# Evidence for the biogenesis of more than 1,000 novel human microRNAs

**DOI:** 10.1186/gb-2014-15-4-r57

**Published:** 2014-04-07

**Authors:** Marc R Friedländer, Esther Lizano, Anna JS Houben, Daniela Bezdan, Mónica Báñez-Coronel, Grzegorz Kudla, Elisabet Mateu-Huertas, Birgit Kagerbauer, Justo González, Kevin C Chen, Emily M LeProust, Eulàlia Martí, Xavier Estivill

**Affiliations:** 1 Genomics and Disease Group, Centre for Genomic Regulation (CRG), Dr. Aiguader 88, Barcelona 08003, Catalonia, Spain; 2 Universitat Pompeu Fabra (UPF), Dr. Aiguader 88, Barcelona 08003, Catalonia, Spain; 3 Centro de Investigación Biomédica en Red Epidemiología y Salud Pública (CIBERESP), Barcelona 08003, Catalonia, Spain; 4 Hospital del Mar Research Institute (IMIM), Dr. Aiguader 88, Barcelona 08003, Catalonia, Spain; 5 Genomic and Epigenomic Variation in Disease Group, Centre for Genomic Regulation (CRG), Dr. Aiguader 88, Barcelona 08003, Catalonia, Spain; 6 MRC Human Genetics Unit, Institute of Genetics and Molecular Medicine, University of Edinburgh, Edinburgh, Scotland; 7 Department of Genetics, Rutgers, State University of New Jersey, Frelinghuysen Road 174, Piscataway, NJ 08854, USA; 8 BioMaPS Institute for Quantitative Biology, Rutgers, State University of New Jersey, Frelinghuysen Road 174, Piscataway, NJ 08854, USA; 9 Genomics Solution Unit, Agilent Technologies Inc., Santa Clara, CA 95051, USA

## Abstract

**Background:**

MicroRNAs (miRNAs) are established regulators of development, cell identity and disease. Although nearly two thousand human miRNA genes are known and new ones are continuously discovered, no attempt has been made to gauge the total miRNA content of the human genome.

**Results:**

Employing an innovative computational method on massively pooled small RNA sequencing data, we report 2,469 novel human miRNA candidates of which 1,098 are validated by in-house and published experiments. Almost 300 candidates are robustly expressed in a neuronal cell system and are regulated during differentiation or when biogenesis factors Dicer, Drosha, DGCR8 or Ago2 are silenced. To improve expression profiling, we devised a quantitative miRNA capture system. In a kidney cell system, 400 candidates interact with DGCR8 at transcript positions that suggest miRNA hairpin recognition, and 1,000 of the new miRNA candidates interact with Ago1 or Ago2, indicating that they are directly bound by miRNA effector proteins. From kidney cell CLASH experiments, in which miRNA-target pairs are ligated and sequenced, we observe hundreds of interactions between novel miRNAs and mRNA targets. The novel miRNA candidates are specifically but lowly expressed, raising the possibility that not all may be functional. Interestingly, the majority are evolutionarily young and overrepresented in the human brain.

**Conclusions:**

In summary, we present evidence that the complement of human miRNA genes is substantially larger than anticipated, and that more are likely to be discovered in the future as more tissues and experimental conditions are sequenced to greater depth.

## Background

Multicellular animals differ widely in complexity of body plan and diversity of cell types. The adult hermaphrodite *Caenorhabditis elegans* nematode is constituted of 959 cells [1], whereas the adult human comprises trillions of cells, including a vast variety of neurons [2]. Although the two species have similar numbers of protein-coding genes, genomic studies show large differences in the complexity of the regulatory networks that orchestrate the expression of proteins during development and in homeostasis [3,4]. These networks include microRNAs (miRNAs): small RNAs that regulate expression of protein-coding genes and play important roles in cell identity, development and disease [5-9]. miRNAs have been detected in all major animal model systems in numbers that largely correlate with organismal complexity, for instance nematodes have approximately 200 whereas humans have approximately 2,000 annotated miRNA genes [10].

Since the first miRNAs were systematically discovered in 2001 [11-13], these regulators have been identified and defined by their biogenesis [14]. During canonical biogenesis, human miRNAs are transcribed as long primary transcripts that each harbor one or more characteristic RNA hairpin structures. These are recognized and cleaved by a protein complex consisting of DGCR8 and Drosha, releasing the so-called miRNA precursor [15]. After being exported to the cytosol, this precursor hairpin is cleaved by the Dicer protein, releasing the terminal loop and two RNA strands about 22 nucleotides in length. One of the two strands is subsequently bound by one of four Argonaute proteins, which form part of the miRISC effector complex. The bound mature miRNA can base pair with 3′-untranslated region binding sites and thus guide the effector complex to target mRNAs, either inhibiting their translation or promoting degradation [16,17].

In practice, most miRNAs have been identified through the use of Sanger sequencing and, later, high-throughput small RNA sequencing (sRNA-seq). miRNAs can be picked out in the large background of cellular sRNAs by their biogenesis: when sequenced miRNA strands are mapped to the precursor hairpin, they will fall in positions characteristic of Drosha and Dicer processing [18,19]. Specifically, sequenced sRNAs should map to positions corresponding to miRNA strands or to the loop, and if both strands are identified, they should form a duplex with overhangs, as is typical of Dicer processing [18].

**Figure 1:**
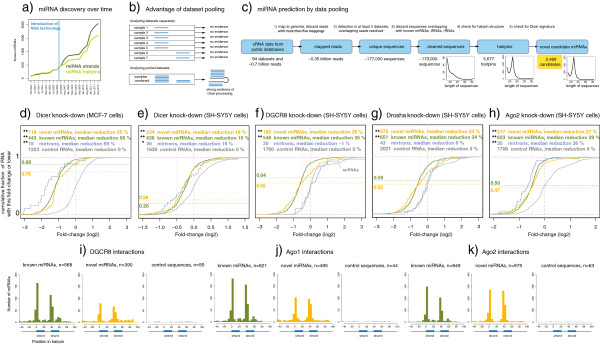
**Evidence for the biogenesis of more than one thousand novel human miRNAs. (a)** Number of human miRNAs in the miRBase database over time. NGS, next-generation sequencing. **(b)** Advantage of dataset pooling. Each of seven sequencing experiments detects a single strand from a miRNA gene (in blue). When the datasets are analyzed separately (top), the single sequencing read does not constitute evidence of miRNA Dicer processing as opposed to random degradation. However, when the datasets are pooled (bottom), the numbers and positions of the reads constitute strong evidence of Dicer processing. **(c)** Synergistic miRNA prediction pipeline. The workflow is described in the Results section. The three plots below the flowchart indicate the length of the sequences retained after each step; notice the approximately 22-nucleotide peak characteristic of Dicer processing. **(d-h)** Four key factors in miRNA biogenesis were silenced with RNA interference. sRNA expression was profiled with high-throughput sequencing in silenced and mock-transfected cells, and the log2 fold-change shown. The curves indicate the cumulative fraction of RNAs with the indicated fold-change or lower. The numbers in the left margins indicate the fractions of miRNAs that are substantially down-regulated (>30% change). Control sequences comprise transfer RNAs (tRNAs), small nucleolar RNAs (snoRNAs) and miscellaneous RNAs (miscRNAs) (in grey). *P <0.01; **P <0.001. **(i-k)** RNAs interacting with DGCR8, Ago1 and Ago2 have previously been detected by crosslinking immunoprecipitation coupled with high-throughput sequencing (CLIP-seq) [22-24]. For each known or novel miRNA the overlaps with these RNAs were plotted as the difference between the 5′ end of the miRNA hairpin and the interacting RNA. The number of miRNAs that are supported by CLIP-seq evidence is shown above each subfigure. In the cases where more than one interacting RNA supported a given miRNA, one random overlap was chosen, such that each data point represents one miRNA. The blue bars indicate the consensus positions of the miRNA strands.

Although nearly two thousand human miRNAs have been identified, and novel ones are reported at a constant rate (Figure 1a), no attempt has been made to provide upper or lower bounds on the miRNA content of the human genome in the last 10 years [20]. To provide such an estimate, we have set out to discover human miRNAs using an innovative computational method employing massively pooled sRNA-seq datasets. We have utilized high-throughput validation methods to provide evidence for the biogenesis of more than one thousand miRNA candidates, and used the first described custom miRNA capture system to show that a substantial fraction of the novel candidates respond to induced cell differentiation. Our new miRNAs appear to be lowly expressed in tissues, raising the possibility that not all may be functional. However, they could be highly expressed and have important functions in individual cells. For instance, the *lsy-6* miRNA is known to be specifically expressed in a single neuron, with important phenotypic effects [21]. Thus as the sRNA field enters the era of single-cell profiling, having a saturated catalog of candidates becomes increasingly important. This catalog should facilitate the evaluation of specific physiological conditions of gene expression that are tightly regulated by miRNAs.

## Results

### Analysis of pooled sRNA-seq datasets yields 2,469 novel human miRNA candidates

When analyzing multiple datasets, it is a critical decision whether to analyze each dataset separately and integrate the results, or to perform a single analysis of the pooled data. In the field of sRNA sequencing, there are numerous advantages to the latter approach [25] (Figure 1b). The presence of both miRNA strands constitutes strong evidence of miRNA Dicer processing; if one set of data contains one strand and another set contains the other, analyzing the two sets simultaneously improves prediction. Pooling data improves the sequencing depth; thus the presence of 10 sequencing reads corresponding to a candidate mature miRNA constitutes stronger evidence than a single read, which could be a random degradation product. Since sRNA-seq includes a PCR amplification step, there is no guarantee that 10 reads within one sample do not represent a single over-amplified RNA; however, a single read in each of 10 samples very likely corresponds to 10 distinct RNAs, and thus constitutes compelling independent evidence that the RNAs are prevalent and the products of specific biogenesis. While mass poolings of dozens of public sRNA-seq datasets have been used to predict mirtrons [26], a subclass of miRNA hairpins that are released by spliceosomal activity, to our knowledge this approach has not previously been used to predict human miRNAs broadly and independent of subclass.

We obtained 94 human sRNA-seq datasets from the Gene Expression Omnibus (GEO) [27], comprising approximately 0.7 billion sequencing reads and representing primary tissues and cell cultures (Additional file 1: Table S1). The datasets were pooled and analyzed with our custom pipeline (Figure 1c, Methods) as follows: reads were mapped to the genome, considering only perfect matches and discarding reads that mapped to more than five genomic locations; sequences not detected in at least two distinct datasets were discarded (in cases where two or more sequences overlapped on the genome, only the sequence that was detected in the largest number of datasets was retained); sequences overlapping known miRNA, rRNA or tRNA genes were discarded; only sequences flanked by RNA hairpin structures similar to miRNA precursors were retained; and reads from the 94 datasets were mapped to the RNA hairpin structures and those with mappings inconsistent with Dicer processing were discarded.

The pipeline yielded 2,469 candidate novel miRNA hairpins. These have features similar to known miRNA precursors, are supported by approximately 22-nucleotide RNAs mapping in accordance with Drosha and Dicer processing, and are each detected in at least two distinct datasets (Additional file 2: Figure S1; Additional file 3: Table S2). Moreover, 420 of the novel candidates were supported by detection of both miRNA strands forming a duplex with typical overhangs (for an example, see Additional file 4: Figure S2).

### Hundreds of novel miRNAs expressed in a neuronal cell system respond to knock-down of biogenesis pathways

To investigate if the expression of these candidate miRNAs depends on Dicer, we re-analyzed sRNA-seq data from a study in which the Dicer transcript was knocked down using RNA interference in MCF-7 breast cancer cells, and sRNA expression in these and control MCF-7 cells was profiled by sequencing [25] (Methods). In total, 119 novel miRNAs could be robustly profiled in this cell type. Expression of known miRNAs was reduced by median 56% upon Dicer knock-down, whereas the expression of the novel miRNAs was reduced by 55% (Figure 1d). Because the novel miRNA candidates had strong representation in human brain (Additional file 4: Figure S3), we repeated the experiment in differentiated SH-SY5Y neuroblastoma cells, knocking down Dicer, Drosha, Argonaute 2 (Ago2) or DGCR8. Each of the knock-downs was efficient at the mRNA levels (31% to 67%), and at the miRNA levels there was good correlation between fold-changes measured by sequencing and by quantitative PCR (qPCR; Pearson’s correlation coefficient 0.75; Additional file 4: Figure S4). In total, 295 distinct novel miRNAs could be profiled in this neuronal cell system. Their expression was consistently and significantly reduced (18% to 35%, *P* <0.001), comparable to already known miRNAs (15% to 35%, Figure 1e-h). Expression of mirtrons was significantly reduced in Dicer and Ago2 but not in the DGCR8 and Drosha knock-downs, consistent with their mode of biogenesis [28,29]. Interestingly, a subset of small nuclear RNA (snRNA) control transcripts appeared up-regulated in the DGCR8 knock-down. This up-regulation was not associated with changes in the length profile of detected snRNAs, suggesting that altered nuclease activity is not the cause (not shown). Summing over the experiments (Methods), 281 of the novel candidates were down-regulated 30% or more upon silencing of the biogenesis pathways.

### More than a thousand novel candidates interact with miRNA key proteins in specific positions

We then investigated if components of the miRNA biogenesis pathways may directly interact with our novel miRNA candidates, by re-analyzing published data from crosslinking immunoprecipitation coupled with high-throughput sequencing (CLIP-seq), which catalog interactions between DGCR8, Ago1, Ago2 and their bound RNAs [22-24]. In the HEK293 kidney cells used in these three studies, interactions with numerous known (568 to 849) and novel (390 to 975) miRNAs were supported by the overlap between miRNA coordinates and the coordinates of the CLIP-seq tags (Figure 1i-k). By contrast, the number of tag overlaps with randomly selected genomic control sequences was much lower (44 to 63 in the three studies, Methods). Further, the interacting RNAs overlapped with both known and novel miRNA hairpins in specific positions corresponding to the two miRNA strands. In the case of Ago1 and Ago2, it is likely that the bound RNAs are in fact mature guide RNAs since a total of 796 novel miRNA strand sequences were identical to one or more Ago CLIP-seq tags, allowing only for minor length variation in the 3′ end. Similarly, 281 novel miRNA sequences were detected in the DGCR8 interaction data.

We decided to complement these methods with classical immunoprecipitation (IP) studies. Because the novel miRNA candidates had good representation in SH-SY5Y neuronal cells, we employed polyclonal antibodies to immunoprecipitate the endogenous Ago2 protein in these cells and compared the results with untreated control cells (Methods). Interacting RNAs were profiled with sRNA sequencing. We found 1,177 known and 720 novel miRNAs in this experiment (Additional file 4: Figure S5). Of these, 85% of the known miRNAs and 65% of the novel miRNAs were detected in the IP data, while the remainder were detected only in the untreated control cells. We further searched published Argonaute immunoprecipitation data for the presence of known and novel miRNAs. The recent sequencing study by Dueck *et al.*[30] covers both input controls and Argonaute 1 to 3. In these data we found 803 known and 341 novel miRNAs. Of these, 87% of the known and 81% of the novel miRNAs are detected in at least one Argonaute IP, while the remainder are only detected in the input sample. This indicates that substantial fractions of the novel miRNAs interact with the Argonaute effector proteins, consistent with the CLIP-seq results.

Overall, these functional evaluations showed that 1,098 new candidates were supported by detection of both strands, robust down-regulation upon silencing of the biogenesis pathways, or interaction with miRNA biogenesis proteins (Additional file 4: Figure S6). While these data do not demonstrate that the novel candidates are functional, they provide compelling evidence that the sequences undergo canonical miRNA biogenesis.

### Experimental identification of novel miRNA targets

An innovative method, crosslinking, ligation and sequencing of hybrids (CLASH), has recently been developed to experimentally identify miRNA-target pairs [31]. Methods such as CLIP-seq can identify miRNAs and mRNAs bound to Argonaute proteins, but CLASH stands out in that it produces information on the exact pairings of miRNAs and mRNAs. The approach relies on directly ligating miRNAs to interacting mRNAs and sequencing the resulting ‘chimeric’ cDNA. We identified interactions for 89 novel miRNA candidates in CLASH interactions from human kidney cell culture [31]. Of these, two candidates interacted with numerous mRNAs, but both had low sequence complexity and neither had support for an miRNA star strand. We therefore concluded that these were more likely endogenous short interfering RNAs (siRNAs) than miRNAs, and omitted them from the rest of the analysis. The remaining 87 novel miRNAs were found to interact with 245 distinct mRNAs (Additional file 5: Table S3; Additional file 6: Table S4).

**Figure 2:**
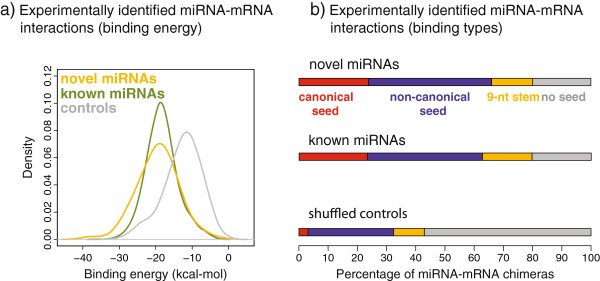
**Experimentally identified miRNA-mRNA interactions. (a)** A method was recently developed to sequence miRNAs ligated to their mRNA targets [31]. In these data, we identified 256 distinct interactions between 87 novel miRNAs and 245 mRNAs. The inferred binding energies of the novel miRNAs are similar to those of known miRNAs, and are significantly stronger than the binding of shuffled control sequences (*P* <0.001, sub-sampling). **(b)** The miRNA-mRNA interactions were grouped based on 5' seed pairing. Both novel and known miRNAs tend to bind by canonical seed pairing, in contrast with control sequences. nt, nucleotide.

The novel miRNAs are predicted to bind their targets with high affinity, comparable to known miRNAs and significantly more strongly than shuffled control sequences (Figure 2a). Similarly, the interactions are enriched for 5'-end seed binding, which is typical of canonical miRNA target recognition (Figure 2b) [32,33]. This constitutes strong evidence that the 87 novel candidates bind their 245 targets by miRNA-specific mechanisms. In the kidney cells, we found that the novel targets were enriched in the functions of protein biosynthesis (*P* = 1.9e^-8^ after Benjamin-Hochberg correction), RNA-binding (*P* = 5.6e^-6^), ubiquitin-like conjugation (*P* = 1.1e^-3^) and nucleoplasm (*P* = 1.6e^-3^). Further, several of the target genes, such as *ABL1*, *CDKN1B*, *TP53*, *YWHAE* and *ZBTB10*, have established roles in disease (Additional file 5: Tables S3; Additional file 6: Table S4).

**Figure 3:**
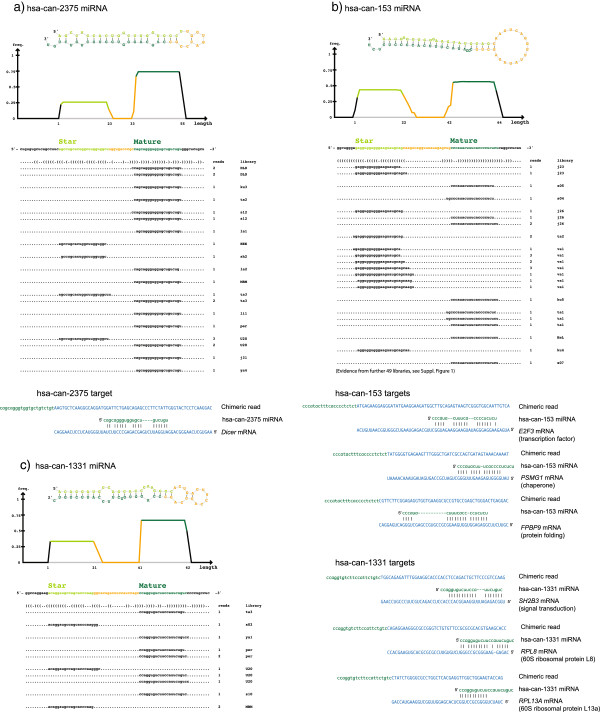
**Three novel miRNAs and experimentally identified targets. (a)** miRNA candidate 2375 targets *Dicer* mRNA. The density plot and read alignments show the distribution of sequenced RNAs mapping to the miRNA precursor, summing over 15 sRNA-seq datasets. The two miRNA strands are indicated in light and dark green. Above, the RNA structure of the precursor. Below, the candidate 2375 miRNA (dark green) ligated to the interacting *Dicer* mRNA (blue). **(b)** Candidate 153 is integrated in protein biosynthesis pathways. The miRNA is derived from an intron of the EIF2B3 translation factor, and interacts with mRNA of *PSGM1*, an established chaperone, and *FPBP9*, involved in protein folding. **(c)** Candidate 1331 targets ribosomes. It interacts with three mRNAs, of which two are distinct parts of the same 60S ribosomal subunit (*RPL8* and *RPL13A*).

We noted that several of the novel miRNAs had multiple targets and appeared to be integrated in regulatory networks (Figure 3a-c). For instance, candidate 2375 had an experimentally identified target in the transcript of Dicer, one of the key proteins in the miRNA biogenesis pathway. Novel miRNA targets were enriched in protein biosynthesis, and candidates 153 and 1331 exemplify this. Candidate 153 was cleaved from the sixth intron of the EIF2B3 translation factor, and interacted with mRNA of *PSGM1*, an established chaperone, and *FPBP9*, involved in protein folding. Candidate 1331 had three identified targets, of which two were distinct parts of the same ribosomal 60S subunit. In sum, the targets of the novel miRNAs did not appear random, but rather were part of regulatory networks.

### The novel miRNA genes are evolutionarily young and specifically expressed

**Figure 4:**
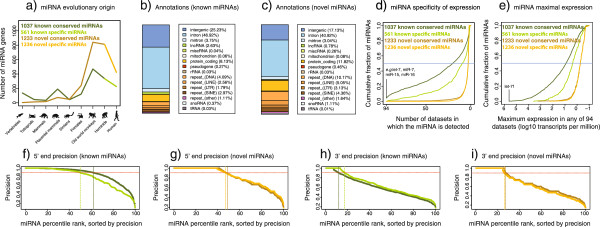
**Features of the identified novel human miRNAs. (a)** Inferred evolutionary origin of known and novel human miRNAs. Representative species are shown for each clade: hominids are represented by chimp; old world monkey, baboon; primate, tarsier; simian, tree shrew; placental mammal, armadillo; mammal, platypus; tetrapod, clawed frog; and vertebrate, zebrafish. The miRNAs have been divided into those specific to hominids and humans (‘specific’ in light green or orange) and those conserved in old world monkeys or beyond (‘conserved’ in dark green or brown). **(b,c)** Genomic sources of known and novel miRNAs. **(d)** Specificity of expression. The horizontal axis shows the number of datasets (out of 94) in which the miRNA is detected. The vertical axis shows the cumulative fraction of miRNAs present in at least these many datasets. The horizontal blue line indicates the median number of datasets in which the miRNAs are present. Examples of miRNAs detected in all 94 datasets are noted. **(e)** Maximal expression. As in the previous figure, except the maximum expression of each miRNA in any of the 94 datasets is shown. Expression is normalized (transcripts per million reads) to adjust for varying sequencing depth between the samples. The miRNA with the highest normalized expression in any dataset is *let-7 f*. **(f-i)** The processing precision of known and novel miRNAs. The precision is defined as the fraction of mapping reads that correspond to the consensus end position of the sequence. The miRNAs are sorted on the x-axis such that the most precisely processed one is at percentile 1 and the least precisely processed one at percentile 100. Dark colors indicate conserved miRNAs, light colors non-conserved.

Some miRNAs originated more than 500 million years ago in the common ancestor of the Cnidaria and Bilateria, whereas others are species-specific [34,35]. We found that 136 (8%) of the known human miRNAs are conserved in vertebrates (Figure 4a) and that there are substantial miRNA gains in the ancestor of the placental mammals (190 genes, 12%) and the ancestor of the old world monkeys (469 genes, 32%), consistent with previous observations [36-38]. By contrast, only three (0.1%) of the novel miRNA candidates are conserved in vertebrates, and the gain in the ancestor of placental mammals is less pronounced than for the known miRNAs (74 genes, 4%). However, 841 of the novel miRNAs (34%) appear to have originated in the ancestor of old world monkeys. Overall, the pattern of evolutionary origin of the novel miRNAs is comparable to that of the known ones, but with more recent gains and almost no deeply conserved genes.

Known miRNAs were often located in intronic (47%) or intergenic (25%) regions, with smaller fractions originating from exons (8%), repeats (12%) or other annotations (4%, Figure 4b,c). The novel candidates had less tendency to locate to intronic (41%) and intergenic (17%) regions, whereas exons (12%) and repeats (24%) were overrepresented, and a comparable fraction (3%) were transcribed from other annotations. These observations are consistent with theories supporting that young miRNAs often originate as exaptations of existing transcripts [39,40].

To investigate specificity of expression, for each miRNA we observed how many samples it was detected in (out of the 94 datasets used for this study). The known miRNAs that are conserved in old world monkeys or beyond were prevalent, being detected in a median 20 samples (Figure 4d). The known miRNAs that are specific to hominids were less prevalent, being detected in a median five samples. Novel miRNAs were specific in their expression, with more than half being detected in only two or three samples. This specific expression can in part explain why the miRNAs have not previously been annotated. To study maximal expression, for each miRNA we identified the dataset in which it had the highest normalized expression (Figure 4e). The known miRNAs conserved in old world monkeys or beyond had the highest expression at median 8.2 transcripts per million (TPM), while the known hominid-specific and novel miRNAs were expressed at a median 2.3 and 0.5 TPM, respectively.

It is a hall-mark of mature miRNAs that they have precisely defined 5′ ends. This is critical because mRNA target recognition depends strongly on this part of the sequence. We here define that a miRNA is precisely processed if nine out of ten derived RNAs map to the consensus position (in our pooled data). We found that 48% of the novel candidates had precisely processed 5′ ends, similar to known miRNAs that are specific to hominids (50%, Figure 4f,g). By contrast, more of the conserved miRNAs were precisely processed (62%). Both known and novel miRNAs had fewer mature sequences with precisely processed 3′ ends (Figure 4h,i). As previously observed [41], several well-studied and deeply conserved miRNAs had imprecise processing, although they were highly expressed (Additional file 4: Figure S7). We conclude that the processing of the novel miRNAs is comparable to that of species-specific known miRNAs, but less precise than that of conserved known miRNAs.

### Selective constraint on novel microRNA candidates

We examined the patterns of natural selection acting on our novel microRNA candidates using single nucleotide polymorphism (SNP) allele frequency data from the 1000 Genomes Project. Specifically, we compared the derived allele frequency spectrum of SNPs in the novel miRNA candidates to an appropriate background set of SNPs chosen from the rest of the genome using a one-sided Wilcoxon test (Methods). A significant enrichment of rare derived alleles in novel miRNA candidates is indicative of selective constraint on these loci.

We decided to restrict our analysis to the approximately 1,300 novel miRNA candidates in intergenic regions. When we compared this set of SNPs to the background set of all intergenic SNPs in the genome, we observed a marginally significant signal of selective constraint on intergenic novel miRNA candidates (one-sided Wilcoxon test, *P =* 0.058). Our analysis suggests that novel miRNA candidates may be under weak selective constraint on average. Inspection of the derived allele frequency spectrum showed no excess of intermediate or high frequency alleles that would be consistent with balancing selection or adaptive evolution models (data not shown). We therefore conclude that there is a marginally significant trend for the novel miRNA candidates to be under weak selective constraint, or alternatively there is a smaller (unknown) set of novel miRNA candidates under high levels of selective constraint within the whole set of novel candidates.

### Enrichment of target miRNAs with a custom capture system

**Figure 5:**
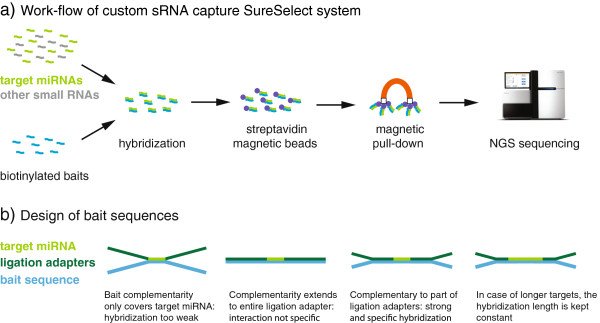
**Design of the custom SureSelect miRNA capture system. (a)** The workflow of the capture system. An sRNA-seq library contains miRNAs of interest and other small RNAs. The library is mixed with biotinylated cRNA baits in solution. The baits are complementary in sequence to the target miRNAs and specifically hybridize with these. Magnetic streptavidin-coated beads are added to the solution and bind to the biotin. Last, the baits and their bound targets are isolated by magnetic separation, and the target miRNA clones can be sequenced. **(b)** The short size of miRNAs requires specific design considerations, since the SureSelect baits are much longer, at 120 nucleotides. If the baits are designed to hybridize with the target miRNA only, the interaction might be weak and targets lost. If baits are complementary to the target miRNA and the entire length of the flanking ligation adapters, which are added during library preparation, then specificity might be lost because most binding is to the universal adapter sequences. We have designed the baits so that they are complementary to the target miRNA and part of the adapters, giving a strong and specific hybridization. For targets longer than typical miRNAs, the length of the hybridized region is kept constant by dynamically varying the part of the adapters which is baited. NGS, next-generation sequencing.

To study the behavior of the novel miRNA candidates during biological processes, we induced SH-SH5Y cells to differentiate to neuron-like state. We used sRNA-seq to measure expression; to achieve superior profiling depth of the novel candidates, we enriched the sequencing libraries with the first described custom miRNA capture system. The system highly enriches a limited set of specific lowly expressed miRNAs at the expense of other miRNA sequences. The system was designed in collaboration with the Agilent Technologies Inc., employing their SureSelect technology. This is a solution-based method that uses biotinylated baits to capture cDNA of interest, including transcripts sequenced by high-throughput platforms (Figure 5a). An inherent challenge in applying this technology to sRNAs is that these transcripts are much shorter than the baits, which are 120 nucleotides in length. Thus, designing baits that are only complementary to the insert sRNAs might cause the binding to be too weak (Figure 5b). By contrast, having complementarity to the sRNA and the full length of the ligation adapters could cause a loss of specificity, because the adapter sequences are shared between target and non-target sRNAs. Balancing binding strength and specificity, we designed the baits to be complementary to the sRNA and part of the adapters, keeping the number of hybridized nucleotides constant. We generated baits for our novel human miRNAs, plus controls consisting of 500 known miRNAs, 200 tRNA sequences and 200 rRNA sequences (Additional file 7: Table S5). All of the controls were lowly expressed in the neuroblastoma cells. sRNA-seq libraries from undifferentiated and differentiated cells were prepared and sequenced both with and without enriching for target sRNAs using the SureSelect capture system (Methods).

### Capture system strongly enriches for target miRNAs and is quantitative

**Figure 6:**
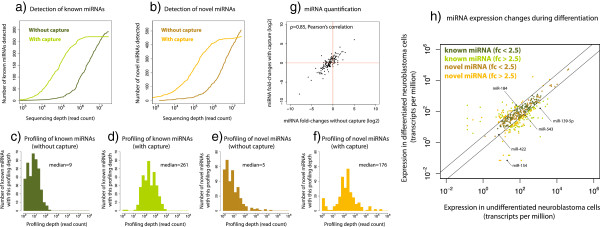
**Profiling novel miRNAs during differentiation with capture. (a)** Saturation curve of miRNA detection with or without SureSelect capture-based enrichment. For sequencing depths ranging from 10 thousand to 30 million reads, the number of known target miRNAs detected is shown. Differentiated neuroblastoma cells were profiled. **(b)** As before, but for novel miRNAs. **(c-f)** Profiling depth with or without capture. The histograms show for each target miRNA how many times it is detected. **(g)** miRNA expression changes measured with or without capture. Neuroblastoma cells were induced to differentiate, and fold-changes were estimated for 140 known miRNAs that could be reliably profiled both with and without using the capture system. **(h)** miRNA expression changes during differentiation. The capture data from the previous figure could be used to robustly profile 428 known and novel target miRNAs, as cells underwent differentiation to neuron-like state. For each miRNA the normalized expression in the two states is shown. Five miRNAs have previously been observed to be regulated during differentiation of SH-SY5Y cells [42]. Our results are in agreement with the up- or down-regulation of four of these miRNAs, while the fifth, miR-422, is a border case in our measurements.

We found that the targeted miRNAs were detected at much lower depth with than without the capture (Figure 6a,b). For instance, 90% of the known target miRNAs in the sample were detected at a depth of 13 million reads without the capture system, whereas only 1.1 million reads were required using the capture system. The sensitivity of the system, estimated as the percentage of target miRNAs detected without the capture that were also detected with the capture, was 93% and 91% for known and novel miRNAs respectively. At a depth of 30 million reads, few target miRNAs were detected with the capture that were not detected without the capture (not shown). This probably reflects that the cDNA library had been sequenced to near saturation at this depth. However, even with this saturated sequencing, the profiling depth of the target miRNAs was substantially improved using the system (Figure 6c-f). After capture, the known miRNAs increased from a median 9 reads to 261 reads, while the novel miRNAs increased from a median 5 reads to 176 reads. This can make the difference between a miRNA that for numerical reasons cannot be profiled, and one that can. The target system also enriched other types of targeted sRNAs (Additional file 4: Figure S8). To see if the capture system retained quantification, we identified 140 known target miRNAs that are robustly profiled with and without the capture (Figure 6g). There was good correlation between the expression fold-changes measured with the capture and without (ρ = 0.85, Pearson’s correlation). Thus the capture system is quantitative and can be used to profile expression changes between conditions.

### A substantial fraction of novel miRNAs respond to induced differentiation

When using the capture system to profile neuroblastoma cells in differentiated and undifferentiated state, we could reliably assign fold-changes to 428 lowly expressed target miRNAs, known and novel (Methods). Interestingly, the same fraction (44%) of the known and novel miRNAs changed expression (Figure 6h). Similarly, we profiled target sequences present in 264 tRNAs of which only 17% changed expression (not shown). None of the four rRNA housekeeping genes altered expression during the differentiation. miRNAs have previously been reported to be regulated during SH-SY5Y differentiation using retinoic acid as in our study [42]. The overlap with our miRNA controls was limited, because we specifically chose transcripts that were lowly expressed in these cell lines to test the limits of the capture system. Thus, most of our target miRNAs were minor strands that were are below detection limits of other technologies. However, we found that four out of five of the overlapping miRNAs were up- or down-regulated in agreement with the previous results, validating our profiling method (Figure 6h). In conclusion, we found that our novel miRNA candidates responded similarly to known miRNAs, but not tRNAs or rRNAs, during induced cell differentiation. This indicates that the novel candidates are not chance side-products of housekeeping RNAs or spurious transcripts, but are in fact linked to core regulatory processes of the cells.

## Discussion

We used synergistic miRNA discovery to analyze 94 human sRNA-seq datasets, yielding 2,469 novel miRNA candidates. These were each supported by a typical RNA hairpin structure and an approximately 22-nucleotide sRNA mapping to the hairpin in accordance with Dicer processing and detected in at least two sequencing experiments. In addition, we characterized the novel miRNA candidates in more detail in two cell systems. In a neuronal cell system, we found that our candidates responded similarly to known miRNAs when components of the biogenesis pathways were knocked down or when the cells were induced to differentiate.

We used public data from a human kidney cell line to show that comparable numbers of novel and known miRNAs interact with key proteins DGCR8, Ago1 and Ago2, in hairpin positions that conform with miRNA biogenesis. The abundance of novel sequences bound to Ago1 and Ago2 suggests that they did not just undergo chance interactions with the biogenesis machinery, but were indeed incorporated by the effector proteins. Last, evidence from CLASH data showed that novel miRNAs had canonical binding to target mRNAs. The interaction strength and seed recognition resembled those of known miRNAs but not random sequences, as would be expected if Argonaute incorporation was spurious. Several of the novel miRNAs interacted with multiple mRNAs in the kidney cells, and appeared to form part of regulatory networks.

While these particular two cell systems do not give saturated coverage of all novel miRNA candidates, we have no reason to doubt that experiments in other cell systems would yield similar positive results. In sum, we have provided additional evidence for the biogenesis of 1,098 novel miRNA candidates (Additional file 4: Figure S6). We have thus presented compelling evidence that the number of human miRNA genes is larger than anticipated at over three thousand genes.

When enriching our novel miRNA candidates with the first described custom miRNA capture system, we showed that they responded similarly to known miRNAs, but not tRNAs and rRNAs, during induced cell differentiation. This suggests that the novel miRNAs were not the results of leaky transcription, but were closely linked to regulatory processes. Further, the SureSelect capture system shows great promise: it strongly enriched for target sRNAs while being fully quantitative. At low-pass sequencing, it improved detection of targets (Figure 6a,b) and at saturated sequencing it improved the profiling depth of targets (Figure 6c-f). With some maturation, a custom miRNA capture system could be used to profile dozens of miRNA samples on an Illumina miSeq instrument in less than one day. This clearly has potential clinical applications with rapid processing of patient sample sets.

Overall, our novel candidates have features similar to known miRNAs, in particular we note that they interacted with Argonaute effector proteins and displayed typical targeting sequence characteristics. The specific and low expression levels of the novel candidates were expected, because there is a strong discovery bias favoring abundant transcripts. The apparent low expression in tissues does not exclude the possibility that some of the novel miRNAs may be highly expressed and have important functions in specific cell types [21]. This is an appealing hypothesis because the novel candidate miRNAs are overrepresented in human brain, which is known to harbor a vast diversity of neuronal cell types. Thus our catalog may provide a valuable resource as the small RNA field enters the single-cell era, facilitating the evaluation of specific physiological conditions of gene expression at the cellular level, which is tightly regulated by miRNAs.

Last, in this study we have presented evidence of the biogenesis of our novel human miRNAs. However, biogenesis does not necessarily imply biological function that confers an adaptive advantage. It is conceivable that hairpins may enter the miRNA biogenesis pathways but have insubstantial impact on the transcriptome because they are lowly expressed or do not recruit the necessary co-factors [43]. In fact, our population genetic studies suggest that many, but likely not all, of our novel human miRNAs are under selection pressure. In general, it is not is easy to discern if a given miRNA has a function. miRNA biochemical function can be validated using reporter assays that express transcripts at physiological levels, but this is extremely time consuming. Deeply conserved miRNAs are likely to be functional, but the reverse does not necessarily hold, as there are examples of species-specific miRNAs with well-defined functions [44]. We think that it is important that miRNA annotations are saturated to ensure that future studies will pick up sequences which change expression patterns during development or in disease, in tissues or in single cells. These miRNAs can then be subjected to careful functional assays. Saturating the miRNA annotations risks diluting out the deeply conserved and well-studied sequences deposited there, but this can easily be avoided by stratifying the sequences according to confidence. miRBase has already curated a ‘core annotation’ of miRNAs with compelling evidence for biogenesis [45], and a recent study has identified a subset of sequences supported by functional evidence [46]. Similarly, we have stratified our novel miRNA candidates into five confidence levels based on the evidence presented in our study (Additional file 3: Table S2), enabling researchers to decide their own levels of stringency.

**Figure 7:**
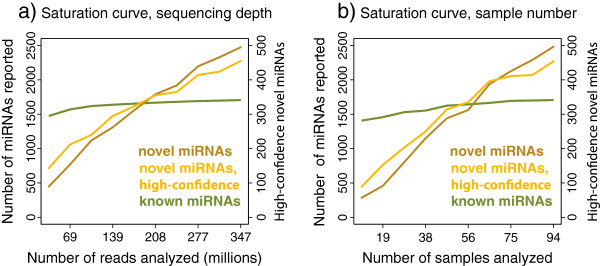
**Saturation of novel miRNA prediction.** To assess the influence of data magnitude on the analyses, saturation curves of the 94 datasets were performed. **(a)** Saturation curve of sequencing depth, from 10% to 100% of reads retained. For each dataset this percentage of (randomly chosen) reads were retained and subsequently the miRNA prediction analysis was repeated. The total number of reported novel miRNAs (brown) or high-confidence novel miRNAs (orange) is shown. The number of known miRNAs that are detected by simple sequence matches is shown in green. **(b)** As before, except that entire datasets rather than individual reads were discarded or retained.

To investigate if other species harbor large numbers of undiscovered miRNAs, we repeated the prediction in mouse, using public sequencing data of a comparable volume to the data used in human, compiled from 11 distinct studies. This yielded 1,520 novel mouse miRNA candidates (unpublished results). Interestingly, this is one-third fewer than the number reported in human, although the mouse data has excellent coverage of tissues, including samples from brain and from several developmental time points [41]. Revisiting the human data with simulations, we found that the number of reported candidates scale almost linearly with the amount of data analyzed (Figure 7), suggesting that human miRNA discovery has not yet reached saturation, even with our added set. This shows that many more miRNAs remain to be discovered, both in well-studied model organisms and in human.

## Conclusions

We discovered 2,469 novel miRNAs, of which we validated 1,098, making this the most comprehensive human miRNA report to date. The novel candidates had features similar to known miRNAs, but most were evolutionarily young, specific in expression, and overrepresented in the human brain. In a kidney cell line the novel miRNAs targeted hundreds of mRNAs, and appeared to form part of regulatory networks. We present evidence that the complement of human miRNA genes is substantially larger than anticipated and that more remain to be discovered.

## Methods

### miRNA discovery using 94 pooled human sRNA-seq datasets

The 94 datasets were obtained from the GEO database [27] and pre-processed as detailed in Additional file 1: Table S1. The sets were chosen according to several criteria, they should originate from human tissues or cells, have a good representation of miRNAs (>50% miRNA content), be sequenced by Illumina protocols, and be free of confounding factors (transfection experiments, RNA interference and so on). The selection was performed in April 2011. The prediction of novel miRNAs was performed in five steps. First, the processed reads were concatenated to a single FASTA file and were mapped to the human genome (version hg19) with bowtie [47], using the following options: bowtie –f –v 0 –a –m 5 --strata --best; reads that did not map using these options were discarded. Second, the remaining reads were collapsed to unique sequences and it was noted how many datasets each sequence was detected in (the ‘prevalence’). Reads that were only detected in a single dataset were discarded, and the remaining ones were assigned weights equal to their prevalence divided by the number of genome mappings. For each pair of sequences that overlapped on the same genome strand, only the sequence with the highest weight was retained, so that in the end no sequences overlapped. This step ensured that only the major miRNA form would be considered at each locus, while minor miRNA variants were discarded. Third, all sequences overlapping with known miRNA hairpins (miRBase version 19 [10]) or annotations of tRNA or rRNA (RepeatMasker hg19 annotations from the University of California, Santa Cruz (UCSC) table browser [48]) were discarded. Fourth, the remaining sequences were excised from the genome twice, once including 10 nucleotides upstream and 70 nucleotides downstream, and once including 70 nucleotides upstream and 10 nucleotides downstream, corresponding to the sequence being the miRNA strand from the 5′ or 3′ hairpin arm respectively. Each excised hairpin was evaluated by the MiPred hairpin structure predictor [49], and only sequences estimated to be real miRNA precursors by their structural features were retained. Fifth, the reads from the concatenated FASTA in step one were mapped to the predicted hairpins with bowtie using these options: bowtie –f –v 0 –a --strata --best, and the read mappings were evaluated for Dicer patterns using miRDeep2 [25] (version 2.0.0.5) with the following options: miRDeep2_core_algorithm.pl –v −100. Even though the score cut-off was set low, the algorithm by default discarded candidates where more than 10% of the reads mapped inconsistently with Dicer processing. Last, in cases where two remaining hairpins overlapped on the same genome strand, only the one with the most prevalent seeding sequence was retained (see step two). This step ensured that only one hairpin was reported from each gene locus. After this last step, 2,469 novel candidate miRNA hairpins remained.

### Induced differentiation and knock-down of miRNA biogenesis

SH-SY5Y cells (human neuroblastoma) were grown in Dulbecco’s Modified Eagle’s Medium (Invitrogen, Carlsbad, CA, USA) supplemented with 10% fetal bovine serum, 2 mM L-glutamine, 100 units/mL penicillin and 100 μg/mL streptomycin (GIBCO, Invitrogen). SH-SY5Y cells were differentiated towards a more post-mitotic neuron phenotype through the administration of 10 μM retinoic acid in the culture medium over five days. The medium was then replaced by growth medium supplemented with 80 nM of 12-O-tetradecanoylphorbol-13-acetate for five additional days. Transfection assays were performed on differentiated cells at a 60% cell confluence. Lipofectamine 2000 (Invitrogen) was used as a transfection reagent following the manufacturer’s protocol. siRNAs against DGCR8 (5′-GAAGCUCAUUACUUUAUCA-dTdT-3′), Drosha (5′- AACGAGUAGGCUUCGUGACUU-dTdT-3′), Dicer (5′- GCUCGAAAUCUUACGCAAAUAdTdT-3′) and Ago2 (5′-GCACGGAAGUCCAUCUGAA-dTdT3′) were purchased from Dharmacon. DGCR8 and Drosha knock-down were performed by a double transfection procedure that consisted of administering each siRNA after neuronal differentiation and 48 hours later. The siRNA concentration for each round of transfection was 50 nM (first round) and 25 nM (second round) for siDGCR8 and 75 nM (first round) and 75 nM (second round) for siDrosha. Dicer and Ago2 knock-down was performed by a single transfection procedure in which siRNAs were administered 40 hours after neuronal differentiation using concentrations of 75 nM for both genes. In all cases, cells were processed 72 hours after transfection for RNA extraction. Transfection efficiency was monitored using siGLO transfection indicator (Dharmacon).

### sRNA library preparation, sequencing and data pre-processing

Total RNA was extracted using miRNeasy Mini kit (Qiagen) according to the manufacturer’s instructions. From each sample, 1 μg of total RNA was used to prepare indexed libraries according to Illumina TruSeq Small RNA Sample Preparation protocol. Sequencing was performed on a HiSeq2000 instrument running TruSeq version 3 chemistry for 50 cycles. Base calling and quality score calculation was performed from raw intensities using Illumina’s pipeline version 1.8.1. The called reads were trimmed with the command line: fastx_trimmer –f 1 –l 36 and low-quality reads discarded with fastx_artifacts_filter using the options –q 10 [50]. Adapters were clipped using the AdRec.jar program from the seqBuster suite [51] with the following options: java -jar AdRec.jar 1 8 0.3. A custom search subsequently clipped shorter adapters: if there were no matches to the first eight nucleotides, then matches to the first seven nucleotides of the adapter were searched in the last seven nucleotides of the read, then matches of the first six to the last six positions and so on. Reads that had no matches were retained, but not clipped. Last, reads shorter than 18 nucleotides were discarded. A detailed overview of the samples sequenced in this study is shown in Additional file 8: Table S6.

### Quantitative PCR measurements of mRNA and miRNA fold-changes

Knock-down efficiency was evaluated by qPCR. Total RNA from SH-SY5Y cells was treated with the DNA-free kit (Ambion). cDNA synthesis was performed with 500 ng of DNA-free RNA using SuperScript III First-Strand Synthesis System for RT-PCR (Life Technologies) following manufacturer’s instructions. The cDNA product was diluted to one-fifth with sterile water. Real-time PCR reaction was performed using TaqMan Gene Expression Assays Hs00229023_m1 for *Dicer*, Hs00377897_m1 for *DGCR8*, Hs01085579_m1 for *Ago2*, Hs00203008_m1 for *Drosha*, Hs00819388_m1 for *MPRIP*, Hs00196523_m1 for *POLR2J*), following manufacturer’s instructions in an AB 7900HT Fast Real-Time PCR System. Amplification was done under the conditions: 15 s at 95°C followed by 55 cycles consisting of 1 min at 60°C and 2 min at 72°C on the ABI PRISM 7000 Detection system (Applied Biosystems). Each sample was run in quadruplicate and the cDNA synthesis repeated at least twice. Data were normalized using two independent endogenous reference genes, *MPRIP* and *POLR2J*. The relative quantification and its statistical significance were obtained from a linear mixed-effects model that accounted for the different sources of variation derived from the experimental design [52]. Gene expression assays for human miR-16-5p, let-7f-5p, miR-324-5p and miR-361-5p were performed using TaqMan MicroRNA Reverse Transcription Kit followed by TaqMan MicroRNA Assays (assay ID: 000391, 000382, 000539 and 000554, respectively). Data were normalized using *RNU6* or *RNU58B* (assay ID: 001093, 001206, respectively). The assays were performed following the manufacturer’s instructions. Briefly, 10 ng of total RNA was used per reverse transcription reaction, performed in duplicate. A 1:15 dilution of the reverse transcriptase reaction was used as input for the qPCR amplification step, performed in quadruplicate per reverse transcription reaction. Amplification conditions were as follows: 2 min at 50°C, 10 min at 95°C, and 40 cycles of 15 s at 95°C and 1 min at 60°C, on a 7900 HT Fast Real-Time PCR System (Applied Biosystems). As before, the relative quantification and its statistical significance were obtained from a linear mixed-effects model [52].

### Estimating sRNA fold-changes upon knock-down of miRNA pathways

To estimate fold-changes, the following datasets were compared, corresponding to Figure 1e-h: Control 2 versus Dicer knock-down; control 1 versus DGCR8 knock-down replicate 1 and 2 (pooled); control 1 versus Drosha knock-down replicate 1 and 2 (pooled); and control 2 versus Ago2 knock-down . For estimating the number of miRNAs that were overall down-regulated by 30% or more upon knock-down of the miRNA biogenesis pathways, the two controls were pooled and compared with the four pooled knock-downs, and normalized as below. For each comparison, the reads were mapped to the following reference sequences: the novel candidate miRNA hairpins; known miRNA hairpins and mirtrons (miRBase version 19 [10] and control sequences (snoRNAs from snoRNABase [53]); tRNAs from tRNAdb [54]; and miscellaneous RNAs from GENCODE version 8 [55]). The latter were excised from GTF coordinates with a custom script. The mapping was performed with bowtie using the following options: −f -v 0 -a --best --strata --norc. For each reference sequence, the sum of reads mapping from the control and the knock-down dataset was calculated. If this sum was less than 30, the sequence was not considered and is not plotted in Figure 1e-h. If this sum was 30 or higher, the log2 fold-change was calculated as follows: f = log2(number of reads mapping from knocked down sample/number of reads mapping from control sample). Because the sequencing depth differed between the samples, the fold-changes were normalized to the control sequences. This normalization correlated well with independent measurements by qPCR (Additional file 4: Figure S4 and Methods). Significance levels were estimated using sub-sampling of the control sequences, generating subsets similar in size to the numbers of known and novel miRNAs profiled. For each knock-down, one million random subsets were generated. The number of transcripts profiled in each sub-experiment differed because of varying sequencing depth and the sharp cut-off of 30 reads, mentioned above. However, there was a tendency for the same novel miRNA candidates to exceed the 30 read cut-off in the different sub-experiments. For instance, 171 novel candidates exceeded the cut-off and were plotted in all four sub-experiments. Note that the higher knock-down efficiency at the miRNA level in MCF-7 versus SH-SY5Y cells as measured by sequencing (56% versus 15% to 35%, respectively) was reflected in the efficiency at the mRNA level as measured by qPCR. The knock-down efficiencies for the MCF-7 cells were 54% to 84% [25]. The efficiencies for the SH-SY5Y cells were 29% (Dicer), 67% (DGCR8), 55% (Drosha) and 32% (Ago2).

### Analysis of DGCR8, Ago1 and Ago2 CLIP-seq data

We obtained the data at the GEO database (DGCR8, SRR518495-8 [22]; Ago1, SRR650318-20 [23]; Ago2, SRR189782-7 [24]). The DGCR8 reads were clipped of adapter with fastx_trimmer from the FASTX suite with this command line: fastx_trimmer –f 1 –l 21 –Q33. The Ago1 and Ago2 reads were processed using the method described above in the section ‘sRNA library preparation, sequencing and data pre-processing’. Reads were mapped to the human genome (hg19) considering only unique mappings: bowtie –v 1 –m 1 --strata --best. For each of the three proteins, the positions on the reads on the genome were intersected with positions of known miRNA hairpins (miRBase version 19 [10]), or the novel candidate hairpins, on the same genome strand using a custom script (available upon demand), and the difference in 5′ position was plotted in Figure 1i-k. CLIP-seq reads mapping within 20 nucleotides of an miRNA hairpin were also considered. In the cases where more than one read overlapped a given hairpin, one read was randomly chosen for plotting, such that each data point in Figure 1i-k represents one hairpin. Because miRBase hairpins differ in the length of flanking sequence, the excision scheme used for predicting miRNAs (see Methods) was used to generate miRBase hairpins of a homogeneous length. The control sequences were generated by randomly sampling positions in the human genome (hg19) with equal probability of selecting each nucleotide in the genome and specific to the strand. The 80 nucleotides bracketing each of these positions were excised and overlapped with the CLIP-seq tags, as described above. In total, 275,400 positions (100 for each candidate hairpin mapping) were excised and the number of overlaps scaled correspondingly.

### Ago2 immunoprecipitation in SH-SY5Y cells

SH-SY5Y neuroblastoma cells were maintained in Dulbecco’s Modified Eagle’s Medium (Invitrogen) supplemented with 10% heat-inactivated fetal bovine serum, 2 mM L-glutamine, 100 units/ml penicillin and 100 mg/ml streptomycin (GIBCO, Invitrogen). Differentiation was induced by growing the cells for three days in standard medium containing 10 mM retinoic acid and an additional five days in fresh standard medium containing 80 nM of 12-O-tetradecanoylphorbol-13-acetate [56]. Ago2 IP and subsequent RNA isolation were performed as described previously [57], with a few adaptations. Cells were lysed in a 1% Triton lysis buffer containing 40 units/μl RNaseOUT (Invitrogen). Endogenous Ago2 was precipitated by incubating the cell lysate with 30 μg of anti-Ago2 polyclonal antibody (ab32381; Abcam) for 3 hours tumbling at 4°C. As a negative control, 30 μg immunoglobulin G from rabbit serum (Sigma) was used. Ago2-RNA complexes were pulled down by incubation with 4 mg protein A Dynabeads (Invitrogen) for 1 hour tumbling at 4°C. Beads were washed three times with lysis buffer and resuspended in RNase-free water. RNA was extracted using UltraPure phenol:chloroform:isoamyl alcohol (25:24:1) (Invitrogen).

For the western blotting, cell lysates were analyzed by gel electrophoresis using 10% SDS-PAGE gels and transferred to nitrocellulose membranes with the iBlot Dry Blotting System (Invitrogen). Membranes were blocked using 3% bovine serum albumin in Tris-buffered saline containing 0.1% Tween-20 for 1 hour, probed with primary antibodies against Ago2 (mouse, 1:1,000; Abnova) or actin (rabbit, 1:10,000; Sigma) overnight at 4°C and subsequently with horseradish peroxidase-conjugated secondary antibodies (1:4,000; Dako) for 1 hour at room temperature. Proteins were visualized using enhanced chemiluminescence (ECL) detection (GE Healthcare, Little Chalfont, United Kingdom) using a FujiFilm Las3000 Imaging System.

### Identification of microRNA interactions by CLASH

miRNA-mRNA interactions were identified in Ago1 CLASH data as described by Helwak *et al*. [31], with some modifications. Briefly, data were downloaded from GEO [GEO: GSE50452], 5′ barcode and 3′ linker sequences were stripped using a homemade perl script and flexbar (settings -ao 4 -m 17), respectively, and reads were mapped to a custom transcriptome database using pblat (settings -stepSize = 5 -tileSize = 11 -minScore = 15). The pblat database contained 2,469 novel candidate microRNAs, 1,223 known human miRNAs from miRBase release 15, and 56,516 protein-coding and non-protein-coding transcripts. Chimeras were called and clustered to identify miRNA-mRNA interactions as described in [31]. miRNA-mRNA binding was analyzed with the hybrid-min program from the UNAFold suite [58]. miRNA-mRNA interactions were classified as in [31].

### Determining the evolutionary origin of known and novel miRNAs

The 46-way vertebrate alignment including human was downloaded from the UCSC browser: [59]. Sequences homologous to human miRNA hairpins (miRBase version 19 and our 2,469 novel candidates) were identified and evaluated with a custom script (available upon demand). A homologous sequence was considered a genuine homologous miRNA hairpin if it folded into a hairpin structure using RNAfold, allowing no bifurcations and minimum 14 nucleotides base paired between the two miRNA strands and a minimum free energy of -14 kcal/mol or lower; and if the seed sequence of either human strand should also have been present in the homologous sequence. The seed sequence was here defined as position two to eight from the 5′ end of the miRNA strand. Once homologs were identified in the 45 species, the evolutionary origin was estimated using a parsimony method. Specifically the branching point was assigned, which minimizes the number of evolutionary gain and losses, at the species level. In the cases of ties, the more recent branching point was chosen. In the cases where a species was not represented in the alignment of a given miRNA, the species was not considered for the estimation of that miRNA. The approach used here is similar to one previously used to estimate evolutionary origin of human miRNA genes [36] and results are comparable.

### Genomic sources of known and novel miRNAs

Known and novel miRNA hairpins (miRBase version 19 and our 2,469 candidates) were mapped to the hg19 genome concatenated with unassembled parts of the human genome (available upon demand) with this command line: bowtie -f -v 1 -a --best --strata. miRNAs were assigned to annotations based on the genome mappings. Annotations used were from GENCODE version 8 [55] supplemented with rRNA and repeat annotations from RepeatMasker hg19 annotations and snoRNA annotations from the UCSC table browser [48]. Annotations were first resolved so that each nucleotide on each strand had exactly one annotation. In cases of nucleotides with more than one annotation, conflicts were resolved using a confidence-based floating hierarchy [60]. The hierarchy used was: mitochondrion > snoRNA > rRNA > tRNA > miscellaneous RNA (GENCODE miscellaneous RNA, snRNA) > long non-coding RNAs (GENCODE long intergenic non-coding RNA, processed_transcript) > pseudogenes > protein_coding > repeats > intergenic. Each read mapping was weighted inversely to the number of genome mappings for the read, for example, a read mapping to two genomic locations got an assigned weight of 0.5. Each mapping was counted towards the annotation of the nucleotide at the center of the mapping.

### Expression of known and novel miRNAs

For the purposes of miRNA specificity, an miRNA was considered to be present in a given dataset if the exact sequence with no sequence mismatches or length variations was present in the dataset. For the purposes of identifying the highest expression of a given miRNA in any of the 94 datasets, the miRNA read counts were noted as the number of exact sequence matches in the dataset. The read counts were normalized to TPM by dividing by the total number of reads in the dataset, after pre-processing, and multiplying by a million. In the differentiation study, TPM values were generated using quantifier.pl from the miRDeep2 suite [25] (version 2.0.0.5) with the following options: quantifier.pl –p hairpins.fa –mature.fa –r reads.fa –c config. Specifically, the normalized columns from the ‘miRNA_expressed_all_samples.csv’ were used. The ‘hairpins.fa’ file and ‘mature.fa file’ contain miRBase version 19 sequences concatenated to our novel candidate sequences.

### Preparation of target-enriched sRNA libraries

Total RNA was extracted from SH-SY5Y differentiated and undifferentiated cells and 1 μg from each extraction was used to prepare indexed libraries according to the Illumina TruSeq Small RNA Sample Preparation protocol with the following modifications: after 5′ Adapter ligation, two aliquots from each ligation reaction product were taken to obtain a total of four indexed libraries; and a total of 15 PCR cycles were performed. The four libraries were concentrated to approximately 30 ng/μl with a vacuum concentrator. Next, the two differentiated libraries were pooled together as well as the two undifferentiated libraries. From each pool, 100 ng were used for hybridization with a custom SureSelect sRNA Target Enrichment library following Agilent’s *SureSelect*^*XT *^*Target Enrichment System for Illumina Paired-End Sequencing Library* (version 1.3.1 [61]), replacing the SureSelect Block mix supplied in the kit with a custom Block mix provided by Agilent. Post-capture PCR amplification was performed using primers complementary to the Illumina P5 and P7 adapter region sequences. Sequencing and data pre-processing were performed as described above.

### Computational analyses of capture data

To estimate the number of detected miRNAs as a function of sequencing depth, the sequencing data from differentiated SH-SH5Y cells, one set with capture and one without, were pre-processed as described in the section ‘sRNA library preparation, sequencing and data pre-processing’ and then shuffled such that the order of reads was random. Using a custom script, the cumulative number of miRNAs detected with each progressive read from 10 thousand to 30 million was noted and plotted. Only known and novel miRNAs, which were targeted in the capture system, were considered, and up to three nucleotides length difference between the read and the miRNA in the 3′ end was tolerated. To estimate profiling depth, the same differentiated datasets were each trimmed to 30 million reads, and the read count of known and novel target miRNA estimated using the quantifier.pl script, using the same parameters as in the previous section. Last, to estimate expression fold-changes of target miRNAs, read counts were again calculated using quantifier.pl. We conservatively only considered miRNAs that had a read count of at least 30 summed between the differentiated and undifferentiated state both with and without the capture. We also discarded miRNAs that had zero read counts in any of the compared conditions. For the remaining miRNAs, read counts were normalized to TPM values by dividing by the total number of reads mapping to miRNAs.

### Population genetics analysis

We obtained SNP data from 1,092 individuals from 14 populations from the 1000 Genomes Project [62]. This dataset contains both low-coverage whole genome sequencing and higher coverage exome sequencing. There are approximately 38 million SNPs that include much more rare variation than the HapMap3 data. We combined allele frequencies from all populations. Importantly, unlike simple measures of SNP occurrence, the derived allele frequency spectrum is robust to mutation biases across the genome, so the patterns we observed should not be due to heterogeneity in the mutation rate. We decided to restrict our analysis to intergenic miRNAs, since the 1000 Genomes Project dataset contains deep exome sequencing data that produces lower allele frequencies in or near exons. This is because a very rare SNP is more likely to be detected with higher sequencing coverage. Initial tests comparing the combined populations with just the African population, which contains the largest amount of diversity of all the populations, showed no significant difference in our results. We thus decided to combine the populations to maximize our statistical power to detect selection. We downloaded RefSeq gene annotations from the UCSC Genome Browser. We searched for a signal of selective constraint on a set of miRNA candidates by comparing the distribution of allele frequencies of SNPs in that set against all SNPs in intergenic regions using a one-sided Wilcoxon test following Akashi [63].

### *In silico* simulation to estimate saturation of miRNA discovery

In the simulation of sequencing depth, the set of pooled reads of the 94 samples was parsed such that for each read it was retained with 10% probability. Following this step, the entire miRNA prediction analysis was performed, from genome mapping to resolving of overlapping hairpins, as described previously in this paper. We noted how many novel miRNA hairpins were reported, and also how many of these hairpins overlapped with our set of high-confidence hairpins (the ones which were supported by at least two types of additional evidence; Additional file 4: Figure S6). This analysis was repeated nine times with probabilities of retaining each read from 10% to 90%. The simulation of datasets was performed in a similar way, except that entire datasets instead of individual reads were retained or discarded. In this case, we ensured that the number of datasets discarded was within one dataset of the mean expected retained number of samples.

### Data access

The data has been submitted to the Sequence Read Archive (SRA) database in study accession number [SRA:SRP028574].

## Competing interests

The authors declare that they have no competing interests.

## Authors’ contributions

MRF performed all analyses except where otherwise noted. EL performed the sRNA capture. AJSH performed Argonaute immunoprecipitation. EL, DB, BK and JG prepared sequencing libraries. AJSH and EMH used qPCR to measure miRNA and mRNA expression, respectively. MB-C performed SH-SY5Y knock-downs and induced cell differentiation. GK performed CLASH data analysis. KCC performed population genetics analyses. EMLeP designed the capture system. EL, AJSH, DB and EM were involved in the study design. MRF and XE designed the study. MRF wrote the paper. All authors discussed the results and commented on the manuscript. All authors read and approved the final manuscript.

## Supplementary Material

Additional file 1: Table S1 Overview of the 94 sRNA-seq datasets that were mined. Click here for file

Additional file 2: Figure S1 Structure and sequence of the 2,469 novel miRNA candidates. Click here for file

Additional file 3: Table S2 Sequences and evidence for the 2,469 novel miRNA candidates. Click here for file

Additional file 4: Figure S2 Example of novel miRNA that is discovered by pooling of sRNA-seq data. **Figure S3.** Representation of novel miRNAs in five human tissues. **Figure S4.** Validation of inferred fold-changes using quantitative PCR. **Figure S5.** Argonaute immunoprecipitation in SH-SY5Y and HeLa cells. **Figure S6.** Stratification of novel miRNAs based on high-throughput evidence. **Figure S7.** Precision of miRNA 5′ end processing. **Figure S8.** sRNA composition of neuroblastoma cells before and after capture. Click here for file

Additional file 5: Table S3 Sequences of known and novel miRNAs ligated and sequenced (CLASH). Click here for file

Additional file 6: Table S4 Structures of known and novel miRNAs ligated and sequenced (CLASH). Click here for file

Additional file 7: Table S5 Sequences of baits used in the custom miRNA capture system. Click here for file

Additional file 8: Table S6 Overview of all samples sequenced. Click here for file
