# Supplementary material for: Evidence for the biogenesis of more than 1,000 novel human microRNAs
Source: Genome Biol. 2014 Apr 7;15(4):R57. doi: 10.1186/gb-2014-15-4-r57 (PMC4054668; doi:10.1186/gb-2014-15-4-r57)
Supplement: Additional file 4: Figure S2 — Example of novel miRNA that is discovered by pooling of sRNA-seq data. Figure S3. Representation of novel miRNAs in five human tissues. Figure S4. Validation of inferred fold-changes using quantitative PCR. Figure S5. Argonaute immunoprecipitation in SH-SY5Y and HeLa cells. Figure S6. Stratification of novel miRNAs based on high-throughput evidence. Figure S7. Precision of miRNA 5′ end processing. Figure S8. sRNA composition of neuroblastoma cells before and after capture. [file gb-2014-15-4-r57-S4.pdf]

SUPPLEMENTARY FIGURES 2-8

Evidence for the biogenesis of more than one thousand novel human miRNAs

Provisional ID	:	hsa_can_406
Score total	:	5.6
Score for star read(s)	:	3.9
Score for read counts	:	0.1
Score for mfe	:	2.2
Score for randfold	:	
Score for cons. seed	:	-0.6
Total read count	:	12
Mature read count	:	10
Loop read count	:	0
Star read count	:	2

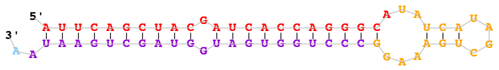

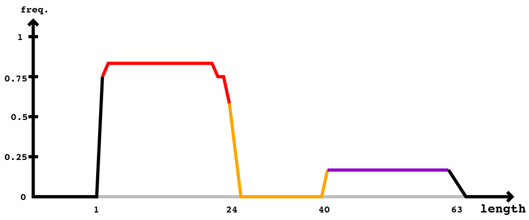


```
           Mature                            Star
5'- ucaauaaaugauucagcuacgaucaccagggcauaucauagcugaaaggcccuggugaugguagcugaauaauuuauugag  -3'   obs
    ucaauaaaugauucagcuacgaucaccagggcauaucauagcugaaaggcccuggugaugguagcugaauaauuuauugag        exp
    (((((((((.((((((((((.(((((((((((...(((....)))...))))))))))).)))))))))).))))))))).   reads   mm   sample
    ..........auucagcuacgaucaccagg...................................................   1       0    NL2
    ...........uucagcuacgaucaccagggca................................................   1       0    j12
    ..........auucagcuacgaucaccagggca................................................   1       0    j19
    .................................................cccuggugaugguagcugaaua..........   1       0    li2
    ..........auucagcuacgaucaccagggc.................................................   1       0    s01
    ..........auucagcuacgaucaccagggc.................................................   1       0    U20
    .................................................cccuggugaugguagcugaaua..........   1       0    s11
    ..........auucagcuacgaucaccagggca................................................   4       0    A54
    ..........auucagcuacgaucaccagggca................................................   1       0    s10
```

Suppl. Figure 2. miRNA hairpin detected through dataset pooling.

Novel miRNA candidate presented in the miRDeep2 format, see figure legend for Suppl. Figure 1. This particular miRNA candidate was only discovered by dataset pooling in which the consistent pattern of the major and minor strands was clear. None of the eight datasets individually provide compelling evidence.

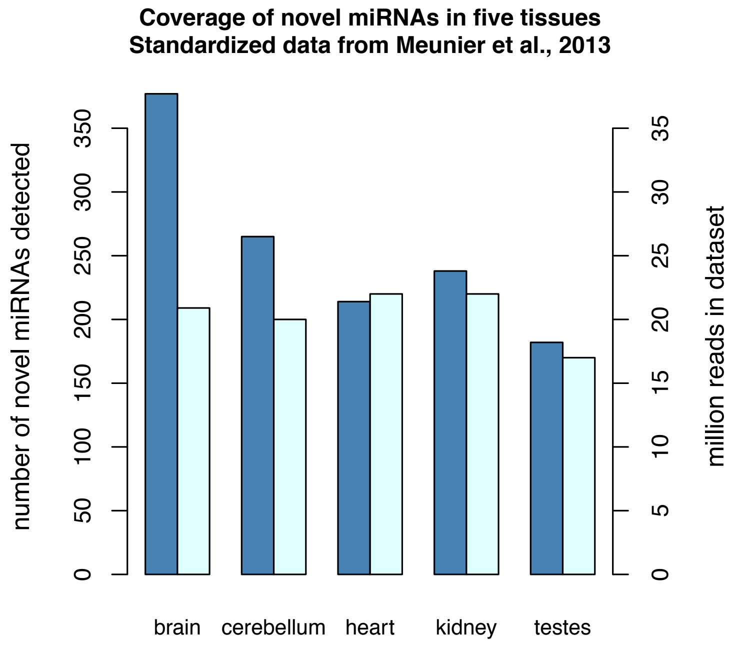

Suppl. Figure 3. Representation of novel miRNAs in five human tissues. The study by Meunier *et al.* (Meunier *et al.*, Genome Research, 2012) is one of the few where multiple human tissues were profiled with sRNA-seq following a standardized protocol within a single laboratory. The dark blue bars shows the number of novel miRNAs that were detected in the dataset (perfect sequence match, but allowing three nucleotides difference in the 3' end), and the light blue bars show the sequence depth of each dataset (in million reads).

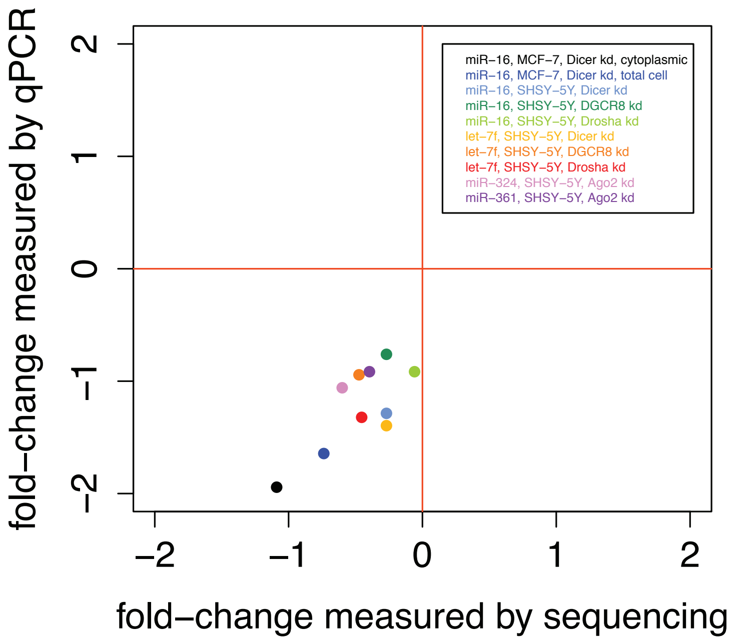

Suppl. Figure 4. miRNA fold-changes measured by sequencing vs. qPCR. The expression fold-change upon Dicer, DGCR8, Drosha or Ago2 knock-down were measured by both high-throughput sequencing and qPCR assay. The fold-changes are plotted in log2, the Pearson's correlation is 0.75. The sequencing appears to systematically slightly underestimate the fold-change relative to the qPCR, suggesting that our conclusions on the effect of the knock-downs are conservative.

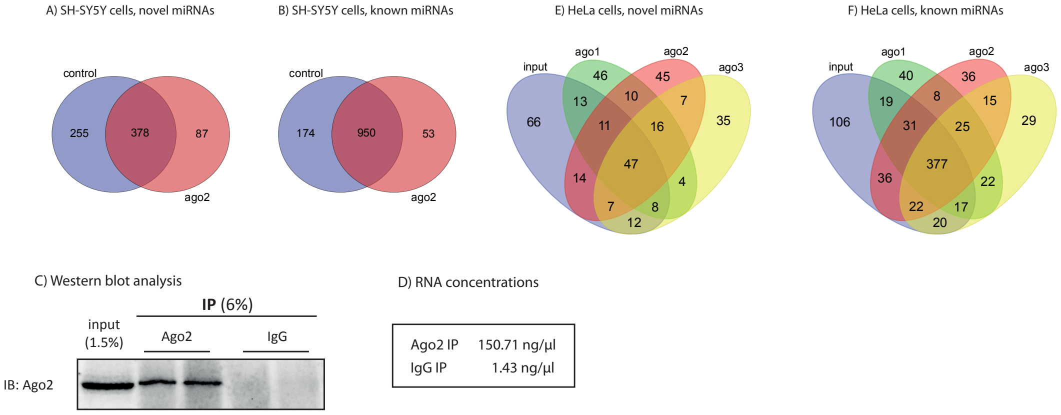

Suppl. Figure 5. Argonaute immunoprecipitation in SH-SY5Y and HeLa cells. (**a-b**) Venn diagrams indicating the number of miRNAs detected in Ago2 immunoprecipitation studies. Endogenous Ago2 was immunoprecipitated from differentiated SH-SY5Y cells and untreated control cells. (**c**) Western blot analysis of Ago2 immunoprecipitation. Ago2 was successfully precipitated (Ago2 IP: lanes 2 and 3), while no binding was detected in the negative control samples (IgG IP: lanes 4 and 5). Lane 1 shows the input sample (1.5% of total lysate used for IP). (**d**) RNA concentrations following IP. (**e-f**) Venn diagrams indicating the number of miRNAs detected in the Ago1-3 IP studies by Dueck *et al.* (Dueck et al., Nucleic Acids Research, 2012).

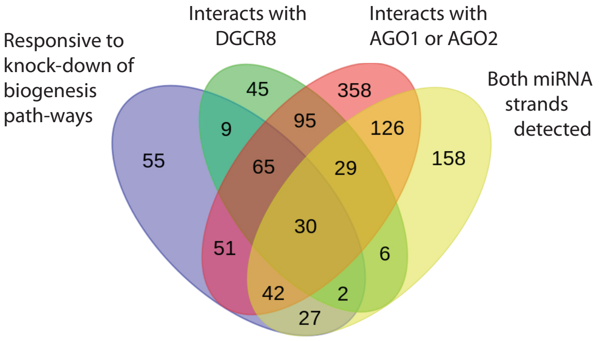

Validated by four methods	30
Validated by three methods	138
Validated by two methods	314
Validated by one method	616
(Validated by min. one method	1098)
No additional validation	1371
Total	2469

Suppl. Figure 6. Compound evidence of novel miRNAs and their validations. All novel miRNA candidates are supported by a stable RNA hairpin structure and a ~22 nucleotide small RNA mapping in accordance to Dicer processing and present in at least two sequencing experiments. This Venn diagrams shows 1,098 novel miRNAs supported by additional information: 1) down-regulation of 30% or more upon knock-down of biogenesis pathways; 2) DGCR8 CLIP-seq reads supporting the exact 5′ end of the mature miRNA; 3) Ago1 or Ago2 CLIP-seq reads supporting the exact 5′ end of the mature miRNA; or 4) the presence of both miRNA strands with exact 2 nucleotides 3′ end overhangs, typical of Dicer processing.

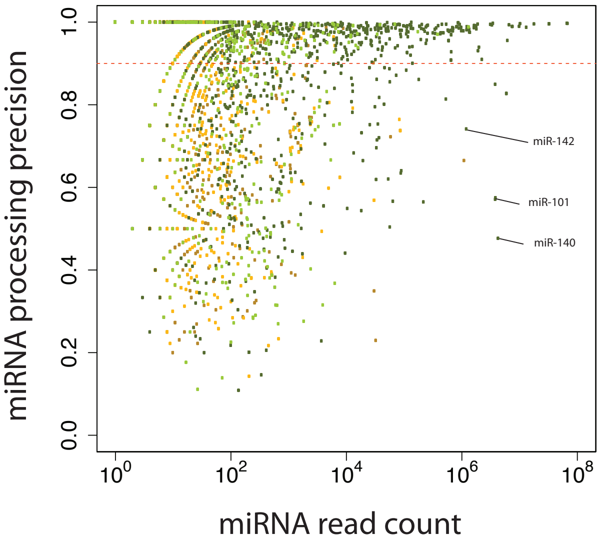

Suppl. Figure 7. Precision of miRNA 5' end processing. For each known and novel miRNAs, the 5' end processing precision is plotted against the read count of the miRNA. The precision is defined as the fraction of mapping reads that correspond to the consensus end position of the sequence. The novel miRNAs are in orange-brown, the known in green. Dark colors indicate conserved miRNAs, light colors non-conserved. Several known miRNAs are conserved and highly expressed, but still display imprecise 5' processing

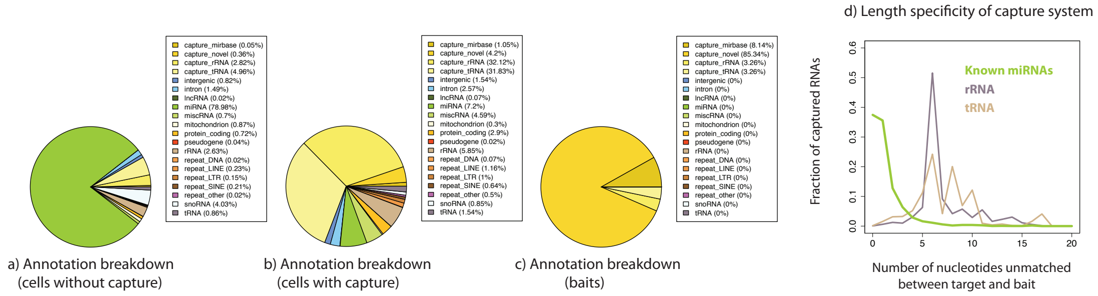

Suppl. Figure 8. Genomic sources of small RNAs with and without SureSelect capture. (**a**) Annotation breakdown of the sequenced small RNAs from differentiated neuroblastoma cells without capture. (**b**) As previous subfigure, but with capture. (**c**) Annotation breakdown of bait sequences included in the capture system. (**d**) Length specificity of the capture system. For each captured RNA we have found the most similar bait sequence, allowing overhangs between the target and the bait, but no sequence mismatches. The number of unmatched (overhanging) nucleotides from both ends were added and are shown. Most captured miRNAs were similar to the corresponding bait sequence, but tRNA and rRNA fragments, being more variable in sequence, typically had six or more mismatched nucleotides. We conclude that the system specifically captures miRNAs, which are of well-defined length, but cannot effectively discriminate between tRNAs and rRNAs which differ in length from the target. The system therefore captures tRNA and rRNA framgnets which are of higher abundance then the low-abundance target sequences. In our next design we will omit tRNA and rRNA controls.
