# Supplementary material for: Evidence for the biogenesis of more than 1,000 novel human microRNAs
Source: Genome Biol. 2014 Apr 7;15(4):R57. doi: 10.1186/gb-2014-15-4-r57 (PMC4054668; doi:10.1186/gb-2014-15-4-r57)
Supplement: Additional file 8: Table S6 — Overview of all samples sequenced. [file gb-2014-15-4-r57-S8.pdf]

Sample name	Cell type	Batch	State	Treatment
Control 1	SH-SY5Y	1	Differentiated	Mock transfection
DGCR8 kd, rep1	SH-SY5Y	1	Differentiated	DGCR8 siRNA
DGCR8 kd, rep2	SH-SY5Y	1	Differentiated	DGCR8 siRNA
Drosha kd, rep1	SH-SY5Y	1	Differentiated	Drosha siRNA
Drosha kd, rep2	SH-SY5Y	1	Differentiated	Drosha siRNA
Control 2	SH-SY5Y	2	Differentiated	Mock transfection
Dicer kd	SH-SY5Y	2	Differentiated	Dicer siRNA
Ago2 kd	SH-SY5Y	2	Differentiated	Ago2 siRNA
Differentiated, part 1	SH-SY5Y	3	Differentiated	None
Differentiated, part 2	SH-SY5Y	3	Differentiated	None
Undifferentiated, part 1	SH-SY5Y	3	Undifferentiated	None
Undifferentiated, part 2	SH-SY5Y	3	Undifferentiated	None
Differentiated capture, part 1	SH-SY5Y	3	Differentiated	SureSelect capture
Differentiated capture, part 2	SH-SY5Y	3	Differentiated	SureSelect capture
Undifferentiated capture, part 1	SH-SY5Y	3	Undifferentiated	SureSelect capture
Undifferentiated capture, part 2	SH-SY5Y	3	Undifferentiated	SureSelect capture

Suppl. Table 6. Overview of the samples sequenced.
