# Supplementary material for: Evidence for the biogenesis of more than 1,000 novel human microRNAs
Source: Genome Biol. 2014 Apr 7;15(4):R57. doi: 10.1186/gb-2014-15-4-r57 (PMC4054668; doi:10.1186/gb-2014-15-4-r57)
Supplement: Additional file 2: Figure S1 — Structure and sequence of the 2,469 novel miRNA candidates. [file gb-2014-15-4-r57-S2.pdf]

Provisional ID	: hsa_can_176	
Score total	:	6
Score for star read(s)	:	3.9
Score for read counts	:	2.2
Score for mfe	:	0.5
Score for randfold	:	
Score for cons. seed	:	-0.6
Total read count	:	16
Mature read count	:	15
Loop read count	:	0
Star read count	:	1

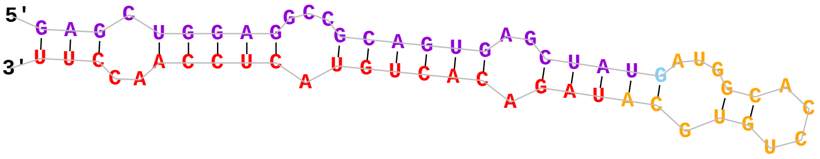

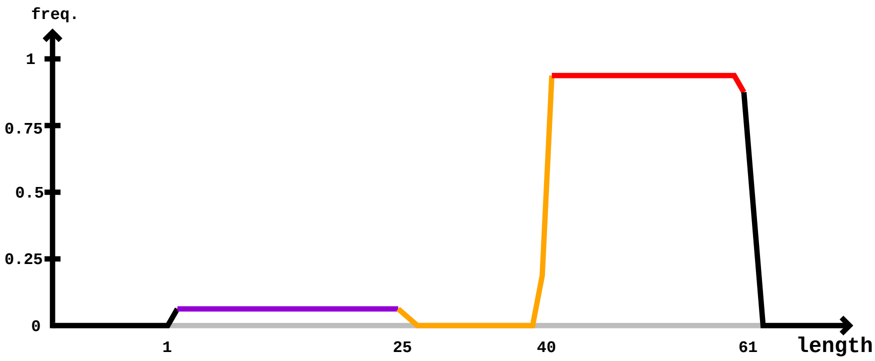

Star Mature

```
5'- cuugagccuaagagcuggaggccgcagugagcuaugauggcaccugugcauagacacuguacuccaaccuugggaacauag  -3'    obs
    cuugagccuaagagcuggaggccgcagugagcuaugauggcaccugugcauagacacuguacuccaaccuugggaacauag        exp
    ..((..((((((.(.(((((...((((((..(((((...((....)).))))).)))))).))))).)))))))..))...    reads     mm        sample
    ..................................................uagacacuguacuccaaccuu..........    3         0         li2

    .................................................auagacacuguacuccaaccuu..........    2         0         li1
    ..................................................uagacacuguacuccaaccu...........    1         0         li1
    ..................................................uagacacuguacuccaaccuu..........    1         0         li1

    .................................................auagacacuguacuccaaccuu..........    1         0         s05

    ..................................................uagacacuguacuccaaccuu..........    5         0         A54

    ..................................................uagacacuguacuccaaccuu..........    1         0         HEK

    ..................................................uagacacuguacuccaaccuu..........    1         0         j15

    ...........gagcuggaggccgcagugagcuau..............................................    1         0         s07
```

Provisional ID	:	hsa_can_279
Score total	:	869.5
Score for star read(s)	:	3.9
Score for read counts	:	860.7
Score for mfe	:	1.9
Score for randfold	:	
Score for cons. seed	:	3
Total read count	:	1700
Mature read count	:	1639
Loop read count	:	0
Star read count	:	61

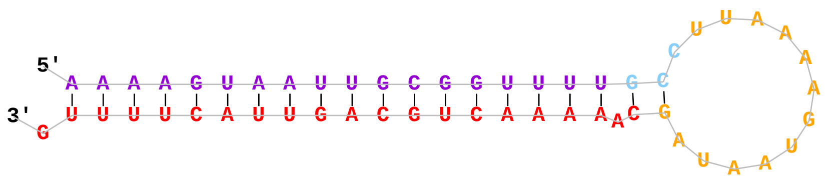

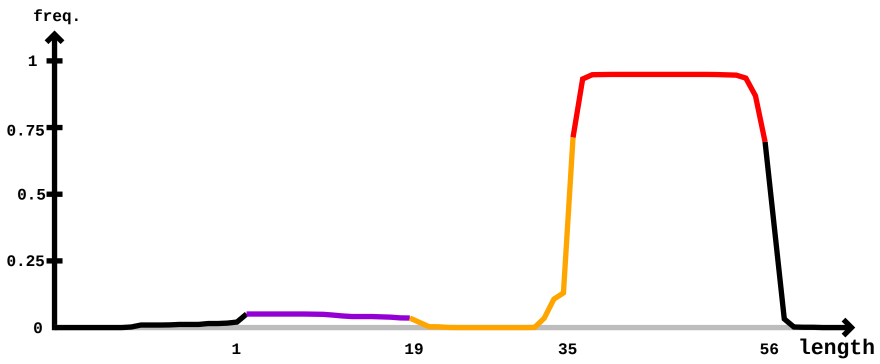

Star Mature

```
5'- aauagaauagguuggugcaaaaguaauugcgguuuugccuuaaaaguaauagcaaaaacugcaguuacuuuugcaccaacc  -3'   obs
    aauagaauagguuggugcaaaaguaauugcgguuuugccuuaaaaguaauagcaaaaacugcaguuacuuuugcaccaacc        exp
    aauagaauagguuggugcaaaaguaauugcgguuuugccuuaaaaguaauagcaaaaacugcaguuacuuuugcaccaacc        known
    .........(((((((((((((((((((((((((((((.............)).)))))))))))))))))))))))))))    reads    mm    sample
    .......uagguuggugcaaaaguaauu.....................................................    2        0     ya1
    ..................aaaaguaauugcgguuuu.............................................    6        0     ya1
    ..................................................agcaaaaacugcaguuacuuuu.........    2        0     ya1
    ....................................................caaaaacugcaguuacuuuu.........    1        0     ya1
    ....................................................caaaaacugcaguuacuuuug........    2        0     ya1
    .....................................................aaaaacugcaguuacuuuu.........    3        0     ya1
    .....................................................aaaaacugcaguuacuuuugc.......    1        0     ya1

    ..................................................agcaaaaacugcaguuacuuu..........    1        0     j26
    ..................................................agcaaaaacugcaguuacuuuu.........    1        0     j26

    .................................................uagcaaaaacugcaguuacuuu..........    1        0     va1
    ..................................................agcaaaaacugcaguuacuuuu.........    9        0     va1
    ...................................................gcaaaaacugcaguuacuuuug........    5        0     va1
    ....................................................caaaaacugcaguuacuuuug........    10       0     va1
    ....................................................caaaaacugcaguuacuuuugc.......    10       0     va1
    .....................................................aaaaacugcaguuacuuuug........    2        0     va1
    .....................................................aaaaacugcaguuacuuuugca......    1        0     va1

    ...........uuggugcaaaaguaauugcggu................................................    1        0     k05
    ....................................................caaaaacugcaguuacuuuug........    1        0     k05
    ....................................................caaaaacugcaguuacuuuugc.......    4        0     k05
    .....................................................aaaaacugcaguuacuuuugc.......    1        0     k05

    ................................................auagcaaaaacugcaguuacuu...........    1        0     s06
    ..................................................agcaaaaacugcaguuacuu...........    1        0     s06

    .................................................uagcaaaaacugcaguuacuu...........    2        0     HeL
    .....................................................aaaaacugcaguuacuuuugc.......    1        0     HeL

    .................................................uagcaaaaacugcaguuacuu...........    1        0     s07

    ....................................................caaaaacugcaguuacuuuu.........    1        0     k08
```

Star **Mature**

aauagaauagguuggugcaaaaguaauugcgguuuugccuuaaaaguaauagcaaaaacugcaguuacuuuugcaccaacc			
....caaaaacugcaguuacuuuug........	4	0	k08
....uagcaaaaacugcaguuacuuu..........	1	0	s10
....caaaaacugcaguuacuuuu.........	1	0	s10
....caaaaacugcaguuacuuuug........	2	0	s10
....caaaaacugcaguuacuuuugc.......	1	0	s10
....aaaaacugcaguuacuuuugc.......	1	0	s10
....aaaaacugcaguuacuuuugca......	1	0	s10
..................aaaaguaauugcgguuuu...	1	0	ta3
..................aaaaguaauugcgguuuu...	16	0	j06
....uagcaaaaacugcaguuacuuu..........	3	0	j10
....uagcaaaaacugcaguuacuuuu.........	1	0	j10
....agcaaaaacugcaguuacuuu..........	2	0	j10
....agcaaaaacugcaguuacuuuu.........	4	0	j10
....gcaaaaacugcaguuacuuuug........	3	0	j10
....caaaaacugcaguuacuuuugc.......	1	0	j10
....caaaaacugcaguuacuuuug........	2	0	j04
....caaaaacugcaguuacuuuug........	1	0	k14
....caaaaacugcaguuacuuuugc.......	3	0	k14
....aaaaacugcaguuacuuuugca......	1	0	k14
.......uagguuggugcaaaaguaauu...	1	0	j02
.......uagguuggugcaaaaguaauug..	1	0	j02
....agcaaaaacugcaguuacuuu..........	3	0	j02
....agcaaaaacugcaguuacuuuu.........	1	0	j02
....gcaaaaacugcaguuacuuuu.........	1	0	j02
....gcaaaaacugcaguuacuuuug........	4	0	j02
....caaaaacugcaguuacuuu..........	2	0	j02
......auagguuggugcaaaaguaa...	1	0	va2
.......uagguuggugcaaaaguaauug..	1	0	va2
..................aaaaguaauugcgguuuu...	1	0	va2
....agcaaaaacugcaguuacuuuu.........	5	0	va2
....gcaaaaacugcaguuacuuuug........	1	0	va2
....caaaaacugcaguuacuuuug........	1	0	va2
....caaaaacugcaguuacuuuugc.......	2	0	va2
....uagcaaaaacugcaguuacuuu..........	1	0	j31
....agcaaaaacugcaguuacuuuu.........	2	0	j31
....gcaaaaacugcaguuacuuuug........	1	0	j31
....caaaaacugcaguuacuuuug........	1	0	j31
....caaaaacugcaguuacuuuugc.......	1	0	j31
....aaaaacugcaguuacuuuugca......	1	0	j31
.................caaaaguaauugcgguuuu...	1	0	j27
..................aaaaguaauugcgguuuug..	1	0	j27
....caaaaacugcaguuacuu...........	2	0	H52
....caaaaacugcaguuacuuuu.........	7	0	H52
....caaaaacugcaguuacuuuug........	31	0	H52
....caaaaacugcaguuacuuuugc.......	78	0	H52
....aaaaacugcaguuacuuuu.........	5	0	H52
....aaaaacugcaguuacuuuug........	6	0	H52
....aaaaacugcaguuacuuuugc.......	13	0	H52
....aaaaacugcaguuacuuuugca......	12	0	H52
....aaaacugcaguuacuuuugc.......	4	0	H52
....gcaaaaacugcaguuacuuuug........	13	0	j05
....uagcaaaaacugcaguuacuuu..........	2	0	s03
....agcaaaaacugcaguuacuuu..........	1	0	s03
....uagcaaaaacugcaguuacuu...........	1	0	j22
....agcaaaaacugcaguuacuuuu.........	4	0	j22
....gcaaaaacugcaguuacuuuug........	2	0	j22

Star Mature

```
aauagaauagguuggugcaaaaguaauugcgguuuugccuuaaaaguaauagcaaaaacugcaguuacuuuugcaccaacc
....................................................caaaaacugcaguuacuuuu.........   1     0     j22
....................................................caaaaacugcaguuacuuuug........   1     0     j22
....................................................caaaaacugcaguuacuuuugc.......   2     0     j22

.......uagguuggugcaaaaguaauug....................................................   1     0     k09
....................................................caaaaacugcaguuacuuuug........   2     0     k09
....................................................caaaaacugcaguuacuuuugc.......   5     0     k09
.....................................................aaaaacugcaguuacuuuug........   1     0     k09
........................................................aacugcaguuacuuuugcacca...   1     0     k09

..................aaaaguaauugcgguuuu.............................................   1     0     B14
..................aaaaguaauugcgguuuugcc..........................................   1     0     B14
.................................................uagcaaaaacugcaguuacu............   1     0     B14
.................................................uagcaaaaacugcaguuacuuu..........   1     0     B14

....................................................caaaaacugcaguuacuuuug........   3     0     j18

.................................................uagcaaaaacugcaguuacuuuug........   1     0     j09
..................................................agcaaaaacugcaguuacuuuu.........   1     0     j09
....................................................caaaaacugcaguuacuuuugc.......   1     0     j09

.................................................uagcaaaaacugcaguuacuuu..........   1     0     ku3
..................................................agcaaaaacugcaguuacuuu..........   1     0     ku3
..................................................agcaaaaacugcaguuacuuuu.........   1     0     ku3
....................................................caaaaacugcaguuacuuuug........   1     0     ku3

.................................................uagcaaaaacugcaguuacuuu..........   1     0     j16
..................................................agcaaaaacugcaguuacuuuu.........   1     0     j16
....................................................caaaaacugcaguuacuuuu.........   2     0     j16
....................................................caaaaacugcaguuacuuuug........   4     0     j16
.....................................................aaaaacugcaguuacuuuu.........   1     0     j16
.....................................................aaaaacugcaguuacuuuug........   6     0     j16
......................................................aaaacugcaguuacuuuug........   3     0     j16

..................................................agcaaaaacugcaguuacuuu..........   1     0     ku6

.................................................uagcaaaaacugcaguuacuuu..........   2     0     j11
..................................................agcaaaaacugcaguuacuuu..........   1     0     j11
..................................................agcaaaaacugcaguuacuuuu.........   5     0     j11

..................................................agcaaaaacugcaguuacuuuu.........   1     0     s12
....................................................caaaaacugcaguuacuuuugc.......   1     0     s12

.......uagguuggugcaaaaguaauu.....................................................   1     0     ya3
................gcaaaaguaauugcgguuuu.............................................   3     0     ya3
..................aaaaguaauugcgguuuu.............................................   9     0     ya3
..................aaaaguaauugcgguuuug............................................   3     0     ya3
..................................................agcaaaaacugcaguuacuuuu.........   3     0     ya3
....................................................caaaaacugcaguuacuuu..........   2     0     ya3
....................................................caaaaacugcaguuacuuuu.........   3     0     ya3
....................................................caaaaacugcaguuacuuuug........   1     0     ya3
.....................................................aaaaacugcaguuacuuuu.........   2     0     ya3
.....................................................aaaaacugcaguuacuuuug........   1     0     ya3
......................................................aaaacugcaguuacuuuu.........   1     0     ya3

..................................................agcaaaaacugcaguuacuuuu.........   2     0     la1
...................................................gcaaaaacugcaguuacuuuu.........   1     0     la1

..............gugcaaaaguaauugcgguuu..............................................   1     0     HEK
.................caaaaguaauugcgguuuugcc..........................................   1     0     HEK
.................................................uagcaaaaacugcaguuacuu...........   2     0     HEK
.................................................uagcaaaaacugcaguuacuuu..........   3     0     HEK
..................................................agcaaaaacugcaguuacuuuu.........   4     0     HEK
....................................................caaaaacugcaguuacuuuugc.......   1     0     HEK

....................................................caaaaacugcaguuacu............   1     0     j15
....................................................caaaaacugcaguuacuuu..........   28    0     j15
....................................................caaaaacugcaguuacuuuu.........   55    0     j15
....................................................caaaaacugcaguuacuuuug........   246   0     j15
....................................................caaaaacugcaguuacuuuugc.......   20    0     j15
```

Star **Mature**

```
aauagaauagguuggugcaaaaguaauugcgguuuugccuuaaaaguaauagcaaaaacugcaguuacuuuugcaccaacc
....................................................caaaaacugcaguuacuuuugcacca...   1     0     j15
.....................................................aaaaacugcaguuacuuu..........   3     0     j15
.....................................................aaaaacugcaguuacuuuu.........   24    0     j15
.....................................................aaaaacugcaguuacuuuug........   107   0     j15
.....................................................aaaaacugcaguuacuuuugc.......   2     0     j15
.....................................................aaaaacugcaguuacuuuugca......   14    0     j15
......................................................aaaacugcaguuacuuuug........   5     0     j15
......................................................aaaacugcaguuacuuuugca......   3     0     j15

..................................................agcaaaaacugcaguuacuuuu.........   1     0     j17
....................................................caaaaacugcaguuacuuuu.........   1     0     j17
....................................................caaaaacugcaguuacuuuug........   4     0     j17
....................................................caaaaacugcaguuacuuuugc.......   4     0     j17
.....................................................aaaaacugcaguuacuuuug........   4     0     j17
.....................................................aaaaacugcaguuacuuuugc.......   1     0     j17

...................................................gcaaaaacugcaguuacuuuug........   1     0     la2

..................aaaaguaauugcgguuuu.............................................   1     0     j12
..................................................agcaaaaacugcaguuacuuuu.........   2     0     j12
....................................................caaaaacugcaguuacuuuug........   1     0     j12
.....................................................aaaaacugcaguuacuuuug........   1     0     j12
......................................................aaaacugcaguuacuuuug........   1     0     j12

.................................................uagcaaaaacugcaguuacuuuu.........   2     0     va4
..................................................agcaaaaacugcaguuacuuuu.........   4     0     va4

.................................................uagcaaaaacugcaguuacuuu..........   1     0     va3
..................................................agcaaaaacugcaguuacuuuu.........   2     0     va3

.......uagguuggugcaaaaguaau......................................................   4     0     ya4
..................aaaaguaauugcgguuuu.............................................   2     0     ya4
...................................................gcaaaaacugcaguuacuuuug........   2     0     ya4
....................................................caaaaacugcaguuacuuuu.........   10    0     ya4
....................................................caaaaacugcaguuacuuuug........   1     0     ya4
.....................................................aaaaacugcaguuacuuuug........   4     0     ya4

.................caaaaguaauugcgguuuugccu.........................................   1     0     SW4
..................................................agcaaaaacugcaguuacuuuu.........   1     0     SW4
......................................................aaaacugcaguuacuuuu.........   2     0     SW4

.................................................uagcaaaaacugcaguuacuuu..........   1     0     ku1
..................................................agcaaaaacugcaguuacuuu..........   1     0     ku1
...................................................gcaaaaacugcaguuacuuu..........   1     0     ku1
....................................................caaaaacugcaguuacuuuug........   2     0     ku1
.....................................................aaaaacugcaguuacuuuu.........   1     0     ku1

..................aaaaguaauugcgguuuu.............................................   3     0     j23
.................................................uagcaaaaacugcaguuacuu...........   2     0     j23
.................................................uagcaaaaacugcaguuacuuu..........   1     0     j23
.................................................uagcaaaaacugcaguuacuuuu.........   1     0     j23
..................................................agcaaaaacugcaguuacuu...........   1     0     j23
..................................................agcaaaaacugcaguuacuuu..........   3     0     j23
..................................................agcaaaaacugcaguuacuuuu.........   14    0     j23
...................................................gcaaaaacugcaguuacuuuug........   1     0     j23
....................................................caaaaacugcaguuacuuuu.........   6     0     j23
....................................................caaaaacugcaguuacuuuug........   34    0     j23
....................................................caaaaacugcaguuacuuuugc.......   11    0     j23
.....................................................aaaaacugcaguuacuuuu.........   2     0     j23
.....................................................aaaaacugcaguuacuuuug........   17    0     j23
.....................................................aaaaacugcaguuacuuuugc.......   5     0     j23
.....................................................aaaaacugcaguuacuuuugca......   4     0     j23
......................................................aaaacugcaguuacuuuugc.......   1     0     j23

.................................................uagcaaaaacugcaguuacuu...........   1     0     s05
.................................................uagcaaaaacugcaguuacuuu..........   1     0     s05
..................................................agcaaaaacugcaguuacuuu..........   2     0     s05

..................................................agcaaaaacugcaguuacuuu..........   1     0     j24

...........uuggugcaaaaguaauugcggu................................................   1     0     k11
```

```
                    Star                              Mature
aauagaauagguuggugcaaaaguaauugcgguuuugccuuaaaaguaauagcaaaaacugcaguuacuuuugcaccaacc
..................................................agcaaaaacugcaguuacuuu..........    2    0    k11
....................................................caaaaacugcaguuacuuuug........    1    0    k11
....................................................caaaaacugcaguuacuuuugc.......    2    0    k11
.....................................................aaaaacugcaguuacuuuu.........    1    0    k11

.................................................uagcaaaaacugcaguuacuu...........    1    0    s09
..................................................agcaaaaacugcaguuacuuuu.........    1    0    s09

......auagguuggugcaaaagua........................................................    1    0    NL2
..................aaaaguaauugcgguuuu.............................................    1    0    NL2
....................................................caaaaacugcaguuacuuu..........    18   0    NL2
....................................................caaaaacugcaguuacuuuug........    10   0    NL2
....................................................caaaaacugcaguuacuuuugc.......    33   0    NL2
.....................................................aaaaacugcaguuacuuu..........    13   0    NL2
.....................................................aaaaacugcaguuacuuuug........    14   0    NL2
.....................................................aaaaacugcaguuacuuuugc.......    10   0    NL2
.....................................................aaaaacugcaguuacuuuugca......    1    0    NL2
......................................................aaaacugcaguuacuuuugc.......    1    0    NL2

....................................................caaaaacugcaguuacuu...........    1    0    k10
....................................................caaaaacugcaguuacuuu..........    1    0    k10
....................................................caaaaacugcaguuacuuuug........    3    0    k10
....................................................caaaaacugcaguuacuuuugc.......    5    0    k10

.................................................uagcaaaaacugcaguuacuu...........    1    0    j13
..................................................agcaaaaacugcaguuacuu...........    3    0    j13
..................................................agcaaaaacugcaguuacuuuu.........    2    0    j13
...................................................gcaaaaacugcaguuacuuuug........    1    0    j13
.....................................................aaaaacugcaguuacuuuug........    1    0    j13

.................caaaaguaauugcgguuuug............................................    1    0    j19
.................................................uagcaaaaacugcaguuacuu...........    1    0    j19
..................................................agcaaaaacugcaguuacuuuu.........    2    0    j19
....................................................caaaaacugcaguuacu............    2    0    j19
....................................................caaaaacugcaguuacuuu..........    4    0    j19
....................................................caaaaacugcaguuacuuuug........    42   0    j19
....................................................caaaaacugcaguuacuuuugc.......    2    0    j19
.....................................................aaaaacugcaguuacuuu..........    1    0    j19
.....................................................aaaaacugcaguuacuuuug........    6    0    j19

..................................................agcaaaaacugcaguuacuu...........    1    0    j03

....................................................caaaaacugcaguuacuuuug........    1    0    j25
.....................................................aaaaacugcaguuacuuuug........    1    0    j25

.................................................uagcaaaaacugcaguuacuu...........    2    0    k01
....................................................caaaaacugcaguuacuuuug........    4    0    k01
....................................................caaaaacugcaguuacuuuugc.......    4    0    k01
.....................................................aaaaacugcaguuacuuuug........    2    0    k01

....................................................caaaaacugcaguuacuuuugc.......    3    0    k04
.....................................................aaaaacugcaguuacuuuug........    1    0    k04
.....................................................aaaaacugcaguuacuuuugca......    1    0    k04

.................................................uagcaaaaacugcaguuacuu...........    1    0    s01
..................................................agcaaaaacugcaguuacuuuu.........    2    0    s01
..................................................agcaaaaacugcaguuacuuuug........    1    0    s01
.....................................................aaaaacugcaguuacuu...........    1    0    s01

..................................................agcaaaaacugcaguuacuu...........    1    0    per
....................................................caaaaacugcaguuacuuuug........    1    0    per

.......uagguuggugcaaaaguaauug....................................................    1    0    ku2
.................................................uagcaaaaacugcaguuacuu...........    2    0    ku2
..................................................agcaaaaacugcaguuacuu...........    1    0    ku2
..................................................agcaaaaacugcaguuacuuuu.........    1    0    ku2

.................................................uagcaaaaacugcagua...............    1    0    U20
.................................................uagcaaaaacugcaguuac.............    2    0    U20
.................................................uagcaaaaacugcaguuacu............    3    0    U20
```

Star Mature

```
aauagaauagguuggugcaaaaguaauugcgguuuugccuuaaaaguaauagcaaaaacugcaguuacuuuugcaccaacc
.................................................uagcaaaaacugcaguuacuuu..........  2   0   U20
..................................................agcaaaaacugcaguuacuu...........  1   0   U20
..................................................agcaaaaacugcaguuacuuuu.........  3   0   U20
...................................................gcaaaaacugcaguuacuuuug........  2   0   U20
....................................................caaaaacugcaguuacuuuugc.......  1   0   U20

.................................................uagcaaaaacugcaguuacuuu..........  2   0   j21
..................................................agcaaaaacugcaguuacuuuu.........  1   0   j21
....................................................caaaaacugcaguuacuu...........  1   0   j21
....................................................caaaaacugcaguuacuuuu.........  2   0   j21
....................................................caaaaacugcaguuacuuuug........  38  0   j21
....................................................caaaaacugcaguuacuuuugc.......  37  0   j21
.....................................................aaaaacugcaguuacuuuu.........  1   0   j21
.....................................................aaaaacugcaguuacuuuug........  8   0   j21
.....................................................aaaaacugcaguuacuuuugc.......  11  0   j21
.....................................................aaaaacugcaguuacuuuugca......  5   0   j21
......................................................aaaacugcaguuacuuuugca......  1   0   j21
......................................................aaaacugcaguuacuuuugcac.....  1   0   j21


....................................................caaaaacugcaguuacuuuugc.......  1   0   s11

..................................................agcaaaaacugcaguuacuuu..........  1   0   j07

....................................................caaaaacugcaguuacuuu..........  1   0   k02
....................................................caaaaacugcaguuacuuuug........  3   0   k02
....................................................caaaaacugcaguuacuuuugc.......  4   0   k02
.....................................................aaaaacugcaguuacuuuugc.......  1   0   k02

.................................................uagcaaaaacugcaguuacuuu..........  2   0   j14
..................................................agcaaaaacugcaguuacuuuu.........  2   0   j14
...................................................gcaaaaacugcaguuacuuuug........  1   0   j14
....................................................caaaaacugcaguuacuuuug........  2   0   j14
....................................................caaaaacugcaguuacuuuugc.......  1   0   j14
.....................................................aaaaacugcaguuacuuuugc.......  1   0   j14

......auagguuggugcaaaaguaauu.....................................................  1   0   j28
.......uagguuggugcaaaaguaauu.....................................................  1   0   j28
..................aaaaguaauugcgguuuu.............................................  3   0   j28
.....................................................aaaaacugcaguuacuuu..........  2   0   j28
.....................................................aaaaacugcaguuacuuuug........  1   0   j28

..................................................agcaaaaacugcaguuacuu...........  2   0   s08
..................................................agcaaaaacugcaguuacuuuu.........  1   0   s08

.................................................uagcaaaaacugcaguuacuuu..........  3   0   DLD

......auagguuggugcaaaaguaau......................................................  1   0   k03
....................................................caaaaacugcaguuacuuuug........  3   0   k03
....................................................caaaaacugcaguuacuuuugc.......  1   0   k03
.....................................................aaaaacugcaguuacuuuugc.......  1   0   k03

....................................................caaaaacugcaguuacuuu..........  5   0   NL3
....................................................caaaaacugcaguuacuuuu.........  15  0   NL3
....................................................caaaaacugcaguuacuuuug........  17  0   NL3
....................................................caaaaacugcaguuacuuuugc.......  27  0   NL3
.....................................................aaaaacugcaguuacuu...........  2   0   NL3
.....................................................aaaaacugcaguuacuuuu.........  2   0   NL3
.....................................................aaaaacugcaguuacuuuug........  5   0   NL3
.....................................................aaaaacugcaguuacuuuugc.......  9   0   NL3
.....................................................aaaaacugcaguuacuuuugca......  1   0   NL3
......................................................aaaacugcaguuacuuuugc.......  2   0   NL3

.................caaaaguaauugcgguuuu.............................................  1   0   ya2
..................aaaaguaauugcgguuuu.............................................  1   0   ya2
..................aaaaguaauugcgguuuug............................................  1   0   ya2
..................................................agcaaaaacugcaguuacuuuu.........  3   0   ya2
...................................................gcaaaaacugcaguuacuuuu.........  1   0   ya2
....................................................caaaaacugcaguuacuuuu.........  9   0   ya2
....................................................caaaaacugcaguuacuuuug........  3   0   ya2
```

Star **Mature**

```
aauagaauagguuggugcaaaaguaauugcgguuuugccuuaaaaguaauagcaaaaacugcaguuacuuuugcaccaacc
....................................................caaaaacugcaguuacuuuugc.......    1    0    ya2
.....................................................aaaaacugcaguuacuuuug........    1    0    ya2

....................................................caaaaacugcaguuacuuuu.........    1    0    k06
....................................................caaaaacugcaguuacuuuug........    6    0    k06
....................................................caaaaacugcaguuacuuuugc.......    3    0    k06
.....................................................aaaaacugcaguuacuuuug........    1    0    k06
.....................................................aaaaacugcaguuacuuuugc.......    1    0    k06
.......................................................aaacugcaguuacuuuug........    1    0    k06

..........guuggugcaaaaguaauugcgg.................................................    1    0    sh2
...........uuggugcaaaaguaauugcgg.................................................    1    0    sh2
.................caaaaguaauugcgguuuugc...........................................    1    0    sh2
..................aaaaguaauugcgguuuu.............................................    1    0    sh2
....................................................caaaaacugcaguuacuuuugc.......    6    0    sh2
.....................................................aaaaacugcaguuacuuuugca......    2    0    sh2

..................................................agcaaaaacugcaguuacuuuu.........    1    0    j29
...................................................gcaaaaacugcaguuacuuuug........    1    0    j29

....................................................caaaaacugcaguuacuuuug........    2    0    j01

.................caaaaguaauugcgguuuugcc..........................................    1    0    MCF
.................................................uagcaaaaacugcaguuacuu...........    1    0    MCF
....................................................caaaaacugcaguuacuuuug........    1    0    MCF
....................................................caaaaacugcaguuacuuuugc.......    1    0    MCF

....................................................caaaaacugcaguuacuuuu.........    1    0    NL1
....................................................caaaaacugcaguuacuuuug........    6    0    NL1
....................................................caaaaacugcaguuacuuuugc.......    9    0    NL1
.....................................................aaaaacugcaguuacuuuu.........    4    0    NL1
.....................................................aaaaacugcaguuacuuuug........    2    0    NL1
.....................................................aaaaacugcaguuacuuuugc.......    2    0    NL1
.....................................................aaaaacugcaguuacuuuugca......    1    0    NL1

..............gugcaaaaguaauugcgguu...............................................    1    0    MBM
..................................................agcaaaaacugcaguuacuu...........    2    0    MBM
....................................................caaaaacugcaguuacuuuug........    2    0    MBM
....................................................caaaaacugcaguuacuuuugc.......    21   0    MBM
.....................................................aaaaacugcaguuacuuuu.........    1    0    MBM
.....................................................aaaaacugcaguuacuuuug........    1    0    MBM
.....................................................aaaaacugcaguuacuuuugc.......    2    0    MBM
.....................................................aaaaacugcaguuacuuuugca......    2    0    MBM
......................................................aaaacugcaguuacuuuugc.......    3    0    MBM
......................................................aaaacugcaguuacuuuugcac.....    1    0    MBM

....................................................caaaaacugcaguuacuuuu.........    1    0    k07
....................................................caaaaacugcaguuacuuuug........    1    0    k07

....................................................caaaaacugcaguuacuuuugc.......    9    0    li1
....................................................caaaaacugcaguuacuuuugca......    1    0    li1

.................................................uagcaaaaacugcaguuacu............    1    0    k13
..................................................agcaaaaacugcaguuacuuuu.........    1    0    k13
....................................................caaaaacugcaguuacuuuug........    3    0    k13
....................................................caaaaacugcaguuacuuuugc.......    5    0    k13
.....................................................aaaaacugcaguuacuuuu.........    1    0    k13
.....................................................aaaaacugcaguuacuuuug........    2    0    k13

.................................................uagcaaaaacugcaguuacuu...........    1    0    j20
.................................................uagcaaaaacugcaguuacuuu..........    1    0    j20
..................................................agcaaaaacugcaguuacuuu..........    1    0    j20
..................................................agcaaaaacugcaguuacuuuu.........    3    0    j20
.....................................................aaaaacugcaguuacuuuug........    1    0    j20

..............gugcaaaaguaauugcgguu...............................................    4    0    A54
..................aaaaguaauugcgguuuugcc..........................................    1    0    A54

....................................................caaaaacugcaguuacuuuug........    18   0    j30
....................................................caaaaacugcaguuacuuuugc.......    10   0    j30
```

Star **Mature**

```
aauagaauagguuggugcaaaaguaauugcgguuuugccuuaaaaguaauagcaaaaacugcaguuacuuuugcaccaacc
.....................................................aaaaacugcaguuacuuuug........    8    0    j30
.....................................................aaaaacugcaguuacuuuugc.......    3    0    j30
.....................................................aaaaacugcaguuacuuuugca......    2    0    j30

..................aaaaguaauugcgguuuu.............................................    1    0    k12
....................................................caaaaacugcaguuacuuuu.........    2    0    k12
....................................................caaaaacugcaguuacuuuug........    3    0    k12
....................................................caaaaacugcaguuacuuuugc.......    1    0    k12
.....................................................aaaaacugcaguuacuuuu.........    1    0    k12
.....................................................aaaaacugcaguuacuuuug........    1    0    k12
.....................................................aaaaacugcaguuacuuuugc.......    1    0    k12
```

Provisional ID	:	hsa_can_356
Score total	:	17.5
Score for star read(s)	:	3.9
Score for read counts	:	12.4
Score for mfe	:	1.9
Score for randfold	:	
Score for cons. seed	:	-0.6
Total read count	:	36
Mature read count	:	32
Loop read count	:	0
Star read count	:	4

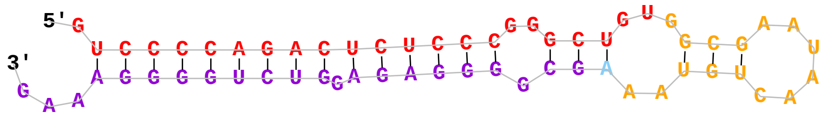

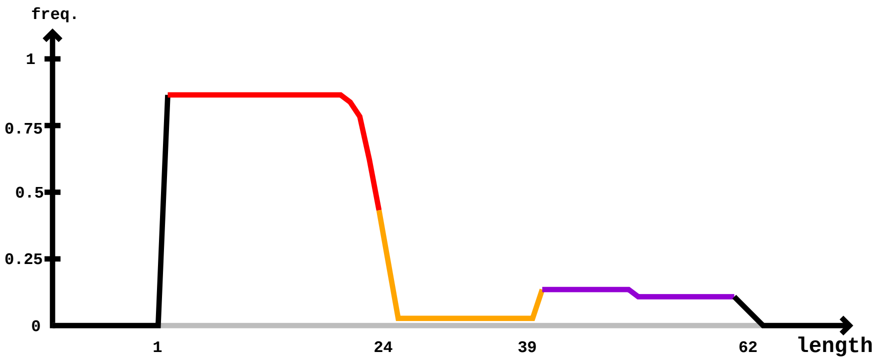

Mature **Star**

```
5'- ccaggcggccguccccagacucucccgggcuguggcgaauaacuguaaagcggggagaggucuggggaaagcugggguagg  -3'   obs
    ccaggcggccguccccagacucucccgggcuguggcgaauaacuguaaagcggggagaggucuggggaaagcugggguagg        exp
    ((...((((..((((((((((((((..(((...(((......)))..))).)))))).)))))))))..)))).))....   reads   mm   sample
    ..........guccccagacucucccgggcu..................................................   1       0    s08
    ..........guccccagacucucccgggcugu................................................   5       0    s08

    ..........guccccagacucucccgggcugu................................................   2       0    s04

    ..........guccccagacucucccgggcug.................................................   1       0    j11

    ..........guccccagacucucccggg....................................................   1       0    ta1

    ..........guccccagacucucccgggcug.................................................   1       0    s09

    ..........guccccagacucucccgggcugu................................................   1       0    sh2

    ..........guccccagacucucccgggcu..................................................   1       0    la1

    ..........guccccagacucucccgggcug.................................................   1       0    j15
    ..........guccccagacucucccgggcugu................................................   2       0    j15
    .................................................gcggggagaggucuggggaaag..........   1       0    j15

    ..........guccccagacucucccgggcu..................................................   1       0    j17

    ..........guccccagacucucccgggc...................................................   1       0    s10
    ..........guccccagacucucccgggcug.................................................   1       0    s10

    ..........guccccagacucucccgggcu..................................................   1       0    la2

    ..........guccccagacucucccgggcu..................................................   1       0    j13

    .................................................gcggggagaggucuggggaaag..........   1       0    j19

    ..........guccccagacucucccgggcug.................................................   2       0    s01
    ..........guccccagacucucccgggcugu................................................   2       0    s01
    .................................................gcggggagaggucuggggaaa...........   2       0    s01
```

Mature Star

```
ccaggcggccguccccagacucucccgggcuguggcgaauaacuguaaagcggggagaggucuggggaaagcuggggguagg

................................uggcgaauaacuguaaagcggggagag......................	1	0	j10

..........guccccagacucucccgggcu..................................................	1	0	j21

..........guccccagacucucccgggc...................................................	1	0	j31

..........guccccagacucucccgggcugu................................................	2	0	va3

..........guccccagacucucccgggcug.................................................	2	0	j28
..........guccccagacucucccgggcugu................................................	1	0	j28
```

Provisional ID	:	hsa_can_406
Score total	:	5.6
Score for star read(s)	:	3.9
Score for read counts	:	0.1
Score for mfe	:	2.2
Score for randfold	:	
Score for cons. seed	:	-0.6
Total read count	:	12
Mature read count	:	10
Loop read count	:	0
Star read count	:	2

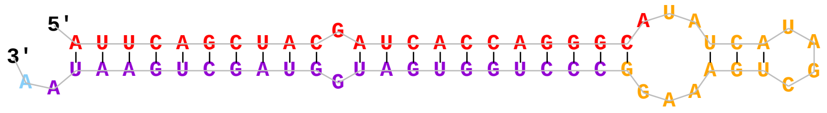

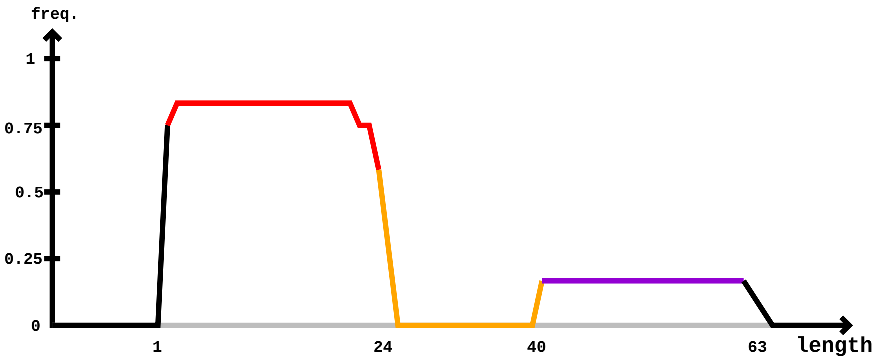

Mature **Star**

```
5'- ucaauaaaugauucagcuacgaucaccagggcauaucauagcugaaaggcccuggugaugguagcugaauaauuuauugag   -3'   obs
    ucaauaaaugauucagcuacgaucaccagggcauaucauagcugaaaggcccuggugaugguagcugaauaauuuauugag         exp
    (((((((((.((((((((((.(((((((((((...(((....)))...))))))))))).)))))))))).))))))))).   reads   mm   sample
    ..........auucagcuacgaucaccagg...................................................   1       0    NL2

    ...........uucagcuacgaucaccagggca................................................   1       0    j12

    ..........auucagcuacgaucaccagggca................................................   1       0    j19

    .................................................cccuggugaugguagcugaaua..........   1       0    li2

    ..........auucagcuacgaucaccagggc.................................................   1       0    s01

    ..........auucagcuacgaucaccagggc.................................................   1       0    U20

    .................................................cccuggugaugguagcugaaua..........   1       0    s11

    ..........auucagcuacgaucaccagggca................................................   4       0    A54

    ..........auucagcuacgaucaccagggca................................................   1       0    s10
```

Provisional ID : hsa_can_409
Score total : 16.2
Score for star read(s) : 3.9
Score for read counts : 10.8
Score for mfe : 2.1
Score for randfold :
Score for cons. seed : -0.6
Total read count : 33
Mature read count : 23
Loop read count : 0
Star read count : 10

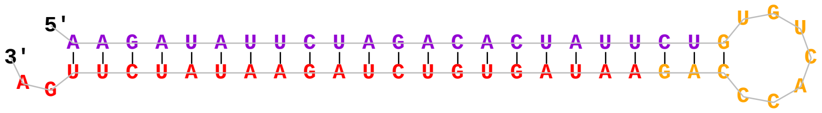

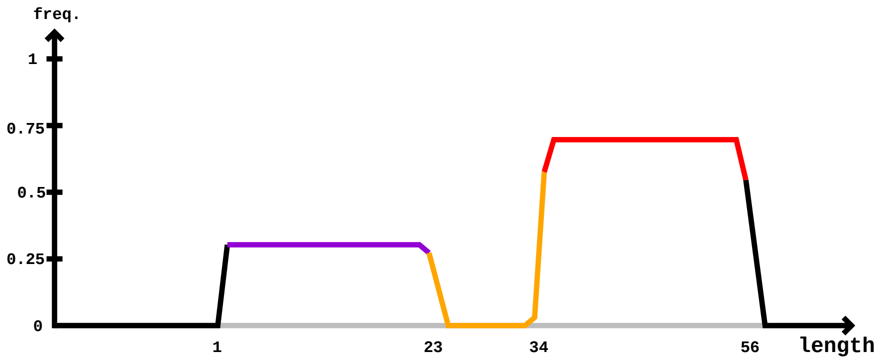


```
                Star                            Mature
5'- uguuugaauuacuguuaagauauucuagacacuauucugugucacccagaauagugucuagaauaucuugacaauaauuca  -3'   obs
    uguuugaauuacuguuaagauauucuagacacuauucugugucacccagaauagugucuagaauaucuugacaauaauuca        exp
    ....(((((((.(((((((((((((((((((((((((((.......))))))))))))))))))))))))))).)))))))    reads   mm   sample
    .................................................aauagugucuagaauaucuuga..........    2       0    j23

    .................................................aauagugucuagaauaucuug...........    1       0    j19
    .................................................aauagugucuagaauaucuuga..........    1       0    j19

    .................................................aauagugucuagaauaucuuga..........    2       0    ku3

    .................................................aauagugucuagaauaucuuga..........    4       0    li1

    ..................................................auagugucuagaauaucuuga..........    1       0    per

    ................aagauauucuagacacuauuc............................................    1       0    va1
    ................aagauauucuagacacuauucu...........................................    3       0    va1
    .................................................aauagugucuagaauaucuug...........    2       0    va1
    .................................................aauagugucuagaauaucuuga..........    3       0    va1
    ..................................................auagugucuagaauaucuuga..........    2       0    va1

    .................................................aauagugucuagaauaucuug...........    1       0    ku2

    ................aagauauucuagacacuauucu...........................................    2       0    U20

    ................aagauauucuagacacuauucu...........................................    1       0    va4

    ..................................................auagugucuagaauaucuuga..........    1       0    j21

    ................aagauauucuagacacuauucu...........................................    1       0    HeL

    ................................................gaauagugucuagaauaucuug...........    1       0    va3
    .................................................aauagugucuagaauaucuuga..........    1       0    va3

    .................................................aauagugucuagaauaucuuga..........    1       0    la2

    ................aagauauucuagacacuauucu...........................................    1       0    MBM
```

Star **Mature**

uguuugaauuacuguuaagauauucuagacacuauucugugucacccagaauagugucuagaauaucuugacaauaauuca

................aagauauucuagacacuauucu... 1 0 j22

Provisional ID	:	hsa_can_410
Score total	:	436
Score for star read(s)	:	3.9
Score for read counts	:	427.4
Score for mfe	:	1.8
Score for randfold	:	
Score for cons. seed	:	3
Total read count	:	850
Mature read count	:	335
Loop read count	:	0
Star read count	:	515

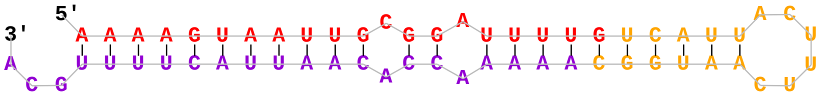

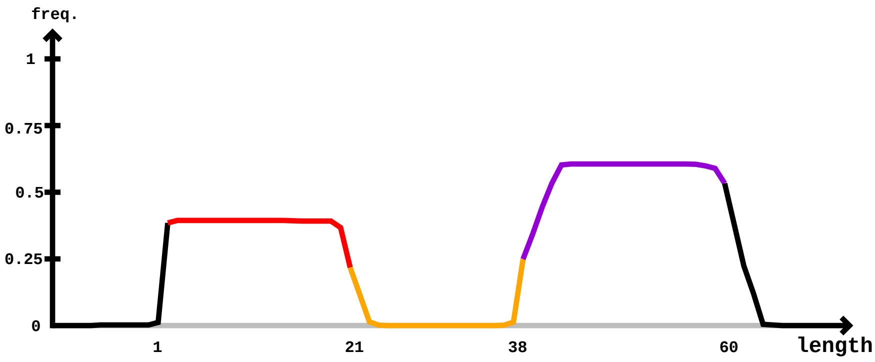

Mature **Star**

```
5'- agguugguacaaaaguaauugcggauuuugucauuacuuucaauggcaaaaaccacaauuacuuuugcaccagccucauag   -3'    obs
    agguugguacaaaaguaauugcggauuuugucauuacuuucaauggcaaaaaccacaauuacuuuugcaccagccucauag          exp
    agguugguacaaaaguaauugcggauuuugucauuacuuucaauggcaaaaaccacaauuacuuuugcaccagccucauag          known
    ((((((((.((((((((((((.((.((((((((((......)))))))))).)).)))))))))))).)))))))).....    reads    mm    sample
    ..........aaaaguaauugcggauuu.....................................................    3        0     ya1
    ..........aaaaguaauugcggauuuu....................................................    19       0     ya1
    ..........aaaaguaauugcggauuuug...................................................    5        0     ya1
    ...............................................aaaaaccacaauuacuuuug..............    1        0     ya1
    ...............................................aaaaaccacaauuacuuuugca............    4        0     ya1
    ................................................aaaaccacaauuacuuuugca............    3        0     ya1
    ................................................aaaaccacaauuacuuuugcac...........    1        0     ya1
    .................................................aaaccacaauuacuuuugca............    1        0     ya1
    ..................................................aaccacaauuacuuuugcacc..........    1        0     ya1

    ...............................................aaaaaccacaauuacuuuugca............    1        0     s04

    ..........aaaaguaauugcggauuuug...................................................    1        0     j26
    ...............................................aaaaaccacaauuacuuuugca............    3        0     j26
    ...................................................accacaauuacuuuugcacca.........    1        0     j26

    ...............................................aaaaaccacaauuacuuuugc.............    1        0     va1
    ...............................................aaaaaccacaauuacuuuugca............    6        0     va1
    ...............................................aaaaaccacaauuacuuuugcac...........    1        0     va1
    ................................................aaaaccacaauuacuuuugcac...........    5        0     va1
    .................................................aaaccacaauuacuuuugcacc..........    1        0     va1

    ...............................................aaaaaccacaauuacuuuugca............    1        0     ku5
    .................................................aaaccacaauuacuuuugcacc..........    1        0     ku5

    .................................................aaaccacaauuacuuuugcacc..........    1        0     k05

    ...............................................aaaaaccacaauuacuuuugca............    1        0     s06
    ..................................................aaccacaauuacuuuugcacca.........    1        0     s06
    ...................................................accacaauuacuuuugcacca.........    1        0     s06

    ...............................................aaaaaccacaauuacuuuugc.............    1        0     HeL
```

Mature Star

```
agguugguacaaaaguaauugcggauuuugucauuacuuucaauggcaaaaaccacaauuacuuuugcaccagccucauag
...............................................aaaaaccacaauuacuuuugca............  3  0  HeL
................................................aaaaccacaauuacuuuugcac...........  2  0  HeL
.................................................aaaccacaauuacuuuugcac...........  1  0  HeL
.................................................aaaccacaauuacuuuugcacc..........  3  0  HeL
..................................................aaccacaauuacuuuugcacca.........  3  0  HeL
...................................................accacaauuacuuuugcacca.........  5  0  HeL

..........aaaaguaauugcggauuuug...................................................  1  0  ku4

.................................................aaaccacaauuacuuuugcacc..........  1  0  s07

..........aaaaguaauugcggauuuug...................................................  1  0  s10
...............................................aaaaaccacaauuacuuuugca............  4  0  s10
.................................................aaaccacaauuacuuuugcac...........  1  0  s10

...............................................aaaaaccacaauuacuuuugca............  4  0  ta3
................................................aaaaccacaauuacuuuugc.............  1  0  ta3
................................................aaaaccacaauuacuuuugcac...........  1  0  ta3
.................................................aaaccacaauuacuuuugcac...........  1  0  ta3
.................................................aaaccacaauuacuuuugcacc..........  1  0  ta3
..................................................aaccacaauuacuuuugcacca.........  2  0  ta3

...........aaaguaauugcggauuuug...................................................  1  0  j06

...............................................aaaaaccacaauuacuuuugca............  5  0  j10
.................................................aaaccacaauuacuuuugcac...........  1  0  j10
..................................................aaccacaauuacuuuugcacca.........  1  0  j10

..........aaaaguaauugcggauuuu....................................................  4  0  j04
..........aaaaguaauugcggauuuug...................................................  4  0  j04

..................................................aaccacaauuacuuuugcac...........  1  0  k14

..........aaaaguaauugcggauuuug...................................................  2  0  j02

..........aaaaguaauugcggauuuug...................................................  1  0  va2
...............................................aaaaaccacaauuacuuuugcac...........  1  0  va2

...............................................aaaaaccacaauuacuuuugca............  1  0  j31

..........aaaaguaauugcggauuu.....................................................  1  0  j27
..........aaaaguaauugcggauuuu....................................................  4  0  j27
..........aaaaguaauugcggauuuug...................................................  7  0  j27
.................................................aaaccacaauuacuuuugca............  1  0  j27

...............................................aaaaaccacaauuacuuuugca............  2  0  s03
................................................aaaaccacaauuacuuuugca............  1  0  s03
................................................aaaaccacaauuacuuuugcac...........  2  0  s03

...............................................aaaaaccacaauuacuuuug..............  2  0  H52
...............................................aaaaaccacaauuacuuuugca............  1  0  H52
................................................aaaaccacaauuacuuuugc.............  1  0  H52
................................................aaaaccacaauuacuuuugca............  2  0  H52
.................................................aaaccacaauuacuuuugcacc..........  2  0  H52
..................................................aaccacaauuacuuuugcacc..........  1  0  H52
..................................................aaccacaauuacuuuugcacca.........  1  0  H52
...................................................accacaauuacuuuugcacca.........  1  0  H52
....................................................ccacaauuacuuuugcaccag........  1  0  H52

...............................................aaaaaccacaauuacuuuugca............  6  0  j22
................................................aaaaccacaauuacuuuugca............  1  0  j22
..................................................aaccacaauuacuuuugcacc..........  1  0  j22

................................................aaaaccacaauuacuuuugcac...........  1  0  k09

...............................................aaaaaccacaauuacuuuugc.............  3  0  B14
...............................................aaaaaccacaauuacuuuugca............  6  0  B14
................................................aaaaccacaauuacuuuugcac...........  1  0  B14
.................................................aaaccacaauuacuuuugca............  2  0  B14
.................................................aaaccacaauuacuuuugcacc..........  2  0  B14
```

Mature **Star**

```
agguugguacaaaaguaauugcggauuuugucauuacuuucaauggcaaaaaccacaauuacuuuugcaccagccucauag
...................................................accacaauuacuuuugcacca.........    2    0    B14

...............................................aaaaaccacaauuacuuuugcac...........    1    0    ku3
.................................................aaaccacaauuacuuuugcacca.........    1    0    ku3

...............................................aaaaaccacaauuacuuuugca............    1    0    j09
...................................................accacaauuacuuuugcacca.........    1    0    j09

...............................................aaaaaccacaauuacuuuugca............    4    0    j16

..........aaaaguaauugcggauuuu....................................................    4    0    j18

..........aaaaguaauugcggauuu.....................................................    1    0    ku6
...............................................aaaaaccacaauuacuuuugca............    2    0    ku6

...............................................aaaaaccacaauuacuuuugca............    2    0    j11

...uugguacaaaaguaauugcg..........................................................    1    0    ya3
.........caaaaguaauugcggauuuu....................................................    1    0    ya3
.........caaaaguaauugcggauuuug...................................................    2    0    ya3
..........aaaaguaauugcggauuu.....................................................    5    0    ya3
..........aaaaguaauugcggauuuu....................................................    33   0    ya3
..........aaaaguaauugcggauuuug...................................................    23   0    ya3
..........aaaaguaauugcggauuuugu..................................................    1    0    ya3
...........aaaguaauugcggauuuu....................................................    1    0    ya3
...........aaaguaauugcggauuuug...................................................    1    0    ya3
...............................................aaaaaccacaauuacuuuugca............    3    0    ya3
................................................aaaaccacaauuacuuuugca............    2    0    ya3
................................................aaaaccacaauuacuuuugcac...........    5    0    ya3
.................................................aaaccacaauuacuuuugca............    1    0    ya3
.................................................aaaccacaauuacuuuugcacc..........    1    0    ya3
..................................................aaccacaauuacuuuugcacc..........    2    0    ya3

...............................................aaaaaccacaauuacuuuugca............    2    0    la1
..................................................aaccacaauuacuuuugcacc..........    1    0    la1

...uugguacaaaaguaauugcgg.........................................................    1    0    HEK
..............................................caaaaaccacaauuacuuuugc.............    3    0    HEK
...............................................aaaaaccacaauuacuuuugc.............    1    0    HEK
...............................................aaaaaccacaauuacuuuugca............    8    0    HEK
................................................aaaaccacaauuacuuuugca............    2    0    HEK
................................................aaaaccacaauuacuuuugcac...........    3    0    HEK
.................................................aaaccacaauuacuuuugca............    1    0    HEK
.................................................aaaccacaauuacuuuugcac...........    3    0    HEK
.................................................aaaccacaauuacuuuugcacc..........    3    0    HEK
..................................................aaccacaauuacuuuugcacc..........    2    0    HEK
..................................................aaccacaauuacuuuugcacca.........    4    0    HEK
...................................................accacaauuacuuuugcacca.........    6    0    HEK

................................................aaaaccacaauuacuuuugca............    1    0    j15
....................................................ccacaauuacuuuugcaccag........    1    0    j15

..........aaaaguaauugcggauuuu....................................................    6    0    j17
..........aaaaguaauugcggauuuug...................................................    47   0    j17
..........aaaaguaauugcggauuuugu..................................................    2    0    j17
..........aaaaguaauugcggauuuuguc.................................................    5    0    j17
................................................aaaaccacaauuacuuuugca............    1    0    j17
..................................................aaccacaauuacuuuugcacca.........    2    0    j17
...................................................accacaauuacuuuugcacca.........    1    0    j17

................................................aaaaccacaauuacuuuugcac...........    1    0    la2
.................................................aaaccacaauuacuuuugca............    1    0    la2
.................................................aaaccacaauuacuuuugcacc..........    1    0    la2
..................................................aaccacaauuacuuuugcacca.........    1    0    la2

...................................................accacaauuacuuuugcacca.........    1    0    j12

..........aaaaguaauugcggauuuuguc.................................................    2    0    li2

...............................................aaaaaccacaauuacuuuugca............    3    0    va4
```

Mature Star

```
agguugguacaaaaguaauugcggauuuugucauuacuuucaauggcaaaaaccacaauuacuuuugcaccagccucauag
...............................................aaaaaccacaauuacuuuugcac...........  1  0  va4
................................................aaaaccacaauuacuuuugcac...........  1  0  va4
..................................................aaccacaauuacuuuugcacc..........  2  0  va4
...................................................accacaauuacuuuugcacca.........  4  0  va4

...............................................aaaaaccacaauuacuuuugca............  12  0  va3
................................................aaaaccacaauuacuuuugcac...........  2  0  va3
.................................................aaaccacaauuacuuuugcac...........  1  0  va3
.................................................aaaccacaauuacuuuugcacc..........  4  0  va3
..................................................aaccacaauuacuuuugcacc..........  2  0  va3
..................................................aaccacaauuacuuuugcacca.........  2  0  va3
...................................................accacaauuacuuuugcacca.........  1  0  va3

...............................................aaaaaccacaauuacuuuugca............  1  0  SW4
................................................aaaaccacaauuacuuuugcac...........  1  0  SW4
.................................................aaaccacaauuacuuuugcac...........  2  0  SW4
.................................................aaaccacaauuacuuuugcacc..........  3  0  SW4
..................................................aaccacaauuacuuuugcacca.........  3  0  SW4
...................................................accacaauuacuuuugcacc..........  1  0  SW4
...................................................accacaauuacuuuugcacca.........  3  0  SW4

.........caaaaguaauugcggauuuug...................................................  2  0  ya4
..........aaaaguaauugcggauuu.....................................................  7  0  ya4
..........aaaaguaauugcggauuuu....................................................  35  0  ya4
..........aaaaguaauugcggauuuug...................................................  21  0  ya4
...............................................aaaaaccacaauuacuuuugca............  4  0  ya4
................................................aaaaccacaauuacuuuugca............  2  0  ya4
................................................aaaaccacaauuacuuuugcac...........  4  0  ya4
.................................................aaaccacaauuacuuuugcac...........  1  0  ya4
.................................................aaaccacaauuacuuuugcacc..........  4  0  ya4
..................................................aaccacaauuacuuuugca............  3  0  ya4
..................................................aaccacaauuacuuuugcacc..........  4  0  ya4
..................................................aaccacaauuacuuuugcacca.........  2  0  ya4

..........aaaaguaauugcggauuuug...................................................  1  0  ku1
...............................................aaaaaccacaauuacuuuugca............  4  0  ku1
..................................................aaccacaauuacuuuugcacc..........  1  0  ku1

.........caaaaguaauugcggauuuug...................................................  1  0  j23
..........aaaaguaauugcggauuuug...................................................  10  0  j23
...............................................aaaaaccacaauuacuuuugc.............  1  0  j23
...............................................aaaaaccacaauuacuuuugca............  2  0  j23
................................................aaaaccacaauuacuuuugca............  1  0  j23
..................................................aaccacaauuacuuuugcacca.........  1  0  j23
...................................................accacaauuacuuuugcacca.........  1  0  j23

..........aaaaguaauugcggauuuug...................................................  1  0  s05
..........aaaaguaauugcggauuuugu..................................................  1  0  s05
...............................................aaaaaccacaauuacuuuugca............  2  0  s05

...............................................aaaaaccacaauuacuuu................  1  0  ta2
................................................aaaaccacaauuacuuuugcac...........  1  0  ta2

..........aaaaguaauugcggauuuug...................................................  13  0  j24

..........aaaaguaauugcggauuuug...................................................  1  0  k11
...............................................aaaaaccacaauuacuuuugca............  1  0  k11

...............................................aaaaaccacaauuacuuuugca............  1  0  ta1

..............................................caaaaaccacaauuacuuuugc.............  1  0  s09
................................................aaaaccacaauuacuuuugca............  1  0  s09
..................................................aaccacaauuacuuuugcacca.........  1  0  s09
...................................................accacaauuacuuuugcacca.........  1  0  s09

..............................................caaaaaccacaauuacuuu................  1  0  NL2
...............................................aaaaaccacaauuacuuuugca............  3  0  NL2
..................................................aaccacaauuacuuuugcacc..........  1  0  NL2
..................................................aaccacaauuacuuuugcacca.........  5  0  NL2

..........aaaaguaauugcggauuuug...................................................  1  0  k10
```

```
          Mature                                Star
agguugguacaaaaguaauugcggauuuugucauuacuuucaauggcaaaaaccacaauuacuuuugcaccagccucauag

...............................................aaaaaccacaauuacuuuugca............      4      0      j13
................................................aaaaccacaauuacuuuugca............      2      0      j13
..................................................aaccacaauuacuuuugcacca.........      2      0      j13

..........aaaaguaauugcggauuuuguc.................................................      1      0      j19
...............................................aaaaaccacaauuacuuuugca............      2      0      j19
.................................................aaaccacaauuacuuuugca............      2      0      j19
..................................................aaccacaauuacuuuugcacca.........      1      0      j19
...................................................accacaauuacuuuugcacca.........      2      0      j19

..........aaaaguaauugcggauuuu....................................................      1      0      j25
..........aaaaguaauugcggauuuug...................................................      1      0      j25
...............................................aaaaaccacaauuacuuuugca............      1      0      j25

................................................aaaaccacaauuacuuuugca............      1      0      k04

...............................................aaaaaccacaauuacuuuugca............      2      0      s01
................................................aaaaccacaauuacuuuugcac...........      2      0      s01
.................................................aaaccacaauuacuuuugcacc..........      2      0      s01

..........aaaaguaauugcggauuuug...................................................      1      0      U20
..............................................caaaaaccacaauuacuuuugc.............      1      0      U20
...............................................aaaaaccacaauuacuuuugc.............      22     0      U20
...............................................aaaaaccacaauuacuuuugca............      17     0      U20
................................................aaaaccacaauuacuuuugca............      1      0      U20
................................................aaaaccacaauuacuuuugcac...........      8      0      U20
.................................................aaaccacaauuacuuuugcac...........      6      0      U20
.................................................aaaccacaauuacuuuugcacc..........      14     0      U20
..................................................aaccacaauuacuuuugcacc..........      6      0      U20
..................................................aaccacaauuacuuuugcacca.........      1      0      U20
...................................................accacaauuacuuuugcacca.........      15     0      U20

.........caaaaguaauugcggauuuugu..................................................      1      0      j21
..........aaaaguaauugcggauuuug...................................................      2      0      j21
...............................................aaaaaccacaauuacuuuugca............      3      0      j21
................................................aaaaccacaauuacuuuugca............      1      0      j21
.................................................aaaccacaauuacuuuugcacc..........      1      0      j21


..................................................aaccacaauuacuuuugcacc..........      1      0      s11
..................................................aaccacaauuacuuuugcacca.........      1      0      s11

...............................................aaaaaccacaauuacuuuugca............      1      0      j07

..........aaaaguaauugcggauuuug...................................................      2      0      j14
.................................................aaaccacaauuacuuuugcacc..........      1      0      j14

...............................................aaaaaccacaauuacuuuugca............      1      0      s08
...................................................accacaauuacuuuugcac...........      1      0      s08

...............................................aaaaaccacaauuacuuuugc.............      1      0      DLD
................................................aaaaccacaauuacuuuugcac...........      1      0      DLD
.................................................aaaccacaauuacuuuugcacc..........      3      0      DLD
...................................................accacaauuacuuuugcacc..........      1      0      DLD
...................................................accacaauuacuuuugcacca.........      4      0      DLD
...................................................accacaauuacuuuugcaccagc.......      1      0      DLD

...........aaaguaauugcggauuuuguca................................................      1      0      k03
...............................................aaaaaccacaauuacuuuugca............      1      0      k03

..........aaaaguaauugcggauuuug...................................................      1      0      NL3
..............................................caaaaaccacaauuacuuuu...............      1      0      NL3
..............................................caaaaaccacaauuacuuuug..............      2      0      NL3
...............................................aaaaaccacaauuacuuuugca............      7      0      NL3
................................................aaaaccacaauuacuuuugcac...........      1      0      NL3
..................................................aaccacaauuacuuuugcacca.........      1      0      NL3

.........caaaaguaauugcggauuuug...................................................      1      0      ya2
..........aaaaguaauugcggauuu.....................................................      4      0      ya2
```

Mature **Star**

```
agguugguacaaaaguaauugcggauuuugucauuacuuucaauggcaaaaaccacaauuacuuuugcaccagccucauag
..........aaaaguaauugcggauuuu....................................................    19    0    ya2
..........aaaaguaauugcggauuuug...................................................    8     0    ya2
...........aaaguaauugcggauuuu....................................................    1     0    ya2
...........aaaguaauugcggauuuug...................................................    3     0    ya2
...............................................aaaaaccacaauuacuuuu...............    2     0    ya2
...............................................aaaaaccacaauuacuuuugc.............    1     0    ya2
...............................................aaaaaccacaauuacuuuugca............    2     0    ya2
................................................aaaaccacaauuacuuuugca............    6     0    ya2
.................................................aaaccacaauuacuuuugcac...........    3     0    ya2
.................................................aaaccacaauuacuuuugcacca.........    1     0    ya2
..................................................aaccacaauuacuuuugcacca.........    2     0    ya2

.................................................aaaccacaauuacuuuugcacc..........    1     0    k06

..........aaaaguaauugcggauuuug...................................................    2     0    j29
..........aaaaguaauugcggauuuuguc.................................................    1     0    j29
...............................................aaaaaccacaauuacuuuugca............    1     0    j29
................................................aaaaccacaauuacuuuugca............    1     0    j29

.............................................caaaaaccacaauuacuuuug...............    1     0    sh2
....................................................ccacaauuacuuuugcaccagc.......    1     0    sh2

...............................................aaaaaccacaauuacuuuu...............    1     0    MCF
...............................................aaaaaccacaauuacuuuugc.............    1     0    MCF
...............................................aaaaaccacaauuacuuuugca............    2     0    MCF
................................................aaaaccacaauuacuuuugcac...........    1     0    MCF

..........aaaaguaauugcggauuuuguc.................................................    2     0    MBM
...............................................aaaaaccacaauuacuuuug..............    1     0    MBM
...............................................aaaaaccacaauuacuuuugc.............    1     0    MBM
...............................................aaaaaccacaauuacuuuugca............    2     0    MBM
................................................aaaaccacaauuacuuuugca............    1     0    MBM
................................................aaaaccacaauuacuuuugcac...........    3     0    MBM
.................................................aaaccacaauuacuuuugcacc..........    4     0    MBM
..................................................aaccacaauuacuuuugcacc..........    1     0    MBM
...................................................accacaauuacuuuugcacca.........    4     0    MBM

............................................gcaaaaaccacaauuacuuuug...............    1     0    li1
...............................................aaaaaccacaauuacuuuugc.............    1     0    li1
................................................aaaaccacaauuacuuuugcac...........    1     0    li1
.................................................aaaccacaauuacuuuugcacc..........    2     0    li1
..................................................aaccacaauuacuuuugcacca.........    1     0    li1

...............................................aaaaaccacaauuacuuuugc.............    7     0    A54
..................................................aaccacaauuacuuuugcacc..........    4     0    A54
..................................................aaccacaauuacuuuugcacca.........    4     0    A54

...................................................accacaauuacuuuugcacca.........    1     0    k12

..........aaaaguaauugcggauuuug...................................................    1     0    j30
```

Provisional ID	:	hsa_can_413
Score total	:	276.7
Score for star read(s)	:	3.9
Score for read counts	:	271.4
Score for mfe	:	2
Score for randfold	:	
Score for cons. seed	:	-0.6
Total read count	:	544
Mature read count	:	516
Loop read count	:	0
Star read count	:	28

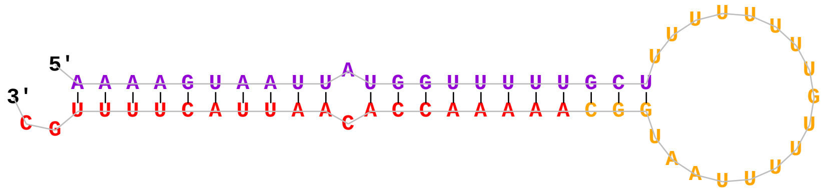

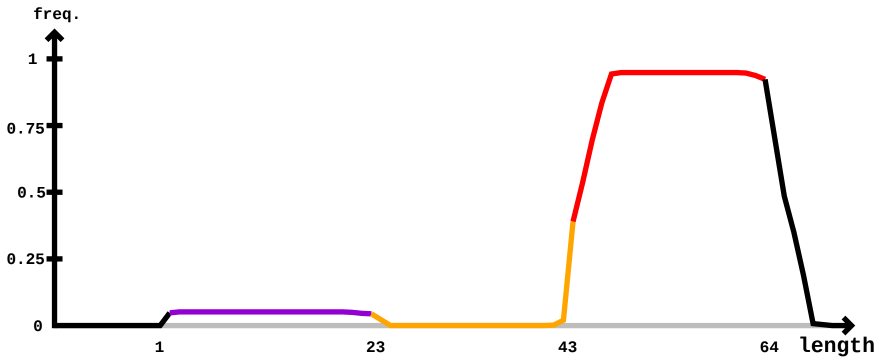


```
           Star                                      Mature
5'- agguuggugcaaaaguaauuaugguuuuugcuuuuuuuuuguuuuuaauggcaaaaaccacaauuacuuuugcaccagccu   -3'    obs
    agguuggugcaaaaguaauuaugguuuuugcuuuuuuuuuguuuuuaauggcaaaaaccacaauuacuuuugcaccagccu          exp
    agguuggugcaaaaguaauuaugguuuuugcuuuuuuuuuguuuuuaauggcaaaaaccacaauuacuuuugcaccagccu          known
    ((((((((((((((((((((.(((((((((((.................))))))))))).))))))))))))))))))))    reads    mm    sample
    ....................................................aaaaaccacaauuacuuuug.........    1        0     ya1
    ....................................................aaaaaccacaauuacuuuugca.......    4        0     ya1
    .....................................................aaaaccacaauuacuuuugca.......    3        0     ya1
    .....................................................aaaaccacaauuacuuuugcac......    1        0     ya1
    ......................................................aaaccacaauuacuuuugca.......    1        0     ya1
    .......................................................aaccacaauuacuuuugcacc.....    1        0     ya1

    ....................................................aaaaaccacaauuacuuuugca.......    1        0     s04

    ....................................................aaaaaccacaauuacuuuugca.......    3        0     j26
    ........................................................accacaauuacuuuugcacca....    1        0     j26

    ....................................................aaaaaccacaauuacuuuugc........    1        0     va1
    ....................................................aaaaaccacaauuacuuuugca.......    6        0     va1
    ....................................................aaaaaccacaauuacuuuugcac......    1        0     va1
    .....................................................aaaaccacaauuacuuuugcac......    5        0     va1
    ......................................................aaaccacaauuacuuuugcacc.....    1        0     va1

    ....................................................aaaaaccacaauuacuuuugca.......    1        0     ku5
    ......................................................aaaccacaauuacuuuugcacc.....    1        0     ku5

    ......................................................aaaccacaauuacuuuugcacc.....    1        0     k05

    ..........aaaaguaauuaugguuuuugcu.................................................    1        0     s06
    ....................................................aaaaaccacaauuacuuuugca.......    1        0     s06
    .......................................................aaccacaauuacuuuugcacca....    1        0     s06
    ........................................................accacaauuacuuuugcacca....    1        0     s06

    ..........aaaaguaauuaugguuuuugcu.................................................    3        0     HeL
    ....................................................aaaaaccacaauuacuuuugc........    1        0     HeL
    ....................................................aaaaaccacaauuacuuuugca.......    3        0     HeL
    .....................................................aaaaccacaauuacuuuugcac......    2        0     HeL
```

Star Mature			
agguuggugcaaaaguaauuaugguuuuugcuuuuuuuuuguuuuuaauggcaaaaaccacaauuacuuuugcaccagccu			
....aaaccacaauuacuuuugcac......	1	0	HeL
....aaaccacaauuacuuuugcacc.....	3	0	HeL
....aaccacaauuacuuuugcacca....	3	0	HeL
....accacaauuacuuuugcacca....	5	0	HeL
....aaaccacaauuacuuuugcacc.....	1	0	s07
....aaaaaccacaauuacuuuugca.......	4	0	s10
....aaaccacaauuacuuuugcac......	1	0	s10
....aaaaaccacaauuacuuuugca.......	4	0	ta3
....aaaaccacaauuacuuuugc........	1	0	ta3
....aaaaccacaauuacuuuugcac......	1	0	ta3
....aaaccacaauuacuuuugcac......	1	0	ta3
....aaaccacaauuacuuuugcacc.....	1	0	ta3
....aaccacaauuacuuuugcacca....	2	0	ta3
....aaaaaccacaauuacuuuugca.......	5	0	j10
....aaaccacaauuacuuuugcac......	1	0	j10
....aaccacaauuacuuuugcacca....	1	0	j10
....aaccacaauuacuuuugcac......	1	0	k14
....aaaaaccacaauuacuuuugcac......	1	0	va2
....aaaccacaauuacuuuugca.......	1	0	j27
....aaaaaccacaauuacuuuugca.......	1	0	j31
....aaaaaccacaauuacuuuug.........	2	0	H52
....aaaaaccacaauuacuuuugca.......	1	0	H52
....aaaaccacaauuacuuuugc........	1	0	H52
....aaaaccacaauuacuuuugca.......	2	0	H52
....aaaccacaauuacuuuugcacc.....	2	0	H52
....aaccacaauuacuuuugcacc.....	1	0	H52
....aaccacaauuacuuuugcacca....	1	0	H52
....accacaauuacuuuugcacca....	1	0	H52
....ccacaauuacuuuugcaccag...	1	0	H52
...........aaaguaauuaugguuuuugcu...	1	0	s03
....aaaaaccacaauuacuuuugca.......	2	0	s03
....aaaaccacaauuacuuuugca.......	1	0	s03
....aaaaccacaauuacuuuugcac......	2	0	s03
....aaaaaccacaauuacuuuugca.......	6	0	j22
....aaaaccacaauuacuuuugca.......	1	0	j22
....aaccacaauuacuuuugcacc.....	1	0	j22
....aaaaccacaauuacuuuugcac......	1	0	k09
....aaaaaccacaauuacuuuugc........	3	0	B14
....aaaaaccacaauuacuuuugca.......	6	0	B14
....aaaaccacaauuacuuuugcac......	1	0	B14
....aaaccacaauuacuuuugca.......	2	0	B14
....aaaccacaauuacuuuugcacc.....	2	0	B14
....accacaauuacuuuugcacca....	2	0	B14
....aaaaaccacaauuacuuuugcac......	1	0	ku3
....aaaccacaauuacuuuugcacca....	1	0	ku3
....aaaaaccacaauuacuuuugca.......	1	0	j09
....accacaauuacuuuugcacca....	1	0	j09
....aaaaaccacaauuacuuuugca.......	4	0	j16
....aaaaaccacaauuacuuuugca.......	2	0	ku6
....aaaaaccacaauuacuuuugca.......	2	0	j11
....aaaaaccacaauuacuuuugca.......	3	0	ya3

Star **Mature**

```
agguuggugcaaaaguaauuaugguuuuugcuuuuuuuuguuuuuaauggcaaaaaccacaauuacuuuugcaccagccu
....................................................aaaaaccacaauuacuuuugca.......  2   0   ya3
.....................................................aaaaccacaauuacuuuugcac......  5   0   ya3
......................................................aaaccacaauuacuuuugca.......  1   0   ya3
......................................................aaaccacaauuacuuuugcacc.....  1   0   ya3
.......................................................aaccacaauuacuuuugcacc.....  2   0   ya3

....................................................aaaaaccacaauuacuuuugca.......  2   0   la1
.......................................................aaccacaauuacuuuugcacc.....  1   0   la1

..........aaaaguaauuaugguuuuugcu.................................................  8   0   HEK
...................................................caaaaaccacaauuacuuuugc........  3   0   HEK
....................................................aaaaaccacaauuacuuuugc........  1   0   HEK
....................................................aaaaaccacaauuacuuuugca.......  8   0   HEK
.....................................................aaaaccacaauuacuuuugca.......  2   0   HEK
.....................................................aaaaccacaauuacuuuugcac......  3   0   HEK
......................................................aaaccacaauuacuuuugca.......  1   0   HEK
......................................................aaaccacaauuacuuuugcac......  3   0   HEK
......................................................aaaccacaauuacuuuugcacc.....  3   0   HEK
.......................................................aaccacaauuacuuuugcacc.....  2   0   HEK
.......................................................aaccacaauuacuuuugcacca....  4   0   HEK
........................................................accacaauuacuuuugcacca....  6   0   HEK

.....................................................aaaaccacaauuacuuuugca.......  1   0   j17
.......................................................aaccacaauuacuuuugcacca....  2   0   j17
........................................................accacaauuacuuuugcacca....  1   0   j17

..........aaaaguaauuaugguuuuug...................................................  1   0   j15
..........aaaaguaauuaugguuuuugcu.................................................  1   0   j15
.....................................................aaaaccacaauuacuuuugca.......  1   0   j15
.........................................................ccacaauuacuuuugcaccag...  1   0   j15

.....................................................aaaaccacaauuacuuuugcac......  1   0   la2
......................................................aaaccacaauuacuuuugca.......  1   0   la2
......................................................aaaccacaauuacuuuugcacc.....  1   0   la2
.......................................................aaccacaauuacuuuugcacca....  1   0   la2

........................................................accacaauuacuuuugcacca....  1   0   j12

....................................................aaaaaccacaauuacuuuugca.......  3   0   va4
....................................................aaaaaccacaauuacuuuugcac......  1   0   va4
.....................................................aaaaccacaauuacuuuugcac......  1   0   va4
.......................................................aaccacaauuacuuuugcacc.....  2   0   va4
........................................................accacaauuacuuuugcacca....  4   0   va4

....................................................aaaaaccacaauuacuuuugca.......  12  0   va3
.....................................................aaaaccacaauuacuuuugcac......  2   0   va3
......................................................aaaccacaauuacuuuugcac......  1   0   va3
......................................................aaaccacaauuacuuuugcacc.....  4   0   va3
.......................................................aaccacaauuacuuuugcacc.....  2   0   va3
.......................................................aaccacaauuacuuuugcacca....  2   0   va3
........................................................accacaauuacuuuugcacca....  1   0   va3

....................................................aaaaaccacaauuacuuuugca.......  4   0   ya4
.....................................................aaaaccacaauuacuuuugca.......  2   0   ya4
.....................................................aaaaccacaauuacuuuugcac......  4   0   ya4
......................................................aaaccacaauuacuuuugcac......  1   0   ya4
......................................................aaaccacaauuacuuuugcacc.....  4   0   ya4
.......................................................aaccacaauuacuuuugca.......  3   0   ya4
.......................................................aaccacaauuacuuuugcacc.....  4   0   ya4
.......................................................aaccacaauuacuuuugcacca....  2   0   ya4

..........aaaaguaauuaugguuuuugcu.................................................  1   0   SW4
....................................................aaaaaccacaauuacuuuugca.......  1   0   SW4
.....................................................aaaaccacaauuacuuuugcac......  1   0   SW4
......................................................aaaccacaauuacuuuugcac......  2   0   SW4
......................................................aaaccacaauuacuuuugcacc.....  3   0   SW4
.......................................................aaccacaauuacuuuugcacca....  3   0   SW4
........................................................accacaauuacuuuugcacc.....  1   0   SW4
........................................................accacaauuacuuuugcacca....  3   0   SW4

....................................................aaaaaccacaauuacuuuugca.......  4   0   ku1
```

Star **Mature**

agguuggugcaaaaguaauuaugguuuuugcuuuuuuuuuguuuuuaauggcaaaaaccacaauuacuuuugcaccagccu

....aaccacaauuacuuuugcacc.....	1	0	ku1
....aaaaaccacaauuacuuuugc........	1	0	j23
....aaaaaccacaauuacuuuugca.......	2	0	j23
....aaaaccacaauuacuuuugca.......	1	0	j23
....aaccacaauuacuuuugcacca....	1	0	j23
....accacaauuacuuuugcacca....	1	0	j23
....aaaaaccacaauuacuuuugca.......	2	0	s05
....aaaaaccacaauuacuuu...........	1	0	ta2
....aaaaccacaauuacuuuugcac......	1	0	ta2
....aaaaaccacaauuacuuuugca.......	1	0	k11
....aaaaaccacaauuacuuuugca.......	1	0	ta1
....caaaaaccacaauuacuuuugc........	1	0	s09
....aaaaccacaauuacuuuugca.......	1	0	s09
....aaccacaauuacuuuugcacca....	1	0	s09
....accacaauuacuuuugcacca....	1	0	s09
....caaaaaccacaauuacuuuu..........	1	0	NL2
....aaaaaccacaauuacuuuugca.......	3	0	NL2
....aaccacaauuacuuuugcacc.....	1	0	NL2
....aaccacaauuacuuuugcacca....	5	0	NL2
..........aaaaguaauuaugguuuuu..	1	0	j13
....aaaaaccacaauuacuuuugca.......	4	0	j13
....aaaaccacaauuacuuuugca.......	2	0	j13
....aaccacaauuacuuuugcacca....	2	0	j13
....aaaaaccacaauuacuuuugca.......	2	0	j19
....aaaccacaauuacuuuugca.......	2	0	j19
....aaccacaauuacuuuugcacca....	1	0	j19
....accacaauuacuuuugcacca....	2	0	j19
....aaaaaccacaauuacuuuugca.......	1	0	j25
....aaaaaccacaauuacuuuugca.......	2	0	s01
....aaaaccacaauuacuuuugcac......	2	0	s01
....aaaccacaauuacuuuugcacc.....	2	0	s01
....aaaaccacaauuacuuuugca.......	1	0	k04
..........aaaaguaauuaugguuuuugcu...	7	0	U20
....caaaaaccacaauuacuuuugc........	1	0	U20
....aaaaaccacaauuacuuuugc........	22	0	U20
....aaaaaccacaauuacuuuugca.......	17	0	U20
....aaaaccacaauuacuuuugca.......	1	0	U20
....aaaaccacaauuacuuuugcac......	8	0	U20
....aaaccacaauuacuuuugcac......	6	0	U20
....aaaccacaauuacuuuugcacc.....	14	0	U20
....aaccacaauuacuuuugcacc.....	6	0	U20
....aaccacaauuacuuuugcacca....	1	0	U20
....accacaauuacuuuugcacca....	15	0	U20
....aaaaaccacaauuacuuuugca.......	3	0	j21
....aaaaccacaauuacuuuugca.......	1	0	j21
....aaaccacaauuacuuuugcacc.....	1	0	j21
....aaccacaauuacuuuugcacc.....	1	0	s11
....aaccacaauuacuuuugcacca....	1	0	s11
....aaaaaccacaauuacuuuugca.......	1	0	j07
....aaaccacaauuacuuuugcacc.....	1	0	j14
....aaaaaccacaauuacuuuugca.......	1	0	s08

```
          Star                                      Mature
agguuggugcaaaaguaauuaugguuuuugcuuuuuuuuuguuuuuaauggcaaaaaccacaauuacuuuugcaccagccu
........................................................accacaauuacuuuugcac......    1    0    s08

..........aaaaguaauuaugguuuuugcu.................................................    1    0    DLD
....................................................aaaaaccacaauuacuuuugc........    1    0    DLD
.....................................................aaaaccacaauuacuuuugcac......    1    0    DLD
......................................................aaaccacaauuacuuuugcacc.....    3    0    DLD
........................................................accacaauuacuuuugcacc.....    1    0    DLD
........................................................accacaauuacuuuugcacca....    4    0    DLD
........................................................accacaauuacuuuugcaccagc..    1    0    DLD

....................................................aaaaaccacaauuacuuuugca.......    1    0    k03

....................................................aaaaaccacaauuacuuuu..........    2    0    ya2
....................................................aaaaaccacaauuacuuuugc........    1    0    ya2
....................................................aaaaaccacaauuacuuuugca.......    2    0    ya2
.....................................................aaaaccacaauuacuuuugca.......    6    0    ya2
......................................................aaaccacaauuacuuuugcac......    3    0    ya2
......................................................aaaccacaauuacuuuugcacca....    1    0    ya2
.......................................................aaccacaauuacuuuugcacca....    2    0    ya2

...................................................caaaaaccacaauuacuuuu..........    1    0    NL3
...................................................caaaaaccacaauuacuuuug.........    2    0    NL3
....................................................aaaaaccacaauuacuuuugca.......    7    0    NL3
.....................................................aaaaccacaauuacuuuugcac......    1    0    NL3
.......................................................aaccacaauuacuuuugcacca....    1    0    NL3

......................................................aaaccacaauuacuuuugcacc.....    1    0    k06

...................................................caaaaaccacaauuacuuuug.........    1    0    sh2
.........................................................ccacaauuacuuuugcaccagc..    1    0    sh2

....................................................aaaaaccacaauuacuuuugca.......    1    0    j29
.....................................................aaaaccacaauuacuuuugca.......    1    0    j29

..........aaaaguaauuaugguuuuugc..................................................    1    0    MCF
....................................................aaaaaccacaauuacuuuu..........    1    0    MCF
....................................................aaaaaccacaauuacuuuugc........    1    0    MCF
....................................................aaaaaccacaauuacuuuugca.......    2    0    MCF
.....................................................aaaaccacaauuacuuuugcac......    1    0    MCF

....................................................aaaaaccacaauuacuuuug.........    1    0    MBM
....................................................aaaaaccacaauuacuuuugc........    1    0    MBM
....................................................aaaaaccacaauuacuuuugca.......    2    0    MBM
.....................................................aaaaccacaauuacuuuugca.......    1    0    MBM
.....................................................aaaaccacaauuacuuuugcac......    3    0    MBM
......................................................aaaccacaauuacuuuugcacc.....    4    0    MBM
.......................................................aaccacaauuacuuuugcacc.....    1    0    MBM
........................................................accacaauuacuuuugcacca....    4    0    MBM

..................................................gcaaaaaccacaauuacuuuug.........    1    0    li1
....................................................aaaaaccacaauuacuuuugc........    1    0    li1
.....................................................aaaaccacaauuacuuuugcac......    1    0    li1
......................................................aaaccacaauuacuuuugcacc.....    2    0    li1
.......................................................aaccacaauuacuuuugcacca....    1    0    li1

..........aaaaguaauuaugguuuuug...................................................    1    0    k13

....................................................aaaaaccacaauuacuuuugc........    7    0    A54
.......................................................aaccacaauuacuuuugcacc.....    4    0    A54
.......................................................aaccacaauuacuuuugcacca....    4    0    A54

...........aaaguaauuaugguuuuugcu.................................................    1    0    k12
........................................................accacaauuacuuuugcacca....    1    0    k12
```

Provisional ID : hsa_can_444
Score total : 43.9
Score for star read(s) : 3.9
Score for read counts : 35.8
Score for mfe : 1.2
Score for randfold :
Score for cons. seed : 3
Total read count : 82
Mature read count : 59
Loop read count : 0
Star read count : 23

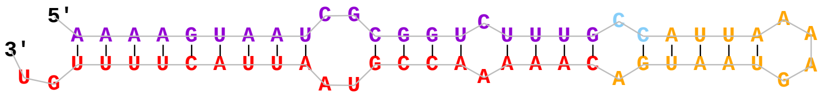

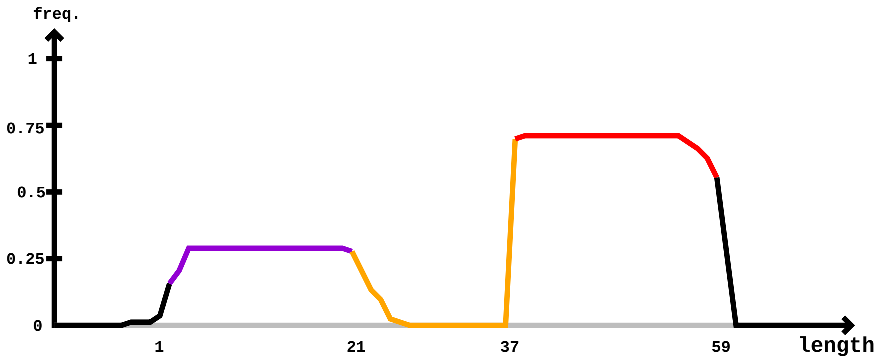

Star **Mature**

```
5'- agguugcugcaaaaguaaucgcggucuuugccauuaaaaguaaugacaaaaaccguaauuacuuuuguaccaacuuauaac -3'   obs
    agguugcugcaaaaguaaucgcggucuuugccauuaaaaguaaugacaaaaaccguaauuacuuuuguaccaacuuauaac       exp
    ((((((.((((((((((((..((((.((((.(((((....))))).)))).)))).)))))))))))).)))))).....    reads   mm   sample
    ...........aaaguaaucgcggucuuugcca................................................    1       0    k09

    ..........aaaaguaaucgcggucuuugcc.................................................    1       0    j23
    ............aaguaaucgcggucuuugccau...............................................    1       0    j23

    ..............................................caaaaaccguaauuacuuuugu.............    1       0    B14

    ..............................................caaaaaccguaauuacuuuu...............    3       0    ya1
    ..............................................caaaaaccguaauuacuuuugua............    3       0    ya1

    ..............................................caaaaaccguaauuacuuuug..............    2       0    j18

    ..........aaaaguaaucgcggucuuug...................................................    1       0    j09

    ..............................................caaaaaccguaauuacuuuugu.............    2       0    j26

    ..............................................caaaaaccguaauuacuuuug..............    1       0    va1
    ..............................................caaaaaccguaauuacuuuugu.............    17      0    va1

    ..........aaaaguaaucgcggucuuug...................................................    3       0    j24

    ..............................................caaaaaccguaauuacuu.................    2       0    ya3

    ..............................................caaaaaccguaauuacuuuugu.............    5       0    j15

    ..........aaaaguaaucgcggucuuugcc.................................................    1       0    j17

    ...........aaaguaaucgcggucuuug...................................................    1       0    k08

    ..........aaaaguaaucgcggucuuugcc.................................................    1       0    j12

    .........caaaaguaaucgcggucuuugc..................................................    1       0    j19
    ...........aaaguaaucgcggucuuug...................................................    2       0    j19
```

Star Mature

```
agguugcugcaaaaguaaucgcggucuuugccauuaaaaguaaugacaaaaaccguaauuacuuuuguaccaacuuauaac
..............................................caaaaaccguaauuacuuuug..............   2    0    j19

............aaguaaucgcggucuuugcca................................................   2    0    sh1

..............................................caaaaaccguaauuacuuuugu.............   1    0    k01

..........aaaaguaaucgcggucuuug...................................................   2    0    j25

............aaguaaucgcggucuuugcca................................................   1    0    k13

.........caaaaguaaucgcggucuuug...................................................   1    0    ku2

............aaguaaucgcggucuuugccauu..............................................   1    0    j21

..............................................caaaaaccguaauuacuuuugu.............   2    0    va2

............aaguaaucgcggucuuugcca................................................   1    0    k14

......cugcaaaaguaaucgcggucuuu....................................................   1    0    va3
..............................................caaaaaccguaauuacuuuugu.............   15   0    va3

..............................................caaaaaccguaauuacuuu................   2    0    ya4

............aaguaaucgcggucuuugcca................................................   1    0    k12

..........aaaaguaaucgcggucuuug...................................................   1    0    ku1

...............................................aaaaaccguaauuacuuuug..............   1    0    j22
```

Provisional ID	:	hsa_can_536
Score total	:	42.3
Score for star read(s)	:	3.9
Score for read counts	:	37.3
Score for mfe	:	1.7
Score for randfold	:	
Score for cons. seed	:	-0.6
Total read count	:	85
Mature read count	:	25
Loop read count	:	0
Star read count	:	60

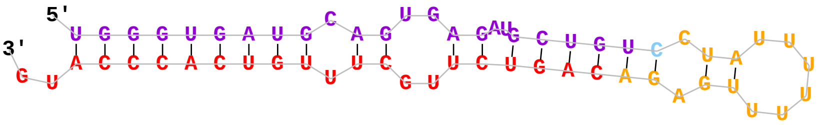

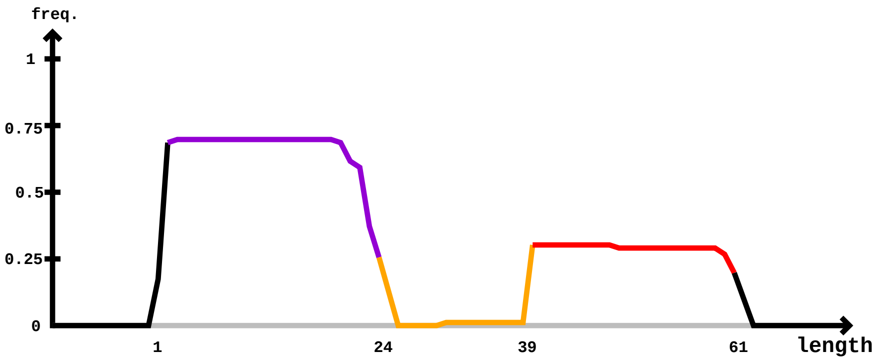


```
                Star                                 Mature
5'- cacuccagccugggugaugcagugagaugcuguccuauuuuuuugagacagucuugcuuugucacccaugcugaagugcag  -3'   obs
    cacuccagccugggugaugcagugagaugcugucuauuuuuuugagacagucuugcuuugucacccaugcugaagugcag         exp
    ((((.((((.(((((((((.((..((..((((((.((......)).))))))))..)).))))))))).)))).))))...   reads     mm      sample
    ..........ugggugaugcagugagaugcugu................................................   1         0       k09

    ..........ugggugaugcagugagaugcug.................................................   1         0       s08

    ................................................cagucuugcuuugucacccaug...........   1         0       DLD

    ..........ugggugaugcagugagaugcugu................................................   2         0       B14
    ................................................cagucuugcuuugucacccaug...........   1         0       B14

    ..........ugggugaugcagugagaug....................................................   2         0       j16
    ..........ugggugaugcagugagaugcu..................................................   1         0       j16
    ..........ugggugaugcagugagaugcugu................................................   1         0       j16

    .........cugggugaugcagugagaugcu..................................................   1         0       s05
    ..........ugggugaugcagugagaug....................................................   2         0       s05
    ..........ugggugaugcagugagaugcugu................................................   1         0       s05

    .........cugggugaugcagugagau.....................................................   1         0       ya2
    .........cugggugaugcagugagaug....................................................   1         0       ya2

    ..........ugggugaugcagugagaugcu..................................................   2         0       s12

    .........cugggugaugcagugagaugcug.................................................   1         0       k11

    ................................................cagucuugcuuugucacccaug...........   1         0       s06

    ................................................cagucuugcuuugucacccau............   1         0       k06
    ................................................cagucuugcuuugucacccaug...........   1         0       k06

    ..........ugggugaugcagugagaugcu..................................................   2         0       s09
    ..........ugggugaugcagugagaugcugu................................................   1         0       s09

    ..........ugggugaugcagugagaugc...................................................   1         0       HeL
```

```
          Star                               Mature
cacuccagccugggugaugcagugagaugcuguccuauuuuuuugagacagucuugcuuugucacccaugcugaagugcag
..........ugggugaugcagugagaugcugu................................................    2    0    HeL
................................................cagucuugcuuugucacccau............    1    0    HeL

..........ugggugaugcagugagaugcugu................................................    1    0    la1

.........cugggugaugcagugagaugcu..................................................    2    0    HEK
..........ugggugaugcagugagaugcugu................................................    3    0    HEK
..........ugggugaugcagugagaugcuguc...............................................    2    0    HEK
................................................cagucuugcuuugucaccca.............    2    0    HEK
................................................cagucuugcuuugucacccau............    1    0    HEK

.........cugggugaugcagugagaugcug.................................................    1    0    sh2

................................................cagucuugcuuugucacccaug...........    3    0    MCF

..........ugggugaugcagugagaugcug.................................................    1    0    NL1

.........cugggugaugcagugagaugcug.................................................    1    0    j13
..........ugggugaugcagugagaugcugu................................................    1    0    j13

..........ugggugaugcagugagaug....................................................    1    0    j12
..........ugggugaugcagugagaugcu..................................................    3    0    j12
..........ugggugaugcagugagaugcugu................................................    1    0    j12
...........gggugaugcagugagaugcu..................................................    1    0    j12

.........cugggugaugcagugagaugcug.................................................    1    0    j19
..........ugggugaugcagugagaugcu..................................................    1    0    j19

................................................cagucuugcuuugucacccaug...........    1    0    k01

.........cugggugaugcagugagaugcug.................................................    1    0    k04
..........ugggugaugcagugagaugc...................................................    1    0    k04
..........ugggugaugcagugagaugcu..................................................    1    0    k04

..........ugggugaugcagugagaugcug.................................................    3    0    U20
................................................cagucuugcuuugucacccaug...........    3    0    U20

..........ugggugaugcagugagaugcu..................................................    1    0    ku2

.........cugggugaugcagugagaugcugu................................................    1    0    j10

.........cugggugaugcagugagaugcugu................................................    1    0    s11

..........ugggugaugcagugagaugcu..................................................    3    0    j07

.........cugggugaugcagugagaugcugu................................................    1    0    va3
..........ugggugaugcagugagaugcugu................................................    1    0    va3

................................................cagucuugcuuugucacccaug...........    6    0    A54

.......................................uuuuugagacagucuugc........................    1    0    SW4

.........cugggugaugcagugagaugcu..................................................    1    0    j28

..........ugggugaugcagugagaugcuguc...............................................    1    0    H52
................................................cagucuugcuuugucacccau............    3    0    H52

.........cugggugaugcagugagaugcugu................................................    1    0    s03
```

Provisional ID	:	hsa_can_541
Score total	:	3.1
Score for star read(s)	:	3.9
Score for read counts	:	-1.8
Score for mfe	:	1.7
Score for randfold	:	
Score for cons. seed	:	-0.6
Total read count	:	8
Mature read count	:	7
Loop read count	:	0
Star read count	:	1

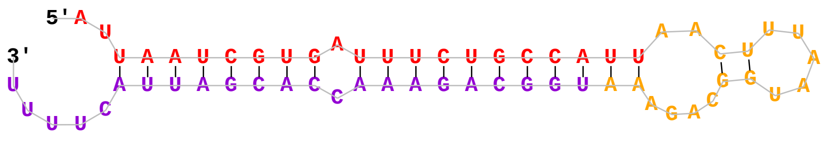

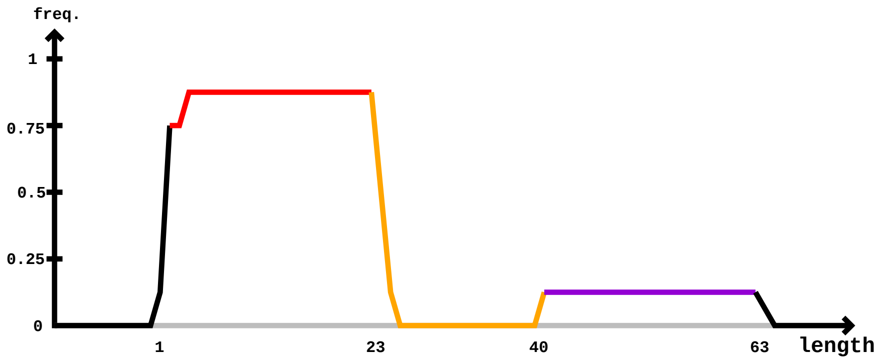

Mature **Star**

```
5'- uuggugcaaaauuaaucgugauuucugccauuaacuuuaaauggcagaaauggcagaaaccacgauuacuuuuguaccaacc  -3'   obs
    uuggugcaaaauuaaucgugauuucugccauuaacuuuaaauggcagaaauggcagaaaccacgauuacuuuuguaccaacc         exp
    ((((((((((.((((((((.(((((((((((..((.....))....))))))))))).)))))))).))))))))))..   reads   mm    sample
    ..........auuaaucgugauuucugccauu..................................................   1       0     j23

    ...........uaaucgugauuucugccauuaa.................................................   1       0     va2

    .........aauuaaucgugauuucugccauu..................................................   1       0     j19

    ..........auuaaucgugauuucugccauu..................................................   1       0     ku3

    ..........auuaaucgugauuucugccauu..................................................   1       0     li1

    ..........auuaaucgugauuucugccauu..................................................   1       0     ku6

    ..................................................uggcagaaaccacgauuacuuuu.........   1       0     per

    ..........auuaaucgugauuucugccauua.................................................   1       0     ku2
```

Provisional ID	:	hsa_can_573
Score total	:	58.7
Score for star read(s)	:	3.9
Score for read counts	:	53.7
Score for mfe	:	1.7
Score for randfold	:	
Score for cons. seed	:	-0.6
Total read count	:	117
Mature read count	:	22
Loop read count	:	0
Star read count	:	95

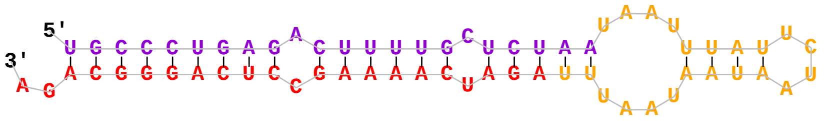

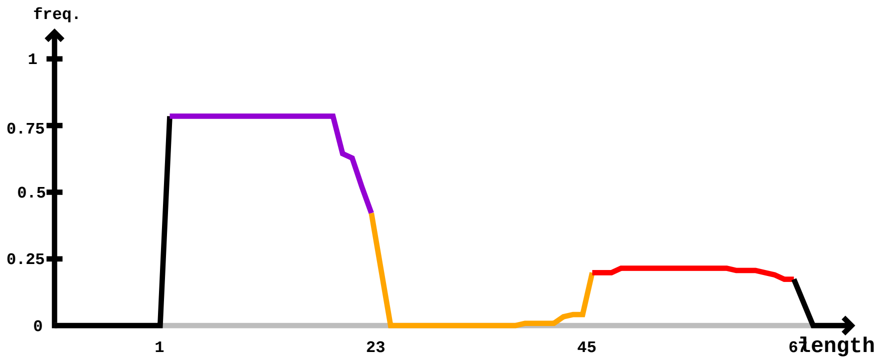

Star **Mature**

```
5'- aaugagaaucugcccugagacuuuugcucuaauaauuuauucuaauaauaauuuagaucaaaagccucagggcagauuuuc  -3'   obs
    aaugagaaucugcccugagacuuuugcucuaauaauuuauucuaauaauaauuuagaucaaaagccucagggcagauuuuc        exp
    ...((((((((((((((((.((((((.(((((....((((....))))....))))).)))))).))))))))))))))))     reads   mm   sample
    ..........ugcccugagacuuuugcucuaa.................................................     2       0    j23

    ..........ugcccugagacuuuugcu.....................................................     2       0    ya1

    ..........ugcccugagacuuuugcucuaa.................................................     1       0    s05

    ..........ugcccugagacuuuugcu.....................................................     1       0    s04

    ..........ugcccugagacuuuugcucuaa.................................................     4       0    va1

    ..........ugcccugagacuuuugcucu...................................................     1       0    s06

    ..........ugcccugagacuuuugcu.....................................................     3       0    HeL
    ..........ugcccugagacuuuugcucua..................................................     2       0    HeL
    ..........ugcccugagacuuuugcucuaa.................................................     1       0    HeL
    ......................................................agaucaaaagccucagggcaga.....     2       0    HeL

    ..........ugcccugagacuuuugcucuaa.................................................     1       0    s10

    ......................................................agaucaaaagccucagggcaga.....     1       0    NL2

    ..........ugcccugagacuuuugcucua..................................................     1       0    j13

    ..........ugcccugagacuuuugcucuaa.................................................     1       0    j03

    ..........ugcccugagacuuuugcucua..................................................     1       0    k01

    ..........ugcccugagacuuuugcucuaa.................................................     2       0    ku2

    ..........ugcccugagacuuuugcucu...................................................     2       0    U20
    ..........ugcccugagacuuuugcucuaa.................................................     2       0    U20
    ..........ugcccugagacuuuugcucuaau................................................     1       0    U20
    ...................................................uuuagaucaaaagccucagggc........     1       0    U20
```

Star Mature

```
aaugagaaucugcccugagacuuuugcucuaauaauuuauucuaauaauaauuuagaucaaaagccucagggcagauuuuc
......................................................agaucaaaagccucagggcaga.....    1    0    U20
.........................................................ucaaaagccucagggcaga.....    1    0    U20

..........ugcccugagacuuuugcucu...................................................    3    0    j10
..........ugcccugagacuuuugcucuaa.................................................    1    0    j10

..........ugcccugagacuuuugcucuaa.................................................    2    0    j21
...............................................auaauuuagaucaaaagccuca............    1    0    j21

..........ugcccugagacuuuugcucuaa.................................................    3    0    j04

..........ugcccugagacuuuugcucuaa.................................................    2    0    va2

..........ugcccugagacuuuugcucua..................................................    1    0    j31
..........ugcccugagacuuuugcucuaa.................................................    5    0    j31

..........ugcccugagacuuuugcucua..................................................    1    0    j22
..........ugcccugagacuuuugcucuaa.................................................    1    0    j22

......................................................agaucaaaagccucagggcaga.....    2    0    B14

..........ugcccugagacuuuugcucua..................................................    1    0    DLD

..........ugcccugagacuuuugcucuaa.................................................    2    0    j16

..........ugcccugagacuuuugcucuaa.................................................    2    0    ku3

..........ugcccugagacuuuugcu.....................................................    5    0    ya2

..........ugcccugagacuuuugcu.....................................................    1    0    NL3

..........ugcccugagacuuuugcucuaa.................................................    1    0    j11

..........ugcccugagacuuuugcucuaa.................................................    1    0    k06

..........ugcccugagacuuuugcucu...................................................    1    0    HEK
...................................................uuuagaucaaaagccucagggca.......    2    0    HEK
......................................................agaucaaaagccucagggcaga.....    8    0    HEK

..........ugcccugagacuuuugcucuaa.................................................    3    0    sh2

..........ugcccugagacuuuugcucu...................................................    1    0    j15
..........ugcccugagacuuuugcucuaa.................................................    1    0    j15

..........ugcccugagacuuuugcucu...................................................    2    0    j17
..........ugcccugagacuuuugcucua..................................................    2    0    j17
..........ugcccugagacuuuugcucuaa.................................................    1    0    j17

..........ugcccugagacuuuugcu.....................................................    1    0    NL1
..........ugcccugagacuuuugcuc....................................................    1    0    NL1

..........ugcccugagacuuuugcucu...................................................    2    0    MBM
..........ugcccugagacuuuugcucua..................................................    2    0    MBM
..........ugcccugagacuuuugcucuaa.................................................    1    0    MBM
......................................................agaucaaaagccucagggcaga.....    4    0    MBM

..........ugcccugagacuuuugcucuaa.................................................    1    0    li1
....................................................uuagaucaaaagccucaggg.........    1    0    li1

..........ugcccugagacuuuugcucua..................................................    1    0    li2
..........ugcccugagacuuuugcucuaa.................................................    1    0    li2

..........ugcccugagacuuuugcucuaa.................................................    1    0    va4

..........ugcccugagacuuuugcucuaa.................................................    3    0    va3

..........ugcccugagacuuuugcu.....................................................    2    0    A54
..........ugcccugagacuuuugcucuaa.................................................    3    0    A54

..........ugcccugagacuuuugcucu...................................................    1    0    SW4
```

```
          Star                                         Mature
aaugagaaucugcccugagacuuuugcucuaauaauuuauucuaauaauaauuuagaucaaaagccucagggcagauuuuc
..........ugcccugagacuuuugcucuaa.................................................    1    0    SW4
......................................................agaucaaaagccucagggcaga.....    1    0    SW4
.........................................................ucaaaagccucagggcaga.....    1    0    SW4

..........ugcccugagacuuuugcu.....................................................    2    0    ya4
..........ugcccugagacuuuugcuc....................................................    1    0    ya4
```

Provisional ID : hsa_can_575
Score total : 499.8
Score for star read(s) : 3.9
Score for read counts : 496.2
Score for mfe : 0.3
Score for randfold :
Score for cons. seed : -0.6
Total read count : 985
Mature read count : 977
Loop read count : 0
Star read count : 8

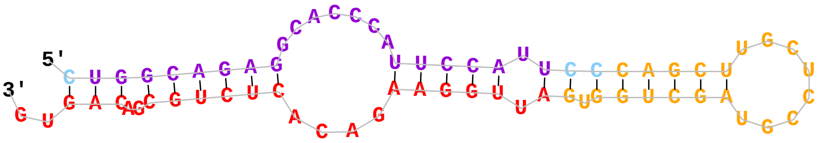

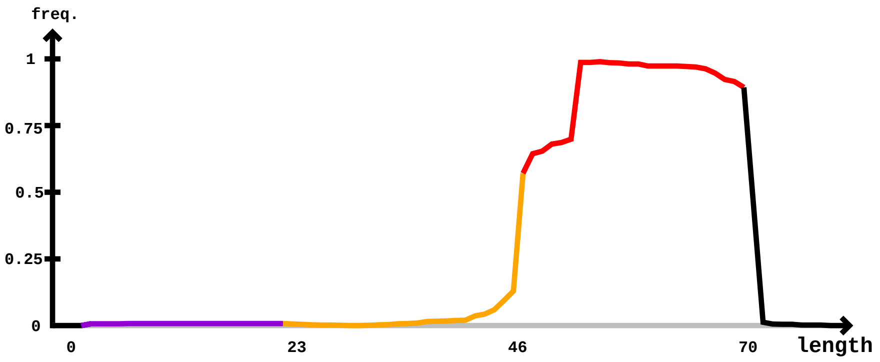


```
        Star                    Mature
5'- ccuggcagaggcacccauuccauucccagcuugcuccguagcuggugauuggaagacacucugcgacaguguucagucccu   -3'    obs
    ccuggcagaggcacccauuccauucccagcuugcuccguagcuggugauuggaagacacucugcgacaguguucagucccu          exp
    .(((((((((.......(((((.((((((((........)))))).)).)))))....))))))..)))............   reads   mm   sample
    ..........................................uggugauuggaagacacu.....................   1       0    ya1
    ..............................................gauuggaagacacucugcgacagug..........   7       0    ya1
    ...............................................auuggaagacacucugcgacagug..........   2       0    ya1
    ....................................................aagacacucugcgacagu...........   1       0    ya1

    ...............................................auuggaagacacucugcgacagug..........   7       0    j26

    ............................................gugauuggaagacacucugcgacagug..........   2       0    va1
    ..............................................gauuggaagacacucugcgacagugu.........   1       0    va1

    ..............................................gauuggaagacacucugcgacagug..........   1       0    ku5

    .....................................guagcuggugauuggaagaca.......................   1       0    k05
    ......................................uagcuggugauuggaagaca.......................   1       0    k05
    .............................................ugauuggaagacacucugcgaca.............   1       0    k05
    ...............................................auuggaagacacucugcgacag............   1       0    k05
    ...............................................auuggaagacacucugcgacagu...........   4       0    k05
    ...............................................auuggaagacacucugcgacagug..........   44      0    k05

    ..................................uccguagcuggugauuggaaga.........................   1       0    HeL
    ..............................................gauuggaagacacucugcgacagug..........   1       0    HeL
    .....................................................agacacucugcgacagug..........   17      0    HeL
    .....................................................agacacucugcgacagugu.........   1       0    HeL
    .......................................................acacucugcgacaguguu........   3       0    HeL

    ...............................................auuggaagacacucugcgacagug..........   1       0    ku4

    .............................................ugauuggaagacacucugcgac..............   1       0    s07
    .............................................ugauuggaagacacucugcgaca.............   1       0    s07

    ...............................................auuggaagacacucugcgacagu...........   1       0    k08
    ...............................................auuggaagacacucugcgacagug..........   18      0    k08
    ................................................uuggaagacacucugcgacagug..........   2       0    k08
```

Star **Mature**

```
ccuggcagaggcacccauuccauucccagcuugcuccguagcuggugauuggaagacacucugcgacaguguucagucccu

..uggcagaggcacccauuccauucc.......................................................   1   0   s10

...............................................auuggaagacacucugcgacagug..........   1   0   j10
.....................................................agacacucugcgacagug..........   1   0   j10

.....................................guagcuggugauuggaaga.........................   4   0   j04
...............................................auuggaagacacucugcgacagug..........   1   0   j04
.....................................................agacacucugcgacagugu.........   2   0   j04

.............................................ugauuggaagacacucugcgaca.............   1   0   k14
...............................................auuggaagacacucugcgacagu...........   1   0   k14
...............................................auuggaagacacucugcgacagug..........   7   0   k14

.........................................cuggugauuggaagacacucugc.................   1   0   va2
.............................................ugauuggaagacacucugcgaca.............   1   0   va2
...............................................auuggaagacacucugcgacagug..........   1   0   va2
................................................uuggaagacacucugcgacagug..........   8   0   va2

.....................................................agacacucugcgacagug..........   1   0   j27

.............................................ugauuggaagacacucugcgac..............   1   0   H52
...............................................auuggaagacacucugcgacagug..........   1   0   H52
.....................................................agacacucugcgacagug..........   4   0   H52

........................................gcuggugauuggaagacacu.....................   1   0   k09
.............................................ugauuggaagacacucugc.................   1   0   k09
...............................................auuggaagacacucugcgaca.............   1   0   k09
...............................................auuggaagacacucugcgacag............   1   0   k09
...............................................auuggaagacacucugcgacagu...........   6   0   k09
...............................................auuggaagacacucugcgacagug..........   72  0   k09
...............................................auuggaagacacucugcgacagugu.........   1   0   k09
................................................uuggaagacacucugcgacagug..........   3   0   k09
..................................................ggaagacacucugcgacagug..........   1   0   k09

..........................................uggugauuggaagacacucugcg................   1   0   B14
....................................................aagacacucugcgacagug..........   3   0   B14
.....................................................agacacucugcgacagug..........   23  0   B14

..................................................ggaagacacucugcgacagug..........   1   0   j16

...............................................auuggaagacacucugcgacagug..........   2   0   j09
................................................uuggaagacacucugcgacagug..........   2   0   j09
..................................................ggaagacacucugcgacagug..........   2   0   j09

...............................................auuggaagacacucugcgacagug..........   16  0   j18
.................................................uggaagacacucugcgacagug..........   1   0   j18
..................................................ggaagacacucugcgacagug..........   1   0   j18

..............................................gauuggaagacacucugcgacagug..........   2   0   ku3
...............................................auuggaagacacucugcgacagug..........   1   0   ku3

..............................................gauuggaagacacucugcgacagug..........   2   0   j11
...............................................auuggaagacacucugcgacagug..........   4   0   j11
................................................uuggaagacacucugcgacagug..........   2   0   j11
................................................uuggaagacacucugcgacagugu.........   1   0   j11

..uggcagaggcacccauuccauu.........................................................   1   0   s12

..........................................uggugauuggaagacacu.....................   1   0   ya3
.............................................ugauuggaagacacucugcgaca.............   1   0   ya3
...............................................auuggaagacacucugcgacagug..........   1   0   ya3
..................................................ggaagacacucugcgacagug..........   1   0   ya3
...................................................gaagacacucugcgacagug..........   1   0   ya3

..............................................gauuggaagacacucugcgacagug..........   2   0   la1
...............................................auuggaagacacucugcgacagug..........   2   0   la1

..............................................gauuggaagacacucugcgacagug..........   1   0   HEK
................................................uuggaagacacucugcgacagug..........   6   0   HEK
```

Star **Mature**

ccuggcagaggcacccauuccauucccagcuugcuccguagcugguggauuggaagacacucugcgacaguguucagucccu			
....gaagacacucugcgacagug..........	1	0	HEK
....aagacacucugcgacagug..........	1	0	HEK
....agacacucugcgacagug..........	35	0	HEK
....agacacucugcgacagugu.........	2	0	HEK
....agacacucugcgacaguguucag.....	2	0	HEK
....cacucugcgacaguguuc.......	1	0	HEK
....agacacucugcgacagug..........	3	0	j17
....ugauuggaagacacucugcgaca.............	1	0	j15
.................................cuccguagcuggugauuggaagaca.......................	1	0	la2
....agcuggugauuggaagac........................	1	0	la2
....auuggaagacacucugcgacagug..........	2	0	j12
................................gcuccguagcuggugauuggaagacacucugcgacagug..........	1	0	li2
....uggugauuggaagacacucugcga...............	1	0	li2
....uggugauuggaagacacucugcgac..............	1	0	li2
....gugauuggaagacacucugcgac..............	1	0	li2
....ugauuggaagacacucugcgac..............	2	0	li2
....ugauuggaagacacucugcgaca.............	5	0	li2
....gauuggaagacacucugcgacagug..........	1	0	li2
....auuggaagacacucugcgacagug..........	3	0	li2
....uuggaagacacucugcgacagug..........	1	0	li2
....gaagacacucugcgacagug..........	1	0	li2
....aagacacucugcgacagug..........	2	0	li2
....agacacucugcgacagug..........	1	0	li2
....ggaagacacucugcgacagug..........	2	0	va4
....uggugauuggaagacacucugcgacagug..........	1	0	va3
....ggugauuggaagacacucugcgacagug..........	1	0	va3
....gugauuggaagacacucugcgacagug..........	3	0	va3
....gugauuggaagacacucugcgacagugu.........	2	0	va3
....ugauuggaagacacucugcgacagug..........	2	0	va3
....gauuggaagacacucugcgacagug..........	1	0	va3
....auuggaagacacucugcgacagug..........	1	0	va3
....ggaagacacucugcgacagug..........	1	0	va3
....aagacacucugcgacagug..........	1	0	va3
....agacacucugcgacagug..........	12	0	SW4
....agacacucugcgacagugu.........	2	0	SW4
.....................................guagcuggugauuggaagacacu.....................	1	0	ya4
....gcuggugauuggaagacacu.....................	1	0	ya4
....uggugauuggaagacacu.....................	1	0	ya4
....ggugauuggaagacacucugcgacag............	1	0	ya4
....ugauuggaagacacucugcgac..............	1	0	ya4
....ugauuggaagacacucugcgacagug..........	1	0	ya4
....gauuggaagacacucugcgacagug..........	2	0	ya4
....ggaagacacucugcgacagug..........	1	0	ya4
....agacacucugcgacagug..........	1	0	ya4
......agaggcacccauuccauucccag..	1	0	ku1
....auuggaagacacucugcgacagug..........	3	0	ku1
....uuggaagacacucugcgacagug..........	1	0	ku1
....agacacucugcgacagug..........	1	0	ku1
....auuggaagacacucugcgacagug..........	1	0	j23
..uggcagaggcacccauuccauu...	1	0	s05
....gugauuggaagacacucugcgac..............	1	0	s05
....gauuggaagacacucugcgacagug..........	1	0	s05
....ggaagacacucugcgacagug..........	1	0	j24
....auuggaagacacucugcgacagug..........	1	0	k11
....gauuggaagacacucugcgacagug..........	1	0	ta1

```
  Star                                        Mature
ccuggcagaggcacccauuccauucccagcuugcuccguagcugguggauuggaagacacucugcgacaguguucagucccu
.................................................uggaagacacucugcgacagug..........    1    0    ta1
.....................................................agacacucugcgacagug..........    2    0    ta1

..............................................gauuggaagacacucugcgacagug..........    3    0    NL2
...............................................auuggaagacacucugcgacagug..........    2    0    NL2
...................................................gaagacacucugcgacagug..........    1    0    NL2

.....................................................agacacucugcgacagug..........    3    0    j13

...............................................auuggaagacacucugcgacag............    1    0    k10
...............................................auuggaagacacucugcgacagu...........    1    0    k10
...............................................auuggaagacacucugcgacagug..........    8    0    k10
...............................................auuggaagacacucugcgacagugu.........    1    0    k10
................................................uuggaagacacucugcgacagug..........    2    0    k10
.................................................uggaagacacucugcgacagug..........    1    0    k10

.................................................uggaagacacucugcgacagug..........    1    0    j19

............................................gugauuggaagacacucugcgacagug..........    1    0    j03
................................................uuggaagacacucugcgacagug..........    3    0    j03

................................................uuggaagacacucugcgacagug..........    1    0    k01

...............................................auuggaagacacucugcgacagug..........    5    0    j25
................................................uuggaagacacucugcgacagug..........    1    0    j25
..................................................ggaagacacucugcgacagug..........    1    0    j25
...................................................gaagacacucugcgacagug..........    1    0    j25

...............................................auuggaagacacucugcgacagug..........    2    0    k04
................................................uuggaagacacucugcgacagug..........    3    0    k04
..................................................ggaagacacucugcgacagug..........    1    0    k04

...........................................ggugauuggaagacacucugcgacagug..........    1    0    per
............................................gugauuggaagacacucugcgacagug..........    1    0    per
................................................uuggaagacacucugcgacagug..........    2    0    per

...............................................auuggaagacacucugcgacagug..........    5    0    s01

.....................................................agacacucugcgacagug..........    12   0    U20
.....................................................agacacucugcgacaguguucagucc..    2    0    U20

..uggcagaggcacccauuccauuccca.....................................................    1    0    s11

...............................................auuggaagacacucugcgacagug..........    1    0    j07

.............................................ugauuggaagacacucugcgaca.............    1    0    k02
...............................................auuggaagacacucugcgacagug..........    4    0    k02
................................................uuggaagacacucugcgacagug..........    1    0    k02
.....................................................agacacucugcgacagug..........    1    0    k02

...............................................auuggaagacacucugcgacagug..........    1    0    j14

..uggcagaggcacccauuccauu.........................................................    3    0    j28
................................................uuggaagacacucugcgacag............    1    0    j28

.............................................ugauuggaagacacucugcga...............    1    0    s08
.............................................ugauuggaagacacucugcgaca.............    1    0    s08

..........................................uggugauuggaagacacucugcga...............    1    0    DLD
.....................................................agacacucugcgacagug..........    4    0    DLD

...............................................auuggaagacacucugcgacag............    2    0    k03
...............................................auuggaagacacucugcgacagu...........    1    0    k03
...............................................auuggaagacacucugcgacagug..........    51   0    k03
...............................................auuggaagacacucugcgacagugu.........    1    0    k03
................................................uuggaagacacucugcgacagu...........    2    0    k03
................................................uuggaagacacucugcgacagug..........    1    0    k03

...............................................auuggaagacacucugcgacagug..........    4    0    NL3

..........................................uggugauuggaagacacu.....................    2    0    ya2
```

Star **Mature**

```
ccuggcagaggcacccauuccauucccagcuugcuccguagcugguguauuggaagacacucugcgacaguguucagucccu
............................................gugauuggaagacacucugcgac..............   1    0    ya2
.............................................ugauuggaagacacucugcgac..............   1    0    ya2
..............................................gauuggaagacacucugcgacagug..........   2    0    ya2
...............................................auuggaagacacucugcgacagug..........   1    0    ya2
..................................................ggaagacacucugcgacagug..........   2    0    ya2

.............................................ugauuggaagacacucugcgac..............   1    0    s02

....................................cguagcuggugauuggaagaca.......................   1    0    k06
............................................gugauuggaagacacucugcgacagug..........   1    0    k06
...............................................auuggaagacacucugcgacagu...........   2    0    k06
...............................................auuggaagacacucugcgacagug..........   57   0    k06
...............................................auuggaagacacucugcgacagugu.........   1    0    k06
.................................................uggaagacacucugcgacagug..........   1    0    k06
...................................................gaagacacucugcgacagug..........   1    0    k06

..........................................uggugauuggaagacacucugcga...............   1    0    sh2
............................................gugauuggaagacacucugcgaca.............   1    0    sh2
.............................................ugauuggaagacacucugcgac..............   1    0    sh2
.............................................ugauuggaagacacucugcgaca.............   2    0    sh2
..............................................gauuggaagacacucugcgacagug..........   2    0    sh2
...............................................auuggaagacacucugcgacagu...........   1    0    sh2
...............................................auuggaagacacucugcgacagug..........   48   0    sh2
...............................................auuggaagacacucugcgacaguguu........   3    0    sh2
................................................uuggaagacacucugcgaca.............   2    0    sh2
................................................uuggaagacacucugcgacagug..........   6    0    sh2
.................................................uggaagacacucugcgacagug..........   1    0    sh2
..................................................ggaagacacucugcgacagug..........   7    0    sh2
....................................................aagacacucugcgacagug..........   2    0    sh2
.....................................................agacacucugcgacagug..........   25   0    sh2

....................................cguagcuggugauuggaagac........................   1    0    j29
.................................................uggaagacacucugcgacagug..........   1    0    j29

...............................................auuggaagacacucugcgacagug..........   3    0    j01
................................................uuggaagacacucugcgacagug..........   3    0    j01

...................................ccguagcuggugauugga............................   1    0    MCF
...........................................ggugauuggaagacacucugcgacagug..........   1    0    MCF
............................................gugauuggaagacacucugcgaca.............   1    0    MCF
................................................uuggaagacacucugcgacagug..........   7    0    MCF
................................................uuggaagacacucugcgacaguguu........   1    0    MCF
.....................................................agacacucugcgacagug..........   72   0    MCF
.....................................................agacacucugcgacagugu.........   1    0    MCF

............................................gugauuggaagacacucugcgacagug..........   1    0    NL1
...............................................auuggaagacacucugcgacagu...........   1    0    NL1
...............................................auuggaagacacucugcgacagug..........   4    0    NL1
.................................................uggaagacacucugcgacagu...........   1    0    NL1
..................................................ggaagacacucugcgacagug..........   3    0    NL1

...............................................auuggaagacacucugcgacagug..........   1    0    MBM
.....................................................agacacucugcgacagug..........   17   0    MBM

...............................................auuggaagacacucugcgacagu...........   1    0    k07
...............................................auuggaagacacucugcgacagug..........   6    0    k07
................................................uuggaagacacucugcgacagug..........   3    0    k07

...............................................auuggaagacacucugcgacagug..........   3    0    sh1
...............................................auuggaagacacucugcgacagugu.........   1    0    sh1
................................................uuggaagacacucugcgacagug..........   1    0    sh1

...............................ugcuccguagcuggugauuggaagacacucugcgacagug..........   1    0    li1
................................gcuccguagcuggugauuggaagacacucugcgacagug..........   1    0    li1
..................................uccguagcuggugauuggaagacacucugcgacagug..........   2    0    li1
..........................................uggugauuggaagacacucugcg................   1    0    li1
..........................................uggugauuggaagacacucugcga...............   2    0    li1
..........................................uggugauuggaagacacucugcgac..............   2    0    li1
...........................................ggugauuggaagacacucugcgacagug..........   1    0    li1
............................................gugauuggaagacacucugcgacagug..........   1    0    li1
```

```
Star                                        Mature
ccuggcagaggcacccauuccauucccagcuugcuccguagcugguguauuggaagacacucugcgacaguguucagucccu
.............................................ugauuggaagacacucugcgac..............   3   0   li1
.............................................ugauuggaagacacucugcgaca.............   5   0   li1
..............................................gauuggaagacacucugcgacagug..........   4   0   li1
................................................uuggaagacacucugcgacagug..........   5   0   li1
.................................................uggaagacacucugcgacagug..........   2   0   li1
..................................................ggaagacacucugcgacagug..........   3   0   li1
....................................................aagacacucugcgacagug..........   3   0   li1
.....................................................agacacucugcgacagug..........   26  0   li1
.....................................................agacacucugcgacagugu.........   1   0   li1
.........................................................acucugcgacaguguucag.....   1   0   li1

...............................................auuggaagacacucugcgacag............   1   0   k13
...............................................auuggaagacacucugcgacagug..........   22  0   k13
................................................uuggaagacacucugcgacagug..........   4   0   k13

...............................................auuggaagacacucugcgacagug..........   1   0   j20

..........................................uggugauuggaagacacucugcga...............   1   0   A54
...........................................ggugauuggaagacacucugcgacagug..........   2   0   A54
..............................................gauuggaagacacucugcgacagug..........   3   0   A54
...............................................auuggaagacacucugcgacagug..........   1   0   A54
................................................uuggaagacacucugcgacagugu.........   2   0   A54
.....................................................agacacucugcgacagug..........   26  0   A54
.....................................................agacacucugcgacagugu.........   3   0   A54

..............................................gauuggaagacacucugcgacagug..........   1   0   j30

...............................................auuggaagacacucugcgacagug..........   6   0   k12
...............................................auuggaagacacucugcgacagugu.........   1   0   k12
```

Provisional ID	:	hsa_can_629
Score total	:	602.5
Score for star read(s)	:	3.9
Score for read counts	:	593.6
Score for mfe	:	2
Score for randfold	:	
Score for cons. seed	:	3
Total read count	:	1176
Mature read count	:	1146
Loop read count	:	0
Star read count	:	30

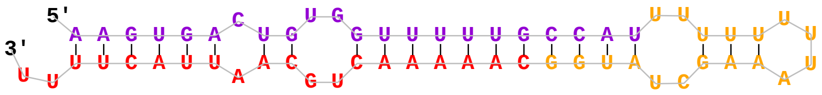

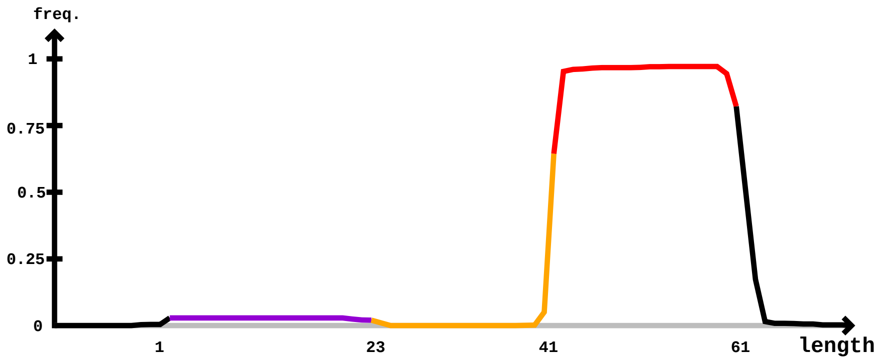

Star **Mature**

```
5'- guuggugcaaaagugacugugguuuuugccauuuuuuuuuaaagcuaugg caaaaacugcaauuacuuuugcaccaaccua  -3'   obs
    guuggugcaaaagugacugugguuuuugccauuuuuuuuuaaagcuaugg caaaaacugcaauuacuuuugcaccaaccua        exp
    guuggugcaaaagugacugugguuuuugccauuuuuuuuuaaagcuauggcaaaaacugcaauuacuuuugcaccaaccua         known
    ((((((((((((((((.((..(((((((((((..(((....)))..)))))))))))..)).))))))))))))))))...    reads   mm   sample
    ..................................................caaaaacugcaauuacuu.............    1       0    ya1
    ..................................................caaaaacugcaauuacuuu............    12      0    ya1
    ..................................................caaaaacugcaauuacuuuu...........    15      0    ya1
    ..................................................caaaaacugcaauuacuuuug..........    2       0    ya1
    ...................................................aaaaacugcaauuacuu.............    3       0    ya1
    ...................................................aaaaacugcaauuacuuu............    7       0    ya1
    ...................................................aaaaacugcaauuacuuuug..........    2       0    ya1
    ....................................................aaaacugcaauuacuuuu...........    4       0    ya1
    ....................................................aaaacugcaauuacuuuugc.........    2       0    ya1

    .......caaaagugacugugguuuuugc....................................................    1       0    va1
    ..........aagugacugugguuuuugccau.................................................    4       0    va1
    .................................................gcaaaaacugcaauuacuuuug..........    6       0    va1
    ..................................................caaaaacugcaauuacuuuug..........    5       0    va1
    ..................................................caaaaacugcaauuacuuuugc.........    2       0    va1
    ...................................................aaaaacugcaauuacuuuug..........    1       0    va1

    ..................................................caaaaacugcaauuacuuuug..........    1       0    ku5

    ..................................................caaaaacugcaauuacuuuugc.........    1       0    s06
    .....................................................aaacugcaauuacuuuugcacca.....    1       0    s06

    ..................................................caaaaacugcaauuacuuuug..........    2       0    HeL
    ..................................................caaaaacugcaauuacuuuugc.........    5       0    HeL
    ...................................................aaaaacugcaauuacuuuugc.........    8       0    HeL
    ...................................................aaaaacugcaauuacuuuugca........    5       0    HeL

    ..................................................caaaaacugcaauuacuuuug..........    1       0    ku4

    .................................................gcaaaaacugcaauuacuuuug..........    1       0    s07

    .................................................gcaaaaacugcaauuacuuuug..........    1       0    s10
```

Star **Mature**

guuggugcaaaagugacugugguuuuugccauuuuuuuuuaaagcuauggcaaaaacugcaauuacuuuugcaccaaccua

....gcaaaaacugcaauuacuuuug..........	1	0	k08
....caaaaacugcaauuacuuuugc.........	1	0	k08
....aaaaacugcaauuacuuuug..........	1	0	k08
....gcaaaaacugcaauuacuuuug..........	1	0	ta3
....caaaaacugcaauuacuuuu...........	2	0	ta3
....gcaaaaacugcaauuacuuuug..........	1	0	j10
....aaaaacugcaauuacuuuug..........	2	0	j10
....caaaaacugcaauuacuuuug..........	4	0	j04
....aaaaacugcaauuacuuuug..........	2	0	k14
....aaaacugcaauuacuuuug..........	1	0	j31
....caaaaacugcaauuacuuu............	1	0	j27
....caaaaacugcaauuacuuuug..........	3	0	j27
....aaaaacugcaauuacuuu............	4	0	j27
....aaaaacugcaauuacuuuu...........	3	0	j27
....aaaaacugcaauuacuuuug..........	7	0	j27
....caaaaacugcaauuacuuuugc.........	1	0	s03
....aacugcaauuacuuuugcacca.....	1	0	s03
....gcaaaaacugcaauuacuuuug..........	2	0	H52
....caaaaacugcaauuacuuuugc.........	3	0	H52
....gcaaaaacugcaauuacuuuug..........	1	0	j22
....caaaaacugcaauuacuuuu...........	1	0	j22
....caaaaacugcaauuacuuuug..........	12	0	j22
....caaaaacugcaauuacuuuugc.........	3	0	j22
....aaaaacugcaauuacuuuu...........	2	0	j22
....aaaaacugcaauuacuuuug..........	2	0	j22
....aaaaacugcaauuacuuuu...........	1	0	k09
....uggcaaaaacugcaauuacuu.............	1	0	B14
....gcaaaaacugcaauuacuuuug..........	2	0	B14
....caaaaacugcaauuacuuuugc.........	5	0	B14
....aaaaacugcaauuacuuuugc.........	1	0	B14
....aacugcaauuacuuuugc.........	1	0	B14
....caaaaacugcaauuacuuuug..........	3	0	j09
....aaaaacugcaauuacuuuu...........	2	0	j09
....aaaaacugcaauuacuuuug..........	1	0	j09
....gcaaaaacugcaauuacuuuug..........	1	0	ku3
....caaaaacugcaauuacuuuug..........	1	0	ku3
....gcaaaaacugcaauuacuuuug..........	2	0	j18
....gcaaaaacugcaauuacuuuug..........	1	0	j16
....caaaaacugcaauuacuuuu...........	1	0	ku6
....gcaaaaacugcaauuacuuuug..........	1	0	s12
....caaaaacugcaauuacuu.............	2	0	ya3
....caaaaacugcaauuacuuu............	6	0	ya3
....caaaaacugcaauuacuuuu...........	11	0	ya3
....caaaaacugcaauuacuuuug..........	4	0	ya3
....aaaaacugcaauuacuuu............	12	0	ya3
....aaaaacugcaauuacuuuu...........	18	0	ya3
....aaaaacugcaauuacuuuug..........	3	0	ya3
....aaaaacugcaauuacuuuugc.........	1	0	ya3
....aaaacugcaauuacuuuug..........	1	0	ya3
....gcaaaaacugcaauuacuuuug..........	1	0	HEK

```
          Star                                   Mature
guuggugcaaaagugacugugguuuuugccauuuuuuuuaaagcuauggcaaaaacugcaauuacuuuugcaccaaccua
..................................................caaaaacugcaauuacuuuug..........   1     0     HEK
..................................................caaaaacugcaauuacuuuugc.........   13    0     HEK
...................................................aaaaacugcaauuacuuuugc.........   3     0     HEK

..........aagugacugugguuuuugc....................................................   1     0     j17
..........aagugacugugguuuuugccau.................................................   2     0     j17
.................................................gcaaaaacugcaauuacuuuug..........   2     0     j17
..................................................caaaaacugcaauuacuuuu...........   2     0     j17
..................................................caaaaacugcaauuacuuuug..........   1     0     j17

.................................................gcaaaaacugcaauuacuuuug..........   1     0     j15
..................................................caaaaacugcaauuacuuuu...........   1     0     j15
..................................................caaaaacugcaauuacuuuug..........   1     0     j15
...................................................aaaaacugcaauuacuuuug..........   1     0     j15

..................................................caaaaacugcaauuacuuuug..........   1     0     la2
..................................................caaaaacugcaauuacuuuugc.........   1     0     la2

.................................................gcaaaaacugcaauuacuuuug..........   2     0     j12
...................................................aaaaacugcaauuacuuuu...........   1     0     j12
...................................................aaaaacugcaauuacuuuug..........   1     0     j12

..................................................caaaaacugcaauuacuuuug..........   1     0     li2
..................................................caaaaacugcaauuacuuuugc.........   14    0     li2
...................................................aaaaacugcaauuacuuuugc.........   1     0     li2

.................................................gcaaaaacugcaauuacuuuug..........   1     0     va4
..................................................caaaaacugcaauuacuuuugc.........   1     0     va4

..........aagugacugugguuuuugccau.................................................   1     0     va3
................................................ggcaaaaacugcaauuacuuuu...........   1     0     va3
.................................................gcaaaaacugcaauuacuuuug..........   2     0     va3
..................................................caaaaacugcaauuacuuuug..........   1     0     va3
..................................................caaaaacugcaauuacuuuugc.........   1     0     va3
....................................................aaaacugcaauuacuuuugc.........   1     0     va3

..................................................caaaaacugcaauuacuuuugc.........   5     0     SW4
......................................................aacugcaauuacuuuugc.........   1     0     SW4

.................................................gcaaaaacugcaauuacuuu............   2     0     ya4
..................................................caaaaacugcaauuacu..............   7     0     ya4
..................................................caaaaacugcaauuacuu.............   8     0     ya4
..................................................caaaaacugcaauuacuuuu...........   23    0     ya4
...................................................aaaaacugcaauuacuu.............   6     0     ya4
...................................................aaaaacugcaauuacuuuug..........   3     0     ya4

..................................................caaaaacugcaauuacuuuug..........   2     0     ku1
..................................................caaaaacugcaauuacuuuugc.........   1     0     ku1

.................................................gcaaaaacugcaauuacuuuug..........   12    0     j23
..................................................caaaaacugcaauuacuuuu...........   1     0     j23
..................................................caaaaacugcaauuacuuuug..........   7     0     j23
..................................................caaaaacugcaauuacuuuugc.........   1     0     j23
...................................................aaaaacugcaauuacuuuu...........   1     0     j23
...................................................aaaaacugcaauuacuuuug..........   1     0     j23

...................................................aaaaacugcaauuacuuuu...........   1     0     s05

..................................................caaaaacugcaauuacuuuug..........   1     0     ta2

.................................................gcaaaaacugcaauuacuuuu...........   1     0     NL2
..................................................caaaaacugcaauuacu..............   8     0     NL2
..................................................caaaaacugcaauuacuu.............   15    0     NL2
..................................................caaaaacugcaauuacuuuu...........   77    0     NL2
..................................................caaaaacugcaauuacuuuug..........   36    0     NL2
..................................................caaaaacugcaauuacuuuugc.........   7     0     NL2
...................................................aaaaacugcaauuacuu.............   14    0     NL2
...................................................aaaaacugcaauuacuuuu...........   40    0     NL2
...................................................aaaaacugcaauuacuuuug..........   32    0     NL2

.................................................gcaaaaacugcaauuacuuuug..........   1     0     j13
```

Star **Mature**

guuggugcaaaagugacugugguuuuugccauuuuuuuuuaaagcuauggcaaaaacugcaauuacuuuugcaccaaccua

....caaaaacugcaauuacuuuu...........	2	0	j19
....caaaaacugcaauuacuuuug..........	4	0	j19
....aaaaacugcaauuacuuuug..........	1	0	j19
....caaaaacugcaauuacuuu............	1	0	j25
....caaaaacugcaauuacuuuu...........	1	0	j25
....aauuacuuuugcaccaaccua	2	0	per
....gcaaaaacugcaauuacuuuug..........	1	0	s01
....aaaaacugcaauuacuuuu...........	2	0	s01
....caaaaacugcaauuacuuu............	1	0	ku2
....caaaaacugcaauuacuuuug..........	2	0	ku2
....caaaaacugcaauuacuuuug..........	3	0	U20
....caaaaacugcaauuacuuuugc.........	12	0	U20
....aaaaacugcaauuacuuuug..........	1	0	U20
....aaaaacugcaauuacuuuugc.........	6	0	U20
....aaaaacugcaauuacuuuugca........	2	0	U20
....acugcaauuacuuuugcaccaac...	2	0	U20
.......caaaagugacugugguuuuugc..	1	0	j21
........aaaagugacugugguuuuugcc...	1	0	j21
..........aagugacugugguuuuugc..	2	0	j21
..........aagugacugugguuuuugcc...	1	0	j21
..........aagugacugugguuuuugcca..	1	0	j21
..........aagugacugugguuuuugccau...	12	0	j21
....gcaaaaacugcaauuacuuuug..........	3	0	j21
....caaaaacugcaauuacuuuug..........	1	0	j21
....caaaaacugcaauuacuuuugc.........	3	0	j21
....caaaaacugcaauuacuuuug..........	1	0	j14
....gcaaaaacugcaauuacuuuug..........	1	0	s08
....gcaaaaacugcaauuacuuuug..........	1	0	DLD
....caaaaacugcaauuacuuuug..........	2	0	DLD
....caaaaacugcaauuacuuuugc.........	6	0	DLD
....caaaaacugcaauuacuuu............	1	0	k03
....aaaaacugcaauuacuuuug..........	1	0	k03
....caaaaacugcaauuacuu.............	7	0	NL3
....caaaaacugcaauuacuuu............	36	0	NL3
....caaaaacugcaauuacuuuu...........	113	0	NL3
....caaaaacugcaauuacuuuug..........	34	0	NL3
....caaaaacugcaauuacuuuugc.........	7	0	NL3
....aaaaacugcaauuacuuu............	7	0	NL3
....aaaaacugcaauuacuuuu...........	60	0	NL3
....aaaaacugcaauuacuuuug..........	36	0	NL3
....aaaaacugcaauuacuuuugc.........	2	0	NL3
....caaaaacugcaauuacuu.............	6	0	ya2
....caaaaacugcaauuacuuu............	3	0	ya2
....caaaaacugcaauuacuuuu...........	4	0	ya2
....aaaaacugcaauuacuuu............	7	0	ya2
....aaaaacugcaauuacuuuu...........	4	0	ya2
....aaaaacugcaauuacuuuug..........	1	0	ya2
....caauuacuuuugcaccaac...	1	0	ya2
....aauuacuuuugcaccaac...	1	0	ya2
....gcaaaaacugcaauuacuuuug..........	1	0	j29
....caaaaacugcaauuacuuuugc.........	1	0	j29
....aaaaacugcaauuacuuuug..........	4	0	j29
....caaaaacugcaauuacuuuugc.........	11	0	sh2
....aaaaacugcaauuacuuuugc.........	2	0	sh2

Star **Mature**

guuggugcaaaagugacugugguuuuugccauuuuuuuuuaaagcuauggcaaaaacugcaauuacuuuugcaccaaccua			
....uuacuuuugcaccaaccua	1	0	sh2
....gcaaaaacugcaauuacuuuug..........	1	0	MCF
....caaaaacugcaauuacuu.............	1	0	MCF
....caaaaacugcaauuacuuu............	4	0	MCF
....caaaaacugcaauuacuuuug..........	2	0	MCF
....caaaaacugcaauuacuuuugc.........	15	0	MCF
....caaaaacugcaauuacuuuugca........	1	0	MCF
....aaaaacugcaauuacuuuug..........	2	0	MCF
....aacugcaauuacuuuugc.........	1	0	MCF
....caaaaacugcaauuacuuuug..........	1	0	j01
....gcaaaaacugcaauuacuuuug..........	1	0	NL1
....caaaaacugcaauuacuu.............	5	0	NL1
....caaaaacugcaauuacuuu............	7	0	NL1
....caaaaacugcaauuacuuuug..........	19	0	NL1
....caaaaacugcaauuacuuuugc.........	3	0	NL1
....aaaaacugcaauuacuuu............	4	0	NL1
....aaaaacugcaauuacuuuug..........	21	0	NL1
....aaaaacugcaauuacuuuugc.........	3	0	NL1
....caaaaacugcaauuacuuuugc.........	6	0	MBM
....aaaaacugcaauuacuuuugc.........	3	0	MBM
....caaaaacugcaauuacuuuug..........	1	0	sh1
.......caaaagugacugugguuuuugcc...	2	0	li1
..........aagugacugugguuuuugccau...	4	0	li1
..........aagugacugugguuuuugccauu..	1	0	li1
....caaaaacugcaauuacuuuug..........	1	0	li1
....caaaaacugcaauuacuuuugc.........	13	0	li1
....aaaaacugcaauuacuuuugc.........	1	0	li1
....aaaaacugcaauuacuuuug..........	1	0	k13
....gcaaaaacugcaauuacuuuug..........	1	0	j20
....caaaaacugcaauuacuuu............	1	0	j20
....caaaaacugcaauuacuuuug..........	4	0	j20
....aaaaacugcaauuacuuu............	1	0	j20
....aaaaacugcaauuacuuuug..........	2	0	j20
....caaaaacugcaauuacuuu............	2	0	A54
....caaaaacugcaauuacuuuugc.........	8	0	A54
....gcaaaaacugcaauuacuuuug..........	2	0	k12
....caaaaacugcaauuacuu.............	1	0	k12
....caaaaacugcaauuacuuuugc.........	1	0	k12
....aaacugcaauuacuuuugcacc......	1	0	k12

Provisional ID	:	hsa_can_645
Score total	:	10.9
Score for star read(s)	:	3.9
Score for read counts	:	5.2
Score for mfe	:	2.4
Score for randfold	:	
Score for cons. seed	:	-0.6
Total read count	:	22
Mature read count	:	18
Loop read count	:	0
Star read count	:	4

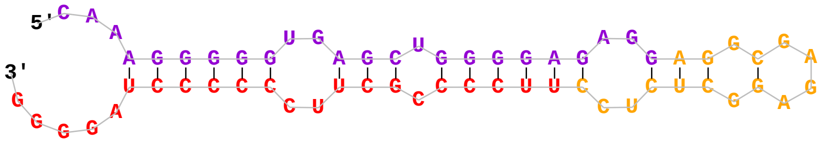

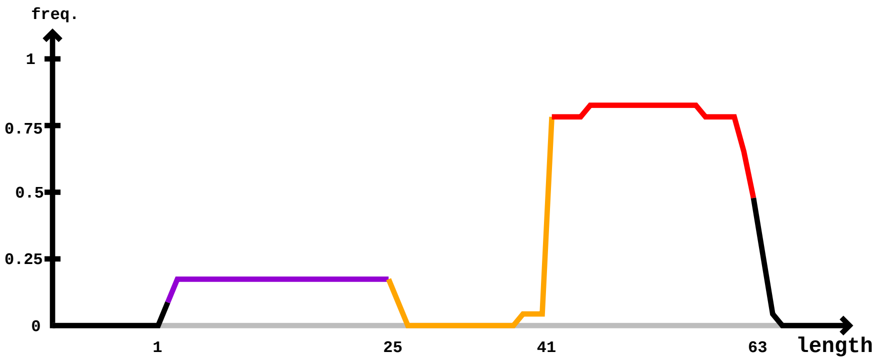

Star **Mature**

```
5'- uuucucagcccaaaggggugagcuggggagaggaggcgagaggcucuccuuccccgcuuccccccuagggguugagggcc  -3'   obs
    uuucucagcccaaaggggugagcuggggagaggaggcgagaggcucuccuuccccgcuuccccccuagggguugagggcc        exp
    .((((((((((..((((((..(((.((((((..(((.(....).)))..)))))).)))..))))))..))))))))))..  reads   mm   sample
    ...........aaaggggugagcuggggagagg...............................................  1       0    j12

    .................................................uuccccgcuuccccccuaggg..........  1       0    DLD

    .................................................uuccccgcuuccccccuagggguu.......  1       0    s05

    .................................................uuccccgcuuccccccuagggg.........  1       0    s01

    .................................................uuccccgcuuccccccuaggggu........  1       0    U20

    ...........aaaggggugagcuggggagagga..............................................  1       0    s12
    .................................................uuccccgcuuccccccuaggg..........  1       0    s12
    .................................................uuccccgcuuccccccuagggg.........  1       0    s12
    .....................................................ccgcuuccccccuaggggu........  1       0    s12

    ..........caaaggggugagcuggggagagg...............................................  1       0    s02

    .................................................uuccccgcuuccccccuaggggu........  2       0    s06

    .................................................uuccccgcuuccccccuaggg..........  1       0    s11

    .................................................uuccccgcuuccccccuagggg.........  2       0    HeL

    ..........caaaggggugagcuggggagagg...............................................  1       0    j31

    .................................................uuccccgcuuccccccuagg...........  2       0    A54
    .................................................uuccccgcuuccccccuagggg.........  2       0    A54

    ..............................................uccuuccccgcuucccccc...............  1       0    MCF
    .................................................uuccccgcuuccccccuagg...........  1       0    MCF
    .................................................uuccccgcuuccccccuaggg..........  1       0    MCF
```

Provisional ID	: hsa_can_657
Score total	: 85.7
Score for star read(s)	: 3.9
Score for read counts	: 80.7
Score for mfe	: 1.8
Score for randfold	:
Score for cons. seed	: -0.6
Total read count	: 170
Mature read count	: 169
Loop read count	: 0
Star read count	: 1

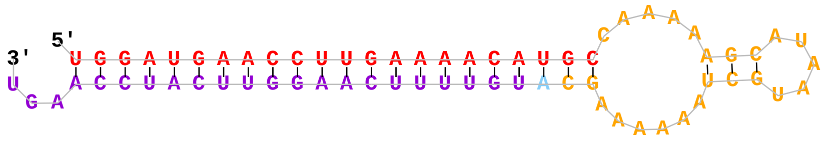

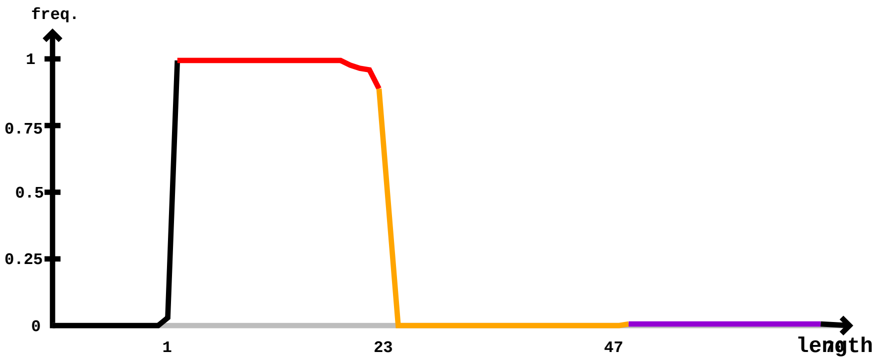

Mature Star

```
5'- guacugcaaauuggaugaaccuugaaaacaugccaaaaagcauaaugcuaaaaaagcauguuuucaagguucauccaaguu   -3'    obs
    guacugcaaauuggaugaaccuugaaaacaugccaaaaagcauaaugcuaaaaaagcauguuuucaagguucauccaaguu          exp
    ..........(((((((((((((((((((((((.....(((.....)))......)))))))))))))))))))))))...    reads   mm   sample
    ...........uggaugaaccuugaaaacaugc................................................    4       0    j23

    ...........uggaugaaccuugaaaacaugc................................................    1       0    ya1

    ...........uggaugaaccuugaaaacaugc................................................    1       0    s05

    ...........uggaugaaccuugaaaacaugc................................................    5       0    va1

    ...........uggaugaaccuugaaaacaugc................................................    1       0    ta2

    ...........uggaugaaccuugaaaacaugc................................................    2       0    k05

    ...........uggaugaaccuugaaaacaugc................................................    3       0    k11

    ...........uggaugaaccuugaaaacaugc................................................    3       0    s06

    ..........uuggaugaaccuugaaaacaug.................................................    1       0    ta1
    ...........uggaugaaccuugaaaacaugc................................................    1       0    ta1

    ...........uggaugaaccuugaaaacaugc................................................    1       0    s09

    ...........uggaugaaccuugaaaacaugc................................................    1       0    s10

    ..........uuggaugaaccuugaaaacaugc................................................    1       0    k10
    ...........uggaugaaccuugaaaacaugc................................................    1       0    k10

    ..........uuggaugaaccuugaaaacaug.................................................    2       0    j19
    ...........uggaugaaccuugaaaacaug.................................................    1       0    j19

    ..........uuggaugaaccuugaaaacaugc................................................    1       0    k04
    ...........uggaugaaccuugaaaacaugc................................................    1       0    k04

    ...........uggaugaaccuugaaaaca...................................................    1       0    s01
```

```
           Mature                                          Star
guacugcaaauuggaugaaccuugaaaacaugccaaaaagcauaaugcuaaaaaagcauguuuucaagguucauccaaguu
...........uggaugaaccuugaaaacaugc................................................   5    0    s01

...........uggaugaaccuugaaaacaug.................................................   1    0    per

...........uggaugaaccuugaaaacaugc................................................   8    0    j10

...........uggaugaaccuugaaaacaugc................................................   1    0    ku2

...........uggaugaaccuugaaaac....................................................   2    0    j21
...........uggaugaaccuugaaaacaugc................................................   14   0    j21

...........uggaugaaccuugaaaacaugc................................................   2    0    va2

...........uggaugaaccuugaaaacaugc................................................   1    0    k14

...........uggaugaaccuugaaaacaugc................................................   3    0    j31

...........uggaugaaccuugaaaacaugc................................................   4    0    j14

...........uggaugaaccuugaaaacaugc................................................   1    0    k02

...........uggaugaaccuugaaaacaugc................................................   1    0    j28

...........uggaugaaccuugaaaacaugc................................................   4    0    j22
...........uggaugaaccuugaaaacaugcc...............................................   1    0    j22

...........uggaugaaccuugaaaacaugc................................................   1    0    k09

...........uggaugaaccuugaaaacaugcc...............................................   1    0    DLD

...........uggaugaaccuugaaaacaugc................................................   3    0    k03

...........uggaugaaccuugaaaacaugc................................................   1    0    j09

...........uggaugaaccuugaaaacaugc................................................   1    0    j11

...........uggaugaaccuugaaaacaugc................................................   2    0    s02

...........uggaugaaccuugaaaacaugc................................................   1    0    ya3

...........uggaugaaccuugaaaacaugc................................................   3    0    k06

...........uggaugaaccuugaaaacaugc................................................   1    0    j29

...........uggaugaaccuugaaaacaug.................................................   2    0    sh2
...........uggaugaaccuugaaaacaugc................................................   1    0    sh2

...........uggaugaaccuugaaaacaugc................................................   1    0    HEK
..........................................................uguuuucaagguucauccaagu.   1    0    HEK

...........uggaugaaccuugaaaacaugc................................................   3    0    j17

...........uggaugaaccuugaaaac....................................................   1    0    MBM

...........uggaugaaccuugaaaacaugc................................................   2    0    k07

...........uggaugaaccuugaaaacaugc................................................   1    0    j12

...........uggaugaaccuugaaaacaugc................................................   18   0    li2

...........uggaugaaccuugaaaacaug.................................................   1    0    li1
...........uggaugaaccuugaaaacaugc................................................   28   0    li1

...........uggaugaaccuugaaaaca...................................................   1    0    k13
...........uggaugaaccuugaaaacaugc................................................   1    0    k13

...........uggaugaaccuugaaaacaugc................................................   4    0    va4

...........uggaugaaccuugaaaacaugc................................................   2    0    j20

...........uggaugaaccuugaaaacaug.................................................   1    0    va3
```

Mature Star

```
guacugcaaauuggaugaaccuugaaaacaugccaaaaagcauaaugcuaaaaaagcauguuuucaagguucauccaaguu
...........uggaugaaccuugaaaacaugc...............................................	2	0	va3

...........uggaugaaccuugaaaacaug................................................	1	0	ya4

...........uggaugaaccuugaaaacaug................................................	1	0	j30
...........uggaugaaccuugaaaacaugc...............................................	1	0	j30

...........uggaugaaccuugaaaacau.................................................	1	0	k12
...........uggaugaaccuugaaaacaug................................................	1	0	k12
...........uggaugaaccuugaaaacaugc...............................................	1	0	k12
```

Provisional ID	:	hsa_can_1031
Score total	:	69.7
Score for star read(s)	:	3.9
Score for read counts	:	64.9
Score for mfe	:	1.5
Score for randfold	:	
Score for cons. seed	:	-0.6
Total read count	:	139
Mature read count	:	114
Loop read count	:	0
Star read count	:	25

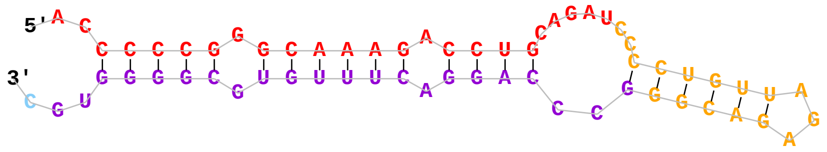

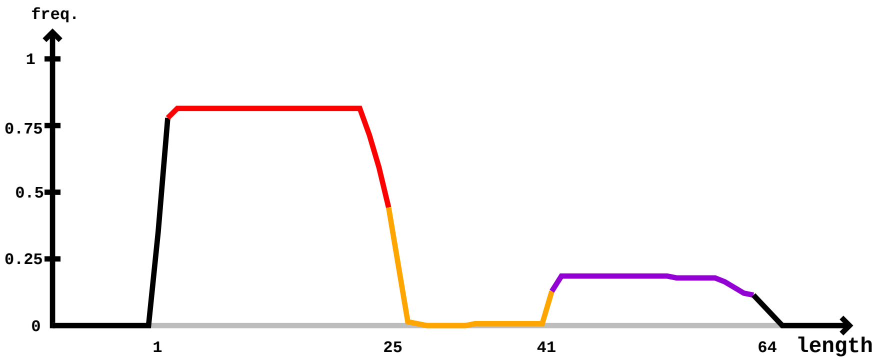

Mature Star

```
5'- uccagugaccacccccgggcaaagaccugcagauccccuguuagagacggggcccaggacuuugugcggggugcccacuguu  -3'   obs
    uccagugaccacccccgggcaaagaccugcagauccccuguuagagacggggcccaggacuuugugcggggugcccacuguu        exp
    ..(((((..((((((.(.((((((.((((.......((((((...))))))..)))).)))))).)))))))..)))))..   reads   mm   sample
    .........cacccccgggcaaagaccugcagau...............................................   3       0    j23
    ..........acccccgggcaaagaccugcagau...............................................   3       0    j23
    ..................................................gcccaggacuuugugcgg.............   2       0    j23

    .........cacccccgggcaaagaccugcagau...............................................   1       0    s04
    ..........acccccgggcaaagaccugcag.................................................   1       0    s04
    ..........acccccgggcaaagaccugcagau...............................................   1       0    s04
    ...................................................cccaggacuuugugcggggug.........   1       0    s04

    .........cacccccgggcaaagaccugca..................................................   1       0    s05
    .........cacccccgggcaaagaccugcaga................................................   2       0    s05
    .........cacccccgggcaaagaccugcagau...............................................   1       0    s05
    ..........acccccgggcaaagaccugcagau...............................................   1       0    s05

    .........cacccccgggcaaagaccugcaga................................................   1       0    va1
    ..........acccccgggcaaagaccugcaga................................................   1       0    va1
    ..........acccccgggcaaagaccugcagau...............................................   1       0    va1
    ..........acccccgggcaaagaccugcagauc..............................................   1       0    va1

    ..........acccccgggcaaagaccugca..................................................   1       0    HeL
    ...........cccccgggcaaagaccugcagauc..............................................   2       0    HeL

    .........cacccccgggcaaagaccugcag.................................................   1       0    s09
    ..........acccccgggcaaagaccugcaga................................................   1       0    s09

    ..........acccccgggcaaagaccugcaga................................................   2       0    ku4

    .........cacccccgggcaaagaccugcag.................................................   2       0    s07
    ..........acccccgggcaaagaccugcagau...............................................   1       0    s07
    ..................................................gcccaggacuuugugcggg............   1       0    s07
    ..................................................gcccaggacuuugugcggggugcc.......   1       0    s07

    ..................................................gcccaggacuuugugcggggug.........   1       0    s10
```

Mature **Star**

```
uccagugaccacccccgggcaaagaccugcagauccccuguuagagacgggcccaggacuuugugcggggugcccacuguu

.........cacccccgggcaaagaccugcag.................................................    1    0    k08

...........cccccgggcaaagaccugcaga................................................    1    0    j03

..........acccccgggcaaagaccugcaga................................................    2    0    s01
..........acccccgggcaaagaccugcagau...............................................    2    0    s01
..........acccccgggcaaagaccugcagauc..............................................    1    0    s01

...........cccccgggcaaagaccugcagauc..............................................    1    0    j10
...................................................cccaggacuuugugcggggugcc.......    1    0    j10

..........acccccgggcaaagaccugcaga................................................    1    0    ku2

.........cacccccgggcaaagaccugca..................................................    1    0    U20
.........cacccccgggcaaagaccugcagau...............................................    2    0    U20
..........acccccgggcaaagaccugcagau...............................................    2    0    U20

.........cacccccgggcaaagaccugcagauc..............................................    1    0    j21
..........acccccgggcaaagaccugcagau...............................................    1    0    j21
..................................................gcccaggacuuugugcggg............    1    0    j21
..................................................gcccaggacuuugugcggggugc........    1    0    j21
...................................................cccaggacuuugugcggggug.........    2    0    j21
...................................................cccaggacuuugugcggggugc........    1    0    j21
...................................................cccaggacuuugugcggggugcc.......    1    0    j21

..........................................agagacgggcccaggacuuug..................    1    0    k14

.........cacccccgggcaaagaccugcag.................................................    1    0    va2
..........acccccgggcaaagaccugcag.................................................    3    0    va2

..........acccccgggcaaagaccugcagau...............................................    1    0    j31

..........acccccgggcaaagaccugcagau...............................................    1    0    s03

.........cacccccgggcaaagaccugcagau...............................................    1    0    H52

..........acccccgggcaaagaccugcagauccc............................................    1    0    k09

.........cacccccgggcaaagaccugcaga................................................    1    0    s08
.........cacccccgggcaaagaccugcagau...............................................    1    0    s08
..........acccccgggcaaagaccugcaga................................................    1    0    s08
...................................................cccaggacuuugugcggggugc........    1    0    s08

..........acccccgggcaaagaccugcaga................................................    1    0    DLD

.........cacccccgggcaaagaccugcaga................................................    1    0    j18

.........cacccccgggcaaagaccugcaga................................................    1    0    j16

..........acccccgggcaaagaccugcagau...............................................    1    0    ku3

.........cacccccgggcaaagaccugcagau...............................................    1    0    j11

..................................................gcccaggacuuugugcgggg...........    1    0    la1

.........cacccccgggcaaagaccugca..................................................    3    0    sh2
...........cccccgggcaaagaccugcagauc..............................................    1    0    sh2
..................................................gcccaggacuuugugcgggg...........    1    0    sh2

..........acccccgggcaaagaccugcagaucc.............................................    1    0    HEK

..................................................gcccaggacuuugugcggggug.........    3    0    j01

.........cacccccgggcaaagaccugca..................................................    2    0    j17
..........acccccgggcaaagaccugcaga................................................    1    0    j17

.........cacccccgggcaaagaccugca..................................................    1    0    j15
..........acccccgggcaaagaccugcagau...............................................    1    0    j15

..........acccccgggcaaagaccugcaga................................................    1    0    j12
```

```
          Mature                                  Star
uccagugaccacccccgggcaaagaccugcagauccccuguuagagacgggcccaggacuuugugcgggguguccacuguu

..................................................gcccaggacuuugugcggggu..........    1    0    li1
..................................................gcccaggacuuugugcggggug.........    1    0    li1
...................................................cccaggacuuugugcggggugc........    1    0    li1

..................................................gcccaggacuuugugcggg............    1    0    va3

.........cacccccgggcaaagaccugca..................................................    5    0    A54
.........cacccccgggcaaagaccugcag.................................................    7    0    A54
.........cacccccgggcaaagaccugcagau...............................................    5    0    A54
..........acccccgggcaaagaccugcaga................................................    1    0    A54
..........acccccgggcaaagaccugcagau...............................................    22   0    A54
..................................................gcccaggacuuugugcgggg...........    1    0    A54
..................................................gcccaggacuuugugcggggugc........    1    0    A54

.........cacccccgggcaaagaccugcaga................................................    1    0    j30

..........acccccgggcaaagaccugcag.................................................    1    0    SW4

.........cacccccgggcaaagaccugcaga................................................    1    0    ku1
```

Provisional ID	:	hsa_can_1129
Score total	:	11.9
Score for star read(s)	:	3.9
Score for read counts	:	7.3
Score for mfe	:	1.3
Score for randfold	:	
Score for cons. seed	:	-0.6
Total read count	:	26
Mature read count	:	25
Loop read count	:	0
Star read count	:	1

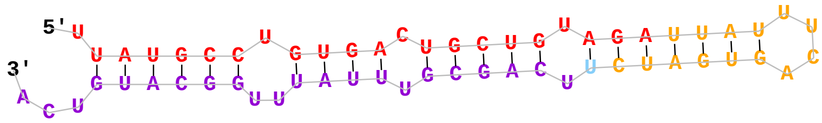

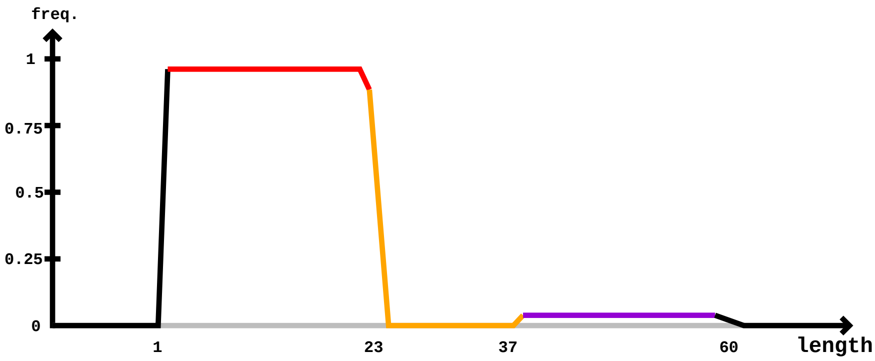


```
                   Mature                                   Star
5'- guguggucuguuaugccugugacugcuguagauuauuucagugaucuucagcguuuauuuggcaugucagacuaucucaga   -3'    obs
    guguggucuguuaugccugugacugcuguagauuauuucagugaucuucagcguuuauuuggcaugucagacuaucucaga          exp
    ..((((((((.((((((.((((.(((((.(((((((....))))))).))))).))))..)))))).))))))))......    reads      mm          sample
    ...............................................ucagcguuuauuuggcaugucа............    1          0           k10

    ..........uuaugccugugacugcuguaga.................................................    1          0           li2

    ..........uuaugccugugacugcuguaga.................................................    20         0           li1

    ..........uuaugccugugacugcuguaga.................................................    1          0           k01

    ..........uuaugccugugacugcuguaga.................................................    1          0           j29

    ..........uuaugccugugacugcuguag..................................................    2          0           U20
```

Provisional ID	:	hsa_can_1150
Score total	:	-0.4
Score for star read(s)	:	3.9
Score for read counts	:	0.1
Score for mfe	:	-3.8
Score for randfold	:	
Score for cons. seed	:	-0.6
Total read count	:	12
Mature read count	:	9
Loop read count	:	0
Star read count	:	3

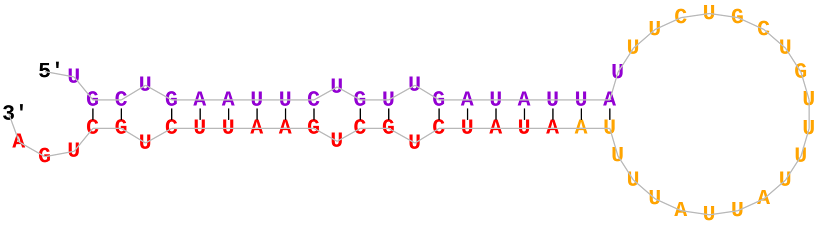

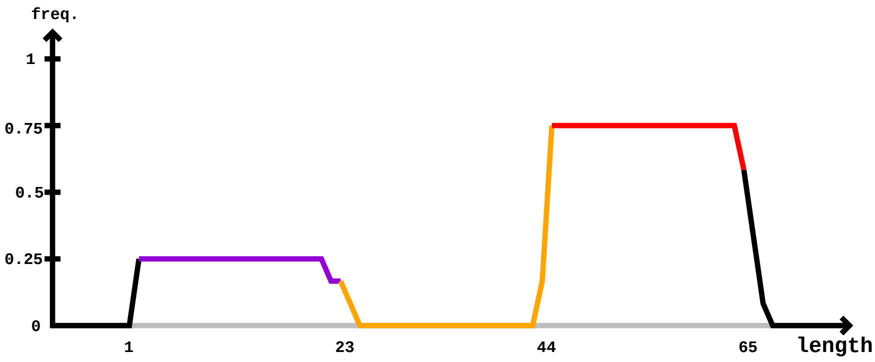

Star **Mature**

```
5'- uaauaucugcugaauucuguugauauuauuucugcuguuuuauuauuuuaauaucugcugaauucugcugauauuaucugc  -3'   obs
    uaauaucugcugaauucuguugauauuauuucugcuguuuuauuauuuuaauaucugcugaauucugcugauauuaucugc         exp
    (((((((.((.((((((.((.(((((((....................))))))).)).)))))).)).))))))).....   reads   mm   sample
    .......ugcugaauucuguugauauuau....................................................   1       0    j23

    .......ugcugaauucuguugauauu......................................................   1       0    li1

    ..................................................auaucugcugaauucugcuga..........   1       0    ta1

    ..................................................auaucugcugaauucugcuga..........   1       0    s06

    .......ugcugaauucuguugauauuau....................................................   1       0    HeL
    ..................................................auaucugcugaauucugcug...........   1       0    HeL

    .................................................aauaucugcugaauucugcuga..........   1       0    HEK
    ..................................................auaucugcugaauucugcuga..........   1       0    HEK

    ..................................................auaucugcugaauucugcugaua........   1       0    ku4

    .................................................aauaucugcugaauucugcug...........   1       0    j17
    ..................................................auaucugcugaauucugcuga..........   2       0    j17
```

Provisional ID	:	hsa_can_1340
Score total	:	35.5
Score for star read(s)	:	3.9
Score for read counts	:	30.7
Score for mfe	:	1.5
Score for randfold	:	
Score for cons. seed	:	-0.6
Total read count	:	72
Mature read count	:	56
Loop read count	:	1
Star read count	:	15

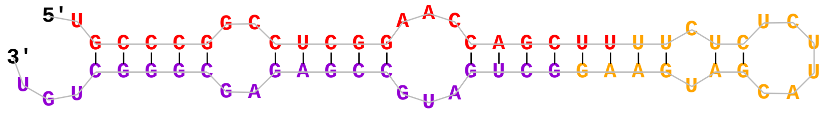

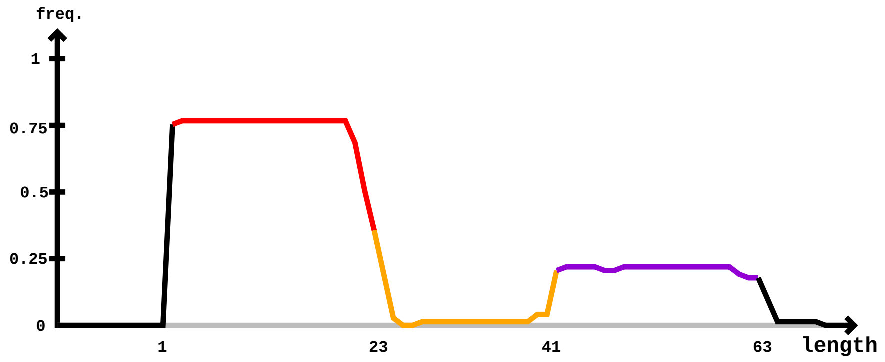

Mature **Star**

```
5'- gccuuucuccugcccggccucggaaccagcuuuucucucuuacgaugaaggcugaugccgagagcgggcugugggcggagc  -3'   obs
    gccuuucuccugcccggccucggaaccagcuuuucucucuuacgaugaaggcugaugccgagagcgggcugugggcggagc        exp
    ..(((..(((.(((((..(((((...((((((((.((......)).))))))))...)))))..)))))...)))..))).    reads   mm   sample
    ..................................................gcugaugccgagagcgggcugu.........    1       0    s08

    ..........ugcccggccucggaaccagcuu.................................................    1       0    k09

    ..........ugcccggccucggaaccagcuu.................................................    1       0    k03
    .........................................................ccgagagcgggcugugggcgg...    1       0    k03

    ..........ugcccggccucggaaccagc...................................................    1       0    j26

    ..........ugcccggccucggaaccagcu..................................................    2       0    j24

    ..................................................gcugaugccgagagcgggcugu.........    1       0    k11

    ..................................................gcugaugccgagagcgggcugu.........    1       0    k06

    ..........ugcccggccucggaaccagcu..................................................    1       0    HeL

    ....................................cucuuacgaugaaggcuga..........................    1       0    la1

    ..........ugcccggccucggaaccag....................................................    5       0    sh2
    ..........ugcccggccucggaaccagc...................................................    6       0    sh2
    ..........ugcccggccucggaaccagcu..................................................    5       0    sh2
    ..........ugcccggccucggaaccagcuu.................................................    8       0    sh2
    ..........ugcccggccucggaaccagcuuu................................................    4       0    sh2
    ..........ugcccggccucggaaccagcuuuu...............................................    1       0    sh2
    ...........gcccggccucggaaccagcu..................................................    1       0    sh2
    ..................................................gcugaugccgagagcgggcugu.........    1       0    sh2

    ..................................................gcugaugccgagagcgggcugu.........    2       0    MCF

    ..........ugcccggccucggaaccagc...................................................    1       0    k08

    ..........ugcccggccucggaaccagc...................................................    1       0    k07
```

```
          Mature                                  Star
gccuuucuccugcccggccucggaaccagcuuuucucucuuacgaugaaggcugaugccgagagcgggcugugggcggagc
..........ugcccggccucggaaccagcuu.................................................    1    0    k07
..........ugcccggccucggaaccagcuuu................................................    3    0    k07

..........ugcccggccucggaaccagcu..................................................    2    0    sh1
..........ugcccggccucggaaccagcuuu................................................    1    0    sh1
..................................................gcugaugccgagagcgggc............    2    0    sh1
...................................................cugaugccgagagcgggcugu.........    1    0    sh1

..................................................gcugaugccgagagcgggcugu.........    1    0    li2

..........ugcccggccucggaaccagc...................................................    1    0    li1

..........ugcccggccucggaaccag....................................................    1    0    k13

..........ugcccggccucggaaccagc...................................................    1    0    per
..........ugcccggccucggaaccagcuu.................................................    1    0    per
................................................aggcugaugccgagagcgggcu...........    1    0    per
................................................aggcugaugccgagagcgggcugu.........    1    0    per
..................................................gcugaugccgagagcgggcugu.........    2    0    per

..........ugcccggccucggaaccagcuu.................................................    1    0    k04
..........ugcccggccucggaaccagcuuu................................................    1    0    k04

..........ugcccggccucggaaccagcuu.................................................    1    0    j21

..................................................gcugaugccgagagcgggcugu.........    1    0    va3

..........ugcccggccucggaaccagc...................................................    1    0    k12
..........ugcccggccucggaaccagcuu.................................................    1    0    k12

..........ugcccggccucggaaccagc...................................................    1    0    ya4
..........ugcccggccucggaaccagcuuuu...............................................    1    0    ya4
```

Provisional ID	: hsa_can_1365	
Score total	:	11.2
Score for star read(s)	:	3.9
Score for read counts	:	6.8
Score for mfe	:	1.1
Score for randfold	:	
Score for cons. seed	:	-0.6
Total read count	:	25
Mature read count	:	21
Loop read count	:	0
Star read count	:	4

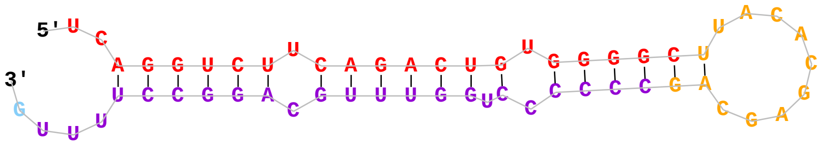

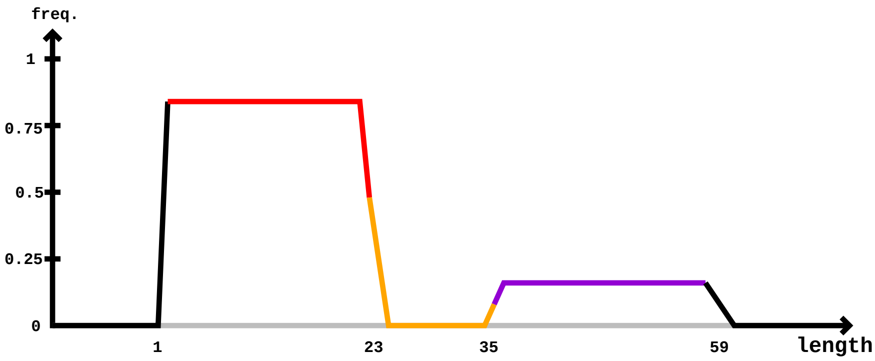

Mature **Star**

```
5'- acaccuggucucaggucuucagacuguggggcuuacacgagcagccccccugguuugcaggccuuuugacuuggucugaau  -3'   obs
    acaccuggucucaggucuucagacuguggggcuuacacgagcagccccccugguuugcaggccuuuugacuuggucugaau        exp
    ..(((.((((..((((((.(((((((.((((((.........)))))).).)))))).))))))...)))).)))......    reads   mm   sample
    ..........ucaggucuucagacuguggggcu................................................    1       0    j12

    ..........ucaggucuucagacuguggggc.................................................    1       0    j19

    ..........ucaggucuucagacuguggggc.................................................    2       0    li1

    ..........ucaggucuucagacugugggg..................................................    4       0    li2
    ..........ucaggucuucagacuguggggc.................................................    7       0    li2

    ............................................ccccccugguuugcaggccuuuu..............    1       0    va1

    .............................................cccccugguuugcaggccuuuu..............    2       0    ku2

    ..........ucaggucuucagacugugggg..................................................    3       0    U20

    ..........ucaggucuucagacuguggggc.................................................    1       0    k06

    ..........ucaggucuucagacugugggg..................................................    2       0    A54

    ............................................ccccccugguuugcaggccuuuu..............    1       0    s07
```

Provisional ID	:	hsa_can_1461
Score total	:	34.4
Score for star read(s)	:	3.9
Score for read counts	:	29.7
Score for mfe	:	1.4
Score for randfold	:	
Score for cons. seed	:	-0.6
Total read count	:	70
Mature read count	:	52
Loop read count	:	1
Star read count	:	17

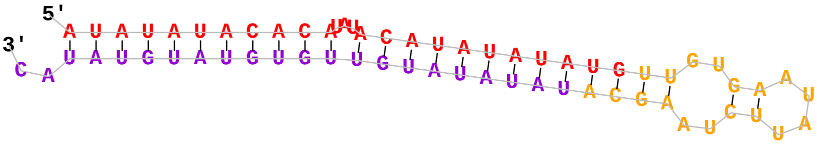

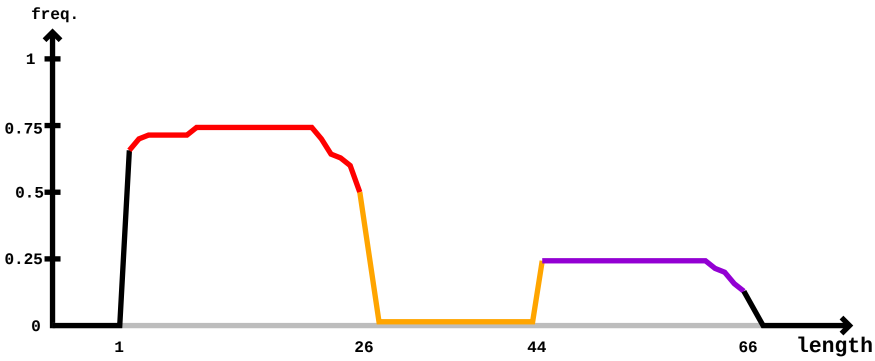

Mature **Star**

```
5'- acauguauauauacacauauacauauauauguugugaauauucuaagcauauauauguuguguauguauacgugugugugu  -3'    obs
    acauguauauauacacauauacauauauauguugugaauauucuaagcauauauauguuguguauguauacgugugugugu          exp
    ((((((((((((((((...((((((((((((((..((....))..))))))))))))))))))))))))))))))......   reads      mm         sample
    ......auauauacacauauacauauauau...................................................   4          0          DLD
    .................................................uauauauguuguguauguauac..........   1          0          DLD

    ......auauauacacauauacauau.......................................................   3          0          ya1
    .......uauauacacauauacauaua......................................................   2          0          ya1

    ......auauauacacauauacauaua......................................................   2          0          ya2

    .................................................uauauauguuguguauguaua...........   1          0          ya3

    .................................................uauauauguuguguaugu..............   1          0          HeL

    ......auauauacacauauacauauau.....................................................   1          0          HEK
    ......auauauacacauauacauauauau...................................................   2          0          HEK
    ......auauauacacauauacauauauaug..................................................   3          0          HEK
    .................................................uauauauguuguguauguauac..........   1          0          HEK

    .......uauauacacauauacauauauaug..................................................   1          0          MCF

    ......auauauacacauauacauauauaug..................................................   1          0          MBM
    ........auauacacauauacauauauaugu.................................................   1          0          MBM

    ......auauauacacauauacauauauaug..................................................   2          0          li2

    ......auauauacacauauacauauauaug..................................................   3          0          li1
    .................................................uauauauguuguguauguauac..........   1          0          li1

    ......auauauacacauauacauauaua....................................................   2          0          U20
    ......auauauacacauauacauauauaugu.................................................   8          0          U20
    .............cacauauacauauauaug..................................................   2          0          U20
    .................................................uauauauguuguguaugua.............   1          0          U20
    .................................................uauauauguuguguauguauac..........   5          0          U20

    .................................................uauauauguuguguauguau............   2          0          j21
```

Mature Star

```
acauguauauauacacauauacauauauaugUugugaauauucuaagcauauauauguuguguauguauacgugugugugu

..................................................uauauauguuguguauguauac..........     1     0     j27

......auauauacacauauacauauauaug..................................................     14    0     A54

......auauauacacauauacauauauau...................................................     1     0     SW4
..................................................uauauauguuguguauguau............     1     0     SW4

..................................................uauauauguuguguaugu..............     1     0     ya4

...............................uugugaauauucuaagca................................     1     0     H52
..................................................uauauauguuguguauguaua...........     1     0     H52
```

Provisional ID : hsa_can_1680
Score total : 13.5
Score for star read(s) : 3.9
Score for read counts : 8.3
Score for mfe : 1.9
Score for randfold :
Score for cons. seed : -0.6
Total read count : 28
Mature read count : 26
Loop read count : 0
Star read count : 2

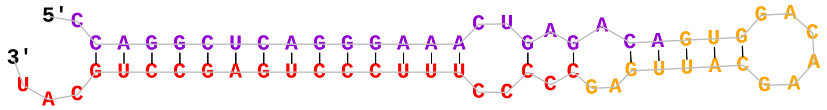

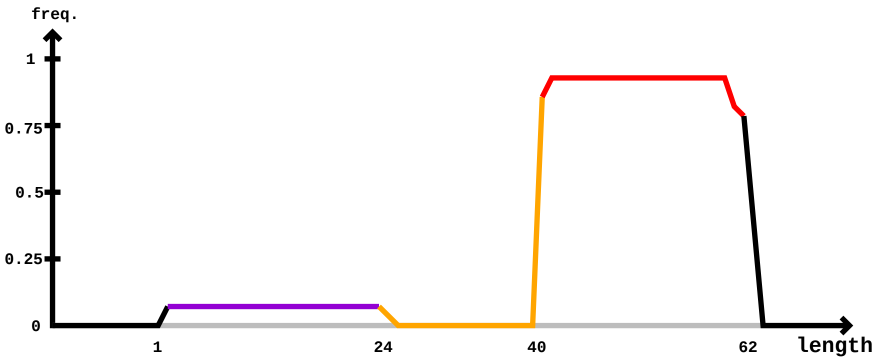


```
                 Star                                            Mature
5'- gggcccagguccaggcucagggaaacugagacaguggacaagcauugagcccccuuucccugagccugcauuuguggccag  -3'   obs
    gggcccagguccaggcucagggaaacugagacaguggacaagcauugagcccccuuucccugagccugcauuuguggccag         exp
    .(((((((((.((((((((((((((..(.(.(((((......)))))..).)..)))))))))))))).))))).))))..    reads   mm   sample
    .................................................cccccuuucccugagccugcau..........    1       0    s08

    .................................................cccccuuucccugagccugcau..........    2       0    DLD

    .................................................cccccuuucccugagccugcau..........    1       0    k03

    .................................................cccccuuucccugagccugc............    1       0    ku3
    .................................................cccccuuucccugagccugcau..........    1       0    ku3

    .................................................cccccuuucccugagccugcau..........    1       0    ya2

    .................................................cccccuuucccugagccugcau..........    1       0    j26

    .................................................cccccuuucccugagccugcau..........    2       0    va1

    .................................................cccccuuucccugagccugcau..........    1       0    s06

    .................................................cccccuuucccugagccugcau..........    1       0    MCF

    .................................................cccccuuucccugagccugcau..........    1       0    k07

    .................................................cccccuuucccugagccugcau..........    1       0    s01

    ..........ccaggcucagggaaacugagaca................................................    2       0    U20
    .................................................cccccuuucccugagccugcau..........    3       0    U20

    .................................................cccccuuucccugagccugcau..........    1       0    va3

    .................................................cccccuuucccugagccugc............    2       0    j28
    .................................................cccccuuucccugagccugca...........    1       0    j28
    .................................................cccccuuucccugagccugcau..........    2       0    j28

    ..................................................ccccuuucccugagccugcau..........    2       0    ya4
```

Star **Mature**

gggcccaggucc aggcucagggaaacugagacaguggacaagcauugagcccccuuucccugagccugcauuuguggccag

....cccccuuucccugagccugcau.......... 1 0 s03

Provisional ID	:	hsa_can_1846
Score total	:	566147.1
Score for star read(s)	:	3.9
Score for read counts	:	566143.1
Score for mfe	:	-2.8
Score for randfold	:	
Score for cons. seed	:	3
Total read count	:	1110477
Mature read count	:	1107837
Loop read count	:	0
Star read count	:	2640

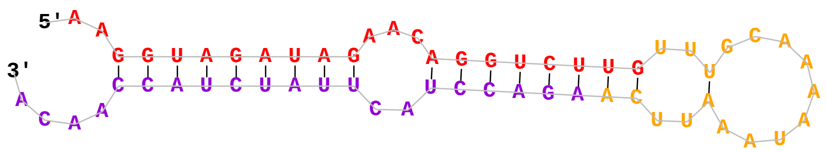

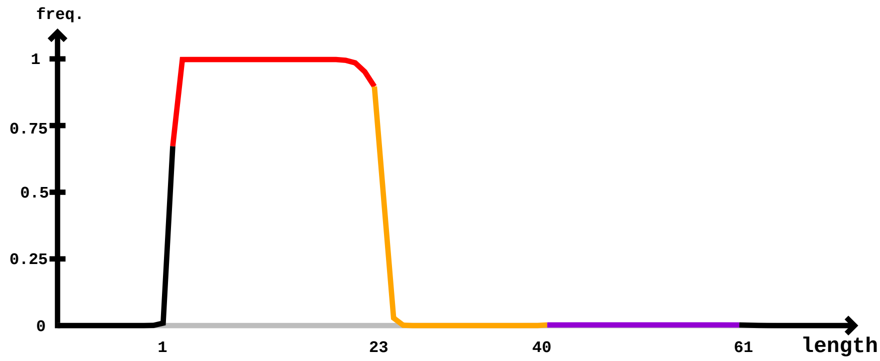

Mature **Star**

```
5'- ccuaaaugaaaagguagauagaacaggucuuguuugcaaaauaaauucaagaccuacuuaucuaccaacagcaauuacacg  -3'   obs
    ccuaaaugaaaagguagauagaacaggucuuguuugcaaaauaaauucaagaccuacuuaucuaccaacagcaauuacacg         exp
    ...........((((((((...((((((((..(.........)..))))))))..))))))))..................   reads   mm   sample
    ........aaaagguagauagaacagg......................................................   1       0    ya1
    ........aaaagguagauagaacaggucu...................................................   2       0    ya1
    ........aaaagguagauagaacaggucuu..................................................   1       0    ya1
    ........aaaagguagauagaacaggucuugu................................................   2       0    ya1
    .........aaagguagauagaacagg......................................................   1       0    ya1
    .........aaagguagauagaacagguc....................................................   1       0    ya1
    .........aaagguagauagaacaggucu...................................................   2       0    ya1
    .........aaagguagauagaacaggucuu..................................................   13      0    ya1
    .........aaagguagauagaacaggucuug.................................................   8       0    ya1
    .........aaagguagauagaacaggucuugu................................................   5       0    ya1
    ..........aagguagauagaacaggu.....................................................   65      0    ya1
    ..........aagguagauagaacagguc....................................................   141     0    ya1
    ..........aagguagauagaacaggucu...................................................   185     0    ya1
    ..........aagguagauagaacaggucuu..................................................   624     0    ya1
    ..........aagguagauagaacaggucuug.................................................   1135    0    ya1
    ..........aagguagauagaacaggucuugu................................................   284     0    ya1
    ..........aagguagauagaacaggucuuguu...............................................   8       0    ya1
    ...........agguagauagaacagguc....................................................   69      0    ya1
    ...........agguagauagaacaggucu...................................................   217     0    ya1
    ...........agguagauagaacaggucuu..................................................   671     0    ya1
    ...........agguagauagaacaggucuug.................................................   1014    0    ya1
    ...........agguagauagaacaggucuugu................................................   257     0    ya1
    ...........agguagauagaacaggucuuguu...............................................   101     0    ya1
    ...........agguagauagaacaggucuuguuu..............................................   1       0    ya1
    ............gguagauagaacaggucuug.................................................   2       0    ya1
    ............gguagauagaacaggucuugu................................................   1       0    ya1
    .................................................agaccuacuuaucuacca..............   3       0    ya1
    .................................................agaccuacuuaucuaccaa.............   1       0    ya1
    .................................................agaccuacuuaucuaccaaca...........   33      0    ya1
    .................................................agaccuacuuaucuaccaacag..........   16      0    ya1
    .................................................agaccuacuuaucuaccaacagc.........   2       0    ya1

    ........aaaagguagauagaacaggucuug.................................................   1       0    s04
```

Mature **Star**

```
ccuaaaugaaaagguagauagaacaggucuuguuugcaaaauaaaauucaagaccuacuuaucuaccaacagcaauuacacg
.........aaagguagauagaacagguc.....................................................	1	0	s04
.........aaagguagauagaacaggucu....................................................	4	0	s04
.........aaagguagauagaacaggucuu...................................................	7	0	s04
.........aaagguagauagaacaggucuug..................................................	40	0	s04
.........aaagguagauagaacaggucuugu.................................................	4	0	s04
..........aagguagauagaacaggu......................................................	23	0	s04
..........aagguagauagaacagguc.....................................................	64	0	s04
..........aagguagauagaacaggucu....................................................	239	0	s04
..........aagguagauagaacaggucuu...................................................	374	0	s04
..........aagguagauagaacaggucuug..................................................	4802	0	s04
..........aagguagauagaacaggucuugu.................................................	568	0	s04
..........aagguagauagaacaggucuuguu................................................	4	0	s04
...........agguagauagaacagguc.....................................................	40	0	s04
...........agguagauagaacaggucu....................................................	60	0	s04
...........agguagauagaacaggucuu...................................................	142	0	s04
...........agguagauagaacaggucuug..................................................	2510	0	s04
...........agguagauagaacaggucuugu.................................................	491	0	s04
...........agguagauagaacaggucuuguu................................................	411	0	s04
...........agguagauagaacaggucuuguuu...............................................	10	0	s04
..............uagauagaacaggucuugu.................................................	1	0	s04
................................................caagaccuacuuaucuaccaaca...........	1	0	s04
..................................................agaccuacuuaucuaccaaca...........	9	0	s04
..................................................agaccuacuuaucuaccaacag..........	1	0	s04
..................................................agaccuacuuaucuaccaacagc.........	1	0	s04

........aaaagguagauagaacaggu......................................................	2	0	j26
........aaaagguagauagaacaggucu....................................................	1	0	j26
........aaaagguagauagaacaggucuug..................................................	1	0	j26
.........aaagguagauagaacaggucu....................................................	4	0	j26
.........aaagguagauagaacaggucuu...................................................	15	0	j26
.........aaagguagauagaacaggucuug..................................................	57	0	j26
.........aaagguagauagaacaggucuugu.................................................	2	0	j26
..........aagguagauagaacaggu......................................................	5	0	j26
..........aagguagauagaacagguc.....................................................	43	0	j26
..........aagguagauagaacaggucu....................................................	203	0	j26
..........aagguagauagaacaggucuu...................................................	302	0	j26
..........aagguagauagaacaggucuug..................................................	4151	0	j26
..........aagguagauagaacaggucuugu.................................................	814	0	j26
..........aagguagauagaacaggucuuguu................................................	6	0	j26
..........aagguagauagaacaggucuuguuu...............................................	1	0	j26
..........aagguagauagaacaggucuuguuug..............................................	2	0	j26
...........agguagauagaacagguc.....................................................	19	0	j26
...........agguagauagaacaggucu....................................................	49	0	j26
...........agguagauagaacaggucuu...................................................	75	0	j26
...........agguagauagaacaggucuug..................................................	1658	0	j26
...........agguagauagaacaggucuugu.................................................	479	0	j26
...........agguagauagaacaggucuuguu................................................	475	0	j26
...........agguagauagaacaggucuuguuu...............................................	29	0	j26
............gguagauagaacaggucuug..................................................	1	0	j26
............gguagauagaacaggucuugu.................................................	2	0	j26
................................................caagaccuacuuaucuaccaacagca........	1	0	j26
..................................................agaccuacuuaucuaccaa.............	1	0	j26
..................................................agaccuacuuaucuaccaaca...........	3	0	j26
..................................................agaccuacuuaucuaccaacag..........	1	0	j26
...................................................gaccuacuuaucuaccaacagca........	1	0	j26
....................................................accuacuuaucuaccaacagca........	1	0	j26

........aaaagguagauagaacaggucuu...................................................	2	0	va1
........aaaagguagauagaacaggucuug..................................................	9	0	va1
........aaaagguagauagaacaggucuugu.................................................	6	0	va1
.........aaagguagauagaacaggu......................................................	1	0	va1
.........aaagguagauagaacagguc.....................................................	2	0	va1
.........aaagguagauagaacaggucuu...................................................	18	0	va1
.........aaagguagauagaacaggucuug..................................................	147	0	va1
.........aaagguagauagaacaggucuugu.................................................	13	0	va1
..........aagguagauagaacaggu......................................................	27	0	va1
..........aagguagauagaacagguc.....................................................	122	0	va1
..........aagguagauagaacaggucu....................................................	132	0	va1
..........aagguagauagaacaggucuu...................................................	476	0	va1
..........aagguagauagaacaggucuug..................................................	8619	0	va1
```

```
          Mature                                   Star
ccuaaaugaaaagguagauagaacaggucuuguuugcaaaauaaauucaagaccuacuuaucuaccaacagcaauuacacg
..........aagguagauagaacaggucuugu................................................    1537    0    va1
..........aagguagauagaacaggucuuguu...............................................    16      0    va1
..........aagguagauagaacaggucuuguuu..............................................    1       0    va1
...........agguagauagaacagguc....................................................    31      0    va1
...........agguagauagaacaggucu...................................................    29      0    va1
...........agguagauagaacaggucuu..................................................    92      0    va1
...........agguagauagaacaggucuug.................................................    3038    0    va1
...........agguagauagaacaggucuugu................................................    440     0    va1
...........agguagauagaacaggucuuguu...............................................    467     0    va1
...........agguagauagaacaggucuuguuu..............................................    17      0    va1
...........agguagauagaacaggucuuguuug.............................................    1       0    va1
...........agguagauagaacaggucuuguuugc............................................    2       0    va1
...........agguagauagaacaggucuuguuugca...........................................    1       0    va1
............gguagauagaacaggucuug.................................................    1       0    va1
................................................aagaccuacuuaucuaccaacagca........    1       0    va1
.................................................agaccuacuuaucuaccaac............    1       0    va1
.................................................agaccuacuuaucuaccaaca...........    1       0    va1

........aaaagguagauagaacagg......................................................    1       0    ku5
........aaaagguagauagaacagguc....................................................    1       0    ku5
........aaaagguagauagaacaggucuug.................................................    1       0    ku5
.........aaagguagauagaacagg......................................................    2       0    ku5
.........aaagguagauagaacagguc....................................................    5       0    ku5
.........aaagguagauagaacaggucu...................................................    29      0    ku5
.........aaagguagauagaacaggucuu..................................................    10      0    ku5
.........aaagguagauagaacaggucuug.................................................    73      0    ku5
.........aaagguagauagaacaggucuugu................................................    15      0    ku5
..........aagguagauagaacaggu.....................................................    4       0    ku5
..........aagguagauagaacagguc....................................................    104     0    ku5
..........aagguagauagaacaggucu...................................................    874     0    ku5
..........aagguagauagaacaggucuu..................................................    478     0    ku5
..........aagguagauagaacaggucuug.................................................    5464    0    ku5
..........aagguagauagaacaggucuugu................................................    1344    0    ku5
..........aagguagauagaacaggucuuguu...............................................    24      0    ku5
..........aagguagauagaacaggucuuguuu..............................................    2       0    ku5
...........agguagauagaacagguc....................................................    10      0    ku5
...........agguagauagaacaggucu...................................................    43      0    ku5
...........agguagauagaacaggucuu..................................................    73      0    ku5
...........agguagauagaacaggucuug.................................................    1603    0    ku5
...........agguagauagaacaggucuugu................................................    917     0    ku5
...........agguagauagaacaggucuuguu...............................................    598     0    ku5
...........agguagauagaacaggucuuguuu..............................................    34      0    ku5
............gguagauagaacaggucuug.................................................    1       0    ku5
...............................................caagaccuacuuaucuaccaac............    1       0    ku5
.................................................agaccuacuuaucuaccaac............    1       0    ku5
.................................................agaccuacuuaucuaccaaca...........    15      0    ku5
.................................................agaccuacuuaucuaccaacag..........    1       0    ku5
.................................................agaccuacuuaucuaccaacagc.........    1       0    ku5

........aaaagguagauagaacaggu.....................................................    1       0    k05
........aaaagguagauagaacagguc....................................................    1       0    k05
........aaaagguagauagaacaggucu...................................................    4       0    k05
........aaaagguagauagaacaggucuu..................................................    15      0    k05
........aaaagguagauagaacaggucuug.................................................    8       0    k05
........aaaagguagauagaacaggucuugu................................................    1       0    k05
.........aaagguagauagaacaggu.....................................................    1       0    k05
.........aaagguagauagaacaggucu...................................................    10      0    k05
.........aaagguagauagaacaggucuu..................................................    42      0    k05
.........aaagguagauagaacaggucuug.................................................    141     0    k05
.........aaagguagauagaacaggucuugu................................................    9       0    k05
.........aaagguagauagaacaggucuuguu...............................................    1       0    k05
..........aagguagauagaacaggu.....................................................    53      0    k05
..........aagguagauagaacagguc....................................................    106     0    k05
..........aagguagauagaacaggucu...................................................    312     0    k05
..........aagguagauagaacaggucuu..................................................    1061    0    k05
..........aagguagauagaacaggucuug.................................................    12999   0    k05
..........aagguagauagaacaggucuugu................................................    1619    0    k05
..........aagguagauagaacaggucuuguu...............................................    21      0    k05
...........agguagauagaacagguc....................................................    25      0    k05
...........agguagauagaacaggucu...................................................    85      0    k05
```

Mature Star

```
ccuaaaugaaaagguagauagaacaggucuuguuugcaaaauaaaauucaagaccuacuuaucuaccaacagcaauuacacg
...........agguagauagaacaggucuu...................................................   331    0   k05
...........agguagauagaacaggucuug..................................................   4767   0   k05
...........agguagauagaacaggucuugu.................................................   937    0   k05
...........agguagauagaacaggucuuguu................................................   537    0   k05
...........agguagauagaacaggucuuguuu...............................................   26     0   k05
...........agguagauagaacaggucuuguuug..............................................   1      0   k05
..................................................agaccuacuuaucuaccaaca...........   2      0   k05
..................................................agaccuacuuaucuaccaacagc.........   1      0   k05

........aaaagguagauagaacaggucuu...................................................   2      0   s06
........aaaagguagauagaacaggucuugu.................................................   2      0   s06
.........aaagguagauagaacagg.......................................................   1      0   s06
.........aaagguagauagaacaggucu....................................................   1      0   s06
.........aaagguagauagaacaggucuu...................................................   1      0   s06
.........aaagguagauagaacaggucuug..................................................   35     0   s06
..........aagguagauagaacaggu......................................................   6      0   s06
..........aagguagauagaacagguc.....................................................   26     0   s06
..........aagguagauagaacaggucu....................................................   47     0   s06
..........aagguagauagaacaggucuu...................................................   115    0   s06
..........aagguagauagaacaggucuug..................................................   2569   0   s06
..........aagguagauagaacaggucuugu.................................................   240    0   s06
..........aagguagauagaacaggucuuguu................................................   7      0   s06
...........agguagauagaacagguc.....................................................   8      0   s06
...........agguagauagaacaggucu....................................................   17     0   s06
...........agguagauagaacaggucuu...................................................   40     0   s06
...........agguagauagaacaggucuug..................................................   1000   0   s06
...........agguagauagaacaggucuugu.................................................   80     0   s06
...........agguagauagaacaggucuuguu................................................   114    0   s06
...........agguagauagaacaggucuuguuu...............................................   1      0   s06
............gguagauagaacaggucuug..................................................   1      0   s06
..................................................agaccuacuuaucuaccaaca...........   4      0   s06
..................................................agaccuacuuaucuaccaacag..........   11     0   s06
..................................................agaccuacuuaucuaccaacagc.........   1      0   s06
...................................................gaccuacuuaucuaccaaca...........   1      0   s06

.........aaagguagauagaacaggucu....................................................   1      0   HeL
.........aaagguagauagaacaggucuu...................................................   4      0   HeL
.........aaagguagauagaacaggucuug..................................................   2      0   HeL
..........aagguagauagaacaggu......................................................   77     0   HeL
..........aagguagauagaacagguc.....................................................   3      0   HeL
..........aagguagauagaacaggucu....................................................   5      0   HeL
..........aagguagauagaacaggucuu...................................................   46     0   HeL
..........aagguagauagaacaggucuug..................................................   232    0   HeL
..........aagguagauagaacaggucuugu.................................................   64     0   HeL
...........agguagauagaacaggucuu...................................................   8      0   HeL
...........agguagauagaacaggucuug..................................................   83     0   HeL
...........agguagauagaacaggucuugu.................................................   9      0   HeL
...........agguagauagaacaggucuuguu................................................   41     0   HeL
...........agguagauagaacaggucuuguuu...............................................   4      0   HeL
............gguagauagaacaggucuug..................................................   2      0   HeL
............gguagauagaacaggucuugu.................................................   2      0   HeL
..............uagauagaacaggucuugu.................................................   1      0   HeL
...............agauagaacaggucuugu.................................................   1      0   HeL
................gauagaacaggucuuguu................................................   2      0   HeL
................................................caagaccuacuuaucuaccaa.............   2      0   HeL
..................................................agaccuacuuaucuacca..............   2      0   HeL
..................................................agaccuacuuaucuaccaac............   3      0   HeL
..................................................agaccuacuuaucuaccaaca...........   18     0   HeL
..................................................agaccuacuuaucuaccaacag..........   52     0   HeL
..................................................agaccuacuuaucuaccaacagc.........   12     0   HeL
....................................................accuacuuaucuaccaaca...........   3      0   HeL
....................................................accuacuuaucuaccaacagc.........   1      0   HeL
.....................................................ccuacuuaucuaccaacag..........   5      0   HeL

........aaaagguagauagaacaggu......................................................   2      0   ku4
........aaaagguagauagaacaggucu....................................................   1      0   ku4
........aaaagguagauagaacaggucuu...................................................   1      0   ku4
.........aaagguagauagaacaggu......................................................   7      0   ku4
.........aaagguagauagaacagguc.....................................................   7      0   ku4
.........aaagguagauagaacaggucu....................................................   32     0   ku4
```

Mature **Star**

```
ccuaaaugaaaagguagauagaacaggucuuguuugcaaaauaaauucaagaccuacuuaucuaccaacagcaauuacacg
.........aaagguagauagaacaggucuu..................................................   3      0   ku4
.........aaagguagauagaacaggucuug.................................................   68     0   ku4
.........aaagguagauagaacaggucuugu................................................   5      0   ku4
..........aagguagauagaacaggu.....................................................   91     0   ku4
..........aagguagauagaacagguc....................................................   249    0   ku4
..........aagguagauagaacaggucu...................................................   1472   0   ku4
..........aagguagauagaacaggucuu..................................................   529    0   ku4
..........aagguagauagaacaggucuug.................................................   5619   0   ku4
..........aagguagauagaacaggucuugu................................................   580    0   ku4
..........aagguagauagaacaggucuuguu...............................................   13     0   ku4
..........aagguagauagaacaggucuuguuu..............................................   1      0   ku4
...........agguagauagaacagguc....................................................   55     0   ku4
...........agguagauagaacaggucu...................................................   289    0   ku4
...........agguagauagaacaggucuu..................................................   260    0   ku4
...........agguagauagaacaggucuug.................................................   4078   0   ku4
...........agguagauagaacaggucuugu................................................   1008   0   ku4
...........agguagauagaacaggucuuguu...............................................   595    0   ku4
...........agguagauagaacaggucuuguuu..............................................   10     0   ku4
............gguagauagaacaggucuug.................................................   1      0   ku4
.................................................agaccuacuuaucuaccaaca...........   18     0   ku4
.................................................agaccuacuuaucuaccaacag..........   1      0   ku4

........aaaagguagauagaacaggucuu..................................................   1      0   s07
........aaaagguagauagaacaggucuug.................................................   1      0   s07
........aaaagguagauagaacaggucuugu................................................   1      0   s07
.........aaagguagauagaacaggu.....................................................   1      0   s07
.........aaagguagauagaacaggucu...................................................   1      0   s07
.........aaagguagauagaacaggucuu..................................................   4      0   s07
.........aaagguagauagaacaggucuug.................................................   58     0   s07
.........aaagguagauagaacaggucuugu................................................   2      0   s07
..........aagguagauagaacaggu.....................................................   9      0   s07
..........aagguagauagaacagguc....................................................   43     0   s07
..........aagguagauagaacaggucu...................................................   74     0   s07
..........aagguagauagaacaggucuu..................................................   253    0   s07
..........aagguagauagaacaggucuug.................................................   3008   0   s07
..........aagguagauagaacaggucuugu................................................   632    0   s07
..........aagguagauagaacaggucuuguu...............................................   3      0   s07
...........agguagauagaacagguc....................................................   29     0   s07
...........agguagauagaacaggucu...................................................   38     0   s07
...........agguagauagaacaggucuu..................................................   114    0   s07
...........agguagauagaacaggucuug.................................................   1847   0   s07
...........agguagauagaacaggucuugu................................................   178    0   s07
...........agguagauagaacaggucuuguu...............................................   61     0   s07
...........agguagauagaacaggucuuguuu..............................................   2      0   s07
...............................................caagaccuacuuaucuaccaacag..........   2      0   s07
................................................aagaccuacuuaucuaccaac............   2      0   s07
.................................................agaccuacuuaucuaccaaca...........   1      0   s07
.................................................agaccuacuuaucuaccaacag..........   14     0   s07
.................................................agaccuacuuaucuaccaacagc.........   1      0   s07
.................................................agaccuacuuaucuaccaacagca........   1      0   s07

........aaaagguagauagaacaggucu...................................................   1      0   s10
........aaaagguagauagaacaggucuu..................................................   1      0   s10
........aaaagguagauagaacaggucuug.................................................   3      0   s10
.........aaagguagauagaacaggucu...................................................   2      0   s10
.........aaagguagauagaacaggucuu..................................................   4      0   s10
.........aaagguagauagaacaggucuug.................................................   51     0   s10
.........aaagguagauagaacaggucuugu................................................   2      0   s10
..........aagguagauagaacaggu.....................................................   8      0   s10
..........aagguagauagaacagguc....................................................   34     0   s10
..........aagguagauagaacaggucu...................................................   47     0   s10
..........aagguagauagaacaggucuu..................................................   126    0   s10
..........aagguagauagaacaggucuug.................................................   3029   0   s10
..........aagguagauagaacaggucuugu................................................   310    0   s10
..........aagguagauagaacaggucuuguu...............................................   6      0   s10
...........agguagauagaacagguc....................................................   15     0   s10
...........agguagauagaacaggucu...................................................   14     0   s10
...........agguagauagaacaggucuu..................................................   49     0   s10
...........agguagauagaacaggucuug.................................................   1341   0   s10
...........agguagauagaacaggucuugu................................................   106    0   s10
```

Mature Star

```
ccuaaaugaaaagguagauagaacaggucuuguuugcaaaauaaauucaagaccuacuuaucuaccaacagcaauuacacg
...........agguagauagaacaggucuuguu...............................................	87	0	s10
...........agguagauagaacaggucuuguuu..............................................	4	0	s10
............gguagauagaacaggucuug.................................................	1	0	s10
.................................................agaccuacuuaucuaccaaca...........	6	0	s10
.................................................agaccuacuuaucuaccaacag..........	8	0	s10
.................................................agaccuacuuaucuaccaacagc.........	2	0	s10

........aaaagguagauagaacaggucu...................................................	6	0	k08
........aaaagguagauagaacaggucuu..................................................	11	0	k08
........aaaagguagauagaacaggucuug.................................................	5	0	k08
........aaaagguagauagaacaggucuugu................................................	1	0	k08
.........aaagguagauagaacaggu.....................................................	3	0	k08
.........aaagguagauagaacagguc....................................................	1	0	k08
.........aaagguagauagaacaggucu...................................................	9	0	k08
.........aaagguagauagaacaggucuu..................................................	29	0	k08
.........aaagguagauagaacaggucuug.................................................	138	0	k08
.........aaagguagauagaacaggucuugu................................................	5	0	k08
..........aagguagauagaacaggu.....................................................	39	0	k08
..........aagguagauagaacagguc....................................................	85	0	k08
..........aagguagauagaacaggucu...................................................	247	0	k08
..........aagguagauagaacaggucuu..................................................	919	0	k08
..........aagguagauagaacaggucuug.................................................	12162	0	k08
..........aagguagauagaacaggucuugu................................................	1214	0	k08
..........aagguagauagaacaggucuuguu...............................................	26	0	k08
...........agguagauagaacagguc....................................................	25	0	k08
...........agguagauagaacaggucu...................................................	62	0	k08
...........agguagauagaacaggucuu..................................................	282	0	k08
...........agguagauagaacaggucuug.................................................	4011	0	k08
...........agguagauagaacaggucuugu................................................	674	0	k08
...........agguagauagaacaggucuuguu...............................................	353	0	k08
...........agguagauagaacaggucuuguuu..............................................	19	0	k08
............gguagauagaacaggucuug.................................................	3	0	k08
.................................................agaccuacuuaucuaccaac............	1	0	k08
.................................................agaccuacuuaucuaccaaca...........	3	0	k08
.................................................agaccuacuuaucuaccaacag..........	2	0	k08
.................................................agaccuacuuaucuaccaacagc.........	1	0	k08

.........aaagguagauagaacagguc....................................................	2	0	ta3
.........aaagguagauagaacaggucuu..................................................	3	0	ta3
.........aaagguagauagaacaggucuug.................................................	18	0	ta3
.........aaagguagauagaacaggucuugu................................................	3	0	ta3
..........aagguagauagaacaggu.....................................................	18	0	ta3
..........aagguagauagaacagguc....................................................	28	0	ta3
..........aagguagauagaacaggucu...................................................	74	0	ta3
..........aagguagauagaacaggucuu..................................................	232	0	ta3
..........aagguagauagaacaggucuug.................................................	1951	0	ta3
..........aagguagauagaacaggucuugu................................................	404	0	ta3
..........aagguagauagaacaggucuuguu...............................................	6	0	ta3
...........agguagauagaacagguc....................................................	19	0	ta3
...........agguagauagaacaggucu...................................................	31	0	ta3
...........agguagauagaacaggucuu..................................................	108	0	ta3
...........agguagauagaacaggucuug.................................................	1186	0	ta3
...........agguagauagaacaggucuugu................................................	236	0	ta3
...........agguagauagaacaggucuuguu...............................................	64	0	ta3
...........agguagauagaacaggucuuguuu..............................................	7	0	ta3
............gguagauagaacaggucuug.................................................	3	0	ta3
............gguagauagaacaggucuugu................................................	1	0	ta3
..............uagauagaacaggucuug.................................................	1	0	ta3
.................................................agaccuacuuaucuacca..............	1	0	ta3
.................................................agaccuacuuaucuaccaa.............	2	0	ta3
.................................................agaccuacuuaucuaccaac............	3	0	ta3
.................................................agaccuacuuaucuaccaaca...........	31	0	ta3
.................................................agaccuacuuaucuaccaacag..........	25	0	ta3
.................................................agaccuacuuaucuaccaacagc.........	3	0	ta3

.........aaagguagauagaacaggucuug.................................................	34	0	j06
..........aagguagauagaacaggu.....................................................	2	0	j06
..........aagguagauagaacagguc....................................................	25	0	j06
..........aagguagauagaacaggucu...................................................	120	0	j06
..........aagguagauagaacaggucuu..................................................	197	0	j06
```

```
          Mature                                Star
ccuaaaugaaaagguagauagaacaggucuuguuugcaaaauaaaauucaagaccuacuuaucuaccaacagcaauuacacg
..........aagguagauagaacaggucuug..................................................    2418    0    j06
..........aagguagauagaacaggucuugu.................................................    465     0    j06
...........agguagauagaacagguc.....................................................    9       0    j06
...........agguagauagaacaggucu....................................................    11      0    j06
...........agguagauagaacaggucuu...................................................    7       0    j06
...........agguagauagaacaggucuug..................................................    939     0    j06
...........agguagauagaacaggucuugu.................................................    134     0    j06
...........agguagauagaacaggucuuguu................................................    148     0    j06
...........agguagauagaacaggucuuguuu...............................................    13      0    j06
.............guagauagaacaggucuu...................................................    2       0    j06

........aaaagguagauagaacagg.......................................................    1       0    j10
........aaaagguagauagaacagguc.....................................................    1       0    j10
........aaaagguagauagaacaggucu....................................................    2       0    j10
........aaaagguagauagaacaggucuu...................................................    5       0    j10
........aaaagguagauagaacaggucuug..................................................    5       0    j10
........aaaagguagauagaacaggucuugu.................................................    3       0    j10
.........aaagguagauagaacaggucu....................................................    5       0    j10
.........aaagguagauagaacaggucuu...................................................    16      0    j10
.........aaagguagauagaacaggucuug..................................................    226     0    j10
.........aaagguagauagaacaggucuugu.................................................    25      0    j10
..........aagguagauagaacaggu......................................................    51      0    j10
..........aagguagauagaacagguc.....................................................    114     0    j10
..........aagguagauagaacaggucu....................................................    199     0    j10
..........aagguagauagaacaggucuu...................................................    694     0    j10
..........aagguagauagaacaggucuug..................................................    14274   0    j10
..........aagguagauagaacaggucuugu.................................................    2832    0    j10
..........aagguagauagaacaggucuuguu................................................    38      0    j10
..........aagguagauagaacaggucuuguuu...............................................    1       0    j10
...........agguagauagaacagguc.....................................................    59      0    j10
...........agguagauagaacaggucu....................................................    60      0    j10
...........agguagauagaacaggucuu...................................................    174     0    j10
...........agguagauagaacaggucuug..................................................    6121    0    j10
...........agguagauagaacaggucuugu.................................................    1152    0    j10
...........agguagauagaacaggucuuguu................................................    1503    0    j10
...........agguagauagaacaggucuuguuu...............................................    101     0    j10
............gguagauagaacaggucu....................................................    1       0    j10
............gguagauagaacaggucuug..................................................    1       0    j10
............gguagauagaacaggucuugu.................................................    1       0    j10
.............guagauagaacaggucuug..................................................    1       0    j10
................................................caagaccuacuuaucuacca..............    1       0    j10
................................................caagaccuacuuaucuaccaa.............    1       0    j10
..................................................agaccuacuuaucuaccaac............    1       0    j10
..................................................agaccuacuuaucuaccaaca...........    7       0    j10
..................................................agaccuacuuaucuaccaacag..........    5       0    j10
..................................................agaccuacuuaucuaccaacagca........    1       0    j10
....................................................accuacuuaucuaccaacagca........    1       0    j10

.........aaagguagauagaacaggucu....................................................    6       0    j04
.........aaagguagauagaacaggucuug..................................................    11      0    j04
..........aagguagauagaacaggu......................................................    1       0    j04
..........aagguagauagaacagguc.....................................................    24      0    j04
..........aagguagauagaacaggucu....................................................    107     0    j04
..........aagguagauagaacaggucuu...................................................    82      0    j04
..........aagguagauagaacaggucuug..................................................    993     0    j04
..........aagguagauagaacaggucuugu.................................................    304     0    j04
..........aagguagauagaacaggucuuguu................................................    1       0    j04
..........aagguagauagaacaggucuuguuu...............................................    4       0    j04
...........agguagauagaacagguc.....................................................    10      0    j04
...........agguagauagaacaggucu....................................................    21      0    j04
...........agguagauagaacaggucuu...................................................    21      0    j04
...........agguagauagaacaggucuug..................................................    523     0    j04
...........agguagauagaacaggucuugu.................................................    229     0    j04
...........agguagauagaacaggucuuguu................................................    110     0    j04
...........agguagauagaacaggucuuguuu...............................................    6       0    j04

.........aaagguagauagaacagguc.....................................................    1       0    j02
.........aaagguagauagaacaggucu....................................................    1       0    j02
.........aaagguagauagaacaggucuu...................................................    1       0    j02
.........aaagguagauagaacaggucuug..................................................    29      0    j02
```

Mature **Star**

```
ccuaaaugaaaagguagauagaacaggucuuguuugcaaaauaaaauucaagaccuacuuaucuaccaacagcaauuacacg
.........aaagguagauagaacaggucuugu.................................................      1       0       j02
..........aagguagauagaacaggu......................................................      5       0       j02
..........aagguagauagaacagguc.....................................................      21      0       j02
..........aagguagauagaacaggucu....................................................      133     0       j02
..........aagguagauagaacaggucuu...................................................      140     0       j02
..........aagguagauagaacaggucuug..................................................      1590    0       j02
..........aagguagauagaacaggucuugu.................................................      299     0       j02
..........aagguagauagaacaggucuuguu................................................      6       0       j02
...........agguagauagaacagguc.....................................................      17      0       j02
...........agguagauagaacaggucu....................................................      22      0       j02
...........agguagauagaacaggucuu...................................................      29      0       j02
...........agguagauagaacaggucuug..................................................      721     0       j02
...........agguagauagaacaggucuugu.................................................      167     0       j02
...........agguagauagaacaggucuuguu................................................      118     0       j02
...........agguagauagaacaggucuuguuu...............................................      3       0       j02

........aaaagguagauagaacaggucuu...................................................      2       0       va2
........aaaagguagauagaacaggucuug..................................................      3       0       va2
.........aaagguagauagaacaggu......................................................      1       0       va2
.........aaagguagauagaacagguc.....................................................      4       0       va2
.........aaagguagauagaacaggucu....................................................      7       0       va2
.........aaagguagauagaacaggucuu...................................................      10      0       va2
.........aaagguagauagaacaggucuug..................................................      78      0       va2
.........aaagguagauagaacaggucuugu.................................................      7       0       va2
..........aagguagauagaacaggu......................................................      22      0       va2
..........aagguagauagaacagguc.....................................................      28      0       va2
..........aagguagauagaacaggucu....................................................      148     0       va2
..........aagguagauagaacaggucuu...................................................      197     0       va2
..........aagguagauagaacaggucuug..................................................      4514    0       va2
..........aagguagauagaacaggucuugu.................................................      1635    0       va2
..........aagguagauagaacaggucuuguu................................................      21      0       va2
..........aagguagauagaacaggucuuguuu...............................................      1       0       va2
...........agguagauagaacagguc.....................................................      18      0       va2
...........agguagauagaacaggucu....................................................      18      0       va2
...........agguagauagaacaggucuu...................................................      66      0       va2
...........agguagauagaacaggucuug..................................................      1743    0       va2
...........agguagauagaacaggucuugu.................................................      547     0       va2
...........agguagauagaacaggucuuguu................................................      163     0       va2
...........agguagauagaacaggucuuguuu...............................................      7       0       va2
..................................................agaccuacuuaucuaccaac............      1       0       va2
..................................................agaccuacuuaucuaccaaca...........      9       0       va2
..................................................agaccuacuuaucuaccaacag..........      13      0       va2
..................................................agaccuacuuaucuaccaacagc.........      2       0       va2
..................................................agaccuacuuaucuaccaacagca........      1       0       va2
..................................................agaccuacuuaucuaccaacagcaa.......      1       0       va2

........aaaagguagauagaacaggu......................................................      1       0       k14
........aaaagguagauagaacaggucu....................................................      4       0       k14
........aaaagguagauagaacaggucuu...................................................      16      0       k14
........aaaagguagauagaacaggucuug..................................................      6       0       k14
........aaaagguagauagaacaggucuugu.................................................      1       0       k14
.........aaagguagauagaacagg.......................................................      1       0       k14
.........aaagguagauagaacaggu......................................................      1       0       k14
.........aaagguagauagaacagguc.....................................................      2       0       k14
.........aaagguagauagaacaggucu....................................................      9       0       k14
.........aaagguagauagaacaggucuu...................................................      42      0       k14
.........aaagguagauagaacaggucuug..................................................      167     0       k14
.........aaagguagauagaacaggucuugu.................................................      8       0       k14
.........aaagguagauagaacaggucuuguu................................................      1       0       k14
..........aagguagauagaacaggu......................................................      74      0       k14
..........aagguagauagaacagguc.....................................................      108     0       k14
..........aagguagauagaacaggucu....................................................      318     0       k14
..........aagguagauagaacaggucuu...................................................      1383    0       k14
..........aagguagauagaacaggucuug..................................................      19252   0       k14
..........aagguagauagaacaggucuugu.................................................      1680    0       k14
..........aagguagauagaacaggucuuguu................................................      18      0       k14
...........agguagauagaacagguc.....................................................      41      0       k14
...........agguagauagaacaggucu....................................................      78      0       k14
...........agguagauagaacaggucuu...................................................      359     0       k14
...........agguagauagaacaggucuug..................................................      6749    0       k14
```

Mature Star

```
ccuaaaugaaaagguagauagaacaggucuuguuugcaaaauaaauucaagaccuacuuaucuaccaacagcaauuacacg
...........agguagauagaacaggucuugu................................................   957     0     k14
...........agguagauagaacaggucuuguu...............................................   405     0     k14
...........agguagauagaacaggucuuguuu..............................................   11      0     k14
..............uagauagaacaggucuug.................................................   1       0     k14
.................................................agaccuacuuaucuaccaaca...........   4       0     k14

........aaaagguagauagaacaggucuug.................................................   1       0     j31
.........aaagguagauagaacagguc....................................................   1       0     j31
.........aaagguagauagaacaggucuu..................................................   3       0     j31
.........aaagguagauagaacaggucuug.................................................   32      0     j31
.........aaagguagauagaacaggucuugu................................................   1       0     j31
..........aagguagauagaacaggu.....................................................   4       0     j31
..........aagguagauagaacagguc....................................................   21      0     j31
..........aagguagauagaacaggucu...................................................   29      0     j31
..........aagguagauagaacaggucuu..................................................   79      0     j31
..........aagguagauagaacaggucuug.................................................   2686    0     j31
..........aagguagauagaacaggucuugu................................................   267     0     j31
..........aagguagauagaacaggucuuguu...............................................   1       0     j31
...........agguagauagaacagguc....................................................   13      0     j31
...........agguagauagaacaggucu...................................................   9       0     j31
...........agguagauagaacaggucuu..................................................   21      0     j31
...........agguagauagaacaggucuug.................................................   1136    0     j31
...........agguagauagaacaggucuugu................................................   127     0     j31
...........agguagauagaacaggucuuguu...............................................   163     0     j31
...........agguagauagaacaggucuuguuu..............................................   11      0     j31
.................................................agaccuacuuaucuaccaaca...........   1       0     j31
.................................................agaccuacuuaucuaccaacag..........   2       0     j31

........aaaagguagauagaacaggu.....................................................   1       0     j27
........aaaagguagauagaacaggucuu..................................................   2       0     j27
........aaaagguagauagaacaggucuug.................................................   1       0     j27
.........aaagguagauagaacagg......................................................   1       0     j27
.........aaagguagauagaacaggu.....................................................   15      0     j27
.........aaagguagauagaacagguc....................................................   15      0     j27
.........aaagguagauagaacaggucu...................................................   33      0     j27
.........aaagguagauagaacaggucuu..................................................   26      0     j27
.........aaagguagauagaacaggucuug.................................................   93      0     j27
.........aaagguagauagaacaggucuugu................................................   8       0     j27
..........aagguagauagaacaggu.....................................................   191     0     j27
..........aagguagauagaacagguc....................................................   518     0     j27
..........aagguagauagaacaggucu...................................................   2030    0     j27
..........aagguagauagaacaggucuu..................................................   825     0     j27
..........aagguagauagaacaggucuug.................................................   7685    0     j27
..........aagguagauagaacaggucuugu................................................   1188    0     j27
..........aagguagauagaacaggucuuguu...............................................   18      0     j27
..........aagguagauagaacaggucuuguuu..............................................   2       0     j27
...........agguagauagaacagguc....................................................   94      0     j27
...........agguagauagaacaggucu...................................................   391     0     j27
...........agguagauagaacaggucuu..................................................   334     0     j27
...........agguagauagaacaggucuug.................................................   5253    0     j27
...........agguagauagaacaggucuugu................................................   1171    0     j27
...........agguagauagaacaggucuuguu...............................................   722     0     j27
...........agguagauagaacaggucuuguuu..............................................   23      0     j27
............gguagauagaacaggucu...................................................   1       0     j27
............gguagauagaacaggucuug.................................................   6       0     j27
...............................................caagaccuacuuaucuaccaa.............   1       0     j27
...............................................caagaccuacuuaucuaccaac............   2       0     j27
.................................................agaccuacuuaucuaccaaca...........   13      0     j27

..........aagguagauagaacagguc....................................................   14      0     j05
..........aagguagauagaacaggucu...................................................   31      0     j05
..........aagguagauagaacaggucuu..................................................   75      0     j05
..........aagguagauagaacaggucuug.................................................   240     0     j05
..........aagguagauagaacaggucuugu................................................   24      0     j05
...........agguagauagaacagguc....................................................   16      0     j05
...........agguagauagaacaggucuu..................................................   25      0     j05
...........agguagauagaacaggucuug.................................................   195     0     j05
...........agguagauagaacaggucuugu................................................   43      0     j05
...........agguagauagaacaggucuuguu...............................................   25      0     j05

........aaaagguagauagaacaggucuug.................................................   4       0     s03
```

Mature **Star**

ccuaaaugaaaagguagauagaacaggucuuguuugcaaaauaaaauucaagaccuacuuaucuaccaacagcaauuacacg

........aaaagguagauagaacaggucuugu...	1	0	s03
.........aaagguagauagaacaggu..	1	0	s03
.........aaagguagauagaacagguc...	2	0	s03
.........aaagguagauagaacaggucuu...	4	0	s03
.........aaagguagauagaacaggucuug..	41	0	s03
.........aaagguagauagaacaggucuugu...	3	0	s03
..........aagguagauagaacaggu..	12	0	s03
..........aagguagauagaacagguc...	40	0	s03
..........aagguagauagaacaggucu..	50	0	s03
..........aagguagauagaacaggucuu...	169	0	s03
..........aagguagauagaacaggucuug..	3072	0	s03
..........aagguagauagaacaggucuugu...	705	0	s03
..........aagguagauagaacaggucuuguu..	11	0	s03
...........agguagauagaacagguc...	20	0	s03
...........agguagauagaacaggucu..	14	0	s03
...........agguagauagaacaggucuu...	74	0	s03
...........agguagauagaacaggucuug..	1985	0	s03
...........agguagauagaacaggucuugu...	217	0	s03
...........agguagauagaacaggucuuguu..	281	0	s03
...........agguagauagaacaggucuuguuu...	27	0	s03
............gguagauagaacaggucuug..	2	0	s03
....caagaccuacuuaucuaccaa.............	1	0	s03
....agaccuacuuaucuaccaaca...........	6	0	s03
....agaccuacuuaucuaccaacag.........	5	0	s03
....agaccuacuuaucuaccaacagca.......	1	0	s03
........aaaagguagauagaacaggucu..	2	0	H52
.........aaagguagauagaacaggu..	2	0	H52
.........aaagguagauagaacaggucu..	2	0	H52
.........aaagguagauagaacaggucuu...	1	0	H52
.........aaagguagauagaacaggucuug..	5	0	H52
.........aaagguagauagaacaggucuugu...	1	0	H52
..........aagguagauagaacaggu..	42	0	H52
..........aagguagauagaacaggucu..	16	0	H52
..........aagguagauagaacaggucuu...	26	0	H52
..........aagguagauagaacaggucuug..	253	0	H52
..........aagguagauagaacaggucuugu...	49	0	H52
..........aagguagauagaacaggucuuguuu...	2	0	H52
...........agguagauagaacagguc...	1	0	H52
...........agguagauagaacaggucu..	8	0	H52
...........agguagauagaacaggucuu...	8	0	H52
...........agguagauagaacaggucuug..	89	0	H52
...........agguagauagaacaggucuugu...	17	0	H52
...........agguagauagaacaggucuuguu..	3	0	H52
............gguagauagaacaggucuugu...	3	0	H52
..............uagauagaacaggucuugu...	1	0	H52
....caagaccuacuuaucuaccaac............	2	0	H52
....agaccuacuuaucuaccaa.............	1	0	H52
....agaccuacuuaucuaccaac............	3	0	H52
....agaccuacuuaucuaccaaca...........	4	0	H52
....agaccuacuuaucuaccaacag.........	49	0	H52
....agaccuacuuaucuaccaacagc........	60	0	H52
....agaccuacuuaucuaccaacagca.......	1	0	H52
....gaccuacuuaucuaccaacagc........	1	0	H52
....cuacuuaucuaccaacagc........	1	0	H52
........aaaagguagauagaacaggu..	1	0	j22
........aaaagguagauagaacaggucuu...	2	0	j22
........aaaagguagauagaacaggucuugu...	1	0	j22
.........aaagguagauagaacagg...	2	0	j22
.........aaagguagauagaacaggucu..	1	0	j22
.........aaagguagauagaacaggucuu...	15	0	j22
.........aaagguagauagaacaggucuug..	64	0	j22
.........aaagguagauagaacaggucuugu...	2	0	j22
..........aagguagauagaacaggu..	7	0	j22
..........aagguagauagaacagguc...	37	0	j22
..........aagguagauagaacaggucu..	159	0	j22
..........aagguagauagaacaggucuu...	367	0	j22
..........aagguagauagaacaggucuug..	5974	0	j22
..........aagguagauagaacaggucuugu...	606	0	j22

```
          Mature                 Star
ccuaaaugaaaagguagauagaacaggucuuguuugcaaaauaaaauucaagaccuacuuaucuaccaacagcaauuacacg
..........aagguagauagaacaggucuuguu................................................   13      0   j22
...........agguagauagaacagguc.....................................................   21      0   j22
...........agguagauagaacaggucu....................................................   37      0   j22
...........agguagauagaacaggucuu...................................................   93      0   j22
...........agguagauagaacaggucuug..................................................   2484    0   j22
...........agguagauagaacaggucuugu.................................................   253     0   j22
...........agguagauagaacaggucuuguu................................................   224     0   j22
...........agguagauagaacaggucuuguuu...............................................   1       0   j22
............gguagauagaacaggucuuguuu...............................................   1       0   j22
..................................................agaccuacuuaucuaccaac............   1       0   j22
..................................................agaccuacuuaucuaccaaca...........   6       0   j22
..................................................agaccuacuuaucuaccaacag..........   2       0   j22

........aaaagguagauagaacaggucu....................................................   3       0   k09
........aaaagguagauagaacaggucuu...................................................   16      0   k09
........aaaagguagauagaacaggucuug..................................................   15      0   k09
........aaaagguagauagaacaggucuugu.................................................   2       0   k09
.........aaagguagauagaacaggu......................................................   1       0   k09
.........aaagguagauagaacagguc.....................................................   2       0   k09
.........aaagguagauagaacaggucu....................................................   4       0   k09
.........aaagguagauagaacaggucuu...................................................   24      0   k09
.........aaagguagauagaacaggucuug..................................................   116     0   k09
.........aaagguagauagaacaggucuugu.................................................   11      0   k09
..........aagguagauagaacaggu......................................................   39      0   k09
..........aagguagauagaacagguc.....................................................   100     0   k09
..........aagguagauagaacaggucu....................................................   303     0   k09
..........aagguagauagaacaggucuu...................................................   1058    0   k09
..........aagguagauagaacaggucuug..................................................   12652   0   k09
..........aagguagauagaacaggucuugu.................................................   1867    0   k09
..........aagguagauagaacaggucuuguu................................................   33      0   k09
..........aagguagauagaacaggucuuguuu...............................................   4       0   k09
...........agguagauagaacagguc.....................................................   27      0   k09
...........agguagauagaacaggucu....................................................   81      0   k09
...........agguagauagaacaggucuu...................................................   266     0   k09
...........agguagauagaacaggucuug..................................................   4289    0   k09
...........agguagauagaacaggucuugu.................................................   786     0   k09
...........agguagauagaacaggucuuguu................................................   364     0   k09
...........agguagauagaacaggucuuguuu...............................................   30      0   k09
.................................................aagaccuacuuaucuaccaaca...........   1       0   k09
..................................................agaccuacuuaucuaccaaca...........   4       0   k09
..................................................agaccuacuuaucuaccaacagca........   1       0   k09

.........aaagguagauagaacaggucu....................................................   1       0   B14
.........aaagguagauagaacaggucuugu.................................................   1       0   B14
..........aagguagauagaacaggu......................................................   17      0   B14
..........aagguagauagaacaggucu....................................................   12      0   B14
..........aagguagauagaacaggucuu...................................................   36      0   B14
..........aagguagauagaacaggucuug..................................................   142     0   B14
..........aagguagauagaacaggucuugu.................................................   91      0   B14
..........aagguagauagaacaggucuuguu................................................   2       0   B14
..........aagguagauagaacaggucuuguuu...............................................   1       0   B14
...........agguagauagaacagguc.....................................................   2       0   B14
...........agguagauagaacaggucu....................................................   4       0   B14
...........agguagauagaacaggucuu...................................................   12      0   B14
...........agguagauagaacaggucuug..................................................   66      0   B14
...........agguagauagaacaggucuugu.................................................   23      0   B14
...........agguagauagaacaggucuuguu................................................   7       0   B14
............gguagauagaacaggucuu...................................................   1       0   B14
............gguagauagaacaggucuug..................................................   2       0   B14
............gguagauagaacaggucuugu.................................................   1       0   B14
.............guagauagaacaggucuugu.................................................   1       0   B14
..............uagauagaacaggucuugu.................................................   3       0   B14
...............agauagaacaggucuugu.................................................   1       0   B14
................................................caagaccuacuuaucuaccaac............   1       0   B14
..................................................agaccuacuuaucuaccaac............   4       0   B14
..................................................agaccuacuuaucuaccaaca...........   4       0   B14
..................................................agaccuacuuaucuaccaacag..........   29      0   B14
..................................................agaccuacuuaucuaccaacagc.........   41      0   B14
..................................................agaccuacuuaucuaccaacagca........   1       0   B14
...................................................gaccuacuuaucuaccaac............   1       0   B14
```

Mature **Star**

```
ccuaaaugaaaagguagauagaacaggucuuguuugcaaaauaaauucaagaccuacuuaucuaccaacagcaauuacacg
..................................................gaccuacuuaucuaccaacagca........   1      0    B14
...................................................accuacuuaucuaccaacagc.........   1      0    B14
...................................................accuacuuaucuaccaacagca........   2      0    B14
....................................................ccuacuuaucuaccaacagca........   2      0    B14

........aaaagguagauagaacagguc....................................................   2      0    j09
........aaaagguagauagaacaggucu...................................................   1      0    j09
.........aaagguagauagaacaggu.....................................................   3      0    j09
.........aaagguagauagaacagguc....................................................   1      0    j09
.........aaagguagauagaacaggucu...................................................   3      0    j09
.........aaagguagauagaacaggucuu..................................................   5      0    j09
.........aaagguagauagaacaggucuug.................................................   16     0    j09
.........aaagguagauagaacaggucuugu................................................   1      0    j09
..........aagguagauagaacaggu.....................................................   12     0    j09
..........aagguagauagaacagguc....................................................   27     0    j09
..........aagguagauagaacaggucu...................................................   140    0    j09
..........aagguagauagaacaggucuu..................................................   195    0    j09
..........aagguagauagaacaggucuug.................................................   1870   0    j09
..........aagguagauagaacaggucuugu................................................   361    0    j09
..........aagguagauagaacaggucuuguu...............................................   8      0    j09
...........agguagauagaacagguc....................................................   6      0    j09
...........agguagauagaacaggucu...................................................   29     0    j09
...........agguagauagaacaggucuu..................................................   54     0    j09
...........agguagauagaacaggucuug.................................................   871    0    j09
...........agguagauagaacaggucuugu................................................   248    0    j09
...........agguagauagaacaggucuuguu...............................................   149    0    j09
...........agguagauagaacaggucuuguuu..............................................   2      0    j09
.................................................agaccuacuuaucuaccaaca...........   2      0    j09
.................................................agaccuacuuaucuaccaacag..........   2      0    j09

........aaaagguagauagaacaggu.....................................................   1      0    j18
........aaaagguagauagaacaggucuug.................................................   3      0    j18
.........aaagguagauagaacagg......................................................   2      0    j18
.........aaagguagauagaacaggu.....................................................   6      0    j18
.........aaagguagauagaacagguc....................................................   3      0    j18
.........aaagguagauagaacaggucu...................................................   18     0    j18
.........aaagguagauagaacaggucuu..................................................   11     0    j18
.........aaagguagauagaacaggucuug.................................................   58     0    j18
.........aaagguagauagaacaggucuugu................................................   1      0    j18
..........aagguagauagaacaggu.....................................................   38     0    j18
..........aagguagauagaacagguc....................................................   135    0    j18
..........aagguagauagaacaggucu...................................................   814    0    j18
..........aagguagauagaacaggucuu..................................................   528    0    j18
..........aagguagauagaacaggucuug.................................................   5622   0    j18
..........aagguagauagaacaggucuugu................................................   622    0    j18
..........aagguagauagaacaggucuuguu...............................................   16     0    j18
...........agguagauagaacagguc....................................................   53     0    j18
...........agguagauagaacaggucu...................................................   132    0    j18
...........agguagauagaacaggucuu..................................................   154    0    j18
...........agguagauagaacaggucuug.................................................   2667   0    j18
...........agguagauagaacaggucuugu................................................   620    0    j18
...........agguagauagaacaggucuuguu...............................................   339    0    j18
...........agguagauagaacaggucuuguuu..............................................   9      0    j18
................................................aagaccuacuuaucuaccaaca...........   1      0    j18
.................................................agaccuacuuaucuaccaaca...........   6      0    j18

........aaaagguagauagaacaggu.....................................................   2      0    j16
........aaaagguagauagaacagguc....................................................   2      0    j16
........aaaagguagauagaacaggucu...................................................   3      0    j16
........aaaagguagauagaacaggucuu..................................................   1      0    j16
........aaaagguagauagaacaggucuug.................................................   12     0    j16
........aaaagguagauagaacaggucuugu................................................   1      0    j16
.........aaagguagauagaacaggu.....................................................   3      0    j16
.........aaagguagauagaacagguc....................................................   6      0    j16
.........aaagguagauagaacaggucu...................................................   22     0    j16
.........aaagguagauagaacaggucuu..................................................   19     0    j16
.........aaagguagauagaacaggucuug.................................................   270    0    j16
.........aaagguagauagaacaggucuugu................................................   10     0    j16
..........aagguagauagaacaggu.....................................................   78     0    j16
..........aagguagauagaacagguc....................................................   296    0    j16
```

```
          Mature                                  Star
ccuaaaugaaaagguagauagaacaggucuuguuugcaaaauaaaauucaagaccuacuuaucuaccaacagcaauuacacg
..........aagguagauagaacaggucu....................................................    1551    0    j16
..........aagguagauagaacaggucuu...................................................    1394    0    j16
..........aagguagauagaacaggucuug..................................................    22659   0    j16
..........aagguagauagaacaggucuugu.................................................    1899    0    j16
..........aagguagauagaacaggucuuguu................................................    19      0    j16
..........aagguagauagaacaggucuuguuu...............................................    2       0    j16
...........agguagauagaacagguc.....................................................    152     0    j16
...........agguagauagaacaggucu....................................................    397     0    j16
...........agguagauagaacaggucuu...................................................    541     0    j16
...........agguagauagaacaggucuug..................................................    14406   0    j16
...........agguagauagaacaggucuugu.................................................    1317    0    j16
...........agguagauagaacaggucuuguu................................................    919     0    j16
...........agguagauagaacaggucuuguuu...............................................    18      0    j16
............gguagauagaacaggucu....................................................    1       0    j16
..............uagauagaacaggucuug..................................................    1       0    j16
................................................caagaccuacuuaucuaccaac............    3       0    j16
................................................caagaccuacuuaucuaccaacag..........    1       0    j16
..................................................agaccuacuuaucuaccaa.............    1       0    j16
..................................................agaccuacuuaucuaccaaca...........    30      0    j16
..................................................agaccuacuuaucuaccaacag.........    8       0    j16
..................................................agaccuacuuaucuaccaacagca.......    1       0    j16

........aaaagguagauagaacagguc.....................................................    1       0    ku3
........aaaagguagauagaacaggucu....................................................    2       0    ku3
.........aaagguagauagaacaggu......................................................    3       0    ku3
.........aaagguagauagaacagguc.....................................................    4       0    ku3
.........aaagguagauagaacaggucu....................................................    29      0    ku3
.........aaagguagauagaacaggucuu...................................................    8       0    ku3
.........aaagguagauagaacaggucuug..................................................    70      0    ku3
.........aaagguagauagaacaggucuugu.................................................    6       0    ku3
..........aagguagauagaacaggu......................................................    18      0    ku3
..........aagguagauagaacagguc.....................................................    75      0    ku3
..........aagguagauagaacaggucu....................................................    637     0    ku3
..........aagguagauagaacaggucuu...................................................    401     0    ku3
..........aagguagauagaacaggucuug..................................................    5911    0    ku3
..........aagguagauagaacaggucuugu.................................................    1643    0    ku3
..........aagguagauagaacaggucuuguu................................................    30      0    ku3
..........aagguagauagaacaggucuuguuu...............................................    1       0    ku3
...........agguagauagaacagguc.....................................................    11      0    ku3
...........agguagauagaacaggucu....................................................    62      0    ku3
...........agguagauagaacaggucuu...................................................    95      0    ku3
...........agguagauagaacaggucuug..................................................    2313    0    ku3
...........agguagauagaacaggucuugu.................................................    953     0    ku3
...........agguagauagaacaggucuuguu................................................    539     0    ku3
...........agguagauagaacaggucuuguuu...............................................    26      0    ku3
..................................................agaccuacuuaucuaccaac............    3       0    ku3
..................................................agaccuacuuaucuaccaaca...........    15      0    ku3
..................................................agaccuacuuaucuaccaacag.........    2       0    ku3

........aaaagguagauagaacaggucuugu.................................................    1       0    ku6
.........aaagguagauagaacagg.......................................................    1       0    ku6
.........aaagguagauagaacaggu......................................................    3       0    ku6
.........aaagguagauagaacaggucu....................................................    7       0    ku6
.........aaagguagauagaacaggucuu...................................................    7       0    ku6
.........aaagguagauagaacaggucuug..................................................    38      0    ku6
.........aaagguagauagaacaggucuugu.................................................    8       0    ku6
..........aagguagauagaacaggu......................................................    10      0    ku6
..........aagguagauagaacagguc.....................................................    45      0    ku6
..........aagguagauagaacaggucu....................................................    309     0    ku6
..........aagguagauagaacaggucuu...................................................    186     0    ku6
..........aagguagauagaacaggucuug..................................................    1834    0    ku6
..........aagguagauagaacaggucuugu.................................................    655     0    ku6
..........aagguagauagaacaggucuuguu................................................    11      0    ku6
..........aagguagauagaacaggucuuguuu...............................................    1       0    ku6
...........agguagauagaacagguc.....................................................    16      0    ku6
...........agguagauagaacaggucu....................................................    38      0    ku6
...........agguagauagaacaggucuu...................................................    55      0    ku6
...........agguagauagaacaggucuug..................................................    689     0    ku6
...........agguagauagaacaggucuugu.................................................    502     0    ku6
...........agguagauagaacaggucuuguu................................................    314     0    ku6
```

```
          Mature                                 Star
ccuaaaugaaaagguagauagaacaggucuuguuugcaaaauaaauucaagaccuacuuaucuaccaacagcaauuacacg
...........agguagauagaacaggucuuguuu..............................................   27     0     ku6
............gguagauagaacaggucuugu................................................   2      0     ku6
.................................................agaccuacuuaucuacca..............   1      0     ku6
.................................................agaccuacuuaucuaccaaca...........   8      0     ku6

........aaaagguagauagaacaggucuug.................................................   3      0     j11
.........aaagguagauagaacaggucu...................................................   3      0     j11
.........aaagguagauagaacaggucuu..................................................   5      0     j11
.........aaagguagauagaacaggucuug.................................................   37     0     j11
..........aagguagauagaacaggu.....................................................   6      0     j11
..........aagguagauagaacagguc....................................................   29     0     j11
..........aagguagauagaacaggucu...................................................   91     0     j11
..........aagguagauagaacaggucuu..................................................   231    0     j11
..........aagguagauagaacaggucuug.................................................   4282   0     j11
..........aagguagauagaacaggucuugu................................................   506    0     j11
..........aagguagauagaacaggucuuguu...............................................   4      0     j11
...........agguagauagaacagguc....................................................   24     0     j11
...........agguagauagaacaggucu...................................................   11     0     j11
...........agguagauagaacaggucuu..................................................   45     0     j11
...........agguagauagaacaggucuug.................................................   1848   0     j11
...........agguagauagaacaggucuugu................................................   207    0     j11
...........agguagauagaacaggucuuguu...............................................   222    0     j11
...........agguagauagaacaggucuuguuu..............................................   6      0     j11

........aaaagguagauagaacaggucuug.................................................   1      0     s12
.........aaagguagauagaacagguc....................................................   1      0     s12
.........aaagguagauagaacaggucuu..................................................   6      0     s12
.........aaagguagauagaacaggucuug.................................................   26     0     s12
..........aagguagauagaacaggu.....................................................   7      0     s12
..........aagguagauagaacagguc....................................................   45     0     s12
..........aagguagauagaacaggucu...................................................   46     0     s12
..........aagguagauagaacaggucuu..................................................   154    0     s12
..........aagguagauagaacaggucuug.................................................   3078   0     s12
..........aagguagauagaacaggucuugu................................................   275    0     s12
..........aagguagauagaacaggucuuguu...............................................   2      0     s12
...........agguagauagaacagguc....................................................   16     0     s12
...........agguagauagaacaggucu...................................................   10     0     s12
...........agguagauagaacaggucuu..................................................   44     0     s12
...........agguagauagaacaggucuug.................................................   1516   0     s12
...........agguagauagaacaggucuugu................................................   82     0     s12
...........agguagauagaacaggucuuguu...............................................   34     0     s12
............gguagauagaacaggucuug.................................................   1      0     s12
..............uagauagaacaggucuug.................................................   2      0     s12
.................................................agaccuacuuaucuaccaaca...........   1      0     s12
.................................................agaccuacuuaucuaccaacag..........   5      0     s12
.................................................agaccuacuuaucuaccaacagca........   4      0     s12
....................................................ccuacuuaucuaccaacagca........   1      0     s12

........aaaagguagauagaacaggu.....................................................   1      0     ya3
.........aaagguagauagaacagg......................................................   2      0     ya3
.........aaagguagauagaacagguc....................................................   1      0     ya3
.........aaagguagauagaacaggucu...................................................   3      0     ya3
.........aaagguagauagaacaggucuu..................................................   6      0     ya3
.........aaagguagauagaacaggucuug.................................................   14     0     ya3
.........aaagguagauagaacaggucuugu................................................   5      0     ya3
..........aagguagauagaacaggu.....................................................   32     0     ya3
..........aagguagauagaacagguc....................................................   132    0     ya3
..........aagguagauagaacaggucu...................................................   191    0     ya3
..........aagguagauagaacaggucuu..................................................   539    0     ya3
..........aagguagauagaacaggucuug.................................................   1368   0     ya3
..........aagguagauagaacaggucuugu................................................   428    0     ya3
..........aagguagauagaacaggucuuguu...............................................   13     0     ya3
..........aagguagauagaacaggucuuguuu..............................................   1      0     ya3
...........agguagauagaacagguc....................................................   50     0     ya3
...........agguagauagaacaggucu...................................................   172    0     ya3
...........agguagauagaacaggucuu..................................................   706    0     ya3
...........agguagauagaacaggucuug.................................................   1226   0     ya3
...........agguagauagaacaggucuugu................................................   442    0     ya3
...........agguagauagaacaggucuuguu...............................................   175    0     ya3
...........agguagauagaacaggucuuguuu..............................................   5      0     ya3
```

Mature **Star**

ccuaaaugaaaagguagauagaacaggucuuguuugcaaaauaaauucaagaccuacuuaucuaccaacagcaauuacacg			
............gguagauagaacaggucuu..	2	0	ya3
............gguagauagaacaggucuug...	3	0	ya3
............gguagauagaacaggucuugu..	2	0	ya3
....aagaccuacuuaucuaccaacagc.........	1	0	ya3
....agaccuacuuaucuacca..............	1	0	ya3
....agaccuacuuaucuaccaa.............	3	0	ya3
....agaccuacuuaucuaccaac............	1	0	ya3
....agaccuacuuaucuaccaaca...........	48	0	ya3
....agaccuacuuaucuaccaacag..........	12	0	ya3
....agaccuacuuaucuaccaacagc.........	5	0	ya3
....gaccuacuuaucuaccaaca...........	1	0	ya3
.........aaagguagauagaacaggu...	1	0	HEK
.........aaagguagauagaacaggucuug...	1	0	HEK
..........aagguagauagaacaggu...	16	0	HEK
..........aagguagauagaacaggucu...	1	0	HEK
..........aagguagauagaacaggucuu..	9	0	HEK
..........aagguagauagaacaggucuug...	91	0	HEK
..........aagguagauagaacaggucuugu..	27	0	HEK
...........agguagauagaacaggucuu..	6	0	HEK
...........agguagauagaacaggucuug...	26	0	HEK
...........agguagauagaacaggucuugu..	1	0	HEK
...............agauagaacaggucuugu..	4	0	HEK
....agaccuacuuaucuaccaac............	2	0	HEK
....agaccuacuuaucuaccaaca...........	3	0	HEK
....agaccuacuuaucuaccaacag..........	10	0	HEK
....agaccuacuuaucuaccaacagc.........	26	0	HEK
....agaccuacuuaucuaccaacagca........	1	0	HEK
....gaccuacuuaucuaccaacagc.........	3	0	HEK
....gaccuacuuaucuaccaacagcaau......	1	0	HEK
....ccuacuuaucuaccaacag..........	1	0	HEK
....cuacuuaucuaccaacagca........	1	0	HEK
........aaaagguagauagaacaggucuu..	1	0	la1
........aaaagguagauagaacaggucuug...	3	0	la1
.........aaagguagauagaacagguc..	1	0	la1
.........aaagguagauagaacaggucuu..	3	0	la1
.........aaagguagauagaacaggucuug...	77	0	la1
.........aaagguagauagaacaggucuugu..	10	0	la1
..........aagguagauagaacaggu...	7	0	la1
..........aagguagauagaacagguc..	27	0	la1
..........aagguagauagaacaggucu...	78	0	la1
..........aagguagauagaacaggucuu..	278	0	la1
..........aagguagauagaacaggucuug...	5809	0	la1
..........aagguagauagaacaggucuugu..	2209	0	la1
..........aagguagauagaacaggucuuguu...	39	0	la1
..........aagguagauagaacaggucuuguuu..	4	0	la1
...........agguagauagaacagguc..	9	0	la1
...........agguagauagaacaggucu...	15	0	la1
...........agguagauagaacaggucuu..	68	0	la1
...........agguagauagaacaggucuug...	1757	0	la1
...........agguagauagaacaggucuugu..	448	0	la1
...........agguagauagaacaggucuuguu...	230	0	la1
...........agguagauagaacaggucuuguuu..	22	0	la1
....agaccuacuuaucuaccaaca...........	5	0	la1
....agaccuacuuaucuaccaacag..........	3	0	la1
....agaccuacuuaucuaccaacagc.........	1	0	la1
........aaaagguagauagaacagg..	1	0	j15
........aaaagguagauagaacaggu...	3	0	j15
........aaaagguagauagaacagguc..	3	0	j15
........aaaagguagauagaacaggucu...	9	0	j15
........aaaagguagauagaacaggucuu..	3	0	j15
........aaaagguagauagaacaggucuug...	9	0	j15
........aaaagguagauagaacaggucuugu..	1	0	j15
.........aaagguagauagaacaggu...	13	0	j15
.........aaagguagauagaacagguc..	7	0	j15
.........aaagguagauagaacaggucu...	25	0	j15
.........aaagguagauagaacaggucuu..	24	0	j15
.........aaagguagauagaacaggucuug...	214	0	j15

Mature Star

```
ccuaaaugaaaagguagauagaacaggucuuguuugcaaaauaaauucaagaccuacuuaucuaccaacagcaauuacacg
.........aaagguagauagaacaggucuugu................................................    14      0    j15
..........aagguagauagaacaggu.....................................................    172     0    j15
..........aagguagauagaacagguc....................................................    396     0    j15
..........aagguagauagaacaggucu...................................................    1756    0    j15
..........aagguagauagaacaggucuu..................................................    1423    0    j15
..........aagguagauagaacaggucuug.................................................    19651   0    j15
..........aagguagauagaacaggucuugu................................................    2125    0    j15
..........aagguagauagaacaggucuuguu...............................................    37      0    j15
..........aagguagauagaacaggucuuguuu..............................................    2       0    j15
...........agguagauagaacagguc....................................................    170     0    j15
...........agguagauagaacaggucu...................................................    455     0    j15
...........agguagauagaacaggucuu..................................................    548     0    j15
...........agguagauagaacaggucuug.................................................    12759   0    j15
...........agguagauagaacaggucuugu................................................    1432    0    j15
...........agguagauagaacaggucuuguu...............................................    1000    0    j15
...........agguagauagaacaggucuuguuu..............................................    14      0    j15
............gguagauagaacaggucuug.................................................    2       0    j15
.............guagauagaacaggucuug.................................................    1       0    j15
...............................................caagaccuacuuaucuaccaa.............    2       0    j15
...............................................caagaccuacuuaucuaccaac............    1       0    j15
.................................................agaccuacuuaucuaccaaca...........    14      0    j15
.................................................agaccuacuuaucuaccaacag..........    4       0    j15
.................................................agaccuacuuaucuaccaacagca........    1       0    j15
..................................................gaccuacuuaucuaccaaca...........    1       0    j15

.......gaaaagguagauagaacaggucu...................................................    1       0    j17
........aaaagguagauagaacagg......................................................    1       0    j17
........aaaagguagauagaacaggucu...................................................    9       0    j17
........aaaagguagauagaacaggucuu..................................................    10      0    j17
........aaaagguagauagaacaggucuug.................................................    9       0    j17
.........aaagguagauagaacagg......................................................    2       0    j17
.........aaagguagauagaacaggu.....................................................    3       0    j17
.........aaagguagauagaacagguc....................................................    9       0    j17
.........aaagguagauagaacaggucu...................................................    28      0    j17
.........aaagguagauagaacaggucuu..................................................    50      0    j17
.........aaagguagauagaacaggucuug.................................................    344     0    j17
.........aaagguagauagaacaggucuugu................................................    29      0    j17
..........aagguagauagaacaggu.....................................................    104     0    j17
..........aagguagauagaacagguc....................................................    321     0    j17
..........aagguagauagaacaggucu...................................................    1133    0    j17
..........aagguagauagaacaggucuu..................................................    2371    0    j17
..........aagguagauagaacaggucuug.................................................    32057   0    j17
..........aagguagauagaacaggucuugu................................................    4670    0    j17
..........aagguagauagaacaggucuuguu...............................................    54      0    j17
...........agguagauagaacagguc....................................................    185     0    j17
...........agguagauagaacaggucu...................................................    306     0    j17
...........agguagauagaacaggucuu..................................................    749     0    j17
...........agguagauagaacaggucuug.................................................    20342   0    j17
...........agguagauagaacaggucuugu................................................    2829    0    j17
...........agguagauagaacaggucuuguu...............................................    953     0    j17
...........agguagauagaacaggucuuguuu..............................................    33      0    j17
............gguagauagaacaggucuu..................................................    1       0    j17
............gguagauagaacaggucuug.................................................    9       0    j17
.................................................agaccuacuuaucuaccaa.............    2       0    j17
.................................................agaccuacuuaucuaccaaca...........    27      0    j17
.................................................agaccuacuuaucuaccaacag..........    10      0    j17
.................................................agaccuacuuaucuaccaacagc.........    6       0    j17

........aaaagguagauagaacaggucuug.................................................    2       0    la2
.........aaagguagauagaacaggu.....................................................    1       0    la2
.........aaagguagauagaacaggucu...................................................    2       0    la2
.........aaagguagauagaacaggucuu..................................................    2       0    la2
.........aaagguagauagaacaggucuug.................................................    33      0    la2
.........aaagguagauagaacaggucuugu................................................    4       0    la2
..........aagguagauagaacaggu.....................................................    3       0    la2
..........aagguagauagaacagguc....................................................    10      0    la2
..........aagguagauagaacaggucu...................................................    25      0    la2
..........aagguagauagaacaggucuu..................................................    91      0    la2
..........aagguagauagaacaggucuug.................................................    2319    0    la2
..........aagguagauagaacaggucuugu................................................    716     0    la2
```

```
          Mature                              Star
ccuaaaugaaaagguagauagaacaggucuuguuugcaaaauaaaauucaagaccuacuuaucuaccaacagcaauuacacg
..........aagguagauagaacaggucuuguu................................................   9      0   la2
..........aagguagauagaacaggucuuguuu...............................................   1      0   la2
..........aagguagauagaacaggucuuguuug..............................................   3      0   la2
...........agguagauagaacagguc.....................................................   4      0   la2
...........agguagauagaacaggucu....................................................   9      0   la2
...........agguagauagaacaggucuu...................................................   24     0   la2
...........agguagauagaacaggucuug..................................................   647    0   la2
...........agguagauagaacaggucuugu.................................................   168    0   la2
...........agguagauagaacaggucuuguu................................................   121    0   la2
...........agguagauagaacaggucuuguuu...............................................   19     0   la2
................................................caagaccuacuuaucuaccaac............   1      0   la2
..................................................agaccuacuuaucuaccaacagca........   2      0   la2

.........aaagguagauagaacaggucu....................................................   1      0   j08
.........aaagguagauagaacaggucuu...................................................   2      0   j08
.........aaagguagauagaacaggucuug..................................................   2      0   j08
..........aagguagauagaacaggu......................................................   3      0   j08
..........aagguagauagaacagguc.....................................................   16     0   j08
..........aagguagauagaacaggucu....................................................   44     0   j08
..........aagguagauagaacaggucuu...................................................   43     0   j08
..........aagguagauagaacaggucuug..................................................   470    0   j08
..........aagguagauagaacaggucuugu.................................................   89     0   j08
..........aagguagauagaacaggucuuguu................................................   3      0   j08
...........agguagauagaacagguc.....................................................   6      0   j08
...........agguagauagaacaggucu....................................................   9      0   j08
...........agguagauagaacaggucuu...................................................   30     0   j08
...........agguagauagaacaggucuug..................................................   247    0   j08
...........agguagauagaacaggucuugu.................................................   65     0   j08
...........agguagauagaacaggucuuguu................................................   30     0   j08

........aaaagguagauagaacagg.......................................................   1      0   j12
........aaaagguagauagaacaggu......................................................   1      0   j12
........aaaagguagauagaacaggucu....................................................   1      0   j12
........aaaagguagauagaacaggucuu...................................................   2      0   j12
........aaaagguagauagaacaggucuug..................................................   1      0   j12
.........aaagguagauagaacagg.......................................................   1      0   j12
.........aaagguagauagaacaggu......................................................   4      0   j12
.........aaagguagauagaacagguc.....................................................   5      0   j12
.........aaagguagauagaacaggucu....................................................   8      0   j12
.........aaagguagauagaacaggucuu...................................................   15     0   j12
.........aaagguagauagaacaggucuug..................................................   115    0   j12
.........aaagguagauagaacaggucuugu.................................................   2      0   j12
..........aagguagauagaacaggu......................................................   68     0   j12
..........aagguagauagaacagguc.....................................................   195    0   j12
..........aagguagauagaacaggucu....................................................   881    0   j12
..........aagguagauagaacaggucuu...................................................   932    0   j12
..........aagguagauagaacaggucuug..................................................   11720  0   j12
..........aagguagauagaacaggucuugu.................................................   1098   0   j12
..........aagguagauagaacaggucuuguu................................................   31     0   j12
..........aagguagauagaacaggucuuguuu...............................................   1      0   j12
...........agguagauagaacagguc.....................................................   69     0   j12
...........agguagauagaacaggucu....................................................   179    0   j12
...........agguagauagaacaggucuu...................................................   270    0   j12
...........agguagauagaacaggucuug..................................................   6237   0   j12
...........agguagauagaacaggucuugu.................................................   870    0   j12
...........agguagauagaacaggucuuguu................................................   938    0   j12
...........agguagauagaacaggucuuguuu...............................................   20     0   j12
..................................................agaccuacuuaucuacca..............   1      0   j12
..................................................agaccuacuuaucuaccaaca...........   2      0   j12

........aaaagguagauagaacaggucuug..................................................   1      0   li2
.........aaagguagauagaacaggucuu...................................................   2      0   li2
.........aaagguagauagaacaggucuug..................................................   13     0   li2
.........aaagguagauagaacaggucuugu.................................................   3      0   li2
..........aagguagauagaacaggucu....................................................   1      0   li2
..........aagguagauagaacaggucuu...................................................   226    0   li2
..........aagguagauagaacaggucuug..................................................   1400   0   li2
..........aagguagauagaacaggucuugu.................................................   307    0   li2
..........aagguagauagaacaggucuuguu................................................   10     0   li2
...........agguagauagaacaggucuu...................................................   21     0   li2
```

Mature Star

```
ccuaaaugaaaagguagauagaacaggucuuguuugcaaaauaaauucaagaccuacuuaucuaccaacagcaauuacacg
...........agguagauagaacaggucuug.................................................    316     0    li2
...........agguagauagaacaggucuugu................................................    44      0    li2
...........agguagauagaacaggucuuguu...............................................    63      0    li2
...........agguagauagaacaggucuuguuu..............................................    1       0    li2
............gguagauagaacaggucuug.................................................    1       0    li2
.............guagauagaacaggucuug.................................................    1       0    li2
................................................aagaccuacuuaucuaccaaca...........    1       0    li2
.................................................agaccuacuuaucuaccaaca...........    1       0    li2
.................................................agaccuacuuaucuaccaacag..........    8       0    li2
.................................................agaccuacuuaucuaccaacagc.........    10      0    li2

........aaaagguagauagaacaggucu...................................................    2       0    va4
........aaaagguagauagaacaggucuu..................................................    10      0    va4
........aaaagguagauagaacaggucuug.................................................    15      0    va4
........aaaagguagauagaacaggucuugu................................................    3       0    va4
.........aaagguagauagaacagg......................................................    3       0    va4
.........aaagguagauagaacagguc....................................................    6       0    va4
.........aaagguagauagaacaggucu...................................................    13      0    va4
.........aaagguagauagaacaggucuu..................................................    29      0    va4
.........aaagguagauagaacaggucuug.................................................    289     0    va4
.........aaagguagauagaacaggucuugu................................................    33      0    va4
..........aagguagauagaacaggu.....................................................    24      0    va4
..........aagguagauagaacagguc....................................................    122     0    va4
..........aagguagauagaacaggucu...................................................    173     0    va4
..........aagguagauagaacaggucuu..................................................    557     0    va4
..........aagguagauagaacaggucuug.................................................    13363   0    va4
..........aagguagauagaacaggucuugu................................................    3330    0    va4
..........aagguagauagaacaggucuuguu...............................................    57      0    va4
..........aagguagauagaacaggucuuguuu..............................................    5       0    va4
...........agguagauagaacagguc....................................................    33      0    va4
...........agguagauagaacaggucu...................................................    34      0    va4
...........agguagauagaacaggucuu..................................................    117     0    va4
...........agguagauagaacaggucuug.................................................    4504    0    va4
...........agguagauagaacaggucuugu................................................    789     0    va4
...........agguagauagaacaggucuuguu...............................................    212     0    va4
...........agguagauagaacaggucuuguuu..............................................    21      0    va4
............gguagauagaacaggucuug.................................................    2       0    va4
............gguagauagaacaggucuugu................................................    1       0    va4
.................................................agaccuacuuaucuaccaaca...........    4       0    va4
.................................................agaccuacuuaucuaccaacag..........    11      0    va4
.................................................agaccuacuuaucuaccaacagc.........    5       0    va4
.................................................agaccuacuuaucuaccaacagca........    1       0    va4

ccuaaaugaaaagguagauagaacaggu.....................................................    1       0    va3
.cuaaaugaaaagguagauagaaca........................................................    1       0    va3
.cuaaaugaaaagguagauagaacag.......................................................    1       0    va3
.cuaaaugaaaagguagauagaacaggu.....................................................    2       0    va3
...aaaugaaaagguagauagaacaggu.....................................................    1       0    va3
...aaaugaaaagguagauagaacaggucuu..................................................    2       0    va3
....aaugaaaagguagauagaacaggucuu..................................................    1       0    va3
......ugaaaagguagauagaacagguc....................................................    1       0    va3
........aaaagguagauagaacagguc....................................................    1       0    va3
........aaaagguagauagaacaggucu...................................................    1       0    va3
........aaaagguagauagaacaggucuu..................................................    5       0    va3
........aaaagguagauagaacaggucuug.................................................    12      0    va3
........aaaagguagauagaacaggucuugu................................................    2       0    va3
........aaaagguagauagaacaggucuuguuu..............................................    1       0    va3
.........aaagguagauagaacagguc....................................................    1       0    va3
.........aaagguagauagaacaggucu...................................................    2       0    va3
.........aaagguagauagaacaggucuu..................................................    8       0    va3
.........aaagguagauagaacaggucuug.................................................    72      0    va3
.........aaagguagauagaacaggucuugu................................................    7       0    va3
..........aagguagauagaacaggu.....................................................    2       0    va3
..........aagguagauagaacagguc....................................................    34      0    va3
..........aagguagauagaacaggucu...................................................    42      0    va3
..........aagguagauagaacaggucuu..................................................    131     0    va3
..........aagguagauagaacaggucuug.................................................    3139    0    va3
..........aagguagauagaacaggucuugu................................................    552     0    va3
..........aagguagauagaacaggucuuguu...............................................    7       0    va3
..........aagguagauagaacaggucuuguuug.............................................    1       0    va3
```

Mature **Star**

```
ccuaaaugaaaagguagauagaacaggucuuguuugcaaaauaaauucaagaccuacuuaucuaccaacagcaauuacacg
...........agguagauagaacagguc....................................................  7     0  va3
...........agguagauagaacaggucu...................................................  9     0  va3
...........agguagauagaacaggucuu..................................................  24    0  va3
...........agguagauagaacaggucuug.................................................  1044  0  va3
...........agguagauagaacaggucuugu................................................  176   0  va3
...........agguagauagaacaggucuuguu...............................................  177   0  va3
...........agguagauagaacaggucuuguuu..............................................  13    0  va3
............gguagauagaacaggucuug.................................................  1     0  va3
...............................................caagaccuacuuaucuaccaacagca........  1     0  va3
................................................aagaccuacuuaucuaccaacagca........  4     0  va3
.................................................agaccuacuuaucuaccaaca...........  1     0  va3
.................................................agaccuacuuaucuaccaacag..........  2     0  va3
.................................................agaccuacuuaucuaccaacagca........  1     0  va3
..................................................gaccuacuuaucuaccaacagca........  1     0  va3
...................................................accuacuuaucuaccaacagca........  11    0  va3

.........aaagguagauagaacaggucuu..................................................  1     0  SW4
..........aagguagauagaacaggu.....................................................  3     0  SW4
..........aagguagauagaacagguc....................................................  3     0  SW4
..........aagguagauagaacaggucuu..................................................  7     0  SW4
..........aagguagauagaacaggucuug.................................................  78    0  SW4
..........aagguagauagaacaggucuugu................................................  20    0  SW4
...........agguagauagaacaggucu...................................................  2     0  SW4
...........agguagauagaacaggucuu..................................................  3     0  SW4
...........agguagauagaacaggucuug.................................................  30    0  SW4
...........agguagauagaacaggucuugu................................................  7     0  SW4
...........agguagauagaacaggucuuguu...............................................  9     0  SW4
.................................................agaccuacuuaucuaccaac............  1     0  SW4
.................................................agaccuacuuaucuaccaaca...........  4     0  SW4
.................................................agaccuacuuaucuaccaacag..........  4     0  SW4
.................................................agaccuacuuaucuaccaacagc.........  12    0  SW4
..................................................gaccuacuuaucuaccaacagcaa.......  1     0  SW4

.........aaagguagauagaacagg......................................................  1     0  ya4
.........aaagguagauagaacagguc....................................................  1     0  ya4
.........aaagguagauagaacaggucu...................................................  1     0  ya4
.........aaagguagauagaacaggucuu..................................................  6     0  ya4
.........aaagguagauagaacaggucuug.................................................  19    0  ya4
.........aaagguagauagaacaggucuugu................................................  2     0  ya4
..........aagguagauagaacaggu.....................................................  40    0  ya4
..........aagguagauagaacagguc....................................................  160   0  ya4
..........aagguagauagaacaggucu...................................................  211   0  ya4
..........aagguagauagaacaggucuu..................................................  680   0  ya4
..........aagguagauagaacaggucuug.................................................  1218  0  ya4
..........aagguagauagaacaggucuugu................................................  387   0  ya4
..........aagguagauagaacaggucuuguu...............................................  5     0  ya4
..........aagguagauagaacaggucuuguuugc............................................  1     0  ya4
...........agguagauagaacagguc....................................................  82    0  ya4
...........agguagauagaacaggucu...................................................  246   0  ya4
...........agguagauagaacaggucuu..................................................  822   0  ya4
...........agguagauagaacaggucuug.................................................  922   0  ya4
...........agguagauagaacaggucuugu................................................  281   0  ya4
...........agguagauagaacaggucuuguu...............................................  121   0  ya4
...........agguagauagaacaggucuuguuu..............................................  6     0  ya4
............gguagauagaacaggucuu..................................................  2     0  ya4
.................................................agaccuacuuaucuaccaa.............  1     0  ya4
.................................................agaccuacuuaucuaccaaca...........  33    0  ya4
.................................................agaccuacuuaucuaccaacag..........  9     0  ya4
.................................................agaccuacuuaucuaccaacagc.........  5     0  ya4
..................................................gaccuacuuaucuaccaacagc.........  1     0  ya4

........aaaagguagauagaacaggucuug.................................................  1     0  ku1
.........aaagguagauagaacaggu.....................................................  1     0  ku1
.........aaagguagauagaacagguc....................................................  1     0  ku1
.........aaagguagauagaacaggucu...................................................  8     0  ku1
.........aaagguagauagaacaggucuu..................................................  7     0  ku1
.........aaagguagauagaacaggucuug.................................................  41    0  ku1
.........aaagguagauagaacaggucuugu................................................  3     0  ku1
.........aaagguagauagaacaggucuuguu...............................................  1     0  ku1
..........aagguagauagaacaggu.....................................................  6     0  ku1
```

```
          Mature                                 Star
ccuaaaugaaaagguagauagaacaggucuuguuugcaaaauaaauucaagaccuacuuaucuaccaacagcaauuacacg
..........aagguagauagaacagguc....................................................    27      0     ku1
..........aagguagauagaacaggucu...................................................    224     0     ku1
..........aagguagauagaacaggucuu..................................................    310     0     ku1
..........aagguagauagaacaggucuug.................................................    3900    0     ku1
..........aagguagauagaacaggucuugu................................................    903     0     ku1
..........aagguagauagaacaggucuuguu...............................................    22      0     ku1
..........aagguagauagaacaggucuuguuu..............................................    2       0     ku1
...........agguagauagaacagguc....................................................    8       0     ku1
...........agguagauagaacaggucu...................................................    39      0     ku1
...........agguagauagaacaggucuu..................................................    70      0     ku1
...........agguagauagaacaggucuug.................................................    1490    0     ku1
...........agguagauagaacaggucuugu................................................    430     0     ku1
...........agguagauagaacaggucuuguu...............................................    259     0     ku1
...........agguagauagaacaggucuuguuu..............................................    9       0     ku1
............gguagauagaacaggucuuguu...............................................    1       0     ku1
...............................................caagaccuacuuaucuaccaa.............    1       0     ku1
.................................................agaccuacuuaucuaccaa.............    1       0     ku1
.................................................agaccuacuuaucuaccaaca...........    3       0     ku1
.................................................agaccuacuuaucuaccaacag..........    1       0     ku1

........aaaagguagauagaacaggucu...................................................    2       0     j23
........aaaagguagauagaacaggucuu..................................................    2       0     j23
........aaaagguagauagaacaggucuug.................................................    3       0     j23
.........aaagguagauagaacagg......................................................    1       0     j23
.........aaagguagauagaacagguc....................................................    2       0     j23
.........aaagguagauagaacaggucu...................................................    3       0     j23
.........aaagguagauagaacaggucuu..................................................    10      0     j23
.........aaagguagauagaacaggucuug.................................................    97      0     j23
.........aaagguagauagaacaggucuugu................................................    2       0     j23
..........aagguagauagaacaggu.....................................................    25      0     j23
..........aagguagauagaacagguc....................................................    84      0     j23
..........aagguagauagaacaggucu...................................................    431     0     j23
..........aagguagauagaacaggucuu..................................................    762     0     j23
..........aagguagauagaacaggucuug.................................................    6326    0     j23
..........aagguagauagaacaggucuugu................................................    422     0     j23
..........aagguagauagaacaggucuuguu...............................................    7       0     j23
...........agguagauagaacagguc....................................................    39      0     j23
...........agguagauagaacaggucu...................................................    118     0     j23
...........agguagauagaacaggucuu..................................................    254     0     j23
...........agguagauagaacaggucuug.................................................    3737    0     j23
...........agguagauagaacaggucuugu................................................    258     0     j23
...........agguagauagaacaggucuuguu...............................................    360     0     j23
...........agguagauagaacaggucuuguuu..............................................    2       0     j23
............gguagauagaacaggucuug.................................................    1       0     j23
.................................................agaccuacuuaucuaccaaca...........    8       0     j23
.................................................agaccuacuuaucuaccaacag..........    6       0     j23
.................................................agaccuacuuaucuaccaacagca........    1       0     j23

.........aaagguagauagaacaggu.....................................................    1       0     s05
.........aaagguagauagaacaggucu...................................................    11      0     s05
.........aaagguagauagaacaggucuu..................................................    5       0     s05
.........aaagguagauagaacaggucuug.................................................    37      0     s05
.........aaagguagauagaacaggucuugu................................................    1       0     s05
..........aagguagauagaacaggu.....................................................    33      0     s05
..........aagguagauagaacagguc....................................................    88      0     s05
..........aagguagauagaacaggucu...................................................    393     0     s05
..........aagguagauagaacaggucuu..................................................    341     0     s05
..........aagguagauagaacaggucuug.................................................    4423    0     s05
..........aagguagauagaacaggucuugu................................................    399     0     s05
..........aagguagauagaacaggucuuguu...............................................    3       0     s05
...........agguagauagaacagguc....................................................    15      0     s05
...........agguagauagaacaggucu...................................................    67      0     s05
...........agguagauagaacaggucuu..................................................    94      0     s05
...........agguagauagaacaggucuug.................................................    2065    0     s05
...........agguagauagaacaggucuugu................................................    219     0     s05
...........agguagauagaacaggucuuguu...............................................    187     0     s05
...........agguagauagaacaggucuuguuu..............................................    2       0     s05
.................................................agaccuacuuaucuaccaac............    2       0     s05
.................................................agaccuacuuaucuaccaaca...........    18      0     s05
.................................................agaccuacuuaucuaccaacag..........    9       0     s05
```

```
         Mature                                   Star
ccuaaaugaaaagguagauagaacaggucuuguuugcaaaauaaaauucaagaccuacuuaucuaccaacagcaauuacacg
..................................................agaccuacuuaucuaccaacagca........	1	0	s05

........aaaagguagauagaacaggucu....................................................	1	0	ta2
........aaaagguagauagaacaggucuu...................................................	1	0	ta2
........aaaagguagauagaacaggucuug..................................................	3	0	ta2
........aaaagguagauagaacaggucuugu.................................................	1	0	ta2
.........aaagguagauagaacaggucuu...................................................	6	0	ta2
.........aaagguagauagaacaggucuug..................................................	39	0	ta2
.........aaagguagauagaacaggucuugu.................................................	9	0	ta2
..........aagguagauagaacaggu......................................................	8	0	ta2
..........aagguagauagaacagguc.....................................................	29	0	ta2
..........aagguagauagaacaggucu....................................................	73	0	ta2
..........aagguagauagaacaggucuu...................................................	179	0	ta2
..........aagguagauagaacaggucuug..................................................	2057	0	ta2
..........aagguagauagaacaggucuugu.................................................	1000	0	ta2
..........aagguagauagaacaggucuuguu................................................	13	0	ta2
...........agguagauagaacagguc.....................................................	14	0	ta2
...........agguagauagaacaggucu....................................................	20	0	ta2
...........agguagauagaacaggucuu...................................................	70	0	ta2
...........agguagauagaacaggucuug..................................................	882	0	ta2
...........agguagauagaacaggucuugu.................................................	437	0	ta2
...........agguagauagaacaggucuuguu................................................	242	0	ta2
...........agguagauagaacaggucuuguuu...............................................	38	0	ta2
...........agguagauagaacaggucuuguuugc.............................................	2	0	ta2
............gguagauagaacaggucuugu.................................................	2	0	ta2
..................................................agaccuacuuaucuaccaaca...........	1	0	ta2

........aaaagguagauagaacagguc.....................................................	1	0	j24
........aaaagguagauagaacaggucuug..................................................	1	0	j24
.........aaagguagauagaacagg.......................................................	2	0	j24
.........aaagguagauagaacaggu......................................................	2	0	j24
.........aaagguagauagaacagguc.....................................................	4	0	j24
.........aaagguagauagaacaggucu....................................................	26	0	j24
.........aaagguagauagaacaggucuu...................................................	14	0	j24
.........aaagguagauagaacaggucuug..................................................	82	0	j24
.........aaagguagauagaacaggucuugu.................................................	3	0	j24
..........aagguagauagaacaggu......................................................	35	0	j24
..........aagguagauagaacagguc.....................................................	62	0	j24
..........aagguagauagaacaggucu....................................................	729	0	j24
..........aagguagauagaacaggucuu...................................................	444	0	j24
..........aagguagauagaacaggucuug..................................................	5219	0	j24
..........aagguagauagaacaggucuugu.................................................	701	0	j24
..........aagguagauagaacaggucuuguu................................................	3	0	j24
...........agguagauagaacagguc.....................................................	22	0	j24
...........agguagauagaacaggucu....................................................	62	0	j24
...........agguagauagaacaggucuu...................................................	141	0	j24
...........agguagauagaacaggucuug..................................................	1972	0	j24
...........agguagauagaacaggucuugu.................................................	730	0	j24
...........agguagauagaacaggucuuguu................................................	412	0	j24
...........agguagauagaacaggucuuguuu...............................................	17	0	j24
............gguagauagaacaggucuug..................................................	1	0	j24
............gguagauagaacaggucuugu.................................................	1	0	j24
..................................................agaccuacuuaucuaccaaca...........	5	0	j24

........aaaagguagauagaacaggucu....................................................	2	0	k11
........aaaagguagauagaacaggucuu...................................................	3	0	k11
........aaaagguagauagaacaggucuug..................................................	2	0	k11
.........aaagguagauagaacagguc.....................................................	1	0	k11
.........aaagguagauagaacaggucu....................................................	2	0	k11
.........aaagguagauagaacaggucuu...................................................	13	0	k11
.........aaagguagauagaacaggucuug..................................................	87	0	k11
.........aaagguagauagaacaggucuugu.................................................	14	0	k11
.........aaagguagauagaacaggucuuguu................................................	1	0	k11
..........aagguagauagaacaggu......................................................	19	0	k11
..........aagguagauagaacagguc.....................................................	48	0	k11
..........aagguagauagaacaggucu....................................................	126	0	k11
..........aagguagauagaacaggucuu...................................................	569	0	k11
..........aagguagauagaacaggucuug..................................................	7466	0	k11
..........aagguagauagaacaggucuugu.................................................	1174	0	k11
..........aagguagauagaacaggucuuguu................................................	30	0	k11
```

Mature **Star**

```
ccuaaaugaaaagguagauagaacaggucuuguuugcaaaauaaauucaagaccuacuuaucuaccaacagcaauuacacg
..........aagguagauagaacaggucuuguuu..............................................    4       0    k11
...........agguagauagaacagguc....................................................    10      0    k11
...........agguagauagaacaggucu...................................................    21      0    k11
...........agguagauagaacaggucuu..................................................    162     0    k11
...........agguagauagaacaggucuug.................................................    2216    0    k11
...........agguagauagaacaggucuugu................................................    402     0    k11
...........agguagauagaacaggucuuguu...............................................    198     0    k11
...........agguagauagaacaggucuuguuu..............................................    19      0    k11
.................................................agaccuacuuaucuaccaac............    1       0    k11
.................................................agaccuacuuaucuaccaaca...........    2       0    k11
.................................................agaccuacuuaucuaccaacagc.........    1       0    k11

........aaaagguagauagaacaggucuu..................................................    1       0    ta1
........aaaagguagauagaacaggucuug.................................................    2       0    ta1
.........aaagguagauagaacaggu.....................................................    2       0    ta1
.........aaagguagauagaacagguc....................................................    1       0    ta1
.........aaagguagauagaacaggucu...................................................    1       0    ta1
.........aaagguagauagaacaggucuu..................................................    14      0    ta1
.........aaagguagauagaacaggucuug.................................................    45      0    ta1
.........aaagguagauagaacaggucuugu................................................    1       0    ta1
..........aagguagauagaacaggu.....................................................    26      0    ta1
..........aagguagauagaacagguc....................................................    32      0    ta1
..........aagguagauagaacaggucu...................................................    99      0    ta1
..........aagguagauagaacaggucuu..................................................    305     0    ta1
..........aagguagauagaacaggucuug.................................................    3535    0    ta1
..........aagguagauagaacaggucuugu................................................    925     0    ta1
..........aagguagauagaacaggucuuguu...............................................    8       0    ta1
..........aagguagauagaacaggucuuguuu..............................................    1       0    ta1
...........agguagauagaacagguc....................................................    16      0    ta1
...........agguagauagaacaggucu...................................................    32      0    ta1
...........agguagauagaacaggucuu..................................................    120     0    ta1
...........agguagauagaacaggucuug.................................................    1460    0    ta1
...........agguagauagaacaggucuugu................................................    411     0    ta1
...........agguagauagaacaggucuuguu...............................................    170     0    ta1
...........agguagauagaacaggucuuguuu..............................................    7       0    ta1
............gguagauagaacaggucuug.................................................    3       0    ta1
.................................................agaccuacuuaucuaccaaca...........    7       0    ta1
.................................................agaccuacuuaucuaccaacag..........    5       0    ta1
.................................................agaccuacuuaucuaccaacagc.........    1       0    ta1

........aaaagguagauagaacaggucuug.................................................    2       0    s09
.........aaagguagauagaacagg......................................................    1       0    s09
.........aaagguagauagaacaggu.....................................................    1       0    s09
.........aaagguagauagaacaggucu...................................................    3       0    s09
.........aaagguagauagaacaggucuu..................................................    4       0    s09
.........aaagguagauagaacaggucuug.................................................    40      0    s09
..........aagguagauagaacaggu.....................................................    17      0    s09
..........aagguagauagaacagguc....................................................    62      0    s09
..........aagguagauagaacaggucu...................................................    192     0    s09
..........aagguagauagaacaggucuu..................................................    322     0    s09
..........aagguagauagaacaggucuug.................................................    4158    0    s09
..........aagguagauagaacaggucuugu................................................    426     0    s09
..........aagguagauagaacaggucuuguu...............................................    9       0    s09
...........agguagauagaacagguc....................................................    18      0    s09
...........agguagauagaacaggucu...................................................    54      0    s09
...........agguagauagaacaggucuu..................................................    109     0    s09
...........agguagauagaacaggucuug.................................................    1875    0    s09
...........agguagauagaacaggucuugu................................................    356     0    s09
...........agguagauagaacaggucuuguu...............................................    584     0    s09
...........agguagauagaacaggucuuguuu..............................................    11      0    s09
............gguagauagaacaggucuu..................................................    1       0    s09
............gguagauagaacaggucuug.................................................    2       0    s09
............gguagauagaacaggucuuguu...............................................    2       0    s09
...............................................caagaccuacuuaucuaccaa.............    1       0    s09
...............................................caagaccuacuuaucuaccaac............    1       0    s09
................................................aagaccuacuuaucuaccaaca...........    1       0    s09
.................................................agaccuacuuaucuacca..............    1       0    s09
.................................................agaccuacuuaucuaccaa.............    1       0    s09
.................................................agaccuacuuaucuaccaaca...........    25      0    s09
.................................................agaccuacuuaucuaccaacag..........    9       0    s09
```

```
         Mature                                   Star
ccuaaaugaaaagguagauagaacaggucuuguuugcaaaauaaaauucaagaccuacuuaucuaccaacagcaauuacacg
...................................................gaccuacuuaucuaccaac............    1    0    s09

........aaaagguagauagaacagguc.....................................................    1    0    NL2
........aaaagguagauagaacaggucu....................................................    2    0    NL2
.........aaagguagauagaacagg.......................................................    3    0    NL2
.........aaagguagauagaacaggucu....................................................    2    0    NL2
.........aaagguagauagaacaggucuu...................................................    2    0    NL2
.........aaagguagauagaacaggucuug..................................................    7    0    NL2
.........aaagguagauagaacaggucuugu.................................................    5    0    NL2
..........aagguagauagaacaggu......................................................    17    0    NL2
..........aagguagauagaacagguc.....................................................    47    0    NL2
..........aagguagauagaacaggucu....................................................    28    0    NL2
..........aagguagauagaacaggucuu...................................................    131    0    NL2
..........aagguagauagaacaggucuug..................................................    772    0    NL2
..........aagguagauagaacaggucuugu.................................................    552    0    NL2
..........aagguagauagaacaggucuuguu................................................    10    0    NL2
..........aagguagauagaacaggucuuguuu...............................................    3    0    NL2
...........agguagauagaacagguc.....................................................    10    0    NL2
...........agguagauagaacaggucu....................................................    19    0    NL2
...........agguagauagaacaggucuu...................................................    88    0    NL2
...........agguagauagaacaggucuug..................................................    726    0    NL2
...........agguagauagaacaggucuugu.................................................    462    0    NL2
...........agguagauagaacaggucuuguu................................................    166    0    NL2
...........agguagauagaacaggucuuguuu...............................................    31    0    NL2
...........agguagauagaacaggucuuguuug..............................................    2    0    NL2
............gguagauagaacaggucuug..................................................    1    0    NL2
............gguagauagaacaggucuugu.................................................    1    0    NL2
................................................caagaccuacuuaucuaccaa.............    2    0    NL2
.................................................aagaccuacuuaucuaccaaca...........    1    0    NL2
..................................................agaccuacuuaucuaccaa.............    1    0    NL2
..................................................agaccuacuuaucuaccaac............    2    0    NL2
..................................................agaccuacuuaucuaccaaca...........    29    0    NL2
..................................................agaccuacuuaucuaccaacag..........    7    0    NL2
..................................................agaccuacuuaucuaccaacagc.........    3    0    NL2
..................................................agaccuacuuaucuaccaacagca........    1    0    NL2

........aaaagguagauagaacaggu......................................................    3    0    j13
........aaaagguagauagaacagguc.....................................................    1    0    j13
........aaaagguagauagaacaggucu....................................................    1    0    j13
........aaaagguagauagaacaggucuu...................................................    1    0    j13
........aaaagguagauagaacaggucuug..................................................    8    0    j13
........aaaagguagauagaacaggucuugu.................................................    2    0    j13
.........aaagguagauagaacagg.......................................................    1    0    j13
.........aaagguagauagaacaggu......................................................    4    0    j13
.........aaagguagauagaacagguc.....................................................    5    0    j13
.........aaagguagauagaacaggucu....................................................    27    0    j13
.........aaagguagauagaacaggucuu...................................................    15    0    j13
.........aaagguagauagaacaggucuug..................................................    180    0    j13
.........aaagguagauagaacaggucuugu.................................................    3    0    j13
.........aaagguagauagaacaggucuuguu................................................    1    0    j13
..........aagguagauagaacaggu......................................................    101    0    j13
..........aagguagauagaacagguc.....................................................    263    0    j13
..........aagguagauagaacaggucu....................................................    1358    0    j13
..........aagguagauagaacaggucuu...................................................    1250    0    j13
..........aagguagauagaacaggucuug..................................................    13958    0    j13
..........aagguagauagaacaggucuugu.................................................    1324    0    j13
..........aagguagauagaacaggucuuguu................................................    26    0    j13
..........aagguagauagaacaggucuuguuu...............................................    1    0    j13
...........agguagauagaacagguc.....................................................    82    0    j13
...........agguagauagaacaggucu....................................................    270    0    j13
...........agguagauagaacaggucuu...................................................    407    0    j13
...........agguagauagaacaggucuug..................................................    6911    0    j13
...........agguagauagaacaggucuugu.................................................    1145    0    j13
...........agguagauagaacaggucuuguu................................................    1273    0    j13
...........agguagauagaacaggucuuguuu...............................................    27    0    j13
............gguagauagaacaggucuug..................................................    3    0    j13
................................................caagaccuacuuaucuaccaa.............    2    0    j13
..................................................agaccuacuuaucuaccaa.............    2    0    j13
..................................................agaccuacuuaucuaccaac............    1    0    j13
..................................................agaccuacuuaucuaccaaca...........    33    0    j13
```

```
      Mature                Star
ccuaaaugaaaagguagauagaacaggucuuguuugcaaaauaaauucaagaccuacuuaucuaccaacagcaauuacacg
.................................................agaccuacuuaucuaccaacag..........    4      0     j13

........aaaagguagauagaacaggucu...................................................    1      0     k10
........aaaagguagauagaacaggucuu..................................................    4      0     k10
........aaaagguagauagaacaggucuug.................................................    4      0     k10
.........aaagguagauagaacaggu.....................................................    2      0     k10
.........aaagguagauagaacaggucu...................................................    2      0     k10
.........aaagguagauagaacaggucuu..................................................    15     0     k10
.........aaagguagauagaacaggucuug.................................................    99     0     k10
.........aaagguagauagaacaggucuugu................................................    7      0     k10
..........aagguagauagaacaggu.....................................................    26     0     k10
..........aagguagauagaacagguc....................................................    73     0     k10
..........aagguagauagaacaggucu...................................................    163    0     k10
..........aagguagauagaacaggucuu..................................................    730    0     k10
..........aagguagauagaacaggucuug.................................................    9846   0     k10
..........aagguagauagaacaggucuugu................................................    1331   0     k10
..........aagguagauagaacaggucuuguu...............................................    22     0     k10
..........aagguagauagaacaggucuuguuu..............................................    2      0     k10
..........aagguagauagaacaggucuuguuug.............................................    1      0     k10
...........agguagauagaacagguc....................................................    22     0     k10
...........agguagauagaacaggucu...................................................    29     0     k10
...........agguagauagaacaggucuu..................................................    166    0     k10
...........agguagauagaacaggucuug.................................................    2783   0     k10
...........agguagauagaacaggucuugu................................................    402    0     k10
...........agguagauagaacaggucuuguu...............................................    159    0     k10
...........agguagauagaacaggucuuguuu..............................................    13     0     k10
............gguagauagaacaggucuug.................................................    1      0     k10
.............guagauagaacaggucuugu................................................    1      0     k10
.................................................agaccuacuuaucuaccaaca...........    2      0     k10
.................................................agaccuacuuaucuaccaacag..........    2      0     k10

........aaaagguagauagaacaggu.....................................................    2      0     j19
........aaaagguagauagaacagguc....................................................    1      0     j19
........aaaagguagauagaacaggucu...................................................    5      0     j19
........aaaagguagauagaacaggucuu..................................................    4      0     j19
........aaaagguagauagaacaggucuug.................................................    4      0     j19
.........aaagguagauagaacagg......................................................    3      0     j19
.........aaagguagauagaacaggu.....................................................    6      0     j19
.........aaagguagauagaacagguc....................................................    13     0     j19
.........aaagguagauagaacaggucu...................................................    54     0     j19
.........aaagguagauagaacaggucuu..................................................    41     0     j19
.........aaagguagauagaacaggucuug.................................................    282    0     j19
.........aaagguagauagaacaggucuugu................................................    8      0     j19
..........aagguagauagaacaggu.....................................................    124    0     j19
..........aagguagauagaacagguc....................................................    346    0     j19
..........aagguagauagaacaggucu...................................................    2397   0     j19
..........aagguagauagaacaggucuu..................................................    1893   0     j19
..........aagguagauagaacaggucuug.................................................    24425  0     j19
..........aagguagauagaacaggucuugu................................................    1865   0     j19
..........aagguagauagaacaggucuuguu...............................................    28     0     j19
...........agguagauagaacagguc....................................................    112    0     j19
...........agguagauagaacaggucu...................................................    435    0     j19
...........agguagauagaacaggucuu..................................................    574    0     j19
...........agguagauagaacaggucuug.................................................    11096  0     j19
...........agguagauagaacaggucuugu................................................    1758   0     j19
...........agguagauagaacaggucuuguu...............................................    1730   0     j19
...........agguagauagaacaggucuuguuu..............................................    25     0     j19
............gguagauagaacaggucuug.................................................    2      0     j19
............gguagauagaacaggucuuguu...............................................    1      0     j19
..............uagauagaacaggucuug.................................................    1      0     j19
...............................................caagaccuacuuaucuaccaac............    2      0     j19
................................................aagaccuacuuaucuaccaaca...........    2      0     j19
.................................................agaccuacuuaucuaccaaca...........    11     0     j19
.................................................agaccuacuuaucuaccaacag..........    7      0     j19

........aaaagguagauagaacagguc....................................................    1      0     j03
........aaaagguagauagaacaggucu...................................................    1      0     j03
........aaaagguagauagaacaggucuug.................................................    1      0     j03
.........aaagguagauagaacagg......................................................    1      0     j03
.........aaagguagauagaacaggu.....................................................    10     0     j03
```

Mature Star

```
ccuaaaugaaaagguagauagaacaggucuuguuugcaaaauaaauucaagaccuacuuaucuaccaacagcaauuacacg
.........aaagguagauagaacagguc....................................................	9	0	j03
.........aaagguagauagaacaggucu...................................................	14	0	j03
.........aaagguagauagaacaggucuu..................................................	6	0	j03
.........aaagguagauagaacaggucuug.................................................	41	0	j03
.........aaagguagauagaacaggucuugu................................................	1	0	j03
..........aagguagauagaacaggu.....................................................	40	0	j03
..........aagguagauagaacagguc....................................................	134	0	j03
..........aagguagauagaacaggucu...................................................	881	0	j03
..........aagguagauagaacaggucuu..................................................	306	0	j03
..........aagguagauagaacaggucuug.................................................	2962	0	j03
..........aagguagauagaacaggucuugu................................................	227	0	j03
..........aagguagauagaacaggucuuguu...............................................	3	0	j03
...........agguagauagaacagguc....................................................	25	0	j03
...........agguagauagaacaggucu...................................................	171	0	j03
...........agguagauagaacaggucuu..................................................	141	0	j03
...........agguagauagaacaggucuug.................................................	1937	0	j03
...........agguagauagaacaggucuugu................................................	419	0	j03
...........agguagauagaacaggucuuguu...............................................	293	0	j03
...........agguagauagaacaggucuuguuu..............................................	5	0	j03
.................................................agaccuacuuaucuacca..............	1	0	j03
.................................................agaccuacuuaucuaccaaca...........	2	0	j03

........aaaagguagauagaacaggucu...................................................	3	0	j25
........aaaagguagauagaacaggucuu..................................................	1	0	j25
........aaaagguagauagaacaggucuug.................................................	1	0	j25
.........aaagguagauagaacagg......................................................	1	0	j25
.........aaagguagauagaacagguc....................................................	1	0	j25
.........aaagguagauagaacaggucu...................................................	6	0	j25
.........aaagguagauagaacaggucuu..................................................	6	0	j25
.........aaagguagauagaacaggucuug.................................................	23	0	j25
..........aagguagauagaacaggu.....................................................	8	0	j25
..........aagguagauagaacagguc....................................................	18	0	j25
..........aagguagauagaacaggucu...................................................	280	0	j25
..........aagguagauagaacaggucuu..................................................	133	0	j25
..........aagguagauagaacaggucuug.................................................	1324	0	j25
..........aagguagauagaacaggucuugu................................................	204	0	j25
..........aagguagauagaacaggucuuguu...............................................	6	0	j25
...........agguagauagaacagguc....................................................	4	0	j25
...........agguagauagaacaggucu...................................................	34	0	j25
...........agguagauagaacaggucuu..................................................	43	0	j25
...........agguagauagaacaggucuug.................................................	444	0	j25
...........agguagauagaacaggucuugu................................................	151	0	j25
...........agguagauagaacaggucuuguu...............................................	158	0	j25
...........agguagauagaacaggucuuguuu..............................................	5	0	j25
.................................................agaccuacuuaucuaccaaca...........	2	0	j25

........aaaagguagauagaacaggucuu..................................................	4	0	k01
........aaaagguagauagaacaggucuug.................................................	2	0	k01
.........aaagguagauagaacaggu.....................................................	2	0	k01
.........aaagguagauagaacaggucuu..................................................	6	0	k01
.........aaagguagauagaacaggucuug.................................................	46	0	k01
.........aaagguagauagaacaggucuugu................................................	9	0	k01
..........aagguagauagaacaggu.....................................................	6	0	k01
..........aagguagauagaacagguc....................................................	33	0	k01
..........aagguagauagaacaggucu...................................................	54	0	k01
..........aagguagauagaacaggucuu..................................................	276	0	k01
..........aagguagauagaacaggucuug.................................................	3271	0	k01
..........aagguagauagaacaggucuugu................................................	767	0	k01
..........aagguagauagaacaggucuuguu...............................................	18	0	k01
..........aagguagauagaacaggucuuguuu..............................................	1	0	k01
...........agguagauagaacagguc....................................................	7	0	k01
...........agguagauagaacaggucu...................................................	13	0	k01
...........agguagauagaacaggucuu..................................................	65	0	k01
...........agguagauagaacaggucuug.................................................	970	0	k01
...........agguagauagaacaggucuugu................................................	276	0	k01
...........agguagauagaacaggucuuguu...............................................	85	0	k01
...........agguagauagaacaggucuuguuu..............................................	7	0	k01
.................................................agaccuacuuaucuaccaaca...........	1	0	k01

........aaaagguagauagaacaggucuug.................................................	5	0	s01
```

Mature Star

```
ccuaaaugaaaagguagauagaacaggucuuguuugcaaaauaaauucaagaccuacuuaucuaccaacagcaauuacacg
........aaaagguagauagaacaggucuugu................................................    1      0    s01
.........aaagguagauagaacaggucu...................................................    4      0    s01
.........aaagguagauagaacaggucuu..................................................    5      0    s01
.........aaagguagauagaacaggucuug.................................................    61     0    s01
.........aaagguagauagaacaggucuugu................................................    7      0    s01
..........aagguagauagaacaggu.....................................................    12     0    s01
..........aagguagauagaacagguc....................................................    39     0    s01
..........aagguagauagaacaggucu...................................................    42     0    s01
..........aagguagauagaacaggucuu..................................................    151    0    s01
..........aagguagauagaacaggucuug.................................................    3506   0    s01
..........aagguagauagaacaggucuugu................................................    807    0    s01
..........aagguagauagaacaggucuuguu...............................................    6      0    s01
..........aagguagauagaacaggucuuguuu..............................................    1      0    s01
...........agguagauagaacagguc....................................................    17     0    s01
...........agguagauagaacaggucu...................................................    14     0    s01
...........agguagauagaacaggucuu..................................................    41     0    s01
...........agguagauagaacaggucuug.................................................    1817   0    s01
...........agguagauagaacaggucuugu................................................    348    0    s01
...........agguagauagaacaggucuuguu...............................................    397    0    s01
...........agguagauagaacaggucuuguuu..............................................    26     0    s01
............gguagauagaacaggucuug.................................................    1      0    s01
.................................................agaccuacuuaucuaccaaca...........    5      0    s01
.................................................agaccuacuuaucuaccaacag..........    3      0    s01
.................................................agaccuacuuaucuaccaacagc.........    2      0    s01
.................................................agaccuacuuaucuaccaacagca........    1      0    s01

........aaaagguagauagaacagguc....................................................    1      0    per
........aaaagguagauagaacaggucuu..................................................    1      0    per
........aaaagguagauagaacaggucuug.................................................    4      0    per
.........aaagguagauagaacaggu.....................................................    1      0    per
.........aaagguagauagaacagguc....................................................    2      0    per
.........aaagguagauagaacaggucu...................................................    12     0    per
.........aaagguagauagaacaggucuu..................................................    11     0    per
.........aaagguagauagaacaggucuug.................................................    81     0    per
.........aaagguagauagaacaggucuugu................................................    5      0    per
..........aagguagauagaacaggu.....................................................    34     0    per
..........aagguagauagaacagguc....................................................    104    0    per
..........aagguagauagaacaggucu...................................................    470    0    per
..........aagguagauagaacaggucuu..................................................    652    0    per
..........aagguagauagaacaggucuug.................................................    8131   0    per
..........aagguagauagaacaggucuugu................................................    1426   0    per
..........aagguagauagaacaggucuuguu...............................................    28     0    per
..........aagguagauagaacaggucuuguuu..............................................    2      0    per
..........aagguagauagaacaggucuuguuug.............................................    1      0    per
..........aagguagauagaacaggucuuguuugca...........................................    1      0    per
...........agguagauagaacagguc....................................................    76     0    per
...........agguagauagaacaggucu...................................................    141    0    per
...........agguagauagaacaggucuu..................................................    229    0    per
...........agguagauagaacaggucuug.................................................    5390   0    per
...........agguagauagaacaggucuugu................................................    1450   0    per
...........agguagauagaacaggucuuguu...............................................    1478   0    per
...........agguagauagaacaggucuuguuu..............................................    69     0    per
............gguagauagaacaggucuug.................................................    1      0    per
.............guagauagaacaggucuu..................................................    1      0    per
...............................................caagaccuacuuaucuaccaac............    1      0    per
................................................aagaccuacuuaucuaccaaca...........    1      0    per
................................................aagaccuacuuaucuaccaacag..........    1      0    per
.................................................agaccuacuuaucuaccaac............    1      0    per
.................................................agaccuacuuaucuaccaaca...........    34     0    per
.................................................agaccuacuuaucuaccaacag..........    20     0    per
.................................................agaccuacuuaucuaccaacagc.........    3      0    per
.................................................agaccuacuuaucuaccaacagca........    3      0    per
...................................................accuacuuaucuaccaacagc.........    1      0    per

........aaaagguagauagaacaggucu...................................................    6      0    k04
........aaaagguagauagaacaggucuu..................................................    13     0    k04
........aaaagguagauagaacaggucuug.................................................    14     0    k04
........aaaagguagauagaacaggucuugu................................................    1      0    k04
.........aaagguagauagaacagg......................................................    2      0    k04
.........aaagguagauagaacagguc....................................................    1      0    k04
```

Mature **Star**

```
ccuaaaugaaaagguagauagaacaggucuuguuugcaaaauaaauucaagaccuacuuaucuaccaacagcaauuacacg
.........aaagguagauagaacaggucu...................................................   5       0   k04
.........aaagguagauagaacaggucuu..................................................   32      0   k04
.........aaagguagauagaacaggucuug.................................................   162     0   k04
.........aaagguagauagaacaggucuugu................................................   18      0   k04
.........aaagguagauagaacaggucuuguu...............................................   1       0   k04
..........aagguagauagaacaggu.....................................................   31      0   k04
..........aagguagauagaacagguc....................................................   121     0   k04
..........aagguagauagaacaggucu...................................................   308     0   k04
..........aagguagauagaacaggucuu..................................................   1599    0   k04
..........aagguagauagaacaggucuug.................................................   17427   0   k04
..........aagguagauagaacaggucuugu................................................   2685    0   k04
..........aagguagauagaacaggucuuguu...............................................   42      0   k04
..........aagguagauagaacaggucuuguuu..............................................   4       0   k04
...........agguagauagaacagguc....................................................   30      0   k04
...........agguagauagaacaggucu...................................................   89      0   k04
...........agguagauagaacaggucuu..................................................   370     0   k04
...........agguagauagaacaggucuug.................................................   5213    0   k04
...........agguagauagaacaggucuugu................................................   904     0   k04
...........agguagauagaacaggucuuguu...............................................   325     0   k04
...........agguagauagaacaggucuuguuu..............................................   20      0   k04
...........agguagauagaacaggucuuguuugca...........................................   1       0   k04
.................................................agaccuacuuaucuaccaaca...........   8       0   k04
.................................................agaccuacuuaucuaccaacag.........   7       0   k04
.................................................agaccuacuuaucuaccaacagc........   2       0   k04

........aaaagguagauagaacaggucuug.................................................   1       0   ku2
........aaaagguagauagaacaggucuugu................................................   1       0   ku2
.........aaagguagauagaacaggucu...................................................   4       0   ku2
.........aaagguagauagaacaggucuu..................................................   6       0   ku2
.........aaagguagauagaacaggucuug.................................................   46      0   ku2
.........aaagguagauagaacaggucuugu................................................   7       0   ku2
..........aagguagauagaacaggu.....................................................   9       0   ku2
..........aagguagauagaacagguc....................................................   26      0   ku2
..........aagguagauagaacaggucu...................................................   171     0   ku2
..........aagguagauagaacaggucuu..................................................   219     0   ku2
..........aagguagauagaacaggucuug.................................................   3337    0   ku2
..........aagguagauagaacaggucuugu................................................   718     0   ku2
..........aagguagauagaacaggucuuguu...............................................   18      0   ku2
..........aagguagauagaacaggucuuguuu..............................................   1       0   ku2
...........agguagauagaacagguc....................................................   7       0   ku2
...........agguagauagaacaggucu...................................................   21      0   ku2
...........agguagauagaacaggucuu..................................................   49      0   ku2
...........agguagauagaacaggucuug.................................................   1333    0   ku2
...........agguagauagaacaggucuugu................................................   306     0   ku2
...........agguagauagaacaggucuuguu...............................................   236     0   ku2
...........agguagauagaacaggucuuguuu..............................................   2       0   ku2
.................................................agaccuacuuaucuaccaaca...........   7       0   ku2

........aaaagguagauagaacaggucuu..................................................   2       0   U20
.........aaagguagauagaacaggucuug.................................................   4       0   U20
..........aagguagauagaacaggu.....................................................   15      0   U20
..........aagguagauagaacaggucu...................................................   4       0   U20
..........aagguagauagaacaggucuu..................................................   17      0   U20
..........aagguagauagaacaggucuug.................................................   103     0   U20
..........aagguagauagaacaggucuugu................................................   30      0   U20
...........agguagauagaacaggucuu..................................................   10      0   U20
...........agguagauagaacaggucuug.................................................   45      0   U20
...........agguagauagaacaggucuugu................................................   11      0   U20
............gguagauagaacaggucuugu................................................   1       0   U20
..............uagauagaacaggucuug.................................................   3       0   U20
..............uagauagaacaggucuugu................................................   1       0   U20
.................................................agaccuacuuaucuaccaaca...........   2       0   U20
.................................................agaccuacuuaucuaccaacag.........   5       0   U20
.................................................agaccuacuuaucuaccaacagc........   25      0   U20

........aaaagguagauagaacaggucu...................................................   2       0   j21
........aaaagguagauagaacaggucuu..................................................   6       0   j21
........aaaagguagauagaacaggucuug.................................................   38      0   j21
........aaaagguagauagaacaggucuugu................................................   2       0   j21
.........aaagguagauagaacagg......................................................   4       0   j21
```

```
          Mature                    Star
ccuaaaugaaaagguagauagaacaggucuuguuugcaaaauaaauucaagaccuacuuaucuaccaacagcaauuacacg
.........aaagguagauagaacagguc....................................................   7       0   j21
.........aaagguagauagaacaggucu...................................................   5       0   j21
.........aaagguagauagaacaggucuu..................................................   31      0   j21
.........aaagguagauagaacaggucuug.................................................   477     0   j21
.........aaagguagauagaacaggucuugu................................................   13      0   j21
..........aagguagauagaacaggu.....................................................   85      0   j21
..........aagguagauagaacagguc....................................................   306     0   j21
..........aagguagauagaacaggucu...................................................   539     0   j21
..........aagguagauagaacaggucuu..................................................   1737    0   j21
..........aagguagauagaacaggucuug.................................................   38694   0   j21
..........aagguagauagaacaggucuugu................................................   2601    0   j21
..........aagguagauagaacaggucuuguu...............................................   20      0   j21
..........aagguagauagaacaggucuuguuu..............................................   1       0   j21
...........agguagauagaacagguc....................................................   134     0   j21
...........agguagauagaacaggucu...................................................   183     0   j21
...........agguagauagaacaggucuu..................................................   502     0   j21
...........agguagauagaacaggucuug.................................................   18419   0   j21
...........agguagauagaacaggucuugu................................................   918     0   j21
...........agguagauagaacaggucuuguu...............................................   171     0   j21
...........agguagauagaacaggucuuguuu..............................................   7       0   j21
............gguagauagaacaggucuug.................................................   2       0   j21
..............uagauagaacaggucuug.................................................   2       0   j21
.................................................agaccuacuuaucuaccaaca...........   9       0   j21
.................................................agaccuacuuaucuaccaacag..........   19      0   j21
.................................................agaccuacuuaucuaccaacagc.........   4       0   j21
.................................................agaccuacuuaucuaccaacagca........   2       0   j21

........aaaagguagauagaacaggucu...................................................   1       0   s11
........aaaagguagauagaacaggucuu..................................................   3       0   s11
........aaaagguagauagaacaggucuug.................................................   1       0   s11
.........aaagguagauagaacaggu.....................................................   1       0   s11
.........aaagguagauagaacagguc....................................................   1       0   s11
.........aaagguagauagaacaggucuu..................................................   8       0   s11
.........aaagguagauagaacaggucuug.................................................   78      0   s11
.........aaagguagauagaacaggucuugu................................................   2       0   s11
..........aagguagauagaacaggu.....................................................   12      0   s11
..........aagguagauagaacagguc....................................................   44      0   s11
..........aagguagauagaacaggucu...................................................   94      0   s11
..........aagguagauagaacaggucuu..................................................   247     0   s11
..........aagguagauagaacaggucuug.................................................   4789    0   s11
..........aagguagauagaacaggucuugu................................................   945     0   s11
..........aagguagauagaacaggucuuguu...............................................   11      0   s11
...........agguagauagaacagguc....................................................   25      0   s11
...........agguagauagaacaggucu...................................................   18      0   s11
...........agguagauagaacaggucuu..................................................   70      0   s11
...........agguagauagaacaggucuug.................................................   2422    0   s11
...........agguagauagaacaggucuugu................................................   280     0   s11
...........agguagauagaacaggucuuguu...............................................   231     0   s11
...........agguagauagaacaggucuuguuu..............................................   11      0   s11
.............guagauagaacaggucuugu................................................   1       0   s11
..............uagauagaacaggucuuguu...............................................   1       0   s11
...............................................caagaccuacuuaucuaccaaca...........   1       0   s11
.................................................agaccuacuuaucuaccaaca...........   11      0   s11
.................................................agaccuacuuaucuaccaacag..........   8       0   s11
.................................................agaccuacuuaucuaccaacagc.........   3       0   s11

........aaaagguagauagaacaggu.....................................................   3       0   j07
........aaaagguagauagaacaggucuug.................................................   5       0   j07
.........aaagguagauagaacagg......................................................   4       0   j07
.........aaagguagauagaacaggu.....................................................   5       0   j07
.........aaagguagauagaacagguc....................................................   1       0   j07
.........aaagguagauagaacaggucu...................................................   13      0   j07
.........aaagguagauagaacaggucuu..................................................   4       0   j07
.........aaagguagauagaacaggucuug.................................................   60      0   j07
..........aagguagauagaacaggu.....................................................   36      0   j07
..........aagguagauagaacagguc....................................................   104     0   j07
..........aagguagauagaacaggucu...................................................   669     0   j07
..........aagguagauagaacaggucuu..................................................   314     0   j07
..........aagguagauagaacaggucuug.................................................   3091    0   j07
..........aagguagauagaacaggucuugu................................................   320     0   j07
```

Mature **Star**

```
ccuaaaugaaaagguagauagaacaggucuuguuugcaaaauaaaauucaagaccuacuuaucuaccaacagcaauuacacg
..........aagguagauagaacaggucuuguu................................................    3       0    j07
..........aagguagauagaacaggucuuguuug..............................................    3       0    j07
...........agguagauagaacagguc.....................................................    19      0    j07
...........agguagauagaacaggucu....................................................    84      0    j07
...........agguagauagaacaggucuu...................................................    116     0    j07
...........agguagauagaacaggucuug..................................................    1773    0    j07
...........agguagauagaacaggucuugu.................................................    374     0    j07
...........agguagauagaacaggucuuguu................................................    263     0    j07
...........agguagauagaacaggucuuguuu...............................................    3       0    j07
..................................................agaccuacuuaucuaccaaca...........    5       0    j07

........aaaagguagauagaacaggucu....................................................    2       0    j14
........aaaagguagauagaacaggucuu...................................................    7       0    j14
........aaaagguagauagaacaggucuug..................................................    11      0    j14
........aaaagguagauagaacaggucuugu.................................................    1       0    j14
.........aaagguagauagaacaggucu....................................................    6       0    j14
.........aaagguagauagaacaggucuu...................................................    20      0    j14
.........aaagguagauagaacaggucuug..................................................    202     0    j14
.........aaagguagauagaacaggucuugu.................................................    17      0    j14
..........aagguagauagaacaggu......................................................    13      0    j14
..........aagguagauagaacagguc.....................................................    67      0    j14
..........aagguagauagaacaggucu....................................................    139     0    j14
..........aagguagauagaacaggucuu...................................................    421     0    j14
..........aagguagauagaacaggucuug..................................................    10107   0    j14
..........aagguagauagaacaggucuugu.................................................    1960    0    j14
..........aagguagauagaacaggucuuguu................................................    23      0    j14
..........aagguagauagaacaggucuuguuu...............................................    3       0    j14
...........agguagauagaacagguc.....................................................    28      0    j14
...........agguagauagaacaggucu....................................................    41      0    j14
...........agguagauagaacaggucuu...................................................    100     0    j14
...........agguagauagaacaggucuug..................................................    4199    0    j14
...........agguagauagaacaggucuugu.................................................    460     0    j14
...........agguagauagaacaggucuuguu................................................    264     0    j14
...........agguagauagaacaggucuuguuu...............................................    18      0    j14
.................................................aagaccuacuuaucuaccaac............    2       0    j14
..................................................agaccuacuuaucuaccaaca...........    1       0    j14
..................................................agaccuacuuaucuaccaacag..........    3       0    j14

........aaaagguagauagaacaggucu....................................................    2       0    k02
........aaaagguagauagaacaggucuu...................................................    4       0    k02
........aaaagguagauagaacaggucuug..................................................    4       0    k02
........aaaagguagauagaacaggucuugu.................................................    1       0    k02
.........aaagguagauagaacaggucu....................................................    3       0    k02
.........aaagguagauagaacaggucuu...................................................    14      0    k02
.........aaagguagauagaacaggucuug..................................................    71      0    k02
.........aaagguagauagaacaggucuugu.................................................    9       0    k02
..........aagguagauagaacaggu......................................................    32      0    k02
..........aagguagauagaacagguc.....................................................    61      0    k02
..........aagguagauagaacaggucu....................................................    160     0    k02
..........aagguagauagaacaggucuu...................................................    647     0    k02
..........aagguagauagaacaggucuug..................................................    8164    0    k02
..........aagguagauagaacaggucuugu.................................................    1116    0    k02
..........aagguagauagaacaggucuuguu................................................    21      0    k02
...........agguagauagaacagguc.....................................................    29      0    k02
...........agguagauagaacaggucu....................................................    30      0    k02
...........agguagauagaacaggucuu...................................................    159     0    k02
...........agguagauagaacaggucuug..................................................    2243    0    k02
...........agguagauagaacaggucuugu.................................................    371     0    k02
...........agguagauagaacaggucuuguu................................................    139     0    k02
...........agguagauagaacaggucuuguuu...............................................    13      0    k02
..................................................agaccuacuuaucuaccaaca...........    1       0    k02
..................................................agaccuacuuaucuaccaacagc.........    1       0    k02

........aaaagguagauagaacagguc.....................................................    1       0    j28
........aaaagguagauagaacaggucuu...................................................    2       0    j28
........aaaagguagauagaacaggucuug..................................................    2       0    j28
.........aaagguagauagaacaggu......................................................    4       0    j28
.........aaagguagauagaacagguc.....................................................    12      0    j28
.........aaagguagauagaacaggucu....................................................    27      0    j28
.........aaagguagauagaacaggucuu...................................................    21      0    j28
```

```
Mature                         Star
ccuaaaugaaaagguagauagaacaggucuuguuugcaaaauaaauucaagaccuacuuaucuaccaacagcaauuacacg
.........aaagguagauagaacaggucuug.................................................   86     0   j28
.........aaagguagauagaacaggucuugu................................................   11     0   j28
..........aagguagauagaacaggu.....................................................   26     0   j28
..........aagguagauagaacagguc....................................................   95     0   j28
..........aagguagauagaacaggucu...................................................   594    0   j28
..........aagguagauagaacaggucuu..................................................   478    0   j28
..........aagguagauagaacaggucuug.................................................   5694   0   j28
..........aagguagauagaacaggucuugu................................................   1384   0   j28
..........aagguagauagaacaggucuuguu...............................................   20     0   j28
..........aagguagauagaacaggucuuguuu..............................................   2      0   j28
...........agguagauagaacagguc....................................................   19     0   j28
...........agguagauagaacaggucu...................................................   66     0   j28
...........agguagauagaacaggucuu..................................................   94     0   j28
...........agguagauagaacaggucuug.................................................   1844   0   j28
...........agguagauagaacaggucuugu................................................   728    0   j28
...........agguagauagaacaggucuuguu...............................................   660    0   j28
...........agguagauagaacaggucuuguuu..............................................   32     0   j28
................................................aagaccuacuuaucuaccaaca...........   1      0   j28
.................................................agaccuacuuaucuaccaa.............   6      0   j28
.................................................agaccuacuuaucuaccaac............   1      0   j28
.................................................agaccuacuuaucuaccaaca...........   221    0   j28
.................................................agaccuacuuaucuaccaacag..........   75     0   j28
.................................................agaccuacuuaucuaccaacagc.........   14     0   j28
.................................................agaccuacuuaucuaccaacagcaa.......   1      0   j28
....................................................ccuacuuaucuaccaacagca........   2      0   j28
......................................................uacuuaucuaccaacagca........   1      0   j28

........aaaagguagauagaacagguc....................................................   1      0   s08
........aaaagguagauagaacaggucuug.................................................   2      0   s08
.........aaagguagauagaacagg......................................................   1      0   s08
.........aaagguagauagaacaggucuu..................................................   2      0   s08
.........aaagguagauagaacaggucuug.................................................   26     0   s08
.........aaagguagauagaacaggucuugu................................................   2      0   s08
..........aagguagauagaacaggu.....................................................   4      0   s08
..........aagguagauagaacagguc....................................................   28     0   s08
..........aagguagauagaacaggucu...................................................   25     0   s08
..........aagguagauagaacaggucuu..................................................   84     0   s08
..........aagguagauagaacaggucuug.................................................   2428   0   s08
..........aagguagauagaacaggucuugu................................................   185    0   s08
..........aagguagauagaacaggucuuguuu..............................................   1      0   s08
...........agguagauagaacagguc....................................................   10     0   s08
...........agguagauagaacaggucu...................................................   10     0   s08
...........agguagauagaacaggucuu..................................................   24     0   s08
...........agguagauagaacaggucuug.................................................   1073   0   s08
...........agguagauagaacaggucuugu................................................   64     0   s08
...........agguagauagaacaggucuuguu...............................................   39     0   s08
...........agguagauagaacaggucuuguuu..............................................   2      0   s08
...............................................caagaccuacuuaucuaccaac............   2      0   s08
.................................................agaccuacuuaucuaccaaca...........   3      0   s08
.................................................agaccuacuuaucuaccaacag..........   4      0   s08

.........aaagguagauagaacaggucuug.................................................   1      0   DLD
..........aagguagauagaacaggu.....................................................   10     0   DLD
..........aagguagauagaacaggucuu..................................................   10     0   DLD
..........aagguagauagaacaggucuug.................................................   68     0   DLD
..........aagguagauagaacaggucuugu................................................   18     0   DLD
...........agguagauagaacaggucuu..................................................   6      0   DLD
...........agguagauagaacaggucuug.................................................   39     0   DLD
...........agguagauagaacaggucuugu................................................   7      0   DLD
...........agguagauagaacaggucuuguu...............................................   4      0   DLD
...........agguagauagaacaggucuuguuu..............................................   1      0   DLD
............gguagauagaacaggucuugu................................................   1      0   DLD
...............................................caagaccuacuuaucuacca..............   1      0   DLD
................................................aagaccuacuuaucuaccaac............   2      0   DLD
.................................................agaccuacuuaucuaccaaca...........   8      0   DLD
.................................................agaccuacuuaucuaccaacag..........   9      0   DLD
.................................................agaccuacuuaucuaccaacagc.........   10     0   DLD
.................................................agaccuacuuaucuaccaacagca........   1      0   DLD
....................................................ccuacuuaucuaccaacagca........   2      0   DLD

........aaaagguagauagaacaggu.....................................................   1      0   k03
```

```
          Mature                                  Star
ccuaaaugaaaagguagauagaacaggucuuguuugcaaaauaaauucaagaccuacuuaucuaccaacagcaauuacacg
........aaaagguagauagaacaggucuu..................................................    4       0    k03
........aaaagguagauagaacaggucuug.................................................    8       0    k03
........aaaagguagauagaacaggucuugu................................................    1       0    k03
.........aaagguagauagaacaggu.....................................................    1       0    k03
.........aaagguagauagaacaggucu...................................................    3       0    k03
.........aaagguagauagaacaggucuu..................................................    16      0    k03
.........aaagguagauagaacaggucuug.................................................    80      0    k03
.........aaagguagauagaacaggucuugu................................................    9       0    k03
..........aagguagauagaacaggu.....................................................    33      0    k03
..........aagguagauagaacagguc....................................................    77      0    k03
..........aagguagauagaacaggucu...................................................    183     0    k03
..........aagguagauagaacaggucuu..................................................    733     0    k03
..........aagguagauagaacaggucuug.................................................    8422    0    k03
..........aagguagauagaacaggucuugu................................................    1281    0    k03
..........aagguagauagaacaggucuuguu...............................................    25      0    k03
...........agguagauagaacagguc....................................................    20      0    k03
...........agguagauagaacaggucu...................................................    40      0    k03
...........agguagauagaacaggucuu..................................................    192     0    k03
...........agguagauagaacaggucuug.................................................    2675    0    k03
...........agguagauagaacaggucuugu................................................    501     0    k03
...........agguagauagaacaggucuuguu...............................................    240     0    k03
...........agguagauagaacaggucuuguuu..............................................    21      0    k03
.................................................agaccuacuuaucuaccaaca...........    2       0    k03

.........aaagguagauagaacagg......................................................    3       0    ya2
.........aaagguagauagaacagguc....................................................    1       0    ya2
.........aaagguagauagaacaggucu...................................................    3       0    ya2
.........aaagguagauagaacaggucuu..................................................    7       0    ya2
.........aaagguagauagaacaggucuug.................................................    13      0    ya2
.........aaagguagauagaacaggucuugu................................................    1       0    ya2
..........aagguagauagaacaggu.....................................................    49      0    ya2
..........aagguagauagaacagguc....................................................    135     0    ya2
..........aagguagauagaacaggucu...................................................    136     0    ya2
..........aagguagauagaacaggucuu..................................................    592     0    ya2
..........aagguagauagaacaggucuug.................................................    1296    0    ya2
..........aagguagauagaacaggucuugu................................................    445     0    ya2
..........aagguagauagaacaggucuuguu...............................................    11      0    ya2
...........agguagauagaacagguc....................................................    61      0    ya2
...........agguagauagaacaggucu...................................................    169     0    ya2
...........agguagauagaacaggucuu..................................................    625     0    ya2
...........agguagauagaacaggucuug.................................................    1016    0    ya2
...........agguagauagaacaggucuugu................................................    367     0    ya2
...........agguagauagaacaggucuuguu...............................................    188     0    ya2
...........agguagauagaacaggucuuguuu..............................................    4       0    ya2
............gguagauagaacaggucu...................................................    1       0    ya2
............gguagauagaacaggucuu..................................................    3       0    ya2
............gguagauagaacaggucuug.................................................    2       0    ya2
............gguagauagaacaggucuugu................................................    2       0    ya2
.................................................agaccuacuuaucuacca..............    2       0    ya2
.................................................agaccuacuuaucuaccaa.............    3       0    ya2
.................................................agaccuacuuaucuaccaaca...........    31      0    ya2
.................................................agaccuacuuaucuaccaacag..........    6       0    ya2
.................................................agaccuacuuaucuaccaacagc.........    2       0    ya2

........aaaagguagauagaacagg......................................................    1       0    NL3
........aaaagguagauagaacaggucuug.................................................    1       0    NL3
.........aaagguagauagaacaggu.....................................................    1       0    NL3
.........aaagguagauagaacagguc....................................................    1       0    NL3
.........aaagguagauagaacaggucuu..................................................    2       0    NL3
.........aaagguagauagaacaggucuug.................................................    4       0    NL3
.........aaagguagauagaacaggucuugu................................................    2       0    NL3
..........aagguagauagaacaggu.....................................................    9       0    NL3
..........aagguagauagaacagguc....................................................    44      0    NL3
..........aagguagauagaacaggucu...................................................    23      0    NL3
..........aagguagauagaacaggucuu..................................................    118     0    NL3
..........aagguagauagaacaggucuug.................................................    545     0    NL3
..........aagguagauagaacaggucuugu................................................    424     0    NL3
..........aagguagauagaacaggucuuguu...............................................    15      0    NL3
...........agguagauagaacaggucu...................................................    18      0    NL3
...........agguagauagaacaggucuu..................................................    79      0    NL3
```

Mature **Star**

```
ccuaaaugaaaagguagauagaacaggucuuguuugcaaaauaaauucaagaccuacuuaucuaccaacagcaauuacacg
...........agguagauagaacaggucuug.................................................  578    0  NL3
...........agguagauagaacaggucuugu................................................  344    0  NL3
...........agguagauagaacaggucuuguu...............................................  95     0  NL3
...........agguagauagaacaggucuuguuu..............................................  13     0  NL3
.................................................agaccuacuuaucuaccaac............  1      0  NL3
.................................................agaccuacuuaucuaccaaca...........  17     0  NL3
.................................................agaccuacuuaucuaccaacag..........  8      0  NL3
.................................................agaccuacuuaucuaccaacagc.........  1      0  NL3

........aaaagguagauagaacaggucuu..................................................  3      0  s02
........aaaagguagauagaacaggucuug.................................................  2      0  s02
........aaaagguagauagaacaggucuugu................................................  1      0  s02
.........aaagguagauagaacaggucu...................................................  1      0  s02
.........aaagguagauagaacaggucuug.................................................  24     0  s02
.........aaagguagauagaacaggucuugu................................................  4      0  s02
..........aagguagauagaacaggu.....................................................  7      0  s02
..........aagguagauagaacagguc....................................................  24     0  s02
..........aagguagauagaacaggucu...................................................  22     0  s02
..........aagguagauagaacaggucuu..................................................  65     0  s02
..........aagguagauagaacaggucuug.................................................  1403   0  s02
..........aagguagauagaacaggucuugu................................................  381    0  s02
..........aagguagauagaacaggucuuguu...............................................  5      0  s02
...........agguagauagaacagguc....................................................  6      0  s02
...........agguagauagaacaggucu...................................................  6      0  s02
...........agguagauagaacaggucuu..................................................  32     0  s02
...........agguagauagaacaggucuug.................................................  984    0  s02
...........agguagauagaacaggucuugu................................................  127    0  s02
...........agguagauagaacaggucuuguu...............................................  81     0  s02
...........agguagauagaacaggucuuguuu..............................................  7      0  s02
.................................................agaccuacuuaucuaccaacag..........  1      0  s02
.................................................agaccuacuuaucuaccaacagc.........  1      0  s02

........aaaagguagauagaacagguc....................................................  2      0  k06
........aaaagguagauagaacaggucu...................................................  4      0  k06
........aaaagguagauagaacaggucuu..................................................  15     0  k06
........aaaagguagauagaacaggucuug.................................................  13     0  k06
........aaaagguagauagaacaggucuugu................................................  1      0  k06
.........aaagguagauagaacagguc....................................................  4      0  k06
.........aaagguagauagaacaggucu...................................................  11     0  k06
.........aaagguagauagaacaggucuu..................................................  26     0  k06
.........aaagguagauagaacaggucuug.................................................  191    0  k06
.........aaagguagauagaacaggucuugu................................................  14     0  k06
..........aagguagauagaacaggu.....................................................  77     0  k06
..........aagguagauagaacagguc....................................................  137    0  k06
..........aagguagauagaacaggucu...................................................  417    0  k06
..........aagguagauagaacaggucuu..................................................  1412   0  k06
..........aagguagauagaacaggucuug.................................................  16817  0  k06
..........aagguagauagaacaggucuugu................................................  2072   0  k06
..........aagguagauagaacaggucuuguu...............................................  36     0  k06
..........aagguagauagaacaggucuuguuu..............................................  1      0  k06
...........agguagauagaacagguc....................................................  56     0  k06
...........agguagauagaacaggucu...................................................  95     0  k06
...........agguagauagaacaggucuu..................................................  391    0  k06
...........agguagauagaacaggucuug.................................................  5328   0  k06
...........agguagauagaacaggucuugu................................................  854    0  k06
...........agguagauagaacaggucuuguu...............................................  382    0  k06
...........agguagauagaacaggucuuguuu..............................................  31     0  k06
...........agguagauagaacaggucuuguuug.............................................  1      0  k06
.................................................agaccuacuuaucuaccaaca...........  4      0  k06
.................................................agaccuacuuaucuaccaacag..........  2      0  k06
.................................................agaccuacuuaucuaccaacagc.........  1      0  k06

........aaaagguagauagaacaggucu...................................................  1      0  sh2
........aaaagguagauagaacaggucuu..................................................  2      0  sh2
.........aaagguagauagaacagg......................................................  2      0  sh2
.........aaagguagauagaacagguc....................................................  2      0  sh2
.........aaagguagauagaacaggucu...................................................  2      0  sh2
.........aaagguagauagaacaggucuu..................................................  8      0  sh2
.........aaagguagauagaacaggucuug.................................................  10     0  sh2
.........aaagguagauagaacaggucuugu................................................  5      0  sh2
```

```
          Mature                                      Star
ccuaaaugaaaagguagauagaacaggucuuguuugcaaaauaaaauucaagaccuacuuaucuaccaacagcaauuacacg
.........aaagguagauagaacaggucuuguu................................................    1      0     sh2
..........aagguagauagaacaggu......................................................    151    0     sh2
..........aagguagauagaacagguc.....................................................    16     0     sh2
..........aagguagauagaacaggucu....................................................    82     0     sh2
..........aagguagauagaacaggucuu...................................................    240    0     sh2
..........aagguagauagaacaggucuug..................................................    871    0     sh2
..........aagguagauagaacaggucuugu.................................................    423    0     sh2
..........aagguagauagaacaggucuuguu................................................    11     0     sh2
...........agguagauagaacagguc.....................................................    14     0     sh2
...........agguagauagaacaggucu....................................................    15     0     sh2
...........agguagauagaacaggucuu...................................................    57     0     sh2
...........agguagauagaacaggucuug..................................................    248    0     sh2
...........agguagauagaacaggucuugu.................................................    225    0     sh2
...........agguagauagaacaggucuuguu................................................    118    0     sh2
...........agguagauagaacaggucuuguuu...............................................    16     0     sh2
...........agguagauagaacaggucuuguuug..............................................    2      0     sh2
............gguagauagaacaggucuug..................................................    2      0     sh2
............gguagauagaacaggucuugu.................................................    1      0     sh2
..............uagauagaacaggucuug..................................................    1      0     sh2
..............uagauagaacaggucuugu.................................................    1      0     sh2
................................................caagaccuacuuaucuacca..............    1      0     sh2
................................................caagaccuacuuaucuaccaa.............    1      0     sh2
................................................caagaccuacuuaucuaccaac............    2      0     sh2
.................................................aagaccuacuuaucuaccaaca...........    2      0     sh2
..................................................agaccuacuuaucuaccaac............    6      0     sh2
..................................................agaccuacuuaucuaccaaca...........    33     0     sh2
..................................................agaccuacuuaucuaccaacag..........    23     0     sh2
..................................................agaccuacuuaucuaccaacagc.........    24     0     sh2
.......................................................uacuuaucuaccaacagca........    1      0     sh2

........aaaagguagauagaacaggucuu...................................................    2      0     j29
.........aaagguagauagaacaggucu....................................................    3      0     j29
.........aaagguagauagaacaggucuu...................................................    4      0     j29
.........aaagguagauagaacaggucuug..................................................    46     0     j29
.........aaagguagauagaacaggucuugu.................................................    7      0     j29
..........aagguagauagaacaggu......................................................    13     0     j29
..........aagguagauagaacagguc.....................................................    44     0     j29
..........aagguagauagaacaggucu....................................................    91     0     j29
..........aagguagauagaacaggucuu...................................................    170    0     j29
..........aagguagauagaacaggucuug..................................................    2696   0     j29
..........aagguagauagaacaggucuugu.................................................    895    0     j29
..........aagguagauagaacaggucuuguu................................................    13     0     j29
...........agguagauagaacagguc.....................................................    12     0     j29
...........agguagauagaacaggucu....................................................    17     0     j29
...........agguagauagaacaggucuu...................................................    43     0     j29
...........agguagauagaacaggucuug..................................................    1112   0     j29
...........agguagauagaacaggucuugu.................................................    283    0     j29
...........agguagauagaacaggucuuguu................................................    107    0     j29
...........agguagauagaacaggucuuguuu...............................................    13     0     j29
..................................................agaccuacuuaucuaccaaca...........    3      0     j29
..................................................agaccuacuuaucuaccaacag..........    1      0     j29

.........aaagguagauagaacaggu......................................................    2      0     MCF
.........aaagguagauagaacaggucu....................................................    5      0     MCF
.........aaagguagauagaacaggucuu...................................................    5      0     MCF
.........aaagguagauagaacaggucuug..................................................    7      0     MCF
..........aagguagauagaacaggu......................................................    63     0     MCF
..........aagguagauagaacagguc.....................................................    9      0     MCF
..........aagguagauagaacaggucu....................................................    54     0     MCF
..........aagguagauagaacaggucuu...................................................    167    0     MCF
..........aagguagauagaacaggucuug..................................................    468    0     MCF
..........aagguagauagaacaggucuugu.................................................    97     0     MCF
..........aagguagauagaacaggucuuguu................................................    3      0     MCF
...........agguagauagaacagguc.....................................................    3      0     MCF
...........agguagauagaacaggucu....................................................    11     0     MCF
...........agguagauagaacaggucuu...................................................    86     0     MCF
...........agguagauagaacaggucuug..................................................    202    0     MCF
...........agguagauagaacaggucuugu.................................................    64     0     MCF
...........agguagauagaacaggucuuguu................................................    66     0     MCF
...........agguagauagaacaggucuuguuu...............................................    4      0     MCF
```

Mature Star

ccuaaaugaaaagguagauagaacaggucuuguuugcaaaauaaauucaagaccuacuuaucuaccaacagcaauuacacg			
...........agguagauagaacaggucuuguuug...	1	0	MCF
............gguagauagaacaggucuug...	1	0	MCF
............gguagauagaacaggucuugu..	2	0	MCF
...............agauagaacaggucuugu..	1	0	MCF
....caagaccuacuuaucuaccaa.............	4	0	MCF
....caagaccuacuuaucuaccaac............	1	0	MCF
....caagaccuacuuaucuaccaaca...........	1	0	MCF
....agaccuacuuaucuaccaa.............	1	0	MCF
....agaccuacuuaucuaccaac............	28	0	MCF
....agaccuacuuaucuaccaaca...........	36	0	MCF
....agaccuacuuaucuaccaacag..........	122	0	MCF
....agaccuacuuaucuaccaacagc.........	69	0	MCF
....gaccuacuuaucuaccaaca...........	2	0	MCF
....gaccuacuuaucuaccaacag..........	4	0	MCF
....gaccuacuuaucuaccaacagc.........	2	0	MCF
....accuacuuaucuaccaacag..........	2	0	MCF
....ccuacuuaucuaccaaca...........	1	0	MCF
....ccuacuuaucuaccaacag..........	1	0	MCF
....ccuacuuaucuaccaacagc.........	2	0	MCF
....ccuacuuaucuaccaacagca........	1	0	MCF
........aaaagguagauagaacaggu...	1	0	j01
........aaaagguagauagaacagguc..	2	0	j01
.........aaagguagauagaacagg..	2	0	j01
.........aaagguagauagaacagguc..	1	0	j01
.........aaagguagauagaacaggucu...	12	0	j01
.........aaagguagauagaacaggucuu..	4	0	j01
.........aaagguagauagaacaggucuug...	33	0	j01
.........aaagguagauagaacaggucuugu..	2	0	j01
..........aagguagauagaacaggu...	13	0	j01
..........aagguagauagaacagguc..	49	0	j01
..........aagguagauagaacaggucu...	370	0	j01
..........aagguagauagaacaggucuu..	268	0	j01
..........aagguagauagaacaggucuug...	2549	0	j01
..........aagguagauagaacaggucuugu..	525	0	j01
..........aagguagauagaacaggucuuguu...	7	0	j01
...........agguagauagaacagguc..	12	0	j01
...........agguagauagaacaggucu...	79	0	j01
...........agguagauagaacaggucuu..	75	0	j01
...........agguagauagaacaggucuug...	1176	0	j01
...........agguagauagaacaggucuugu..	484	0	j01
...........agguagauagaacaggucuuguu...	224	0	j01
...........agguagauagaacaggucuuguuu..	5	0	j01
....agaccuacuuaucuaccaaca...........	1	0	j01
.........aaagguagauagaacaggucuug...	4	0	NL1
.........aaagguagauagaacaggucuugu..	1	0	NL1
..........aagguagauagaacaggu...	1	0	NL1
..........aagguagauagaacagguc..	2	0	NL1
..........aagguagauagaacaggucu...	6	0	NL1
..........aagguagauagaacaggucuu..	11	0	NL1
..........aagguagauagaacaggucuug...	173	0	NL1
..........aagguagauagaacaggucuugu..	46	0	NL1
...........agguagauagaacagguc..	1	0	NL1
...........agguagauagaacaggucu...	4	0	NL1
...........agguagauagaacaggucuu..	21	0	NL1
...........agguagauagaacaggucuug...	158	0	NL1
...........agguagauagaacaggucuugu..	43	0	NL1
...........agguagauagaacaggucuuguu...	9	0	NL1
...........agguagauagaacaggucuuguuu..	7	0	NL1
....agaccuacuuaucuaccaaca...........	4	0	NL1
.........aaagguagauagaacaggucuu..	2	0	MBM
.........aaagguagauagaacaggucuug...	5	0	MBM
..........aagguagauagaacaggu...	12	0	MBM
..........aagguagauagaacagguc..	2	0	MBM
..........aagguagauagaacaggucu...	5	0	MBM
..........aagguagauagaacaggucuu..	29	0	MBM
..........aagguagauagaacaggucuug...	212	0	MBM
..........aagguagauagaacaggucuugu..	72	0	MBM

Mature **Star**

```
ccuaaaugaaaagguagauagaacaggucuuguuugcaaaauaaauucaagaccuacuuaucuaccaacagcaauuacacg
..........aagguagauagaacaggucuuguu...............................................   2      0      MBM
...........agguagauagaacaggucu...................................................   2      0      MBM
...........agguagauagaacaggucuu..................................................   11     0      MBM
...........agguagauagaacaggucuug.................................................   72     0      MBM
...........agguagauagaacaggucuugu................................................   45     0      MBM
...........agguagauagaacaggucuuguu...............................................   65     0      MBM
...........agguagauagaacaggucuuguuu..............................................   12     0      MBM
............gguagauagaacaggucuug.................................................   2      0      MBM
............gguagauagaacaggucuugu................................................   2      0      MBM
............gguagauagaacaggucuuguu...............................................   1      0      MBM
..............uagauagaacaggucuuguuu..............................................   2      0      MBM
...............agauagaacaggucuugu................................................   1      0      MBM
................gauagaacaggucuuguu...............................................   3      0      MBM
............................................auucaagaccuacuuaucuaccaacagca........   2      0      MBM
...............................................caagaccuacuuaucuaccaa.............   2      0      MBM
...............................................caagaccuacuuaucuaccaac............   4      0      MBM
.................................................agaccuacuuaucuaccaac............   9      0      MBM
.................................................agaccuacuuaucuaccaaca...........   13     0      MBM
.................................................agaccuacuuaucuaccaacag..........   11     0      MBM
.................................................agaccuacuuaucuaccaacagc.........   12     0      MBM
.................................................agaccuacuuaucuaccaacagca........   1      0      MBM
...................................................gaccuacuuaucuaccaacagc.........   2      0      MBM
...................................................gaccuacuuaucuaccaacagca........   1      0      MBM
.....................................................ccuacuuaucuaccaacag..........   1      0      MBM
.....................................................ccuacuuaucuaccaacagc.........   1      0      MBM
.....................................................ccuacuuaucuaccaacagca........   2      0      MBM

........aaaagguagauagaacaggucu...................................................   2      0      k07
........aaaagguagauagaacaggucuu..................................................   5      0      k07
........aaaagguagauagaacaggucuug.................................................   4      0      k07
........aaaagguagauagaacaggucuugu................................................   1      0      k07
.........aaagguagauagaacaggu.....................................................   1      0      k07
.........aaagguagauagaacagguc....................................................   2      0      k07
.........aaagguagauagaacaggucu...................................................   8      0      k07
.........aaagguagauagaacaggucuu..................................................   24     0      k07
.........aaagguagauagaacaggucuug.................................................   105    0      k07
.........aaagguagauagaacaggucuugu................................................   6      0      k07
..........aagguagauagaacaggu.....................................................   42     0      k07
..........aagguagauagaacagguc....................................................   69     0      k07
..........aagguagauagaacaggucu...................................................   183    0      k07
..........aagguagauagaacaggucuu..................................................   661    0      k07
..........aagguagauagaacaggucuug.................................................   7910   0      k07
..........aagguagauagaacaggucuugu................................................   1152   0      k07
..........aagguagauagaacaggucuuguu...............................................   14     0      k07
..........aagguagauagaacaggucuuguuu..............................................   2      0      k07
...........agguagauagaacagguc....................................................   22     0      k07
...........agguagauagaacaggucu...................................................   55     0      k07
...........agguagauagaacaggucuu..................................................   223    0      k07
...........agguagauagaacaggucuug.................................................   3261   0      k07
...........agguagauagaacaggucuugu................................................   725    0      k07
...........agguagauagaacaggucuuguu...............................................   471    0      k07
...........agguagauagaacaggucuuguuu..............................................   25     0      k07
...........agguagauagaacaggucuuguuug.............................................   1      0      k07
............gguagauagaacaggucuug.................................................   1      0      k07
.................................................agaccuacuuaucuaccaac............   1      0      k07
.................................................agaccuacuuaucuaccaaca...........   6      0      k07

........aaaagguagauagaacaggucuu..................................................   2      0      sh1
........aaaagguagauagaacaggucuug.................................................   2      0      sh1
.........aaagguagauagaacagguc....................................................   3      0      sh1
.........aaagguagauagaacaggucuug.................................................   33     0      sh1
.........aaagguagauagaacaggucuugu................................................   10     0      sh1
..........aagguagauagaacaggu.....................................................   5      0      sh1
..........aagguagauagaacagguc....................................................   38     0      sh1
..........aagguagauagaacaggucu...................................................   77     0      sh1
..........aagguagauagaacaggucuu..................................................   157    0      sh1
..........aagguagauagaacaggucuug.................................................   1629   0      sh1
..........aagguagauagaacaggucuugu................................................   679    0      sh1
..........aagguagauagaacaggucuuguu...............................................   11     0      sh1
...........agguagauagaacagguc....................................................   8      0      sh1
```

```
          Mature                                 Star
ccuaaaugaaaagguagauagaacaggucuuguuugcaaaauaaauucaagaccuacuuaucuaccaacagcaauuacacg
...........agguagauagaacaggucu...................................................   15      0   sh1
...........agguagauagaacaggucuu..................................................   72      0   sh1
...........agguagauagaacaggucuug.................................................   768     0   sh1
...........agguagauagaacaggucuugu................................................   149     0   sh1
...........agguagauagaacaggucuuguu...............................................   88      0   sh1
...........agguagauagaacaggucuuguuu..............................................   15      0   sh1
............gguagauagaacaggucuug.................................................   11      0   sh1
............gguagauagaacaggucuuguu...............................................   1       0   sh1
.................................................agaccuacuuaucuaccaaca...........   1       0   sh1

.........aaagguagauagaacaggucuu..................................................   1       0   li1
.........aaagguagauagaacaggucuug.................................................   3       0   li1
..........aagguagauagaacagguc....................................................   1       0   li1
..........aagguagauagaacaggucu...................................................   1       0   li1
..........aagguagauagaacaggucuu..................................................   26      0   li1
..........aagguagauagaacaggucuug.................................................   475     0   li1
..........aagguagauagaacaggucuugu................................................   53      0   li1
...........agguagauagaacaggucu...................................................   3       0   li1
...........agguagauagaacaggucuu..................................................   9       0   li1
...........agguagauagaacaggucuug.................................................   106     0   li1
...........agguagauagaacaggucuugu................................................   9       0   li1
...........agguagauagaacaggucuuguu...............................................   11      0   li1
.................................................agaccuacuuaucuaccaacag..........   18      0   li1
.................................................agaccuacuuaucuaccaacagc.........   31      0   li1
.................................................agaccuacuuaucuaccaacagca........   1       0   li1

........aaaagguagauagaacagg......................................................   1       0   k13
........aaaagguagauagaacaggucu...................................................   5       0   k13
........aaaagguagauagaacaggucuu..................................................   24      0   k13
........aaaagguagauagaacaggucuug.................................................   3       0   k13
.........aaagguagauagaacaggu.....................................................   1       0   k13
.........aaagguagauagaacagguc....................................................   1       0   k13
.........aaagguagauagaacaggucu...................................................   6       0   k13
.........aaagguagauagaacaggucuu..................................................   33      0   k13
.........aaagguagauagaacaggucuug.................................................   177     0   k13
.........aaagguagauagaacaggucuugu................................................   4       0   k13
..........aagguagauagaacaggu.....................................................   50      0   k13
..........aagguagauagaacagguc....................................................   133     0   k13
..........aagguagauagaacaggucu...................................................   297     0   k13
..........aagguagauagaacaggucuu..................................................   1300    0   k13
..........aagguagauagaacaggucuug.................................................   19067   0   k13
..........aagguagauagaacaggucuugu................................................   1713    0   k13
..........aagguagauagaacaggucuuguu...............................................   9       0   k13
...........agguagauagaacagguc....................................................   51      0   k13
...........agguagauagaacaggucu...................................................   106     0   k13
...........agguagauagaacaggucuu..................................................   419     0   k13
...........agguagauagaacaggucuug.................................................   7010    0   k13
...........agguagauagaacaggucuugu................................................   1103    0   k13
...........agguagauagaacaggucuuguu...............................................   558     0   k13
...........agguagauagaacaggucuuguuu..............................................   20      0   k13
............gguagauagaacaggucuug.................................................   1       0   k13
...............................................caagaccuacuuaucuaccaa.............   1       0   k13
.................................................agaccuacuuaucuaccaac............   1       0   k13
.................................................agaccuacuuaucuaccaaca...........   6       0   k13
.................................................agaccuacuuaucuaccaacag..........   5       0   k13
.................................................agaccuacuuaucuaccaacagc.........   2       0   k13

........aaaagguagauagaacag.......................................................   1       0   j20
........aaaagguagauagaacaggu.....................................................   1       0   j20
........aaaagguagauagaacaggucu...................................................   2       0   j20
........aaaagguagauagaacaggucuu..................................................   4       0   j20
........aaaagguagauagaacaggucuug.................................................   4       0   j20
.........aaagguagauagaacagg......................................................   2       0   j20
.........aaagguagauagaacaggu.....................................................   2       0   j20
.........aaagguagauagaacagguc....................................................   3       0   j20
.........aaagguagauagaacaggucu...................................................   8       0   j20
.........aaagguagauagaacaggucuu..................................................   21      0   j20
.........aaagguagauagaacaggucuug.................................................   146     0   j20
.........aaagguagauagaacaggucuugu................................................   1       0   j20
..........aagguagauagaacaggu.....................................................   65      0   j20
```

Mature **Star**

```
ccuaaaugaaaagguagauagaacaggucuuguuugcaaaauaaauucaagaccuacuuaucuaccaacagcaauuacacg
..........aagguagauagaacagguc....................................................  166    0  j20
..........aagguagauagaacaggucu...................................................  763    0  j20
..........aagguagauagaacaggucuu..................................................  1132   0  j20
..........aagguagauagaacaggucuug.................................................  14773  0  j20
..........aagguagauagaacaggucuugu................................................  1215   0  j20
..........aagguagauagaacaggucuuguu...............................................  13     0  j20
..........aagguagauagaacaggucuuguuu..............................................  2      0  j20
...........agguagauagaacagguc....................................................  70     0  j20
...........agguagauagaacaggucu...................................................  176    0  j20
...........agguagauagaacaggucuu..................................................  336    0  j20
...........agguagauagaacaggucuug.................................................  7390   0  j20
...........agguagauagaacaggucuugu................................................  848    0  j20
...........agguagauagaacaggucuuguu...............................................  670    0  j20
...........agguagauagaacaggucuuguuu..............................................  12     0  j20
............gguagauagaacaggucuug.................................................  1      0  j20
.................................................agaccuacuuaucuaccaa.............  1      0  j20
.................................................agaccuacuuaucuaccaac............  1      0  j20
.................................................agaccuacuuaucuaccaaca...........  11     0  j20
.................................................agaccuacuuaucuaccaacag..........  7      0  j20

.........aaagguagauagaacagg......................................................  2      0  A54
..........aagguagauagaacaggu.....................................................  19     0  A54
..........aagguagauagaacaggucu...................................................  9      0  A54
..........aagguagauagaacaggucuu..................................................  55     0  A54
..........aagguagauagaacaggucuug.................................................  153    0  A54
..........aagguagauagaacaggucuugu................................................  37     0  A54
...........agguagauagaacaggucuu..................................................  15     0  A54
...........agguagauagaacaggucuug.................................................  72     0  A54
...........agguagauagaacaggucuugu................................................  21     0  A54
...........agguagauagaacaggucuuguu...............................................  14     0  A54
.............................................uucaagaccuacuuaucuaccaacagca........  3      0  A54
...............................................caagaccuacuuaucuaccaa.............  4      0  A54
.................................................agaccuacuuaucuaccaac............  6      0  A54
.................................................agaccuacuuaucuaccaaca...........  24     0  A54
.................................................agaccuacuuaucuaccaacag..........  37     0  A54
.................................................agaccuacuuaucuaccaacagc.........  14     0  A54
..................................................gaccuacuuaucuaccaaca...........  10     0  A54
...................................................accuacuuaucuaccaacagc.........  3      0  A54
...................................................accuacuuaucuaccaacagca........  12     0  A54
....................................................ccuacuuaucuaccaacagca........  8      0  A54
.....................................................cuacuuaucuaccaacagca........  3      0  A54

........aaaagguagauagaacaggucuug.................................................  2      0  j30
.........aaagguagauagaacaggu.....................................................  1      0  j30
.........aaagguagauagaacagguc....................................................  1      0  j30
.........aaagguagauagaacaggucu...................................................  1      0  j30
.........aaagguagauagaacaggucuu..................................................  6      0  j30
.........aaagguagauagaacaggucuug.................................................  45     0  j30
.........aaagguagauagaacaggucuugu................................................  1      0  j30
..........aagguagauagaacaggu.....................................................  12     0  j30
..........aagguagauagaacagguc....................................................  36     0  j30
..........aagguagauagaacaggucu...................................................  126    0  j30
..........aagguagauagaacaggucuu..................................................  248    0  j30
..........aagguagauagaacaggucuug.................................................  4066   0  j30
..........aagguagauagaacaggucuugu................................................  270    0  j30
..........aagguagauagaacaggucuuguu...............................................  3      0  j30
...........agguagauagaacagguc....................................................  15     0  j30
...........agguagauagaacaggucu...................................................  41     0  j30
...........agguagauagaacaggucuu..................................................  99     0  j30
...........agguagauagaacaggucuug.................................................  2863   0  j30
...........agguagauagaacaggucuugu................................................  235    0  j30
...........agguagauagaacaggucuuguu...............................................  158    0  j30
...........agguagauagaacaggucuuguuu..............................................  4      0  j30
............gguagauagaacaggucuug.................................................  1      0  j30
.................................................agaccuacuuaucuaccaaca...........  5      0  j30
.................................................agaccuacuuaucuaccaacag..........  1      0  j30

........aaaagguagauagaacaggu.....................................................  1      0  k12
........aaaagguagauagaacagguc....................................................  2      0  k12
........aaaagguagauagaacaggucu...................................................  5      0  k12
```

```
          Mature                                  Star
ccuaaaugaaaagguagauagaacaggucuuguuugcaaaauaaauucaagaccuacuuaucuaccaacagcaauuacacg
........aaaagguagauagaacaggucuu..................................................   5      0   k12
........aaaagguagauagaacaggucuug.................................................   3      0   k12
.........aaagguagauagaacaggucu...................................................   5      0   k12
.........aaagguagauagaacaggucuu..................................................   18     0   k12
.........aaagguagauagaacaggucuug.................................................   47     0   k12
.........aaagguagauagaacaggucuugu................................................   3      0   k12
..........aagguagauagaacaggu.....................................................   64     0   k12
..........aagguagauagaacagguc....................................................   62     0   k12
..........aagguagauagaacaggucu...................................................   435    0   k12
..........aagguagauagaacaggucuu..................................................   1229   0   k12
..........aagguagauagaacaggucuug.................................................   6624   0   k12
..........aagguagauagaacaggucuugu................................................   386    0   k12
..........aagguagauagaacaggucuuguu...............................................   4      0   k12
..........aagguagauagaacaggucuuguuug.............................................   1      0   k12
...........agguagauagaacagguc....................................................   33     0   k12
...........agguagauagaacaggucu...................................................   145    0   k12
...........agguagauagaacaggucuu..................................................   662    0   k12
...........agguagauagaacaggucuug.................................................   4812   0   k12
...........agguagauagaacaggucuugu................................................   474    0   k12
...........agguagauagaacaggucuuguu...............................................   160    0   k12
...........agguagauagaacaggucuuguuu..............................................   6      0   k12
............gguagauagaacaggucuugu................................................   1      0   k12
.........................................uaaauucaagaccuacuuaucu..................   1      0   k12
...............................................caagaccuacuuaucuaccaa.............   1      0   k12
................................................aagaccuacuuaucuaccaaca...........   2      0   k12
................................................aagaccuacuuaucuaccaacagc.........   1      0   k12
.................................................agaccuacuuaucuaccaac............   1      0   k12
.................................................agaccuacuuaucuaccaaca...........   12     0   k12
.................................................agaccuacuuaucuaccaacag..........   5      0   k12
```

Provisional ID	:	hsa_can_1943
Score total	:	55
Score for star read(s)	:	3.9
Score for read counts	:	50.1
Score for mfe	:	1.6
Score for randfold	:	
Score for cons. seed	:	-0.6
Total read count	:	110
Mature read count	:	102
Loop read count	:	0
Star read count	:	8

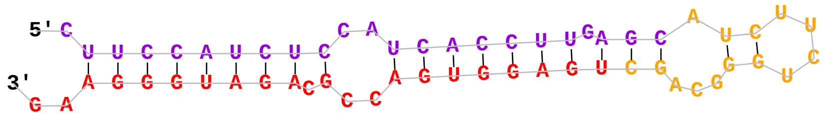

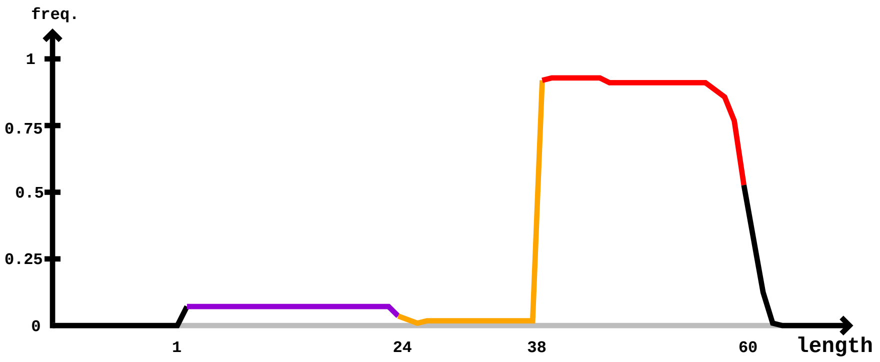

Star **Mature**

```
5'- uccugcccuuuccuuccaucuccaucaccuugagcaucuucugggcagcugaggugaccgcagaugggaaggaagggcgca   -3'    obs
    uccugcccuuuccuuccaucuccaucaccuugagcaucuucugggcagcugaggugaccgcagaugggaaggaagggcgca          exp
    ....((((((((.(((((((((..(((((((.(((.((....))...))))))))))..).)))))))).))))))))...    reads      mm         sample
    .................................................ugaggugaccgcagaugggaa...........    2          0          va1
    .................................................ugaggugaccgcagaugggaagg.........    1          0          va1
    .................................................ugaggugaccgcagaugggaagga........    3          0          va1

    ............cuuccaucuccaucaccuugagc..............................................    1          0          ta2
    .................................................ugaggugaccgcagauggg.............    1          0          ta2
    .................................................ugaggugaccgcagaugggaa...........    1          0          ta2

    .................................................ugaggugaccgcagaugggaagg.........    1          0          k05

    .................................................ugaggugaccgcagaugggaag..........    1          0          k11
    .................................................ugaggugaccgcagaugggaagga........    1          0          k11

    .................................................ugaggugaccgcagaugggaa...........    1          0          s06
    .................................................ugaggugaccgcagaugggaagg.........    1          0          s06
    .................................................ugaggugaccgcagaugggaagga........    1          0          s06

    .................................................ugaggugaccgcagauggg.............    1          0          ta1
    .................................................ugaggugaccgcagauggga............    1          0          ta1
    .................................................ugaggugaccgcagaugggaag..........    1          0          ta1
    .................................................ugaggugaccgcagaugggaagg.........    1          0          ta1
    .................................................ugaggugaccgcagaugggaagga........    1          0          ta1

    .................................................ugaggugaccgcagaugggaag..........    1          0          ku4

    .................................................ugaggugaccgcagaugggaa...........    1          0          k08

    .................................................ugaggugaccgcagauggga............    1          0          ta3
    .................................................ugaggugaccgcagaugggaag..........    1          0          ta3

    .................................................ugaggugaccgcagauggga............    1          0          k10
    .................................................ugaggugaccgcagaugggaag..........    1          0          k10

    .................................................ugaggugaccgcagauggga............    1          0          j19
```

Star Mature

```
uccugcccuuuccuuccaucuccaucaccuugagcaucuucugggcagcugaggugaccgcagaugggaaggaagggcgca

.................................................ugaggugaccgcagaugggaa...........    3    0    k04
.................................................ugaggugaccgcagaugggaag..........    1    0    k04
.................................................ugaggugaccgcagaugggaagg.........    1    0    k04

.................................................ugaggugaccgcagauggga............    1    0    per
.................................................ugaggugaccgcagaugggaag..........    1    0    per

............cuuccaucuccaucaccuugag...............................................    1    0    ku2

............cuuccaucuccaucaccuugagc..............................................    2    0    U20
.................................................ugaggugaccgcagaugggaa...........    1    0    U20
.................................................ugaggugaccgcagaugggaag..........    2    0    U20
.................................................ugaggugaccgcagaugggaaggaa.......    1    0    U20

.................................................ugaggugaccgcagaugggaag..........    1    0    va2

.................................................ugaggugaccgcagaugg..............    1    0    k14

.................................................ugaggugaccgcagaugggaagg.........    1    0    s11

.................................................ugaggugaccgcagaugggaa...........    1    0    k02
.................................................ugaggugaccgcagaugggaag..........    1    0    k02

.................................................ugaggugaccgcagaugggaag..........    1    0    j28

............cuuccaucuccaucaccuugag...............................................    2    0    H52
.................................................ugaggugaccgcagaugggaa...........    2    0    H52
.................................................ugaggugaccgcagaugggaag..........    6    0    H52
.................................................ugaggugaccgcagaugggaagg.........    1    0    H52
.................................................ugaggugaccgcagaugggaagga........    1    0    H52

.................................................ugaggugaccgcagaugggaa...........    3    0    j22

.................................................ugaggugaccgcagaugggaagga........    1    0    k09

.................................................ugaggugaccgcagaugggaa...........    1    0    s08
.................................................ugaggugaccgcagaugggaag..........    1    0    s08
.................................................ugaggugaccgcagaugggaagg.........    1    0    s08

.................................................ugaggugaccgcagauggga............    1    0    B14
.................................................ugaggugaccgcagaugggaa...........    1    0    B14
.................................................ugaggugaccgcagaugggaag..........    3    0    B14
.................................................ugaggugaccgcagaugggaagg.........    1    0    B14
.................................................ugaggugaccgcagaugggaagga........    1    0    B14

.................................................ugaggugaccgcagauggga............    1    0    ku3
.................................................ugaggugaccgcagaugggaa...........    1    0    ku3

.................................................ugaggugaccgcagaugggaagga........    1    0    ya2

....................................ucuucugggcagcugaggug.........................    1    0    la1
.....................................cuucugggcagcugaggug.........................    1    0    la1
.................................................ugaggugaccgcagauggga............    1    0    la1
.................................................ugaggugaccgcagaugggaa...........    2    0    la1
.................................................ugaggugaccgcagaugggaag..........    1    0    la1

.................................................ugaggugaccgcagauggga............    1    0    sh2
.................................................ugaggugaccgcagaugggaag..........    3    0    sh2

.................................................ugaggugaccgcagauggga............    1    0    MCF
.................................................ugaggugaccgcagaugggaa...........    1    0    MCF
.................................................ugaggugaccgcagaugggaagg.........    1    0    MCF

.................................................ugaggugaccgcagaugggaa...........    1    0    j15
.................................................ugaggugaccgcagaugggaag..........    1    0    j15

.................................................ugaggugaccgcagaugggaa...........    1    0    la2
.................................................ugaggugaccgcagaugggaag..........    4    0    la2

.................................................ugaggugaccgcagaugggaa...........    2    0    NL1
```

```
            Star                                    Mature
uccugcccuuuccuuccaucuccaucaccuugagcaucuucugggcagcugaggugaccgcagaugggaaggaagggcgca
................................................ugaggugaccgcagaugggaagga.........    1    0    NL1

............cuuccaucuccaucaccuugagc..............................................    1    0    li2
................................................ugaggugaccgcagaugggaagg..........    1    0    li2

................................................ugaggugaccgcagaugg...............    1    0    k13

................................................ugaggugaccgcagaugggaagga.........    1    0    j20

................................................ugaggugaccgcagaugggaa............    1    0    va3
................................................ugaggugaccgcagaugggaag...........    1    0    va3
................................................ugaggugaccgcagaugggaagga.........    1    0    va3

............cuuccaucuccaucaccuugag...............................................    1    0    SW4
................................................ugaggugaccgcagaugggaag...........    1    0    SW4
................................................ugaggugaccgcagaugggaagg..........    1    0    SW4

................................................ugaggugaccgcagaugg...............    1    0    ya4
.................................................gaggugaccgcagauggg..............    1    0    ya4

................................................ugaggugaccgcagaugggaa............    1    0    j30
```

Provisional ID	:	hsa_can_2013
Score total	:	5.1
Score for star read(s)	:	3.9
Score for read counts	:	0.6
Score for mfe	:	1.2
Score for randfold	:	
Score for cons. seed	:	-0.6
Total read count	:	13
Mature read count	:	12
Loop read count	:	0
Star read count	:	1

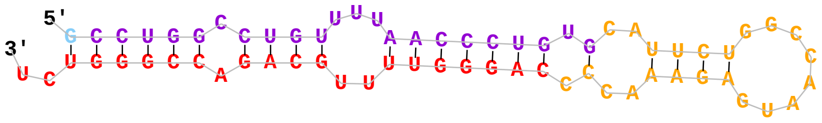

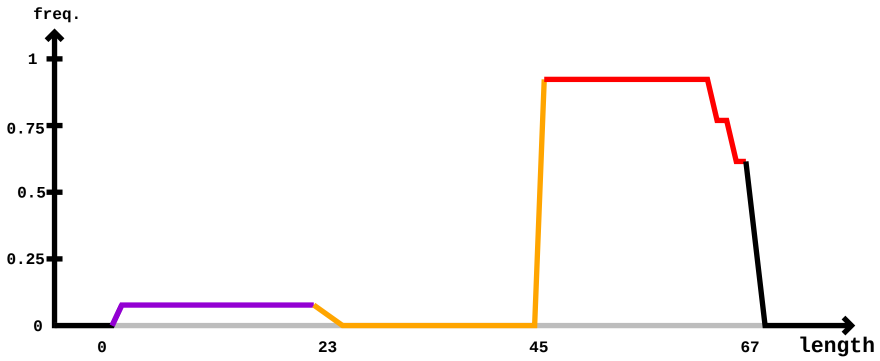


```
      Star                                          Mature
5'- cccagccuggccuguuuuaacccugugcauucuggccaaugagaaaccccaggguuuugcagaccgggucuggaggaugug   -3'    obs
    cccagccuggccuguuuuaacccugugcauucuggccaaugagaaaccccaggguuuugcagaccgggucuggaggaugug          exp
    .(((((((((.((((...(((((((.(..((((........))))..).)))))))..)))).))))).))))........    reads      mm          sample
    .................................................caggguuuugcagaccgggucu..........    1          0           k10

    .................................................caggguuuugcagaccgggu............    2          0           DLD
    .................................................caggguuuugcagaccgggucu..........    1          0           DLD

    .....ccuggccuguuuuaacccugug......................................................    1          0           li1
    .................................................caggguuuugcagaccgggucu..........    1          0           li1

    .................................................caggguuuugcagaccgggucu..........    2          0           U20

    .................................................caggguuuugcagaccgg..............    1          0           j21

    .................................................caggguuuugcagaccgg..............    1          0           k11

    .................................................caggguuuugcagaccgggucu..........    1          0           va3

    .................................................caggguuuugcagaccgggucu..........    1          0           HeL

    .................................................caggguuuugcagaccgggucu..........    1          0           HEK
```

Provisional ID	:	hsa_can_2015
Score total	:	601.1
Score for star read(s)	:	3.9
Score for read counts	:	592
Score for mfe	:	2.2
Score for randfold	:	
Score for cons. seed	:	3
Total read count	:	1173
Mature read count	:	1084
Loop read count	:	0
Star read count	:	89

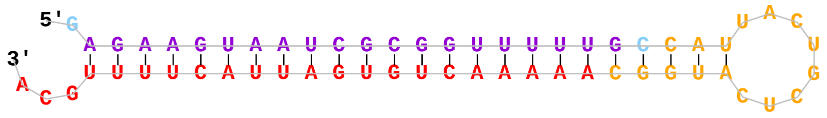

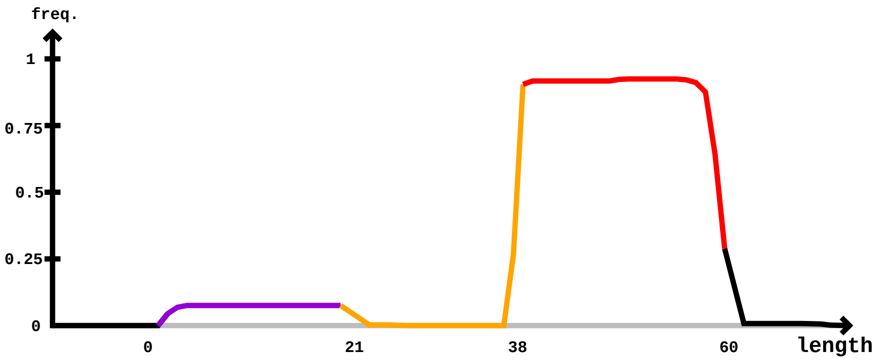


```
             Star                       Mature
5'- cagguuggugagaaguaaucgcgguuuuugccauuacugcucauggcaaaaacugugauuacuuuugcaccaaccuaauau  -3'   obs
    cagguugguggagaaguaaucgcgguuuuugccauuacugcucauggcaaaaacugugauuacuuuugcaccaaccuaauau         exp
    .(((((((((((((((((((((((((((((((((........))))))))))))))))))))))))).)))))))))).....    reads   mm   sample
    ..............................................caaaaacugugauuacuuuu...............    3       0    ya1
    ...............................................aaaaacugugauuacuuuug..............    3       0    ya1
    ...............................................aaaaacugugauuacuuuugc.............    5       0    ya1
    ...............................................aaaaacugugauuacuuuugca............    3       0    ya1

    ...............................................aaaaacugugauuacuuuug..............    1       0    s04

    ...............................................aaaaacugugauuacuuuu...............    1       0    j26
    ...............................................aaaaacugugauuacuuuug..............    3       0    j26
    ...............................................aaaaacugugauuacuuuugca............    1       0    j26

    ..............................................caaaaacugugauuacuuuug..............    5       0    va1
    ..............................................caaaaacugugauuacuuuugc.............    52      0    va1
    ...............................................aaaaacugugauuacuuuug..............    8       0    va1
    ...............................................aaaaacugugauuacuuuugc.............    48      0    va1
    ...............................................aaaaacugugauuacuuuugca............    62      0    va1
    ................................................aaaacugugauuacuuuugc.............    1       0    va1

    ...............................................aaaaacugugauuacuuuug..............    1       0    s06

    ...............................................aaaaacugugauuacuuuugca............    3       0    HeL

    ..............................................caaaaacugugauuacuuuug..............    3       0    ku4
    ...............................................aaaaacugugauuacuuuug..............    1       0    ku4
    ...............................................aaaaacugugauuacuuuugc.............    1       0    ku4
    ...............................................aaaaacugugauuacuuuugca............    1       0    ku4

    ..............................................caaaaacugugauuacuuuu...............    1       0    s07
    ..............................................caaaaacugugauuacuuuugc.............    1       0    s07

    ...............................................aaaaacugugauuacuuuugc.............    1       0    s10
    ...............................................aaaaacugugauuacuuuugca............    1       0    s10

    ...............................................aaaaacugugauuacuuuugca............    1       0    ta3
```

Star **Mature**

```
cagguuggugagaaguaaucgcgguuuuugccauuacugcucauggcaaaaacugugauuacuuuugcaccaaccuaauau

...............................................aaaaacugugauuacuuuugca............     7     0     j06

..............................................caaaaacugugauuacuuuug..............     3     0     j10
..............................................caaaaacugugauuacuuuugc.............     8     0     j10
...............................................aaaaacugugauuacuuuug..............     4     0     j10
...............................................aaaaacugugauuacuuuugc.............     10    0     j10
...............................................aaaaacugugauuacuuuugca............     13    0     j10

..............................................caaaaacugugauuacuuuug..............     3     0     j04
...............................................aaaaacugugauuacuuuugc.............     5     0     j04
................................................aaaacugugauuacuuuugca............     4     0     j04

..............................................caaaaacugugauuacuuuug..............     2     0     j02
..............................................caaaaacugugauuacuuuugc.............     8     0     j02
...............................................aaaaacugugauuacuuuu...............     1     0     j02
...............................................aaaaacugugauuacuuuug..............     8     0     j02
...............................................aaaaacugugauuacuuuugc.............     6     0     j02
...............................................aaaaacugugauuacuuuugca............     9     0     j02

..............................................caaaaacugugauuacuuu................     1     0     va2
...............................................aaaaacugugauuacuuuugc.............     1     0     va2
...............................................aaaaacugugauuacuuuugca............     4     0     va2

...............................................aaaaacugugauuacuuuug..............     1     0     j27
...............................................aaaaacugugauuacuuuugc.............     5     0     j27
...............................................aaaaacugugauuacuuuugca............     1     0     j27

..............................................caaaaacugugauuacuuuugc.............     2     0     j31
...............................................aaaaacugugauuacuuuug..............     1     0     j31
...............................................aaaaacugugauuacuuuugc.............     4     0     j31
...............................................aaaaacugugauuacuuuugca............     7     0     j31
................................................aaaacugugauuacuuuugca............     1     0     j31

............aaguaaucgcgguuuuugccauu..............................................     1     0     H52

..............................................caaaaacugugauuacuuuu...............     1     0     j22
..............................................caaaaacugugauuacuuuug..............     6     0     j22
..............................................caaaaacugugauuacuuuugc.............     15    0     j22
...............................................aaaaacugugauuacuuu................     1     0     j22
...............................................aaaaacugugauuacuuuug..............     13    0     j22
...............................................aaaaacugugauuacuuuugc.............     14    0     j22
...............................................aaaaacugugauuacuuuugca............     20    0     j22

...............................................aaaaacugugauuacuuuugc.............     1     0     B14
...............................................aaaaacugugauuacuuuugca............     4     0     B14

..............................................caaaaacugugauuacuuuug..............     1     0     j16

..........agaaguaaucgcgguuuuug...................................................     1     0     j09
..............................................caaaaacugugauuacuuuu...............     1     0     j09
..............................................caaaaacugugauuacuuuug..............     1     0     j09
...............................................aaaaacugugauuacuuuugc.............     2     0     j09
...............................................aaaaacugugauuacuuuugca............     1     0     j09

..............................................caaaaacugugauuacuuuug..............     1     0     j18

..............................................caaaaacugugauuacuuuug..............     1     0     ku3
..............................................caaaaacugugauuacuuuugc.............     2     0     ku3
...............................................aaaaacugugauuacuuuug..............     3     0     ku3
...............................................aaaaacugugauuacuuuugc.............     2     0     ku3
...............................................aaaaacugugauuacuuuugca............     2     0     ku3

..............................................caaaaacugugauuacuuuugc.............     1     0     ku6
...............................................aaaaacugugauuacuuuug..............     2     0     ku6
...............................................aaaaacugugauuacuuuugca............     1     0     ku6

...............................................aaaaacugugauuacuuuug..............     2     0     j11
...............................................aaaaacugugauuacuuuugca............     3     0     j11

..............................................caaaaacugugauuacuuuug..............     1     0     s12
```

Star **Mature**

```
cagguuggugagaaguaaucgcgguuuuugccauuacugcucauggcaaaaacugugauuacuuuugcaccaaccuaauau

..........agaaguaaucgcgguuuu.....................................................   1    0    ya3
...........gaaguaaucgcgguuuu.....................................................   2    0    ya3
..............................................caaaaacugugauuacuuuu...............   2    0    ya3
...............................................aaaaacugugauuacuuu................   1    0    ya3
...............................................aaaaacugugauuacuuuu...............   6    0    ya3
...............................................aaaaacugugauuacuuuugc.............   2    0    ya3
...............................................aaaaacugugauuacuuuugca............   1    0    ya3

..............................................caaaaacugugauuacuuuugc.............   1    0    HEK
...............................................aaaaacugugauuacuuuugca............   3    0    HEK

..........agaaguaaucgcgguuuuugc..................................................   1    0    la1
...............................................aaaaacugugauuacuuuugca............   2    0    la1

..............................................caaaaacugugauuacuuu................   1    0    j17
..............................................caaaaacugugauuacuuuug..............   1    0    j17
..............................................caaaaacugugauuacuuuugc.............   1    0    j17
...............................................aaaaacugugauuacuuuug..............   5    0    j17
...............................................aaaaacugugauuacuuuugc.............   7    0    j17
...............................................aaaaacugugauuacuuuugca............   4    0    j17

..............................................caaaaacugugauuacuuuu...............   1    0    j15
..............................................caaaaacugugauuacuuuug..............   7    0    j15
...............................................aaaaacugugauuacuuu................   2    0    j15
...............................................aaaaacugugauuacuuuug..............   14   0    j15
...............................................aaaaacugugauuacuuuugca............   4    0    j15

..............................................caaaaacugugauuacuuuugc.............   2    0    la2
...............................................aaaaacugugauuacuuuugc.............   1    0    la2
...............................................aaaaacugugauuacuuuugca............   1    0    la2

...............................................aaaaacugugauuacuuuug..............   14   0    j08

...............................................aaaaacugugauuacuuuug..............   4    0    j12
...............................................aaaaacugugauuacuuuugc.............   2    0    j12
...............................................aaaaacugugauuacuuuugca............   5    0    j12

............aaguaaucgcgguuuuugccau...............................................   1    0    li2
..............................................caaaaacugugauuacuuuugc.............   13   0    li2
...............................................aaaaacugugauuacuuuugc.............   7    0    li2
...............................................aaaaacugugauuacuuuugca............   12   0    li2
................................................aaaacugugauuacuuuugca............   3    0    li2

..............................................caaaaacugugauuacuuuugc.............   3    0    va4
...............................................aaaaacugugauuacuuuugc.............   5    0    va4
...............................................aaaaacugugauuacuuuugca............   1    0    va4

..............................................caaaaacugugauuacuuuugc.............   11   0    va3
...............................................aaaaacugugauuacuuuug..............   2    0    va3
...............................................aaaaacugugauuacuuuugc.............   6    0    va3
...............................................aaaaacugugauuacuuuugca............   11   0    va3

..........agaaguaaucgcgguuuu.....................................................   1    0    ya4
..............................................caaaaacugugauuacuuuu...............   2    0    ya4
...............................................aaaaacugugauuacuuu................   1    0    ya4
...............................................aaaaacugugauuacuuuugc.............   7    0    ya4

..............................................caaaaacugugauuacuuuug..............   6    0    ku1
..............................................caaaaacugugauuacuuuugc.............   4    0    ku1
...............................................aaaaacugugauuacuuuug..............   7    0    ku1
...............................................aaaaacugugauuacuuuugca............   2    0    ku1

..............................................caaaaacugugauuacuuuu...............   3    0    j23
..............................................caaaaacugugauuacuuuug..............   7    0    j23
..............................................caaaaacugugauuacuuuugc.............   26   0    j23
...............................................aaaaacugugauuacuuu................   2    0    j23
...............................................aaaaacugugauuacuuuu...............   1    0    j23
...............................................aaaaacugugauuacuuuug..............   34   0    j23
...............................................aaaaacugugauuacuuuugc.............   30   0    j23
```

```
        Star                                   Mature
cagguuggugagaaguaaucgcgguuuuugccauuacugcucauggcaaaaacugugauuacuuuugcaccaaccuaauau
...............................................aaaaacugugauuacuuuugca............    29    0    j23

...............................................aaaaacugugauuacuuuug..............    1     0    s05

...............................................aaaaacugugauuacuuuugc.............    1     0    ta2

...............................................aaaaacugugauuacuuuu...............    3     0    j24
...............................................aaaaacugugauuacuuuug..............    5     0    j24
...............................................aaaaacugugauuacuuuugca............    4     0    j24

...............................................aaaaacugugauuacuuuugc.............    2     0    k11
...............................................aaaaacugugauuacuuuugca............    1     0    k11

...............................................aaaaacugugauuacuuuugc.............    4     0    ta1
...............................................aaaaacugugauuacuuuugca............    2     0    ta1

...............................................aaaaacugugauuacuuuu...............    1     0    s09

..........agaaguaaucgcgguuuuu....................................................    11    0    NL2
..........agaaguaaucgcgguuuuug...................................................    11    0    NL2
...........gaaguaaucgcgguuuuu....................................................    1     0    NL2
...........gaaguaaucgcgguuuuug...................................................    5     0    NL2
..............................................caaaaacugugauuacuuuugc.............    1     0    NL2
...............................................aaaaacugugauuacuuuu...............    2     0    NL2
...............................................aaaaacugugauuacuuuug..............    1     0    NL2
...............................................aaaaacugugauuacuuuugc.............    1     0    NL2
................................................aaaacugugauuacuuuugca............    1     0    NL2

..............................................caaaaacugugauuacuuuug..............    5     0    j13
..............................................caaaaacugugauuacuuuugc.............    1     0    j13
...............................................aaaaacugugauuacuuuugca............    8     0    j13

..............................................caaaaacugugauuacuuuugc.............    1     0    k10
...............................................aaaaacugugauuacuuuugc.............    1     0    k10
................................................aaaacugugauuacuuuugca............    1     0    k10

..............................................caaaaacugugauuacuuuu...............    2     0    j19
..............................................caaaaacugugauuacuuuug..............    3     0    j19
..............................................caaaaacugugauuacuuuugc.............    2     0    j19
...............................................aaaaacugugauuacuuuu...............    2     0    j19
...............................................aaaaacugugauuacuuuug..............    21    0    j19
...............................................aaaaacugugauuacuuuugc.............    2     0    j19
...............................................aaaaacugugauuacuuuugca............    13    0    j19

..............................................caaaaacugugauuacuuuug..............    2     0    j03
...............................................aaaaacugugauuacuuu................    1     0    j03
...............................................aaaaacugugauuacuuuug..............    6     0    j03
...............................................aaaaacugugauuacuuuugca............    3     0    j03

...............................................aaaaacugugauuacuuuugc.............    1     0    k01
...............................................aaaaacugugauuacuuuugca............    1     0    k01

..............................................caaaaacugugauuacuuuug..............    2     0    j25
...............................................aaaaacugugauuacuuuug..............    3     0    j25
...............................................aaaaacugugauuacuuuugc.............    2     0    j25
...............................................aaaaacugugauuacuuuugca............    3     0    j25

.........................................................auuacuuuugcaccaaccuaau..    1     0    k04

...............................................aaaaacugugauuacuuuugca............    5     0    s01

...............................................aaaaacugugauuacuuuug..............    1     0    per

..............................................caaaaacugugauuacuuuu...............    1     0    ku2
..............................................caaaaacugugauuacuuuug..............    9     0    ku2
..............................................caaaaacugugauuacuuuugc.............    4     0    ku2
...............................................aaaaacugugauuacuuuu...............    1     0    ku2
...............................................aaaaacugugauuacuuuug..............    5     0    ku2
...............................................aaaaacugugauuacuuuugc.............    3     0    ku2
...............................................aaaaacugugauuacuuuugca............    3     0    ku2
```

Star **Mature**

cagguuggugagaaguaaucgcgguuuuugccauuacugcucauggcaaaaacugugauuacuuuugcaccaaccuaauau

....caaaaacugugauuacuuuugc.............	1	0	U20
....aaaacugugauuacuuuugca............	1	0	U20
....caaaaacugugauuacuuuug..............	1	0	j21
....caaaaacugugauuacuuuugc.............	5	0	j21
....aaaaacugugauuacuuuug..............	5	0	j21
....aaaaacugugauuacuuuugca............	4	0	j21
....aaaacugugauuacuuuug..............	2	0	j21
....caaaaacugugauuacuuuug..............	1	0	s11
....caaaaacugugauuacuuuug..............	4	0	j07
....caaaaacugugauuacuuuugc.............	1	0	j07
....aaaaacugugauuacuuuug..............	7	0	j07
....aaaaacugugauuacuuuugc.............	2	0	j07
....caaaaacugugauuacuuuugc.............	13	0	j14
....aaaaacugugauuacuuuugc.............	1	0	j14
....aaaaacugugauuacuuuugca............	7	0	j14
....aaaaacugugauuacuuuugcac..........	1	0	j14
....caaaaacugugauuacuuuugc.............	1	0	k02
....aaaaacugugauuacuuuugc.............	2	0	k02
....aaaaacugugauuacuuuugca............	1	0	j28
....caaaaacugugauuacuu.................	1	0	s08
....aaaaacugugauuacuuuugca............	1	0	s08
....aaaaacugugauuacuuuugca............	2	0	DLD
....caaaaacugugauuacuuuug..............	1	0	k03
....caaaaacugugauuacuu.................	3	0	ya2
....caaaaacugugauuacuuuu...............	1	0	ya2
....aaaaacugugauuacuuuugca............	4	0	ya2
....auuacuuuugcaccaaccuaauau	1	0	ya2
..........agaaguaaucgcgguuuuu..	11	0	NL3
..........agaaguaaucgcgguuuuug...	7	0	NL3
...........gaaguaaucgcgguuuuu..	15	0	NL3
...........gaaguaaucgcgguuuuug...	3	0	NL3
............aaguaaucgcgguuuuug...	3	0	NL3
....caaaaacugugauuacuuuug..............	1	0	NL3
....caaaaacugugauuacuuuugc.............	4	0	NL3
....aaaaacugugauuacuuuugc.............	3	0	NL3
....aaaacugugauuacuuuugca............	1	0	NL3
....auuacuuuugcaccaaccuaa...	1	0	NL3
....caaaaacugugauuacuuuugc.............	1	0	k06
....aaaaacugugauuacuuuug..............	1	0	k06
....uuacuuuugcaccaaccua....	1	0	sh2
....aaaaacugugauuacuuuug..............	2	0	j29
....aaaaacugugauuacuuuugca............	1	0	MCF
....auuacuuuugcaccaaccuaau..	4	0	MCF
....caaaaacugugauuacuuuu...............	2	0	j01
....aaaaacugugauuacuuuugca............	2	0	j01
..........agaaguaaucgcgguuuuu..	2	0	NL1
..........agaaguaaucgcgguuuuug...	7	0	NL1
...........gaaguaaucgcgguuuuug...	2	0	NL1
............aaguaaucgcgguuuuug...	2	0	NL1
............aaguaaucgcgguuuuugccau...	1	0	NL1
....aaaaacugugauuacuuuugca............	1	0	NL1
....caaaaacugugauuacuuuugc.............	2	0	MBM

Star   Mature

```
cagguuggugagaaguaaucgcgguuuuugccauuacugcucauggcaaaaacugugauuacuuuugcaccaaccuaauau
...............................................aaaaacugugauuacuuuugca............     5     0     MBM

...............................................aaaaacugugauuacuuuugca............     1     0     k07

..............................................caaaaacugugauuacuuuugc.............     20    0     li1
...............................................aaaaacugugauuacuuuugc.............     5     0     li1
...............................................aaaaacugugauuacuuuugca............     24    0     li1

..........................................................uuacuuuugcaccaaccuaaua.     1     0     k13

..............................................caaaaacugugauuacuuuug..............     1     0     j20
..............................................caaaaacugugauuacuuuugc.............     2     0     j20
...............................................aaaaacugugauuacuuuu...............     3     0     j20
...............................................aaaaacugugauuacuuuug..............     8     0     j20
...............................................aaaaacugugauuacuuuugc.............     3     0     j20
...............................................aaaaacugugauuacuuuugca............     2     0     j20

..............................................caaaaacugugauuacuuuu...............     2     0     j30
..............................................caaaaacugugauuacuuuugc.............     1     0     j30
...............................................aaaaacugugauuacuuuugca............     2     0     j30

...............................................aaaaacugugauuacuuuu...............     1     0     k12
...............................................aaaaacugugauuacuuuugc.............     1     0     k12
```

Provisional ID	:	hsa_can_2183
Score total	:	3.4
Score for star read(s)	:	3.9
Score for read counts	:	0.6
Score for mfe	:	-0.5
Score for randfold	:	
Score for cons. seed	:	-0.6
Total read count	:	13
Mature read count	:	11
Loop read count	:	0
Star read count	:	2

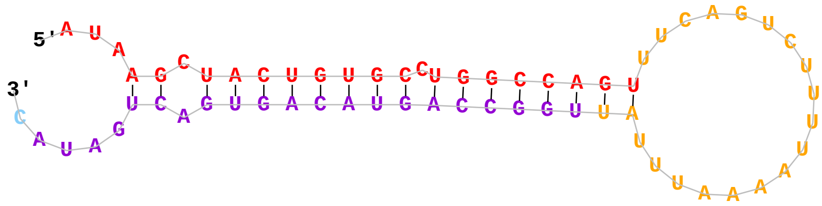

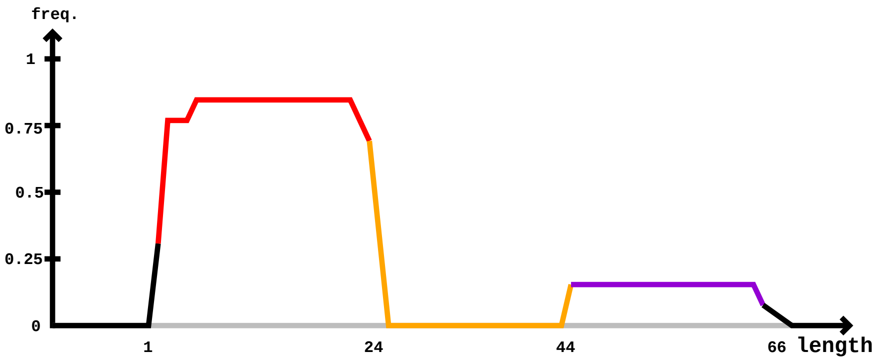

Mature **Star**

```
5'- auuauuggcauaagcuacugugccuggccaguuucagucuuuuaaaauuuauuggccaguacagugacugauaccuauaau  -3'   obs
    auuauuggcauaagcuacugugccuggccaguuucagucuuuuaaaauuuauuggccaguacagugacugauaccuauaau        exp
    (((((.((.((.((.((((((((.((((((((..................)))))))))))))))).)).)).)).)))))   reads     mm        sample
    ..........uaagcuacugugccuggcca...................................................   1         0         j20

    .............gcuacugugccuggccagu.................................................   1         0         B14

    ....................................................uggccaguacagugacugaua........   1         0         va3

    ..........uaagcuacugugccuggccagu.................................................   2         0         SW4
    ....................................................uggccaguacagugacugau.........   1         0         SW4

    ..........uaagcuacugugccuggccagu.................................................   1         0         HEK

    ..........uaagcuacugugccuggccagu.................................................   1         0         j10

    ..........uaagcuacugugccuggccag..................................................   1         0         MCF

    .........auaagcuacugugccuggccagu.................................................   4         0         H52
```

Provisional ID	:	hsa_can_2196
Score total	:	30.2
Score for star read(s)	:	3.9
Score for read counts	:	24.6
Score for mfe	:	2.3
Score for randfold	:	
Score for cons. seed	:	-0.6
Total read count	:	60
Mature read count	:	46
Loop read count	:	0
Star read count	:	14

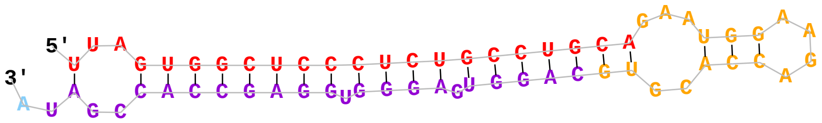

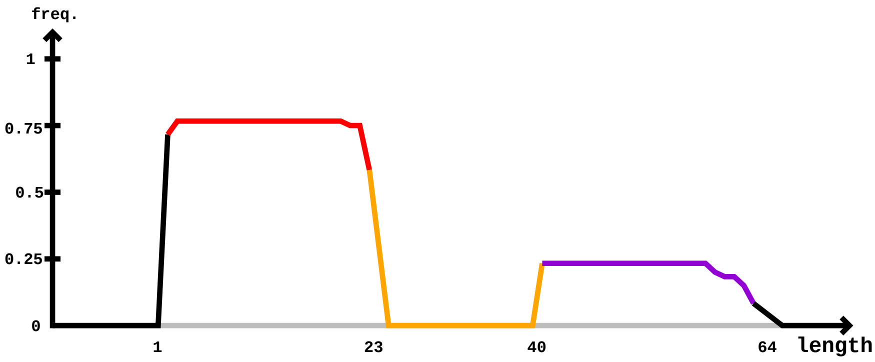

Mature **Star**

```
5'- gacccagguuuuaguggcucccucugccugcagaauggaagaccacgugcaggugaggguggagccaccgauaccugggug  -3'   obs
    gacccagguuuuaguggcucccucugccugcagaauggaagaccacgugcaggugaggguggagccaccgauaccugggug         exp
    .((((((((.(..((((((((((((((((((...(((....)))..))))))).)))).))))))))..).)))))))).   reads   mm   sample
    ..........uuaguggcucccucugccu....................................................   1       0    ya1

    ..........uuaguggcucccucugccugca.................................................   1       0    j26

    ..........uuaguggcucccucugccugca.................................................   7       0    va1
    ...........uaguggcucccucugccugca.................................................   1       0    va1

    ..........uuaguggcucccucugccugca.................................................   1       0    ta2

    .................................................caggugaggguggagccaccga..........   1       0    s06

    ..........uuaguggcucccucugccugca.................................................   1       0    ta1
    .................................................caggugaggguggagccaccgau.........   1       0    ta1

    ..........uuaguggcucccucugccugc..................................................   1       0    HeL
    .................................................caggugaggguggagccaccgau.........   2       0    HeL

    ..........uuaguggcucccucugccugca.................................................   1       0    ku4

    ..........uuaguggcucccucugccugca.................................................   1       0    s10
    .................................................caggugaggguggagccaccga..........   2       0    s10

    ..........uuaguggcucccucugccugca.................................................   2       0    k10

    ..........uuaguggcucccucugccugca.................................................   1       0    j13

    ..........uuaguggcucccucugccugca.................................................   1       0    j19

    ..........uuaguggcucccucugccugca.................................................   1       0    s01
    .................................................caggugaggguggagccaccg...........   2       0    s01

    ..........uuaguggcucccucugccugc..................................................   1       0    k04

    ..........uuaguggcucccucugccugca.................................................   2       0    U20
```

```
          Mature                                Star
gacccagguuuuaguggcucccucugccugcagaauggaagaccacgugcaggugaggguggagccaccgauaccugggug

..........uuaguggcucccucugccugca.................................................    1    0    j21

..........uuaguggcucccucugccugca.................................................    1    0    j27

..........uuaguggcucccucugccugca.................................................    1    0    k02
...........uaguggcucccucugccugc..................................................    1    0    k02

..........uuaguggcucccucugccugca.................................................    1    0    j28

..........uuaguggcucccucugccugc..................................................    1    0    H52

.................................................caggugaggguggagccaccgau.........    2    0    j22

..........uuaguggcucccucugccugca.................................................    1    0    s08

..........uuaguggcucccucugccugca.................................................    1    0    B14

..........uuaguggcucccucugccugca.................................................    1    0    ku3

..........uuaguggcucccucugccugca.................................................    2    0    j18
...........uaguggcucccucugccugca.................................................    1    0    j18

.................................................caggugaggguggagcca..............    2    0    ya2

.................................................caggugaggguggagccaccga..........    1    0    s12

..........uuaguggcucccucugccugc..................................................    5    0    sh2

.................................................caggugaggguggagccac.............    1    0    la1

..........uuaguggcucccucugccugc..................................................    1    0    MBM
..........uuaguggcucccucugccugca.................................................    1    0    MBM

..........uuaguggcucccucugccugca.................................................    1    0    li1

..........uuaguggcucccucugccugca.................................................    1    0    va4

..........uuaguggcucccucugccugca.................................................    1    0    va3

..........uuaguggcucccucugccugca.................................................    1    0    ku1
```

Provisional ID	:	hsa_can_2335
Score total	:	1.2
Score for star read(s)	:	3.9
Score for read counts	:	-3.9
Score for mfe	:	1.9
Score for randfold	:	
Score for cons. seed	:	-0.6
Total read count	:	4
Mature read count	:	3
Loop read count	:	0
Star read count	:	1

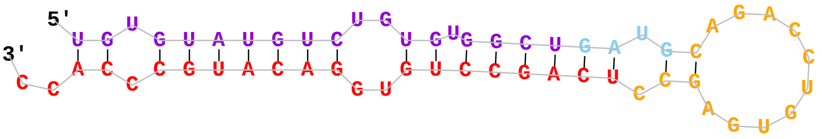

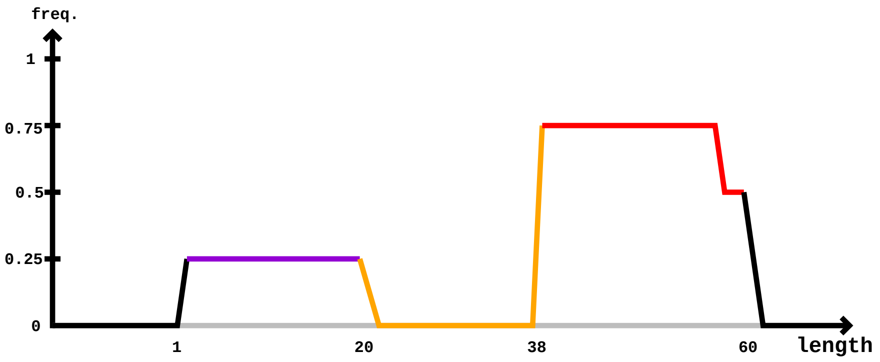

Star **Mature**

```
5'- ggggugagcccguguguaugucuguguggcugaugcagaccugugagccucagccuguggacaugcccaccggcgggccuc   -3'    obs
    ggggugagcccguguguaugucuguguggcugaugcagaccugugagccucagccuguggacaugcccaccggcgggccuc          exp
    (((((..(((.(((.(((((((..((.((((((.((..........)).))))))))..))))))).))).)))..)))))    reads      mm         sample
    ............uguguaugucuguguggcu..................................................    1          0          k09

    .................................................ucagccuguggacaugcccacc..........    1          0          ta2

    .................................................ucagccuguggacaugcccacc..........    1          0          s01

    .................................................ucagccuguggacaugccc.............    1          0          j30
```

```
Provisional ID            : hsa_can_2403
Score total               :    910.5
Score for star read(s)    :      3.9
Score for read counts     :    901.5
Score for mfe             :      2.1
Score for randfold        :
Score for cons. seed      :        3
Total read count          :     1780
Mature read count         :     1639
Loop read count           :        0
Star read count           :      141
```

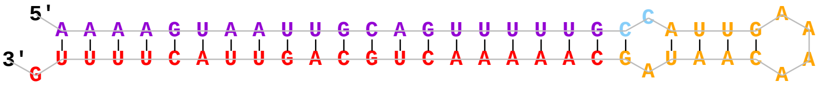

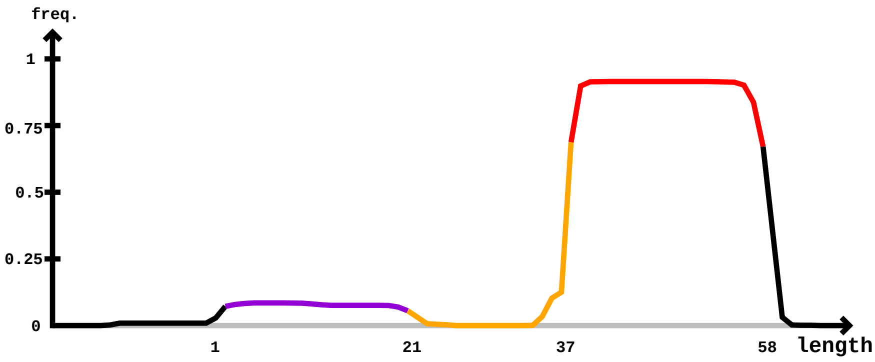

Star Mature

```
5'- aacuauagguuggugcaaaaguaauugcaguuuuugccauugaaaacaauagcaaaaacugcaguuacuuuugcaccaauu -3'  obs
    aacuauagguuggugcaaaaguaauugcaguuuuugccauugaaaacaauagcaaaaacugcaguuacuuuugcaccaauu      exp
    aacuauagguuggugcaaaaguaauugcaguuuuugccauugaaaacaauagcaaaaacugcaguuacuuuugcaccaauu      known
    .......((((((((((((((((((((((((((((((.((((....)))).))))))))))))))))))))))))))))))  reads  mm  sample
    .....uagguuggugcaaaaguaauu.......................................................  2      0   ya1
    ...............caaaaguaauugcaguuuuu..............................................  1      0   ya1
    ..................................................agcaaaaacugcaguuacuuuu.........  2      0   ya1
    ....................................................caaaaacugcaguuacuuuu.........  1      0   ya1
    ....................................................caaaaacugcaguuacuuuug........  2      0   ya1
    .....................................................aaaaacugcaguuacuuuu.........  3      0   ya1
    .....................................................aaaaacugcaguuacuuuugc.......  1      0   ya1

    ..................................................agcaaaaacugcaguuacuuu..........  1      0   j26
    ..................................................agcaaaaacugcaguuacuuuu.........  1      0   j26

    .................................................uagcaaaaacugcaguuacuuu..........  1      0   va1
    ..................................................agcaaaaacugcaguuacuuuu.........  9      0   va1
    ...................................................gcaaaaacugcaguuacuuuug........  5      0   va1
    ....................................................caaaaacugcaguuacuuuug........  10     0   va1
    ....................................................caaaaacugcaguuacuuuugc.......  10     0   va1
    .....................................................aaaaacugcaguuacuuuug........  2      0   va1
    .....................................................aaaaacugcaguuacuuuugca......  1      0   va1

    ................aaaaguaauugcaguuuuug.............................................  1      0   ku5

    ....................................................caaaaacugcaguuacuuuug........  1      0   k05
    ....................................................caaaaacugcaguuacuuuugc.......  4      0   k05
    .....................................................aaaaacugcaguuacuuuugc.......  1      0   k05

    ................................................auagcaaaaacugcaguuacuu...........  1      0   s06
    ..................................................agcaaaaacugcaguuacuuu..........  1      0   s06

    ................aaaaguaauugcaguuuuugc............................................  2      0   HeL
    ..................aaguaauugcaguuuuugccau.........................................  2      0   HeL
    .................................................uagcaaaaacugcaguuacuuu..........  2      0   HeL
    .....................................................aaaaacugcaguuacuuuugc.......  1      0   HeL
```

```
            Star                                Mature
aacuauagguuggugcaaaaguaauugcaguuuuugccauugaaaacaauagcaaaaacugcaguuacuuuugcaccaauu

.................................................uagcaaaaacugcaguuacuuu..........  1    0    s07

....................................................caaaaacugcaguuacuuuu.........  1    0    k08
....................................................caaaaacugcaguuacuuuug........  4    0    k08

.................................................uagcaaaaacugcaguuacuuu..........  1    0    s10
....................................................caaaaacugcaguuacuuuu.........  1    0    s10
....................................................caaaaacugcaguuacuuuug........  2    0    s10
....................................................caaaaacugcaguuacuuuugc.......  1    0    s10
.....................................................aaaaacugcaguuacuuuugc.......  1    0    s10
.....................................................aaaaacugcaguuacuuuugca......  1    0    s10

.................................................uagcaaaaacugcaguuacuuu..........  3    0    j10
.................................................uagcaaaaacugcaguuacuuuu.........  1    0    j10
..................................................agcaaaaacugcaguuacuuu..........  2    0    j10
..................................................agcaaaaacugcaguuacuuuu.........  4    0    j10
...................................................gcaaaaacugcaguuacuuuug........  3    0    j10
....................................................caaaaacugcaguuacuuuugc.......  1    0    j10

.................aaaguaauugcaguuuuug.............................................  2    0    j04
....................................................caaaaacugcaguuacuuuug........  2    0    j04

....................................................caaaaacugcaguuacuuuug........  1    0    k14
....................................................caaaaacugcaguuacuuuugc.......  3    0    k14
.....................................................aaaaacugcaguuacuuuugca......  1    0    k14

.....uagguuggugcaaaaguaauu.......................................................  1    0    j02
.....uagguuggugcaaaaguaauug......................................................  1    0    j02
................aaaaguaauugcaguuuuug.............................................  2    0    j02
..................................................agcaaaaacugcaguuacuuu..........  3    0    j02
..................................................agcaaaaacugcaguuacuuuu.........  1    0    j02
...................................................gcaaaaacugcaguuacuuuu.........  1    0    j02
...................................................gcaaaaacugcaguuacuuuug........  4    0    j02
....................................................caaaaacugcaguuacuuu..........  2    0    j02

....auagguuggugcaaaaguaa.........................................................  1    0    va2
.....uagguuggugcaaaaguaauug......................................................  1    0    va2
..................................................agcaaaaacugcaguuacuuuu.........  5    0    va2
...................................................gcaaaaacugcaguuacuuuug........  1    0    va2
....................................................caaaaacugcaguuacuuuug........  1    0    va2
....................................................caaaaacugcaguuacuuuugc.......  2    0    va2

.................................................uagcaaaaacugcaguuacuuu..........  1    0    j31
..................................................agcaaaaacugcaguuacuuuu.........  2    0    j31
...................................................gcaaaaacugcaguuacuuuug........  1    0    j31
....................................................caaaaacugcaguuacuuuug........  1    0    j31
....................................................caaaaacugcaguuacuuuugc.......  1    0    j31
.....................................................aaaaacugcaguuacuuuugca......  1    0    j31

................aaaaguaauugcaguuuuu..............................................  3    0    j27

....................................................caaaaacugcaguuacuu...........  2    0    H52
....................................................caaaaacugcaguuacuuuu.........  7    0    H52
....................................................caaaaacugcaguuacuuuug........  31   0    H52
....................................................caaaaacugcaguuacuuuugc.......  78   0    H52
.....................................................aaaaacugcaguuacuuuu.........  5    0    H52
.....................................................aaaaacugcaguuacuuuug........  6    0    H52
.....................................................aaaaacugcaguuacuuuugc.......  13   0    H52
.....................................................aaaaacugcaguuacuuuugca......  12   0    H52
......................................................aaaacugcaguuacuuuugc.......  4    0    H52

...................................................gcaaaaacugcaguuacuuuug........  13   0    j05

.................................................uagcaaaaacugcaguuacuuu..........  2    0    s03
..................................................agcaaaaacugcaguuacuuu..........  1    0    s03

.................................................uagcaaaaacugcaguuacuu...........  1    0    j22
..................................................agcaaaaacugcaguuacuuuu.........  4    0    j22
...................................................gcaaaaacugcaguuacuuuug........  2    0    j22
```

```
                Star                                Mature
aacuauagguuggugcaaaaguaauugcaguuuuugccauugaaaacaauagcaaaaacugcaguuacuuuugcaccaauu
....................................................caaaaacugcaguuacuuuu.........   1   0   j22
....................................................caaaaacugcaguuacuuuug........   1   0   j22
....................................................caaaaacugcaguuacuuuugc.......   2   0   j22

.....uagguuggugcaaaaguaauug......................................................   1   0   k09
................aaaaguaauugcaguuuuu..............................................   3   0   k09
....................................................caaaaacugcaguuacuuuug........   2   0   k09
....................................................caaaaacugcaguuacuuuugc.......   5   0   k09
.....................................................aaaaacugcaguuacuuuug........   1   0   k09
........................................................aacugcaguuacuuuugcacca...   1   0   k09

.................aaaguaauugcaguuuuugc............................................   1   0   B14
..................aaguaauugcaguuuuugccau.........................................   1   0   B14
.................................................uagcaaaaacugcaguuacu............   1   0   B14
.................................................uagcaaaaacugcaguuacuuu..........   1   0   B14

....................................................caaaaacugcaguuacuuuug........   3   0   j18

.................................................uagcaaaaacugcaguuacuuu..........   1   0   j16
..................................................agcaaaaacugcaguuacuuuu.........   1   0   j16
....................................................caaaaacugcaguuacuuuu.........   2   0   j16
....................................................caaaaacugcaguuacuuuug........   4   0   j16
.....................................................aaaaacugcaguuacuuuu.........   1   0   j16
.....................................................aaaaacugcaguuacuuuug........   6   0   j16
......................................................aaaacugcaguuacuuuug........   3   0   j16

................aaaaguaauugcaguuuuu..............................................   1   0   ku3
................aaaaguaauugcaguuuuug.............................................   2   0   ku3
.................................................uagcaaaaacugcaguuacuu...........   1   0   ku3
..................................................agcaaaaacugcaguuacuu...........   1   0   ku3
..................................................agcaaaaacugcaguuacuuuu.........   1   0   ku3
....................................................caaaaacugcaguuacuuuug........   1   0   ku3

.................aaaguaauugcaguuuuu..............................................   1   0   j09
.................................................uagcaaaaacugcaguuacuuuug........   1   0   j09
..................................................agcaaaaacugcaguuacuuuu.........   1   0   j09
....................................................caaaaacugcaguuacuuuugc.......   1   0   j09

..................................................agcaaaaacugcaguuacuu...........   1   0   ku6

.................................................uagcaaaaacugcaguuacuu...........   2   0   j11
..................................................agcaaaaacugcaguuacuu...........   1   0   j11
..................................................agcaaaaacugcaguuacuuuu.........   5   0   j11

..................................................agcaaaaacugcaguuacuuuu.........   1   0   s12
....................................................caaaaacugcaguuacuuuugc.......   1   0   s12

.....uagguuggugcaaaaguaauu.......................................................   1   0   ya3
...............caaaaguaauugcaguuu................................................   2   0   ya3
................aaaaguaauugcaguuu................................................   1   0   ya3
................aaaaguaauugcaguuuu...............................................   1   0   ya3
..................................................agcaaaaacugcaguuacuuuu.........   3   0   ya3
....................................................caaaaacugcaguuacuu...........   2   0   ya3
....................................................caaaaacugcaguuacuuu..........   3   0   ya3
....................................................caaaaacugcaguuacuuuug........   1   0   ya3
.....................................................aaaaacugcaguuacuuuu.........   2   0   ya3
.....................................................aaaaacugcaguuacuuuug........   1   0   ya3
......................................................aaaacugcaguuacuuuu.........   1   0   ya3

..................................................agcaaaaacugcaguuacuuuu.........   2   0   la1
...................................................gcaaaaacugcaguuacuuuu.........   1   0   la1

...............caaaaguaauugcaguuuuu..............................................   2   0   HEK
...............caaaaguaauugcaguuuuug.............................................   1   0   HEK
................aaaaguaauugcaguuuuugcc...........................................   1   0   HEK
.................................................uagcaaaaacugcaguuacu............   2   0   HEK
.................................................uagcaaaaacugcaguuacuuu..........   3   0   HEK
..................................................agcaaaaacugcaguuacuuuu.........   4   0   HEK
....................................................caaaaacugcaguuacuuuugc.......   1   0   HEK

..................................................agcaaaaacugcaguuacuuuu.........   1   0   j17
```

Star **Mature**

aacuauagguuggugcaaaaguaauugcaguuuuugccauugaaaacaauagcaaaaacugcaguuacuuuugcaccaauu			
....caaaaacugcaguuacuuuu.........	1	0	j17
....caaaaacugcaguuacuuuug........	4	0	j17
....caaaaacugcaguuacuuuugc.......	4	0	j17
....aaaaacugcaguuacuuuug........	4	0	j17
....aaaaacugcaguuacuuuugc.......	1	0	j17
................aaaaguaauugcaguuuuu..	1	0	j15
................aaaaguaauugcaguuuuug...	5	0	j15
....caaaaacugcaguuacuu...........	1	0	j15
....caaaaacugcaguuacuuu..........	28	0	j15
....caaaaacugcaguuacuuuu.........	55	0	j15
....caaaaacugcaguuacuuuug........	246	0	j15
....caaaaacugcaguuacuuuugc.......	20	0	j15
....caaaaacugcaguuacuuuugcacca...	1	0	j15
....aaaaacugcaguuacuuu..........	3	0	j15
....aaaaacugcaguuacuuuu.........	24	0	j15
....aaaaacugcaguuacuuuug........	107	0	j15
....aaaaacugcaguuacuuuugc.......	2	0	j15
....aaaaacugcaguuacuuuugca......	14	0	j15
....aaaacugcaguuacuuuug........	5	0	j15
....aaaacugcaguuacuuuugca......	3	0	j15
....gcaaaaacugcaguuacuuuug........	1	0	la2
....agcaaaaacugcaguuacuuuu.........	2	0	j12
....caaaaacugcaguuacuuuug........	1	0	j12
....aaaaacugcaguuacuuuug........	1	0	j12
....aaaacugcaguuacuuuug........	1	0	j12
....uagcaaaaacugcaguuacuuuu.........	2	0	va4
....agcaaaaacugcaguuacuuuu.........	4	0	va4
....uagcaaaaacugcaguuacuuu..........	1	0	va3
....agcaaaaacugcaguuacuuuu.........	2	0	va3
.....uagguuggugcaaaaguaau..	4	0	ya4
....gcaaaaacugcaguuacuuuug........	2	0	ya4
....caaaaacugcaguuacuuuu.........	10	0	ya4
....caaaaacugcaguuacuuuug........	1	0	ya4
....aaaaacugcaguuacuuuug........	4	0	ya4
................aaaaguaauugcaguuuuugcc...	1	0	SW4
....agcaaaaacugcaguuacuuuu.........	1	0	SW4
....aaaacugcaguuacuuuu.........	2	0	SW4
................aaaaguaauugcaguuuuu..	1	0	ku1
................aaaaguaauugcaguuuuug...	3	0	ku1
....uagcaaaaacugcaguuacuuu..........	1	0	ku1
....agcaaaaacugcaguuacuuu..........	1	0	ku1
....gcaaaaacugcaguuacuuu..........	1	0	ku1
....caaaaacugcaguuacuuuug........	2	0	ku1
....aaaaacugcaguuacuuuu.........	1	0	ku1
................aaaaguaauugcaguuuuu..	1	0	j23
.................aaaguaauugcaguuuuug...	1	0	j23
....uagcaaaaacugcaguuacuu...........	2	0	j23
....uagcaaaaacugcaguuacuuu..........	1	0	j23
....uagcaaaaacugcaguuacuuuu.........	1	0	j23
....agcaaaaacugcaguuacuu...........	1	0	j23
....agcaaaaacugcaguuacuuu..........	3	0	j23
....agcaaaaacugcaguuacuuuu.........	14	0	j23
....gcaaaaacugcaguuacuuuug........	1	0	j23
....caaaaacugcaguuacuuuu.........	6	0	j23
....caaaaacugcaguuacuuuug........	34	0	j23
....caaaaacugcaguuacuuuugc.......	11	0	j23
....aaaaacugcaguuacuuuu.........	2	0	j23
....aaaaacugcaguuacuuuug........	17	0	j23
....aaaaacugcaguuacuuuugc.......	5	0	j23
....aaaaacugcaguuacuuuugca......	4	0	j23
....aaaacugcaguuacuuuugc.......	1	0	j23

```
                Star                              Mature
aacuauagguuggugcaaaaguaauugcaguuuuugccauugaaaacaauagcaaaaacugcaguuacuuuugcaccaauu

.................................................uagcaaaaacugcaguuacuu...........  1   0   s05
.................................................uagcaaaaacugcaguuacuuu..........  1   0   s05
..................................................agcaaaaacugcaguuacuuu..........  2   0   s05

..................................................agcaaaaacugcaguuacuuu..........  1   0   j24

..................................................agcaaaaacugcaguuacuuu..........  2   0   k11
....................................................caaaaacugcaguuacuuuug........  1   0   k11
....................................................caaaaacugcaguuacuuuugc.......  2   0   k11
.....................................................aaaaacugcaguuacuuuu.........  1   0   k11

.................................................uagcaaaaacugcaguuacuuu..........  1   0   s09
..................................................agcaaaaacugcaguuacuuuu.........  1   0   s09

....auagguuggugcaaaagua..........................................................  1   0   NL2
...............caaaaguaauugcaguuuu...............................................  1   0   NL2
................aaaaguaauugcaguuuu...............................................  2   0   NL2
.................aaaguaauugcaguuuuu..............................................  1   0   NL2
....................................................caaaaacugcaguuacuuuu.........  18  0   NL2
....................................................caaaaacugcaguuacuuuug........  10  0   NL2
....................................................caaaaacugcaguuacuuuugc.......  33  0   NL2
.....................................................aaaaacugcaguuacuuuu.........  13  0   NL2
.....................................................aaaaacugcaguuacuuuug........  14  0   NL2
.....................................................aaaaacugcaguuacuuuugc.......  10  0   NL2
.....................................................aaaaacugcaguuacuuuugca......  1   0   NL2
......................................................aaaacugcaguuacuuuugc.......  1   0   NL2

....................................................caaaaacugcaguuacuuu..........  1   0   k10
....................................................caaaaacugcaguuacuuuu.........  1   0   k10
....................................................caaaaacugcaguuacuuuug........  3   0   k10
....................................................caaaaacugcaguuacuuuugc.......  5   0   k10

.................aaaguaauugcaguuuuu..............................................  2   0   j13
.................aaaguaauugcaguuuuug.............................................  1   0   j13
.................................................uagcaaaaacugcaguuacuuu..........  1   0   j13
..................................................agcaaaaacugcaguuacuuu..........  3   0   j13
..................................................agcaaaaacugcaguuacuuuu.........  2   0   j13
...................................................gcaaaaacugcaguuacuuuug........  1   0   j13
.....................................................aaaaacugcaguuacuuuug........  1   0   j13

...............caaaaguaauugcaguuuuu..............................................  1   0   j19
.................................................uagcaaaaacugcaguuacuuu..........  1   0   j19
..................................................agcaaaaacugcaguuacuuuu.........  2   0   j19
....................................................caaaaacugcaguuacuu...........  2   0   j19
....................................................caaaaacugcaguuacuuuu.........  4   0   j19
....................................................caaaaacugcaguuacuuuug........  42  0   j19
....................................................caaaaacugcaguuacuuuugc.......  2   0   j19
.....................................................aaaaacugcaguuacuuuu.........  1   0   j19
.....................................................aaaaacugcaguuacuuuug........  6   0   j19

..................................................agcaaaaacugcaguuacuuu..........  1   0   j03

....................................................caaaaacugcaguuacuuuug........  1   0   j25
.....................................................aaaaacugcaguuacuuuug........  1   0   j25

.................................................uagcaaaaacugcaguuacuuu..........  2   0   k01
....................................................caaaaacugcaguuacuuuug........  4   0   k01
....................................................caaaaacugcaguuacuuuugc.......  4   0   k01
.....................................................aaaaacugcaguuacuuuug........  2   0   k01

................aaaaguaauugcaguuuuu..............................................  2   0   k04
....................................................caaaaacugcaguuacuuuugc.......  3   0   k04
.....................................................aaaaacugcaguuacuuuug........  1   0   k04
.....................................................aaaaacugcaguuacuuuugca......  1   0   k04

.................................................uagcaaaaacugcaguuacuuu..........  1   0   s01
..................................................agcaaaaacugcaguuacuuuu.........  2   0   s01
..................................................agcaaaaacugcaguuacuuuug........  1   0   s01
.....................................................aaaaacugcaguuacuuu..........  1   0   s01
```

Star **Mature**

aacuauagguuggugcaaaaguaauugcaguuuuugccauugaaaacaauagcaaaaacugcaguuacuuuugcaccaauu

....agcaaaaacugcaguuacuuu..........	1	0	per
....caaaaacugcaguuacuuuug........	1	0	per
.....uagguuggugcaaaaguaauug..	1	0	ku2
....uagcaaaaacugcaguuacuuu..........	2	0	ku2
....agcaaaaacugcaguuacuuu..........	1	0	ku2
....agcaaaaacugcaguuacuuuu.........	1	0	ku2
...............caaaaguaauugcaguuuuugc..	1	0	U20
................aaaaguaauugcaguuuuug...	1	0	U20
................aaaaguaauugcaguuuuugc..	1	0	U20
.................aaaguaauugcaguuuuugcca..	2	0	U20
..................aaguaauugcaguuuuugccau...	3	0	U20
....uagcaaaaacugcaguua..............	1	0	U20
....uagcaaaaacugcaguuac.............	2	0	U20
....uagcaaaaacugcaguuacuu...........	3	0	U20
....uagcaaaaacugcaguuacuuu..........	2	0	U20
....agcaaaaacugcaguuacuu...........	1	0	U20
....agcaaaaacugcaguuacuuuu.........	3	0	U20
....gcaaaaacugcaguuacuuuug........	2	0	U20
....caaaaacugcaguuacuuuugc.......	1	0	U20
...............caaaaguaauugcaguuuuug...	1	0	j21
...............caaaaguaauugcaguuuuugc..	3	0	j21
................aaaaguaauugcaguuuuug...	3	0	j21
....uagcaaaaacugcaguuacuuu..........	2	0	j21
....agcaaaaacugcaguuacuuuu.........	1	0	j21
....caaaaacugcaguuacuu...........	1	0	j21
....caaaaacugcaguuacuuuu.........	2	0	j21
....caaaaacugcaguuacuuuug........	38	0	j21
....caaaaacugcaguuacuuuugc.......	37	0	j21
....aaaaacugcaguuacuuuu.........	1	0	j21
....aaaaacugcaguuacuuuug........	8	0	j21
....aaaaacugcaguuacuuuugc.......	11	0	j21
....aaaaacugcaguuacuuuugca......	5	0	j21
....aaaacugcaguuacuuuugca......	1	0	j21
....aaaacugcaguuacuuuugcac.....	1	0	j21
....caaaaacugcaguuacuuuugc.......	1	0	s11
....agcaaaaacugcaguuacuu...........	1	0	j07
................aaaaguaauugcaguuuuug...	1	0	k02
....caaaaacugcaguuacuu...........	1	0	k02
....caaaaacugcaguuacuuuug........	3	0	k02
....caaaaacugcaguuacuuuugc.......	4	0	k02
....aaaaacugcaguuacuuuugc.......	1	0	k02
....uagcaaaaacugcaguuacuu...........	2	0	j14
....agcaaaaacugcaguuacuuu..........	2	0	j14
....gcaaaaacugcaguuacuuuug........	1	0	j14
....caaaaacugcaguuacuuuug........	2	0	j14
....caaaaacugcaguuacuuuugc.......	1	0	j14
....aaaaacugcaguuacuuuugc.......	1	0	j14
....auagguuggugcaaaaguaauu...	1	0	j28
.....uagguuggugcaaaaguaauu...	1	0	j28
................aaaaguaauugcaguuuu...	2	0	j28
................aaaaguaauugcaguuuuug...	1	0	j28
................aaaaguaauugcaguuuuugcc...	2	0	j28
....aaaaacugcaguuacuuuu.........	2	0	j28
....aaaaacugcaguuacuuuug........	1	0	j28
....agcaaaaacugcaguuacuu...........	2	0	s08
....agcaaaaacugcaguuacuuuu.........	1	0	s08
...............caaaaguaauugcaguuuuugc..	1	0	DLD
....uagcaaaaacugcaguuacuu...........	3	0	DLD

Star **Mature**

aacuauagguuggugcaaaaguaauugcaguuuuugccauugaaaacaauagcaaaaacugcaguuacuuuugcaccaauu

....auagguuggugcaaaaguaau..	1	0	k03
....caaaaacugcaguuacuuuug........	3	0	k03
....caaaaacugcaguuacuuuugc.......	1	0	k03
....aaaaacugcaguuacuuuugc.......	1	0	k03
...............caaaaguaauugcaguuuuu..	1	0	ya2
................aaaaguaauugcaguuuu...	1	0	ya2
....agcaaaaacugcaguuacuuuu.........	3	0	ya2
....gcaaaaacugcaguuacuuuu.........	1	0	ya2
....caaaaacugcaguuacuuuu.........	9	0	ya2
....caaaaacugcaguuacuuuug........	3	0	ya2
....caaaaacugcaguuacuuuugc.......	1	0	ya2
....aaaaacugcaguuacuuuug........	1	0	ya2
...............caaaaguaauugcaguuu..	1	0	NL3
...............caaaaguaauugcaguuuu...	1	0	NL3
................aaaaguaauugcaguuuu...	3	0	NL3
....caaaaacugcaguuacuuu..........	5	0	NL3
....caaaaacugcaguuacuuuu.........	15	0	NL3
....caaaaacugcaguuacuuuug........	17	0	NL3
....caaaaacugcaguuacuuuugc.......	27	0	NL3
....aaaaacugcaguuacuuu..........	2	0	NL3
....aaaaacugcaguuacuuuu.........	2	0	NL3
....aaaaacugcaguuacuuuug........	5	0	NL3
....aaaaacugcaguuacuuuugc.......	9	0	NL3
....aaaaacugcaguuacuuuugca......	1	0	NL3
....aaaacugcaguuacuuuugc.......	2	0	NL3
....caaaaacugcaguuacuuuu.........	1	0	k06
....caaaaacugcaguuacuuuug........	6	0	k06
....caaaaacugcaguuacuuuugc.......	3	0	k06
....aaaaacugcaguuacuuuug........	1	0	k06
....aaaaacugcaguuacuuuugc.......	1	0	k06
....aaacugcaguuacuuuug........	1	0	k06
....caaaaacugcaguuacuuuugc.......	6	0	sh2
....aaaaacugcaguuacuuuugca......	2	0	sh2
....agcaaaaacugcaguuacuuuu.........	1	0	j29
....gcaaaaacugcaguuacuuuug........	1	0	j29
....caaaaacugcaguuacuuuug........	2	0	j01
.................aaaguaauugcaguuuuug...	2	0	MCF
....uagcaaaaacugcaguuacuu...........	1	0	MCF
....caaaaacugcaguuacuuuug........	1	0	MCF
....caaaaacugcaguuacuuuugc.......	1	0	MCF
....caaaaacugcaguuacuuuu.........	1	0	NL1
....caaaaacugcaguuacuuuug........	6	0	NL1
....caaaaacugcaguuacuuuugc.......	9	0	NL1
....aaaaacugcaguuacuuuu.........	4	0	NL1
....aaaaacugcaguuacuuuug........	2	0	NL1
....aaaaacugcaguuacuuuugc.......	2	0	NL1
....aaaaacugcaguuacuuuugca......	1	0	NL1
...............caaaaguaauugcaguuuuu..	1	0	MBM
...............caaaaguaauugcaguuuuug...	1	0	MBM
...............caaaaguaauugcaguuuuugc..	16	0	MBM
................aaaaguaauugcaguuuuug...	9	0	MBM
................aaaaguaauugcaguuuuugc..	24	0	MBM
...................aguaauugcaguuuuugc..	4	0	MBM
....agcaaaaacugcaguuacuuu..........	2	0	MBM
....caaaaacugcaguuacuuuug........	2	0	MBM
....caaaaacugcaguuacuuuugc.......	21	0	MBM
....aaaaacugcaguuacuuuu.........	1	0	MBM
....aaaaacugcaguuacuuuug........	1	0	MBM
....aaaaacugcaguuacuuuugc.......	2	0	MBM
....aaaaacugcaguuacuuuugca......	2	0	MBM

```
                Star                                Mature
aacuauagguuggugcaaaaguaauugcaguuuuugccauugaaaacaauagcaaaaacugcaguuacuuuugcaccaauu
......................................................aaaacugcaguuacuuuugc.......    3    0    MBM
......................................................aaaacugcaguuacuuuugcac.....    1    0    MBM

....................................................caaaaacugcaguuacuuuu.........    1    0    k07
....................................................caaaaacugcaguuacuuuug........    1    0    k07

....................................................caaaaacugcaguuacuuuugc.......    9    0    li1
....................................................caaaaacugcaguuacuuuugca......    1    0    li1

.................................................uagcaaaaacugcaguuacu............    1    0    k13
..................................................agcaaaaacugcaguuacuuuu.........    1    0    k13
....................................................caaaaacugcaguuacuuuug........    3    0    k13
....................................................caaaaacugcaguuacuuuugc.......    5    0    k13
.....................................................aaaaacugcaguuacuuuu.........    1    0    k13
.....................................................aaaaacugcaguuacuuuug........    2    0    k13

...............caaaaguaauugcaguuuuu..............................................    1    0    j20
.................................................uagcaaaaacugcaguuacuu...........    1    0    j20
.................................................uagcaaaaacugcaguuacuuu..........    1    0    j20
..................................................agcaaaaacugcaguuacuuu..........    1    0    j20
..................................................agcaaaaacugcaguuacuuuu.........    3    0    j20
.....................................................aaaaacugcaguuacuuuug........    1    0    j20

..................aaguaauugcaguuuuugcca..........................................    1    0    A54

....................................................caaaaacugcaguuacuuuug........    18   0    j30
....................................................caaaaacugcaguuacuuuugc.......    10   0    j30
.....................................................aaaaacugcaguuacuuuug........    8    0    j30
.....................................................aaaaacugcaguuacuuuugc.......    3    0    j30
.....................................................aaaaacugcaguuacuuuugca......    2    0    j30

....................................................caaaaacugcaguuacuuuu.........    2    0    k12
....................................................caaaaacugcaguuacuuuug........    3    0    k12
....................................................caaaaacugcaguuacuuuugc.......    1    0    k12
.....................................................aaaaacugcaguuacuuuu.........    1    0    k12
.....................................................aaaaacugcaguuacuuuug........    1    0    k12
.....................................................aaaaacugcaguuacuuuugc.......    1    0    k12
```

Provisional ID	:	hsa_can_2
Score total	:	-4.4
Score for star read(s)	:	-1.3
Score for read counts	:	-4.9
Score for mfe	:	2.3
Score for randfold	:	
Score for cons. seed	:	-0.6
Total read count	:	2
Mature read count	:	2
Loop read count	:	0
Star read count	:	0

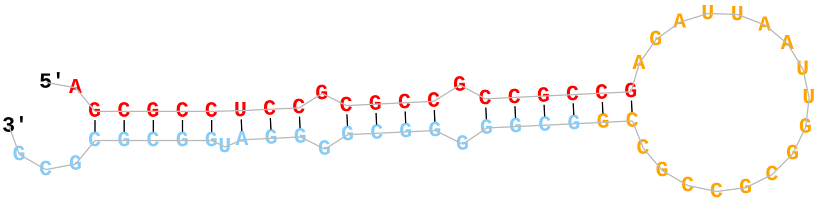

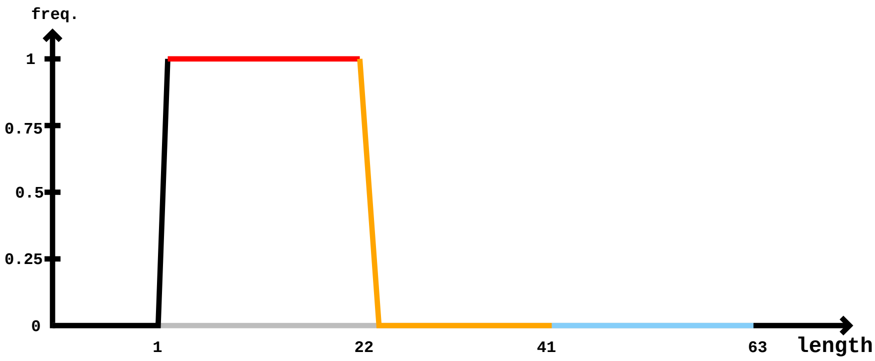

Mature **Star**

```
5'- ccgccgguca gcgccuccgcgccgccgccgagauuaauuggcgccgccggcgggggcggggauggcgcgcgaccuggggc   -3'    exp
    ((.(((((((.((((((((.((((.((((((.................)))))).)))).))).))))).))))))))).      reads      mm         sample
    ..........agcgccuccgcgccgccgccg.................................................      1          0          HEK

    ..........agcgccuccgcgccgccgccg.................................................      1          0          la2
```

Provisional ID	:	hsa_can_13
Score total	:	1812.5
Score for star read(s)	:	3.9
Score for read counts	:	1803.4
Score for mfe	:	2.3
Score for randfold	:	
Score for cons. seed	:	3
Total read count	:	3549
Mature read count	:	3511
Loop read count	:	0
Star read count	:	38

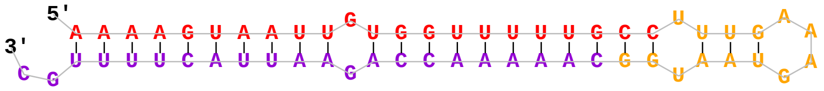

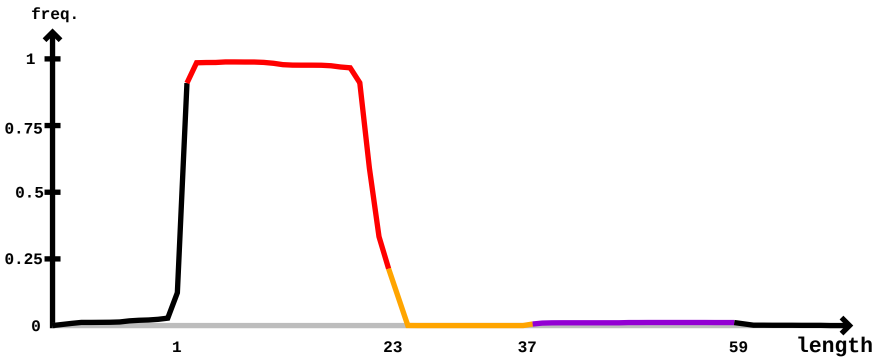

Mature Star

```
5'- uuagguuggugcaaaaguaauugugguuuuugccuuugaaaguaauggcaaaaccagaauuacuuuugcaccaaccuagu  -3'  obs
    uuagguuggugcaaaaguaauugugguuuuugccuuugaaaguaauggcaaaaccagaauuacuuuugcaccaaccuagu       exp
    uuagguuggugcaaaaguaauugugguuuuugccuuugaaaguaauggcaaaaccagaauuacuuuugcaccaaccuagu       known
    ((((((((((((((((((((((.(((((((((((.(((....))).))))))))))).))))))))))))))))))))))).    reads   mm   sample
    .uagguuggugcaaaaguaauu..........................................................    2       0    ya1
    ...........caaaaguaauugugguuuu..................................................    5       0    ya1
    ...........caaaaguaauugugguuuuu.................................................    22      0    ya1
    ...........caaaaguaauugugguuuuug................................................    1       0    ya1
    ............aaaaguaauugugguuuu..................................................    16      0    ya1
    ............aaaaguaauugugguuuuu.................................................    111     0    ya1
    ............aaaaguaauugugguuuuug................................................    8       0    ya1
    .............aaaguaauugugguuuuu.................................................    19      0    ya1

    ............aaaaguaauugugguuuuug................................................    1       0    s04

    uuagguuggugcaaaaguaauug.........................................................    1       0    j26
    ............aaaaguaauugugguuuuug................................................    1       0    j26

    ......uggugcaaaaguaauugugguu....................................................    1       0    va1
    ...........caaaaguaauugugguuuuugc...............................................    1       0    va1
    ............aaaaguaauugugguuuuu.................................................    2       0    va1
    ............aaaaguaauugugguuuuug................................................    25      0    va1
    ............aaaaguaauugugguuuuugc...............................................    55      0    va1
    ............aaaaguaauugugguuuuugcc..............................................    35      0    va1
    .............aaaguaauugugguuuuug................................................    2       0    va1
    .............aaaguaauugugguuuuugc...............................................    1       0    va1
    .............aaaguaauugugguuuuugcc..............................................    1       0    va1

    .....uuggugcaaaaguaauugugguu....................................................    1       0    ku5
    ......uggugcaaaaguaauuguggu.....................................................    1       0    ku5
    ........gugcaaaaguaauugugguu....................................................    1       0    ku5
    ...........caaaaguaauugugguuuuug................................................    1       0    ku5
    ............aaaaguaauugugguuuuug................................................    3       0    ku5

    ...........caaaaguaauugugguuuuu.................................................    2       0    k05
    ...........caaaaguaauugugguuuuug................................................    2       0    k05
```

Mature **Star**

```
uuagguuggugcaaaaguaauugugguuuuugccuuugaaaguaauggcaaaaaccagaauuacuuuugcaccaaccuagu
...........caaaaguaauugugguuuuugc................................................    1    0    k05
............aaaaguaauugugguuuuug.................................................    3    0    k05
............aaaaguaauugugguuuuugc................................................    1    0    k05
.............aaaguaauugugguuuuug.................................................    1    0    k05

uuagguuggugcaaaaguaauu...........................................................    1    0    s06
...........caaaaguaauugugguuuuug.................................................    1    0    s06
............aaaaguaauugugguuuuugc................................................    1    0    s06
............aaaaguaauugugguuuuugcc...............................................    2    0    s06

uuagguuggugcaaaaguaauu...........................................................    1    0    HeL
............aaaaguaauugugguuuuugc................................................    5    0    HeL
............aaaaguaauugugguuuuugcc...............................................    49   0    HeL
.............aaaguaauugugguuuuugc................................................    1    0    HeL
.............aaaguaauugugguuuuugcc...............................................    1    0    HeL
................guaauugugguuuuugcc...............................................    5    0    HeL
................................................caaaaaccagaauuacuuuugc...........    1    0    HeL

........gugcaaaaguaauugugguu.....................................................    1    0    ku4
............aaaaguaauugugguuuuu..................................................    5    0    ku4
............aaaaguaauugugguuuuug.................................................    11   0    ku4

............aaaaguaauugugguuuuugcc...............................................    1    0    s07

............aaaaguaauugugguuuuug.................................................    2    0    s10
............aaaaguaauugugguuuuugc................................................    1    0    s10

...........caaaaguaauugugguuuuu..................................................    2    0    k08
...........caaaaguaauugugguuuuug.................................................    1    0    k08
............aaaaguaauugugguuuuu..................................................    1    0    k08
............aaaaguaauugugguuuuug.................................................    1    0    k08
.............aaaguaauugugguuuuugcc...............................................    1    0    k08

............aaaaguaauugugguuuu...................................................    1    0    ta3
............aaaaguaauugugguuuuug.................................................    7    0    ta3
............aaaaguaauugugguuuuugc................................................    1    0    ta3
.............aaaguaauugugguuuuug.................................................    1    0    ta3

...........caaaaguaauugugguuuuugc................................................    1    0    j10
............aaaaguaauugugguuuuu..................................................    2    0    j10
............aaaaguaauugugguuuuug.................................................    6    0    j10
............aaaaguaauugugguuuuugc................................................    5    0    j10

............aaaaguaauugugguuuuug.................................................    3    0    j04

...........caaaaguaauugugguuuuu..................................................    2    0    k14
...........caaaaguaauugugguuuuug.................................................    4    0    k14
............aaaaguaauugugguuuuu..................................................    2    0    k14
............aaaaguaauugugguuuuug.................................................    4    0    k14
.............aaaguaauugugguuuuug.................................................    1    0    k14

.uagguuggugcaaaaguaauug..........................................................    1    0    va2
...........caaaaguaauugugguuuuu..................................................    1    0    va2
...........caaaaguaauugugguuuuugc................................................    1    0    va2
............aaaaguaauugugguuuuu..................................................    1    0    va2
............aaaaguaauugugguuuuug.................................................    4    0    va2
............aaaaguaauugugguuuuugc................................................    4    0    va2
.............aaaguaauugugguuuuugc................................................    1    0    va2

.uagguuggugcaaaaguaauu...........................................................    1    0    j02
.uagguuggugcaaaaguaauug..........................................................    1    0    j02
............aaaaguaauugugguuuu...................................................    1    0    j02
............aaaaguaauugugguuuuu..................................................    5    0    j02
............aaaaguaauugugguuuuug.................................................    22   0    j02
.............aaaguaauugugguuuuug.................................................    1    0    j02

.......ggugcaaaaguaauugugguuu....................................................    1    0    j27
...........caaaaguaauugugguuuuu..................................................    1    0    j27
...........caaaaguaauugugguuuuug.................................................    4    0    j27
............aaaaguaauugugguuuu...................................................    1    0    j27
```

```
            Mature                              Star
uuagguuggugcaaaaguaauugugguuuuugccuuugaaaguaauggcaaaaaccagaauuacuuuugcaccaaccuagu
............aaaaguaauugugguuuuu..................................................   2    0    j27
............aaaaguaauugugguuuuug.................................................   13   0    j27
.............aaaguaauugugguuuuugc................................................   1    0    j27

uuagguuggugcaaaaguaauug..........................................................   1    0    j31
.....uuggugcaaaaguaauuguggu......................................................   1    0    j31
......uggugcaaaaguaauugugguu.....................................................   1    0    j31
...........caaaaguaauugugguuuuu..................................................   1    0    j31
...........caaaaguaauugugguuuuug.................................................   2    0    j31
............aaaaguaauugugguuuuug.................................................   1    0    j31
............aaaaguaauugugguuuuugcc...............................................   1    0    j31

............aaaaguaauugugguuuuugc................................................   2    0    s03

uuagguuggugcaaaaguaauu...........................................................   1    0    H52
...........caaaaguaauugugguuuuugc................................................   1    0    H52
............aaaaguaauugugguuuuu..................................................   2    0    H52
............aaaaguaauugugguuuuugc................................................   4    0    H52
............aaaaguaauugugguuuuugcc...............................................   16   0    H52
................................................caaaaaccagaauuacuuuugc...........   2    0    H52

...........caaaaguaauugugguuuuu..................................................   1    0    j22
...........caaaaguaauugugguuuuug.................................................   1    0    j22
...........caaaaguaauugugguuuuugc................................................   2    0    j22
............aaaaguaauugugguuuuu..................................................   1    0    j22
............aaaaguaauugugguuuuug.................................................   33   0    j22
............aaaaguaauugugguuuuugc................................................   1    0    j22
............aaaaguaauugugguuuuugcc...............................................   2    0    j22
.............aaaguaauugugguuuuug.................................................   3    0    j22
...................................................aaaccagaauuacuuuugcacc........   1    0    j22

.uagguuggugcaaaaguaauug..........................................................   1    0    k09
.....uuggugcaaaaguaauugugg.......................................................   1    0    k09
.........ugcaaaaguaauugugguuuuu..................................................   1    0    k09
...........caaaaguaauugugguuuuu..................................................   1    0    k09
...........caaaaguaauugugguuuuug.................................................   2    0    k09
............aaaaguaauugugguuuuu..................................................   2    0    k09
............aaaaguaauugugguuuuug.................................................   4    0    k09
............aaaaguaauugugguuuuugc................................................   1    0    k09
............aaaaguaauugugguuuuugcc...............................................   1    0    k09

...........caaaaguaauugugguuuuug.................................................   3    0    B14
...........caaaaguaauugugguuuuugc................................................   1    0    B14
............aaaaguaauugugguuuuugc................................................   6    0    B14
............aaaaguaauugugguuuuugcc...............................................   15   0    B14
.............aaaguaauugugguuuuugcc...............................................   1    0    B14

...........caaaaguaauugugguuuuug.................................................   1    0    j16
............aaaaguaauugugguuuu...................................................   1    0    j16
............aaaaguaauugugguuuuu..................................................   12   0    j16
............aaaaguaauugugguuuuug.................................................   25   0    j16
.............aaaguaauugugguuuuugc................................................   1    0    j16
.................................................aaaaaccagaauuacuuuugca..........   1    0    j16

...........caaaaguaauugugguuuuu..................................................   3    0    j18
...........caaaaguaauugugguuuuug.................................................   1    0    j18
............aaaaguaauugugguuuuug.................................................   1    0    j18

...........caaaaguaauugugguuuuu..................................................   2    0    j09
............aaaaguaauugugguuuuu..................................................   4    0    j09
............aaaaguaauugugguuuuug.................................................   9    0    j09
.............aaaguaauugugguuuuug.................................................   1    0    j09

uuagguuggugcaaaaguaau............................................................   2    0    ku3
......uggugcaaaaguaauuguggu......................................................   1    0    ku3
...........caaaaguaauugugguuuuug.................................................   1    0    ku3
............aaaaguaauugugguuuuug.................................................   6    0    ku3

uuagguuggugcaaaaguaa.............................................................   1    0    ku6
...........caaaaguaauugugguuuu...................................................   1    0    ku6
```

```
            Mature                   Star
uuagguuggugcaaaaguaauugugguuuuugccuuugaaaguaauggcaaaaaccagaauuacuuuugcaccaaccuagu
...........caaaaguaauugugguuuuu..................................................    1    0    ku6
............aaaaguaauugugguuuuug.................................................    2    0    ku6

............aaaaguaauugugguuuuu..................................................    4    0    j11
............aaaaguaauugugguuuuug.................................................    7    0    j11
.............aaaguaauugugguuuuug.................................................    1    0    j11

............aaaaguaauugugguuuuug.................................................    4    0    s12
.............aaaguaauugugguuuuug.................................................    2    0    s12

uuagguuggugcaaaaguaauu...........................................................    1    0    ya3
.uagguuggugcaaaaguaauu...........................................................    1    0    ya3
..........gcaaaaguaauugugguuuuu..................................................    2    0    ya3
...........caaaaguaauugugguuuu...................................................    9    0    ya3
...........caaaaguaauugugguuuuu..................................................    22   0    ya3
............aaaaguaauugugguuuu...................................................    44   0    ya3
............aaaaguaauugugguuuuu..................................................    238  0    ya3
............aaaaguaauugugguuuuug.................................................    27   0    ya3
............aaaaguaauugugguuuuugc................................................    1    0    ya3
.............aaaguaauugugguuuuu..................................................    45   0    ya3
.............aaaguaauugugguuuuug.................................................    8    0    ya3
................................................caaaaaccagaauuacuuuugca..........    1    0    ya3
.................................................aaaaaccagaauuacuuuugca..........    1    0    ya3

...........caaaaguaauugugguuuuu..................................................    1    0    la1
...........caaaaguaauugugguuuuugc................................................    1    0    la1
............aaaaguaauugugguuuu...................................................    1    0    la1
............aaaaguaauugugguuuuu..................................................    1    0    la1
............aaaaguaauugugguuuuug.................................................    3    0    la1
............aaaaguaauugugguuuuugcc...............................................    1    0    la1
.................................................aaaaaccagaauuacuuuugca..........    1    0    la1

......uggugcaaaaguaauugugguu.....................................................    1    0    HEK
...........caaaaguaauugugguuuuugc................................................    7    0    HEK
............aaaaguaauugugguuuuugc................................................    9    0    HEK
............aaaaguaauugugguuuuugcc...............................................    75   0    HEK
.............aaaguaauugugguuuuugcc...............................................    2    0    HEK
..............aaguaauugugguuuuugcc...............................................    1    0    HEK
................................................caaaaaccagaauuacuuuugc...........    2    0    HEK

...........caaaaguaauugugguuuuug.................................................    4    0    j15
...........caaaaguaauugugguuuuugc................................................    1    0    j15
............aaaaguaauugugguuuu...................................................    1    0    j15
............aaaaguaauugugguuuuu..................................................    3    0    j15
............aaaaguaauugugguuuuug.................................................    16   0    j15
.............aaaguaauugugguuuuu..................................................    1    0    j15
.............aaaguaauugugguuuuug.................................................    1    0    j15

uuagguuggugcaaaaguaauu...........................................................    1    0    j17
............aaaaguaauugugguuuu...................................................    1    0    j17
............aaaaguaauugugguuuuu..................................................    8    0    j17
............aaaaguaauugugguuuuug.................................................    8    0    j17

uuagguuggugcaaaagu...............................................................    1    0    la2
............aaaaguaauugugguuuuu..................................................    1    0    la2
............aaaaguaauugugguuuuug.................................................    3    0    la2
............aaaaguaauugugguuuuugcc...............................................    6    0    la2
.............aaaguaauugugguuuuugcc...............................................    1    0    la2
.................................................aaaaaccagaauuacuuuugca..........    1    0    la2

......uggugcaaaaguaauugugguu.....................................................    1    0    j12
...........caaaaguaauugugguuuuug.................................................    5    0    j12
............aaaaguaauugugguuuuu..................................................    6    0    j12
............aaaaguaauugugguuuuug.................................................    24   0    j12
............aaaaguaauugugguuuuugc................................................    2    0    j12
............aaaaguaauugugguuuuugcc...............................................    1    0    j12
.............aaaguaauugugguuuuug.................................................    2    0    j12

......uggugcaaaaguaauugugguu.....................................................    1    0    li2
...........caaaaguaauugugguuuuug.................................................    4    0    li2
```

```
            Mature                              Star
uuagguuggugcaaaaguaauugugguuuuugccuuugaaaguaauggcaaaaaccagaauuacuuuugcaccaaccuagu
...........caaaaguaauugugguuuuugc................................................   9     0     li2
............aaaaguaauugugguuuuugc................................................   56    0     li2
............aaaaguaauugugguuuuugcc...............................................   3     0     li2
.............aaaguaauugugguuuuugc................................................   3     0     li2
................................................caaaaaccagaauuacuuuugc...........   2     0     li2

...........caaaaguaauugugguuuuug.................................................   1     0     va4
...........caaaaguaauugugguuuuugc................................................   1     0     va4
............aaaaguaauugugguuuuu..................................................   1     0     va4
............aaaaguaauugugguuuuug.................................................   5     0     va4
............aaaaguaauugugguuuuugc................................................   11    0     va4
............aaaaguaauugugguuuuugcc...............................................   11    0     va4
............aaaaguaauugugguuuuugccu..............................................   1     0     va4

...........caaaaguaauugugguuuuug.................................................   2     0     va3
...........caaaaguaauugugguuuuugc................................................   1     0     va3
............aaaaguaauugugguuuuu..................................................   2     0     va3
............aaaaguaauugugguuuuug.................................................   10    0     va3
............aaaaguaauugugguuuuugc................................................   13    0     va3
............aaaaguaauugugguuuuugcc...............................................   3     0     va3
............aaaaguaauugugguuuuugccu..............................................   1     0     va3
.............aaaguaauugugguuuuug.................................................   2     0     va3
.............aaaguaauugugguuuuugcc...............................................   1     0     va3

uuagguuggugcaaaaguaauu...........................................................   1     0     SW4
...........caaaaguaauugugguuuuu..................................................   1     0     SW4
...........caaaaguaauugugguuuuugc................................................   1     0     SW4
............aaaaguaauugugguuuuu..................................................   1     0     SW4
............aaaaguaauugugguuuuug.................................................   3     0     SW4
............aaaaguaauugugguuuuugc................................................   12    0     SW4
............aaaaguaauugugguuuuugcc...............................................   20    0     SW4
.............aaaguaauugugguuuuugc................................................   1     0     SW4

.uagguuggugcaaaaguaau............................................................   4     0     ya4
...........caaaaguaauugugguuuu...................................................   16    0     ya4
...........caaaaguaauugugguuuuu..................................................   16    0     ya4
...........caaaaguaauugugguuuuug.................................................   4     0     ya4
............aaaaguaauugugguuuu...................................................   33    0     ya4
............aaaaguaauugugguuuuu..................................................   213   0     ya4
............aaaaguaauugugguuuuug.................................................   5     0     ya4
............aaaaguaauugugguuuuugcc...............................................   4     0     ya4
.............aaaguaauugugguuuuu..................................................   43    0     ya4
.............aaaguaauugugguuuuug.................................................   3     0     ya4

uuagguuggugcaaaaguaauu...........................................................   1     0     ku1
...........caaaaguaauugugguuuuu..................................................   1     0     ku1
...........caaaaguaauugugguuuuug.................................................   1     0     ku1
...........caaaaguaauugugguuuuugc................................................   1     0     ku1
............aaaaguaauugugguuuuu..................................................   5     0     ku1
............aaaaguaauugugguuuuug.................................................   38    0     ku1
............aaaaguaauugugguuuuugc................................................   1     0     ku1
.............aaaguaauugugguuuuug.................................................   1     0     ku1

.........ugcaaaaguaauugugguuuuu..................................................   1     0     j23
...........caaaaguaauugugguuuuug.................................................   4     0     j23
............aaaaguaauugugguuuu...................................................   1     0     j23
............aaaaguaauugugguuuuu..................................................   8     0     j23
............aaaaguaauugugguuuuug.................................................   89    0     j23
............aaaaguaauugugguuuuugc................................................   5     0     j23
............aaaaguaauugugguuuuugcc...............................................   13    0     j23
.............aaaguaauugugguuuuug.................................................   11    0     j23
.............aaaguaauugugguuuuugc................................................   1     0     j23
.............aaaguaauugugguuuuugcc...............................................   2     0     j23
.................................................aaaaaccagaauuacuuuugca..........   4     0     j23

............aaaaguaauugugguuuuug.................................................   7     0     s05
............aaaaguaauugugguuuuugc................................................   1     0     s05
.............aaaguaauugugguuuuug.................................................   1     0     s05

............aaaaguaauugugguuuuugc................................................   4     0     ta2
```

```
            Mature                              Star
uuagguuggugcaaaaguaauugugguuuuugccuuugaaaguaauggcaaaaaccagaauuacuuuugcaccaaccuagu
.............aaaguaauugugguuuuug.................................................    1    0    ta2

.......ggugcaaaaguaauugugguuu....................................................    5    0    j24
...........caaaaguaauugugguuuu...................................................    3    0    j24
...........caaaaguaauugugguuuuug.................................................    1    0    j24
............aaaaguaauugugguuuuu..................................................    3    0    j24
............aaaaguaauugugguuuuug.................................................    8    0    j24

uuagguuggugcaaaaguaa.............................................................    1    0    k11
uuagguuggugcaaaaguaau............................................................    1    0    k11
...........caaaaguaauugugguuuuug.................................................    1    0    k11
............aaaaguaauugugguuuuu..................................................    2    0    k11
............aaaaguaauugugguuuuug.................................................    2    0    k11
............aaaaguaauugugguuuuugc................................................    3    0    k11

............aaaaguaauugugguuuu...................................................    1    0    ta1
............aaaaguaauugugguuuuu..................................................    1    0    ta1
............aaaaguaauugugguuuuug.................................................    3    0    ta1
............aaaaguaauugugguuuuugcc...............................................    1    0    ta1
.............aaaguaauugugguuuuug.................................................    1    0    ta1

...........caaaaguaauugugguuuuug.................................................    1    0    s09
............aaaaguaauugugguuuuu..................................................    1    0    s09
............aaaaguaauugugguuuuug.................................................    3    0    s09
.............aaaguaauugugguuuuug.................................................    1    0    s09

uuagguuggugcaaaaguaauu...........................................................    1    0    NL2
...........caaaaguaauugugguuu....................................................    1    0    NL2
...........caaaaguaauugugguuuu...................................................    1    0    NL2
...........caaaaguaauugugguuuuu..................................................    4    0    NL2
............aaaaguaauugugguuuu...................................................    10   0    NL2
............aaaaguaauugugguuuuu..................................................    2    0    NL2
............aaaaguaauugugguuuuug.................................................    2    0    NL2

...........caaaaguaauugugguuuuug.................................................    1    0    j13
...........caaaaguaauugugguuuuugc................................................    1    0    j13
............aaaaguaauugugguuuu...................................................    1    0    j13
............aaaaguaauugugguuuuu..................................................    6    0    j13
............aaaaguaauugugguuuuug.................................................    33   0    j13
............aaaaguaauugugguuuuugc................................................    4    0    j13
............aaaaguaauugugguuuuugcc...............................................    1    0    j13
.............aaaguaauugugguuuuug.................................................    1    0    j13
.............aaaguaauugugguuuuugc................................................    1    0    j13

uuagguuggugcaaaaguaauu...........................................................    2    0    k10
......uggugcaaaaguaauugugguu.....................................................    1    0    k10
...........caaaaguaauugugguuuuu..................................................    1    0    k10
...........caaaaguaauugugguuuuug.................................................    2    0    k10
............aaaaguaauugugguuuuug.................................................    4    0    k10
............aaaaguaauugugguuuuugc................................................    2    0    k10

...........caaaaguaauugugguuuuu..................................................    2    0    j19
...........caaaaguaauugugguuuuug.................................................    1    0    j19
............aaaaguaauugugguuuu...................................................    2    0    j19
............aaaaguaauugugguuuuu..................................................    5    0    j19
............aaaaguaauugugguuuuug.................................................    37   0    j19
............aaaaguaauugugguuuuugc................................................    2    0    j19

............aaaaguaauugugguuuuug.................................................    4    0    j03

............aaaaguaauugugguuuuug.................................................    1    0    k01
............aaaaguaauugugguuuuugc................................................    3    0    k01

uuagguuggugcaaaaguaa.............................................................    1    0    j25
......uggugcaaaaguaauugugguu.....................................................    1    0    j25
.........ugcaaaaguaauugugguu.....................................................    2    0    j25
...........caaaaguaauugugguuuu...................................................    1    0    j25
............aaaaguaauugugguuuuug.................................................    10   0    j25

uuagguuggugcaaaaguaa.............................................................    1    0    per
```

```
            Mature                              Star
uuagguuggugcaaaaguaauugugguuuuugccuuugaaaguaauggcaaaaaccagaauuacuuuugcaccaaccuagu
uuagguuggugcaaaaguaau............................................................    1    0    per
..........gcaaaaguaauugugguuuuug.................................................    1    0    per
...........caaaaguaauugugguuuuug.................................................    1    0    per
............aaaaguaauugugguuuu...................................................    1    0    per
............aaaaguaauugugguuuuug.................................................    8    0    per
.............aaaguaauugugguuuuug.................................................    1    0    per
..........................................................aauuacuuuugcaccaaccua..    2    0    per

..........gcaaaaguaauugugguuuuug.................................................    1    0    s01

...........caaaaguaauugugguuuuu..................................................    2    0    k04
...........caaaaguaauugugguuuuug.................................................    1    0    k04
............aaaaguaauugugguuuu...................................................    1    0    k04
............aaaaguaauugugguuuuug.................................................    5    0    k04
............aaaaguaauugugguuuuugc................................................    1    0    k04

.uagguuggugcaaaaguaauug..........................................................    1    0    ku2
...........caaaaguaauugugguuuuu..................................................    2    0    ku2
............aaaaguaauugugguuuuu..................................................    5    0    ku2
............aaaaguaauugugguuuuug.................................................    16   0    ku2
............aaaaguaauugugguuuuugc................................................    1    0    ku2
............aaaaguaauugugguuuuugcc...............................................    1    0    ku2

......uggugcaaaaguaauuguggu......................................................    2    0    U20
......uggugcaaaaguaauugugguu.....................................................    1    0    U20
........gugcaaaaguaauugugguuuu...................................................    1    0    U20
...........caaaaguaauugugguuuuug.................................................    1    0    U20
...........caaaaguaauugugguuuuugc................................................    18   0    U20
...........caaaaguaauugugguuuuugcc...............................................    1    0    U20
............aaaaguaauugugguuuu...................................................    1    0    U20
............aaaaguaauugugguuuuug.................................................    6    0    U20
............aaaaguaauugugguuuuugc................................................    48   0    U20
............aaaaguaauugugguuuuugcc...............................................    243  0    U20
.............aaaguaauugugguuuuugc................................................    11   0    U20
.............aaaguaauugugguuuuugcc...............................................    24   0    U20
...............aguaauugugguuuuugc................................................    1    0    U20
................................................caaaaaccagaauuacuuuugc...........    9    0    U20
.................................................aaaaaccagaauuacuuuugca..........    2    0    U20
..................................................aaaaccagaauuacuuuugca..........    3    0    U20

...........caaaaguaauugugguuuuu..................................................    1    0    j21
...........caaaaguaauugugguuuuug.................................................    3    0    j21
............aaaaguaauugugguuuuu..................................................    1    0    j21
............aaaaguaauugugguuuuug.................................................    14   0    j21
............aaaaguaauugugguuuuugc................................................    3    0    j21
............aaaaguaauugugguuuuugcc...............................................    2    0    j21
.............aaaguaauugugguuuuug.................................................    3    0    j21
.............aaaguaauugugguuuuugcc...............................................    2    0    j21
..............aaguaauugugguuuuugccuu.............................................    1    0    j21
.................................................aaaaaccagaauuacuuuugca..........    2    0    j21


............aaaaguaauugugguuuuug.................................................    1    0    s11

............aaaaguaauugugguuuuu..................................................    4    0    j07
............aaaaguaauugugguuuuug.................................................    2    0    j07
.............aaaguaauugugguuuuug.................................................    1    0    j07

uuagguuggugcaaaaguaau............................................................    1    0    k02
...........caaaaguaauugugguuuuug.................................................    2    0    k02
............aaaaguaauugugguuuuu..................................................    2    0    k02
............aaaaguaauugugguuuuugc................................................    1    0    k02

...........caaaaguaauugugguuuuug.................................................    5    0    j14
............aaaaguaauugugguuuuug.................................................    9    0    j14
............aaaaguaauugugguuuuugc................................................    5    0    j14
............aaaaguaauugugguuuuugcc...............................................    1    0    j14

uuagguuggugcaaaaguaau............................................................    1    0    j28
uuagguuggugcaaaaguaauu...........................................................    1    0    j28
```

```
                Mature                              Star
uuagguuggugcaaaaguaauugugguuuuugccuuugaaaguaauggcaaaaaccagaauuacuuuugcaccaaccuagu
.uagguuggugcaaaaguaauu...........................................................    1     0     j28
...........caaaaguaauugugguuuuu..................................................    1     0     j28
............aaaaguaauugugguuuuu..................................................    3     0     j28
............aaaaguaauugugguuuuug.................................................    17    0     j28

............aaaaguaauugugguuuuug.................................................    1     0     s08

...........caaaaguaauugugguuuuugc................................................    1     0     DLD
............aaaaguaauugugguuuuu..................................................    2     0     DLD
............aaaaguaauugugguuuuug.................................................    1     0     DLD
............aaaaguaauugugguuuuugc................................................    13    0     DLD
............aaaaguaauugugguuuuugcc...............................................    73    0     DLD
.............aaaguaauugugguuuuugcc...............................................    4     0     DLD
................guaauugugguuuuugcc...............................................    1     0     DLD
................................................caaaaaccagaauuacuuu..............    1     0     DLD
................................................caaaaaccagaauuacuuuugc...........    1     0     DLD

....guuggugcaaaaguaauuguggu......................................................    1     0     k03
............aaaaguaauugugguuuuug.................................................    5     0     k03
............aaaaguaauugugguuuuugc................................................    3     0     k03

uuagguuggugcaaaaguaauu...........................................................    2     0     ya2
...........caaaaguaauugugguuu....................................................    1     0     ya2
...........caaaaguaauugugguuuu...................................................    6     0     ya2
...........caaaaguaauugugguuuuu..................................................    13    0     ya2
...........caaaaguaauugugguuuuug.................................................    2     0     ya2
............aaaaguaauugugguuuu...................................................    24    0     ya2
............aaaaguaauugugguuuuu..................................................    188   0     ya2
............aaaaguaauugugguuuuug.................................................    17    0     ya2
............aaaaguaauugugguuuuugcc...............................................    1     0     ya2
.............aaaguaauugugguuuuu..................................................    43    0     ya2
.............aaaguaauugugguuuuug.................................................    4     0     ya2
..........................................................aauuacuuuugcaccaac.....    1     0     ya2

uuagguuggugcaaaaguaauu...........................................................    1     0     NL3
...........caaaaguaauugugguuu....................................................    1     0     NL3
...........caaaaguaauugugguuuu...................................................    1     0     NL3
...........caaaaguaauugugguuuuu..................................................    4     0     NL3
...........caaaaguaauugugguuuuug.................................................    2     0     NL3
............aaaaguaauugugguuuu...................................................    15    0     NL3
............aaaaguaauugugguuuuu..................................................    6     0     NL3
............aaaaguaauugugguuuuug.................................................    5     0     NL3

...........caaaaguaauugugguuuuug.................................................    1     0     s02
............aaaaguaauugugguuuuugcc...............................................    1     0     s02

...........caaaaguaauugugguuuuug.................................................    3     0     k06
............aaaaguaauugugguuuuug.................................................    2     0     k06

uuagguuggugcaaaaguaa.............................................................    1     0     sh2
...gguuggugcaaaaguaauugu.........................................................    1     0     sh2
.....uuggugcaaaaguaauugugguu.....................................................    1     0     sh2
........gugcaaaaguaauugugguu.....................................................    1     0     sh2
...........caaaaguaauugugguuuuu..................................................    1     0     sh2
...........caaaaguaauugugguuuuugc................................................    1     0     sh2
............aaaaguaauugugguuuuug.................................................    1     0     sh2
............aaaaguaauugugguuuuugc................................................    3     0     sh2
............aaaaguaauugugguuuuugcc...............................................    3     0     sh2
............................................................uuacuuuugcaccaaccua..    1     0     sh2

..........gcaaaaguaauugugguuuuug.................................................    1     0     j29
...........caaaaguaauugugguuuuug.................................................    3     0     j29
............aaaaguaauugugguuuuug.................................................    10    0     j29
............aaaaguaauugugguuuuugc................................................    8     0     j29
............aaaaguaauugugguuuuugcc...............................................    1     0     j29

......uggugcaaaaguaauuguggu......................................................    1     0     MCF
...........caaaaguaauugugguuuuugc................................................    1     0     MCF
............aaaaguaauugugguuuuug.................................................    4     0     MCF
............aaaaguaauugugguuuuugc................................................    11    0     MCF
```

```
            Mature                              Star
uuagguuggugcaaaaguaauugugguuuuugccuuugaaaguaauggcaaaaaccagaauuacuuuugcaccaaccuagu
............aaaaguaauugugguuuuugcc...............................................   42   0   MCF
.............aaaguaauugugguuuuugcc...............................................   1    0   MCF

.......ggugcaaaaguaauugugguu.....................................................   1    0   j01
.........ugcaaaaguaauugugguuuu...................................................   3    0   j01
...........caaaaguaauugugguuuuug.................................................   1    0   j01
............aaaaguaauugugguuuuug.................................................   5    0   j01

...........caaaaguaauugugguuuuugc................................................   1    0   MBM
............aaaaguaauugugguuuuug.................................................   2    0   MBM
............aaaaguaauugugguuuuugc................................................   14   0   MBM
............aaaaguaauugugguuuuugcc...............................................   33   0   MBM
.............aaaguaauugugguuuuugc................................................   1    0   MBM
.............aaaguaauugugguuuuugcc...............................................   1    0   MBM
................guaauugugguuuuugcc...............................................   1    0   MBM

uuagguuggugcaaaaguaau............................................................   2    0   NL1
............aaaaguaauugugguuuu...................................................   2    0   NL1
............aaaaguaauugugguuuuug.................................................   2    0   NL1
............aaaaguaauugugguuuuugc................................................   1    0   NL1

.......ggugcaaaaguaauuguggu......................................................   1    0   k07
.........ugcaaaaguaauugugguuuu...................................................   1    0   k07
...........caaaaguaauugugguuuuug.................................................   9    0   k07
............aaaaguaauugugguuuuug.................................................   3    0   k07
............aaaaguaauugugguuuuugc................................................   1    0   k07

.........ugcaaaaguaauugugguuuu...................................................   1    0   sh1
...........caaaaguaauugugguuuuug.................................................   6    0   sh1
............aaaaguaauugugguuuuug.................................................   4    0   sh1

......uggugcaaaaguaauuguggu......................................................   1    0   li1
...........caaaaguaauugugguuuuug.................................................   3    0   li1
...........caaaaguaauugugguuuuugc................................................   1    0   li1
............aaaaguaauugugguuuuugc................................................   23   0   li1
............aaaaguaauugugguuuuugcc...............................................   2    0   li1
.............aaaguaauugugguuuuugc................................................   1    0   li1

...........caaaaguaauugugguuuu...................................................   1    0   k13
...........caaaaguaauugugguuuuug.................................................   4    0   k13
............aaaaguaauugugguuuuug.................................................   1    0   k13

...........caaaaguaauugugguuu....................................................   1    0   j20
...........caaaaguaauugugguuuu...................................................   2    0   j20
...........caaaaguaauugugguuuuug.................................................   7    0   j20
............aaaaguaauugugguuuu...................................................   3    0   j20
............aaaaguaauugugguuuuug.................................................   27   0   j20
............aaaaguaauugugguuuuugcc...............................................   1    0   j20
..............aaguaauugugguuuuugc................................................   1    0   j20

..........gcaaaaguaauugugguuuuug.................................................   11   0   A54
............aaaaguaauugugguuuuug.................................................   3    0   A54
............aaaaguaauugugguuuuugc................................................   3    0   A54
............aaaaguaauugugguuuuugcc...............................................   50   0   A54
.............aaaguaauugugguuuuugcc...............................................   5    0   A54
................................................caaaaaccagaauuacuuuugc...........   2    0   A54

......uggugcaaaaguaauuguggu......................................................   1    0   j30
............aaaaguaauugugguuuuug.................................................   2    0   j30
............aaaaguaauugugguuuuugcc...............................................   1    0   j30
.................................................aaaaaccagaauuacuuuugca..........   1    0   j30

...........caaaaguaauugugguuuu...................................................   8    0   k12
...........caaaaguaauugugguuuuug.................................................   12   0   k12
............aaaaguaauugugguuuu...................................................   6    0   k12
............aaaaguaauugugguuuuug.................................................   18   0   k12
............aaaaguaauugugguuuuugc................................................   2    0   k12
............aaaaguaauugugguuuuugcc...............................................   1    0   k12
```

Provisional ID	:	hsa_can_19
Score total	:	3.4
Score for star read(s)	:	-1.3
Score for read counts	:	0
Score for mfe	:	1.7
Score for randfold	:	
Score for cons. seed	:	3
Total read count	:	759
Mature read count	:	254
Loop read count	:	0
Star read count	:	505

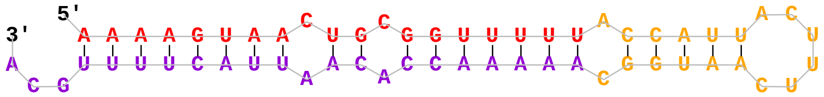

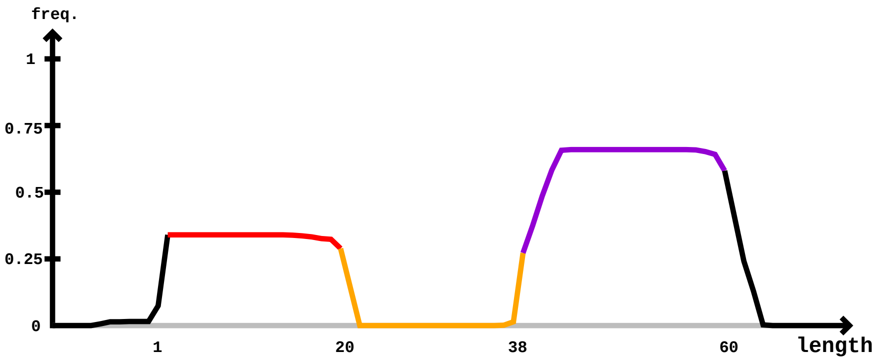

Mature　　　　Star

```
5'- agguuggugcaaaaguaacugcgguuuuuaccauuacuuucaaggcaaaaaccacaauuacuuuugcaccagucuaauag  -3'  obs
    agguuggugcaaaaguaacugcgguuuuuaccauuacuuucaaggcaaaaaccacaauuacuuuugcaccagucuaauag       exp
    agguuggugcaaaaguaacugcgguuuuuaccauuacuuucaaggcaaaaaccacaauuacuuuugcaccagucuaauag       known
    ((..((((((((((((((.((.(((((((.(((((......))))).))))))).)).))))))))))))))..))....      reads   mm   sample
    ..........aaaaguaacugcgguuuu....................................................      2       0    ya1
    ..........aaaaguaacugcgguuuuu...................................................      21      0    ya1
    ..............................................aaaaaccacaauuacuuuug..............      1       0    ya1
    ..............................................aaaaaccacaauuacuuuugca............      4       0    ya1
    ...............................................aaaaccacaauuacuuuugca............      3       0    ya1
    ...............................................aaaaccacaauuacuuuugcac...........      1       0    ya1
    ................................................aaaccacaauuacuuuugca............      1       0    ya1
    .................................................aaccacaauuacuuuugcacc..........      1       0    ya1

    ..............................................aaaaaccacaauuacuuuugca............      1       0    s04

    ..............................................aaaaaccacaauuacuuuugca............      3       0    j26
    ..................................................accacaauuacuuuugcacca.........      1       0    j26

    ..............................................aaaaaccacaauuacuuuugc.............      1       0    va1
    ..............................................aaaaaccacaauuacuuuugca............      6       0    va1
    ..............................................aaaaaccacaauuacuuuugcac...........      1       0    va1
    ...............................................aaaaccacaauuacuuuugcac...........      5       0    va1
    ................................................aaaccacaauuacuuuugcacc..........      1       0    va1

    .........caaaaguaacugcgguuuuu...................................................      1       0    ku5
    ..........aaaaguaacugcgguuuuu...................................................      4       0    ku5
    ..............................................aaaaaccacaauuacuuuugca............      1       0    ku5
    ................................................aaaccacaauuacuuuugcacc..........      1       0    ku5

    ................................................aaaccacaauuacuuuugcacc..........      1       0    k05

    ..............................................aaaaaccacaauuacuuuugca............      1       0    s06
    .................................................aaccacaauuacuuuugcacca.........      1       0    s06
    ..................................................accacaauuacuuuugcacca.........      1       0    s06

    ..............................................aaaaaccacaauuacuuuugc.............      1       0    HeL
```

```
          Mature                                     Star
agguuggugcaaaaguaacugcgguuuuuaccauuacuuucaauggcaaaaaccacaauuacuuuugcaccagucuaauag
...............................................aaaaaccacaauuacuuuugca............   3    0    HeL
................................................aaaaccacaauuacuuuugcac...........   2    0    HeL
.................................................aaaccacaauuacuuuugcac...........   1    0    HeL
.................................................aaaccacaauuacuuuugcacc..........   3    0    HeL
..................................................aaccacaauuacuuuugcacca.........   3    0    HeL
...................................................accacaauuacuuuugcacca.........   5    0    HeL

..........aaaaguaacugcgguuuuu....................................................   5    0    ku4

.................................................aaaccacaauuacuuuugcacc..........   1    0    s07

...............................................aaaaaccacaauuacuuuugca............   4    0    s10
.................................................aaaccacaauuacuuuugcac...........   1    0    s10

..........aaaaguaacugcgguuuuu....................................................   1    0    ta3
...............................................aaaaaccacaauuacuuuugca............   4    0    ta3
................................................aaaaccacaauuacuuuugc.............   1    0    ta3
................................................aaaaccacaauuacuuuugcac...........   1    0    ta3
.................................................aaaccacaauuacuuuugcac...........   1    0    ta3
.................................................aaaccacaauuacuuuugcacc..........   1    0    ta3
..................................................aaccacaauuacuuuugcacca.........   2    0    ta3

..........aaaaguaacugcgguuuuu....................................................   1    0    j10
...............................................aaaaaccacaauuacuuuugca............   5    0    j10
.................................................aaaccacaauuacuuuugcac...........   1    0    j10
..................................................aaccacaauuacuuuugcacca.........   1    0    j10

..........aaaaguaacugcgguuuuua...................................................   9    0    j04

..........aaaaguaacugcgguuuuu....................................................   1    0    k14
..................................................aaccacaauuacuuuugcac...........   1    0    k14

.........caaaaguaacugcgguuuuu....................................................   2    0    va2
..........aaaaguaacugcgguuuu.....................................................   1    0    va2
..........aaaaguaacugcgguuuuu....................................................   2    0    va2
...............................................aaaaaccacaauuacuuuugcac...........   1    0    va2

...............................................aaaaaccacaauuacuuuugca............   1    0    j31

.........caaaaguaacugcgguuuuu....................................................   2    0    j27
..........aaaaguaacugcgguuuuu....................................................   18   0    j27
.................................................aaaccacaauuacuuuugca............   1    0    j27

...............................................aaaaaccacaauuacuuuugca............   2    0    s03
................................................aaaaccacaauuacuuuugca............   1    0    s03
................................................aaaaccacaauuacuuuugcac...........   2    0    s03

...............................................aaaaaccacaauuacuuuug..............   2    0    H52
...............................................aaaaaccacaauuacuuuugca............   1    0    H52
................................................aaaaccacaauuacuuuugc.............   1    0    H52
................................................aaaaccacaauuacuuuugca............   2    0    H52
.................................................aaaccacaauuacuuuugcacc..........   2    0    H52
..................................................aaccacaauuacuuuugcacc..........   1    0    H52
..................................................aaccacaauuacuuuugcacca.........   1    0    H52
...................................................accacaauuacuuuugcacca.........   1    0    H52
....................................................ccacaauuacuuuugcaccag........   1    0    H52

....uggugcaaaaguaacugcgguu.......................................................   1    0    j22
.........caaaaguaacugcgguuuu.....................................................   1    0    j22
..........aaaaguaacugcgguuuu.....................................................   1    0    j22
...............................................aaaaaccacaauuacuuuugca............   6    0    j22
................................................aaaaccacaauuacuuuugca............   1    0    j22
..................................................aaccacaauuacuuuugcacc..........   1    0    j22

................................................aaaaccacaauuacuuuugcac...........   1    0    k09

...............................................aaaaaccacaauuacuuuugc.............   3    0    B14
...............................................aaaaaccacaauuacuuuugca............   6    0    B14
................................................aaaaccacaauuacuuuugcac...........   1    0    B14
.................................................aaaccacaauuacuuuugca............   2    0    B14
```

```
          Mature                                     Star
agguuggugcaaaaguaacugcgguuuuuaccauuacuuucaauggcaaaaaccacaauuacuuuugcaccagucuaauag
.................................................aaaccacaauuacuuuugcacc..........   2   0   B14
...................................................accacaauuacuuuugcacca.........   2   0   B14

...............................................aaaaaccacaauuacuuuugca............   1   0   j09
...................................................accacaauuacuuuugcacca.........   1   0   j09

.........caaaaguaacugcgguuuu.....................................................   1   0   ku3
.........caaaaguaacugcgguuuuu....................................................   5   0   ku3
..........aaaaguaacugcgguuuuu....................................................   1   0   ku3
...............................................aaaaaccacaauuacuuuugcac...........   1   0   ku3
.................................................aaaccacaauuacuuuugcacca.........   1   0   ku3

.........caaaaguaacugcgguuuuu....................................................   2   0   j18

..........aaaaguaacugcgguuuu.....................................................   1   0   j16
..........aaaaguaacugcgguuuuu....................................................   1   0   j16
...............................................aaaaaccacaauuacuuuugca............   4   0   j16

.........caaaaguaacugcgguuuuu....................................................   2   0   ku6
..........aaaaguaacugcgguuuuu....................................................   5   0   ku6
...............................................aaaaaccacaauuacuuuugca............   2   0   ku6

...............................................aaaaaccacaauuacuuuugca............   2   0   j11

.........caaaaguaacugcgguuu......................................................   1   0   ya3
.........caaaaguaacugcgguuuuu....................................................   3   0   ya3
..........aaaaguaacugcgguuuu.....................................................   3   0   ya3
..........aaaaguaacugcgguuuuu....................................................   23  0   ya3
...............................................aaaaaccacaauuacuuuugca............   3   0   ya3
................................................aaaaccacaauuacuuuugca............   2   0   ya3
................................................aaaaccacaauuacuuuugcac...........   5   0   ya3
.................................................aaaccacaauuacuuuugca............   1   0   ya3
.................................................aaaccacaauuacuuuugcacc..........   1   0   ya3
..................................................aaccacaauuacuuuugcacc..........   2   0   ya3

...............................................aaaaaccacaauuacuuuugca............   2   0   la1
..................................................aaccacaauuacuuuugcacc..........   1   0   la1

..............................................caaaaaccacaauuacuuuugc.............   3   0   HEK
...............................................aaaaaccacaauuacuuuugc.............   1   0   HEK
...............................................aaaaaccacaauuacuuuugca............   8   0   HEK
................................................aaaaccacaauuacuuuugca............   2   0   HEK
................................................aaaaccacaauuacuuuugcac...........   3   0   HEK
.................................................aaaccacaauuacuuuugca............   1   0   HEK
.................................................aaaccacaauuacuuuugcac...........   3   0   HEK
.................................................aaaccacaauuacuuuugcacc..........   3   0   HEK
..................................................aaccacaauuacuuuugcacc..........   2   0   HEK
..................................................aaccacaauuacuuuugcacca.........   4   0   HEK
...................................................accacaauuacuuuugcacca.........   6   0   HEK

................................................aaaaccacaauuacuuuugca............   1   0   j17
..................................................aaccacaauuacuuuugcacca.........   2   0   j17
...................................................accacaauuacuuuugcacca.........   1   0   j17

.........caaaaguaacugcgguuuu.....................................................   1   0   j15
..........aaaaguaacugcgguuuuu....................................................   5   0   j15
................................................aaaaccacaauuacuuuugca............   1   0   j15
....................................................ccacaauuacuuuugcaccag........   1   0   j15

................................................aaaaccacaauuacuuuugcac...........   1   0   la2
.................................................aaaccacaauuacuuuugca............   1   0   la2
.................................................aaaccacaauuacuuuugcacc..........   1   0   la2
..................................................aaccacaauuacuuuugcacca.........   1   0   la2

...................................................accacaauuacuuuugcacca.........   1   0   j12

...uuggugcaaaaguaacugcggu........................................................   1   0   li2

...............................................aaaaaccacaauuacuuuugca............   3   0   va4
...............................................aaaaaccacaauuacuuuugcac...........   1   0   va4
```

```
          Mature                                    Star
agguuggugcaaaaguaacugcgguuuuuaccauuacuuucaauggcaaaaaccacaauuacuuuugcaccagucuaauag
................................................aaaaccacaauuacuuuugcac...........   1    0    va4
..................................................aaccacaauuacuuuugcacc..........   2    0    va4
...................................................accacaauuacuuuugcacca.........   4    0    va4

...............................................aaaaaccacaauuacuuuugca............   12   0    va3
................................................aaaaccacaauuacuuuugcac...........   2    0    va3
.................................................aaaccacaauuacuuuugcac...........   1    0    va3
.................................................aaaccacaauuacuuuugcacc..........   4    0    va3
..................................................aaccacaauuacuuuugcacc..........   2    0    va3
..................................................aaccacaauuacuuuugcacca.........   2    0    va3
...................................................accacaauuacuuuugcacca.........   1    0    va3

..........aaaaguaacugcgguuuuu....................................................   14   0    ya4
...............................................aaaaaccacaauuacuuuugca............   4    0    ya4
................................................aaaaccacaauuacuuuugca............   2    0    ya4
................................................aaaaccacaauuacuuuugcac...........   4    0    ya4
.................................................aaaccacaauuacuuuugcac...........   1    0    ya4
.................................................aaaccacaauuacuuuugcacc..........   4    0    ya4
..................................................aaccacaauuacuuuugca............   3    0    ya4
..................................................aaccacaauuacuuuugcacc..........   4    0    ya4
..................................................aaccacaauuacuuuugcacca.........   2    0    ya4

..........aaaaguaacugcgguuuuu....................................................   1    0    SW4
...............................................aaaaaccacaauuacuuuugca............   1    0    SW4
................................................aaaaccacaauuacuuuugcac...........   1    0    SW4
.................................................aaaccacaauuacuuuugcac...........   2    0    SW4
.................................................aaaccacaauuacuuuugcacc..........   3    0    SW4
..................................................aaccacaauuacuuuugcacca.........   3    0    SW4
...................................................accacaauuacuuuugcacc..........   1    0    SW4
...................................................accacaauuacuuuugcacca.........   3    0    SW4

.........caaaaguaacugcgguuuu.....................................................   1    0    ku1
..........aaaaguaacugcgguuuuu....................................................   1    0    ku1
...............................................aaaaaccacaauuacuuuugca............   4    0    ku1
..................................................aaccacaauuacuuuugcacc..........   1    0    ku1

...............................................aaaaaccacaauuacuuuugc.............   1    0    j23
...............................................aaaaaccacaauuacuuuugca............   2    0    j23
................................................aaaaccacaauuacuuuugca............   1    0    j23
..................................................aaccacaauuacuuuugcacca.........   1    0    j23
...................................................accacaauuacuuuugcacca.........   1    0    j23

..........aaaaguaacugcgguuuuu....................................................   1    0    s05
...............................................aaaaaccacaauuacuuuugca............   2    0    s05

...............................................aaaaaccacaauuacuuu................   1    0    ta2
................................................aaaaccacaauuacuuuugcac...........   1    0    ta2

.........caaaaguaacugcgguuuu.....................................................   3    0    j24

...............................................aaaaaccacaauuacuuuugca............   1    0    k11

...............................................aaaaaccacaauuacuuuugca............   1    0    ta1

..............................................caaaaaccacaauuacuuuugc.............   1    0    s09
................................................aaaaccacaauuacuuuugca............   1    0    s09
..................................................aaccacaauuacuuuugcacca.........   1    0    s09
...................................................accacaauuacuuuugcacca.........   1    0    s09

....uggugcaaaaguaacugcg..........................................................   1    0    NL2
....uggugcaaaaguaacugcgguu.......................................................   2    0    NL2
.........caaaaguaacugcgguuuuu....................................................   1    0    NL2
..........aaaaguaacugcgguuuuu....................................................   14   0    NL2
..........aaaaguaacugcgguuuuua...................................................   1    0    NL2
..............................................caaaaaccacaauuacuuu................   1    0    NL2
...............................................aaaaaccacaauuacuuuugca............   3    0    NL2
..................................................aaccacaauuacuuuugcacc..........   1    0    NL2
..................................................aaccacaauuacuuuugcacca.........   5    0    NL2

..........aaaaguaacugcgguuuuu....................................................   2    0    j13
```

Mature Star

agguuggugcaaaaguaacugcgguuuuuaccauuacuuucaauggcaaaaaccacaauuacuuuugcaccagucuaauag

....aaaaaccacaauuacuuuugca............	4	0	j13
....aaaaccacaauuacuuuugca............	2	0	j13
....aaccacaauuacuuuugcacca.........	2	0	j13
.........caaaaguaacugcgguuuuu..	1	0	j19
..........aaaaguaacugcgguuuu...	3	0	j19
..........aaaaguaacugcgguuuuu..	5	0	j19
....aaaaaccacaauuacuuuugca............	2	0	j19
....aaaccacaauuacuuuugca............	2	0	j19
....aaccacaauuacuuuugcacca.........	1	0	j19
....accacaauuacuuuugcacca.........	2	0	j19
.........caaaaguaacugcgguuuuu..	2	0	j25
..........aaaaguaacugcgguuuuu..	4	0	j25
....aaaaaccacaauuacuuuugca............	1	0	j25
..........aaaaguaacugcgguuuu...	1	0	k01
....aaaaccacaauuacuuuugca............	1	0	k04
....aaaaaccacaauuacuuuugca............	2	0	s01
....aaaaccacaauuacuuuugcac...........	2	0	s01
....aaaccacaauuacuuuugcacc..........	2	0	s01
....caaaaaccacaauuacuuuugc.............	1	0	U20
....aaaaaccacaauuacuuuugc.............	22	0	U20
....aaaaaccacaauuacuuuugca............	17	0	U20
....aaaaccacaauuacuuuugca............	1	0	U20
....aaaaccacaauuacuuuugcac...........	8	0	U20
....aaaccacaauuacuuuugcac...........	6	0	U20
....aaaccacaauuacuuuugcacc..........	14	0	U20
....aaccacaauuacuuuugcacc..........	6	0	U20
....aaccacaauuacuuuugcacca.........	1	0	U20
....accacaauuacuuuugcacca.........	15	0	U20
....aaaaaccacaauuacuuuugca............	3	0	j21
....aaaaccacaauuacuuuugca............	1	0	j21
....aaaccacaauuacuuuugcacc..........	1	0	j21
....aaccacaauuacuuuugcacc..........	1	0	s11
....aaccacaauuacuuuugcacca.........	1	0	s11
....aaaaaccacaauuacuuuugca............	1	0	j07
....aaaccacaauuacuuuugcacc..........	1	0	j14
.........caaaaguaacugcgguuuuu..	5	0	j28
..........aaaaguaacugcgguuuu...	2	0	j28
..........aaaaguaacugcgguuuuu..	5	0	j28
....aaaaaccacaauuacuuuugca............	1	0	s08
....accacaauuacuuuugcac...........	1	0	s08
....aaaaaccacaauuacuuuugc.............	1	0	DLD
....aaaaccacaauuacuuuugcac...........	1	0	DLD
....aaaccacaauuacuuuugcacc..........	3	0	DLD
....accacaauuacuuuugcacc..........	1	0	DLD
....accacaauuacuuuugcacca.........	4	0	DLD
....aaaaaccacaauuacuuuugca............	1	0	k03
.........caaaaguaacugcgguuuuu..	5	0	ya2
..........aaaaguaacugcgguuuu...	4	0	ya2
..........aaaaguaacugcgguuuuu..	20	0	ya2
....aaaaaccacaauuacuuuu...............	2	0	ya2
....aaaaaccacaauuacuuuugc.............	1	0	ya2
....aaaaaccacaauuacuuuugca............	2	0	ya2
....aaaaccacaauuacuuuugca............	6	0	ya2
....aaaccacaauuacuuuugcac...........	3	0	ya2

```
          Mature                                Star
agguuggugcaaaaguaacugcgguuuuuaccauuacuuucaaggcaaaaaccacaauuacuuuugcaccagucuaauag
................................................aaaccacaauuacuuuugcacca.........	1	0	ya2
.................................................aaccacaauuacuuuugcacca.........	2	0	ya2

.........caaaaguaacugcgguuuuu...................................................	6	0	NL3
..........aaaaguaacugcgguuuu....................................................	1	0	NL3
..........aaaaguaacugcgguuuuu...................................................	18	0	NL3
.............................................caaaaaccacaauuacuuuu...............	1	0	NL3
.............................................caaaaaccacaauuacuuuug..............	2	0	NL3
..............................................aaaaaccacaauuacuuuugca............	7	0	NL3
...............................................aaaaccacaauuacuuuugcac...........	1	0	NL3
.................................................aaccacaauuacuuuugcacca.........	1	0	NL3

...uuggugcaaaaguaacugcgg........................................................	1	0	k06
...uuggugcaaaaguaacugcggu.......................................................	1	0	k06
................................................aaaccacaauuacuuuugcacc..........	1	0	k06

.............................................caaaaaccacaauuacuuuug..............	1	0	sh2

..............................................aaaaaccacaauuacuuuugca............	1	0	j29
...............................................aaaaccacaauuacuuuugca............	1	0	j29

...uuggugcaaaaguaacugcgg........................................................	1	0	MCF
......gugcaaaaguaacugcgguuu.....................................................	1	0	MCF
..............................................aaaaaccacaauuacuuuu...............	1	0	MCF
..............................................aaaaaccacaauuacuuuugc.............	1	0	MCF
..............................................aaaaaccacaauuacuuuugca............	2	0	MCF
...............................................aaaaccacaauuacuuuugcac...........	1	0	MCF

.........caaaaguaacugcgguuuu....................................................	1	0	j01
..........aaaaguaacugcgguuuuu...................................................	5	0	j01

...uuggugcaaaaguaacugcggu.......................................................	1	0	NL1
....uggugcaaaaguaacugcgguu......................................................	1	0	NL1

..............................................aaaaaccacaauuacuuuug..............	1	0	MBM
..............................................aaaaaccacaauuacuuuugc.............	1	0	MBM
..............................................aaaaaccacaauuacuuuugca............	2	0	MBM
...............................................aaaaccacaauuacuuuugca............	1	0	MBM
...............................................aaaaccacaauuacuuuugcac...........	3	0	MBM
................................................aaaccacaauuacuuuugcacc..........	4	0	MBM
.................................................aaccacaauuacuuuugcacc..........	1	0	MBM
..................................................accacaauuacuuuugcacca.........	4	0	MBM

............................................gcaaaaaccacaauuacuuuug..............	1	0	li1
..............................................aaaaaccacaauuacuuuugc.............	1	0	li1
...............................................aaaaccacaauuacuuuugcac...........	1	0	li1
................................................aaaccacaauuacuuuugcacc..........	2	0	li1
.................................................aaccacaauuacuuuugcacca.........	1	0	li1

..........aaaaguaacugcgguuuuu...................................................	1	0	k13

..............................................aaaaaccacaauuacuuuugc.............	7	0	A54
.................................................aaccacaauuacuuuugcacc..........	4	0	A54
.................................................aaccacaauuacuuuugcacca.........	4	0	A54

....uggugcaaaaguaacugcgguu......................................................	1	0	k12
..................................................accacaauuacuuuugcacca.........	1	0	k12
```

Provisional ID	:	hsa_can_42
Score total	:	2.5
Score for star read(s)	:	3.9
Score for read counts	:	-2.3
Score for mfe	:	1.6
Score for randfold	:	
Score for cons. seed	:	-0.6
Total read count	:	7
Mature read count	:	4
Loop read count	:	0
Star read count	:	3

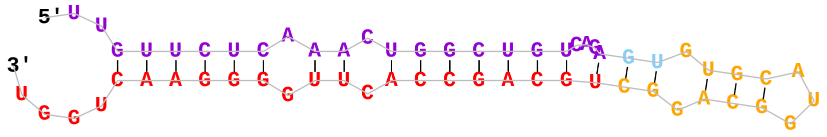

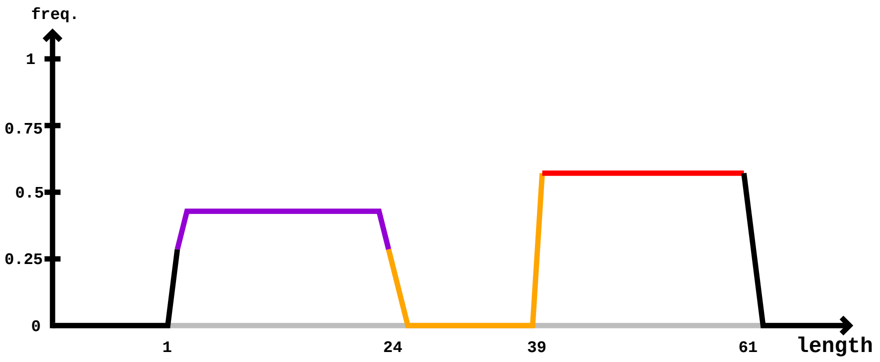

Star Mature

```
5'- uucugcagggcuuguucucaaacuggcugucagagugugcauggcaggcugcagccacuuggggaacugguucugugguua   -3'   obs
    uucugcagggcuuguucucaaacuggcugucagagugugcauggcaggcugcagccacuuggggaacugguucugugguua         exp
    ..(..(((((((.((((((.((.(((((((...(((.(((...))).))))))))))).)).)))))).)))))))..)...   reads   mm   sample
    ...........uuguucucaaacuggcugucag................................................   1       0    ya3

    .................................................ugcagccacuuggggaacuggu..........   1       0    k12

    ...........uuguucucaaacuggcugucaga...............................................   1       0    sh2
    .................................................ugcagccacuuggggaacuggu..........   3       0    sh2

    ............uguucucaaacuggcugucaga...............................................   1       0    U20
```

Provisional ID	:	hsa_can_64
Score total	:	143.2
Score for star read(s)	:	3.9
Score for read counts	:	135.2
Score for mfe	:	1
Score for randfold	:	
Score for cons. seed	:	3
Total read count	:	277
Mature read count	:	276
Loop read count	:	0
Star read count	:	1

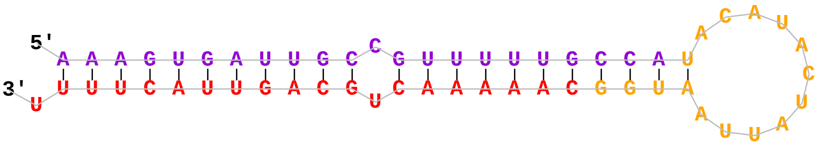

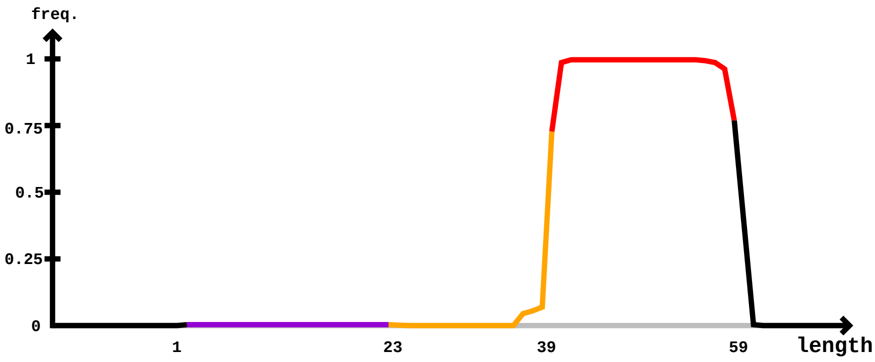

Star **Mature**

```
5'- uaaguuggugcgaaagugauugccguuuuugccauacauacuauuaauggcaaaaacugcaguuacuuuuacucacuauau  -3'   obs
    uaaguuggugcgaaagugauugccguuuuugccauacauacuauuaauggcaaaaacugcaguuacuuuuacucacuauau        exp
    .....(((((.((((((((((((.(((((((((((...........))))))))))).))))))))))))...)))))...     reads   mm   sample
    ...............................................uggcaaaaacugcaguuacuu.............     1       0    j23
    ...............................................uggcaaaaacugcaguuacuuu............     2       0    j23
    ..................................................caaaaacugcaguuacuuuu...........     6       0    j23
    ...................................................aaaaacugcaguuacuuuu...........     2       0    j23

    ..................................................caaaaacugcaguuacuuuu...........     1       0    ya1
    ...................................................aaaaacugcaguuacuuuu...........     3       0    ya1

    ...............................................uggcaaaaacugcaguuacuuu............     1       0    va1

    ...............................................uggcaaaaacugcaguuacuuuu...........     1       0    j24

    ...................................................aaaaacugcaguuacuuuu...........     1       0    k11

    ..................................................caaaaacugcaguuacuuuua..........     1       0    k05

    ..................................................caaaaacugcaguuacuuuu...........     1       0    k08

    ..................................................caaaaacugcaguuacuuuu...........     1       0    s10

    ..................................................caaaaacugcaguuacuuuu...........     18      0    NL2
    ...................................................aaaaacugcaguuacuuuu...........     13      0    NL2
    ...................................................aaaaacugcaguuacuuuua..........     2       0    NL2

    ..................................................caaaaacugcaguuacuuu............     1       0    k10
    ..................................................caaaaacugcaguuacuuuu...........     1       0    k10

    ..................................................caaaaacugcaguuacu..............     2       0    j19
    ..................................................caaaaacugcaguuacuuuu...........     4       0    j19
    ...................................................aaaaacugcaguuacuuuu...........     1       0    j19

    ...................................................aaaaacugcaguuacuuuua..........     1       0    k01

    ...................................................aaaaacugcaguuacuuu............     1       0    s01
```

```
            Star                                      Mature
uaaguuggugcgaaagugauugccguuuuugccauacauacuauuaaugGcaaaaacugcaguuacuuuuacucacuauau

...............................................uggcaaaaacugcaguuacuuu............   1   0   U20
................................................ggcaaaaacugcaguuac...............   1   0   U20

............aaagugauugccguuuuugcca...............................................   1   0   j21
................................................ggcaaaaacugcaguuacu..............   1   0   j21
..................................................caaaaacugcaguuacuu.............   1   0   j21
..................................................caaaaacugcaguuacuuuu...........   2   0   j21
...................................................aaaaacugcaguuacuuuu...........   1   0   j21

...................................................aaaaacugcaguuacuuuua..........   1   0   va2

.................................................gcaaaaacugcaguuacuuuu...........   1   0   j02
..................................................caaaaacugcaguuacuuu............   2   0   j02

..................................................caaaaacugcaguuacuuu............   1   0   k02

...................................................aaaaacugcaguuacuuuu...........   2   0   j28

..................................................caaaaacugcaguuacu..............   2   0   H52
..................................................caaaaacugcaguuacuuuu...........   7   0   H52
..................................................caaaaacugcaguuacuuuuac.........   1   0   H52
...................................................aaaaacugcaguuacuuuu...........   5   0   H52

..................................................caaaaacugcaguuacuuuu...........   1   0   j22

...............................................uggcaaaaacugcaguuacu..............   1   0   ku3

..................................................caaaaacugcaguuacuuuu...........   2   0   j16
..................................................caaaaacugcaguuacuuuua..........   1   0   j16
...................................................aaaaacugcaguuacuuuu...........   1   0   j16

.................................................gcaaaaacugcaguuacuuuu...........   1   0   ya2
..................................................caaaaacugcaguuacuuuu...........   9   0   ya2

..................................................caaaaacugcaguuacuuu............   5   0   NL3
..................................................caaaaacugcaguuacuuuu...........   15  0   NL3
...................................................aaaaacugcaguuacuuu............   2   0   NL3
...................................................aaaaacugcaguuacuuuu...........   2   0   NL3

...............................................uggcaaaaacugcaguuacuuu............   4   0   j11

..................................................caaaaacugcaguuacuuu............   2   0   ya3
..................................................caaaaacugcaguuacuuuu...........   3   0   ya3
...................................................aaaaacugcaguuacuuuu...........   2   0   ya3
....................................................aaaacugcaguuacuuuu...........   1   0   ya3

..................................................caaaaacugcaguuacuuuu...........   1   0   k06

.................................................gcaaaaacugcaguuacuuuu...........   1   0   la1

...............................................uggcaaaaacugcaguuacuuu............   2   0   j17
..................................................caaaaacugcaguuacuuuu...........   1   0   j17

..................................................caaaaacugcaguuacu..............   1   0   j15
..................................................caaaaacugcaguuacuuu............   28  0   j15
..................................................caaaaacugcaguuacuuuu...........   55  0   j15
..................................................caaaaacugcaguuacuuuua..........   1   0   j15
...................................................aaaaacugcaguuacuuu............   3   0   j15
...................................................aaaaacugcaguuacuuuu...........   24  0   j15

..................................................caaaaacugcaguuacuuuu...........   1   0   NL1
...................................................aaaaacugcaguuacuuuu...........   4   0   NL1

...................................................aaaaacugcaguuacuuuu...........   1   0   MBM

..................................................caaaaacugcaguuacuuuu...........   1   0   k07

...................................................aaaaacugcaguuacuuuu...........   1   0   k13

....................................................aaaacugcaguuacuuuu...........   2   0   SW4
```

```
            Star                                  Mature
uaaguuggugcgaaagugauugccguuuuugccauacauacuauuaaauggcaaaaacugcaguuacuuuuacucacuauau

.................................................ggcaaaaacugcaguuacuuuu...........    1    0    k12
...................................................caaaaacugcaguuacuuuu...........    2    0    k12
....................................................aaaaacugcaguuacuuuu...........    1    0    k12

...................................................caaaaacugcaguuacuuuu...........    10   0    ya4

..................................................gcaaaaacugcaguuacuuu............    1    0    ku1
....................................................aaaaacugcaguuacuuuu...........    1    0    ku1
```

Provisional ID	:	hsa_can_65
Score total	:	5.2
Score for star read(s)	:	3.9
Score for read counts	:	0.1
Score for mfe	:	1.8
Score for randfold	:	
Score for cons. seed	:	-0.6
Total read count	:	12
Mature read count	:	7
Loop read count	:	0
Star read count	:	5

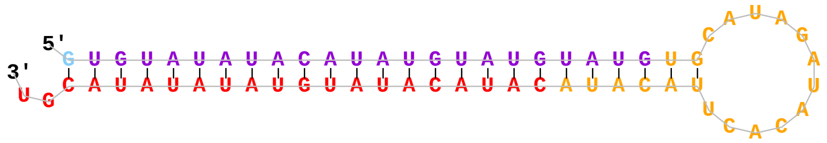

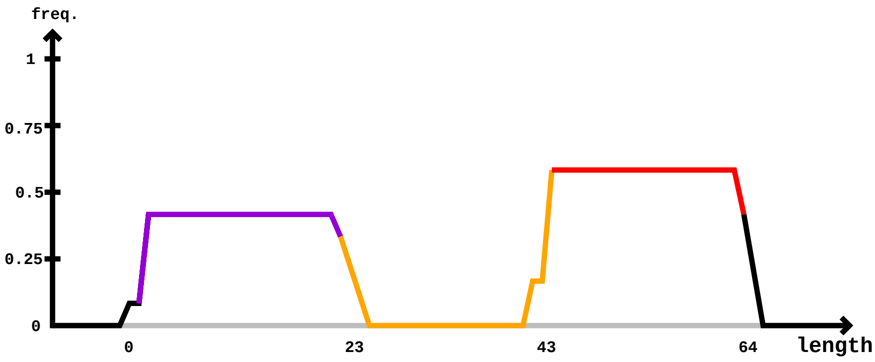

Star **Mature**

```
5'- uguaaauguguauauacauauguauguaugugcauagauacacuuacauacauacauauguauauauacguuuacauacua   -3'   obs
    uguaaauguguauauacauauguauguaugugcauagauacacuuacauacauacauauguauauauacguuuacauacua         exp
    ((((((((((((((((((((((((((((((((............))))))))))))))))))))))))))))))))....     reads      mm         sample
    ........uguauauacauauguauguaug...................................................     2          0          DLD

    ........uguauauacauauguauguaug...................................................     1          0          A54

    ......uguguauauacauauguaugua.....................................................     1          0          HEK
    ........uguauauacauauguauguaug...................................................     1          0          HEK
    ..................................................cauacauauguauauauacgu..........     2          0          HEK

    ................................................uacauacauauguauauauacg...........     2          0          U20
    ..................................................cauacauauguauauauacgu..........     3          0          U20
```

Provisional ID	:	hsa_can_72
Score total	:	-0.1
Score for star read(s)	:	-1.3
Score for read counts	:	0
Score for mfe	:	1.7
Score for randfold	:	
Score for cons. seed	:	-0.6
Total read count	:	81
Mature read count	:	81
Loop read count	:	0
Star read count	:	0

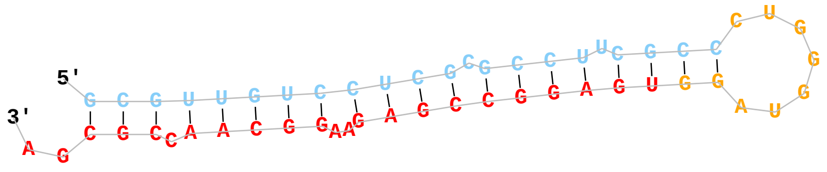

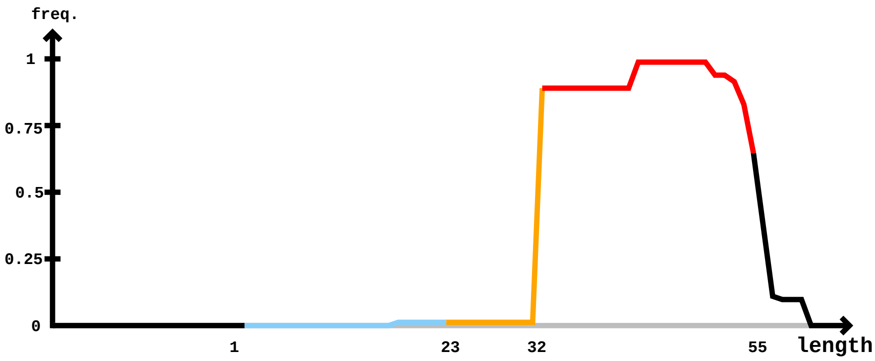

Star Mature

```
5'- cgggucgccucccaacccgcguuguccucgcgccuucgcccuggguaggugaggccgagaaggcaaccgcgaaaaggaggc -3'   exp
    ......((((((.....(((((((((((((.((((.((((.......))))))))))))..))))).)))).....))))))   reads   mm   sample
    ................................................ugaggccgagaaggcaaccgcg...........   1       0    j23

    ................................................ugaggccgagaaggcaaccgc............   1       0    DLD
    ................................................ugaggccgagaaggcaaccgcg...........   1       0    DLD
    ................................................ugaggccgagaaggcaaccgcga..........   1       0    DLD

    ................................................ugaggccgagaaggcaac...............   2       0    B14
    ................................................ugaggccgagaaggcaaccg.............   1       0    B14
    ................................................ugaggccgagaaggcaaccgc............   1       0    B14
    ................................................ugaggccgagaaggcaaccgcg...........   2       0    B14
    ................................................ugaggccgagaaggcaaccgcga..........   5       0    B14

    ..........................................................aaggcaaccgcgaaaagg.....   8       0    j18

    ................................................ugaggccgagaaggcaaccgcg...........   1       0    j16

    ................................................ugaggccgagaaggcaac...............   1       0    ya3
    ................................................ugaggccgagaaggcaaccgcgaa.........   1       0    ya3

    ................................................ugaggccgagaaggcaaccgcg...........   1       0    s09
    ................................................ugaggccgagaaggcaaccgcga..........   2       0    s09

    ..................................uucgcccuggguaggugagg...........................   1       0    HeL
    ................................................ugaggccgagaaggcaaccgcga..........   1       0    HeL

    ................................................ugaggccgagaaggcaaccg.............   1       0    HEK
    ................................................ugaggccgagaaggcaaccgcga..........   5       0    HEK

    ................................................ugaggccgagaaggcaaccgcga..........   1       0    MCF

    ................................................ugaggccgagaaggcaaccgcg...........   1       0    j17

    ................................................ugaggccgagaaggcaaccgcg...........   1       0    ta3

    ................................................ugaggccgagaaggcaaccgc............   3       0    li2
```

```
                  Star                          Mature
cgggucgccucccaacccgcguuguccucgcgccuucgcccuggguagguaggccgagaaggcaaccgcgaaaaggaggc
.................................................ugaggccgagaaggcaaccgcga.........   1    0    li2

.................................................ugaggccgagaaggcaaccgcga.........   1    0    ku2

.................................................ugaggccgagaaggcaac..............   1    0    U20
.................................................ugaggccgagaaggcaaccgcg..........   1    0    U20
.................................................ugaggccgagaaggcaaccgcga.........   11   0    U20

.................................................ugaggccgagaaggcaaccgc...........   1    0    j21
.................................................ugaggccgagaaggcaaccgcg..........   1    0    j21

......................................................ccgagaaggcaaccgcgaaa.......   1    0    va2

.................................................ugaggccgagaaggcaaccgcga.........   1    0    j30

.................................................ugaggccgagaaggcaaccgc...........   1    0    k12

.................................................ugaggccgagaaggcaaccgcg..........   4    0    H52
.................................................ugaggccgagaaggcaaccgcga.........   14   0    H52

.................................................ugaggccgagaaggcaaccgcg..........   1    0    s03
```

Provisional ID : hsa_can_90
Score total : 0
Score for star read(s) : -1.3
Score for read counts : 0
Score for mfe : 1.9
Score for randfold :
Score for cons. seed : -0.6
Total read count : 96
Mature read count : 86
Loop read count : 0
Star read count : 10

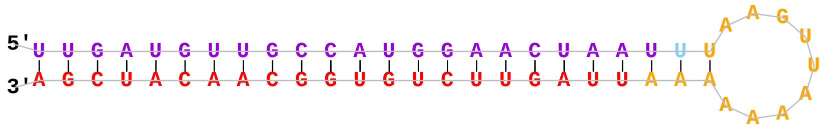

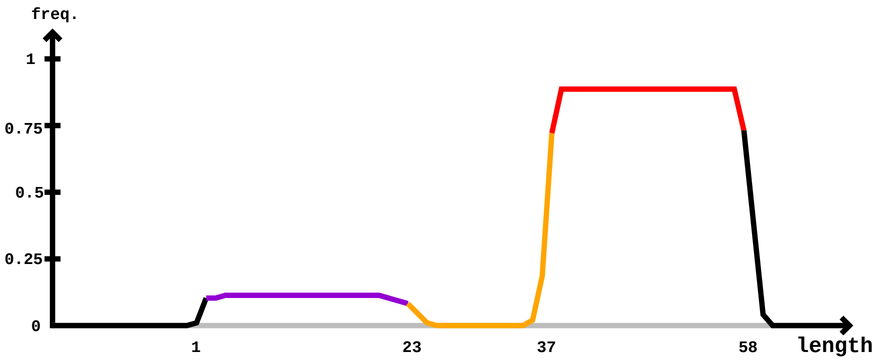


```
                       Star                                        Mature
5'- gaaaauugauuuuauugauguugccauggaacuaauuuaaguuaaaaaaauuaguucuguggcaacaucgauaaaauugau   -3'    obs
    gaaaauugauuuuauugauguugccauggaacuaauuuaaguuaaaaaaauuaguucuguggcaacaucgauaaaauugau          exp
    ....((..((((((((((((((((((((((((((((((.........))))))))))))))))))))))))))))))..))     reads      mm         sample
    ..................................................uuaguucuguggcaacaucga..........     1          0          j09

    .................................................auuaguucuguggcaacaucga..........     3          0          j18
    ..................................................uuaguucuguggcaacaucga..........     2          0          j18
    ...................................................uaguucuguggcaacaucga..........     4          0          j18

    ..............uugauguugccauggaacuaau.............................................     1          0          ku3
    .................................................auuaguucuguggcaacaucg...........     1          0          ku3
    ..................................................uuaguucuguggcaacaucga..........     2          0          ku3
    ...................................................uaguucuguggcaacaucga..........     1          0          ku3

    ..............uugauguugccauggaacuaau.............................................     1          0          s04

    .................................................auuaguucuguggcaacaucg...........     2          0          j26
    ...................................................uaguucuguggcaacaucg...........     1          0          j26

    ..............uugauguugccauggaacuaau.............................................     2          0          va1
    ................gauguugccauggaacuaauuu...........................................     1          0          va1

    .................................................auuaguucuguggcaacaucga..........     2          0          j24
    ..................................................uuaguucuguggcaacaucga..........     1          0          j24

    ..................................................uuaguucuguggcaacaucg...........     1          0          ku5
    ..................................................uuaguucuguggcaacaucga..........     2          0          ku5

    ..................................................uuaguucuguggcaacaucgau.........     1          0          s06

    ...................................................uaguucuguggcaacaucgau.........     1          0          s09

    ..................................................uuaguucuguggcaacaucga..........     1          0          la1

    ..................................................uuaguucuguggcaacaucga..........     1          0          HEK
    ...................................................uaguucuguggcaacaucgau.........     1          0          HEK
```

```
              Star                                Mature
gaaaauugauuuuauugauguugccauggaacuaauuuaaguuaaaaaaauuaguucuguggcaacaucgauaaaauugau

...................................................uaguucuguggcaacaucgau.........    1    0    s07

..................................................uuaguucuguggcaacaucga..........    2    0    j01

..............uugauguugccauggaacuaa..............................................    1    0    j17
................................................aauuaguucuguggcaacaucg...........    1    0    j17
..................................................uuaguucuguggcaacaucg...........    2    0    j17
..................................................uuaguucuguggcaacaucga..........    1    0    j17
..................................................uuaguucuguggcaacaucgau.........    4    0    j17
...................................................uaguucuguggcaacaucgau.........    2    0    j17

..................................................uuaguucuguggcaacaucga..........    1    0    s10

..................................................uuaguucuguggcaacaucgau.........    4    0    j06

................................................aauuaguucuguggcaacaucg...........    1    0    li1
.................................................auuaguucuguggcaacaucga..........    3    0    li1
..................................................uuaguucuguggcaacaucg...........    1    0    li1
..................................................uuaguucuguggcaacaucga..........    11   0    li1
..................................................uuaguucuguggcaacaucgaua........    1    0    li1

.................................................auuaguucuguggcaacaucga..........    1    0    li2
..................................................uuaguucuguggcaacaucga..........    1    0    li2

.............auugauguugccauggaacu................................................    1    0    j25
..................................................uuaguucuguggcaacaucg...........    1    0    j25
..................................................uuaguucuguggcaacaucga..........    3    0    j25

.................................................auuaguucuguggcaacaucga..........    1    0    ku2
..................................................uuaguucuguggcaacaucg...........    1    0    ku2
..................................................uuaguucuguggcaacaucgau.........    1    0    ku2
...................................................uaguucuguggcaacaucgaua........    1    0    ku2

..................................................uuaguucuguggcaacaucga..........    1    0    U20

..................................................uuaguucuguggcaacaucgau.........    1    0    j20

.................................................auuaguucuguggcaacaucga..........    3    0    j04

...................................................uaguucuguggcaacaucga..........    1    0    j07

...................................................uaguucuguggcaacaucg...........    1    0    j27

..............uugauguugccauggaacua...............................................    1    0    j31
..............uugauguugccauggaacuaau.............................................    1    0    j31
..................................................uuaguucuguggcaacaucg...........    2    0    j31
..................................................uuaguucuguggcaacaucga..........    2    0    j31
...................................................uaguucuguggcaacaucgaua........    2    0    j31

..............uugauguugccauggaacuaau.............................................    2    0    j28
```

Provisional ID	:	hsa_can_103
Score total	:	40.1
Score for star read(s)	:	3.9
Score for read counts	:	36.8
Score for mfe	:	0
Score for randfold	:	
Score for cons. seed	:	-0.6
Total read count	:	84
Mature read count	:	43
Loop read count	:	0
Star read count	:	41

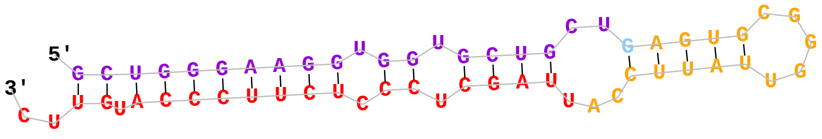

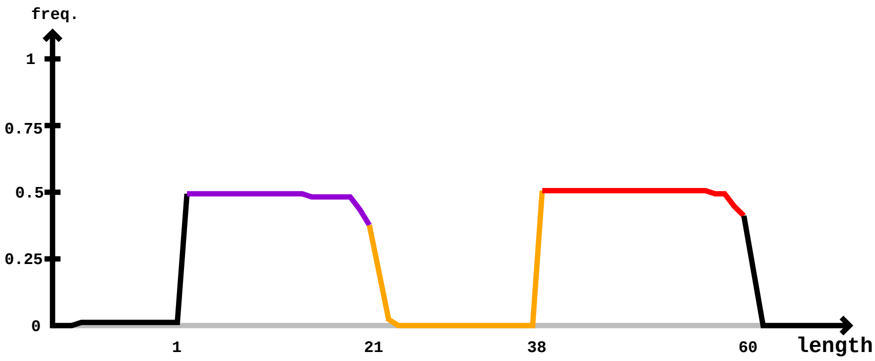

Star **Mature**

```
5'- ugucugcugugggcugggaagguggugcugcugagugcggguuauuccauuagcucccucuucccauguucucaugugaau  -3'   obs
    ugucugcugugggcugggaagguggugcugcugagugcggguuauuccauuagcucccucuucccauguucucaugugaau        exp
    ..((.((((.((((((((((((.((.((((..(((((.....)))))...)))).)).)))))))).)))).)).))))..     reads   mm   sample
    ............gcugggaagguggugcugcug................................................     1       0    s08

    ............gcugggaagguggugcugcu.................................................     1       0    k09
    ............gcugggaagguggugcugcug................................................     1       0    k09

    ............gcugggaagguggugcugcu.................................................     1       0    ya1

    ............gcugggaagguggugcugcu.................................................     1       0    k03

    ............gcugggaagguggugcugc..................................................     1       0    s04
    ............gcugggaagguggugcugcu.................................................     1       0    s04

    ............gcugggaagguggugcugcu.................................................     1       0    ya2

    ............gcugggaagguggugcugc..................................................     1       0    j26

    ............gcugggaagguggugcugcug................................................     2       0    k11
    ............gcugggaagguggugcugcuga...............................................     2       0    k11

    .gucugcugugggcugggaaggugg........................................................     1       0    k05
    ............gcugggaagguggugcugcu.................................................     1       0    k05

    ............gcugggaagguggugcug...................................................     1       0    k06
    ............gcugggaagguggugcugc..................................................     1       0    k06
    ............gcugggaagguggugcugcu.................................................     2       0    k06

    ............gcugggaagguggugcugcug................................................     2       0    sh2
    ................................................uuagcucccucuucccau...............     1       0    sh2
    ................................................uuagcucccucuucccaugu.............     4       0    sh2
    ................................................uuagcucccucuucccauguu............     1       0    sh2
    ................................................uuagcucccucuucccauguuc...........     15      0    sh2
    ................................................uuagcucccucuucccauguucu..........     11      0    sh2

    ............gcugggaagguggugcugcu.................................................     2       0    k08
```

```
            Star                                Mature
ugucugcugugggcugggaagguggugcugcugagugcggguuauuccauuagcucccucuucccauguucucaugugaau
............gcugggaagguggugcugcug................................................   1   0   k08

............gcugggaagguggugcugcu.................................................   2   0   k07

............gcugggaagguggugcugc..................................................   1   0   k10
............gcugggaagguggugcugcu.................................................   3   0   k10
.................................................uuagcucccucuucccauguuc..........   1   0   k10

............gcugggaagguggugcugcu.................................................   2   0   k13
.................................................uuagcucccucuucccauguucu.........   1   0   k13

.................................................uuagcucccucuucccauguu...........   2   0   U20
.................................................uuagcucccucuucccauguuc..........   6   0   U20

............gcugggaagguggugcugcug................................................   1   0   k14

............gcugggaagguggugcug...................................................   2   0   k02
............gcugggaagguggugcugc..................................................   1   0   k02
............gcugggaagguggugcugcug................................................   1   0   k02

............gcugggaagguggugcug...................................................   1   0   k12
............gcugggaagguggugcugcu.................................................   1   0   k12
............gcugggaagguggugcugcug................................................   1   0   k12
.................................................uuagcucccucuucccauguuc..........   1   0   k12

............gcugggaagguggugcugcug................................................   2   0   s03
```

Provisional ID	:	hsa_can_116
Score total	:	-0.9
Score for star read(s)	:	-1.3
Score for read counts	:	0
Score for mfe	:	0.9
Score for randfold	:	
Score for cons. seed	:	-0.6
Total read count	:	37
Mature read count	:	37
Loop read count	:	0
Star read count	:	0

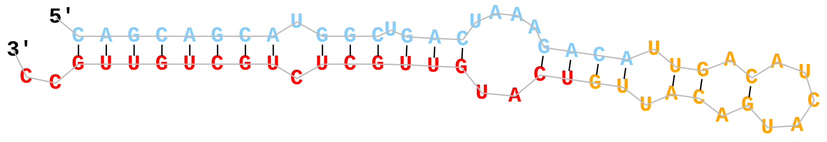

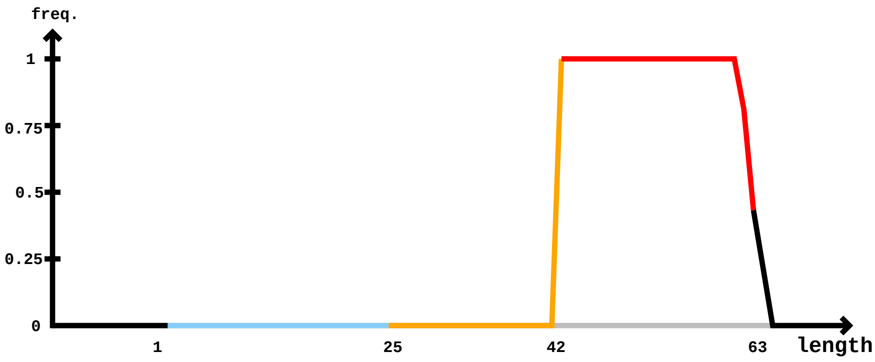

Star Mature

```
5'- uugucaggcucagcagcauggcugacuaaagacauugacaucaugacauugucauguugcucugcuguugccccuggcucu  -3'    exp
    ..((((((..((((((((.(((.(((....((((.((.(.....).)).))))..)))))).))))))))..))))))...       reads     mm     sample
    ...................................................ucauguugcucugcuguugcc.........        1         0      s08

    ...................................................ucauguugcucugcuguug............        1         0      j23

    ...................................................ucauguugcucugcuguugc...........        3         0      B14

    ...................................................ucauguugcucugcuguug............        1         0      ku3

    ...................................................ucauguugcucugcuguug............        1         0      j16

    ...................................................ucauguugcucugcuguugc...........        1         0      va1

    ...................................................ucauguugcucugcuguugcc.........        3         0      HEK

    ...................................................ucauguugcucugcuguugc...........        1         0      sh2

    ...................................................ucauguugcucugcuguugc...........        1         0      MCF

    ...................................................ucauguugcucugcuguugcc.........        2         0      NL2

    ...................................................ucauguugcucugcuguug............        1         0      j19

    ...................................................ucauguugcucugcuguug............        1         0      li1
    ...................................................ucauguugcucugcuguugc...........        5         0      li1
    ...................................................ucauguugcucugcuguugcc.........        7         0      li1

    ...................................................ucauguugcucugcuguugc...........        1         0      li2
    ...................................................ucauguugcucugcuguugcc.........        1         0      li2

    ...................................................ucauguugcucugcuguugc...........        1         0      k04

    ...................................................ucauguugcucugcuguugc...........        1         0      U20

    ...................................................ucauguugcucugcuguug............        1         0      SW4
```

```
          Star                                    Mature
uugucaggcucagcagcauggcugacuaaagacauugacaucaugacauugucauguugcucugcuguugccccuggcucu
...................................................ucauguugcucugcuguugcc.........    1    0    SW4

...................................................ucauguugcucugcuguugcc.........    1    0    k12

...................................................ucauguugcucugcuguug...........    1    0    j22
```

Provisional ID	:	hsa_can_123
Score total	:	6.7
Score for star read(s)	:	3.9
Score for read counts	:	2.7
Score for mfe	:	0.8
Score for randfold	:	
Score for cons. seed	:	-0.6
Total read count	:	17
Mature read count	:	13
Loop read count	:	0
Star read count	:	4

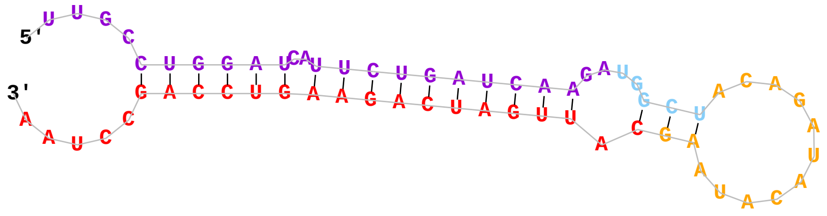

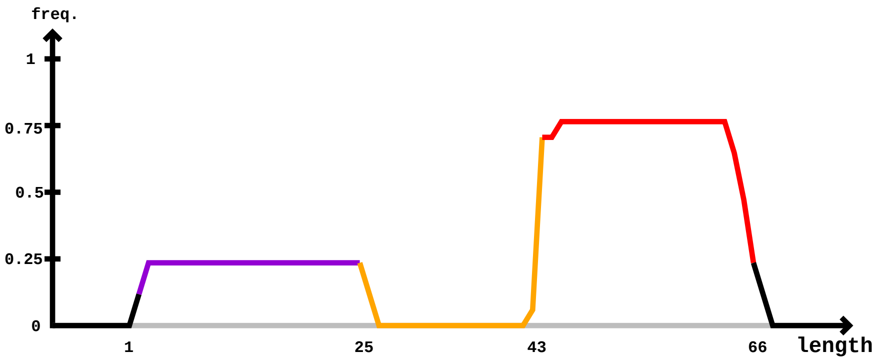

Star **Mature**

```
5'- gcggcccuugccuggaucauucugaucaagauggcuacagauacauaagcauugaucagaaguccagccuaaggccuucau  -3'   obs
    gcggcccuugccuggaucauucugaucaagauggcuacagauacauaagcauugaucagaaguccagccuaaggccuucau         exp
    ..((((...(.((((((..((((((((((....(((...........))).)))))))))))))))).)...)))).....    reads    mm    sample
    .................................................cauugaucagaaguccagccua..........    1        0     NL2

    ................................................gcauugaucagaaguccagccu...........    1        0     ta3
    .................................................cauugaucagaaguccagccua..........    1        0     ta3

    ........ugccuggaucauucugaucaaga..................................................    1        0     li1

    .......uugccuggaucauucugaucaaga..................................................    1        0     li2

    .................................................cauugaucagaaguccagccuaa.........    3        0     ya2

    ...................................................uugaucagaaguccagccuaa.........    1        0     s01

    .......uugccuggaucauucugaucaaga..................................................    1        0     per

    .................................................cauugaucagaaguccagccu...........    2        0     U20

    .................................................cauugaucagaaguccagccua..........    1        0     j21

    .................................................cauugaucagaaguccagcc............    2        0     HEK

    ........ugccuggaucauucugaucaagau.................................................    1        0     ku1

    .................................................cauugaucagaaguccagccua..........    1        0     j15
```

Provisional ID	:	hsa_can_127
Score total	:	-0.1
Score for star read(s)	:	-1.3
Score for read counts	:	0
Score for mfe	:	1.7
Score for randfold	:	
Score for cons. seed	:	-0.6
Total read count	:	68
Mature read count	:	68
Loop read count	:	0
Star read count	:	0

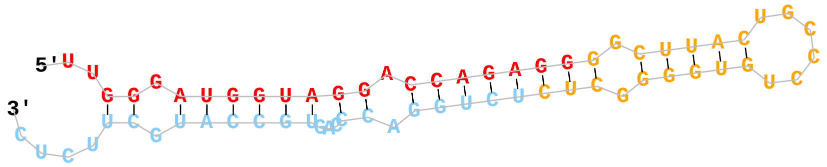

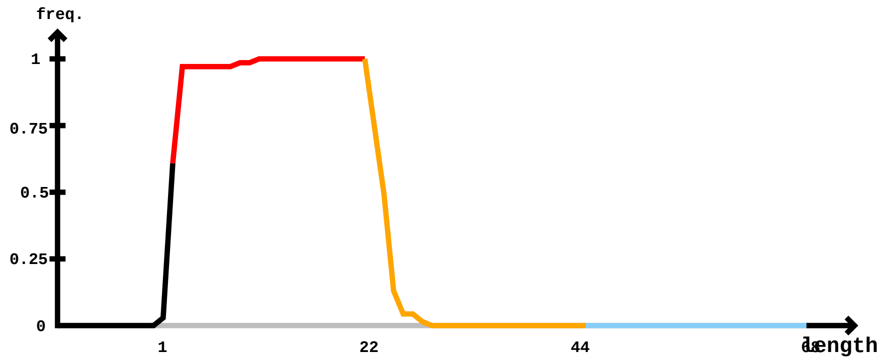

Mature **Star**

```
5'- gugagcuagauugggaugguaggaccagaggggcuuacugcccuguggggcucucuggacccagugccaugcuucucugcu  -3'   exp
    ...(((.(((..((.((((((((.((((((((.(((((......))))).)))))))).))...)))))).))..))))))   reads   mm   sample
    ...........ugggaugguaggaccagaggg.................................................   1       0    j23

    ..........uugggaugguaggaccagaggg.................................................   1       0    s08
    ...........ugggaugguaggaccagagggg................................................   1       0    s08
    ...........ugggaugguaggaccagaggggc...............................................   1       0    s08

    ..........uugggaugguaggaccagagg..................................................   1       0    j16
    ..........uugggaugguaggaccagagggg................................................   1       0    j16
    ...........ugggaugguaggaccagaggggc...............................................   2       0    j16

    ..........uugggaugguaggaccagagg..................................................   2       0    s05

    ..........uugggaugguaggaccagagg..................................................   1       0    va1
    ..........uugggaugguaggaccagaggg.................................................   1       0    va1
    ..........uugggaugguaggaccagagggg................................................   3       0    va1
    ...........ugggaugguaggaccagagg..................................................   1       0    va1

    ...........ugggaugguaggaccagaggggcuu.............................................   1       0    s12

    .........auugggaugguaggaccagagggg................................................   1       0    s06
    ..........uugggaugguaggaccagaggg.................................................   1       0    s06

    ..........uugggaugguaggaccagagggg................................................   1       0    la1

    ..........uugggaugguaggaccagaggg.................................................   2       0    j17
    ...........ugggaugguaggaccagaggg.................................................   1       0    j17

    .........auugggaugguaggaccagagggg................................................   1       0    j15

    ..........uugggaugguaggaccagagg..................................................   1       0    s07
    ..........uugggaugguaggaccagagggg................................................   2       0    s07
    ...........ugggaugguaggaccagagggg................................................   1       0    s07

    .................gguaggaccagaggggcuu.............................................   1       0    la2
```

```
          Mature                                    Star
gugagcuagauugggaugguaggaccagaggggcuuacugcccuguggggcucucuggacccagugccaugcuucucugcu

..........uugggaugguaggaccagagggg................................................    2    0    s10

..........uugggaugguaggaccagaggg.................................................    1    0    j12
..........uugggaugguaggaccagagggg................................................    1    0    j12

...........ugggaugguaggaccagagggg................................................    1    0    j19
...........ugggaugguaggaccagaggggc...............................................    1    0    j19

..........uugggaugguaggaccagagg..................................................    3    0    li1
..........uugggaugguaggaccagaggg.................................................    1    0    li1
..........uugggaugguaggaccagagggg................................................    2    0    li1
...........ugggaugguaggaccagagggg................................................    1    0    li1

..........uugggaugguaggaccagaggg.................................................    1    0    li2
..........uugggaugguaggaccagagggg................................................    1    0    li2
...........ugggaugguaggaccagaggggc...............................................    2    0    li2

..........uugggaugguaggaccagagggg................................................    1    0    U20

..........uugggaugguaggaccagaggg.................................................    1    0    va4
...........ugggaugguaggaccagagggg................................................    1    0    va4

..........uugggaugguaggaccagagg..................................................    5    0    j21
..........uugggaugguaggaccagagggg................................................    1    0    j21
...........ugggaugguaggaccagagg..................................................    4    0    j21
...........ugggaugguaggaccagaggg.................................................    2    0    j21
...........ugggaugguaggaccagagggg................................................    1    0    j21

...........ugggaugguaggaccagagggg................................................    1    0    s11

...................uaggaccagaggggcuua............................................    1    0    j27

..........uugggaugguaggaccagagg..................................................    1    0    j30
..........uugggaugguaggaccagaggg.................................................    1    0    j30
...........ugggaugguaggaccagagggg................................................    1    0    j30

..........uugggaugguaggaccagaggg.................................................    1    0    j22
...........ugggaugguaggaccagaggg.................................................    1    0    j22
```

Provisional ID	:	hsa_can_152
Score total	:	34.6
Score for star read(s)	:	3.9
Score for read counts	:	36.3
Score for mfe	:	-4.9
Score for randfold	:	
Score for cons. seed	:	-0.6
Total read count	:	83
Mature read count	:	80
Loop read count	:	2
Star read count	:	1

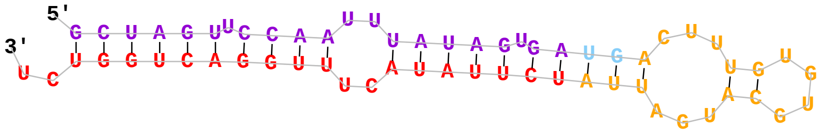

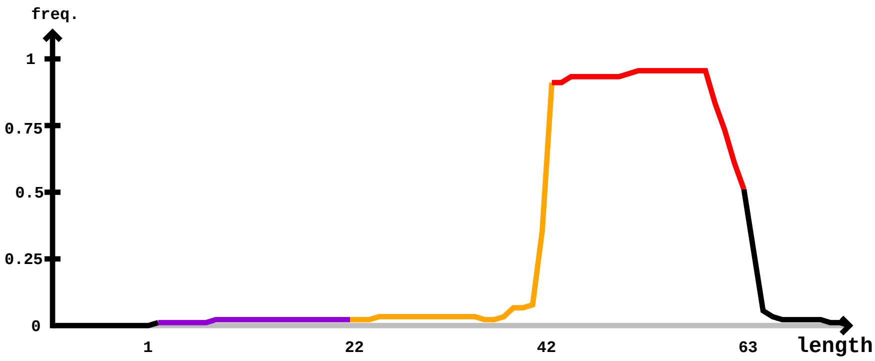

Star **Mature**

```
5'- uucuagugugcuaguuccaauuuauagugaugacuuugugugcaugauuaucuuauacuuuggacuggucucauauuugaa  -3'   obs
    uucuagugugcuaguuccaauuuauagugaugacuuugugugcaugauuaucuuauacuuuggacuggucucauauuugaa        exp
    .((.((((((((((.((((..(((((.(((((...((....))...))))))))))..)))))))))...)))))).)).     reads    mm    sample
    .................................................aucuuauacuuuggacug..............     1        0     s08

    ..............................................auuaucuuauacuuuggacug..............     2        0     j23
    ..................................................ucuuauacuuuggacuggucu..........     5        0     j23
    ...........................................................uuggacuggucucauauuugaa     1        0     j23

    ..................................................ucuuauacuuuggacuggucu..........     1        0     j18

    ..................................................ucuuauacuuuggacugguc...........     3        0     j16
    ..................................................ucuuauacuuuggacuggucu..........     4        0     j16
    ..................................................ucuuauacuuuggacuggucuc.........     1        0     j16

    ..................................................ucuuauacuuuggacugg.............     1        0     s05

    ..................................................ucuuauacuuuggacuggucuc.........     1        0     va1

    .................................................aucuuauacuuuggacuggucu..........     1        0     s12
    ..................................................ucuuauacuuuggacugguc...........     1        0     s12
    ..................................................ucuuauacuuuggacuggucu..........     1        0     s12

    ..................................................ucuuauacuuuggacuggucu..........     1        0     HeL

    .................................................aucuuauacuuuggacugg.............     1        0     ku4
    ..................................................ucuuauacuuuggacuggu............     1        0     ku4

    .................................................aucuuauacuuuggacugg.............     3        0     la1

    .................................................aucuuauacuuuggacugguc...........     1        0     j15
    ..................................................ucuuauacuuuggacuggu............     1        0     j15
    ..................................................ucuuauacuuuggacuggucu..........     1        0     j15
    ....................................................uuauacuuuggacuggucu..........     1        0     j15

    .................................................aucuuauacuuuggacug..............     3        0     j17
```

Star **Mature**

```
uucuagugugcuaguuccaauuuauagugaugacuuugugugcaugauuaucuuauacuuuggacuggucucauauuugaa
..................................................ucuuauacuuuggacuggucu..........  1  0  j17

..................................................ucuuauacuuuggacuggu............  5  0  j06

..............................ugacuuugugugcaugauuauc.............................  1  0  j12

.................................................aucuuauacuuuggacugg.............  1  0  j19
..................................................ucuuauacuuuggacuggucu..........  1  0  j19

.................................................aucuuauacuuuggacugg.............  1  0  li1
.................................................aucuuauacuuuggacuggu............  2  0  li1
..................................................ucuuauacuuuggacuggucu..........  1  0  li1

..................................................ucuuauacuuuggacuggucu..........  1  0  va4

.................................................aucuuauacuuuggacug..............  1  0  j10

.........gcuaguuccaauuuauaguga...................................................  1  0  j21
................................acuuugugugcaugauua...............................  1  0  j21
.............................................gauuaucuuauacuuuggacug..............  1  0  j21
..............................................auuaucuuauacuuuggacuggu............  1  0  j21
................................................uaucuuauacuuuggacugguc...........  1  0  j21
.................................................aucuuauacuuuggacug..............  3  0  j21
.................................................aucuuauacuuuggacugg.............  1  0  j21
.................................................aucuuauacuuuggacuggucu..........  2  0  j21
.................................................aucuuauacuuuggacuggucuc.........  1  0  j21
..................................................ucuuauacuuuggacugguc...........  1  0  j21
..................................................ucuuauacuuuggacuggucu..........  8  0  j21
..................................................ucuuauacuuuggacuggucuc.........  6  0  j21
....................................................uuauacuuuggacuggucuca........  1  0  j21
....................................................uuauacuuuggacuggucucau.......  1  0  j21
..........................................................uuuggacuggucucauauuug..  1  0  j21

.................................................aucuuauacuuuggacugg.............  1  0  j20
.................................................aucuuauacuuuggacuggu............  1  0  j20

..................................................ucuuauacuuuggacugguc...........  1  0  s11

..................................................ucuuauacuuuggacuggucuca........  1  0  va3

..................................................ucuuauacuuuggacuggucu..........  1  0  j31

..................................................ucuuauacuuuggacuggucu..........  1  0  j14

...............uccaauuuauagugaugacuuugugugc......................................  1  0  SW4

.................................................aucuuauacuuuggacugguc...........  1  0  j22
..................................................ucuuauacuuuggacuggucu..........  1  0  j22
```

Provisional ID	:	hsa_can_153
Score total	:	78.6
Score for star read(s)	:	3.9
Score for read counts	:	73.5
Score for mfe	:	1.8
Score for randfold	:	
Score for cons. seed	:	-0.6
Total read count	:	156
Mature read count	:	68
Loop read count	:	0
Star read count	:	88

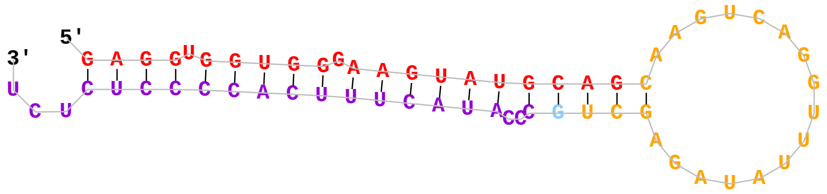

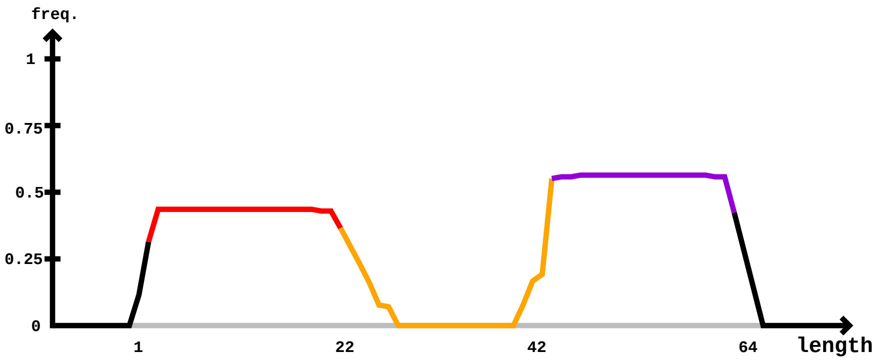

Mature **Star**

```
5'- ggcugggagagguggugggaaguaugcagcaagucagguuuauagagcugcccauacuuucaccccucucucaggccucau  -3'    obs
    ggcugggagagguggugggaaguaugcagcaagucagguuuauagagcugcccauacuuucaccccucucucaggccucau          exp
    ((((((((((((.(((((.(((((((((((................)))))..)))))))))))))))))))).)))....   reads      mm         sample
    ........gagguggugggaaguaugca.....................................................   1          0          j23
    ........gagguggugggaaguaugcagca..................................................   1          0          j23

    ..................................................cccauacuuucaccccucu............   1          0          s05

    ................................................ugcccauacuuucaccccucu............   1          0          s04

    ........gagguggugggaaguaugcag....................................................   1          0          j26
    ..................................................cccauacuuucaccccucu............   1          0          j26
    ..................................................cccauacuuucaccccucucu..........   2          0          j26

    .......agagguggugggaaguaugcag....................................................   2          0          ta2

    .......agagguggugggaaguaugca.....................................................   1          0          va1
    ........gagguggugggaaguaugca.....................................................   3          0          va1
    ........gagguggugggaaguaugcagc...................................................   2          0          va1
    ........gagguggugggaaguaugcagcaa.................................................   3          0          va1
    ........gagguggugggaaguaugcagcaagu...............................................   1          0          va1
    .........agguggugggaaguaugcagcaag................................................   1          0          va1
    .........agguggugggaaguaugcagcaagu...............................................   1          0          va1

    ..................................................cccauacuuucaccccucucu..........   1          0          ku5

    ................................................ugcccauacuuucaccccucuc...........   1          0          ta1
    ................................................ugcccauacuuucaccccucucu..........   1          0          ta1
    ..................................................cccauacuuucaccccucucu..........   1          0          ta1

    ..................................................cccauacuuucaccccucucu..........   1          0          HeL

    .........agguggugggaaguaugcagcaa.................................................   1          0          ku4

    ..................................................cccauacuuucaccccucucu..........   1          0          s07

    ..................................................cccauacuuucaccccucucu..........   1          0          s10
```

Mature **Star**

```
ggcugggagagguggugggaaguaugcagcaagucagguuuauagagcugcccauacuuucaccccucucucaggccucau

..................................................cccauacuuucaccccucuc...........  1  0  ta3
..................................................cccauacuuucaccccucucu..........  6  0  ta3

..................................................cccauacuuucaccccucuc...........  1  0  NL2

..................................................cccauacuuucaccccucucuc.........  1  0  k10

..................................................cccauacuuucaccccucu............  1  0  j13
.....................................................auacuuucaccccucucu..........  1  0  j13

........gagguggugggaaguaugcag....................................................  7  0  j06

..................................................cccauacuuucaccccucucu..........  1  0  per

...............................................cugcccauacuuucaccccucu............  1  0  k04
..................................................cccauacuuucaccccucucu..........  1  0  k04

........gagguggugggaaguaugcagcaa.................................................  1  0  s01
.........agguggugggaaguaugcagcaa.................................................  1  0  s01

................................................ugcccauacuuucaccccucucu..........  1  0  ku2
..................................................cccauacuuucaccccucu............  1  0  ku2
..................................................cccauacuuucaccccucucu..........  3  0  ku2

................................................ugcccauacuuucaccccucuc...........  5  0  U20

.......agagguggugggaaguaugcagca..................................................  1  0  j10

.......agagguggugggaaguaugca.....................................................  1  0  j21
........gagguggugggaaguaugcagc...................................................  2  0  j21
........gagguggugggaaguaugcagca..................................................  3  0  j21
...............................................cugcccauacuuucaccccucu............  1  0  j21
................................................ugcccauacuuucaccccucucu..........  1  0  j21
..................................................cccauacuuucaccccucucu..........  1  0  j21

.........agguggugggaaguaugcagcaagu...............................................  1  0  s11
...............................................cugcccauacuuucaccccucu............  1  0  s11
..................................................cccauacuuucaccccucucu..........  2  0  s11

.........agguggugggaaguaugcagcaa.................................................  2  0  j27

........gagguggugggaaguaugca.....................................................  1  0  j07

.......agagguggugggaaguaugcagca..................................................  1  0  j31
........gagguggugggaaguaugcagcaa.................................................  1  0  j31
.........agguggugggaaguaugcagcaagu...............................................  1  0  j31
...............................................cugcccauacuuucaccccucu............  1  0  j31

..................................................cccauacuuucaccccucucu..........  1  0  k02

.......agagguggugggaaguaugcagca..................................................  1  0  j14

................................................ugcccauacuuucaccccucucu..........  1  0  j28
.................................................gcccauacuuucaccccucucu..........  4  0  j28
..................................................cccauacuuucaccccucu............  2  0  j28
..................................................cccauacuuucaccccucucu..........  4  0  j28

.......agagguggugggaaguaugcag....................................................  1  0  s03
.........agguggugggaaguaugcagcaagu...............................................  1  0  s03
..................................................cccauacuuucaccccucuc...........  1  0  s03

........gagguggugggaaguaugca.....................................................  1  0  H52
.........agguggugggaaguaugcagcaagu...............................................  2  0  H52

...............................................cugcccauacuuucaccccucu............  1  0  j22

.........agguggugggaaguaugcagcaa.................................................  1  0  k09
..................................................cccauacuuucaccccucucu..........  1  0  k09

.........agguggugggaaguaugcagcaa.................................................  1  0  s08
```

```
        Mature                                    Star
ggcugggagagguggugggaaguaugcagcaagucagguuuauagagcugcccauacuuucaccccucucucaggccucau
..................................................cccauacuuucaccccucucu..........    1          0          s08

..................................................cccauacuuucaccccucucu..........    2          0          DLD

..................................................cccauacuuucaccccucucu..........    1          0          B14

.......agagguggugggaaguaugca.....................................................    1          0          ku3
.........agguggugggaaguaugcagcaa.................................................    1          0          ku3
................................................ugcccauacuuucaccccucucu..........    1          0          ku3

.........agguggugggaaguaugcagcaa.................................................    1          0          j09

..................................................cccauacuuucaccccucu............    1          0          ku6

..................................................cccauacuuucaccccucu............    1          0          NL3
..................................................cccauacuuucaccccucucu..........    1          0          NL3

................................................ugcccauacuuucaccccu..............    1          0          ya2

........gagguggugggaaguaugcagcaagu...............................................    1          0          s12
.........agguggugggaaguaugcagcaagu...............................................    1          0          s12

.......agagguggugggaaguaug.......................................................    1          0          ya3
.......agagguggugggaaguaugcagc...................................................    1          0          ya3
...............................................cugcccauacuuucaccccucu............    1          0          ya3

................................................ugcccauacuuucaccccucucu..........    1          0          k06

..................................................cccauacuuucaccccucucu..........    1          0          HEK

.......agagguggugggaaguaugcag....................................................    1          0          j29
.......agagguggugggaaguaugcagc...................................................    1          0          j29

.........agguggugggaaguaugcagcaagu...............................................    1          0          la1

.......agagguggugggaaguaugca.....................................................    1          0          j17
.......agagguggugggaaguaugcagca..................................................    1          0          j17
...............................................cugcccauacuuucaccccucu............    1          0          j17
..................................................cccauacuuucaccccucucu..........    1          0          j17

.......agagguggugggaaguaugcagca..................................................    1          0          j15
........gagguggugggaaguaugcagc...................................................    1          0          j15
........gagguggugggaaguaugcagca..................................................    1          0          j15
.........agguggugggaaguaugcag....................................................    1          0          j15

...............................................cugcccauacuuucaccccucu............    1          0          MCF
..................................................cccauacuuucaccccucucu..........    2          0          MCF

.........agguggugggaaguaugcagcaagu...............................................    1          0          NL1

..................................................cccauacuuucaccccucucu..........    1          0          k07

.......agagguggugggaaguaugcagca..................................................    1          0          j12

...............................................cugcccauacuuucaccccucu............    3          0          va4
..................................................cccauacuuucaccccucucu..........    2          0          va4

...............................................cugcccauacuuucaccccucu............    1          0          va3
..................................................cccauacuuucaccccucuc...........    1          0          va3

..................................................cccauacuuucaccccucucu..........    3          0          A54

.......agagguggugggaaguaugcagc...................................................    1          0          ya4
...................................................ccauacuuucaccccucucu..........    1          0          ya4

..................................................cccauacuuucaccccucucu..........    1          0          ku1
```

Provisional ID	:	hsa_can_163
Score total	:	3.2
Score for star read(s)	:	-1.3
Score for read counts	:	0
Score for mfe	:	1.5
Score for randfold	:	
Score for cons. seed	:	3
Total read count	:	2910
Mature read count	:	2407
Loop read count	:	0
Star read count	:	503

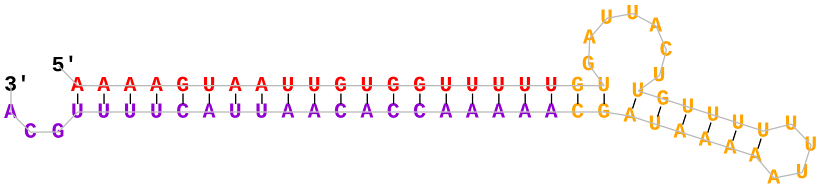

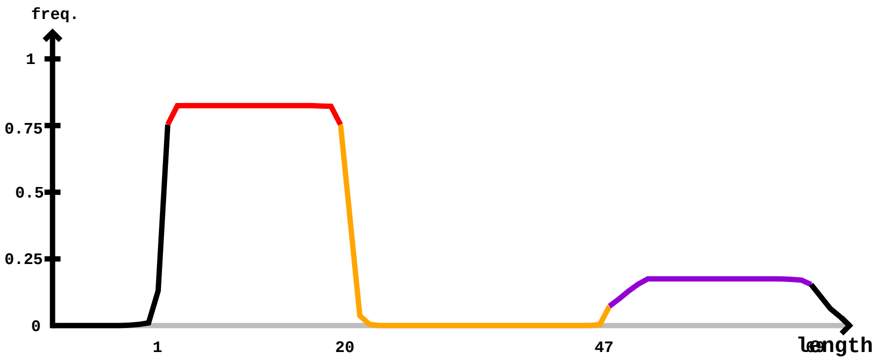

Mature Star

```
5'- agguuagugcaaaaguaauugugguuuuugugauuacuuguuuuuuuaaaaauagcaaaaaccacaauuacuuuugcacca  -3'   obs
    agguuagugcaaaaguaauugugguuuuugugauuacuuguuuuuuuaaaaauagcaaaaaccacaauuacuuuugcacca        exp
    agguuagugcaaaaguaauugugguuuuugugauuacuuguuuuuuuaaaaauagcaaaaaccacaauuacuuuugcacca        known
    ......(((((((((((((((((((((((((.......((((((....)))))))))))))))))))))))))))))))..    reads   mm    sample
    .........caaaaguaauugugguuuu.....................................................    5       0     ya1
    .........caaaaguaauugugguuuuu....................................................    22      0     ya1
    .........caaaaguaauugugguuuuug...................................................    1       0     ya1
    .........caaaaguaauugugguuuuugu..................................................    1       0     ya1
    ..........aaaaguaauugugguuuu.....................................................    16      0     ya1
    ..........aaaaguaauugugguuuuu....................................................    111     0     ya1
    ..........aaaaguaauugugguuuuug...................................................    8       0     ya1
    ...........aaaguaauugugguuuuu....................................................    19      0     ya1
    ...........aaaguaauugugguuuuugu..................................................    1       0     ya1
    ........................................................aaaaaccacaauuacuuuug.....    1       0     ya1
    ........................................................aaaaaccacaauuacuuuugca...    4       0     ya1
    .........................................................aaaaccacaauuacuuuugca...    3       0     ya1
    .........................................................aaaaccacaauuacuuuugcac..    1       0     ya1
    ..........................................................aaaccacaauuacuuuugca...    1       0     ya1
    ...........................................................aaccacaauuacuuuugcacc.    1       0     ya1

    ..........aaaaguaauugugguuuuug...................................................    1       0     s04
    ........................................................aaaaaccacaauuacuuuugca...    1       0     s04

    .........caaaaguaauugugguuuuugu..................................................    1       0     j26
    ..........aaaaguaauugugguuuuug...................................................    1       0     j26
    ........................................................aaaaaccacaauuacuuuugca...    3       0     j26
    ............................................................accacaauuacuuuugcacca    1       0     j26

    ..........aaaaguaauugugguuuuu....................................................    2       0     va1
    ..........aaaaguaauugugguuuuug...................................................    25      0     va1
    ..........aaaaguaauugugguuuuugu..................................................    2       0     va1
    ...........aaaguaauugugguuuuug...................................................    2       0     va1
    ........................................................aaaaaccacaauuacuuuugc....    1       0     va1
    ........................................................aaaaaccacaauuacuuuugca...    6       0     va1
    ........................................................aaaaaccacaauuacuuuugcac..    1       0     va1
    .........................................................aaaaccacaauuacuuuugcac..    5       0     va1
```

```
          Mature                                       Star
agguuagugcaaaaguaauugugguuuuugugauuacuuguuuuuuuaaaaauagcaaaaaccacaauuacuuuugcacca
..........................................................aaaccacaauuacuuuugcacc.    1    0    va1

......gugcaaaaguaauugugguu.......................................................    1    0    ku5
.........caaaaguaauugugguuuuug...................................................    1    0    ku5
..........aaaaguaauugugguuuuug...................................................    3    0    ku5
........................................................aaaaaccacaauuacuuuugca...    1    0    ku5
..........................................................aaaccacaauuacuuuugcacc.    1    0    ku5

.........caaaaguaauugugguuuuu....................................................    2    0    k05
.........caaaaguaauugugguuuuug...................................................    2    0    k05
.........caaaaguaauugugguuuuugu..................................................    3    0    k05
..........aaaaguaauugugguuuuug...................................................    3    0    k05
...........aaaguaauugugguuuuug...................................................    1    0    k05
..........................................................aaaccacaauuacuuuugcacc.    1    0    k05

.........caaaaguaauugugguuuuug...................................................    1    0    s06
........................................................aaaaaccacaauuacuuuugca...    1    0    s06
...........................................................aaccacaauuacuuuugcacca    1    0    s06
............................................................accacaauuacuuuugcacca    1    0    s06

..........aaaaguaauugugguuuuugu..................................................    1    0    HeL
........................................................aaaaaccacaauuacuuuugc....    1    0    HeL
........................................................aaaaaccacaauuacuuuugca...    3    0    HeL
.........................................................aaaaccacaauuacuuuugcac..    2    0    HeL
..........................................................aaaccacaauuacuuuugcac..    1    0    HeL
..........................................................aaaccacaauuacuuuugcacc.    3    0    HeL
...........................................................aaccacaauuacuuuugcacca    3    0    HeL
............................................................accacaauuacuuuugcacca    5    0    HeL

......gugcaaaaguaauugugguu.......................................................    1    0    ku4
..........aaaaguaauugugguuuuu....................................................    5    0    ku4
..........aaaaguaauugugguuuuug...................................................    11   0    ku4

..........................................................aaaccacaauuacuuuugcacc.    1    0    s07

..........aaaaguaauugugguuuuug...................................................    2    0    s10
..........aaaaguaauugugguuuuugu..................................................    1    0    s10
........................................................aaaaaccacaauuacuuuugca...    4    0    s10
..........................................................aaaccacaauuacuuuugcac..    1    0    s10

.........caaaaguaauugugguuuuu....................................................    2    0    k08
.........caaaaguaauugugguuuuug...................................................    1    0    k08
.........caaaaguaauugugguuuuugu..................................................    2    0    k08
..........aaaaguaauugugguuuuu....................................................    1    0    k08
..........aaaaguaauugugguuuuug...................................................    1    0    k08

..........aaaaguaauugugguuuu.....................................................    1    0    ta3
..........aaaaguaauugugguuuuug...................................................    7    0    ta3
...........aaaguaauugugguuuuug...................................................    1    0    ta3
........................................................aaaaaccacaauuacuuuugca...    4    0    ta3
.........................................................aaaaccacaauuacuuuugc....    1    0    ta3
.........................................................aaaaccacaauuacuuuugcac..    1    0    ta3
..........................................................aaaccacaauuacuuuugcac..    1    0    ta3
..........................................................aaaccacaauuacuuuugcacc.    1    0    ta3
...........................................................aaccacaauuacuuuugcacca    2    0    ta3

..........aaaaguaauugugguuuuu....................................................    2    0    j10
..........aaaaguaauugugguuuuug...................................................    6    0    j10
........................................................aaaaaccacaauuacuuuugca...    5    0    j10
..........................................................aaaccacaauuacuuuugcac..    1    0    j10
...........................................................aaccacaauuacuuuugcacca    1    0    j10

..........aaaaguaauugugguuuuug...................................................    3    0    j04

..........aaaaguaauugugguuuu.....................................................    1    0    j02
..........aaaaguaauugugguuuuu....................................................    5    0    j02
..........aaaaguaauugugguuuuug...................................................    22   0    j02
..........aaaaguaauugugguuuuugu..................................................    2    0    j02
...........aaaguaauugugguuuuug...................................................    1    0    j02

.........caaaaguaauugugguuuuu....................................................    1    0    va2
```

```
          Mature                                             Star
agguuagugcaaaaguaauugugguuuuugugauuacuuguuuuuuuaaaaauagcaaaaaccacaauuacuuuugcacca
.........caaaaguaauugugguuuuugu..................................................   1    0    va2
..........aaaaguaauugugguuuuu....................................................   1    0    va2
..........aaaaguaauugugguuuuug...................................................   4    0    va2
..........aaaaguaauugugguuuuugug.................................................   1    0    va2
........................................................aaaaaccacaauuacuuuugcac..   1    0    va2

.........caaaaguaauugugguuuuu....................................................   2    0    k14
.........caaaaguaauugugguuuuug...................................................   4    0    k14
.........caaaaguaauugugguuuuugu..................................................   1    0    k14
..........aaaaguaauugugguuuuu....................................................   2    0    k14
..........aaaaguaauugugguuuuug...................................................   4    0    k14
..........aaaaguaauugugguuuuugu..................................................   1    0    k14
...........aaaguaauugugguuuuug...................................................   1    0    k14
............aaguaauugugguuuuugu..................................................   1    0    k14
...........................................................aaccacaauuacuuuugcac..   1    0    k14

.........caaaaguaauugugguuuuu....................................................   1    0    j31
.........caaaaguaauugugguuuuug...................................................   2    0    j31
.........caaaaguaauugugguuuuugu..................................................   3    0    j31
..........aaaaguaauugugguuuuug...................................................   1    0    j31
........................................................aaaaaccacaauuacuuuugca...   1    0    j31

.........caaaaguaauugugguuuuu....................................................   1    0    j27
.........caaaaguaauugugguuuuug...................................................   4    0    j27
.........caaaaguaauugugguuuuugu..................................................   2    0    j27
..........aaaaguaauugugguuuu.....................................................   1    0    j27
..........aaaaguaauugugguuuuu....................................................   2    0    j27
..........aaaaguaauugugguuuuug...................................................   13   0    j27
..........................................................aaaccacaauuacuuuugca...   1    0    j27

........................................................aaaaaccacaauuacuuuugca...   2    0    s03
.........................................................aaaaccacaauuacuuuugca...   1    0    s03
.........................................................aaaaccacaauuacuuuugcac..   2    0    s03

..........aaaaguaauugugguuuuu....................................................   2    0    H52
........................................................aaaaaccacaauuacuuuug.....   2    0    H52
........................................................aaaaaccacaauuacuuuugca...   1    0    H52
.........................................................aaaaccacaauuacuuuugc....   1    0    H52
.........................................................aaaaccacaauuacuuuugca...   2    0    H52
..........................................................aaaccacaauuacuuuugcacc.   2    0    H52
...........................................................aaccacaauuacuuuugcacc.   1    0    H52
...........................................................aaccacaauuacuuuugcacca   1    0    H52
............................................................accacaauuacuuuugcacca   1    0    H52

.........caaaaguaauugugguuuuu....................................................   1    0    j22
.........caaaaguaauugugguuuuug...................................................   1    0    j22
.........caaaaguaauugugguuuuugu..................................................   3    0    j22
..........aaaaguaauugugguuuuu....................................................   1    0    j22
..........aaaaguaauugugguuuuug...................................................   33   0    j22
..........aaaaguaauugugguuuuugu..................................................   3    0    j22
..........aaaaguaauugugguuuuugug.................................................   2    0    j22
...........aaaguaauugugguuuuug...................................................   3    0    j22
........................................................aaaaaccacaauuacuuuugca...   6    0    j22
.........................................................aaaaccacaauuacuuuugca...   1    0    j22
...........................................................aaccacaauuacuuuugcacc.   1    0    j22

.......ugcaaaaguaauugugguuuuu....................................................   1    0    k09
.........caaaaguaauugugguuuuu....................................................   1    0    k09
.........caaaaguaauugugguuuuug...................................................   2    0    k09
.........caaaaguaauugugguuuuugu..................................................   1    0    k09
..........aaaaguaauugugguuuuu....................................................   2    0    k09
..........aaaaguaauugugguuuuug...................................................   4    0    k09
..........aaaaguaauugugguuuuugu..................................................   2    0    k09
.........................................................aaaaccacaauuacuuuugcac..   1    0    k09

.........caaaaguaauugugguuuuug...................................................   3    0    B14
.........caaaaguaauugugguuuuugu..................................................   1    0    B14
........................................................aaaaaccacaauuacuuuugc....   3    0    B14
........................................................aaaaaccacaauuacuuuugca...   6    0    B14
.........................................................aaaaccacaauuacuuuugcac..   1    0    B14
```

Mature	Star

```
agguuagugcaaaaguaauugugguuuuugugauuacuuguuuuuuuaaaaauagcaaaaaccacaauuacuuuugcacca
..........................................................aaaccacaauuacuuuugca...	2	0	B14
..........................................................aaaccacaauuacuuuugcacc.	2	0	B14
............................................................accacaauuacuuuugcacca	2	0	B14

.........caaaaguaauugugguuuuug...................................................	1	0	ku3
.........caaaaguaauugugguuuuugu..................................................	1	0	ku3
..........aaaaguaauugugguuuuug...................................................	6	0	ku3
........................................................aaaaaccacaauuacuuuugcac..	1	0	ku3
..........................................................aaaccacaauuacuuuugcacca	1	0	ku3

.........caaaaguaauugugguuuuug...................................................	1	0	j16
..........aaaaguaauugugguuuu.....................................................	1	0	j16
..........aaaaguaauugugguuuuu....................................................	12	0	j16
..........aaaaguaauugugguuuuug...................................................	25	0	j16
........................................................aaaaaccacaauuacuuuugca...	4	0	j16

.........caaaaguaauugugguuuuu....................................................	2	0	j09
..........aaaaguaauugugguuuuu....................................................	4	0	j09
..........aaaaguaauugugguuuuug...................................................	9	0	j09
..........aaaaguaauugugguuuuugug.................................................	2	0	j09
...........aaaguaauugugguuuuug...................................................	1	0	j09
........................................................aaaaaccacaauuacuuuugca...	1	0	j09
............................................................accacaauuacuuuugcacca	1	0	j09

.........caaaaguaauugugguuuuu....................................................	3	0	j18
.........caaaaguaauugugguuuuug...................................................	1	0	j18
..........aaaaguaauugugguuuuug...................................................	1	0	j18
..........aaaaguaauugugguuuuugu..................................................	1	0	j18

.........caaaaguaauugugguuuu.....................................................	1	0	ku6
.........caaaaguaauugugguuuuu....................................................	1	0	ku6
.........caaaaguaauugugguuuuugu..................................................	1	0	ku6
..........aaaaguaauugugguuuuug...................................................	2	0	ku6
........................................................aaaaaccacaauuacuuuugca...	2	0	ku6

..........aaaaguaauugugguuuuu....................................................	4	0	j11
..........aaaaguaauugugguuuuug...................................................	7	0	j11
...........aaaguaauugugguuuuug...................................................	1	0	j11
........................................................aaaaaccacaauuacuuuugca...	2	0	j11

..........aaaaguaauugugguuuuug...................................................	4	0	s12
...........aaaguaauugugguuuuug...................................................	2	0	s12

........gcaaaaguaauugugguuuuu....................................................	2	0	ya3
.........caaaaguaauugugguuuu.....................................................	9	0	ya3
.........caaaaguaauugugguuuuu....................................................	22	0	ya3
.........caaaaguaauugugguuuuugu..................................................	4	0	ya3
..........aaaaguaauugugguuuu.....................................................	44	0	ya3
..........aaaaguaauugugguuuuu....................................................	238	0	ya3
..........aaaaguaauugugguuuuug...................................................	27	0	ya3
..........aaaaguaauugugguuuuugu..................................................	1	0	ya3
...........aaaguaauugugguuuuu....................................................	45	0	ya3
...........aaaguaauugugguuuuug...................................................	8	0	ya3
........................................................aaaaaccacaauuacuuuugca...	3	0	ya3
.........................................................aaaaccacaauuacuuuugca...	2	0	ya3
.........................................................aaaaccacaauuacuuuugcac..	5	0	ya3
..........................................................aaaccacaauuacuuuugca...	1	0	ya3
..........................................................aaaccacaauuacuuuugcacc.	1	0	ya3
...........................................................aaccacaauuacuuuugcacc.	2	0	ya3

.........caaaaguaauugugguuuuugu..................................................	2	0	HEK
.......................................................caaaaaccacaauuacuuuugc....	3	0	HEK
........................................................aaaaaccacaauuacuuuugc....	1	0	HEK
........................................................aaaaaccacaauuacuuuugca...	8	0	HEK
.........................................................aaaaccacaauuacuuuugca...	2	0	HEK
.........................................................aaaaccacaauuacuuuugcac..	3	0	HEK
..........................................................aaaccacaauuacuuuugca...	1	0	HEK
..........................................................aaaccacaauuacuuuugcac..	3	0	HEK
..........................................................aaaccacaauuacuuuugcacc.	3	0	HEK
...........................................................aaccacaauuacuuuugcacc.	2	0	HEK
```

Mature Star			
agguuagugcaaaaguaauugugguuuuugugauuacuuguuuuuuuaaaaauagcaaaaaccacaauuacuuuugcacca			
....aaccacaauuacuuuugcacca	4	0	HEK
....accacaauuacuuuugcacca	6	0	HEK
.........caaaaguaauugugguuuuu..	1	0	la1
.........caaaaguaauugugguuuuugu..	1	0	la1
..........aaaaguaauugugguuuu...	1	0	la1
..........aaaaguaauugugguuuuu..	1	0	la1
..........aaaaguaauugugguuuuug...	3	0	la1
....aaaaaccacaauuacuuuugca...	2	0	la1
....aaccacaauuacuuuugcacc.	1	0	la1
..........aaaaguaauugugguuuu...	1	0	j17
..........aaaaguaauugugguuuuu..	8	0	j17
..........aaaaguaauugugguuuuug...	8	0	j17
....aaaaccacaauuacuuuugca...	1	0	j17
....aaccacaauuacuuuugcacca	2	0	j17
....accacaauuacuuuugcacca	1	0	j17
.........caaaaguaauugugguuuuug...	4	0	j15
..........aaaaguaauugugguuuu...	1	0	j15
..........aaaaguaauugugguuuuu..	3	0	j15
..........aaaaguaauugugguuuuug...	16	0	j15
...........aaaguaauugugguuuuu..	1	0	j15
...........aaaguaauugugguuuuug...	1	0	j15
....aaaaccacaauuacuuuugca...	1	0	j15
..........aaaaguaauugugguuuuu..	1	0	la2
..........aaaaguaauugugguuuuug...	3	0	la2
....aaaaccacaauuacuuuugcac..	1	0	la2
....aaaccacaauuacuuuugca...	1	0	la2
....aaaccacaauuacuuuugcacc.	1	0	la2
....aaccacaauuacuuuugcacca	1	0	la2
.........caaaaguaauugugguuuuug...	5	0	j12
..........aaaaguaauugugguuuuu..	6	0	j12
..........aaaaguaauugugguuuuug...	24	0	j12
..........aaaaguaauugugguuuuugug...	1	0	j12
...........aaaguaauugugguuuuug...	2	0	j12
....accacaauuacuuuugcacca	1	0	j12
.........caaaaguaauugugguuuuug...	4	0	li2
.........caaaaguaauugugguuuuug...	1	0	va4
..........aaaaguaauugugguuuuu..	1	0	va4
..........aaaaguaauugugguuuuug...	5	0	va4
..........aaaaguaauugugguuuuugu..	1	0	va4
....aaaaaccacaauuacuuuugca...	3	0	va4
....aaaaaccacaauuacuuuugcac..	1	0	va4
....aaaaccacaauuacuuuugcac..	1	0	va4
....aaccacaauuacuuuugcacc.	2	0	va4
....accacaauuacuuuugcacca	4	0	va4
.........caaaaguaauugugguuuuug...	2	0	va3
..........aaaaguaauugugguuuuu..	2	0	va3
..........aaaaguaauugugguuuuug...	10	0	va3
...........aaaguaauugugguuuuug...	2	0	va3
....aaaaaccacaauuacuuuugca...	12	0	va3
....aaaaccacaauuacuuuugcac..	2	0	va3
....aaaccacaauuacuuuugcac..	1	0	va3
....aaaccacaauuacuuuugcacc.	4	0	va3
....aaccacaauuacuuuugcacc.	2	0	va3
....aaccacaauuacuuuugcacca	2	0	va3
....accacaauuacuuuugcacca	1	0	va3
.........caaaaguaauugugguuuu...	16	0	ya4
.........caaaaguaauugugguuuuu..	16	0	ya4
.........caaaaguaauugugguuuuug...	4	0	ya4
..........aaaaguaauugugguuuu...	33	0	ya4
..........aaaaguaauugugguuuuu..	213	0	ya4
..........aaaaguaauugugguuuuug...	5	0	ya4

Mature **Star**

```
agguuagugcaaaaguaauugugguuuuugugauuacuuguuuuuuuaaaaauagcaaaaaccacaauuacuuuugcacca
..........aaaaguaauugugguuuuugu..................................................   1    0    ya4
...........aaaguaauugugguuuuu....................................................   43   0    ya4
...........aaaguaauugugguuuuug...................................................   3    0    ya4
........................................................aaaaaccacaauuacuuuugca...   4    0    ya4
.........................................................aaaaccacaauuacuuuugca...   2    0    ya4
.........................................................aaaaccacaauuacuuuugcac..   4    0    ya4
..........................................................aaaccacaauuacuuuugcac..   1    0    ya4
..........................................................aaaccacaauuacuuuugcacc.   4    0    ya4
...........................................................aaccacaauuacuuuugca...   3    0    ya4
...........................................................aaccacaauuacuuuugcacc.   4    0    ya4
...........................................................aaccacaauuacuuuugcacca   2    0    ya4

.........caaaaguaauugugguuuuu....................................................   1    0    SW4
.........caaaaguaauugugguuuuugu..................................................   1    0    SW4
..........aaaaguaauugugguuuuu....................................................   1    0    SW4
..........aaaaguaauugugguuuuug...................................................   3    0    SW4
..........aaaaguaauugugguuuuugu..................................................   2    0    SW4
........................................................aaaaaccacaauuacuuuugca...   1    0    SW4
.........................................................aaaaccacaauuacuuuugcac..   1    0    SW4
..........................................................aaaccacaauuacuuuugcac..   2    0    SW4
..........................................................aaaccacaauuacuuuugcacc.   3    0    SW4
...........................................................aaccacaauuacuuuugcacca   3    0    SW4
............................................................accacaauuacuuuugcacc.   1    0    SW4
............................................................accacaauuacuuuugcacca   3    0    SW4

.........caaaaguaauugugguuuuu....................................................   1    0    ku1
.........caaaaguaauugugguuuuug...................................................   1    0    ku1
.........caaaaguaauugugguuuuugu..................................................   1    0    ku1
..........aaaaguaauugugguuuuu....................................................   5    0    ku1
..........aaaaguaauugugguuuuug...................................................   38   0    ku1
...........aaaguaauugugguuuuug...................................................   1    0    ku1
........................................................aaaaaccacaauuacuuuugca...   4    0    ku1
...........................................................aaccacaauuacuuuugcacc.   1    0    ku1

.......ugcaaaaguaauugugguuuuu....................................................   1    0    j23
.........caaaaguaauugugguuuuug...................................................   4    0    j23
.........caaaaguaauugugguuuuugu..................................................   1    0    j23
..........aaaaguaauugugguuuu.....................................................   1    0    j23
..........aaaaguaauugugguuuuu....................................................   8    0    j23
..........aaaaguaauugugguuuuug...................................................   89   0    j23
..........aaaaguaauugugguuuuugu..................................................   2    0    j23
..........aaaaguaauugugguuuuugug.................................................   4    0    j23
...........aaaguaauugugguuuuug...................................................   11   0    j23
........................................................aaaaaccacaauuacuuuugc....   1    0    j23
........................................................aaaaaccacaauuacuuuugca...   2    0    j23
.........................................................aaaaccacaauuacuuuugca...   1    0    j23
...........................................................aaccacaauuacuuuugcacca   1    0    j23
............................................................accacaauuacuuuugcacca   1    0    j23

.........caaaaguaauugugguuuuugu..................................................   1    0    s05
..........aaaaguaauugugguuuuug...................................................   7    0    s05
...........aaaguaauugugguuuuug...................................................   1    0    s05
........................................................aaaaaccacaauuacuuuugca...   2    0    s05

...........aaaguaauugugguuuuug...................................................   1    0    ta2
........................................................aaaaaccacaauuacuuu.......   1    0    ta2
.........................................................aaaaccacaauuacuuuugcac..   1    0    ta2

.........caaaaguaauugugguuuu.....................................................   3    0    j24
.........caaaaguaauugugguuuuug...................................................   1    0    j24
.........caaaaguaauugugguuuuugu..................................................   2    0    j24
..........aaaaguaauugugguuuuu....................................................   3    0    j24
..........aaaaguaauugugguuuuug...................................................   8    0    j24

.........caaaaguaauugugguuuuug...................................................   1    0    k11
.........caaaaguaauugugguuuuugu..................................................   2    0    k11
..........aaaaguaauugugguuuuu....................................................   2    0    k11
..........aaaaguaauugugguuuuug...................................................   2    0    k11
........................................................aaaaaccacaauuacuuuugca...   1    0    k11

..........aaaaguaauugugguuuu.....................................................   1    0    ta1
```

Mature Star

```
agguuagugcaaaaguaauugugguuuuugugauuacuuguuuuuuaaaaauagcaaaaaccacaauuacuuuugcacca
..........aaaaguaauugugguuuuu...................................................    1    0    ta1
..........aaaaguaauugugguuuuug..................................................    3    0    ta1
...........aaaguaauugugguuuuug..................................................    1    0    ta1
.......................................................aaaaaccacaauuacuuuugca...    1    0    ta1

.........caaaaguaauugugguuuuug..................................................    1    0    s09
..........aaaaguaauugugguuuuu...................................................    1    0    s09
..........aaaaguaauugugguuuuug..................................................    3    0    s09
...........aaaguaauugugguuuuug..................................................    1    0    s09
......................................................caaaaaccacaauuacuuuugc....    1    0    s09
........................................................aaaaccacaauuacuuuugca...    1    0    s09
..........................................................aaccacaauuacuuuugcacca    1    0    s09
...........................................................accacaauuacuuuugcacca    1    0    s09

.........caaaaguaauugugguuu.....................................................    1    0    NL2
.........caaaaguaauugugguuuu....................................................    1    0    NL2
.........caaaaguaauugugguuuuu...................................................    4    0    NL2
.........caaaaguaauugugguuuuugu.................................................    1    0    NL2
..........aaaaguaauugugguuuu....................................................    10   0    NL2
..........aaaaguaauugugguuuuu...................................................    2    0    NL2
..........aaaaguaauugugguuuuug..................................................    2    0    NL2
..........aaaaguaauugugguuuuugu.................................................    2    0    NL2
......................................................caaaaaccacaauuacuuuu......    1    0    NL2
.......................................................aaaaaccacaauuacuuuugca...    3    0    NL2
..........................................................aaccacaauuacuuuugcacc.    1    0    NL2
..........................................................aaccacaauuacuuuugcacca    5    0    NL2

.........caaaaguaauugugguuuuu...................................................    1    0    k10
.........caaaaguaauugugguuuuug..................................................    2    0    k10
.........caaaaguaauugugguuuuugu.................................................    2    0    k10
..........aaaaguaauugugguuuuug..................................................    4    0    k10

.........caaaaguaauugugguuuuug..................................................    1    0    j13
..........aaaaguaauugugguuuu....................................................    1    0    j13
..........aaaaguaauugugguuuuu...................................................    6    0    j13
..........aaaaguaauugugguuuuug..................................................    33   0    j13
...........aaaguaauugugguuuuug..................................................    1    0    j13
.......................................................aaaaaccacaauuacuuuugca...    4    0    j13
........................................................aaaaccacaauuacuuuugca...    2    0    j13
..........................................................aaccacaauuacuuuugcacca    2    0    j13

.........caaaaguaauugugguuuuu...................................................    2    0    j19
.........caaaaguaauugugguuuuug..................................................    1    0    j19
.........caaaaguaauugugguuuuugu.................................................    1    0    j19
..........aaaaguaauugugguuuu....................................................    2    0    j19
..........aaaaguaauugugguuuuu...................................................    5    0    j19
..........aaaaguaauugugguuuuug..................................................    37   0    j19
.......................................................aaaaaccacaauuacuuuugca...    2    0    j19
.........................................................aaaccacaauuacuuuugca...    2    0    j19
..........................................................aaccacaauuacuuuugcacca    1    0    j19
...........................................................accacaauuacuuuugcacca    2    0    j19

..........aaaaguaauugugguuuuug..................................................    4    0    j03

.......ugcaaaaguaauugugguu......................................................    2    0    j25
.........caaaaguaauugugguuu.....................................................    1    0    j25
..........aaaaguaauugugguuuuug..................................................    10   0    j25
.......................................................aaaaaccacaauuacuuuugca...    1    0    j25

.........caaaaguaauugugguuuuugu.................................................    1    0    k01
..........aaaaguaauugugguuuuug..................................................    1    0    k01
..........aaaaguaauugugguuuuugu.................................................    1    0    k01

........gcaaaaguaauugugguuuuug..................................................    1    0    s01
.......................................................aaaaaccacaauuacuuuugca...    2    0    s01
........................................................aaaaccacaauuacuuuugcac..    2    0    s01
.........................................................aaaccacaauuacuuuugcacc.    2    0    s01

........gcaaaaguaauugugguuuuug..................................................    1    0    per
.........caaaaguaauugugguuuuug..................................................    1    0    per
```

```
          Mature                                        Star
agguuagugcaaaaguaauugugguuuuugugauuacuuguuuuuuuaaaaauagcaaaaaccacaauuacuuuugcacca
.........caaaaguaauugugguuuuugu..................................................    3    0    per
..........aaaaguaauugugguuuu.....................................................    1    0    per
..........aaaaguaauugugguuuuug...................................................    8    0    per
...........aaaguaauugugguuuuug...................................................    1    0    per

.........caaaaguaauugugguuuuu....................................................    2    0    k04
.........caaaaguaauugugguuuuug...................................................    1    0    k04
.........caaaaguaauugugguuuuugu..................................................    2    0    k04
..........aaaaguaauugugguuuu.....................................................    1    0    k04
..........aaaaguaauugugguuuuug...................................................    5    0    k04
..........aaaaguaauugugguuuuugu..................................................    1    0    k04
.........................................................aaaaccacaauuacuuuugca...    1    0    k04

......gugcaaaaguaauugugguuuu.....................................................    1    0    U20
.........caaaaguaauugugguuuuug...................................................    1    0    U20
.........caaaaguaauugugguuuuugu..................................................    1    0    U20
..........aaaaguaauugugguuuuu....................................................    1    0    U20
..........aaaaguaauugugguuuuug...................................................    6    0    U20
..........aaaaguaauugugguuuuugug.................................................    1    0    U20
...........aaaguaauugugguuuuugu..................................................    1    0    U20
.......................................................caaaaaccacaauuacuuuugc....    1    0    U20
........................................................aaaaaccacaauuacuuuugc....    22   0    U20
........................................................aaaaaccacaauuacuuuugca...    17   0    U20
.........................................................aaaaccacaauuacuuuugca...    1    0    U20
.........................................................aaaaccacaauuacuuuugcac..    8    0    U20
..........................................................aaaccacaauuacuuuugcac..    6    0    U20
..........................................................aaaccacaauuacuuuugcacc.    14   0    U20
...........................................................aaccacaauuacuuuugcacc.    6    0    U20
...........................................................aaccacaauuacuuuugcacca    1    0    U20
............................................................accacaauuacuuuugcacca    15   0    U20

.........caaaaguaauugugguuuuu....................................................    2    0    ku2
.........caaaaguaauugugguuuuugu..................................................    2    0    ku2
..........aaaaguaauugugguuuuu....................................................    5    0    ku2
..........aaaaguaauugugguuuuug...................................................    16   0    ku2

.........caaaaguaauugugguuuuu....................................................    1    0    j21
.........caaaaguaauugugguuuuug...................................................    3    0    j21
..........aaaaguaauugugguuuuu....................................................    1    0    j21
..........aaaaguaauugugguuuuug...................................................    14   0    j21
...........aaaguaauugugguuuuug...................................................    3    0    j21
........................................................aaaaaccacaauuacuuuugca...    3    0    j21
.........................................................aaaaccacaauuacuuuugca...    1    0    j21
..........................................................aaaccacaauuacuuuugcacc.    1    0    j21


..........aaaaguaauugugguuuuug...................................................    1    0    s11
...........................................................aaccacaauuacuuuugcacc.    1    0    s11
...........................................................aaccacaauuacuuuugcacca    1    0    s11

..........aaaaguaauugugguuuuu....................................................    4    0    j07
..........aaaaguaauugugguuuuug...................................................    2    0    j07
...........aaaguaauugugguuuuug...................................................    1    0    j07
........................................................aaaaaccacaauuacuuuugca...    1    0    j07

.........caaaaguaauugugguuuuug...................................................    5    0    j14
..........aaaaguaauugugguuuuug...................................................    9    0    j14
..........aaaaguaauugugguuuuugu..................................................    1    0    j14
..........................................................aaaccacaauuacuuuugcacc.    1    0    j14

.........caaaaguaauugugguuuuug...................................................    2    0    k02
.........caaaaguaauugugguuuuugu..................................................    1    0    k02
..........aaaaguaauugugguuuuu....................................................    2    0    k02

.........caaaaguaauugugguuuuu....................................................    1    0    j28
..........aaaaguaauugugguuuuu....................................................    3    0    j28
..........aaaaguaauugugguuuuug...................................................    17   0    j28

..........aaaaguaauugugguuuuug...................................................    1    0    s08
........................................................aaaaaccacaauuacuuuugca...    1    0    s08
```

Mature **Star**

```
agguuagugcaaaaguaauugugguuuuugugauuacuuguuuuuuuaaaaauagcaaaaaccacaauuacuuuugcacca
............................................................accacaauuacuuuugcac..   1     0    s08

.........caaaaguaauugugguuuuugu..................................................   1     0    DLD
..........aaaaguaauugugguuuuu....................................................   2     0    DLD
..........aaaaguaauugugguuuuug...................................................   1     0    DLD
........................................................aaaaaccacaauuacuuuugc....   1     0    DLD
.........................................................aaaaccacaauuacuuuugcac..   1     0    DLD
..........................................................aaaccacaauuacuuuugcacc.   3     0    DLD
............................................................accacaauuacuuuugcacc.   1     0    DLD
............................................................accacaauuacuuuugcacca   4     0    DLD

..........aaaaguaauugugguuuuug...................................................   5     0    k03
........................................................aaaaaccacaauuacuuuugca...   1     0    k03

.........caaaaguaauugugguuu......................................................   1     0    NL3
.........caaaaguaauugugguuuu.....................................................   1     0    NL3
.........caaaaguaauugugguuuuu....................................................   4     0    NL3
.........caaaaguaauugugguuuuug...................................................   2     0    NL3
..........aaaaguaauugugguuuu.....................................................   15    0    NL3
..........aaaaguaauugugguuuuu....................................................   6     0    NL3
..........aaaaguaauugugguuuuug...................................................   5     0    NL3
.......................................................caaaaaccacaauuacuuuu......   1     0    NL3
.......................................................caaaaaccacaauuacuuuug.....   2     0    NL3
........................................................aaaaaccacaauuacuuuugca...   7     0    NL3
.........................................................aaaaccacaauuacuuuugcac..   1     0    NL3
...........................................................aaccacaauuacuuuugcacca   1     0    NL3

.........caaaaguaauugugguuu......................................................   1     0    ya2
.........caaaaguaauugugguuuu.....................................................   6     0    ya2
.........caaaaguaauugugguuuuu....................................................   13    0    ya2
.........caaaaguaauugugguuuuug...................................................   2     0    ya2
..........aaaaguaauugugguuuu.....................................................   24    0    ya2
..........aaaaguaauugugguuuuu....................................................   188   0    ya2
..........aaaaguaauugugguuuuug...................................................   17    0    ya2
..........aaaaguaauugugguuuuugu..................................................   4     0    ya2
...........aaaguaauugugguuuuu....................................................   43    0    ya2
...........aaaguaauugugguuuuug...................................................   4     0    ya2
........................................................aaaaaccacaauuacuuuu......   2     0    ya2
........................................................aaaaaccacaauuacuuuugc....   1     0    ya2
........................................................aaaaaccacaauuacuuuugca...   2     0    ya2
.........................................................aaaaccacaauuacuuuugca...   6     0    ya2
..........................................................aaaccacaauuacuuuugcac..   3     0    ya2
..........................................................aaaccacaauuacuuuugcacca   1     0    ya2
...........................................................aaccacaauuacuuuugcacca   2     0    ya2

.........caaaaguaauugugguuuuug...................................................   1     0    s02

.........caaaaguaauugugguuuuug...................................................   3     0    k06
..........aaaaguaauugugguuuuug...................................................   2     0    k06
..........................................................aaaccacaauuacuuuugcacc.   1     0    k06

........gcaaaaguaauugugguuuuug...................................................   1     0    j29
.........caaaaguaauugugguuuuug...................................................   3     0    j29
.........caaaaguaauugugguuuuugu..................................................   2     0    j29
..........aaaaguaauugugguuuuug...................................................   10    0    j29
........................................................aaaaaccacaauuacuuuugca...   1     0    j29
.........................................................aaaaccacaauuacuuuugca...   1     0    j29

......gugcaaaaguaauugugguu.......................................................   1     0    sh2
.........caaaaguaauugugguuuuu....................................................   1     0    sh2
..........aaaaguaauugugguuuuug...................................................   1     0    sh2
.......................................................caaaaaccacaauuacuuuug.....   1     0    sh2

.......ugcaaaaguaauugugguuuu.....................................................   3     0    j01
.........caaaaguaauugugguuuuug...................................................   1     0    j01
..........aaaaguaauugugguuuuug...................................................   5     0    j01

.........caaaaguaauugugguuuuugu..................................................   1     0    MCF
..........aaaaguaauugugguuuuug...................................................   4     0    MCF
........................................................aaaaaccacaauuacuuuu......   1     0    MCF
```

```
          Mature                                         Star
agguuagugcaaaaguaauugugguuuuugugauuacuuguuuuuuuaaaaauagcaaaaaccacaauuacuuuugcacca
........................................................aaaaaccacaauuacuuuugc....    1    0    MCF
........................................................aaaaaccacaauuacuuuugca...    2    0    MCF
.........................................................aaaaccacaauuacuuuugcac..    1    0    MCF

..........aaaaguaauugugguuuuug...................................................    2    0    MBM
........................................................aaaaaccacaauuacuuuug.....    1    0    MBM
........................................................aaaaaccacaauuacuuuugc....    1    0    MBM
........................................................aaaaaccacaauuacuuuugca...    2    0    MBM
.........................................................aaaaccacaauuacuuuugca...    1    0    MBM
.........................................................aaaaccacaauuacuuuugcac..    3    0    MBM
..........................................................aaaccacaauuacuuuugcacc.    4    0    MBM
...........................................................aaccacaauuacuuuugcacc.    1    0    MBM
............................................................accacaauuacuuuugcacca    4    0    MBM

..........aaaaguaauugugguuuuu....................................................    2    0    NL1
..........aaaaguaauugugguuuuug...................................................    2    0    NL1

.......ugcaaaaguaauugugguuuuu....................................................    1    0    k07
.........caaaaguaauugugguuuuug...................................................    9    0    k07
.........caaaaguaauugugguuuuugu..................................................    3    0    k07
..........aaaaguaauugugguuuuug...................................................    3    0    k07

.......ugcaaaaguaauugugguuuuu....................................................    1    0    sh1
.........caaaaguaauugugguuuuug...................................................    6    0    sh1
..........aaaaguaauugugguuuuug...................................................    4    0    sh1

.........caaaaguaauugugguuuuug...................................................    3    0    li1
..........aaaaguaauugugguuuuugug.................................................    2    0    li1
......................................................gcaaaaaccacaauuacuuuug.....    1    0    li1
........................................................aaaaaccacaauuacuuuugc....    1    0    li1
.........................................................aaaaccacaauuacuuuugcac..    1    0    li1
..........................................................aaaccacaauuacuuuugcacc.    2    0    li1
...........................................................aaccacaauuacuuuugcacca    1    0    li1

.........caaaaguaauugugguuuuu....................................................    1    0    k13
.........caaaaguaauugugguuuuug...................................................    4    0    k13
.........caaaaguaauugugguuuuugu..................................................    3    0    k13
..........aaaaguaauugugguuuuug...................................................    1    0    k13

.........caaaaguaauugugguuuu.....................................................    1    0    j20
.........caaaaguaauugugguuuuu....................................................    2    0    j20
.........caaaaguaauugugguuuuug...................................................    7    0    j20
..........aaaaguaauugugguuuuu....................................................    3    0    j20
..........aaaaguaauugugguuuuug...................................................    27   0    j20
..........aaaaguaauugugguuuuugug.................................................    1    0    j20

........gcaaaaguaauugugguuuuug...................................................    11   0    A54
.........caaaaguaauugugguuuuugu..................................................    1    0    A54
..........aaaaguaauugugguuuuug...................................................    3    0    A54
........................................................aaaaaccacaauuacuuuugc....    7    0    A54
...........................................................aaccacaauuacuuuugcacc.    4    0    A54
...........................................................aaccacaauuacuuuugcacca    4    0    A54

..........aaaaguaauugugguuuuug...................................................    2    0    j30

.........caaaaguaauugugguuuuu....................................................    8    0    k12
.........caaaaguaauugugguuuuug...................................................    12   0    k12
..........aaaaguaauugugguuuuu....................................................    6    0    k12
..........aaaaguaauugugguuuuug...................................................    18   0    k12
..........aaaaguaauugugguuuuugu..................................................    2    0    k12
............................................................accacaauuacuuuugcacca    1    0    k12
```

Provisional ID	:	hsa_can_172
Score total	:	742.7
Score for star read(s)	:	3.9
Score for read counts	:	733.8
Score for mfe	:	2.1
Score for randfold	:	
Score for cons. seed	:	3
Total read count	:	1451
Mature read count	:	1450
Loop read count	:	0
Star read count	:	1

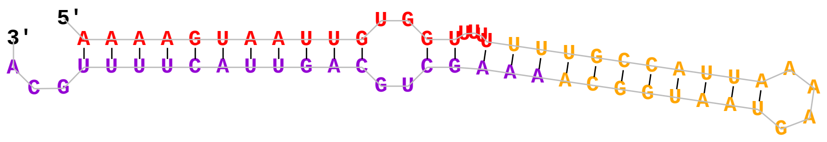

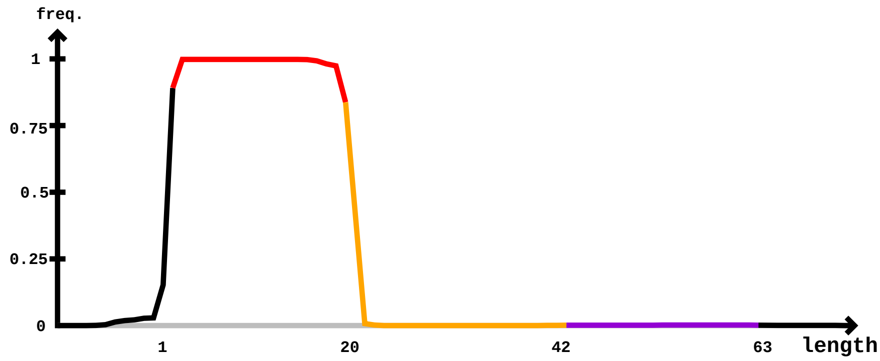

Mature Star

```
5'- aaguuggugcaaaaguaauugugguuuuuuuugccauuaaaaguaauggcaaaagcugcaguuacuuuugcaccaaccuaa   -3'   obs
    aaguuggugcaaaaguaauugugguuuuuuuugccauuaaaaguaauggcaaaagcugcaguuacuuuugcaccaaccuaa         exp
    ..((((((((((((((((((..((...((((((((((((....))))))))))))..))))))))))))))))))))....    reads    mm    sample
    .........caaaaguaauugugguuuu.....................................................    5        0     ya1
    .........caaaaguaauugugguuuuu....................................................    22       0     ya1
    ..........aaaaguaauugugguuuu.....................................................    16       0     ya1
    ..........aaaaguaauugugguuuuu....................................................    111      0     ya1
    ..........aaaaguaauugugguuuuuu...................................................    4        0     ya1
    ...........aaaguaauugugguuuuu....................................................    19       0     ya1

    ....uggugcaaaaguaauugugguu.......................................................    1        0     va1
    ..........aaaaguaauugugguuuuu....................................................    2        0     va1
    ..........aaaaguaauugugguuuuuu...................................................    1        0     va1
    ..........aaaaguaauugugguuuuuuu..................................................    1        0     va1
    ...........aaaguaauugugguuuuuu...................................................    1        0     va1

    ...uuggugcaaaaguaauugugguuu.......................................................    1        0     ku5
    ....uggugcaaaaguaauuguggu........................................................    1        0     ku5
    ......gugcaaaaguaauugugguu.......................................................    1        0     ku5

    .........caaaaguaauugugguuuuu....................................................    2        0     k05
    .........caaaaguaauugugguuuuuu...................................................    1        0     k05
    .........caaaaguaauugugguuuuuuu..................................................    1        0     k05

    ..........aaaaguaauugugguuuuuuu..................................................    1        0     HeL

    ......gugcaaaaguaauugugguu.......................................................    1        0     ku4
    ..........aaaaguaauugugguuuuu....................................................    5        0     ku4

    .........caaaaguaauugugguuuuu....................................................    2        0     k08
    ..........aaaaguaauugugguuuuu....................................................    1        0     k08
    ..........aaaaguaauugugguuuuuu...................................................    1        0     k08

    ..........aaaaguaauugugguuuu.....................................................    1        0     ta3
    ..........aaaaguaauugugguuuuuu...................................................    2        0     ta3

    ..........aaaaguaauugugguuuuu....................................................    2        0     j10
```

Mature Star

```
aaguuggugcaaaaguaauugugguuuuuuuugccauuaaaaguaauggcaaaagcugcaguuacuuuugcaccaaccuaa

..........aaaaguaauugugguuuu.....................................................   1     0    j02
..........aaaaguaauugugguuuuu....................................................   5     0    j02
..........aaaaguaauugugguuuuuu...................................................   3     0    j02
..........aaaaguaauugugguuuuuuuu.................................................   1     0    j02

.........caaaaguaauugugguuuuu....................................................   2     0    k14
..........aaaaguaauugugguuuuu....................................................   2     0    k14

.........caaaaguaauugugguuuuu....................................................   1     0    va2
..........aaaaguaauugugguuuuu....................................................   1     0    va2

.....ggugcaaaaguaauugugguuu......................................................   1     0    j27
.........caaaaguaauugugguuuuu....................................................   1     0    j27
..........aaaaguaauugugguuuu.....................................................   1     0    j27
..........aaaaguaauugugguuuuu....................................................   2     0    j27

...uuggugcaaaaguaauuguggu........................................................   1     0    j31
....uggugcaaaaguaauugugguu.......................................................   1     0    j31
.........caaaaguaauugugguuuuu....................................................   1     0    j31
.................................................caaaagcugcaguuacuuuugc..........   1     0    j31

..........aaaaguaauugugguuuuu....................................................   2     0    H52

.........caaaaguaauugugguuuuu....................................................   1     0    j22
..........aaaaguaauugugguuuuu....................................................   1     0    j22
..........aaaaguaauugugguuuuuu...................................................   2     0    j22

...uuggugcaaaaguaauugugg.........................................................   1     0    k09
.......ugcaaaaguaauugugguuuuu....................................................   1     0    k09
.........caaaaguaauugugguuuuu....................................................   1     0    k09
..........aaaaguaauugugguuuuu....................................................   2     0    k09
..........aaaaguaauugugguuuuuu...................................................   1     0    k09

.........caaaaguaauugugguuuuuu...................................................   3     0    B14
.........caaaaguaauugugguuuuuuuu.................................................   1     0    B14

..........aaaaguaauugugguuuu.....................................................   1     0    j16
..........aaaaguaauugugguuuuu....................................................   12    0    j16

.........caaaaguaauugugguuuuu....................................................   2     0    j09
.........caaaaguaauugugguuuuuu...................................................   1     0    j09
..........aaaaguaauugugguuuuu....................................................   4     0    j09

.........caaaaguaauugugguuuuu....................................................   3     0    j18

....uggugcaaaaguaauuguggu........................................................   1     0    ku3
..........aaaaguaauugugguuuuuu...................................................   1     0    ku3

.........caaaaguaauugugguuuu.....................................................   1     0    ku6
.........caaaaguaauugugguuuuu....................................................   1     0    ku6
..........aaaaguaauugugguuuuuuuu.................................................   1     0    ku6

..........aaaaguaauugugguuuuu....................................................   4     0    j11
..........aaaaguaauugugguuuuuu...................................................   1     0    j11

........gcaaaaguaauugugguuuuu....................................................   2     0    ya3
.........caaaaguaauugugguuuu.....................................................   9     0    ya3
.........caaaaguaauugugguuuuu....................................................   22    0    ya3
.........caaaaguaauugugguuuuuu...................................................   1     0    ya3
..........aaaaguaauugugguuuu.....................................................   44    0    ya3
..........aaaaguaauugugguuuuu....................................................   238   0    ya3
..........aaaaguaauugugguuuuuu...................................................   2     0    ya3
...........aaaguaauugugguuuuu....................................................   45    0    ya3
...........aaaguaauugugguuuuuu...................................................   4     0    ya3

.........caaaaguaauugugguuuuu....................................................   1     0    la1
..........aaaaguaauugugguuuu.....................................................   1     0    la1
..........aaaaguaauugugguuuuu....................................................   1     0    la1
..........aaaaguaauugugguuuuuu...................................................   1     0    la1
```

Mature Star

aaguuggugcaaaaguaauugugguuuuuuuugccauuaaaaguaauggcaaaagcugcaguuacuuuugcaccaaccuaa

....uggugcaaaaguaauugugguu...	1	0	HEK
..........aaaaguaauugugguuuu...	1	0	j17
..........aaaaguaauugugguuuuu..	8	0	j17
..........aaaaguaauugugguuuuuu...	1	0	j17
..........aaaaguaauugugguuuu...	1	0	j15
..........aaaaguaauugugguuuuu..	3	0	j15
...........aaaguaauugugguuuuu..	1	0	j15
..........aaaaguaauugugguuuuu..	1	0	la2
....uggugcaaaaguaauugugguu...	1	0	j12
..........aaaaguaauugugguuuuu..	6	0	j12
....uggugcaaaaguaauugugguu...	1	0	li2
..........aaaaguaauugugguuuuu..	1	0	va4
..........aaaaguaauugugguuuuuu...	2	0	va4
..........aaaaguaauugugguuuuu..	2	0	va3
..........aaaaguaauugugguuuuuu...	1	0	va3
.........caaaaguaauugugguuuuu..	1	0	SW4
..........aaaaguaauugugguuuuu..	1	0	SW4
..........aaaaguaauugugguuuuuu...	1	0	SW4
.........caaaaguaauugugguuuu...	16	0	ya4
.........caaaaguaauugugguuuuu..	16	0	ya4
..........aaaaguaauugugguuuu...	33	0	ya4
..........aaaaguaauugugguuuuu..	213	0	ya4
..........aaaaguaauugugguuuuuu...	5	0	ya4
...........aaaguaauugugguuuuu..	43	0	ya4
...........aaaguaauugugguuuuuu...	2	0	ya4
.........caaaaguaauugugguuuuu..	1	0	ku1
..........aaaaguaauugugguuuuu..	5	0	ku1
..........aaaaguaauugugguuuuuu...	1	0	ku1
.......ugcaaaaguaauugugguuuuu..	1	0	j23
..........aaaaguaauugugguuuu...	1	0	j23
..........aaaaguaauugugguuuuu..	8	0	j23
.....ggugcaaaaguaauugugguuu..	5	0	j24
.........caaaaguaauugugguuuu...	3	0	j24
..........aaaaguaauugugguuuuu..	3	0	j24
..........aaaaguaauugugguuuuu..	2	0	k11
..........aaaaguaauugugguuuu...	1	0	ta1
..........aaaaguaauugugguuuuu..	1	0	ta1
..........aaaaguaauugugguuuuu..	1	0	s09
.........caaaaguaauugugguuu..	1	0	NL2
.........caaaaguaauugugguuuu...	1	0	NL2
.........caaaaguaauugugguuuuu..	4	0	NL2
..........aaaaguaauugugguuuu...	10	0	NL2
..........aaaaguaauugugguuuuu..	2	0	NL2
....uggugcaaaaguaauugugguu...	1	0	k10
.........caaaaguaauugugguuuuu..	1	0	k10
..........aaaaguaauugugguuuu...	1	0	j13
..........aaaaguaauugugguuuuu..	6	0	j13
..........aaaaguaauugugguuuuuu...	7	0	j13
.........caaaaguaauugugguuuuu..	2	0	j19
..........aaaaguaauugugguuuu...	2	0	j19

```
          Mature                                  Star
aaguuggugcaaaaguaauugugguuuuuuuugccauuaaaaguaauggcaaaagcugcaguuacuuuugcaccaaccuaa
..........aaaaguaauugugguuuuu....................................................    5    0    j19

..........aaaaguaauugugguuuuuu...................................................    2    0    k01
..........aaaaguaauugugguuuuuuu..................................................    1    0    k01

....uggugcaaaaguaauugugguu.......................................................    1    0    j25
.......ugcaaaaguaauugugguu.......................................................    2    0    j25
.........caaaaguaauugugguuuu.....................................................    1    0    j25

.........caaaaguaauugugguuuuu....................................................    2    0    k04
..........aaaaguaauugugguuuu.....................................................    1    0    k04
..........aaaaguaauugugguuuuuu...................................................    1    0    k04

..........aaaaguaauugugguuuu.....................................................    1    0    per
..........aaaaguaauugugguuuuuu...................................................    2    0    per

....uggugcaaaaguaauuguggu........................................................    2    0    U20
....uggugcaaaaguaauugugguu.......................................................    1    0    U20
......gugcaaaaguaauugugguuuu.....................................................    1    0    U20
..........aaaaguaauugugguuuuu....................................................    1    0    U20
..........aaaaguaauugugguuuuuu...................................................    1    0    U20

.........caaaaguaauugugguuuuu....................................................    2    0    ku2
..........aaaaguaauugugguuuuu....................................................    5    0    ku2
..........aaaaguaauugugguuuuuu...................................................    2    0    ku2

.........caaaaguaauugugguuuuu....................................................    1    0    j21
..........aaaaguaauugugguuuuu....................................................    1    0    j21
..........aaaaguaauugugguuuuuu...................................................    1    0    j21

..........aaaaguaauugugguuuuuu...................................................    1    0    s11

..........aaaaguaauugugguuuuu....................................................    4    0    j07
..........aaaaguaauugugguuuuuu...................................................    1    0    j07

..........aaaaguaauugugguuuuu....................................................    2    0    k02

.........caaaaguaauugugguuuuuu...................................................    1    0    j14
..........aaaaguaauugugguuuuuu...................................................    1    0    j14

.........caaaaguaauugugguuuuu....................................................    1    0    j28
..........aaaaguaauugugguuuuu....................................................    3    0    j28

..........aaaaguaauugugguuuuu....................................................    2    0    DLD

..guuggugcaaaaguaauuguggu........................................................    1    0    k03
.........caaaaguaauugugguuuuuu...................................................    1    0    k03

.........caaaaguaauugugguuu......................................................    1    0    NL3
.........caaaaguaauugugguuuu.....................................................    1    0    NL3
.........caaaaguaauugugguuuuu....................................................    4    0    NL3
..........aaaaguaauugugguuuu.....................................................    15   0    NL3
..........aaaaguaauugugguuuuu....................................................    6    0    NL3

.........caaaaguaauugugguuu......................................................    1    0    ya2
.........caaaaguaauugugguuuu.....................................................    6    0    ya2
.........caaaaguaauugugguuuuu....................................................    13   0    ya2
.........caaaaguaauugugguuuuuu...................................................    1    0    ya2
..........aaaaguaauugugguuuu.....................................................    24   0    ya2
..........aaaaguaauugugguuuuu....................................................    188  0    ya2
..........aaaaguaauugugguuuuuu...................................................    3    0    ya2
..........aaaaguaauugugguuuuuuuu.................................................    1    0    ya2
...........aaaguaauugugguuuuu....................................................    43   0    ya2
...........aaaguaauugugguuuuuu...................................................    1    0    ya2

...uuggugcaaaaguaauugugguu.......................................................    1    0    sh2
......gugcaaaaguaauugugguu.......................................................    1    0    sh2
.........caaaaguaauugugguuuuu....................................................    1    0    sh2
.............................................................uuacuuuugcaccaaccua.    1    0    sh2

.....ggugcaaaaguaauugugguuu......................................................    1    0    j01
```

Mature Star

```
aaguuggugcaaaaguaauugugguuuuuuugccauuaaaaguaauggcaaaagcugcaguuacuuuugcaccaaccuaa
.......ugcaaaaguaauugugguuuu.....................................................   3   0   j01

....uggugcaaaaguaauuguggu........................................................   1   0   MCF
..........aaaaguaauugugguuuuuu...................................................   1   0   MCF

..........aaaaguaauugugguuuuu....................................................   2   0   NL1

.....ggugcaaaaguaauugugguu.......................................................   1   0   k07
.......ugcaaaaguaauugugguuuuu....................................................   1   0   k07
.........caaaaguaauugugguuuuuuu..................................................   1   0   k07
..........aaaaguaauugugguuuuuu...................................................   2   0   k07

.......ugcaaaaguaauugugguuuuu....................................................   1   0   sh1
.........caaaaguaauugugguuuuuu...................................................   2   0   sh1

....uggugcaaaaguaauugugguu.......................................................   1   0   li1
...................................................aaagcugcaguuacuuuugca.........   1   0   li1

.........caaaaguaauugugguuuuu....................................................   1   0   k13
.........caaaaguaauugugguuuuuuu..................................................   1   0   k13

.........caaaaguaauugugguuuu.....................................................   1   0   j20
.........caaaaguaauugugguuuuu....................................................   2   0   j20
..........aaaaguaauugugguuuuu....................................................   3   0   j20
...........aaaguaauugugguuuuuu...................................................   1   0   j20

.........caaaaguaauugugguuuuuuu..................................................   1   0   A54

....uggugcaaaaguaauugugguu.......................................................   1   0   j30

.........caaaaguaauugugguuuuu....................................................   8   0   k12
..........aaaaguaauugugguuuuu....................................................   6   0   k12
```

Provisional ID	:	hsa_can_202
Score total	:	33.5
Score for star read(s)	:	3.9
Score for read counts	:	29.2
Score for mfe	:	1.1
Score for randfold	:	
Score for cons. seed	:	-0.6
Total read count	:	69
Mature read count	:	56
Loop read count	:	0
Star read count	:	13

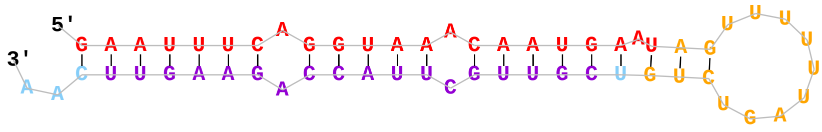

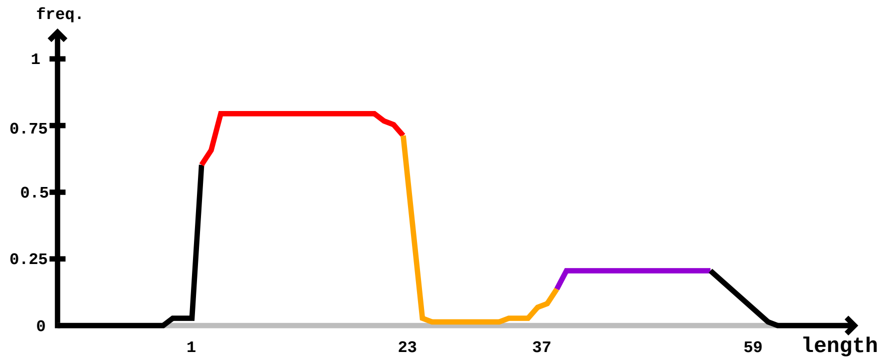


```
                 Mature                      Star
5'- cccaguuaaaauuugaauuucagguaaacaaugaauaguuuuuuagucugucguugcuuaccagaaguucaaauuuaucugg  -3'  obs
    cccaguuaaaauuugaauuucagguaaacaaugaauaguuuuuuagucugucguugcuuaccagaaguucaaauuuaucugg       exp
    .((((.((((((((((((((.(((((.((((((.(((..........))))))))).))))).)))))))))))))).))))   reads    mm    sample
    ...............aauuucagguaaacaaugaau..............................................   2        0     DLD

    ..............gaauuucagguaaacaauga................................................   1        0     B14
    ..............gaauuucagguaaacaaugaau..............................................   2        0     B14
    ...............aauuucagguaaacaaugaau..............................................   1        0     B14

    ....................................................guugcuuaccagaaguuca...........   1        0     j09

    ...............aauuucagguaaacaaugaauag............................................   1        0     ya2

    ..............gaauuucagguaaacaaugaaua.............................................   1        0     va1

    ..............gaauuucagguaaacaaugaau..............................................   1        0     s12
    .................................................gucguugcuuaccagaaguuca...........   1        0     s12
    ..................................................ucguugcuuaccagaaguuca...........   1        0     s12

    ..............gaauuucagguaaacaaugaau..............................................   1        0     HeL
    ................auuucagguaaacaaugaau..............................................   3        0     HeL
    ....................................................guugcuuaccagaaguucaaau........   1        0     HeL

    ....................................................guugcuuaccagaaguucaa..........   1        0     sh2

    ..............gaauuucagguaaacaaugaau..............................................   6        0     HEK
    ....................................................guugcuuaccagaaguucaa..........   1        0     HEK

    ..............gaauuucagguaaacaaugaau..............................................   2        0     s07

    ..............gaauuucagguaaacaaugaau..............................................   1        0     MCF

    ..............gaauuucagguaaacaaugaau..............................................   1        0     s10
    ...................................................cguugcuuaccagaaguucaa..........   1        0     s10

    ...........uuugaauuucagguaaacaaug.................................................   2        0     NL2
```

```
             Mature                                    Star
cccaguuaaauuugaauuucagguaaacaaugaauaguuuuuuagucugucguugcuuaccagaaguucaaauuuaucugg

.............gaauuucagguaaacaaugaau..............................................    1    0    li1
...................................aguuuuuuagucugucguugcuuaccagaaguucaa..........    1    0    li1
................................................gucguugcuuaccagaaguuca...........    1    0    li1

.............................................ucugucguugcuuaccagaaguu.............    1    0    va4
...................................................guugcuuaccagaaguucaa..........    1    0    va4

.............gaauuucagguaaacaaugaa...............................................    3    0    U20
.............gaauuucagguaaacaaugaau..............................................    15   0    U20
...............auuucagguaaacaaugaau..............................................    2    0    U20

.............gaauuucagguaaacaaugaau..............................................    1    0    j21

.............gaauuucagguaaacaaugaau..............................................    1    0    A54
...............auuucagguaaacaaugaau..............................................    5    0    A54
..................................................cguugcuuaccagaaguu.............    3    0    A54

................................................gucguugcuuaccagaaguuc............    1    0    j28

.............gaauuucagguaaacaaugaau..............................................    1    0    SW4

.............gaauuucagguaaacaaugaau..............................................    1    0    j30

.............gaauuucagguaaacaaugaaua.............................................    1    0    ya4

.............gaauuucagguaaacaaugaau..............................................    2    0    H52
```

Provisional ID	:	hsa_can_263
Score total	:	0
Score for star read(s)	:	-1.3
Score for read counts	:	0
Score for mfe	:	1.9
Score for randfold	:	
Score for cons. seed	:	-0.6
Total read count	:	606
Mature read count	:	606
Loop read count	:	0
Star read count	:	0

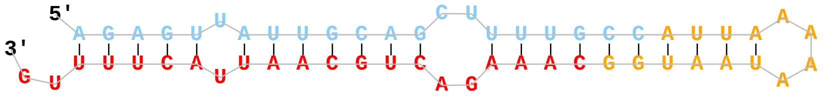

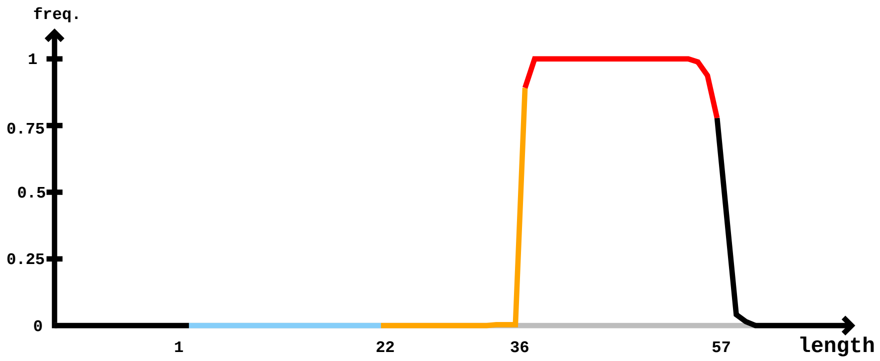

Star Mature

```
5'- uagguucgugcaagaguuauugcagcuuuugccauuaaaaaauaauggcaaagacugcaauuacuuuugcaacaaccuaaua  -3'   exp
    ((((((..(((((((((.(((((((..((((((((((....))))))))))..))))))).)))))))))..))))))...    reads   mm   sample
    ................................................caaagacugcaauuacuu................   3       0    ya1
    ................................................caaagacugcaauuacuuu...............   3       0    ya1
    ................................................caaagacugcaauuacuuuugc............   3       0    ya1
    .................................................aaagacugcaauuacuu................   3       0    ya1
    .................................................aaagacugcaauuacuuuu..............   1       0    ya1

    ................................................caaagacugcaauuacuuuug.............   1       0    s04
    .................................................aaagacugcaauuacuuuug.............   1       0    s04

    ................................................caaagacugcaauuacuuuug.............   2       0    j26

    ................................................caaagacugcaauuacuu................   1       0    va1
    ................................................caaagacugcaauuacuuuug.............   9       0    va1
    ................................................caaagacugcaauuacuuuugc............   10      0    va1
    ................................................caaagacugcaauuacuuuugca...........   2       0    va1

    ................................................caaagacugcaauuacuuuu..............   3       0    ku5
    ................................................caaagacugcaauuacuuuug.............   2       0    ku5

    ................................................caaagacugcaauuacuuuug.............   1       0    k05

    ................................................caaagacugcaauuacuuuug.............   1       0    s06

    ................................................caaagacugcaauuacuuuugc............   1       0    HeL
    .................................................aaagacugcaauuacuuuugc............   1       0    HeL

    ................................................caaagacugcaauuacuu................   1       0    ku4
    ................................................caaagacugcaauuacuuuu..............   1       0    ku4
    ................................................caaagacugcaauuacuuuug.............   8       0    ku4

    ................................................caaagacugcaauuacuuuug.............   3       0    s07
    ................................................caaagacugcaauuacuuuugc............   2       0    s07

    ................................................caaagacugcaauuacuuuug.............   2       0    s10
```

Star Mature

```
uagguucgugcaagaguuauugcagcuuuugccauuaaaaaauaauggcaaagacugcaauuacuuuugcaacaaccuaaua

...............................................caaagacugcaauuacuuu................   1   0   k08
...............................................caaagacugcaauuacuuuug..............   1   0   k08

...............................................caaagacugcaauuacuuu................   2   0   ta3
...............................................caaagacugcaauuacuuuug..............   9   0   ta3
................................................aaagacugcaauuacuuuug..............   1   0   ta3
................................................aaagacugcaauuacuuuugca............   1   0   ta3

...............................................caaagacugcaauuacuuuu...............   1   0   j10
...............................................caaagacugcaauuacuuuug..............   3   0   j10
...............................................caaagacugcaauuacuuuugc.............   1   0   j10

...............................................caaagacugcaauuacuuuug..............   2   0   k14
................................................aaagacugcaauuacuuuu...............   1   0   k14

...............................................caaagacugcaauuacuuuu...............   1   0   j02
...............................................caaagacugcaauuacuuuug..............   3   0   j02
...............................................caaagacugcaauuacuuuugc.............   3   0   j02

...............................................caaagacugcaauuacuuuu...............   1   0   va2
...............................................caaagacugcaauuacuuuug..............   5   0   va2
................................................aaagacugcaauuacuuuug..............   2   0   va2

...............................................caaagacugcaauuacuuuug..............   1   0   j31
...............................................caaagacugcaauuacuuuugc.............   1   0   j31

...............................................caaagacugcaauuacuuuu...............   1   0   j27
...............................................caaagacugcaauuacuuuug..............   3   0   j27

...............................................caaagacugcaauuacuuuugc.............   1   0   H52

...............................................caaagacugcaauuacuuu................   4   0   j22
...............................................caaagacugcaauuacuuuu...............   1   0   j22
...............................................caaagacugcaauuacuuuug..............   11  0   j22
...............................................caaagacugcaauuacuuuugc.............   2   0   j22

...............................................caaagacugcaauuacuuuug..............   1   0   B14
...............................................caaagacugcaauuacuuuugc.............   1   0   B14

...............................................caaagacugcaauuacuuuug..............   4   0   j18

...............................................caaagacugcaauuacuuu................   1   0   ku3
...............................................caaagacugcaauuacuuuu...............   1   0   ku3
...............................................caaagacugcaauuacuuuug..............   1   0   ku3
...............................................caaagacugcaauuacuuuugc.............   1   0   ku3

...............................................caaagacugcaauuacuuuu...............   3   0   j16
...............................................caaagacugcaauuacuuuug..............   10  0   j16
...............................................caaagacugcaauuacuuuugc.............   2   0   j16
...............................................caaagacugcaauuacuuuugcaa...........   2   0   j16

...............................................caaagacugcaauuacuuuu...............   1   0   j09
...............................................caaagacugcaauuacuuuug..............   3   0   j09
...............................................caaagacugcaauuacuuuugc.............   1   0   j09

...............................................caaagacugcaauuacuuuug..............   4   0   ku6

...............................................caaagacugcaauuacuuuugc.............   4   0   s12

...............................................caaagacugcaauuacuuu................   2   0   ya3
...............................................caaagacugcaauuacuuuu...............   8   0   ya3
...............................................caaagacugcaauuacuuuug..............   2   0   ya3
................................................aaagacugcaauuacuuuu...............   2   0   ya3

...............................................caaagacugcaauuacuuuug..............   1   0   la1
...............................................caaagacugcaauuacuuuugcaa...........   1   0   la1

...............................................caaagacugcaauuacuuuu...............   6   0   j17
...............................................caaagacugcaauuacuuuug..............   6   0   j17
```

```
              Star                                 Mature
uagguucgugcaagaguuauugcagcuuuugccauuaaaaaauaauggcaaagacugcaauuacuuuugcaacaaccuaaua
................................................caaagacugcaauuacuuuugc............	4	0	j17

................................................caaagacugcaauuacuu................	1	0	j15
................................................caaagacugcaauuacuuu...............	10	0	j15
................................................caaagacugcaauuacuuuug.............	21	0	j15
................................................caaagacugcaauuacuuuugc............	2	0	j15
.................................................aaagacugcaauuacuu................	1	0	j15
.................................................aaagacugcaauuacuuu...............	3	0	j15
.................................................aaagacugcaauuacuuuug.............	10	0	j15
.................................................aaagacugcaauuacuuuugc............	1	0	j15
.................................................aaagacugcaauuacuuuugca...........	3	0	j15

................................................caaagacugcaauuacuuuug.............	1	0	la2

................................................caaagacugcaauuacuu................	2	0	j12
................................................caaagacugcaauuacuuuug.............	5	0	j12

................................................caaagacugcaauuacuuuug.............	4	0	va4
.................................................aaagacugcaauuacuuuug.............	1	0	va4
.................................................aaagacugcaauuacuuuugc............	2	0	va4
.................................................aaagacugcaauuacuuuugca...........	1	0	va4

................................................caaagacugcaauuacuuuug.............	1	0	va3
................................................caaagacugcaauuacuuuugc............	3	0	va3

................................................caaagacugcaauuacuuuug.............	1	0	SW4
................................................caaagacugcaauuacuuuugc............	1	0	SW4

................................................caaagacugcaauuacuu................	2	0	ya4
................................................caaagacugcaauuacuuu...............	4	0	ya4
................................................caaagacugcaauuacuuuug.............	4	0	ya4
................................................caaagacugcaauuacuuuugc............	1	0	ya4
.................................................aaagacugcaauuacuuu...............	3	0	ya4
.................................................aaagacugcaauuacuuuugca...........	1	0	ya4

................................................caaagacugcaauuacuuu...............	2	0	ku1
................................................caaagacugcaauuacuuuug.............	7	0	ku1
.................................................aaagacugcaauuacuuuugca...........	1	0	ku1

................................................caaagacugcaauuacuuu...............	10	0	j23
................................................caaagacugcaauuacuuuug.............	47	0	j23
................................................caaagacugcaauuacuuuugc............	7	0	j23
................................................caaagacugcaauuacuuuugcaa..........	1	0	j23
.................................................aaagacugcaauuacuuu...............	1	0	j23
.................................................aaagacugcaauuacuuuug.............	3	0	j23

................................................caaagacugcaauuacuuu...............	1	0	s05
................................................caaagacugcaauuacuuuug.............	14	0	s05

................................................caaagacugcaauuacuuuug.............	4	0	ta2
.................................................aaagacugcaauuacuuuugc............	1	0	ta2

................................................caaagacugcaauuacuuuug.............	4	0	j24
................................................caaagacugcaauuacuuuugca...........	6	0	j24

................................................caaagacugcaauuacuuuug.............	3	0	k11

................................................caaagacugcaauuacuuuug.............	2	0	ta1

................................................caaagacugcaauuacuuuug.............	3	0	s09

................................................caaagacugcaauuacuuuug.............	1	0	NL2
.................................................aaagacugcaauuacuuu...............	1	0	NL2

................................................caaagacugcaauuacu.................	1	0	k10
................................................caaagacugcaauuacuuuugc............	1	0	k10

................................................caaagacugcaauuacuuu...............	2	0	j13
................................................caaagacugcaauuacuuuug.............	14	0	j13
.................................................aaagacugcaauuacuuuug.............	3	0	j13
```

```
                Star                      Mature
uagguucgugcaagaguuauugcagcuuuugccauuaaaaaauaauggcaaagacugcaauuacuuuugcaacaaccuaaua

................................................caaagacugcaauuacuuu...............   1     0     j19
................................................caaagacugcaauuacuuuu..............   11    0     j19
................................................caaagacugcaauuacuuuug.............   9     0     j19
................................................caaagacugcaauuacuuuugc............   1     0     j19
.................................................aaagacugcaauuacuuuugcaa..........   2     0     j19

................................................caaagacugcaauuacuuuug.............   2     0     j03
.................................................aaagacugcaauuacuuuug.............   1     0     j03

................................................caaagacugcaauuacuuuug.............   3     0     j25

................................................caaagacugcaauuacuuuu..............   1     0     k01
................................................caaagacugcaauuacuuuug.............   1     0     k01
................................................caaagacugcaauuacuuuugcaa..........   1     0     k01

................................................caaagacugcaauuacuuuugc............   1     0     k04

................................................caaagacugcaauuacuuuu..............   1     0     s01
................................................caaagacugcaauuacuuuugc............   2     0     s01

................................................caaagacugcaauuacuuuu..............   4     0     per
................................................caaagacugcaauuacuuuug.............   6     0     per
................................................caaagacugcaauuacuuuugc............   1     0     per

................................................caaagacugcaauuacuuu...............   1     0     ku2
................................................caaagacugcaauuacuuuu..............   1     0     ku2
................................................caaagacugcaauuacuuuug.............   8     0     ku2
.................................................aaagacugcaauuacuuuug.............   1     0     ku2

................................................caaagacugcaauuacuuuug.............   3     0     U20
................................................caaagacugcaauuacuuuugc............   7     0     U20

.............................................uggcaaagacugcaauuacu.................   2     0     j21
................................................caaagacugcaauuacuuuug.............   18    0     j21
................................................caaagacugcaauuacuuuugc............   7     0     j21
................................................caaagacugcaauuacuuuugcaa..........   2     0     j21

................................................caaagacugcaauuacuuuug.............   1     0     s11
................................................caaagacugcaauuacuuuugc............   2     0     s11

................................................caaagacugcaauuacuuuug.............   9     0     j07

................................................caaagacugcaauuacuuuug.............   1     0     k02
................................................caaagacugcaauuacuuuugc............   1     0     k02
.................................................aaagacugcaauuacuuuug.............   1     0     k02

................................................caaagacugcaauuacuuuug.............   2     0     j14

................................................caaagacugcaauuacuuuugc............   2     0     s08

................................................caaagacugcaauuacuuu...............   1     0     NL3
................................................caaagacugcaauuacuuuug.............   4     0     NL3
.................................................aaagacugcaauuacuuuu..............   1     0     NL3

................................................caaagacugcaauuacuuu...............   3     0     ya2
................................................caaagacugcaauuacuuuu..............   2     0     ya2
.................................................aaagacugcaauuacuuu...............   1     0     ya2
.................................................aaagacugcaauuacuuuu..............   1     0     ya2
.................................................aaagacugcaauuacuuuug.............   1     0     ya2
.................................................aaagacugcaauuacuuuugc............   1     0     ya2

................................................caaagacugcaauuacuuuug.............   1     0     s02

................................................caaagacugcaauuacuuuu..............   1     0     k06
................................................caaagacugcaauuacuuuug.............   2     0     k06

................................................caaagacugcaauuacuuuug.............   1     0     j29

................................................caaagacugcaauuacuuuugc............   3     0     MCF
```

```
            Star                                   Mature
uagguucgugcaagaguuauugcagcuuuugccauuaaaaaauaauggcaaagacugcaauuacuuuugcaacaaccuaaua
.................................................aaagacugcaauuacuuuugc............   1   0   MCF

................................................caaagacugcaauuacuuuug.............   5   0   j01

................................................caaagacugcaauuacuuuugc............   2   0   MBM

................................................caaagacugcaauuacuuuug.............   1   0   NL1
................................................caaagacugcaauuacuuuugc............   1   0   NL1
.................................................aaagacugcaauuacuuuug.............   1   0   NL1
.................................................aaagacugcaauuacuuuugca...........   1   0   NL1

................................................caaagacugcaauuacuuuug.............   1   0   k07

................................................caaagacugcaauuacuuuugc............   1   0   li1

................................................caaagacugcaauuacuuuug.............   3   0   k13
................................................caaagacugcaauuacuuuugc............   1   0   k13

................................................caaagacugcaauuacuuuu..............   4   0   j20
................................................caaagacugcaauuacuuuug.............   2   0   j20
................................................caaagacugcaauuacuuuugc............   1   0   j20
.................................................aaagacugcaauuacuuuug.............   4   0   j20

................................................caaagacugcaauuacuuuugc............   4   0   A54

................................................caaagacugcaauuacuuu...............   1   0   k12
................................................caaagacugcaauuacuuuu..............   1   0   k12
................................................caaagacugcaauuacuuuug.............   2   0   k12

................................................caaagacugcaauuacuuuug.............   1   0   j30
................................................caaagacugcaauuacuuuugc............   1   0   j30
```

Provisional ID	:	hsa_can_269
Score total	:	7.5
Score for star read(s)	:	3.9
Score for read counts	:	4.7
Score for mfe	:	-0.4
Score for randfold	:	
Score for cons. seed	:	-0.6
Total read count	:	21
Mature read count	:	20
Loop read count	:	0
Star read count	:	1

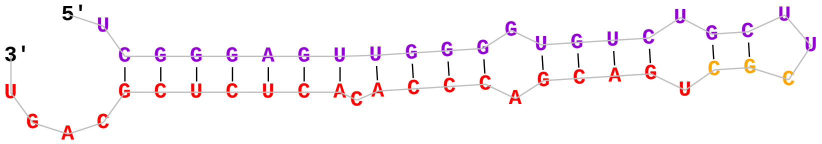

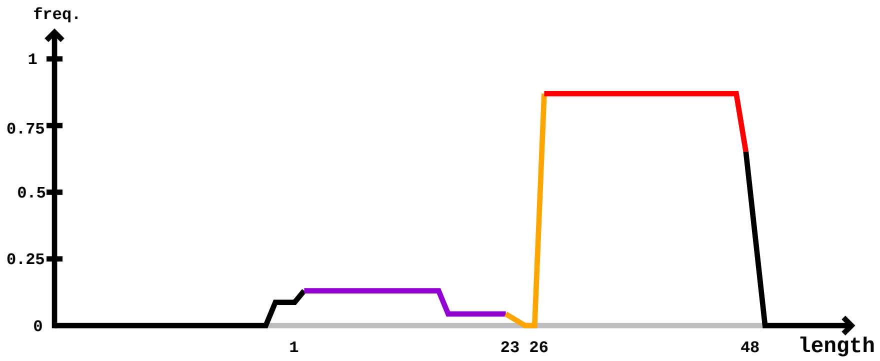

Star Mature

```
5'- augccucuuuuuccacugcaucucucgggaguuggggugucugcuucgcugacgacccacacucucgcaguguucuugcac   -3'    obs
    augccucuuuuuccacugcaucucucgggaguuggggugucugcuucgcugacgacccacacucucgcaguguucuugcac          exp
    ................((((.(.(((((((((((((.((((.((...)).)))).)))).))))))).)).)....)))).    reads      mm        sample
    .................................................ugacgacccacacucucgcag...........    4          0         j23
    .................................................ugacgacccacacucucgcagu..........    6          0         j23

    .....................cucucgggaguuggggug..........................................    1          0         j26

    ........................ucgggaguuggggugucugcuu...................................    1          0         U20

    .................................................ugacgacccacacucucgcagu..........    2          0         j21

    .................................................ugacgacccacacucucgcag...........    1          0         j20

    .................................................ugacgacccacacucucgcagu..........    5          0         HeL

    .....................cucucgggaguuggggug..........................................    1          0         j28

    .................................................ugacgacccacacucucgcagu..........    1          0         HEK

    .................................................ugacgacccacacucucgcagu..........    1          0         ku1
```

Provisional ID	:	hsa_can_275
Score total	:	-2.6
Score for star read(s)	:	-1.3
Score for read counts	:	-1.8
Score for mfe	:	1.1
Score for randfold	:	
Score for cons. seed	:	-0.6
Total read count	:	8
Mature read count	:	8
Loop read count	:	0
Star read count	:	0

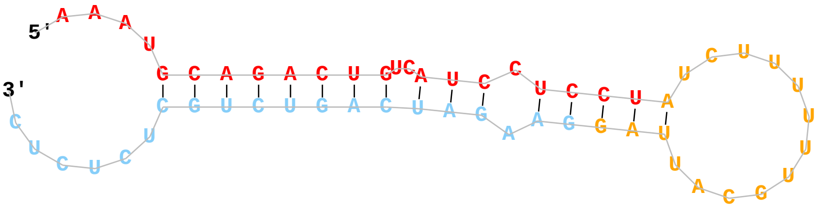

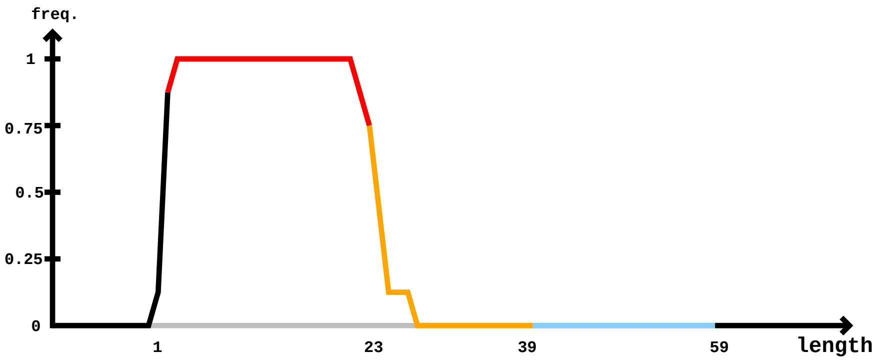

Mature **Star**

```
5'- aacuuggagaaaaugcagacugucauccuccuaucuuuuuugcauuaggaagaucagucugcucucucuccaagagggccc   -3'    exp
    ..((((((((....((((((((..(((.(((((............))))).)))))))))))....))))))))........   reads      mm          sample
    ..........aaaugcagacugucauccuccua................................................   1          0           k14

    ..........aaaugcagacugucauccuc...................................................   1          0           SW4

    .........aaaaugcagacugucauccucc..................................................   1          0           k12

    ..........aaaugcagacugucauccuccu.................................................   3          0           U20
    ..........aaaugcagacugucauccuccua................................................   1          0           U20

    ...........aaugcagacugucauccuccuaucu.............................................   1          0           j11
```

Provisional ID	:	hsa_can_299
Score total	:	0.2
Score for star read(s)	:	-1.3
Score for read counts	:	0
Score for mfe	:	2.1
Score for randfold	:	
Score for cons. seed	:	-0.6
Total read count	:	15
Mature read count	:	15
Loop read count	:	0
Star read count	:	0

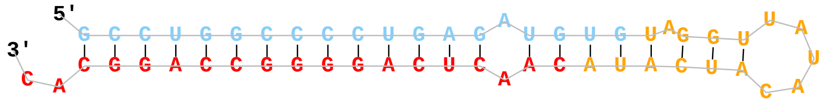

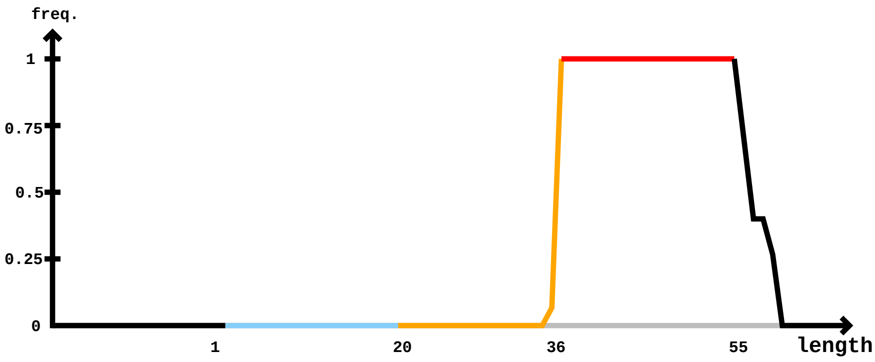

Star Mature

```
5'- auguauugggaaugggGccuggccccugagaugugUagguuauacaucauaCaacucaggggccaggcacCauucacauca   -3'    exp
    ......((.((((((.((((((((((((((.(((((.(((.....)))))))).)))))))))))))).)))))).))...    reads     mm       sample
    ...................................................caacucaggggccaggcaccauu.......    1         0        ya1

    ...................................................caacucaggggccaggcaccau........    1         0        B14

    ...................................................caacucaggggccaggcacc..........    1         0        j09

    ..................................................acaacucaggggccaggcac...........    1         0        li2
    ...................................................caacucaggggccaggcacc..........    1         0        li2

    ...................................................caacucaggggccaggcacc..........    2         0        HEK
    ...................................................caacucaggggccaggcaccau........    1         0        HEK
    ...................................................caacucaggggccaggcaccauu.......    1         0        HEK

    ...................................................caacucaggggccaggcacc..........    1         0        H52

    ...................................................caacucaggggccaggcac...........    3         0        U20
    ...................................................caacucaggggccaggcaccauu.......    2         0        U20
```

Provisional ID	:	hsa_can_302
Score total	:	281.7
Score for star read(s)	:	3.9
Score for read counts	:	276.5
Score for mfe	:	2
Score for randfold	:	
Score for cons. seed	:	-0.6
Total read count	:	554
Mature read count	:	521
Loop read count	:	0
Star read count	:	33

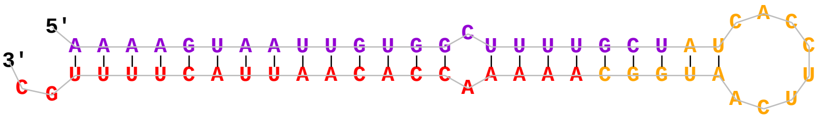

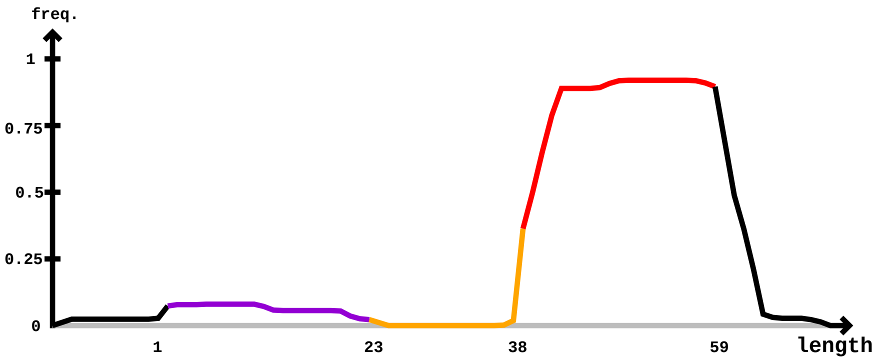

Star **Mature**

```
5'- agguuguugcaaaaguaauuguggcuuuugcuaucaccuucaauggcaaaaaccacaauuacuuuugcaccaaccuaauua   -3'    obs
    agguuguugcaaaaguaauuguggcuuuugcuaucaccuucaauggcaaaaaccacaauuacuuuugcaccaaccuaauua          exp
    agguuguugcaaaaguaauuguggcuuuugcuaucaccuucaauggcaaaaaccacaauuacuuuugcaccaaccuaauua          known
    (((((.((((((((((((((((.(((((((((........))))))))).)))))))))))))))).)))))......... reads  mm  sample
    ...............................................aaaaaccacaauuacuuuug..............  1     0   ya1
    ...............................................aaaaaccacaauuacuuuugca............  4     0   ya1
    ................................................aaaaccacaauuacuuuugca............  3     0   ya1
    ................................................aaaaccacaauuacuuuugcac...........  1     0   ya1
    .................................................aaaccacaauuacuuuugca............  1     0   ya1
    ..................................................aaccacaauuacuuuugcacc..........  1     0   ya1

    ...............................................aaaaaccacaauuacuuuugca............  1     0   s04

    ..........aaaaguaauuguggcuuuugcu.................................................  3     0   j26
    ...............................................aaaaaccacaauuacuuuugca............  3     0   j26
    ...................................................accacaauuacuuuugcacca.........  1     0   j26

    ..........aaaaguaauuguggcuuuug...................................................  1     0   va1
    ..........aaaaguaauuguggcuuuugc..................................................  1     0   va1
    ..........aaaaguaauuguggcuuuugcu.................................................  1     0   va1
    ...............................................aaaaaccacaauuacuuuugc.............  1     0   va1
    ...............................................aaaaaccacaauuacuuuugca............  6     0   va1
    ...............................................aaaaaccacaauuacuuuugcac...........  1     0   va1
    ................................................aaaaccacaauuacuuuugcac...........  5     0   va1
    .................................................aaaccacaauuacuuuugcacc..........  1     0   va1

    ...............................................aaaaaccacaauuacuuuugca............  1     0   ku5
    .................................................aaaccacaauuacuuuugcacc..........  1     0   ku5

    .................................................aaaccacaauuacuuuugcacc..........  1     0   k05

    ...............................................aaaaaccacaauuacuuuugca............  1     0   s06
    ..................................................aaccacaauuacuuuugcacca.........  1     0   s06
    ...................................................accacaauuacuuuugcacca.........  1     0   s06

    ...............................................aaaaaccacaauuacuuuugc.............  1     0   HeL
```

Star **Mature**

```
agguguugcaaaaguaauuguggcuuuugcuaucaccuucaauggcaaaaaccacaauuacuuuugcaccaaccuaauua
..............................................aaaaaccacaauuacuuuugca............   3   0   HeL
...............................................aaaaccacaauuacuuuugcac...........   2   0   HeL
................................................aaaccacaauuacuuuugcac...........   1   0   HeL
................................................aaaccacaauuacuuuugcacc..........   3   0   HeL
.................................................aaccacaauuacuuuugcacca.........   3   0   HeL
.................................................aaccacaauuacuuuugcaccaa........   3   0   HeL
..................................................accacaauuacuuuugcacca.........   5   0   HeL

.........aaaaguaauuguggcuuuu....................................................   1   0   ku4

................................................aaaccacaauuacuuuugcacc..........   1   0   s07

..............................................aaaaaccacaauuacuuuugca............   4   0   s10
................................................aaaccacaauuacuuuugcac...........   1   0   s10

.........aaaaguaauuguggcuuuu....................................................   1   0   ta3
.........aaaaguaauuguggcuuuugcu.................................................   1   0   ta3
..........aaaguaauuguggcuuuug...................................................   1   0   ta3
..............................................aaaaaccacaauuacuuuugca............   4   0   ta3
...............................................aaaaccacaauuacuuuugc.............   1   0   ta3
...............................................aaaaccacaauuacuuuugcac...........   1   0   ta3
................................................aaaccacaauuacuuuugcac...........   1   0   ta3
................................................aaaccacaauuacuuuugcacc..........   1   0   ta3
.................................................aaccacaauuacuuuugcacca.........   2   0   ta3

agguguugcaaaaguaauug............................................................   3   0   j10
..............................................aaaaaccacaauuacuuuugca............   5   0   j10
................................................aaaccacaauuacuuuugcac...........   1   0   j10
.................................................aaccacaauuacuuuugcacca.........   1   0   j10

.................................................aaccacaauuacuuuugcac...........   1   0   k14

..............................................aaaaaccacaauuacuuuugcac...........   1   0   va2
......................................................caauuacuuuugcaccaaccuaa...   1   0   va2

agguguugcaaaaguaauug............................................................   2   0   j02

..............................................aaaaaccacaauuacuuuugca............   1   0   j31

..........aaaguaauuguggcuuuu....................................................   1   0   j27
..........aaaguaauuguggcuuuugc..................................................   1   0   j27
................................................aaaccacaauuacuuuugca............   1   0   j27

..............................................aaaaaccacaauuacuuuugca............   2   0   s03
...............................................aaaaccacaauuacuuuugca............   1   0   s03
...............................................aaaaccacaauuacuuuugcac...........   2   0   s03

..............................................aaaaaccacaauuacuuuug..............   2   0   H52
..............................................aaaaaccacaauuacuuuugca............   1   0   H52
...............................................aaaaccacaauuacuuuugc.............   1   0   H52
...............................................aaaaccacaauuacuuuugca............   2   0   H52
................................................aaaccacaauuacuuuugcacc..........   2   0   H52
.................................................aaccacaauuacuuuugcacc..........   1   0   H52
.................................................aaccacaauuacuuuugcacca.........   1   0   H52
..................................................accacaauuacuuuugcacca.........   1   0   H52

..............................................aaaaaccacaauuacuuuugca............   6   0   j22
...............................................aaaaccacaauuacuuuugca............   1   0   j22
.................................................aaccacaauuacuuuugcacc..........   1   0   j22

...............................................aaaaccacaauuacuuuugcac...........   1   0   k09
................................................aaaccacaauuacuuuugcaccaa........   1   0   k09

agguguugcaaaaguaauu.............................................................   1   0   B14
agguguugcaaaaguaauug............................................................   1   0   B14
..............................................aaaaaccacaauuacuuuugc.............   3   0   B14
..............................................aaaaaccacaauuacuuuugca............   6   0   B14
...............................................aaaaccacaauuacuuuugcac...........   1   0   B14
................................................aaaccacaauuacuuuugca............   2   0   B14
................................................aaaccacaauuacuuuugcacc..........   2   0   B14
```

Star **Mature**

```
agguuguugcaaaaguaauuguggcuuuugcuaucaccuucaauggcaaaaaccacaauuacuuuugcaccaaccuaauua
...................................................accacaauuacuuuugcacca.........   2   0   B14

...............................................aaaaaccacaauuacuuuugca............   4   0   j16

..........aaaaguaauuguggcuuuu....................................................   1   0   j09
...............................................aaaaaccacaauuacuuuugca............   1   0   j09
...................................................accacaauuacuuuugcacca.........   1   0   j09

..........aaaaguaauuguggcuuuugcu.................................................   1   0   ku3
...............................................aaaaaccacaauuacuuuugcac...........   1   0   ku3
.................................................aaaccacaauuacuuuugcacca.........   1   0   ku3

...............................................aaaaaccacaauuacuuuugca............   2   0   ku6

...............................................aaaaaccacaauuacuuuugca............   2   0   j11

..........aaaaguaauuguggcuuu.....................................................   1   0   ya3
..........aaaaguaauuguggcuuuu....................................................   2   0   ya3
...............................................aaaaaccacaauuacuuuugca............   3   0   ya3
................................................aaaaccacaauuacuuuugca............   2   0   ya3
................................................aaaaccacaauuacuuuugcac...........   5   0   ya3
.................................................aaaccacaauuacuuuugca............   1   0   ya3
.................................................aaaccacaauuacuuuugcacc..........   1   0   ya3
..................................................aaccacaauuacuuuugcacc..........   2   0   ya3

...............................................aaaaaccacaauuacuuuugca............   2   0   la1
..................................................aaccacaauuacuuuugcacc..........   1   0   la1

..............................................caaaaaccacaauuacuuuugc.............   3   0   HEK
...............................................aaaaaccacaauuacuuuugc.............   1   0   HEK
...............................................aaaaaccacaauuacuuuugca............   8   0   HEK
................................................aaaaccacaauuacuuuugca............   2   0   HEK
................................................aaaaccacaauuacuuuugcac...........   3   0   HEK
.................................................aaaccacaauuacuuuugca............   1   0   HEK
.................................................aaaccacaauuacuuuugcac...........   3   0   HEK
.................................................aaaccacaauuacuuuugcacc..........   3   0   HEK
..................................................aaccacaauuacuuuugcacc..........   2   0   HEK
..................................................aaccacaauuacuuuugcacca.........   4   0   HEK
...................................................accacaauuacuuuugcacca.........   6   0   HEK

................................................aaaaccacaauuacuuuugca............   1   0   j17
..................................................aaccacaauuacuuuugcacca.........   2   0   j17
...................................................accacaauuacuuuugcacca.........   1   0   j17

agguuguugcaaaaguaauug............................................................   1   0   j15
................................................aaaaccacaauuacuuuugca............   1   0   j15

................................................aaaaccacaauuacuuuugcac...........   1   0   la2
.................................................aaaccacaauuacuuuugca............   1   0   la2
.................................................aaaccacaauuacuuuugcacc..........   1   0   la2
..................................................aaccacaauuacuuuugcacca.........   1   0   la2

...................................................accacaauuacuuuugcacca.........   1   0   j12

...............................................aaaaaccacaauuacuuuugca............   3   0   va4
...............................................aaaaaccacaauuacuuuugcac...........   1   0   va4
................................................aaaaccacaauuacuuuugcac...........   1   0   va4
..................................................aaccacaauuacuuuugcacc..........   2   0   va4
...................................................accacaauuacuuuugcacca.........   4   0   va4

...............................................aaaaaccacaauuacuuuugca............   12  0   va3
................................................aaaaccacaauuacuuuugcac...........   2   0   va3
.................................................aaaccacaauuacuuuugcac...........   1   0   va3
.................................................aaaccacaauuacuuuugcacc..........   4   0   va3
..................................................aaccacaauuacuuuugcacc..........   2   0   va3
..................................................aaccacaauuacuuuugcacca.........   2   0   va3
...................................................accacaauuacuuuugcacca.........   1   0   va3

.........caaaaguaauuguggcuuuu....................................................   1   0   ya4
...............................................aaaaaccacaauuacuuuugca............   4   0   ya4
```

Star **Mature**

agguuguugcaaaaguaauuguggcuuuugcuaucaccuucaauggcaaaaaccacaauuacuuuugcaccaaccuaauua

....aaaaccacaauuacuuuugca............	2	0	ya4
....aaaaccacaauuacuuuugcac...........	4	0	ya4
....aaaccacaauuacuuuugcac...........	1	0	ya4
....aaaccacaauuacuuuugcacc..........	4	0	ya4
....aaccacaauuacuuuugca............	3	0	ya4
....aaccacaauuacuuuugcacc..........	4	0	ya4
....aaccacaauuacuuuugcacca.........	2	0	ya4
..........aaaaguaauuguggcuuuugcu...	1	0	SW4
....aaaaaccacaauuacuuuugca............	1	0	SW4
....aaaaccacaauuacuuuugcac...........	1	0	SW4
....aaaccacaauuacuuuugcac...........	2	0	SW4
....aaaccacaauuacuuuugcacc..........	3	0	SW4
....aaccacaauuacuuuugcacca.........	3	0	SW4
....accacaauuacuuuugcacc..........	1	0	SW4
....accacaauuacuuuugcacca.........	3	0	SW4
....aaaaaccacaauuacuuuugca............	4	0	ku1
....aaccacaauuacuuuugcacc..........	1	0	ku1
..........aaaaguaauuguggcuuuugcu...	1	0	j23
....aaaaaccacaauuacuuuugc.............	1	0	j23
....aaaaaccacaauuacuuuugca............	2	0	j23
....aaaaccacaauuacuuuugca............	1	0	j23
....aaccacaauuacuuuugcacca.........	1	0	j23
....accacaauuacuuuugcacca.........	1	0	j23
....aaaaaccacaauuacuuuugca............	2	0	s05
....aaaaaccacaauuacuuu................	1	0	ta2
....aaaaccacaauuacuuuugcac...........	1	0	ta2
....aaaaaccacaauuacuuuugca............	1	0	k11
....aaaaaccacaauuacuuuugca............	1	0	ta1
....caaaaaccacaauuacuuuugc.............	1	0	s09
....aaaaccacaauuacuuuugca............	1	0	s09
....aaccacaauuacuuuugcacca.........	1	0	s09
....accacaauuacuuuugcacca.........	1	0	s09
....caaaaaccacaauuacuuuu...............	1	0	NL2
....aaaaaccacaauuacuuuugca............	3	0	NL2
....aaccacaauuacuuuugcacc..........	1	0	NL2
....aaccacaauuacuuuugcacca.........	5	0	NL2
....aaaaaccacaauuacuuuugca............	4	0	j13
....aaaaccacaauuacuuuugca............	2	0	j13
....aaccacaauuacuuuugcacca.........	2	0	j13
..........aaaaguaauuguggcuuuug...	1	0	j19
....aaaaaccacaauuacuuuugca............	2	0	j19
....aaaccacaauuacuuuugca............	2	0	j19
....aaccacaauuacuuuugcacca.........	1	0	j19
....accacaauuacuuuugcacca.........	2	0	j19
....aaaaaccacaauuacuuuugca............	1	0	j25
....aauuacuuuugcaccaaccua....	2	0	per
....aaaaaccacaauuacuuuugca............	2	0	s01
....aaaaccacaauuacuuuugcac...........	2	0	s01
....aaaccacaauuacuuuugcacc..........	2	0	s01
....aaaaccacaauuacuuuugca............	1	0	k04
....auuacuuuugcaccaaccuaau..	1	0	k04
.........caaaaguaauuguggcuuuug...	1	0	U20
..............guaauuguggcuuuugcu...	1	0	U20
....caaaaaccacaauuacuuuugc.............	1	0	U20

Star   Mature

agguuguugcaaaaguaauuguggcuuuugcuaucaccuucaauggcaaaaaccacaauuacuuuugcaccaaccuaauua

....aaaaaccacaauuacuuuugc.............	22	0	U20
....aaaaaccacaauuacuuuugca............	17	0	U20
....aaaaccacaauuacuuuugca............	1	0	U20
....aaaaccacaauuacuuuugcac...........	8	0	U20
....aaaccacaauuacuuuugcac...........	6	0	U20
....aaaccacaauuacuuuugcacc..........	14	0	U20
....aaccacaauuacuuuugcacc..........	6	0	U20
....aaccacaauuacuuuugcacca.........	1	0	U20
....accacaauuacuuuugcacca.........	15	0	U20
....accacaauuacuuuugcaccaa........	1	0	U20
..........aaaaguaauuguggcuuuugcu...	2	0	ku2
agguuguugcaaaaguaauug..	1	0	j21
....aaaaaccacaauuacuuuugca............	3	0	j21
....aaaaccacaauuacuuuugca............	1	0	j21
....aaaccacaauuacuuuugcacc..........	1	0	j21
....aaccacaauuacuuuugcacc..........	1	0	s11
....aaccacaauuacuuuugcacca.........	1	0	s11
....aaaaaccacaauuacuuuugca............	1	0	j07
....aaaccacaauuacuuuugcacc..........	1	0	j14
..........aaaaguaauuguggcuuuugcu...	1	0	s08
....aaaaaccacaauuacuuuugca............	1	0	s08
....accacaauuacuuuugcac...........	1	0	s08
agguuguugcaaaaguaauugu...	1	0	DLD
....aaaaaccacaauuacuuuugc.............	1	0	DLD
....aaaaccacaauuacuuuugcac...........	1	0	DLD
....aaaccacaauuacuuuugcacc..........	3	0	DLD
....accacaauuacuuuugcacc..........	1	0	DLD
....accacaauuacuuuugcacca.........	4	0	DLD
....aaaaaccacaauuacuuuugca............	1	0	k03
....caaaaaccacaauuacuuuu...............	1	0	NL3
....caaaaaccacaauuacuuuug..............	2	0	NL3
....aaaaaccacaauuacuuuugca............	7	0	NL3
....aaaaccacaauuacuuuugcac...........	1	0	NL3
....aaccacaauuacuuuugcacca.........	1	0	NL3
....auuacuuuugcaccaaccuaa...	1	0	NL3
agguuguugcaaaaguaauu...	3	0	ya2
..........aaaaguaauuguggcuuuu..	4	0	ya2
....aaaaaccacaauuacuuuu...............	2	0	ya2
....aaaaaccacaauuacuuuugc.............	1	0	ya2
....aaaaaccacaauuacuuuugca............	2	0	ya2
....aaaaccacaauuacuuuugca............	6	0	ya2
....aaaccacaauuacuuuugcac...........	3	0	ya2
....aaaccacaauuacuuuugcacca.........	1	0	ya2
....aaccacaauuacuuuugcacca.........	2	0	ya2
....caauuacuuuugcaccaac.......	1	0	ya2
....aauuacuuuugcaccaac.......	1	0	ya2
....aaaccacaauuacuuuugcacc..........	1	0	k06
....caaaaaccacaauuacuuuug..............	1	0	sh2
....aaccacaauuacuuuugcaccaa........	2	0	sh2
....uuacuuuugcaccaaccua....	1	0	sh2
....aaaaaccacaauuacuuuugca............	1	0	j29
....aaaaccacaauuacuuuugca............	1	0	j29
agguuguugcaaaaguaauu...	1	0	MCF
..........aaaaguaauuguggcuuuug...	1	0	MCF
....aaaaaccacaauuacuuuu...............	1	0	MCF

```
          Star                                Mature
agguuguugcaaaaguaauuguggcuuuugcuaucaccuucaauggcaaaaaccacaauuacuuuugcaccaaccuaauua
...............................................aaaaaccacaauuacuuuugc.............    1    0    MCF
...............................................aaaaaccacaauuacuuuugca............    2    0    MCF
................................................aaaaccacaauuacuuuugcac...........    1    0    MCF
........................................................aauuacuuuugcaccaaccuaa...    2    0    MCF
........................................................aauuacuuuugcaccaaccuaau..    3    0    MCF
.........................................................auuacuuuugcaccaaccuaau..    4    0    MCF

..........aaaaguaauuguggcuuuugcu.................................................    1    0    MBM
...............................................aaaaaccacaauuacuuuug..............    1    0    MBM
...............................................aaaaaccacaauuacuuuugc.............    1    0    MBM
...............................................aaaaaccacaauuacuuuugca............    2    0    MBM
................................................aaaaccacaauuacuuuugca............    1    0    MBM
................................................aaaaccacaauuacuuuugcac...........    3    0    MBM
.................................................aaaccacaauuacuuuugcacc..........    4    0    MBM
..................................................aaccacaauuacuuuugcacc..........    1    0    MBM
...................................................accacaauuacuuuugcacca.........    4    0    MBM

.............................................gcaaaaaccacaauuacuuuug..............    1    0    li1
...............................................aaaaaccacaauuacuuuugc.............    1    0    li1
................................................aaaaccacaauuacuuuugcac...........    1    0    li1
.................................................aaaccacaauuacuuuugcacc..........    2    0    li1
..................................................aaccacaauuacuuuugcacca.........    1    0    li1

........................................................aauuacuuuugcaccaaccuaa...    1    0    k13

..........aaaaguaauuguggcuuuug...................................................    1    0    j20

...............................................aaaaaccacaauuacuuuugc.............    7    0    A54
..................................................aaccacaauuacuuuugcacc..........    4    0    A54
..................................................aaccacaauuacuuuugcacca.........    4    0    A54

...................................................accacaauuacuuuugcacca.........    1    0    k12
```

Provisional ID	:	hsa_can_418
Score total	:	-3
Score for star read(s)	:	-1.3
Score for read counts	:	0
Score for mfe	:	-1.1
Score for randfold	:	
Score for cons. seed	:	-0.6
Total read count	:	33
Mature read count	:	33
Loop read count	:	0
Star read count	:	0

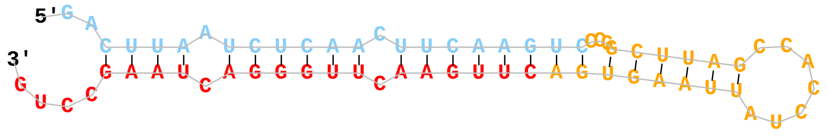

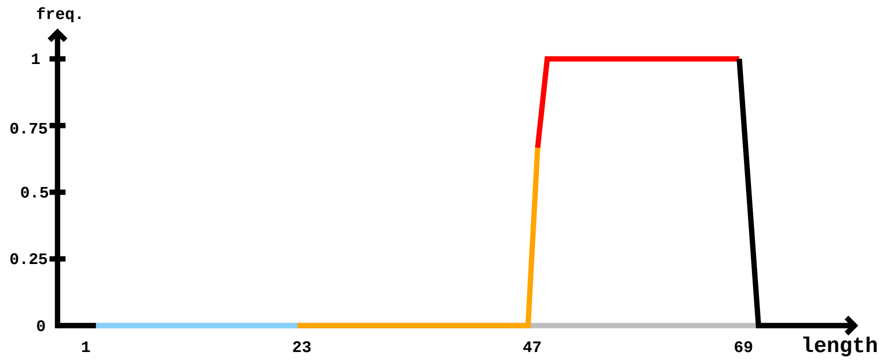

Star Mature

```
5'- cagacuuaaucucaacuucaaguccggcuuagccaccuauuaagugacuugaacuugggacuaagccuguugucgguuca   -3'    exp
    (((.((((.((((((.((((((((...((((((.......))))))))))))))).)))))).)))).)))...........    reads      mm          sample
    ................................................cuugaacuugggacuaagccug...........    1          0           j23
    .................................................uugaacuugggacuaagccug...........    1          0           j23

    ................................................cuugaacuugggacuaagccug...........    1          0           s08
    ................................................cuugaacuugggacuaagccugu..........    1          0           s08

    ................................................cuugaacuugggacuaagccugu..........    1          0           li1
    .................................................uugaacuugggacuaagccugu..........    2          0           li1

    ................................................cuugaacuugggacuaagccug...........    1          0           j16
    ................................................cuugaacuugggacuaagccugu..........    1          0           j16

    ................................................cuugaacuugggacuaagccug...........    1          0           li2
    ................................................cuugaacuugggacuaagccugu..........    1          0           li2
    .................................................uugaacuugggacuaagccug...........    1          0           li2

    ................................................cuugaacuugggacuaagccug...........    3          0           va1
    ................................................cuugaacuugggacuaagccugu..........    1          0           va1

    ................................................cuugaacuugggacuaagccugu..........    1          0           ku2

    ................................................cuugaacuugggacuaagccug...........    1          0           va4

    ................................................cuugaacuugggacuaagccug...........    1          0           j21
    ................................................cuugaacuugggacuaagccugu..........    2          0           j21
    .................................................uugaacuugggacuaagccug...........    1          0           j21
    .................................................uugaacuugggacuaagccugu..........    2          0           j21

    ................................................cuugaacuugggacuaagccugu..........    1          0           j20

    .................................................uugaacuugggacuaagccugu..........    1          0           s06

    ................................................cuugaacuugggacuaagccug...........    1          0           va3

    ................................................cuugaacuugggacuaagccugu..........    2          0           j14
```

Star **Mature**

cagacuuaaucucaacuucaagucccggcuuagccaccuauuaagugacuugaacuugggacuaagccuguugucgguuca

....uugaacuugggacuaagccugu..........	2	0	j28
....uugaacuugggacuaagccugu..........	1	0	MCF
....cuugaacuugggacuaagccugu..........	1	0	s10

Provisional ID	:	hsa_can_437
Score total	:	305.2
Score for star read(s)	:	3.9
Score for read counts	:	299.9
Score for mfe	:	2
Score for randfold	:	
Score for cons. seed	:	-0.6
Total read count	:	600
Mature read count	:	521
Loop read count	:	0
Star read count	:	79

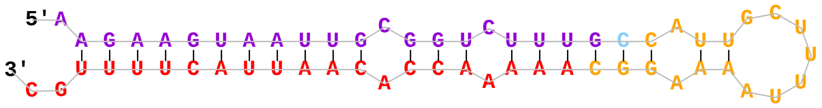

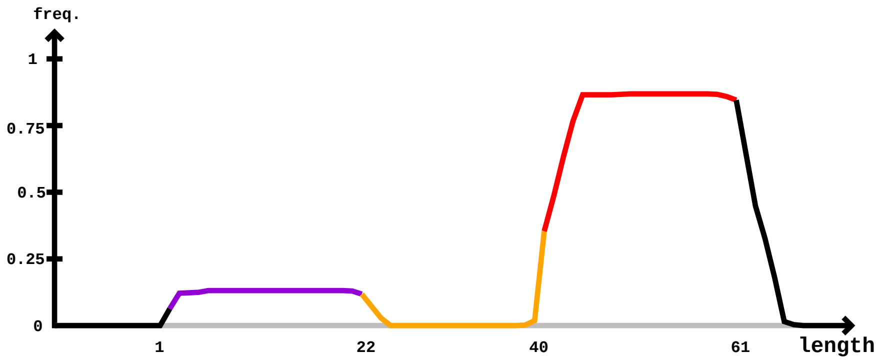

Star **Mature**

```
5'- gagguuggugaagaaguaauugcggucuuugccauugcuuuuaaaaggcaaaaaccacaauuacuuuugcaccaaccucau  -3'   obs
    gagguuggugaagaaguaauugcggucuuugccauugcuuuuaaaaggcaaaaaccacaauuacuuuugcaccaaccucau        exp
    gagguuggugaagaaguaauugcggucuuugccauugcuuuuaaaaggcaaaaaccacaauuacuuuugcaccaaccucau        known
    ((((((((((.(((((((((((.(((.((((((.((.......)).)))))).))).))))))))))).))))))))))..    reads   mm   sample
    .................................................aaaaaccacaauuacuuuug............    1       0    ya1
    .................................................aaaaaccacaauuacuuuugca..........    4       0    ya1
    ..................................................aaaaccacaauuacuuuugca..........    3       0    ya1
    ..................................................aaaaccacaauuacuuuugcac.........    1       0    ya1
    ...................................................aaaccacaauuacuuuugca..........    1       0    ya1
    ....................................................aaccacaauuacuuuugcacc........    1       0    ya1

    .................................................aaaaaccacaauuacuuuugca..........    1       0    s04

    ..........aagaaguaauugcggucuuu...................................................    2       0    j26
    ..........aagaaguaauugcggucuuug..................................................    1       0    j26
    ...........agaaguaauugcggucuuug..................................................    3       0    j26
    .................................................aaaaaccacaauuacuuuugca..........    3       0    j26
    .....................................................accacaauuacuuuugcacca.......    1       0    j26

    .................................................aaaaaccacaauuacuuuugc...........    1       0    va1
    .................................................aaaaaccacaauuacuuuugca..........    6       0    va1
    .................................................aaaaaccacaauuacuuuugcac.........    1       0    va1
    ..................................................aaaaccacaauuacuuuugcac.........    5       0    va1
    ...................................................aaaccacaauuacuuuugcacc........    1       0    va1

    .................................................aaaaaccacaauuacuuuugca..........    1       0    ku5
    ...................................................aaaccacaauuacuuuugcacc........    1       0    ku5

    ...................................................aaaccacaauuacuuuugcacc........    1       0    k05

    .................................................aaaaaccacaauuacuuuugca..........    1       0    s06
    ....................................................aaccacaauuacuuuugcacca.......    1       0    s06
    .....................................................accacaauuacuuuugcacca.......    1       0    s06

    ..........aagaaguaauugcggucuuugc.................................................    4       0    HeL
    ..........aagaaguaauugcggucuuugcc................................................    1       0    HeL
```

Star **Mature**

```
gagguuggugaagaaguaauugcggucuuugccauugcuuuuaaaaggcaaaaaccacaauuacuuuugcaccaaccucau
...........agaaguaauugcggucuuugcc................................................	5	0	HeL
.............aaguaauugcggucuuugc.................................................	1	0	HeL
..............aguaauugcggucuuugc.................................................	1	0	HeL
..............aguaauugcggucuuugcc................................................	3	0	HeL
.................................................aaaaaccacaauuacuuuugc...........	1	0	HeL
.................................................aaaaaccacaauuacuuuugca..........	3	0	HeL
..................................................aaaaccacaauuacuuuugcac.........	2	0	HeL
...................................................aaaccacaauuacuuuugcac.........	1	0	HeL
...................................................aaaccacaauuacuuuugcacc........	3	0	HeL
....................................................aaccacaauuacuuuugcacca.......	3	0	HeL
....................................................aaccacaauuacuuuugcaccaa......	3	0	HeL
.....................................................accacaauuacuuuugcacca.......	5	0	HeL

..........aagaaguaauugcggucuuug..................................................	2	0	ku4

...................................................aaaccacaauuacuuuugcacc........	1	0	s07

.................................................aaaaaccacaauuacuuuugca..........	4	0	s10
...................................................aaaccacaauuacuuuugcac.........	1	0	s10

..........aagaaguaauugcggucuuugc.................................................	1	0	ta3
.................................................aaaaaccacaauuacuuuugca..........	4	0	ta3
..................................................aaaaccacaauuacuuuugc...........	1	0	ta3
..................................................aaaaccacaauuacuuuugcac.........	1	0	ta3
...................................................aaaccacaauuacuuuugcac.........	1	0	ta3
...................................................aaaccacaauuacuuuugcacc........	1	0	ta3
....................................................aaccacaauuacuuuugcacca.......	2	0	ta3

..........aagaaguaauugcggucuuug..................................................	1	0	j10
.................................................aaaaaccacaauuacuuuugca..........	5	0	j10
...................................................aaaccacaauuacuuuugcac.........	1	0	j10
....................................................aaccacaauuacuuuugcacca.......	1	0	j10

..........aagaaguaauugcggucuuug..................................................	4	0	j04

....................................................aaccacaauuacuuuugcac.........	1	0	k14

.................................................aaaaaccacaauuacuuuugcac.........	1	0	va2

...........agaaguaauugcggucuuug..................................................	2	0	j02

.................................................aaaaaccacaauuacuuuugca..........	1	0	j31

..........aagaaguaauugcggucuuug..................................................	4	0	j27
...........agaaguaauugcggucuuug..................................................	7	0	j27
...................................................aaaccacaauuacuuuugca..........	1	0	j27

.................................................aaaaaccacaauuacuuuugca..........	2	0	s03
..................................................aaaaccacaauuacuuuugca..........	1	0	s03
..................................................aaaaccacaauuacuuuugcac.........	2	0	s03

.................................................aaaaaccacaauuacuuuug............	2	0	H52
.................................................aaaaaccacaauuacuuuugca..........	1	0	H52
..................................................aaaaccacaauuacuuuugc...........	1	0	H52
..................................................aaaaccacaauuacuuuugca..........	2	0	H52
...................................................aaaccacaauuacuuuugcacc........	2	0	H52
....................................................aaccacaauuacuuuugcacc........	1	0	H52
....................................................aaccacaauuacuuuugcacca.......	1	0	H52
.....................................................accacaauuacuuuugcacca.......	1	0	H52

.................................................aaaaaccacaauuacuuuugca..........	6	0	j22
..................................................aaaaccacaauuacuuuugca..........	1	0	j22
....................................................aaccacaauuacuuuugcacc........	1	0	j22

..................................................aaaaccacaauuacuuuugcac.........	1	0	k09
...................................................aaaccacaauuacuuuugcaccaa......	1	0	k09

.................................................aaaaaccacaauuacuuuugc...........	3	0	B14
.................................................aaaaaccacaauuacuuuugca..........	6	0	B14
..................................................aaaaccacaauuacuuuugcac.........	1	0	B14
```

Star **Mature**

```
gagguuggugaagaaguaauugcggucuuugccauugcuuuuaaaaggcaaaaaccacaauuacuuuugcaccaaccucau
...................................................aaaccacaauuacuuuugca..........  2   0   B14
...................................................aaaccacaauuacuuuugcacc........  2   0   B14
.....................................................accacaauuacuuuugcacca.......  2   0   B14

.................................................aaaaaccacaauuacuuuugcac.........  1   0   ku3
...................................................aaaccacaauuacuuuugcacca.......  1   0   ku3

.................................................aaaaaccacaauuacuuuugca..........  4   0   j16

..........aagaaguaauugcggucuuug..................................................  1   0   j09
.................................................aaaaaccacaauuacuuuugca..........  1   0   j09
.....................................................accacaauuacuuuugcacca.......  1   0   j09

..........aagaaguaauugcggucuu....................................................  1   0   ku6
...........agaaguaauugcggucuuu...................................................  1   0   ku6
...........agaaguaauugcggucuuug..................................................  1   0   ku6
.................................................aaaaaccacaauuacuuuugca..........  2   0   ku6

..........aagaaguaauugcggucuuug..................................................  1   0   j11
.................................................aaaaaccacaauuacuuuugca..........  2   0   j11

.................................................aaaaaccacaauuacuuuugca..........  3   0   ya3
..................................................aaaaccacaauuacuuuugca..........  2   0   ya3
..................................................aaaaccacaauuacuuuugcac.........  5   0   ya3
...................................................aaaccacaauuacuuuugca..........  1   0   ya3
...................................................aaaccacaauuacuuuugcacc........  1   0   ya3
....................................................aaccacaauuacuuuugcacc........  2   0   ya3

..........aagaaguaauugcggucuuug..................................................  1   0   la1
.................................................aaaaaccacaauuacuuuugca..........  2   0   la1
....................................................aaccacaauuacuuuugcacc........  1   0   la1

................................................caaaaaccacaauuacuuuugc...........  3   0   HEK
.................................................aaaaaccacaauuacuuuugc...........  1   0   HEK
.................................................aaaaaccacaauuacuuuugca..........  8   0   HEK
..................................................aaaaccacaauuacuuuugca..........  2   0   HEK
..................................................aaaaccacaauuacuuuugcac.........  3   0   HEK
...................................................aaaccacaauuacuuuugca..........  1   0   HEK
...................................................aaaccacaauuacuuuugcac.........  3   0   HEK
...................................................aaaccacaauuacuuuugcacc........  3   0   HEK
....................................................aaccacaauuacuuuugcacc........  2   0   HEK
....................................................aaccacaauuacuuuugcacca.......  4   0   HEK
.....................................................accacaauuacuuuugcacca.......  6   0   HEK

..................................................aaaaccacaauuacuuuugca..........  1   0   j15

..........aagaaguaauugcggucuuug..................................................  3   0   j17
..........aagaaguaauugcggucuuugc.................................................  1   0   j17
..................................................aaaaccacaauuacuuuugca..........  1   0   j17
....................................................aaccacaauuacuuuugcacca.......  2   0   j17
.....................................................accacaauuacuuuugcacca.......  1   0   j17

..........aagaaguaauugcggucuuug..................................................  1   0   la2
..................................................aaaaccacaauuacuuuugcac.........  1   0   la2
...................................................aaaccacaauuacuuuugca..........  1   0   la2
...................................................aaaccacaauuacuuuugcacc........  1   0   la2
....................................................aaccacaauuacuuuugcacca.......  1   0   la2

...........agaaguaauugcggucuuu...................................................  1   0   j12
...........agaaguaauugcggucuuug..................................................  1   0   j12
.....................................................accacaauuacuuuugcacca.......  1   0   j12

.................................................aaaaaccacaauuacuuuugca..........  3   0   va4
.................................................aaaaaccacaauuacuuuugcac.........  1   0   va4
..................................................aaaaccacaauuacuuuugcac.........  1   0   va4
....................................................aaccacaauuacuuuugcacc........  2   0   va4
.....................................................accacaauuacuuuugcacca.......  4   0   va4

.................................................aaaaaccacaauuacuuuugca..........  12  0   va3
..................................................aaaaccacaauuacuuuugcac.........  2   0   va3
```

Star   Mature

```
gagguuggugaagaaguaauugcggucuuugccauugcuuuuaaaaggcaaaaaccacaauuacuuuugcaccaaccucau
...................................................aaaccacaauuacuuuugcac.........    1    0    va3
...................................................aaaccacaauuacuuuugcacc........    4    0    va3
....................................................aaccacaauuacuuuugcacc........    2    0    va3
....................................................aaccacaauuacuuuugcacca.......    2    0    va3
.....................................................accacaauuacuuuugcacca.......    1    0    va3

.................................................aaaaaccacaauuacuuuugca..........    4    0    ya4
..................................................aaaaccacaauuacuuuugca..........    2    0    ya4
..................................................aaaaccacaauuacuuuugcac.........    4    0    ya4
...................................................aaaccacaauuacuuuugcac.........    1    0    ya4
...................................................aaaccacaauuacuuuugcacc........    4    0    ya4
....................................................aaccacaauuacuuuugca..........    3    0    ya4
....................................................aaccacaauuacuuuugcacc........    4    0    ya4
....................................................aaccacaauuacuuuugcacca.......    2    0    ya4

...........agaaguaauugcggucuuugcc................................................    1    0    SW4
.................................................aaaaaccacaauuacuuuugca..........    1    0    SW4
..................................................aaaaccacaauuacuuuugcac.........    1    0    SW4
...................................................aaaccacaauuacuuuugcac.........    2    0    SW4
...................................................aaaccacaauuacuuuugcacc........    3    0    SW4
....................................................aaccacaauuacuuuugcacca.......    3    0    SW4
.....................................................accacaauuacuuuugcacc........    1    0    SW4
.....................................................accacaauuacuuuugcacca.......    3    0    SW4

..........aagaaguaauugcggucuuug..................................................    1    0    ku1
...........agaaguaauugcggucuuug..................................................    1    0    ku1
.................................................aaaaaccacaauuacuuuugca..........    4    0    ku1
....................................................aaccacaauuacuuuugcacc........    1    0    ku1

.................................................aaaaaccacaauuacuuuugc...........    1    0    j23
.................................................aaaaaccacaauuacuuuugca..........    2    0    j23
..................................................aaaaccacaauuacuuuugca..........    1    0    j23
....................................................aaccacaauuacuuuugcacca.......    1    0    j23
.....................................................accacaauuacuuuugcacca.......    1    0    j23

.................................................aaaaaccacaauuacuuuugca..........    2    0    s05

.................................................aaaaaccacaauuacuuu..............    1    0    ta2
..................................................aaaaccacaauuacuuuugcac.........    1    0    ta2

.................................................aaaaaccacaauuacuuuugca..........    1    0    k11

..........aagaaguaauugcggucuuugc.................................................    1    0    ta1
.................................................aaaaaccacaauuacuuuugca..........    1    0    ta1

................................................caaaaaccacaauuacuuuugc...........    1    0    s09
..................................................aaaaccacaauuacuuuugca..........    1    0    s09
....................................................aaccacaauuacuuuugcacca.......    1    0    s09
.....................................................accacaauuacuuuugcacca.......    1    0    s09

..........aagaaguaauugcggucuuu...................................................    1    0    NL2
................................................caaaaaccacaauuacuuuu.............    1    0    NL2
.................................................aaaaaccacaauuacuuuugca..........    3    0    NL2
....................................................aaccacaauuacuuuugcacc........    1    0    NL2
....................................................aaccacaauuacuuuugcacca.......    5    0    NL2

.................................................aaaaaccacaauuacuuuugca..........    4    0    j13
..................................................aaaaccacaauuacuuuugca..........    2    0    j13
....................................................aaccacaauuacuuuugcacca.......    2    0    j13

..........aagaaguaauugcggucuuu...................................................    1    0    j19
..........aagaaguaauugcggucuuug..................................................    1    0    j19
...........agaaguaauugcggucuuug..................................................    1    0    j19
.................................................aaaaaccacaauuacuuuugca..........    2    0    j19
...................................................aaaccacaauuacuuuugca..........    2    0    j19
....................................................aaccacaauuacuuuugcacca.......    1    0    j19
.....................................................accacaauuacuuuugcacca.......    2    0    j19

.................................................aaaaaccacaauuacuuuugca..........    1    0    j25

..................................................aaaaccacaauuacuuuugca..........    1    0    k04
```

```
          Star                   Mature
gagguuggugaagaaguaauugcggucuuugccauugcuuuuaaaaggcaaaaaccacaauuacuuuugcaccaaccucau

.................................................aaaaaccacaauuacuuuugca..........   2     0     s01
..................................................aaaaccacaauuacuuuugcac.........   2     0     s01
...................................................aaaccacaauuacuuuugcacc........   2     0     s01

..........aagaaguaauugcggucuuug..................................................   3     0     ku2
...........agaaguaauugcggucuuug..................................................   1     0     ku2

..........aagaaguaauugcggucuuugc.................................................   1     0     U20
...........agaaguaauugcggucuuugcc................................................   3     0     U20
................................................caaaaaccacaauuacuuuugc...........   1     0     U20
.................................................aaaaaccacaauuacuuuugc...........   22    0     U20
.................................................aaaaaccacaauuacuuuugca..........   17    0     U20
..................................................aaaaccacaauuacuuuugca..........   1     0     U20
..................................................aaaaccacaauuacuuuugcac.........   8     0     U20
...................................................aaaccacaauuacuuuugcac.........   6     0     U20
...................................................aaaccacaauuacuuuugcacc........   14    0     U20
....................................................aaccacaauuacuuuugcacc........   6     0     U20
....................................................aaccacaauuacuuuugcacca.......   1     0     U20
.....................................................accacaauuacuuuugcacca.......   15    0     U20
.....................................................accacaauuacuuuugcaccaa......   1     0     U20

.................................................aaaaaccacaauuacuuuugca..........   3     0     j21
..................................................aaaaccacaauuacuuuugca..........   1     0     j21
...................................................aaaccacaauuacuuuugcacc........   1     0     j21


....................................................aaccacaauuacuuuugcacc........   1     0     s11
....................................................aaccacaauuacuuuugcacca.......   1     0     s11

.................................................aaaaaccacaauuacuuuugca..........   1     0     j07

...................................................aaaccacaauuacuuuugcacc........   1     0     j14

...........agaaguaauugcggucuuug..................................................   2     0     j28
............gaaguaauugcggucuuug..................................................   1     0     j28

.................................................aaaaaccacaauuacuuuugca..........   1     0     s08
.....................................................accacaauuacuuuugcac.........   1     0     s08

.................................................aaaaaccacaauuacuuuugc...........   1     0     DLD
..................................................aaaaccacaauuacuuuugcac.........   1     0     DLD
...................................................aaaccacaauuacuuuugcacc........   3     0     DLD
.....................................................accacaauuacuuuugcacc........   1     0     DLD
.....................................................accacaauuacuuuugcacca.......   4     0     DLD

.................................................aaaaaccacaauuacuuuugca..........   1     0     k03

.................................................aaaaaccacaauuacuuuu.............   2     0     ya2
.................................................aaaaaccacaauuacuuuugc...........   1     0     ya2
.................................................aaaaaccacaauuacuuuugca..........   2     0     ya2
..................................................aaaaccacaauuacuuuugca..........   6     0     ya2
...................................................aaaccacaauuacuuuugcac.........   3     0     ya2
...................................................aaaccacaauuacuuuugcacca.......   1     0     ya2
....................................................aaccacaauuacuuuugcacca.......   2     0     ya2
.........................................................caauuacuuuugcaccaac.....   1     0     ya2
..........................................................aauuacuuuugcaccaac.....   1     0     ya2

................................................caaaaaccacaauuacuuuu.............   1     0     NL3
................................................caaaaaccacaauuacuuuug............   2     0     NL3
.................................................aaaaaccacaauuacuuuugca..........   7     0     NL3
..................................................aaaaccacaauuacuuuugcac.........   1     0     NL3
....................................................aaccacaauuacuuuugcacca.......   1     0     NL3

...................................................aaaccacaauuacuuuugcacc........   1     0     k06

................................................caaaaaccacaauuacuuuug............   1     0     sh2
....................................................aaccacaauuacuuuugcaccaa......   2     0     sh2

.................................................aaaaaccacaauuacuuuugca..........   1     0     j29
```

Star **Mature**

```
gagguuggugaagaaguaauugcggucuuugccauugcuuuuaaaaggcaaaaaccacaauuacuuuugcaccaaccucau
..................................................aaaaccacaauuacuuuugca..........    1    0    j29

.................................................aaaaaccacaauuacuuuu.............    1    0    MCF
.................................................aaaaaccacaauuacuuuugc...........    1    0    MCF
.................................................aaaaaccacaauuacuuuugca..........    2    0    MCF
..................................................aaaaccacaauuacuuuugcac.........    1    0    MCF

...........agaaguaauugcggucuuu...................................................    1    0    NL1

.................................................aaaaaccacaauuacuuuug............    1    0    MBM
.................................................aaaaaccacaauuacuuuugc...........    1    0    MBM
.................................................aaaaaccacaauuacuuuugca..........    2    0    MBM
..................................................aaaaccacaauuacuuuugca..........    1    0    MBM
..................................................aaaaccacaauuacuuuugcac.........    3    0    MBM
...................................................aaaccacaauuacuuuugcacc........    4    0    MBM
....................................................aaccacaauuacuuuugcacc........    1    0    MBM
.....................................................accacaauuacuuuugcacca.......    4    0    MBM

...............................................gcaaaaaccacaauuacuuuug............    1    0    li1
.................................................aaaaaccacaauuacuuuugc...........    1    0    li1
..................................................aaaaccacaauuacuuuugcac.........    1    0    li1
...................................................aaaccacaauuacuuuugcacc........    2    0    li1
....................................................aaccacaauuacuuuugcacca.......    1    0    li1

...........agaaguaauugcggucuuugcc................................................    4    0    A54
.................................................aaaaaccacaauuacuuuugc...........    7    0    A54
....................................................aaccacaauuacuuuugcacc........    4    0    A54
....................................................aaccacaauuacuuuugcacca.......    4    0    A54

.....................................................accacaauuacuuuugcacca.......    1    0    k12
```

Provisional ID	:	hsa_can_477
Score total	:	2.9
Score for star read(s)	:	-1.3
Score for read counts	:	0
Score for mfe	:	1.2
Score for randfold	:	
Score for cons. seed	:	3
Total read count	:	1513
Mature read count	:	1513
Loop read count	:	0
Star read count	:	0

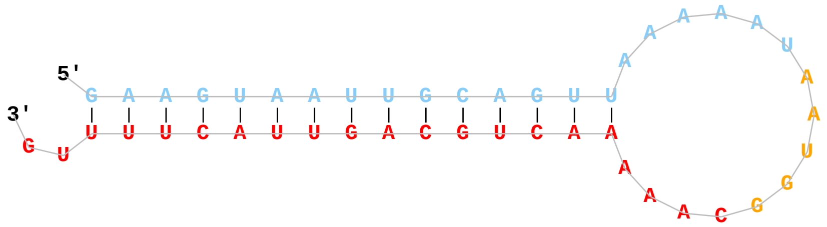

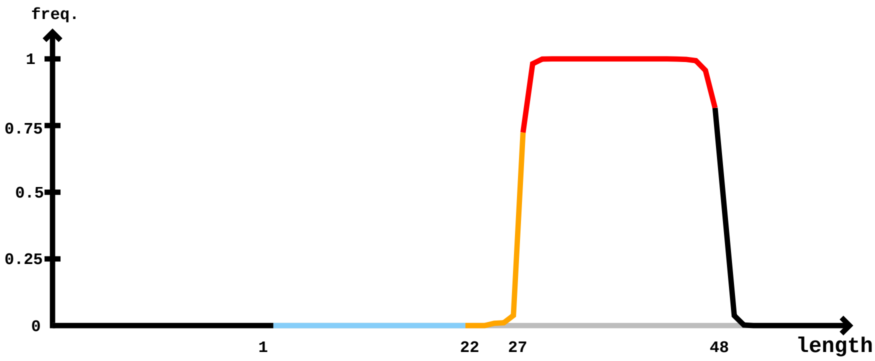

Star Mature

```
5'- uugauguguuagguuggcacagaaguaauugcaguuaaaaauaauggcaaaaacugcaguuacuuuugcaacaaccuaaua  -3'   exp
    uugauguguuagguuggcacagaaguaauugcaguuaaaaauaauggcaaaaacugcaguuacuuuugcaacaaccuaaua        known
    ......((((((((((...((((((((((((((((.................))))))))))))))))...))))))))))   reads   mm   sample
    ...............................................caaaaacugcaguuacuuuu..............   1       0    ya1
    ...............................................caaaaacugcaguuacuuuug.............   2       0    ya1
    ................................................aaaaacugcaguuacuuuu..............   3       0    ya1
    ................................................aaaaacugcaguuacuuuugc............   1       0    ya1

    ............................................uggcaaaaacugcaguuacuu................   1       0    va1
    ..............................................gcaaaaacugcaguuacuuuug.............   5       0    va1
    ...............................................caaaaacugcaguuacuuuug.............   10      0    va1
    ...............................................caaaaacugcaguuacuuuugc............   10      0    va1
    ...............................................caaaaacugcaguuacuuuugcaa..........   1       0    va1
    ................................................aaaaacugcaguuacuuuug.............   2       0    va1
    ................................................aaaaacugcaguuacuuuugca...........   1       0    va1

    ...............................................caaaaacugcaguuacuuuug.............   1       0    k05
    ...............................................caaaaacugcaguuacuuuugc............   4       0    k05
    ................................................aaaaacugcaguuacuuuugc............   1       0    k05

    ................................................aaaaacugcaguuacuuuugc............   1       0    HeL

    ...............................................caaaaacugcaguuacuuuu..............   1       0    k08
    ...............................................caaaaacugcaguuacuuuug.............   4       0    k08

    ...............................................caaaaacugcaguuacuuuu..............   1       0    s10
    ...............................................caaaaacugcaguuacuuuug.............   2       0    s10
    ...............................................caaaaacugcaguuacuuuugc............   1       0    s10
    ................................................aaaaacugcaguuacuuuugc............   1       0    s10
    ................................................aaaaacugcaguuacuuuugca...........   1       0    s10

    ..............................................gcaaaaacugcaguuacuuuug.............   3       0    j10
    ...............................................caaaaacugcaguuacuuuugc............   1       0    j10

    ...............................................caaaaacugcaguuacuuuug.............   2       0    j04

    ...............................................caaaaacugcaguuacuuuug.............   1       0    k14
```

```
                     Star                         Mature
uugauguguuagguuggcacagaaguaauugcaguuaaaaauaauggcaaaaacugcaguuacuuuugcaacaaccuaaua
...............................................caaaaacugcaguuacuuuugc............  3   0   k14
................................................aaaaacugcaguuacuuuugca...........  1   0   k14

..............................................gcaaaaacugcaguuacuuuu..............  1   0   j02
..............................................gcaaaaacugcaguuacuuuug.............  4   0   j02
...............................................caaaaacugcaguuacuuu...............  2   0   j02

..............................................gcaaaaacugcaguuacuuuug.............  1   0   va2
...............................................caaaaacugcaguuacuuuug.............  1   0   va2
...............................................caaaaacugcaguuacuuuugc............  2   0   va2

..............................................gcaaaaacugcaguuacuuuug.............  1   0   j31
...............................................caaaaacugcaguuacuuuug.............  1   0   j31
...............................................caaaaacugcaguuacuuuugc............  1   0   j31
................................................aaaaacugcaguuacuuuugca...........  1   0   j31

...............................................caaaaacugcaguuacuu................  2   0   H52
...............................................caaaaacugcaguuacuuuu..............  7   0   H52
...............................................caaaaacugcaguuacuuuug.............  31  0   H52
...............................................caaaaacugcaguuacuuuugc............  78  0   H52
................................................aaaaacugcaguuacuuuu..............  5   0   H52
................................................aaaaacugcaguuacuuuug.............  6   0   H52
................................................aaaaacugcaguuacuuuugc............  13  0   H52
................................................aaaaacugcaguuacuuuugca...........  12  0   H52
.................................................aaaacugcaguuacuuuugc............  4   0   H52

..............................................gcaaaaacugcaguuacuuuug.............  13  0   j05

..............................................gcaaaaacugcaguuacuuuug.............  2   0   j22
...............................................caaaaacugcaguuacuuuu..............  1   0   j22
...............................................caaaaacugcaguuacuuuug.............  1   0   j22
...............................................caaaaacugcaguuacuuuugc............  2   0   j22

...............................................caaaaacugcaguuacuuuug.............  2   0   k09
...............................................caaaaacugcaguuacuuuugc............  5   0   k09
................................................aaaaacugcaguuacuuuug.............  1   0   k09

...............................................caaaaacugcaguuacuuuugc............  1   0   j09

...............................................caaaaacugcaguuacuuuug.............  3   0   j18

...............................................caaaaacugcaguuacuuuu..............  2   0   j16
...............................................caaaaacugcaguuacuuuug.............  4   0   j16
................................................aaaaacugcaguuacuuuu..............  1   0   j16
................................................aaaaacugcaguuacuuuug.............  6   0   j16
.................................................aaaacugcaguuacuuuug.............  3   0   j16

............................................uggcaaaaacugcaguuacu.................  1   0   ku3
...............................................caaaaacugcaguuacuuuug.............  1   0   ku3

............................................uggcaaaaacugcaguuacuuu...............  4   0   j11

...............................................caaaaacugcaguuacuuuugc............  1   0   s12

...............................................caaaaacugcaguuacuuu...............  2   0   ya3
...............................................caaaaacugcaguuacuuuu..............  3   0   ya3
...............................................caaaaacugcaguuacuuuug.............  1   0   ya3
................................................aaaaacugcaguuacuuuu..............  2   0   ya3
................................................aaaaacugcaguuacuuuug.............  1   0   ya3
.................................................aaaacugcaguuacuuuu..............  1   0   ya3

...............................................caaaaacugcaguuacuuuugc............  1   0   HEK

..............................................gcaaaaacugcaguuacuuuu..............  1   0   la1

............................................uggcaaaaacugcaguuacuuu...............  2   0   j17
...............................................caaaaacugcaguuacuuuu..............  1   0   j17
...............................................caaaaacugcaguuacuuuug.............  4   0   j17
...............................................caaaaacugcaguuacuuuugc............  4   0   j17
................................................aaaaacugcaguuacuuuug.............  4   0   j17
```

Star Mature

uugauguguuagguuggcacagaaguaauugcaguuaaaaauaauggcaaaaacugcaguuacuuuugcaacaaccuaaua			
....aaaaacugcaguuacuuuugc............	1	0	j17
....caaaaacugcaguuacuu................	1	0	j15
....caaaaacugcaguuacuuu...............	28	0	j15
....caaaaacugcaguuacuuuu..............	55	0	j15
....caaaaacugcaguuacuuuug.............	246	0	j15
....caaaaacugcaguuacuuuugc............	20	0	j15
....aaaaacugcaguuacuuu...............	3	0	j15
....aaaaacugcaguuacuuuu..............	24	0	j15
....aaaaacugcaguuacuuuug.............	107	0	j15
....aaaaacugcaguuacuuuugc............	2	0	j15
....aaaaacugcaguuacuuuugca...........	14	0	j15
....aaaacugcaguuacuuuug.............	5	0	j15
....aaaacugcaguuacuuuugca...........	3	0	j15
....gcaaaaacugcaguuacuuuug.............	1	0	la2
....caaaaacugcaguuacuuuug.............	1	0	j12
....aaaaacugcaguuacuuuug.............	1	0	j12
....aaaacugcaguuacuuuug.............	1	0	j12
....aaaacugcaguuacuuuu..............	2	0	SW4
....gcaaaaacugcaguuacuuuug.............	2	0	ya4
....caaaaacugcaguuacuuuu..............	10	0	ya4
....caaaaacugcaguuacuuuug.............	1	0	ya4
....aaaaacugcaguuacuuuug.............	4	0	ya4
....gcaaaaacugcaguuacuu................	1	0	ku1
....caaaaacugcaguuacuuuug.............	2	0	ku1
....aaaaacugcaguuacuuuu..............	1	0	ku1
....uggcaaaaacugcaguuacuu................	1	0	j23
....uggcaaaaacugcaguuacuuu...............	2	0	j23
....gcaaaaacugcaguuacuuuug.............	1	0	j23
....caaaaacugcaguuacuuuu..............	6	0	j23
....caaaaacugcaguuacuuuug.............	34	0	j23
....caaaaacugcaguuacuuuugc............	11	0	j23
....aaaaacugcaguuacuuuu..............	2	0	j23
....aaaaacugcaguuacuuuug.............	17	0	j23
....aaaaacugcaguuacuuuugc............	5	0	j23
....aaaaacugcaguuacuuuugca...........	4	0	j23
....aaaacugcaguuacuuuugc............	1	0	j23
....uggcaaaaacugcaguuacuuuu..............	1	0	j24
....caaaaacugcaguuacuuuug.............	1	0	k11
....caaaaacugcaguuacuuuugc............	2	0	k11
....aaaaacugcaguuacuuuu..............	1	0	k11
....caaaaacugcaguuacuuuu..............	18	0	NL2
....caaaaacugcaguuacuuuug.............	10	0	NL2
....caaaaacugcaguuacuuuugc............	33	0	NL2
....aaaaacugcaguuacuuuu..............	13	0	NL2
....aaaaacugcaguuacuuuug.............	14	0	NL2
....aaaaacugcaguuacuuuugc............	10	0	NL2
....aaaaacugcaguuacuuuugca...........	1	0	NL2
....aaaacugcaguuacuuuugc............	1	0	NL2
....gcaaaaacugcaguuacuuuug.............	1	0	j13
....aaaaacugcaguuacuuuug.............	1	0	j13
....caaaaacugcaguuacuuu...............	1	0	k10
....caaaaacugcaguuacuuuu..............	1	0	k10
....caaaaacugcaguuacuuuug.............	3	0	k10
....caaaaacugcaguuacuuuugc............	5	0	k10
....caaaaacugcaguuacuu................	2	0	j19
....caaaaacugcaguuacuuuu..............	4	0	j19
....caaaaacugcaguuacuuuug.............	42	0	j19

```
                    Star                      Mature
uugauguguuagguuggcacagaaguaauugcaguuaaaaauaauggcaaaaacugcaguuacuuuugcaacaaccuaaua
...............................................caaaaacugcaguuacuuuugc............    2    0    j19
................................................aaaaacugcaguuacuuuu..............    1    0    j19
................................................aaaaacugcaguuacuuuug.............    6    0    j19

...............................................caaaaacugcaguuacuuuug.............    1    0    j25
................................................aaaaacugcaguuacuuuug.............    1    0    j25

...............................................caaaaacugcaguuacuuuug.............    4    0    k01
...............................................caaaaacugcaguuacuuuugc............    4    0    k01
................................................aaaaacugcaguuacuuuug.............    2    0    k01

...............................................caaaaacugcaguuacuuuug.............    1    0    per

...............................................caaaaacugcaguuacuuuugc............    3    0    k04
................................................aaaaacugcaguuacuuuug.............    1    0    k04
................................................aaaaacugcaguuacuuuugca...........    1    0    k04

................................................aaaaacugcaguuacuuu...............    1    0    s01

............................................uggcaaaaacugcaguuacuuu...............    1    0    U20
.............................................ggcaaaaacugcaguuac..................    1    0    U20
..............................................gcaaaaacugcaguuacuuuug.............    2    0    U20
...............................................caaaaacugcaguuacuuuugc............    1    0    U20

.............................................ggcaaaaacugcaguuacu.................    1    0    j21
...............................................caaaaacugcaguuacu.................    1    0    j21
...............................................caaaaacugcaguuacuuuu..............    2    0    j21
...............................................caaaaacugcaguuacuuuug.............    38   0    j21
...............................................caaaaacugcaguuacuuuugc............    37   0    j21
................................................aaaaacugcaguuacuuuu..............    1    0    j21
................................................aaaaacugcaguuacuuuug.............    8    0    j21
................................................aaaaacugcaguuacuuuugc............    11   0    j21
................................................aaaaacugcaguuacuuuugca...........    5    0    j21
................................................aaaaacugcaguuacuuuugcaa..........    1    0    j21
.................................................aaaacugcaguuacuuuugca...........    1    0    j21


...............................................caaaaacugcaguuacuuuugc............    1    0    s11
...............................................caaaaacugcaguuacuuuugcaa..........    1    0    s11

..............................................gcaaaaacugcaguuacuuuug.............    1    0    j14
...............................................caaaaacugcaguuacuuuug.............    2    0    j14
...............................................caaaaacugcaguuacuuuugc............    1    0    j14
................................................aaaaacugcaguuacuuuugc............    1    0    j14

...............................................caaaaacugcaguuacuuu...............    1    0    k02
...............................................caaaaacugcaguuacuuuug.............    3    0    k02
...............................................caaaaacugcaguuacuuuugc............    4    0    k02
................................................aaaaacugcaguuacuuuugc............    1    0    k02

................................................aaaaacugcaguuacuuuu..............    2    0    j28
................................................aaaaacugcaguuacuuuug.............    1    0    j28

...............................................caaaaacugcaguuacuuuug.............    3    0    k03
...............................................caaaaacugcaguuacuuuugc............    1    0    k03
................................................aaaaacugcaguuacuuuugc............    1    0    k03

...............................................caaaaacugcaguuacuuu...............    5    0    NL3
...............................................caaaaacugcaguuacuuuu..............    15   0    NL3
...............................................caaaaacugcaguuacuuuug.............    17   0    NL3
...............................................caaaaacugcaguuacuuuugc............    27   0    NL3
................................................aaaaacugcaguuacuuu...............    2    0    NL3
................................................aaaaacugcaguuacuuuu..............    2    0    NL3
................................................aaaaacugcaguuacuuuug.............    5    0    NL3
................................................aaaaacugcaguuacuuuugc............    9    0    NL3
................................................aaaaacugcaguuacuuuugca...........    1    0    NL3
.................................................aaaacugcaguuacuuuugc............    2    0    NL3

..............................................gcaaaaacugcaguuacuuuu..............    1    0    ya2
...............................................caaaaacugcaguuacuuuu..............    9    0    ya2
```

Star Mature

```
uugauguguuagguuggcacagaaguaauugcaguuaaaaauaauggcaaaaacugcaguuacuuuugcaacaaccuaaua
...............................................caaaaacugcaguuacuuuug.............   3   0   ya2
...............................................caaaaacugcaguuacuuuugc............   1   0   ya2
................................................aaaaacugcaguuacuuuug.............   1   0   ya2

...............................................caaaaacugcaguuacuuuu..............   1   0   k06
...............................................caaaaacugcaguuacuuuug.............   6   0   k06
...............................................caaaaacugcaguuacuuuugc............   3   0   k06
................................................aaaaacugcaguuacuuuug.............   1   0   k06
................................................aaaaacugcaguuacuuuugc............   1   0   k06
..................................................aaacugcaguuacuuuug.............   1   0   k06

..............................................gcaaaaacugcaguuacuuuug.............   1   0   j29

...............................................caaaaacugcaguuacuuuugc............   6   0   sh2
................................................aaaaacugcaguuacuuuugca...........   2   0   sh2

...............................................caaaaacugcaguuacuuuug.............   2   0   j01

...............................................caaaaacugcaguuacuuuug.............   1   0   MCF
...............................................caaaaacugcaguuacuuuugc............   1   0   MCF

...............................................caaaaacugcaguuacuuuu..............   1   0   NL1
...............................................caaaaacugcaguuacuuuug.............   6   0   NL1
...............................................caaaaacugcaguuacuuuugc............   9   0   NL1
................................................aaaaacugcaguuacuuuu..............   4   0   NL1
................................................aaaaacugcaguuacuuuug.............   2   0   NL1
................................................aaaaacugcaguuacuuuugc............   2   0   NL1
................................................aaaaacugcaguuacuuuugca...........   1   0   NL1

...............................................caaaaacugcaguuacuuuug.............   2   0   MBM
...............................................caaaaacugcaguuacuuuugc............   21  0   MBM
................................................aaaaacugcaguuacuuuu..............   1   0   MBM
................................................aaaaacugcaguuacuuuug.............   1   0   MBM
................................................aaaaacugcaguuacuuuugc............   2   0   MBM
................................................aaaaacugcaguuacuuuugca...........   2   0   MBM
.................................................aaaacugcaguuacuuuugc............   3   0   MBM

...............................................caaaaacugcaguuacuuuu..............   1   0   k07
...............................................caaaaacugcaguuacuuuug.............   1   0   k07

...............................................caaaaacugcaguuacuuuugc............   9   0   li1
...............................................caaaaacugcaguuacuuuugca...........   1   0   li1

...............................................caaaaacugcaguuacuuuug.............   3   0   k13
...............................................caaaaacugcaguuacuuuugc............   5   0   k13
................................................aaaaacugcaguuacuuuu..............   1   0   k13
................................................aaaaacugcaguuacuuuug.............   2   0   k13

................................................aaaaacugcaguuacuuuug.............   1   0   j20

.............................................ggcaaaaacugcaguuacuuuu..............   1   0   k12
...............................................caaaaacugcaguuacuuuu..............   2   0   k12
...............................................caaaaacugcaguuacuuuug.............   3   0   k12
...............................................caaaaacugcaguuacuuuugc............   1   0   k12
................................................aaaaacugcaguuacuuuu..............   1   0   k12
................................................aaaaacugcaguuacuuuug.............   1   0   k12
................................................aaaaacugcaguuacuuuugc............   1   0   k12

...............................................caaaaacugcaguuacuuuug.............   18  0   j30
...............................................caaaaacugcaguuacuuuugc............   10  0   j30
................................................aaaaacugcaguuacuuuug.............   8   0   j30
................................................aaaaacugcaguuacuuuugc............   3   0   j30
................................................aaaaacugcaguuacuuuugca...........   2   0   j30
```

Provisional ID	:	hsa_can_516
Score total	:	-3.3
Score for star read(s)	:	-1.3
Score for read counts	:	0
Score for mfe	:	-1.4
Score for randfold	:	
Score for cons. seed	:	-0.6
Total read count	:	59281
Mature read count	:	59240
Loop read count	:	0
Star read count	:	41

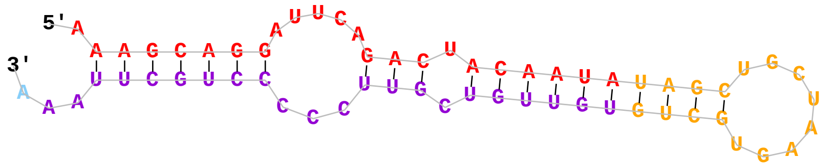

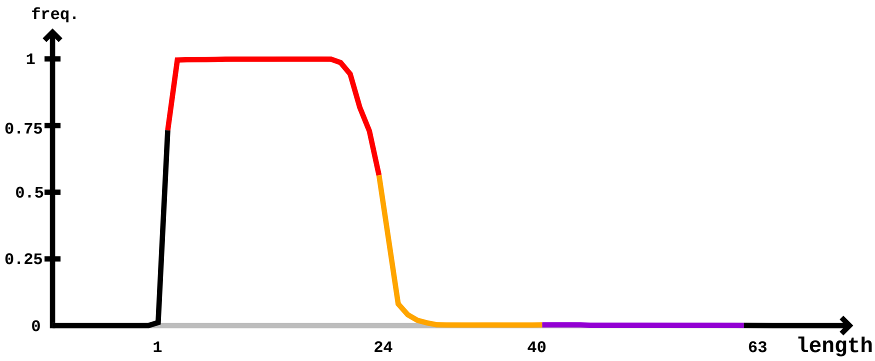

Mature **Star**

```
5'- gauuuccacuaaagcaggauucagacuacaauauagcugcuaagugcuguguugucguucccccugcuuaaaauaaaguug  -3'   obs
    gauuuccacuaaagcaggauucagacuacaauauagcugcuaagugcuguguugucguucccccugcuuaaaauaaaguug         exp
    ...........(((((((.....(((.((((((((((........)))))))))).)))...)))))))............    reads   mm    sample
    .........uaaagcaggauucagacuaca...................................................    1       0     ya1
    .........uaaagcaggauucagacuacaau.................................................    2       0     ya1
    ..........aaagcaggauucagacua.....................................................    1       0     ya1
    ..........aaagcaggauucagacuac....................................................    1       0     ya1
    ..........aaagcaggauucagacuaca...................................................    9       0     ya1
    ..........aaagcaggauucagacuacaa..................................................    17      0     ya1
    ..........aaagcaggauucagacuacaau.................................................    22      0     ya1
    ..........aaagcaggauucagacuacaaua................................................    4       0     ya1
    ..........aaagcaggauucagacuacaauau...............................................    26      0     ya1
    ..........aaagcaggauucagacuacaauauagcu...........................................    2       0     ya1
    ...........aagcaggauucagacuacaau.................................................    5       0     ya1
    ...........aagcaggauucagacuacaaua................................................    1       0     ya1
    ...........aagcaggauucagacuacaauau...............................................    11      0     ya1
    ...........aagcaggauucagacuacaauaua..............................................    2       0     ya1
    ...........aagcaggauucagacuacaauauag.............................................    3       0     ya1
    ...........aagcaggauucagacuacaauauagc............................................    1       0     ya1
    ...........aagcaggauucagacuacaauauagcu...........................................    1       0     ya1

    .........uaaagcaggauucagacuacaau.................................................    1       0     s04
    .........uaaagcaggauucagacuacaaua................................................    1       0     s04
    .........uaaagcaggauucagacuacaauau...............................................    1       0     s04
    ..........aaagcaggauucagacua.....................................................    2       0     s04
    ..........aaagcaggauucagacuac....................................................    11      0     s04
    ..........aaagcaggauucagacuaca...................................................    33      0     s04
    ..........aaagcaggauucagacuacaa..................................................    18      0     s04
    ..........aaagcaggauucagacuacaau.................................................    56      0     s04
    ..........aaagcaggauucagacuacaaua................................................    24      0     s04
    ..........aaagcaggauucagacuacaauau...............................................    164     0     s04
    ..........aaagcaggauucagacuacaauauagc............................................    1       0     s04
    ...........aagcaggauucagacuac....................................................    4       0     s04
    ...........aagcaggauucagacuaca...................................................    4       0     s04
    ...........aagcaggauucagacuacaa..................................................    3       0     s04
    ...........aagcaggauucagacuacaau.................................................    5       0     s04
```

```
          Mature                                   Star
gauuuccacuaaagcaggauucagacuacaauauagcugcuaagugcugugugucguucccccugcuuaaaauaaaguug
...........aagcaggauucagacuacaaua................................................    5     0    s04
...........aagcaggauucagacuacaauau...............................................    24    0    s04
...........aagcaggauucagacuacaauauag.............................................    1     0    s04
...........aagcaggauucagacuacaauauagc............................................    3     0    s04
...........aagcaggauucagacuacaauauagcug..........................................    1     0    s04

.........uaaagcaggauucagacua.....................................................    1     0    j26
.........uaaagcaggauucagacuacaau.................................................    1     0    j26
..........aaagcaggauucagacua.....................................................    29    0    j26
..........aaagcaggauucagacuac....................................................    37    0    j26
..........aaagcaggauucagacuaca...................................................    92    0    j26
..........aaagcaggauucagacuacaa..................................................    89    0    j26
..........aaagcaggauucagacuacaau.................................................    179   0    j26
..........aaagcaggauucagacuacaaua................................................    50    0    j26
..........aaagcaggauucagacuacaauau...............................................    174   0    j26
..........aaagcaggauucagacuacaauaua..............................................    6     0    j26
..........aaagcaggauucagacuacaauauag.............................................    3     0    j26
...........aagcaggauucagacuac....................................................    46    0    j26
...........aagcaggauucagacuaca...................................................    27    0    j26
...........aagcaggauucagacuacaa..................................................    32    0    j26
...........aagcaggauucagacuacaau.................................................    117   0    j26
...........aagcaggauucagacuacaaua................................................    23    0    j26
...........aagcaggauucagacuacaauau...............................................    69    0    j26
...........aagcaggauucagacuacaauaua..............................................    9     0    j26
...........aagcaggauucagacuacaauauag.............................................    3     0    j26
...........aagcaggauucagacuacaauauagc............................................    2     0    j26
......................agacuacaauauagcugcu........................................    1     0    j26
...........................................gugcuguguugucguucc....................    1     0    j26
...........................................gugcuguguugucguucccccugc..............    1     0    j26

.........uaaagcaggauucagacuacaau.................................................    1     0    va1
.........uaaagcaggauucagacuacaauau...............................................    1     0    va1
..........aaagcaggauucagacua.....................................................    3     0    va1
..........aaagcaggauucagacuac....................................................    4     0    va1
..........aaagcaggauucagacuaca...................................................    1     0    va1
..........aaagcaggauucagacuacaa..................................................    8     0    va1
..........aaagcaggauucagacuacaau.................................................    36    0    va1
..........aaagcaggauucagacuacaaua................................................    7     0    va1
..........aaagcaggauucagacuacaauau...............................................    77    0    va1
..........aaagcaggauucagacuacaauaua..............................................    7     0    va1
..........aaagcaggauucagacuacaauauag.............................................    1     0    va1
..........aaagcaggauucagacuacaauauagc............................................    10    0    va1
...........aagcaggauucagacuacaa..................................................    3     0    va1
...........aagcaggauucagacuacaau.................................................    14    0    va1
...........aagcaggauucagacuacaaua................................................    4     0    va1
...........aagcaggauucagacuacaauau...............................................    5     0    va1
...........aagcaggauucagacuacaauauag.............................................    9     0    va1
...........aagcaggauucagacuacaauauagc............................................    10    0    va1
...........aagcaggauucagacuacaauauagcu...........................................    3     0    va1
............agcaggauucagacuacaauauagc............................................    1     0    va1

.........uaaagcaggauucagacuaca...................................................    5     0    ku5
.........uaaagcaggauucagacuacaa..................................................    2     0    ku5
.........uaaagcaggauucagacuacaau.................................................    1     0    ku5
.........uaaagcaggauucagacuacaaua................................................    1     0    ku5
.........uaaagcaggauucagacuacaauau...............................................    1     0    ku5
..........aaagcaggauucagacuac....................................................    4     0    ku5
..........aaagcaggauucagacuaca...................................................    148   0    ku5
..........aaagcaggauucagacuacaa..................................................    68    0    ku5
..........aaagcaggauucagacuacaau.................................................    59    0    ku5
..........aaagcaggauucagacuacaaua................................................    35    0    ku5
..........aaagcaggauucagacuacaauau...............................................    72    0    ku5
..........aaagcaggauucagacuacaauaua..............................................    1     0    ku5
..........aaagcaggauucagacuacaauauag.............................................    2     0    ku5
..........aaagcaggauucagacuacaauauagc............................................    1     0    ku5
...........aagcaggauucagacuac....................................................    2     0    ku5
...........aagcaggauucagacuaca...................................................    1     0    ku5
...........aagcaggauucagacuacaau.................................................    1     0    ku5
...........aagcaggauucagacuacaaua................................................    9     0    ku5
...........aagcaggauucagacuacaauau...............................................    12    0    ku5
```

Mature **Star**

```
gauuuccacuaaagcaggauucagacuacaauauagcugcuaagugcuguguugucguucccccugcuuaaaauaaaguug
...........aagcaggauucagacuacaauauag.............................................  1    0  ku5
...........aagcaggauucagacuacaauauagc............................................  1    0  ku5
.................................................uguugucguucccccugcuuaaa.........  1    0  ku5

.........uaaagcaggauucagacuac....................................................  1    0  k05
.........uaaagcaggauucagacuaca...................................................  1    0  k05
.........uaaagcaggauucagacuacaau.................................................  3    0  k05
.........uaaagcaggauucagacuacaauau...............................................  1    0  k05
..........aaagcaggauucagacua.....................................................  6    0  k05
..........aaagcaggauucagacuac....................................................  31   0  k05
..........aaagcaggauucagacuaca...................................................  133  0  k05
..........aaagcaggauucagacuacaa..................................................  77   0  k05
..........aaagcaggauucagacuacaau.................................................  162  0  k05
..........aaagcaggauucagacuacaaua................................................  56   0  k05
..........aaagcaggauucagacuacaauau...............................................  308  0  k05
..........aaagcaggauucagacuacaauaua..............................................  1    0  k05
...........aagcaggauucagacuac....................................................  9    0  k05
...........aagcaggauucagacuaca...................................................  6    0  k05
...........aagcaggauucagacuacaa..................................................  5    0  k05
...........aagcaggauucagacuacaau.................................................  16   0  k05
...........aagcaggauucagacuacaaua................................................  14   0  k05
...........aagcaggauucagacuacaauau...............................................  118  0  k05
............agcaggauucagacuacaauau...............................................  1    0  k05

.........uaaagcaggauucagacu......................................................  2    0  s06
.........uaaagcaggauucagacua.....................................................  1    0  s06
.........uaaagcaggauucagacuac....................................................  2    0  s06
.........uaaagcaggauucagacuaca...................................................  1    0  s06
.........uaaagcaggauucagacuacaa..................................................  1    0  s06
.........uaaagcaggauucagacuacaau.................................................  2    0  s06
..........aaagcaggauucagacua.....................................................  19   0  s06
..........aaagcaggauucagacuac....................................................  50   0  s06
..........aaagcaggauucagacuaca...................................................  24   0  s06
..........aaagcaggauucagacuacaa..................................................  36   0  s06
..........aaagcaggauucagacuacaau.................................................  171  0  s06
..........aaagcaggauucagacuacaaua................................................  31   0  s06
..........aaagcaggauucagacuacaauau...............................................  219  0  s06
..........aaagcaggauucagacuacaauaua..............................................  2    0  s06
..........aaagcaggauucagacuacaauauag.............................................  2    0  s06
..........aaagcaggauucagacuacaauauagc............................................  1    0  s06
...........aagcaggauucagacuac....................................................  52   0  s06
...........aagcaggauucagacuaca...................................................  35   0  s06
...........aagcaggauucagacuacaa..................................................  25   0  s06
...........aagcaggauucagacuacaau.................................................  170  0  s06
...........aagcaggauucagacuacaaua................................................  7    0  s06
...........aagcaggauucagacuacaauau...............................................  31   0  s06
...........aagcaggauucagacuacaauaua..............................................  1    0  s06
...........aagcaggauucagacuacaauauag.............................................  2    0  s06
...........aagcaggauucagacuacaauauagc............................................  5    0  s06

.........uaaagcaggauucagacua.....................................................  1    0  HeL
..........aaagcaggauucagacua.....................................................  2    0  HeL
...............aggauucagacuacaauauagcugc.........................................  1    0  HeL
................ggauucagacuacaauauagcu...........................................  3    0  HeL
.................gauucagacuacaauauagc............................................  1    0  HeL
....................ucagacuacaauauagcu...........................................  1    0  HeL
.......................gacuacaauauagcugcu........................................  1    0  HeL

.........uaaagcaggauucagacuac....................................................  1    0  ku4
.........uaaagcaggauucagacuaca...................................................  4    0  ku4
.........uaaagcaggauucagacuacaa..................................................  1    0  ku4
..........aaagcaggauucagacua.....................................................  5    0  ku4
..........aaagcaggauucagacuac....................................................  11   0  ku4
..........aaagcaggauucagacuaca...................................................  244  0  ku4
..........aaagcaggauucagacuacaa..................................................  65   0  ku4
..........aaagcaggauucagacuacaau.................................................  58   0  ku4
..........aaagcaggauucagacuacaaua................................................  69   0  ku4
..........aaagcaggauucagacuacaauau...............................................  82   0  ku4
..........aaagcaggauucagacuacaauaua..............................................  4    0  ku4
..........aaagcaggauucagacuacaauauag.............................................  2    0  ku4
```

Mature Star

```
gauuuccacuaaagcaggauucagacuacaauauagcugcuaagugcuguguugucguucccccugcuuaaaauaaaguug
..........aaagcaggauucagacuacaauauagc............................................    1     0    ku4
..........aaagcaggauucagacuacaauauagcug..........................................    2     0    ku4
...........aagcaggauucagacuac....................................................    2     0    ku4
...........aagcaggauucagacuaca...................................................    7     0    ku4
...........aagcaggauucagacuacaa..................................................    1     0    ku4
...........aagcaggauucagacuacaau.................................................    3     0    ku4
...........aagcaggauucagacuacaaua................................................    10    0    ku4
...........aagcaggauucagacuacaauau...............................................    9     0    ku4
...........aagcaggauucagacuacaauaua..............................................    4     0    ku4
...........aagcaggauucagacuacaauauag.............................................    2     0    ku4
...........aagcaggauucagacuacaauauagcug..........................................    1     0    ku4

.........uaaagcaggauucagacuacaa..................................................    1     0    s07
.........uaaagcaggauucagacuacaau.................................................    4     0    s07
.........uaaagcaggauucagacuacaauau...............................................    4     0    s07
..........aaagcaggauucagacua.....................................................    3     0    s07
..........aaagcaggauucagacuac....................................................    9     0    s07
..........aaagcaggauucagacuaca...................................................    14    0    s07
..........aaagcaggauucagacuacaa..................................................    29    0    s07
..........aaagcaggauucagacuacaau.................................................    80    0    s07
..........aaagcaggauucagacuacaaua................................................    26    0    s07
..........aaagcaggauucagacuacaauau...............................................    492   0    s07
..........aaagcaggauucagacuacaauaua..............................................    3     0    s07
..........aaagcaggauucagacuacaauauag.............................................    1     0    s07
...........aagcaggauucagacuac....................................................    7     0    s07
...........aagcaggauucagacuaca...................................................    4     0    s07
...........aagcaggauucagacuacaa..................................................    5     0    s07
...........aagcaggauucagacuacaau.................................................    25    0    s07
...........aagcaggauucagacuacaaua................................................    6     0    s07
...........aagcaggauucagacuacaauau...............................................    134   0    s07
...........aagcaggauucagacuacaauauagc............................................    3     0    s07

.........uaaagcaggauucagacuaca...................................................    2     0    k08
.........uaaagcaggauucagacuacaa..................................................    5     0    k08
.........uaaagcaggauucagacuacaau.................................................    4     0    k08
.........uaaagcaggauucagacuacaauau...............................................    1     0    k08
..........aaagcaggauucagacua.....................................................    5     0    k08
..........aaagcaggauucagacuac....................................................    24    0    k08
..........aaagcaggauucagacuaca...................................................    93    0    k08
..........aaagcaggauucagacuacaa..................................................    67    0    k08
..........aaagcaggauucagacuacaau.................................................    82    0    k08
..........aaagcaggauucagacuacaaua................................................    47    0    k08
..........aaagcaggauucagacuacaauau...............................................    204   0    k08
...........aagcaggauucagacuac....................................................    3     0    k08
...........aagcaggauucagacuaca...................................................    4     0    k08
...........aagcaggauucagacuacaa..................................................    4     0    k08
...........aagcaggauucagacuacaau.................................................    6     0    k08
...........aagcaggauucagacuacaaua................................................    5     0    k08
...........aagcaggauucagacuacaauau...............................................    80    0    k08
............agcaggauucagacuacaauau...............................................    1     0    k08
..................................................guugucguucccccugcuuaaa.........    1     0    k08

.........uaaagcaggauucagacuac....................................................    1     0    s10
.........uaaagcaggauucagacuacaau.................................................    3     0    s10
.........uaaagcaggauucagacuacaaua................................................    1     0    s10
..........aaagcaggauucagacua.....................................................    4     0    s10
..........aaagcaggauucagacuac....................................................    19    0    s10
..........aaagcaggauucagacuaca...................................................    5     0    s10
..........aaagcaggauucagacuacaa..................................................    8     0    s10
..........aaagcaggauucagacuacaau.................................................    78    0    s10
..........aaagcaggauucagacuacaaua................................................    6     0    s10
..........aaagcaggauucagacuacaauau...............................................    53    0    s10
..........aaagcaggauucagacuacaauaua..............................................    1     0    s10
..........aaagcaggauucagacuacaauauag.............................................    2     0    s10
..........aaagcaggauucagacuacaauauagc............................................    1     0    s10
...........aagcaggauucagacuac....................................................    28    0    s10
...........aagcaggauucagacuaca...................................................    14    0    s10
...........aagcaggauucagacuacaa..................................................    13    0    s10
...........aagcaggauucagacuacaau.................................................    80    0    s10
...........aagcaggauucagacuacaaua................................................    6     0    s10
```

Mature **Star**

```
gauuuccacuaaagcaggauucagacuacaauauagcugcuaagugcuguguugucguucccccugcuuaaaauaaagguug
...........aagcaggauucagacuacaauau................................................ 11   0   s10
...........aagcaggauucagacuacaauauag.............................................. 2    0   s10
...........aagcaggauucagacuacaauauagc............................................. 2    0   s10

.........uaaagcaggauucagacuaca.................................................... 2    0   ta3
.........uaaagcaggauucagacuacaau.................................................. 1    0   ta3
..........aaagcaggauucagacua...................................................... 1    0   ta3
..........aaagcaggauucagacuac..................................................... 4    0   ta3
..........aaagcaggauucagacuaca.................................................... 24   0   ta3
..........aaagcaggauucagacuacaa................................................... 30   0   ta3
..........aaagcaggauucagacuacaau.................................................. 38   0   ta3
..........aaagcaggauucagacuacaaua................................................. 19   0   ta3
..........aaagcaggauucagacuacaauau................................................ 35   0   ta3
...........aagcaggauucagacuac..................................................... 3    0   ta3
...........aagcaggauucagacuaca.................................................... 1    0   ta3
...........aagcaggauucagacuacaa................................................... 1    0   ta3
...........aagcaggauucagacuacaau.................................................. 2    0   ta3
...........aagcaggauucagacuacaaua................................................. 1    0   ta3
...........aagcaggauucagacuacaauau................................................ 16   0   ta3

.........uaaagcaggauucagacua...................................................... 6    0   j06
..........aaagcaggauucagacua...................................................... 4    0   j06
..........aaagcaggauucagacuac..................................................... 9    0   j06
..........aaagcaggauucagacuaca.................................................... 14   0   j06
..........aaagcaggauucagacuacaau.................................................. 48   0   j06
..........aaagcaggauucagacuacaaua................................................. 17   0   j06
..........aaagcaggauucagacuacaauau................................................ 29   0   j06
..........aaagcaggauucagacuacaauaua............................................... 4    0   j06
...........aagcaggauucagacuacaauau................................................ 19   0   j06
...........aagcaggauucagacuacaauaua............................................... 14   0   j06
...........aagcaggauucagacuacaauauagcug........................................... 8    0   j06

.........uaaagcaggauucagacuacaa................................................... 1    0   j10
.........uaaagcaggauucagacuacaau.................................................. 1    0   j10
.........uaaagcaggauucagacuacaaua................................................. 3    0   j10
..........aaagcaggauucagacua...................................................... 1    0   j10
..........aaagcaggauucagacuaca.................................................... 1    0   j10
..........aaagcaggauucagacuacaa................................................... 4    0   j10
..........aaagcaggauucagacuacaau.................................................. 20   0   j10
..........aaagcaggauucagacuacaaua................................................. 14   0   j10
..........aaagcaggauucagacuacaauau................................................ 109  0   j10
..........aaagcaggauucagacuacaauauag.............................................. 2    0   j10
...........aagcaggauucagacuac..................................................... 2    0   j10
...........aagcaggauucagacuaca.................................................... 1    0   j10
...........aagcaggauucagacuacaau.................................................. 4    0   j10
...........aagcaggauucagacuacaauau................................................ 16   0   j10
...........aagcaggauucagacuacaauaua............................................... 2    0   j10
...........aagcaggauucagacuacaauauag.............................................. 3    0   j10
...............aggauucagacuacaauau................................................ 1    0   j10
.................................................uguugucguucccccugcuuaa........... 1    0   j10

..........aaagcaggauucagacua...................................................... 6    0   j04
..........aaagcaggauucagacuac..................................................... 14   0   j04
..........aaagcaggauucagacuaca.................................................... 49   0   j04
..........aaagcaggauucagacuacaa................................................... 33   0   j04
..........aaagcaggauucagacuacaau.................................................. 19   0   j04
..........aaagcaggauucagacuacaaua................................................. 19   0   j04
..........aaagcaggauucagacuacaauau................................................ 18   0   j04
..........aaagcaggauucagacuacaauaua............................................... 2    0   j04
...........aagcaggauucagacuac..................................................... 27   0   j04
...........aagcaggauucagacuaca.................................................... 5    0   j04
...........aagcaggauucagacuacaa................................................... 22   0   j04
...........aagcaggauucagacuacaau.................................................. 13   0   j04
...........aagcaggauucagacuacaaua................................................. 5    0   j04
...........aagcaggauucagacuacaauau................................................ 3    0   j04
...........aagcaggauucagacuacaauauagc............................................. 2    0   j04
............agcaggauucagacuacaaua................................................. 8    0   j04
............agcaggauucagacuacaauau................................................ 2    0   j04
.......................................cuaagugcuguguugucguucc..................... 3    0   j04

.........uaaagcaggauucagacuacaa................................................... 4    0   va2
```

Mature **Star**

gauuuccacuaaagcaggauucagacuacaauauagcugcuaagugcugguguugucguucccccugcuuaaaauaaaguug

.........uaaagcaggauucagacuacaau...	2	0	va2
.........uaaagcaggauucagacuacaauau...	1	0	va2
..........aaagcaggauucagacua...	1	0	va2
..........aaagcaggauucagacuac..	8	0	va2
..........aaagcaggauucagacuaca...	66	0	va2
..........aaagcaggauucagacuacaa..	40	0	va2
..........aaagcaggauucagacuacaau...	47	0	va2
..........aaagcaggauucagacuacaaua..	48	0	va2
..........aaagcaggauucagacuacaauau...	181	0	va2
..........aaagcaggauucagacuacaauaua..	1	0	va2
...........aagcaggauucagacuac..	5	0	va2
...........aagcaggauucagacuacaa..	1	0	va2
...........aagcaggauucagacuacaau...	5	0	va2
...........aagcaggauucagacuacaaua..	4	0	va2
...........aagcaggauucagacuacaauau...	33	0	va2
.........uaaagcaggauucagacuaca...	1	0	j02
..........aaagcaggauucagacua...	1	0	j02
..........aaagcaggauucagacuaca...	17	0	j02
..........aaagcaggauucagacuacaa..	15	0	j02
..........aaagcaggauucagacuacaau...	18	0	j02
..........aaagcaggauucagacuacaaua..	24	0	j02
..........aaagcaggauucagacuacaauau...	47	0	j02
...........aagcaggauucagacuac..	1	0	j02
...........aagcaggauucagacuacaa..	2	0	j02
...........aagcaggauucagacuacaau...	4	0	j02
...........aagcaggauucagacuacaaua..	5	0	j02
...........aagcaggauucagacuacaauau...	11	0	j02
...........aagcaggauucagacuacaauaua..	2	0	j02
.........uaaagcaggauucagacua...	1	0	k14
.........uaaagcaggauucagacuac..	1	0	k14
.........uaaagcaggauucagacuaca...	3	0	k14
.........uaaagcaggauucagacuacaa..	2	0	k14
..........aaagcaggauucagacua...	4	0	k14
..........aaagcaggauucagacuac..	23	0	k14
..........aaagcaggauucagacuaca...	84	0	k14
..........aaagcaggauucagacuacaa..	65	0	k14
..........aaagcaggauucagacuacaau...	107	0	k14
..........aaagcaggauucagacuacaaua..	64	0	k14
..........aaagcaggauucagacuacaauau...	187	0	k14
..........aaagcaggauucagacuacaauaua..	1	0	k14
...........aagcaggauucagacuac..	2	0	k14
...........aagcaggauucagacuaca...	5	0	k14
...........aagcaggauucagacuacaa..	3	0	k14
...........aagcaggauucagacuacaau...	14	0	k14
...........aagcaggauucagacuacaaua..	8	0	k14
...........aagcaggauucagacuacaauau...	93	0	k14
............agcaggauucagacuacaauau...	1	0	k14
....guguugucguucccccugcuua...........	2	0	k14
.........uaaagcaggauucagacuacaauau...	3	0	j31
..........aaagcaggauucagacuacaa..	1	0	j31
..........aaagcaggauucagacuacaau...	3	0	j31
..........aaagcaggauucagacuacaaua..	5	0	j31
..........aaagcaggauucagacuacaauau...	27	0	j31
...........aagcaggauucagacuacaauau...	2	0	j31
.........uaaagcaggauucagacua...	2	0	j27
.........uaaagcaggauucagacuaca...	5	0	j27
.........uaaagcaggauucagacuacaa..	2	0	j27
.........uaaagcaggauucagacuacaau...	1	0	j27
..........aaagcaggauucagacua...	29	0	j27
..........aaagcaggauucagacuac..	57	0	j27
..........aaagcaggauucagacuaca...	709	0	j27
..........aaagcaggauucagacuacaa..	222	0	j27
..........aaagcaggauucagacuacaau...	194	0	j27
..........aaagcaggauucagacuacaaua..	95	0	j27
..........aaagcaggauucagacuacaauau...	156	0	j27
..........aaagcaggauucagacuacaauaua..	1	0	j27

Mature **Star**

gauuuccacuaaagcaggauucagacuacaauauagcugcuaagugcuguguugucguucccccugcuuaaaauaaaguug			
...........aagcaggauucagacuac..	10	0	j27
...........aagcaggauucagacuaca...	30	0	j27
...........aagcaggauucagacuacaa..	15	0	j27
...........aagcaggauucagacuacaau...	20	0	j27
...........aagcaggauucagacuacaaua..	11	0	j27
...........aagcaggauucagacuacaauau...	70	0	j27
...........aagcaggauucagacuacaauaua..	1	0	j27
...........aagcaggauucagacuacaauauag...	5	0	j27
............agcaggauucagacuacaaua..	2	0	j27
..........aaagcaggauucagacuaca...	10	0	j05
..........aaagcaggauucagacuac..	5	0	H52
..........aaagcaggauucagacuacaauau...	3	0	H52
...............aggauucagacuacaauau...	2	0	H52
................ggauucagacuacaauau...	2	0	H52
.........uaaagcaggauucagacuac..	1	0	s03
.........uaaagcaggauucagacuacaau...	6	0	s03
.........uaaagcaggauucagacuacaauau...	3	0	s03
..........aaagcaggauucagacua...	9	0	s03
..........aaagcaggauucagacuac..	11	0	s03
..........aaagcaggauucagacuaca...	9	0	s03
..........aaagcaggauucagacuacaa..	16	0	s03
..........aaagcaggauucagacuacaau...	81	0	s03
..........aaagcaggauucagacuacaaua..	28	0	s03
..........aaagcaggauucagacuacaauau...	291	0	s03
..........aaagcaggauucagacuacaauaua..	2	0	s03
..........aaagcaggauucagacuacaauauag...	1	0	s03
..........aaagcaggauucagacuacaauauagc..	3	0	s03
..........aaagcaggauucagacuacaauauagcu...	1	0	s03
...........aagcaggauucagacuac..	14	0	s03
...........aagcaggauucagacuaca...	9	0	s03
...........aagcaggauucagacuacaa..	6	0	s03
...........aagcaggauucagacuacaau...	42	0	s03
...........aagcaggauucagacuacaaua..	6	0	s03
...........aagcaggauucagacuacaauau...	38	0	s03
...........aagcaggauucagacuacaauaua..	1	0	s03
...........aagcaggauucagacuacaauauag...	1	0	s03
...........aagcaggauucagacuacaauauagc..	4	0	s03
...........aagcaggauucagacuacaauauagcugc...	1	0	s03
....uguugucguucccccugcuuaa..........	1	0	s03
.........uaaagcaggauucagacuaca...	1	0	j22
.........uaaagcaggauucagacuacaaua..	1	0	j22
..........aaagcaggauucagacua...	1	0	j22
..........aaagcaggauucagacuac..	4	0	j22
..........aaagcaggauucagacuaca...	9	0	j22
..........aaagcaggauucagacuacaa..	8	0	j22
..........aaagcaggauucagacuacaau...	15	0	j22
..........aaagcaggauucagacuacaaua..	14	0	j22
..........aaagcaggauucagacuacaauau...	137	0	j22
...........aagcaggauucagacuacaa..	1	0	j22
...........aagcaggauucagacuacaau...	3	0	j22
...........aagcaggauucagacuacaaua..	1	0	j22
...........aagcaggauucagacuacaauau...	5	0	j22
...........aagcaggauucagacuacaauauagc..	2	0	j22
.........uaaagcaggauucagacua...	1	0	k09
.........uaaagcaggauucagacuaca...	3	0	k09
.........uaaagcaggauucagacuacaau...	2	0	k09
.........uaaagcaggauucagacuacaauau...	4	0	k09
..........aaagcaggauucagacua...	3	0	k09
..........aaagcaggauucagacuac..	21	0	k09
..........aaagcaggauucagacuaca...	74	0	k09
..........aaagcaggauucagacuacaa..	53	0	k09
..........aaagcaggauucagacuacaau...	97	0	k09
..........aaagcaggauucagacuacaaua..	69	0	k09
..........aaagcaggauucagacuacaauau...	361	0	k09
..........aaagcaggauucagacuacaauaua..	2	0	k09

```
          Mature                                 Star
gauuuccacuaaagcaggauucagacuacaauauagcugcuaagugcuguguugucguucccccugcuuaaaauaaaguug
...........aagcaggauucagacuac....................................................    8      0      k09
...........aagcaggauucagacuaca...................................................    5      0      k09
...........aagcaggauucagacuacaa..................................................    3      0      k09
...........aagcaggauucagacuacaau.................................................    14     0      k09
...........aagcaggauucagacuacaaua................................................    11     0      k09
...........aagcaggauucagacuacaauau...............................................    92     0      k09
............agcaggauucagacuacaauau...............................................    1      0      k09
.................................................uguugucguucccccugcuuaa..........    1      0      k09

..........aaagcaggauucagacua.....................................................    1      0      B14
..........aaagcaggauucagacuac....................................................    2      0      B14
..........aaagcaggauucagacuacaa..................................................    1      0      B14
..........aaagcaggauucagacuacaau.................................................    2      0      B14
..........aaagcaggauucagacuacaauau...............................................    1      0      B14
..........aaagcaggauucagacuacaauauagcug..........................................    1      0      B14
..........aaagcaggauucagacuacaauauagcugc.........................................    1      0      B14
................ggauucagacuacaauau...............................................    2      0      B14
................ggauucagacuacaauauagc............................................    1      0      B14
................ggauucagacuacaauauagcugc.........................................    2      0      B14
.................gauucagacuacaauauagcugcuaagug...................................    1      0      B14

.........uaaagcaggauucagacuacaau.................................................    1      0      j09
..........aaagcaggauucagacua.....................................................    3      0      j09
..........aaagcaggauucagacuac....................................................    5      0      j09
..........aaagcaggauucagacuaca...................................................    54     0      j09
..........aaagcaggauucagacuacaa..................................................    24     0      j09
..........aaagcaggauucagacuacaau.................................................    38     0      j09
..........aaagcaggauucagacuacaaua................................................    25     0      j09
..........aaagcaggauucagacuacaauau...............................................    39     0      j09
..........aaagcaggauucagacuacaauauagc............................................    1      0      j09
...........aagcaggauucagacuac....................................................    9      0      j09
...........aagcaggauucagacuaca...................................................    7      0      j09
...........aagcaggauucagacuacaa..................................................    1      0      j09
...........aagcaggauucagacuacaau.................................................    26     0      j09
...........aagcaggauucagacuacaaua................................................    8      0      j09
...........aagcaggauucagacuacaauau...............................................    6      0      j09
...........aagcaggauucagacuacaauaua..............................................    2      0      j09

.........uaaagcaggauucagacuaca...................................................    5      0      j16
..........aaagcaggauucagacua.....................................................    4      0      j16
..........aaagcaggauucagacuac....................................................    6      0      j16
..........aaagcaggauucagacuaca...................................................    104    0      j16
..........aaagcaggauucagacuacaa..................................................    55     0      j16
..........aaagcaggauucagacuacaau.................................................    64     0      j16
..........aaagcaggauucagacuacaaua................................................    60     0      j16
..........aaagcaggauucagacuacaauau...............................................    190    0      j16
..........aaagcaggauucagacuacaauaua..............................................    2      0      j16
..........aaagcaggauucagacuacaauauagc............................................    1      0      j16
...........aagcaggauucagacuac....................................................    1      0      j16
...........aagcaggauucagacuaca...................................................    4      0      j16
...........aagcaggauucagacuacaa..................................................    7      0      j16
...........aagcaggauucagacuacaau.................................................    10     0      j16
...........aagcaggauucagacuacaaua................................................    11     0      j16
...........aagcaggauucagacuacaauau...............................................    38     0      j16
...........aagcaggauucagacuacaauaua..............................................    6      0      j16
...........aagcaggauucagacuacaauauagcu...........................................    1      0      j16
...............aggauucagacuacaauauag.............................................    1      0      j16
.................................................uguugucguucccccugcuuaaa.........    2      0      j16

.........uaaagcaggauucagacuac....................................................    3      0      j18
..........aaagcaggauucagacua.....................................................    1      0      j18
..........aaagcaggauucagacuaca...................................................    160    0      j18
..........aaagcaggauucagacuacaa..................................................    51     0      j18
..........aaagcaggauucagacuacaau.................................................    46     0      j18
..........aaagcaggauucagacuacaaua................................................    39     0      j18
..........aaagcaggauucagacuacaauau...............................................    64     0      j18
...........aagcaggauucagacuaca...................................................    3      0      j18
...........aagcaggauucagacuacaa..................................................    3      0      j18
...........aagcaggauucagacuacaaua................................................    6      0      j18
...........aagcaggauucagacuacaauau...............................................    11     0      j18
```

Mature **Star**

gauuuccacuaaagcaggauucagacuacaauauagcugcuaagugcuguguugucguucccccugcuuaaaauaaaguug			
...........aagcaggauucagacuacaauaua..	3	0	j18
...........aagcaggauucagacuacaauauagc..	3	0	j18
...........aagcaggauucagacuacaauauagcu...	2	0	j18
.....................cagacuacaauauagcug..	1	0	j18
.........uaaagcaggauucagacuac..	1	0	ku3
.........uaaagcaggauucagacuaca...	2	0	ku3
.........uaaagcaggauucagacuacaa..	2	0	ku3
.........uaaagcaggauucagacuacaauau...	1	0	ku3
..........aaagcaggauucagacua...	2	0	ku3
..........aaagcaggauucagacuac..	4	0	ku3
..........aaagcaggauucagacuaca...	189	0	ku3
..........aaagcaggauucagacuacaa..	105	0	ku3
..........aaagcaggauucagacuacaau...	132	0	ku3
..........aaagcaggauucagacuacaaua..	78	0	ku3
..........aaagcaggauucagacuacaauau...	205	0	ku3
..........aaagcaggauucagacuacaauaua..	1	0	ku3
..........aaagcaggauucagacuacaauauagcu...	2	0	ku3
...........aagcaggauucagacuaca...	2	0	ku3
...........aagcaggauucagacuacaau...	5	0	ku3
...........aagcaggauucagacuacaaua..	6	0	ku3
...........aagcaggauucagacuacaauau...	33	0	ku3
...........aagcaggauucagacuacaauaua..	4	0	ku3
...........aagcaggauucagacuacaauauag...	1	0	ku3
...........aagcaggauucagacuacaauauagc..	3	0	ku3
...........aagcaggauucagacuacaauauagcu...	1	0	ku3
...............aggauucagacuacaauauagc..	1	0	ku3
..........aaagcaggauucagacua...	2	0	ku6
..........aaagcaggauucagacuac..	4	0	ku6
..........aaagcaggauucagacuaca...	134	0	ku6
..........aaagcaggauucagacuacaa..	60	0	ku6
..........aaagcaggauucagacuacaau...	63	0	ku6
..........aaagcaggauucagacuacaaua..	34	0	ku6
..........aaagcaggauucagacuacaauau...	66	0	ku6
..........aaagcaggauucagacuacaauaua..	6	0	ku6
..........aaagcaggauucagacuacaauauagc..	1	0	ku6
...........aagcaggauucagacuac..	2	0	ku6
...........aagcaggauucagacuaca...	4	0	ku6
...........aagcaggauucagacuacaa..	1	0	ku6
...........aagcaggauucagacuacaau...	5	0	ku6
...........aagcaggauucagacuacaaua..	4	0	ku6
...........aagcaggauucagacuacaauau...	9	0	ku6
...........aagcaggauucagacuacaauaua..	2	0	ku6
...........aagcaggauucagacuacaauauag...	1	0	ku6
...........aagcaggauucagacuacaauauagc..	1	0	ku6
....aagugcuguguugucguucc....................	1	0	ku6
.........uaaagcaggauucagacuaca...	6	0	j11
..........aaagcaggauucagacua...	3	0	j11
..........aaagcaggauucagacuac..	7	0	j11
..........aaagcaggauucagacuaca...	21	0	j11
..........aaagcaggauucagacuacaa..	15	0	j11
..........aaagcaggauucagacuacaau...	39	0	j11
..........aaagcaggauucagacuacaaua..	27	0	j11
..........aaagcaggauucagacuacaauau...	159	0	j11
...........aagcaggauucagacuac..	4	0	j11
...........aagcaggauucagacuaca...	5	0	j11
...........aagcaggauucagacuacaa..	2	0	j11
...........aagcaggauucagacuacaau...	19	0	j11
...........aagcaggauucagacuacaaua..	12	0	j11
...........aagcaggauucagacuacaauau...	16	0	j11
...........aagcaggauucagacuacaauaua..	1	0	j11
.........uaaagcaggauucagacuac..	1	0	s12
.........uaaagcaggauucagacuacaa..	2	0	s12
.........uaaagcaggauucagacuacaau...	1	0	s12
.........uaaagcaggauucagacuacaaua..	1	0	s12
.........uaaagcaggauucagacuacaauau...	3	0	s12
..........aaagcaggauucagacua...	12	0	s12

Mature Star

gauuuccacuaaagcaggauucagacuacaauauagcugcuaagugcuguguugucguucccccugcuuaaaauaaaguug

..........aaagcaggauucagacuac..	16	0	s12
..........aaagcaggauucagacuaca...	14	0	s12
..........aaagcaggauucagacuacaa..	11	0	s12
..........aaagcaggauucagacuacaau...	74	0	s12
..........aaagcaggauucagacuacaaua..	20	0	s12
..........aaagcaggauucagacuacaauau...	147	0	s12
..........aaagcaggauucagacuacaauaua..	3	0	s12
..........aaagcaggauucagacuacaauauag...	2	0	s12
...........aagcaggauucagacuac..	34	0	s12
...........aagcaggauucagacuaca...	17	0	s12
...........aagcaggauucagacuacaa..	23	0	s12
...........aagcaggauucagacuacaau...	105	0	s12
...........aagcaggauucagacuacaaua..	4	0	s12
...........aagcaggauucagacuacaauau...	33	0	s12
...........aagcaggauucagacuacaauauag...	1	0	s12
...........aagcaggauucagacuacaauauagc..	3	0	s12
.........uaaagcaggauucagacuacaau...	1	0	ya3
..........aaagcaggauucagacuaca...	14	0	ya3
..........aaagcaggauucagacuacaa..	7	0	ya3
..........aaagcaggauucagacuacaau...	34	0	ya3
..........aaagcaggauucagacuacaaua..	4	0	ya3
..........aaagcaggauucagacuacaauau...	24	0	ya3
...........aagcaggauucagacuac..	1	0	ya3
...........aagcaggauucagacuaca...	1	0	ya3
...........aagcaggauucagacuacaau...	1	0	ya3
...........aagcaggauucagacuacaaua..	4	0	ya3
...........aagcaggauucagacuacaauau...	8	0	ya3
...........aagcaggauucagacuacaauaua..	1	0	ya3
...........aagcaggauucagacuacaauauag...	1	0	ya3
...........aagcaggauucagacuacaauauagc..	2	0	ya3
...........aagcaggauucagacuacaauauagcu...	2	0	ya3
............agcaggauucagacuacaauau...	1	0	ya3
..........aaagcaggauucagacua...	1	0	HEK
..........aaagcaggauucagacuac..	5	0	HEK
..........aaagcaggauucagacuacaau...	2	0	HEK
...........aagcaggauucagacuac..	2	0	HEK
................ggauucagacuacaauauagcugcua.......................................	1	0	HEK
.........uaaagcaggauucagacu..	1	0	la1
.........uaaagcaggauucagacua...	1	0	la1
.........uaaagcaggauucagacuaca...	1	0	la1
.........uaaagcaggauucagacuacaauauagc..	1	0	la1
..........aaagcaggauucagacua...	11	0	la1
..........aaagcaggauucagacuac..	2	0	la1
..........aaagcaggauucagacuaca...	9	0	la1
..........aaagcaggauucagacuacaa..	6	0	la1
..........aaagcaggauucagacuacaau...	26	0	la1
..........aaagcaggauucagacuacaaua..	3	0	la1
..........aaagcaggauucagacuacaauau...	40	0	la1
..........aaagcaggauucagacuacaauauagc..	4	0	la1
..........aaagcaggauucagacuacaauauagcu...	2	0	la1
..........aaagcaggauucagacuacaauauagcug..	1	0	la1
...........aagcaggauucagacuac..	6	0	la1
...........aagcaggauucagacuaca...	3	0	la1
...........aagcaggauucagacuacaa..	5	0	la1
...........aagcaggauucagacuacaau...	11	0	la1
...........aagcaggauucagacuacaauau...	5	0	la1
...........aagcaggauucagacuacaauaua..	1	0	la1
...........aagcaggauucagacuacaauauag...	1	0	la1
...........aagcaggauucagacuacaauauagc..	4	0	la1
...............aggauucagacuacaauauagc..	1	0	la1
.........uaaagcaggauucagacua...	1	0	j15
.........uaaagcaggauucagacuacaa..	1	0	j15
.........uaaagcaggauucagacuacaau...	4	0	j15
..........aaagcaggauucagacua...	9	0	j15
..........aaagcaggauucagacuac..	9	0	j15
..........aaagcaggauucagacuaca...	116	0	j15

```
          Mature                              Star
gauuuccacuaaagcaggauucagacuacaauauagcugcuaagugcuguguugucguucccccugcuuaaaauaaaguug
..........aaagcaggauucagacuacaa..................................................   76    0    j15
..........aaagcaggauucagacuacaau.................................................   86    0    j15
..........aaagcaggauucagacuacaaua................................................   91    0    j15
..........aaagcaggauucagacuacaauau...............................................   213   0    j15
..........aaagcaggauucagacuacaauaua..............................................   2     0    j15
..........aaagcaggauucagacuacaauauagc............................................   2     0    j15
...........aagcaggauucagacuac....................................................   2     0    j15
...........aagcaggauucagacuaca...................................................   6     0    j15
...........aagcaggauucagacuacaa..................................................   5     0    j15
...........aagcaggauucagacuacaau.................................................   13    0    j15
...........aagcaggauucagacuacaaua................................................   12    0    j15
...........aagcaggauucagacuacaauau...............................................   61    0    j15
...........aagcaggauucagacuacaauaua..............................................   2     0    j15
...........aagcaggauucagacuacaauauag.............................................   3     0    j15
...........aagcaggauucagacuacaauauagc............................................   2     0    j15

.........uaaagcaggauucagacua.....................................................   2     0    j17
.........uaaagcaggauucagacuaca...................................................   11    0    j17
.........uaaagcaggauucagacuacaa..................................................   7     0    j17
.........uaaagcaggauucagacuacaau.................................................   5     0    j17
.........uaaagcaggauucagacuacaaua................................................   1     0    j17
..........aaagcaggauucagacua.....................................................   11    0    j17
..........aaagcaggauucagacuac....................................................   26    0    j17
..........aaagcaggauucagacuaca...................................................   117   0    j17
..........aaagcaggauucagacuacaa..................................................   122   0    j17
..........aaagcaggauucagacuacaau.................................................   155   0    j17
..........aaagcaggauucagacuacaaua................................................   86    0    j17
..........aaagcaggauucagacuacaauau...............................................   393   0    j17
..........aaagcaggauucagacuacaauaua..............................................   2     0    j17
...........aagcaggauucagacuac....................................................   9     0    j17
...........aagcaggauucagacuaca...................................................   7     0    j17
...........aagcaggauucagacuacaa..................................................   7     0    j17
...........aagcaggauucagacuacaau.................................................   11    0    j17
...........aagcaggauucagacuacaaua................................................   16    0    j17
...........aagcaggauucagacuacaauau...............................................   107   0    j17
...........aagcaggauucagacuacaauaua..............................................   2     0    j17
.................................................uguugucguucccccugcuuaa..........   1     0    j17

.........uaaagcaggauucagacuacaa..................................................   1     0    la2
.........uaaagcaggauucagacuacaaua................................................   1     0    la2
..........aaagcaggauucagacuaca...................................................   3     0    la2
..........aaagcaggauucagacuacaa..................................................   6     0    la2
..........aaagcaggauucagacuacaau.................................................   7     0    la2
..........aaagcaggauucagacuacaaua................................................   3     0    la2
..........aaagcaggauucagacuacaauau...............................................   14    0    la2
..........aaagcaggauucagacuacaauauag.............................................   1     0    la2
...........aagcaggauucagacuacaaua................................................   1     0    la2
...........aagcaggauucagacuacaauau...............................................   3     0    la2

..........aaagcaggauucagacuaca...................................................   5     0    j08
..........aaagcaggauucagacuacaa..................................................   2     0    j08
..........aaagcaggauucagacuacaau.................................................   12    0    j08
..........aaagcaggauucagacuacaaua................................................   4     0    j08
..........aaagcaggauucagacuacaauau...............................................   4     0    j08
...........aagcaggauucagacuaca...................................................   2     0    j08
...........aagcaggauucagacuacaaua................................................   4     0    j08
...........aagcaggauucagacuacaauau...............................................   6     0    j08

.........uaaagcaggauucagacua.....................................................   1     0    j12
.........uaaagcaggauucagacuaca...................................................   2     0    j12
.........uaaagcaggauucagacuacaauau...............................................   1     0    j12
..........aaagcaggauucagacua.....................................................   7     0    j12
..........aaagcaggauucagacuac....................................................   4     0    j12
..........aaagcaggauucagacuaca...................................................   31    0    j12
..........aaagcaggauucagacuacaa..................................................   19    0    j12
..........aaagcaggauucagacuacaau.................................................   39    0    j12
..........aaagcaggauucagacuacaaua................................................   33    0    j12
..........aaagcaggauucagacuacaauau...............................................   153   0    j12
..........aaagcaggauucagacuacaauaua..............................................   2     0    j12
..........aaagcaggauucagacuacaauauag.............................................   1     0    j12
```

Mature **Star**

```
gauuuccacuaaagcaggauucagacuacaauauagcugcuaagugcuguguugucguucccccugcuuaaaauaaaguug
..........aaagcaggauucagacuacaauauagc............................................    2      0    j12
...........aagcaggauucagacuac....................................................    11     0    j12
...........aagcaggauucagacuaca...................................................    5      0    j12
...........aagcaggauucagacuacaa..................................................    2      0    j12
...........aagcaggauucagacuacaau.................................................    16     0    j12
...........aagcaggauucagacuacaaua................................................    4      0    j12
...........aagcaggauucagacuacaauau...............................................    7      0    j12
...........aagcaggauucagacuacaauaua..............................................    2      0    j12
...........aagcaggauucagacuacaauauag.............................................    4      0    j12
...........aagcaggauucagacuacaauauagc............................................    6      0    j12
...............aggauucagacuacaauau...............................................    1      0    j12
.................................................uguugucguucccccugcuuaaa.........    1      0    j12

.........uaaagcaggauucagacuacaa..................................................    1      0    va4
.........uaaagcaggauucagacuacaau.................................................    4      0    va4
.........uaaagcaggauucagacuacaaua................................................    1      0    va4
.........uaaagcaggauucagacuacaauau...............................................    6      0    va4
..........aaagcaggauucagacua.....................................................    11     0    va4
..........aaagcaggauucagacuac....................................................    4      0    va4
..........aaagcaggauucagacuaca...................................................    15     0    va4
..........aaagcaggauucagacuacaa..................................................    22     0    va4
..........aaagcaggauucagacuacaau.................................................    117    0    va4
..........aaagcaggauucagacuacaaua................................................    86     0    va4
..........aaagcaggauucagacuacaauau...............................................    432    0    va4
..........aaagcaggauucagacuacaauaua..............................................    29     0    va4
..........aaagcaggauucagacuacaauauag.............................................    41     0    va4
..........aaagcaggauucagacuacaauauagc............................................    23     0    va4
..........aaagcaggauucagacuacaauauagcu...........................................    5      0    va4
...........aagcaggauucagacuac....................................................    7      0    va4
...........aagcaggauucagacuaca...................................................    3      0    va4
...........aagcaggauucagacuacaa..................................................    7      0    va4
...........aagcaggauucagacuacaau.................................................    31     0    va4
...........aagcaggauucagacuacaaua................................................    19     0    va4
...........aagcaggauucagacuacaauau...............................................    33     0    va4
...........aagcaggauucagacuacaauaua..............................................    8      0    va4
...........aagcaggauucagacuacaauauag.............................................    28     0    va4
...........aagcaggauucagacuacaauauagc............................................    38     0    va4
...........aagcaggauucagacuacaauauagcu...........................................    6      0    va4
...........aagcaggauucagacuacaauauagcug..........................................    1      0    va4

........cuaaagcaggauucagacuacaaua................................................    2      0    va3
........cuaaagcaggauucagacuacaauaua..............................................    1      0    va3
........cuaaagcaggauucagacuacaauauag.............................................    1      0    va3
.........uaaagcaggauucagacua.....................................................    4      0    va3
.........uaaagcaggauucagacuac....................................................    1      0    va3
.........uaaagcaggauucagacuaca...................................................    10     0    va3
.........uaaagcaggauucagacuacaa..................................................    18     0    va3
.........uaaagcaggauucagacuacaau.................................................    9      0    va3
.........uaaagcaggauucagacuacaaua................................................    113    0    va3
.........uaaagcaggauucagacuacaauau...............................................    17     0    va3
.........uaaagcaggauucagacuacaauaua..............................................    44     0    va3
.........uaaagcaggauucagacuacaauauag.............................................    7      0    va3
.........uaaagcaggauucagacuacaauauagc............................................    2      0    va3
.........uaaagcaggauucagacuacaauauagcu...........................................    2      0    va3
..........aaagcaggauucagacua.....................................................    99     0    va3
..........aaagcaggauucagacuac....................................................    164    0    va3
..........aaagcaggauucagacuaca...................................................    748    0    va3
..........aaagcaggauucagacuacaa..................................................    1040   0    va3
..........aaagcaggauucagacuacaau.................................................    505    0    va3
..........aaagcaggauucagacuacaaua................................................    6206   0    va3
..........aaagcaggauucagacuacaauau...............................................    1157   0    va3
..........aaagcaggauucagacuacaauaua..............................................    1614   0    va3
..........aaagcaggauucagacuacaauauag.............................................    664    0    va3
..........aaagcaggauucagacuacaauauagc............................................    133    0    va3
..........aaagcaggauucagacuacaauauagcu...........................................    203    0    va3
...........aagcaggauucagacuac....................................................    78     0    va3
...........aagcaggauucagacuaca...................................................    313    0    va3
...........aagcaggauucagacuacaa..................................................    453    0    va3
...........aagcaggauucagacuacaau.................................................    209    0    va3
...........aagcaggauucagacuacaaua................................................    3963   0    va3
```

```
          Mature                            Star
gauuuccacuaaagcaggauucagacuacaauauagcugcuaagugcuguguugucguucccccugcuuaaaauaaaguug
...........aagcaggauucagacuacaauau...............................................    614   0   va3
...........aagcaggauucagacuacaauaua..............................................    477   0   va3
...........aagcaggauucagacuacaauauag.............................................    304   0   va3
...........aagcaggauucagacuacaauauagc............................................    85    0   va3
...........aagcaggauucagacuacaauauagcu...........................................    141   0   va3
...........aagcaggauucagacuacaauauagcug..........................................    1     0   va3
............agcaggauucagacuaca...................................................    1     0   va3
............agcaggauucagacuacaa..................................................    3     0   va3
............agcaggauucagacuacaau.................................................    2     0   va3
............agcaggauucagacuacaaua................................................    9     0   va3
............agcaggauucagacuacaauauag.............................................    2     0   va3
.............gcaggauucagacuacaaua................................................    1     0   va3
.............gcaggauucagacuacaauau...............................................    2     0   va3
.............gcaggauucagacuacaauaua..............................................    2     0   va3
.............gcaggauucagacuacaauauag.............................................    3     0   va3
..............caggauucagacuacaaua................................................    1     0   va3
...............aggauucagacuacaaua................................................    1     0   va3
...............aggauucagacuacaauau...............................................    2     0   va3
...............aggauucagacuacaauaua..............................................    5     0   va3
...............aggauucagacuacaauauag.............................................    5     0   va3
...............aggauucagacuacaauauagc............................................    1     0   va3
..................................agcugcuaagugcuguguugucguu......................    1     0   va3
....................................cugcuaagugcuguguugucguucccccu................    2     0   va3
.....................................ugcuaagugcuguguugucguucccc..................    1     0   va3
.....................................ugcuaagugcuguguugucguucccccu................    1     0   va3
......................................gcuaagugcuguguugucguucccccug...............    1     0   va3

..........aaagcaggauucagacuac....................................................    2     0   SW4
..........aaagcaggauucagacuacaau.................................................    1     0   SW4
................ggauucagacuacaauauagc............................................    2     0   SW4

.........uaaagcaggauucagacuacaa..................................................    4     0   ya4
.........uaaagcaggauucagacuacaau.................................................    1     0   ya4
..........aaagcaggauucagacuaca...................................................    12    0   ya4
..........aaagcaggauucagacuacaa..................................................    7     0   ya4
..........aaagcaggauucagacuacaau.................................................    39    0   ya4
..........aaagcaggauucagacuacaaua................................................    6     0   ya4
..........aaagcaggauucagacuacaauau...............................................    15    0   ya4
..........aaagcaggauucagacuacaauaua..............................................    1     0   ya4
...........aagcaggauucagacuacaaua................................................    2     0   ya4
...........aagcaggauucagacuacaauau...............................................    11    0   ya4
...........aagcaggauucagacuacaauauag.............................................    2     0   ya4
...........aagcaggauucagacuacaauauagcu...........................................    2     0   ya4
............................caauauagcugcuaagugcu.................................    1     0   ya4

.........uaaagcaggauucagacuaca...................................................    2     0   ku1
.........uaaagcaggauucagacuacaa..................................................    2     0   ku1
.........uaaagcaggauucagacuacaau.................................................    3     0   ku1
.........uaaagcaggauucagacuacaauau...............................................    2     0   ku1
..........aaagcaggauucagacua.....................................................    1     0   ku1
..........aaagcaggauucagacuaca...................................................    55    0   ku1
..........aaagcaggauucagacuacaa..................................................    38    0   ku1
..........aaagcaggauucagacuacaau.................................................    50    0   ku1
..........aaagcaggauucagacuacaaua................................................    42    0   ku1
..........aaagcaggauucagacuacaauau...............................................    201   0   ku1
..........aaagcaggauucagacuacaauaua..............................................    1     0   ku1
..........aaagcaggauucagacuacaauauag.............................................    1     0   ku1
..........aaagcaggauucagacuacaauauagc............................................    1     0   ku1
...........aagcaggauucagacuac....................................................    1     0   ku1
...........aagcaggauucagacuacaa..................................................    2     0   ku1
...........aagcaggauucagacuacaau.................................................    6     0   ku1
...........aagcaggauucagacuacaaua................................................    7     0   ku1
...........aagcaggauucagacuacaauau...............................................    24    0   ku1
...........aagcaggauucagacuacaauaua..............................................    1     0   ku1
........................................uaagugcuguguugucguuccc...................    1     0   ku1

.........uaaagcaggauucagacuac....................................................    1     0   j23
.........uaaagcaggauucagacuaca...................................................    1     0   j23
.........uaaagcaggauucagacuacaa..................................................    4     0   j23
.........uaaagcaggauucagacuacaau.................................................    1     0   j23
```

Mature Star

gauuuccacuaaagcaggauucagacuacaauauagcugcuaagugcuguguugucguucccccugcuuaaaauaaaguug			
..........aaagcaggauucagacuac..	1	0	j23
..........aaagcaggauucagacuaca...	12	0	j23
..........aaagcaggauucagacuacaa..	17	0	j23
..........aaagcaggauucagacuacaau...	25	0	j23
..........aaagcaggauucagacuacaaua..	24	0	j23
..........aaagcaggauucagacuacaauau...	158	0	j23
..........aaagcaggauucagacuacaauaua..	1	0	j23
...........aagcaggauucagacuac..	1	0	j23
...........aagcaggauucagacuacaa..	2	0	j23
...........aagcaggauucagacuacaau...	1	0	j23
...........aagcaggauucagacuacaaua..	1	0	j23
...........aagcaggauucagacuacaauau...	28	0	j23
...........aagcaggauucagacuacaauaua..	1	0	j23
....uguugucguucccccugcuuaa..........	1	0	j23
.........uaaagcaggauucagacu..	1	0	s05
.........uaaagcaggauucagacuacaa..	1	0	s05
.........uaaagcaggauucagacuacaau...	3	0	s05
.........uaaagcaggauucagacuacaauau...	1	0	s05
..........aaagcaggauucagacua...	3	0	s05
..........aaagcaggauucagacuac..	5	0	s05
..........aaagcaggauucagacuaca...	35	0	s05
..........aaagcaggauucagacuacaa..	22	0	s05
..........aaagcaggauucagacuacaau...	33	0	s05
..........aaagcaggauucagacuacaaua..	29	0	s05
..........aaagcaggauucagacuacaauau...	80	0	s05
...........aagcaggauucagacuac..	4	0	s05
...........aagcaggauucagacuaca...	2	0	s05
...........aagcaggauucagacuacaa..	5	0	s05
...........aagcaggauucagacuacaau...	26	0	s05
...........aagcaggauucagacuacaaua..	6	0	s05
...........aagcaggauucagacuacaauau...	20	0	s05
...........aagcaggauucagacuacaauaua..	2	0	s05
...........aagcaggauucagacuacaauauag...	2	0	s05
...........aagcaggauucagacuacaauauagc..	2	0	s05
............agcaggauucagacuacaau...	2	0	s05
.........uaaagcaggauucagacuaca...	1	0	ta2
.........uaaagcaggauucagacuacaau...	2	0	ta2
.........uaaagcaggauucagacuacaauau...	1	0	ta2
..........aaagcaggauucagacua...	1	0	ta2
..........aaagcaggauucagacuac..	4	0	ta2
..........aaagcaggauucagacuaca...	20	0	ta2
..........aaagcaggauucagacuacaa..	27	0	ta2
..........aaagcaggauucagacuacaau...	42	0	ta2
..........aaagcaggauucagacuacaaua..	28	0	ta2
..........aaagcaggauucagacuacaauau...	170	0	ta2
..........aaagcaggauucagacuacaauaua..	1	0	ta2
..........aaagcaggauucagacuacaauauagcu...	2	0	ta2
...........aagcaggauucagacuac..	1	0	ta2
...........aagcaggauucagacuacaa..	2	0	ta2
...........aagcaggauucagacuacaau...	5	0	ta2
...........aagcaggauucagacuacaaua..	4	0	ta2
...........aagcaggauucagacuacaauau...	37	0	ta2
...........aagcaggauucagacuacaauauagcu...	1	0	ta2
...........aagcaggauucagacuacaauauagcug..	1	0	ta2
.........uaaagcaggauucagacuac..	2	0	j24
.........uaaagcaggauucagacuaca...	4	0	j24
..........aaagcaggauucagacua...	3	0	j24
..........aaagcaggauucagacuac..	12	0	j24
..........aaagcaggauucagacuaca...	427	0	j24
..........aaagcaggauucagacuacaa..	134	0	j24
..........aaagcaggauucagacuacaau...	146	0	j24
..........aaagcaggauucagacuacaaua..	83	0	j24
..........aaagcaggauucagacuacaauau...	67	0	j24
..........aaagcaggauucagacuacaauaua..	1	0	j24
...........aagcaggauucagacuac..	9	0	j24
...........aagcaggauucagacuaca...	13	0	j24
...........aagcaggauucagacuacaa..	3	0	j24

```
                 Mature                            Star
gauuuccacuaaagcaggauucagacuacaauauagcugcuaagugcuguguugucguucccccugcuuaaaauaaaguug
...........aagcaggauucagacuacaau.................................................    16    0    j24
...........aagcaggauucagacuacaaua................................................    14    0    j24
...........aagcaggauucagacuacaauau...............................................    27    0    j24
............agcaggauucagacuacaau.................................................    3     0    j24

.........uaaagcaggauucagacuac....................................................    1     0    k11
.........uaaagcaggauucagacuaca...................................................    1     0    k11
.........uaaagcaggauucagacuacaa..................................................    3     0    k11
.........uaaagcaggauucagacuacaau.................................................    2     0    k11
.........uaaagcaggauucagacuacaauau...............................................    2     0    k11
..........aaagcaggauucagacua.....................................................    2     0    k11
..........aaagcaggauucagacuac....................................................    15    0    k11
..........aaagcaggauucagacuaca...................................................    30    0    k11
..........aaagcaggauucagacuacaa..................................................    26    0    k11
..........aaagcaggauucagacuacaau.................................................    55    0    k11
..........aaagcaggauucagacuacaaua................................................    19    0    k11
..........aaagcaggauucagacuacaauau...............................................    231   0    k11
..........aaagcaggauucagacuacaauauag.............................................    1     0    k11
...........aagcaggauucagacuac....................................................    11    0    k11
...........aagcaggauucagacuaca...................................................    5     0    k11
...........aagcaggauucagacuacaa..................................................    2     0    k11
...........aagcaggauucagacuacaau.................................................    18    0    k11
...........aagcaggauucagacuacaaua................................................    4     0    k11
...........aagcaggauucagacuacaauau...............................................    42    0    k11
...........aagcaggauucagacuacaauaua..............................................    1     0    k11
...........aagcaggauucagacuacaauauag.............................................    1     0    k11
...............aggauucagacuacaauau...............................................    1     0    k11

.........uaaagcaggauucagacua.....................................................    1     0    ta1
.........uaaagcaggauucagacuaca...................................................    3     0    ta1
.........uaaagcaggauucagacuacaa..................................................    1     0    ta1
..........aaagcaggauucagacua.....................................................    12    0    ta1
..........aaagcaggauucagacuac....................................................    3     0    ta1
..........aaagcaggauucagacuaca...................................................    9     0    ta1
..........aaagcaggauucagacuacaa..................................................    17    0    ta1
..........aaagcaggauucagacuacaau.................................................    42    0    ta1
..........aaagcaggauucagacuacaaua................................................    18    0    ta1
..........aaagcaggauucagacuacaauau...............................................    38    0    ta1
..........aaagcaggauucagacuacaauaua..............................................    2     0    ta1
..........aaagcaggauucagacuacaauauag.............................................    1     0    ta1
..........aaagcaggauucagacuacaauauagc............................................    1     0    ta1
...........aagcaggauucagacuac....................................................    6     0    ta1
...........aagcaggauucagacuaca...................................................    3     0    ta1
...........aagcaggauucagacuacaa..................................................    5     0    ta1
...........aagcaggauucagacuacaau.................................................    8     0    ta1
...........aagcaggauucagacuacaaua................................................    11    0    ta1
...........aagcaggauucagacuacaauau...............................................    22    0    ta1
...........aagcaggauucagacuacaauaua..............................................    3     0    ta1
...........aagcaggauucagacuacaauauag.............................................    2     0    ta1
...........aagcaggauucagacuacaauauagcu...........................................    1     0    ta1

.........uaaagcaggauucagacua.....................................................    2     0    s09
.........uaaagcaggauucagacuac....................................................    1     0    s09
.........uaaagcaggauucagacuacaau.................................................    1     0    s09
..........aaagcaggauucagacua.....................................................    20    0    s09
..........aaagcaggauucagacuac....................................................    35    0    s09
..........aaagcaggauucagacuaca...................................................    25    0    s09
..........aaagcaggauucagacuacaa..................................................    16    0    s09
..........aaagcaggauucagacuacaau.................................................    96    0    s09
..........aaagcaggauucagacuacaaua................................................    24    0    s09
..........aaagcaggauucagacuacaauau...............................................    69    0    s09
..........aaagcaggauucagacuacaauauagc............................................    2     0    s09
...........aagcaggauucagacuac....................................................    49    0    s09
...........aagcaggauucagacuaca...................................................    24    0    s09
...........aagcaggauucagacuacaa..................................................    17    0    s09
...........aagcaggauucagacuacaau.................................................    106   0    s09
...........aagcaggauucagacuacaaua................................................    7     0    s09
...........aagcaggauucagacuacaauau...............................................    12    0    s09
...........aagcaggauucagacuacaauaua..............................................    3     0    s09
...........aagcaggauucagacuacaauauag.............................................    2     0    s09
```

```
          Mature                                 Star
gauuuccacuaaagcaggauucagacuacaauauagcugcuaagugcuguguugucguucccccugcuuaaaauaaaguug
...........aagcaggauucagacuacaauauagc............................................    6      0      s09
...........aagcaggauucagacuacaauauagcug..........................................    1      0      s09

.........uaaagcaggauucagacuacaau.................................................    1      0      NL2
..........aaagcaggauucagacuac....................................................    2      0      NL2
..........aaagcaggauucagacuaca...................................................    8      0      NL2
..........aaagcaggauucagacuacaa..................................................    10     0      NL2
..........aaagcaggauucagacuacaau.................................................    29     0      NL2
..........aaagcaggauucagacuacaaua................................................    4      0      NL2
..........aaagcaggauucagacuacaauau...............................................    20     0      NL2
..........aaagcaggauucagacuacaauaua..............................................    1      0      NL2
..........aaagcaggauucagacuacaauauagc............................................    1      0      NL2
...........aagcaggauucagacuaca...................................................    1      0      NL2
...........aagcaggauucagacuacaau.................................................    5      0      NL2
...........aagcaggauucagacuacaaua................................................    2      0      NL2
...........aagcaggauucagacuacaauau...............................................    8      0      NL2
...........aagcaggauucagacuacaauaua..............................................    9      0      NL2
...........aagcaggauucagacuacaauauag.............................................    3      0      NL2
...........aagcaggauucagacuacaauauagc............................................    19     0      NL2
...........aagcaggauucagacuacaauauagcu...........................................    4      0      NL2
............agcaggauucagacuacaau.................................................    1      0      NL2
............agcaggauucagacuacaauauagc............................................    1      0      NL2
.............gcaggauucagacuacaauauagc............................................    1      0      NL2

..........aaagcaggauucagacua.....................................................    1      0      j13
..........aaagcaggauucagacuac....................................................    4      0      j13
..........aaagcaggauucagacuaca...................................................    63     0      j13
..........aaagcaggauucagacuacaa..................................................    28     0      j13
..........aaagcaggauucagacuacaau.................................................    36     0      j13
..........aaagcaggauucagacuacaaua................................................    43     0      j13
..........aaagcaggauucagacuacaauau...............................................    171    0      j13
..........aaagcaggauucagacuacaauaua..............................................    2      0      j13
...........aagcaggauucagacuaca...................................................    3      0      j13
...........aagcaggauucagacuacaa..................................................    2      0      j13
...........aagcaggauucagacuacaau.................................................    4      0      j13
...........aagcaggauucagacuacaaua................................................    2      0      j13
...........aagcaggauucagacuacaauau...............................................    19     0      j13
...........aagcaggauucagacuacaauaua..............................................    1      0      j13
...........aagcaggauucagacuacaauauagc............................................    2      0      j13
...........aagcaggauucagacuacaauauagcu...........................................    1      0      j13

.........uaaagcaggauucagacuaca...................................................    3      0      k10
.........uaaagcaggauucagacuacaau.................................................    1      0      k10
.........uaaagcaggauucagacuacaauau...............................................    2      0      k10
..........aaagcaggauucagacua.....................................................    2      0      k10
..........aaagcaggauucagacuac....................................................    12     0      k10
..........aaagcaggauucagacuaca...................................................    25     0      k10
..........aaagcaggauucagacuacaa..................................................    27     0      k10
..........aaagcaggauucagacuacaau.................................................    43     0      k10
..........aaagcaggauucagacuacaaua................................................    20     0      k10
..........aaagcaggauucagacuacaauau...............................................    150    0      k10
...........aagcaggauucagacuac....................................................    3      0      k10
...........aagcaggauucagacuaca...................................................    4      0      k10
...........aagcaggauucagacuacaa..................................................    1      0      k10
...........aagcaggauucagacuacaau.................................................    15     0      k10
...........aagcaggauucagacuacaaua................................................    1      0      k10
...........aagcaggauucagacuacaauau...............................................    33     0      k10
...........aagcaggauucagacuacaauaua..............................................    3      0      k10
............agcaggauucagacuacaau.................................................    1      0      k10

.........uaaagcaggauucagacuaca...................................................    2      0      j19
.........uaaagcaggauucagacuacaa..................................................    2      0      j19
.........uaaagcaggauucagacuacaau.................................................    3      0      j19
..........aaagcaggauucagacua.....................................................    1      0      j19
..........aaagcaggauucagacuac....................................................    4      0      j19
..........aaagcaggauucagacuaca...................................................    140    0      j19
..........aaagcaggauucagacuacaa..................................................    75     0      j19
..........aaagcaggauucagacuacaau.................................................    79     0      j19
..........aaagcaggauucagacuacaaua................................................    78     0      j19
..........aaagcaggauucagacuacaauau...............................................    167    0      j19
```

Mature **Star**

gauuuccacuaaagcaggauucagacuacaauauagcugcuaagugcuguguugucguucccccugcuuaaaauaaaguug			
..........aaagcaggauucagacuacaauaua..	1	0	j19
...........aagcaggauucagacuaca...	3	0	j19
...........aagcaggauucagacuacaa..	5	0	j19
...........aagcaggauucagacuacaau...	6	0	j19
...........aagcaggauucagacuacaaua..	10	0	j19
...........aagcaggauucagacuacaauau...	36	0	j19
...........aagcaggauucagacuacaauaua..	1	0	j19
............agcaggauucagacuacaauau...	1	0	j19
....guugucguucccccugcuuaaa.........	1	0	j19
..........aaagcaggauucagacuac..	3	0	j03
..........aaagcaggauucagacuaca...	101	0	j03
..........aaagcaggauucagacuacaa..	20	0	j03
..........aaagcaggauucagacuacaau...	20	0	j03
..........aaagcaggauucagacuacaaua..	33	0	j03
..........aaagcaggauucagacuacaauau...	46	0	j03
..........aaagcaggauucagacuacaauaua..	2	0	j03
..........aaagcaggauucagacuacaauauagcu...	2	0	j03
...........aagcaggauucagacuaca...	2	0	j03
...........aagcaggauucagacuacaau...	5	0	j03
...........aagcaggauucagacuacaaua..	2	0	j03
...........aagcaggauucagacuacaauau...	5	0	j03
...........aagcaggauucagacuacaauaua..	1	0	j03
...........aagcaggauucagacuacaauauag...	1	0	j03
.........uaaagcaggauucagacuaca...	1	0	k01
..........aaagcaggauucagacua...	1	0	k01
..........aaagcaggauucagacuac..	6	0	k01
..........aaagcaggauucagacuaca...	16	0	k01
..........aaagcaggauucagacuacaa..	3	0	k01
..........aaagcaggauucagacuacaau...	26	0	k01
..........aaagcaggauucagacuacaaua..	8	0	k01
..........aaagcaggauucagacuacaauau...	49	0	k01
...........aagcaggauucagacuaca...	1	0	k01
...........aagcaggauucagacuacaa..	1	0	k01
...........aagcaggauucagacuacaaua..	1	0	k01
...........aagcaggauucagacuacaauau...	12	0	k01
....uguugucguucccccugcuua...........	1	0	k01
.........uaaagcaggauucagacuaca...	2	0	j25
.........uaaagcaggauucagacuacaa..	2	0	j25
.........uaaagcaggauucagacuacaau...	1	0	j25
..........aaagcaggauucagacua...	13	0	j25
..........aaagcaggauucagacuac..	10	0	j25
..........aaagcaggauucagacuaca...	240	0	j25
..........aaagcaggauucagacuacaa..	71	0	j25
..........aaagcaggauucagacuacaau...	40	0	j25
..........aaagcaggauucagacuacaaua..	47	0	j25
..........aaagcaggauucagacuacaauau...	96	0	j25
..........aaagcaggauucagacuacaauaua..	2	0	j25
..........aaagcaggauucagacuacaauauag...	1	0	j25
...........aagcaggauucagacuac..	2	0	j25
...........aagcaggauucagacuaca...	4	0	j25
...........aagcaggauucagacuacaa..	7	0	j25
...........aagcaggauucagacuacaau...	8	0	j25
...........aagcaggauucagacuacaaua..	13	0	j25
...........aagcaggauucagacuacaauau...	16	0	j25
...........aagcaggauucagacuacaauaua..	4	0	j25
...........aagcaggauucagacuacaauauag...	1	0	j25
...........aagcaggauucagacuacaauauagc..	1	0	j25
...........aagcaggauucagacuacaauauagcu...	1	0	j25
...........aagcaggauucagacuacaauauagcug..	1	0	j25
.........uaaagcaggauucagacua...	1	0	s01
.........uaaagcaggauucagacuacaaua..	2	0	s01
.........uaaagcaggauucagacuacaauau...	5	0	s01
..........aaagcaggauucagacuac..	2	0	s01
..........aaagcaggauucagacuaca...	7	0	s01
..........aaagcaggauucagacuacaa..	6	0	s01
..........aaagcaggauucagacuacaau...	45	0	s01

Mature **Star**

```
gauuuccacuaaagcaggauucagacuacaauauagcugcuaagugcuguguugucguucccccugcuuaaaauaaaguug
..........aaagcaggauucagacuacaaua................................................  34    0    s01
..........aaagcaggauucagacuacaauau...............................................  517   0    s01
..........aaagcaggauucagacuacaauaua..............................................  1     0    s01
..........aaagcaggauucagacuacaauauag.............................................  1     0    s01
...........aagcaggauucagacuaca...................................................  1     0    s01
...........aagcaggauucagacuacaau.................................................  9     0    s01
...........aagcaggauucagacuacaaua................................................  3     0    s01
...........aagcaggauucagacuacaauau...............................................  46    0    s01
...........aagcaggauucagacuacaauaua..............................................  2     0    s01
...........aagcaggauucagacuacaauauag.............................................  1     0    s01
............agcaggauucagacuacaauau...............................................  1     0    s01
.......................................cuaagugcuguguugucguucc....................  1     0    s01

.........uaaagcaggauucagacuac....................................................  1     0    per
.........uaaagcaggauucagacuaca...................................................  3     0    per
.........uaaagcaggauucagacuacaa..................................................  2     0    per
.........uaaagcaggauucagacuacaau.................................................  1     0    per
.........uaaagcaggauucagacuacaauau...............................................  3     0    per
..........aaagcaggauucagacuac....................................................  4     0    per
..........aaagcaggauucagacuaca...................................................  52    0    per
..........aaagcaggauucagacuacaa..................................................  44    0    per
..........aaagcaggauucagacuacaau.................................................  60    0    per
..........aaagcaggauucagacuacaaua................................................  21    0    per
..........aaagcaggauucagacuacaauau...............................................  114   0    per
..........aaagcaggauucagacuacaauaua..............................................  2     0    per
..........aaagcaggauucagacuacaauauagcugcuaagugcuguguu............................  7     0    per
..........aaagcaggauucagacuacaauauagcugcuaagugcuguguug...........................  8     0    per
...........aagcaggauucagacuac....................................................  1     0    per
...........aagcaggauucagacuaca...................................................  7     0    per
...........aagcaggauucagacuacaa..................................................  1     0    per
...........aagcaggauucagacuacaau.................................................  8     0    per
...........aagcaggauucagacuacaaua................................................  12    0    per
...........aagcaggauucagacuacaauau...............................................  48    0    per
...........aagcaggauucagacuacaauauagcu...........................................  1     0    per
...........aagcaggauucagacuacaauauagcug..........................................  3     0    per
...........aagcaggauucagacuacaauauagcugcuaagugcugug..............................  1     0    per
...........aagcaggauucagacuacaauauagcugcuaagugcuguguug...........................  92    0    per

.........uaaagcaggauucagacuaca...................................................  1     0    k04
.........uaaagcaggauucagacuacaa..................................................  1     0    k04
.........uaaagcaggauucagacuacaau.................................................  3     0    k04
.........uaaagcaggauucagacuacaauau...............................................  1     0    k04
..........aaagcaggauucagacua.....................................................  4     0    k04
..........aaagcaggauucagacuac....................................................  18    0    k04
..........aaagcaggauucagacuaca...................................................  52    0    k04
..........aaagcaggauucagacuacaa..................................................  30    0    k04
..........aaagcaggauucagacuacaau.................................................  87    0    k04
..........aaagcaggauucagacuacaaua................................................  41    0    k04
..........aaagcaggauucagacuacaauau...............................................  262   0    k04
...........aagcaggauucagacuac....................................................  3     0    k04
...........aagcaggauucagacuaca...................................................  3     0    k04
...........aagcaggauucagacuacaa..................................................  4     0    k04
...........aagcaggauucagacuacaau.................................................  15    0    k04
...........aagcaggauucagacuacaaua................................................  9     0    k04
...........aagcaggauucagacuacaauau...............................................  95    0    k04
.................................................uguugucguucccccugcuua...........  1     0    k04

..........aaagcaggauucagacuacaauau...............................................  1     0    U20
.................................................uguugucguucccccugcuuaaa.........  1     0    U20
..................................................guugucguucccccugcuuaa..........  1     0    U20

.........uaaagcaggauucagacuaca...................................................  1     0    ku2
.........uaaagcaggauucagacuacaa..................................................  2     0    ku2
.........uaaagcaggauucagacuacaau.................................................  1     0    ku2
.........uaaagcaggauucagacuacaaua................................................  2     0    ku2
..........aaagcaggauucagacuac....................................................  1     0    ku2
..........aaagcaggauucagacuaca...................................................  37    0    ku2
..........aaagcaggauucagacuacaa..................................................  18    0    ku2
..........aaagcaggauucagacuacaau.................................................  32    0    ku2
..........aaagcaggauucagacuacaaua................................................  35    0    ku2
```

Mature **Star**

gauuuccacuaaagcaggauucagacuacaauauagcugcuaagugcuguguugucguucccccugcuuaaaauaaaguug

..........aaagcaggauucagacuacaauau...	160	0	ku2
..........aaagcaggauucagacuacaauaua..	1	0	ku2
..........aaagcaggauucagacuacaauauagc..	1	0	ku2
...........aagcaggauucagacuac..	1	0	ku2
...........aagcaggauucagacuacaa..	1	0	ku2
...........aagcaggauucagacuacaau...	4	0	ku2
...........aagcaggauucagacuacaaua..	3	0	ku2
...........aagcaggauucagacuacaauau...	14	0	ku2
...........aagcaggauucagacuacaauaua..	1	0	ku2
...........aagcaggauucagacuacaauauag...	2	0	ku2
...........aagcaggauucagacuacaauauagc..	3	0	ku2
...........aagcaggauucagacuacaauauagcu...	1	0	ku2
...........aagcaggauucagacuacaauauagcug..	1	0	ku2
....uguugucguucccccugcuua...........	1	0	ku2
.........uaaagcaggauucagacuacaau...	3	0	j21
.........uaaagcaggauucagacuacaauau...	1	0	j21
..........aaagcaggauucagacua...	6	0	j21
..........aaagcaggauucagacuac..	1	0	j21
..........aaagcaggauucagacuaca...	10	0	j21
..........aaagcaggauucagacuacaa..	15	0	j21
..........aaagcaggauucagacuacaau...	28	0	j21
..........aaagcaggauucagacuacaaua..	46	0	j21
..........aaagcaggauucagacuacaauau...	292	0	j21
..........aaagcaggauucagacuacaauaua..	2	0	j21
...........aagcaggauucagacuac..	3	0	j21
...........aagcaggauucagacuaca...	3	0	j21
...........aagcaggauucagacuacaa..	1	0	j21
...........aagcaggauucagacuacaau...	7	0	j21
...........aagcaggauucagacuacaaua..	4	0	j21
...........aagcaggauucagacuacaauau...	34	0	j21
...........aagcaggauucagacuacaauaua..	1	0	j21
...........aagcaggauucagacuacaauauag...	2	0	j21
...........aagcaggauucagacuacaauauagc..	2	0	j21
............agcaggauucagacuacaauau...	1	0	j21
.............gcaggauucagacuacaauaua..	1	0	j21
.........uaaagcaggauucagacuacaau...	1	0	s11
.........uaaagcaggauucagacuacaauau...	2	0	s11
..........aaagcaggauucagacua...	6	0	s11
..........aaagcaggauucagacuac..	6	0	s11
..........aaagcaggauucagacuaca...	6	0	s11
..........aaagcaggauucagacuacaa..	12	0	s11
..........aaagcaggauucagacuacaau...	63	0	s11
..........aaagcaggauucagacuacaaua..	22	0	s11
..........aaagcaggauucagacuacaauau...	186	0	s11
..........aaagcaggauucagacuacaauaua..	3	0	s11
..........aaagcaggauucagacuacaauauag...	2	0	s11
..........aaagcaggauucagacuacaauauagc..	4	0	s11
..........aaagcaggauucagacuacaauauagcug..	1	0	s11
...........aagcaggauucagacuac..	8	0	s11
...........aagcaggauucagacuaca...	7	0	s11
...........aagcaggauucagacuacaa..	6	0	s11
...........aagcaggauucagacuacaau...	25	0	s11
...........aagcaggauucagacuacaaua..	8	0	s11
...........aagcaggauucagacuacaauau...	32	0	s11
...........aagcaggauucagacuacaauaua..	3	0	s11
...........aagcaggauucagacuacaauauag...	2	0	s11
...........aagcaggauucagacuacaauauagc..	1	0	s11
...........aagcaggauucagacuacaauauagcug..	1	0	s11
...........aagcaggauucagacuacaauauagcugc...	1	0	s11
..........aaagcaggauucagacuaca...	52	0	j07
..........aaagcaggauucagacuacaa..	7	0	j07
..........aaagcaggauucagacuacaau...	22	0	j07
..........aaagcaggauucagacuacaaua..	23	0	j07
..........aaagcaggauucagacuacaauau...	15	0	j07
...........aagcaggauucagacuac..	2	0	j07
...........aagcaggauucagacuaca...	1	0	j07
...........aagcaggauucagacuacaa..	3	0	j07

Mature **Star**

```
gauuuccacuaaagcaggauucagacuacaauauagcugcuaagugcuguguugucguucccccugcuuaaaauaaaguug
...........aagcaggauucagacuacaau.................................................	8	0	j07
...........aagcaggauucagacuacaaua................................................	5	0	j07
...........aagcaggauucagacuacaauau...............................................	4	0	j07
...........aagcaggauucagacuacaauaua..............................................	5	0	j07
...........aagcaggauucagacuacaauauag.............................................	5	0	j07
...........aagcaggauucagacuacaauauagc............................................	3	0	j07
...........aagcaggauucagacuacaauauagcu...........................................	1	0	j07
...........aagcaggauucagacuacaauauagcug..........................................	4	0	j07

.........uaaagcaggauucagacuacaau.................................................	2	0	j14
.........uaaagcaggauucagacuacaauau...............................................	1	0	j14
..........aaagcaggauucagacua.....................................................	5	0	j14
..........aaagcaggauucagacuaca...................................................	12	0	j14
..........aaagcaggauucagacuacaa..................................................	17	0	j14
..........aaagcaggauucagacuacaau.................................................	33	0	j14
..........aaagcaggauucagacuacaaua................................................	22	0	j14
..........aaagcaggauucagacuacaauau...............................................	136	0	j14
..........aaagcaggauucagacuacaauaua..............................................	1	0	j14
...........aagcaggauucagacuacaa..................................................	1	0	j14
...........aagcaggauucagacuacaau.................................................	1	0	j14
...........aagcaggauucagacuacaauau...............................................	18	0	j14
...........aagcaggauucagacuacaauauagcug..........................................	1	0	j14

.........uaaagcaggauucagacuaca...................................................	2	0	k02
.........uaaagcaggauucagacuacaau.................................................	3	0	k02
.........uaaagcaggauucagacuacaauau...............................................	1	0	k02
..........aaagcaggauucagacua.....................................................	2	0	k02
..........aaagcaggauucagacuac....................................................	6	0	k02
..........aaagcaggauucagacuaca...................................................	21	0	k02
..........aaagcaggauucagacuacaa..................................................	11	0	k02
..........aaagcaggauucagacuacaau.................................................	36	0	k02
..........aaagcaggauucagacuacaaua................................................	15	0	k02
..........aaagcaggauucagacuacaauau...............................................	145	0	k02
...........aagcaggauucagacuaca...................................................	2	0	k02
...........aagcaggauucagacuacaau.................................................	4	0	k02
...........aagcaggauucagacuacaaua................................................	4	0	k02
...........aagcaggauucagacuacaauau...............................................	23	0	k02
............agcaggauucagacuacaa..................................................	1	0	k02
.................................................uguugucguucccccugcuuaa..........	1	0	k02

.........uaaagcaggauucagacu......................................................	1	0	j28
.........uaaagcaggauucagacua.....................................................	7	0	j28
.........uaaagcaggauucagacuac....................................................	8	0	j28
.........uaaagcaggauucagacuaca...................................................	4	0	j28
.........uaaagcaggauucagacuacaa..................................................	2	0	j28
.........uaaagcaggauucagacuacaau.................................................	17	0	j28
.........uaaagcaggauucagacuacaaua................................................	3	0	j28
..........aaagcaggauucagacua.....................................................	284	0	j28
..........aaagcaggauucagacuac....................................................	401	0	j28
..........aaagcaggauucagacuaca...................................................	350	0	j28
..........aaagcaggauucagacuacaa..................................................	271	0	j28
..........aaagcaggauucagacuacaau.................................................	1406	0	j28
..........aaagcaggauucagacuacaaua................................................	124	0	j28
..........aaagcaggauucagacuacaauau...............................................	102	0	j28
..........aaagcaggauucagacuacaauaua..............................................	10	0	j28
..........aaagcaggauucagacuacaauauag.............................................	28	0	j28
..........aaagcaggauucagacuacaauauagc............................................	18	0	j28
..........aaagcaggauucagacuacaauauagcu...........................................	4	0	j28
..........aaagcaggauucagacuacaauauagcug..........................................	2	0	j28
...........aagcaggauucagacuac....................................................	564	0	j28
...........aagcaggauucagacuaca...................................................	254	0	j28
...........aagcaggauucagacuacaa..................................................	289	0	j28
...........aagcaggauucagacuacaau.................................................	1678	0	j28
...........aagcaggauucagacuacaaua................................................	68	0	j28
...........aagcaggauucagacuacaauau...............................................	32	0	j28
...........aagcaggauucagacuacaauaua..............................................	8	0	j28
...........aagcaggauucagacuacaauauag.............................................	53	0	j28
...........aagcaggauucagacuacaauauagc............................................	97	0	j28
...........aagcaggauucagacuacaauauagcu...........................................	15	0	j28
...........aagcaggauucagacuacaauauagcug..........................................	9	0	j28
```

Mature Star

```
gauuuccacuaaagcaggauucagacuacaauauagcugcuaagugcuguguugucguucccccugcuuaaaauaaaguug
...........aagcaggauucagacuacaauauagcugc.........................................  1    0  j28
............agcaggauucagacuaca...................................................  3    0  j28
............agcaggauucagacuacaa..................................................  1    0  j28
............agcaggauucagacuacaau.................................................  13   0  j28
............agcaggauucagacuacaauauagc............................................  1    0  j28
............agcaggauucagacuacaauauagcug..........................................  3    0  j28
................................................guguugucguucccccugcuu............  1    0  j28
.................................................uguugucguucccccugcu.............  1    0  j28
.................................................uguugucguucccccugcuuaa..........  1    0  j28
..................................................guugucguucccccugcuuaa..........  1    0  j28
...........................................................cccccugcuuaaaauaaag...  1    0  j28

.........uaaagcaggauucagacuaca...................................................  1    0  s08
.........uaaagcaggauucagacuacaa..................................................  4    0  s08
.........uaaagcaggauucagacuacaau.................................................  1    0  s08
.........uaaagcaggauucagacuacaaua................................................  2    0  s08
.........uaaagcaggauucagacuacaauau...............................................  3    0  s08
..........aaagcaggauucagacua.....................................................  11   0  s08
..........aaagcaggauucagacuac....................................................  19   0  s08
..........aaagcaggauucagacuaca...................................................  11   0  s08
..........aaagcaggauucagacuacaa..................................................  12   0  s08
..........aaagcaggauucagacuacaau.................................................  55   0  s08
..........aaagcaggauucagacuacaaua................................................  12   0  s08
..........aaagcaggauucagacuacaauau...............................................  124  0  s08
..........aaagcaggauucagacuacaauaua..............................................  1    0  s08
..........aaagcaggauucagacuacaauauagc............................................  1    0  s08
...........aagcaggauucagacuac....................................................  42   0  s08
...........aagcaggauucagacuaca...................................................  13   0  s08
...........aagcaggauucagacuacaa..................................................  15   0  s08
...........aagcaggauucagacuacaau.................................................  80   0  s08
...........aagcaggauucagacuacaaua................................................  4    0  s08
...........aagcaggauucagacuacaauau...............................................  31   0  s08
...........aagcaggauucagacuacaauauagc............................................  2    0  s08
............agcaggauucagacuacaau.................................................  3    0  s08
............agcaggauucagacuacaauau...............................................  1    0  s08

..........aaagcaggauucagacuacaauauagcugcuaagu....................................  2    0  DLD
...........aagcaggauucagacuacaau.................................................  1    0  DLD
..............caggauucagacuacaau.................................................  3    0  DLD
................ggauucagacuacaauau...............................................  1    0  DLD

.........uaaagcaggauucagacuacaa..................................................  2    0  k03
.........uaaagcaggauucagacuacaau.................................................  1    0  k03
.........uaaagcaggauucagacuacaauau...............................................  1    0  k03
..........aaagcaggauucagacuac....................................................  9    0  k03
..........aaagcaggauucagacuaca...................................................  21   0  k03
..........aaagcaggauucagacuacaa..................................................  24   0  k03
..........aaagcaggauucagacuacaau.................................................  34   0  k03
..........aaagcaggauucagacuacaaua................................................  22   0  k03
..........aaagcaggauucagacuacaauau...............................................  126  0  k03
..........aaagcaggauucagacuacaauaua..............................................  1    0  k03
...........aagcaggauucagacuac....................................................  2    0  k03
...........aagcaggauucagacuaca...................................................  2    0  k03
...........aagcaggauucagacuacaa..................................................  4    0  k03
...........aagcaggauucagacuacaau.................................................  8    0  k03
...........aagcaggauucagacuacaaua................................................  3    0  k03
...........aagcaggauucagacuacaauau...............................................  35   0  k03

..........aaagcaggauucagacuaca...................................................  7    0  ya2
..........aaagcaggauucagacuacaa..................................................  9    0  ya2
..........aaagcaggauucagacuacaau.................................................  39   0  ya2
..........aaagcaggauucagacuacaaua................................................  2    0  ya2
..........aaagcaggauucagacuacaauau...............................................  30   0  ya2
..........aaagcaggauucagacuacaauaua..............................................  1    0  ya2
..........aaagcaggauucagacuacaauauagc............................................  2    0  ya2
...........aagcaggauucagacuacaa..................................................  2    0  ya2
...........aagcaggauucagacuacaaua................................................  4    0  ya2
...........aagcaggauucagacuacaauau...............................................  7    0  ya2
...........aagcaggauucagacuacaauaua..............................................  1    0  ya2
...........aagcaggauucagacuacaauauag.............................................  1    0  ya2
```

Mature Star

gauuuccacuaaagcaggauucagacuacaauauagcugcuaagugcuguguugucguucccccugcuuaaaauaaaguug			
...........aagcaggauucagacuacaauauagc..	1	0	ya2
...........aagcaggauucagacuacaauauagcu...	5	0	ya2
...............aggauucagacuacaauauagcu...	1	0	ya2
....uguugucguucccccugcuuaa..........	1	0	ya2
.........uaaagcaggauucagacuacaau...	1	0	NL3
..........aaagcaggauucagacuac..	1	0	NL3
..........aaagcaggauucagacuaca...	14	0	NL3
..........aaagcaggauucagacuacaa..	8	0	NL3
..........aaagcaggauucagacuacaau...	24	0	NL3
..........aaagcaggauucagacuacaaua..	4	0	NL3
..........aaagcaggauucagacuacaauau...	20	0	NL3
..........aaagcaggauucagacuacaauauag...	1	0	NL3
...........aagcaggauucagacuacaau...	1	0	NL3
...........aagcaggauucagacuacaaua..	3	0	NL3
...........aagcaggauucagacuacaauau...	5	0	NL3
...........aagcaggauucagacuacaauaua..	2	0	NL3
...........aagcaggauucagacuacaauauagc..	2	0	NL3
.........uaaagcaggauucagacuaca...	1	0	s02
.........uaaagcaggauucagacuacaa..	1	0	s02
.........uaaagcaggauucagacuacaau...	1	0	s02
.........uaaagcaggauucagacuacaaua..	2	0	s02
.........uaaagcaggauucagacuacaauau...	1	0	s02
..........aaagcaggauucagacuac..	2	0	s02
..........aaagcaggauucagacuaca...	5	0	s02
..........aaagcaggauucagacuacaa..	8	0	s02
..........aaagcaggauucagacuacaau...	16	0	s02
..........aaagcaggauucagacuacaaua..	12	0	s02
..........aaagcaggauucagacuacaauau...	107	0	s02
..........aaagcaggauucagacuacaauaua..	1	0	s02
...........aagcaggauucagacuaca...	1	0	s02
...........aagcaggauucagacuacaa..	3	0	s02
...........aagcaggauucagacuacaau...	2	0	s02
...........aagcaggauucagacuacaauau...	16	0	s02
...........aagcaggauucagacuacaauauag...	1	0	s02
...........aagcaggauucagacuacaauauagc..	1	0	s02
.........uaaagcaggauucagacuaca...	1	0	k06
.........uaaagcaggauucagacuacaa..	1	0	k06
.........uaaagcaggauucagacuacaau...	1	0	k06
.........uaaagcaggauucagacuacaaua..	1	0	k06
.........uaaagcaggauucagacuacaauau...	2	0	k06
..........aaagcaggauucagacua...	3	0	k06
..........aaagcaggauucagacuac..	15	0	k06
..........aaagcaggauucagacuaca...	70	0	k06
..........aaagcaggauucagacuacaa..	46	0	k06
..........aaagcaggauucagacuacaau...	73	0	k06
..........aaagcaggauucagacuacaaua..	40	0	k06
..........aaagcaggauucagacuacaauau...	272	0	k06
..........aaagcaggauucagacuacaauauagcu...	1	0	k06
...........aagcaggauucagacuac..	3	0	k06
...........aagcaggauucagacuaca...	4	0	k06
...........aagcaggauucagacuacaa..	7	0	k06
...........aagcaggauucagacuacaau...	7	0	k06
...........aagcaggauucagacuacaaua..	5	0	k06
...........aagcaggauucagacuacaauau...	74	0	k06
...........aagcaggauucagacuacaauaua..	1	0	k06
............agcaggauucagacuacaaua..	1	0	k06
.........uaaagcaggauucagacua...	1	0	sh2
..........aaagcaggauucagacuac..	1	0	sh2
..........aaagcaggauucagacuaca...	28	0	sh2
..........aaagcaggauucagacuacaa..	10	0	sh2
..........aaagcaggauucagacuacaau...	59	0	sh2
..........aaagcaggauucagacuacaaua..	14	0	sh2
..........aaagcaggauucagacuacaauau...	33	0	sh2
..........aaagcaggauucagacuacaauaua..	3	0	sh2
..........aaagcaggauucagacuacaauauag...	1	0	sh2
...........aagcaggauucagacuacaa..	3	0	sh2

```
          Mature                                Star
gauuuccacuaaagcaggauucagacuacaauauagcugcuaagugcuguguugucguucccccugcuuaaaauaaaguug
...........aagcaggauucagacuacaau.................................................   7    0    sh2
...........aagcaggauucagacuacaaua................................................   6    0    sh2
...........aagcaggauucagacuacaauau...............................................   5    0    sh2
...........aagcaggauucagacuacaauaua..............................................   2    0    sh2
...........aagcaggauucagacuacaauauagc............................................   2    0    sh2
............agcaggauucagacuacaauaua..............................................   1    0    sh2
.............gcaggauucagacuacaaua................................................   1    0    sh2
..............caggauucagacuacaau.................................................   4    0    sh2
..............caggauucagacuacaauau...............................................   3    0    sh2
...............aggauucagacuacaauaua..............................................   1    0    sh2
...............aggauucagacuacaauauagc............................................   1    0    sh2
................ggauucagacuacaauau...............................................   20   0    sh2
.................gauucagacuacaauaua..............................................   1    0    sh2
.................gauucagacuacaauauag.............................................   4    0    sh2
.................................................uguugucguucccccugcuua...........   2    0    sh2
.................................................uguugucguucccccugcuuaa..........   5    0    sh2
.................................................uguugucguucccccugcuuaaa.........   1    0    sh2
..................................................guugucguucccccugcuuaa..........   2    0    sh2

.........uaaagcaggauucagacuaca...................................................   2    0    j29
.........uaaagcaggauucagacuacaa..................................................   1    0    j29
.........uaaagcaggauucagacuacaau.................................................   1    0    j29
.........uaaagcaggauucagacuacaauau...............................................   2    0    j29
..........aaagcaggauucagacua.....................................................   1    0    j29
..........aaagcaggauucagacuac....................................................   9    0    j29
..........aaagcaggauucagacuaca...................................................   31   0    j29
..........aaagcaggauucagacuacaa..................................................   42   0    j29
..........aaagcaggauucagacuacaau.................................................   27   0    j29
..........aaagcaggauucagacuacaaua................................................   28   0    j29
..........aaagcaggauucagacuacaauau...............................................   86   0    j29
..........aaagcaggauucagacuacaauaua..............................................   3    0    j29
..........aaagcaggauucagacuacaauauag.............................................   1    0    j29
..........aaagcaggauucagacuacaauauagc............................................   1    0    j29
..........aaagcaggauucagacuacaauauagcug..........................................   1    0    j29
...........aagcaggauucagacuac....................................................   3    0    j29
...........aagcaggauucagacuaca...................................................   5    0    j29
...........aagcaggauucagacuacaa..................................................   3    0    j29
...........aagcaggauucagacuacaau.................................................   20   0    j29
...........aagcaggauucagacuacaaua................................................   4    0    j29
...........aagcaggauucagacuacaauau...............................................   18   0    j29
...........aagcaggauucagacuacaauaua..............................................   2    0    j29
...........aagcaggauucagacuacaauauagc............................................   2    0    j29

..........aaagcaggauucagacuac....................................................   1    0    MCF
..........aaagcaggauucagacuaca...................................................   2    0    MCF
..........aaagcaggauucagacuacaa..................................................   2    0    MCF
..........aaagcaggauucagacuacaau.................................................   1    0    MCF
...........aagcaggauucagacuac....................................................   2    0    MCF
...........aagcaggauucagacuacaau.................................................   1    0    MCF
...........aagcaggauucagacuacaauau...............................................   1    0    MCF
...............aggauucagacuacaauauagcug..........................................   1    0    MCF
................ggauucagacuacaauauagc............................................   3    0    MCF
................ggauucagacuacaauauagcugcuaagug...................................   1    0    MCF
.................................................uguugucguucccccugcuuaaa.........   2    0    MCF

.........uaaagcaggauucagacuaca...................................................   3    0    j01
..........aaagcaggauucagacua.....................................................   3    0    j01
..........aaagcaggauucagacuac....................................................   12   0    j01
..........aaagcaggauucagacuaca...................................................   88   0    j01
..........aaagcaggauucagacuacaa..................................................   16   0    j01
..........aaagcaggauucagacuacaau.................................................   23   0    j01
..........aaagcaggauucagacuacaaua................................................   18   0    j01
..........aaagcaggauucagacuacaauau...............................................   35   0    j01
...........aagcaggauucagacuaca...................................................   1    0    j01
...........aagcaggauucagacuacaau.................................................   5    0    j01
...........aagcaggauucagacuacaaua................................................   1    0    j01
...........aagcaggauucagacuacaauau...............................................   1    0    j01
...........aagcaggauucagacuacaauaua..............................................   1    0    j01
...........aagcaggauucagacuacaauauag.............................................   4    0    j01

..........aaagcaggauucagacua.....................................................   2    0    NL1
```

Mature   Star

```
gauuuccacuaaagcaggauucagacuacaauauagcugcuaagugcuguguugucguucccccugcuuaaaauaaaguug
..........aaagcaggauucagacuaca...................................................    6      0      NL1
..........aaagcaggauucagacuacaa..................................................    2      0      NL1
..........aaagcaggauucagacuacaau.................................................    3      0      NL1
..........aaagcaggauucagacuacaauau...............................................    6      0      NL1
...........aagcaggauucagacuaca...................................................    1      0      NL1
...........aagcaggauucagacuacaauau...............................................    2      0      NL1
...........aagcaggauucagacuacaauaua..............................................    1      0      NL1
...........................acaauauagcugcuaagugcu.................................    1      0      NL1

.........uaaagcaggauucagacuacaau.................................................    2      0      MBM
..........aaagcaggauucagacuac....................................................    1      0      MBM
..........aaagcaggauucagacuaca...................................................    1      0      MBM
..........aaagcaggauucagacuacaau.................................................    5      0      MBM
..........aaagcaggauucagacuacaauau...............................................    3      0      MBM
..........aaagcaggauucagacuacaauauagcug..........................................    1      0      MBM
...........aagcaggauucagacuac....................................................    1      0      MBM
...........aagcaggauucagacuacaauauagcug..........................................    1      0      MBM
................ggauucagacuacaauau...............................................    2      0      MBM
................ggauucagacuacaauauagcugc.........................................    1      0      MBM

.........uaaagcaggauucagacuaca...................................................    1      0      k07
.........uaaagcaggauucagacuacaa..................................................    1      0      k07
..........aaagcaggauucagacua.....................................................    1      0      k07
..........aaagcaggauucagacuac....................................................    11     0      k07
..........aaagcaggauucagacuaca...................................................    88     0      k07
..........aaagcaggauucagacuacaa..................................................    44     0      k07
..........aaagcaggauucagacuacaau.................................................    63     0      k07
..........aaagcaggauucagacuacaaua................................................    26     0      k07
..........aaagcaggauucagacuacaauau...............................................    141    0      k07
..........aaagcaggauucagacuacaauaua..............................................    1      0      k07
...........aagcaggauucagacuac....................................................    1      0      k07
...........aagcaggauucagacuaca...................................................    4      0      k07
...........aagcaggauucagacuacaa..................................................    4      0      k07
...........aagcaggauucagacuacaau.................................................    8      0      k07
...........aagcaggauucagacuacaaua................................................    8      0      k07
...........aagcaggauucagacuacaauau...............................................    49     0      k07
............agcaggauucagacuacaauau...............................................    1      0      k07
.................................................uguugucguucccccugcuu............    1      0      k07

..........aaagcaggauucagacua.....................................................    12     0      sh1
..........aaagcaggauucagacuac....................................................    25     0      sh1
..........aaagcaggauucagacuaca...................................................    153    0      sh1
..........aaagcaggauucagacuacaa..................................................    42     0      sh1
..........aaagcaggauucagacuacaau.................................................    48     0      sh1
..........aaagcaggauucagacuacaaua................................................    33     0      sh1
..........aaagcaggauucagacuacaauau...............................................    191    0      sh1
..........aaagcaggauucagacuacaauaua..............................................    2      0      sh1
...........aagcaggauucagacuac....................................................    3      0      sh1
...........aagcaggauucagacuaca...................................................    11     0      sh1
...........aagcaggauucagacuacaa..................................................    10     0      sh1
...........aagcaggauucagacuacaau.................................................    6      0      sh1
...........aagcaggauucagacuacaaua................................................    4      0      sh1
...........aagcaggauucagacuacaauau...............................................    42     0      sh1
...........aagcaggauucagacuacaauaua..............................................    1      0      sh1
............agcaggauucagacuacaau.................................................    1      0      sh1
.................................................uguugucguucccccugcuuaaa.........    1      0      sh1

.........uaaagcaggauucagacuacaa..................................................    2      0      li1
..........aaagcaggauucagacuacaau.................................................    2      0      li1
...........aagcaggauucagacuacaau.................................................    1      0      li1
...............aggauucagacuacaauauagcugc.........................................    1      0      li1

.........uaaagcaggauucagacuac....................................................    1      0      k13
.........uaaagcaggauucagacuaca...................................................    8      0      k13
.........uaaagcaggauucagacuacaa..................................................    3      0      k13
.........uaaagcaggauucagacuacaau.................................................    2      0      k13
..........aaagcaggauucagacua.....................................................    3      0      k13
..........aaagcaggauucagacuac....................................................    28     0      k13
..........aaagcaggauucagacuaca...................................................    127    0      k13
..........aaagcaggauucagacuacaa..................................................    96     0      k13
```

Mature **Star**

```
gauuuccacuaaagcaggauucagacuacaauauagcugcuaagugcuguguugucguucccccugcuuaaaauaaaguug
..........aaagcaggauucagacuacaau.................................................  154  0  k13
..........aaagcaggauucagacuacaaua................................................  75   0  k13
..........aaagcaggauucagacuacaauau...............................................  265  0  k13
...........aagcaggauucagacuac....................................................  5    0  k13
...........aagcaggauucagacuaca...................................................  12   0  k13
...........aagcaggauucagacuacaa..................................................  10   0  k13
...........aagcaggauucagacuacaau.................................................  23   0  k13
...........aagcaggauucagacuacaaua................................................  15   0  k13
...........aagcaggauucagacuacaauau...............................................  119  0  k13
...........aagcaggauucagacuacaauaua..............................................  1    0  k13
............agcaggauucagacuaca...................................................  1    0  k13
............agcaggauucagacuacaau.................................................  1    0  k13
...............aggauucagacuacaauau...............................................  1    0  k13
.................................................uguugucguucccccugcu.............  1    0  k13
..................................................guugucguucccccugcuua...........  1    0  k13

.........uaaagcaggauucagacuaca...................................................  1    0  j20
.........uaaagcaggauucagacuacaa..................................................  1    0  j20
.........uaaagcaggauucagacuacaau.................................................  2    0  j20
.........uaaagcaggauucagacuacaauau...............................................  1    0  j20
..........aaagcaggauucagacua.....................................................  1    0  j20
..........aaagcaggauucagacuac....................................................  3    0  j20
..........aaagcaggauucagacuaca...................................................  32   0  j20
..........aaagcaggauucagacuacaa..................................................  46   0  j20
..........aaagcaggauucagacuacaau.................................................  40   0  j20
..........aaagcaggauucagacuacaaua................................................  29   0  j20
..........aaagcaggauucagacuacaauau...............................................  127  0  j20
..........aaagcaggauucagacuacaauaua..............................................  4    0  j20
...........aagcaggauucagacuac....................................................  3    0  j20
...........aagcaggauucagacuaca...................................................  1    0  j20
...........aagcaggauucagacuacaa..................................................  1    0  j20
...........aagcaggauucagacuacaau.................................................  2    0  j20
...........aagcaggauucagacuacaaua................................................  4    0  j20
...........aagcaggauucagacuacaauau...............................................  22   0  j20
...........aagcaggauucagacuacaauaua..............................................  1    0  j20
..................................................guugucguucccccugcuuaa..........  1    0  j20

..........aaagcaggauucagacuac....................................................  1    0  A54
...........aagcaggauucagacuac....................................................  4    0  A54
...........aagcaggauucagacuaca...................................................  1    0  A54
...........aagcaggauucagacuacaau.................................................  3    0  A54
................ggauucagacuacaauau...............................................  4    0  A54
................ggauucagacuacaauauagc............................................  4    0  A54
................ggauucagacuacaauauagcugc.........................................  2    0  A54

.........uaaagcaggauucagacuacaauau...............................................  1    0  j30
..........aaagcaggauucagacua.....................................................  2    0  j30
..........aaagcaggauucagacuaca...................................................  4    0  j30
..........aaagcaggauucagacuacaa..................................................  7    0  j30
..........aaagcaggauucagacuacaau.................................................  8    0  j30
..........aaagcaggauucagacuacaaua................................................  1    0  j30
..........aaagcaggauucagacuacaauau...............................................  32   0  j30
..........aaagcaggauucagacuacaauaua..............................................  1    0  j30
...........aagcaggauucagacuacaaua................................................  1    0  j30
...........aagcaggauucagacuacaauau...............................................  3    0  j30

.........uaaagcaggauucagacuac....................................................  1    0  k12
.........uaaagcaggauucagacuaca...................................................  2    0  k12
.........uaaagcaggauucagacuacaa..................................................  1    0  k12
.........uaaagcaggauucagacuacaau.................................................  2    0  k12
..........aaagcaggauucagacua.....................................................  5    0  k12
..........aaagcaggauucagacuac....................................................  76   0  k12
..........aaagcaggauucagacuaca...................................................  265  0  k12
..........aaagcaggauucagacuacaa..................................................  103  0  k12
..........aaagcaggauucagacuacaau.................................................  101  0  k12
..........aaagcaggauucagacuacaaua................................................  21   0  k12
..........aaagcaggauucagacuacaauau...............................................  44   0  k12
...........aagcaggauucagacuac....................................................  12   0  k12
...........aagcaggauucagacuaca...................................................  14   0  k12
...........aagcaggauucagacuacaa..................................................  15   0  k12
```

Mature **Star**

```
gauuuccacuaaagcaggauucagacuacaauauagcugcuaagugcuguguugucguucccccugcuuaaaauaaaguug
...........aagcaggauucagacuacaau................................................   9    0    k12
...........aagcaggauucagacuacaaua...............................................   7    0    k12
...........aagcaggauucagacuacaauau..............................................   37   0    k12
...........aagcaggauucagacuacaauauagc...........................................   1    0    k12
............agcaggauucagacuacaauau..............................................   1    0    k12
.................................................uguugucguucccccugcuu............   2    0    k12
.................................................uguugucguucccccugcuuaa..........   1    0    k12
```

Provisional ID : hsa_can_537
Score total : 78.1
Score for star read(s) : 3.9
Score for read counts : 73.5
Score for mfe : 1.3
Score for randfold :
Score for cons. seed : -0.6
Total read count : 156
Mature read count : 145
Loop read count : 0
Star read count : 11

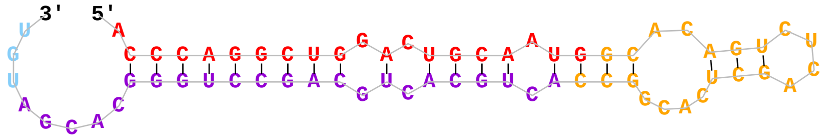

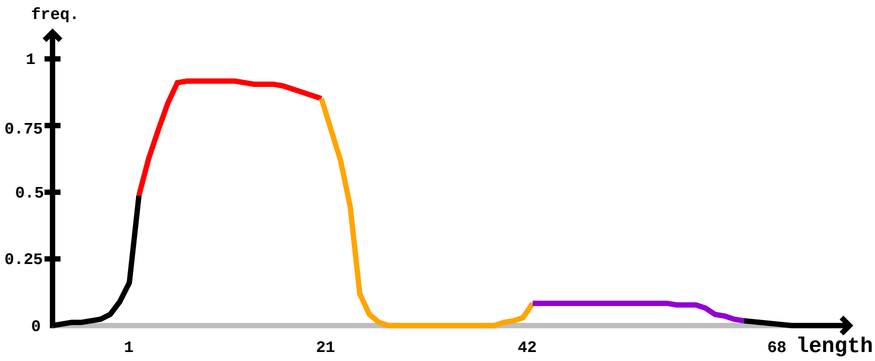


```
              Mature                                    Star
5'- cucugucacccaggcuggacugcaauggcacagucucagcucacggccacugcacugcagccugggcacgaugucggcuca  -3'   obs
    cucugucacccaggcuggacugcaauggcacagucucagcucacggccacugcacugcagccugggcacgaugucggcuca        exp
    ..(((.(((((((((((.(.((((.((((..(((....)))....)))).)))).).))))))))).....)).)))....   reads    mm    sample
    .......acccaggcuggacugcaauggc....................................................   2        0     j23

    .....ucacccaggcuggacugcaaug......................................................   1        0     ya1
    .......acccaggcuggacugcaauggca...................................................   2        0     ya1
    ..........aggcuggacugcaauggca....................................................   1        0     ya1

    ........cccaggcuggacugcaaugg.....................................................   1        0     s05

    ........cccaggcuggacugcaauggca...................................................   1        0     j26
    ........cccaggcuggacugcaauggcac..................................................   1        0     j26
    ..........aggcuggacugcaauggca....................................................   2        0     j26
    ..........aggcuggacugcaauggcaca..................................................   2        0     j26

    .......acccaggcuggacugcaaug......................................................   2        0     j24

    .......acccaggcuggacugcaauggc....................................................   1        0     k05
    .......acccaggcuggacugcaauggca...................................................   1        0     k05
    ........cccaggcuggacugcaaugg.....................................................   1        0     k05
    ........cccaggcuggacugcaauggc....................................................   1        0     k05
    .........ccaggcuggacugcaauggca...................................................   3        0     k05
    ..........caggcuggacugcaauggcaca.................................................   1        0     k05

    .......acccaggcuggacugcaaugg.....................................................   1        0     k11

    ....gucacccaggcuggacugc..........................................................   1        0     ta1
    ..........aggcuggacugcaauggca....................................................   1        0     ta1

    ........cccaggcuggacugcaaugg.....................................................   1        0     ku4
    .........ccaggcuggacugcaauggca...................................................   1        0     ku4

    .......acccaggcuggacugcaauggc....................................................   1        0     k08
    ..........caggcuggacugcaauggcac..................................................   1        0     k08

    ................................................acugcacugcagccugggcacga..........   1        0     ta3
```

```
      Mature                                  Star
cucugucacccaggcuggacugcaauggcacagucucagcucacggccacugcacugcagccugggcacgaugucggcuca

...........aggcuggacugcaauggcac..................................................    1    0    NL2

........cccaggcuggacugcaauggca...................................................    1    0    k10
.........ccaggcuggacugcaauggca...................................................    1    0    k10

.......acccaggcuggacugcaauggc....................................................    1    0    j19
.........ccaggcuggacugcaauggca...................................................    1    0    j19

................................................acugcacugcagccugggcacg...........    1    0    j03

...........aggcuggacugcaauggca...................................................    1    0    j25

.....ucacccaggcuggacugcaauggca...................................................    1    0    k01

.......acccaggcuggacugcaauggc....................................................    1    0    k04
........cccaggcuggacugcaauggca...................................................    2    0    k04

.......acccaggcuggacugcaauggc....................................................    1    0    per

....gucacccaggcuggacugca.........................................................    1    0    s01
.......acccaggcuggacugcaauggc....................................................    1    0    s01
................................................acugcacugcagccugggcac............    1    0    s01

.............................................gccacugcacugcagccu..................    1    0    U20

.....ucacccaggcuggacugcaau.......................................................    2    0    j21
.......acccaggcuggacugcaaugg.....................................................    1    0    j21
.......acccaggcuggacugcaauggc....................................................    1    0    j21
.......acccaggcuggacugcaauggca...................................................    1    0    j21
........cccaggcuggacugcaauggca...................................................    1    0    j21
................................................acugcacugcagccugggc..............    1    0    j21

.......acccaggcuggacugcaauggca...................................................    1    0    va2

......cacccaggcuggacugcaauggca...................................................    1    0    k14
.......acccaggcuggacugcaauggc....................................................    1    0    k14
........cccaggcuggacugcaauggc....................................................    1    0    k14

...............................................cacugcacugcagccugggc..............    1    0    s11

.......acccaggcuggacugcaauggc....................................................    1    0    j31
........cccaggcuggacugcaauggcacag................................................    2    0    j31

.......acccaggcuggacugcaaug......................................................    1    0    j27

.......acccaggcuggacugcaauggc....................................................    1    0    j14

......cacccaggcuggacugcaaugg.....................................................    1    0    k02
.......acccaggcuggacugcaauggca...................................................    2    0    k02
........cccaggcuggacugcaauggca...................................................    1    0    k02

................................................acugcacugcagccugggcacga..........    1    0    j28

......cacccaggcuggacugcaauggca...................................................    1    0    j22
.......acccaggcuggacugcaauggca...................................................    1    0    j22
.........ccaggcuggacugcaaug......................................................    1    0    j22

.......acccaggcuggacugcaauggc....................................................    1    0    s08

......cacccaggcuggacugcaaugg.....................................................    1    0    k09
.......acccaggcuggacugcaauggca...................................................    1    0    k09
..........caggcuggacugcaauggcaca.................................................    1    0    k09
...........aggcuggacugcaauggc....................................................    1    0    k09
..............................................ccacugcacugcagccugggca.............    1    0    k09

..........caggcuggacugcaauggcac..................................................    1    0    DLD

.....ucacccaggcuggacugcaaug......................................................    1    0    j16
.......acccaggcuggacugcaaugg.....................................................    1    0    j16
```

Mature Star

```
cucugucacccaggcuggacugcaauggcacagucucagcucacggccacugcacugcagccugggcacgaugucggcuca
.........ccaggcuggacugcaauggca...................................................   1   0   j16
..........caggcuggacugcaauggca...................................................   1   0   j16
..........caggcuggacugcaauggcac..................................................   2   0   j16
................................................acugcacugcagccugggcacga..........   1   0   j16

.....ucacccaggcuggacugcaaugg.....................................................   1   0   k03
.......acccaggcuggacugcaauggc....................................................   1   0   k03
.......acccaggcuggacugcaauggca...................................................   1   0   k03
........cccaggcuggacugcaauggca...................................................   1   0   k03
...........aggcuggacugcaauggca...................................................   1   0   k03
............ggcuggacugcaauggcac..................................................   1   0   k03

......cacccaggcuggacugcaaugg.....................................................   1   0   j09
.........ccaggcuggacugcaauggca...................................................   1   0   j09

.......acccaggcuggacugcaaugg.....................................................   1   0   ku3

..cugucacccaggcuggacugca.........................................................   1   0   ku6

.......acccaggcuggacugcaaug......................................................   1   0   ya2

.......acccaggcuggacugcaauggc....................................................   1   0   s12
...........aggcuggacugcaauggc....................................................   1   0   s12

.......acccaggcuggacugcaaug......................................................   4   0   ya3

.....ucacccaggcuggacugc..........................................................   1   0   k06
......cacccaggcuggacugcaa........................................................   1   0   k06
......cacccaggcuggacugcaaug......................................................   2   0   k06
........cccaggcuggacugcaauggca...................................................   1   0   k06
.........ccaggcuggacugcaauggcac..................................................   1   0   k06
..........caggcuggacugcaauggca...................................................   1   0   k06
..........caggcuggacugcaauggcac..................................................   1   0   k06

...............................................cacugcacugcagccugggc..............   1   0   HEK

.........ccaggcuggacugcaauggca...................................................   1   0   j29

cucugucacccaggcugga..............................................................   1   0   la1
...ugucacccaggcuggacug...........................................................   1   0   la1

......cacccaggcuggacugcaaugg.....................................................   1   0   sh2
.............................................gccacugcacugcagccugggc..............   1   0   sh2

........cccaggcuggacugcaaugg.....................................................   2   0   j15
........cccaggcuggacugcaauggca...................................................   1   0   j15
.........ccaggcuggacugcaauggca...................................................   1   0   j15
.........ccaggcuggacugcaauggcaca.................................................   1   0   j15

.....ucacccaggcuggacugcaauggc....................................................   1   0   j17
.......acccaggcuggacugcaauggc....................................................   2   0   j17
.......acccaggcuggacugcaauggca...................................................   1   0   j17
.........ccaggcuggacugcaauggc....................................................   1   0   j17
.........ccaggcuggacugcaauggca...................................................   1   0   j17

................................................acugcacugcagccuggg...............   2   0   MBM

.......acccaggcuggacugcaaug......................................................   1   0   NL1
.......acccaggcuggacugcaauggca...................................................   1   0   NL1

......cacccaggcuggacugcaaugg.....................................................   1   0   k07

......cacccaggcuggacugcaauggca...................................................   1   0   j12
.........ccaggcuggacugcaauggc....................................................   1   0   j12
..........caggcuggacugcaauggca...................................................   1   0   j12

cucugucacccaggcugg...............................................................   1   0   li2
.......acccaggcuggacugcaaugg.....................................................   1   0   li2
.......acccaggcuggacugcaauggc....................................................   1   0   li2
.........ccaggcuggacugcaauggc....................................................   1   0   li2
```

```
      Mature                                  Star
cucugucacccaggcuggacugcaauggcacagucucagcucacggccacugcacugcagccugggcacgaugucggcuca

......cacccaggcuggacugcaauggc....................................................    1    0    li1
........cccaggcuggacugcaauggc....................................................    1    0    li1

.......acccaggcuggacugcaaugg.....................................................    1    0    k13
.........ccaggcuggacugcaauggca...................................................    1    0    k13
................................................acugcacugcagccugggcac............    1    0    k13

.......acccaggcuggacugcaauggca...................................................    1    0    va4

...........aggcuggacugcaauggca...................................................    1    0    va3

..........caggcuggacugcaauggca...................................................    1    0    j30

....gucacccaggcuggacugcaa........................................................    1    0    ya4
.......acccaggcuggacugcaaug......................................................    5    0    ya4
........cccaggcuggacugcaauggc....................................................    1    0    ya4
..........caggcuggacugcaauggcac..................................................    1    0    ya4

.......acccaggcuggacugcaaugg.....................................................    2    0    k12
.......acccaggcuggacugcaauggca...................................................    1    0    k12
........cccaggcuggacugcaauggca...................................................    2    0    k12
..........caggcuggacugcaauggca...................................................    2    0    k12
..........caggcuggacugcaauggcac..................................................    3    0    k12

.......acccaggcuggacugcaaugg.....................................................    1    0    ku1
...........aggcuggacugcaauggca...................................................    1    0    ku1
```

Provisional ID : hsa_can_550
Score total : 8
Score for star read(s) : 3.9
Score for read counts : 3.7
Score for mfe : 1
Score for randfold :
Score for cons. seed : -0.6
Total read count : 19
Mature read count : 18
Loop read count : 0
Star read count : 1

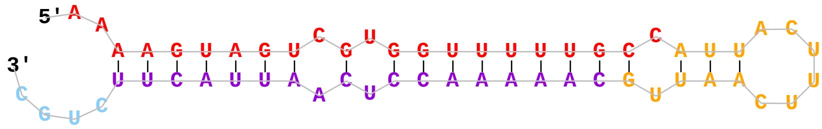

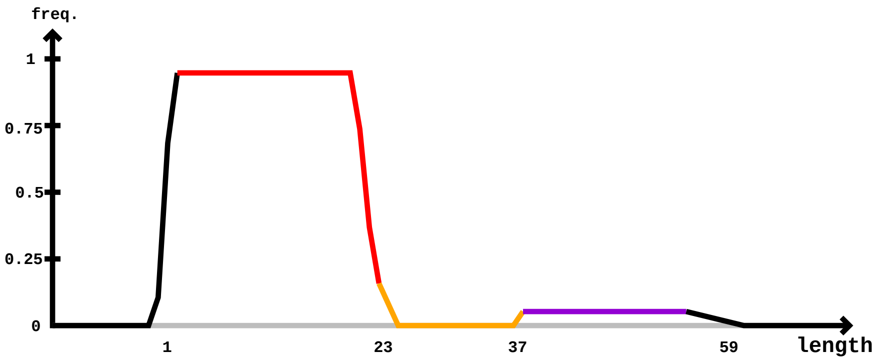

Mature Star

```
5'- uagcuuggugcaaaaguagucgugguuuuugccauuacuuucaauugcaaaaaccucaauuacuucugcacuaaccuaaua  -3'   obs
    uagcuuggugcaaaaguagucgugguuuuugccauuacuuucaauugcaaaaaccucaauuacuucugcacuaaccuaaua        exp
    (((.((((((((.(((((((.(.(((((((((.(((......))).))))))))).).))))))).)))))))).)))...    reads   mm   sample
    ..........caaaaguagucgugguuuuugc.................................................    1       0    k09
    ..........caaaaguagucgugguuuuug..................................................    1       0    k10
    ..........caaaaguagucgugguuuuug..................................................    1       0    sh1
    ...........aaaaguagucgugguuuuugcc................................................    1       0    li2
    ..........caaaaguagucgugguuuuug..................................................    1       0    j09
    .........gcaaaaguagucgugguuuu....................................................    2       0    j16
    ...............................................caaaaaccucaauuacuu................    1       0    ya3
    ..........caaaaguagucgugguuuuugc.................................................    1       0    s11
    ..........caaaaguagucgugguuuuug..................................................    1       0    s06
    ..........caaaaguagucgugguuuuug..................................................    1       0    j31
    ...........aaaaguagucgugguuuuug..................................................    2       0    j28
    ..........caaaaguagucgugguuuuugc.................................................    2       0    HEK
    ..........caaaaguagucgugguuuu....................................................    1       0    ya4
    ..........caaaaguagucgugguuuu....................................................    1       0    k12
    ...........aaaaguagucgugguuuuugcc................................................    2       0    MBM
```

Provisional ID	:	hsa_can_560
Score total	:	3.4
Score for star read(s)	:	-1.3
Score for read counts	:	0
Score for mfe	:	1.7
Score for randfold	:	
Score for cons. seed	:	3
Total read count	:	184
Mature read count	:	177
Loop read count	:	0
Star read count	:	7

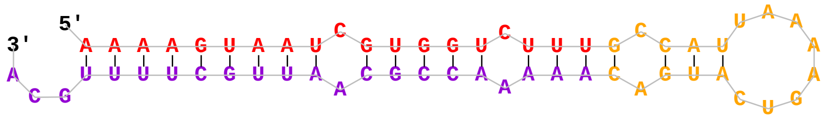

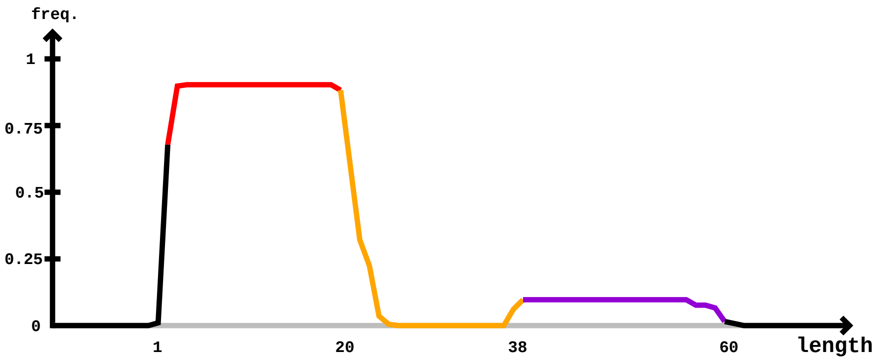

Mature Star

```
5'- agguuggugcaaaaguaaucguggucuuugccauuaaaagucaugacaaaaaccgcaauugcuuuugcaccaacuuaauaa   -3'   obs
    agguuggugcaaaaguaaucguggucuuugccauuaaaagucaugacaaaaaccgcaauugcuuuugcaccaacuuaauaa         exp
    (((((((((((((((((((.(((((.((((.(((........))).)))).))))).)))))))))))))))))))).....     reads    mm    sample
    ..........aaaaguaaucguggucuuugc..................................................     2        0     j23
    ..........aaaaguaaucguggucuuugcc.................................................     1        0     j23

    ..........aaaaguaaucguggucuuu....................................................     2        0     ya1
    ...........aaaguaaucguggucuuu....................................................     5        0     ya1
    ...............................................aaaaaccgcaauugcuuu................     1        0     ya1

    ..........aaaaguaaucguggucuuugcc.................................................     1        0     s04

    ..........aaaaguaaucguggucuu.....................................................     1        0     s05
    ..........aaaaguaaucguggucuuug...................................................     1        0     s05

    ..........aaaaguaaucguggucuuu....................................................     1        0     j26
    ..........aaaaguaaucguggucuuug...................................................     1        0     j26
    ..........aaaaguaaucguggucuuugc..................................................     1        0     j26
    ...........aaaguaaucguggucuuug...................................................     1        0     j26

    ..........aaaaguaaucguggucuuugc..................................................     2        0     va1
    ..........aaaaguaaucguggucuuugcc.................................................     1        0     va1
    ...........aaaguaaucguggucuuugcc.................................................     1        0     va1

    ..........aaaaguaaucguggucuuu....................................................     4        0     j24
    ..........aaaaguaaucguggucuuug...................................................     1        0     j24
    ...........aaaguaaucguggucuuugc..................................................     1        0     j24

    ...........aaaguaaucguggucuuug...................................................     1        0     ku5
    ...........aaaguaaucguggucuuugcca................................................     1        0     ku5

    .........caaaaguaaucguggucuuug...................................................     1        0     k11

    ..........aaaaguaaucguggucuu.....................................................     1        0     ta1

    ..........aaaaguaaucguggucuuu....................................................     2        0     s09
```

Mature   Star

```
agguuggugcaaaaguaaucguggucuuugccauuaaaagucaugacaaaaaccgcaauugcuuuugcaccaacuuaauaa

..........aaaaguaaucguggucuuu....................................................    6    0    ku4
..........aaaaguaaucguggucuuug...................................................    1    0    ku4
..............................................caaaaaccgcaauugcuuu................    1    0    ku4

..........aaaaguaaucguggucuuugc..................................................    1    0    s07

..........aaaaguaaucguggucuuug...................................................    1    0    ta3

..........aaaaguaaucguggucuuugcc.................................................    5    0    j06

..........aaaaguaaucguggucuu.....................................................    1    0    j13
..........aaaaguaaucguggucuuug...................................................    2    0    j13

..........aaaaguaaucguggucuuu....................................................    3    0    j19
..........aaaaguaaucguggucuuug...................................................    4    0    j19
..........aaaaguaaucguggucuuugc..................................................    1    0    j19
...........aaaguaaucguggucuuug...................................................    1    0    j19
...........aaaguaaucguggucuuugc..................................................    2    0    j19

..........aaaaguaaucguggucuuu....................................................    2    0    j25
...........aaaguaaucguggucuuugc..................................................    1    0    j25

..........aaaaguaaucguggucuuugcc.................................................    1    0    U20

..........aaaaguaaucguggucuuugcc.................................................    2    0    j10

..........aaaaguaaucguggucuuug...................................................    5    0    ku2
..........aaaaguaaucguggucuuugc..................................................    1    0    ku2
..........aaaaguaaucguggucuuugcc.................................................    1    0    ku2
...........aaaguaaucguggucuuugcca................................................    1    0    ku2

..........aaaaguaaucguggucuuugcc.................................................    2    0    j21
..........aaaaguaaucguggucuuugcca................................................    1    0    j21
...........aaaguaaucguggucuuu....................................................    1    0    j21

..........aaaaguaaucguggucuuug...................................................    3    0    j04
...........aaaguaaucguggucuuug...................................................    2    0    j04

..........aaaaguaaucguggucuuug...................................................    1    0    va2

..........aaaaguaaucguggucuuug...................................................    1    0    j02
...........aaaguaaucguggucuuug...................................................    1    0    j02

..........aaaaguaaucguggucuuugcc.................................................    1    0    s11

..........aaaaguaaucguggucuuugc..................................................    1    0    j31
..........aaaaguaaucguggucuuugcc.................................................    1    0    j31
...........aaaguaaucguggucuuugcc.................................................    1    0    j31

..........aaaaguaaucguggucuuug...................................................    4    0    j27
...........aaaguaaucguggucuuug...................................................    2    0    j27
...........aaaguaaucguggucuuugc..................................................    2    0    j27
...........aaaguaaucguggucuuugcc.................................................    1    0    j27

..........aaaaguaaucguggucuuu....................................................    1    0    j07
..........aaaaguaaucguggucuuug...................................................    1    0    j07

..............................................caaaaaccgcaauugcuuuugc.............    2    0    j14

..........aaaaguaaucguggucuuug...................................................    1    0    k02

...........aaaguaaucguggucuuug...................................................    1    0    j28

...........aaaguaaucguggucuuug...................................................    1    0    s03
...............................................aaaaaccgcaauugcuuuug..............    2    0    s03

..........aaaaguaaucguggucuuu....................................................    1    0    j22
..........aaaaguaaucguggucuuug...................................................    2    0    j22
..........aaaaguaaucguggucuuugcc.................................................    1    0    j22
```

Mature **Star**

```
agguuggugcaaaaguaaucguggucuuugccauuaaaagucaugacaaaaaccgcaauugcuuuugcaccaacuuaauaa
............aaguaaucguggucuuugccau...............................................     1     0     j22

..........aaaaguaaucguggucuuugcc.................................................     1     0     s08

..............................................caaaaaccgcaauugcuuuugc.............     2     0     DLD

..........aaaaguaaucguggucuuu....................................................     1     0     ku3
..........aaaaguaaucguggucuuug...................................................     1     0     ku3
...........aaaguaaucguggucuuugc..................................................     2     0     ku3

..........aaaaguaaucguggucuuug...................................................     1     0     j09

..........aaaaguaaucguggucuuug...................................................     4     0     j16
...........aaaguaaucguggucuuug...................................................     1     0     j16

..........aaaaguaaucguggucuuug...................................................     3     0     j18
...........aaaguaaucguggucuuug...................................................     1     0     j18

..........aaaaguaaucguggucuuu....................................................     3     0     ya2
...........aaaguaaucguggucuuu....................................................     1     0     ya2
..............................................caaaaaccgcaauugcuuu................     1     0     ya2

..........aaaaguaaucguggucuuu....................................................     1     0     NL3

..........aaaaguaaucguggucuuug...................................................     1     0     ku6
...........aaaguaaucguggucuuugcc.................................................     1     0     ku6

..........aaaaguaaucguggucuuug...................................................     2     0     j11

..........aaaaguaaucguggucuuugcc.................................................     1     0     s12

..........aaaaguaaucguggucuuu....................................................     1     0     ya3
..............................................caaaaaccgcaauugcuuu................     1     0     ya3

...........aaaguaaucguggucuuugcca................................................     1     0     HEK

...........aaaguaaucguggucuuug...................................................     1     0     la1

..........aaaaguaaucguggucuuugcc.................................................     2     0     j29

..............................................caaaaaccgcaauugcuuuugc.............     5     0     MCF
...............................................aaaaaccgcaauugcuuuugc.............     1     0     MCF

..........aaaaguaaucguggucuu.....................................................     1     0     j15
..........aaaaguaaucguggucuuug...................................................     3     0     j15
...........aaaguaaucguggucuuu....................................................     1     0     j15
...........aaaguaaucguggucuuugcca................................................     1     0     j15

..........aaaaguaaucguggucuuug...................................................     3     0     j17
..........aaaaguaaucguggucuuugcc.................................................     2     0     j17
...........aaaguaaucguggucuuug...................................................     1     0     j17

..........aaaaguaaucguggucuuugcc.................................................     2     0     la2

..........aaaaguaaucguggucuuugcc.................................................     1     0     MBM

..........aaaaguaaucguggucuuu....................................................     1     0     j08

..........aaaaguaaucguggucuuugcc.................................................     1     0     li1
...........aaaguaaucguggucuuugcc.................................................     1     0     li1

..........aaaaguaaucguggucuuugcc.................................................     2     0     li2

...........aaaguaaucguggucuuugc..................................................     1     0     va4
...........aaaguaaucguggucuuugcca................................................     1     0     va4

..........aaaaguaaucguggucuuug...................................................     1     0     j20
..........aaaaguaaucguggucuuugc..................................................     1     0     j20

..........aaaaguaaucguggucuuugcc.................................................     2     0     va3
```

Mature **Star**

```
agguuggugcaaaaguaaucguggucuuugccauuaaaagucaugacaaaaaccgcaauugcuuuugcaccaacuuaauaa

...............................................aaaaaccgcaauugcuuuugca............   3   0   A54

...........aaaguaaucguggucuuu....................................................   1   0   k12

..........aaaaguaaucguggucuuu....................................................   2   0   ya4

..........aaaaguaaucguggucuuug...................................................   2   0   j30

.........caaaaguaaucguggucuuug...................................................   1   0   ku1
..........aaaaguaaucguggucuuu....................................................   1   0   ku1
..........aaaaguaaucguggucuuug...................................................   3   0   ku1
...........aaaguaaucguggucuuugcc.................................................   1   0   ku1
```

Provisional ID	:	hsa_can_582
Score total	:	3.2
Score for star read(s)	:	-1.3
Score for read counts	:	0
Score for mfe	:	1.5
Score for randfold	:	
Score for cons. seed	:	3
Total read count	:	1083
Mature read count	:	1083
Loop read count	:	0
Star read count	:	0

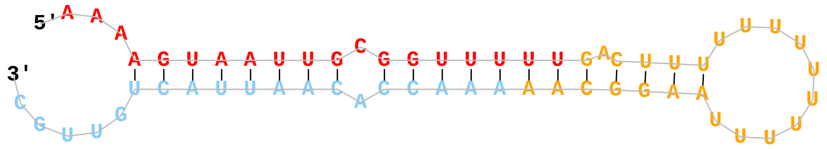

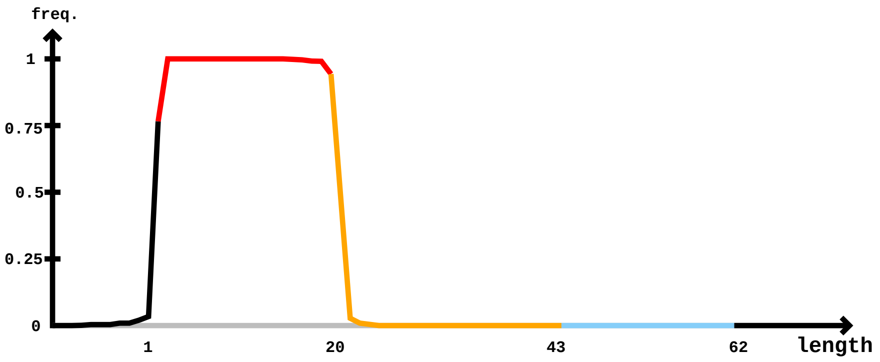

Mature Star

```
5'- gguuggugcaaaaguaauugcgguuuuugacuuuuuuuuuuuuuuaaggcaaaaaccacaauuacuguugcaccaauauaau   -3'    exp
    .((((((((((.((((((((.((((((((.((((..........)))))))))))).)))))))).))))))))))..... reads      mm         sample
    .......gcaaaaguaauugcgguuuuug.....................................................   1          0          ya1
    .........aaaaguaauugcgguuuu.......................................................   6          0          ya1
    .........aaaaguaauugcgguuuuu......................................................   44         0          ya1
    ..........aaaguaauugcgguuuuu......................................................   7          0          ya1
    ..........aaaguaauugcgguuuuug.....................................................   3          0          ya1

    .........aaaaguaauugcgguuuuug.....................................................   4          0          j26
    ..........aaaguaauugcgguuuuug.....................................................   1          0          j26

    .........aaaaguaauugcgguuuuug.....................................................   1          0          va1
    ..........aaaguaauugcgguuuuuga....................................................   1          0          va1

    .........aaaaguaauugcgguuuuug.....................................................   7          0          ku5

    ..uuggugcaaaaguaauugcggu..........................................................   1          0          k05
    .........aaaaguaauugcgguuuuu......................................................   2          0          k05
    .........aaaaguaauugcgguuuuug.....................................................   1          0          k05

    ........caaaaguaauugcgguuuuug.....................................................   1          0          s06
    .........aaaaguaauugcgguuuuug.....................................................   2          0          s06

    ..........aaaguaauugcgguuuuugac...................................................   1          0          HeL

    .........aaaaguaauugcgguuuuu......................................................   2          0          ku4
    .........aaaaguaauugcgguuuuug.....................................................   5          0          ku4
    ..........aaaguaauugcgguuuuug.....................................................   5          0          ku4

    .........aaaaguaauugcgguuuuug.....................................................   1          0          s10
    ..........aaaguaauugcgguuuuug.....................................................   2          0          s10

    ........caaaaguaauugcgguuuuug.....................................................   1          0          ta3
    .........aaaaguaauugcgguuuu.......................................................   1          0          ta3
    .........aaaaguaauugcgguuuuu......................................................   1          0          ta3
    .........aaaaguaauugcgguuuuug.....................................................   11         0          ta3
```

```
          Mature                                   Star
gguuggugcaaaaguaauugcgguuuuugacuuuuuuuuuuuuuaaggcaaaaaccacaauuacuguugcaccaauauaau
..........aaaguaauugcgguuuuu.....................................................    2     0     ta3

.........aaaaguaauugcgguuuu......................................................    16    0     j06
.........aaaaguaauugcgguuuuu.....................................................    4     0     j06
.........aaaaguaauugcgguuuuug....................................................    3     0     j06
..........aaaguaauugcgguuuuug....................................................    13    0     j06

.........aaaaguaauugcgguuuuug....................................................    3     0     j10

.........aaaaguaauugcgguuuuu.....................................................    1     0     j04
.........aaaaguaauugcgguuuuug....................................................    6     0     j04

..........aaaguaauugcgguuuuuga...................................................    1     0     k14

.........aaaaguaauugcgguuuu......................................................    1     0     va2
.........aaaaguaauugcgguuuuu.....................................................    5     0     va2
..........aaaguaauugcgguuuuugacu.................................................    1     0     va2

........caaaaguaauugcgguuuuu.....................................................    1     0     j02
.........aaaaguaauugcgguuuuu.....................................................    5     0     j02
..........aaaguaauugcgguuuuug....................................................    6     0     j02

.........aaaaguaauugcgguuuuug....................................................    1     0     j31

........caaaaguaauugcgguuuu......................................................    1     0     j27
.........aaaaguaauugcgguuuuu.....................................................    11    0     j27
.........aaaaguaauugcgguuuuug....................................................    14    0     j27
..........aaaguaauugcgguuuuug....................................................    11    0     j27

..........aaaguaauugcgguuuuug....................................................    1     0     s03

.........aaaaguaauugcgguuuuu.....................................................    1     0     j22
.........aaaaguaauugcgguuuuug....................................................    4     0     j22
..........aaaguaauugcgguuuuu.....................................................    1     0     j22
..........aaaguaauugcgguuuuug....................................................    5     0     j22

.........aaaaguaauugcgguuuuu.....................................................    2     0     k09

.........aaaaguaauugcgguuuu......................................................    1     0     B14
.........aaaaguaauugcgguuuuug....................................................    3     0     B14
..........aaaguaauugcgguuuuug....................................................    3     0     B14

.........aaaaguaauugcgguuuuu.....................................................    3     0     ku3
.........aaaaguaauugcgguuuuug....................................................    11    0     ku3
.........aaaaguaauugcgguuuuuga...................................................    2     0     ku3
..........aaaguaauugcgguuuuug....................................................    1     0     ku3

.........aaaaguaauugcgguuuuu.....................................................    1     0     j18
.........aaaaguaauugcgguuuuug....................................................    1     0     j18
.........aaaaguaauugcgguuuuuga...................................................    1     0     j18
..........aaaguaauugcgguuuuug....................................................    1     0     j18

.........aaaaguaauugcgguuuuu.....................................................    8     0     j09
.........aaaaguaauugcgguuuuug....................................................    9     0     j09
.........aaaaguaauugcgguuuuuga...................................................    1     0     j09

.........aaaaguaauugcgguuuuu.....................................................    3     0     j16
.........aaaaguaauugcgguuuuug....................................................    4     0     j16
..........aaaguaauugcgguuuuug....................................................    7     0     j16

........caaaaguaauugcgguuuuug....................................................    1     0     ku6
.........aaaaguaauugcgguuuuu.....................................................    7     0     ku6
.........aaaaguaauugcgguuuuug....................................................    9     0     ku6
..........aaaguaauugcgguuuuug....................................................    2     0     ku6

.........aaaaguaauugcgguuuuu.....................................................    1     0     j11
.........aaaaguaauugcgguuuuug....................................................    2     0     j11

.......gcaaaaguaauugcgguuuu......................................................    3     0     ya3
.........aaaaguaauugcgguuuu......................................................    9     0     ya3
```

Mature Star

```
gguuggugcaaaaguaauugcgguuuuugacuuuuuuuuuuuuuaaggcaaaaaccacaauuacuguugcaccaauauaau
.........aaaaguaauugcgguuuuu.....................................................    64    0    ya3
.........aaaaguaauugcgguuuuug....................................................    21    0    ya3
..........aaaguaauugcgguuuuu.....................................................    26    0    ya3
..........aaaguaauugcgguuuuug....................................................    3     0    ya3

.........aaaaguaauugcgguuuuug....................................................    2     0    la1

.....gugcaaaaguaauugcgguuu.......................................................    1     0    HEK
.......gcaaaaguaauugcgguuuuug....................................................    1     0    HEK
.........aaaaguaauugcgguuuuuga...................................................    1     0    HEK

.........aaaaguaauugcgguuuuu.....................................................    5     0    j15
.........aaaaguaauugcgguuuuug....................................................    10    0    j15
..........aaaguaauugcgguuuuug....................................................    7     0    j15

.......gcaaaaguaauugcgguuuuu.....................................................    1     0    j17
.........aaaaguaauugcgguuuuu.....................................................    9     0    j17
.........aaaaguaauugcgguuuuug....................................................    17    0    j17
.........aaaaguaauugcgguuuuuga...................................................    1     0    j17
..........aaaguaauugcgguuuuu.....................................................    1     0    j17
..........aaaguaauugcgguuuuug....................................................    9     0    j17

.........aaaaguaauugcgguuuu......................................................    1     0    j12
.........aaaaguaauugcgguuuuu.....................................................    2     0    j12
.........aaaaguaauugcgguuuuug....................................................    7     0    j12
..........aaaguaauugcgguuuuug....................................................    5     0    j12

.........aaaaguaauugcgguuuuug....................................................    2     0    va3

.........aaaaguaauugcgguuuuug....................................................    3     0    SW4
..........aaaguaauugcgguuuuu.....................................................    2     0    SW4

........caaaaguaauugcgguuuuu.....................................................    2     0    ya4
.........aaaaguaauugcgguuuu......................................................    2     0    ya4
.........aaaaguaauugcgguuuuu.....................................................    49    0    ya4
.........aaaaguaauugcgguuuuug....................................................    1     0    ya4
..........aaaguaauugcgguuuuu.....................................................    15    0    ya4

.........aaaaguaauugcgguuuuu.....................................................    3     0    ku1
.........aaaaguaauugcgguuuuug....................................................    14    0    ku1
..........aaaguaauugcgguuuuu.....................................................    2     0    ku1
..........aaaguaauugcgguuuuug....................................................    2     0    ku1

........caaaaguaauugcgguuuuug....................................................    2     0    j23
.........aaaaguaauugcgguuuu......................................................    3     0    j23
.........aaaaguaauugcgguuuuu.....................................................    3     0    j23
.........aaaaguaauugcgguuuuug....................................................    23    0    j23
.........aaaaguaauugcgguuuuuga...................................................    1     0    j23
..........aaaguaauugcgguuuuu.....................................................    2     0    j23
..........aaaguaauugcgguuuuug....................................................    14    0    j23
..........aaaguaauugcgguuuuuga...................................................    1     0    j23
..........aaaguaauugcgguuuuugac..................................................    1     0    j23

.........aaaaguaauugcgguuuuu.....................................................    2     0    s05
.........aaaaguaauugcgguuuuug....................................................    2     0    s05
..........aaaguaauugcgguuuuug....................................................    1     0    s05

.........aaaaguaauugcgguuuuu.....................................................    1     0    ta2
.........aaaaguaauugcgguuuuug....................................................    1     0    ta2
.........aaaaguaauugcgguuuuugacu.................................................    2     0    ta2

.........aaaaguaauugcgguuuuu.....................................................    1     0    j24
.........aaaaguaauugcgguuuuug....................................................    21    0    j24
.........aaaaguaauugcgguuuuuga...................................................    1     0    j24
..........aaaguaauugcgguuuuug....................................................    12    0    j24

..uuggugcaaaaguaauugcggu.........................................................    1     0    k11
.........aaaaguaauugcgguuuuug....................................................    2     0    k11
..........aaaguaauugcgguuuuug....................................................    1     0    k11

.........aaaaguaauugcgguuuuug....................................................    4     0    ta1
```

Mature   Star

```
gguuggugcaaaaguaauugcgguuuuugacuuuuuuuuuuuuuaaggcaaaaaccacaauuacuguugcaccaauauaau
.........aaaaguaauugcgguuuuuga...................................................    1    0    ta1
.........aaaaguaauugcgguuuuugacu.................................................    1    0    ta1
..........aaaguaauugcgguuuuug....................................................    2    0    ta1

.........aaaaguaauugcgguuuuu.....................................................    1    0    s09
.........aaaaguaauugcgguuuuug....................................................    5    0    s09
..........aaaguaauugcgguuuuu.....................................................    1    0    s09
..........aaaguaauugcgguuuuug....................................................    2    0    s09

.........aaaaguaauugcgguuuu......................................................    1    0    NL2
.........aaaaguaauugcgguuuuu.....................................................    3    0    NL2
.........aaaaguaauugcgguuuuug....................................................    6    0    NL2
..........aaaguaauugcgguuuuu.....................................................    1    0    NL2
..........aaaguaauugcgguuuuug....................................................    1    0    NL2

..........aaaguaauugcgguuuuuga...................................................    1    0    k10

.........aaaaguaauugcgguuuuu.....................................................    1    0    j13
.........aaaaguaauugcgguuuuug....................................................    10   0    j13
..........aaaguaauugcgguuuuu.....................................................    1    0    j13
..........aaaguaauugcgguuuuug....................................................    5    0    j13

.........aaaaguaauugcgguuuuu.....................................................    6    0    j19
.........aaaaguaauugcgguuuuug....................................................    21   0    j19
.........aaaaguaauugcgguuuuuga...................................................    1    0    j19
..........aaaguaauugcgguuuuu.....................................................    1    0    j19
..........aaaguaauugcgguuuuug....................................................    3    0    j19

.........aaaaguaauugcgguuuuu.....................................................    3    0    j03
.........aaaaguaauugcgguuuuug....................................................    4    0    j03

.........aaaaguaauugcgguuuuu.....................................................    5    0    j25
.........aaaaguaauugcgguuuuug....................................................    3    0    j25
.........aaaaguaauugcgguuuuuga...................................................    2    0    j25
..........aaaguaauugcgguuuuug....................................................    1    0    j25

.........aaaaguaauugcgguuuuug....................................................    1    0    k01

.........aaaaguaauugcgguuuuug....................................................    3    0    k04
..........aaaguaauugcgguuuuug....................................................    1    0    k04

.........aaaaguaauugcgguuuuu.....................................................    2    0    per
.........aaaaguaauugcgguuuuug....................................................    8    0    per
..........aaaguaauugcgguuuuu.....................................................    1    0    per
..........aaaguaauugcgguuuuug....................................................    4    0    per

........caaaaguaauugcgguuuuug....................................................    2    0    U20
.........aaaaguaauugcgguuuuug....................................................    7    0    U20
.........aaaaguaauugcgguuuuugac..................................................    1    0    U20

.........aaaaguaauugcgguuuuu.....................................................    1    0    ku2
.........aaaaguaauugcgguuuuug....................................................    7    0    ku2
..........aaaguaauugcgguuuuug....................................................    5    0    ku2

.........aaaaguaauugcgguuuuug....................................................    4    0    j21
..........aaaguaauugcgguuuuug....................................................    2    0    j21

.........aaaaguaauugcgguuuuu.....................................................    2    0    j07
.........aaaaguaauugcgguuuuug....................................................    7    0    j07

........caaaaguaauugcgguuuuug....................................................    1    0    k02

........caaaaguaauugcgguuuuug....................................................    1    0    j28
.........aaaaguaauugcgguuuu......................................................    3    0    j28
.........aaaaguaauugcgguuuuug....................................................    37   0    j28
..........aaaguaauugcgguuuuug....................................................    4    0    j28

.......gcaaaaguaauugcgguuuuug....................................................    1    0    s08

.........aaaaguaauugcgguuuuu.....................................................    3    0    DLD
```

Mature **Star**

gguuggugcaaaaguaauugcgguuuuugacuuuuuuuuuuuuuaaggcaaaaaccacaauuacuguugcaccaauauaau

.......gcaaaaguaauugcgguuuuu...	1	0	k03
........caaaaguaauugcgguuuu..	1	0	ya2
.........aaaaguaauugcgguuuu..	1	0	ya2
.........aaaaguaauugcgguuuuu...	40	0	ya2
.........aaaaguaauugcgguuuuug..	3	0	ya2
..........aaaguaauugcgguuuuu...	12	0	ya2
..........aaaguaauugcgguuuuug..	1	0	ya2
.........aaaaguaauugcgguuuuu...	2	0	NL3
.........aaaaguaauugcgguuuuug..	8	0	NL3
..........aaaguaauugcgguuuuu...	3	0	NL3
..........aaaguaauugcgguuuuug..	3	0	NL3
.guuggugcaaaaguaauugcgg..	1	0	sh2
..uuggugcaaaaguaauugcgg..	1	0	sh2
.........aaaaguaauugcgguuuu..	1	0	sh2
.........aaaaguaauugcgguuuuugac..	1	0	sh2
.........aaaaguaauugcgguuuuu...	1	0	j29
.........aaaaguaauugcgguuuuug..	4	0	j29
.........aaaaguaauugcgguuuuu...	4	0	j01
.........aaaaguaauugcgguuuuug..	9	0	j01
.........aaaaguaauugcgguuuuug..	11	0	MCF
.........aaaaguaauugcgguuuuuga...	2	0	MCF
..........aaaguaauugcgguuuuu...	1	0	MCF
..........aaaguaauugcgguuuuug..	1	0	MCF
.........aaaaguaauugcgguuuuug..	2	0	NL1
.........aaaaguaauugcgguuuuuga...	1	0	NL1
.....gugcaaaaguaauugcgguu..	1	0	MBM
.........aaaaguaauugcgguuuuug..	2	0	MBM
..........aaaguaauugcgguuuuug..	3	0	MBM
.........aaaaguaauugcgguuuuug..	1	0	k07
.........aaaaguaauugcgguuuuug..	3	0	sh1
.......gcaaaaguaauugcgguuuuug..	1	0	li1
.........aaaaguaauugcgguuuuugac..	1	0	li1
........caaaaguaauugcgguuuuu...	1	0	k13
.........aaaaguaauugcgguuuuu...	1	0	k13
.........aaaaguaauugcgguuuuu...	4	0	j20
.........aaaaguaauugcgguuuuug..	6	0	j20
..........aaaguaauugcgguuuuug..	3	0	j20
.....gugcaaaaguaauugcgguu..	4	0	A54
.......gcaaaaguaauugcgguuuuu...	3	0	A54
.........aaaaguaauugcgguuuuug..	4	0	A54
..........aaaguaauugcgguuuuug..	10	0	A54
.........aaaaguaauugcgguuuu..	1	0	k12
.........aaaaguaauugcgguuuuu...	1	0	k12
.........aaaaguaauugcgguuuuug..	4	0	k12
..........aaaguaauugcgguuuuug..	2	0	k12
..........aaaguaauugcgguuuuuga...	1	0	k12
..........aaaguaauugcgguuuuugacu...	1	0	k12

Provisional ID	:	hsa_can_633
Score total	:	812
Score for star read(s)	:	3.9
Score for read counts	:	803.1
Score for mfe	:	2
Score for randfold	:	
Score for cons. seed	:	3
Total read count	:	1587
Mature read count	:	1514
Loop read count	:	0
Star read count	:	73

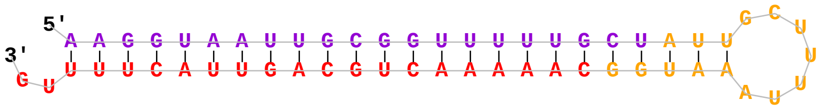

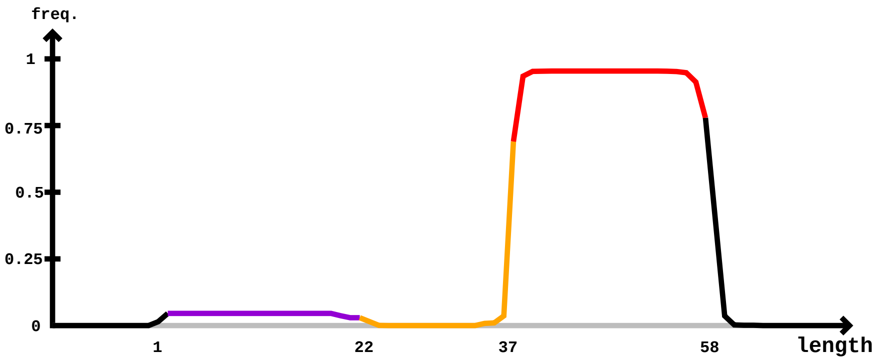


```
                Star                                Mature
5'- ggcuagugcaaagguaauugcgguuuuugcuauugcuuuuaaauggcaaaaacugcaguuacuuuugcaccaaccaauauu   -3'    obs
    ggcuagugcaaagguaauugcgguuuuugcuauugcuuuuaaauggcaaaaacugcaguuacuuuugcaccaaccaauauu          exp
    ggcuagugcaaagguaauugcgguuuuugcuauugcuuuuaaauggcaaaaacugcaguuacuuuugcaccaaccaauauu          known
    ((...(((((((((((((((((((((((((((((.......)))))))))))))))))))))))))))))...))......    reads      mm         sample
    ..........aagguaauugcgguuuuu.....................................................    1          0          ya1
    ..............................................caaaaacugcaguuacuuuu...............    1          0          ya1
    ..............................................caaaaacugcaguuacuuuug..............    2          0          ya1
    ...............................................aaaaacugcaguuacuuuu...............    3          0          ya1
    ...............................................aaaaacugcaguuacuuuugc.............    1          0          ya1

    .........aaagguaauugcgguuuuu.....................................................    1          0          j26
    ..........aagguaauugcgguuuuugcu..................................................    2          0          j26

    ...........................................uggcaaaaacugcaguuacuuu................    1          0          va1
    .............................................gcaaaaacugcaguuacuuuug..............    5          0          va1
    ..............................................caaaaacugcaguuacuuuug..............    10         0          va1
    ..............................................caaaaacugcaguuacuuuugc.............    10         0          va1
    ...............................................aaaaacugcaguuacuuuug..............    2          0          va1
    ...............................................aaaaacugcaguuacuuuugca............    1          0          va1

    ..............................................caaaaacugcaguuacuuuug..............    1          0          k05
    ..............................................caaaaacugcaguuacuuuugc.............    4          0          k05
    ...............................................aaaaacugcaguuacuuuugc.............    1          0          k05

    ...............................................aaaaacugcaguuacuuuugc.............    1          0          HeL

    ..........aagguaauugcgguuuuugcu..................................................    2          0          ku4

    ..............................................caaaaacugcaguuacuuuu...............    1          0          s10
    ..............................................caaaaacugcaguuacuuuug..............    2          0          s10
    ..............................................caaaaacugcaguuacuuuugc.............    1          0          s10
    ...............................................aaaaacugcaguuacuuuugc.............    1          0          s10
    ...............................................aaaaacugcaguuacuuuugca............    1          0          s10

    .........aaagguaauugcgguuuuugcu..................................................    1          0          k08
    ..............................................caaaaacugcaguuacuuuu...............    1          0          k08
```

```
          Star                               Mature
ggcuagugcaaagguaauugcgguuuuugcuauugcuuuuaaauggcaaaaacugcaguuacuuuugcaccaaccaauauu
..............................................caaaaacugcaguuacuuuug..............    4     0     k08

.........aaagguaauugcgguuuuug....................................................    1     0     ta3
..........aagguaauugcgguuuuu.....................................................    1     0     ta3

.............................................gcaaaaacugcaguuacuuuug..............    3     0     j10
..............................................caaaaacugcaguuacuuuugc.............    1     0     j10

..............................................caaaaacugcaguuacuuuug..............    2     0     j04

.............................................gcaaaaacugcaguuacuuuu...............    1     0     j02
.............................................gcaaaaacugcaguuacuuuug..............    4     0     j02
..............................................caaaaacugcaguuacuuu................    2     0     j02

..........aagguaauugcgguuuuugcu..................................................    2     0     va2
.............................................gcaaaaacugcaguuacuuuug..............    1     0     va2
..............................................caaaaacugcaguuacuuuug..............    1     0     va2
..............................................caaaaacugcaguuacuuuugc.............    2     0     va2

..........aagguaauugcgguuuuugcu..................................................    1     0     k14
..............................................caaaaacugcaguuacuuuug..............    1     0     k14
..............................................caaaaacugcaguuacuuuugc.............    3     0     k14
...............................................aaaaacugcaguuacuuuugca............    1     0     k14

.............................................gcaaaaacugcaguuacuuuug..............    1     0     j31
..............................................caaaaacugcaguuacuuuug..............    1     0     j31
..............................................caaaaacugcaguuacuuuugc.............    1     0     j31
...............................................aaaaacugcaguuacuuuugca............    1     0     j31

..........aagguaauugcgguuuuug....................................................    1     0     j27

..............................................caaaaacugcaguuacu..................    2     0     H52
..............................................caaaaacugcaguuacuuuu...............    7     0     H52
..............................................caaaaacugcaguuacuuuug..............    31    0     H52
..............................................caaaaacugcaguuacuuuugc.............    78    0     H52
...............................................aaaaacugcaguuacuuuu...............    5     0     H52
...............................................aaaaacugcaguuacuuuug..............    6     0     H52
...............................................aaaaacugcaguuacuuuugc.............    13    0     H52
...............................................aaaaacugcaguuacuuuugca............    12    0     H52
................................................aaaacugcaguuacuuuugc.............    4     0     H52

.............................................gcaaaaacugcaguuacuuuug..............    13    0     j05

..........aagguaauugcgguuuuugcu..................................................    1     0     s03

.............................................gcaaaaacugcaguuacuuuug..............    2     0     j22
..............................................caaaaacugcaguuacuuuu...............    1     0     j22
..............................................caaaaacugcaguuacuuuug..............    1     0     j22
..............................................caaaaacugcaguuacuuuugc.............    2     0     j22

..............................................caaaaacugcaguuacuuuug..............    2     0     k09
..............................................caaaaacugcaguuacuuuugc.............    5     0     k09
...............................................aaaaacugcaguuacuuuug..............    1     0     k09
..................................................aacugcaguuacuuuugcacca.........    1     0     k09

..............................................caaaaacugcaguuacuuuug..............    3     0     j18

.........aaagguaauugcgguuuuu.....................................................    1     0     j09
.........aaagguaauugcgguuuuugcu..................................................    1     0     j09
..............................................caaaaacugcaguuacuuuugc.............    1     0     j09

.........aaagguaauugcgguuuuugcu..................................................    1     0     ku3
..........aagguaauugcgguuuuugcu..................................................    1     0     ku3
..........aagguaauugcgguuuuugcua.................................................    1     0     ku3
...........................................uggcaaaaacugcaguuacu..................    1     0     ku3
..............................................caaaaacugcaguuacuuuug..............    1     0     ku3

..........aagguaauugcgguuuuug....................................................    1     0     j16
..............................................caaaaacugcaguuacuuuu...............    2     0     j16
..............................................caaaaacugcaguuacuuuug..............    4     0     j16
```

Star **Mature**

```
ggcuagugcaaagguaauugcgguuuuugcuauugcuuuuaaauggcaaaaacugcaguuacuuuugcaccaaccaauauu
...............................................aaaaacugcaguuacuuuu...............  1    0  j16
...............................................aaaaacugcaguuacuuuug..............  6    0  j16
................................................aaaacugcaguuacuuuug..............  3    0  j16

.........aaagguaauugcgguuuuu.....................................................  3    0  j11
..........aagguaauugcgguuuuugcuau................................................  1    0  j11
...........................................uggcaaaaacugcaguuacuuu................  4    0  j11

..............................................caaaaacugcaguuacuuuugc.............  1    0  s12

.........aaagguaauugcgguuuuu.....................................................  1    0  ya3
..........aagguaauugcgguuuuu.....................................................  2    0  ya3
..............................................caaaaacugcaguuacuuu................  2    0  ya3
..............................................caaaaacugcaguuacuuuu...............  3    0  ya3
..............................................caaaaacugcaguuacuuuug..............  1    0  ya3
...............................................aaaaacugcaguuacuuuu...............  2    0  ya3
...............................................aaaaacugcaguuacuuuug..............  1    0  ya3
................................................aaaacugcaguuacuuuu...............  1    0  ya3

.........aaagguaauugcgguuuuugcu..................................................  3    0  HEK
..........aagguaauugcgguuuuugcu..................................................  1    0  HEK
..............................................caaaaacugcaguuacuuuugc.............  1    0  HEK

..........aagguaauugcgguuuuugcu..................................................  2    0  la1
.............................................gcaaaaacugcaguuacuuuu...............  1    0  la1

..............................................caaaaacugcaguuacuu.................  1    0  j15
..............................................caaaaacugcaguuacuuu................  28   0  j15
..............................................caaaaacugcaguuacuuuu...............  55   0  j15
..............................................caaaaacugcaguuacuuuug..............  246  0  j15
..............................................caaaaacugcaguuacuuuugc.............  20   0  j15
..............................................caaaaacugcaguuacuuuugcacca.........  1    0  j15
...............................................aaaaacugcaguuacuuu................  3    0  j15
...............................................aaaaacugcaguuacuuuu...............  24   0  j15
...............................................aaaaacugcaguuacuuuug..............  107  0  j15
...............................................aaaaacugcaguuacuuuugc.............  2    0  j15
...............................................aaaaacugcaguuacuuuugca............  14   0  j15
................................................aaaacugcaguuacuuuug..............  5    0  j15
................................................aaaacugcaguuacuuuugca............  3    0  j15

..........aagguaauugcgguuuuugcu..................................................  1    0  j17
..........aagguaauugcgguuuuugcua.................................................  1    0  j17
...........................................uggcaaaaacugcaguuacuuu................  2    0  j17
..............................................caaaaacugcaguuacuuuu...............  1    0  j17
..............................................caaaaacugcaguuacuuuug..............  4    0  j17
..............................................caaaaacugcaguuacuuuugc.............  4    0  j17
...............................................aaaaacugcaguuacuuuug..............  4    0  j17
...............................................aaaaacugcaguuacuuuugc.............  1    0  j17

.............................................gcaaaaacugcaguuacuuuug..............  1    0  la2

..........aagguaauugcgguuuuugcu..................................................  1    0  j12
..............................................caaaaacugcaguuacuuuug..............  1    0  j12
...............................................aaaaacugcaguuacuuuug..............  1    0  j12
................................................aaaacugcaguuacuuuug..............  1    0  j12

................................................aaaacugcaguuacuuuu...............  2    0  SW4

.........aaagguaauugcgguuuuug....................................................  1    0  ya4
.............................................gcaaaaacugcaguuacuuuug..............  2    0  ya4
..............................................caaaaacugcaguuacuuuu...............  10   0  ya4
..............................................caaaaacugcaguuacuuuug..............  1    0  ya4
...............................................aaaaacugcaguuacuuuug..............  4    0  ya4

.........aaagguaauugcgguuuuu.....................................................  1    0  ku1
.........aaagguaauugcgguuuuugcu..................................................  1    0  ku1
..........aagguaauugcgguuuuug....................................................  2    0  ku1
..........aagguaauugcgguuuuugcu..................................................  3    0  ku1
.............................................gcaaaaacugcaguuacuuu................  1    0  ku1
..............................................caaaaacugcaguuacuuuug..............  2    0  ku1
```

```
          Star                                 Mature
ggcuagugcaaagguaauugcgguuuuugcuauugcuuuuaaauggcaaaaacugcaguuacuuuugcaccaaccaauauu
...............................................aaaaacugcaguuacuuuu...............    1    0    ku1

.........aaagguaauugcgguuuuu.....................................................    1    0    j23
...........................................uggcaaaaacugcaguuacuu.................    1    0    j23
...........................................uggcaaaaacugcaguuacuuu................    2    0    j23
.............................................gcaaaaacugcaguuacuuuug.............    1    0    j23
..............................................caaaaacugcaguuacuuuu...............    6    0    j23
..............................................caaaaacugcaguuacuuuug..............   34    0    j23
..............................................caaaaacugcaguuacuuuugc.............   11    0    j23
...............................................aaaaacugcaguuacuuuu...............    2    0    j23
...............................................aaaaacugcaguuacuuuug..............   17    0    j23
...............................................aaaaacugcaguuacuuuugc.............    5    0    j23
...............................................aaaaacugcaguuacuuuugca............    4    0    j23
................................................aaaacugcaguuacuuuugc.............    1    0    j23

..........aagguaauugcgguuuuugcu..................................................    2    0    s05

...........................................uggcaaaaacugcaguuacuuuu...............    1    0    j24

..............................................caaaaacugcaguuacuuuug..............    1    0    k11
..............................................caaaaacugcaguuacuuuugc.............    2    0    k11
...............................................aaaaacugcaguuacuuuu...............    1    0    k11

..........aagguaauugcgguuuuugcu..................................................    1    0    s09

..............................................caaaaacugcaguuacuuuu...............   18    0    NL2
..............................................caaaaacugcaguuacuuuug..............   10    0    NL2
..............................................caaaaacugcaguuacuuuugc.............   33    0    NL2
...............................................aaaaacugcaguuacuuuu...............   13    0    NL2
...............................................aaaaacugcaguuacuuuug..............   14    0    NL2
...............................................aaaaacugcaguuacuuuugc.............   10    0    NL2
...............................................aaaaacugcaguuacuuuugca............    1    0    NL2
................................................aaaacugcaguuacuuuugc.............    1    0    NL2

..........aagguaauugcgguuuuug....................................................    1    0    j13
.............................................gcaaaaacugcaguuacuuuug.............    1    0    j13
...............................................aaaaacugcaguuacuuuug..............    1    0    j13

..........aagguaauugcgguuuuugcua.................................................    1    0    k10
..............................................caaaaacugcaguuacuuu................    1    0    k10
..............................................caaaaacugcaguuacuuuu...............    1    0    k10
..............................................caaaaacugcaguuacuuuug..............    3    0    k10
..............................................caaaaacugcaguuacuuuugc.............    5    0    k10

.........aaagguaauugcgguuuuug....................................................    2    0    j19
..............................................caaaaacugcaguuacuu.................    2    0    j19
..............................................caaaaacugcaguuacuuuu...............    4    0    j19
..............................................caaaaacugcaguuacuuuug..............   42    0    j19
..............................................caaaaacugcaguuacuuuugc.............    2    0    j19
...............................................aaaaacugcaguuacuuuu...............    1    0    j19
...............................................aaaaacugcaguuacuuuug..............    6    0    j19

..............................................caaaaacugcaguuacuuuug..............    1    0    j25
...............................................aaaaacugcaguuacuuuug..............    1    0    j25

..............................................caaaaacugcaguuacuuuug..............    4    0    k01
..............................................caaaaacugcaguuacuuuugc.............    4    0    k01
...............................................aaaaacugcaguuacuuuug..............    2    0    k01

...............................................aaaaacugcaguuacuu.................    1    0    s01

..............................................caaaaacugcaguuacuuuugc.............    3    0    k04
...............................................aaaaacugcaguuacuuuug..............    1    0    k04
...............................................aaaaacugcaguuacuuuugca............    1    0    k04

.........aaagguaauugcgguuuuug....................................................    1    0    per
..............................................caaaaacugcaguuacuuuug..............    1    0    per

.........aaagguaauugcgguuuuugcu..................................................    1    0    ku2
..........aagguaauugcgguuuuugcu..................................................    2    0    ku2
```

Star **Mature**

```
ggcuagugcaaagguaauugcgguuuuugcuauugcuuuuaaauggcaaaaacugcaguuacuuuugcaccaaccaauauu

..........aagguaauugcgguuuuugcu..................................................    6     0    U20
...........................................uggcaaaaacugcaguuacuuu................    1     0    U20
............................................ggcaaaaacugcaguuac...................    1     0    U20
.............................................gcaaaaacugcaguuacuuuug..............    2     0    U20
..............................................caaaaacugcaguuacuuuugc.............    1     0    U20

..........aagguaauugcgguuuuug....................................................    1     0    j21
..........aagguaauugcgguuuuugcu..................................................    1     0    j21
............................................ggcaaaaacugcaguuacu..................    1     0    j21
..............................................caaaaacugcaguuacuu.................    1     0    j21
..............................................caaaaacugcaguuacuuuu...............    2     0    j21
..............................................caaaaacugcaguuacuuuug..............    38    0    j21
..............................................caaaaacugcaguuacuuuugc.............    37    0    j21
...............................................aaaaacugcaguuacuuuu...............    1     0    j21
...............................................aaaaacugcaguuacuuuug..............    8     0    j21
...............................................aaaaacugcaguuacuuuugc.............    11    0    j21
...............................................aaaaacugcaguuacuuuugca............    5     0    j21
................................................aaaacugcaguuacuuuugca............    1     0    j21
................................................aaaacugcaguuacuuuugcac...........    1     0    j21


..............................................caaaaacugcaguuacuuuugc.............    1     0    s11

.........aaagguaauugcgguuuuugcu..................................................    1     0    j07

..........aagguaauugcgguuuuugcu..................................................    1     0    k02
..............................................caaaaacugcaguuacuuu................    1     0    k02
..............................................caaaaacugcaguuacuuuug..............    3     0    k02
..............................................caaaaacugcaguuacuuuugc.............    4     0    k02
...............................................aaaaacugcaguuacuuuugc.............    1     0    k02

.............................................gcaaaaacugcaguuacuuuug..............    1     0    j14
..............................................caaaaacugcaguuacuuuug..............    2     0    j14
..............................................caaaaacugcaguuacuuuugc.............    1     0    j14
...............................................aaaaacugcaguuacuuuugc.............    1     0    j14

...............................................aaaaacugcaguuacuuuu...............    2     0    j28
...............................................aaaaacugcaguuacuuuug..............    1     0    j28

..........aagguaauugcgguuuuugcua.................................................    4     0    DLD

..............................................caaaaacugcaguuacuuuug..............    3     0    k03
..............................................caaaaacugcaguuacuuuugc.............    1     0    k03
...............................................aaaaacugcaguuacuuuugc.............    1     0    k03

.........aaagguaauugcgguuuuug....................................................    1     0    ya2
..........aagguaauugcgguuuuu.....................................................    2     0    ya2
.............................................gcaaaaacugcaguuacuuuu...............    1     0    ya2
..............................................caaaaacugcaguuacuuuu...............    9     0    ya2
..............................................caaaaacugcaguuacuuuug..............    3     0    ya2
..............................................caaaaacugcaguuacuuuugc.............    1     0    ya2
...............................................aaaaacugcaguuacuuuug..............    1     0    ya2

..............................................caaaaacugcaguuacuuu................    5     0    NL3
..............................................caaaaacugcaguuacuuuu...............    15    0    NL3
..............................................caaaaacugcaguuacuuuug..............    17    0    NL3
..............................................caaaaacugcaguuacuuuugc.............    27    0    NL3
...............................................aaaaacugcaguuacuuu................    2     0    NL3
...............................................aaaaacugcaguuacuuuu...............    2     0    NL3
...............................................aaaaacugcaguuacuuuug..............    5     0    NL3
...............................................aaaaacugcaguuacuuuugc.............    9     0    NL3
...............................................aaaaacugcaguuacuuuugca............    1     0    NL3
................................................aaaacugcaguuacuuuugc.............    2     0    NL3

..............................................caaaaacugcaguuacuuuu...............    1     0    k06
..............................................caaaaacugcaguuacuuuug..............    6     0    k06
..............................................caaaaacugcaguuacuuuugc.............    3     0    k06
...............................................aaaaacugcaguuacuuuug..............    1     0    k06
...............................................aaaaacugcaguuacuuuugc.............    1     0    k06
```

Star **Mature**

```
ggcuagugcaaagguaauugcgguuuuugcuauugcuuuuaaaugg caaaaacugcaguuacuuuugcaccaaccaauauu
.................................................aaacugcaguuacuuuug..............    1    0    k06

..............................................caaaaacugcaguuacuuuugc.............    6    0    sh2
...............................................aaaaacugcaguuacuuuugca............    2    0    sh2

.............................................gcaaaaacugcaguuacuuuug..............    1    0    j29

..............................................caaaaacugcaguuacuuuug..............    2    0    j01

..............................................caaaaacugcaguuacuuuug..............    1    0    MCF
..............................................caaaaacugcaguuacuuuugc.............    1    0    MCF

..............................................caaaaacugcaguuacuuuu...............    1    0    NL1
..............................................caaaaacugcaguuacuuuug..............    6    0    NL1
..............................................caaaaacugcaguuacuuuugc.............    9    0    NL1
...............................................aaaaacugcaguuacuuuu...............    4    0    NL1
...............................................aaaaacugcaguuacuuuug..............    2    0    NL1
...............................................aaaaacugcaguuacuuuugc.............    2    0    NL1
...............................................aaaaacugcaguuacuuuugca............    1    0    NL1

..............................................caaaaacugcaguuacuuuug..............    2    0    MBM
..............................................caaaaacugcaguuacuuuugc.............    21   0    MBM
...............................................aaaaacugcaguuacuuuu...............    1    0    MBM
...............................................aaaaacugcaguuacuuuug..............    1    0    MBM
...............................................aaaaacugcaguuacuuuugc.............    2    0    MBM
...............................................aaaaacugcaguuacuuuugca............    2    0    MBM
................................................aaaacugcaguuacuuuugc.............    3    0    MBM
................................................aaaacugcaguuacuuuugcac...........    1    0    MBM

..............................................caaaaacugcaguuacuuuu...............    1    0    k07
..............................................caaaaacugcaguuacuuuug..............    1    0    k07

..............................................caaaaacugcaguuacuuuugc.............    9    0    li1
..............................................caaaaacugcaguuacuuuugca............    1    0    li1

..............................................caaaaacugcaguuacuuuug..............    3    0    k13
..............................................caaaaacugcaguuacuuuugc.............    5    0    k13
...............................................aaaaacugcaguuacuuuu...............    1    0    k13
...............................................aaaaacugcaguuacuuuug..............    2    0    k13

...............................................aaaaacugcaguuacuuuug..............    1    0    j20

............................................ggcaaaaacugcaguuacuuuu...............    1    0    k12
..............................................caaaaacugcaguuacuuuu...............    2    0    k12
..............................................caaaaacugcaguuacuuuug..............    3    0    k12
..............................................caaaaacugcaguuacuuuugc.............    1    0    k12
...............................................aaaaacugcaguuacuuuu...............    1    0    k12
...............................................aaaaacugcaguuacuuuug..............    1    0    k12
...............................................aaaaacugcaguuacuuuugc.............    1    0    k12

..............................................caaaaacugcaguuacuuuug..............    18   0    j30
..............................................caaaaacugcaguuacuuuugc.............    10   0    j30
...............................................aaaaacugcaguuacuuuug..............    8    0    j30
...............................................aaaaacugcaguuacuuuugc.............    3    0    j30
...............................................aaaaacugcaguuacuuuugca............    2    0    j30
```

Provisional ID	:	hsa_can_635
Score total	:	-0.7
Score for star read(s)	:	-1.3
Score for read counts	:	0
Score for mfe	:	1.1
Score for randfold	:	
Score for cons. seed	:	-0.6
Total read count	:	118
Mature read count	:	97
Loop read count	:	0
Star read count	:	21

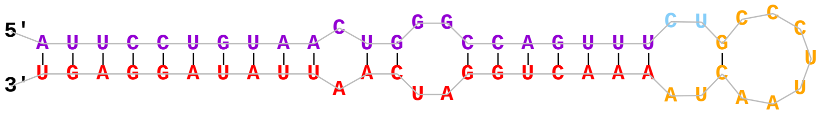

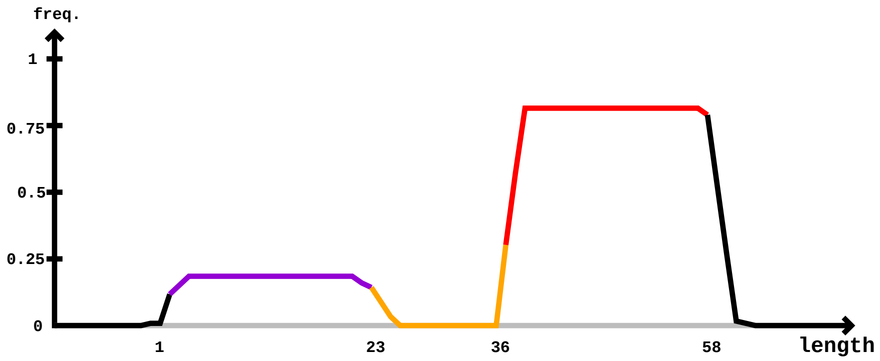

Star **Mature**

```
5'- cuuuuauuuuauuccuguaacugggccaguuucugcccuuaacuaaaacuggaucaauuauaggagugaaauaaaggucca  -3'   obs
    cuuuuauuuuauuccuguaacugggccaguuucugcccuuaacuaaaacuggaucaauuauaggagugaaauaaaggucca        exp
    cuuuuauuuuauuccuguaacugggccaguuucugcccuuaacuaaaacuggaucaauuauaggagugaaauaaaggucca        known
    ((((((((((((((((((.((..(((((((..(.......)..)))))))..)).)))))))))))))))))))....    reads      mm          sample
    .............................................aaacuggaucaauuauaggagu..............    1          0           j23
    ..............................................aacuggaucaauuauaggagug.............    1          0           j23

    ..............................................aacuggaucaauuauaggagu..............    1          0           k05

    .............................................aaacuggaucaauuauaggag...............    1          0           k11
    ..............................................aacuggaucaauuauaggagu..............    1          0           k11
    ..............................................aacuggaucaauuauaggagug.............    1          0           k11

    ...............................................acuggaucaauuauaggaguga............    1          0           ta1

    ..........auuccuguaacugggccaguuu.................................................    1          0           s06
    ..............................................aacuggaucaauuauaggagug.............    1          0           s06

    .............................................aaacuggaucaauuauaggagu..............    1          0           s09

    ...............................................acuggaucaauuauaggaguga............    1          0           k08

    ..........auuccuguaacugggccaguu..................................................    1          0           s10
    ............uccuguaacugggccaguuucu...............................................    1          0           s10

    ............uccuguaacugggccaguuucu...............................................    1          0           ta3
    .............................................aaacuggaucaauuauaggagu..............    1          0           ta3

    ............uccuguaacugggccaguuucu...............................................    1          0           k10
    ...............................................acuggaucaauuauaggaguga............    1          0           k10
    ...............................................acuggaucaauuauaggagugaaa..........    1          0           k10

    ..............................................aacuggaucaauuauaggag...............    1          0           j19

    ...............................................acuggaucaauuauaggagugaa...........    1          0           k01

    .............................................aaacuggaucaauuauaggagu..............    1          0           k04
```

Star **Mature**

```
cuuuuauuuuauuccuguaacugggccaguuucugcccuuaacuaaaacuggaucaauuauaggagugaaauaaaggucca

..............................................aacuggaucaauuauaggagug.............   1   0   j10

........uuauuccuguaacugggccagu...................................................   1   0   U20
.............................................aaacuggaucaauuauaggagu..............   4   0   U20
..............................................aacuggaucaauuauaggaguga............   4   0   U20

..........auuccuguaacugggccaguu..................................................   1   0   j21
.............................................aaacuggaucaauuauaggagu..............   3   0   j21
..............................................aacuggaucaauuauaggagu..............   5   0   j21


..............................................aacuggaucaauuauaggagu..............   1   0   k14
...............................................acuggaucaauuauaggaguga............   1   0   k14

..............................................aacuggaucaauuauaggagu..............   1   0   s11
..............................................aacuggaucaauuauaggaguga............   1   0   s11

..............................................aacuggaucaauuauaggag...............   1   0   k02
...............................................acuggaucaauuauaggaguga............   1   0   k02

.............................................aaacuggaucaauuauaggagu..............   2   0   H52

..............................................aacuggaucaauuauaggagug.............   1   0   j22
...............................................acuggaucaauuauaggaguga............   2   0   j22

..........auuccuguaacugggccaguuu.................................................   1   0   s08

.............................................aaacuggaucaauuauaggagu..............   1   0   k09

.............................................aaacuggaucaauuauaggagu..............   1   0   B14
...............................................acuggaucaauuauaggaguga............   1   0   B14

...............................................acuggaucaauuauaggaguga............   1   0   k03

.............................................aaacuggaucaauuauaggagu..............   1   0   j16

..............................................aacuggaucaauuauaggagu..............   1   0   s12

...............................................acuggaucaauuauaggaguga............   2   0   k06

...............................................acuggaucaauuauaggaguga............   1   0   la1

..........auuccuguaacugggccagu...................................................   1   0   sh2
..........auuccuguaacugggccaguu..................................................   2   0   sh2
...........uuccuguaacugggccaguu..................................................   2   0   sh2
.............................................aaacuggaucaauuauaggagu..............   7   0   sh2
..............................................aacuggaucaauuauaggagu..............   1   0   sh2

..........auuccuguaacugggccagu...................................................   1   0   HEK
.............................................aaacuggaucaauuauaggagu..............   1   0   HEK

...........uuccuguaacugggccaguuu.................................................   1   0   j17
..............................................aacuggaucaauuauaggagug.............   1   0   j17
...............................................acuggaucaauuauaggagu..............   1   0   j17

.............................................aaacuggaucaauuauaggagu..............   1   0   MBM
..............................................aacuggaucaauuauaggaguga............   1   0   MBM

..............................................aacuggaucaauuauaggagu..............   2   0   j12

..........auuccuguaacugggccaguuu.................................................   2   0   sh1
.............................................aaacuggaucaauuauaggagu..............   3   0   sh1
...............................................acuggaucaauuauaggaguga............   3   0   sh1

..........auuccuguaacugggccaguuu.................................................   2   0   li1
...........uuccuguaacugggccaguuuc................................................   1   0   li1
............uccuguaacugggccaguuucu...............................................   1   0   li1
.............................................aaacuggaucaauuauaggagu..............   5   0   li1
..............................................aacuggaucaauuauaggagu..............   2   0   li1
```

Star **Mature**

```
cuuuuauuuuauuccuguaacugggccaguuucugcccuuaacuaaaacuggaucaauuauaggagugaaauaaaggucca
..............................................aacuggaucaauuauaggagug.............   1   0   li1
...............................................acuggaucaauuauaggagug.............   2   0   li1
...............................................acuggaucaauuauaggaguga............   6   0   li1

.............................................aaacuggaucaauuauaggagu..............   2   0   k13
...............................................acuggaucaauuauaggaguga............   1   0   k13

..............................................aacuggaucaauuauaggagu..............   1   0   j20
...............................................acuggaucaauuauaggaguga............   1   0   j20

...............................................acuggaucaauuauaggaguga............   1   0   SW4

..........auuccuguaacugggccaguuu.................................................   1   0   k12
..............................................aacuggaucaauuauaggagu..............   1   0   k12
```

Provisional ID	:	hsa_can_667
Score total	:	200.3
Score for star read(s)	:	3.9
Score for read counts	:	195.4
Score for mfe	:	1.6
Score for randfold	:	
Score for cons. seed	:	-0.6
Total read count	:	395
Mature read count	:	392
Loop read count	:	0
Star read count	:	3

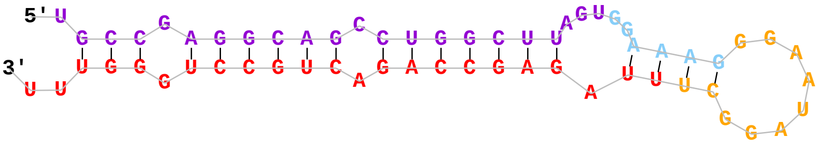

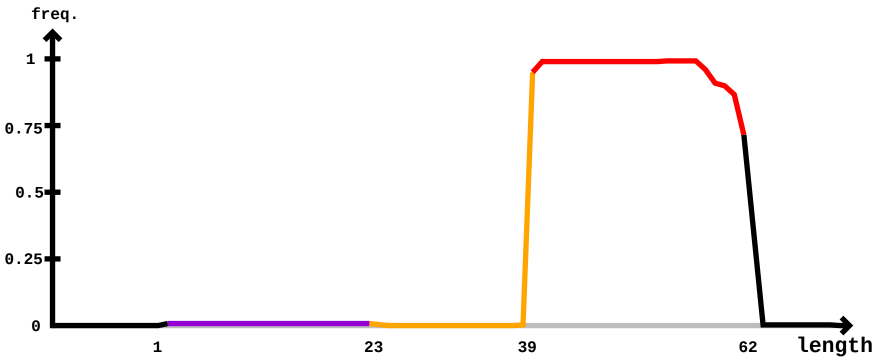


```
                Star                                  Mature
5'- ucagggaggcugccgaggcagccuggcuuaguggaaagggaauaggcuuuagagccagacugccuggguuugaaucccagc  -3'   obs
    ucagggaggcugccgaggcagccuggcuuaguggaaagggaauaggcuuuagagccagacugccuggguuugaaucccagc         exp
    ...((((..(.(((.((((((.(((((((.....((((........)))).))))))).)))))).)))..)..))))...   reads   mm   sample
    ................................................uuagagccagacugccugggu............   1       0    va1
    ................................................uuagagccagacugccuggguu...........   3       0    va1
    ................................................uuagagccagacugccuggguuu..........   2       0    va1

    ................................................uuagagccagacugccuggguuu..........   5       0    k05

    ................................................uuagagccagacugccug...............   1       0    k11
    ................................................uuagagccagacugccuggguuu..........   1       0    k11

    ................................................uuagagccagacugccuggguu...........   2       0    s06
    ................................................uuagagccagacugccuggguuu..........   3       0    s06
    ..............................................................ccuggguuugaaucccag.   1       0    s06

    ................................................uuagagccagacugccuggguuu..........   1       0    s09

    ................................................uuagagccagacugccuggguu...........   2       0    HeL

    ................................................uuagagccagacugccugggu............   1       0    s10
    ................................................uuagagccagacugccuggguu...........   4       0    s10
    ................................................uuagagccagacugccuggguuu..........   9       0    s10

    ................................................uuagagccagacugccug...............   1       0    k08
    ................................................uuagagccagacugccuggguuu..........   6       0    k08

    ................................................uuagagccagacugccuggguu...........   1       0    j13

    ................................................uuagagccagacugccug...............   2       0    k10
    ................................................uuagagccagacugccuggguuu..........   6       0    k10
    .................................................uagagccagacugccuggguuu..........   1       0    k10

    ................................................uuagagccagacugccuggguuu..........   7       0    k04

    ................................................uuagagccagacugccug...............   1       0    per
```

```
          Star                                  Mature
ucagggaggcugccgaggcagccuggcuuaguggaaagggaauaggcuuuagagccagacugccuggguuugaaucccagc
................................................uuagagccagacugccuggguu...........   4    0    per
................................................uuagagccagacugccuggguuu..........   5    0    per
.................................................uagagccagacugccuggguuu..........   1    0    per

................................................uuagagccagacugccuggguu...........   1    0    ku2

................................................uuagagccagacugccuggguu...........   2    0    U20
................................................uuagagccagacugccuggguuu..........   8    0    U20

................................................uuagagccagacugccuggguuu..........   13   0    k14

................................................uuagagccagacugccuggguuu..........   7    0    k02
.................................................uagagccagacugccuggguuu..........   1    0    k02

..........ugccgaggcagccuggcuuagu.................................................   1    0    H52
................................................uuagagccagacugccuggguu...........   1    0    H52
................................................uuagagccagacugccuggguuu..........   1    0    H52
.................................................uagagccagacugccuggguuu..........   2    0    H52

................................................uuagagccagacugccuggguu...........   1    0    j22

................................................uuagagccagacugccug...............   1    0    k09
................................................uuagagccagacugccuggguu...........   3    0    k09
................................................uuagagccagacugccuggguuu..........   18   0    k09
.................................................uagagccagacugccuggguuu..........   1    0    k09

................................................uuagagccagacugccugggu............   1    0    DLD
................................................uuagagccagacugccuggguuu..........   1    0    DLD
.................................................uagagccagacugccuggguuu..........   2    0    DLD

................................................uuagagccagacugccuggguuu..........   2    0    B14

................................................uuagagccagacugccuggguu...........   1    0    k03
................................................uuagagccagacugccuggguuu..........   4    0    k03
.................................................uagagccagacugccuggguuu..........   1    0    k03

................................................uuagagccagacugccuggguuu..........   2    0    s12

................................................uuagagccagacugccugggu............   1    0    k06
................................................uuagagccagacugccuggguuu..........   7    0    k06

................................................uuagagccagacugccug...............   1    0    la1
................................................uuagagccagacugccugggu............   1    0    la1
................................................uuagagccagacugccuggguuu..........   1    0    la1

...............................................uuuagagccagacugccugggu............   1    0    sh2
................................................uuagagccagacugccugg..............   12   0    sh2
................................................uuagagccagacugccuggg.............   3    0    sh2
................................................uuagagccagacugccugggu............   5    0    sh2
................................................uuagagccagacugccuggguu...........   17   0    sh2
................................................uuagagccagacugccuggguuu..........   80   0    sh2
.................................................uagagccagacugccuggguu...........   1    0    sh2
.................................................uagagccagacugccuggguuu..........   2    0    sh2

................................................uuagagccagacugccuggguu...........   2    0    HEK

................................................uuagagccagacugccuggguuu..........   2    0    j15

................................................uuagagccagacugccug...............   1    0    MCF
................................................uuagagccagacugccugggu............   1    0    MCF
................................................uuagagccagacugccuggguu...........   5    0    MCF
................................................uuagagccagacugccuggguuu..........   12   0    MCF
.................................................uagagccagacugccuggguu...........   1    0    MCF
.................................................uagagccagacugccuggguuu..........   1    0    MCF

................................................uuagagccagacugccuggguuu..........   2    0    la2

................................................uuagagccagacugccuggguuu..........   8    0    k07

................................................uuagagccagacugccug...............   2    0    sh1
```

Star   Mature

```
ucagggaggcugccgaggcagccuggcuuaguggaaagggaauaggcuuuagagccagacugccuggguuugaaucccagc
................................................uuagagccagacugccuggguu...........    3    0    sh1
................................................uuagagccagacugccuggguuu..........    9    0    sh1
.................................................uagagccagacugccuggguu...........    2    0    sh1

................................................uuagagccagacugccug...............    1    0    li1
................................................uuagagccagacugccugg..............    8    0    li1
................................................uuagagccagacugccuggguu...........    1    0    li1
................................................uuagagccagacugccuggguuu..........    2    0    li1

................................................uuagagccagacugccuggguu...........    1    0    k13
................................................uuagagccagacugccuggguuu..........    16   0    k13

................................................uuagagccagacugccuggguuu..........    1    0    j20

................................................uuagagccagacugccuggg.............    1    0    va3

..........ugccgaggcagccuggcuuagu.................................................    1    0    A54
................................................uuagagccagacugccug...............    2    0    A54
................................................uuagagccagacugccugggu............    1    0    A54
................................................uuagagccagacugccuggguuu..........    3    0    A54

..........ugccgaggcagccuggcuuagu.................................................    1    0    k12
................................................uuagagccagacugccuggguu...........    1    0    k12
................................................uuagagccagacugccuggguuu..........    18   0    k12

................................................uuagagccagacugccuggguu...........    1    0    SW4
................................................uuagagccagacugccuggguuu..........    8    0    SW4
```

Provisional ID	:	hsa_can_671
Score total	:	172.4
Score for star read(s)	:	3.9
Score for read counts	:	165.3
Score for mfe	:	0.1
Score for randfold	:	
Score for cons. seed	:	3
Total read count	:	336
Mature read count	:	84
Loop read count	:	0
Star read count	:	252

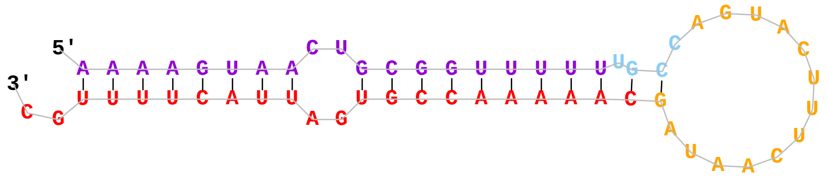

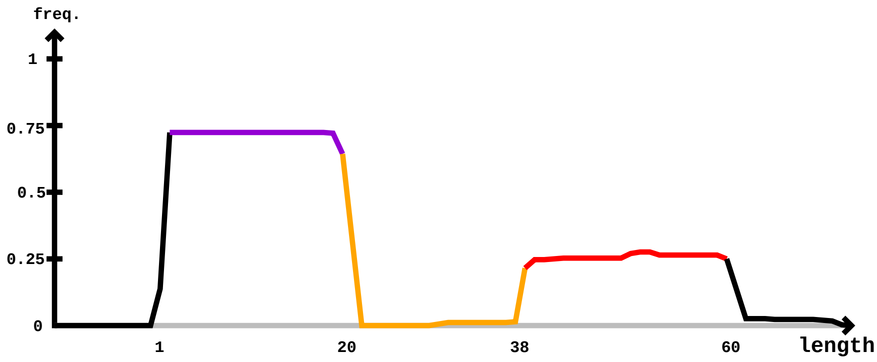

Star Mature

```
5'- agauuggguacaaaaguaacugcgguuuuuugccaguacuuucaaauagcaaaaaccgugauuacuuuugcaccaaccuaaua  -3'    obs
    agauuggguacaaaaguaacugcgguuuuuuugccaguacuuucaaauagcaaaaaccgugauuacuuuugcaccaaccuaaua        exp
    ((.(((((.(((((((((..((((((((.((..............)))))))))))..)))))))))).))))).))....   reads   mm   sample
    ..........aaaaguaacugcgguuuu......................................................   2       0    ya1
    ..........aaaaguaacugcgguuuuu.....................................................   21      0    ya1

    ..........aaaaguaacugcgguuuuu.....................................................   1       0    s05

    .........caaaaguaacugcgguuuu......................................................   3       0    j24

    .........caaaaguaacugcgguuuuu.....................................................   1       0    ku5
    ..........aaaaguaacugcgguuuuu.....................................................   4       0    ku5

    ................................................caaaaaccgugauuacuuuugc............   2       0    HeL

    ..........aaaaguaacugcgguuuuu.....................................................   5       0    ku4
    ..........aaaaguaacugcgguuuuuu....................................................   1       0    ku4

    ..........aaaaguaacugcgguuuuu.....................................................   1       0    ta3
    ..........aaaaguaacugcgguuuuuu....................................................   1       0    ta3

    .........caaaaguaacugcgguuuuu.....................................................   1       0    NL2
    ..........aaaaguaacugcgguuuuu.....................................................   14      0    NL2

    ..........aaaaguaacugcgguuuuu.....................................................   2       0    j13

    .........caaaaguaacugcgguuuuu.....................................................   1       0    j19
    ..........aaaaguaacugcgguuuu......................................................   3       0    j19
    ..........aaaaguaacugcgguuuuu.....................................................   5       0    j19

    ..........aaaaguaacugcgguuuu......................................................   1       0    k01

    .........caaaaguaacugcgguuuuu.....................................................   2       0    j25
    ..........aaaaguaacugcgguuuuu.....................................................   4       0    j25

    ...........................................................auuacuuuugcaccaaccuaau.   1       0    k04
```

Star Mature

agauugguacaaaaguaacugcgguuuuuugccaguacuuucaauagcaaaaaccgugauuacuuuugcaccaaccuaaua

....gcaaaaaccgugauuacuuuug.............	1	0	U20
....caaaaaccgugauuacuuuug.............	1	0	U20
....caaaaaccgugauuacuuuugc............	26	0	U20
....aaaaaccgugauuacuuuugc............	2	0	U20
....aaaaaccgugauuacuuuugca...........	3	0	U20
..........aaaaguaacugcgguuuuu..	1	0	j10
....caaaaaccgugauuacuuuugc............	5	0	j21
....aaaaaccgugauuacuuuugca...........	1	0	j21
..........aaaaguaacugcgguuuuuu...	1	0	j02
..........aaaaguaacugcgguuuuu..	1	0	k14
.........caaaaguaacugcgguuuuu..	2	0	va2
.........caaaaguaacugcgguuuuuu...	2	0	va2
..........aaaaguaacugcgguuuu...	1	0	va2
..........aaaaguaacugcgguuuuu..	2	0	va2
..........aaaaguaacugcgguuuuuu...	1	0	va2
....aaaaaccgugauuacuuuugc............	1	0	j07
....caaaaaccgugauuacuuuugc............	2	0	j31
....aaaaaccgugauuacuuuugca...........	1	0	j31
.........caaaaguaacugcgguuuuu..	2	0	j27
..........aaaaguaacugcgguuuuu..	18	0	j27
.........caaaaguaacugcgguuuuu..	5	0	j28
..........aaaaguaacugcgguuuu...	2	0	j28
..........aaaaguaacugcgguuuuu..	5	0	j28
....caaaaaccgugauuacuuuugc............	4	0	H52
.........caaaaguaacugcgguuuu...	1	0	j22
..........aaaaguaacugcgguuuu...	1	0	j22
.........caaaaguaacugcgguuuu...	1	0	ku3
.........caaaaguaacugcgguuuuu..	5	0	ku3
..........aaaaguaacugcgguuuuu..	1	0	ku3
.........caaaaguaacugcgguuuuu..	2	0	j18
..........aaaaguaacugcgguuuu...	1	0	j16
..........aaaaguaacugcgguuuuu..	1	0	j16
.........caaaaguaacugcgguuuuu..	6	0	NL3
..........aaaaguaacugcgguuuu...	1	0	NL3
..........aaaaguaacugcgguuuuu..	18	0	NL3
....auuacuuuugcaccaaccuaa..	1	0	NL3
.........caaaaguaacugcgguuuuu..	2	0	ku6
..........aaaaguaacugcgguuuuu..	5	0	ku6
..........aaaaguaacugcgguuuuuu...	1	0	ku6
.........caaaaguaacugcgguuuuu..	5	0	ya2
..........aaaaguaacugcgguuuu...	4	0	ya2
..........aaaaguaacugcgguuuuu..	20	0	ya2
.........caaaaguaacugcgguuu..	1	0	ya3
.........caaaaguaacugcgguuuuu..	3	0	ya3
..........aaaaguaacugcgguuuu...	3	0	ya3
..........aaaaguaacugcgguuuuu..	23	0	ya3
....aaaccgugauuacuuuugca...........	1	0	ya3
....caaaaaccgugauuacuuuugc............	1	0	sh2
....uuacuuuugcaccaaccua...	1	0	sh2
......................................uuucaauagcaaaaaccgugauu....................	2	0	HEK

```
          Star                                    Mature
agauugguacaaaaguaacugcgguuuuuugccaguacuuucaauagcaaaaaccgugauuacuuuugcaccaaccuaaua
.......................................uucaauagcaaaaaccgugauu....................    2    0    HEK
...............................................caaaaaccgugauuacuuuugc............    10   0    HEK

..........................................................auuacuuuugcaccaaccuaau.    4    0    MCF

.........caaaaguaacugcgguuuu.....................................................    1    0    j15
..........aaaaguaacugcgguuuuu....................................................    5    0    j15

.........caaaaguaacugcgguuuu.....................................................    1    0    j01
..........aaaaguaacugcgguuuuu....................................................    5    0    j01

..........aaaaguaacugcgguuuuuu...................................................    1    0    j17

...............................................caaaaaccgugauuacuuuugc............    10   0    MBM
................................................aaaaaccgugauuacuuuug.............    2    0    MBM

...............................................caaaaaccgugauuacuuuugc............    1    0    j12

...............................................caaaaaccgugauuacuuuugc............    4    0    li1
................................................aaaaaccgugauuacuuuugca...........    1    0    li1

..........aaaaguaacugcgguuuuu....................................................    1    0    k13
...........................................................uuacuuuugcaccaaccuaaua    1    0    k13

...............................................caaaaaccgugauuacuuuugc............    3    0    A54

..........aaaaguaacugcgguuuuu....................................................    1    0    SW4
...............................................caaaaaccgugauuacuuuug.............    1    0    SW4

..........aaaaguaacugcgguuuuu....................................................    14   0    ya4
...................................................aaccgugauuacuuuugcacca........    1    0    ya4

.........caaaaguaacugcgguuuu.....................................................    1    0    ku1
..........aaaaguaacugcgguuuuu....................................................    1    0    ku1
```

Provisional ID	:	hsa_can_679
Score total	:	-2.8
Score for star read(s)	:	-1.3
Score for read counts	:	0
Score for mfe	:	-0.9
Score for randfold	:	
Score for cons. seed	:	-0.6
Total read count	:	4546
Mature read count	:	4546
Loop read count	:	0
Star read count	:	0

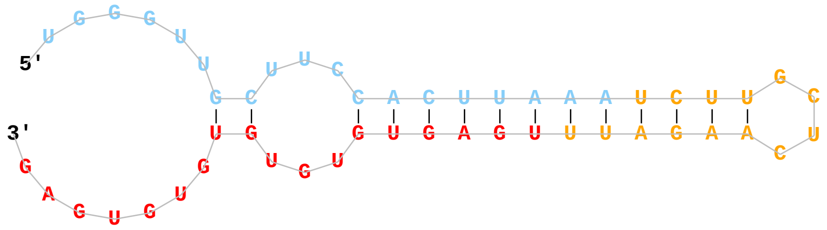

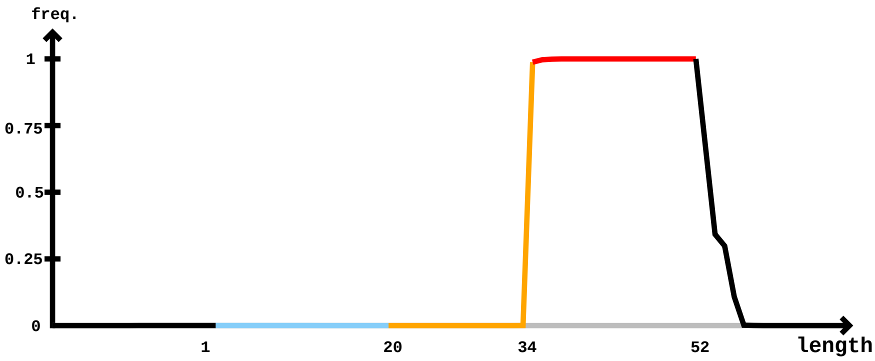

Star Mature

```
5'- ucaucaauggacacuuggguugcuuccacuuaaaucuugcucaagauuugagugugugugugugagugugcauguauguca -3'    exp
    .(((..(((.(((((((...(((...((((((((((((....))))))))))))...)))..))))))).)))..)))...   reads      mm         sample
    ...............................................uugagugugugugugugagu..............   1          0          ya1
    ................................................ugagugugugugugugag...............   195        0          ya1
    ................................................ugagugugugugugugagu..............   160        0          ya1
    ................................................ugagugugugugugugagug.............   13         0          ya1
    ................................................ugagugugugugugugagugu............   21         0          ya1
    ................................................ugagugugugugugugagugug...........   4          0          ya1
    .................................................gagugugugugugugagu..............   3          0          ya1
    .................................................gagugugugugugugagug.............   1          0          ya1
    .................................................gagugugugugugugagugug...........   1          0          ya1
    ..................................................agugugugugugugagug.............   1          0          ya1

    ................................................ugagugugugugugugag...............   1          0          s04
    ................................................ugagugugugugugugagu..............   1          0          s04
    ................................................ugagugugugugugugagugu............   1          0          s04
    ................................................ugagugugugugugugagugug...........   1          0          s04

    ................................................ugagugugugugugugagu..............   1          0          j26
    ................................................ugagugugugugugugagugu............   6          0          j26
    ................................................ugagugugugugugugagugug...........   2          0          j26

    ................................................ugagugugugugugugagug.............   2          0          va1
    ................................................ugagugugugugugugagugu............   21         0          va1
    ................................................ugagugugugugugugagugug...........   11         0          va1
    ................................................ugagugugugugugugagugugc..........   1          0          va1

    .......uggacacuuggguugcuuc.......................................................   1          0          k05
    ................................................ugagugugugugugugagugu............   2          0          k05

    ................................................ugagugugugugugugag...............   1          0          s06
    ................................................ugagugugugugugugagu..............   23         0          s06
    ................................................ugagugugugugugugagug.............   5          0          s06
    ................................................ugagugugugugugugagugu............   49         0          s06
    ................................................ugagugugugugugugagugug...........   24         0          s06
    ................................................ugagugugugugugugagugugc..........   1          0          s06
```

```
                 Star                           Mature
ucaucaauggacacuuggguugcuuccacuuaaaucuugcucaagauuugagugugugugugugagugugcauguauguca
..................................................agugugugugugugagugu............	1	0	s06

................................................ugagugugugugugugag...............	1	0	HeL
................................................ugagugugugugugugagu..............	12	0	HeL
................................................ugagugugugugugugagug.............	4	0	HeL
................................................ugagugugugugugugagugu............	43	0	HeL
................................................ugagugugugugugugagugug...........	23	0	HeL

................................................ugagugugugugugugag...............	12	0	ku4
................................................ugagugugugugugugagu..............	16	0	ku4
................................................ugagugugugugugugagug.............	7	0	ku4
................................................ugagugugugugugugagugu............	11	0	ku4
................................................ugagugugugugugugagugug...........	6	0	ku4

................................................ugagugugugugugugagugu............	1	0	k08

................................................ugagugugugugugugag...............	1	0	s10
................................................ugagugugugugugugagu..............	3	0	s10
................................................ugagugugugugugugagugu............	11	0	s10
................................................ugagugugugugugugagugug...........	2	0	s10

................................................ugagugugugugugugagu..............	2	0	ta3
................................................ugagugugugugugugagug.............	1	0	ta3
................................................ugagugugugugugugagugu............	5	0	ta3
................................................ugagugugugugugugagugug...........	1	0	ta3

................................................ugagugugugugugugagugu............	1	0	j02

................................................ugagugugugugugugagugu............	2	0	va2

................................................ugagugugugugugugagu..............	1	0	k14

................................................ugagugugugugugugag...............	3	0	j27
................................................ugagugugugugugugagu..............	4	0	j27
................................................ugagugugugugugugagug.............	3	0	j27
................................................ugagugugugugugugagugu............	19	0	j27
................................................ugagugugugugugugagugug...........	3	0	j27
.................................................gagugugugugugugagugu............	1	0	j27
..................................................agugugugugugugagug.............	2	0	j27

................................................ugagugugugugugugagu..............	5	0	H52
................................................ugagugugugugugugagug.............	2	0	H52
................................................ugagugugugugugugagugu............	9	0	H52
................................................ugagugugugugugugagugug...........	11	0	H52

................................................ugagugugugugugugagu..............	2	0	s03
................................................ugagugugugugugugagugu............	3	0	s03

................................................ugagugugugugugugagugu............	2	0	k09

................................................ugagugugugugugugagu..............	2	0	B14
................................................ugagugugugugugugagug.............	1	0	B14
................................................ugagugugugugugugagugu............	14	0	B14
................................................ugagugugugugugugagugug...........	11	0	B14

................................................ugagugugugugugugagugu............	3	0	j16
................................................ugagugugugugugugagugug...........	2	0	j16

................................................ugagugugugugugugagug.............	1	0	j18
................................................ugagugugugugugugagugu............	1	0	j18

................................................ugagugugugugugugagu..............	2	0	ku3
................................................ugagugugugugugugagugu............	1	0	ku3

................................................ugagugugugugugugagu..............	1	0	j09
................................................ugagugugugugugugagugu............	1	0	j09
................................................ugagugugugugugugagugug...........	3	0	j09

................................................ugagugugugugugugagug.............	2	0	j11
................................................ugagugugugugugugagugu............	4	0	j11
```

```
                  Star                            Mature
ucaucaauggacacuuggguugcuuccacuuaaaucuugcucaagauuugagugugugugugugagugugcauguauguca

................................................ugagugugugugugugag...............    3     0    s12
................................................ugagugugugugugugagu..............    2     0    s12
................................................ugagugugugugugugagug.............    2     0    s12
................................................ugagugugugugugugagugu............    10    0    s12
................................................ugagugugugugugugagugug...........    4     0    s12

................................................ugagugugugugugugag...............    360   0    ya3
................................................ugagugugugugugugagu..............    250   0    ya3
................................................ugagugugugugugugagug.............    29    0    ya3
................................................ugagugugugugugugagugu............    29    0    ya3
................................................ugagugugugugugugagugug...........    8     0    ya3
.................................................gagugugugugugugagu..............    4     0    ya3
.................................................gagugugugugugugagugu............    1     0    ya3
..................................................agugugugugugugagugug...........    2     0    ya3

................................................ugagugugugugugugagu..............    6     0    la1
................................................ugagugugugugugugagugu............    5     0    la1
................................................ugagugugugugugugagugug...........    4     0    la1

................................................ugagugugugugugugag...............    1     0    HEK
................................................ugagugugugugugugagugu............    2     0    HEK
................................................ugagugugugugugugagugug...........    3     0    HEK
.................................................gagugugugugugugagugu............    1     0    HEK

................................................ugagugugugugugugagug.............    3     0    j17

................................................ugagugugugugugugagu..............    2     0    j15
................................................ugagugugugugugugagug.............    1     0    j15
................................................ugagugugugugugugagugu............    9     0    j15
................................................ugagugugugugugugagugug...........    6     0    j15
.................................................gagugugugugugugagugug...........    1     0    j15

................................................ugagugugugugugugag...............    2     0    la2
................................................ugagugugugugugugagu..............    3     0    la2
................................................ugagugugugugugugagug.............    2     0    la2
................................................ugagugugugugugugagugu............    12    0    la2
................................................ugagugugugugugugagugug...........    5     0    la2

................................................ugagugugugugugugag...............    17    0    li2
................................................ugagugugugugugugagu..............    17    0    li2
................................................ugagugugugugugugagug.............    2     0    li2
................................................ugagugugugugugugagugu............    46    0    li2
................................................ugagugugugugugugagugug...........    140   0    li2
................................................ugagugugugugugugagugugc..........    1     0    li2
................................................ugagugugugugugugagugugca.........    1     0    li2

................................................ugagugugugugugugagugu............    2     0    va4
................................................ugagugugugugugugagugug...........    2     0    va4

................................................ugagugugugugugugagug.............    1     0    va3
................................................ugagugugugugugugagugu............    2     0    va3
................................................ugagugugugugugugagugug...........    2     0    va3

................................................ugagugugugugugugagu..............    1     0    SW4
................................................ugagugugugugugugagugu............    5     0    SW4
................................................ugagugugugugugugagugug...........    2     0    SW4

................................................ugagugugugugugugag...............    638   0    ya4
................................................ugagugugugugugugagu..............    347   0    ya4
................................................ugagugugugugugugagug.............    41    0    ya4
................................................ugagugugugugugugagugu............    21    0    ya4
................................................ugagugugugugugugagugug...........    8     0    ya4
.................................................gagugugugugugugagu..............    13    0    ya4
.................................................gagugugugugugugagug.............    3     0    ya4

................................................ugagugugugugugugagug.............    2     0    ku1
................................................ugagugugugugugugagugu............    1     0    ku1

................................................ugagugugugugugugagugu............    1     0    j23
```

```
                   Star                        Mature
ucaucaauggacacuuggguugcuuccacuuaaaucuugcucaagauuugagugugugugugugagugugcauguaugca
................................................ugagugugugugugugagugug..........   2   0   j23

................................................ugagugugugugugugag..............   4   0   s05
................................................ugagugugugugugugagu.............   9   0   s05
................................................ugagugugugugugugagug............   1   0   s05
................................................ugagugugugugugugagugu...........   6   0   s05
................................................ugagugugugugugugagugug..........   3   0   s05
.................................................gagugugugugugugagugug..........   1   0   s05

................................................ugagugugugugugugagug............   1   0   ta2
................................................ugagugugugugugugagugu...........   5   0   ta2

................................................ugagugugugugugugagug............   7   0   j24
................................................ugagugugugugugugagugu...........   14  0   j24
................................................ugagugugugugugugagugug..........   3   0   j24

................................................ugagugugugugugugagu.............   1   0   k11

................................................ugagugugugugugugagu.............   1   0   ta1
................................................ugagugugugugugugagug............   4   0   ta1
................................................ugagugugugugugugagugu...........   7   0   ta1
................................................ugagugugugugugugagugug..........   2   0   ta1

................................................ugagugugugugugugag..............   8   0   s09
................................................ugagugugugugugugagu.............   11  0   s09
................................................ugagugugugugugugagug............   2   0   s09
................................................ugagugugugugugugagugu...........   49  0   s09
................................................ugagugugugugugugagugug..........   11  0   s09

................................................ugagugugugugugugag..............   22  0   NL2
................................................ugagugugugugugugagu.............   26  0   NL2
................................................ugagugugugugugugagug............   2   0   NL2
................................................ugagugugugugugugagugu...........   4   0   NL2
..................................................agugugugugugugagugu...........   1   0   NL2

................................................ugagugugugugugugag..............   1   0   j19
................................................ugagugugugugugugagu.............   2   0   j19
................................................ugagugugugugugugagug............   1   0   j19
................................................ugagugugugugugugagugu...........   17  0   j19
................................................ugagugugugugugugagugug..........   10  0   j19

................................................ugagugugugugugugag..............   1   0   j03
................................................ugagugugugugugugagugu...........   6   0   j03
................................................ugagugugugugugugagugug..........   4   0   j03

................................................ugagugugugugugugagugu...........   3   0   j25
................................................ugagugugugugugugagugug..........   2   0   j25
.................................................gagugugugugugugagugug..........   1   0   j25

................................................ugagugugugugugugagugu...........   1   0   k04

................................................ugagugugugugugugag..............   5   0   per
................................................ugagugugugugugugagu.............   4   0   per
................................................ugagugugugugugugagug............   2   0   per
................................................ugagugugugugugugagugu...........   21  0   per
................................................ugagugugugugugugagugug..........   3   0   per

................................................ugagugugugugugugagu.............   2   0   s01
................................................ugagugugugugugugagugug..........   2   0   s01

................................................ugagugugugugugugag..............   5   0   U20
................................................ugagugugugugugugagu.............   22  0   U20
................................................ugagugugugugugugagug............   1   0   U20
................................................ugagugugugugugugagugu...........   83  0   U20
................................................ugagugugugugugugagugug..........   50  0   U20
................................................ugagugugugugugugagugugc.........   1   0   U20
................................................ugagugugugugugugagugugca........   1   0   U20
...................................................gugugugugugugagugu...........   3   0   U20

................................................ugagugugugugugugagu.............   1   0   ku2
```

```
                   Star                    Mature
ucaucaauggacacuuggguugcuuccacuuaaaucuugcucaagauuugagugugugugugugagugugcauguauguca
................................................ugagugugugugugugagugu............    1     0     ku2
................................................ugagugugugugugugagugug...........    1     0     ku2

................................................ugagugugugugugugagu..............    2     0     s11
................................................ugagugugugugugugagugu............    5     0     s11
................................................ugagugugugugugugagugug...........    2     0     s11

................................................ugagugugugugugugag...............    1     0     j07
................................................ugagugugugugugugagu..............    4     0     j07
................................................ugagugugugugugugagug.............    1     0     j07
................................................ugagugugugugugugagugu............    8     0     j07

................................................ugagugugugugugugagugu............    1     0     k02
................................................ugagugugugugugugagugug...........    1     0     k02

................................................ugagugugugugugugag...............    15    0     j28
................................................ugagugugugugugugagu..............    45    0     j28
................................................ugagugugugugugugagug.............    10    0     j28
................................................ugagugugugugugugagugu............    75    0     j28
................................................ugagugugugugugugagugug...........    20    0     j28
.................................................gagugugugugugugagu..............    1     0     j28
.................................................gagugugugugugugagug.............    2     0     j28
...................................................gugugugugugugagugug...........    1     0     j28

................................................ugagugugugugugugag...............    1     0     s08
................................................ugagugugugugugugagu..............    5     0     s08
................................................ugagugugugugugugagug.............    2     0     s08
................................................ugagugugugugugugagugu............    19    0     s08
................................................ugagugugugugugugagugug...........    5     0     s08

................................................ugagugugugugugugagu..............    3     0     DLD
................................................ugagugugugugugugagugu............    20    0     DLD
................................................ugagugugugugugugagugug...........    4     0     DLD
..................................................agugugugugugugagugu............    1     0     DLD

................................................ugagugugugugugugag...............    265   0     ya2
................................................ugagugugugugugugagu..............    177   0     ya2
................................................ugagugugugugugugagug.............    15    0     ya2
................................................ugagugugugugugugagugu............    16    0     ya2
................................................ugagugugugugugugagugug...........    3     0     ya2
.................................................gagugugugugugugagug.............    1     0     ya2
..................................................agugugugugugugagug.............    2     0     ya2

................................................ugagugugugugugugag...............    47    0     NL3
................................................ugagugugugugugugagu..............    58    0     NL3
................................................ugagugugugugugugagugu............    6     0     NL3
.................................................gagugugugugugugagu..............    2     0     NL3

................................................ugagugugugugugugagugu............    1     0     s02

................................................ugagugugugugugugagugu............    1     0     k06
................................................ugagugugugugugugagugug...........    1     0     k06

................................................ugagugugugugugugagugu............    3     0     j29

................................................ugagugugugugugugagug.............    1     0     sh2
................................................ugagugugugugugugagugu............    15    0     sh2
................................................ugagugugugugugugagugug...........    4     0     sh2
.................................................gagugugugugugugagugu............    1     0     sh2

................................................ugagugugugugugugagugu............    7     0     MCF
................................................ugagugugugugugugagugug...........    2     0     MCF

................................................ugagugugugugugugag...............    1     0     MBM
................................................ugagugugugugugugagu..............    4     0     MBM
................................................ugagugugugugugugagug.............    4     0     MBM
................................................ugagugugugugugugagugu............    29    0     MBM
................................................ugagugugugugugugagugug...........    10    0     MBM
.................................................gagugugugugugugagugu............    2     0     MBM

................................................ugagugugugugugugag...............    40    0     NL1
```

```
               Star                               Mature
ucaucaauggacacuuggguugcuuccacuuaaaucuugcucaagauuugagugugugugugugagugugcauguauguca
................................................ugagugugugugugugagu..............  74   0   NL1
................................................ugagugugugugugugagug.............  3    0   NL1
................................................ugagugugugugugugagugu............  20   0   NL1
................................................ugagugugugugugugagugug...........  2    0   NL1
.................................................gagugugugugugugagugu............  1    0   NL1

................................................ugagugugugugugugagugu............  3    0   k07

................................................ugagugugugugugugagugu............  1    0   sh1

................................................ugagugugugugugugag...............  1    0   li1
................................................ugagugugugugugugagu..............  1    0   li1
................................................ugagugugugugugugagugu............  2    0   li1
................................................ugagugugugugugugagugug...........  8    0   li1

................................................ugagugugugugugugagugug...........  1    0   k13

................................................ugagugugugugugugagug.............  1    0   j20
................................................ugagugugugugugugagugu............  3    0   j20
................................................ugagugugugugugugagugug...........  3    0   j20

................................................ugagugugugugugugag...............  1    0   A54
................................................ugagugugugugugugagug.............  3    0   A54
................................................ugagugugugugugugagugu............  26   0   A54
................................................ugagugugugugugugagugug...........  30   0   A54

................................................ugagugugugugugugagugu............  1    0   k12

................................................ugagugugugugugugagug.............  1    0   j30
```

Provisional ID	:	hsa_can_682
Score total	:	1810.2
Score for star read(s)	:	3.9
Score for read counts	:	1801.3
Score for mfe	:	1.9
Score for randfold	:	
Score for cons. seed	:	3
Total read count	:	3545
Mature read count	:	3542
Loop read count	:	0
Star read count	:	3

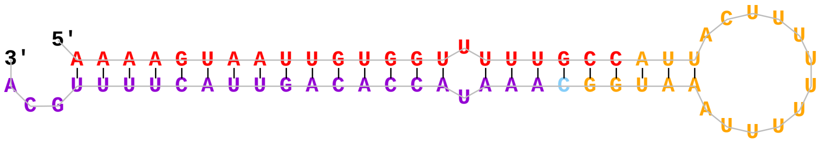

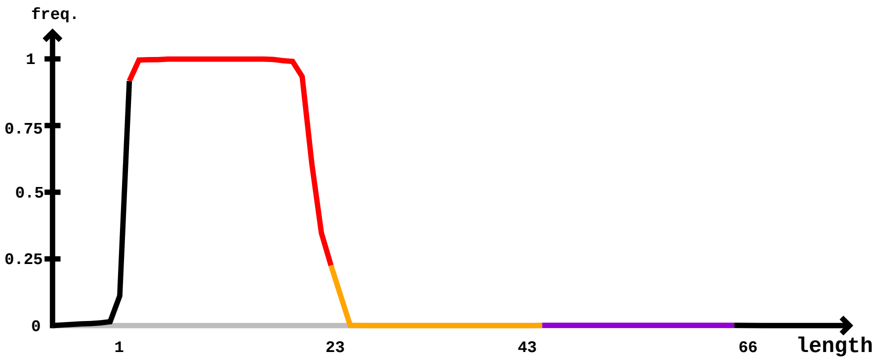

Mature Star

```
5'- uggugcaaaaguaauugugguuuuugccauuacuuuuuuuuuaaauggcaaauaccacaguuacuuuugcacuaauguaau  -3'  obs
    uggugcaaaaguaauugugguuuuugccauuacuuuuuuuuuaaauggcaaauaccacaguuacuuuugcacuaauguaau        exp
    uggugcaaaaguaauugugguuuuugccauuacuuuuuuuuuaaauggcaaauaccacaguuacuuuugcacuaauguaau        known
    ((((((((((((((((((((.(((((((((...........))))))))).))))))))))))))))))))).......   reads    mm    sample
    .....caaaaguaauugugguuuu.......................................................   5        0     ya1
    .....caaaaguaauugugguuuuu......................................................   22       0     ya1
    .....caaaaguaauugugguuuuug.....................................................   1        0     ya1
    ......aaaaguaauugugguuuu.......................................................   16       0     ya1
    ......aaaaguaauugugguuuuu......................................................   111      0     ya1
    ......aaaaguaauugugguuuuug.....................................................   8        0     ya1
    .......aaaguaauugugguuuuu......................................................   19       0     ya1

    ......aaaaguaauugugguuuuug.....................................................   1        0     s04

    ......aaaaguaauugugguuuuug.....................................................   1        0     j26

    uggugcaaaaguaauugugguu.........................................................   1        0     va1
    .....caaaaguaauugugguuuuugc....................................................   1        0     va1
    ......aaaaguaauugugguuuuu......................................................   2        0     va1
    ......aaaaguaauugugguuuuug.....................................................   25       0     va1
    ......aaaaguaauugugguuuuugc....................................................   55       0     va1
    ......aaaaguaauugugguuuuugcc...................................................   35       0     va1
    ......aaaaguaauugugguuuuugcca..................................................   3        0     va1
    .......aaaguaauugugguuuuug.....................................................   2        0     va1
    .......aaaguaauugugguuuuugc....................................................   1        0     va1
    .......aaaguaauugugguuuuugcc...................................................   1        0     va1

    uggugcaaaaguaauuguggu..........................................................   1        0     ku5
    ..gugcaaaaguaauugugguu.........................................................   1        0     ku5
    .....caaaaguaauugugguuuuug.....................................................   1        0     ku5
    ......aaaaguaauugugguuuuug.....................................................   3        0     ku5

    .....caaaaguaauugugguuuuu......................................................   2        0     k05
    .....caaaaguaauugugguuuuug.....................................................   2        0     k05
    .....caaaaguaauugugguuuuugc....................................................   1        0     k05
    ......aaaaguaauugugguuuuug.....................................................   3        0     k05
```

```
      Mature                                Star
uggugcaaaaguaauugugguuuuugccauuacuuuuuuuuuaaauggcaaauaccacaguuacuuuugcacuaauguaau
......aaaaguaauugugguuuuugc......................................................   1    0    k05
.......aaaguaauugugguuuuug.......................................................   1    0    k05

.....caaaaguaauugugguuuuug.......................................................   1    0    s06
......aaaaguaauugugguuuuugc......................................................   1    0    s06
......aaaaguaauugugguuuuugcc.....................................................   2    0    s06

......aaaaguaauugugguuuuugc......................................................   5    0    HeL
......aaaaguaauugugguuuuugcc.....................................................   49   0    HeL
......aaaaguaauugugguuuuugcca....................................................   6    0    HeL
.......aaaguaauugugguuuuugc......................................................   1    0    HeL
.......aaaguaauugugguuuuugcc.....................................................   1    0    HeL
........aaguaauugugguuuuugccauu..................................................   1    0    HeL
..........guaauugugguuuuugcc.....................................................   5    0    HeL

..gugcaaaaguaauuguggu............................................................   1    0    ku4
......aaaaguaauugugguuuu.........................................................   5    0    ku4
......aaaaguaauugugguuuuug.......................................................   11   0    ku4

......aaaaguaauugugguuuuugcc.....................................................   1    0    s07

......aaaaguaauugugguuuuug.......................................................   2    0    s10
......aaaaguaauugugguuuuugc......................................................   1    0    s10

.....caaaaguaauugugguuuu.........................................................   2    0    k08
.....caaaaguaauugugguuuuug.......................................................   1    0    k08
......aaaaguaauugugguuuu.........................................................   1    0    k08
......aaaaguaauugugguuuuug.......................................................   1    0    k08
.......aaaguaauugugguuuuugcc.....................................................   1    0    k08

......aaaaguaauugugguuu..........................................................   1    0    ta3
......aaaaguaauugugguuuuug.......................................................   7    0    ta3
......aaaaguaauugugguuuuugc......................................................   1    0    ta3
.......aaaguaauugugguuuuug.......................................................   1    0    ta3

.....caaaaguaauugugguuuuugc......................................................   1    0    j10
......aaaaguaauugugguuuu.........................................................   2    0    j10
......aaaaguaauugugguuuuug.......................................................   6    0    j10
......aaaaguaauugugguuuuugc......................................................   5    0    j10

......aaaaguaauugugguuuuug.......................................................   3    0    j04

......aaaaguaauugugguuu..........................................................   1    0    j02
......aaaaguaauugugguuuu.........................................................   5    0    j02
......aaaaguaauugugguuuuug.......................................................   22   0    j02
.......aaaguaauugugguuuuug.......................................................   1    0    j02

.....caaaaguaauugugguuuu.........................................................   2    0    k14
.....caaaaguaauugugguuuuug.......................................................   4    0    k14
......aaaaguaauugugguuuu.........................................................   2    0    k14
......aaaaguaauugugguuuuug.......................................................   4    0    k14
.......aaaguaauugugguuuuug.......................................................   1    0    k14

.....caaaaguaauugugguuuu.........................................................   1    0    va2
.....caaaaguaauugugguuuuugc......................................................   1    0    va2
......aaaaguaauugugguuuu.........................................................   1    0    va2
......aaaaguaauugugguuuuug.......................................................   4    0    va2
......aaaaguaauugugguuuuugc......................................................   4    0    va2
.......aaaguaauugugguuuuugc......................................................   1    0    va2

uggugcaaaaguaauuguggu............................................................   1    0    j31
.....caaaaguaauugugguuuu.........................................................   1    0    j31
.....caaaaguaauugugguuuuug.......................................................   2    0    j31
......aaaaguaauugugguuuuug.......................................................   1    0    j31
......aaaaguaauugugguuuuugcc.....................................................   1    0    j31

.ggugcaaaaguaauugugguu...........................................................   1    0    j27
.....caaaaguaauugugguuuu.........................................................   1    0    j27
.....caaaaguaauugugguuuuug.......................................................   4    0    j27
......aaaaguaauugugguuu..........................................................   1    0    j27
```

```
      Mature                                     Star
uggugcaaaaguaauugugguuuuugccauuacuuuuuuuuuaaauggcaaauaccacaguuacuuuugcacuaauguaau
......aaaaguaauugugguuuuu........................................................	2	0	j27
......aaaaguaauugugguuuuug.......................................................	13	0	j27
.......aaaguaauugugguuuuugc......................................................	1	0	j27

......aaaaguaauugugguuuuugc......................................................	2	0	s03

.....caaaaguaauugugguuuuugc......................................................	1	0	H52
......aaaaguaauugugguuuuu........................................................	2	0	H52
......aaaaguaauugugguuuuugc......................................................	4	0	H52
......aaaaguaauugugguuuuugcc.....................................................	16	0	H52
.......aaaguaauugugguuuuugcca....................................................	1	0	H52

.....caaaaguaauugugguuuuu........................................................	1	0	j22
.....caaaaguaauugugguuuuug.......................................................	1	0	j22
.....caaaaguaauugugguuuuugc......................................................	2	0	j22
......aaaaguaauugugguuuuu........................................................	1	0	j22
......aaaaguaauugugguuuuug.......................................................	33	0	j22
......aaaaguaauugugguuuuugc......................................................	1	0	j22
......aaaaguaauugugguuuuugcc.....................................................	2	0	j22
.......aaaguaauugugguuuuug.......................................................	3	0	j22

...ugcaaaaguaauugugguuuuu........................................................	1	0	k09
.....caaaaguaauugugguuuuu........................................................	1	0	k09
.....caaaaguaauugugguuuuug.......................................................	2	0	k09
......aaaaguaauugugguuuuu........................................................	2	0	k09
......aaaaguaauugugguuuuug.......................................................	4	0	k09
......aaaaguaauugugguuuuugc......................................................	1	0	k09
......aaaaguaauugugguuuuugcc.....................................................	1	0	k09

.....caaaaguaauugugguuuuug.......................................................	3	0	B14
.....caaaaguaauugugguuuuugc......................................................	1	0	B14
......aaaaguaauugugguuuuugc......................................................	6	0	B14
......aaaaguaauugugguuuuugcc.....................................................	15	0	B14
.......aaaguaauugugguuuuugcc.....................................................	1	0	B14
.................................................aaauaccacaguuacuuuugca..........	1	0	B14

.....caaaaguaauugugguuuuug.......................................................	1	0	j16
......aaaaguaauugugguuuu.........................................................	1	0	j16
......aaaaguaauugugguuuuu........................................................	12	0	j16
......aaaaguaauugugguuuuug.......................................................	25	0	j16
.......aaaguaauugugguuuuugc......................................................	1	0	j16

.....caaaaguaauugugguuuuu........................................................	2	0	j09
......aaaaguaauugugguuuuu........................................................	4	0	j09
......aaaaguaauugugguuuuug.......................................................	9	0	j09
.......aaaguaauugugguuuuug.......................................................	1	0	j09

.....caaaaguaauugugguuuuu........................................................	3	0	j18
.....caaaaguaauugugguuuuug.......................................................	1	0	j18
......aaaaguaauugugguuuuug.......................................................	1	0	j18

uggugcaaaaguaauuguggu............................................................	1	0	ku3
.....caaaaguaauugugguuuuug.......................................................	1	0	ku3
......aaaaguaauugugguuuuug.......................................................	6	0	ku3

.....caaaaguaauugugguuuu.........................................................	1	0	ku6
.....caaaaguaauugugguuuuu........................................................	1	0	ku6
......aaaaguaauugugguuuuug.......................................................	2	0	ku6

......aaaaguaauugugguuuuu........................................................	4	0	j11
......aaaaguaauugugguuuuug.......................................................	7	0	j11
.......aaaguaauugugguuuuug.......................................................	1	0	j11

......aaaaguaauugugguuuuug.......................................................	4	0	s12
.......aaaguaauugugguuuuug.......................................................	2	0	s12

....gcaaaaguaauugugguuuuu........................................................	2	0	ya3
.....caaaaguaauugugguuuu.........................................................	9	0	ya3
.....caaaaguaauugugguuuuu........................................................	22	0	ya3
......aaaaguaauugugguuuu.........................................................	44	0	ya3
```

```
    Mature                                   Star
uggugcaaaaguaauugugguuuuugccauuacuuuuuuuuuaaauggcaaauaccacaguuacuuuugcacuaauguaau
......aaaaguaauugugguuuuu........................................................    238    0    ya3
......aaaaguaauugugguuuuug.......................................................    27     0    ya3
......aaaaguaauugugguuuuugc......................................................    1      0    ya3
.......aaaguaauugugguuuuu........................................................    45     0    ya3
.......aaaguaauugugguuuuug.......................................................    8      0    ya3

.....caaaaguaauugugguuuuu........................................................    1      0    la1
.....caaaaguaauugugguuuuugc......................................................    1      0    la1
......aaaaguaauugugguuuu.........................................................    1      0    la1
......aaaaguaauugugguuuuu........................................................    1      0    la1
......aaaaguaauugugguuuuug.......................................................    3      0    la1
......aaaaguaauugugguuuuugcc.....................................................    1      0    la1

uggugcaaaaguaauuguggu............................................................    1      0    HEK
.....caaaaguaauugugguuuuugc......................................................    7      0    HEK
......aaaaguaauugugguuuuugc......................................................    9      0    HEK
......aaaaguaauugugguuuuugcc.....................................................    75     0    HEK
.......aaaguaauugugguuuuugcc.....................................................    2      0    HEK
.......aaaguaauugugguuuuugcca....................................................    1      0    HEK
........aaguaauugugguuuuugcc.....................................................    1      0    HEK
........aaguaauugugguuuuugccau...................................................    1      0    HEK

......aaaaguaauugugguuuu.........................................................    1      0    j17
......aaaaguaauugugguuuuu........................................................    8      0    j17
......aaaaguaauugugguuuuug.......................................................    8      0    j17

.....caaaaguaauugugguuuuug.......................................................    4      0    j15
.....caaaaguaauugugguuuuugc......................................................    1      0    j15
......aaaaguaauugugguuuu.........................................................    1      0    j15
......aaaaguaauugugguuuuu........................................................    3      0    j15
......aaaaguaauugugguuuuug.......................................................    16     0    j15
.......aaaguaauugugguuuuu........................................................    1      0    j15
.......aaaguaauugugguuuuug.......................................................    1      0    j15

......aaaaguaauugugguuuuu........................................................    1      0    la2
......aaaaguaauugugguuuuug.......................................................    3      0    la2
......aaaaguaauugugguuuuugcc.....................................................    6      0    la2
.......aaaguaauugugguuuuugcc.....................................................    1      0    la2

uggugcaaaaguaauuguggu............................................................    1      0    j12
.....caaaaguaauugugguuuuug.......................................................    5      0    j12
......aaaaguaauugugguuuuu........................................................    6      0    j12
......aaaaguaauugugguuuuug.......................................................    24     0    j12
......aaaaguaauugugguuuuugc......................................................    2      0    j12
......aaaaguaauugugguuuuugcc.....................................................    1      0    j12
.......aaaguaauugugguuuuug.......................................................    2      0    j12

uggugcaaaaguaauuguggu............................................................    1      0    li2
.....caaaaguaauugugguuuuug.......................................................    4      0    li2
.....caaaaguaauugugguuuuugc......................................................    9      0    li2
......aaaaguaauugugguuuuugc......................................................    56     0    li2
......aaaaguaauugugguuuuugcc.....................................................    3      0    li2
.......aaaguaauugugguuuuugc......................................................    3      0    li2

.....caaaaguaauugugguuuuug.......................................................    1      0    va4
.....caaaaguaauugugguuuuugc......................................................    1      0    va4
......aaaaguaauugugguuuuu........................................................    1      0    va4
......aaaaguaauugugguuuuug.......................................................    5      0    va4
......aaaaguaauugugguuuuugc......................................................    11     0    va4
......aaaaguaauugugguuuuugcc.....................................................    11     0    va4

.....caaaaguaauugugguuuuug.......................................................    2      0    va3
.....caaaaguaauugugguuuuugc......................................................    1      0    va3
......aaaaguaauugugguuuuu........................................................    2      0    va3
......aaaaguaauugugguuuuug.......................................................    10     0    va3
......aaaaguaauugugguuuuugc......................................................    13     0    va3
......aaaaguaauugugguuuuugcc.....................................................    3      0    va3
.......aaaguaauugugguuuuug.......................................................    2      0    va3
.......aaaguaauugugguuuuugcc.....................................................    1      0    va3

.....caaaaguaauugugguuuuu........................................................    1      0    SW4
```

```
    Mature                                  Star
uggugcaaaaguaauugugguuuuugccauuacuuuuuuuuuaaauggcaaauaccacaguuacuuuugcacuaauguaau
.....caaaaguaauugugguuuuugc......................................................    1     0     SW4
......aaaaguaauugugguuuuu........................................................    1     0     SW4
......aaaaguaauugugguuuuug.......................................................    3     0     SW4
......aaaaguaauugugguuuuugc......................................................    12    0     SW4
......aaaaguaauugugguuuuugcc.....................................................    20    0     SW4
.......aaaguaauugugguuuuugc......................................................    1     0     SW4
.......aaaguaauugugguuuuugcca....................................................    1     0     SW4

.....caaaaguaauugugguuuu.........................................................    16    0     ya4
.....caaaaguaauugugguuuuu........................................................    16    0     ya4
.....caaaaguaauugugguuuuug.......................................................    4     0     ya4
......aaaaguaauugugguuuu.........................................................    33    0     ya4
......aaaaguaauugugguuuuu........................................................    213   0     ya4
......aaaaguaauugugguuuuug.......................................................    5     0     ya4
......aaaaguaauugugguuuuugcc.....................................................    4     0     ya4
.......aaaguaauugugguuuuu........................................................    43    0     ya4
.......aaaguaauugugguuuuug.......................................................    3     0     ya4

.....caaaaguaauugugguuuuu........................................................    1     0     ku1
.....caaaaguaauugugguuuuug.......................................................    1     0     ku1
.....caaaaguaauugugguuuuugc......................................................    1     0     ku1
......aaaaguaauugugguuuuu........................................................    5     0     ku1
......aaaaguaauugugguuuuug.......................................................    38    0     ku1
......aaaaguaauugugguuuuugc......................................................    1     0     ku1
.......aaaguaauugugguuuuug.......................................................    1     0     ku1

...ugcaaaaguaauugugguuuuu........................................................    1     0     j23
.....caaaaguaauugugguuuuug.......................................................    4     0     j23
......aaaaguaauugugguuuu.........................................................    1     0     j23
......aaaaguaauugugguuuuu........................................................    8     0     j23
......aaaaguaauugugguuuuug.......................................................    89    0     j23
......aaaaguaauugugguuuuugc......................................................    5     0     j23
......aaaaguaauugugguuuuugcc.....................................................    13    0     j23
.......aaaguaauugugguuuuug.......................................................    11    0     j23
.......aaaguaauugugguuuuugc......................................................    1     0     j23
.......aaaguaauugugguuuuugcc.....................................................    2     0     j23

......aaaaguaauugugguuuuug.......................................................    7     0     s05
......aaaaguaauugugguuuuugc......................................................    1     0     s05
.......aaaguaauugugguuuuug.......................................................    1     0     s05

......aaaaguaauugugguuuuugc......................................................    4     0     ta2
.......aaaguaauugugguuuuug.......................................................    1     0     ta2

.ggugcaaaaguaauugugguuu..........................................................    5     0     j24
.....caaaaguaauugugguuuu.........................................................    3     0     j24
.....caaaaguaauugugguuuuug.......................................................    1     0     j24
......aaaaguaauugugguuuuu........................................................    3     0     j24
......aaaaguaauugugguuuuug.......................................................    8     0     j24

.....caaaaguaauugugguuuuug.......................................................    1     0     k11
......aaaaguaauugugguuuuu........................................................    2     0     k11
......aaaaguaauugugguuuuug.......................................................    2     0     k11
......aaaaguaauugugguuuuugc......................................................    3     0     k11

......aaaaguaauugugguuuu.........................................................    1     0     ta1
......aaaaguaauugugguuuuu........................................................    1     0     ta1
......aaaaguaauugugguuuuug.......................................................    3     0     ta1
......aaaaguaauugugguuuuugcc.....................................................    1     0     ta1
.......aaaguaauugugguuuuug.......................................................    1     0     ta1

.....caaaaguaauugugguuuuug.......................................................    1     0     s09
......aaaaguaauugugguuuuu........................................................    1     0     s09
......aaaaguaauugugguuuuug.......................................................    3     0     s09
.......aaaguaauugugguuuuug.......................................................    1     0     s09

.....caaaaguaauugugguuu..........................................................    1     0     NL2
.....caaaaguaauugugguuuu.........................................................    1     0     NL2
.....caaaaguaauugugguuuuu........................................................    4     0     NL2
......aaaaguaauugugguuuu.........................................................    10    0     NL2
```

Mature Star

```
uggugcaaaaguaauugugguuuuugccauuacuuuuuuuuuaaauggcaaauaccacaguuacuuuugcacuaauguaau
......aaaaguaauugugguuuuu........................................................   2    0    NL2
......aaaaguaauugugguuuuug.......................................................   2    0    NL2

uggugcaaaaguaauugugguu...........................................................   1    0    k10
.....caaaaguaauugugguuuuu........................................................   1    0    k10
.....caaaaguaauugugguuuuug.......................................................   2    0    k10
......aaaaguaauugugguuuuug.......................................................   4    0    k10
......aaaaguaauugugguuuuugc......................................................   2    0    k10

.....caaaaguaauugugguuuuug.......................................................   1    0    j13
.....caaaaguaauugugguuuuugc......................................................   1    0    j13
......aaaaguaauugugguuuu.........................................................   1    0    j13
......aaaaguaauugugguuuuu........................................................   6    0    j13
......aaaaguaauugugguuuuug.......................................................   33   0    j13
......aaaaguaauugugguuuuugc......................................................   4    0    j13
......aaaaguaauugugguuuuugcc.....................................................   1    0    j13
.......aaaguaauugugguuuuug.......................................................   1    0    j13
.......aaaguaauugugguuuuugc......................................................   1    0    j13
.................................................aaauaccacaguuacuuuugca..........   1    0    j13

.....caaaaguaauugugguuuuu........................................................   2    0    j19
.....caaaaguaauugugguuuuug.......................................................   1    0    j19
......aaaaguaauugugguuuu.........................................................   2    0    j19
......aaaaguaauugugguuuuu........................................................   5    0    j19
......aaaaguaauugugguuuuug.......................................................   37   0    j19
......aaaaguaauugugguuuuugc......................................................   2    0    j19

......aaaaguaauugugguuuuug.......................................................   4    0    j03

......aaaaguaauugugguuuuug.......................................................   1    0    k01
......aaaaguaauugugguuuuugc......................................................   3    0    k01

uggugcaaaaguaauugugguu...........................................................   1    0    j25
...ugcaaaaguaauugugguu...........................................................   2    0    j25
.....caaaaguaauugugguuuu.........................................................   1    0    j25
......aaaaguaauugugguuuuug.......................................................   10   0    j25

....gcaaaaguaauugugguuuuug.......................................................   1    0    s01

....gcaaaaguaauugugguuuuug.......................................................   1    0    per
.....caaaaguaauugugguuuuug.......................................................   1    0    per
......aaaaguaauugugguuuu.........................................................   1    0    per
......aaaaguaauugugguuuuug.......................................................   8    0    per
.......aaaguaauugugguuuuug.......................................................   1    0    per

.....caaaaguaauugugguuuuu........................................................   2    0    k04
.....caaaaguaauugugguuuuug.......................................................   1    0    k04
......aaaaguaauugugguuuu.........................................................   1    0    k04
......aaaaguaauugugguuuuug.......................................................   5    0    k04
......aaaaguaauugugguuuuugc......................................................   1    0    k04

uggugcaaaaguaauuguggu............................................................   2    0    U20
uggugcaaaaguaauugugguu...........................................................   1    0    U20
..gugcaaaaguaauugugguuuu.........................................................   1    0    U20
.....caaaaguaauugugguuuuug.......................................................   1    0    U20
.....caaaaguaauugugguuuuugc......................................................   18   0    U20
.....caaaaguaauugugguuuuugcc.....................................................   1    0    U20
......aaaaguaauugugguuuuu........................................................   1    0    U20
......aaaaguaauugugguuuuug.......................................................   6    0    U20
......aaaaguaauugugguuuuugc......................................................   48   0    U20
......aaaaguaauugugguuuuugcc.....................................................   243  0    U20
......aaaaguaauugugguuuuugcca....................................................   3    0    U20
.......aaaguaauugugguuuuugc......................................................   11   0    U20
.......aaaguaauugugguuuuugcc.....................................................   24   0    U20
.......aaaguaauugugguuuuugcca....................................................   1    0    U20
.........aguaauugugguuuuugc......................................................   1    0    U20

.....caaaaguaauugugguuuuu........................................................   2    0    ku2
......aaaaguaauugugguuuuu........................................................   5    0    ku2
......aaaaguaauugugguuuuug.......................................................   16   0    ku2
```

Mature **Star**

```
uggugcaaaaguaauugugguuuuugccauuacuuuuuuuuuaaauggcaaauaccacaguuacuuuugcacuaauguaau
......aaaaguaauugugguuuuugc......................................................   1     0    ku2
......aaaaguaauugugguuuuugcc.....................................................   1     0    ku2

.....caaaaguaauugugguuuuu........................................................   1     0    j21
.....caaaaguaauugugguuuuug.......................................................   3     0    j21
......aaaaguaauugugguuuuu........................................................   1     0    j21
......aaaaguaauugugguuuuug.......................................................   14    0    j21
......aaaaguaauugugguuuuugc......................................................   3     0    j21
......aaaaguaauugugguuuuugcc.....................................................   2     0    j21
.......aaaguaauugugguuuuug.......................................................   3     0    j21
.......aaaguaauugugguuuuugcc.....................................................   2     0    j21


......aaaaguaauugugguuuuug.......................................................   1     0    s11

......aaaaguaauugugguuuuu........................................................   4     0    j07
......aaaaguaauugugguuuuug.......................................................   2     0    j07
.......aaaguaauugugguuuuug.......................................................   1     0    j07

.....caaaaguaauugugguuuuug.......................................................   5     0    j14
......aaaaguaauugugguuuuug.......................................................   9     0    j14
......aaaaguaauugugguuuuugc......................................................   5     0    j14
......aaaaguaauugugguuuuugcc.....................................................   1     0    j14

.....caaaaguaauugugguuuuug.......................................................   2     0    k02
......aaaaguaauugugguuuuu........................................................   2     0    k02
......aaaaguaauugugguuuuugc......................................................   1     0    k02

.....caaaaguaauugugguuuuu........................................................   1     0    j28
......aaaaguaauugugguuuuu........................................................   3     0    j28
......aaaaguaauugugguuuuug.......................................................   17    0    j28
.................................................aaauaccacaguuacuuuugca..........   1     0    j28

......aaaaguaauugugguuuuug.......................................................   1     0    s08

.....caaaaguaauugugguuuuugc......................................................   1     0    DLD
......aaaaguaauugugguuuuu........................................................   2     0    DLD
......aaaaguaauugugguuuuug.......................................................   1     0    DLD
......aaaaguaauugugguuuuugc......................................................   13    0    DLD
......aaaaguaauugugguuuuugcc.....................................................   73    0    DLD
......aaaaguaauugugguuuuugcca....................................................   1     0    DLD
.......aaaguaauugugguuuuugcc.....................................................   4     0    DLD
..........guaauugugguuuuugcc.....................................................   1     0    DLD

......aaaaguaauugugguuuuug.......................................................   5     0    k03
......aaaaguaauugugguuuuugc......................................................   3     0    k03

.....caaaaguaauugugguuu..........................................................   1     0    NL3
.....caaaaguaauugugguuuu.........................................................   1     0    NL3
.....caaaaguaauugugguuuuu........................................................   4     0    NL3
.....caaaaguaauugugguuuuug.......................................................   2     0    NL3
......aaaaguaauugugguuuu.........................................................   15    0    NL3
......aaaaguaauugugguuuuu........................................................   6     0    NL3
......aaaaguaauugugguuuuug.......................................................   5     0    NL3

.....caaaaguaauugugguuu..........................................................   1     0    ya2
.....caaaaguaauugugguuuu.........................................................   6     0    ya2
.....caaaaguaauugugguuuuu........................................................   13    0    ya2
.....caaaaguaauugugguuuuug.......................................................   2     0    ya2
......aaaaguaauugugguuuu.........................................................   24    0    ya2
......aaaaguaauugugguuuuu........................................................   188   0    ya2
......aaaaguaauugugguuuuug.......................................................   17    0    ya2
......aaaaguaauugugguuuuugcc.....................................................   1     0    ya2
......aaaaguaauugugguuuuugcca....................................................   1     0    ya2
.......aaaguaauugugguuuuu........................................................   43    0    ya2
.......aaaguaauugugguuuuug.......................................................   4     0    ya2

.....caaaaguaauugugguuuuug.......................................................   1     0    s02
......aaaaguaauugugguuuuugcc.....................................................   1     0    s02

.....caaaaguaauugugguuuuug.......................................................   3     0    k06
```

```
      Mature                                      Star
uggugcaaaaguaauugugguuuuugccauuacuuuuuuuuuaaauggcaaauaccacaguuacuuuugcacuaauguaau
......aaaaguaauugugguuuuug.......................................................    2     0     k06

....gcaaaaguaauugugguuuuug.......................................................    1     0     j29
.....caaaaguaauugugguuuuug.......................................................    3     0     j29
......aaaaguaauugugguuuuug.......................................................    10    0     j29
......aaaaguaauugugguuuuugc......................................................    8     0     j29
......aaaaguaauugugguuuuugcc.....................................................    1     0     j29

..gugcaaaaguaauuguggu............................................................    1     0     sh2
.....caaaaguaauugugguuuuu........................................................    1     0     sh2
.....caaaaguaauugugguuuuugc......................................................    1     0     sh2
......aaaaguaauugugguuuuug.......................................................    1     0     sh2
......aaaaguaauugugguuuuugc......................................................    3     0     sh2
......aaaaguaauugugguuuuugcc.....................................................    3     0     sh2
......aaaaguaauugugguuuuugcca....................................................    2     0     sh2

.ggugcaaaaguaauugugguu...........................................................    1     0     j01
...ugcaaaaguaauugugguuuu.........................................................    3     0     j01
.....caaaaguaauugugguuuuug.......................................................    1     0     j01
......aaaaguaauugugguuuuug.......................................................    5     0     j01

uggugcaaaaguaauuguggu............................................................    1     0     MCF
.....caaaaguaauugugguuuuugc......................................................    1     0     MCF
......aaaaguaauugugguuuuug.......................................................    4     0     MCF
......aaaaguaauugugguuuuugc......................................................    11    0     MCF
......aaaaguaauugugguuuuugcc.....................................................    42    0     MCF
......aaaaguaauugugguuuuugcca....................................................    1     0     MCF
.......aaaguaauugugguuuuugcc.....................................................    1     0     MCF

......aaaaguaauugugguuuuu........................................................    2     0     NL1
......aaaaguaauugugguuuuug.......................................................    2     0     NL1
......aaaaguaauugugguuuuugc......................................................    1     0     NL1

.....caaaaguaauugugguuuuugc......................................................    1     0     MBM
......aaaaguaauugugguuuuug.......................................................    2     0     MBM
......aaaaguaauugugguuuuugc......................................................    14    0     MBM
......aaaaguaauugugguuuuugcc.....................................................    33    0     MBM
......aaaaguaauugugguuuuugcca....................................................    6     0     MBM
.......aaaguaauugugguuuuugc......................................................    1     0     MBM
.......aaaguaauugugguuuuugcc.....................................................    1     0     MBM
.........guaauugugguuuuugcc......................................................    1     0     MBM

.ggugcaaaaguaauuguggu............................................................    1     0     k07
...ugcaaaaguaauugugguuuuu........................................................    1     0     k07
.....caaaaguaauugugguuuuug.......................................................    9     0     k07
......aaaaguaauugugguuuuug.......................................................    3     0     k07
......aaaaguaauugugguuuuugc......................................................    1     0     k07

...ugcaaaaguaauugugguuuuu........................................................    1     0     sh1
.....caaaaguaauugugguuuuug.......................................................    6     0     sh1
......aaaaguaauugugguuuuug.......................................................    4     0     sh1

uggugcaaaaguaauugugguu...........................................................    1     0     li1
.....caaaaguaauugugguuuuug.......................................................    3     0     li1
.....caaaaguaauugugguuuuugc......................................................    1     0     li1
......aaaaguaauugugguuuuugc......................................................    23    0     li1
......aaaaguaauugugguuuuugcc.....................................................    2     0     li1
.......aaaguaauugugguuuuugc......................................................    1     0     li1

.....caaaaguaauugugguuuuu........................................................    1     0     k13
.....caaaaguaauugugguuuuug.......................................................    4     0     k13
......aaaaguaauugugguuuuug.......................................................    1     0     k13

.....caaaaguaauugugguuuu.........................................................    1     0     j20
.....caaaaguaauugugguuuuu........................................................    2     0     j20
.....caaaaguaauugugguuuuug.......................................................    7     0     j20
......aaaaguaauugugguuuuu........................................................    3     0     j20
......aaaaguaauugugguuuuug.......................................................    27    0     j20
......aaaaguaauugugguuuuugcc.....................................................    1     0     j20
........aaguaauugugguuuuugc......................................................    1     0     j20
```

Mature Star

```
uggugcaaaaguaauugugguuuuugccauuacuuuuuuuuuaaauggcaaauaccacaguuacuuuugcacuaauguaau

....gcaaaaguaauugugguuuuug.......................................................   11   0   A54
......aaaaguaauugugguuuuug.......................................................   3    0   A54
......aaaaguaauugugguuuuugc......................................................   3    0   A54
......aaaaguaauugugguuuuugcc.....................................................   50   0   A54
......aaaaguaauugugguuuuugcca....................................................   5    0   A54
.......aaaguaauugugguuuuugcc.....................................................   5    0   A54

uggugcaaaaguaauugugguu...........................................................   1    0   j30
......aaaaguaauugugguuuuug.......................................................   2    0   j30
......aaaaguaauugugguuuuugcc.....................................................   1    0   j30

.....caaaaguaauugugguuuuu........................................................   8    0   k12
.....caaaaguaauugugguuuuug.......................................................   12   0   k12
......aaaaguaauugugguuuuu........................................................   6    0   k12
......aaaaguaauugugguuuuug.......................................................   18   0   k12
......aaaaguaauugugguuuuugc......................................................   2    0   k12
......aaaaguaauugugguuuuugcc.....................................................   1    0   k12
```

Provisional ID	:	hsa_can_685
Score total	:	15.9
Score for star read(s)	:	3.9
Score for read counts	:	10.3
Score for mfe	:	2.3
Score for randfold	:	
Score for cons. seed	:	-0.6
Total read count	:	32
Mature read count	:	26
Loop read count	:	0
Star read count	:	6

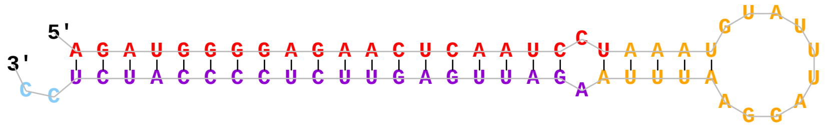

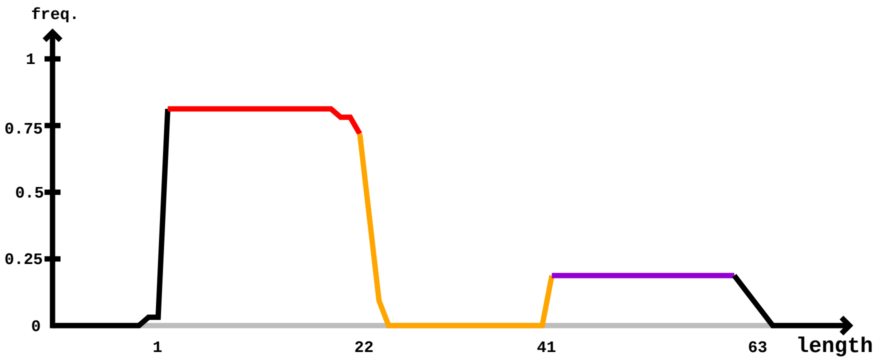

Mature **Star**

```
5'- agagaugaggagauggggagaacucaauccuaaauguauuuaggaauuuaagauugaguucuccccaucuccccaucucua   -3'    obs
    agagaugaggagauggggagaacucaauccuaaauguauuuaggaauuuaagauugaguucuccccaucucccaucucua          exp
    ((((((.((((((((((((((((((((.(((((..........))))).))))))))))))))))))))).))))))).    reads      mm         sample
    ..........agauggggagaacucaauccu..................................................   2          0          NL2
    ..........agauggggagaacucaauccua.................................................   1          0          NL2

    ..........agauggggagaacucaauccuaa................................................   1          0          j13

    ..........agauggggagaacucaaucc...................................................   1          0          j19
    ..................................................agauugaguucuccccaucu...........   2          0          j19

    ..........agauggggagaacucaauccua.................................................   2          0          va1
    ..........agauggggagaacucaauccuaa................................................   1          0          va1
    ..................................................agauugaguucuccccaucu...........   1          0          va1
    ..................................................agauugaguucuccccaucuc..........   2          0          va1

    ..........agauggggagaacucaauccua.................................................   1          0          j10

    ..........agauggggagaacucaauccu..................................................   1          0          j21
    ..........agauggggagaacucaauccua.................................................   1          0          j21

    ..........agauggggagaacucaauccua.................................................   1          0          ya3

    ..........agauggggagaacucaauccuaa................................................   1          0          j31

    ..........agauggggagaacucaauccu..................................................   4          0          va3
    ..........agauggggagaacucaauccua.................................................   1          0          va3

    ........ggagauggggagaacucaau.....................................................   1          0          ku4

    ..........agauggggagaacucaauccu..................................................   2          0          ya4

    ..........agauggggagaacucaaucc...................................................   1          0          j15
    ..........agauggggagaacucaauccu..................................................   1          0          j15

    ..........agauggggagaacucaauccu..................................................   1          0          j17
```

Mature Star

agagaugaggagauggggagaacucaauccuaaauguauuuaggaauuuaagauugaguucuccccaucuccccaucucua

..........agauggggagaacucaauccua... 2 0 j17

....agauugaguucuccccaucucc......... 1 0 MBM

Provisional ID : hsa_can_688
Score total : 42.9
Score for star read(s) : 3.9
Score for read counts : 37.3
Score for mfe : 2.2
Score for randfold :
Score for cons. seed : -0.6
Total read count : 85
Mature read count : 75
Loop read count : 0
Star read count : 10

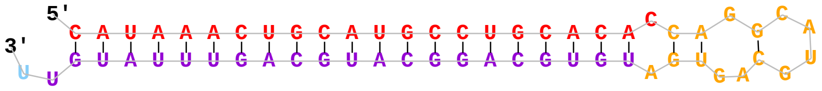

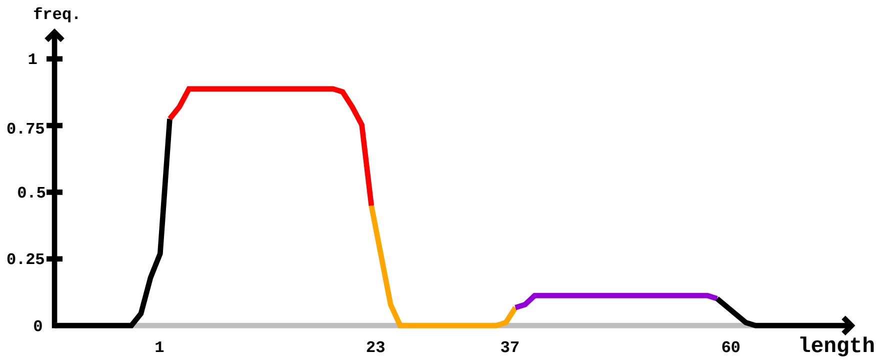


```
                 Mature                              Star
5'- caucacucagcauaaacugcaugccugcacaccaggcaugcagugaugugcaggcaugcaguuuauguuaagugaugcuuu  -3'   obs
    caucacucagcauaaacugcaugccugcacaccaggcaugcagugaugugcaggcaugcaguuuauguuaagugaugcuuu        exp
    (((((((.(((((((((((((((((((((((.((.(....)..)).))))))))))))))))))))))).))))))).... reads   mm    sample
    .........gcauaaacugcaugccugcaca.................................................. 1       0     j23
    ..........cauaaacugcaugccugcaca.................................................. 1       0     j23
    ..........cauaaacugcaugccugcacac................................................. 1       0     j23

    .......cagcauaaacugcaugccugca.................................................... 1       0     ya1
    .........gcauaaacugcaugccugcaca.................................................. 1       0     ya1

    ..............................................ugugcaggcaugcaguuuauguu............ 1       0     s04

    ..........cauaaacugcaugccugcaca.................................................. 1       0     j26

    ..........cauaaacugcaugccugcaca.................................................. 1       0     ta2

    .......cagcauaaacugcaugccugca.................................................... 1       0     va1
    ..........cauaaacugcaugccugcacac................................................. 1       0     va1
    ..............................................ugugcaggcaugcaguuuaugu............. 1       0     va1

    ..........cauaaacugcaugccugcaca.................................................. 1       0     k05

    ........agcauaaacugcaugccugcac................................................... 1       0     s06
    ..........cauaaacugcaugccugcacac................................................. 1       0     s06

    ..........cauaaacugcaugccugca.................................................... 1       0     s09

    ........agcauaaacugcaugccugca.................................................... 1       0     ku4
    .........gcauaaacugcaugccugcaca.................................................. 1       0     ku4

    ..........cauaaacugcaugccugcacacc................................................ 3       0     s07

    ..........cauaaacugcaugccugcacacc................................................ 1       0     s10

    ...........uaaacugcaugccugcacac.................................................. 1       0     ta3

    ..........cauaaacugcaugccugcaca.................................................. 1       0     NL2
```

Mature **Star**

```
caucacucagcauaaacugcaugccugcacaccaggcaugcagugaugugcaggcaugcaguuuauguuaagugaugcuuu
............uaaacugcaugccugcacacca...............................................   1   0   NL2

..........cauaaacugcaugccugcaca..................................................   1   0   j03

............uaaacugcaugccugcacacc................................................   1   0   k04
................................................ugcaggcaugcaguuuauguu............   2   0   k04

..........cauaaacugcaugccugcacac.................................................   1   0   per

.........gcauaaacugcaugccugcaca..................................................   1   0   s01

..........cauaaacugcaugccugcacacca...............................................   1   0   ku2

..........cauaaacugcaugccugcacacca...............................................   2   0   j10

..........cauaaacugcaugccugcacac.................................................   6   0   j21
...........auaaacugcaugccugcaca..................................................   1   0   j21

..........cauaaacugcaugccugcacac.................................................   1   0   j31
............uaaacugcaugccugcacacca...............................................   1   0   j31
................................................ugcaggcaugcaguuuauguuaa..........   1   0   j31

........agcauaaacugcaugccugcac...................................................   2   0   j14

........agcauaaacugcaugccugcaca..................................................   1   0   j22
.........gcauaaacugcaugccugcaca..................................................   4   0   j22
..........cauaaacugcaugccugcacac.................................................   3   0   j22
..........cauaaacugcaugccugcacacc................................................   1   0   j22

........agcauaaacugcaugccugcaca..................................................   2   0   DLD

........agcauaaacugcaugccugcaca..................................................   1   0   B14
..............................................ugugcaggcaugcaguuuaugu.............   1   0   B14

..........cauaaacugcaugccugcacac.................................................   1   0   NL3

..........cauaaacugcaugccugcacac.................................................   1   0   ya2

........agcauaaacugcaugccugcaca..................................................   2   0   j11

.......cagcauaaacugcaugccugca....................................................   1   0   ya3

.......cagcauaaacugcaugccugc.....................................................   1   0   HEK

..........cauaaacugcaugccugcaca..................................................   1   0   sh2
..........cauaaacugcaugccugcacacc................................................   3   0   sh2

...........auaaacugcaugccugcac...................................................   3   0   MCF

..........cauaaacugcaugccugcacac.................................................   3   0   j17
..........cauaaacugcaugccugcacacc................................................   1   0   j17

..........cauaaacugcaugccugcaca..................................................   1   0   NL1

..........cauaaacugcaugccugcacac.................................................   3   0   MBM

............uaaacugcaugccugcacacca...............................................   1   0   k07

..............................................ugugcaggcaugcaguuuauguu............   1   0   li1
...............................................gugcaggcaugcaguuuauguu............   1   0   li1

.............................................augugcaggcaugcaguuuaug..............   1   0   li2

..........cauaaacugcaugccugcaca..................................................   1   0   k13

........agcauaaacugcaugccugcaca..................................................   1   0   j20
............uaaacugcaugccugcacacca...............................................   1   0   j20

..............................................ugugcaggcaugcaguuuaugu.............   1   0   SW4

..........cauaaacugcaugccugcaca..................................................   1   0   ya4
```

Mature **Star**

caucacucagcauaaacugcaugccugcacaccaggcaugcagugaugugcaggcaugcaguuuauguuaagugaugcuuu

........agcauaaacugcaugccugcaca.. 1 0 ku1

Provisional ID	:	hsa_can_689
Score total	:	0
Score for star read(s)	:	-1.3
Score for read counts	:	0
Score for mfe	:	1.8
Score for randfold	:	
Score for cons. seed	:	-0.6
Total read count	:	75
Mature read count	:	75
Loop read count	:	0
Star read count	:	0

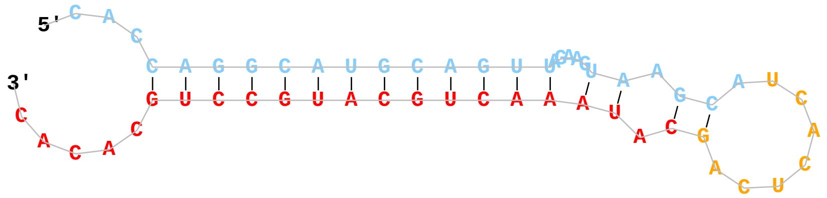

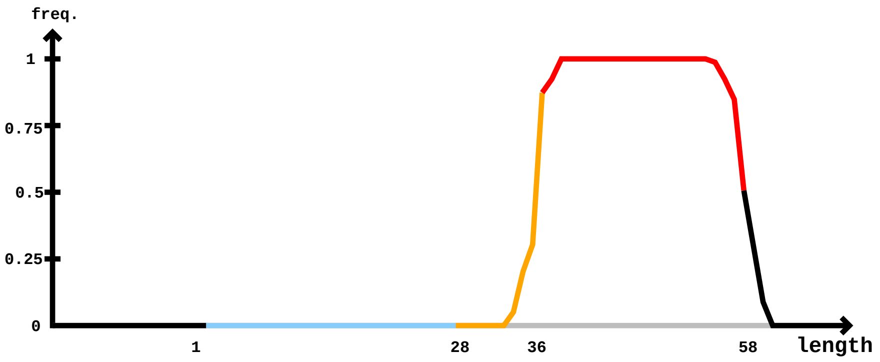

Star Mature

```
5'- acugcaugccugcacaccaggcaugcaguuagaaguaagcaucacucagcauaaacugcaugccugcacaccaggcaugca   -3'    exp
    ...((((((((.....((((((((((((.....((.((........)).))))))))))))))).....))))))))).     reads      mm         sample
    ................................................gcauaaacugcaugccugcaca...........     1          0          j23
    .................................................cauaaacugcaugccugcaca...........     1          0          j23
    .................................................cauaaacugcaugccugcacac..........     1          0          j23

    ..............................................cagcauaaacugcaugccugca.............     1          0          ya1
    ................................................gcauaaacugcaugccugcaca...........     1          0          ya1

    .................................................cauaaacugcaugccugcaca...........     1          0          j26

    .................................................cauaaacugcaugccugcaca...........     1          0          ta2

    ..............................................cagcauaaacugcaugccugca.............     1          0          va1
    .................................................cauaaacugcaugccugcacac..........     1          0          va1

    .................................................cauaaacugcaugccugcaca...........     1          0          k05

    ...............................................agcauaaacugcaugccugcac............     1          0          s06
    .................................................cauaaacugcaugccugcacac..........     1          0          s06

    .................................................cauaaacugcaugccugca.............     1          0          s09

    ...............................................agcauaaacugcaugccugca.............     1          0          ku4
    ................................................gcauaaacugcaugccugcaca...........     1          0          ku4

    .................................................cauaaacugcaugccugcacacc.........     3          0          s07

    .................................................cauaaacugcaugccugcacacc.........     1          0          s10

    ...................................................uaaacugcaugccugcacac..........     1          0          ta3

    .................................................cauaaacugcaugccugcaca...........     1          0          NL2
    ...................................................uaaacugcaugccugcacacca........     1          0          NL2

    .................................................cauaaacugcaugccugcaca...........     1          0          j03
```

```
             Star                            Mature
acugcaugccugcacaccaggcaugcaguuagaaguaagcaucacucagcauaaacugcaugccugcacaccaggcaugca

...................................................uaaacugcaugccugcacacc.........   1   0   k04

.................................................cauaaacugcaugccugcacac..........   1   0   per

................................................gcauaaacugcaugccugcaca...........   1   0   s01

.................................................cauaaacugcaugccugcacacca........   1   0   ku2

.................................................cauaaacugcaugccugcacacca........   2   0   j10

.................................................cauaaacugcaugccugcacac..........   6   0   j21
..................................................auaaacugcaugccugcaca...........   1   0   j21

.................................................cauaaacugcaugccugcacac..........   1   0   j31
...................................................uaaacugcaugccugcacacca........   1   0   j31

...............................................agcauaaacugcaugccugcac............   2   0   j14

...............................................agcauaaacugcaugccugcaca...........   1   0   j22
................................................gcauaaacugcaugccugcaca...........   4   0   j22
.................................................cauaaacugcaugccugcacac..........   3   0   j22
.................................................cauaaacugcaugccugcacacc.........   1   0   j22

...............................................agcauaaacugcaugccugcaca...........   2   0   DLD

...............................................agcauaaacugcaugccugcaca...........   1   0   B14

.................................................cauaaacugcaugccugcacac..........   1   0   NL3

.................................................cauaaacugcaugccugcacac..........   1   0   ya2

...............................................agcauaaacugcaugccugcaca...........   2   0   j11

..............................................cagcauaaacugcaugccugca.............   1   0   ya3

..............................................cagcauaaacugcaugccugc..............   1   0   HEK

.................................................cauaaacugcaugccugcaca...........   1   0   sh2
.................................................cauaaacugcaugccugcacacc.........   3   0   sh2

..................................................auaaacugcaugccugcac............   3   0   MCF

.................................................cauaaacugcaugccugcacac..........   3   0   j17
.................................................cauaaacugcaugccugcacacc.........   1   0   j17

.................................................cauaaacugcaugccugcaca...........   1   0   NL1

.................................................cauaaacugcaugccugcacac..........   3   0   MBM

...................................................uaaacugcaugccugcacacca........   1   0   k07

.................................................cauaaacugcaugccugcaca...........   1   0   k13

...............................................agcauaaacugcaugccugcaca...........   1   0   j20
...................................................uaaacugcaugccugcacacca........   1   0   j20

.................................................cauaaacugcaugccugcaca...........   1   0   ya4

...............................................agcauaaacugcaugccugcaca...........   1   0   ku1
```

Provisional ID	:	hsa_can_698
Score total	:	271.7
Score for star read(s)	:	3.9
Score for read counts	:	266.8
Score for mfe	:	1.6
Score for randfold	:	
Score for cons. seed	:	-0.6
Total read count	:	535
Mature read count	:	521
Loop read count	:	0
Star read count	:	14

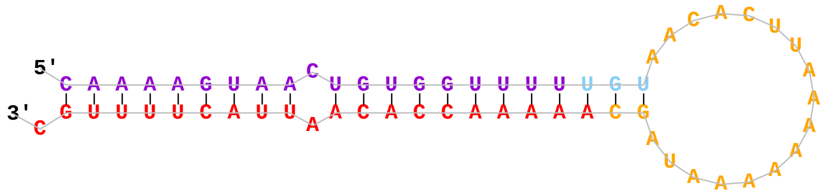

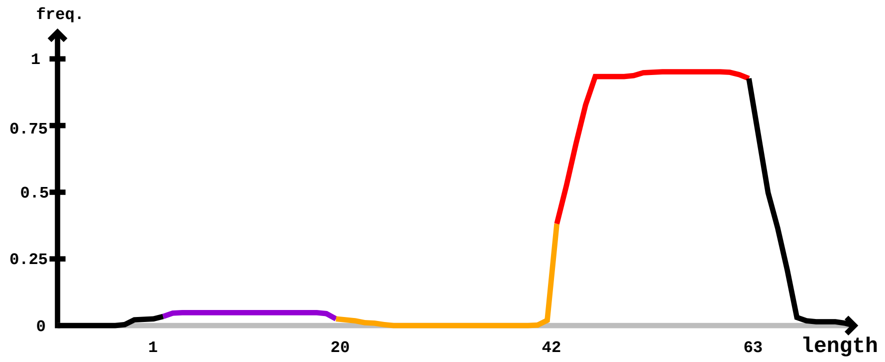

Star **Mature**

```
5'- agauuggugcaaaaguaacugugguuuuuuguaacacuuaaaaaaaaauagcaaaaaccacaauuacuuuugcaccaaccuaa   -3'   obs
    agauuggugcaaaaguaacugugguuuuuuguaacacuuaaaaaaaaauagcaaaaaccacaauuacuuuugcaccaaccuaa         exp
    agauuggugcaaaaguaacugugguuuuuuguaacacuuaaaaaaaaauagcaaaaaccacaauuacuuuugcaccaaccuaa         known
    ((.((((((((((((((.(((((((((((.................))))))))))).)))))))))))))).))..    reads   mm   sample
    ....................................................aaaaaccacaauuacuuuug...........    1   0   ya1
    ....................................................aaaaaccacaauuacuuuugca.........    4   0   ya1
    .....................................................aaaaccacaauuacuuuugca.........    3   0   ya1
    .....................................................aaaaccacaauuacuuuugcac........    1   0   ya1
    ......................................................aaaccacaauuacuuuugca.........    1   0   ya1
    .......................................................aaccacaauuacuuuugcacc.......    1   0   ya1

    ....................................................aaaaaccacaauuacuuuugca.........    1   0   s04

    ....................................................aaaaaccacaauuacuuuugca.........    3   0   j26
    ........................................................accacaauuacuuuugcacca......    1   0   j26

    .......ugcaaaaguaacugugguuuuug.....................................................    1   0   va1
    ....................................................aaaaaccacaauuacuuuugc..........    1   0   va1
    ....................................................aaaaaccacaauuacuuuugca.........    6   0   va1
    ....................................................aaaaaccacaauuacuuuugcac........    1   0   va1
    .....................................................aaaaccacaauuacuuuugcac........    5   0   va1
    ......................................................aaaccacaauuacuuuugcacc.......    1   0   va1

    ....................................................aaaaaccacaauuacuuuugca.........    1   0   ku5
    ......................................................aaaccacaauuacuuuugcacc.......    1   0   ku5

    ......................................................aaaccacaauuacuuuugcacc.......    1   0   k05

    ....................................................aaaaaccacaauuacuuuugca.........    1   0   s06
    .......................................................aaccacaauuacuuuugcacca......    1   0   s06
    ........................................................accacaauuacuuuugcacca......    1   0   s06

    ....................................................aaaaaccacaauuacuuuugc..........    1   0   HeL
    ....................................................aaaaaccacaauuacuuuugca.........    3   0   HeL
    .....................................................aaaaccacaauuacuuuugcac........    2   0   HeL
    ......................................................aaaccacaauuacuuuugcac........    1   0   HeL
```

Star **Mature**

```
Star                                           Mature
agauuggugcaaaaguaacugugguuuuuguaacacuuaaaaaaaauagcaaaaaccacaauuacuuuugcaccaaccuaa
....................................................aaaccacaauuacuuuugcacc.......	3	0	HeL
.....................................................aaccacaauuacuuuugcacca......	3	0	HeL
.....................................................aaccacaauuacuuuugcaccaa.....	3	0	HeL
......................................................accacaauuacuuuugcacca......	5	0	HeL

....................................................aaaccacaauuacuuuugcacc.......	1	0	s07

..................................................aaaaaccacaauuacuuuugca.........	4	0	s10
....................................................aaaccacaauuacuuuugcac........	1	0	s10

..................................................aaaaaccacaauuacuuuugca.........	4	0	ta3
...................................................aaaaccacaauuacuuuugc..........	1	0	ta3
...................................................aaaaccacaauuacuuuugcac........	1	0	ta3
....................................................aaaccacaauuacuuuugcac........	1	0	ta3
....................................................aaaccacaauuacuuuugcacc.......	1	0	ta3
.....................................................aaccacaauuacuuuugcacca......	2	0	ta3

..................................................aaaaaccacaauuacuuuugca.........	5	0	j10
....................................................aaaccacaauuacuuuugcac........	1	0	j10
.....................................................aaccacaauuacuuuugcacca......	1	0	j10

.....................................................aaccacaauuacuuuugcac........	1	0	k14

..................................................aaaaaccacaauuacuuuugcac........	1	0	va2
..........................................................caauuacuuuugcaccaaccuaa	1	0	va2

....................................................aaaccacaauuacuuuugca.........	1	0	j27

..................................................aaaaaccacaauuacuuuugca.........	1	0	j31

..................................................aaaaaccacaauuacuuuugca.........	2	0	s03
...................................................aaaaccacaauuacuuuugca.........	1	0	s03
...................................................aaaaccacaauuacuuuugcac........	2	0	s03

..................................................aaaaaccacaauuacuuuug...........	2	0	H52
..................................................aaaaaccacaauuacuuuugca.........	1	0	H52
...................................................aaaaccacaauuacuuuugc..........	1	0	H52
...................................................aaaaccacaauuacuuuugca.........	2	0	H52
....................................................aaaccacaauuacuuuugcacc.......	2	0	H52
.....................................................aaccacaauuacuuuugcacc.......	1	0	H52
.....................................................aaccacaauuacuuuugcacca......	1	0	H52
......................................................accacaauuacuuuugcacca......	1	0	H52

..................................................aaaaaccacaauuacuuuugca.........	6	0	j22
...................................................aaaaccacaauuacuuuugca.........	1	0	j22
.....................................................aaccacaauuacuuuugcacc.......	1	0	j22

...................................................aaaaccacaauuacuuuugcac........	1	0	k09
....................................................aaaccacaauuacuuuugcaccaa.....	1	0	k09

..................................................aaaaaccacaauuacuuuugc..........	3	0	B14
..................................................aaaaaccacaauuacuuuugca.........	6	0	B14
...................................................aaaaccacaauuacuuuugcac........	1	0	B14
....................................................aaaccacaauuacuuuugca.........	2	0	B14
....................................................aaaccacaauuacuuuugcacc.......	2	0	B14
......................................................accacaauuacuuuugcacca......	2	0	B14

..................................................aaaaaccacaauuacuuuugcac........	1	0	ku3
....................................................aaaccacaauuacuuuugcacca......	1	0	ku3

..................................................aaaaaccacaauuacuuuugca.........	4	0	j16

..................................................aaaaaccacaauuacuuuugca.........	1	0	j09
......................................................accacaauuacuuuugcacca......	1	0	j09

..................................................aaaaaccacaauuacuuuugca.........	2	0	ku6

..................................................aaaaaccacaauuacuuuugca.........	2	0	j11

......gugcaaaaguaacugugguuu......................................................	1	0	ya3
```

Star Mature

```
agauuggugcaaaaguaacugugguuuuuguaacacuuaaaaaaaauagcaaaaaccacaauuacuuuugcaccaaccuaa
..................................................aaaaaccacaauuacuuuugca.........    3    0    ya3
...................................................aaaaccacaauuacuuuugca.........    2    0    ya3
...................................................aaaaccacaauuacuuuugcac........    5    0    ya3
....................................................aaaccacaauuacuuuugca.........    1    0    ya3
....................................................aaaccacaauuacuuuugcacc.......    1    0    ya3
.....................................................aaccacaauuacuuuugcacc.......    2    0    ya3

..................................................aaaaaccacaauuacuuuugca.........    2    0    la1
.....................................................aaccacaauuacuuuugcacc.......    1    0    la1

.................................................caaaaaccacaauuacuuuugc..........    3    0    HEK
..................................................aaaaaccacaauuacuuuugc..........    1    0    HEK
..................................................aaaaaccacaauuacuuuugca.........    8    0    HEK
...................................................aaaaccacaauuacuuuugca.........    2    0    HEK
...................................................aaaaccacaauuacuuuugcac........    3    0    HEK
....................................................aaaccacaauuacuuuugca.........    1    0    HEK
....................................................aaaccacaauuacuuuugcac........    3    0    HEK
....................................................aaaccacaauuacuuuugcacc.......    3    0    HEK
.....................................................aaccacaauuacuuuugcacc.......    2    0    HEK
.....................................................aaccacaauuacuuuugcacca......    4    0    HEK
......................................................accacaauuacuuuugcacca......    6    0    HEK

........gcaaaaguaacugugguuuu.....................................................    1    0    j15
...................................................aaaaccacaauuacuuuugca.........    1    0    j15

......gugcaaaaguaacugugguuu......................................................    1    0    j17
.........caaaaguaacugugguuu......................................................    1    0    j17
.........caaaaguaacugugguuuu.....................................................    2    0    j17
...................................................aaaaccacaauuacuuuugca.........    1    0    j17
.....................................................aaccacaauuacuuuugcacca......    2    0    j17
......................................................accacaauuacuuuugcacca......    1    0    j17

...................................................aaaaccacaauuacuuuugcac........    1    0    la2
....................................................aaaccacaauuacuuuugca.........    1    0    la2
....................................................aaaccacaauuacuuuugcacc.......    1    0    la2
.....................................................aaccacaauuacuuuugcacca......    1    0    la2

......................................................accacaauuacuuuugcacca......    1    0    j12

..................................................aaaaaccacaauuacuuuugca.........    3    0    va4
..................................................aaaaaccacaauuacuuuugcac........    1    0    va4
...................................................aaaaccacaauuacuuuugcac........    1    0    va4
.....................................................aaccacaauuacuuuugcacc.......    2    0    va4
......................................................accacaauuacuuuugcacca......    4    0    va4

...........aaaguaacugugguuuuuguaa................................................    1    0    va3
..................................................aaaaaccacaauuacuuuugca.........    12   0    va3
...................................................aaaaccacaauuacuuuugcac........    2    0    va3
....................................................aaaccacaauuacuuuugcac........    1    0    va3
....................................................aaaccacaauuacuuuugcacc.......    4    0    va3
.....................................................aaccacaauuacuuuugcacc.......    2    0    va3
.....................................................aaccacaauuacuuuugcacca......    2    0    va3
......................................................accacaauuacuuuugcacca......    1    0    va3

..................................................aaaaaccacaauuacuuuugca.........    4    0    ya4
...................................................aaaaccacaauuacuuuugca.........    2    0    ya4
...................................................aaaaccacaauuacuuuugcac........    4    0    ya4
....................................................aaaccacaauuacuuuugcac........    1    0    ya4
....................................................aaaccacaauuacuuuugcacc.......    4    0    ya4
.....................................................aaccacaauuacuuuugca.........    3    0    ya4
.....................................................aaccacaauuacuuuugcacc.......    4    0    ya4
.....................................................aaccacaauuacuuuugcacca......    2    0    ya4

.........caaaaguaacugugguuuu.....................................................    1    0    SW4
..................................................aaaaaccacaauuacuuuugca.........    1    0    SW4
...................................................aaaaccacaauuacuuuugcac........    1    0    SW4
....................................................aaaccacaauuacuuuugcac........    2    0    SW4
....................................................aaaccacaauuacuuuugcacc.......    3    0    SW4
.....................................................aaccacaauuacuuuugcacca......    3    0    SW4
......................................................accacaauuacuuuugcacc.......    1    0    SW4
```

```
         Star                                   Mature
agauuggugcaaaaguaacugugguuuuuguaacacuuaaaaaaaauagcaaaaaccacaauuacuuuugcaccaaccuaa
......................................................accacaauuacuuuugcacca......    3     0     SW4

..................................................aaaaaccacaauuacuuuugca.........    4     0     ku1
.....................................................aaccacaauuacuuuugcacc.......    1     0     ku1

.....ggugcaaaaguaacugugguuu......................................................    1     0     j23
......gugcaaaaguaacugugguuu......................................................    5     0     j23
..................................................aaaaaccacaauuacuuuugc..........    1     0     j23
..................................................aaaaaccacaauuacuuuugca.........    2     0     j23
...................................................aaaaccacaauuacuuuugca.........    1     0     j23
.....................................................aaccacaauuacuuuugcacca......    1     0     j23
......................................................accacaauuacuuuugcacca......    1     0     j23

..................................................aaaaaccacaauuacuuuugca.........    2     0     s05

..................................................aaaaaccacaauuacuuu.............    1     0     ta2
...................................................aaaaccacaauuacuuuugcac........    1     0     ta2

.....ggugcaaaaguaacugugguuu......................................................    1     0     k11
..................................................aaaaaccacaauuacuuuugca.........    1     0     k11

..................................................aaaaaccacaauuacuuuugca.........    1     0     ta1

.................................................caaaaaccacaauuacuuuugc..........    1     0     s09
...................................................aaaaccacaauuacuuuugca.........    1     0     s09
.....................................................aaccacaauuacuuuugcacca......    1     0     s09
......................................................accacaauuacuuuugcacca......    1     0     s09

.................................................caaaaaccacaauuacuuu.............    1     0     NL2
..................................................aaaaaccacaauuacuuuugca.........    3     0     NL2
.....................................................aaccacaauuacuuuugcacc.......    1     0     NL2
.....................................................aaccacaauuacuuuugcacca......    5     0     NL2

..................................................aaaaaccacaauuacuuuugca.........    4     0     j13
...................................................aaaaccacaauuacuuuugca.........    2     0     j13
.....................................................aaccacaauuacuuuugcacca......    2     0     j13

..................................................aaaaaccacaauuacuuuugca.........    2     0     j19
....................................................aaaccacaauuacuuuugca.........    2     0     j19
.....................................................aaccacaauuacuuuugcacca......    1     0     j19
......................................................accacaauuacuuuugcacca......    2     0     j19

..................................................aaaaaccacaauuacuuuugca.........    1     0     j25

..........aaaaguaacugugguuuuuguaa................................................    1     0     per
...........................................................aauuacuuuugcaccaaccua.    2     0     per

..................................................aaaaaccacaauuacuuuugca.........    2     0     s01
...................................................aaaaccacaauuacuuuugcac........    2     0     s01
....................................................aaaccacaauuacuuuugcacc.......    2     0     s01

...................................................aaaaccacaauuacuuuugca.........    1     0     k04

.................................................caaaaaccacaauuacuuuugc..........    1     0     U20
..................................................aaaaaccacaauuacuuuugc..........    22    0     U20
..................................................aaaaaccacaauuacuuuugca.........    17    0     U20
...................................................aaaaccacaauuacuuuugca.........    1     0     U20
...................................................aaaaccacaauuacuuuugcac........    8     0     U20
....................................................aaaccacaauuacuuuugcac........    6     0     U20
....................................................aaaccacaauuacuuuugcacc.......    14    0     U20
.....................................................aaccacaauuacuuuugcacc.......    6     0     U20
.....................................................aaccacaauuacuuuugcacca......    1     0     U20
......................................................accacaauuacuuuugcacca......    15    0     U20
......................................................accacaauuacuuuugcaccaa.....    1     0     U20

..........aaaaguaacugugguuuuug...................................................    1     0     j21
..........aaaaguaacugugguuuuugua.................................................    1     0     j21
..................................................aaaaaccacaauuacuuuugca.........    3     0     j21
...................................................aaaaccacaauuacuuuugca.........    1     0     j21
....................................................aaaccacaauuacuuuugcacc.......    1     0     j21
```

```
         Star                  Mature
agauugguggcaaaaguaacugugguuuuuguaacacuuaaaaaaaauagcaaaaaccacaauuacuuuugcaccaaccuaa
```

```
.....................................................aaccacaauuacuuuugcacc.......   1   0   s11
.....................................................aaccacaauuacuuuugcacca......   1   0   s11

..................................................aaaaaccacaauuacuuuugca.........   1   0   j07

..........aaaaguaacugugguuuuugua.................................................   2   0   j14
....................................................aaaccacaauuacuuuugcacc.......   1   0   j14

......gugcaaaaguaacugugguuu......................................................   1   0   s08
..................................................aaaaaccacaauuacuuuugca.........   1   0   s08
......................................................accacaauuacuuuugcac........   1   0   s08

..................................................aaaaaccacaauuacuuuugc..........   1   0   DLD
...................................................aaaaccacaauuacuuuugcac........   1   0   DLD
....................................................aaaccacaauuacuuuugcacc.......   3   0   DLD
......................................................accacaauuacuuuugcacc.......   1   0   DLD
......................................................accacaauuacuuuugcacca......   4   0   DLD

..................................................aaaaaccacaauuacuuuugca.........   1   0   k03

..................................................aaaaaccacaauuacuuuu............   2   0   ya2
..................................................aaaaaccacaauuacuuuugc..........   1   0   ya2
..................................................aaaaaccacaauuacuuuugca.........   2   0   ya2
...................................................aaaaccacaauuacuuuugca.........   6   0   ya2
....................................................aaaccacaauuacuuuugcac........   3   0   ya2
....................................................aaaccacaauuacuuuugcacca......   1   0   ya2
.....................................................aaccacaauuacuuuugcacca......   2   0   ya2
..........................................................caauuacuuuugcaccaac....   1   0   ya2
...........................................................aauuacuuuugcaccaac....   1   0   ya2

.................................................caaaaaccacaauuacuuuu............   1   0   NL3
.................................................caaaaaccacaauuacuuuug...........   2   0   NL3
..................................................aaaaaccacaauuacuuuugca.........   7   0   NL3
...................................................aaaaccacaauuacuuuugcac........   1   0   NL3
.....................................................aaccacaauuacuuuugcacca......   1   0   NL3
............................................................auuacuuuugcaccaaccuaa   1   0   NL3

....................................................aaaccacaauuacuuuugcacc.......   1   0   k06

.................................................caaaaaccacaauuacuuuug...........   1   0   sh2
.....................................................aaccacaauuacuuuugcaccaa.....   2   0   sh2
.............................................................uuacuuuugcaccaaccua.   1   0   sh2

..................................................aaaaaccacaauuacuuuugca.........   1   0   j29
...................................................aaaaccacaauuacuuuugca.........   1   0   j29

..................................................aaaaaccacaauuacuuuu............   1   0   MCF
..................................................aaaaaccacaauuacuuuugc..........   1   0   MCF
..................................................aaaaaccacaauuacuuuugca.........   2   0   MCF
...................................................aaaaccacaauuacuuuugcac........   1   0   MCF
...........................................................aauuacuuuugcaccaaccuaa   2   0   MCF

..................................................aaaaaccacaauuacuuuug...........   1   0   MBM
..................................................aaaaaccacaauuacuuuugc..........   1   0   MBM
..................................................aaaaaccacaauuacuuuugca.........   2   0   MBM
...................................................aaaaccacaauuacuuuugca.........   1   0   MBM
...................................................aaaaccacaauuacuuuugcac........   3   0   MBM
....................................................aaaccacaauuacuuuugcacc.......   4   0   MBM
.....................................................aaccacaauuacuuuugcacc.......   1   0   MBM
......................................................accacaauuacuuuugcacca......   4   0   MBM

.........caaaaguaacugugguuuuugu..................................................   1   0   li1
................................................gcaaaaaccacaauuacuuuug...........   1   0   li1
..................................................aaaaaccacaauuacuuuugc..........   1   0   li1
...................................................aaaaccacaauuacuuuugcac........   1   0   li1
....................................................aaaccacaauuacuuuugcacc.......   2   0   li1
.....................................................aaccacaauuacuuuugcacca......   1   0   li1

...........................................................aauuacuuuugcaccaaccuaa   1   0   k13
```

Star **Mature**

```
agauuggugcaaaaguaacugugguuuuuguaacacuuaaaaaaaauagcaaaaaccacaauuacuuuugcaccaaccuaa

......gugcaaaaguaacugugguu.......................................................	2	0	j20
..........aaaaguaacugugguuuuug...................................................	1	0	j20

..................................................aaaaaccacaauuacuuuugc..........	7	0	A54
.....................................................aaccacaauuacuuuugcacc.......	4	0	A54
.....................................................aaccacaauuacuuuugcacca......	4	0	A54

..........aaaaguaacugugguuuuug...................................................	1	0	k12
......................................................accacaauuacuuuugcacca......	1	0	k12
```

Provisional ID	: hsa_can_720
Score total	: 0
Score for star read(s)	: -1.3
Score for read counts	: 0
Score for mfe	: 1.9
Score for randfold	:
Score for cons. seed	: -0.6
Total read count	: 167
Mature read count	: 167
Loop read count	: 0
Star read count	: 0

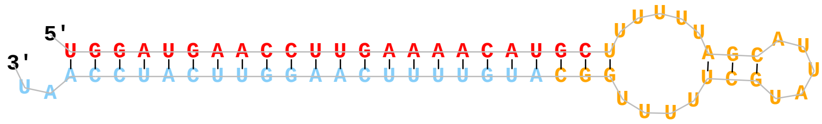

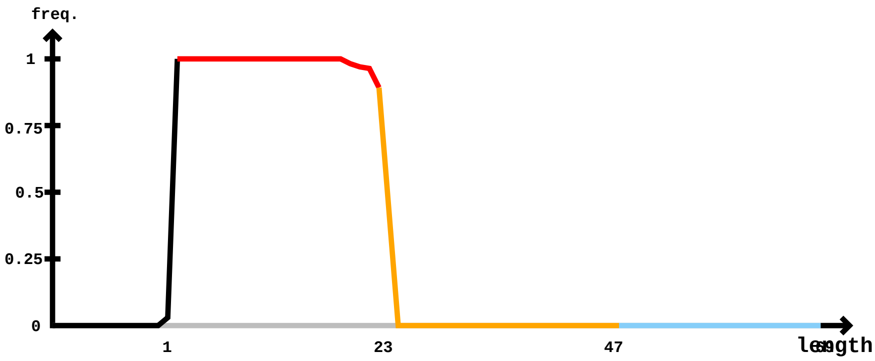

Mature **Star**

5'- guacugcaacuuggaugaaccuugaaaacaugcuuuuuuagcauuaugcuuuuuggcauguuuucaagguucauccaauuu -3'	exp		
..........((((((((((((((((((((((((.....(((.....)))....))))))))))))))))))))))))....	reads	mm	sample
...........uggaugaaccuugaaaacaugc..	4	0	j23
...........uggaugaaccuugaaaacaugc..	1	0	ya1
...........uggaugaaccuugaaaacaugc..	1	0	s05
...........uggaugaaccuugaaaacaugc..	5	0	va1
...........uggaugaaccuugaaaacaugc..	1	0	ta2
...........uggaugaaccuugaaaacaugc..	3	0	k11
...........uggaugaaccuugaaaacaugc..	2	0	k05
...........uggaugaaccuugaaaacaugc..	3	0	s06
..........uuggaugaaccuugaaaacaug...	1	0	ta1
...........uggaugaaccuugaaaacaugc..	1	0	ta1
...........uggaugaaccuugaaaacaugc..	1	0	s09
...........uggaugaaccuugaaaacaugc..	1	0	s10
..........uuggaugaaccuugaaaacaugc..	1	0	k10
...........uggaugaaccuugaaaacaugc..	1	0	k10
..........uuggaugaaccuugaaaacaug...	2	0	j19
...........uggaugaaccuugaaaacaug...	1	0	j19
..........uuggaugaaccuugaaaacaugc..	1	0	k04
...........uggaugaaccuugaaaacaugc..	1	0	k04
...........uggaugaaccuugaaaaca...	1	0	s01
...........uggaugaaccuugaaaacaugc..	5	0	s01

Mature Star

guacugcaacuuggaugaaccuugaaaacaugcuuuuuuagcauuaugcuuuuuggcauguuuucaagguucauccaauuu

...........uggaugaaccuugaaaacaug...	1	0	per
...........uggaugaaccuugaaaacaugc..	1	0	ku2
...........uggaugaaccuugaaaacaugc..	8	0	j10
...........uggaugaaccuugaaaac..	2	0	j21
...........uggaugaaccuugaaaacaugc..	14	0	j21
...........uggaugaaccuugaaaacaugc..	2	0	va2
...........uggaugaaccuugaaaacaugc..	1	0	k14
...........uggaugaaccuugaaaacaugc..	3	0	j31
...........uggaugaaccuugaaaacaugc..	4	0	j14
...........uggaugaaccuugaaaacaugc..	1	0	k02
...........uggaugaaccuugaaaacaugc..	1	0	j28
...........uggaugaaccuugaaaacaugc..	4	0	j22
...........uggaugaaccuugaaaacaugc..	1	0	k09
...........uggaugaaccuugaaaacaugc..	3	0	k03
...........uggaugaaccuugaaaacaugc..	1	0	j09
...........uggaugaaccuugaaaacaugc..	1	0	j11
...........uggaugaaccuugaaaacaugc..	2	0	s02
...........uggaugaaccuugaaaacaugc..	1	0	ya3
...........uggaugaaccuugaaaacaugc..	3	0	k06
...........uggaugaaccuugaaaacaugc..	1	0	j29
...........uggaugaaccuugaaaacaug...	2	0	sh2
...........uggaugaaccuugaaaacaugc..	1	0	sh2
...........uggaugaaccuugaaaacaugc..	1	0	HEK
...........uggaugaaccuugaaaacaugc..	3	0	j17
...........uggaugaaccuugaaaac..	1	0	MBM
...........uggaugaaccuugaaaacaugc..	2	0	k07
...........uggaugaaccuugaaaacaugc..	1	0	j12
...........uggaugaaccuugaaaacaugc..	18	0	li2
...........uggaugaaccuugaaaacaug...	1	0	li1
...........uggaugaaccuugaaaacaugc..	28	0	li1
...........uggaugaaccuugaaaaca...	1	0	k13
...........uggaugaaccuugaaaacaugc..	1	0	k13
...........uggaugaaccuugaaaacaugc..	4	0	va4
...........uggaugaaccuugaaaacaugc..	2	0	j20
...........uggaugaaccuugaaaacaug...	1	0	va3
...........uggaugaaccuugaaaacaugc..	2	0	va3
...........uggaugaaccuugaaaacaug...	1	0	ya4
...........uggaugaaccuugaaaacaug...	1	0	j30

```
           Mature                                          Star
guacugcaacuuggaugaaccuugaaaacaugcuuuuuuagcauuaugcuuuuuggcauguuuucaagguucauccaauuu
...........uggaugaaccuugaaaacaugc................................................    1    0    j30

...........uggaugaaccuugaaaacau..................................................    1    0    k12
...........uggaugaaccuugaaaacaug.................................................    1    0    k12
...........uggaugaaccuugaaaacaugc................................................    1    0    k12
```

Provisional ID	: hsa_can_742
Score total	: 3.6
Score for star read(s)	: -1.3
Score for read counts	: 0
Score for mfe	: 1.9
Score for randfold	:
Score for cons. seed	: 3
Total read count	: 2407
Mature read count	: 2407
Loop read count	: 0
Star read count	: 0

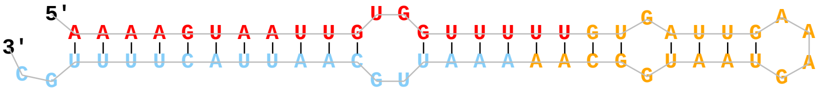

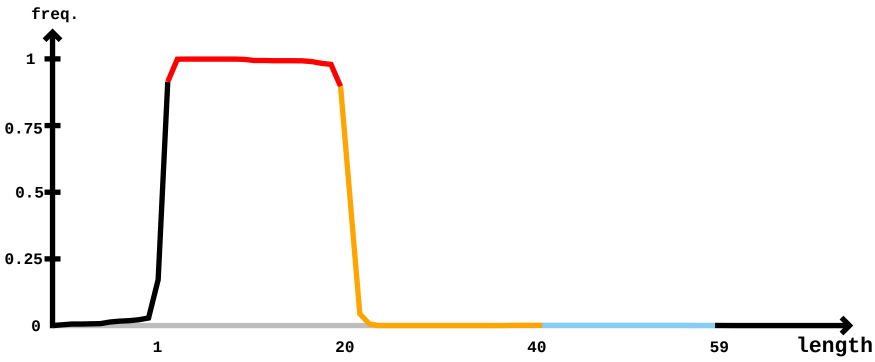

Mature **Star**

5'- aggcuggugcaaaaguaauugugguuuuugugauugaaaguaaauggcaaaaauugcaauuacuuuugcacuaaccuaaaua -3'	exp		
(((.((((((((((((((((..((((((((.((((....)))).))))))))..)))))))))))))))).))).....	reads	mm	sample
aggcuggugcaaaaguaau...	3	0	ya1
.........caaaaguaauugugguuuu..	5	0	ya1
.........caaaaguaauugugguuuuu...	22	0	ya1
.........caaaaguaauugugguuuuug..	1	0	ya1
.........caaaaguaauugugguuuuugu...	1	0	ya1
..........aaaaguaauugugguuuu..	16	0	ya1
..........aaaaguaauugugguuuuu...	111	0	ya1
..........aaaaguaauugugguuuuug..	8	0	ya1
...........aaaguaauugugguuuuu...	19	0	ya1
...........aaaguaauugugguuuuugu...	1	0	ya1
..........aaaaguaauugugguuuuug..	1	0	s04
.........caaaaguaauugugguuuuugu...	1	0	j26
..........aaaaguaauugugguuuuug..	1	0	j26
....uggugcaaaaguaauugugguu..	1	0	va1
..........aaaaguaauugugguuuuu...	2	0	va1
..........aaaaguaauugugguuuuug..	25	0	va1
..........aaaaguaauugugguuuuugu...	2	0	va1
...........aaaguaauugugguuuuug..	2	0	va1
...cuggugcaaaaguaauuguggu...	1	0	ku5
....uggugcaaaaguaauuguggu...	1	0	ku5
......gugcaaaaguaauugugguu..	1	0	ku5
.........caaaaguaauugugguuuuug..	1	0	ku5
..........aaaaguaauugugguuuuug..	3	0	ku5
.........caaaaguaauugugguuuuu...	2	0	k05
.........caaaaguaauugugguuuuug..	2	0	k05
.........caaaaguaauugugguuuuugu...	3	0	k05
..........aaaaguaauugugguuuuug..	3	0	k05
...........aaaguaauugugguuuuug..	1	0	k05
.........caaaaguaauugugguuuuug..	1	0	s06

Mature Star

```
aggcuggugcaaaaguaauugugguuuuugugauugaaaguaauggcaaaaauugcaauuacuuuugcacuaaccuaaaua

..........aaaaguaauugugguuuuugu..................................................    1     0     HeL
..............................................caaaaauugcaauuacuuuugc.............    1     0     HeL

......gugcaaaaguaauugugguu.......................................................    1     0     ku4
..........aaaaguaauugugguuuuu....................................................    5     0     ku4
..........aaaaguaauugugguuuuug...................................................    11    0     ku4

..........aaaaguaauugugguuuuug...................................................    2     0     s10
..........aaaaguaauugugguuuuugu..................................................    1     0     s10

.........caaaaguaauugugguuuuu....................................................    2     0     k08
.........caaaaguaauugugguuuuug...................................................    1     0     k08
.........caaaaguaauugugguuuuugu..................................................    2     0     k08
..........aaaaguaauugugguuuuu....................................................    1     0     k08
..........aaaaguaauugugguuuuug...................................................    1     0     k08

..........aaaaguaauugugguuuu.....................................................    1     0     ta3
..........aaaaguaauugugguuuuug...................................................    7     0     ta3
...........aaaguaauugugguuuuug...................................................    1     0     ta3

..........aaaaguaauugugguuuuu....................................................    2     0     j10
..........aaaaguaauugugguuuuug...................................................    6     0     j10

..........aaaaguaauugugguuuuug...................................................    3     0     j04

..........aaaaguaauugugguuuu.....................................................    1     0     j02
..........aaaaguaauugugguuuuu....................................................    5     0     j02
..........aaaaguaauugugguuuuug...................................................    22    0     j02
..........aaaaguaauugugguuuuugu..................................................    2     0     j02
...........aaaguaauugugguuuuug...................................................    1     0     j02

.........caaaaguaauugugguuuuu....................................................    2     0     k14
.........caaaaguaauugugguuuuug...................................................    4     0     k14
.........caaaaguaauugugguuuuugu..................................................    1     0     k14
..........aaaaguaauugugguuuuu....................................................    2     0     k14
..........aaaaguaauugugguuuuug...................................................    4     0     k14
..........aaaaguaauugugguuuuugu..................................................    1     0     k14
...........aaaguaauugugguuuuug...................................................    1     0     k14
............aaguaauugugguuuuugu..................................................    1     0     k14

.........caaaaguaauugugguuuuu....................................................    1     0     va2
.........caaaaguaauugugguuuuugu..................................................    1     0     va2
..........aaaaguaauugugguuuuu....................................................    1     0     va2
..........aaaaguaauugugguuuuug...................................................    4     0     va2
..........aaaaguaauugugguuuuugug.................................................    1     0     va2

.....ggugcaaaaguaauugugguuu......................................................    1     0     j27
.........caaaaguaauugugguuuuu....................................................    1     0     j27
.........caaaaguaauugugguuuuug...................................................    4     0     j27
.........caaaaguaauugugguuuuugu..................................................    2     0     j27
..........aaaaguaauugugguuuu.....................................................    1     0     j27
..........aaaaguaauugugguuuuu....................................................    2     0     j27
..........aaaaguaauugugguuuuug...................................................    13    0     j27

....uggugcaaaaguaauugugguu.......................................................    1     0     j31
.........caaaaguaauugugguuuuu....................................................    1     0     j31
.........caaaaguaauugugguuuuug...................................................    2     0     j31
.........caaaaguaauugugguuuuugu..................................................    3     0     j31
..........aaaaguaauugugguuuuug...................................................    1     0     j31

..........aaaaguaauugugguuuuu....................................................    2     0     H52

.........caaaaguaauugugguuuuu....................................................    1     0     j22
.........caaaaguaauugugguuuuug...................................................    1     0     j22
.........caaaaguaauugugguuuuugu..................................................    3     0     j22
..........aaaaguaauugugguuuuu....................................................    1     0     j22
..........aaaaguaauugugguuuuug...................................................    33    0     j22
..........aaaaguaauugugguuuuugu..................................................    3     0     j22
..........aaaaguaauugugguuuuugug.................................................    2     0     j22
```

```
          Mature                                Star
aggcuggugcaaaaguaauugugguuuuugugauugaaaguaauggcaaaaauugcaauuacuuuugcacuaaccuaaaua
...........aaaguaauugugguuuuug...................................................   3     0     j22

.......ugcaaaaguaauugugguuuuu....................................................   1     0     k09
.........caaaaguaauugugguuuuu....................................................   1     0     k09
.........caaaaguaauugugguuuuug...................................................   2     0     k09
.........caaaaguaauugugguuuuugu..................................................   1     0     k09
..........aaaaguaauugugguuuuu....................................................   2     0     k09
..........aaaaguaauugugguuuuug...................................................   4     0     k09
..........aaaaguaauugugguuuuugu..................................................   2     0     k09

.........caaaaguaauugugguuuuug...................................................   3     0     B14
.........caaaaguaauugugguuuuugu..................................................   1     0     B14

.........caaaaguaauugugguuuuug...................................................   1     0     j16
..........aaaaguaauugugguuuu.....................................................   1     0     j16
..........aaaaguaauugugguuuuu....................................................   12    0     j16
..........aaaaguaauugugguuuuug...................................................   25    0     j16

.........caaaaguaauugugguuuuu....................................................   3     0     j18
.........caaaaguaauugugguuuuug...................................................   1     0     j18
..........aaaaguaauugugguuuuug...................................................   1     0     j18
..........aaaaguaauugugguuuuugu..................................................   1     0     j18

.........caaaaguaauugugguuuuu....................................................   2     0     j09
..........aaaaguaauugugguuuuu....................................................   4     0     j09
..........aaaaguaauugugguuuuug...................................................   9     0     j09
..........aaaaguaauugugguuuuugug.................................................   2     0     j09
...........aaaguaauugugguuuuug...................................................   1     0     j09

....uggugcaaaaguaauuguggu........................................................   1     0     ku3
.........caaaaguaauugugguuuuug...................................................   1     0     ku3
.........caaaaguaauugugguuuuugu..................................................   1     0     ku3
..........aaaaguaauugugguuuuug...................................................   6     0     ku3

.........caaaaguaauugugguuuu.....................................................   1     0     ku6
.........caaaaguaauugugguuuuu....................................................   1     0     ku6
.........caaaaguaauugugguuuuugu..................................................   1     0     ku6
..........aaaaguaauugugguuuuug...................................................   2     0     ku6

..........aaaaguaauugugguuuuu....................................................   4     0     j11
..........aaaaguaauugugguuuuug...................................................   7     0     j11
...........aaaguaauugugguuuuug...................................................   1     0     j11

..........aaaaguaauugugguuuuug...................................................   4     0     s12
...........aaaguaauugugguuuuug...................................................   2     0     s12

........gcaaaaguaauugugguuuuu....................................................   2     0     ya3
.........caaaaguaauugugguuuu.....................................................   9     0     ya3
.........caaaaguaauugugguuuuu....................................................   22    0     ya3
.........caaaaguaauugugguuuuugu..................................................   4     0     ya3
..........aaaaguaauugugguuuu.....................................................   44    0     ya3
..........aaaaguaauugugguuuuu....................................................   238   0     ya3
..........aaaaguaauugugguuuuug...................................................   27    0     ya3
..........aaaaguaauugugguuuuugu..................................................   1     0     ya3
...........aaaguaauugugguuuuu....................................................   45    0     ya3
...........aaaguaauugugguuuuug...................................................   8     0     ya3

aggcuggugcaaaaguaa...............................................................   1     0     la1
.........caaaaguaauugugguuuuug...................................................   1     0     la1
.........caaaaguaauugugguuuuugu..................................................   1     0     la1
..........aaaaguaauugugguuuu.....................................................   1     0     la1
..........aaaaguaauugugguuuuu....................................................   1     0     la1
..........aaaaguaauugugguuuuug...................................................   3     0     la1

....uggugcaaaaguaauugugguu.......................................................   1     0     HEK
.........caaaaguaauugugguuuuugu..................................................   2     0     HEK

..........aaaaguaauugugguuuuu....................................................   1     0     j17
..........aaaaguaauugugguuuuu....................................................   8     0     j17
..........aaaaguaauugugguuuuug...................................................   8     0     j17
```

```
         Mature                                       Star
aggcuggugcaaaaguaauugugguuuuugugauugaaaguaauggcaaaaauugcaauuacuuuugcacuaaccuaaaua

.........caaaaguaauugugguuuuug...................................................    4     0     j15
..........aaaaguaauugugguuuu.....................................................    1     0     j15
..........aaaaguaauugugguuuuu....................................................    3     0     j15
..........aaaaguaauugugguuuuug...................................................    16    0     j15
...........aaaguaauugugguuuuu....................................................    1     0     j15
...........aaaguaauugugguuuuug...................................................    1     0     j15

..........aaaaguaauugugguuuuu....................................................    1     0     la2
..........aaaaguaauugugguuuuug...................................................    3     0     la2

....uggugcaaaaguaauugugguu.......................................................    1     0     j12
.........caaaaguaauugugguuuuug...................................................    5     0     j12
..........aaaaguaauugugguuuuu....................................................    6     0     j12
..........aaaaguaauugugguuuuug...................................................    24    0     j12
..........aaaaguaauugugguuuuugug.................................................    1     0     j12
...........aaaguaauugugguuuuug...................................................    2     0     j12

....uggugcaaaaguaauugugguu.......................................................    1     0     li2
.........caaaaguaauugugguuuuug...................................................    4     0     li2

.........caaaaguaauugugguuuuug...................................................    1     0     va4
..........aaaaguaauugugguuuuu....................................................    1     0     va4
..........aaaaguaauugugguuuuug...................................................    5     0     va4
..........aaaaguaauugugguuuuugu..................................................    1     0     va4

.........caaaaguaauugugguuuuug...................................................    2     0     va3
..........aaaaguaauugugguuuuu....................................................    2     0     va3
..........aaaaguaauugugguuuuug...................................................    10    0     va3
...........aaaguaauugugguuuuug...................................................    2     0     va3

...cuggugcaaaaguaauuguggu........................................................    1     0     SW4
.........caaaaguaauugugguuuuu....................................................    1     0     SW4
.........caaaaguaauugugguuuuugu..................................................    1     0     SW4
..........aaaaguaauugugguuuuu....................................................    1     0     SW4
..........aaaaguaauugugguuuuug...................................................    3     0     SW4
..........aaaaguaauugugguuuuugu..................................................    2     0     SW4

aggcuggugcaaaaguaau..............................................................    1     0     ya4
..gcuggugcaaaaguaauug............................................................    1     0     ya4
.........caaaaguaauugugguuuu.....................................................    16    0     ya4
.........caaaaguaauugugguuuuu....................................................    16    0     ya4
.........caaaaguaauugugguuuuug...................................................    4     0     ya4
..........aaaaguaauugugguuuu.....................................................    33    0     ya4
..........aaaaguaauugugguuuuu....................................................    213   0     ya4
..........aaaaguaauugugguuuuug...................................................    5     0     ya4
..........aaaaguaauugugguuuuugu..................................................    1     0     ya4
...........aaaguaauugugguuuuu....................................................    43    0     ya4
...........aaaguaauugugguuuuug...................................................    3     0     ya4

.........caaaaguaauugugguuuuu....................................................    1     0     ku1
.........caaaaguaauugugguuuuug...................................................    1     0     ku1
.........caaaaguaauugugguuuuugu..................................................    1     0     ku1
..........aaaaguaauugugguuuuu....................................................    5     0     ku1
..........aaaaguaauugugguuuuug...................................................    38    0     ku1
...........aaaguaauugugguuuuug...................................................    1     0     ku1

.......ugcaaaaguaauugugguuuuu....................................................    1     0     j23
.........caaaaguaauugugguuuuug...................................................    4     0     j23
.........caaaaguaauugugguuuuugu..................................................    1     0     j23
..........aaaaguaauugugguuuu.....................................................    1     0     j23
..........aaaaguaauugugguuuuu....................................................    8     0     j23
..........aaaaguaauugugguuuuug...................................................    89    0     j23
..........aaaaguaauugugguuuuugu..................................................    2     0     j23
..........aaaaguaauugugguuuuugug.................................................    4     0     j23
...........aaaguaauugugguuuuug...................................................    11    0     j23

.........caaaaguaauugugguuuuugu..................................................    1     0     s05
..........aaaaguaauugugguuuuug...................................................    7     0     s05
...........aaaguaauugugguuuuug...................................................    1     0     s05
```

Mature **Star**

```
aggcuggugcaaaaguaauugugguuuuugugauugaaaguaauggcaaaaauugcaauuacuuuugcacuaaccuaaaua

...........aaaguaauugugguuuuug...................................................  1   0   ta2

.....ggugcaaaaguaauugugguuu......................................................  5   0   j24
.........caaaaguaauugugguuuu.....................................................  3   0   j24
.........caaaaguaauugugguuuuug...................................................  1   0   j24
.........caaaaguaauugugguuuuugu..................................................  2   0   j24
..........aaaaguaauugugguuuu.....................................................  3   0   j24
..........aaaaguaauugugguuuuug...................................................  8   0   j24

.........caaaaguaauugugguuuuug...................................................  1   0   k11
.........caaaaguaauugugguuuuugu..................................................  2   0   k11
..........aaaaguaauugugguuuu.....................................................  2   0   k11
..........aaaaguaauugugguuuuug...................................................  2   0   k11

..........aaaaguaauugugguuu......................................................  1   0   ta1
..........aaaaguaauugugguuuu.....................................................  1   0   ta1
..........aaaaguaauugugguuuuug...................................................  3   0   ta1
...........aaaguaauugugguuuuug...................................................  1   0   ta1

.........caaaaguaauugugguuuuug...................................................  1   0   s09
..........aaaaguaauugugguuuu.....................................................  1   0   s09
..........aaaaguaauugugguuuuug...................................................  3   0   s09
...........aaaguaauugugguuuuug...................................................  1   0   s09

aggcuggugcaaaaguaau..............................................................  1   0   NL2
.........caaaaguaauugugguu.......................................................  1   0   NL2
.........caaaaguaauugugguuu......................................................  1   0   NL2
.........caaaaguaauugugguuuu.....................................................  4   0   NL2
.........caaaaguaauugugguuuuugu..................................................  1   0   NL2
..........aaaaguaauugugguuu......................................................  10  0   NL2
..........aaaaguaauugugguuuu.....................................................  2   0   NL2
..........aaaaguaauugugguuuuug...................................................  2   0   NL2
..........aaaaguaauugugguuuuugu..................................................  2   0   NL2

....uggugcaaaaguaauugugguu.......................................................  1   0   k10
.........caaaaguaauugugguuuu.....................................................  1   0   k10
.........caaaaguaauugugguuuuug...................................................  2   0   k10
.........caaaaguaauugugguuuuugu..................................................  2   0   k10
..........aaaaguaauugugguuuuug...................................................  4   0   k10

.........caaaaguaauugugguuuuug...................................................  1   0   j13
..........aaaaguaauugugguuu......................................................  1   0   j13
..........aaaaguaauugugguuuu.....................................................  6   0   j13
..........aaaaguaauugugguuuuug...................................................  33  0   j13
...........aaaguaauugugguuuuug...................................................  1   0   j13

.........caaaaguaauugugguuuu.....................................................  2   0   j19
.........caaaaguaauugugguuuuug...................................................  1   0   j19
.........caaaaguaauugugguuuuugu..................................................  1   0   j19
..........aaaaguaauugugguuu......................................................  2   0   j19
..........aaaaguaauugugguuuu.....................................................  5   0   j19
..........aaaaguaauugugguuuuug...................................................  37  0   j19

..........aaaaguaauugugguuuuug...................................................  4   0   j03

....uggugcaaaaguaauugugguu.......................................................  1   0   j25
.......ugcaaaaguaauugugguu.......................................................  2   0   j25
.........caaaaguaauugugguuu......................................................  1   0   j25
..........aaaaguaauugugguuuuug...................................................  10  0   j25
..............................................caaaaauugcaauuacuuu................  1   0   j25

.........caaaaguaauugugguuuuugu..................................................  1   0   k01
..........aaaaguaauugugguuuuug...................................................  1   0   k01
..........aaaaguaauugugguuuuugu..................................................  1   0   k01

aggcuggugcaaaaguaa...............................................................  1   0   per
........gcaaaaguaauugugguuuuug...................................................  1   0   per
.........caaaaguaauugugguuuuug...................................................  1   0   per
.........caaaaguaauugugguuuuugu..................................................  3   0   per
```

```
          Mature                                Star
aggcuggugcaaaaguaauugugguuuuugugauugaaaguaauggcaaaaauugcaauuacuuuugcacuaaccuaaaua
..........aaaaguaauugugguuuu.....................................................    1    0    per
..........aaaaguaauugugguuuuug...................................................    8    0    per
...........aaaguaauugugguuuuug...................................................    1    0    per

........gcaaaaguaauugugguuuuug...................................................    1    0    s01

.........caaaaguaauugugguuuuu....................................................    2    0    k04
.........caaaaguaauugugguuuuug...................................................    1    0    k04
.........caaaaguaauugugguuuuugu..................................................    2    0    k04
..........aaaaguaauugugguuuu.....................................................    1    0    k04
..........aaaaguaauugugguuuuug...................................................    5    0    k04
..........aaaaguaauugugguuuuugu..................................................    1    0    k04

....uggugcaaaaguaauuguggu........................................................    2    0    U20
....uggugcaaaaguaauugugguu.......................................................    1    0    U20
......gugcaaaaguaauugugguuuu.....................................................    1    0    U20
.........caaaaguaauugugguuuuug...................................................    1    0    U20
.........caaaaguaauugugguuuuugu..................................................    1    0    U20
..........aaaaguaauugugguuuuu....................................................    1    0    U20
..........aaaaguaauugugguuuuug...................................................    6    0    U20
..........aaaaguaauugugguuuuugug.................................................    1    0    U20
...........aaaguaauugugguuuuugu..................................................    1    0    U20

.........caaaaguaauugugguuuuu....................................................    2    0    ku2
.........caaaaguaauugugguuuuugu..................................................    2    0    ku2
..........aaaaguaauugugguuuuu....................................................    5    0    ku2
..........aaaaguaauugugguuuuug...................................................    16   0    ku2

.........caaaaguaauugugguuuuu....................................................    1    0    j21
.........caaaaguaauugugguuuuug...................................................    3    0    j21
..........aaaaguaauugugguuuuu....................................................    1    0    j21
..........aaaaguaauugugguuuuug...................................................    14   0    j21
...........aaaguaauugugguuuuug...................................................    3    0    j21

..........aaaaguaauugugguuuuug...................................................    1    0    s11

..........aaaaguaauugugguuuuu....................................................    4    0    j07
..........aaaaguaauugugguuuuug...................................................    2    0    j07
...........aaaguaauugugguuuuug...................................................    1    0    j07

.........caaaaguaauugugguuuuug...................................................    2    0    k02
.........caaaaguaauugugguuuuugu..................................................    1    0    k02
..........aaaaguaauugugguuuuu....................................................    2    0    k02

.........caaaaguaauugugguuuuug...................................................    5    0    j14
..........aaaaguaauugugguuuuug...................................................    9    0    j14
..........aaaaguaauugugguuuuugu..................................................    1    0    j14

.........caaaaguaauugugguuuuu....................................................    1    0    j28
..........aaaaguaauugugguuuuu....................................................    3    0    j28
..........aaaaguaauugugguuuuug...................................................    17   0    j28

..........aaaaguaauugugguuuuug...................................................    1    0    s08

.........caaaaguaauugugguuuuugu..................................................    1    0    DLD
..........aaaaguaauugugguuuuu....................................................    2    0    DLD
..........aaaaguaauugugguuuuug...................................................    1    0    DLD

..........aaaaguaauugugguuuuug...................................................    5    0    k03

aggcuggugcaaaaguaauug............................................................    1    0    NL3
.........caaaaguaauugugguuu......................................................    1    0    NL3
.........caaaaguaauugugguuuu.....................................................    1    0    NL3
.........caaaaguaauugugguuuuu....................................................    4    0    NL3
.........caaaaguaauugugguuuuug...................................................    2    0    NL3
..........aaaaguaauugugguuuu.....................................................    15   0    NL3
..........aaaaguaauugugguuuuu....................................................    6    0    NL3
..........aaaaguaauugugguuuuug...................................................    5    0    NL3

aggcuggugcaaaaguaa...............................................................    1    0    ya2
```

```
         Mature                                  Star
aggcuggugcaaaaguaauugugguuuuugugauugaaaguaauggcaaaaauugcaauuacuuuugcacuaaccuaaaua
aggcuggugcaaaaguaau..............................................................   5     0    ya2
.........caaaaguaauugugguuu......................................................   1     0    ya2
.........caaaaguaauugugguuuu.....................................................   6     0    ya2
.........caaaaguaauugugguuuuu....................................................   13    0    ya2
.........caaaaguaauugugguuuuug...................................................   2     0    ya2
..........aaaaguaauugugguuuu.....................................................   24    0    ya2
..........aaaaguaauugugguuuuu....................................................   188   0    ya2
..........aaaaguaauugugguuuuug...................................................   17    0    ya2
..........aaaaguaauugugguuuuugu..................................................   4     0    ya2
...........aaaguaauugugguuuuu....................................................   43    0    ya2
...........aaaguaauugugguuuuug...................................................   4     0    ya2

.........caaaaguaauugugguuuuug...................................................   1     0    s02

.........caaaaguaauugugguuuuug...................................................   3     0    k06
..........aaaaguaauugugguuuuug...................................................   2     0    k06

........gcaaaaguaauugugguuuuug...................................................   1     0    j29
.........caaaaguaauugugguuuuug...................................................   3     0    j29
.........caaaaguaauugugguuuuugu..................................................   2     0    j29
..........aaaaguaauugugguuuuug...................................................   10    0    j29

......gugcaaaaguaauuguggu........................................................   1     0    sh2
.........caaaaguaauugugguuuuu....................................................   1     0    sh2
..........aaaaguaauugugguuuuug...................................................   1     0    sh2

.....ggugcaaaaguaauugugguu.......................................................   1     0    j01
.......ugcaaaaguaauugugguuu......................................................   3     0    j01
.........caaaaguaauugugguuuuug...................................................   1     0    j01
..........aaaaguaauugugguuuuug...................................................   5     0    j01

....uggugcaaaaguaauuguggu........................................................   1     0    MCF
.........caaaaguaauugugguuuuugu..................................................   1     0    MCF
..........aaaaguaauugugguuuuug...................................................   4     0    MCF

..........aaaaguaauugugguuuuug...................................................   2     0    MBM

..........aaaaguaauugugguuuuu....................................................   2     0    NL1
..........aaaaguaauugugguuuuug...................................................   2     0    NL1

.....ggugcaaaaguaauuguggu........................................................   1     0    k07
.......ugcaaaaguaauugugguuuuu....................................................   1     0    k07
.........caaaaguaauugugguuuuug...................................................   9     0    k07
.........caaaaguaauugugguuuuugu..................................................   3     0    k07
..........aaaaguaauugugguuuuug...................................................   3     0    k07

.......ugcaaaaguaauugugguuuuu....................................................   1     0    sh1
.........caaaaguaauugugguuuuug...................................................   6     0    sh1
..........aaaaguaauugugguuuuug...................................................   4     0    sh1

..gcuggugcaaaaguaauugugg.........................................................   1     0    li1
....uggugcaaaaguaauugugguu.......................................................   1     0    li1
.........caaaaguaauugugguuuuug...................................................   3     0    li1
..........aaaaguaauugugguuuuugug.................................................   2     0    li1

.........caaaaguaauugugguuuuu....................................................   1     0    k13
.........caaaaguaauugugguuuuug...................................................   4     0    k13
.........caaaaguaauugugguuuuugu..................................................   3     0    k13
..........aaaaguaauugugguuuuug...................................................   1     0    k13

.........caaaaguaauugugguuuu.....................................................   1     0    j20
.........caaaaguaauugugguuuuu....................................................   2     0    j20
.........caaaaguaauugugguuuuug...................................................   7     0    j20
..........aaaaguaauugugguuuuu....................................................   3     0    j20
..........aaaaguaauugugguuuuug...................................................   27    0    j20
..........aaaaguaauugugguuuuugug.................................................   1     0    j20

........gcaaaaguaauugugguuuuug...................................................   11    0    A54
.........caaaaguaauugugguuuuugu..................................................   1     0    A54
..........aaaaguaauugugguuuuug...................................................   3     0    A54
```

Mature Star

```
aggcuggugcaaaaguaauugugguuuuugugauugaaaguaauggcaaaaauugcaauuacuuuugcacuaaccuaaaua

....uggugcaaaaguaauugugguu......................................................    1    0    j30
..........aaaaguaauugugguuuuug..................................................    2    0    j30

.........caaaaguaauugugguuuuu...................................................    8    0    k12
.........caaaaguaauugugguuuuug..................................................    12   0    k12
..........aaaaguaauugugguuuuu...................................................    6    0    k12
..........aaaaguaauugugguuuuug..................................................    18   0    k12
..........aaaaguaauugugguuuuugu.................................................    2    0    k12
```

Provisional ID	:	hsa_can_743
Score total	:	265.2
Score for star read(s)	:	3.9
Score for read counts	:	259.6
Score for mfe	:	2.2
Score for randfold	:	
Score for cons. seed	:	-0.6
Total read count	:	521
Mature read count	:	511
Loop read count	:	0
Star read count	:	10

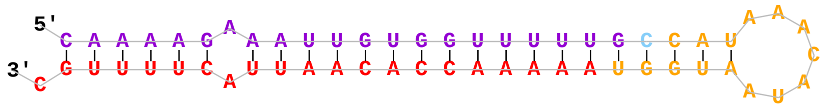

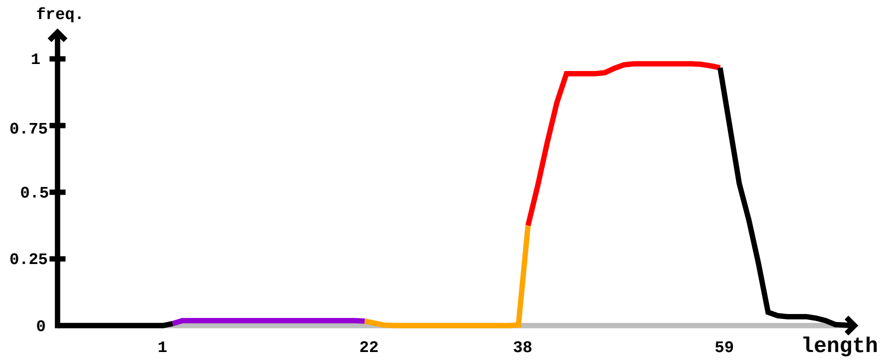


```
           Star                      Mature
5'- uagguuggugcaaaagaaauugugguuuuugccauaaacauaaaugguaaaaaccacaauuacuuuugcaccaaccuaauau  -3'   obs
    uagguuggugcaaaagaaauugugguuuuugccauaaacauaaaugguaaaaaccacaauuacuuuugcaccaaccuaauau        exp
    uagguuggugcaaaagaaauugugguuuuugccauaaacauaaaugguaaaaaccacaauuacuuuugcaccaaccuaauau        known
    ((((((((((((((((.(((((((((((((((((((.......)))))))))))))))))).)))))))))))))))).... reads  mm  sample
    ................................................aaaaaccacaauuacuuuug..............  1     0   ya1
    ................................................aaaaaccacaauuacuuuugca............  4     0   ya1
    .................................................aaaaccacaauuacuuuugca............  3     0   ya1
    .................................................aaaaccacaauuacuuuugcac...........  1     0   ya1
    ..................................................aaaccacaauuacuuuugca............  1     0   ya1
    ...................................................aaccacaauuacuuuugcacc..........  1     0   ya1

    ................................................aaaaaccacaauuacuuuugca............  1     0   s04

    ................................................aaaaaccacaauuacuuuugca............  3     0   j26
    ....................................................accacaauuacuuuugcacca.........  1     0   j26

    ................................................aaaaaccacaauuacuuuugc.............  1     0   va1
    ................................................aaaaaccacaauuacuuuugca............  6     0   va1
    ................................................aaaaaccacaauuacuuuugcac...........  1     0   va1
    .................................................aaaaccacaauuacuuuugcac...........  5     0   va1
    ..................................................aaaccacaauuacuuuugcacc..........  1     0   va1

    ................................................aaaaaccacaauuacuuuugca............  1     0   ku5
    ..................................................aaaccacaauuacuuuugcacc..........  1     0   ku5

    ..........caaaagaaauugugguuuuug...................................................  1     0   k05
    ..................................................aaaccacaauuacuuuugcacc..........  1     0   k05

    ................................................aaaaaccacaauuacuuuugca............  1     0   s06
    ...................................................aaccacaauuacuuuugcacca.........  1     0   s06
    ....................................................accacaauuacuuuugcacca.........  1     0   s06

    ................................................aaaaaccacaauuacuuuugc.............  1     0   HeL
    ................................................aaaaaccacaauuacuuuugca............  3     0   HeL
    .................................................aaaaccacaauuacuuuugcac...........  2     0   HeL
    ..................................................aaaccacaauuacuuuugcac...........  1     0   HeL
```

Star **Mature**

uagguuggugcaaaagaaauugugguuuuugccauaaacauaaagguaaaaaccacaauuacuuuugcaccaaccuaauau			
....aaaccacaauuacuuuugcacc..........	3	0	HeL
....aaccacaauuacuuuugcacca.........	3	0	HeL
....aaccacaauuacuuuugcaccaa........	3	0	HeL
....accacaauuacuuuugcacca.........	5	0	HeL
....aaaccacaauuacuuuugcacc..........	1	0	s07
....aaaaaccacaauuacuuuugca............	4	0	s10
....aaaccacaauuacuuuugcac...........	1	0	s10
....aaaaaccacaauuacuuuugca............	4	0	ta3
....aaaaccacaauuacuuuugc.............	1	0	ta3
....aaaaccacaauuacuuuugcac...........	1	0	ta3
....aaaccacaauuacuuuugcac...........	1	0	ta3
....aaaccacaauuacuuuugcacc..........	1	0	ta3
....aaccacaauuacuuuugcacca.........	2	0	ta3
....aaaaaccacaauuacuuuugca............	5	0	j10
....aaaccacaauuacuuuugcac...........	1	0	j10
....aaccacaauuacuuuugcacca.........	1	0	j10
....aaccacaauuacuuuugcac...........	1	0	k14
....aaaaaccacaauuacuuuugcac...........	1	0	va2
....caauuacuuuugcaccaaccuaa...	1	0	va2
....aaaccacaauuacuuuugca............	1	0	j27
....aaaaaccacaauuacuuuugca............	1	0	j31
....aaaaaccacaauuacuuuugca............	2	0	s03
....aaaaccacaauuacuuuugca............	1	0	s03
....aaaaccacaauuacuuuugcac...........	2	0	s03
....aaaaaccacaauuacuuuug..............	2	0	H52
....aaaaaccacaauuacuuuugca............	1	0	H52
....aaaaccacaauuacuuuugc.............	1	0	H52
....aaaaccacaauuacuuuugca............	2	0	H52
....aaaccacaauuacuuuugcacc..........	2	0	H52
....aaccacaauuacuuuugcacc..........	1	0	H52
....aaccacaauuacuuuugcacca.........	1	0	H52
....accacaauuacuuuugcacca.........	1	0	H52
....aaaaaccacaauuacuuuugca............	6	0	j22
....aaaaccacaauuacuuuugca............	1	0	j22
....aaccacaauuacuuuugcacc..........	1	0	j22
....aaaaccacaauuacuuuugcac...........	1	0	k09
....aaaccacaauuacuuuugcaccaa........	1	0	k09
....aaaaaccacaauuacuuuugc.............	3	0	B14
....aaaaaccacaauuacuuuugca............	6	0	B14
....aaaaccacaauuacuuuugcac...........	1	0	B14
....aaaccacaauuacuuuugca............	2	0	B14
....aaaccacaauuacuuuugcacc..........	2	0	B14
....accacaauuacuuuugcacca.........	2	0	B14
....aaaaaccacaauuacuuuugcac...........	1	0	ku3
....aaaccacaauuacuuuugcacca.........	1	0	ku3
....aaaaaccacaauuacuuuugca............	1	0	j09
....accacaauuacuuuugcacca.........	1	0	j09
....aaaaaccacaauuacuuuugca............	4	0	j16
....aaaaaccacaauuacuuuugca............	2	0	ku6
...........aaaagaaauugugguuuuug..	1	0	j11
....aaaaaccacaauuacuuuugca............	2	0	j11
....aaaaaccacaauuacuuuugca............	3	0	ya3

Star **Mature**

```
uagguuggugcaaaagaaauugugguuuuugccauaaacauaauggguaaaaaccacaauuacuuuugcaccaaccuaauau
................................................aaaaccacaauuacuuuugca............   2   0   ya3
................................................aaaaccacaauuacuuuugcac...........   5   0   ya3
.................................................aaaccacaauuacuuuugca............   1   0   ya3
.................................................aaaccacaauuacuuuugcacc..........   1   0   ya3
..................................................aaccacaauuacuuuugcacc..........   2   0   ya3

...............................................aaaaaccacaauuacuuuugca............   2   0   la1
..................................................aaccacaauuacuuuugcacc..........   1   0   la1

...............................................aaaaaccacaauuacuuuugc.............   1   0   HEK
...............................................aaaaaccacaauuacuuuugca............   8   0   HEK
................................................aaaaccacaauuacuuuugca............   2   0   HEK
................................................aaaaccacaauuacuuuugcac...........   3   0   HEK
.................................................aaaccacaauuacuuuugca............   1   0   HEK
.................................................aaaccacaauuacuuuugcac...........   3   0   HEK
.................................................aaaccacaauuacuuuugcacc..........   3   0   HEK
..................................................aaccacaauuacuuuugcacc..........   2   0   HEK
..................................................aaccacaauuacuuuugcacca.........   4   0   HEK
...................................................accacaauuacuuuugcacca.........   6   0   HEK

................................................aaaaccacaauuacuuuugca............   1   0   j17
..................................................aaccacaauuacuuuugcacca.........   2   0   j17
...................................................accacaauuacuuuugcacca.........   1   0   j17

................................................aaaaccacaauuacuuuugca............   1   0   j15

................................................aaaaccacaauuacuuuugcac...........   1   0   la2
.................................................aaaccacaauuacuuuugca............   1   0   la2
.................................................aaaccacaauuacuuuugcacc..........   1   0   la2
..................................................aaccacaauuacuuuugcacca.........   1   0   la2

...................................................accacaauuacuuuugcacca.........   1   0   j12

...........aaaagaaauugugguuuuugc.................................................   1   0   li2

...............................................aaaaaccacaauuacuuuugca............   3   0   va4
...............................................aaaaaccacaauuacuuuugcac...........   1   0   va4
................................................aaaaccacaauuacuuuugcac...........   1   0   va4
..................................................aaccacaauuacuuuugcacc..........   2   0   va4
...................................................accacaauuacuuuugcacca.........   4   0   va4

...........aaaagaaauugugguuuuugcc................................................   1   0   va3
..............................................uaaaaaccacaauuacuuuugca............   1   0   va3
...............................................aaaaaccacaauuacuuuugca............   12  0   va3
................................................aaaaccacaauuacuuuugcac...........   2   0   va3
.................................................aaaccacaauuacuuuugcac...........   1   0   va3
.................................................aaaccacaauuacuuuugcacc..........   4   0   va3
..................................................aaccacaauuacuuuugcacc..........   2   0   va3
..................................................aaccacaauuacuuuugcacca.........   2   0   va3
...................................................accacaauuacuuuugcacca.........   1   0   va3

...............................................aaaaaccacaauuacuuuugca............   4   0   ya4
................................................aaaaccacaauuacuuuugca............   2   0   ya4
................................................aaaaccacaauuacuuuugcac...........   4   0   ya4
.................................................aaaccacaauuacuuuugcac...........   1   0   ya4
.................................................aaaccacaauuacuuuugcacc..........   4   0   ya4
..................................................aaccacaauuacuuuugca............   3   0   ya4
..................................................aaccacaauuacuuuugcacc..........   4   0   ya4
..................................................aaccacaauuacuuuugcacca.........   2   0   ya4

...............................................aaaaaccacaauuacuuuugca............   1   0   SW4
................................................aaaaccacaauuacuuuugcac...........   1   0   SW4
.................................................aaaccacaauuacuuuugcac...........   2   0   SW4
.................................................aaaccacaauuacuuuugcacc..........   3   0   SW4
..................................................aaccacaauuacuuuugcacca.........   3   0   SW4
...................................................accacaauuacuuuugcacc..........   1   0   SW4
...................................................accacaauuacuuuugcacca.........   3   0   SW4

...............................................aaaaaccacaauuacuuuugca............   4   0   ku1
..................................................aaccacaauuacuuuugcacc..........   1   0   ku1
```

Star Mature

```
uagguuggugcaaaagaaauugugguuuuugccauaaacauaaugguaaaaaccacaauuacuuuugcaccaaccuaauau

..........caaaagaaauugugguuuuugc.................................................	1	0	j23
...............................................aaaaaccacaauuacuuuugc.............	1	0	j23
...............................................aaaaaccacaauuacuuuugca............	2	0	j23
................................................aaaaccacaauuacuuuugca............	1	0	j23
..................................................aaccacaauuacuuuugcacca.........	1	0	j23
...................................................accacaauuacuuuugcacca.........	1	0	j23

...............................................aaaaaccacaauuacuuuugca............	2	0	s05

...............................................aaaaaccacaauuacuuu................	1	0	ta2
................................................aaaaccacaauuacuuuugcac...........	1	0	ta2

...............................................aaaaaccacaauuacuuuugca............	1	0	k11

...........aaaagaaauugugguuuuug..................................................	1	0	ta1
...............................................aaaaaccacaauuacuuuugca............	1	0	ta1

................................................aaaaccacaauuacuuuugca............	1	0	s09
..................................................aaccacaauuacuuuugcacca.........	1	0	s09
...................................................accacaauuacuuuugcacca.........	1	0	s09

...............................................aaaaaccacaauuacuuuugca............	3	0	NL2
..................................................aaccacaauuacuuuugcacc..........	1	0	NL2
..................................................aaccacaauuacuuuugcacca.........	5	0	NL2

...........aaaagaaauugugguuuuug..................................................	1	0	j13
...............................................aaaaaccacaauuacuuuugca............	4	0	j13
................................................aaaaccacaauuacuuuugca............	2	0	j13
..................................................aaccacaauuacuuuugcacca.........	2	0	j13

...............................................aaaaaccacaauuacuuuugca............	2	0	j19
.................................................aaaccacaauuacuuuugca............	2	0	j19
..................................................aaccacaauuacuuuugcacca.........	1	0	j19
...................................................accacaauuacuuuugcacca.........	2	0	j19

...............................................aaaaaccacaauuacuuuugca............	1	0	j25

........................................................aauuacuuuugcaccaaccua....	2	0	per

...............................................aaaaaccacaauuacuuuugca............	2	0	s01
................................................aaaaccacaauuacuuuugcac...........	2	0	s01
.................................................aaaccacaauuacuuuugcacc..........	2	0	s01

................................................aaaaccacaauuacuuuugca............	1	0	k04
.........................................................auuacuuuugcaccaaccuaau..	1	0	k04

...............................................aaaaaccacaauuacuuuugc.............	22	0	U20
...............................................aaaaaccacaauuacuuuugca............	17	0	U20
................................................aaaaccacaauuacuuuugca............	1	0	U20
................................................aaaaccacaauuacuuuugcac...........	8	0	U20
.................................................aaaccacaauuacuuuugcac...........	6	0	U20
.................................................aaaccacaauuacuuuugcacc..........	14	0	U20
..................................................aaccacaauuacuuuugcacc..........	6	0	U20
..................................................aaccacaauuacuuuugcacca.........	1	0	U20
...................................................accacaauuacuuuugcacca.........	15	0	U20
...................................................accacaauuacuuuugcaccaa........	1	0	U20

...............................................aaaaaccacaauuacuuuugca............	3	0	j21
................................................aaaaccacaauuacuuuugca............	1	0	j21
.................................................aaaccacaauuacuuuugcacc..........	1	0	j21


..................................................aaccacaauuacuuuugcacc..........	1	0	s11
..................................................aaccacaauuacuuuugcacca.........	1	0	s11

...............................................aaaaaccacaauuacuuuugca............	1	0	j07

..........caaaagaaauugugguuuuug..................................................	2	0	j14
.................................................aaaccacaauuacuuuugcacc..........	1	0	j14
```

```
      Star                    Mature
uagguuggugcaaaagaaauugugguuuuugccauaaacauaaugguaaaaaccacaauuacuuuugcaccaaccuaauau

...............................................aaaaaccacaauuacuuuugca............    1    0    s08
...................................................accacaauuacuuuugcac...........    1    0    s08

...............................................aaaaaccacaauuacuuuugc.............    1    0    DLD
................................................aaaaccacaauuacuuuugcac...........    1    0    DLD
.................................................aaaccacaauuacuuuugcacc..........    3    0    DLD
...................................................accacaauuacuuuugcacc..........    1    0    DLD
...................................................accacaauuacuuuugcacca.........    4    0    DLD

...............................................aaaaaccacaauuacuuuugca............    1    0    k03

...........aaaagaaauugugguuuuu...................................................    1    0    ya2
...............................................aaaaaccacaauuacuuuu...............    2    0    ya2
...............................................aaaaaccacaauuacuuuugc.............    1    0    ya2
...............................................aaaaaccacaauuacuuuugca............    2    0    ya2
................................................aaaaccacaauuacuuuugca............    6    0    ya2
.................................................aaaccacaauuacuuuugcac...........    3    0    ya2
.................................................aaaccacaauuacuuuugcacca.........    1    0    ya2
..................................................aaccacaauuacuuuugcacca.........    2    0    ya2
.......................................................caauuacuuuugcaccaac.......    1    0    ya2
........................................................aauuacuuuugcaccaac.......    1    0    ya2
.........................................................auuacuuuugcaccaaccuaauau    1    0    ya2

...............................................aaaaaccacaauuacuuuugca............    7    0    NL3
................................................aaaaccacaauuacuuuugcac...........    1    0    NL3
..................................................aaccacaauuacuuuugcacca.........    1    0    NL3
.........................................................auuacuuuugcaccaaccuaa...    1    0    NL3

.................................................aaaccacaauuacuuuugcacc..........    1    0    k06

..................................................aaccacaauuacuuuugcaccaa........    2    0    sh2
..........................................................uuacuuuugcaccaaccua....    1    0    sh2

...............................................aaaaaccacaauuacuuuugca............    1    0    j29
................................................aaaaccacaauuacuuuugca............    1    0    j29

...............................................aaaaaccacaauuacuuuu...............    1    0    MCF
...............................................aaaaaccacaauuacuuuugc.............    1    0    MCF
...............................................aaaaaccacaauuacuuuugca............    2    0    MCF
................................................aaaaccacaauuacuuuugcac...........    1    0    MCF
........................................................aauuacuuuugcaccaaccuaa...    2    0    MCF
........................................................aauuacuuuugcaccaaccuaau..    3    0    MCF
.........................................................auuacuuuugcaccaaccuaau..    4    0    MCF

...............................................aaaaaccacaauuacuuuug..............    1    0    MBM
...............................................aaaaaccacaauuacuuuugc.............    1    0    MBM
...............................................aaaaaccacaauuacuuuugca............    2    0    MBM
................................................aaaaccacaauuacuuuugca............    1    0    MBM
................................................aaaaccacaauuacuuuugcac...........    3    0    MBM
.................................................aaaccacaauuacuuuugcacc..........    4    0    MBM
..................................................aaccacaauuacuuuugcacc..........    1    0    MBM
...................................................accacaauuacuuuugcacca.........    4    0    MBM

...............................................aaaaaccacaauuacuuuugc.............    1    0    li1
................................................aaaaccacaauuacuuuugcac...........    1    0    li1
.................................................aaaccacaauuacuuuugcacc..........    2    0    li1
..................................................aaccacaauuacuuuugcacca.........    1    0    li1

........................................................aauuacuuuugcaccaaccuaa...    1    0    k13
..........................................................uuacuuuugcaccaaccuaaua.    1    0    k13

...............................................aaaaaccacaauuacuuuugc.............    7    0    A54
..................................................aaccacaauuacuuuugcacc..........    4    0    A54
..................................................aaccacaauuacuuuugcacca.........    4    0    A54

...................................................accacaauuacuuuugcacca.........    1    0    k12
```

Provisional ID	:	hsa_can_845
Score total	:	3.1
Score for star read(s)	:	-1.3
Score for read counts	:	0
Score for mfe	:	1.4
Score for randfold	:	
Score for cons. seed	:	3
Total read count	:	2396
Mature read count	:	2396
Loop read count	:	0
Star read count	:	0

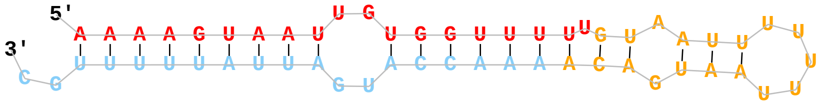

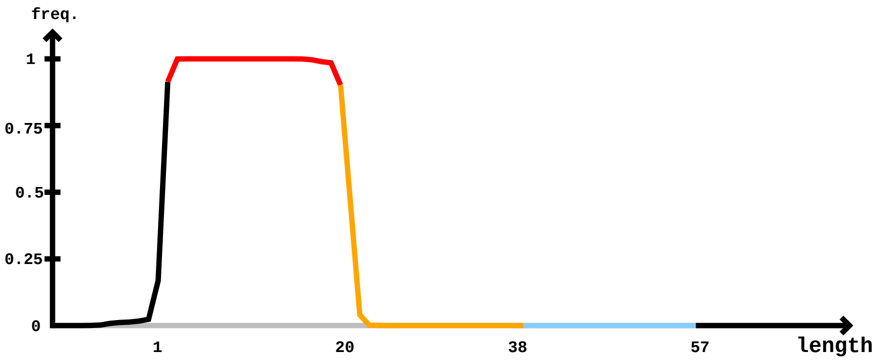

Mature **Star**

```
5'- aaguuggugcaaaaguaauugugguuuuuguaauuuuuuuaaugacaaaaccaugauuauuuuugcaccaaccuaauauca  -3'   exp
    ..((((((((((((((((..(((((((.((.(((.....))).)))))))))..))))))))))))))))...........    reads    mm     sample
    .........caaaaguaauugugguuuu.....................................................    5        0      ya1
    .........caaaaguaauugugguuuuu....................................................    22       0      ya1
    .........caaaaguaauugugguuuuug...................................................    1        0      ya1
    .........caaaaguaauugugguuuuugu..................................................    1        0      ya1
    ..........aaaaguaauugugguuuu.....................................................    16       0      ya1
    ..........aaaaguaauugugguuuuu....................................................    111      0      ya1
    ..........aaaaguaauugugguuuuug...................................................    8        0      ya1
    ...........aaaguaauugugguuuuu....................................................    19       0      ya1
    ...........aaaguaauugugguuuuugu..................................................    1        0      ya1

    ..........aaaaguaauugugguuuuug...................................................    1        0      s04

    .........caaaaguaauugugguuuuugu..................................................    1        0      j26
    ..........aaaaguaauugugguuuuug...................................................    1        0      j26

    ....uggugcaaaaguaauugugguu.......................................................    1        0      va1
    ..........aaaaguaauugugguuuuu....................................................    2        0      va1
    ..........aaaaguaauugugguuuuug...................................................    25       0      va1
    ..........aaaaguaauugugguuuuugu..................................................    2        0      va1
    ...........aaaguaauugugguuuuug...................................................    2        0      va1

    ...uuggugcaaaaguaauugugguu.......................................................    1        0      ku5
    ....uggugcaaaaguaauuguggu........................................................    1        0      ku5
    ......gugcaaaaguaauugugguu.......................................................    1        0      ku5
    .........caaaaguaauugugguuuuug...................................................    1        0      ku5
    ..........aaaaguaauugugguuuuug...................................................    3        0      ku5

    .........caaaaguaauugugguuuuu....................................................    2        0      k05
    .........caaaaguaauugugguuuuug...................................................    2        0      k05
    .........caaaaguaauugugguuuuugu..................................................    3        0      k05
    ..........aaaaguaauugugguuuuug...................................................    3        0      k05
    ...........aaaguaauugugguuuuug...................................................    1        0      k05

    .........caaaaguaauugugguuuuug...................................................    1        0      s06
```

```
          Mature                    Star
aaguuggugcaaaaguaauugugguuuuuguaauuuuuuuaaugacaaaaccaugauuauuuuugcaccaaccuaauauca

..........aaaaguaauugugguuuuugu..................................................   1    0    HeL

......gugcaaaaguaauugugguu.......................................................   1    0    ku4
..........aaaaguaauugugguuuuu....................................................   5    0    ku4
..........aaaaguaauugugguuuuug...................................................   11   0    ku4

..........aaaaguaauugugguuuuug...................................................   2    0    s10
..........aaaaguaauugugguuuuugu..................................................   1    0    s10

.........caaaaguaauugugguuuuu....................................................   2    0    k08
.........caaaaguaauugugguuuuug...................................................   1    0    k08
.........caaaaguaauugugguuuuugu..................................................   2    0    k08
..........aaaaguaauugugguuuuu....................................................   1    0    k08
..........aaaaguaauugugguuuuug...................................................   1    0    k08
..........aaaaguaauugugguuuuugua.................................................   1    0    k08

..........aaaaguaauugugguuuu.....................................................   1    0    ta3
..........aaaaguaauugugguuuuug...................................................   7    0    ta3
...........aaaguaauugugguuuuug...................................................   1    0    ta3

..........aaaaguaauugugguuuuu....................................................   2    0    j10
..........aaaaguaauugugguuuuug...................................................   6    0    j10

..........aaaaguaauugugguuuuug...................................................   3    0    j04

..........aaaaguaauugugguuuu.....................................................   1    0    j02
..........aaaaguaauugugguuuuu....................................................   5    0    j02
..........aaaaguaauugugguuuuug...................................................   22   0    j02
..........aaaaguaauugugguuuuugu..................................................   2    0    j02
...........aaaguaauugugguuuuug...................................................   1    0    j02

.........caaaaguaauugugguuuuu....................................................   1    0    va2
.........caaaaguaauugugguuuuugu..................................................   1    0    va2
..........aaaaguaauugugguuuuu....................................................   1    0    va2
..........aaaaguaauugugguuuuug...................................................   4    0    va2

.........caaaaguaauugugguuuuu....................................................   2    0    k14
.........caaaaguaauugugguuuuug...................................................   4    0    k14
.........caaaaguaauugugguuuuugu..................................................   1    0    k14
..........aaaaguaauugugguuuuu....................................................   2    0    k14
..........aaaaguaauugugguuuuug...................................................   4    0    k14
..........aaaaguaauugugguuuuugu..................................................   1    0    k14
...........aaaguaauugugguuuuug...................................................   1    0    k14
............aaguaauugugguuuuugu..................................................   1    0    k14

.....ggugcaaaaguaauugugguu.......................................................   1    0    j27
.........caaaaguaauugugguuuuu....................................................   1    0    j27
.........caaaaguaauugugguuuuug...................................................   4    0    j27
.........caaaaguaauugugguuuuugu..................................................   2    0    j27
..........aaaaguaauugugguuuu.....................................................   1    0    j27
..........aaaaguaauugugguuuuu....................................................   2    0    j27
..........aaaaguaauugugguuuuug...................................................   13   0    j27

...uuggugcaaaaguaauuguggu........................................................   1    0    j31
....uggugcaaaaguaauugugguu.......................................................   1    0    j31
.........caaaaguaauugugguuuuu....................................................   1    0    j31
.........caaaaguaauugugguuuuug...................................................   2    0    j31
.........caaaaguaauugugguuuuugu..................................................   3    0    j31
..........aaaaguaauugugguuuuug...................................................   1    0    j31

..........aaaaguaauugugguuuuu....................................................   2    0    H52

.........caaaaguaauugugguuuuu....................................................   1    0    j22
.........caaaaguaauugugguuuuug...................................................   1    0    j22
.........caaaaguaauugugguuuuugu..................................................   3    0    j22
..........aaaaguaauugugguuuuu....................................................   1    0    j22
..........aaaaguaauugugguuuuug...................................................   33   0    j22
..........aaaaguaauugugguuuuugu..................................................   3    0    j22
..........aaaaguaauugugguuuuuguaa................................................   1    0    j22
```

```
          Mature                                  Star
aaguuggugcaaaaguaauuugugguuuuuguaauuuuuuuaaugacaaaaccaugauuauuuuugcaccaaccuaauauca
...........aaaguaauuugugguuuuug...................................................    3      0      j22

...uuggugcaaaaguaauuugugg.........................................................    1      0      k09
.......ugcaaaaguaauuugugguuuuu....................................................    1      0      k09
.........caaaaguaauuugugguuuuu....................................................    1      0      k09
.........caaaaguaauuugugguuuuug...................................................    2      0      k09
.........caaaaguaauuugugguuuuugu..................................................    1      0      k09
..........aaaaguaauuugugguuuuu....................................................    2      0      k09
..........aaaaguaauuugugguuuuug...................................................    4      0      k09
..........aaaaguaauuugugguuuuugu..................................................    2      0      k09

.........caaaaguaauuugugguuuuug...................................................    3      0      B14
.........caaaaguaauuugugguuuuugu..................................................    1      0      B14

.........caaaaguaauuugugguuuuug...................................................    1      0      j16
..........aaaaguaauuugugguuuu.....................................................    1      0      j16
..........aaaaguaauuugugguuuuu....................................................    12     0      j16
..........aaaaguaauuugugguuuuug...................................................    25     0      j16

.........caaaaguaauuugugguuuuu....................................................    3      0      j18
.........caaaaguaauuugugguuuuug...................................................    1      0      j18
..........aaaaguaauuugugguuuuug...................................................    1      0      j18
..........aaaaguaauuugugguuuuugu..................................................    1      0      j18

.........caaaaguaauuugugguuuuu....................................................    2      0      j09
..........aaaaguaauuugugguuuuu....................................................    4      0      j09
..........aaaaguaauuugugguuuuug...................................................    9      0      j09
...........aaaguaauuugugguuuuug...................................................    1      0      j09

....uggugcaaaaguaauuuguggu........................................................    1      0      ku3
.........caaaaguaauuugugguuuuug...................................................    1      0      ku3
.........caaaaguaauuugugguuuuugu..................................................    1      0      ku3
..........aaaaguaauuugugguuuuug...................................................    6      0      ku3

.........caaaaguaauuugugguuuu.....................................................    1      0      ku6
.........caaaaguaauuugugguuuuu....................................................    1      0      ku6
.........caaaaguaauuugugguuuuugu..................................................    1      0      ku6
..........aaaaguaauuugugguuuuug...................................................    2      0      ku6

..........aaaaguaauuugugguuuuu....................................................    4      0      j11
..........aaaaguaauuugugguuuuug...................................................    7      0      j11
...........aaaguaauuugugguuuuug...................................................    1      0      j11

..........aaaaguaauuugugguuuuug...................................................    4      0      s12
...........aaaguaauuugugguuuuug...................................................    2      0      s12

........gcaaaaguaauuugugguuuuu....................................................    2      0      ya3
.........caaaaguaauuugugguuuu.....................................................    9      0      ya3
.........caaaaguaauuugugguuuuu....................................................    22     0      ya3
.........caaaaguaauuugugguuuuugu..................................................    4      0      ya3
..........aaaaguaauuugugguuuu.....................................................    44     0      ya3
..........aaaaguaauuugugguuuuu....................................................    238    0      ya3
..........aaaaguaauuugugguuuuug...................................................    27     0      ya3
..........aaaaguaauuugugguuuuugu..................................................    1      0      ya3
...........aaaguaauuugugguuuuu....................................................    45     0      ya3
...........aaaguaauuugugguuuuug...................................................    8      0      ya3

.........caaaaguaauuugugguuuuu....................................................    1      0      la1
.........caaaaguaauuugugguuuuugu..................................................    1      0      la1
..........aaaaguaauuugugguuuu.....................................................    1      0      la1
..........aaaaguaauuugugguuuuu....................................................    1      0      la1
..........aaaaguaauuugugguuuuug...................................................    3      0      la1

....uggugcaaaaguaauuugugguu.......................................................    1      0      HEK
.........caaaaguaauuugugguuuuugu..................................................    2      0      HEK

..........aaaaguaauuugugguuuu.....................................................    1      0      j17
..........aaaaguaauuugugguuuuu....................................................    8      0      j17
..........aaaaguaauuugugguuuuug...................................................    8      0      j17

.........caaaaguaauuugugguuuuug...................................................    4      0      j15
```

Mature Star

aaguuggugcaaaaguaauugugguuuuuguaauuuuuuuaaugacaaaaccaugauuauuuuugcaccaaccuaauauca			
..........aaaaguaauugugguuuu...	1	0	j15
..........aaaaguaauugugguuuuu..	3	0	j15
..........aaaaguaauugugguuuuug...	16	0	j15
...........aaaguaauugugguuuuu..	1	0	j15
...........aaaguaauugugguuuuug...	1	0	j15
..........aaaaguaauugugguuuuu..	1	0	la2
..........aaaaguaauugugguuuuug...	3	0	la2
....uggugcaaaaguaauugugguu...	1	0	j12
.........caaaaguaauugugguuuuug...	5	0	j12
..........aaaaguaauugugguuuuu..	6	0	j12
..........aaaaguaauugugguuuuug...	24	0	j12
...........aaaguaauugugguuuuug...	2	0	j12
....uggugcaaaaguaauugugguu...	1	0	li2
.........caaaaguaauugugguuuuug...	4	0	li2
.........caaaaguaauugugguuuuug...	1	0	va4
..........aaaaguaauugugguuuuu..	1	0	va4
..........aaaaguaauugugguuuuug...	5	0	va4
..........aaaaguaauugugguuuuugu..	1	0	va4
.........caaaaguaauugugguuuuug...	2	0	va3
..........aaaaguaauugugguuuuu..	2	0	va3
..........aaaaguaauugugguuuuug...	10	0	va3
...........aaaguaauugugguuuuug...	2	0	va3
.........caaaaguaauugugguuuuu..	1	0	SW4
.........caaaaguaauugugguuuuugu..	1	0	SW4
..........aaaaguaauugugguuuuu..	1	0	SW4
..........aaaaguaauugugguuuuug...	3	0	SW4
..........aaaaguaauugugguuuuugu..	2	0	SW4
.........caaaaguaauugugguuuu...	16	0	ya4
.........caaaaguaauugugguuuuu..	16	0	ya4
.........caaaaguaauugugguuuuug...	4	0	ya4
..........aaaaguaauugugguuuu...	33	0	ya4
..........aaaaguaauugugguuuuu..	213	0	ya4
..........aaaaguaauugugguuuuug...	5	0	ya4
..........aaaaguaauugugguuuuugu..	1	0	ya4
..........aaaaguaauugugguuuuugua...	1	0	ya4
...........aaaguaauugugguuuuu..	43	0	ya4
...........aaaguaauugugguuuuug...	3	0	ya4
.........caaaaguaauugugguuuuu..	1	0	ku1
.........caaaaguaauugugguuuuug...	1	0	ku1
.........caaaaguaauugugguuuuugu..	1	0	ku1
..........aaaaguaauugugguuuuu..	5	0	ku1
..........aaaaguaauugugguuuuug...	38	0	ku1
...........aaaguaauugugguuuuug...	1	0	ku1
.......ugcaaaaguaauugugguuuuu..	1	0	j23
.........caaaaguaauugugguuuuug...	4	0	j23
.........caaaaguaauugugguuuuugu..	1	0	j23
..........aaaaguaauugugguuuu...	1	0	j23
..........aaaaguaauugugguuuuu..	8	0	j23
..........aaaaguaauugugguuuuug...	89	0	j23
..........aaaaguaauugugguuuuugu..	2	0	j23
...........aaaguaauugugguuuuug...	11	0	j23
.........caaaaguaauugugguuuuugu..	1	0	s05
..........aaaaguaauugugguuuuug...	7	0	s05
...........aaaguaauugugguuuuug...	1	0	s05
...........aaaguaauugugguuuuug...	1	0	ta2
.....ggugcaaaaguaauugugguuu..	5	0	j24
.........caaaaguaauugugguuuu...	3	0	j24
.........caaaaguaauugugguuuuug...	1	0	j24

```
                Mature                   Star
aaguuggugcaaaaguaauugugguuuuuguaauuuuuuuaaugacaaaaccaugauuauuuuugcaccaaccuaauauca
.........caaaaguaauugugguuuuugu..................................................    2    0    j24
..........aaaaguaauugugguuuuu....................................................    3    0    j24
..........aaaaguaauugugguuuuug...................................................    8    0    j24

.........caaaaguaauugugguuuuug...................................................    1    0    k11
.........caaaaguaauugugguuuuugu..................................................    2    0    k11
..........aaaaguaauugugguuuuu....................................................    2    0    k11
..........aaaaguaauugugguuuuug...................................................    2    0    k11

..........aaaaguaauugugguuuu.....................................................    1    0    ta1
..........aaaaguaauugugguuuuu....................................................    1    0    ta1
..........aaaaguaauugugguuuuug...................................................    3    0    ta1
...........aaaguaauugugguuuuug...................................................    1    0    ta1

.........caaaaguaauugugguuuuug...................................................    1    0    s09
..........aaaaguaauugugguuuuu....................................................    1    0    s09
..........aaaaguaauugugguuuuug...................................................    3    0    s09
...........aaaguaauugugguuuuug...................................................    1    0    s09

.........caaaaguaauugugguuu......................................................    1    0    NL2
.........caaaaguaauugugguuuu.....................................................    1    0    NL2
.........caaaaguaauugugguuuuu....................................................    4    0    NL2
.........caaaaguaauugugguuuuugu..................................................    1    0    NL2
..........aaaaguaauugugguuuu.....................................................    10   0    NL2
..........aaaaguaauugugguuuuu....................................................    2    0    NL2
..........aaaaguaauugugguuuuug...................................................    2    0    NL2
..........aaaaguaauugugguuuuugu..................................................    2    0    NL2

....uggugcaaaaguaauuguggu........................................................    1    0    k10
.........caaaaguaauugugguuuuu....................................................    1    0    k10
.........caaaaguaauugugguuuuug...................................................    2    0    k10
.........caaaaguaauugugguuuuugu..................................................    2    0    k10
..........aaaaguaauugugguuuuug...................................................    4    0    k10

.........caaaaguaauugugguuuuug...................................................    1    0    j13
..........aaaaguaauugugguuuu.....................................................    1    0    j13
..........aaaaguaauugugguuuuu....................................................    6    0    j13
..........aaaaguaauugugguuuuug...................................................    33   0    j13
...........aaaguaauugugguuuuug...................................................    1    0    j13

.........caaaaguaauugugguuuuu....................................................    2    0    j19
.........caaaaguaauugugguuuuug...................................................    1    0    j19
.........caaaaguaauugugguuuuugu..................................................    1    0    j19
..........aaaaguaauugugguuuu.....................................................    2    0    j19
..........aaaaguaauugugguuuuu....................................................    5    0    j19
..........aaaaguaauugugguuuuug...................................................    37   0    j19

..........aaaaguaauugugguuuuug...................................................    4    0    j03

.........caaaaguaauugugguuuuugu..................................................    1    0    k01
..........aaaaguaauugugguuuuug...................................................    1    0    k01
..........aaaaguaauugugguuuuugu..................................................    1    0    k01

....uggugcaaaaguaauuguggu........................................................    1    0    j25
.......ugcaaaaguaauuguggu........................................................    2    0    j25
.........caaaaguaauugugguuuu.....................................................    1    0    j25
..........aaaaguaauugugguuuuug...................................................    10   0    j25

........gcaaaaguaauugugguuuuug...................................................    1    0    per
.........caaaaguaauugugguuuuug...................................................    1    0    per
.........caaaaguaauugugguuuuugu..................................................    3    0    per
..........aaaaguaauugugguuuu.....................................................    1    0    per
..........aaaaguaauugugguuuuug...................................................    8    0    per
...........aaaguaauugugguuuuug...................................................    1    0    per

........gcaaaaguaauugugguuuuug...................................................    1    0    s01

.........caaaaguaauugugguuuuu....................................................    2    0    k04
.........caaaaguaauugugguuuuug...................................................    1    0    k04
.........caaaaguaauugugguuuuugu..................................................    2    0    k04
```

```
                Mature                      Star
aaguuggugcaaaaguaauugugguuuuuguaauuuuuuuaaugacaaaaccaugauuauuuuugcaccaaccuaauauca
..........aaaaguaauugugguuuu.....................................................    1     0     k04
..........aaaaguaauugugguuuuug...................................................    5     0     k04
..........aaaaguaauugugguuuuugu..................................................    1     0     k04

....uggugcaaaaguaauuguggu........................................................    2     0     U20
....uggugcaaaaguaauugugguu.......................................................    1     0     U20
......gugcaaaaguaauugugguuuu.....................................................    1     0     U20
.........caaaaguaauugugguuuuug...................................................    1     0     U20
.........caaaaguaauugugguuuuugu..................................................    1     0     U20
..........aaaaguaauugugguuuuu....................................................    1     0     U20
..........aaaaguaauugugguuuuug...................................................    6     0     U20
...........aaaguaauugugguuuuugu..................................................    1     0     U20

.........caaaaguaauugugguuuuu....................................................    2     0     ku2
.........caaaaguaauugugguuuuugu..................................................    2     0     ku2
..........aaaaguaauugugguuuuu....................................................    5     0     ku2
..........aaaaguaauugugguuuuug...................................................    16    0     ku2

.........caaaaguaauugugguuuuu....................................................    1     0     j21
.........caaaaguaauugugguuuuug...................................................    3     0     j21
..........aaaaguaauugugguuuuu....................................................    1     0     j21
..........aaaaguaauugugguuuuug...................................................    14    0     j21
...........aaaguaauugugguuuuug...................................................    3     0     j21

..........aaaaguaauugugguuuuug...................................................    1     0     s11

..........aaaaguaauugugguuuuu....................................................    4     0     j07
..........aaaaguaauugugguuuuug...................................................    2     0     j07
...........aaaguaauugugguuuuug...................................................    1     0     j07

.........caaaaguaauugugguuuuug...................................................    5     0     j14
..........aaaaguaauugugguuuuug...................................................    9     0     j14
..........aaaaguaauugugguuuuugu..................................................    1     0     j14

.........caaaaguaauugugguuuuug...................................................    2     0     k02
.........caaaaguaauugugguuuuugu..................................................    1     0     k02
..........aaaaguaauugugguuuuu....................................................    2     0     k02

.........caaaaguaauugugguuuuu....................................................    1     0     j28
..........aaaaguaauugugguuuuu....................................................    3     0     j28
..........aaaaguaauugugguuuuug...................................................    17    0     j28

..........aaaaguaauugugguuuuug...................................................    1     0     s08

.........caaaaguaauugugguuuuugu..................................................    1     0     DLD
..........aaaaguaauugugguuuuu....................................................    2     0     DLD
..........aaaaguaauugugguuuuug...................................................    1     0     DLD

..guuggugcaaaaguaauuguggu........................................................    1     0     k03
..........aaaaguaauugugguuuuug...................................................    5     0     k03

.........caaaaguaauugugguuu......................................................    1     0     NL3
.........caaaaguaauugugguuuu.....................................................    1     0     NL3
.........caaaaguaauugugguuuuu....................................................    4     0     NL3
.........caaaaguaauugugguuuuug...................................................    2     0     NL3
..........aaaaguaauugugguuuu.....................................................    15    0     NL3
..........aaaaguaauugugguuuuu....................................................    6     0     NL3
..........aaaaguaauugugguuuuug...................................................    5     0     NL3

.........caaaaguaauugugguuu......................................................    1     0     ya2
.........caaaaguaauugugguuuu.....................................................    6     0     ya2
.........caaaaguaauugugguuuuu....................................................    13    0     ya2
.........caaaaguaauugugguuuuug...................................................    2     0     ya2
..........aaaaguaauugugguuuu.....................................................    24    0     ya2
..........aaaaguaauugugguuuuu....................................................    188   0     ya2
..........aaaaguaauugugguuuuug...................................................    17    0     ya2
..........aaaaguaauugugguuuuugu..................................................    4     0     ya2
...........aaaguaauugugguuuuu....................................................    43    0     ya2
...........aaaguaauugugguuuuug...................................................    4     0     ya2

.........caaaaguaauugugguuuuug...................................................    1     0     s02
```

Mature Star

aaguuggugcaaaaguaauugugguuuuuguaauuuuuuuaaugacaaaaccaugauuauuuuugcaccaaccuaauauca

.........caaaaguaauugugguuuuug...	3	0	k06
..........aaaaguaauugugguuuuug...	2	0	k06
........gcaaaaguaauugugguuuuug...	1	0	j29
.........caaaaguaauugugguuuuug...	3	0	j29
.........caaaaguaauugugguuuuugu..	2	0	j29
..........aaaaguaauugugguuuuug...	10	0	j29
...uuggugcaaaaguaauugugguu...	1	0	sh2
......gugcaaaaguaauugugguu...	1	0	sh2
.........caaaaguaauugugguuuuu..	1	0	sh2
..........aaaaguaauugugguuuuug...	1	0	sh2
.....ggugcaaaaguaauugugguuu..	1	0	j01
.......ugcaaaaguaauugugguuuu...	3	0	j01
.........caaaaguaauugugguuuuug...	1	0	j01
..........aaaaguaauugugguuuuug...	5	0	j01
....uggugcaaaaguaauuguggu..	1	0	MCF
.........caaaaguaauugugguuuuugu..	1	0	MCF
..........aaaaguaauugugguuuuug...	4	0	MCF
..........aaaaguaauugugguuuuug...	2	0	MBM
..........aaaaguaauugugguuuuu..	2	0	NL1
..........aaaaguaauugugguuuuug...	2	0	NL1
.....ggugcaaaaguaauugugguu...	1	0	k07
.......ugcaaaaguaauugugguuuuu..	1	0	k07
.........caaaaguaauugugguuuuug...	9	0	k07
.........caaaaguaauugugguuuuugu..	3	0	k07
..........aaaaguaauugugguuuuug...	3	0	k07
.......ugcaaaaguaauugugguuuuu..	1	0	sh1
.........caaaaguaauugugguuuuug...	6	0	sh1
..........aaaaguaauugugguuuuug...	4	0	sh1
....uggugcaaaaguaauugugguu...	1	0	li1
.........caaaaguaauugugguuuuug...	3	0	li1
.........caaaaguaauugugguuuuu..	1	0	k13
.........caaaaguaauugugguuuuug...	4	0	k13
.........caaaaguaauugugguuuuugu..	3	0	k13
..........aaaaguaauugugguuuuug...	1	0	k13
.........caaaaguaauugugguuuu...	1	0	j20
.........caaaaguaauugugguuuuu..	2	0	j20
.........caaaaguaauugugguuuuug...	7	0	j20
..........aaaaguaauugugguuuuu..	3	0	j20
..........aaaaguaauugugguuuuug...	27	0	j20
........gcaaaaguaauugugguuuuug...	11	0	A54
.........caaaaguaauugugguuuuugu..	1	0	A54
..........aaaaguaauugugguuuuug...	3	0	A54
....uggugcaaaaguaauugugguu...	1	0	j30
..........aaaaguaauugugguuuuug...	2	0	j30
.........caaaaguaauugugguuuuu..	8	0	k12
.........caaaaguaauugugguuuuug...	12	0	k12
..........aaaaguaauugugguuuuu..	6	0	k12
..........aaaaguaauugugguuuuug...	18	0	k12
..........aaaaguaauugugguuuuugu..	2	0	k12

Provisional ID	:	hsa_can_857
Score total	:	271.1
Score for star read(s)	:	3.9
Score for read counts	:	266.3
Score for mfe	:	1.6
Score for randfold	:	
Score for cons. seed	:	-0.6
Total read count	:	534
Mature read count	:	521
Loop read count	:	0
Star read count	:	13

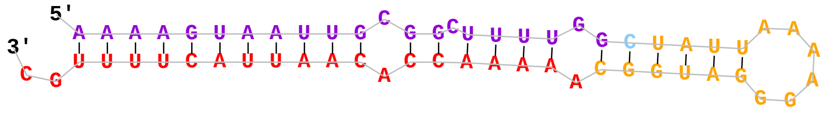

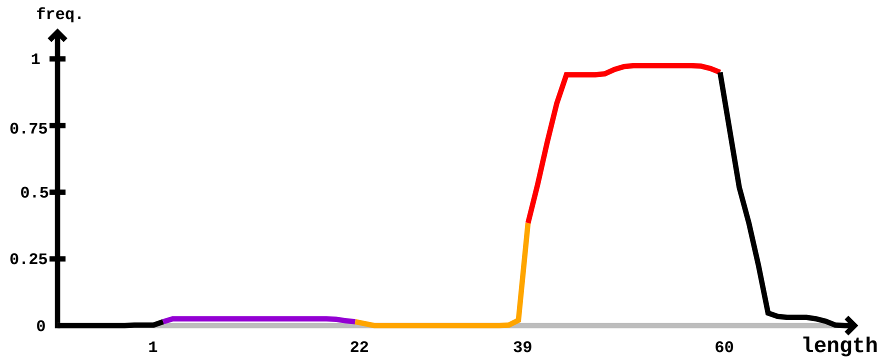

Star Mature

```
5'- gguuugugcaaaaguaauugcggcuuuuggcuauuaaaagggauggcaaaaaccacaauuacuuuugcaccaaccuaauaa  -3'   obs
    gguuugugcaaaaguaauugcggcuuuuggcuauuaaaagggauggcaaaaaccacaauuacuuuugcaccaaccuaauaa        exp
    gguuugugcaaaaguaauugcggcuuuuggcuauuaaaagggauggcaaaaaccacaauuacuuuugcaccaaccuaauaa        known
    ((((.((((((((((((((((.((.((((.((((((......)))))).)))))).))))))))))))))).)))).....    reads    mm    sample
    ...............................................aaaaaccacaauuacuuuug..............    1        0     ya1
    ...............................................aaaaaccacaauuacuuuugca............    4        0     ya1
    ................................................aaaaccacaauuacuuuugca............    3        0     ya1
    ................................................aaaaccacaauuacuuuugcac...........    1        0     ya1
    .................................................aaaccacaauuacuuuugca............    1        0     ya1
    ..................................................aaccacaauuacuuuugcacc..........    1        0     ya1

    ...............................................aaaaaccacaauuacuuuugca............    1        0     s04

    ...............................................aaaaaccacaauuacuuuugca............    3        0     j26
    ...................................................accacaauuacuuuugcacca.........    1        0     j26

    ...............................................aaaaaccacaauuacuuuugc.............    1        0     va1
    ...............................................aaaaaccacaauuacuuuugca............    6        0     va1
    ...............................................aaaaaccacaauuacuuuugcac...........    1        0     va1
    ................................................aaaaccacaauuacuuuugcac...........    5        0     va1
    .................................................aaaccacaauuacuuuugcacc..........    1        0     va1

    ...............................................aaaaaccacaauuacuuuugca............    1        0     ku5
    .................................................aaaccacaauuacuuuugcacc..........    1        0     ku5

    ..........aaaguaauugcggcuuuugg...................................................    1        0     k05
    .................................................aaaccacaauuacuuuugcacc..........    1        0     k05

    ...............................................aaaaaccacaauuacuuuugca............    1        0     s06
    ..................................................aaccacaauuacuuuugcacca.........    1        0     s06
    ...................................................accacaauuacuuuugcacca.........    1        0     s06

    ...............................................aaaaaccacaauuacuuuugc.............    1        0     HeL
    ...............................................aaaaaccacaauuacuuuugca............    3        0     HeL
    ................................................aaaaccacaauuacuuuugcac...........    2        0     HeL
    .................................................aaaccacaauuacuuuugcac...........    1        0     HeL
```

Star | Mature

```
gguuugugcaaaaguaauugcggcuuuuggcuauuaaaagggauggcaaaaaccacaauuacuuuugcaccaaccuaauaa
.................................................aaaccacaauuacuuuugcacc..........	3	0	HeL
..................................................aaccacaauuacuuuugcacca.........	3	0	HeL
..................................................aaccacaauuacuuuugcaccaa........	3	0	HeL
...................................................accacaauuacuuuugcacca.........	5	0	HeL

.................................................aaaccacaauuacuuuugcacc..........	1	0	s07

...............................................aaaaaccacaauuacuuuugca............	4	0	s10
.................................................aaaccacaauuacuuuugcac...........	1	0	s10

.........aaaaguaauugcggcuuuugg...................................................	1	0	ta3
...............................................aaaaaccacaauuacuuuugca............	4	0	ta3
................................................aaaaccacaauuacuuuugc.............	1	0	ta3
................................................aaaaccacaauuacuuuugcac...........	1	0	ta3
.................................................aaaccacaauuacuuuugcac...........	1	0	ta3
.................................................aaaccacaauuacuuuugcacc..........	1	0	ta3
..................................................aaccacaauuacuuuugcacca.........	2	0	ta3

...............................................aaaaaccacaauuacuuuugca............	5	0	j10
.................................................aaaccacaauuacuuuugcac...........	1	0	j10
..................................................aaccacaauuacuuuugcacca.........	1	0	j10

..................................................aaccacaauuacuuuugcac...........	1	0	k14

...............................................aaaaaccacaauuacuuuugcac...........	1	0	va2
.......................................................caauuacuuuugcaccaaccuaa...	1	0	va2

.................................................aaaccacaauuacuuuugca............	1	0	j27

...............................................aaaaaccacaauuacuuuugca............	1	0	j31

...............................................aaaaaccacaauuacuuuugca............	2	0	s03
................................................aaaaccacaauuacuuuugca............	1	0	s03
................................................aaaaccacaauuacuuuugcac...........	2	0	s03

..........aaaguaauugcggcuuuuggc..................................................	1	0	H52
...............................................aaaaaccacaauuacuuuug..............	2	0	H52
...............................................aaaaaccacaauuacuuuugca............	1	0	H52
................................................aaaaccacaauuacuuuugc.............	1	0	H52
................................................aaaaccacaauuacuuuugca............	2	0	H52
.................................................aaaccacaauuacuuuugcacc..........	2	0	H52
..................................................aaccacaauuacuuuugcacc..........	1	0	H52
..................................................aaccacaauuacuuuugcacca.........	1	0	H52
...................................................accacaauuacuuuugcacca.........	1	0	H52

...............................................aaaaaccacaauuacuuuugca............	6	0	j22
................................................aaaaccacaauuacuuuugca............	1	0	j22
..................................................aaccacaauuacuuuugcacc..........	1	0	j22

................................................aaaaccacaauuacuuuugcac...........	1	0	k09
.................................................aaaccacaauuacuuuugcaccaa........	1	0	k09

...............................................aaaaaccacaauuacuuuugc.............	3	0	B14
...............................................aaaaaccacaauuacuuuugca............	6	0	B14
................................................aaaaccacaauuacuuuugcac...........	1	0	B14
.................................................aaaccacaauuacuuuugca............	2	0	B14
.................................................aaaccacaauuacuuuugcacc..........	2	0	B14
...................................................accacaauuacuuuugcacca.........	2	0	B14

...............................................aaaaaccacaauuacuuuugcac...........	1	0	ku3
.................................................aaaccacaauuacuuuugcacca.........	1	0	ku3

...............................................aaaaaccacaauuacuuuugca............	4	0	j16

...............................................aaaaaccacaauuacuuuugca............	1	0	j09
...................................................accacaauuacuuuugcacca.........	1	0	j09

...............................................aaaaaccacaauuacuuuugca............	2	0	ku6

...............................................aaaaaccacaauuacuuuugca............	2	0	j11
```

Star   Mature

```
gguuugugcaaaaguaauugcggcuuuuggcuauuaaaagggauggcaaaaaccacaauuacuuuugcaccaaccuaauaa

...............................................aaaaaccacaauuacuuuugca............   3   0   ya3
................................................aaaaccacaauuacuuuugca............   2   0   ya3
................................................aaaaccacaauuacuuuugcac...........   5   0   ya3
.................................................aaaccacaauuacuuuugca............   1   0   ya3
.................................................aaaccacaauuacuuuugcacc..........   1   0   ya3
..................................................aaccacaauuacuuuugcacc..........   2   0   ya3

...............................................aaaaaccacaauuacuuuugca............   2   0   la1
..................................................aaccacaauuacuuuugcacc..........   1   0   la1

.........aaaaguaauugcggcuuuuggc..................................................   1   0   HEK
..............................................caaaaaccacaauuacuuuugc.............   3   0   HEK
...............................................aaaaaccacaauuacuuuugc.............   1   0   HEK
...............................................aaaaaccacaauuacuuuugca............   8   0   HEK
................................................aaaaccacaauuacuuuugca............   2   0   HEK
................................................aaaaccacaauuacuuuugcac...........   3   0   HEK
.................................................aaaccacaauuacuuuugca............   1   0   HEK
.................................................aaaccacaauuacuuuugcac...........   3   0   HEK
.................................................aaaccacaauuacuuuugcacc..........   3   0   HEK
..................................................aaccacaauuacuuuugcacc..........   2   0   HEK
..................................................aaccacaauuacuuuugcacca.........   4   0   HEK
...................................................accacaauuacuuuugcacca.........   6   0   HEK

................................................aaaaccacaauuacuuuugca............   1   0   j15

.........aaaaguaauugcggcuuuug....................................................   1   0   j17
................................................aaaaccacaauuacuuuugca............   1   0   j17
..................................................aaccacaauuacuuuugcacca.........   2   0   j17
...................................................accacaauuacuuuugcacca.........   1   0   j17

................................................aaaaccacaauuacuuuugcac...........   1   0   la2
.................................................aaaccacaauuacuuuugca............   1   0   la2
.................................................aaaccacaauuacuuuugcacc..........   1   0   la2
..................................................aaccacaauuacuuuugcacca.........   1   0   la2

...................................................accacaauuacuuuugcacca.........   1   0   j12

...............................................aaaaaccacaauuacuuuugca............   3   0   va4
...............................................aaaaaccacaauuacuuuugcac...........   1   0   va4
................................................aaaaccacaauuacuuuugcac...........   1   0   va4
..................................................aaccacaauuacuuuugcacc..........   2   0   va4
...................................................accacaauuacuuuugcacca.........   4   0   va4

...............................................aaaaaccacaauuacuuuugca............   12  0   va3
................................................aaaaccacaauuacuuuugcac...........   2   0   va3
.................................................aaaccacaauuacuuuugcac...........   1   0   va3
.................................................aaaccacaauuacuuuugcacc..........   4   0   va3
..................................................aaccacaauuacuuuugcacc..........   2   0   va3
..................................................aaccacaauuacuuuugcacca.........   2   0   va3
...................................................accacaauuacuuuugcacca.........   1   0   va3

...............................................aaaaaccacaauuacuuuugca............   1   0   SW4
................................................aaaaccacaauuacuuuugcac...........   1   0   SW4
.................................................aaaccacaauuacuuuugcac...........   2   0   SW4
.................................................aaaccacaauuacuuuugcacc..........   3   0   SW4
..................................................aaccacaauuacuuuugcacca.........   3   0   SW4
...................................................accacaauuacuuuugcacc..........   1   0   SW4
...................................................accacaauuacuuuugcacca.........   3   0   SW4

.........aaaaguaauugcggcuuu......................................................   1   0   ya4
...............................................aaaaaccacaauuacuuuugca............   4   0   ya4
................................................aaaaccacaauuacuuuugca............   2   0   ya4
................................................aaaaccacaauuacuuuugcac...........   4   0   ya4
.................................................aaaccacaauuacuuuugcac...........   1   0   ya4
.................................................aaaccacaauuacuuuugcacc..........   4   0   ya4
..................................................aaccacaauuacuuuugca............   3   0   ya4
..................................................aaccacaauuacuuuugcacc..........   4   0   ya4
..................................................aaccacaauuacuuuugcacca.........   2   0   ya4

...............................................aaaaaccacaauuacuuuugca............   4   0   ku1
```

```
          Star                                    Mature
gguuugugcaaaaguaauugcggcuuuggcuauuaaaagggauggcaaaaaccacaauuacuuuugcaccaaccuaauaa
.................................................aaccacaauuacuuuugcacc..........   1    0    ku1

..........aaaguaauugcggcuuug....................................................   1    0    j23
..........aaaguaauugcggcuuugg...................................................   1    0    j23
..............................................aaaaaccacaauuacuuuugc.............   1    0    j23
..............................................aaaaaccacaauuacuuuugca............   2    0    j23
...............................................aaaaccacaauuacuuuugca............   1    0    j23
.................................................aaccacaauuacuuuugcacca.........   1    0    j23
..................................................accacaauuacuuuugcacca.........   1    0    j23

..............................................aaaaaccacaauuacuuuugca............   2    0    s05

..............................................aaaaaccacaauuacuuu................   1    0    ta2
...............................................aaaaccacaauuacuuuugcac...........   1    0    ta2

..............................................aaaaaccacaauuacuuuugca............   1    0    k11

..............................................aaaaaccacaauuacuuuugca............   1    0    ta1

.............................................caaaaaccacaauuacuuuugc.............   1    0    s09
...............................................aaaaccacaauuacuuuugca............   1    0    s09
.................................................aaccacaauuacuuuugcacca.........   1    0    s09
..................................................accacaauuacuuuugcacca.........   1    0    s09

.............................................caaaaaccacaauuacuuuu...............   1    0    NL2
..............................................aaaaaccacaauuacuuuugca............   3    0    NL2
.................................................aaccacaauuacuuuugcacc..........   1    0    NL2
.................................................aaccacaauuacuuuugcacca.........   5    0    NL2

.........aaaaguaauugcggcuuuu....................................................   1    0    j13
..............................................aaaaaccacaauuacuuuugca............   4    0    j13
...............................................aaaaccacaauuacuuuugca............   2    0    j13
.................................................aaccacaauuacuuuugcacca.........   2    0    j13

..............................................aaaaaccacaauuacuuuugca............   2    0    j19
................................................aaaccacaauuacuuuugca............   2    0    j19
.................................................aaccacaauuacuuuugcacca.........   1    0    j19
..................................................accacaauuacuuuugcacca.........   2    0    j19

..............................................aaaaaccacaauuacuuuugca............   1    0    j25

.......................................................aauuacuuuugcaccaaccua....   2    0    per

..............................................aaaaaccacaauuacuuuugca............   2    0    s01
...............................................aaaaccacaauuacuuuugcac...........   2    0    s01
................................................aaaccacaauuacuuuugcacc..........   2    0    s01

...............................................aaaaccacaauuacuuuugca............   1    0    k04
........................................................auuacuuuugcaccaaccuaau..   1    0    k04

......ugcaaaaguaauugcggcuuuu....................................................   1    0    U20
.............................................caaaaaccacaauuacuuuugc.............   1    0    U20
..............................................aaaaaccacaauuacuuuugc.............   22   0    U20
..............................................aaaaaccacaauuacuuuugca............   17   0    U20
...............................................aaaaccacaauuacuuuugca............   1    0    U20
...............................................aaaaccacaauuacuuuugcac...........   8    0    U20
................................................aaaccacaauuacuuuugcac...........   6    0    U20
................................................aaaccacaauuacuuuugcacc..........   14   0    U20
.................................................aaccacaauuacuuuugcacc..........   6    0    U20
.................................................aaccacaauuacuuuugcacca.........   1    0    U20
..................................................accacaauuacuuuugcacca.........   15   0    U20
..................................................accacaauuacuuuugcaccaa........   1    0    U20

..............................................aaaaaccacaauuacuuuugca............   3    0    j21
...............................................aaaaccacaauuacuuuugca............   1    0    j21
................................................aaaccacaauuacuuuugcacc..........   1    0    j21


.................................................aaccacaauuacuuuugcacc..........   1    0    s11
.................................................aaccacaauuacuuuugcacca.........   1    0    s11
```

Star **Mature**

```
gguuugugcaaaaguaauugcggcuuuuggcuauuaaaagggauggcaaaaaccacaauuacuuuugcaccaaccuaauaa

...............................................aaaaaccacaauuacuuuugca............	1	0	j07

.................................................aaaccacaauuacuuuugcacc..........	1	0	j14

.........aaaaguaauugcggcuuuugg...................................................	2	0	j28

...............................................aaaaaccacaauuacuuuugca............	1	0	s08
...................................................accacaauuacuuuugcac...........	1	0	s08

...............................................aaaaaccacaauuacuuuugc.............	1	0	DLD
................................................aaaaccacaauuacuuuugcac...........	1	0	DLD
.................................................aaaccacaauuacuuuugcacc..........	3	0	DLD
...................................................accacaauuacuuuugcacc..........	1	0	DLD
...................................................accacaauuacuuuugcacca.........	4	0	DLD

...............................................aaaaaccacaauuacuuuugca............	1	0	k03

..............................................caaaaaccacaauuacuuuu...............	1	0	NL3
..............................................caaaaaccacaauuacuuuug..............	2	0	NL3
...............................................aaaaaccacaauuacuuuugca............	7	0	NL3
................................................aaaaccacaauuacuuuugcac...........	1	0	NL3
..................................................aaccacaauuacuuuugcacca.........	1	0	NL3
.........................................................auuacuuuugcaccaaccuaa...	1	0	NL3

..........aaaguaauugcggcuuuu.....................................................	1	0	ya2
...............................................aaaaaccacaauuacuuuu...............	2	0	ya2
...............................................aaaaaccacaauuacuuuugc.............	1	0	ya2
...............................................aaaaaccacaauuacuuuugca............	2	0	ya2
................................................aaaaccacaauuacuuuugca............	6	0	ya2
.................................................aaaccacaauuacuuuugcac...........	3	0	ya2
.................................................aaaccacaauuacuuuugcacca.........	1	0	ya2
..................................................aaccacaauuacuuuugcacca.........	2	0	ya2
.......................................................caauuacuuuugcaccaac.......	1	0	ya2
........................................................aauuacuuuugcaccaac.......	1	0	ya2

.................................................aaaccacaauuacuuuugcacc..........	1	0	k06

..............................................caaaaaccacaauuacuuuug..............	1	0	sh2
..................................................aaccacaauuacuuuugcaccaa........	2	0	sh2
..........................................................uuacuuuugcaccaaccua....	1	0	sh2

...............................................aaaaaccacaauuacuuuugca............	1	0	j29
................................................aaaaccacaauuacuuuugca............	1	0	j29

...............................................aaaaaccacaauuacuuuu...............	1	0	MCF
...............................................aaaaaccacaauuacuuuugc.............	1	0	MCF
...............................................aaaaaccacaauuacuuuugca............	2	0	MCF
................................................aaaaccacaauuacuuuugcac...........	1	0	MCF
........................................................aauuacuuuugcaccaaccuaa...	2	0	MCF
........................................................aauuacuuuugcaccaaccuaau..	3	0	MCF
.........................................................auuacuuuugcaccaaccuaau..	4	0	MCF

...............................................aaaaaccacaauuacuuuug..............	1	0	MBM
...............................................aaaaaccacaauuacuuuugc.............	1	0	MBM
...............................................aaaaaccacaauuacuuuugca............	2	0	MBM
................................................aaaaccacaauuacuuuugca............	1	0	MBM
................................................aaaaccacaauuacuuuugcac...........	3	0	MBM
.................................................aaaccacaauuacuuuugcacc..........	4	0	MBM
..................................................aaccacaauuacuuuugcacc..........	1	0	MBM
...................................................accacaauuacuuuugcacca.........	4	0	MBM

.............................................gcaaaaaccacaauuacuuuug..............	1	0	li1
...............................................aaaaaccacaauuacuuuugc.............	1	0	li1
................................................aaaaccacaauuacuuuugcac...........	1	0	li1
.................................................aaaccacaauuacuuuugcacc..........	2	0	li1
..................................................aaccacaauuacuuuugcacca.........	1	0	li1

........................................................aauuacuuuugcaccaaccuaa...	1	0	k13
..........................................................uuacuuuugcaccaaccuaaua.	1	0	k13
```

Star **Mature**

```
gguuugugcaaaaguaauugcggcuuuuggcuauuaaaagggauggcaaaaaccacaauuacuuuugcaccaaccuaauaa

...............................................aaaaaccacaauuacuuuugc.............   7   0   A54
..................................................aaccacaauuacuuuugcacc..........   4   0   A54
..................................................aaccacaauuacuuuugcacca.........   4   0   A54

..........aaaguaauugcggcuuuugg...................................................   1   0   k12
...................................................accacaauuacuuuugcacca.........   1   0   k12
```

Provisional ID	:	hsa_can_859
Score total	:	-3.5
Score for star read(s)	:	-1.3
Score for read counts	:	-2.8
Score for mfe	:	1.2
Score for randfold	:	
Score for cons. seed	:	-0.6
Total read count	:	6
Mature read count	:	6
Loop read count	:	0
Star read count	:	0

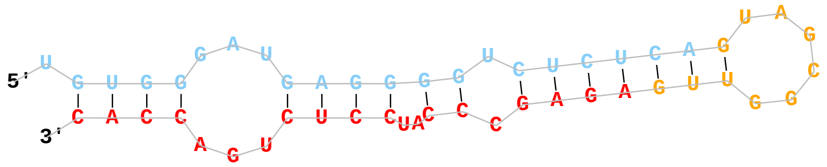

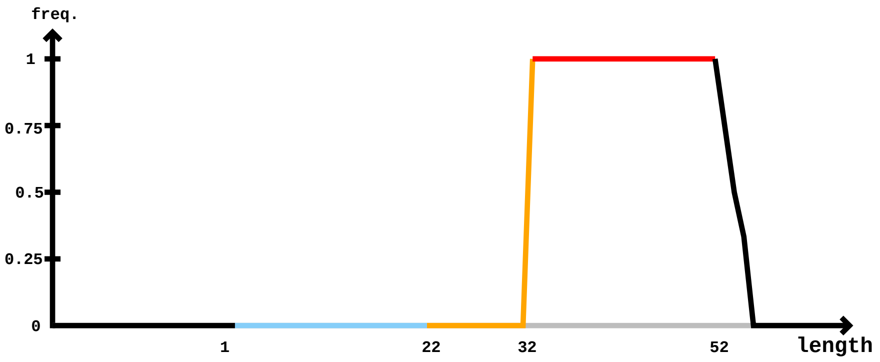

Star Mature

```
5'- ggugggugcugagcgguuguggaugaggggucucucaguagcgguugagagcccauccucugaccacugcuuuuucaugu  -3'   exp
    .(((((....(((((((.((.(((.((.(((.(((((((......)))))))))).)).))).)).))))))).))))).. reads     mm        sample
    .............................................agagcccauccucugaccacugc..........    1        0         j23

    .............................................agagcccauccucugaccac.............    2        0         j04

    .............................................agagcccauccucugaccacugc..........    1        0         j13

    .............................................agagcccauccucugaccacu............    1        0         k02

    .............................................agagcccauccucugaccacug...........    1        0         j21
```

Provisional ID	:	hsa_can_873
Score total	:	0.1
Score for star read(s)	:	-1.3
Score for read counts	:	0
Score for mfe	:	2
Score for randfold	:	
Score for cons. seed	:	-0.6
Total read count	:	44
Mature read count	:	44
Loop read count	:	0
Star read count	:	0

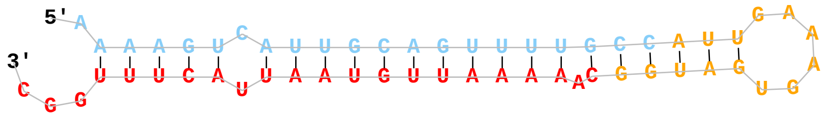

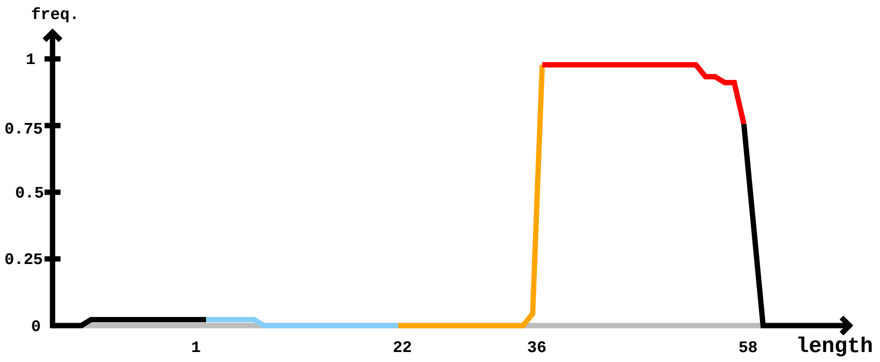

Star **Mature**

```
5'- uguuagguuggugcaaaagucauugcaguuuugccauugaaagugauggcaaaaauuguaauuacuuuggcaccaaccuaa   -3'    exp
    ..((((((((((((.(((((.(((((((((((((((((......)))))).)))))))))))).))))).))))))))))))    reads      mm         sample
    .................................................caaaaauuguaauuacuuugg...........    1          0          j13

    ................................................gcaaaaauuguaauuacu...............    2          0          ya1

    .................................................caaaaauuguaauuacuuuggc..........    2          0          li1
    .................................................caaaaauuguaauuacuuuggca.........    1          0          li1

    .................................................caaaaauuguaauuacuuuggc..........    18         0          li2
    .................................................caaaaauuguaauuacuuuggca.........    2          0          li2

    .................................................caaaaauuguaauuacuuugg...........    3          0          j16
    .................................................caaaaauuguaauuacuuuggc..........    1          0          j16

    .................................................caaaaauuguaauuacuuu.............    1          0          NL3
    .................................................caaaaauuguaauuacuuuggc..........    1          0          NL3

    .................................................caaaaauuguaauuacuuuggc..........    2          0          s01

    .................................................caaaaauuguaauuacuuuggc..........    1          0          j10

    .................................................caaaaauuguaauuacuuuggc..........    2          0          j21

    .................................................caaaaauuguaauuacuuugg...........    1          0          ku5

    .................................................caaaaauuguaauuacuuuggc..........    1          0          s06

    .................................................caaaaauuguaauuacuuugg...........    2          0          j28

    .................................................caaaaauuguaauuacuuuggc..........    2          0          j15

    .................................................caaaaauuguaauuacuuuggc..........    1          0          MBM

    ..uuagguuggugcaaaagu.............................................................    1          0          la2
```

Provisional ID	:	hsa_can_931
Score total	:	3.7
Score for star read(s)	:	-1.3
Score for read counts	:	0
Score for mfe	:	2
Score for randfold	:	
Score for cons. seed	:	3
Total read count	:	443
Mature read count	:	443
Loop read count	:	0
Star read count	:	0

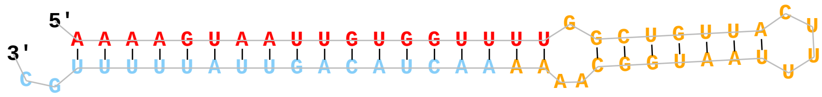

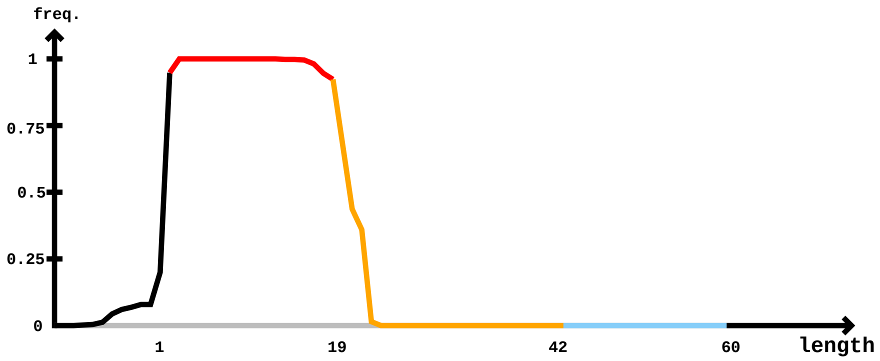

Mature **Star**

```
5'- agguuggugcaaaaguaauugugguuuuggcuguuacuuuuaauggcaaaaaacuacaguuauuuuugcaccaaccuaaua   -3'   exp
    ((((((((((((((((((((((((((((.(((((((....)))))))..))))))))))))))))))))))))))))....    reads      mm         sample
    .........caaaaguaauugugguuuuggc..................................................    1          0          j23
    ..........aaaaguaauugugguuuu.....................................................    1          0          j23
    ..........aaaaguaauugugguuuugg...................................................    2          0          j23

    .........caaaaguaauugugguuuu.....................................................    5          0          ya1
    ..........aaaaguaauugugguuuu.....................................................    16         0          ya1
    ..........aaaaguaauugugguuuug....................................................    3          0          ya1

    ..........aaaaguaauugugguuuug....................................................    1          0          s05
    ..........aaaaguaauugugguuuuggc..................................................    1          0          s05

    ..........aaaaguaauugugguuuuggc..................................................    1          0          j26

    ...........aaaguaauugugguuuuggc..................................................    2          0          ta2

    ....uggugcaaaaguaauugugguu.......................................................    1          0          va1
    ..........aaaaguaauugugguuuug....................................................    1          0          va1
    ..........aaaaguaauugugguuuugg...................................................    7          0          va1
    ..........aaaaguaauugugguuuuggc..................................................    32         0          va1
    ...........aaaguaauugugguuuuggc..................................................    6          0          va1

    .....ggugcaaaaguaauugugguuu......................................................    5          0          j24
    .........caaaaguaauugugguuuu.....................................................    3          0          j24

    ...uuggugcaaaaguaauugugguuu......................................................    1          0          ku5
    ....uggugcaaaaguaauuguggu........................................................    1          0          ku5
    ......gugcaaaaguaauugugguu.......................................................    1          0          ku5

    ..........aaaaguaauugugguuuu.....................................................    1          0          ta1
    ..........aaaaguaauugugguuuuggc..................................................    1          0          ta1

    ...........aaaguaauugugguuuuggc..................................................    1          0          HeL

    ......gugcaaaaguaauuguggu........................................................    1          0          ku4
```

Mature Star

```
agguuggugcaaaaguaauugugguuuuggcuguuacuuuuaaauggcaaaaaacuacaguuauuuuugcaccaaccuaaua

..........aaaaguaauugugguuuuggc...................................................	2	0	s07

..........aaaaguaauugugguuuu......................................................	1	0	ta3
..........aaaaguaauugugguuuugg....................................................	1	0	ta3
..........aaaaguaauugugguuuuggc...................................................	1	0	ta3

.........caaaaguaauugugguu........................................................	1	0	NL2
.........caaaaguaauugugguuu.......................................................	1	0	NL2
.........caaaaguaauugugguuuug.....................................................	2	0	NL2
..........aaaaguaauugugguuuu......................................................	10	0	NL2
..........aaaaguaauugugguuuuggcu..................................................	1	0	NL2

..........aaaaguaauugugguuuu......................................................	1	0	j13

....uggugcaaaaguaauugugguu........................................................	1	0	k10

..........aaaaguaauugugguuuu......................................................	2	0	j19

..........aaaaguaauugugguuuuggcu..................................................	1	0	k01

....uggugcaaaaguaauugugguu........................................................	1	0	j25
.......ugcaaaaguaauugugguu........................................................	2	0	j25
.........caaaaguaauugugguuu.......................................................	1	0	j25

..........aaaaguaauugugguuuu......................................................	1	0	per
..........aaaaguaauugugguuuug.....................................................	1	0	per

..........aaaaguaauugugguuuu......................................................	1	0	k04
..........aaaaguaauugugguuuuggc...................................................	1	0	k04

..........aaaaguaauugugguuuugg....................................................	2	0	j10

..........aaaaguaauugugguuuug.....................................................	1	0	ku2
..........aaaaguaauugugguuuugg....................................................	6	0	ku2
..........aaaaguaauugugguuuuggc...................................................	5	0	ku2
..........aaaaguaauugugguuuuggcu..................................................	2	0	ku2
...........aaaguaauugugguuuuggc...................................................	1	0	ku2

....uggugcaaaaguaauuguggu.........................................................	2	0	U20
....uggugcaaaaguaauugugguu........................................................	1	0	U20
......gugcaaaaguaauugugguuuu......................................................	1	0	U20

..........aaaaguaauugugguuuug.....................................................	2	0	j21
..........aaaaguaauugugguuuugg....................................................	3	0	j21
..........aaaaguaauugugguuuuggc...................................................	14	0	j21
...........aaaguaauugugguuuuggc...................................................	4	0	j21

..........aaaaguaauugugguuuu......................................................	1	0	j02
..........aaaaguaauugugguuuug.....................................................	8	0	j02
..........aaaaguaauugugguuuugg....................................................	2	0	j02
..........aaaaguaauugugguuuuggc...................................................	3	0	j02

.....ggugcaaaaguaauugugguuu.......................................................	1	0	j27
..........aaaaguaauugugguuuu......................................................	1	0	j27

...uuggugcaaaaguaauuguggu.........................................................	1	0	j31
....uggugcaaaaguaauugugguu........................................................	1	0	j31
..........aaaaguaauugugguuuuggc...................................................	3	0	j31

..........aaaaguaauugugguuuuggc...................................................	1	0	k02

..........aaaaguaauugugguuuuggc...................................................	1	0	j28

..........aaaaguaauugugguuuuggc...................................................	2	0	H52

...uuggugcaaaaguaauugugg..........................................................	1	0	k09

..........aaaaguaauugugguuuuggc...................................................	3	0	B14

..........aaaaguaauugugguuuugg....................................................	1	0	j09
```

```
          Mature                                  Star
agguuggugcaaaaguaauugugguuuuggcuguuacuuuuaauggcaaaaaacuacaguuauuuuugcaccaaccuaaua
..........aaaaguaauugugguuuuggc..................................................  3   0  j09

..........aaaaguaauugugguuuu.....................................................  1   0  j16
..........aaaaguaauugugguuuugg...................................................  1   0  j16

..guuggugcaaaaguaauuguggu........................................................  1   0  k03
...........aaaguaauugugguuuuggc..................................................  2   0  k03

....uggugcaaaaguaauuguggu........................................................  1   0  ku3

.........caaaaguaauugugguuu......................................................  1   0  NL3
.........caaaaguaauugugguuuu.....................................................  1   0  NL3
..........aaaaguaauugugguuuu.....................................................  15  0  NL3
..........aaaaguaauugugguuuuggc..................................................  1   0  NL3

.........caaaaguaauugugguuu......................................................  1   0  ya2
.........caaaaguaauugugguuuu.....................................................  6   0  ya2
..........aaaaguaauugugguuuu.....................................................  24  0  ya2

.........caaaaguaauugugguuuu.....................................................  1   0  ku6
..........aaaaguaauugugguuuuggc..................................................  1   0  ku6

.........caaaaguaauugugguuuu.....................................................  9   0  ya3
.........caaaaguaauugugguuuug....................................................  5   0  ya3
..........aaaaguaauugugguuuu.....................................................  44  0  ya3

..........aaaaguaauugugguuuu.....................................................  1   0  la1
..........aaaaguaauugugguuuuggc..................................................  1   0  la1
...........aaaguaauugugguuuugg...................................................  1   0  la1

.gguuggugcaaaaguaauugu...........................................................  1   0  sh2
...uuggugcaaaaguaauugugguu.......................................................  1   0  sh2
......gugcaaaaguaauugugguu.......................................................  1   0  sh2
..........aaaaguaauugugguuuuggc..................................................  2   0  sh2

....uggugcaaaaguaauugugguu.......................................................  1   0  HEK
..........aaaaguaauugugguuuugg...................................................  2   0  HEK
..........aaaaguaauugugguuuuggc..................................................  11  0  HEK
...........aaaguaauugugguuuuggc..................................................  2   0  HEK

..........aaaaguaauugugguuuu.....................................................  1   0  j17
..........aaaaguaauugugguuuuggc..................................................  4   0  j17
..........aaaaguaauugugguuuuggcu.................................................  1   0  j17
...........aaaguaauugugguuuuggc..................................................  1   0  j17

..........aaaaguaauugugguuuu.....................................................  1   0  j15

....uggugcaaaaguaauuguggu........................................................  1   0  MCF
..........aaaaguaauugugguuuuggc..................................................  1   0  MCF

.....ggugcaaaaguaauugugguuu......................................................  1   0  j01
.......ugcaaaaguaauugugguuuu.....................................................  3   0  j01

.........caaaaguaauugugguuuug....................................................  1   0  la2
..........aaaaguaauugugguuuug....................................................  2   0  la2
..........aaaaguaauugugguuuuggc..................................................  7   0  la2

.....ggugcaaaaguaauugugguu.......................................................  1   0  k07

....uggugcaaaaguaauugugguu.......................................................  1   0  j12

....uggugcaaaaguaauugugguu.......................................................  1   0  li1
..........aaaaguaauugugguuuuggc..................................................  19  0  li1
...........aaaguaauugugguuuuggc..................................................  4   0  li1

....uggugcaaaaguaauugugguu.......................................................  1   0  li2
...........aaaguaauugugguuuuggc..................................................  1   0  li2

..........aaaaguaauugugguuuuggc..................................................  1   0  k13

..........aaaaguaauugugguuuug....................................................  1   0  va4
```

```
          Mature                                  Star
agguuggugcaaaaguaauugugguuuuggcuguuacuuuuaauggcaaaaaacuacaguuauuuuugcaccaaccuaaua

.........caaaaguaauugugguuuu.....................................................    1     0     j20
..........aaaaguaauugugguuuug....................................................    1     0     j20
..........aaaaguaauugugguuuugg...................................................    4     0     j20
..........aaaaguaauugugguuuuggc..................................................    4     0     j20

..........aaaaguaauugugguuuugg...................................................    1     0     va3
..........aaaaguaauugugguuuuggc..................................................    11    0     va3
..........aaaaguaauugugguuuuggcu.................................................    2     0     va3

..........aaaaguaauugugguuuugg...................................................    1     0     SW4
..........aaaaguaauugugguuuuggc..................................................    1     0     SW4

.........caaaaguaauugugguuuuggc..................................................    1     0     k12
..........aaaaguaauugugguuuuggc..................................................    1     0     k12

....uggugcaaaaguaauuguggu.......................................................    1     0     j30

.........caaaaguaauugugguuuu.....................................................    16    0     ya4
..........aaaaguaauugugguuuu.....................................................    33    0     ya4

.........caaaaguaauugugguuuugg...................................................    1     0     ku1
..........aaaaguaauugugguuuug....................................................    1     0     ku1
..........aaaaguaauugugguuuugg...................................................    2     0     ku1
..........aaaaguaauugugguuuuggc..................................................    1     0     ku1
```

Provisional ID : hsa_can_1010
Score total : 21
Score for star read(s) : 3.9
Score for read counts : 19
Score for mfe : -1.2
Score for randfold :
Score for cons. seed : -0.6
Total read count : 49
Mature read count : 47
Loop read count : 1
Star read count : 1

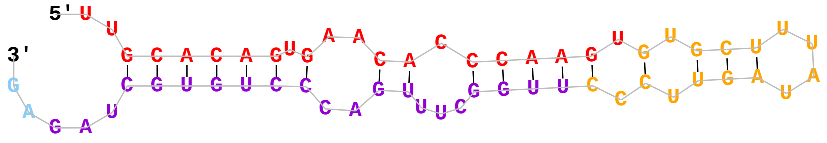

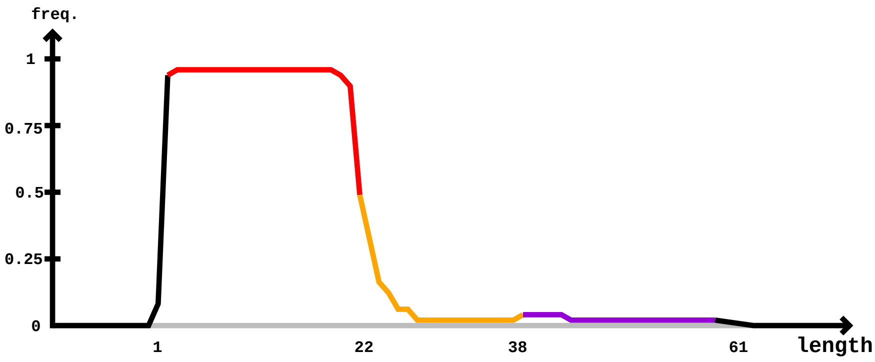

Mature **Star**

```
5'- ucagagugguuugcacagugaacacccaagugugcuuuauaguucccuuggcuuugacccugugcuagagcauucauuacc  -3'   obs
    ucagagugguuugcacagugaacacccaagugugcuuuauaguucccuuggcuuugacccugugcuagagcauucauuacc        exp
    ...(((((.(((((((((.(..((.(((((.(.(((....))).).)))))...))..))))))).))).)))))......   reads   mm   sample
    .........uugcacagugaacacccaagugug................................................   2       0    k09

    .........uugcacagugaacacccaagu...................................................   1       0    ta2

    .........uugcacagugaacacccaa.....................................................   1       0    va1
    .........uugcacagugaacacccaag....................................................   2       0    va1
    .........uugcacagugaacacccaagug..................................................   1       0    va1

    .........uugcacagugaacacccaag....................................................   1       0    j11

    ...............................................uuggcuuugacccugugcuag.............   1       0    s06

    ........uuugcacagugaacacccaag....................................................   1       0    ta1
    .........uugcacagugaacacccaagu...................................................   1       0    ta1
    .........uugcacagugaacacccaagugugcu..............................................   1       0    ta1

    ..............................gugcuuuauaguucccuuggc..............................   1       0    HeL

    .........uugcacagugaacacccaag....................................................   1       0    HEK
    .........uugcacagugaacacccaagug..................................................   1       0    HEK

    .........uugcacagugaacacccaag....................................................   2       0    j17
    .........uugcacagugaacacccaagu...................................................   1       0    j17

    .........uugcacagugaacaccca......................................................   1       0    MCF

    .........uugcacagugaacacccaag....................................................   1       0    NL1

    ........uuugcacagugaacacccaag....................................................   1       0    j12

    .........uugcacagugaacacccaag....................................................   5       0    li1
    .........uugcacagugaacacccaagu...................................................   1       0    li1

    .........uugcacagugaacacccaagug..................................................   1       0    li2
```

```
          Mature                               Star
ucagagugguuugcacagugaacacccaagugugcuuuauaguucccuuggcuuugacccugugcuagagcauucauuacc

..........uugcacagugaacacccaag...................................................    3    0    per

.........uuugcacagugaacacccaag...................................................    1    0    va3
.........uuugcacagugaacacccaagu..................................................    1    0    va3
..........uugcacagugaacacccaa....................................................    1    0    va3
..........uugcacagugaacacccaag...................................................    2    0    va3
..........uugcacagugaacacccaagu..................................................    7    0    va3
..........uugcacagugaacacccaagugu................................................    2    0    va3
...........ugcacagugaacacccaagugug...............................................    1    0    va3

..........uugcacagugaacacccaagu..................................................    1    0    j14
..........uugcacagugaacacccaagugugcu.............................................    1    0    j14

..........uugcacagugaacacccaagu..................................................    1    0    SW4
```

Provisional ID	:	hsa_can_1046
Score total	:	6.7
Score for star read(s)	:	3.9
Score for read counts	:	1.7
Score for mfe	:	1.8
Score for randfold	:	
Score for cons. seed	:	-0.6
Total read count	:	15
Mature read count	:	12
Loop read count	:	0
Star read count	:	3

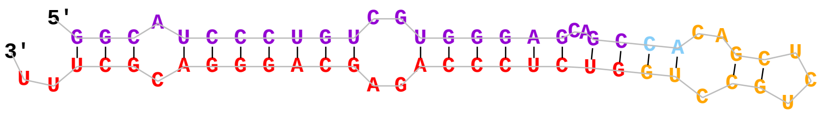

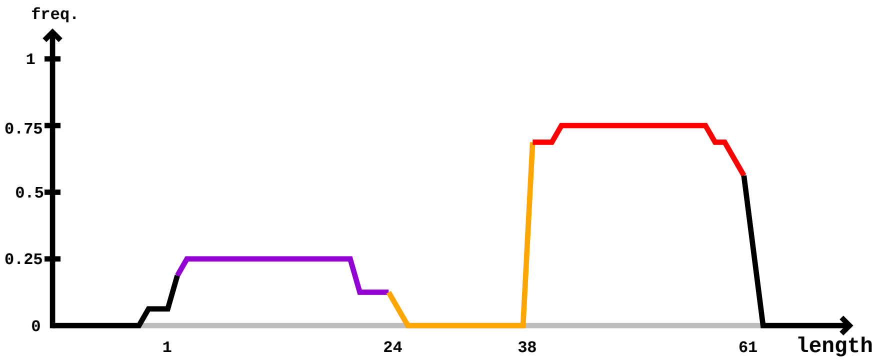

Star Mature

```
5'- ugucugggugaggcaucccugucgugggagcagccacagcucugccuggucucccagagcagggacgcuuuguccgcauuu -3'   obs
    ugucugggugaggcaucccugucgugggagcagccacagcucugccuggucucccagagcagggacgcuuuguccgcauuu       exp
    .....(((..((((.(((((((..((((((..((((..((...)).))))))))))..))))))).))))..)))......   reads   mm   sample
    ...........ggcaucccugucgugggagcagc...............................................   1       0    U20
    ................................................gucucccagagcagggacgcuu...........   1       0    U20
    ................................................gucucccagagcagggacgcuuu..........   2       0    U20

    ........ugaggcaucccugucgugggag...................................................   1       0    va3

    ............gcaucccugucgugggag...................................................   1       0    sh2
    ...................................................ucccagagcagggacgcuuu..........   1       0    sh2

    ................................................gucucccagagcagggacgcu............   1       0    HEK
    ................................................gucucccagagcagggacgcuuu..........   3       0    HEK

    ................................................gucucccagagcagggacgcuuu..........   2       0    SW4

    ...........ggcaucccugucgugggagcagc...............................................   1       0    H52

    ................................................gucucccagagcagggacg..............   1       0    MCF

    ................................................gucucccagagcagggacgcuuu..........   1       0    MBM
```

Provisional ID	:	hsa_can_1051
Score total	:	45.3
Score for star read(s)	:	3.9
Score for read counts	:	37.3
Score for mfe	:	1.1
Score for randfold	:	
Score for cons. seed	:	3
Total read count	:	85
Mature read count	:	83
Loop read count	:	0
Star read count	:	2

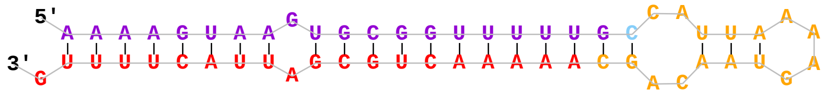

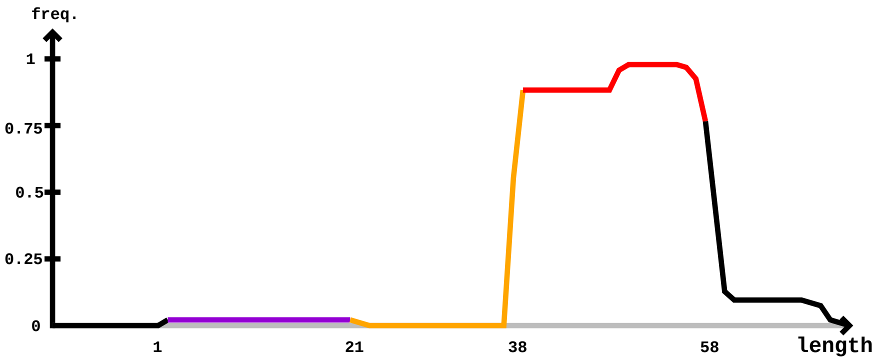

Star Mature

```
5'- agcuuagugcaaaaguaagugcgguuuuugccauuaaaaguaacagcaaaaacugcgauuacuuuugcaccaaccuaauau  -3'   obs
    agcuuagugcaaaaguaagugcgguuuuugccauuaaaaguaacagcaaaaacugcgauuacuuuugcaccaaccuaauau         exp
    ......((((((((((((.((((((((((((..(((....)))..)))))))))))).))))))))))))...........    reads   mm    sample
    ..............................................caaaaacugcgauuacuu.................    1       0     ya1
    ..............................................caaaaacugcgauuacuuu................    1       0     ya1
    ...............................................aaaaacugcgauuacuuu................    1       0     ya1
    ...............................................aaaaacugcgauuacuuuu...............    1       0     ya1

    ...............................................aaaaacugcgauuacuuuug..............    1       0     ta2

    ..............................................caaaaacugcgauuacuuuug..............    3       0     va1
    ..............................................caaaaacugcgauuacuuuugc.............    1       0     va1

    ..............................................caaaaacugcgauuacuuuug..............    1       0     k05

    ..............................................caaaaacugcgauuacuuuug..............    1       0     k11

    ..........aaaaguaagugcgguuuuug...................................................    1       0     ku4

    ..............................................caaaaacugcgauuacuuuug..............    1       0     s07
    ...............................................aaaaacugcgauuacuuuugca............    1       0     s07

    ..............................................caaaaacugcgauuacuuuu...............    2       0     NL2

    ..............................................caaaaacugcgauuacuuuug..............    3       0     j13
    ...............................................aaaaacugcgauuacuuu................    1       0     j13
    ...............................................aaaaacugcgauuacuuuu...............    2       0     j13
    ...............................................aaaaacugcgauuacuuuug..............    5       0     j13

    ...............................................aaaaacugcgauuacuuuugc.............    1       0     j19

    ...............................................aaaaacugcgauuacuuuug..............    1       0     j03

    .........................................................auuacuuuugcaccaaccuaau..    1       0     k04

    ..............................................caaaaacugcgauuacuuuugc.............    1       0     s01
```

Star **Mature**

```
agcuuagugcaaaaguaagugcgguuuuugccauuaaaaguaacagcaaaaacugcgauuacuuuugcaccaaccuaauau

...............................................aaaaacugcgauuacuuuug..............    1    0    ku2
...............................................aaaaacugcgauuacuuuugc.............    1    0    ku2

..............................................caaaaacugcgauuacuuu................    5    0    U20
..............................................caaaaacugcgauuacuuuugc.............    4    0    U20

..............................................caaaaacugcgauuacuuuugc.............    1    0    j21

...............................................aaaaacugcgauuacuuuug..............    1    0    j02

..............................................caaaaacugcgauuacuuuugc.............    1    0    j31

..........aaaaguaagugcgguuuuug...................................................    1    0    j27

..............................................caaaaacugcgauuacuuuug..............    1    0    k02

..............................................caaaaacugcgauuacuuuugc.............    2    0    j22
...............................................aaaaacugcgauuacuuuug..............    1    0    j22

..............................................caaaaacugcgauuacuuuugc.............    1    0    s08

..............................................caaaaacugcgauuacuuuugc.............    1    0    B14

..............................................caaaaacugcgauuacuuu................    1    0    j16
...............................................aaaaacugcgauuacuuuugca............    1    0    j16

...............................................aaaaacugcgauuacuuuug..............    3    0    ya2
.........................................................auuacuuuugcaccaaccuaauau    1    0    ya2

..............................................caaaaacugcgauuacuuuu...............    2    0    NL3
.........................................................auuacuuuugcaccaaccuaa...    1    0    NL3

..............................................caaaaacugcgauuacuuuu...............    1    0    ya3
...............................................aaaaacugcgauuacuuuu...............    1    0    ya3

..........................................................uuacuuuugcaccaaccua....    1    0    sh2

..............................................caaaaacugcgauuacuuuugc.............    5    0    HEK
...............................................aaaaacugcgauuacuuuugc.............    1    0    HEK

.........................................................auuacuuuugcaccaaccuaau..    4    0    MCF

..............................................caaaaacugcgauuacuuuug..............    1    0    j15
..............................................caaaaacugcgauuacuuuugc.............    2    0    j15
...............................................aaaaacugcgauuacuuuug..............    1    0    j15

...............................................aaaaacugcgauuacuuuu...............    1    0    j01

..............................................caaaaacugcgauuacuuuug..............    2    0    MBM

..............................................caaaaacugcgauuacuuuug..............    1    0    k07

..............................................caaaaacugcgauuacuuuug..............    1    0    li1
..............................................caaaaacugcgauuacuuuugc.............    1    0    li1
...............................................aaaaacugcgauuacuuuugca............    1    0    li1

..............................................caaaaacugcgauuacuuuugc.............    3    0    li2

..........................................................uuacuuuugcaccaaccuaaua.    1    0    k13

...............................................aaaaacugcgauuacuuuug..............    1    0    j20
...............................................aaaaacugcgauuacuuuugc.............    1    0    j20

..............................................caaaaacugcgauuacuuuugc.............    1    0    va3
...............................................aaaaacugcgauuacuuuugc.............    1    0    va3

...............................................aaaaacugcgauuacuuuug..............    1    0    ku1
```

Provisional ID	:	hsa_can_1067
Score total	:	3.2
Score for star read(s)	:	-1.3
Score for read counts	:	0
Score for mfe	:	1.5
Score for randfold	:	
Score for cons. seed	:	3
Total read count	:	2302
Mature read count	:	2302
Loop read count	:	0
Star read count	:	0

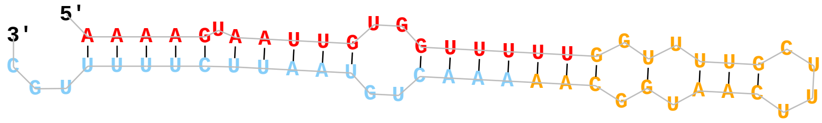

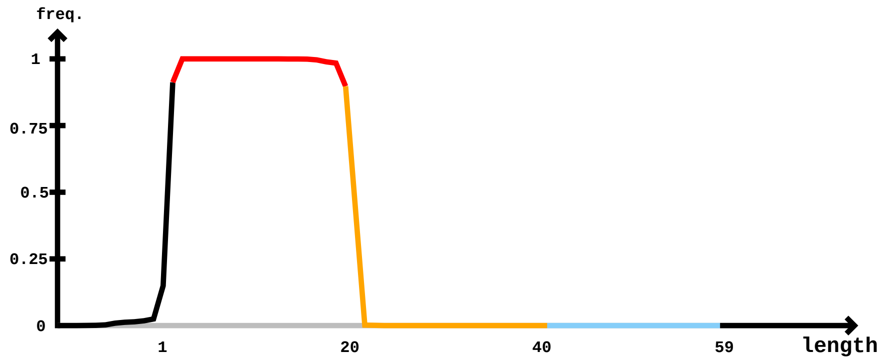

Mature Star

```
5'- cgguuggugcaaaaguaauugugguuuuugguuuugcuuucaauggcaaaaacuguaauucuuuuugcaccaaccuauaaa -3'   exp
    .((((((((((((((.(((((..(((((((.(.(((....))).).)))))))..))))).))))))))))))))......    reads   mm   sample
    .........caaaaguaauugugguuuu.....................................................    5       0    ya1
    .........caaaaguaauugugguuuuu....................................................    22      0    ya1
    .........caaaaguaauugugguuuuug...................................................    1       0    ya1
    ..........aaaaguaauugugguuuu.....................................................    16      0    ya1
    ..........aaaaguaauugugguuuuu....................................................    111     0    ya1
    ..........aaaaguaauugugguuuuug...................................................    8       0    ya1
    ...........aaaguaauugugguuuuu....................................................    19      0    ya1

    ..........aaaaguaauugugguuuuug...................................................    1       0    s04

    ..........aaaaguaauugugguuuuug...................................................    1       0    j26

    ....uggugcaaaaguaauugugguu.......................................................    1       0    va1
    ..........aaaaguaauugugguuuuu....................................................    2       0    va1
    ..........aaaaguaauugugguuuuug...................................................    25      0    va1
    ...........aaaguaauugugguuuuug...................................................    2       0    va1

    ...uuggugcaaaaguaauugugguuu......................................................    1       0    ku5
    ....uggugcaaaaguaauuguggu........................................................    1       0    ku5
    ......gugcaaaaguaauugugguu.......................................................    1       0    ku5
    .........caaaaguaauugugguuuuug...................................................    1       0    ku5
    ..........aaaaguaauugugguuuuug...................................................    3       0    ku5

    .........caaaaguaauugugguuuuu....................................................    2       0    k05
    .........caaaaguaauugugguuuuug...................................................    2       0    k05
    ..........aaaaguaauugugguuuuug...................................................    3       0    k05
    ...........aaaguaauugugguuuuug...................................................    1       0    k05

    .........caaaaguaauugugguuuuug...................................................    1       0    s06

    ......gugcaaaaguaauugugguu.......................................................    1       0    ku4
    ..........aaaaguaauugugguuuuu....................................................    5       0    ku4
    ..........aaaaguaauugugguuuuug...................................................    11      0    ku4

    ..........aaaaguaauugugguuuuug...................................................    2       0    s10
```

Mature **Star**

```
cgguuggugcaaaaguaauugugguuuuugguuuugcuuucaauggcaaaaacuguaauucuuuuugcaccaaccuauaaa

.........caaaaguaauugugguuuuu....................................................    2    0    k08
.........caaaaguaauugugguuuuug...................................................    1    0    k08
..........aaaaguaauugugguuuuu....................................................    1    0    k08
..........aaaaguaauugugguuuuug...................................................    1    0    k08

..........aaaaguaauugugguuuu.....................................................    1    0    ta3
..........aaaaguaauugugguuuuug...................................................    7    0    ta3
...........aaaguaauugugguuuuug...................................................    1    0    ta3

..........aaaaguaauugugguuuuu....................................................    2    0    j10
..........aaaaguaauugugguuuuug...................................................    6    0    j10

..........aaaaguaauugugguuuuug...................................................    3    0    j04

..........aaaaguaauugugguuuu.....................................................    1    0    j02
..........aaaaguaauugugguuuuu....................................................    5    0    j02
..........aaaaguaauugugguuuuug...................................................    22   0    j02
...........aaaguaauugugguuuuug...................................................    1    0    j02

.........caaaaguaauugugguuuuu....................................................    1    0    va2
..........aaaaguaauugugguuuuu....................................................    1    0    va2
..........aaaaguaauugugguuuuug...................................................    4    0    va2

.........caaaaguaauugugguuuuu....................................................    2    0    k14
.........caaaaguaauugugguuuuug...................................................    4    0    k14
..........aaaaguaauugugguuuuu....................................................    2    0    k14
..........aaaaguaauugugguuuuug...................................................    4    0    k14
..........aaaaguaauugugguuuuugg..................................................    1    0    k14
...........aaaguaauugugguuuuug...................................................    1    0    k14

.....ggugcaaaaguaauugugguuu......................................................    1    0    j27
.........caaaaguaauugugguuuuu....................................................    1    0    j27
.........caaaaguaauugugguuuuug...................................................    4    0    j27
..........aaaaguaauugugguuuu.....................................................    1    0    j27
..........aaaaguaauugugguuuuu....................................................    2    0    j27
..........aaaaguaauugugguuuuug...................................................    13   0    j27

...uuggugcaaaaguaauuguggu........................................................    1    0    j31
....uggugcaaaaguaauugugguu.......................................................    1    0    j31
.........caaaaguaauugugguuuuu....................................................    1    0    j31
.........caaaaguaauugugguuuuug...................................................    2    0    j31
..........aaaaguaauugugguuuuug...................................................    1    0    j31

..........aaaaguaauugugguuuuuggu.................................................    1    0    s03

..........aaaaguaauugugguuuuu....................................................    2    0    H52

.........caaaaguaauugugguuuuu....................................................    1    0    j22
.........caaaaguaauugugguuuuug...................................................    1    0    j22
..........aaaaguaauugugguuuuu....................................................    1    0    j22
..........aaaaguaauugugguuuuug...................................................    33   0    j22
...........aaaguaauugugguuuuug...................................................    3    0    j22

...uuggugcaaaaguaauugugg.........................................................    1    0    k09
.......ugcaaaaguaauugugguuuuu....................................................    1    0    k09
.........caaaaguaauugugguuuuu....................................................    1    0    k09
.........caaaaguaauugugguuuuug...................................................    2    0    k09
..........aaaaguaauugugguuuuu....................................................    2    0    k09
..........aaaaguaauugugguuuuug...................................................    4    0    k09

.........caaaaguaauugugguuuuug...................................................    3    0    B14

.........caaaaguaauugugguuuuug...................................................    1    0    j16
..........aaaaguaauugugguuuu.....................................................    1    0    j16
..........aaaaguaauugugguuuuu....................................................    12   0    j16
..........aaaaguaauugugguuuuug...................................................    25   0    j16

.........caaaaguaauugugguuuuu....................................................    3    0    j18
.........caaaaguaauugugguuuuug...................................................    1    0    j18
```

Mature Star

```
cgguuggugcaaaaguaauugugguuuuugguuuugcuuucaauggcaaaaacuguaauucuuuuugcaccaaccuauaaa
..........aaaaguaauugugguuuuug...................................................   1     0   j18

.........caaaaguaauugugguuuuu....................................................   2     0   j09
..........aaaaguaauugugguuuuu....................................................   4     0   j09
..........aaaaguaauugugguuuuug...................................................   9     0   j09
...........aaaguaauugugguuuuug...................................................   1     0   j09

....uggugcaaaaguaauuguggu........................................................   1     0   ku3
.........caaaaguaauugugguuuuug...................................................   1     0   ku3
..........aaaaguaauugugguuuuug...................................................   6     0   ku3

.........caaaaguaauugugguuuu.....................................................   1     0   ku6
.........caaaaguaauugugguuuuu....................................................   1     0   ku6
..........aaaaguaauugugguuuuug...................................................   2     0   ku6

..........aaaaguaauugugguuuuu....................................................   4     0   j11
..........aaaaguaauugugguuuuug...................................................   7     0   j11
...........aaaguaauugugguuuuug...................................................   1     0   j11

..........aaaaguaauugugguuuuug...................................................   4     0   s12
...........aaaguaauugugguuuuug...................................................   2     0   s12

........gcaaaaguaauugugguuuuu....................................................   2     0   ya3
.........caaaaguaauugugguuuu.....................................................   9     0   ya3
.........caaaaguaauugugguuuuu....................................................   22    0   ya3
..........aaaaguaauugugguuuu.....................................................   44    0   ya3
..........aaaaguaauugugguuuuu....................................................   238   0   ya3
..........aaaaguaauugugguuuuug...................................................   27    0   ya3
...........aaaguaauugugguuuuu....................................................   45    0   ya3
...........aaaguaauugugguuuuug...................................................   8     0   ya3

.........caaaaguaauugugguuuuu....................................................   1     0   la1
..........aaaaguaauugugguuuu.....................................................   1     0   la1
..........aaaaguaauugugguuuuu....................................................   1     0   la1
..........aaaaguaauugugguuuuug...................................................   3     0   la1

....uggugcaaaaguaauugugguu.......................................................   1     0   HEK

..........aaaaguaauugugguuuu.....................................................   1     0   j17
..........aaaaguaauugugguuuuu....................................................   8     0   j17
..........aaaaguaauugugguuuuug...................................................   8     0   j17

.........caaaaguaauugugguuuuug...................................................   4     0   j15
..........aaaaguaauugugguuuu.....................................................   1     0   j15
..........aaaaguaauugugguuuuu....................................................   3     0   j15
..........aaaaguaauugugguuuuug...................................................   16    0   j15
...........aaaguaauugugguuuuu....................................................   1     0   j15
...........aaaguaauugugguuuuug...................................................   1     0   j15

..........aaaaguaauugugguuuuu....................................................   1     0   la2
..........aaaaguaauugugguuuuug...................................................   3     0   la2

....uggugcaaaaguaauugugguu.......................................................   1     0   j12
.........caaaaguaauugugguuuuug...................................................   5     0   j12
..........aaaaguaauugugguuuuu....................................................   6     0   j12
..........aaaaguaauugugguuuuug...................................................   24    0   j12
...........aaaguaauugugguuuuug...................................................   2     0   j12

....uggugcaaaaguaauugugguu.......................................................   1     0   li2
.........caaaaguaauugugguuuuug...................................................   4     0   li2

.........caaaaguaauugugguuuuug...................................................   1     0   va4
..........aaaaguaauugugguuuuu....................................................   1     0   va4
..........aaaaguaauugugguuuuug...................................................   5     0   va4

.........caaaaguaauugugguuuuug...................................................   2     0   va3
..........aaaaguaauugugguuuuu....................................................   2     0   va3
..........aaaaguaauugugguuuuug...................................................   10    0   va3
...........aaaguaauugugguuuuug...................................................   2     0   va3

.........caaaaguaauugugguuuuu....................................................   1     0   SW4
```

Mature **Star**

cgguuggugcaaaaguaauugugguuuuugguuuugcuuucaauggcaaaaacuguaauucuuuuugcaccaaccuauaaa

..........aaaaguaauugugguuuuu..	1	0	SW4
..........aaaaguaauugugguuuuug...	3	0	SW4
.........caaaaguaauugugguuuu...	16	0	ya4
.........caaaaguaauugugguuuuu..	16	0	ya4
.........caaaaguaauugugguuuuug...	4	0	ya4
..........aaaaguaauugugguuuu...	33	0	ya4
..........aaaaguaauugugguuuuu..	213	0	ya4
..........aaaaguaauugugguuuuug...	5	0	ya4
...........aaaguaauugugguuuuu..	43	0	ya4
...........aaaguaauugugguuuuug...	3	0	ya4
.........caaaaguaauugugguuuuu..	1	0	ku1
.........caaaaguaauugugguuuuug...	1	0	ku1
..........aaaaguaauugugguuuuu..	5	0	ku1
..........aaaaguaauugugguuuuug...	38	0	ku1
...........aaaguaauugugguuuuug...	1	0	ku1
.......ugcaaaaguaauugugguuuuu..	1	0	j23
.........caaaaguaauugugguuuuug...	4	0	j23
..........aaaaguaauugugguuuu...	1	0	j23
..........aaaaguaauugugguuuuu..	8	0	j23
..........aaaaguaauugugguuuuug...	89	0	j23
...........aaaguaauugugguuuuug...	11	0	j23
..........aaaaguaauugugguuuuug...	7	0	s05
...........aaaguaauugugguuuuug...	1	0	s05
...........aaaguaauugugguuuuug...	1	0	ta2
.....ggugcaaaaguaauugugguuu..	5	0	j24
.........caaaaguaauugugguuuu...	3	0	j24
.........caaaaguaauugugguuuuug...	1	0	j24
..........aaaaguaauugugguuuuu..	3	0	j24
..........aaaaguaauugugguuuuug...	8	0	j24
.........caaaaguaauugugguuuuug...	1	0	k11
..........aaaaguaauugugguuuuu..	2	0	k11
..........aaaaguaauugugguuuuug...	2	0	k11
..........aaaaguaauugugguuuu...	1	0	ta1
..........aaaaguaauugugguuuuu..	1	0	ta1
..........aaaaguaauugugguuuuug...	3	0	ta1
...........aaaguaauugugguuuuug...	1	0	ta1
.........caaaaguaauugugguuuuug...	1	0	s09
..........aaaaguaauugugguuuuu..	1	0	s09
..........aaaaguaauugugguuuuug...	3	0	s09
...........aaaguaauugugguuuuug...	1	0	s09
.........caaaaguaauugugguuu..	1	0	NL2
.........caaaaguaauugugguuuu...	1	0	NL2
.........caaaaguaauugugguuuuu..	4	0	NL2
..........aaaaguaauugugguuuu...	10	0	NL2
..........aaaaguaauugugguuuuu..	2	0	NL2
..........aaaaguaauugugguuuuug...	2	0	NL2
..........aaaaguaauugugguuuuuggu...	1	0	NL2
....uggugcaaaaguaauugugguu...	1	0	k10
.........caaaaguaauugugguuuuu..	1	0	k10
.........caaaaguaauugugguuuuug...	2	0	k10
..........aaaaguaauugugguuuuug...	4	0	k10
.........caaaaguaauugugguuuuug...	1	0	j13
..........aaaaguaauugugguuuu...	1	0	j13
..........aaaaguaauugugguuuuu..	6	0	j13
..........aaaaguaauugugguuuuug...	33	0	j13
...........aaaguaauugugguuuuug...	1	0	j13
.........caaaaguaauugugguuuuu..	2	0	j19

```
          Mature                                  Star
cgguuggugcaaaaguaauugugguuuuugguuuugcuuucaauggcaaaaacuguaauucuuuuugcaccaaccuauaaa
.........caaaaguaauugugguuuuug...................................................   1    0    j19
..........aaaaguaauugugguuuu.....................................................   2    0    j19
..........aaaaguaauugugguuuuu....................................................   5    0    j19
..........aaaaguaauugugguuuuug...................................................   37   0    j19

..........aaaaguaauugugguuuuug...................................................   4    0    j03

..........aaaaguaauugugguuuuug...................................................   1    0    k01

....uggugcaaaaguaauugugguu.......................................................   1    0    j25
.......ugcaaaaguaauugugguu.......................................................   2    0    j25
.........caaaaguaauugugguuuu.....................................................   1    0    j25
..........aaaaguaauugugguuuuug...................................................   10   0    j25

........gcaaaaguaauugugguuuuug...................................................   1    0    per
.........caaaaguaauugugguuuuug...................................................   1    0    per
..........aaaaguaauugugguuuu.....................................................   1    0    per
..........aaaaguaauugugguuuuug...................................................   8    0    per
...........aaaguaauugugguuuuug...................................................   1    0    per

........gcaaaaguaauugugguuuuug...................................................   1    0    s01

.........caaaaguaauugugguuuuu....................................................   2    0    k04
.........caaaaguaauugugguuuuug...................................................   1    0    k04
..........aaaaguaauugugguuuu.....................................................   1    0    k04
..........aaaaguaauugugguuuuug...................................................   5    0    k04

....uggugcaaaaguaauuguggu........................................................   2    0    U20
....uggugcaaaaguaauugugguu.......................................................   1    0    U20
......gugcaaaaguaauugugguuuu.....................................................   1    0    U20
.........caaaaguaauugugguuuuug...................................................   1    0    U20
..........aaaaguaauugugguuuuu....................................................   1    0    U20
..........aaaaguaauugugguuuuug...................................................   6    0    U20

.........caaaaguaauugugguuuuu....................................................   2    0    ku2
..........aaaaguaauugugguuuuu....................................................   5    0    ku2
..........aaaaguaauugugguuuuug...................................................   16   0    ku2

.........caaaaguaauugugguuuuu....................................................   1    0    j21
.........caaaaguaauugugguuuuug...................................................   3    0    j21
..........aaaaguaauugugguuuuu....................................................   1    0    j21
..........aaaaguaauugugguuuuug...................................................   14   0    j21
...........aaaguaauugugguuuuug...................................................   3    0    j21

..........aaaaguaauugugguuuuug...................................................   1    0    s11

..........aaaaguaauugugguuuuu....................................................   4    0    j07
..........aaaaguaauugugguuuuug...................................................   2    0    j07
...........aaaguaauugugguuuuug...................................................   1    0    j07

.........caaaaguaauugugguuuuug...................................................   2    0    k02
..........aaaaguaauugugguuuuu....................................................   2    0    k02

.........caaaaguaauugugguuuuug...................................................   5    0    j14
..........aaaaguaauugugguuuuug...................................................   9    0    j14

.........caaaaguaauugugguuuuu....................................................   1    0    j28
..........aaaaguaauugugguuuuu....................................................   3    0    j28
..........aaaaguaauugugguuuuug...................................................   17   0    j28

..........aaaaguaauugugguuuuug...................................................   1    0    s08

..........aaaaguaauugugguuuuu....................................................   2    0    DLD
..........aaaaguaauugugguuuuug...................................................   1    0    DLD

..guuggugcaaaaguaauuguggu........................................................   1    0    k03
..........aaaaguaauugugguuuuug...................................................   5    0    k03

.........caaaaguaauugugguu.......................................................   1    0    NL3
.........caaaaguaauugugguuu......................................................   1    0    NL3
```

```
         Mature                                       Star
cgguuggugcaaaaguaauugugguuuuugguuuugcuuucaauggcaaaaacuguaauucuuuuugcaccaaccuauaaa
.........caaaaguaauugugguuuuu....................................................    4     0    NL3
.........caaaaguaauugugguuuuug...................................................    2     0    NL3
..........aaaaguaauugugguuuu.....................................................    15    0    NL3
..........aaaaguaauugugguuuuu....................................................    6     0    NL3
..........aaaaguaauugugguuuuug...................................................    5     0    NL3

.........caaaaguaauugugguuu......................................................    1     0    ya2
.........caaaaguaauugugguuuu.....................................................    6     0    ya2
.........caaaaguaauugugguuuuu....................................................    13    0    ya2
.........caaaaguaauugugguuuuug...................................................    2     0    ya2
..........aaaaguaauugugguuuu.....................................................    24    0    ya2
..........aaaaguaauugugguuuuu....................................................    188   0    ya2
..........aaaaguaauugugguuuuug...................................................    17    0    ya2
...........aaaguaauugugguuuuu....................................................    43    0    ya2
...........aaaguaauugugguuuuug...................................................    4     0    ya2

.........caaaaguaauugugguuuuug...................................................    1     0    s02

.........caaaaguaauugugguuuuug...................................................    3     0    k06
..........aaaaguaauugugguuuuug...................................................    2     0    k06

........gcaaaaguaauugugguuuuug...................................................    1     0    j29
.........caaaaguaauugugguuuuug...................................................    3     0    j29
..........aaaaguaauugugguuuuug...................................................    10    0    j29

.gguuggugcaaaaguaauugu...........................................................    1     0    sh2
...uuggugcaaaaguaauugugguu.......................................................    1     0    sh2
......gugcaaaaguaauugugguu.......................................................    1     0    sh2
.........caaaaguaauugugguuuuu....................................................    1     0    sh2
..........aaaaguaauugugguuuuug...................................................    1     0    sh2

.....ggugcaaaaguaauugugguuu......................................................    1     0    j01
.......ugcaaaaguaauugugguuuu.....................................................    3     0    j01
.........caaaaguaauugugguuuuug...................................................    1     0    j01
..........aaaaguaauugugguuuuug...................................................    5     0    j01

....uggugcaaaaguaauuguggu........................................................    1     0    MCF
..........aaaaguaauugugguuuuug...................................................    4     0    MCF

..........aaaaguaauugugguuuuug...................................................    2     0    MBM

..........aaaaguaauugugguuuuu....................................................    2     0    NL1
..........aaaaguaauugugguuuuug...................................................    2     0    NL1

.....ggugcaaaaguaauugugguu.......................................................    1     0    k07
.......ugcaaaaguaauugugguuuuu....................................................    1     0    k07
.........caaaaguaauugugguuuuug...................................................    9     0    k07
..........aaaaguaauugugguuuuug...................................................    3     0    k07

.......ugcaaaaguaauugugguuuuu....................................................    1     0    sh1
.........caaaaguaauugugguuuuug...................................................    6     0    sh1
..........aaaaguaauugugguuuuug...................................................    4     0    sh1

....uggugcaaaaguaauugugguu.......................................................    1     0    li1
.........caaaaguaauugugguuuuug...................................................    3     0    li1

.........caaaaguaauugugguuuuu....................................................    1     0    k13
.........caaaaguaauugugguuuuug...................................................    4     0    k13
..........aaaaguaauugugguuuuug...................................................    1     0    k13

.........caaaaguaauugugguuuu.....................................................    1     0    j20
.........caaaaguaauugugguuuuu....................................................    2     0    j20
.........caaaaguaauugugguuuuug...................................................    7     0    j20
..........aaaaguaauugugguuuuu....................................................    3     0    j20
..........aaaaguaauugugguuuuug...................................................    27    0    j20
..........aaaaguaauugugguuuuugg..................................................    1     0    j20

........gcaaaaguaauugugguuuuug...................................................    11    0    A54
..........aaaaguaauugugguuuuug...................................................    3     0    A54

....uggugcaaaaguaauugugguu.......................................................    1     0    j30
```

Mature   Star

```
cggguuggugcaaaaguaauuguggguuuuuggguuuugcuuucaauggcaaaaacuguaauucuuuuugcaccaaccuauaaa
..........aaaaguaauuguggguuuuug..................................................    2     0     j30

.........caaaaguaauuguggguuuuu...................................................    8     0     k12
.........caaaaguaauuguggguuuuug..................................................    12    0     k12
..........aaaaguaauuguggguuuuu...................................................    6     0     k12
..........aaaaguaauuguggguuuuug..................................................    18    0     k12
```

Provisional ID	:	hsa_can_1081
Score total	:	4.5
Score for star read(s)	:	3.9
Score for read counts	:	-1.3
Score for mfe	:	2.6
Score for randfold	:	
Score for cons. seed	:	-0.6
Total read count	:	9
Mature read count	:	8
Loop read count	:	0
Star read count	:	1

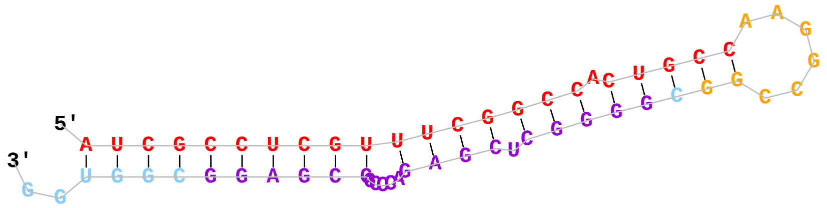

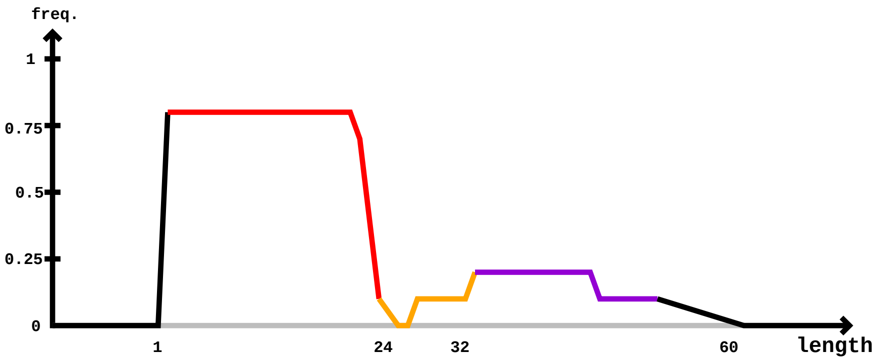

Mature **Star**

```
5'- ccccacccccaucgccucguuucggccacugccaaggccggcggggcucgagacccggcgaggcgguggggguggggguag  -3'   obs
    ccccacccccaucgccucguuucggccacugccaaggccggcggggcucgagacccggcgaggcgguggggguggggguag         exp
    ((((((((((((((((((((((((.(((((......)))))))).)))).....))))))))))))))))))))....     reads   mm   sample
    .........aucgccucguuucggccacugc...............................................     1       0    sh1
    .........aucgccucguuucggccacu.................................................     1       0    ya1
    .........aucgccucguuucggccacugc...............................................     1       0    li1
    .........aucgccucguuucggccacug................................................     1       0    s05
    .........aucgccucguuucggccacugc...............................................     1       0    k01
    .........aucgccucguuucggccacug................................................     1       0    j02
    ......................................ggggcucgagacccggcgagg.................     1       0    s11
    .........aucgccucguuucggccacugcc..............................................     1       0    k02
    ..................................gccggcggggcucgagacc.........................     1       0    sh2
    .........aucgccucguuucggccacug................................................     1       0    ku1
```

Provisional ID	:	hsa_can_1109
Score total	:	1811.7
Score for star read(s)	:	3.9
Score for read counts	:	1802.9
Score for mfe	:	1.9
Score for randfold	:	
Score for cons. seed	:	3
Total read count	:	3548
Mature read count	:	3542
Loop read count	:	0
Star read count	:	6

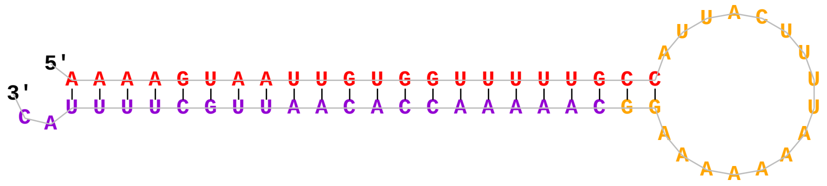

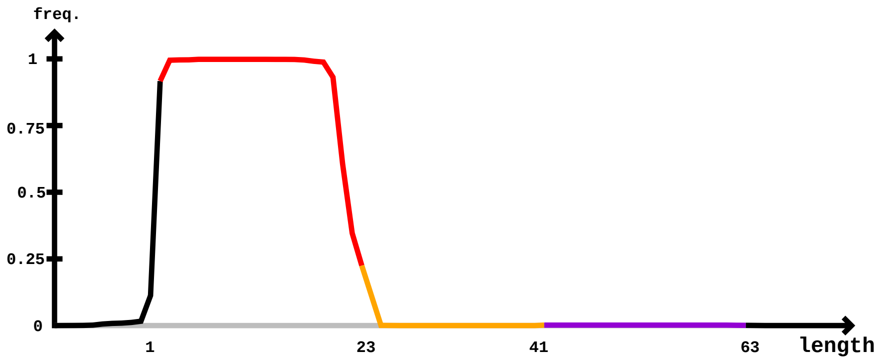

Mature **Star**

```
5'- gguuggugcaaaaguaauugugguuuuugccauuacuuuuaaaaaaaggcaaaaaccacaauugcuuuuacaucaaccuaa  -3'   obs
    gguuggugcaaaaguaauugugguuuuugccauuacuuuuaaaaaaaggcaaaaaccacaauugcuuuuacaucaaccuaa         exp
    gguuggugcaaaaguaauugugguuuuugcauuacuuuuaaaaaaaggcaaaaaccacaauugcuuuuacaucaaccuaa          known
    ((((((((.((((((((((((((((((((((................))))))))))))))))))))))).))))))))...    reads    mm    sample
    ........caaaaguaauugugguuuu......................................................    5        0     ya1
    ........caaaaguaauugugguuuuu.....................................................    22       0     ya1
    ........caaaaguaauugugguuuuug....................................................    1        0     ya1
    .........aaaaguaauugugguuuu......................................................    16       0     ya1
    .........aaaaguaauugugguuuuu.....................................................    111      0     ya1
    .........aaaaguaauugugguuuuug....................................................    8        0     ya1
    ..........aaaguaauugugguuuuu.....................................................    19       0     ya1

    .........aaaaguaauugugguuuuug....................................................    1        0     s04

    .........aaaaguaauugugguuuuug....................................................    1        0     j26
    .................................................caaaaaccacaauugcuuuu............    1        0     j26

    ...uggugcaaaaguaauugugguu........................................................    1        0     va1
    ........caaaaguaauugugguuuuugc...................................................    1        0     va1
    .........aaaaguaauugugguuuuu.....................................................    2        0     va1
    .........aaaaguaauugugguuuuug....................................................    25       0     va1
    .........aaaaguaauugugguuuuugc...................................................    55       0     va1
    .........aaaaguaauugugguuuuugcc..................................................    35       0     va1
    .........aaaaguaauugugguuuuugcca.................................................    3        0     va1
    ..........aaaguaauugugguuuuug....................................................    2        0     va1
    ..........aaaguaauugugguuuuugc...................................................    1        0     va1
    ..........aaaguaauugugguuuuugcc..................................................    1        0     va1

    ..uuggugcaaaaguaauugugguuu.......................................................    1        0     ku5
    ...uggugcaaaaguaauuguggu.........................................................    1        0     ku5
    .....gugcaaaaguaauugugguu........................................................    1        0     ku5
    ........caaaaguaauugugguuuuug....................................................    1        0     ku5
    .........aaaaguaauugugguuuuug....................................................    3        0     ku5

    ........caaaaguaauugugguuuuu.....................................................    2        0     k05
    ........caaaaguaauugugguuuuug....................................................    2        0     k05
```

```
         Mature                                  Star
gguuggugcaaaaguaauugugguuuuugccauuacuuuuaaaaaaaggcaaaaaccacaauugcuuuuacaucaaccuaa
........caaaaguaauugugguuuuugc...................................................    1    0    k05
.........aaaaguaauugugguuuuug....................................................    3    0    k05
.........aaaaguaauugugguuuuugc...................................................    1    0    k05
..........aaaguaauugugguuuuug....................................................    1    0    k05

........caaaaguaauugugguuuuug....................................................    1    0    s06
.........aaaaguaauugugguuuuugc...................................................    1    0    s06
.........aaaaguaauugugguuuuugcc..................................................    2    0    s06

.........aaaaguaauugugguuuuugc...................................................    5    0    HeL
.........aaaaguaauugugguuuuugcc..................................................    49   0    HeL
.........aaaaguaauugugguuuuugcca.................................................    6    0    HeL
..........aaaguaauugugguuuuugc...................................................    1    0    HeL
..........aaaguaauugugguuuuugcc..................................................    1    0    HeL
...........aaguaauugugguuuuugccauu...............................................    1    0    HeL
.............guaauugugguuuuugcc..................................................    5    0    HeL

.....gugcaaaaguaauugugguu........................................................    1    0    ku4
.........aaaaguaauugugguuuu......................................................    5    0    ku4
.........aaaaguaauugugguuuuug....................................................    11   0    ku4

.........aaaaguaauugugguuuuugcc..................................................    1    0    s07

.........aaaaguaauugugguuuuug....................................................    2    0    s10
.........aaaaguaauugugguuuuugc...................................................    1    0    s10
.................................................caaaaaccacaauugcuuuua...........    1    0    s10
.................................................caaaaaccacaauugcuuuuac..........    2    0    s10

........caaaaguaauugugguuuu......................................................    2    0    k08
........caaaaguaauugugguuuuug....................................................    1    0    k08
.........aaaaguaauugugguuuu......................................................    1    0    k08
.........aaaaguaauugugguuuuug....................................................    1    0    k08
..........aaaguaauugugguuuuugcc..................................................    1    0    k08

.........aaaaguaauugugguuu.......................................................    1    0    ta3
.........aaaaguaauugugguuuuug....................................................    7    0    ta3
.........aaaaguaauugugguuuuugc...................................................    1    0    ta3
..........aaaguaauugugguuuuug....................................................    1    0    ta3

........caaaaguaauugugguuuuugc...................................................    1    0    j10
.........aaaaguaauugugguuuu......................................................    2    0    j10
.........aaaaguaauugugguuuuug....................................................    6    0    j10
.........aaaaguaauugugguuuuugc...................................................    5    0    j10

.........aaaaguaauugugguuuuug....................................................    3    0    j04

.........aaaaguaauugugguuu.......................................................    1    0    j02
.........aaaaguaauugugguuuu......................................................    5    0    j02
.........aaaaguaauugugguuuuug....................................................    22   0    j02
..........aaaguaauugugguuuuug....................................................    1    0    j02

........caaaaguaauugugguuuu......................................................    1    0    va2
........caaaaguaauugugguuuuugc...................................................    1    0    va2
.........aaaaguaauugugguuuu......................................................    1    0    va2
.........aaaaguaauugugguuuuug....................................................    4    0    va2
.........aaaaguaauugugguuuuugc...................................................    4    0    va2
..........aaaguaauugugguuuuugc...................................................    1    0    va2

........caaaaguaauugugguuuu......................................................    2    0    k14
........caaaaguaauugugguuuuug....................................................    4    0    k14
.........aaaaguaauugugguuuu......................................................    2    0    k14
.........aaaaguaauugugguuuuug....................................................    4    0    k14
..........aaaguaauugugguuuuug....................................................    1    0    k14

....ggugcaaaaguaauugugguu........................................................    1    0    j27
........caaaaguaauugugguuuu......................................................    1    0    j27
........caaaaguaauugugguuuuug....................................................    4    0    j27
.........aaaaguaauugugguuu.......................................................    1    0    j27
.........aaaaguaauugugguuuu......................................................    2    0    j27
.........aaaaguaauugugguuuuug....................................................    13   0    j27
```

```
         Mature                                      Star
gguuggugcaaaaguaauugugguuuuugccauuacuuuuaaaaaaaggcaaaaaccacaauugcuuuuacaucaaccuaa
..........aaaguaauugugguuuuugc...................................................    1    0    j27

..uuggugcaaaaguaauuguggu.........................................................    1    0    j31
...uggugcaaaaguaauugugguu........................................................    1    0    j31
........caaaaguaauugugguuuu......................................................    1    0    j31
........caaaaguaauugugguuuuug....................................................    2    0    j31
.........aaaaguaauugugguuuuug....................................................    1    0    j31
.........aaaaguaauugugguuuuugcc..................................................    1    0    j31

.........aaaaguaauugugguuuuugc...................................................    2    0    s03

........caaaaguaauugugguuuuugc...................................................    1    0    H52
.........aaaaguaauugugguuuu......................................................    2    0    H52
.........aaaaguaauugugguuuuugc...................................................    4    0    H52
.........aaaaguaauugugguuuuugcc..................................................    16   0    H52
..........aaaguaauugugguuuuugcca.................................................    1    0    H52

........caaaaguaauugugguuuu......................................................    1    0    j22
........caaaaguaauugugguuuuug....................................................    1    0    j22
........caaaaguaauugugguuuuugc...................................................    2    0    j22
.........aaaaguaauugugguuuu......................................................    1    0    j22
.........aaaaguaauugugguuuuug....................................................    33   0    j22
.........aaaaguaauugugguuuuugc...................................................    1    0    j22
.........aaaaguaauugugguuuuugcc..................................................    2    0    j22
..........aaaguaauugugguuuuug....................................................    3    0    j22

..uuggugcaaaaguaauugugg..........................................................    1    0    k09
......ugcaaaaguaauugugguuuu......................................................    1    0    k09
........caaaaguaauugugguuuu......................................................    1    0    k09
........caaaaguaauugugguuuuug....................................................    2    0    k09
.........aaaaguaauugugguuuu......................................................    2    0    k09
.........aaaaguaauugugguuuuug....................................................    4    0    k09
.........aaaaguaauugugguuuuugc...................................................    1    0    k09
.........aaaaguaauugugguuuuugcc..................................................    1    0    k09

........caaaaguaauugugguuuuug....................................................    3    0    B14
........caaaaguaauugugguuuuugc...................................................    1    0    B14
.........aaaaguaauugugguuuuugc...................................................    6    0    B14
.........aaaaguaauugugguuuuugcc..................................................    15   0    B14
..........aaaguaauugugguuuuugcc..................................................    1    0    B14

........caaaaguaauugugguuuuug....................................................    1    0    j16
.........aaaaguaauugugguuu.......................................................    1    0    j16
.........aaaaguaauugugguuuu......................................................    12   0    j16
.........aaaaguaauugugguuuuug....................................................    25   0    j16
..........aaaguaauugugguuuuugc...................................................    1    0    j16

........caaaaguaauugugguuuu......................................................    3    0    j18
........caaaaguaauugugguuuuug....................................................    1    0    j18
.........aaaaguaauugugguuuuug....................................................    1    0    j18

........caaaaguaauugugguuuu......................................................    2    0    j09
.........aaaaguaauugugguuuu......................................................    4    0    j09
.........aaaaguaauugugguuuuug....................................................    9    0    j09
..........aaaguaauugugguuuuug....................................................    1    0    j09

...uggugcaaaaguaauuguggu.........................................................    1    0    ku3
........caaaaguaauugugguuuuug....................................................    1    0    ku3
.........aaaaguaauugugguuuuug....................................................    6    0    ku3

........caaaaguaauugugguuu.......................................................    1    0    ku6
........caaaaguaauugugguuuu......................................................    1    0    ku6
.........aaaaguaauugugguuuuug....................................................    2    0    ku6

.........aaaaguaauugugguuuu......................................................    4    0    j11
.........aaaaguaauugugguuuuug....................................................    7    0    j11
..........aaaguaauugugguuuuug....................................................    1    0    j11

.........aaaaguaauugugguuuuug....................................................    4    0    s12
..........aaaguaauugugguuuuug....................................................    2    0    s12
```

Mature Star

```
gguuggugcaaaaguaauugugguuuuugccauuacuuuuaaaaaaaggcaaaaaccacaauugcuuuuacaucaaccuaa

.......gcaaaaguaauugugguuuuu.....................................................    2      0     ya3
........caaaaguaauugugguuuu......................................................    9      0     ya3
........caaaaguaauugugguuuuu.....................................................    22     0     ya3
.........aaaaguaauugugguuuu......................................................    44     0     ya3
.........aaaaguaauugugguuuuu.....................................................    238    0     ya3
.........aaaaguaauugugguuuuug....................................................    27     0     ya3
.........aaaaguaauugugguuuuugc...................................................    1      0     ya3
..........aaaguaauugugguuuuu.....................................................    45     0     ya3
..........aaaguaauugugguuuuug....................................................    8      0     ya3

........caaaaguaauugugguuuuu.....................................................    1      0     la1
........caaaaguaauugugguuuuugc...................................................    1      0     la1
.........aaaaguaauugugguuuu......................................................    1      0     la1
.........aaaaguaauugugguuuuu.....................................................    1      0     la1
.........aaaaguaauugugguuuuug....................................................    3      0     la1
.........aaaaguaauugugguuuuugcc..................................................    1      0     la1

...uggugcaaaaguaauugugguu........................................................    1      0     HEK
........caaaaguaauugugguuuuugc...................................................    7      0     HEK
.........aaaaguaauugugguuuuugc...................................................    9      0     HEK
.........aaaaguaauugugguuuuugcc..................................................    75     0     HEK
..........aaaguaauugugguuuuugcc..................................................    2      0     HEK
..........aaaguaauugugguuuuugcca.................................................    1      0     HEK
...........aaguaauugugguuuuugcc..................................................    1      0     HEK
...........aaguaauugugguuuuugccau................................................    1      0     HEK

.........aaaaguaauugugguuuu......................................................    1      0     j17
.........aaaaguaauugugguuuuu.....................................................    8      0     j17
.........aaaaguaauugugguuuuug....................................................    8      0     j17

........caaaaguaauugugguuuuug....................................................    4      0     j15
........caaaaguaauugugguuuuugc...................................................    1      0     j15
.........aaaaguaauugugguuuu......................................................    1      0     j15
.........aaaaguaauugugguuuuu.....................................................    3      0     j15
.........aaaaguaauugugguuuuug....................................................    16     0     j15
..........aaaguaauugugguuuuu.....................................................    1      0     j15
..........aaaguaauugugguuuuug....................................................    1      0     j15

.........aaaaguaauugugguuuuu.....................................................    1      0     la2
.........aaaaguaauugugguuuuug....................................................    3      0     la2
.........aaaaguaauugugguuuuugcc..................................................    6      0     la2
..........aaaguaauugugguuuuugcc..................................................    1      0     la2

...uggugcaaaaguaauugugguu........................................................    1      0     j12
........caaaaguaauugugguuuuug....................................................    5      0     j12
.........aaaaguaauugugguuuuu.....................................................    6      0     j12
.........aaaaguaauugugguuuuug....................................................    24     0     j12
.........aaaaguaauugugguuuuugc...................................................    2      0     j12
.........aaaaguaauugugguuuuugcc..................................................    1      0     j12
..........aaaguaauugugguuuuug....................................................    2      0     j12

...uggugcaaaaguaauugugguu........................................................    1      0     li2
........caaaaguaauugugguuuuug....................................................    4      0     li2
........caaaaguaauugugguuuuugc...................................................    9      0     li2
.........aaaaguaauugugguuuuugc...................................................    56     0     li2
.........aaaaguaauugugguuuuugcc..................................................    3      0     li2
..........aaaguaauugugguuuuugc...................................................    3      0     li2

........caaaaguaauugugguuuuug....................................................    1      0     va4
........caaaaguaauugugguuuuugc...................................................    1      0     va4
.........aaaaguaauugugguuuuu.....................................................    1      0     va4
.........aaaaguaauugugguuuuug....................................................    5      0     va4
.........aaaaguaauugugguuuuugc...................................................    11     0     va4
.........aaaaguaauugugguuuuugcc..................................................    11     0     va4

........caaaaguaauugugguuuuug....................................................    2      0     va3
........caaaaguaauugugguuuuugc...................................................    1      0     va3
.........aaaaguaauugugguuuuu.....................................................    2      0     va3
.........aaaaguaauugugguuuuug....................................................    10     0     va3
```

```
         Mature                                 Star
gguuggugcaaaaguaauugugguuuuugccauuacuuuuaaaaaaaggcaaaaaccacaauugcuuuuacaucaaccuaa
.........aaaaguaauugugguuuuugc...................................................    13      0      va3
.........aaaaguaauugugguuuuugcc..................................................    3       0      va3
..........aaaguaauugugguuuuug....................................................    2       0      va3
..........aaaguaauugugguuuuugcc..................................................    1       0      va3

........caaaaguaauugugguuuuu.....................................................    1       0      SW4
........caaaaguaauugugguuuuugc...................................................    1       0      SW4
.........aaaaguaauugugguuuuu.....................................................    1       0      SW4
.........aaaaguaauugugguuuuug....................................................    3       0      SW4
.........aaaaguaauugugguuuuugc...................................................    12      0      SW4
.........aaaaguaauugugguuuuugcc..................................................    20      0      SW4
..........aaaguaauugugguuuuugc...................................................    1       0      SW4
..........aaaguaauugugguuuuugcca.................................................    1       0      SW4

........caaaaguaauugugguuuu......................................................    16      0      ya4
........caaaaguaauugugguuuuu.....................................................    16      0      ya4
........caaaaguaauugugguuuuug....................................................    4       0      ya4
.........aaaaguaauugugguuuu......................................................    33      0      ya4
.........aaaaguaauugugguuuuu.....................................................    213     0      ya4
.........aaaaguaauugugguuuuug....................................................    5       0      ya4
.........aaaaguaauugugguuuuugcc..................................................    4       0      ya4
..........aaaguaauugugguuuuu.....................................................    43      0      ya4
..........aaaguaauugugguuuuug....................................................    3       0      ya4

........caaaaguaauugugguuuuu.....................................................    1       0      ku1
........caaaaguaauugugguuuuug....................................................    1       0      ku1
........caaaaguaauugugguuuuugc...................................................    1       0      ku1
.........aaaaguaauugugguuuuu.....................................................    5       0      ku1
.........aaaaguaauugugguuuuug....................................................    38      0      ku1
.........aaaaguaauugugguuuuugc...................................................    1       0      ku1
..........aaaguaauugugguuuuug....................................................    1       0      ku1

......ugcaaaaguaauugugguuuuu.....................................................    1       0      j23
........caaaaguaauugugguuuuug....................................................    4       0      j23
.........aaaaguaauugugguuuu......................................................    1       0      j23
.........aaaaguaauugugguuuuu.....................................................    8       0      j23
.........aaaaguaauugugguuuuug....................................................    89      0      j23
.........aaaaguaauugugguuuuugc...................................................    5       0      j23
.........aaaaguaauugugguuuuugcc..................................................    13      0      j23
..........aaaguaauugugguuuuug....................................................    11      0      j23
..........aaaguaauugugguuuuugc...................................................    1       0      j23
..........aaaguaauugugguuuuugcc..................................................    2       0      j23

.........aaaaguaauugugguuuuug....................................................    7       0      s05
.........aaaaguaauugugguuuuugc...................................................    1       0      s05
..........aaaguaauugugguuuuug....................................................    1       0      s05
.................................................caaaaaccacaauugcuuuu............    1       0      s05

.........aaaaguaauugugguuuuugc...................................................    4       0      ta2
..........aaaguaauugugguuuuug....................................................    1       0      ta2

....ggugcaaaaguaauugugguuu.......................................................    5       0      j24
........caaaaguaauugugguuuu......................................................    3       0      j24
........caaaaguaauugugguuuuug....................................................    1       0      j24
.........aaaaguaauugugguuuuu.....................................................    3       0      j24
.........aaaaguaauugugguuuuug....................................................    8       0      j24

........caaaaguaauugugguuuuug....................................................    1       0      k11
.........aaaaguaauugugguuuuu.....................................................    2       0      k11
.........aaaaguaauugugguuuuug....................................................    2       0      k11
.........aaaaguaauugugguuuuugc...................................................    3       0      k11

.........aaaaguaauugugguuuu......................................................    1       0      ta1
.........aaaaguaauugugguuuuu.....................................................    1       0      ta1
.........aaaaguaauugugguuuuug....................................................    3       0      ta1
.........aaaaguaauugugguuuuugcc..................................................    1       0      ta1
..........aaaguaauugugguuuuug....................................................    1       0      ta1

........caaaaguaauugugguuuuug....................................................    1       0      s09
.........aaaaguaauugugguuuuu.....................................................    1       0      s09
```

```
         Mature                                  Star
gguuggugcaaaaguaauugugguuuuugccauuacuuuuaaaaaaaggcaaaaaccacaauugcuuuuacaucaaccuaa
.........aaaaguaauugugguuuuug....................................................    3     0     s09
..........aaaguaauugugguuuuug....................................................    1     0     s09

........caaaaguaauugugguuu.......................................................    1     0     NL2
........caaaaguaauugugguuuu......................................................    1     0     NL2
........caaaaguaauugugguuuuu.....................................................    4     0     NL2
.........aaaaguaauugugguuuu......................................................    10    0     NL2
.........aaaaguaauugugguuuuu.....................................................    2     0     NL2
.........aaaaguaauugugguuuuug....................................................    2     0     NL2

...uggugcaaaaguaauugugguu........................................................    1     0     k10
........caaaaguaauugugguuuuu.....................................................    1     0     k10
........caaaaguaauugugguuuuug....................................................    2     0     k10
.........aaaaguaauugugguuuuug....................................................    4     0     k10
.........aaaaguaauugugguuuuugc...................................................    2     0     k10

........caaaaguaauugugguuuuug....................................................    1     0     j13
........caaaaguaauugugguuuuugc...................................................    1     0     j13
.........aaaaguaauugugguuuu......................................................    1     0     j13
.........aaaaguaauugugguuuuu.....................................................    6     0     j13
.........aaaaguaauugugguuuuug....................................................    33    0     j13
.........aaaaguaauugugguuuuugc...................................................    4     0     j13
.........aaaaguaauugugguuuuugcc..................................................    1     0     j13
..........aaaguaauugugguuuuug....................................................    1     0     j13
..........aaaguaauugugguuuuugc...................................................    1     0     j13

........caaaaguaauugugguuuuu.....................................................    2     0     j19
........caaaaguaauugugguuuuug....................................................    1     0     j19
.........aaaaguaauugugguuuu......................................................    2     0     j19
.........aaaaguaauugugguuuuu.....................................................    5     0     j19
.........aaaaguaauugugguuuuug....................................................    37    0     j19
.........aaaaguaauugugguuuuugc...................................................    2     0     j19

.........aaaaguaauugugguuuuug....................................................    4     0     j03

.........aaaaguaauugugguuuuug....................................................    1     0     k01
.........aaaaguaauugugguuuuugc...................................................    3     0     k01

...uggugcaaaaguaauugugguu........................................................    1     0     j25
......ugcaaaaguaauugugguu........................................................    2     0     j25
........caaaaguaauugugguuuu......................................................    1     0     j25
.........aaaaguaauugugguuuuug....................................................    10    0     j25

.......gcaaaaguaauugugguuuuug....................................................    1     0     per
........caaaaguaauugugguuuuug....................................................    1     0     per
.........aaaaguaauugugguuuu......................................................    1     0     per
.........aaaaguaauugugguuuuug....................................................    8     0     per
..........aaaguaauugugguuuuug....................................................    1     0     per

.......gcaaaaguaauugugguuuuug....................................................    1     0     s01

........caaaaguaauugugguuuuu.....................................................    2     0     k04
........caaaaguaauugugguuuuug....................................................    1     0     k04
.........aaaaguaauugugguuuu......................................................    1     0     k04
.........aaaaguaauugugguuuuug....................................................    5     0     k04
.........aaaaguaauugugguuuuugc...................................................    1     0     k04

...uggugcaaaaguaauugugg..........................................................    2     0     U20
...uggugcaaaaguaauugugguu........................................................    1     0     U20
.....gugcaaaaguaauugugguuuu......................................................    1     0     U20
........caaaaguaauugugguuuuug....................................................    1     0     U20
........caaaaguaauugugguuuuugc...................................................    18    0     U20
........caaaaguaauugugguuuuugcc..................................................    1     0     U20
.........aaaaguaauugugguuuuu.....................................................    1     0     U20
.........aaaaguaauugugguuuuug....................................................    6     0     U20
.........aaaaguaauugugguuuuugc...................................................    48    0     U20
.........aaaaguaauugugguuuuugcc..................................................    243   0     U20
.........aaaaguaauugugguuuuugcca.................................................    3     0     U20
..........aaaguaauugugguuuuugc...................................................    11    0     U20
..........aaaguaauugugguuuuugcc..................................................    24    0     U20
```

Mature Star

```
gguuggugcaaaaguaauugugguuuuugccauuacuuuuaaaaaaaggcaaaaaccacaauugcuuuuacaucaaccuaa
..........aaaguaauugugguuuuugcca.................................................   1    0    U20
............aguaauugugguuuuugc...................................................   1    0    U20
.................................................caaaaaccacaauugcuuuuac..........   1    0    U20

........caaaaguaauugugguuuuu.....................................................   2    0    ku2
.........aaaaguaauugugguuuuu.....................................................   5    0    ku2
.........aaaaguaauugugguuuuug....................................................   16   0    ku2
.........aaaaguaauugugguuuuugc...................................................   1    0    ku2
.........aaaaguaauugugguuuuugcc..................................................   1    0    ku2

........caaaaguaauugugguuuuu.....................................................   1    0    j21
........caaaaguaauugugguuuuug....................................................   3    0    j21
.........aaaaguaauugugguuuuu.....................................................   1    0    j21
.........aaaaguaauugugguuuuug....................................................   14   0    j21
.........aaaaguaauugugguuuuugc...................................................   3    0    j21
.........aaaaguaauugugguuuuugcc..................................................   2    0    j21
..........aaaguaauugugguuuuug....................................................   3    0    j21
..........aaaguaauugugguuuuugcc..................................................   2    0    j21


.........aaaaguaauugugguuuuug....................................................   1    0    s11

.........aaaaguaauugugguuuuu.....................................................   4    0    j07
.........aaaaguaauugugguuuuug....................................................   2    0    j07
..........aaaguaauugugguuuuug....................................................   1    0    j07

........caaaaguaauugugguuuuug....................................................   5    0    j14
.........aaaaguaauugugguuuuug....................................................   9    0    j14
.........aaaaguaauugugguuuuugc...................................................   5    0    j14
.........aaaaguaauugugguuuuugcc..................................................   1    0    j14

........caaaaguaauugugguuuuug....................................................   2    0    k02
.........aaaaguaauugugguuuuu.....................................................   2    0    k02
.........aaaaguaauugugguuuuugc...................................................   1    0    k02

........caaaaguaauugugguuuuu.....................................................   1    0    j28
.........aaaaguaauugugguuuuu.....................................................   3    0    j28
.........aaaaguaauugugguuuuug....................................................   17   0    j28

.........aaaaguaauugugguuuuug....................................................   1    0    s08

........caaaaguaauugugguuuuugc...................................................   1    0    DLD
.........aaaaguaauugugguuuuu.....................................................   2    0    DLD
.........aaaaguaauugugguuuuug....................................................   1    0    DLD
.........aaaaguaauugugguuuuugc...................................................   13   0    DLD
.........aaaaguaauugugguuuuugcc..................................................   73   0    DLD
.........aaaaguaauugugguuuuugcca.................................................   1    0    DLD
..........aaaguaauugugguuuuugcc..................................................   4    0    DLD
.............guaauugugguuuuugcc..................................................   1    0    DLD

.guuggugcaaaaguaauuguggu.........................................................   1    0    k03
.........aaaaguaauugugguuuuug....................................................   5    0    k03
.........aaaaguaauugugguuuuugc...................................................   3    0    k03

........caaaaguaauugugguuu.......................................................   1    0    NL3
........caaaaguaauugugguuuu......................................................   1    0    NL3
........caaaaguaauugugguuuuu.....................................................   4    0    NL3
........caaaaguaauugugguuuuug....................................................   2    0    NL3
.........aaaaguaauugugguuuu......................................................   15   0    NL3
.........aaaaguaauugugguuuuu.....................................................   6    0    NL3
.........aaaaguaauugugguuuuug....................................................   5    0    NL3

........caaaaguaauugugguuu.......................................................   1    0    ya2
........caaaaguaauugugguuuu......................................................   6    0    ya2
........caaaaguaauugugguuuuu.....................................................   13   0    ya2
........caaaaguaauugugguuuuug....................................................   2    0    ya2
.........aaaaguaauugugguuuu......................................................   24   0    ya2
.........aaaaguaauugugguuuuu.....................................................   188  0    ya2
.........aaaaguaauugugguuuuug....................................................   17   0    ya2
.........aaaaguaauugugguuuuugcc..................................................   1    0    ya2
```

```
         Mature                                  Star
gguuggugcaaaaguaauugugguuuuugccauuacuuuuaaaaaaaggcaaaaaccacaauugcuuuuacaucaaccuaa
.........aaaaguaauugugguuuuugcca.................................................   1    0    ya2
..........aaaguaauugugguuuuu.....................................................   43   0    ya2
..........aaaguaauugugguuuuug....................................................   4    0    ya2

........caaaaguaauugugguuuuug....................................................   1    0    s02
.........aaaaguaauugugguuuuugcc..................................................   1    0    s02

........caaaaguaauugugguuuuug....................................................   3    0    k06
.........aaaaguaauugugguuuuug....................................................   2    0    k06

.......gcaaaaguaauugugguuuuug....................................................   1    0    j29
........caaaaguaauugugguuuuug....................................................   3    0    j29
.........aaaaguaauugugguuuuug....................................................   10   0    j29
.........aaaaguaauugugguuuuugc...................................................   8    0    j29
.........aaaaguaauugugguuuuugcc..................................................   1    0    j29

gguuggugcaaaaguaauugu............................................................   1    0    sh2
..uuggugcaaaaguaauugugguu........................................................   1    0    sh2
.....gugcaaaaguaauugugguu........................................................   1    0    sh2
........caaaaguaauugugguuuuu.....................................................   1    0    sh2
........caaaaguaauugugguuuuugc...................................................   1    0    sh2
.........aaaaguaauugugguuuuug....................................................   1    0    sh2
.........aaaaguaauugugguuuuugc...................................................   3    0    sh2
.........aaaaguaauugugguuuuugcc..................................................   3    0    sh2
.........aaaaguaauugugguuuuugcca.................................................   2    0    sh2

....ggugcaaaaguaauugugguuu.......................................................   1    0    j01
......ugcaaaaguaauugugguuuu......................................................   3    0    j01
........caaaaguaauugugguuuuug....................................................   1    0    j01
.........aaaaguaauugugguuuuug....................................................   5    0    j01

...uggugcaaaaguaauuguggu.........................................................   1    0    MCF
........caaaaguaauugugguuuuugc...................................................   1    0    MCF
.........aaaaguaauugugguuuuug....................................................   4    0    MCF
.........aaaaguaauugugguuuuugc...................................................   11   0    MCF
.........aaaaguaauugugguuuuugcc..................................................   42   0    MCF
.........aaaaguaauugugguuuuugcca.................................................   1    0    MCF
..........aaaguaauugugguuuuugcc..................................................   1    0    MCF

.........aaaaguaauugugguuuuu.....................................................   2    0    NL1
.........aaaaguaauugugguuuuug....................................................   2    0    NL1
.........aaaaguaauugugguuuuugc...................................................   1    0    NL1

........caaaaguaauugugguuuuugc...................................................   1    0    MBM
.........aaaaguaauugugguuuuug....................................................   2    0    MBM
.........aaaaguaauugugguuuuugc...................................................   14   0    MBM
.........aaaaguaauugugguuuuugcc..................................................   33   0    MBM
.........aaaaguaauugugguuuuugcca.................................................   6    0    MBM
..........aaaguaauugugguuuuugc...................................................   1    0    MBM
..........aaaguaauugugguuuuugcc..................................................   1    0    MBM
.............guaauugugguuuuugcc..................................................   1    0    MBM

....ggugcaaaaguaauugugguu........................................................   1    0    k07
......ugcaaaaguaauugugguuuuu.....................................................   1    0    k07
........caaaaguaauugugguuuuug....................................................   9    0    k07
.........aaaaguaauugugguuuuug....................................................   3    0    k07
.........aaaaguaauugugguuuuugc...................................................   1    0    k07

......ugcaaaaguaauugugguuuuu.....................................................   1    0    sh1
........caaaaguaauugugguuuuug....................................................   6    0    sh1
.........aaaaguaauugugguuuuug....................................................   4    0    sh1

...uggugcaaaaguaauugugguu........................................................   1    0    li1
........caaaaguaauugugguuuuug....................................................   3    0    li1
........caaaaguaauugugguuuuugc...................................................   1    0    li1
.........aaaaguaauugugguuuuugc...................................................   23   0    li1
.........aaaaguaauugugguuuuugcc..................................................   2    0    li1
..........aaaguaauugugguuuuugc...................................................   1    0    li1

........caaaaguaauugugguuuuu.....................................................   1    0    k13
```

```
         Mature                                   Star
gguuggugcaaaaguaauugugguuuuugccauuacuuuuaaaaaaaggcaaaaaccacaauugcuuuuacaucaaccuaa
........caaaaguaauugugguuuuug....................................................    4     0     k13
.........aaaaguaauugugguuuuug....................................................    1     0     k13

........caaaaguaauugugguuuu......................................................    1     0     j20
........caaaaguaauugugguuuuu.....................................................    2     0     j20
........caaaaguaauugugguuuuug....................................................    7     0     j20
.........aaaaguaauugugguuuuu.....................................................    3     0     j20
.........aaaaguaauugugguuuuug....................................................    27    0     j20
.........aaaaguaauugugguuuuugcc..................................................    1     0     j20
...........aaguaauugugguuuuugc...................................................    1     0     j20

.......gcaaaaguaauugugguuuuug....................................................    11    0     A54
.........aaaaguaauugugguuuuug....................................................    3     0     A54
.........aaaaguaauugugguuuuugc...................................................    3     0     A54
.........aaaaguaauugugguuuuugcc..................................................    50    0     A54
.........aaaaguaauugugguuuuugcca.................................................    5     0     A54
..........aaaguaauugugguuuuugcc..................................................    5     0     A54

...uggugcaaaaguaauugugguu........................................................    1     0     j30
.........aaaaguaauugugguuuuug....................................................    2     0     j30
.........aaaaguaauugugguuuuugcc..................................................    1     0     j30

........caaaaguaauugugguuuuu.....................................................    8     0     k12
........caaaaguaauugugguuuuug....................................................    12    0     k12
.........aaaaguaauugugguuuuu.....................................................    6     0     k12
.........aaaaguaauugugguuuuug....................................................    18    0     k12
.........aaaaguaauugugguuuuugc...................................................    2     0     k12
.........aaaaguaauugugguuuuugcc..................................................    1     0     k12
```

Provisional ID	:	hsa_can_1112
Score total	:	2.2
Score for star read(s)	:	-1.3
Score for read counts	:	0
Score for mfe	:	0.5
Score for randfold	:	
Score for cons. seed	:	3
Total read count	:	1300
Mature read count	:	1092
Loop read count	:	0
Star read count	:	208

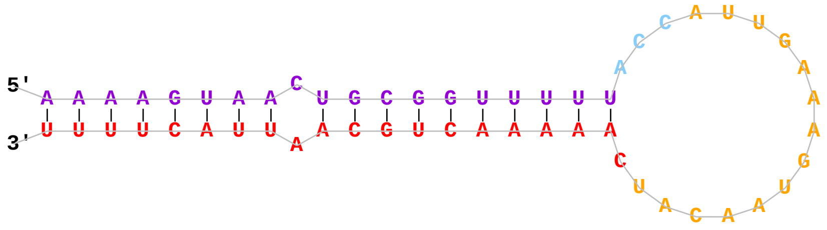

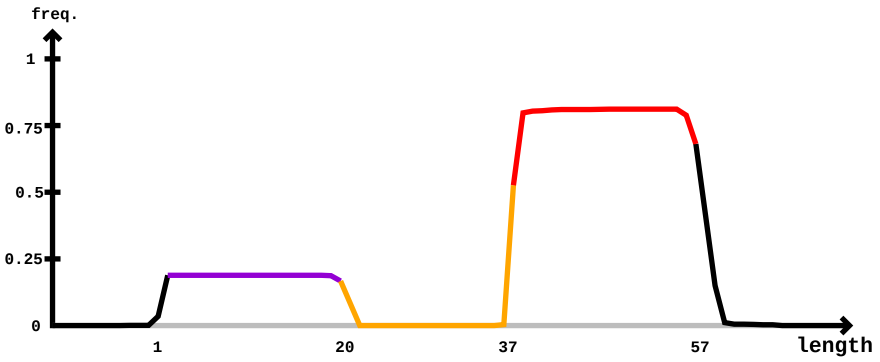

Star Mature

```
5'- aggucagugcaaaaguaacugcgguuuuuaccauugaaaguaacaucaaaaacugcaauuacuuuugcaccaacccaauac   -3'    obs
    aggucagugcaaaaguaacugcgguuuuuaccauugaaaguaacaucaaaaacugcaauuacuuuugcaccaacccaauac          exp
    aggucagugcaaaaguaacugcgguuuuuaccauugaaaguaacaucaaaaacugcaauuacuuuugcaccaacccaauac          known
    .(((..((((((((((((.((((((((((.................)))))))))).)))))))))))..))).......    reads      mm         sample
    .........aaaaguaacugcgguuuuu..................................................    2          0          ya1
    .........aaaaguaacugcgguuuuuu.................................................    21         0          ya1
    ..............................................caaaaacugcaauuacuu................    1          0          ya1
    ..............................................caaaaacugcaauuacuuu...............    12         0          ya1
    ..............................................caaaaacugcaauuacuuuu..............    15         0          ya1
    ..............................................caaaaacugcaauuacuuuug.............    2          0          ya1
    ...............................................aaaaacugcaauuacuuu...............    3          0          ya1
    ...............................................aaaaacugcaauuacuuuu..............    7          0          ya1
    ...............................................aaaaacugcaauuacuuuug.............    2          0          ya1
    ................................................aaaacugcaauuacuuuu..............    4          0          ya1
    ................................................aaaacugcaauuacuuuugc............    2          0          ya1

    ..............................................caaaaacugcaauuacuuuug.............    5          0          va1
    ..............................................caaaaacugcaauuacuuuugc............    2          0          va1
    ...............................................aaaaacugcaauuacuuuug.............    1          0          va1

    ........caaaaguaacugcgguuuuuu.................................................    1          0          ku5
    .........aaaaguaacugcgguuuuuu.................................................    4          0          ku5
    ..............................................caaaaacugcaauuacuuuug.............    1          0          ku5

    ..............................................caaaaacugcaauuacuuuugc............    1          0          s06
    ..................................................aaacugcaauuacuuuugcacca........    1          0          s06

    ..............................................caaaaacugcaauuacuuuug.............    2          0          HeL
    ..............................................caaaaacugcaauuacuuuugc............    5          0          HeL
    ...............................................aaaaacugcaauuacuuuugc............    8          0          HeL
    ...............................................aaaaacugcaauuacuuuugca...........    5          0          HeL

    .........aaaaguaacugcgguuuuuu.................................................    5          0          ku4
    ..............................................caaaaacugcaauuacuuuug.............    1          0          ku4

    ..............................................caaaaacugcaauuacuuuugc............    1          0          k08
```

Star **Mature**

aggucagugcaaaaguaacugcgguuuuuaccauugaaaguaacaucaaaaacugcaauuacuuuugcaccaacccaauac			
....aaaaacugcaauuacuuuug..............	1	0	k08
..........aaaaguaacugcgguuuuu..	1	0	ta3
....caaaaacugcaauuacuuuu...............	2	0	ta3
..........aaaaguaacugcgguuuuu..	1	0	j10
....aaaaacugcaauuacuuuug..............	2	0	j10
..........aaaaguaacugcgguuuuua...	9	0	j04
....caaaaacugcaauuacuuuug..............	4	0	j04
..........aaaaguaacugcgguuuuu..	1	0	k14
....aaaaacugcaauuacuuuug..............	2	0	k14
.........caaaaguaacugcgguuuuu..	2	0	va2
..........aaaaguaacugcgguuuu...	1	0	va2
..........aaaaguaacugcgguuuuu..	2	0	va2
....aaacugcaauuacuuuug..............	1	0	j31
.........caaaaguaacugcgguuuuu..	2	0	j27
..........aaaaguaacugcgguuuuu..	18	0	j27
....caaaaacugcaauuacuu.................	1	0	j27
....caaaaacugcaauuacuuuug..............	3	0	j27
....aaaaacugcaauuacuu.................	4	0	j27
....aaaaacugcaauuacuuu................	3	0	j27
....aaaaacugcaauuacuuuug..............	7	0	j27
....caaaaacugcaauuacuuuugc.............	1	0	s03
....aacugcaauuacuuuugcacca.........	1	0	s03
....caaaaacugcaauuacuuuugc.............	3	0	H52
.........caaaaguaacugcgguuuu...	1	0	j22
..........aaaaguaacugcgguuuu...	1	0	j22
....caaaaacugcaauuacuuu................	1	0	j22
....caaaaacugcaauuacuuuug..............	12	0	j22
....caaaaacugcaauuacuuuugc.............	3	0	j22
....aaaaacugcaauuacuuu................	2	0	j22
....aaaaacugcaauuacuuuug..............	2	0	j22
....aaaaacugcaauuacuuu................	1	0	k09
....ucaaaaacugcaauuacuu.................	1	0	B14
....caaaaacugcaauuacuuuugc.............	5	0	B14
....aaaaacugcaauuacuuuugc.............	1	0	B14
....aacugcaauuacuuuugc.............	1	0	B14
....caaaaacugcaauuacuuuug..............	3	0	j09
....aaaaacugcaauuacuuu................	2	0	j09
....aaaaacugcaauuacuuuug..............	1	0	j09
.........caaaaguaacugcgguuuu...	1	0	ku3
.........caaaaguaacugcgguuuuu..	5	0	ku3
..........aaaaguaacugcgguuuuu..	1	0	ku3
....caaaaacugcaauuacuuuug..............	1	0	ku3
.........caaaaguaacugcgguuuuu..	2	0	j18
..........aaaaguaacugcgguuuu...	1	0	j16
..........aaaaguaacugcgguuuuu..	1	0	j16
.........caaaaguaacugcgguuuuu..	2	0	ku6
..........aaaaguaacugcgguuuuu..	5	0	ku6
....caaaaacugcaauuacuuu................	1	0	ku6
.........caaaaguaacugcgguu...	1	0	ya3
.........caaaaguaacugcgguuuuu..	3	0	ya3
..........aaaaguaacugcgguuuu...	3	0	ya3
..........aaaaguaacugcgguuuuu..	23	0	ya3

```
          Star                                  Mature
aggucagugcaaaaguaacugcgguuuuuaccauugaaaguaacaucaaaaacugcaauuacuuuugcaccaacccaauac
..............................................caaaaacugcaauuacuu.................    2    0    ya3
..............................................caaaaacugcaauuacuuu................    6    0    ya3
..............................................caaaaacugcaauuacuuuu...............    11   0    ya3
..............................................caaaaacugcaauuacuuuug..............    4    0    ya3
...............................................aaaaacugcaauuacuu.................    12   0    ya3
...............................................aaaaacugcaauuacuuu................    18   0    ya3
...............................................aaaaacugcaauuacuuuug..............    3    0    ya3
...............................................aaaaacugcaauuacuuuugc.............    1    0    ya3
................................................aaaacugcaauuacuuuug..............    1    0    ya3

..............................................caaaaacugcaauuacuuuug..............    1    0    HEK
..............................................caaaaacugcaauuacuuuugc.............    13   0    HEK
...............................................aaaaacugcaauuacuuuugc.............    3    0    HEK

..............................................caaaaacugcaauuacuuuu...............    2    0    j17
..............................................caaaaacugcaauuacuuuug..............    1    0    j17

.........caaaaguaacugcgguuuu.....................................................    1    0    j15
..........aaaaguaacugcgguuuuu....................................................    5    0    j15
..............................................caaaaacugcaauuacuuuu...............    1    0    j15
..............................................caaaaacugcaauuacuuuug..............    1    0    j15
...............................................aaaaacugcaauuacuuuug..............    1    0    j15

..............................................caaaaacugcaauuacuuuug..............    1    0    la2
..............................................caaaaacugcaauuacuuuugc.............    1    0    la2

...............................................aaaaacugcaauuacuuuu...............    1    0    j12
...............................................aaaaacugcaauuacuuuug..............    1    0    j12

..............................................caaaaacugcaauuacuuuug..............    1    0    li2
..............................................caaaaacugcaauuacuuuugc.............    14   0    li2
...............................................aaaaacugcaauuacuuuugc.............    1    0    li2

..............................................caaaaacugcaauuacuuuugc.............    1    0    va4

.............................................ucaaaaacugcaauuacuu.................    1    0    va3
..............................................caaaaacugcaauuacuuuug..............    1    0    va3
..............................................caaaaacugcaauuacuuuugc.............    1    0    va3
................................................aaaacugcaauuacuuuugc.............    1    0    va3

..........aaaaguaacugcgguuuuu....................................................    14   0    ya4
..............................................caaaaacugcaauuacuu.................    7    0    ya4
..............................................caaaaacugcaauuacuuu................    8    0    ya4
..............................................caaaaacugcaauuacuuuu...............    23   0    ya4
...............................................aaaaacugcaauuacuu.................    6    0    ya4
...............................................aaaaacugcaauuacuuuug..............    3    0    ya4

..........aaaaguaacugcgguuuuu....................................................    1    0    SW4
.............................................ucaaaaacugcaauuacuuuug..............    3    0    SW4
..............................................caaaaacugcaauuacuuuugc.............    5    0    SW4
..................................................aacugcaauuacuuuugc.............    1    0    SW4

.........caaaaguaacugcgguuuu.....................................................    1    0    ku1
..........aaaaguaacugcgguuuuu....................................................    1    0    ku1
..............................................caaaaacugcaauuacuuuug..............    2    0    ku1
..............................................caaaaacugcaauuacuuuugc.............    1    0    ku1

..............................................caaaaacugcaauuacuuuu...............    1    0    j23
..............................................caaaaacugcaauuacuuuug..............    7    0    j23
..............................................caaaaacugcaauuacuuuugc.............    1    0    j23
...............................................aaaaacugcaauuacuuuu...............    1    0    j23
...............................................aaaaacugcaauuacuuuug..............    1    0    j23

..........aaaaguaacugcgguuuuu....................................................    1    0    s05
...............................................aaaaacugcaauuacuuuu...............    1    0    s05

..............................................caaaaacugcaauuacuuuug..............    1    0    ta2

.........caaaaguaacugcgguuuu.....................................................    3    0    j24

.........caaaaguaacugcgguuuuu....................................................    1    0    NL2
```

Star **Mature**

aggucagugcaaaaguaacugcgguuuuuaccauugaaaguaacaucaaaaacugcaauuacuuuugcaccaacccaauac			
..........aaaaguaacugcgguuuuu..	14	0	NL2
..........aaaaguaacugcgguuuuua...	1	0	NL2
....caaaaacugcaauuacuu.................	8	0	NL2
....caaaaacugcaauuacuuu................	15	0	NL2
....caaaaacugcaauuacuuuu...............	77	0	NL2
....caaaaacugcaauuacuuuug..............	36	0	NL2
....caaaaacugcaauuacuuuugc.............	7	0	NL2
....aaaaacugcaauuacuuu................	14	0	NL2
....aaaaacugcaauuacuuuu...............	40	0	NL2
....aaaaacugcaauuacuuuug..............	32	0	NL2
..........aaaaguaacugcgguuuuu..	2	0	j13
.........caaaaguaacugcgguuuuu..	1	0	j19
..........aaaaguaacugcgguuuu...	3	0	j19
..........aaaaguaacugcgguuuuu..	5	0	j19
....caaaaacugcaauuacuuuu...............	2	0	j19
....caaaaacugcaauuacuuuug..............	4	0	j19
....aaaaacugcaauuacuuuug..............	1	0	j19
.........caaaaguaacugcgguuuuu..	2	0	j25
..........aaaaguaacugcgguuuuu..	4	0	j25
....caaaaacugcaauuacuuu................	1	0	j25
....caaaaacugcaauuacuuuu...............	1	0	j25
..........aaaaguaacugcgguuuu...	1	0	k01
....aaaaacugcaauuacuuuu...............	2	0	s01
....caaaaacugcaauuacuuu................	1	0	ku2
....caaaaacugcaauuacuuuug..............	2	0	ku2
....caaaaacugcaauuacuuuug..............	3	0	U20
....caaaaacugcaauuacuuuugc.............	12	0	U20
....aaaaacugcaauuacuuuug..............	1	0	U20
....aaaaacugcaauuacuuuugc.............	6	0	U20
....aaaaacugcaauuacuuuugca............	2	0	U20
....acugcaauuacuuuugcaccaac.......	2	0	U20
....caaaaacugcaauuacuuuug..............	1	0	j21
....caaaaacugcaauuacuuuugc.............	3	0	j21
....caaaaacugcaauuacuuuug..............	1	0	j14
.........caaaaguaacugcgguuuuu..	5	0	j28
..........aaaaguaacugcgguuuu...	2	0	j28
..........aaaaguaacugcgguuuuu..	5	0	j28
....caaaaacugcaauuacuuuug..............	2	0	DLD
....caaaaacugcaauuacuuuugc.............	6	0	DLD
....caaaaacugcaauuacuuu................	1	0	k03
....aaaaacugcaauuacuuuug..............	1	0	k03
.........caaaaguaacugcgguuuuu..	6	0	NL3
..........aaaaguaacugcgguuuu...	1	0	NL3
..........aaaaguaacugcgguuuuu..	18	0	NL3
....caaaaacugcaauuacuu.................	7	0	NL3
....caaaaacugcaauuacuuu................	36	0	NL3
....caaaaacugcaauuacuuuu...............	113	0	NL3
....caaaaacugcaauuacuuuug..............	34	0	NL3
....caaaaacugcaauuacuuuugc.............	7	0	NL3
....aaaaacugcaauuacuuu................	7	0	NL3
....aaaaacugcaauuacuuuu...............	60	0	NL3
....aaaaacugcaauuacuuuug..............	36	0	NL3
....aaaaacugcaauuacuuuugc.............	2	0	NL3
.........caaaaguaacugcgguuuuu..	5	0	ya2
..........aaaaguaacugcgguuuu...	4	0	ya2

```
          Star                               Mature
aggucagugcaaaaguaacugcgguuuuuaccauugaaaguaacaucaaaaacugcaauuacuuuugcaccaacccaauac
..........aaaaguaacugcgguuuuu....................................................    20    0    ya2
..............................................caaaaacugcaauuacuu.................    6     0    ya2
..............................................caaaaacugcaauuacuuu................    3     0    ya2
..............................................caaaaacugcaauuacuuuu...............    4     0    ya2
...............................................aaaaacugcaauuacuuu................    7     0    ya2
...............................................aaaaacugcaauuacuuuu...............    4     0    ya2
...............................................aaaaacugcaauuacuuuug..............    1     0    ya2
.......................................................caauuacuuuugcaccaac.......    1     0    ya2
........................................................aauuacuuuugcaccaac.......    1     0    ya2

..............................................caaaaacugcaauuacuuuugc.............    1     0    j29
...............................................aaaaacugcaauuacuuuug..............    4     0    j29

..............................................caaaaacugcaauuacuuuugc.............    11    0    sh2
...............................................aaaaacugcaauuacuuuugc.............    2     0    sh2

......gugcaaaaguaacugcgguuu......................................................    1     0    MCF
..............................................caaaaacugcaauuacuuu................    1     0    MCF
..............................................caaaaacugcaauuacuuuu...............    4     0    MCF
..............................................caaaaacugcaauuacuuuug..............    2     0    MCF
..............................................caaaaacugcaauuacuuuugc.............    15    0    MCF
..............................................caaaaacugcaauuacuuuugca............    1     0    MCF
...............................................aaaaacugcaauuacuuuug..............    2     0    MCF
..................................................aacugcaauuacuuuugc.............    1     0    MCF

.........caaaaguaacugcgguuuu.....................................................    1     0    j01
..........aaaaguaacugcgguuuuu....................................................    5     0    j01
..............................................caaaaacugcaauuacuuuug..............    1     0    j01

..............................................caaaaacugcaauuacuuu................    5     0    NL1
..............................................caaaaacugcaauuacuuuu...............    7     0    NL1
..............................................caaaaacugcaauuacuuuug..............    19    0    NL1
..............................................caaaaacugcaauuacuuuugc.............    3     0    NL1
...............................................aaaaacugcaauuacuuuu...............    4     0    NL1
...............................................aaaaacugcaauuacuuuug..............    21    0    NL1
...............................................aaaaacugcaauuacuuuugc.............    3     0    NL1

..............................................caaaaacugcaauuacuuuugc.............    6     0    MBM
...............................................aaaaacugcaauuacuuuugc.............    3     0    MBM

..............................................caaaaacugcaauuacuuuug..............    1     0    sh1

..............................................caaaaacugcaauuacuuuug..............    1     0    li1
..............................................caaaaacugcaauuacuuuugc.............    13    0    li1
...............................................aaaaacugcaauuacuuuugc.............    1     0    li1

..........aaaaguaacugcgguuuuu....................................................    1     0    k13
...............................................aaaaacugcaauuacuuuug..............    1     0    k13

..............................................caaaaacugcaauuacuuuu...............    1     0    j20
..............................................caaaaacugcaauuacuuuug..............    4     0    j20
...............................................aaaaacugcaauuacuuuu...............    1     0    j20
...............................................aaaaacugcaauuacuuuug..............    2     0    j20

..............................................caaaaacugcaauuacuuuu...............    2     0    A54
..............................................caaaaacugcaauuacuuuugc.............    8     0    A54

..............................................caaaaacugcaauuacuu.................    1     0    k12
..............................................caaaaacugcaauuacuuuugc.............    1     0    k12
.................................................aaacugcaauuacuuuugcacc..........    1     0    k12
```

Provisional ID	:	hsa_can_1135
Score total	:	0
Score for star read(s)	:	-1.3
Score for read counts	:	0
Score for mfe	:	1.8
Score for randfold	:	
Score for cons. seed	:	-0.6
Total read count	:	875
Mature read count	:	363
Loop read count	:	0
Star read count	:	512

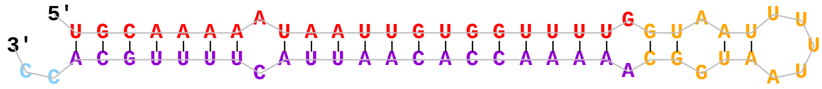

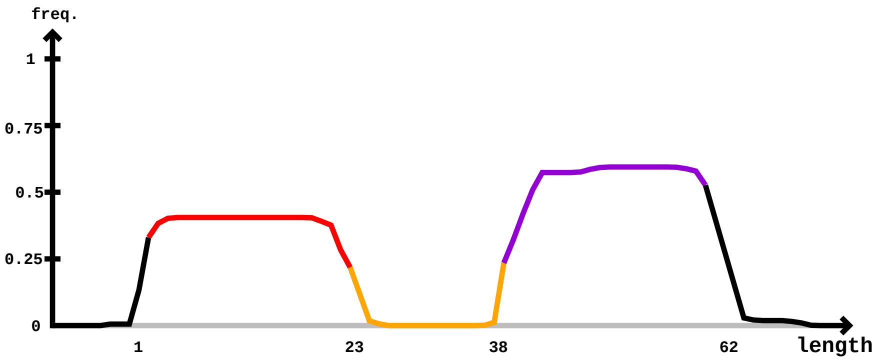

Mature **Star**

```
5'- uagguuggugcaaaaauaauugugguuuugguaauuuuuaauggcaaaaaccacaauuacuuuugcaccaaccuaauaacu   -3'    obs
    uagguuggugcaaaaauaauugugguuuugguaauuuuuaauggcaaaaaccacaauuacuuuugcaccaaccuaauaacu          exp
    uagguuggugcaaaaauaauugugguuuugguaauuuuuaauggcaaaaaccacaauuacuuuugcaccaaccuaauaacu          known
    (((((((((((((((.(((((((((((((.((.((.....)).)).))))))))))))).)))))))))))))))......    reads    mm    sample
    ........ugcaaaaauaauugugguuuu....................................................    1        0     ya1
    .........gcaaaaauaauugugguu......................................................    1        0     ya1
    .........gcaaaaauaauugugguuuu....................................................    1        0     ya1
    ..........caaaaauaauugugguuuuggua................................................    4        0     ya1
    .............................................aaaaaccacaauuacuuuug................    1        0     ya1
    .............................................aaaaaccacaauuacuuuugca..............    4        0     ya1
    ..............................................aaaaccacaauuacuuuugca..............    3        0     ya1
    ..............................................aaaaccacaauuacuuuugcac.............    1        0     ya1
    ...............................................aaaccacaauuacuuuugca..............    1        0     ya1
    ................................................aaccacaauuacuuuugcacc............    1        0     ya1

    .............................................aaaaaccacaauuacuuuugca..............    1        0     s04

    .............................................aaaaaccacaauuacuuuugca..............    3        0     j26
    ..................................................accacaauuacuuuugcacca..........    1        0     j26

    .......gugcaaaaauaauugugguu......................................................    1        0     va1
    .......gugcaaaaauaauugugguuuu....................................................    1        0     va1
    ........ugcaaaaauaauugugguuuug...................................................    3        0     va1
    .............................................aaaaaccacaauuacuuuugc...............    1        0     va1
    .............................................aaaaaccacaauuacuuuugca..............    6        0     va1
    .............................................aaaaaccacaauuacuuuugcac.............    1        0     va1
    ..............................................aaaaccacaauuacuuuugcac.............    5        0     va1
    ...............................................aaaccacaauuacuuuugcacc............    1        0     va1

    ........ugcaaaaauaauugugguuuug...................................................    1        0     ku5
    .............................................aaaaaccacaauuacuuuugca..............    1        0     ku5
    ...............................................aaaccacaauuacuuuugcacc............    1        0     ku5

    ...............................................aaaccacaauuacuuuugcacc............    1        0     k05

    .............................................aaaaaccacaauuacuuuugca..............    1        0     s06
```

Mature   Star

```
uagguuggugcaaaaauaauugugguuuugguaauuuuuaauggcaaaaaccacaauuacuuuugcaccaaccuaauaacu
................................................aaccacaauuacuuuugcacca...........    1     0     s06
.................................................accacaauuacuuuugcacca...........    1     0     s06

.......gugcaaaaauaauugugguuu.....................................................    2     0     HeL
.......gugcaaaaauaauugugguuuu....................................................    1     0     HeL
........ugcaaaaauaauugugguuuu....................................................    3     0     HeL
........ugcaaaaauaauugugguuuug...................................................    15    0     HeL
.............................................aaaaaccacaauuacuuuugc...............    1     0     HeL
.............................................aaaaaccacaauuacuuuugca..............    3     0     HeL
..............................................aaaaccacaauuacuuuugcac.............    2     0     HeL
...............................................aaaccacaauuacuuuugcac.............    1     0     HeL
...............................................aaaccacaauuacuuuugcacc............    3     0     HeL
................................................aaccacaauuacuuuugcacca...........    3     0     HeL
................................................aaccacaauuacuuuugcaccaa..........    3     0     HeL
.................................................accacaauuacuuuugcacca...........    5     0     HeL

........ugcaaaaauaauugugguuuug...................................................    1     0     ku4

.......gugcaaaaauaauugugguuu.....................................................    1     0     s07
...............................................aaaccacaauuacuuuugcacc............    1     0     s07

.........gcaaaaauaauugugguuuugg..................................................    3     0     s10
.............................................aaaaaccacaauuacuuuugca..............    4     0     s10
...............................................aaaccacaauuacuuuugcac.............    1     0     s10

.......gugcaaaaauaauugugguu......................................................    1     0     k08

.......gugcaaaaauaauugugguuu.....................................................    1     0     ta3
.........gcaaaaauaauugugguuuugg..................................................    1     0     ta3
.............................................aaaaaccacaauuacuuuugca..............    4     0     ta3
..............................................aaaaccacaauuacuuuugc...............    1     0     ta3
..............................................aaaaccacaauuacuuuugcac.............    1     0     ta3
...............................................aaaccacaauuacuuuugcac.............    1     0     ta3
...............................................aaaccacaauuacuuuugcacc............    1     0     ta3
................................................aaccacaauuacuuuugcacca...........    2     0     ta3

.......gugcaaaaauaauugugguuuu....................................................    1     0     j10
........ugcaaaaauaauugugguuuug...................................................    1     0     j10
.............................................aaaaaccacaauuacuuuugca..............    5     0     j10
...............................................aaaccacaauuacuuuugcac.............    1     0     j10
................................................aaccacaauuacuuuugcacca...........    1     0     j10

.............................................aaaaaccacaauuacuuuugcac.............    1     0     va2
.....................................................caauuacuuuugcaccaaccuaa.....    1     0     va2

.........gcaaaaauaauugugguuuugg..................................................    4     0     j02

.......gugcaaaaauaauugugguuu.....................................................    1     0     k14
................................................aaccacaauuacuuuugcac.............    1     0     k14

........ugcaaaaauaauugugguuuug...................................................    1     0     j31
.............................................aaaaaccacaauuacuuuugca..............    1     0     j31

.......gugcaaaaauaauugugguu......................................................    1     0     j27
.........gcaaaaauaauugugguuuugg..................................................    1     0     j27
..........caaaaauaauugugguuuuggu.................................................    1     0     j27
...............................................aaaccacaauuacuuuugca..............    1     0     j27

.............................................aaaaaccacaauuacuuuugca..............    2     0     s03
..............................................aaaaccacaauuacuuuugca..............    1     0     s03
..............................................aaaaccacaauuacuuuugcac.............    2     0     s03

.......gugcaaaaauaauugugguuuu....................................................    1     0     H52
........ugcaaaaauaauugugguuuug...................................................    1     0     H52
.........gcaaaaauaauugugguuuugg..................................................    1     0     H52
.............................................aaaaaccacaauuacuuuug................    2     0     H52
.............................................aaaaaccacaauuacuuuugca..............    1     0     H52
..............................................aaaaccacaauuacuuuugc...............    1     0     H52
..............................................aaaaccacaauuacuuuugca..............    2     0     H52
...............................................aaaccacaauuacuuuugcacc............    2     0     H52
```

Mature **Star**

```
uagguuggugcaaaaauaauugugguuuugguaauuuuuaauggcaaaaaccacaauuacuuuugcaccaaccuaauaacu
................................................aaccacaauuacuuuugcacc............    1    0    H52
................................................aaccacaauuacuuuugcacca...........    1    0    H52
.................................................accacaauuacuuuugcacca...........    1    0    H52

.......gugcaaaaauaauugugguu......................................................    1    0    j22
.......gugcaaaaauaauugugguuu.....................................................    1    0    j22
.........gcaaaaauaauugugguuuugg..................................................    1    0    j22
.............................................aaaaaccacaauuacuuuugca..............    6    0    j22
..............................................aaaaccacaauuacuuuugca..............    1    0    j22
................................................aaccacaauuacuuuugcacc............    1    0    j22

.......gugcaaaaauaauugugguu......................................................    1    0    k09
..............................................aaaaccacaauuacuuuugcac.............    1    0    k09
...............................................aaaccacaauuacuuuugcaccaa..........    1    0    k09

.......gugcaaaaauaauugugguuu.....................................................    3    0    B14
........ugcaaaaauaauugugguuu.....................................................    2    0    B14
........ugcaaaaauaauugugguuuug...................................................    4    0    B14
.............................................aaaaaccacaauuacuuuugc...............    3    0    B14
.............................................aaaaaccacaauuacuuuugca..............    6    0    B14
..............................................aaaaccacaauuacuuuugcac.............    1    0    B14
...............................................aaaccacaauuacuuuugca..............    2    0    B14
...............................................aaaccacaauuacuuuugcacc............    2    0    B14
.................................................accacaauuacuuuugcacca...........    2    0    B14

.......gugcaaaaauaauugugguu......................................................    1    0    ku3
.............................................aaaaaccacaauuacuuuugcac.............    1    0    ku3
...............................................aaaccacaauuacuuuugcacca...........    1    0    ku3

.......gugcaaaaauaauugugguuu.....................................................    2    0    j16
........ugcaaaaauaauugugguuuug...................................................    4    0    j16
.............................................aaaaaccacaauuacuuuugca..............    4    0    j16

.......gugcaaaaauaauugugguuu.....................................................    1    0    j09
..........caaaaauaauugugguuuuggu.................................................    3    0    j09
.............................................aaaaaccacaauuacuuuugca..............    1    0    j09
.................................................accacaauuacuuuugcacca...........    1    0    j09

........ugcaaaaauaauugugguuuug...................................................    1    0    ku6
.............................................aaaaaccacaauuacuuuugca..............    2    0    ku6

.......gugcaaaaauaauugugguuu.....................................................    4    0    j11
.............................................aaaaaccacaauuacuuuugca..............    2    0    j11

.......gugcaaaaauaauugugguuu.....................................................    2    0    ya3
........ugcaaaaauaauugugguuuu....................................................    1    0    ya3
.........gcaaaaauaauugugguuuu....................................................    3    0    ya3
.........gcaaaaauaauugugguuuug...................................................    1    0    ya3
.............................................aaaaaccacaauuacuuuugca..............    3    0    ya3
..............................................aaaaccacaauuacuuuugca..............    2    0    ya3
..............................................aaaaccacaauuacuuuugcac.............    5    0    ya3
...............................................aaaccacaauuacuuuugca..............    1    0    ya3
...............................................aaaccacaauuacuuuugcacc............    1    0    ya3
................................................aaccacaauuacuuuugcacc............    2    0    ya3

.............................................aaaaaccacaauuacuuuugca..............    2    0    la1
................................................aaccacaauuacuuuugcacc............    1    0    la1

.......gugcaaaaauaauuguggu.......................................................    1    0    HEK
.......gugcaaaaauaauugugguuu.....................................................    13   0    HEK
.......gugcaaaaauaauugugguuuu....................................................    2    0    HEK
........ugcaaaaauaauuguggu.......................................................    1    0    HEK
........ugcaaaaauaauugugguuu.....................................................    1    0    HEK
........ugcaaaaauaauugugguuuu....................................................    1    0    HEK
........ugcaaaaauaauugugguuuug...................................................    20   0    HEK
.........gcaaaaauaauugugguuuugg..................................................    1    0    HEK
..........caaaaauaauugugguuuuggu.................................................    1    0    HEK
............................................caaaaaccacaauuacuuuugc...............    3    0    HEK
.............................................aaaaaccacaauuacuuuugc...............    1    0    HEK
.............................................aaaaaccacaauuacuuuugca..............    8    0    HEK
```

```
        Mature                    Star
uagguuggugcaaaaauaauugugguuuugguaauuuuuaauggcaaaaaccacaauuacuuuugcaccaaccuaauaacu
..............................................aaaaccacaauuacuuuugca..............   2   0   HEK
..............................................aaaaccacaauuacuuuugcac.............   3   0   HEK
...............................................aaaccacaauuacuuuugca..............   1   0   HEK
...............................................aaaccacaauuacuuuugcac.............   3   0   HEK
...............................................aaaccacaauuacuuuugcacc............   3   0   HEK
................................................aaccacaauuacuuuugcacc............   2   0   HEK
................................................aaccacaauuacuuuugcacca...........   4   0   HEK
.................................................accacaauuacuuuugcacca...........   6   0   HEK

.......gugcaaaaauaauugugguu......................................................   2   0   j15
..............................................aaaaccacaauuacuuuugca..............   1   0   j15

.......gugcaaaaauaauugugguuu.....................................................   1   0   j17
..............................................aaaaccacaauuacuuuugca..............   1   0   j17
................................................aaccacaauuacuuuugcacca...........   2   0   j17
.................................................accacaauuacuuuugcacca...........   1   0   j17

..............................................aaaaccacaauuacuuuugcac.............   1   0   la2
...............................................aaaccacaauuacuuuugca..............   1   0   la2
...............................................aaaccacaauuacuuuugcacc............   1   0   la2
................................................aaccacaauuacuuuugcacca...........   1   0   la2

........ugcaaaaauaauugugguuuug...................................................   2   0   j08

.................................................accacaauuacuuuugcacca...........   1   0   j12

........ugcaaaaauaauugugguuuug...................................................   2   0   li2
.........gcaaaaauaauugugguuuugg..................................................   2   0   li2

.......gugcaaaaauaauugugguuu.....................................................   1   0   va4
........ugcaaaaauaauugugguuuug...................................................   2   0   va4
.............................................aaaaaccacaauuacuuuugca..............   3   0   va4
.............................................aaaaaccacaauuacuuuugcac.............   1   0   va4
..............................................aaaaccacaauuacuuuugcac.............   1   0   va4
................................................aaccacaauuacuuuugcacc............   2   0   va4
.................................................accacaauuacuuuugcacca...........   4   0   va4

........ugcaaaaauaauugugguuuug...................................................   3   0   va3
.........gcaaaaauaauugugguuuugg..................................................   2   0   va3
..........caaaaauaauugugguuuuggua................................................   1   0   va3
.............................................aaaaaccacaauuacuuuugca..............  12   0   va3
..............................................aaaaccacaauuacuuuugcac.............   2   0   va3
...............................................aaaccacaauuacuuuugcac.............   1   0   va3
...............................................aaaccacaauuacuuuugcacc............   4   0   va3
................................................aaccacaauuacuuuugcacc............   2   0   va3
................................................aaccacaauuacuuuugcacca...........   2   0   va3
.................................................accacaauuacuuuugcacca...........   1   0   va3

...........aaaaauaauugugguuuu....................................................   2   0   ya4
.............................................aaaaaccacaauuacuuuugca..............   4   0   ya4
..............................................aaaaccacaauuacuuuugca..............   2   0   ya4
..............................................aaaaccacaauuacuuuugcac.............   4   0   ya4
...............................................aaaccacaauuacuuuugcac.............   1   0   ya4
...............................................aaaccacaauuacuuuugcacc............   4   0   ya4
................................................aaccacaauuacuuuugca..............   3   0   ya4
................................................aaccacaauuacuuuugcacc............   4   0   ya4
................................................aaccacaauuacuuuugcacca...........   2   0   ya4

.......gugcaaaaauaauugugguu......................................................   2   0   SW4
.......gugcaaaaauaauugugguuu.....................................................   3   0   SW4
........ugcaaaaauaauugugguuuu....................................................   1   0   SW4
........ugcaaaaauaauugugguuuug...................................................   4   0   SW4
.............................................aaaaaccacaauuacuuuugca..............   1   0   SW4
..............................................aaaaccacaauuacuuuugcac.............   1   0   SW4
...............................................aaaccacaauuacuuuugcac.............   2   0   SW4
...............................................aaaccacaauuacuuuugcacc............   3   0   SW4
................................................aaccacaauuacuuuugcacca...........   3   0   SW4
.................................................accacaauuacuuuugcacc............   1   0   SW4
.................................................accacaauuacuuuugcacca...........   3   0   SW4

........ugcaaaaauaauugugguuuug...................................................   4   0   ku1
```

Mature   Star

```
uagguuggugcaaaaauaauugugguuuugguaauuuuuaauggcaaaaaccacaauuacuuuugcaccaaccuaauaacu
.............................................aaaaaccacaauuacuuuugca..............    4    0    ku1
................................................aaccacaauuacuuuugcacc............    1    0    ku1

.........gcaaaaauaauugugguuuugg..................................................    1    0    j23
.............................................aaaaaccacaauuacuuuugc...............    1    0    j23
.............................................aaaaaccacaauuacuuuugca..............    2    0    j23
..............................................aaaaccacaauuacuuuugca..............    1    0    j23
................................................aaccacaauuacuuuugcacca...........    1    0    j23
.................................................accacaauuacuuuugcacca...........    1    0    j23

.............................................aaaaaccacaauuacuuuugca..............    2    0    s05

.............................................aaaaaccacaauuacuuu..................    1    0    ta2
..............................................aaaaccacaauuacuuuugcac.............    1    0    ta2

.............................................aaaaaccacaauuacuuuugca..............    1    0    k11

.............................................aaaaaccacaauuacuuuugca..............    1    0    ta1

.........gcaaaaauaauugugguuuugg..................................................    2    0    s09
..........caaaaauaauugugguuuuggu.................................................    1    0    s09
............................................caaaaaccacaauuacuuuugc...............    1    0    s09
..............................................aaaaccacaauuacuuuugca..............    1    0    s09
................................................aaccacaauuacuuuugcacca...........    1    0    s09
.................................................accacaauuacuuuugcacca...........    1    0    s09

............................................caaaaaccacaauuacuuuu.................    1    0    NL2
.............................................aaaaaccacaauuacuuuugca..............    3    0    NL2
................................................aaccacaauuacuuuugcacc............    1    0    NL2
................................................aaccacaauuacuuuugcacca...........    5    0    NL2

.......gugcaaaaauaauugugguuu.....................................................    1    0    j13
........ugcaaaaauaauugugguuuug...................................................    1    0    j13
.........gcaaaaauaauugugguuuugg..................................................    1    0    j13
.............................................aaaaaccacaauuacuuuugca..............    4    0    j13
..............................................aaaaccacaauuacuuuugca..............    2    0    j13
................................................aaccacaauuacuuuugcacca...........    2    0    j13

.......gugcaaaaauaauugugguu......................................................    1    0    j19
.............................................aaaaaccacaauuacuuuugca..............    2    0    j19
...............................................aaaccacaauuacuuuugca..............    2    0    j19
................................................aaccacaauuacuuuugcacca...........    1    0    j19
.................................................accacaauuacuuuugcacca...........    2    0    j19

.............................................aaaaaccacaauuacuuuugca..............    1    0    j25

......................................................aauuacuuuugcaccaaccua......    2    0    per

.......gugcaaaaauaauugugguuu.....................................................    1    0    k04
..............................................aaaaccacaauuacuuuugca..............    1    0    k04
.......................................................auuacuuuugcaccaaccuaau....    1    0    k04

.............................................aaaaaccacaauuacuuuugca..............    2    0    s01
..............................................aaaaccacaauuacuuuugcac.............    2    0    s01
...............................................aaaccacaauuacuuuugcacc............    2    0    s01

.......gugcaaaaauaauugugguu......................................................    1    0    U20
.......gugcaaaaauaauugugguuu.....................................................    25   0    U20
.......gugcaaaaauaauugugguuuu....................................................    8    0    U20
........ugcaaaaauaauugugguuuu....................................................    22   0    U20
........ugcaaaaauaauugugguuuug...................................................    31   0    U20
.........gcaaaaauaauugugguuuugg..................................................    2    0    U20
............................................caaaaaccacaauuacuuuugc...............    1    0    U20
.............................................aaaaaccacaauuacuuuugc...............    22   0    U20
.............................................aaaaaccacaauuacuuuugca..............    17   0    U20
..............................................aaaaccacaauuacuuuugca..............    1    0    U20
..............................................aaaaccacaauuacuuuugcac.............    8    0    U20
...............................................aaaccacaauuacuuuugcac.............    6    0    U20
...............................................aaaccacaauuacuuuugcacc............    14   0    U20
................................................aaccacaauuacuuuugcacc............    6    0    U20
```

Mature **Star**

uagguuggugcaaaaauaauugugguuuugguaauuuuuaauggcaaaaaccacaauuacuuuugcaccaaccuaauaacu			
....aaccacaauuacuuuugcacca...........	1	0	U20
....accacaauuacuuuugcacca...........	15	0	U20
....accacaauuacuuuugcaccaa..........	1	0	U20
.......gugcaaaaauaauugugguuu...	1	0	ku2
........ugcaaaaauaauugugguuuug...	1	0	ku2
........ugcaaaaauaauugugguuuug...	1	0	j21
.........gcaaaaauaauugugguuuugg..	3	0	j21
....aaaaaccacaauuacuuuugca..............	3	0	j21
....aaaaccacaauuacuuuugca..............	1	0	j21
....aaaccacaauuacuuuugcacc............	1	0	j21
....aaccacaauuacuuuugcacc............	1	0	s11
....aaccacaauuacuuuugcacca...........	1	0	s11
....aaaaaccacaauuacuuuugca..............	1	0	j07
.......gugcaaaaauaauuguggu...	1	0	j14
.......gugcaaaaauaauugugguuu...	2	0	j14
.........gcaaaaauaauugugguuuugg..	1	0	j14
....aaaccacaauuacuuuugcacc............	1	0	j14
.......gugcaaaaauaauuguggu...	1	0	j28
.........gcaaaaauaauugugguuuugg..	5	0	j28
..........caaaaauaauugugguuuugg..	1	0	s08
....aaaaaccacaauuacuuuugca..............	1	0	s08
....accacaauuacuuuugcac.............	1	0	s08
.......gugcaaaaauaauugugguuu...	4	0	DLD
........ugcaaaaauaauugugguuuug...	4	0	DLD
....aaaaaccacaauuacuuuugc...............	1	0	DLD
....aaaaccacaauuacuuuugcac.............	1	0	DLD
....aaaccacaauuacuuuugcacc............	3	0	DLD
....accacaauuacuuuugcacc............	1	0	DLD
....accacaauuacuuuugcacca...........	4	0	DLD
....aaaaaccacaauuacuuuugca..............	1	0	k03
.......gugcaaaaauaauugugguuu...	2	0	ya2
........ugcaaaaauaauugugguuuug...	1	0	ya2
...........aaaaauaauugugguuuu..	1	0	ya2
....aaaaaccacaauuacuuuu.................	2	0	ya2
....aaaaaccacaauuacuuuugc...............	1	0	ya2
....aaaaaccacaauuacuuuugca..............	2	0	ya2
....aaaaccacaauuacuuuugca..............	6	0	ya2
....aaaccacaauuacuuuugcac.............	3	0	ya2
....aaaccacaauuacuuuugcacca...........	1	0	ya2
....aaccacaauuacuuuugcacca...........	2	0	ya2
....caauuacuuuugcaccaac.........	1	0	ya2
....aauuacuuuugcaccaac.........	1	0	ya2
........ugcaaaaauaauugugguuuug...	1	0	NL3
.........gcaaaaauaauugugguuuugg..	1	0	NL3
....caaaaaccacaauuacuuuu.................	1	0	NL3
....caaaaaccacaauuacuuuug................	2	0	NL3
....aaaaaccacaauuacuuuugca..............	7	0	NL3
....aaaaccacaauuacuuuugcac.............	1	0	NL3
....aaccacaauuacuuuugcacca...........	1	0	NL3
....auuacuuuugcaccaaccuaa.....	1	0	NL3
........ugcaaaaauaauugugguuuug...	1	0	k06
....aaaccacaauuacuuuugcacc............	1	0	k06
....aaaaaccacaauuacuuuugca..............	1	0	j29
....aaaaccacaauuacuuuugca..............	1	0	j29
....uuggugcaaaaauaauuguggu...	3	0	sh2

Mature **Star**

```
uagguuggugcaaaaauaauugugguuuugguaauuuuuaauggcaaaaaccacaauuacuuuugcaccaaccuaauaacu
........ugcaaaaauaauugugguuuug...................................................	2	0	sh2
............................................caaaaaccacaauuacuuuug................	1	0	sh2
................................................aaccacaauuacuuuugcaccaa..........	2	0	sh2
........................................................uuacuuuugcaccaaccua......	1	0	sh2

.......gugcaaaaauaauugugguuuu....................................................	1	0	MCF
........ugcaaaaauaauugugguuuug...................................................	1	0	MCF
.............................................aaaaaccacaauuacuuuu.................	1	0	MCF
.............................................aaaaaccacaauuacuuuugc...............	1	0	MCF
.............................................aaaaaccacaauuacuuuugca..............	2	0	MCF
..............................................aaaaccacaauuacuuuugcac.............	1	0	MCF
......................................................aauuacuuuugcaccaaccuaa.....	2	0	MCF
......................................................aauuacuuuugcaccaaccuaau....	3	0	MCF
.......................................................auuacuuuugcaccaaccuaau....	4	0	MCF

.......gugcaaaaauaauugugg........................................................	1	0	MBM
.......gugcaaaaauaauugugguuu.....................................................	6	0	MBM
.......gugcaaaaauaauugugguuuu....................................................	4	0	MBM
........ugcaaaaauaauugugguuuu....................................................	4	0	MBM
........ugcaaaaauaauugugguuuug...................................................	14	0	MBM
..........caaaaauaauugugguuuuggu.................................................	3	0	MBM
.............................................aaaaaccacaauuacuuuug................	1	0	MBM
.............................................aaaaaccacaauuacuuuugc...............	1	0	MBM
.............................................aaaaaccacaauuacuuuugca..............	2	0	MBM
..............................................aaaaccacaauuacuuuugca..............	1	0	MBM
..............................................aaaaccacaauuacuuuugcac.............	3	0	MBM
...............................................aaaccacaauuacuuuugcacc............	4	0	MBM
................................................aaccacaauuacuuuugcacc............	1	0	MBM
.................................................accacaauuacuuuugcacca...........	4	0	MBM

....uuggugcaaaaauaauuguggu.......................................................	2	0	li1
........ugcaaaaauaauugugguuuug...................................................	6	0	li1
........ugcaaaaauaauugugguuuugg..................................................	1	0	li1
.........gcaaaaauaauugugguuuugg..................................................	2	0	li1
..........caaaaauaauugugguuuuggu.................................................	1	0	li1
...........................................gcaaaaaccacaauuacuuuug................	1	0	li1
.............................................aaaaaccacaauuacuuuugc...............	1	0	li1
..............................................aaaaccacaauuacuuuugcac.............	1	0	li1
...............................................aaaccacaauuacuuuugcacc............	2	0	li1
................................................aaccacaauuacuuuugcacca...........	1	0	li1

......................................................aauuacuuuugcaccaaccuaa.....	1	0	k13
........................................................uuacuuuugcaccaaccuaaua...	1	0	k13

.......gugcaaaaauaauugugguuu.....................................................	1	0	j20
..........caaaaauaauugugguuuuggua................................................	1	0	j20

.......gugcaaaaauaauugugguuuu....................................................	1	0	A54
........ugcaaaaauaauuguggu.......................................................	4	0	A54
........ugcaaaaauaauugugguuuug...................................................	4	0	A54
.........gcaaaaauaauugugguuuug...................................................	2	0	A54
.........gcaaaaauaauugugguuuugg..................................................	6	0	A54
.............................................aaaaaccacaauuacuuuugc...............	7	0	A54
................................................aaccacaauuacuuuugcacc............	4	0	A54
................................................aaccacaauuacuuuugcacca...........	4	0	A54

.......gugcaaaaauaauugugguu......................................................	1	0	k12
.................................................accacaauuacuuuugcacca...........	1	0	k12
```

Provisional ID	:	hsa_can_1136
Score total	:	2.8
Score for star read(s)	:	-1.3
Score for read counts	:	0
Score for mfe	:	1.1
Score for randfold	:	
Score for cons. seed	:	3
Total read count	:	616
Mature read count	:	616
Loop read count	:	0
Star read count	:	0

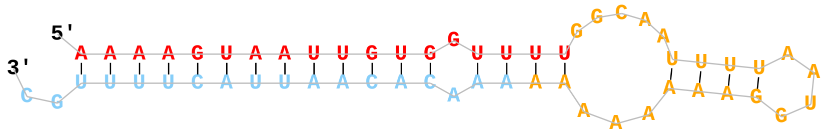

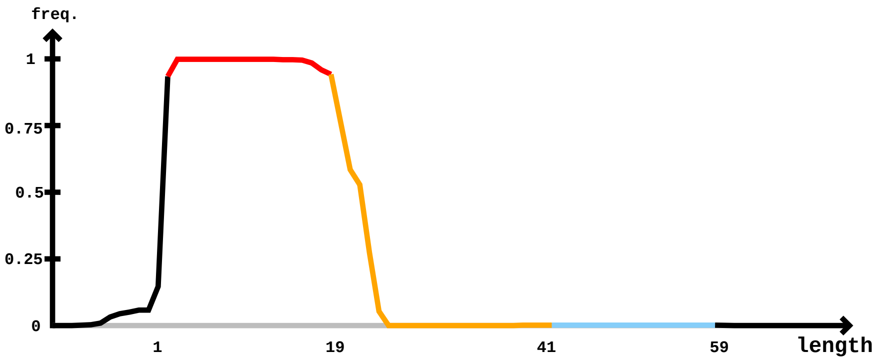

Mature **Star**

```
5'- agguuggugcaaaaguaauuugugguuuuggcaauuuuaauggaaaaaaaaaaacacaauuacuuuugcaccgacauuuuuu  -3'    exp
    ..(((((((((((((((((((((.((((.....((((....))))...)))).)))))))))))))))))))))........   reads      mm         sample
    .........caaaaguaauuugugguuuu.....................................................   5          0          ya1
    ..........aaaaguaauuugugguuuu.....................................................   16         0          ya1
    ..........aaaaguaauuugugguuuug....................................................   3          0          ya1

    ..........aaaaguaauuugugguuuuggc..................................................   1          0          j26
    ..........aaaaguaauuugugguuuuggca.................................................   1          0          j26

    ....uggugcaaaaguaauuugugguu.......................................................   1          0          va1
    ..........aaaaguaauuugugguuuug....................................................   1          0          va1
    ..........aaaaguaauuugugguuuugg...................................................   7          0          va1
    ..........aaaaguaauuugugguuuuggc..................................................   32         0          va1
    ..........aaaaguaauuugugguuuuggca.................................................   20         0          va1
    ..........aaaaguaauuugugguuuuggcaa................................................   5          0          va1
    ...........aaaguaauuugugguuuuggc..................................................   6          0          va1

    ...uuggugcaaaaguaauuugugguuu......................................................   1          0          ku5
    ....uggugcaaaaguaauuuguggu........................................................   1          0          ku5
    ......gugcaaaaguaauuugugguu.......................................................   1          0          ku5

    ...........aaaguaauuugugguuuuggc..................................................   1          0          HeL

    ......gugcaaaaguaauuugugguu.......................................................   1          0          ku4
    ...........aaaguaauuugugguuuuggca.................................................   1          0          ku4
    ...............................................aaaaaacacaauuacuuuugca.............   1          0          ku4

    ..........aaaaguaauuugugguuuuggc..................................................   2          0          s07
    ..........aaaaguaauuugugguuuuggcaa................................................   1          0          s07

    ..........aaaaguaauuugugguuuuggca.................................................   3          0          k08

    ..........aaaaguaauuugugguuuu.....................................................   1          0          ta3
    ..........aaaaguaauuugugguuuugg...................................................   1          0          ta3
    ..........aaaaguaauuugugguuuuggc..................................................   1          0          ta3
    ..........aaaaguaauuugugguuuuggca.................................................   1          0          ta3
```

```
          Mature                                   Star
agguuggugcaaaaguaauugugguuuuggcaauuuuaauggaaaaaaaaaaacacaauuacuuuugcaccgacauuuuuu
..........aaaaguaauugugguuuuggcaa................................................    1    0    ta3

..........aaaaguaauugugguuuuggca.................................................    7    0    j06

..........aaaaguaauugugguuuugg...................................................    2    0    j10
..........aaaaguaauugugguuuuggcaa................................................    2    0    j10

..........aaaaguaauugugguuuuggca.................................................    1    0    k14
...........aaaguaauugugguuuuggca.................................................    1    0    k14
...........aaaguaauugugguuuuggcaa................................................    1    0    k14

..........aaaaguaauugugguuuuggca.................................................    1    0    va2

..........aaaaguaauugugguuuu.....................................................    1    0    j02
..........aaaaguaauugugguuuug....................................................    8    0    j02
..........aaaaguaauugugguuuugg...................................................    2    0    j02
..........aaaaguaauugugguuuuggc..................................................    3    0    j02
..........aaaaguaauugugguuuuggca.................................................    1    0    j02
..........aaaaguaauugugguuuuggcaa................................................    1    0    j02
...........aaaguaauugugguuuuggca.................................................    3    0    j02

.....ggugcaaaaguaauugugguuu......................................................    1    0    j27
..........aaaaguaauugugguuuu.....................................................    1    0    j27
..........aaaaguaauugugguuuuggca.................................................    1    0    j27

...uuggugcaaaaguaauuguggu........................................................    1    0    j31
....uggugcaaaaguaauugugguu.......................................................    1    0    j31
..........aaaaguaauugugguuuuggc..................................................    3    0    j31
..........aaaaguaauugugguuuuggca.................................................    3    0    j31

..........aaaaguaauugugguuuuggc..................................................    2    0    H52

...uuggugcaaaaguaauugugg.........................................................    1    0    k09
..........aaaaguaauugugguuuuggca.................................................    1    0    k09

..........aaaaguaauugugguuuuggc..................................................    3    0    B14

..........aaaaguaauugugguuuugg...................................................    1    0    j09
..........aaaaguaauugugguuuuggc..................................................    3    0    j09
...........aaaguaauugugguuuuggca.................................................    1    0    j09

..........aaaaguaauugugguuuu.....................................................    1    0    j16
..........aaaaguaauugugguuuugg...................................................    1    0    j16

....uggugcaaaaguaauuguggu........................................................    1    0    ku3
..........aaaaguaauugugguuuuggca.................................................    1    0    ku3

.........caaaaguaauugugguuuu.....................................................    1    0    ku6
..........aaaaguaauugugguuuuggc..................................................    1    0    ku6

..........aaaaguaauugugguuuuggca.................................................    2    0    s12

.........caaaaguaauugugguuuu.....................................................    9    0    ya3
.........caaaaguaauugugguuuug....................................................    5    0    ya3
..........aaaaguaauugugguuuu.....................................................    44   0    ya3
..........aaaaguaauugugguuuuggca.................................................    1    0    ya3

..........aaaaguaauugugguuuu.....................................................    1    0    la1
..........aaaaguaauugugguuuuggc..................................................    1    0    la1
...........aaaguaauugugguuuugg...................................................    1    0    la1

....uggugcaaaaguaauugugguu.......................................................    1    0    HEK
..........aaaaguaauugugguuuugg...................................................    2    0    HEK
..........aaaaguaauugugguuuuggc..................................................    11   0    HEK
..........aaaaguaauugugguuuuggca.................................................    2    0    HEK
..........aaaaguaauugugguuuuggcaa................................................    1    0    HEK
...........aaaguaauugugguuuuggc..................................................    2    0    HEK

..........aaaaguaauugugguuuu.....................................................    1    0    j15

..........aaaaguaauugugguuuu.....................................................    1    0    j17
```

```
          Mature                                  Star
agguuggugcaaaaguaauugugguuuuggcaauuuuaauggaaaaaaaaaaacacaauuacuuuugcaccgacauuuuuu
..........aaaaguaauugugguuuuggc..................................................    4     0     j17
..........aaaaguaauugugguuuuggca.................................................    6     0     j17
..........aaaaguaauugugguuuuggcaa................................................    2     0     j17
...........aaaguaauugugguuuuggc..................................................    1     0     j17
...........aaaguaauugugguuuuggca.................................................    1     0     j17

.........caaaaguaauugugguuuug....................................................    1     0     la2
..........aaaaguaauugugguuuug....................................................    2     0     la2
..........aaaaguaauugugguuuuggc..................................................    7     0     la2
..........aaaaguaauugugguuuuggca.................................................    2     0     la2

....uggugcaaaaguaauugugguu.......................................................    1     0     j12

....uggugcaaaaguaauugugguu.......................................................    1     0     li2
...........aaaguaauugugguuuuggc..................................................    1     0     li2

..........aaaaguaauugugguuuug....................................................    1     0     va4

..........aaaaguaauugugguuuugg...................................................    1     0     va3
..........aaaaguaauugugguuuuggc..................................................    11    0     va3
..........aaaaguaauugugguuuuggca.................................................    9     0     va3
..........aaaaguaauugugguuuuggcaa................................................    3     0     va3
...........aaaguaauugugguuuuggca.................................................    2     0     va3

..........aaaaguaauugugguuuugg...................................................    1     0     SW4
..........aaaaguaauugugguuuuggc..................................................    1     0     SW4

.........caaaaguaauugugguuuu.....................................................    16    0     ya4
..........aaaaguaauugugguuuu.....................................................    33    0     ya4

.........caaaaguaauugugguuuugg...................................................    1     0     ku1
..........aaaaguaauugugguuuug....................................................    1     0     ku1
..........aaaaguaauugugguuuugg...................................................    2     0     ku1
..........aaaaguaauugugguuuuggc..................................................    1     0     ku1
..........aaaaguaauugugguuuuggca.................................................    3     0     ku1

.........caaaaguaauugugguuuuggc..................................................    1     0     j23
..........aaaaguaauugugguuuu.....................................................    1     0     j23
..........aaaaguaauugugguuuugg...................................................    2     0     j23
...........aaaguaauugugguuuuggca.................................................    1     0     j23

..........aaaaguaauugugguuuug....................................................    1     0     s05
..........aaaaguaauugugguuuuggc..................................................    1     0     s05

..........aaaaguaauugugguuuuggca.................................................    2     0     ta2
...........aaaguaauugugguuuuggc..................................................    2     0     ta2

.....ggugcaaaaguaauugugguuu......................................................    5     0     j24
.........caaaaguaauugugguuuu.....................................................    3     0     j24

..........aaaaguaauugugguuuuggcaa................................................    1     0     k11

..........aaaaguaauugugguuuu.....................................................    1     0     ta1
..........aaaaguaauugugguuuuggc..................................................    1     0     ta1
..........aaaaguaauugugguuuuggca.................................................    3     0     ta1
..........aaaaguaauugugguuuuggcaa................................................    2     0     ta1

.........caaaaguaauugugguuu......................................................    1     0     NL2
.........caaaaguaauugugguuuu.....................................................    1     0     NL2
.........caaaaguaauugugguuuug....................................................    2     0     NL2
..........aaaaguaauugugguuuu.....................................................    10    0     NL2
..........aaaaguaauugugguuuuggca.................................................    3     0     NL2
..........aaaaguaauugugguuuuggcaa................................................    1     0     NL2
...........aaaguaauugugguuuuggcaa................................................    2     0     NL2

....uggugcaaaaguaauugugguu.......................................................    1     0     k10
..........aaaaguaauugugguuuuggca.................................................    2     0     k10

..........aaaaguaauugugguuuu.....................................................    1     0     j13

..........aaaaguaauugugguuuu.....................................................    2     0     j19
```

```
          Mature                                  Star
agguuggugcaaaaguaauugugguuuuggcaauuuuaauggaaaaaaaaaaacacaauuacuuuugcaccgacauuuuuu
..........aaaaguaauugugguuuuggca.................................................    1    0    j19

..........aaaaguaauugugguuuuggca.................................................    4    0    j03

..........aaaaguaauugugguuuuggcaa................................................    1    0    k01

....uggugcaaaaguaauugugguu.......................................................    1    0    j25
.......ugcaaaaguaauugugguu.......................................................    2    0    j25
.........caaaaguaauugugguuuu.....................................................    1    0    j25

..........aaaaguaauugugguuuu.....................................................    1    0    per
..........aaaaguaauugugguuuug....................................................    1    0    per

..........aaaaguaauugugguuuu.....................................................    1    0    k04
..........aaaaguaauugugguuuuggc..................................................    1    0    k04
..........aaaaguaauugugguuuuggcaa................................................    1    0    k04

....uggugcaaaaguaauuguggu........................................................    2    0    U20
....uggugcaaaaguaauugugguu.......................................................    1    0    U20
......gugcaaaaguaauugugguuuu.....................................................    1    0    U20

..........aaaaguaauugugguuuug....................................................    1    0    ku2
..........aaaaguaauugugguuuugg...................................................    6    0    ku2
..........aaaaguaauugugguuuuggc..................................................    5    0    ku2
..........aaaaguaauugugguuuuggca.................................................    2    0    ku2
...........aaaguaauugugguuuuggc..................................................    1    0    ku2

..........aaaaguaauugugguuuug....................................................    2    0    j21
..........aaaaguaauugugguuuugg...................................................    3    0    j21
..........aaaaguaauugugguuuuggc..................................................    14   0    j21
..........aaaaguaauugugguuuuggca.................................................    13   0    j21
..........aaaaguaauugugguuuuggcaa................................................    2    0    j21
...........aaaguaauugugguuuuggc..................................................    4    0    j21
...........aaaguaauugugguuuuggca.................................................    1    0    j21

..........aaaaguaauugugguuuuggca.................................................    2    0    j07
..........aaaaguaauugugguuuuggcaa................................................    1    0    j07

..........aaaaguaauugugguuuuggc..................................................    1    0    k02

..........aaaaguaauugugguuuuggc..................................................    1    0    j28
..........aaaaguaauugugguuuuggca.................................................    4    0    j28

..guuggugcaaaaguaauuguggu........................................................    1    0    k03
..........aaaaguaauugugguuuuggca.................................................    3    0    k03
...........aaaguaauugugguuuuggc..................................................    2    0    k03

.........caaaaguaauugugguuu......................................................    1    0    NL3
.........caaaaguaauugugguuuu.....................................................    1    0    NL3
..........aaaaguaauugugguuuu.....................................................    15   0    NL3
..........aaaaguaauugugguuuuggc..................................................    1    0    NL3
..........aaaaguaauugugguuuuggca.................................................    4    0    NL3
..........aaaaguaauugugguuuuggcaa................................................    2    0    NL3

.........caaaaguaauugugguuu......................................................    1    0    ya2
.........caaaaguaauugugguuuu.....................................................    6    0    ya2
..........aaaaguaauugugguuuu.....................................................    24   0    ya2

..........aaaaguaauugugguuuuggca.................................................    2    0    k06
..........aaaaguaauugugguuuuggcaa................................................    2    0    k06

..........aaaaguaauugugguuuuggca.................................................    2    0    j29
..........aaaaguaauugugguuuuggcaa................................................    1    0    j29

.gguuggugcaaaaguaauugu...........................................................    1    0    sh2
...uuggugcaaaaguaauugugguu.......................................................    1    0    sh2
......gugcaaaaguaauugugguu.......................................................    1    0    sh2
..........aaaaguaauugugguuuuggc..................................................    2    0    sh2
..........aaaaguaauugugguuuuggca.................................................    2    0    sh2

.....ggugcaaaaguaauugugguuu......................................................    1    0    j01
```

```
      Mature                                   Star
agguuggugcaaaaguaauugugguuuuggcaauuuuaauggaaaaaaaaaaacacaauuacuuuugcaccgacauuuuuu
.......ugcaaaaguaauugugguuuu.....................................................    3    0    j01

....uggugcaaaaguaauuguggu........................................................    1    0    MCF
..........aaaaguaauugugguuuuggc..................................................    1    0    MCF
..........aaaaguaauugugguuuuggca.................................................    1    0    MCF

..........aaaaguaauugugguuuuggca.................................................    5    0    NL1
...........aaaguaauugugguuuuggca.................................................    1    0    NL1

.....ggugcaaaaguaauugugguu.......................................................    1    0    k07
..........aaaaguaauugugguuuuggca.................................................    3    0    k07

....uggugcaaaaguaauugugguu.......................................................    1    0    li1
..........aaaaguaauugugguuuuggc..................................................    19   0    li1
..........aaaaguaauugugguuuuggca.................................................    1    0    li1
..........aaaaguaauugugguuuuggcaa................................................    1    0    li1
...........aaaguaauugugguuuuggc..................................................    4    0    li1

..........aaaaguaauugugguuuuggc..................................................    1    0    k13
..........aaaaguaauugugguuuuggca.................................................    2    0    k13

.........caaaaguaauugugguuuu.....................................................    1    0    j20
..........aaaaguaauugugguuuug....................................................    1    0    j20
..........aaaaguaauugugguuuugg...................................................    4    0    j20
..........aaaaguaauugugguuuuggc..................................................    4    0    j20
..........aaaaguaauugugguuuuggca.................................................    4    0    j20
..........aaaaguaauugugguuuuggcaa................................................    1    0    j20

....uggugcaaaaguaauugugguu.......................................................    1    0    j30

.........caaaaguaauugugguuuuggc..................................................    1    0    k12
..........aaaaguaauugugguuuuggc..................................................    1    0    k12
...........aaaguaauugugguuuuggca.................................................    2    0    k12
```

Provisional ID	:	hsa_can_1155
Score total	:	-5.9
Score for star read(s)	:	-1.3
Score for read counts	:	0
Score for mfe	:	-4
Score for randfold	:	
Score for cons. seed	:	-0.6
Total read count	:	1282
Mature read count	:	1282
Loop read count	:	0
Star read count	:	0

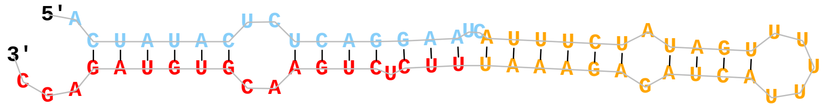

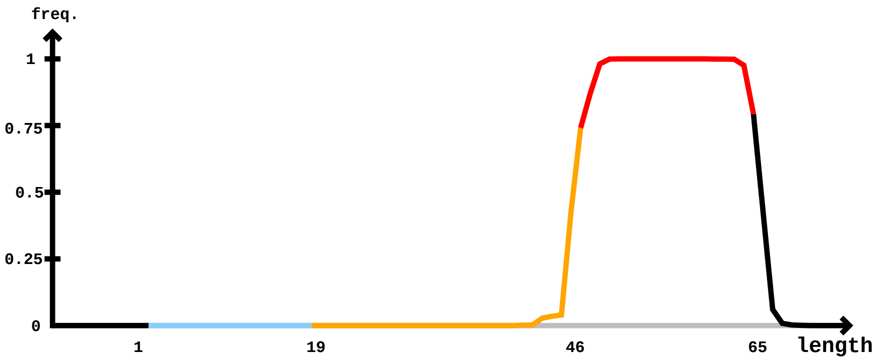

Star Mature

```
5'- ucagaaagacuauacucucaggaaucauuucuauaguuuuuuacuagagaaauuucucugaacguguagagcacuggaaac  -3'   exp
    ((((.....((((((..(((((((..((((((.((((.....)))).))))))))).))))..))))))....))))....    reads   mm   sample
    ....................................................uuucucugaacguguagag..........    1       0    ya1
    ....................................................uuucucugaacguguagagcac.......    1       0    ya1

    .....................................................uucucugaacguguagagc.........    3       0    s04

    ................................................gaaauuucucugaacguguagag..........    1       0    j26
    ...................................................auuucucugaacguguagag..........    1       0    j26
    ....................................................uuucucugaacguguaga...........    4       0    j26
    ....................................................uuucucugaacguguagag..........    41      0    j26
    ....................................................uuucucugaacguguagagc.........    22      0    j26
    ....................................................uuucucugaacguguagagca........    42      0    j26
    ....................................................uuucucugaacguguagagcac.......    1       0    j26
    .....................................................uucucugaacguguagag..........    30      0    j26
    .....................................................uucucugaacguguagagc.........    12      0    j26
    .....................................................uucucugaacguguagagca........    9       0    j26
    ......................................................ucucugaacguguagagc.........    25      0    j26
    ......................................................ucucugaacguguagagca........    17      0    j26
    ......................................................ucucugaacguguagagcac.......    2       0    j26
    ......................................................ucucugaacguguagagcacug.....    1       0    j26
    .......................................................cucugaacguguagagca........    29      0    j26

    ....................................................uuucucugaacguguagagc.........    1       0    va1
    ....................................................uuucucugaacguguagagca........    1       0    va1
    .....................................................uucucugaacguguagagca........    1       0    va1
    ......................................................ucucugaacguguagagca........    2       0    va1

    ....................................................uuucucugaacguguagagca........    2       0    ku5
    .....................................................uucucugaacguguagagca........    2       0    ku5

    .................................................aaauuucucugaacguguagag..........    2       0    k05
    ....................................................uuucucugaacguguagag..........    1       0    k05
    ....................................................uuucucugaacguguagagca........    14      0    k05
    ....................................................uuucucugaacguguagagcac.......    3       0    k05
    .....................................................uucucugaacguguagagca........    3       0    k05
```

Star **Mature**

ucagaaagacuauacucucaggaaucauuucuauaguuuuuuacuagagaaauuucucugaacguguagagcacuggaaac			
....uucucugaacguguagagcac.......	1	0	k05
....ucucugaacguguagagc.........	2	0	k05
....ucucugaacguguagagca........	8	0	k05
....ucucugaacguguagagcac.......	2	0	k05
....cucugaacguguagagca........	7	0	k05
....ucugaacguguagagcac.......	1	0	k05
....uuucucugaacguguagagca........	2	0	s06
....uucucugaacguguagagc.........	4	0	s06
....uuucucugaacguguagagca........	1	0	ku4
....uucucugaacguguagag..........	1	0	ku4
....uucucugaacguguagagca........	3	0	ku4
....uuucucugaacguguagagca........	2	0	s07
....uuucucugaacguguagagcac.......	1	0	s07
....uuucucugaacguguaga...........	1	0	s10
....uuucucugaacguguagagcac.......	1	0	s10
....uucucugaacguguagagc.........	2	0	s10
....ucucugaacguguagagca........	1	0	s10
....aaauuucucugaacguguaga...........	1	0	k08
....uucucugaacguguagagca........	1	0	k08
....ucucugaacguguagagc.........	1	0	k08
....uuucucugaacguguagagca........	5	0	j06
....uucucugaacguguagagca........	9	0	j06
....uuucucugaacguguaga...........	2	0	j10
....uuucucugaacguguagagca........	1	0	j10
....uucucugaacguguagagca........	2	0	j10
....ucucugaacguguagagca........	1	0	j10
....cucugaacguguagagca........	2	0	j10
....uuucucugaacguguagagca........	1	0	j04
....uucucugaacguguagag..........	3	0	j04
....uucucugaacguguagagc.........	1	0	j04
....uuucucugaacguguagagca........	2	0	k14
....uucucugaacguguagag..........	1	0	k14
....ucucugaacguguagagca........	1	0	k14
....ucucugaacguguagagcac.......	1	0	k14
....uuucucugaacguguagag..........	1	0	j02
....uuucucugaacguguagagc.........	3	0	j02
....uuucucugaacguguagagca........	3	0	j02
....uucucugaacguguagagca........	1	0	j02
....uuucucugaacguguaga...........	1	0	va2
....uuucucugaacguguagagca........	2	0	va2
....uucucugaacguguagag..........	1	0	va2
....uucucugaacguguagagca........	2	0	va2
....ucucugaacguguagagca........	1	0	va2
....cucugaacguguagagca........	1	0	va2
....uuucucugaacguguagagc.........	2	0	j27
....uuucucugaacguguagagca........	1	0	j27
....uucucugaacguguagag..........	13	0	j27
....uucucugaacguguagagc.........	5	0	j27
....uucucugaacguguagagca........	8	0	j27
....cucugaacguguagagca........	9	0	j27
....aaauuucucugaacguguag............	1	0	j31
....aaauuucucugaacguguaga...........	2	0	j31
....aaauuucucugaacguguagag..........	3	0	j31
....aaauuucucugaacguguagagc.........	3	0	j31
....aauuucucugaacguguagag..........	1	0	j31
....aauuucucugaacguguagagc.........	1	0	j31
....auuucucugaacguguagagc.........	2	0	j31

Star Mature

```
ucagaaagacuauacucucaggaaucauuucuauaguuuuuuacuagagaaauuucucugaacguguagagcacuggaaac
...................................................auuucucugaacguguagagca........	4	0	j31
.....................................................uucucugaacguguagag..........	1	0	j31
.....................................................uucucugaacguguagagc.........	1	0	j31
.....................................................uucucugaacguguagagca........	1	0	j31
.......................................................cucugaacguguagagca........	1	0	j31

....................................................uuucucugaacguguagagcac.......	2	0	H52

.................................................aaauuucucugaacguguagag..........	1	0	s03
.....................................................uucucugaacguguagagca........	1	0	s03

....................................................uuucucugaacguguagagca........	6	0	j22
.....................................................uucucugaacguguagag..........	1	0	j22
.......................................................cucugaacguguagagca........	4	0	j22

.................................................aaauuucucugaacguguagagc.........	1	0	k09
....................................................uuucucugaacguguaga...........	3	0	k09
....................................................uuucucugaacguguagag..........	5	0	k09
....................................................uuucucugaacguguagagc.........	2	0	k09
....................................................uuucucugaacguguagagca........	16	0	k09
.....................................................uucucugaacguguagag..........	4	0	k09
.....................................................uucucugaacguguagagc.........	3	0	k09
.....................................................uucucugaacguguagagca........	5	0	k09
.....................................................uucucugaacguguagagcac.......	1	0	k09
......................................................ucucugaacguguagagc.........	6	0	k09
......................................................ucucugaacguguagagca........	18	0	k09
......................................................ucucugaacguguagagcac.......	1	0	k09
.......................................................cucugaacguguagagca........	11	0	k09
.......................................................cucugaacguguagagcac.......	2	0	k09
........................................................ucugaacguguagagcac.......	2	0	k09

....................................................uuucucugaacguguagag..........	1	0	B14
....................................................uuucucugaacguguagagc.........	1	0	B14

....................................................uuucucugaacguguagagca........	5	0	j18
.....................................................uucucugaacguguagagca........	4	0	j18
.....................................................uucucugaacguguagagcac.......	4	0	j18

....................................................uuucucugaacguguagagca........	1	0	ku3
.....................................................uucucugaacguguagagca........	1	0	ku3

.................................................aaauuucucugaacguguaga...........	1	0	j16
....................................................uuucucugaacguguagagc.........	2	0	j16
....................................................uuucucugaacguguagagca........	8	0	j16
.....................................................uucucugaacguguagag..........	1	0	j16
.....................................................uucucugaacguguagagca........	1	0	j16

.................................................aaauuucucugaacguguagag..........	1	0	j09
....................................................uuucucugaacguguagag..........	1	0	j09
....................................................uuucucugaacguguagagc.........	1	0	j09
....................................................uuucucugaacguguagagca........	1	0	j09
.....................................................uucucugaacguguagag..........	5	0	j09
.....................................................uucucugaacguguagagc.........	1	0	j09
.....................................................uucucugaacguguagagca........	1	0	j09
......................................................ucucugaacguguagagc.........	1	0	j09
.......................................................cucugaacguguagagcac.......	1	0	j09

.....................................................uucucugaacguguagagca........	2	0	ku6

.................................................aaauuucucugaacguguagagc.........	1	0	j11
....................................................uuucucugaacguguaga...........	1	0	j11
....................................................uuucucugaacguguagag..........	3	0	j11
....................................................uuucucugaacguguagagc.........	1	0	j11
....................................................uuucucugaacguguagagca........	4	0	j11
....................................................uuucucugaacguguagagcacu......	2	0	j11
.....................................................uucucugaacguguagag..........	2	0	j11
.....................................................uucucugaacguguagagca........	4	0	j11
......................................................ucucugaacguguagagca........	7	0	j11
.......................................................cucugaacguguagagca........	7	0	j11

.................................................aaauuucucugaacguguagag..........	1	0	s12
```

Star Mature

ucagaaagacuauacucucaggaaucauuucuauaguuuuuuacuagagaaauuucucugaacguguagagcacuggaaac

....uuucucugaacguguaga...........	1	0	s12
....uuucucugaacguguagagc.........	1	0	s12
....uuucucugaacguguagagca........	7	0	s12
....uuucucugaacguguagagcac.......	1	0	s12
....uucucugaacguguagag..........	4	0	s12
....uucucugaacguguagagc.........	20	0	s12
....ucucugaacguguagagc.........	1	0	s12
....ucucugaacguguagagca........	1	0	s12
....uucucugaacguguagagc.........	1	0	ya3
....uuucucugaacguguagag..........	2	0	la1
....uuucucugaacguguagagc.........	1	0	la1
....uuucucugaacguguagagca........	5	0	la1
....uucucugaacguguagag..........	5	0	la1
....uucucugaacguguagagc.........	1	0	la1
....uucucugaacguguagagca........	1	0	la1
....uucucugaacguguagagcac.......	1	0	la1
....ucucugaacguguagagc.........	1	0	la1
....ucucugaacguguagagca........	2	0	la1
....cucugaacguguagagca........	2	0	la1
....ucugaacguguagagcac.......	1	0	la1
....aaauuucucugaacguguagag..........	1	0	j17
....uuucucugaacguguagagca........	6	0	j17
....uucucugaacguguagagca........	2	0	j17
....cucugaacguguagagca........	6	0	j17
....aaauuucucugaacguguagagc.........	1	0	j15
....uuucucugaacguguagag..........	2	0	j15
....uuucucugaacguguagagc.........	1	0	j15
....uuucucugaacguguagagca........	6	0	j15
....uucucugaacguguagag..........	1	0	j15
....uucucugaacguguagagc.........	1	0	j15
....ucucugaacguguagagca........	1	0	j15
....uuucucugaacguguagag..........	2	0	la2
....uuucucugaacguguagagc.........	3	0	la2
....uuucucugaacguguagagca........	5	0	la2
....uucucugaacguguagag..........	6	0	la2
....uucucugaacguguagagc.........	43	0	la2
....uucucugaacguguagagca........	7	0	la2
....ucucugaacguguagagc.........	2	0	la2
....ucucugaacguguagagca........	1	0	la2
....uuucucugaacguguagag..........	4	0	j12
....uuucucugaacguguagagca........	4	0	j12
....uucucugaacguguagag..........	1	0	j12
....uucucugaacguguagagca........	1	0	j12
....ucucugaacguguagagca........	2	0	j12
....cucugaacguguagagca........	6	0	j12
....ucugaacguguagagcac.......	2	0	j12
....aauuucucugaacguguagagc.........	1	0	li2
....uuucucugaacguguagagc.........	2	0	li2
....uuucucugaacguguagagcac.......	1	0	li2
....uuucucugaacguguagagcacu......	1	0	li2
....uucucugaacguguagagc.........	3	0	li2
....ucucugaacguguagagc.........	4	0	li2
....cucugaacguguagagca........	1	0	li2
....uuucucugaacguguagagc.........	1	0	va4
....uuucucugaacguguagagca........	4	0	va4
....uuucucugaacguguagagcac.......	1	0	va4
....uuucucugaacguguagagca........	6	0	va3
....uucucugaacguguagag..........	2	0	va3
....uucucugaacguguagagc.........	17	0	va3
....uucucugaacguguagagca........	6	0	va3
....uucucugaacguguagagcac.......	1	0	va3

```
         Star                                       Mature
ucagaaagacuauacucucaggaaucauuucuauaguuuuuuacuagagaaauuucucugaacguguagagcacuggaaac
......................................................ucucugaacguguagagc.........	3	0	va3
......................................................ucucugaacguguagagca........	1	0	va3
.......................................................cucugaacguguagagca........	4	0	va3

...............................................agaaauuucucugaacgugu..............	1	0	ya4

....................................................uuucucugaacguguagag..........	1	0	ku1
....................................................uuucucugaacguguagagca........	3	0	ku1
......................................................ucucugaacguguagagca........	1	0	ku1
.......................................................cucugaacguguagagca........	2	0	ku1

...............................................agaaauuucucugaacguguaga...........	1	0	j23
.................................................aaauuucucugaacguguagag..........	1	0	j23
..................................................aauuucucugaacguguagag..........	1	0	j23
..................................................aauuucucugaacguguagagca........	1	0	j23
....................................................uuucucugaacguguagag..........	4	0	j23
....................................................uuucucugaacguguagagc.........	7	0	j23
....................................................uuucucugaacguguagagca........	32	0	j23
.....................................................uucucugaacguguagag..........	3	0	j23
.......................................................cucugaacguguagagca........	1	0	j23

....................................................uuucucugaacguguagagca........	1	0	s05
.....................................................uucucugaacguguagag..........	1	0	s05

......................................................ucucugaacguguagagc.........	1	0	ta2

....................................................uuucucugaacguguaga...........	1	0	j24
....................................................uuucucugaacguguagagca........	1	0	j24
.....................................................uucucugaacguguagagc.........	2	0	j24
.....................................................uucucugaacguguagagca........	4	0	j24
.....................................................uucucugaacguguagagcac.......	2	0	j24
......................................................ucucugaacguguagagca........	3	0	j24
......................................................ucucugaacguguagagcac.......	1	0	j24
.......................................................cucugaacguguagagca........	7	0	j24

....................................................uuucucugaacguguagagca........	1	0	k11
......................................................ucucugaacguguagagc.........	1	0	k11
......................................................ucucugaacguguagagca........	1	0	k11

....................................................uuucucugaacguguagagca........	6	0	s09
.....................................................uucucugaacguguagag..........	2	0	s09
.....................................................uucucugaacguguagagc.........	7	0	s09
.....................................................uucucugaacguguagagca........	2	0	s09
......................................................ucucugaacguguagagc.........	2	0	s09

.....................................................uucucugaacguguagagc.........	5	0	NL2
.....................................................uucucugaacguguagagca........	1	0	NL2

.................................................aaauuucucugaacguguaga...........	1	0	j13
.................................................aaauuucucugaacguguagag..........	3	0	j13
..................................................aauuucucugaacguguagagc.........	1	0	j13
....................................................uuucucugaacguguagag..........	1	0	j13
....................................................uuucucugaacguguagagca........	3	0	j13
.......................................................cucugaacguguagagca........	1	0	j13

....................................................uuucucugaacguguagagc.........	1	0	k10
....................................................uuucucugaacguguagagca........	2	0	k10
....................................................uuucucugaacguguagagcac.......	1	0	k10
.....................................................uucucugaacguguagag..........	2	0	k10
......................................................ucucugaacguguagagc.........	1	0	k10
......................................................ucucugaacguguagagca........	2	0	k10

.................................................aaauuucucugaacguguagag..........	4	0	j19
..................................................aauuucucugaacguguagagc.........	1	0	j19
....................................................uuucucugaacguguagagca........	3	0	j19
.....................................................uucucugaacguguagag..........	2	0	j19
.......................................................cucugaacguguagagca........	4	0	j19

....................................................uuucucugaacguguagag..........	1	0	j25
....................................................uuucucugaacguguagagca........	1	0	j25
```

Star Mature

```
ucagaaagacuauacucucaggaaucauuucuauaguuuuuuacuagagaaauuucucugaacguguagagcacuggaaac
.....................................................uucucugaacguguagag..........    5    0    j25
.....................................................uucucugaacguguagagc.........    1    0    j25
.......................................................cucugaacguguagagca........    1    0    j25

....................................................uuucucugaacguguagag..........    1    0    k01
....................................................uuucucugaacguguagagca........    1    0    k01
....................................................uuucucugaacguguagagcacu......    1    0    k01
.....................................................uucucugaacguguagag..........    1    0    k01
.....................................................uucucugaacguguagagca........    1    0    k01
.......................................................cucugaacguguagagca........    1    0    k01

.................................................aaauuucucugaacguguagag..........    1    0    s01
....................................................uuucucugaacguguagag..........    1    0    s01
....................................................uuucucugaacguguagagca........    1    0    s01
.....................................................uucucugaacguguagag..........    3    0    s01
.....................................................uucucugaacguguagagc.........    2    0    s01
.....................................................uucucugaacguguagagca........    9    0    s01
......................................................ucucugaacguguagagca........    4    0    s01
.......................................................cucugaacguguagagcac.......    1    0    s01

....................................................uuucucugaacguguagag..........    1    0    per
......................................................ucucugaacguguagagca........    1    0    per

....................................................uuucucugaacguguagagc.........    1    0    k04
....................................................uuucucugaacguguagagca........    7    0    k04
....................................................uuucucugaacguguagagcac.......    1    0    k04
....................................................uuucucugaacguguagagcacu......    1    0    k04
.....................................................uucucugaacguguagagc.........    1    0    k04

.....................................................uucucugaacguguagag..........    1    0    ku2
.....................................................uucucugaacguguagagca........    2    0    ku2
.......................................................cucugaacguguagagca........    2    0    ku2
........................................................ucugaacguguagagcac.......    2    0    ku2

....................................................uuucucugaacguguagag..........    1    0    j21
....................................................uuucucugaacguguagagc.........    3    0    j21
....................................................uuucucugaacguguagagca........    11   0    j21
....................................................uuucucugaacguguagagcac.......    1    0    j21
.....................................................uucucugaacguguagagca........    1    0    j21
......................................................ucucugaacguguagagc.........    1    0    j21
......................................................ucucugaacguguagagca........    1    0    j21
.......................................................cucugaacguguagagca........    1    0    j21
........................................................ucugaacguguagagcac.......    1    0    j21

....................................................uuucucugaacguguagagca........    3    0    s11
.......................................................cucugaacguguagagca........    1    0    s11

.....................................................uucucugaacguguagag..........    3    0    j07
.....................................................uucucugaacguguagagca........    1    0    j07

....................................................uuucucugaacguguagagc.........    1    0    j14
....................................................uuucucugaacguguagagca........    2    0    j14
.....................................................uucucugaacguguagagca........    3    0    j14
......................................................ucucugaacguguagagc.........    1    0    j14
.......................................................cucugaacguguagagca........    2    0    j14
.........................................................cugaacguguagagcacugg....    1    0    j14

....................................................uuucucugaacguguagagc.........    1    0    k02
....................................................uuucucugaacguguagagca........    1    0    k02
.....................................................uucucugaacguguagag..........    1    0    k02
.....................................................uucucugaacguguagagc.........    2    0    k02
.....................................................uucucugaacguguagagca........    1    0    k02
......................................................ucucugaacguguagagca........    1    0    k02

....................................................uuucucugaacguguagagca........    1    0    j28
.....................................................uucucugaacguguagagca........    3    0    j28

....................................................uuucucugaacguguagagc.........    2    0    s08
....................................................uuucucugaacguguagagca........    4    0    s08
.....................................................uucucugaacguguagag..........    3    0    s08
```

Star   Mature

```
ucagaaagacuauacucucaggaaucauuucuauaguuuuuuacuagagaaauuucucugaacguguagagcacuggaaac
.....................................................uucucugaacguguagagc.........   8   0   s08

....................................................uuucucugaacguguagagc.........   2   0   k03
....................................................uuucucugaacguguagagca........   7   0   k03
....................................................uuucucugaacguguagagcac.......   1   0   k03
.....................................................uucucugaacguguagag..........   1   0   k03
.....................................................uucucugaacguguagagc.........   1   0   k03
.....................................................uucucugaacguguagagca........   6   0   k03
.....................................................uucucugaacguguagagcac.......   1   0   k03
......................................................ucucugaacguguagagc.........   3   0   k03
......................................................ucucugaacguguagagca........   10  0   k03
......................................................ucucugaacguguagagcac.......   3   0   k03
......................................................ucucugaacguguagagcacu......   1   0   k03
.......................................................cucugaacguguagagca........   10  0   k03
.......................................................cucugaacguguagagcac.......   2   0   k03

....................................................uuucucugaacguguagag..........   1   0   NL3
.....................................................uucucugaacguguagagca........   2   0   NL3

....................................................uuucucugaacguguagag..........   1   0   ya2
.....................................................uucucugaacguguagag..........   1   0   ya2

.................................................aaauuucucugaacguguagag..........   1   0   s02
....................................................uuucucugaacguguagagc.........   1   0   s02
....................................................uuucucugaacguguagagca........   1   0   s02

....................................................uuucucugaacguguagagca........   6   0   k06
.....................................................uucucugaacguguagag..........   1   0   k06
.....................................................uucucugaacguguagagca........   1   0   k06
......................................................ucucugaacguguagagc.........   1   0   k06
......................................................ucucugaacguguagagca........   2   0   k06
.......................................................cucugaacguguagagca........   2   0   k06

....................................................uuucucugaacguguaga...........   3   0   sh2
....................................................uuucucugaacguguagag..........   2   0   sh2
.....................................................uucucugaacguguagag..........   1   0   sh2

....................................................uuucucugaacguguagagc.........   1   0   j29
....................................................uuucucugaacguguagagca........   1   0   j29
.....................................................uucucugaacguguagagc.........   2   0   j29
.....................................................uucucugaacguguagagca........   3   0   j29
......................................................ucucugaacguguagagca........   1   0   j29
.......................................................cucugaacguguagagca........   1   0   j29

....................................................uuucucugaacguguagagc.........   2   0   j01
.....................................................uucucugaacguguagagca........   1   0   j01
........................................................ucugaacguguagagcac.......   1   0   j01

.....................................................uucucugaacguguagag..........   1   0   NL1

......................................................ucucugaacguguagagca........   1   0   k07

.....................................................uucucugaacguguagagc.........   2   0   sh1

....................................................uuucucugaacguguaga...........   3   0   li1
....................................................uuucucugaacguguagag..........   2   0   li1
....................................................uuucucugaacguguagagca........   1   0   li1
....................................................uuucucugaacguguagagcacu......   2   0   li1
.....................................................uucucugaacguguagag..........   1   0   li1
.......................................................cucugaacguguagagcac.......   1   0   li1
........................................................ucugaacguguagagcac.......   4   0   li1

....................................................uuucucugaacguguagag..........   1   0   k13
....................................................uuucucugaacguguagagca........   1   0   k13
.....................................................uucucugaacguguagag..........   1   0   k13
......................................................ucucugaacguguagagca........   3   0   k13

.................................................aaauuucucugaacguguaga...........   1   0   j20
.................................................aaauuucucugaacguguagag..........   2   0   j20
..................................................aauuucucugaacguguagagc.........   1   0   j20
```

Star Mature

```
ucagaaagacuauacucucaggaaucauuucuauaguuuuuuacuagagaaauuucucugaacguguagagcacuggaaac
....................................................uuucucugaacguguaga...........    3    0    j20
....................................................uuucucugaacguguagag..........    10   0    j20
....................................................uuucucugaacguguagagc.........    5    0    j20
....................................................uuucucugaacguguagagca........    24   0    j20
....................................................uuucucugaacguguagagcac.......    2    0    j20
.....................................................uucucugaacguguagag..........    8    0    j20
.....................................................uucucugaacguguagagc.........    4    0    j20
.....................................................uucucugaacguguagagca........    3    0    j20
......................................................ucucugaacguguagagc.........    3    0    j20
......................................................ucucugaacguguagagca........    1    0    j20
.......................................................cucugaacguguagagca........    13   0    j20
........................................................ucugaacguguagagcac.......    9    0    j20
........................................................ucugaacguguagagcacug.....    1    0    j20

..................................................aauuucucugaacguguagagc.........    1    0    k12
....................................................uuucucugaacguguagagc.........    2    0    k12
....................................................uuucucugaacguguagagca........    5    0    k12
.....................................................uucucugaacguguagag..........    2    0    k12
.....................................................uucucugaacguguagagc.........    1    0    k12
......................................................ucucugaacguguagagca........    3    0    k12

....................................................uuucucugaacguguagagc.........    1    0    j30
....................................................uuucucugaacguguagagca........    8    0    j30
.....................................................uucucugaacguguagagc.........    1    0    j30
......................................................ucucugaacguguagagca........    1    0    j30
.......................................................cucugaacguguagagca........    1    0    j30
```

Provisional ID	:	hsa_can_1194
Score total	:	9.1
Score for star read(s)	:	3.9
Score for read counts	:	5.2
Score for mfe	:	0.6
Score for randfold	:	
Score for cons. seed	:	-0.6
Total read count	:	22
Mature read count	:	21
Loop read count	:	0
Star read count	:	1

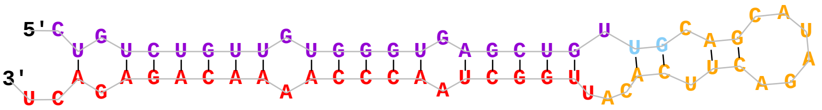

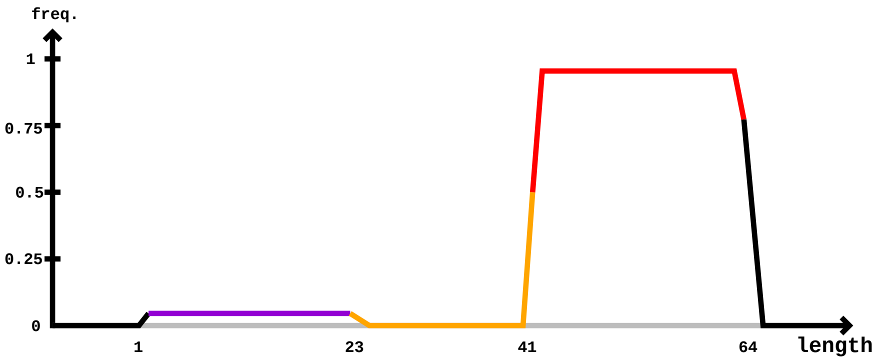

Star **Mature**

```
5'- uccccgggcugucuguugugggugagcuguugcagcauagacuucacauuggcuaacccaaaacagagacugggaggagcc  -3'   obs
    uccccgggcugucuguugugggugagcuguugcagcauagacuucacauuggcuaacccaaaacagagacugggaggagcc         exp
    (((((.((.(.((((((.(((((.(((((.((.((......)).))...))))).))))).)))))).))).)).)))...    reads   mm   sample
    ................................................uuggcuaacccaaaacagagacu..........    1       0    j23
    .................................................uggcuaacccaaaacagagacu..........    1       0    j23

    ................................................uuggcuaacccaaaacagagacu..........    1       0    j26

    .................................................uggcuaacccaaaacagagacu..........    1       0    k04

    ................................................uuggcuaacccaaaacagagacu..........    1       0    U20
    .................................................uggcuaacccaaaacagagacu..........    4       0    U20

    ................................................uuggcuaacccaaaacagagacu..........    1       0    k14

    .................................................uggcuaacccaaaacagagacu..........    1       0    s06

    ................................................uuggcuaacccaaaacagagacu..........    1       0    HEK
    .................................................uggcuaacccaaaacagagac...........    2       0    HEK

    ................................................uuggcuaacccaaaacagagacu..........    4       0    sh2

    ........cugucuguugugggugagcugu...................................................    1       0    ku1

    .................................................uggcuaacccaaaacagagacu..........    1       0    j15

    ................................................uuggcuaacccaaaacagagac...........    2       0    MBM
```

Provisional ID	:	hsa_can_1203
Score total	:	36.8
Score for star read(s)	:	3.9
Score for read counts	:	31.2
Score for mfe	:	2.3
Score for randfold	:	
Score for cons. seed	:	-0.6
Total read count	:	73
Mature read count	:	30
Loop read count	:	0
Star read count	:	43

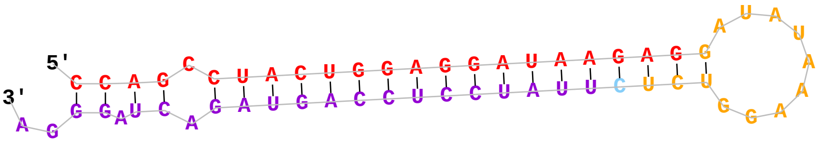

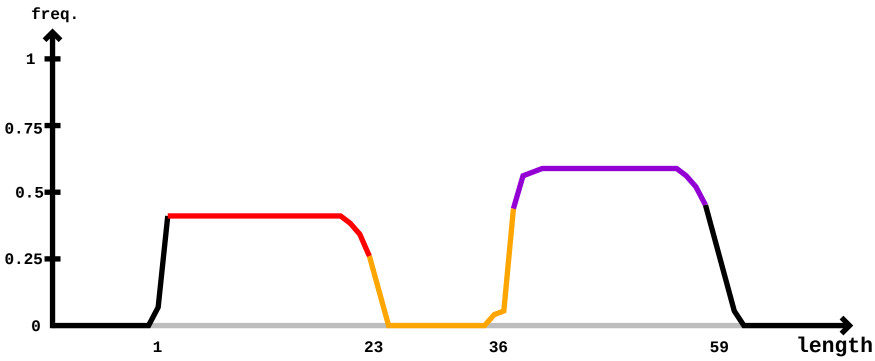

Mature Star

```
5'- cucugguucccca gccuacuggaggauaagaggauauaaaggucucuuauccuccaguagacuagggagccagagcuggua  -3'   obs
```

```
5'- cucugguucccagccuacuggaggauaagaggauauaaaggucucuuauccuccaguagacuagggagccagagcuggua  -3'   obs
    cucugguucccagccuacuggaggauaagaggauauaaaggucucuuauccuccaguagacuagggagccagagcuggua         exp
    ((((((((((.((.(((((((((((((((((((.........))))))))))))))))))).)).))))))))))......    reads   mm   sample
    ..........ccagccuacuggaggauaagag.................................................    3       0    DLD
    ..............................................uuauccuccaguagacuaggga.............    1       0    DLD
    ..............................................uuauccuccaguagacuagggag............    2       0    DLD

    ..........ccagccuacuggaggauaagag.................................................    1       0    B14
    ..............................................uuauccuccaguagacuagg...............    1       0    B14
    ..............................................uuauccuccaguagacuaggga.............    1       0    B14
    ..............................................uuauccuccaguagacuagggag............    1       0    B14

    ..........ccagccuacuggaggauaagag.................................................    1       0    ya1

    ..........ccagccuacuggaggauaagag.................................................    1       0    ku3

    .................................................uccuccaguagacuaggga.............    1       0    NL3

    ............................................ucuuauccuccaguagacuag................    2       0    ya3

    .........cccagccuacuggaggauaa....................................................    1       0    s06

    .........cccagccuacuggaggauaaga..................................................    1       0    HeL

    .........cccagccuacuggaggauaag...................................................    1       0    HEK
    ..........ccagccuacuggaggauaagag.................................................    4       0    HEK
    .............................................cuuauccuccaguagacuagg...............    1       0    HEK
    ..............................................uuauccuccaguagacuagggag............    1       0    HEK
    ...............................................uauccuccaguagacuaggga.............    1       0    HEK

    ..........ccagccuacuggaggauaaga..................................................    1       0    MCF
    ..........ccagccuacuggaggauaagag.................................................    3       0    MCF
    ..............................................uuauccuccaguagacuagg...............    2       0    MCF
    ..............................................uuauccuccaguagacuaggga.............    2       0    MCF

    ...............................................uauccuccaguagacuagggag............    1       0    MBM
```

```
          Mature                              Star
cucugguuccccagccuacuggaggauaagaggauauaaaggucucuuauccuccaguagacuagggagccagagcuggua

..........ccagccuacuggaggauaa....................................................    1    0    NL1
..........ccagccuacuggaggauaagag.................................................    1    0    NL1

..........ccagccuacuggaggauaag...................................................    1    0    NL2
............................................ucuuauccuccaguagacuag................    1    0    NL2

..............................................uuauccuccaguagacuaggga.............    1    0    li2
...............................................uauccuccaguagacuagggagc...........    1    0    li2

.........cccagccuacuggaggauaag...................................................    1    0    li1
.........cccagccuacuggaggauaaga..................................................    1    0    li1
..........ccagccuacuggaggauaagag.................................................    3    0    li1
..............................................uuauccuccaguagacuaggga.............    1    0    li1
...............................................uauccuccaguagacuagggagc...........    2    0    li1

..........ccagccuacuggaggauaaga..................................................    3    0    U20
..........ccagccuacuggaggauaagag.................................................    1    0    U20
..............................................uuauccuccaguagacua.................    2    0    U20
..............................................uuauccuccaguagacuaggga.............    3    0    U20
..............................................uuauccuccaguagacuagggag............    4    0    U20
...............................................uauccuccaguagacuagggag............    2    0    U20
................................................auccuccaguagacuagggagc...........    1    0    U20

..........ccagccuacuggaggauaagagg................................................    1    0    A54

..............................................uuauccuccaguagacuaggga.............    1    0    k12

..............................................uuauccuccaguagacuaggg..............    1    0    ya4
...............................................uauccuccaguagacuagg...............    1    0    ya4

..............................................uuauccuccaguagacuaggg..............    3    0    SW4
...............................................uauccuccaguagacuaggga.............    1    0    SW4

..............................................uuauccuccaguagacuaggg..............    1    0    H52
```

Provisional ID : hsa_can_1220
Score total : 261.6
Score for star read(s) : 3.9
Score for read counts : 264.2
Score for mfe : -5.8
Score for randfold :
Score for cons. seed : -0.6
Total read count : 530
Mature read count : 516
Loop read count : 0
Star read count : 14

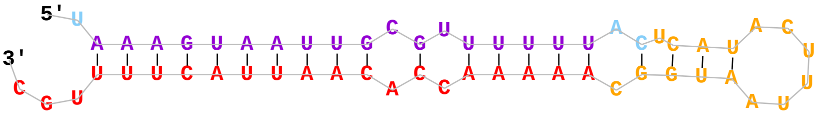

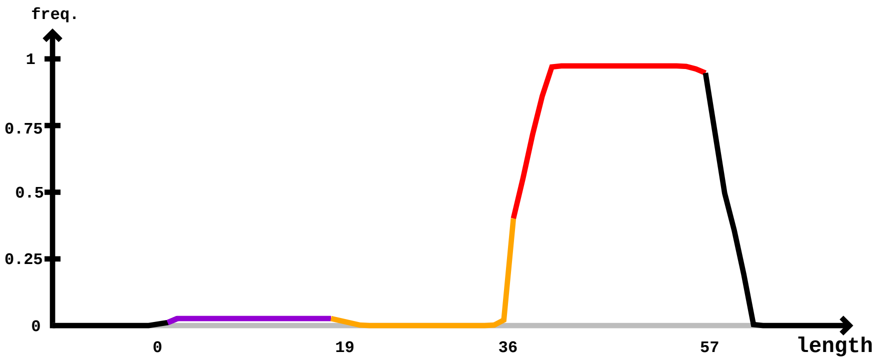

Star Mature

```
5'- agguugacgcuaaaguaauugcguuuuuuacucauacuuuaauggcaaaaaccacaauuacuuuugcaccagucuaauaau   -3'    obs
    agguugacgcuaaaguaauugcguuuuuuacucauacuuuaauggcaaaaaccacaauuacuuuugcaccagucuaauaau          exp
    agguugacgcuaaaguaauugcguuuuuuacucauacuuuaauggcaaaaaccacaauuacuuuugcaccagucuaauaau          known
    ((..((..((.((((((((((.(.(((((.(.(((......)))).))))).).)))))))))).))..))..))......   reads   mm   sample
    ..............................................aaaaaccacaauuacuuuug...............   1       0    ya1
    ..............................................aaaaaccacaauuacuuuugca.............   4       0    ya1
    ...............................................aaaaccacaauuacuuuugca.............   3       0    ya1
    ...............................................aaaaccacaauuacuuuugcac............   1       0    ya1
    ................................................aaaccacaauuacuuuugca.............   1       0    ya1
    .................................................aaccacaauuacuuuugcacc...........   1       0    ya1

    ..............................................aaaaaccacaauuacuuuugca.............   1       0    s04

    ..............................................aaaaaccacaauuacuuuugca.............   3       0    j26
    ..................................................accacaauuacuuuugcacca..........   1       0    j26

    ..............................................aaaaaccacaauuacuuuugc..............   1       0    va1
    ..............................................aaaaaccacaauuacuuuugca.............   6       0    va1
    ..............................................aaaaaccacaauuacuuuugcac............   1       0    va1
    ...............................................aaaaccacaauuacuuuugcac............   5       0    va1
    ................................................aaaccacaauuacuuuugcacc...........   1       0    va1

    ..............................................aaaaaccacaauuacuuuugca.............   1       0    ku5
    ................................................aaaccacaauuacuuuugcacc...........   1       0    ku5

    ................................................aaaccacaauuacuuuugcacc...........   1       0    k05

    ..............................................aaaaaccacaauuacuuuugca.............   1       0    s06
    .................................................aaccacaauuacuuuugcacca..........   1       0    s06
    ..................................................accacaauuacuuuugcacca..........   1       0    s06

    .........cuaaaguaauugcguuuuuua...................................................   1       0    HeL
    ..............................................aaaaaccacaauuacuuuugc..............   1       0    HeL
    ..............................................aaaaaccacaauuacuuuugca.............   3       0    HeL
    ...............................................aaaaccacaauuacuuuugcac............   2       0    HeL
    ................................................aaaccacaauuacuuuugcac............   1       0    HeL
```

Star Mature

agguugacgcuaaaguaauugcguuuuuuacucauacuuuaauggcaaaaaccacaauuacuuuugcaccagucuaauaau			
....aaaccacaauuacuuuugcacc...........	3	0	HeL
....aaccacaauuacuuuugcacca..........	3	0	HeL
....accacaauuacuuuugcacca..........	5	0	HeL
....aaaccacaauuacuuuugcacc...........	1	0	s07
....aaaaaccacaauuacuuuugca.............	4	0	s10
....aaaccacaauuacuuuugcac............	1	0	s10
....aaaaaccacaauuacuuuugca.............	4	0	ta3
....aaaaccacaauuacuuuugc..............	1	0	ta3
....aaaaccacaauuacuuuugcac............	1	0	ta3
....aaaccacaauuacuuuugcac............	1	0	ta3
....aaaccacaauuacuuuugcacc...........	1	0	ta3
....aaccacaauuacuuuugcacca..........	2	0	ta3
....aaaaaccacaauuacuuuugca.............	5	0	j10
....aaaccacaauuacuuuugcac............	1	0	j10
....aaccacaauuacuuuugcacca..........	1	0	j10
....aaccacaauuacuuuugcac............	1	0	k14
....aaaaaccacaauuacuuuugcac............	1	0	va2
..........uaaaguaauugcguuuuuu..	1	0	j02
...........aaaguaauugcguuuuuu..	1	0	j02
....aaaccacaauuacuuuugca.............	1	0	j27
....aaaaaccacaauuacuuuugca.............	1	0	j31
....aaaaaccacaauuacuuuugca.............	2	0	s03
....aaaaccacaauuacuuuugca.............	1	0	s03
....aaaaccacaauuacuuuugcac............	2	0	s03
....aaaaaccacaauuacuuuug...............	2	0	H52
....aaaaaccacaauuacuuuugca.............	1	0	H52
....aaaaccacaauuacuuuugc..............	1	0	H52
....aaaaccacaauuacuuuugca.............	2	0	H52
....aaaccacaauuacuuuugcacc...........	2	0	H52
....aaccacaauuacuuuugcacc...........	1	0	H52
....aaccacaauuacuuuugcacca..........	1	0	H52
....accacaauuacuuuugcacca..........	1	0	H52
....ccacaauuacuuuugcaccag.........	1	0	H52
....aaaaaccacaauuacuuuugca.............	6	0	j22
....aaaaccacaauuacuuuugca.............	1	0	j22
....aaccacaauuacuuuugcacc...........	1	0	j22
....aaaaccacaauuacuuuugcac............	1	0	k09
....aaaaaccacaauuacuuuugc..............	3	0	B14
....aaaaaccacaauuacuuuugca.............	6	0	B14
....aaaaccacaauuacuuuugcac............	1	0	B14
....aaaccacaauuacuuuugca.............	2	0	B14
....aaaccacaauuacuuuugcacc...........	2	0	B14
....accacaauuacuuuugcacca..........	2	0	B14
....aaaaaccacaauuacuuuugcac............	1	0	ku3
....aaaccacaauuacuuuugcacca..........	1	0	ku3
....aaaaaccacaauuacuuuugca.............	1	0	j09
....accacaauuacuuuugcacca..........	1	0	j09
....aaaaaccacaauuacuuuugca.............	4	0	j16
....aaaaaccacaauuacuuuugca.............	2	0	ku6
....aaaaaccacaauuacuuuugca.............	2	0	j11
.........cuaaaguaauugcguuuuuuac..	1	0	ya3

Star **Mature**

agguugacgcuaaaguaauugcguuuuuuacucauacuuuaauggcaaaaaccacaauuacuuuugcaccagucuaauaau

...........aaaguaauugcguuuuuu..	2	0	ya3
....aaaaaccacaauuacuuuugca.............	3	0	ya3
....aaaaccacaauuacuuuugca.............	2	0	ya3
....aaaaccacaauuacuuuugcac............	5	0	ya3
....aaaccacaauuacuuuugca.............	1	0	ya3
....aaaccacaauuacuuuugcacc...........	1	0	ya3
....aaccacaauuacuuuugcacc...........	2	0	ya3
....aaaaaccacaauuacuuuugca.............	2	0	la1
....aaccacaauuacuuuugcacc...........	1	0	la1
....caaaaaccacaauuacuuuugc..............	3	0	HEK
....aaaaaccacaauuacuuuugc..............	1	0	HEK
....aaaaaccacaauuacuuuugca.............	8	0	HEK
....aaaaccacaauuacuuuugca.............	2	0	HEK
....aaaaccacaauuacuuuugcac............	3	0	HEK
....aaaccacaauuacuuuugca.............	1	0	HEK
....aaaccacaauuacuuuugcac............	3	0	HEK
....aaaccacaauuacuuuugcacc...........	3	0	HEK
....aaccacaauuacuuuugcacc...........	2	0	HEK
....aaccacaauuacuuuugcacca..........	4	0	HEK
....accacaauuacuuuugcacca..........	6	0	HEK
....aaaaccacaauuacuuuugca.............	1	0	j17
....aaccacaauuacuuuugcacca..........	2	0	j17
....accacaauuacuuuugcacca..........	1	0	j17
....aaaaccacaauuacuuuugca.............	1	0	j15
....ccacaauuacuuuugcaccag.........	1	0	j15
....aaaaccacaauuacuuuugcac............	1	0	la2
....aaaccacaauuacuuuugca.............	1	0	la2
....aaaccacaauuacuuuugcacc...........	1	0	la2
....aaccacaauuacuuuugcacca..........	1	0	la2
....accacaauuacuuuugcacca..........	1	0	j12
....aaaaaccacaauuacuuuugca.............	3	0	va4
....aaaaaccacaauuacuuuugcac............	1	0	va4
....aaaaccacaauuacuuuugcac............	1	0	va4
....aaccacaauuacuuuugcacc...........	2	0	va4
....accacaauuacuuuugcacca..........	4	0	va4
....aaaaaccacaauuacuuuugca.............	12	0	va3
....aaaaccacaauuacuuuugcac............	2	0	va3
....aaaccacaauuacuuuugcac............	1	0	va3
....aaaccacaauuacuuuugcacc...........	4	0	va3
....aaccacaauuacuuuugcacc...........	2	0	va3
....aaccacaauuacuuuugcacca..........	2	0	va3
....accacaauuacuuuugcacca..........	1	0	va3
....aaaaaccacaauuacuuuugca.............	1	0	SW4
....aaaaccacaauuacuuuugcac............	1	0	SW4
....aaaccacaauuacuuuugcac............	2	0	SW4
....aaaccacaauuacuuuugcacc...........	3	0	SW4
....aaccacaauuacuuuugcacca..........	3	0	SW4
....accacaauuacuuuugcacc...........	1	0	SW4
....accacaauuacuuuugcacca..........	3	0	SW4
...........aaaguaauugcguuuuuu..	3	0	ya4
....aaaaaccacaauuacuuuugca.............	4	0	ya4
....aaaaccacaauuacuuuugca.............	2	0	ya4
....aaaaccacaauuacuuuugcac............	4	0	ya4
....aaaccacaauuacuuuugcac............	1	0	ya4
....aaaccacaauuacuuuugcacc...........	4	0	ya4
....aaccacaauuacuuuugca.............	3	0	ya4
....aaccacaauuacuuuugcacc...........	4	0	ya4
....aaccacaauuacuuuugcacca..........	2	0	ya4
....aaaaaccacaauuacuuuugca.............	4	0	ku1

```
          Star                Mature
agguugacgcuaaaguaauugcguuuuuuacucauacuuuaauggcaaaaaccacaauuacuuuugcaccagucuaauaau
.................................................aaccacaauuacuuuugcacc...........    1    0    ku1

..........uaaaguaauugcguuuuuu....................................................    1    0    j23
..............................................aaaaaccacaauuacuuuugc..............    1    0    j23
..............................................aaaaaccacaauuacuuuugca.............    2    0    j23
...............................................aaaaccacaauuacuuuugca.............    1    0    j23
.................................................aaccacaauuacuuuugcacca..........    1    0    j23
..................................................accacaauuacuuuugcacca..........    1    0    j23

..............................................aaaaaccacaauuacuuuugca.............    2    0    s05

..............................................aaaaaccacaauuacuuu.................    1    0    ta2
...............................................aaaaccacaauuacuuuugcac............    1    0    ta2

..............................................aaaaaccacaauuacuuuugca.............    1    0    k11

..............................................aaaaaccacaauuacuuuugca.............    1    0    ta1

.............................................caaaaaccacaauuacuuuugc..............    1    0    s09
...............................................aaaaccacaauuacuuuugca.............    1    0    s09
.................................................aaccacaauuacuuuugcacca..........    1    0    s09
..................................................accacaauuacuuuugcacca..........    1    0    s09

.............................................caaaaaccacaauuacuuu.................    1    0    NL2
..............................................aaaaaccacaauuacuuuugca.............    3    0    NL2
.................................................aaccacaauuacuuuugcacc...........    1    0    NL2
.................................................aaccacaauuacuuuugcacca..........    5    0    NL2

..............................................aaaaaccacaauuacuuuugca.............    4    0    j13
...............................................aaaaccacaauuacuuuugca.............    2    0    j13
.................................................aaccacaauuacuuuugcacca..........    2    0    j13

..........uaaaguaauugcguuuuu.....................................................    1    0    j19
..............................................aaaaaccacaauuacuuuugca.............    2    0    j19
................................................aaaccacaauuacuuuugca.............    2    0    j19
.................................................aaccacaauuacuuuugcacca..........    1    0    j19
..................................................accacaauuacuuuugcacca..........    2    0    j19

..............................................aaaaaccacaauuacuuuugca.............    1    0    j25

..............................................aaaaaccacaauuacuuuugca.............    2    0    s01
...............................................aaaaccacaauuacuuuugcac............    2    0    s01
................................................aaaccacaauuacuuuugcacc...........    2    0    s01

...............................................aaaaccacaauuacuuuugca.............    1    0    k04

.............................................caaaaaccacaauuacuuuugc..............    1    0    U20
..............................................aaaaaccacaauuacuuuugc..............    22   0    U20
..............................................aaaaaccacaauuacuuuugca.............    17   0    U20
...............................................aaaaccacaauuacuuuugca.............    1    0    U20
...............................................aaaaccacaauuacuuuugcac............    8    0    U20
................................................aaaccacaauuacuuuugcac............    6    0    U20
................................................aaaccacaauuacuuuugcacc...........    14   0    U20
.................................................aaccacaauuacuuuugcacc...........    6    0    U20
.................................................aaccacaauuacuuuugcacca..........    1    0    U20
..................................................accacaauuacuuuugcacca..........    15   0    U20

..............................................aaaaaccacaauuacuuuugca.............    3    0    j21
...............................................aaaaccacaauuacuuuugca.............    1    0    j21
................................................aaaccacaauuacuuuugcacc...........    1    0    j21


.................................................aaccacaauuacuuuugcacc...........    1    0    s11
.................................................aaccacaauuacuuuugcacca..........    1    0    s11

..............................................aaaaaccacaauuacuuuugca.............    1    0    j07

................................................aaaccacaauuacuuuugcacc...........    1    0    j14

..............................................aaaaaccacaauuacuuuugca.............    1    0    s08
```

```
                 Star                             Mature
agguugacgcuaaaguaauugcguuuuuuacucauacuuuaauggcaaaaaccacaauuacuuuugcaccagucuaauaau
..................................................accacaauuacuuuugcac............      1      0      s08

..............................................aaaaaccacaauuacuuuugc..............      1      0      DLD
...............................................aaaaccacaauuacuuuugcac............      1      0      DLD
................................................aaaccacaauuacuuuugcacc...........      3      0      DLD
..................................................accacaauuacuuuugcacc...........      1      0      DLD
..................................................accacaauuacuuuugcacca..........      4      0      DLD

..............................................aaaaaccacaauuacuuuugca.............      1      0      k03

.............................................caaaaaccacaauuacuuuu................      1      0      NL3
.............................................caaaaaccacaauuacuuuug...............      2      0      NL3
..............................................aaaaaccacaauuacuuuugca.............      7      0      NL3
...............................................aaaaccacaauuacuuuugcac............      1      0      NL3
.................................................aaccacaauuacuuuugcacca..........      1      0      NL3

...........aaaguaauugcguuuuuu....................................................      2      0      ya2
..............................................aaaaaccacaauuacuuuu................      2      0      ya2
..............................................aaaaaccacaauuacuuuugc..............      1      0      ya2
..............................................aaaaaccacaauuacuuuugca.............      2      0      ya2
...............................................aaaaccacaauuacuuuugca.............      6      0      ya2
................................................aaaccacaauuacuuuugcac............      3      0      ya2
................................................aaaccacaauuacuuuugcacca..........      1      0      ya2
.................................................aaccacaauuacuuuugcacca..........      2      0      ya2

................................................aaaccacaauuacuuuugcacc...........      1      0      k06

..............................................aaaaaccacaauuacuuuugca.............      1      0      j29
...............................................aaaaccacaauuacuuuugca.............      1      0      j29

.............................................caaaaaccacaauuacuuuug...............      1      0      sh2

.........cuaaaguaauugcguuuuu.....................................................      1      0      MCF
..............................................aaaaaccacaauuacuuuu................      1      0      MCF
..............................................aaaaaccacaauuacuuuugc..............      1      0      MCF
..............................................aaaaaccacaauuacuuuugca.............      2      0      MCF
...............................................aaaaccacaauuacuuuugcac............      1      0      MCF

..............................................aaaaaccacaauuacuuuug...............      1      0      MBM
..............................................aaaaaccacaauuacuuuugc..............      1      0      MBM
..............................................aaaaaccacaauuacuuuugca.............      2      0      MBM
...............................................aaaaccacaauuacuuuugca.............      1      0      MBM
...............................................aaaaccacaauuacuuuugcac............      3      0      MBM
................................................aaaccacaauuacuuuugcacc...........      4      0      MBM
.................................................aaccacaauuacuuuugcacc...........      1      0      MBM
..................................................accacaauuacuuuugcacca..........      4      0      MBM

............................................gcaaaaaccacaauuacuuuug...............      1      0      li1
..............................................aaaaaccacaauuacuuuugc..............      1      0      li1
...............................................aaaaccacaauuacuuuugcac............      1      0      li1
................................................aaaccacaauuacuuuugcacc...........      2      0      li1
.................................................aaccacaauuacuuuugcacca..........      1      0      li1

..............................................aaaaaccacaauuacuuuugc..............      7      0      A54
.................................................aaccacaauuacuuuugcacc...........      4      0      A54
.................................................aaccacaauuacuuuugcacca..........      4      0      A54

..................................................accacaauuacuuuugcacca..........      1      0      k12
```

Provisional ID	:	hsa_can_1241
Score total	:	2.9
Score for star read(s)	:	-1.3
Score for read counts	:	0
Score for mfe	:	1.2
Score for randfold	:	
Score for cons. seed	:	3
Total read count	:	1091
Mature read count	:	1091
Loop read count	:	0
Star read count	:	0

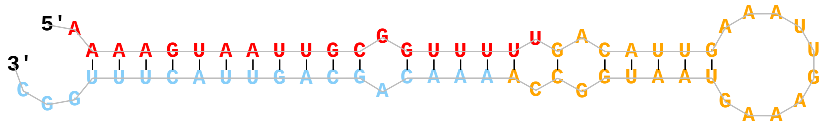

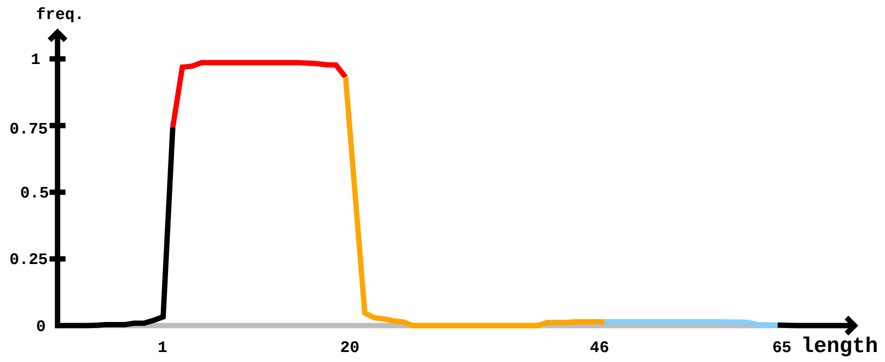

Mature **Star**

```
5'- agguuggugcaaaaguaauugcgguuuuugacauugaaauugaaaguaauggccaaaacagcaguuacuuuggcaucaacc  -3'    exp
    .(((((((((.(((((((((((.(((((.(.(((((..........))))).).))))).))))))))))).)))))))))    reads      mm         sample
    ........gcaaaaguaauugcgguuuuug...................................................    1          0          ya1
    ..........aaaaguaauugcgguuuu.....................................................    6          0          ya1
    ..........aaaaguaauugcgguuuuu....................................................    44         0          ya1
    ..........aaaaguaauugcgguuuuugaca................................................    1          0          ya1
    ...........aaaguaauugcgguuuuu....................................................    7          0          ya1
    ...........aaaguaauugcgguuuuug...................................................    3          0          ya1
    ............aaguaauugcgguuuuugacau...............................................    1          0          ya1
    .............aguaauugcgguuuuugacauu..............................................    1          0          ya1
    .................................................uggccaaaacagcaguuacuuu..........    1          0          ya1

    ..........aaaaguaauugcgguuuuug...................................................    4          0          j26
    ...........aaaguaauugcgguuuuug...................................................    1          0          j26

    ..........aaaaguaauugcgguuuuug...................................................    1          0          va1
    ...........aaaguaauugcgguuuuuga..................................................    1          0          va1

    ..........aaaaguaauugcgguuuuug...................................................    7          0          ku5

    ...uuggugcaaaaguaauugcggu........................................................    1          0          k05
    ..........aaaaguaauugcgguuuuu....................................................    2          0          k05
    ..........aaaaguaauugcgguuuuug...................................................    1          0          k05
    .............aguaauugcgguuuuugacauu..............................................    1          0          k05

    .........caaaaguaauugcgguuuuug...................................................    1          0          s06
    ..........aaaaguaauugcgguuuuug...................................................    2          0          s06

    ...........aaaguaauugcgguuuuugac.................................................    1          0          HeL

    ..........aaaaguaauugcgguuuuu....................................................    2          0          ku4
    ..........aaaaguaauugcgguuuuug...................................................    5          0          ku4
    ...........aaaguaauugcgguuuuug...................................................    5          0          ku4

    ..........aaaaguaauugcgguuuuug...................................................    1          0          s10
    ...........aaaguaauugcgguuuuug...................................................    2          0          s10
```

```
          Mature                                            Star
agguuggugcaaaaguaauugcgguuuuugacauugaaauugaaaguaauggccaaaacagcaguuacuuuggcaucaacc

.........caaaaguaauugcgguuuuug...................................................  1   0   ta3
..........aaaaguaauugcgguuuu.....................................................  1   0   ta3
..........aaaaguaauugcgguuuuu....................................................  1   0   ta3
..........aaaaguaauugcgguuuuug...................................................  11  0   ta3
...........aaaguaauugcgguuuuu....................................................  2   0   ta3
...........aaaguaauugcgguuuuugaca................................................  1   0   ta3

..........aaaaguaauugcgguuuu.....................................................  16  0   j06
..........aaaaguaauugcgguuuuu....................................................  4   0   j06
..........aaaaguaauugcgguuuuug...................................................  3   0   j06
...........aaaguaauugcgguuuuug...................................................  13  0   j06

..........aaaaguaauugcgguuuuug...................................................  3   0   j10

..........aaaaguaauugcgguuuuu....................................................  1   0   j04
..........aaaaguaauugcgguuuuug...................................................  6   0   j04

...........aaaguaauugcgguuuuuga..................................................  1   0   k14

..........aaaaguaauugcgguuuu.....................................................  1   0   va2
..........aaaaguaauugcgguuuuu....................................................  5   0   va2
.............aguaauugcgguuuuugacauu..............................................  2   0   va2

.........caaaaguaauugcgguuuuu....................................................  1   0   j02
..........aaaaguaauugcgguuuuu....................................................  5   0   j02
...........aaaguaauugcgguuuuug...................................................  6   0   j02

..........aaaaguaauugcgguuuuug...................................................  1   0   j31

.........caaaaguaauugcgguuuu.....................................................  1   0   j27
..........aaaaguaauugcgguuuuu....................................................  11  0   j27
..........aaaaguaauugcgguuuuug...................................................  14  0   j27
...........aaaguaauugcgguuuuug...................................................  11  0   j27
....................................................ccaaaacagcaguuacuuugg........  1   0   j27

.................................................uggccaaaacagcaguuacuuu..........  1   0   H52

...........aaaguaauugcgguuuuug...................................................  1   0   s03

..........aaaaguaauugcgguuuuu....................................................  1   0   j22
..........aaaaguaauugcgguuuuug...................................................  4   0   j22
...........aaaguaauugcgguuuuu....................................................  1   0   j22
...........aaaguaauugcgguuuuug...................................................  5   0   j22

..........aaaaguaauugcgguuuuu....................................................  2   0   k09

..........aaaaguaauugcgguuuu.....................................................  1   0   B14
..........aaaaguaauugcgguuuuug...................................................  3   0   B14
...........aaaguaauugcgguuuuug...................................................  3   0   B14
.................................................uggccaaaacagcaguuacuuu..........  1   0   B14

..........aaaaguaauugcgguuuuu....................................................  3   0   j16
..........aaaaguaauugcgguuuuug...................................................  4   0   j16
...........aaaguaauugcgguuuuug...................................................  7   0   j16

..........aaaaguaauugcgguuuuu....................................................  1   0   j18
..........aaaaguaauugcgguuuuug...................................................  1   0   j18
..........aaaaguaauugcgguuuuuga..................................................  1   0   j18
...........aaaguaauugcgguuuuug...................................................  1   0   j18

..........aaaaguaauugcgguuuuu....................................................  8   0   j09
..........aaaaguaauugcgguuuuug...................................................  9   0   j09
..........aaaaguaauugcgguuuuuga..................................................  1   0   j09

..........aaaaguaauugcgguuuuu....................................................  3   0   ku3
..........aaaaguaauugcgguuuuug...................................................  11  0   ku3
..........aaaaguaauugcgguuuuuga..................................................  2   0   ku3
...........aaaguaauugcgguuuuug...................................................  1   0   ku3

.........caaaaguaauugcgguuuuug...................................................  1   0   ku6
```

Mature **Star**

```
agguuggugcaaaaguaauugcgguuuuugacauugaaauugaaaguaauggccaaaacagcaguuacuuuggcaucaacc
..........aaaaguaauugcgguuuuu....................................................   7    0   ku6
..........aaaaguaauugcgguuuuug...................................................   9    0   ku6
...........aaaguaauugcgguuuuug...................................................   2    0   ku6

..........aaaaguaauugcgguuuuu....................................................   1    0   j11
..........aaaaguaauugcgguuuuug...................................................   2    0   j11

........gcaaaaguaauugcgguuuu.....................................................   3    0   ya3
..........aaaaguaauugcgguuuu.....................................................   9    0   ya3
..........aaaaguaauugcgguuuuu....................................................   64   0   ya3
..........aaaaguaauugcgguuuuug...................................................   21   0   ya3
...........aaaguaauugcgguuuuu....................................................   26   0   ya3
...........aaaguaauugcgguuuuug...................................................   3    0   ya3
.............aguaauugcgguuuuugacauu..............................................   1    0   ya3

..........aaaaguaauugcgguuuuug...................................................   2    0   la1

......gugcaaaaguaauugcgguuu......................................................   1    0   HEK
........gcaaaaguaauugcgguuuuug...................................................   1    0   HEK
..........aaaaguaauugcgguuuuuga..................................................   1    0   HEK
.............aguaauugcgguuuuugacauu..............................................   1    0   HEK
.................................................uggccaaaacagcaguuac.............   1    0   HEK
.................................................uggccaaaacagcaguuacuuu..........   3    0   HEK

..........aaaaguaauugcgguuuuu....................................................   5    0   j15
..........aaaaguaauugcgguuuuug...................................................   10   0   j15
...........aaaguaauugcgguuuuug...................................................   7    0   j15

........gcaaaaguaauugcgguuuuu....................................................   1    0   j17
..........aaaaguaauugcgguuuuu....................................................   9    0   j17
..........aaaaguaauugcgguuuuug...................................................   17   0   j17
..........aaaaguaauugcgguuuuuga..................................................   1    0   j17
...........aaaguaauugcgguuuuu....................................................   1    0   j17
...........aaaguaauugcgguuuuug...................................................   9    0   j17

..........aaaaguaauugcgguuuu.....................................................   1    0   j12
..........aaaaguaauugcgguuuuu....................................................   2    0   j12
..........aaaaguaauugcgguuuuug...................................................   7    0   j12
...........aaaguaauugcgguuuuug...................................................   5    0   j12
.............aguaauugcgguuuuugacauu..............................................   1    0   j12

..........aaaaguaauugcgguuuuug...................................................   2    0   va3

..........aaaaguaauugcgguuuuug...................................................   3    0   SW4
...........aaaguaauugcgguuuuu....................................................   2    0   SW4

.........caaaaguaauugcgguuuuu....................................................   2    0   ya4
..........aaaaguaauugcgguuuu.....................................................   2    0   ya4
..........aaaaguaauugcgguuuuu....................................................   49   0   ya4
..........aaaaguaauugcgguuuuug...................................................   1    0   ya4
...........aaaguaauugcgguuuuu....................................................   15   0   ya4
.............aguaauugcgguuuuugacauu..............................................   4    0   ya4

..........aaaaguaauugcgguuuuu....................................................   3    0   ku1
..........aaaaguaauugcgguuuuug...................................................   14   0   ku1
...........aaaguaauugcgguuuuu....................................................   2    0   ku1
...........aaaguaauugcgguuuuug...................................................   2    0   ku1

.........caaaaguaauugcgguuuuug...................................................   2    0   j23
..........aaaaguaauugcgguuuu.....................................................   3    0   j23
..........aaaaguaauugcgguuuuu....................................................   3    0   j23
..........aaaaguaauugcgguuuuug...................................................   23   0   j23
..........aaaaguaauugcgguuuuuga..................................................   1    0   j23
...........aaaguaauugcgguuuuu....................................................   2    0   j23
...........aaaguaauugcgguuuuug...................................................   14   0   j23
...........aaaguaauugcgguuuuuga..................................................   1    0   j23
...........aaaguaauugcgguuuuugac.................................................   1    0   j23

..........aaaaguaauugcgguuuuu....................................................   2    0   s05
..........aaaaguaauugcgguuuuug...................................................   2    0   s05
```

Mature **Star**

```
agguuggugcaaaaguaauugcgguuuuugacauugaaauugaaaguaauggccaaaacagcaguuacuuuggcaucaacc
...........aaaguaauugcgguuuuug...................................................  1   0   s05

..........aaaaguaauugcgguuuuu....................................................  1   0   ta2
..........aaaaguaauugcgguuuuug...................................................  1   0   ta2
..........aaaaguaauugcgguuuuugaca................................................  4   0   ta2
...........aaaguaauugcgguuuuugacau...............................................  1   0   ta2
............aaguaauugcgguuuuugacau...............................................  3   0   ta2

..........aaaaguaauugcgguuuuu....................................................  1   0   j24
..........aaaaguaauugcgguuuuug...................................................  21  0   j24
..........aaaaguaauugcgguuuuuga..................................................  1   0   j24
...........aaaguaauugcgguuuuug...................................................  12  0   j24

...uuggugcaaaaguaauugcggu........................................................  1   0   k11
..........aaaaguaauugcgguuuuug...................................................  2   0   k11
...........aaaguaauugcgguuuuug...................................................  1   0   k11

..........aaaaguaauugcgguuuuug...................................................  4   0   ta1
..........aaaaguaauugcgguuuuuga..................................................  1   0   ta1
..........aaaaguaauugcgguuuuugaca................................................  1   0   ta1
...........aaaguaauugcgguuuuug...................................................  2   0   ta1

..........aaaaguaauugcgguuuuu....................................................  1   0   s09
..........aaaaguaauugcgguuuuug...................................................  5   0   s09
...........aaaguaauugcgguuuuu....................................................  1   0   s09
...........aaaguaauugcgguuuuug...................................................  2   0   s09

..........aaaaguaauugcgguuuu.....................................................  1   0   NL2
..........aaaaguaauugcgguuuuu....................................................  3   0   NL2
..........aaaaguaauugcgguuuuug...................................................  6   0   NL2
...........aaaguaauugcgguuuuu....................................................  1   0   NL2
...........aaaguaauugcgguuuuug...................................................  1   0   NL2

...........aaaguaauugcgguuuuuga..................................................  1   0   k10

..........aaaaguaauugcgguuuuu....................................................  1   0   j13
..........aaaaguaauugcgguuuuug...................................................  10  0   j13
...........aaaguaauugcgguuuuu....................................................  1   0   j13
...........aaaguaauugcgguuuuug...................................................  5   0   j13

..........aaaaguaauugcgguuuuu....................................................  6   0   j19
..........aaaaguaauugcgguuuuug...................................................  21  0   j19
..........aaaaguaauugcgguuuuuga..................................................  1   0   j19
...........aaaguaauugcgguuuuu....................................................  1   0   j19
...........aaaguaauugcgguuuuug...................................................  3   0   j19

..........aaaaguaauugcgguuuuu....................................................  3   0   j03
..........aaaaguaauugcgguuuuug...................................................  4   0   j03

..........aaaaguaauugcgguuuuug...................................................  1   0   k01
....................................................ccaaaacagcaguuacuuuggca......  2   0   k01

..........aaaaguaauugcgguuuuu....................................................  5   0   j25
..........aaaaguaauugcgguuuuug...................................................  3   0   j25
..........aaaaguaauugcgguuuuuga..................................................  2   0   j25
...........aaaguaauugcgguuuuug...................................................  1   0   j25

..........aaaaguaauugcgguuuuu....................................................  2   0   per
..........aaaaguaauugcgguuuuug...................................................  8   0   per
...........aaaguaauugcgguuuuu....................................................  1   0   per
...........aaaguaauugcgguuuuug...................................................  4   0   per

..........aaaaguaauugcgguuuuug...................................................  3   0   k04
...........aaaguaauugcgguuuuug...................................................  1   0   k04

.........caaaaguaauugcgguuuuug...................................................  2   0   U20
..........aaaaguaauugcgguuuuug...................................................  7   0   U20
..........aaaaguaauugcgguuuuugac.................................................  1   0   U20

..........aaaaguaauugcgguuuuu....................................................  1   0   ku2
```

```
          Mature                        Star
agguuggugcaaaaguaauugcgguuuuugacauugaaauugaaaguaauggccaaaacagcaguuacuuuggcaucaacc
..........aaaaguaauugcgguuuuug...................................................    7     0     ku2
...........aaaguaauugcgguuuuug...................................................    5     0     ku2

..........aaaaguaauugcgguuuuug...................................................    4     0     j21
...........aaaguaauugcgguuuuug...................................................    2     0     j21

..........aaaaguaauugcgguuuuu....................................................    2     0     j07
..........aaaaguaauugcgguuuuug...................................................    7     0     j07

..........aaaaguaauugcgguuuuugaca................................................    1     0     j14

.........caaaaguaauugcgguuuuug...................................................    1     0     k02
.............aguaauugcgguuuuugacauu..............................................    1     0     k02

.........caaaaguaauugcgguuuuug...................................................    1     0     j28
..........aaaaguaauugcgguuuu.....................................................    3     0     j28
..........aaaaguaauugcgguuuuug...................................................    37    0     j28
...........aaaguaauugcgguuuuug...................................................    4     0     j28

........gcaaaaguaauugcgguuuuug...................................................    1     0     s08

..........aaaaguaauugcgguuuuu....................................................    3     0     DLD

........gcaaaaguaauugcgguuuuu....................................................    1     0     k03

.........caaaaguaauugcgguuuu.....................................................    1     0     ya2
..........aaaaguaauugcgguuuu.....................................................    1     0     ya2
..........aaaaguaauugcgguuuuu....................................................    40    0     ya2
..........aaaaguaauugcgguuuuug...................................................    3     0     ya2
...........aaaguaauugcgguuuuu....................................................    12    0     ya2
...........aaaguaauugcgguuuuug...................................................    1     0     ya2
.............aguaauugcgguuuuugacauu..............................................    1     0     ya2

..........aaaaguaauugcgguuuuu....................................................    2     0     NL3
..........aaaaguaauugcgguuuuug...................................................    8     0     NL3
...........aaaguaauugcgguuuuu....................................................    3     0     NL3
...........aaaguaauugcgguuuuug...................................................    3     0     NL3

..guuggugcaaaaguaauugcgg.........................................................    1     0     sh2
...uuggugcaaaaguaauugcgg.........................................................    1     0     sh2
..........aaaaguaauugcgguuuu.....................................................    1     0     sh2
..........aaaaguaauugcgguuuuugac.................................................    1     0     sh2

..........aaaaguaauugcgguuuuu....................................................    1     0     j29
..........aaaaguaauugcgguuuuug...................................................    4     0     j29

..........aaaaguaauugcgguuuuug...................................................    11    0     MCF
..........aaaaguaauugcgguuuuuga..................................................    2     0     MCF
...........aaaguaauugcgguuuuu....................................................    1     0     MCF
...........aaaguaauugcgguuuuug...................................................    1     0     MCF
.............aguaauugcgguuuuugacauu..............................................    1     0     MCF
.................................................uggccaaaacagcaguuacuuu..........    1     0     MCF

..........aaaaguaauugcgguuuuu....................................................    4     0     j01
..........aaaaguaauugcgguuuuug...................................................    9     0     j01

......gugcaaaaguaauugcgguu.......................................................    1     0     MBM
..........aaaaguaauugcgguuuuug...................................................    2     0     MBM
...........aaaguaauugcgguuuuug...................................................    3     0     MBM
.................................................uggccaaaacagcaguuacuuu..........    1     0     MBM

..........aaaaguaauugcgguuuuug...................................................    2     0     NL1
..........aaaaguaauugcgguuuuuga..................................................    1     0     NL1

..........aaaaguaauugcgguuuuug...................................................    1     0     k07

..........aaaaguaauugcgguuuuug...................................................    3     0     sh1

........gcaaaaguaauugcgguuuuug...................................................    1     0     li1
..........aaaaguaauugcgguuuuugac.................................................    1     0     li1
```

Mature Star

```
agguuggugcaaaaguaauugcgguuuuugacauugaaauugaaaguaauggccaaaacagcaguuacuuuggcaucaacc
.................................................uggccaaaacagcaguuacuu...........    2    0    li1
.................................................uggccaaaacagcaguuacuuu..........    2    0    li1

.........caaaaguaauugcgguuuuu....................................................    1    0    k13
..........aaaaguaauugcgguuuuu....................................................    1    0    k13

..........aaaaguaauugcgguuuuu....................................................    4    0    j20
..........aaaaguaauugcgguuuuug...................................................    6    0    j20
...........aaaguaauugcgguuuuug...................................................    3    0    j20

......gugcaaaaguaauugcgguu.......................................................    4    0    A54
........gcaaaaguaauugcgguuuuu....................................................    3    0    A54
..........aaaaguaauugcgguuuuug...................................................    4    0    A54
...........aaaguaauugcgguuuuug...................................................    10   0    A54

..........aaaaguaauugcgguuuu.....................................................    1    0    k12
..........aaaaguaauugcgguuuuu....................................................    1    0    k12
..........aaaaguaauugcgguuuuug...................................................    4    0    k12
...........aaaguaauugcgguuuuug...................................................    2    0    k12
...........aaaguaauugcgguuuuuga..................................................    1    0    k12
.............aguaauugcgguuuuugacauu..............................................    1    0    k12
```

Provisional ID : hsa_can_1250
Score total : -3.3
Score for star read(s) : -1.3
Score for read counts : -3.3
Score for mfe : 1.9
Score for randfold :
Score for cons. seed : -0.6
Total read count : 5
Mature read count : 5
Loop read count : 0
Star read count : 0

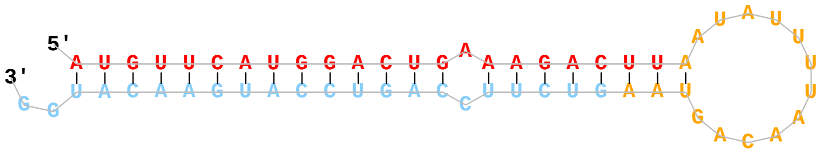

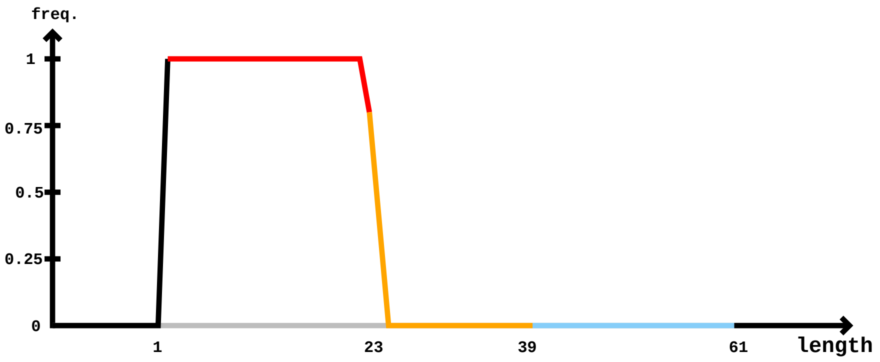

Mature Star

```
5'- gggacauuccauguucauggacugaaagacuuaauauuuuaacaguaagucuuccaguccaugaacauggaauaucuuuuc   -3'    exp
    ((((.(((((((((((((((((((.((((((((............)))))))).))))))))))))))))))).))))...     reads      mm         sample
    ..........auguucauggacugaaagacuu.................................................     3          0          li1

    ..........auguucauggacugaaagacuu.................................................     1          0          HeL

    ..........auguucauggacugaaagacu..................................................     1          0          ya4
```

Provisional ID : hsa_can_1252
Score total : 1.5
Score for star read(s) : -1.3
Score for read counts : 0
Score for mfe : -0.1
Score for randfold :
Score for cons. seed : 3
Total read count : 605
Mature read count : 605
Loop read count : 0
Star read count : 0

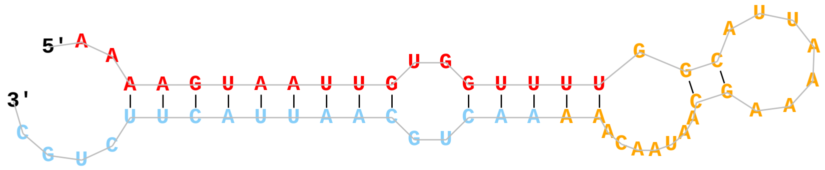

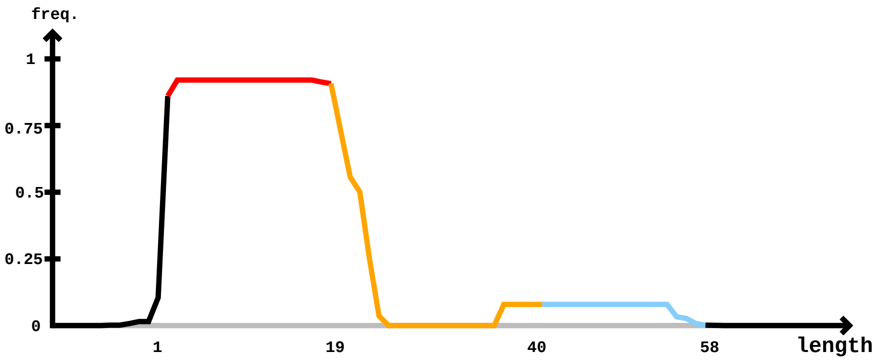

Mature Star

```
5'- agguuugugcaaaaguaauugugguuuuggcauuaaaagcaauaacaaaaacugcaauuacuucugcaccaacauaauaga -3'   exp
    ..(((.(((((.(((((((((..(((((.((.......)).......)))))..))))))))).))))).)))........     reads   mm   sample
    .........caaaaguaauugugguuuu.....................................................     5       0    ya1
    ..........aaaaguaauugugguuuu.....................................................     16      0    ya1
    ..........aaaaguaauugugguuuug....................................................     3       0    ya1
    .............................................caaaaacugcaauuacuu..................     1       0    ya1
    .............................................caaaaacugcaauuacuuc.................     2       0    ya1
    .............................................caaaaacugcaauuacuucu................     7       0    ya1

    ..........aaaaguaauugugguuuuggc..................................................     1       0    j26
    ..........aaaaguaauugugguuuuggca.................................................     1       0    j26

    ..........aaaaguaauugugguuuug....................................................     1       0    va1
    ..........aaaaguaauugugguuuugg...................................................     7       0    va1
    ..........aaaaguaauugugguuuuggc..................................................     32      0    va1
    ..........aaaaguaauugugguuuuggca.................................................     20      0    va1
    ..........aaaaguaauugugguuuuggcau................................................     1       0    va1
    ...........aaaguaauugugguuuuggc..................................................     6       0    va1

    ......gugcaaaaguaauugugguu.......................................................     1       0    ku5

    ..........aaaaguaauugugguuuuggcau................................................     1       0    k05

    ...........aaaguaauugugguuuuggc..................................................     1       0    HeL
    .............................................caaaaacugcaauuacuucug...............     1       0    HeL

    ......gugcaaaaguaauugugguu.......................................................     1       0    ku4
    ...........aaaguaauugugguuuuggca.................................................     1       0    ku4

    ..........aaaaguaauugugguuuuggc..................................................     2       0    s07

    ..........aaaaguaauugugguuuuggca.................................................     3       0    k08

    ..........aaaaguaauugugguuuu.....................................................     1       0    ta3
    ..........aaaaguaauugugguuuugg...................................................     1       0    ta3
    ..........aaaaguaauugugguuuuggc..................................................     1       0    ta3
```

Mature / Star			
agguuugugcaaaaguaauugugguuuuggcauuaaaagcaauaacaaaaacugcaauuacuucugcaccaacauaauaga			
..........aaaaguaauugugguuuuggca...	1	0	ta3
..........aaaaguaauugugguuuuggca...	7	0	j06
..........aaaaguaauugugguuuugg...	2	0	j10
..........aaaaguaauugugguuuuggcau..	1	0	j10
..........aaaaguaauugugguuuuggca...	1	0	k14
...........aaaguaauugugguuuuggca...	1	0	k14
..........aaaaguaauugugguuuu...	1	0	j02
..........aaaaguaauugugguuuug..	8	0	j02
..........aaaaguaauugugguuuugg...	2	0	j02
..........aaaaguaauugugguuuuggc..	3	0	j02
..........aaaaguaauugugguuuuggca...	1	0	j02
...........aaaguaauugugguuuuggca...	3	0	j02
....uugugcaaaaguaauugugguuu..	1	0	va2
..........aaaaguaauugugguuuuggca...	1	0	va2
..........aaaaguaauugugguuuuggc..	3	0	j31
..........aaaaguaauugugguuuuggca...	3	0	j31
..........aaaaguaauugugguuuu...	1	0	j27
..........aaaaguaauugugguuuuggca...	1	0	j27
..........aaaaguaauugugguuuuggc..	2	0	H52
..........aaaaguaauugugguuuuggca...	1	0	k09
..........aaaaguaauugugguuuuggc..	3	0	B14
..........aaaaguaauugugguuuuggca...	1	0	ku3
..........aaaaguaauugugguuuugg...	1	0	j09
..........aaaaguaauugugguuuuggc..	3	0	j09
...........aaaguaauugugguuuuggca...	1	0	j09
..........aaaaguaauugugguuuu...	1	0	j16
..........aaaaguaauugugguuuugg...	1	0	j16
.........caaaaguaauugugguuuu...	1	0	ku6
..........aaaaguaauugugguuuuggc..	1	0	ku6
..........aaaaguaauugugguuuuggca...	2	0	s12
.........caaaaguaauugugguuuu...	9	0	ya3
.........caaaaguaauugugguuuug..	5	0	ya3
..........aaaaguaauugugguuuu...	44	0	ya3
..........aaaaguaauugugguuuuggca...	1	0	ya3
..........aaaaguaauugugguuuuggcau..	3	0	ya3
....caaaaacugcaauuacuu..................	2	0	ya3
....caaaaacugcaauuacuuc.................	1	0	ya3
..........aaaaguaauugugguuuugg...	2	0	HEK
..........aaaaguaauugugguuuuggc..	11	0	HEK
..........aaaaguaauugugguuuuggca...	2	0	HEK
...........aaaguaauugugguuuuggc..	2	0	HEK
..........aaaaguaauugugguuuu...	1	0	la1
..........aaaaguaauugugguuuuggc..	1	0	la1
...........aaaguaauugugguuuugg...	1	0	la1
..........aaaaguaauugugguuuu...	1	0	j15
....caaaaacugcaauuacuucug...............	1	0	j15
..........aaaaguaauugugguuuu...	1	0	j17
..........aaaaguaauugugguuuuggc..	4	0	j17
..........aaaaguaauugugguuuuggca...	6	0	j17
..........aaaaguaauugugguuuuggcau..	1	0	j17

```
          Mature                                  Star
agguuugugcaaaaguaauugugguuuuggcauuaaaagcaauaacaaaaacugcaauuacuucugcaccaacauaauaga
...........aaaguaauugugguuuuggc..................................................   1    0    j17
...........aaaguaauugugguuuuggca.................................................   1    0    j17
.............................................caaaaacugcaauuacuucug...............   1    0    j17

.........caaaaguaauugugguuuug....................................................   1    0    la2
..........aaaaguaauugugguuuug....................................................   2    0    la2
..........aaaaguaauugugguuuuggc..................................................   7    0    la2
..........aaaaguaauugugguuuuggca.................................................   2    0    la2

...........aaaguaauugugguuuuggc..................................................   1    0    li2

..........aaaaguaauugugguuuug....................................................   1    0    va4

..........aaaaguaauugugguuuugg...................................................   1    0    va3
..........aaaaguaauugugguuuuggc..................................................   11   0    va3
..........aaaaguaauugugguuuuggca.................................................   9    0    va3
..........aaaaguaauugugguuuuggcau................................................   1    0    va3
...........aaaguaauugugguuuuggca.................................................   2    0    va3

..........aaaaguaauugugguuuugg...................................................   1    0    SW4
..........aaaaguaauugugguuuuggc..................................................   1    0    SW4

.........caaaaguaauugugguuuu.....................................................   16   0    ya4
..........aaaaguaauugugguuuu.....................................................   33   0    ya4
.............................................caaaaacugcaauuacuu..................   7    0    ya4

.........caaaaguaauugugguuuugg...................................................   1    0    ku1
..........aaaaguaauugugguuuug....................................................   1    0    ku1
..........aaaaguaauugugguuuugg...................................................   2    0    ku1
..........aaaaguaauugugguuuuggc..................................................   1    0    ku1
..........aaaaguaauugugguuuuggca.................................................   3    0    ku1
..........aaaaguaauugugguuuuggcau................................................   1    0    ku1

.........caaaaguaauugugguuuuggc..................................................   1    0    j23
..........aaaaguaauugugguuuu.....................................................   1    0    j23
..........aaaaguaauugugguuuugg...................................................   2    0    j23
...........aaaguaauugugguuuuggca.................................................   1    0    j23

..........aaaaguaauugugguuuug....................................................   1    0    s05
..........aaaaguaauugugguuuuggc..................................................   1    0    s05

..........aaaaguaauugugguuuuggca.................................................   2    0    ta2
...........aaaguaauugugguuuuggc..................................................   2    0    ta2

.........caaaaguaauugugguuuu.....................................................   3    0    j24

..........aaaaguaauugugguuuu.....................................................   1    0    ta1
..........aaaaguaauugugguuuuggc..................................................   1    0    ta1
..........aaaaguaauugugguuuuggca.................................................   3    0    ta1
...........aaaguaauugugguuuuggcau................................................   1    0    ta1

.........caaaaguaauugugguuu......................................................   1    0    NL2
.........caaaaguaauugugguuuu.....................................................   1    0    NL2
.........caaaaguaauugugguuuug....................................................   2    0    NL2
..........aaaaguaauugugguuuu.....................................................   10   0    NL2
..........aaaaguaauugugguuuuggca.................................................   3    0    NL2
..........aaaaguaauugugguuuuggcau................................................   3    0    NL2
.............................................caaaaacugcaauuacuu..................   8    0    NL2
.............................................caaaaacugcaauuacuucu................   1    0    NL2

..........aaaaguaauugugguuuu.....................................................   1    0    j13
.............................................caaaaacugcaauuacuucugc..............   1    0    j13

..........aaaaguaauugugguuuuggca.................................................   2    0    k10

..........aaaaguaauugugguuuu.....................................................   2    0    j19
..........aaaaguaauugugguuuuggca.................................................   1    0    j19

..........aaaaguaauugugguuuuggca.................................................   4    0    j03

.......ugcaaaaguaauugugguu.......................................................   2    0    j25
```

Mature **Star**

```
agguuugugcaaaaguaauugugguuuuggcauuaaaagcaauaacaaaaacugcaauuacuucugcaccaacauaauaga
.........caaaaguaauugugguuuu.....................................................   1   0   j25

..........aaaaguaauugugguuuu.....................................................   1   0   per
..........aaaaguaauugugguuuug....................................................   1   0   per

..........aaaaguaauugugguuuu.....................................................   1   0   k04
..........aaaaguaauugugguuuuggc..................................................   1   0   k04

......gugcaaaaguaauugugguuuu.....................................................   1   0   U20

..........aaaaguaauugugguuuug....................................................   1   0   ku2
..........aaaaguaauugugguuuugg...................................................   6   0   ku2
..........aaaaguaauugugguuuuggc..................................................   5   0   ku2
..........aaaaguaauugugguuuuggca.................................................   2   0   ku2
..........aaaaguaauugugguuuuggcau................................................   1   0   ku2
...........aaaguaauugugguuuuggc..................................................   1   0   ku2

..........aaaaguaauugugguuuug....................................................   2   0   j21
..........aaaaguaauugugguuuugg...................................................   3   0   j21
..........aaaaguaauugugguuuuggc..................................................   14  0   j21
..........aaaaguaauugugguuuuggca.................................................   13  0   j21
..........aaaaguaauugugguuuuggcau................................................   1   0   j21
...........aaaguaauugugguuuuggc..................................................   4   0   j21
...........aaaguaauugugguuuuggca.................................................   1   0   j21

..........aaaaguaauugugguuuuggca.................................................   2   0   j07

..........aaaaguaauugugguuuuggc..................................................   1   0   k02
..........aaaaguaauugugguuuuggcau................................................   1   0   k02

..........aaaaguaauugugguuuuggc..................................................   1   0   j28
..........aaaaguaauugugguuuuggca.................................................   4   0   j28

..........aaaaguaauugugguuuuggca.................................................   3   0   k03
...........aaaguaauugugguuuuggc..................................................   2   0   k03

.........caaaaguaauugugguuu......................................................   1   0   ya2
.........caaaaguaauugugguuuu.....................................................   6   0   ya2
..........aaaaguaauugugguuuu.....................................................   24  0   ya2
..........aaaaguaauugugguuuuggcau................................................   3   0   ya2
.............................................caaaaacugcaauuacuu..................   6   0   ya2
.............................................caaaaacugcaauuacuuc.................   1   0   ya2
.............................................caaaaacugcaauuacuucu................   3   0   ya2

.........caaaaguaauugugguuu......................................................   1   0   NL3
.........caaaaguaauugugguuuu.....................................................   1   0   NL3
.........caaaaguaauugugguuuuggcau................................................   2   0   NL3
..........aaaaguaauugugguuuu.....................................................   15  0   NL3
..........aaaaguaauugugguuuuggc..................................................   1   0   NL3
..........aaaaguaauugugguuuuggca.................................................   4   0   NL3
.............................................caaaaacugcaauuacuu..................   7   0   NL3
.............................................caaaaacugcaauuacuucu................   2   0   NL3

..........aaaaguaauugugguuuuggca.................................................   2   0   k06
..........aaaaguaauugugguuuuggcau................................................   1   0   k06

......gugcaaaaguaauugugguu.......................................................   1   0   sh2
..........aaaaguaauugugguuuuggc..................................................   2   0   sh2
..........aaaaguaauugugguuuuggca.................................................   2   0   sh2

..........aaaaguaauugugguuuuggca.................................................   2   0   j29

.......ugcaaaaguaauugugguuuu.....................................................   3   0   j01

..........aaaaguaauugugguuuuggc..................................................   1   0   MCF
..........aaaaguaauugugguuuuggca.................................................   1   0   MCF

..........aaaaguaauugugguuuuggca.................................................   5   0   NL1
..........aaaaguaauugugguuuuggcau................................................   2   0   NL1
...........aaaguaauugugguuuuggca.................................................   1   0   NL1
```

```
          Mature                                  Star
agguuugugcaaaaguaauugugguuuuggcauuaaaagcaauaacaaaaacugcaauuacuucugcaccaacauaauaga

..........aaaaguaauugugguuuuggca..................................................   3     0     k07

..........aaaaguaauugugguuuuggc...................................................   19    0     li1
..........aaaaguaauugugguuuuggca..................................................   1     0     li1
...........aaaguaauugugguuuuggc...................................................   4     0     li1

..........aaaaguaauugugguuuuggc...................................................   1     0     k13
..........aaaaguaauugugguuuuggca..................................................   2     0     k13

.........caaaaguaauugugguuuu......................................................   1     0     j20
..........aaaaguaauugugguuuug.....................................................   1     0     j20
..........aaaaguaauugugguuuugg....................................................   4     0     j20
..........aaaaguaauugugguuuuggc...................................................   4     0     j20
..........aaaaguaauugugguuuuggca..................................................   4     0     j20
............................................caaaaacugcaauuacuucug.................   1     0     j20

.........caaaaguaauugugguuuuggc...................................................   1     0     k12
..........aaaaguaauugugguuuuggc...................................................   1     0     k12
...........aaaguaauugugguuuuggca..................................................   2     0     k12
```

Provisional ID	:	hsa_can_1262
Score total	:	180.2
Score for star read(s)	:	3.9
Score for read counts	:	175.5
Score for mfe	:	1.4
Score for randfold	:	
Score for cons. seed	:	-0.6
Total read count	:	356
Mature read count	:	253
Loop read count	:	0
Star read count	:	103

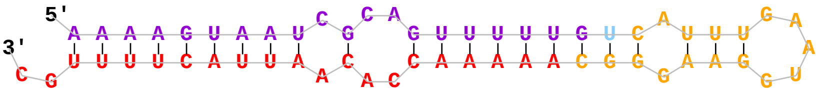

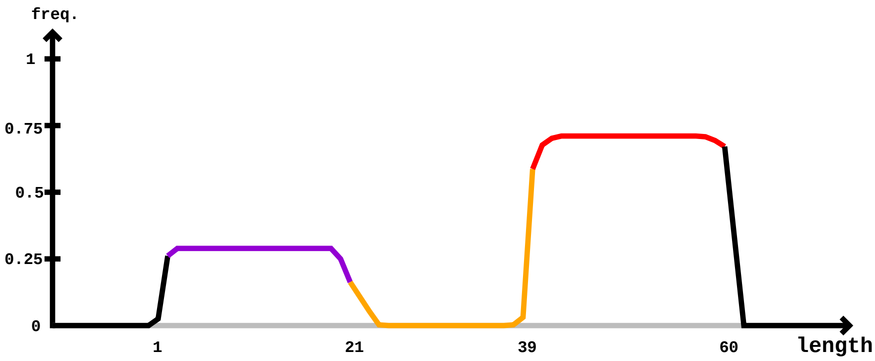

Star **Mature**

```
5'- agguuggugcaaaaguaaucgcaguuuuugucauuugaauggaagggcaaaaaccacaauuacuuuugcagcaaccugaua   -3'   obs
    agguuggugcaaaaguaaucgcaguuuuugucauuugaauggaagggcaaaaaccacaauuacuuuugcagcaaccugaua         exp
    agguuggugcaaaaguaaucgcaguuuuugucauuugaauggaagggcaaaaaccacaauuacuuuugcagcaaccugaua         known
    ((((((.((((((((((((.(..(((((((((.(((.....))).)))))))))..).)))))))))))).))))))....    reads    mm    sample
    ..........aaaaguaaucgcaguuuuu....................................................    7        0     ya1
    ................................................aaaaaccacaauuacuuuug.............    1        0     ya1
    ................................................aaaaaccacaauuacuuuugca...........    4        0     ya1
    .................................................aaaaccacaauuacuuuugca...........    3        0     ya1
    ..................................................aaaccacaauuacuuuugca...........    1        0     ya1

    ................................................aaaaaccacaauuacuuuugca...........    1        0     s04

    ................................................aaaaaccacaauuacuuuugca...........    3        0     j26

    ................................................aaaaaccacaauuacuuuugc............    1        0     va1
    ................................................aaaaaccacaauuacuuuugca...........    6        0     va1

    ................................................aaaaaccacaauuacuuuugca...........    1        0     ku5

    ................................................aaaaaccacaauuacuuuugca...........    1        0     s06

    ..........aaaaguaaucgcaguuuuuguc.................................................    1        0     HeL
    ...........aaaguaaucgcaguuuuuguc.................................................    1        0     HeL
    ................................................aaaaaccacaauuacuuuugc............    1        0     HeL
    ................................................aaaaaccacaauuacuuuugca...........    3        0     HeL

    .........caaaaguaaucgcaguuuuu....................................................    1        0     ku4
    ..........aaaaguaaucgcaguuuuu....................................................    3        0     ku4
    ..........aaaaguaaucgcaguuuuug...................................................    1        0     ku4

    ................................................aaaaaccacaauuacuuuugca...........    4        0     s10

    ..........aaaaguaaucgcaguuuuug...................................................    2        0     ta3
    ................................................aaaaaccacaauuacuuuugca...........    4        0     ta3
    .................................................aaaaccacaauuacuuuugc............    1        0     ta3

    ..........aaaaguaaucgcaguuuuug...................................................    6        0     j06
```

```
      Star                           Mature
agguuggugcaaaaguaaucgcaguuuuugucauuugaauggaagggcaaaaaccacaauuacuuuugcagcaaccugaua

.........caaaaguaaucgcaguuuuug...................................................    1    0    j10
..........aaaaguaaucgcaguuuuug...................................................    1    0    j10
................................................aaaaaccacaauuacuuuugca...........    5    0    j10

..........aaaaguaaucgcaguuuu.....................................................    3    0    j04

..........aaaaguaaucgcaguuuuu....................................................    2    0    j02

.........caaaaguaaucgcaguuuuu....................................................    2    0    va2

..................................................aaaccacaauuacuuuugca...........    1    0    j27

................................................aaaaaccacaauuacuuuugca...........    1    0    j31

................................................aaaaaccacaauuacuuuugca...........    2    0    s03
.................................................aaaaccacaauuacuuuugca...........    1    0    s03

..........aaaaguaaucgcaguuuuuguc.................................................    2    0    H52
...........aaaguaaucgcaguuuuuguc.................................................    1    0    H52
................................................aaaaaccacaauuacuuuug.............    2    0    H52
................................................aaaaaccacaauuacuuuugca...........    1    0    H52
.................................................aaaaccacaauuacuuuugc............    1    0    H52
.................................................aaaaccacaauuacuuuugca...........    2    0    H52

................................................aaaaaccacaauuacuuuugca...........    6    0    j22
.................................................aaaaccacaauuacuuuugca...........    1    0    j22

...........aaaguaaucgcaguuuuugu..................................................    1    0    B14
................................................aaaaaccacaauuacuuuugc............    3    0    B14
................................................aaaaaccacaauuacuuuugca...........    6    0    B14
..................................................aaaccacaauuacuuuugca...........    2    0    B14

................................................aaaaaccacaauuacuuuugca...........    4    0    j16

................................................aaaaaccacaauuacuuuugca...........    1    0    j09

..........aaaaguaaucgcaguuuuug...................................................    1    0    ku3

..........aaaaguaaucgcaguuuuu....................................................    1    0    ku6
..........aaaaguaaucgcaguuuuug...................................................    1    0    ku6
................................................aaaaaccacaauuacuuuugca...........    2    0    ku6

................................................aaaaaccacaauuacuuuugca...........    2    0    j11

..........aaaaguaaucgcaguuuu.....................................................    2    0    ya3
..........aaaaguaaucgcaguuuuu....................................................    2    0    ya3
................................................aaaaaccacaauuacuuuugca...........    3    0    ya3
.................................................aaaaccacaauuacuuuugca...........    2    0    ya3
..................................................aaaccacaauuacuuuugca...........    1    0    ya3

................................................aaaaaccacaauuacuuuugca...........    2    0    la1

...............................................caaaaaccacaauuacuuuugc............    3    0    HEK
................................................aaaaaccacaauuacuuuugc............    1    0    HEK
................................................aaaaaccacaauuacuuuugca...........    8    0    HEK
.................................................aaaaccacaauuacuuuugca...........    2    0    HEK
..................................................aaaccacaauuacuuuugca...........    1    0    HEK

.................................................aaaaccacaauuacuuuugca...........    1    0    j15

..........aaaaguaaucgcaguuuuu....................................................    1    0    j17
.................................................aaaaccacaauuacuuuugca...........    1    0    j17

..................................................aaaccacaauuacuuuugca...........    1    0    la2

...........aaaguaaucgcaguuuuuguc.................................................    1    0    li2

................................................aaaaaccacaauuacuuuugca...........    3    0    va4

................................................aaaaaccacaauuacuuuugca...........    12   0    va3
```

```
          Star                                  Mature
agguuggugcaaaaguaaucgcaguuuuugucauuugaauggaagggcaaaaaccacaauuacuuuugcagcaaccugaua

..........aaaaguaaucgcaguuuu.....................................................    3    0    ya4
..........aaaaguaaucgcaguuuuug...................................................    3    0    ya4
................................................aaaaaccacaauuacuuuugca...........    4    0    ya4
.................................................aaaaccacaauuacuuuugca...........    2    0    ya4
...................................................aaccacaauuacuuuugca...........    3    0    ya4

..........aaaaguaaucgcaguuuuuguc.................................................    2    0    SW4
................................................aaaaaccacaauuacuuuugca...........    1    0    SW4

.........caaaaguaaucgcaguuuuu....................................................    1    0    ku1
..........aaaaguaaucgcaguuuuu....................................................    3    0    ku1
..........aaaaguaaucgcaguuuuug...................................................    2    0    ku1
................................................aaaaaccacaauuacuuuugca...........    4    0    ku1

.........caaaaguaaucgcaguuuuug...................................................    2    0    j23
..........aaaaguaaucgcaguuuuug...................................................    1    0    j23
................................................aaaaaccacaauuacuuuugc............    1    0    j23
................................................aaaaaccacaauuacuuuugca...........    2    0    j23
.................................................aaaaccacaauuacuuuugca...........    1    0    j23

................................................aaaaaccacaauuacuuuugca...........    2    0    s05

................................................aaaaaccacaauuacuuu...............    1    0    ta2

................................................aaaaaccacaauuacuuuugca...........    1    0    k11

................................................aaaaaccacaauuacuuuugca...........    1    0    ta1

...............................................caaaaaccacaauuacuuuugc............    1    0    s09
.................................................aaaaccacaauuacuuuugca...........    1    0    s09

...............................................caaaaaccacaauuacuuuu..............    1    0    NL2
................................................aaaaaccacaauuacuuuugca...........    3    0    NL2

.........caaaaguaaucgcaguuuu.....................................................    1    0    j13
..........aaaaguaaucgcaguuuuug...................................................    2    0    j13
................................................aaaaaccacaauuacuuuugca...........    4    0    j13
.................................................aaaaccacaauuacuuuugca...........    2    0    j13

.........caaaaguaaucgcaguuuuu....................................................    1    0    j19
..........aaaaguaaucgcaguuuuug...................................................    3    0    j19
................................................aaaaaccacaauuacuuuugca...........    2    0    j19
..................................................aaaccacaauuacuuuugca...........    2    0    j19

..........aaaaguaaucgcaguuuuug...................................................    2    0    j25
................................................aaaaaccacaauuacuuuugca...........    1    0    j25

................................................aaaaaccacaauuacuuuugca...........    2    0    s01

.................................................aaaaccacaauuacuuuugca...........    1    0    k04

..........aaaaguaaucgcaguuuuu....................................................    1    0    ku2
..........aaaaguaaucgcaguuuuug...................................................    4    0    ku2

...........aaaguaaucgcaguuuuuguca................................................    1    0    U20
...............................................caaaaaccacaauuacuuuugc............    1    0    U20
................................................aaaaaccacaauuacuuuugc............    22   0    U20
................................................aaaaaccacaauuacuuuugca...........    17   0    U20
.................................................aaaaccacaauuacuuuugca...........    1    0    U20

..........aaaaguaaucgcaguuuuug...................................................    1    0    j21
................................................aaaaaccacaauuacuuuugca...........    3    0    j21
.................................................aaaaccacaauuacuuuugca...........    1    0    j21


................................................aaaaaccacaauuacuuuugca...........    1    0    j07

................................................aaaaaccacaauuacuuuugca...........    1    0    s08

..........aaaaguaaucgcaguuuuugu..................................................    2    0    DLD
```

```
          Star                                   Mature
agguuggugcaaaaguaaucgcaguuuuugucauuugaauggaagggcaaaaaccacaauuacuuuugcagcaaccugaua
..........aaaaguaaucgcaguuuuuguc.................................................      1      0      DLD
................................................aaaaaccacaauuacuuuugc............      1      0      DLD

................................................aaaaaccacaauuacuuuugca...........      1      0      k03

..........aaaaguaaucgcaguuuu.....................................................      3      0      ya2
..........aaaaguaaucgcaguuuuu....................................................      6      0      ya2
................................................aaaaaccacaauuacuuuu..............      2      0      ya2
................................................aaaaaccacaauuacuuuugc............      1      0      ya2
................................................aaaaaccacaauuacuuuugca...........      2      0      ya2
.................................................aaaaccacaauuacuuuugca...........      6      0      ya2

..........aaaaguaaucgcaguuuu.....................................................      1      0      NL3
...............................................caaaaaccacaauuacuuuu..............      1      0      NL3
...............................................caaaaaccacaauuacuuuug.............      2      0      NL3
................................................aaaaaccacaauuacuuuugca...........      7      0      NL3

..........aaaaguaaucgcaguuuuug...................................................      1      0      j29
................................................aaaaaccacaauuacuuuugca...........      1      0      j29
.................................................aaaaccacaauuacuuuugca...........      1      0      j29

...............................................caaaaaccacaauuacuuuug.............      1      0      sh2

................................................aaaaaccacaauuacuuuu..............      1      0      MCF
................................................aaaaaccacaauuacuuuugc............      1      0      MCF
................................................aaaaaccacaauuacuuuugca...........      2      0      MCF

..........aaaaguaaucgcaguuuuuguc.................................................      2      0      MBM
................................................aaaaaccacaauuacuuuug.............      1      0      MBM
................................................aaaaaccacaauuacuuuugc............      1      0      MBM
................................................aaaaaccacaauuacuuuugca...........      2      0      MBM
.................................................aaaaccacaauuacuuuugca...........      1      0      MBM

..............................................gcaaaaaccacaauuacuuuug.............      1      0      li1
................................................aaaaaccacaauuacuuuugc............      1      0      li1

..........aaaaguaaucgcaguuuu.....................................................      1      0      j20
..........aaaaguaaucgcaguuuuug...................................................      2      0      j20

..........aaaaguaaucgcaguuuuuguc.................................................      2      0      A54
...........aaaguaaucgcaguuuuuguc.................................................      5      0      A54
................................................aaaaaccacaauuacuuuugc............      7      0      A54
```

Provisional ID	:	hsa_can_1270
Score total	:	613.8
Score for star read(s)	:	3.9
Score for read counts	:	608.3
Score for mfe	:	2.1
Score for randfold	:	
Score for cons. seed	:	-0.6
Total read count	:	1205
Mature read count	:	1176
Loop read count	:	0
Star read count	:	29

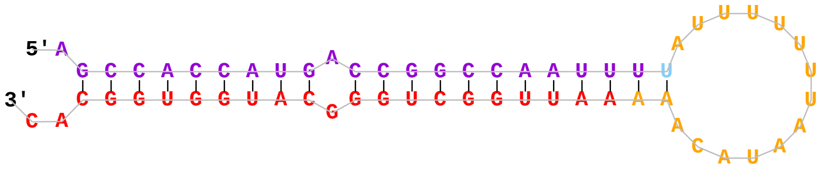

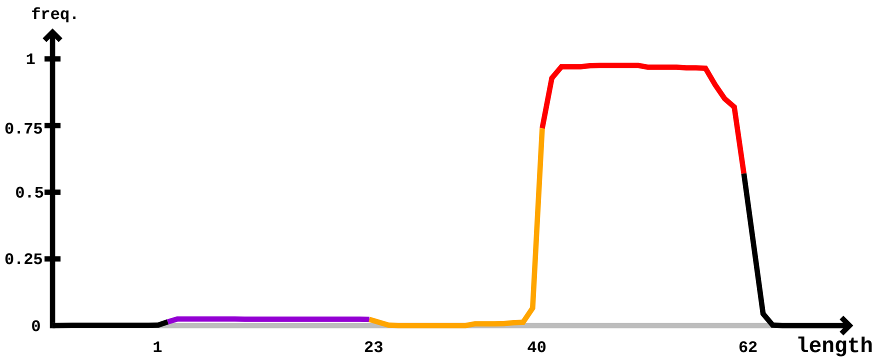

Star Mature

```
5'- uacaggcgugagccaccaugaccggccaauuuuauuuuuuuaauacaaaaauuggcugggcaugguggcacaugcccguag  -3'   obs
    uacaggcgugagccaccaugaccggccaauuuuauuuuuuuaauacaaaaauuggcugggcaugguggcacaugcccguag        exp
    (((.((((((.(((((((((.(((((((((((.............))))))))))).))))))))).)))))).))).      reads   mm   sample
    ..........agccaccaugaccggccaauu..................................................   1       0    j23
    ..........agccaccaugaccggccaauuu.................................................   3       0    j23
    ..........agccaccaugaccggccaauuuu................................................   1       0    j23
    ................................................aaauuggcugggcauggug..............   2       0    j23
    ................................................aaauuggcugggcaugguggg.............   6       0    j23
    ................................................aaauuggcugggcaugguggca...........   10      0    j23
    ................................................aaauuggcugggcaugguggcac..........   8       0    j23
    ................................................aaauuggcugggcaugguggcaca.........   1       0    j23
    .................................................aauuggcugggcauggug..............   17      0    j23
    .................................................aauuggcugggcauggugg.............   26      0    j23
    .................................................aauuggcugggcaugguggc............   13      0    j23
    .................................................aauuggcugggcaugguggca...........   121     0    j23
    .................................................aauuggcugggcaugguggcac..........   123     0    j23
    .................................................aauuggcugggcaugguggcaca.........   37      0    j23
    .................................................aauuggcugggcaugguggcacau........   3       0    j23
    ..................................................auuggcugggcaugguggca...........   9       0    j23
    ..................................................auuggcugggcaugguggcac..........   52      0    j23
    ..................................................auuggcugggcaugguggcaca.........   45      0    j23
    ..................................................auuggcugggcaugguggcacau........   6       0    j23
    ...................................................uuggcugggcaugguggca...........   5       0    j23
    ...................................................uuggcugggcaugguggcac..........   8       0    j23
    ...................................................uuggcugggcaugguggcaca.........   4       0    j23
    ...................................................uuggcugggcaugguggcacau........   15      0    j23
    ...................................................uuggcugggcaugguggcacaug.......   1       0    j23

    .................................................aauuggcugggcauggug..............   1       0    ya1

    .................................................aauuggcugggcaugguggca...........   3       0    s04
    .................................................aauuggcugggcaugguggcac..........   2       0    s04
    ..................................................auuggcugggcaugguggcaca.........   1       0    s04

    ..............................................aaaaauuggcugggcaugguggca...........   1       0    j26
    ................................................aaauuggcugggcauggu...............   1       0    j26
```

Star Mature			
uacaggcgugagccaccaugaccggccaauuuuauuuuuuuaauacaaaaauuggcugggcaugguggcacaugcccguag			
....gcugggcaugguggcaca.........	3	0	j26
....auuggcugggcaugguggcac..........	3	0	j24
....aaaaauuggcugggcaugguggcac..........	1	0	k05
....aaauuggcugggcauggug..............	1	0	s06
....aauuggcugggcauggugg.............	1	0	s06
....aauuggcugggcaugguggca...........	1	0	s06
....aauuggcugggcaugguggcac..........	3	0	s06
....auuggcugggcaugguggcaca.........	1	0	s10
....aauuggcugggcaugguggcac..........	1	0	ta3
..........agccaccaugaccggccaauuu...	1	0	NL2
...........gccaccaugaccggccaauuu...	1	0	NL2
...........gccaccaugaccggccaauuuu..	5	0	NL2
....aaauuggcugggcauggug..............	3	0	NL2
....aaauuggcugggcaugguggc............	1	0	NL2
....aaauuggcugggcaugguggcac..........	1	0	NL2
....aauuggcugggcauggug..............	3	0	NL2
....aauuggcugggcaugguggca...........	2	0	NL2
....aauuggcugggcaugguggcac..........	1	0	NL2
....auuggcugggcaugguggca...........	1	0	NL2
....auacaaaaauuggcuggg.....................	8	0	j06
....aauuggcugggcaugguggca...........	2	0	j06
....aaauuggcugggcaugguggcaca.........	2	0	j19
....aauuggcugggcauggug..............	2	0	j19
....aauuggcugggcaugguggc............	1	0	j19
....aauuggcugggcaugguggca...........	11	0	j19
....aauuggcugggcaugguggcac..........	7	0	j19
....auuggcugggcaugguggcac..........	2	0	j19
....auuggcugggcaugguggcaca.........	2	0	j19
....auuggcugggcaugguggcacau........	1	0	j19
....aauuggcugggcaugguggca...........	1	0	j03
....aauuggcugggcauggugg.............	1	0	j25
....gcugggcaugguggcaca.........	2	0	s01
....aauuggcugggcauggug..............	1	0	j10
....aauuggcugggcaugguggcac..........	1	0	j10
..........agccaccaugaccggccaauuu...	1	0	j21
..........agccaccaugaccggccaauuuu..	1	0	j21
...........gccaccaugaccggccaauuuu..	1	0	j21
....aaauuggcugggcaugguggc............	1	0	j21
....aaauuggcugggcaugguggcac..........	2	0	j21
....aauuggcugggcauggug..............	7	0	j21
....aauuggcugggcauggugg.............	9	0	j21
....aauuggcugggcaugguggc............	4	0	j21
....aauuggcugggcaugguggca...........	36	0	j21
....aauuggcugggcaugguggcac..........	78	0	j21
....aauuggcugggcaugguggcaca.........	30	0	j21
....aauuggcugggcaugguggcacau........	8	0	j21
....auuggcugggcauggugg.............	2	0	j21
....auuggcugggcaugguggca...........	4	0	j21
....auuggcugggcaugguggcac..........	12	0	j21
....auuggcugggcaugguggcaca.........	21	0	j21
....auuggcugggcaugguggcacau........	3	0	j21
....uuggcugggcaugguggcaca.........	2	0	j21
....uuggcugggcaugguggcacau........	8	0	j21
....aauuggcugggcaugguggca...........	2	0	j07
....aauuggcugggcaugguggcaca.........	1	0	j07
....aaauuggcugggcauggug..............	1	0	j27

```
          Star                                    Mature
uacaggcgugagccaccaugaccggccaauuuuauuuuuuuaauacaaaaauuggcugggcaugguggcacaugcccguag
.................................................aauuggcugggcauggug..............    2    0    j27
..................................................auuggcugggcaugguggcac..........    1    0    j27

.................................................aauuggcugggcaugguggcac..........    1    0    j22

..............................................aaaaauuggcugggcaug.................    1    0    k09
.................................................aauuggcugggcaugguggcaca.........    1    0    k09

..................................................auuggcugggcaugguggcaca.........    1    0    k03

.................................................aauuggcugggcaugguggca...........    1    0    ku3

.................................................aauuggcugggcaugguggcac..........    1    0    j18

.........gagccaccaugaccggccaauuu.................................................    1    0    NL3
..........agccaccaugaccggccaauuu.................................................    6    0    NL3
..........agccaccaugaccggccaauuuu................................................    1    0    NL3
...........gccaccaugaccggccaauuuu................................................    1    0    NL3
...........gccaccaugaccggccaauuuua...............................................    2    0    NL3
................................................aaauuggcugggcauggug..............    3    0    NL3
.................................................aauuggcugggcauggug..............    9    0    NL3
.................................................aauuggcugggcaugguggc............    1    0    NL3
.................................................aauuggcugggcaugguggca...........    5    0    NL3
.................................................aauuggcugggcaugguggcac..........    6    0    NL3
.................................................aauuggcugggcaugguggcaca.........    1    0    NL3
..................................................auuggcugggcaugguggca...........    1    0    NL3
..................................................auuggcugggcaugguggcac..........    1    0    NL3
...................................................uuggcugggcaugguggcacau........    1    0    NL3

.................................................aauuggcugggcauggug..............    1    0    ya2
.................................................aauuggcugggcaugguggca...........    1    0    ya2
..................................................auuggcugggcaugguggca...........    1    0    ya2
..................................................auuggcugggcaugguggcac..........    1    0    ya2

..................................................auuggcugggcaugguggca...........    1    0    j11

.................................................aauuggcugggcauggug..............    1    0    ya3

.................................................aauuggcugggcauggug..............    1    0    k06

.................................................aauuggcugggcaugguggcac..........    2    0    j29

.............................................caaaaauuggcugggcaug.................    1    0    la1
..............................................aaaaauuggcugggcaug.................    1    0    la1

.................................................aauuggcugggcaugguggca...........    1    0    HEK
.................................................aauuggcugggcaugguggcac..........    1    0    HEK
..................................................auuggcugggcaugguggcaca.........    1    0    HEK
...................................................uuggcugggcaugguggcacau........    1    0    HEK

.................................................aauuggcugggcaugguggca...........    1    0    sh2

...........gccaccaugaccggccaauuuu................................................    1    0    j15
...............................................aaaauuggcugggcaugguggcac..........    1    0    j15
................................................aaauuggcugggcaugguggc............    1    0    j15
................................................aaauuggcugggcaugguggca...........    3    0    j15
................................................aaauuggcugggcaugguggcaca.........    1    0    j15
.................................................aauuggcugggcauggug..............    6    0    j15
.................................................aauuggcugggcauggugg.............    7    0    j15
.................................................aauuggcugggcaugguggc............    4    0    j15
.................................................aauuggcugggcaugguggca...........    47   0    j15
.................................................aauuggcugggcaugguggcac..........    35   0    j15
.................................................aauuggcugggcaugguggcaca.........    18   0    j15
..................................................auuggcugggcaugguggca...........    4    0    j15
..................................................auuggcugggcaugguggcac..........    10   0    j15
..................................................auuggcugggcaugguggcaca.........    15   0    j15
...................................................uuggcugggcaugguggcaca.........    1    0    j15
...................................................uuggcugggcaugguggcacau........    1    0    j15

................................................aaauuggcugggcauggug..............    2    0    j17
```

Star   Mature

```
uacaggcgugagccaccaugaccggccaauuuuauuuuuuuaauacaaaaauuggcugggcaugguggcacaugcccguag
................................................aaauuggcugggcaugguggcac..........   2    0    j17
.................................................aauuggcugggcauggug..............   1    0    j17
.................................................aauuggcugggcaugguggc............   1    0    j17
.................................................aauuggcugggcaugguggca...........   10   0    j17
.................................................aauuggcugggcaugguggcac..........   12   0    j17
.................................................aauuggcugggcaugguggcaca.........   3    0    j17
..................................................auuggcugggcaugguggcac..........   5    0    j17
..................................................auuggcugggcaugguggcaca.........   2    0    j17
..................................................auuggcugggcaugguggcacau........   1    0    j17
...................................................uuggcugggcaugguggcaca.........   3    0    j17

.................................................aauuggcugggcaugguggca...........   1    0    j01

...............................................aaaauuggcugggcauggug..............   1    0    NL1
................................................aaauuggcugggcauggu...............   1    0    NL1
................................................aaauuggcugggcauggug..............   2    0    NL1
................................................aaauuggcugggcaugugg.............   1    0    NL1
................................................aaauuggcugggcaugguggc............   1    0    NL1
................................................aaauuggcugggcaugguggca...........   1    0    NL1
................................................aaauuggcugggcaugguggcac..........   4    0    NL1
.................................................aauuggcugggcauggug..............   5    0    NL1
.................................................aauuggcugggcaugguggc............   4    0    NL1
.................................................aauuggcugggcaugguggca...........   6    0    NL1
.................................................aauuggcugggcaugguggcac..........   23   0    NL1
.................................................aauuggcugggcaugguggcaca.........   1    0    NL1
.................................................aauuggcugggcaugguggcacau........   2    0    NL1
..................................................auuggcugggcaugguggc............   4    0    NL1
..................................................auuggcugggcaugguggcac..........   7    0    NL1
..................................................auuggcugggcaugguggcaca.........   1    0    NL1
..................................................auuggcugggcaugguggcacau........   1    0    NL1
...................................................uuggcugggcaugguggcac..........   1    0    NL1

...................................................uuggcugggcaugguggca...........   1    0    j12
.......................................................cugggcaugguggcacaug.......   1    0    j12

................................................aaauuggcugggcauggug..............   1    0    li1
................................................aaauuggcugggcauggugg.............   1    0    li1
................................................aaauuggcugggcaugguggca...........   1    0    li1
.................................................aauuggcugggcauggug..............   2    0    li1
.................................................aauuggcugggcauggugg.............   8    0    li1
.................................................aauuggcugggcaugguggc............   2    0    li1
.................................................aauuggcugggcaugguggca...........   9    0    li1
.................................................aauuggcugggcaugguggcac..........   16   0    li1
..................................................auuggcugggcauggugg.............   1    0    li1
..................................................auuggcugggcaugguggcac..........   3    0    li1
..................................................auuggcugggcaugguggcaca.........   1    0    li1

...........gccaccaugaccggccaauuuu................................................   2    0    A54
.................................................aauuggcugggcauggugg.............   1    0    A54
.................................................aauuggcugggcaugguggcacau........   1    0    A54
..................................................auuggcugggcaugguggcac..........   1    0    A54

uacaggcgugagccacca...............................................................   1    0    ya4
```

Provisional ID : hsa_can_1306
Score total : 3.6
Score for star read(s) : -1.3
Score for read counts : 0
Score for mfe : 1.9
Score for randfold :
Score for cons. seed : 3
Total read count : 2411
Mature read count : 2407
Loop read count : 0
Star read count : 4

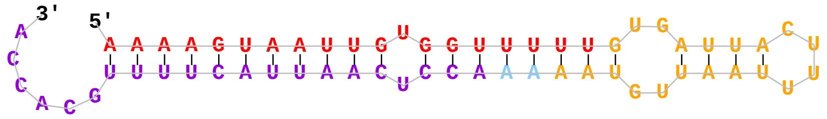

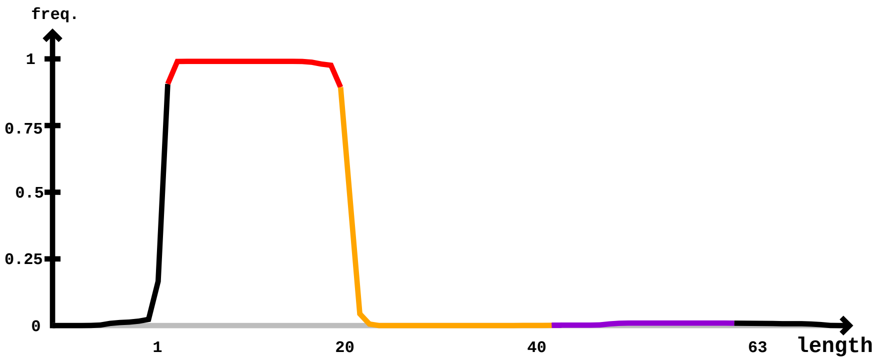

Mature Star

```
5'- caguuggugcaaaaguaauugugguuuuugugauuacuuuuaauuguaaaaaccucaauuacuuuugcaccaaccuaauaa   -3'    obs
    caguuggugcaaaaguaauugugguuuuugugauuacuuuuaauuguaaaaaccucaauuacuuuugcaccaaccuaauaa          exp
    ..(((((((((((((((((((.((((((((..((((....))))..)))))))).)))))))))))))))))))).......    reads   mm   sample
    .........caaaaguaauugugguuuu.....................................................    5       0    ya1
    .........caaaaguaauugugguuuuu....................................................    22      0    ya1
    .........caaaaguaauugugguuuuug...................................................    1       0    ya1
    .........caaaaguaauugugguuuuugu..................................................    1       0    ya1
    ..........aaaaguaauugugguuuu.....................................................    16      0    ya1
    ..........aaaaguaauugugguuuuu....................................................    111     0    ya1
    ..........aaaaguaauugugguuuuug...................................................    8       0    ya1
    ...........aaaguaauugugguuuuu....................................................    19      0    ya1
    ...........aaaguaauugugguuuuugu..................................................    1       0    ya1

    ..........aaaaguaauugugguuuuug...................................................    1       0    s04

    .........caaaaguaauugugguuuuugu..................................................    1       0    j26
    ..........aaaaguaauugugguuuuug...................................................    1       0    j26

    ....uggugcaaaaguaauugugguu.......................................................    1       0    va1
    ..........aaaaguaauugugguuuuu....................................................    2       0    va1
    ..........aaaaguaauugugguuuuug...................................................    25      0    va1
    ..........aaaaguaauugugguuuuugu..................................................    2       0    va1
    ...........aaaguaauugugguuuuug...................................................    2       0    va1

    ...uuggugcaaaaguaauugugguuu......................................................    1       0    ku5
    ....uggugcaaaaguaauuguggu........................................................    1       0    ku5
    ......gugcaaaaguaauugugguu.......................................................    1       0    ku5
    .........caaaaguaauugugguuuuug...................................................    1       0    ku5
    ..........aaaaguaauugugguuuuug...................................................    3       0    ku5

    .........caaaaguaauugugguuuuu....................................................    2       0    k05
    .........caaaaguaauugugguuuuug...................................................    2       0    k05
    .........caaaaguaauugugguuuuugu..................................................    3       0    k05
    ..........aaaaguaauugugguuuuug...................................................    3       0    k05
    ...........aaaguaauugugguuuuug...................................................    1       0    k05

    .........caaaaguaauugugguuuuug...................................................    1       0    s06
```

```
          Mature                                   Star
caguuggugcaaaaguaauugugguuuuugugauuacuuuuaauuguaaaaaccucaauuacuuuugcaccaaccuaauaa

..........aaaaguaauugugguuuuugu..................................................   1    0    HeL

......gugcaaaaguaauugugguu.......................................................   1    0    ku4
..........aaaaguaauugugguuuuu....................................................   5    0    ku4
..........aaaaguaauugugguuuuug...................................................   11   0    ku4

..........aaaaguaauugugguuuuug...................................................   2    0    s10
..........aaaaguaauugugguuuuugu..................................................   1    0    s10

.........caaaaguaauugugguuuuu....................................................   2    0    k08
.........caaaaguaauugugguuuuug...................................................   1    0    k08
.........caaaaguaauugugguuuuugu..................................................   2    0    k08
..........aaaaguaauugugguuuuu....................................................   1    0    k08
..........aaaaguaauugugguuuuug...................................................   1    0    k08

..........aaaaguaauugugguuu......................................................   1    0    ta3
..........aaaaguaauugugguuuuug...................................................   7    0    ta3
...........aaaguaauugugguuuuug...................................................   1    0    ta3

..........aaaaguaauugugguuuuu....................................................   2    0    j10
..........aaaaguaauugugguuuuug...................................................   6    0    j10

..........aaaaguaauugugguuuuug...................................................   3    0    j04

..........aaaaguaauugugguuu......................................................   1    0    j02
..........aaaaguaauugugguuuuu....................................................   5    0    j02
..........aaaaguaauugugguuuuug...................................................   22   0    j02
..........aaaaguaauugugguuuuugu..................................................   2    0    j02
...........aaaguaauugugguuuuug...................................................   1    0    j02

.........caaaaguaauugugguuuuu....................................................   2    0    k14
.........caaaaguaauugugguuuuug...................................................   4    0    k14
.........caaaaguaauugugguuuuugu..................................................   1    0    k14
..........aaaaguaauugugguuuuu....................................................   2    0    k14
..........aaaaguaauugugguuuuug...................................................   4    0    k14
..........aaaaguaauugugguuuuugu..................................................   1    0    k14
...........aaaguaauugugguuuuug...................................................   1    0    k14
............aaguaauugugguuuuugu..................................................   1    0    k14

.........caaaaguaauugugguuuuu....................................................   1    0    va2
.........caaaaguaauugugguuuuugu..................................................   1    0    va2
..........aaaaguaauugugguuuuu....................................................   1    0    va2
..........aaaaguaauugugguuuuug...................................................   4    0    va2
..........aaaaguaauugugguuuuugug.................................................   1    0    va2
.......................................................caauuacuuuugcaccaaccuaa...   1    0    va2

.....ggugcaaaaguaauugugguu.......................................................   1    0    j27
.........caaaaguaauugugguuuuu....................................................   1    0    j27
.........caaaaguaauugugguuuuug...................................................   4    0    j27
.........caaaaguaauugugguuuuugu..................................................   2    0    j27
..........aaaaguaauugugguuu......................................................   1    0    j27
..........aaaaguaauugugguuuuu....................................................   2    0    j27
..........aaaaguaauugugguuuuug...................................................   13   0    j27

...uuggugcaaaaguaauuguggu........................................................   1    0    j31
....uggugcaaaaguaauugugguu.......................................................   1    0    j31
.........caaaaguaauugugguuuuu....................................................   1    0    j31
.........caaaaguaauugugguuuuug...................................................   2    0    j31
.........caaaaguaauugugguuuuugu..................................................   3    0    j31
..........aaaaguaauugugguuuuug...................................................   1    0    j31

..........aaaaguaauugugguuuuu....................................................   2    0    H52

.........caaaaguaauugugguuuuu....................................................   1    0    j22
.........caaaaguaauugugguuuuug...................................................   1    0    j22
.........caaaaguaauugugguuuuugu..................................................   3    0    j22
..........aaaaguaauugugguuuuu....................................................   1    0    j22
..........aaaaguaauugugguuuuug...................................................   33   0    j22
..........aaaaguaauugugguuuuugu..................................................   3    0    j22
```

```
          Mature                                    Star
caguuggugcaaaaguaauugugguuuuugugauuacuuuuaauuguaaaaaccucaauuacuuuugcaccaaccuaauaa
..........aaaaguaauugugguuuuugug.................................................   2    0    j22
...........aaaguaauugugguuuuug...................................................   3    0    j22

...uuggugcaaaaguaauugugg.........................................................   1    0    k09
.......ugcaaaaguaauugugguuuuu....................................................   1    0    k09
.........caaaaguaauugugguuuuu....................................................   1    0    k09
.........caaaaguaauugugguuuuug...................................................   2    0    k09
.........caaaaguaauugugguuuuugu..................................................   1    0    k09
..........aaaaguaauugugguuuuu....................................................   2    0    k09
..........aaaaguaauugugguuuuug...................................................   4    0    k09
..........aaaaguaauugugguuuuugu..................................................   2    0    k09

.........caaaaguaauugugguuuuug...................................................   3    0    B14
.........caaaaguaauugugguuuuugu..................................................   1    0    B14

.........caaaaguaauugugguuuuug...................................................   1    0    j16
..........aaaaguaauugugguuuu.....................................................   1    0    j16
..........aaaaguaauugugguuuuu....................................................   12   0    j16
..........aaaaguaauugugguuuuug...................................................   25   0    j16

.........caaaaguaauugugguuuuu....................................................   2    0    j09
..........aaaaguaauugugguuuuu....................................................   4    0    j09
..........aaaaguaauugugguuuuug...................................................   9    0    j09
..........aaaaguaauugugguuuuugug.................................................   2    0    j09
...........aaaguaauugugguuuuug...................................................   1    0    j09

.........caaaaguaauugugguuuuu....................................................   3    0    j18
.........caaaaguaauugugguuuuug...................................................   1    0    j18
..........aaaaguaauugugguuuuug...................................................   1    0    j18
..........aaaaguaauugugguuuuugu..................................................   1    0    j18

....uggugcaaaaguaauuguggu........................................................   1    0    ku3
.........caaaaguaauugugguuuuug...................................................   1    0    ku3
.........caaaaguaauugugguuuuugu..................................................   1    0    ku3
..........aaaaguaauugugguuuuug...................................................   6    0    ku3

.........caaaaguaauugugguuuu.....................................................   1    0    ku6
.........caaaaguaauugugguuuuu....................................................   1    0    ku6
.........caaaaguaauugugguuuuugu..................................................   1    0    ku6
..........aaaaguaauugugguuuuug...................................................   2    0    ku6

..........aaaaguaauugugguuuuu....................................................   4    0    j11
..........aaaaguaauugugguuuuug...................................................   7    0    j11
...........aaaguaauugugguuuuug...................................................   1    0    j11

..........aaaaguaauugugguuuuug...................................................   4    0    s12
...........aaaguaauugugguuuuug...................................................   2    0    s12

........gcaaaaguaauugugguuuuu....................................................   2    0    ya3
.........caaaaguaauugugguuuu.....................................................   9    0    ya3
.........caaaaguaauugugguuuuu....................................................   22   0    ya3
.........caaaaguaauugugguuuuugu..................................................   4    0    ya3
..........aaaaguaauugugguuuu.....................................................   44   0    ya3
..........aaaaguaauugugguuuuu....................................................   238  0    ya3
..........aaaaguaauugugguuuuug...................................................   27   0    ya3
..........aaaaguaauugugguuuuugu..................................................   1    0    ya3
...........aaaguaauugugguuuuu....................................................   45   0    ya3
...........aaaguaauugugguuuuug...................................................   8    0    ya3

.........caaaaguaauugugguuuuu....................................................   1    0    la1
.........caaaaguaauugugguuuuugu..................................................   1    0    la1
..........aaaaguaauugugguuuu.....................................................   1    0    la1
..........aaaaguaauugugguuuuu....................................................   1    0    la1
..........aaaaguaauugugguuuuug...................................................   3    0    la1
...................................................accucaauuacuuuugcacca.........   1    0    la1

....uggugcaaaaguaauugugguu.......................................................   1    0    HEK
.........caaaaguaauugugguuuuugu..................................................   2    0    HEK

..........aaaaguaauugugguuuu.....................................................   1    0    j17
```

```
            Mature                              Star
caguuggugcaaaaguaauugugguuuuugugauuacuuuuaauuguaaaaaccucaauuacuuuugcaccaaccuaauaa
..........aaaaguaauugugguuuuu....................................................    8    0    j17
..........aaaaguaauugugguuuuug...................................................    8    0    j17

.........caaaaguaauugugguuuuug...................................................    4    0    j15
..........aaaaguaauugugguuuu.....................................................    1    0    j15
..........aaaaguaauugugguuuuu....................................................    3    0    j15
..........aaaaguaauugugguuuuug...................................................    16   0    j15
...........aaaguaauugugguuuuu....................................................    1    0    j15
...........aaaguaauugugguuuuug...................................................    1    0    j15

..........aaaaguaauugugguuuuu....................................................    1    0    la2
..........aaaaguaauugugguuuuug...................................................    3    0    la2
...................................................accucaauuacuuuugcacca.........    1    0    la2

....uggugcaaaaguaauugugguu.......................................................    1    0    j12
.........caaaaguaauugugguuuuug...................................................    5    0    j12
..........aaaaguaauugugguuuuu....................................................    6    0    j12
..........aaaaguaauugugguuuuug...................................................    24   0    j12
..........aaaaguaauugugguuuuugug.................................................    1    0    j12
...........aaaguaauugugguuuuug...................................................    2    0    j12

....uggugcaaaaguaauugugguu.......................................................    1    0    li2
.........caaaaguaauugugguuuuug...................................................    4    0    li2

.........caaaaguaauugugguuuuug...................................................    1    0    va4
..........aaaaguaauugugguuuuu....................................................    1    0    va4
..........aaaaguaauugugguuuuug...................................................    5    0    va4
..........aaaaguaauugugguuuuugu..................................................    1    0    va4

.........caaaaguaauugugguuuuug...................................................    2    0    va3
..........aaaaguaauugugguuuuu....................................................    2    0    va3
..........aaaaguaauugugguuuuug...................................................    10   0    va3
...........aaaguaauugugguuuuug...................................................    2    0    va3

.........caaaaguaauugugguuuuu....................................................    1    0    SW4
.........caaaaguaauugugguuuuugu..................................................    1    0    SW4
..........aaaaguaauugugguuuuu....................................................    1    0    SW4
..........aaaaguaauugugguuuuug...................................................    3    0    SW4
..........aaaaguaauugugguuuuugu..................................................    2    0    SW4

.........caaaaguaauugugguuuu.....................................................    16   0    ya4
.........caaaaguaauugugguuuuu....................................................    16   0    ya4
.........caaaaguaauugugguuuuug...................................................    4    0    ya4
..........aaaaguaauugugguuuu.....................................................    33   0    ya4
..........aaaaguaauugugguuuuu....................................................    213  0    ya4
..........aaaaguaauugugguuuuug...................................................    5    0    ya4
..........aaaaguaauugugguuuuugu..................................................    1    0    ya4
...........aaaguaauugugguuuuu....................................................    43   0    ya4
...........aaaguaauugugguuuuug...................................................    3    0    ya4

.........caaaaguaauugugguuuuu....................................................    1    0    ku1
.........caaaaguaauugugguuuuug...................................................    1    0    ku1
.........caaaaguaauugugguuuuugu..................................................    1    0    ku1
..........aaaaguaauugugguuuuu....................................................    5    0    ku1
..........aaaaguaauugugguuuuug...................................................    38   0    ku1
...........aaaguaauugugguuuuug...................................................    1    0    ku1

.......ugcaaaaguaauugugguuuuu....................................................    1    0    j23
.........caaaaguaauugugguuuuug...................................................    4    0    j23
.........caaaaguaauugugguuuuugu..................................................    1    0    j23
..........aaaaguaauugugguuuu.....................................................    1    0    j23
..........aaaaguaauugugguuuuu....................................................    8    0    j23
..........aaaaguaauugugguuuuug...................................................    89   0    j23
..........aaaaguaauugugguuuuugu..................................................    2    0    j23
..........aaaaguaauugugguuuuugug.................................................    4    0    j23
...........aaaguaauugugguuuuug...................................................    11   0    j23

.........caaaaguaauugugguuuuugu..................................................    1    0    s05
..........aaaaguaauugugguuuuug...................................................    7    0    s05
...........aaaguaauugugguuuuug...................................................    1    0    s05
```

```
          Mature                                   Star
caguuggugcaaaaguaauugugguuuuugugauuacuuuuaauuguaaaaaccucaauuacuuuugcaccaaccuaauaa

...........aaaguaauugugguuuuug...................................................	1	0	ta2

.....ggugcaaaaguaauugugguuu......................................................	5	0	j24
.........caaaaguaauugugguuuu.....................................................	3	0	j24
.........caaaaguaauugugguuuuug...................................................	1	0	j24
.........caaaaguaauugugguuuuugu..................................................	2	0	j24
..........aaaaguaauugugguuuuu....................................................	3	0	j24
..........aaaaguaauugugguuuuug...................................................	8	0	j24

.........caaaaguaauugugguuuuug...................................................	1	0	k11
.........caaaaguaauugugguuuuugu..................................................	2	0	k11
..........aaaaguaauugugguuuuu....................................................	2	0	k11
..........aaaaguaauugugguuuuug...................................................	2	0	k11

..........aaaaguaauugugguuuu.....................................................	1	0	ta1
..........aaaaguaauugugguuuuu....................................................	1	0	ta1
..........aaaaguaauugugguuuuug...................................................	3	0	ta1
...........aaaguaauugugguuuuug...................................................	1	0	ta1

.........caaaaguaauugugguuuuug...................................................	1	0	s09
..........aaaaguaauugugguuuuu....................................................	1	0	s09
..........aaaaguaauugugguuuuug...................................................	3	0	s09
...........aaaguaauugugguuuuug...................................................	1	0	s09

.........caaaaguaauugugguuu......................................................	1	0	NL2
.........caaaaguaauugugguuuu.....................................................	1	0	NL2
.........caaaaguaauugugguuuuu....................................................	4	0	NL2
.........caaaaguaauugugguuuuugu..................................................	1	0	NL2
..........aaaaguaauugugguuuu.....................................................	10	0	NL2
..........aaaaguaauugugguuuuu....................................................	2	0	NL2
..........aaaaguaauugugguuuuug...................................................	2	0	NL2
..........aaaaguaauugugguuuuugu..................................................	2	0	NL2

....uggugcaaaaguaauuguggu........................................................	1	0	k10
.........caaaaguaauugugguuuuu....................................................	1	0	k10
.........caaaaguaauugugguuuuug...................................................	2	0	k10
.........caaaaguaauugugguuuuugu..................................................	2	0	k10
..........aaaaguaauugugguuuuug...................................................	4	0	k10

.........caaaaguaauugugguuuuug...................................................	1	0	j13
..........aaaaguaauugugguuuu.....................................................	1	0	j13
..........aaaaguaauugugguuuuu....................................................	6	0	j13
..........aaaaguaauugugguuuuug...................................................	33	0	j13
...........aaaguaauugugguuuuug...................................................	1	0	j13

.........caaaaguaauugugguuuuu....................................................	2	0	j19
.........caaaaguaauugugguuuuug...................................................	1	0	j19
.........caaaaguaauugugguuuuugu..................................................	1	0	j19
..........aaaaguaauugugguuuu.....................................................	2	0	j19
..........aaaaguaauugugguuuuu....................................................	5	0	j19
..........aaaaguaauugugguuuuug...................................................	37	0	j19

..........aaaaguaauugugguuuuug...................................................	4	0	j03

....uggugcaaaaguaauuguggu........................................................	1	0	j25
.......ugcaaaaguaauuguggu........................................................	2	0	j25
.........caaaaguaauugugguuuu.....................................................	1	0	j25
..........aaaaguaauugugguuuuug...................................................	10	0	j25

.........caaaaguaauugugguuuuugu..................................................	1	0	k01
..........aaaaguaauugugguuuuug...................................................	1	0	k01
..........aaaaguaauugugguuuuugu..................................................	1	0	k01

........gcaaaaguaauugugguuuuug...................................................	1	0	per
.........caaaaguaauugugguuuuug...................................................	1	0	per
.........caaaaguaauugugguuuuugu..................................................	3	0	per
..........aaaaguaauugugguuuu.....................................................	1	0	per
..........aaaaguaauugugguuuuug...................................................	8	0	per
...........aaaguaauugugguuuuug...................................................	1	0	per
```

```
          Mature                                  Star
caguuggugcaaaaguaauugugguuuuugugauuacuuuuaauuguaaaaaccucaauuacuuuugcaccaaccuaauaa
........................................................aauuacuuuugcaccaaccua....    2    0    per

........gcaaaaguaauugugguuuuug...................................................    1    0    s01

.........caaaaguaauugugguuuuu....................................................    2    0    k04
.........caaaaguaauugugguuuuug...................................................    1    0    k04
.........caaaaguaauugugguuuuugu..................................................    2    0    k04
..........aaaaguaauugugguuuu.....................................................    1    0    k04
..........aaaaguaauugugguuuuug...................................................    5    0    k04
..........aaaaguaauugugguuuuugu..................................................    1    0    k04
.........................................................auuacuuuugcaccaaccuaau..    1    0    k04

....uggugcaaaaguaauuguggu........................................................    2    0    U20
....uggugcaaaaguaauugugguu.......................................................    1    0    U20
......gugcaaaaguaauugugguuuu.....................................................    1    0    U20
.........caaaaguaauugugguuuuug...................................................    1    0    U20
.........caaaaguaauugugguuuuugu..................................................    1    0    U20
..........aaaaguaauugugguuuuu....................................................    1    0    U20
..........aaaaguaauugugguuuuug...................................................    6    0    U20
..........aaaaguaauugugguuuuugug.................................................    1    0    U20
...........aaaguaauugugguuuuugu..................................................    1    0    U20

.........caaaaguaauugugguuuuu....................................................    2    0    ku2
.........caaaaguaauugugguuuuugu..................................................    2    0    ku2
..........aaaaguaauugugguuuuu....................................................    5    0    ku2
..........aaaaguaauugugguuuuug...................................................    16   0    ku2

.........caaaaguaauugugguuuuu....................................................    1    0    j21
.........caaaaguaauugugguuuuug...................................................    3    0    j21
..........aaaaguaauugugguuuuu....................................................    1    0    j21
..........aaaaguaauugugguuuuug...................................................    14   0    j21
...........aaaguaauugugguuuuug...................................................    3    0    j21

..........aaaaguaauugugguuuuug...................................................    1    0    s11

..........aaaaguaauugugguuuuu....................................................    4    0    j07
..........aaaaguaauugugguuuuug...................................................    2    0    j07
...........aaaguaauugugguuuuug...................................................    1    0    j07
...............................................aaaaaccucaauuacuuuugca............    1    0    j07

.........caaaaguaauugugguuuuug...................................................    2    0    k02
.........caaaaguaauugugguuuuugu..................................................    1    0    k02
..........aaaaguaauugugguuuuu....................................................    2    0    k02

.........caaaaguaauugugguuuuug...................................................    5    0    j14
..........aaaaguaauugugguuuuug...................................................    9    0    j14
..........aaaaguaauugugguuuuugu..................................................    1    0    j14

.........caaaaguaauugugguuuuu....................................................    1    0    j28
..........aaaaguaauugugguuuuu....................................................    3    0    j28
..........aaaaguaauugugguuuuug...................................................    17   0    j28

..........aaaaguaauugugguuuuug...................................................    1    0    s08

.........caaaaguaauugugguuuuugu..................................................    1    0    DLD
..........aaaaguaauugugguuuuu....................................................    2    0    DLD
..........aaaaguaauugugguuuuug...................................................    1    0    DLD

..guuggugcaaaaguaauuguggu........................................................    1    0    k03
..........aaaaguaauugugguuuuug...................................................    5    0    k03

.........caaaaguaauugugguuu......................................................    1    0    NL3
.........caaaaguaauugugguuuu.....................................................    1    0    NL3
.........caaaaguaauugugguuuuu....................................................    4    0    NL3
.........caaaaguaauugugguuuuug...................................................    2    0    NL3
..........aaaaguaauugugguuuu.....................................................    15   0    NL3
..........aaaaguaauugugguuuuu....................................................    6    0    NL3
..........aaaaguaauugugguuuuug...................................................    5    0    NL3
.........................................................auuacuuuugcaccaaccuaa...    1    0    NL3

.........caaaaguaauugugguuu......................................................    1    0    ya2
```

Mature   Star

```
caguuggugcaaaaguaauugugguuuuugugauuacuuuuaauuguaaaaaccucaauuacuuuugcaccaaccuaauaa
.........caaaaguaauugugguuuu.....................................................     6      0     ya2
.........caaaaguaauugugguuuuu....................................................     13     0     ya2
.........caaaaguaauugugguuuuug...................................................     2      0     ya2
..........aaaaguaauugugguuuu.....................................................     24     0     ya2
..........aaaaguaauugugguuuuu....................................................     188    0     ya2
..........aaaaguaauugugguuuuug...................................................     17     0     ya2
..........aaaaguaauugugguuuuugu..................................................     4      0     ya2
...........aaaguaauugugguuuuu....................................................     43     0     ya2
...........aaaguaauugugguuuuug...................................................     4      0     ya2
.......................................................caauuacuuuugcaccaac.......     1      0     ya2
........................................................aauuacuuuugcaccaac.......     1      0     ya2

.........caaaaguaauugugguuuuug...................................................     1      0     s02

.........caaaaguaauugugguuuuug...................................................     3      0     k06
..........aaaaguaauugugguuuuug...................................................     2      0     k06

........gcaaaaguaauugugguuuuug...................................................     1      0     j29
.........caaaaguaauugugguuuuug...................................................     3      0     j29
.........caaaaguaauugugguuuuugu..................................................     2      0     j29
..........aaaaguaauugugguuuuug...................................................     10     0     j29

...uuggugcaaaaguaauugugguu.......................................................     1      0     sh2
......gugcaaaaguaauugugguu.......................................................     1      0     sh2
.........caaaaguaauugugguuuuu....................................................     1      0     sh2
..........aaaaguaauugugguuuuug...................................................     1      0     sh2
..........................................................uuacuuuugcaccaaccua....     1      0     sh2

.....ggugcaaaaguaauugugguuu......................................................     1      0     j01
.......ugcaaaaguaauugugguuuu.....................................................     3      0     j01
.........caaaaguaauugugguuuuug...................................................     1      0     j01
..........aaaaguaauugugguuuuug...................................................     5      0     j01

....uggugcaaaaguaauuguggu........................................................     1      0     MCF
.........caaaaguaauugugguuuuugu..................................................     1      0     MCF
..........aaaaguaauugugguuuuug...................................................     4      0     MCF
........................................................aauuacuuuugcaccaaccuaa...     2      0     MCF
........................................................aauuacuuuugcaccaaccuaau..     3      0     MCF
.........................................................auuacuuuugcaccaaccuaau..     4      0     MCF

..........aaaaguaauugugguuuuug...................................................     2      0     MBM

..........aaaaguaauugugguuuuu....................................................     2      0     NL1
..........aaaaguaauugugguuuuug...................................................     2      0     NL1

.....ggugcaaaaguaauugugguu.......................................................     1      0     k07
.......ugcaaaaguaauugugguuuuu....................................................     1      0     k07
.........caaaaguaauugugguuuuug...................................................     9      0     k07
.........caaaaguaauugugguuuuugu..................................................     3      0     k07
..........aaaaguaauugugguuuuug...................................................     3      0     k07

.......ugcaaaaguaauugugguuuuu....................................................     1      0     sh1
.........caaaaguaauugugguuuuug...................................................     6      0     sh1
..........aaaaguaauugugguuuuug...................................................     4      0     sh1

....uggugcaaaaguaauugugguu.......................................................     1      0     li1
.........caaaaguaauugugguuuuug...................................................     3      0     li1
..........aaaaguaauugugguuuuugug.................................................     2      0     li1

.........caaaaguaauugugguuuuu....................................................     1      0     k13
.........caaaaguaauugugguuuuug...................................................     4      0     k13
.........caaaaguaauugugguuuuugu..................................................     3      0     k13
..........aaaaguaauugugguuuuug...................................................     1      0     k13
........................................................aauuacuuuugcaccaaccuaa...     1      0     k13
..........................................................uuacuuuugcaccaaccuaaua.     1      0     k13

.........caaaaguaauugugguuuu.....................................................     1      0     j20
.........caaaaguaauugugguuuuu....................................................     2      0     j20
.........caaaaguaauugugguuuuug...................................................     7      0     j20
..........aaaaguaauugugguuuu.....................................................     3      0     j20
```

Mature **Star**

```
caguuggugcaaaaguaauugugguuuuugugauuacuuuuaauuguaaaaaccucaauuacuuuugcaccaaccuaauaa
..........aaaaguaauugugguuuuug..................................................  27   0   j20
..........aaaaguaauugugguuuuugug................................................  1    0   j20

........gcaaaaguaauugugguuuuug..................................................  11   0   A54
.........caaaaguaauugugguuuuugu.................................................  1    0   A54
..........aaaaguaauugugguuuuug..................................................  3    0   A54
..................................................aaccucaauuacuuuugcacc..........  1    0   A54

....uggugcaaaaguaauugugguu......................................................  1    0   j30
..........aaaaguaauugugguuuuug..................................................  2    0   j30

.........caaaaguaauugugguuuuu...................................................  8    0   k12
.........caaaaguaauugugguuuuug..................................................  12   0   k12
..........aaaaguaauugugguuuuu...................................................  6    0   k12
..........aaaaguaauugugguuuuug..................................................  18   0   k12
..........aaaaguaauugugguuuuugu.................................................  2    0   k12
```

Provisional ID : hsa_can_1341
Score total : -0.1
Score for star read(s) : -1.3
Score for read counts : 0
Score for mfe : 1.7
Score for randfold :
Score for cons. seed : -0.6
Total read count : 21
Mature read count : 19
Loop read count : 0
Star read count : 2

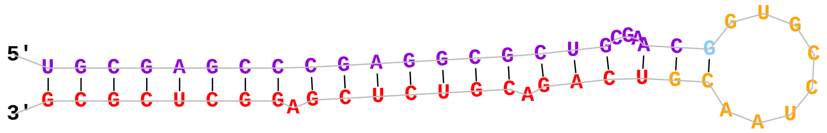

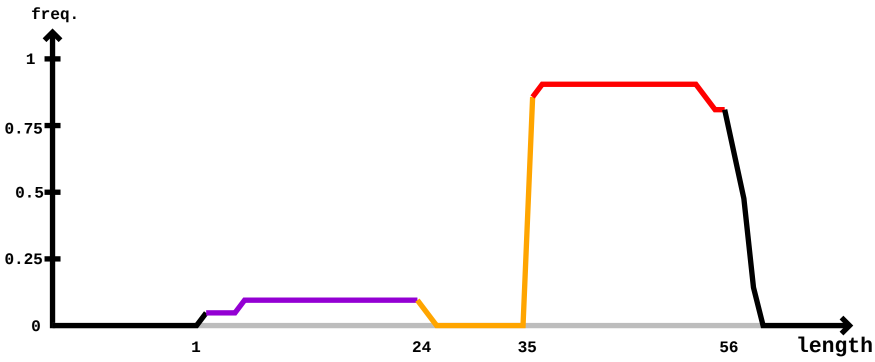


```
                  Star                            Mature
5'- uuucgauccugccgugcgagcccgaggcgcugcgaacggugccuaaacgucagacgucucgaggcucgcguuccauggagga  -3'   obs
    uuucgauccugccgugcgagcccgaggcgcugcgaacggugccuaaacgucagacgucucgaggcucgcguuccauggagga        exp
    ......((((.(((((((((((((((((((((...(((.........)))))).))))))).))))))).....))))))))   reads   mm   sample
    ..................................................cagacgucucgaggcucgcguu..........   1       0    ya1

    .................................................ucagacgucucgaggcucgcg............   1       0    B14

    .................................................ucagacgucucgaggcucgcg............   2       0    j18

    .................................................ucagacgucucgaggcucgcguu..........   1       0    ya2

    .................................................ucagacgucucgaggcucgcguu..........   1       0    ku5

    .................................................ucagacgucucgaggcucgcguuc.........   1       0    k05

    ..............ugcgagcccgaggcgcugcgaac.............................................   1       0    HeL

    .................................................ucagacgucucgaggcucgcg............   2       0    j12

    .................................................ucagacgucucgaggcucgcguu..........   1       0    j19

    .................................................ucagacgucucgaggcucgcguuc.........   1       0    s01

    ..................agcccgaggcgcugcgaacg............................................   1       0    ku2
    .................................................ucagacgucucgaggcuc...............   1       0    ku2

    .................................................ucagacgucucgaggcucgcguu..........   1       0    j21

    .................................................ucagacgucucgaggcucg..............   1       0    j07

    .................................................ucagacgucucgaggcucgcgu...........   1       0    ya4
    .................................................ucagacgucucgaggcucgcguu..........   1       0    ya4

    .................................................ucagacgucucgaggcucgcg............   1       0    k12

    .................................................ucagacgucucgaggcucgcguu..........   1       0    ku1
```

Star Mature

uuucgauccugccgugcgagcccgaggcgcugcgaacggugccuaacgucagacgucucgaggcucgcguuccauggagga

....ucagacgucucgaggcucgcguuc........ 1 0 H52

Provisional ID	: hsa_can_1354	
Score total	:	0.4
Score for star read(s)	:	-1.3
Score for read counts	:	0
Score for mfe	:	2.3
Score for randfold	:	
Score for cons. seed	:	-0.6
Total read count	:	12
Mature read count	:	8
Loop read count	:	0
Star read count	:	4

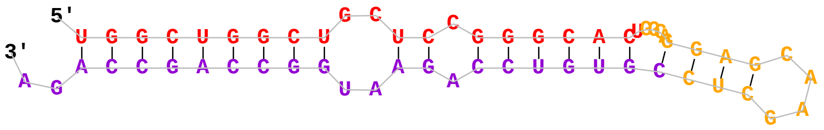

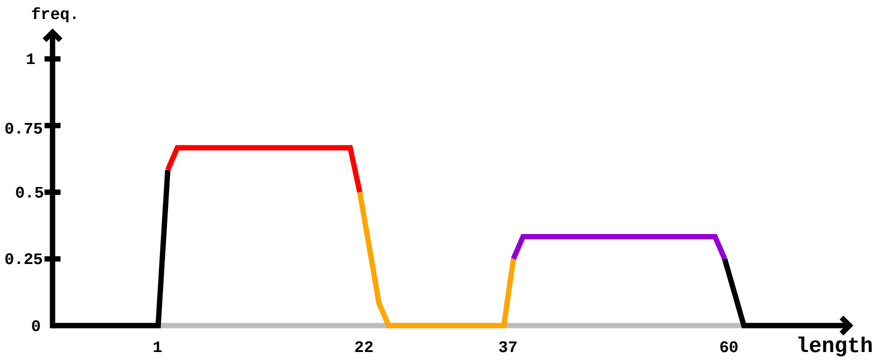

Mature Star

```
5'- gugcgggaucuggcuggcugcuccgggcacuggcaggagcaagcuccguguccagaauggccagccagaucccacacuugc  -3'   obs
    gugcgggaucuggcuggcugcuccgggcacuggcaggagcaagcuccguguccagaauggccagccagaucccacacuugc        exp
    (((.((((((((((((((..((.((((((.....((((....)))))))))).))..)))))))))))))))).)))....      reads      mm         sample
    .........uggcuggcugcuccgggcacu...............................................    2          0          j06

    .........uggcuggcugcuccgggcac................................................    1          0          ya1

    .........uggcuggcugcuccgggcacu...............................................    1          0          li2

    .........................................cguguccagaauggccagccaga...........    1          0          va2

    .........uggcuggcugcuccgggcacu...............................................    1          0          s09

    .........uggcuggcugcuccgggcacu...............................................    1          0          j31

    ..........................................guguccagaauggccagccaga...........    1          0          A54

    .........................................cguguccagaauggccagccag............    1          0          j28
    .........................................cguguccagaauggccagccaga...........    1          0          j28

    ..........ggcuggcugcuccgggcac................................................    1          0          H52

    .........uggcuggcugcuccgggcacugg.............................................    1          0          j22
```

Provisional ID : hsa_can_1359
Score total : -0.2
Score for star read(s) : -1.3
Score for read counts : 0
Score for mfe : 1.6
Score for randfold :
Score for cons. seed : -0.6
Total read count : 31
Mature read count : 31
Loop read count : 0
Star read count : 0

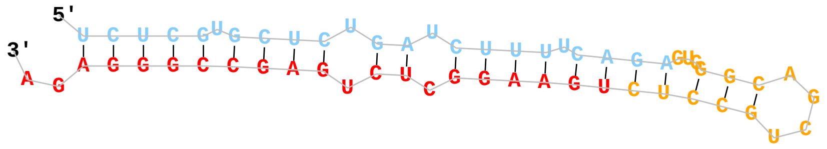

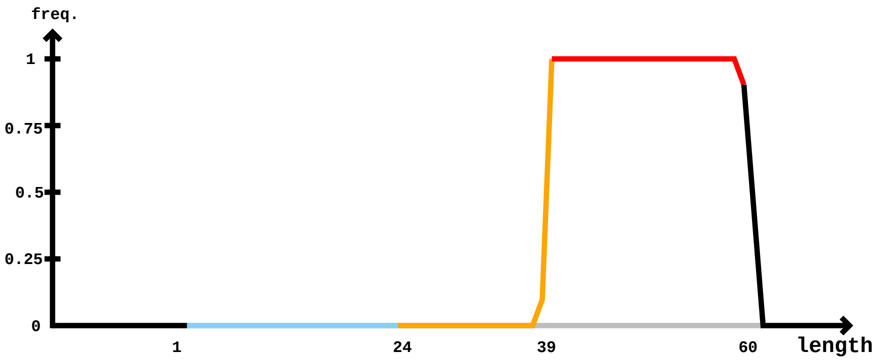


```
                    Star                                      Mature
5'- ccgggcucagugucucgugcucugaucuuuucagagugggcagcugccucugaaggcucugagccgggagacugugcuuuu  -3'   exp
    ..((((.((((.(((((.((((.((.((((.((((...(((....))))))))))).)).))))))))).)))).))))..   reads      mm         sample
    ..................................................ugaaggcucugagccgggaga..........   2          0          s08
    ..................................................ugaaggcucugagccgggaga..........   5          0          j23
    ..................................................ugaaggcucugagccgggaga..........   1          0          ya1
    ..................................................ugaaggcucugagccgggaga..........   3          0          j16
    ..................................................ugaaggcucugagccgggaga..........   1          0          ku3
    ..................................................ugaaggcucugagccgggaga..........   1          0          k11
    ..................................................ugaaggcucugagccgggaga..........   1          0          j17
    ..................................................ugaaggcucugagccgggag...........   1          0          j19
    ..................................................ugaaggcucugagccgggaga..........   1          0          j19
    ..................................................ugaaggcucugagccgggaga..........   1          0          sh1
    ..................................................ugaaggcucugagccgggaga..........   1          0          j25
    ..................................................ugaaggcucugagccgggaga..........   1          0          s01
    .................................................cugaaggcucugagccgggaga..........   3          0          U20
    ..................................................ugaaggcucugagccgggaga..........   3          0          j10
    ..................................................ugaaggcucugagccgggag...........   1          0          j21
    ..................................................ugaaggcucugagccgggaga..........   2          0          j21
    ..................................................ugaaggcucugagccgggag...........   1          0          SW4
    ..................................................ugaaggcucugagccgggaga..........   1          0          ya4
    ..................................................ugaaggcucugagccgggaga..........   1          0          j22
```

Star Mature

ccgggcucagugucucgugcucugaucuuuucagagugggcagcugccucugaaggcucugagccgggagacugugcuuuu

Provisional ID	:	hsa_can_1372
Score total	:	78.1
Score for star read(s)	:	3.9
Score for read counts	:	80.7
Score for mfe	:	-5.8
Score for randfold	:	
Score for cons. seed	:	-0.6
Total read count	:	170
Mature read count	:	169
Loop read count	:	0
Star read count	:	1

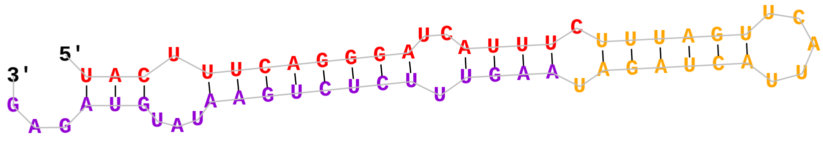

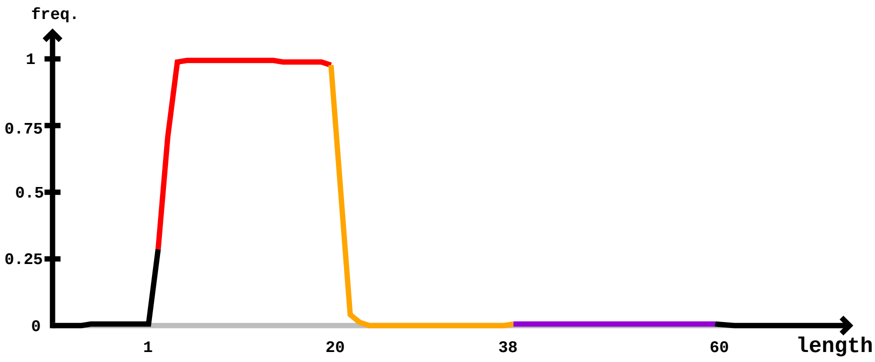

Mature **Star**

```
5'- cuaagacuguacuuucagggaucauuucuuuagguucauuacuagauaaguuucucugaauauguagagcgcugaauuauaa  -3'   obs
    cuaagacuguacuuucagggaucauuucuuuagguucauuacuagauaaguuucucugaauauguagagcgcugaauuauaa         exp
    ....(.((.(((.((((((((..((((.((((((.....)))))).)))).))))))))...))).))).............    reads     mm      sample
    .........uacuuucagggaucauuucu.....................................................    1         0       k09

    .........uacuuucagggaucauuuc......................................................    9         0       DLD
    ..........acuuucagggaucauuuc......................................................    1         0       DLD
    ...........cuuucagggaucauuucu.....................................................    4         0       DLD

    .........uacuuucagggaucauuu.......................................................    2         0       B14
    .........uacuuucagggaucauuuc......................................................    2         0       B14
    ..........acuuucagggaucauuuc......................................................    6         0       B14
    ..........acuuucagggaucauuucuu....................................................    1         0       B14
    ..........acuuucagggaucauuucuuu...................................................    1         0       B14
    ...........cuuucagggaucauuucu.....................................................    7         0       B14

    ..........acuuucagggaucauuuc......................................................    2         0       ya3

    .........uacuuucagggaucauuuc......................................................    4         0       HeL
    ..........acuuucagggaucauuuc......................................................    2         0       HeL
    ..........acuuucagggaucauuucu.....................................................    9         0       HeL
    ..........acuuucagggaucauuucuuu...................................................    1         0       HeL
    ...........cuuucagggaucauuucu.....................................................    10        0       HeL

    ..........acuuucagggaucauuucu.....................................................    2         0       j29

    ..........acuuucagggaucauuucu.....................................................    3         0       sh2

    .........uacuuucagggaucauuuc......................................................    2         0       HEK
    .........uacuuucagggaucauuucu.....................................................    1         0       HEK
    ............uuucagggaucauuucuu....................................................    1         0       HEK

    .........uacuuucagggaucauuuc......................................................    2         0       MCF
    ..........acuuucagggaucauuuc......................................................    2         0       MCF
    ...........cuuucagggaucauuucu.....................................................    3         0       MCF

    ...............................................aaguuucucugaauauguagag.............    1         0       k08
```

Mature **Star**

```
cuaagacuguacuuucagggaucauuucuuuaguucauuacuagauaaguuucucugaauauguagagcgcugaauuauaa

..........acuuucagggaucauuucu....................................................  1   0   MBM
...........cuuucagggaucauuucu....................................................  6   0   MBM

.........uacuuucagggaucauuucu....................................................  1   0   k07

...........cuuucagggaucauuucu....................................................  1   0   li2

.........uacuuucagggaucauuucu....................................................  1   0   k13

..........acuuucagggaucauuuc.....................................................  8   0   U20
..........acuuucagggaucauuucu....................................................  6   0   U20
...........cuuucagggaucauuucu....................................................  1   0   U20

.........uacuuucagggaucauuuc.....................................................  1   0   va3

.........uacuuucagggaucauuuc.....................................................  14  0   A54
..........acuuucagggaucauuuc.....................................................  4   0   A54
..........acuuucagggaucauuucu....................................................  5   0   A54
...........cuuucagggaucauuucu....................................................  7   0   A54

.........uacuuucagggaucauuuc.....................................................  4   0   SW4
..........acuuucagggaucauuuc.....................................................  4   0   SW4
..........acuuucagggaucauuucu....................................................  7   0   SW4
...........cuuucagggaucauuucu....................................................  2   0   SW4
...........cuuucagggaucauuucuu...................................................  1   0   SW4

.........uacuuucagggaucauuucu....................................................  1   0   k12

.........uacuuucagggaucauuuc.....................................................  3   0   H52
..........acuuucagggaucauuuc.....................................................  2   0   H52
..........acuuucagggaucauuucu....................................................  3   0   H52
..........acuuucagggaucauuucuu...................................................  2   0   H52
...........cuuucagggaucauuucu....................................................  6   0   H52

..aagacuguacuuucagggau...........................................................  1   0   ku1
```

Provisional ID	:	hsa_can_1416
Score total	:	0.3
Score for star read(s)	:	-1.3
Score for read counts	:	0
Score for mfe	:	2.2
Score for randfold	:	
Score for cons. seed	:	-0.6
Total read count	:	62
Mature read count	:	59
Loop read count	:	0
Star read count	:	3

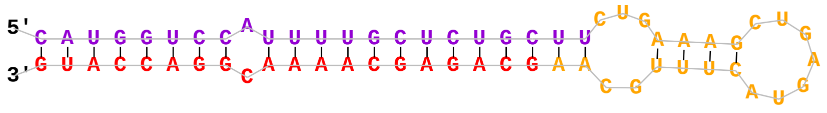

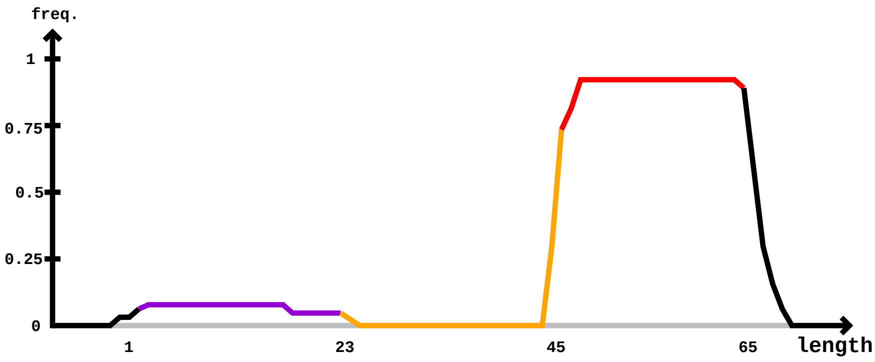

Star **Mature**

```
5'- gguuaugcaugguccauuuugcucugcuucugaaagcugaguacuuugcaagcagagcaaaacggaccaugcauaaccugg  -3'   obs
    gguuaugcaugguccauuuugcucugcuucugaaagcugaguacuuugcaagcagagcaaaacggaccaugcauaaccugg        exp
    ((((((((((((((.(((((((((((((...((((.......))))..))))))))))))).))))))))))))))...    reads   mm   sample
    ....................................................cagagcaaaacggaccaugcau.......    2       0    j18

    ..................................................agcagagcaaaacggaccaug..........    2       0    ku3
    ....................................................cagagcaaaacggaccaug..........    1       0    ku3
    ....................................................cagagcaaaacggaccaugcau.......    1       0    ku3
    .....................................................agagcaaaacggaccaugcaua......    1       0    ku3

    ...................................................gcagagcaaaacggaccaug..........    1       0    ku6
    ...................................................gcagagcaaaacggaccaugc.........    1       0    ku6

    ..................................................agcagagcaaaacggaccaug..........    2       0    ta2

    ........augguccauuuugcucugcuu....................................................    1       0    va1
    ..................................................agcagagcaaaacggaccaug..........    2       0    va1
    ..................................................agcagagcaaaacggaccaugc.........    2       0    va1
    ...................................................gcagagcaaaacggaccau...........    2       0    va1
    ...................................................gcagagcaaaacggaccaug..........    2       0    va1
    ...................................................gcagagcaaaacggaccaugca........    1       0    va1
    .....................................................agagcaaaacggaccaugcau.......    1       0    va1

    .....ugcaugguccauuuugcu..........................................................    1       0    ya3

    ...................................................gcagagcaaaacggaccaug..........    1       0    la1

    ...................................................gcagagcaaaacggaccaug..........    1       0    j15

    ...................................................gcagagcaaaacggaccaug..........    1       0    s10

    ..................................................agcagagcaaaacggaccaug..........    2       0    la2

    ..................................................agcagagcaaaacggaccaug..........    1       0    NL2

    ..................................................agcagagcaaaacggaccaugca........    1       0    ta3
```

Star **Mature**

gguuaugcaugguccauuuugcucugcuucugaaagcugaguacuuugcaagcagagcaaaacggaccaugcauaaccugg

....agcagagcaaaacggaccaug..........	1	0	j13
....gcagagcaaaacggaccaugca........	1	0	j13
....cagagcaaaacggaccaugcaua......	1	0	j13
....agcagagcaaaacggaccaug..........	1	0	j19
....agagcaaaacggaccaugca........	1	0	j03
....gcagagcaaaacggaccaugca........	1	0	s01
.......caugguccauuuugcucugcuu..	1	0	va4
....agcagagcaaaacggaccaugc.........	1	0	va4
....gcagagcaaaacggaccaug..........	1	0	va4
....gcagagcaaaacggaccaugc.........	1	0	va4
....gcagagcaaaacggaccaugca........	1	0	va4
....agcagagcaaaacggaccaug..........	1	0	ku2
....gcagagcaaaacggaccaug..........	2	0	ku2
....agagcaaaacggaccaugcau.......	1	0	ku2
.......caugguccauuuugcucugcuu..	1	0	j21
....gcagagcaaaacggaccaug..........	1	0	j21
....agagcaaaacggaccaugcaua......	2	0	j02
....gcagagcaaaacggaccaugc.........	1	0	j31
....gcagagcaaaacggaccaugca........	2	0	j31
....gcagagcaaaacggaccaug..........	3	0	j07
....agcagagcaaaacggaccaug..........	1	0	va3
....agcagagcaaaacggaccaugc.........	1	0	va3
....agagcaaaacggaccaugcau.......	1	0	va3
....gcagagcaaaacggaccaug..........	1	0	k12
.....ugcaugguccauuuugcu..	1	0	ya4
....agcagagcaaaacggaccaug..........	1	0	ya4
....gcagagcaaaacggaccaugca........	1	0	ya4
....gcagagcaaaacggaccaug..........	2	0	ku1

Provisional ID	:	hsa_can_1424
Score total	:	18.3
Score for star read(s)	:	3.9
Score for read counts	:	13.4
Score for mfe	:	1.6
Score for randfold	:	
Score for cons. seed	:	-0.6
Total read count	:	38
Mature read count	:	29
Loop read count	:	0
Star read count	:	9

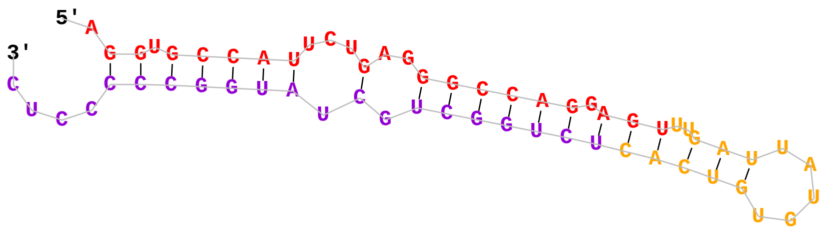

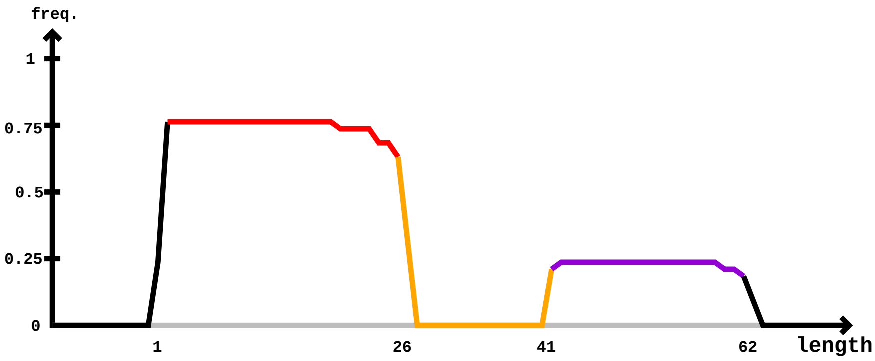

Mature Star

```
5'- uacucugggaaggugccauucugagggccaggaguuugauuaugugucacucuggcugcuauggcccccucccagggucug  -3'   obs
    uacucugggaaggugccauucugagggccaggaguuugauuaugugucacucuggcugcuauggcccccucccagggucug        exp
    .(((((((((.((.(((((...(..((((((.(((..(((.....)))))))))))).).))))).)).)))))))))...    reads      mm         sample
    ..........aggugccauucugagggccagg.................................................    1          0          k09
    ..........aggugccauucugagggccaggagu..............................................    1          0          k09

    .........aaggugccauucugagggccaggagu..............................................    1          0          k10
    .........aaggugccauucugagggccaggaguu.............................................    1          0          k10
    ..........aggugccauucugagggccaggagu..............................................    2          0          k10
    ..........aggugccauucugagggccaggaguu.............................................    1          0          k10

    ..........aggugccauucugagggccaggag...............................................    1          0          k13
    ..........aggugccauucugagggccaggagu..............................................    1          0          k13

    ..........aggugccauucugagggccaggagu..............................................    1          0          k04

    .........aaggugccauucugagggccaggagu..............................................    2          0          k11

    .........aaggugccauucugagggccaggagu..............................................    1          0          k06
    ..........aggugccauucugagggccaggagu..............................................    1          0          k06

    ..................................................ucuggcugcuauggcccccuc..........    1          0          HeL

    .........aaggugccauucugagggccaggagu..............................................    1          0          k02
    ..........aggugccauucugagggccaggagu..............................................    2          0          k02
    ..................................................ucuggcugcuauggcccccuc..........    1          0          k02

    .........aaggugccauucugagggc.....................................................    1          0          sh2
    .........aaggugccauucugagggccagg.................................................    1          0          sh2
    .........aaggugccauucugagggccaggagu..............................................    1          0          sh2
    ..........aggugccauucugagggccaggagu..............................................    2          0          sh2
    ..................................................ucuggcugcuauggcccccuc..........    2          0          sh2
    ..................................................ucuggcugcuauggcccccucc.........    1          0          sh2
    ...................................................cuggcugcuauggcccccuc..........    1          0          sh2

    ..........aggugccauucugagggccaggag...............................................    1          0          s10
```

Mature Star

```
uacucugggaaggugccauucugagggccaggaguuugauuaugugucacucuggcugcuauggcccccucccagggucug
..........aggugccauucugagggccaggagu.............................................   6   0   s10
..................................................ucuggcugcuauggcccc.............   1   0   s10
..................................................ucuggcugcuauggcccccu...........   1   0   s10
..................................................ucuggcugcuauggcccccuc..........   1   0   s10
```

Provisional ID	:	hsa_can_1444
Score total	:	561.3
Score for star read(s)	:	3.9
Score for read counts	:	553.3
Score for mfe	:	1.1
Score for randfold	:	
Score for cons. seed	:	3
Total read count	:	1097
Mature read count	:	1064
Loop read count	:	0
Star read count	:	33

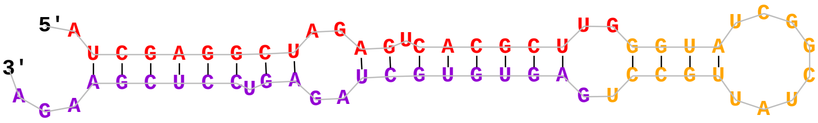

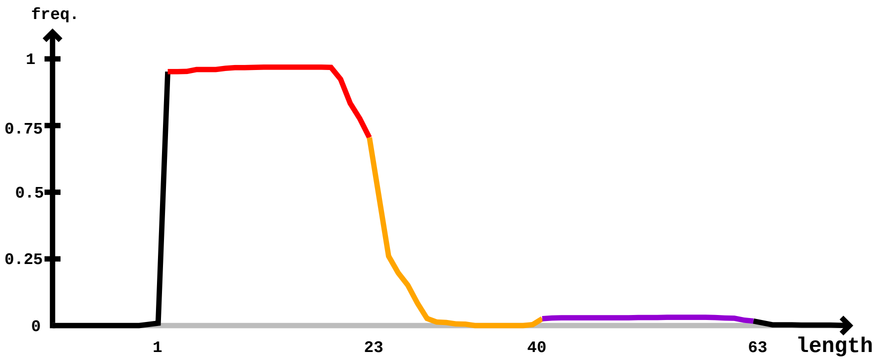

Mature **Star**

```
5'- agaaccugucaucgaggcuagagucacgcuuggguaucggcuauugccugagugugcuagaguccucgaagaguaacugcu  -3'    obs
    agaaccugucaucgaggcuagagucacgcuuggguaucggcuauugccugagugugcuagaguccucgaagaguaacugcu         exp
    ........((.((((((((..((.((((((..((((........))))..))))))))..)).)))))).)).........    reads     mm       sample
    ..........aucgaggcuagagucacg.....................................................    1         0        ya1
    ..........aucgaggcuagagucacgc....................................................    1         0        ya1
    ..........aucgaggcuagagucacgcu...................................................    1         0        ya1
    ..........aucgaggcuagagucacgcuug.................................................    2         0        ya1
    ..........aucgaggcuagagucacgcuugggua.............................................    2         0        ya1
    ..........aucgaggcuagagucacgcuuggguau............................................    1         0        ya1
    .................................................gagugugcuagaguccucgaaga.........    1         0        ya1

    ..........aucgaggcuagagucacg.....................................................    1         0        j26
    ..........aucgaggcuagagucacgcuugg................................................    4         0        j26
    ..........aucgaggcuagagucacgcuuggg...............................................    2         0        j26
    ..........aucgaggcuagagucacgcuugggua.............................................    1         0        j26
    ..........aucgaggcuagagucacgcuuggguau............................................    2         0        j26
    .................................................gagugugcuagaguccucgaa...........    3         0        j26

    ..........aucgaggcuagagucacgcuu..................................................    2         0        va1
    ..........aucgaggcuagagucacgcuugggua.............................................    1         0        va1
    ..........aucgaggcuagagucacgcuuggguau............................................    2         0        va1
    ...........................................................gaguccucgaagaguaacugc.    1         0        va1

    .........caucgaggcuagagucacgcuugggu..............................................    1         0        ku5
    ..........aucgaggcuagagucacgcu...................................................    1         0        ku5
    ..........aucgaggcuagagucacgcuu..................................................    1         0        ku5
    ..........aucgaggcuagagucacgcuugg................................................    3         0        ku5
    ..........aucgaggcuagagucacgcuugggu..............................................    1         0        ku5
    ..........aucgaggcuagagucacgcuugggua.............................................    2         0        ku5
    .................................................gagugugcuagaguccucgaag..........    1         0        ku5

    ..........aucgaggcuagagucacgcuugg................................................    1         0        k05

    ..........aucgaggcuagagucacgcuugg................................................    3         0        s06
    .............gaggcuagagucacgcuugg................................................    1         0        s06

    ..........aucgaggcuagagucacg.....................................................    2         0        HeL
```

Mature **Star**

```
agaaccugucaucgaggcuagagucacgcuuggguaucggcuauugccugagugugcuagaguccucgaagaguaacugcu
..........aucgaggcuagagucacgc....................................................   2   0   HeL
..........aucgaggcuagagucacgcu...................................................   1   0   HeL
..........aucgaggcuagagucacgcuug.................................................   3   0   HeL
..........aucgaggcuagagucacgcuugg................................................   3   0   HeL
..........aucgaggcuagagucacgcuuggg...............................................   2   0   HeL
..........aucgaggcuagagucacgcuugggua.............................................   3   0   HeL
..........aucgaggcuagagucacgcuuggguau............................................   1   0   HeL
..........aucgaggcuagagucacgcuuggguaucgg........................................   2   0   HeL
.............gaggcuagagucacgcuugggu..............................................   1   0   HeL
.................cuagagucacgcuugggu..............................................   1   0   HeL

..........aucgaggcuagagucacgcuugg................................................   1   0   ku4
..........aucgaggcuagagucacgcuuggg...............................................   1   0   ku4
..........aucgaggcuagagucacgcuugggua.............................................   4   0   ku4
..........aucgaggcuagagucacgcuuggguauc...........................................   1   0   ku4

..........aucgaggcuagagucacgcuugg................................................   5   0   s10
..........aucgaggcuagagucacgcuuggg...............................................   1   0   s10
..........aucgaggcuagagucacgcuugggua.............................................   1   0   s10

..........aucgaggcuagagucacgcuug.................................................   1   0   ta3
..........aucgaggcuagagucacgcuugg................................................   1   0   ta3
..........aucgaggcuagagucacgcuuggguauc...........................................   1   0   ta3
..............................................................uccucgaagaguaacugcu   1   0   ta3

..........aucgaggcuagagucacgc....................................................   1   0   j10
..........aucgaggcuagagucacgcuug.................................................   1   0   j10

..........aucgaggcuagagucacgc....................................................   2   0   j04
..........aucgaggcuagagucacgcuug.................................................   4   0   j04

..........aucgaggcuagagucacgcuugg................................................   1   0   va2

..........aucgaggcuagagucacgcuu..................................................   1   0   k14
..........aucgaggcuagagucacgcuug.................................................   1   0   k14
..........aucgaggcuagagucacgcuugg................................................   1   0   k14

..........aucgaggcuagagucacgcuu..................................................   1   0   j02
..........aucgaggcuagagucacgcuug.................................................   2   0   j02

..........aucgaggcuagagucacg.....................................................   2   0   j27
..........aucgaggcuagagucacgcu...................................................   1   0   j27
..........aucgaggcuagagucacgcuug.................................................   1   0   j27
..........aucgaggcuagagucacgcuugg................................................   1   0   j27
..........aucgaggcuagagucacgcuugggu..............................................   1   0   j27
..........aucgaggcuagagucacgcuugggua.............................................   2   0   j27
..........aucgaggcuagagucacgcuuggguau............................................   3   0   j27
.................................................gagugugcuagaguccucgaa...........   1   0   j27

..........aucgaggcuagagucacgcuugg................................................   2   0   s03
..........aucgaggcuagagucacgcuugggua.............................................   3   0   s03

..........aucgaggcuagagucacgcuugg................................................   6   0   H52
..........aucgaggcuagagucacgcuuggguaucggcu......................................   1   0   H52

..........aucgaggcuagagucacgcuug.................................................   3   0   k09
..........aucgaggcuagagucacgcuugg................................................   1   0   k09

..........aucgaggcuagagucacg.....................................................   1   0   B14
..........aucgaggcuagagucacgcu...................................................   1   0   B14
..........aucgaggcuagagucacgcuugg................................................   1   0   B14
..........aucgaggcuagagucacgcuuggguauc...........................................   1   0   B14
.............gaggcuagagucacgcuu..................................................   1   0   B14
....................gagucacgcuuggguauc...........................................   1   0   B14

..........aucgaggcuagagucacgcuug.................................................   1   0   j16

..........aucgaggcuagagucacgcuu..................................................   1   0   ku3
..........aucgaggcuagagucacgcuug.................................................   2   0   ku3
..........aucgaggcuagagucacgcuugg................................................   3   0   ku3
```

Mature

Star

```
agaaccugucaucgaggcuagagucacgcuugggguaucggcuauugccugagugugcuagaguccucgaagaguaacugcu
..........aucgaggcuagagucacgcuuggg...............................................    1    0    ku3
..........aucgaggcuagagucacgcuugggu..............................................    1    0    ku3
..........aucgaggcuagagucacgcuugggua.............................................    2    0    ku3
..........aucgaggcuagagucacgcuuggguauc...........................................    1    0    ku3

..........aucgaggcuagagucacg.....................................................    5    0    j18
..........aucgaggcuagagucacgc....................................................    3    0    j18
..........aucgaggcuagagucacgcuu..................................................    3    0    j18
..........aucgaggcuagagucacgcuug.................................................    5    0    j18
..........aucgaggcuagagucacgcuugg................................................    6    0    j18
..........aucgaggcuagagucacgcuuggg...............................................    6    0    j18
..........aucgaggcuagagucacgcuugggu..............................................    2    0    j18
..........aucgaggcuagagucacgcuugggua.............................................    2    0    j18
..........aucgaggcuagagucacgcuuggguau............................................    5    0    j18
..........aucgaggcuagagucacgcuuggguauc...........................................    2    0    j18
................gcuagagucacgcuuggguaucggcu.......................................    5    0    j18
.................................................gagugugcuagaguccucgaa...........    1    0    j18
.................................................gagugugcuagaguccucgaag..........    2    0    j18
.................................................gagugugcuagaguccucgaaga.........    1    0    j18

..........aucgaggcuagagucacgc....................................................    1    0    j09
..........aucgaggcuagagucacgcuu..................................................    1    0    j09
..........aucgaggcuagagucacgcuug.................................................    1    0    j09
..........aucgaggcuagagucacgcuugg................................................    1    0    j09
............cgaggcuagagucacgcuug.................................................    1    0    j09
...................................................gugugcuagaguccucgaagaguaa.....    1    0    j09

..........aucgaggcuagagucacgcuugggua.............................................    1    0    ku6

..........aucgaggcuagagucacgcuug.................................................    6    0    j11
..........aucgaggcuagagucacgcuugg................................................    3    0    j11

..........aucgaggcuagagucacgc....................................................    2    0    s12
..........aucgaggcuagagucacgcu...................................................    3    0    s12
..........aucgaggcuagagucacgcuug.................................................    1    0    s12
..........aucgaggcuagagucacgcuugg................................................    5    0    s12
..........aucgaggcuagagucacgcuuggg...............................................    1    0    s12

..........aucgaggcuagagucacgc....................................................    1    0    ya3
..........aucgaggcuagagucacgcu...................................................    3    0    ya3
..........aucgaggcuagagucacgcuug.................................................    2    0    ya3
..........aucgaggcuagagucacgcuuggguauc...........................................    1    0    ya3
.............gaggcuagagucacgcuu..................................................    2    0    ya3
................................................ugagugugcuagaguccuc..............    1    0    ya3

..........aucgaggcuagagucacgc....................................................    1    0    HEK
..........aucgaggcuagagucacgcuug.................................................    2    0    HEK
..........aucgaggcuagagucacgcuugg................................................    1    0    HEK
..........aucgaggcuagagucacgcuuggguaucgg.........................................    1    0    HEK

..........aucgaggcuagagucacgc....................................................    1    0    la1
..........aucgaggcuagagucacgcu...................................................    1    0    la1
..........aucgaggcuagagucacgcuugg................................................    3    0    la1
..........aucgaggcuagagucacgcuugggu..............................................    1    0    la1
..........aucgaggcuagagucacgcuugggua.............................................    1    0    la1
..........aucgaggcuagagucacgcuuggguau............................................    2    0    la1
...................agagucacgcuuggguau............................................    1    0    la1

..........aucgaggcuagagucacgcuug.................................................    1    0    j17
..........aucgaggcuagagucacgcuugg................................................    4    0    j17
.................................................gagugugcuagaguccucgaaga.........    2    0    j17

..........aucgaggcuagagucacgc....................................................    1    0    j15
..........aucgaggcuagagucacgcuug.................................................    1    0    j15
..........aucgaggcuagagucacgcuugg................................................    2    0    j15

..........aucgaggcuagagucacgcuug.................................................    1    0    la2

..........aucgaggcuagagucacg.....................................................    1    0    j08
..........aucgaggcuagagucacgcuug.................................................    1    0    j08
```

```
          Mature                                 Star
agaaccugucaucgaggcuagagucacgcuuggguaucggcuauugccugagugugcuagaguccucgaagaguaacugcu

..........aucgaggcuagagucacgcuu..................................................    1      0      j12
..........aucgaggcuagagucacgcuugg................................................    1      0      j12
..........aucgaggcuagagucacgcuuggg...............................................    1      0      j12
..........aucgaggcuagagucacgcuuggguaucgg.........................................    1      0      j12

..........aucgaggcuagagucacgcuug.................................................    1      0      li2
..........aucgaggcuagagucacgcuugg................................................    6      0      li2
..........aucgaggcuagagucacgcuugggu..............................................    1      0      li2
..........aucgaggcuagagucacgcuugggua.............................................    1      0      li2
..........aucgaggcuagagucacgcuuggguaucggc........................................    1      0      li2
.................................................gagugugcuagaguccucgaaga.........    1      0      li2

..........aucgaggcuagagucacg.....................................................    2      0      va4
..........aucgaggcuagagucacgc....................................................    1      0      va4
..........aucgaggcuagagucacgcu...................................................    2      0      va4
..........aucgaggcuagagucacgcuug.................................................    4      0      va4
..........aucgaggcuagagucacgcuugg................................................    10     0      va4
..........aucgaggcuagagucacgcuuggg...............................................    1      0      va4
..........aucgaggcuagagucacgcuugggua.............................................    2      0      va4
..........aucgaggcuagagucacgcuuggguau............................................    10     0      va4

.........caucgaggcuagagucacgc....................................................    1      0      va3
.........caucgaggcuagagucacgcu...................................................    1      0      va3
.........caucgaggcuagagucacgcuugg................................................    1      0      va3
..........aucgaggcuagagucacg.....................................................    26     0      va3
..........aucgaggcuagagucacgc....................................................    46     0      va3
..........aucgaggcuagagucacgcu...................................................    32     0      va3
..........aucgaggcuagagucacgcuu..................................................    27     0      va3
..........aucgaggcuagagucacgcuug.................................................    105    0      va3
..........aucgaggcuagagucacgcuugg................................................    85     0      va3
..........aucgaggcuagagucacgcuuggg...............................................    26     0      va3
..........aucgaggcuagagucacgcuugggu..............................................    19     0      va3
..........aucgaggcuagagucacgcuugggua.............................................    18     0      va3
..........aucgaggcuagagucacgcuuggguau............................................    15     0      va3
..........aucgaggcuagagucacgcuuggguauc...........................................    2      0      va3

..........aucgaggcuagagucacgcuugggu..............................................    2      0      SW4
..........aucgaggcuagagucacgcuuggguauc...........................................    2      0      SW4

..........aucgaggcuagagucacgc....................................................    2      0      ya4
..........aucgaggcuagagucacgcu...................................................    1      0      ya4
..........aucgaggcuagagucacgcuu..................................................    4      0      ya4
..........aucgaggcuagagucacgcuug.................................................    1      0      ya4
..........aucgaggcuagagucacgcuugggua.............................................    1      0      ya4
..................................................agugugcuagaguccucgaaga.........    1      0      ya4

..........aucgaggcuagagucacgc....................................................    1      0      ku1
..........aucgaggcuagagucacgcuu..................................................    1      0      ku1
..........aucgaggcuagagucacgcuug.................................................    1      0      ku1
..........aucgaggcuagagucacgcuugg................................................    2      0      ku1
..........aucgaggcuagagucacgcuuggg...............................................    1      0      ku1
..........aucgaggcuagagucacgcuugggua.............................................    2      0      ku1
..........aucgaggcuagagucacgcuuggguau............................................    2      0      ku1
..........aucgaggcuagagucacgcuuggguaucgg.........................................    1      0      ku1
.................................................gagugugcuagaguccucgaa...........    1      0      ku1

..........aucgaggcuagagucacgcuugg................................................    1      0      j23
.................................................gagugugcuagaguccucgaaga.........    1      0      j23

.................................................gagugugcuagaguccucgaa...........    1      0      s05
.................................................gagugugcuagaguccucgaaga.........    1      0      s05

..........aucgaggcuagagucacgcuug.................................................    1      0      ta2
..........aucgaggcuagagucacgcuugggua.............................................    1      0      ta2

..........aucgaggcuagagucacgc....................................................    1      0      j24
..........aucgaggcuagagucacgcu...................................................    1      0      j24
..........aucgaggcuagagucacgcuu..................................................    1      0      j24
..........aucgaggcuagagucacgcuug.................................................    1      0      j24
```

Mature Star

```
agaaccugucaucgaggcuagagucacgcuuggguaucggcuauugccugagugugcuagaguccucgaagaguaacugcu
..........aucgaggcuagagucacgcuugg................................................     2     0     j24

..........aucgaggcuagagucacg.....................................................     6     0     ta1
..........aucgaggcuagagucacgc....................................................     7     0     ta1
..........aucgaggcuagagucacgcu...................................................     4     0     ta1
..........aucgaggcuagagucacgcuu..................................................     5     0     ta1
..........aucgaggcuagagucacgcuug.................................................     13    0     ta1
..........aucgaggcuagagucacgcuugg................................................     11    0     ta1
..........aucgaggcuagagucacgcuuggg...............................................     1     0     ta1
..........aucgaggcuagagucacgcuugggu..............................................     1     0     ta1
.................................................gagugugcuagaguccucgaaga.........     1     0     ta1
.................................................gagugugcuagaguccucgaagag........     1     0     ta1

..........aucgaggcuagagucacgcuu..................................................     1     0     s09
..........aucgaggcuagagucacgcuug.................................................     1     0     s09
..........aucgaggcuagagucacgcuugg................................................     7     0     s09
..........aucgaggcuagagucacgcuugggu..............................................     1     0     s09

..........aucgaggcuagagucacgcuugggu..............................................     1     0     NL2
..........aucgaggcuagagucacgcuuggguau............................................     1     0     NL2

..........aucgaggcuagagucacgc....................................................     1     0     j13
..........aucgaggcuagagucacgcuu..................................................     1     0     j13
..........aucgaggcuagagucacgcuug.................................................     2     0     j13
..........aucgaggcuagagucacgcuugg................................................     1     0     j13
..........aucgaggcuagagucacgcuuggg...............................................     1     0     j13

..........aucgaggcuagagucacgcu...................................................     4     0     j19
..........aucgaggcuagagucacgcuu..................................................     1     0     j19
..........aucgaggcuagagucacgcuug.................................................     1     0     j19
..........aucgaggcuagagucacgcuugg................................................     1     0     j19
..........aucgaggcuagagucacgcuuggg...............................................     1     0     j19

..........aucgaggcuagagucacgcuu..................................................     1     0     j03
.............gaggcuagagucacgcuugg................................................     2     0     j03

.........caucgaggcuagagucac......................................................     1     0     j25
..........aucgaggcuagagucacgc....................................................     2     0     j25
..........aucgaggcuagagucacgcuu..................................................     1     0     j25
..........aucgaggcuagagucacgcuug.................................................     1     0     j25
..........aucgaggcuagagucacgcuuggg...............................................     2     0     j25
..........aucgaggcuagagucacgcuugggua.............................................     4     0     j25

..........aucgaggcuagagucacgcu...................................................     1     0     per
..........aucgaggcuagagucacgcuugg................................................     1     0     per
.................................................gagugugcuagaguccucgaaga.........     2     0     per

..........aucgaggcuagagucacgcuug.................................................     1     0     s01
..........aucgaggcuagagucacgcuugg................................................     3     0     s01
..........aucgaggcuagagucacgcuugggua.............................................     1     0     s01
..........aucgaggcuagagucacgcuuggguau............................................     2     0     s01

..........aucgaggcuagagucacgcuuggg...............................................     3     0     U20

..........aucgaggcuagagucacgc....................................................     1     0     ku2
..........aucgaggcuagagucacgcuugg................................................     1     0     ku2

..........aucgaggcuagagucacgcuu..................................................     2     0     j21
..........aucgaggcuagagucacgcuugg................................................     2     0     j21

..........aucgaggcuagagucacgcuu..................................................     1     0     s11
..........aucgaggcuagagucacgcuugg................................................     2     0     s11
..........aucgaggcuagagucacgcuugggu..............................................     1     0     s11

..........aucgaggcuagagucacgcuuggguau............................................     1     0     j07

..........aucgaggcuagagucacg.....................................................     2     0     j28
..........aucgaggcuagagucacgc....................................................     15    0     j28
..........aucgaggcuagagucacgcu...................................................     3     0     j28
..........aucgaggcuagagucacgcuu..................................................     10    0     j28
```

Mature   Star

```
agaaccugucaucgaggcuagagucacgcuuggguaucggcuauugccugagugugcuagaguccucgaagaguaacugcu
..........aucgaggcuagagucacgcuug.................................................    14    0    j28
..........aucgaggcuagagucacgcuugg................................................    48    0    j28
..........aucgaggcuagagucacgcuuggg...............................................    11    0    j28
..........aucgaggcuagagucacgcuugggu..............................................    8     0    j28
..........aucgaggcuagagucacgcuuggua..............................................    7     0    j28
..........aucgaggcuagagucacgcuuggguau............................................    11    0    j28
..........aucgaggcuagagucacgcuuggguauc...........................................    2     0    j28
.................................................gagugugcuagaguccucga............    1     0    j28

..........aucgaggcuagagucacgc....................................................    2     0    s08
..........aucgaggcuagagucacgcuu..................................................    1     0    s08
..........aucgaggcuagagucacgcuug.................................................    1     0    s08
..........aucgaggcuagagucacgcuugg................................................    8     0    s08
..........aucgaggcuagagucacgcuuggg...............................................    4     0    s08

..........aucgaggcuagagucacgcuug.................................................    1     0    DLD
..........aucgaggcuagagucacgcuugg................................................    3     0    DLD
..........aucgaggcuagagucacgcuugggua.............................................    1     0    DLD
..................................................agugugcuagaguccucgaaga.........    1     0    DLD

..........aucgaggcuagagucacgcuugg................................................    1     0    k03

..........aucgaggcuagagucacgcuuggguau............................................    1     0    NL3

..........aucgaggcuagagucacgcuugggua.............................................    1     0    ya2
................................................ugagugugcuagaguccucgaa...........    1     0    ya2

..........aucgaggcuagagucacgcuugg................................................    1     0    s02

........ucaucgaggcuagagucacgcuugg................................................    5     0    sh2
..........aucgaggcuagagucacgc....................................................    2     0    sh2
..........aucgaggcuagagucacgcu...................................................    4     0    sh2
..........aucgaggcuagagucacgcuu..................................................    6     0    sh2
..........aucgaggcuagagucacgcuug.................................................    5     0    sh2
..........aucgaggcuagagucacgcuugg................................................    15    0    sh2
..........aucgaggcuagagucacgcuugggu..............................................    4     0    sh2
..........aucgaggcuagagucacgcuugggua.............................................    3     0    sh2
................................................ugagugugcuagaguccucgaag..........    1     0    sh2
.................................................gagugugcuagaguccucg.............    2     0    sh2
..................................................agugugcuagaguccucgaaga.........    1     0    sh2

..........aucgaggcuagagucacgcuug.................................................    1     0    j29
..........aucgaggcuagagucacgcuugg................................................    2     0    j29
..........aucgaggcuagagucacgcuuggg...............................................    1     0    j29
..........aucgaggcuagagucacgcuugggu..............................................    2     0    j29
..........aucgaggcuagagucacgcuugggua.............................................    3     0    j29
..........aucgaggcuagagucacgcuuggguau............................................    3     0    j29

..........aucgaggcuagagucacgcuugg................................................    1     0    MCF

..........aucgaggcuagagucacgc....................................................    1     0    MBM
..........aucgaggcuagagucacgcuug.................................................    1     0    MBM
..........aucgaggcuagagucacgcuugg................................................    1     0    MBM
..........aucgaggcuagagucacgcuuggg...............................................    1     0    MBM
..........aucgaggcuagagucacgcuugggua.............................................    3     0    MBM
..........aucgaggcuagagucacgcuuggguau............................................    1     0    MBM
..........aucgaggcuagagucacgcuuggguaucgg.........................................    1     0    MBM
.................cuagagucacgcuugggu..............................................    1     0    MBM
.................cuagagucacgcuugggua.............................................    1     0    MBM

..........aucgaggcuagagucacg.....................................................    1     0    NL1
..........aucgaggcuagagucacgc....................................................    1     0    NL1
..........aucgaggcuagagucacgcuug.................................................    2     0    NL1
..........aucgaggcuagagucacgcuugg................................................    2     0    NL1
..........aucgaggcuagagucacgcuugggu..............................................    1     0    NL1
..........aucgaggcuagagucacgcuuggguau............................................    1     0    NL1

..........aucgaggcuagagucacgcuugg................................................    1     0    k07
.................................................gagugugcuagaguccucgaaga.........    1     0    k07

..........aucgaggcuagagucacgcuug.................................................    2     0    sh1
```

```
          Mature                                 Star
agaaccugucaucgaggcuagagucacgcuuggguaucggcuauugccugagugugcuagaguccucgaagaguaacugcu
..........aucgaggcuagagucacgcuugg................................................    6    0    sh1
..........aucgaggcuagagucacgcuugggua.............................................    2    0    sh1

..........aucgaggcuagagucacgc....................................................    2    0    li1
..........aucgaggcuagagucacgcuuggg...............................................    1    0    li1
..........aucgaggcuagagucacgcuuggguauc...........................................    1    0    li1
..........aucgaggcuagagucacgcuuggguaucg..........................................    2    0    li1
................................................ugagugugcuagaguccucgaaga.........    1    0    li1

..........aucgaggcuagagucacgcuug.................................................    1    0    k13
..........aucgaggcuagagucacgcuugg................................................    1    0    k13
..........aucgaggcuagagucacgcuuggg...............................................    1    0    k13

..........aucgaggcuagagucacgcuuggg...............................................    1    0    j20

..........aucgaggcuagagucacgcuu..................................................    3    0    A54
.............gaggcuagagucacgcuugggu..............................................    1    0    A54

..........aucgaggcuagagucacgcuugg................................................    1    0    j30
```

Provisional ID	:	hsa_can_1447
Score total	:	10.5
Score for star read(s)	:	3.9
Score for read counts	:	6.8
Score for mfe	:	0.5
Score for randfold	:	
Score for cons. seed	:	-0.6
Total read count	:	25
Mature read count	:	19
Loop read count	:	0
Star read count	:	6

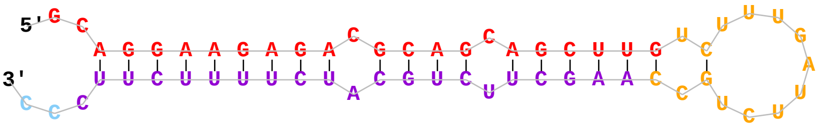

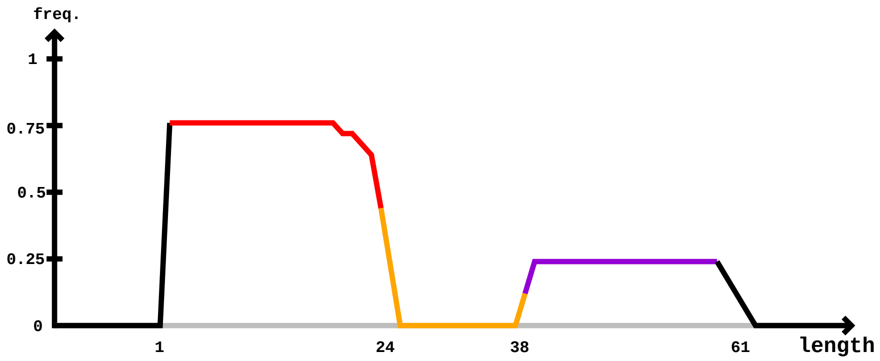

Mature **Star**

```
5'- agagacuaaggcaggaagagacgcagcagcuugucuuugauucugccaagcuucugcaucuuuucuucccuagcccuuagc  -3'   obs
    agagacuaaggcaggaagagacgcagcagcuugucuuugauucugccaagcuucugcaucuuuucuucccuagcccuuagc        exp
    .(((.(((.((.(((((((((.((((.((((((.(.........).)))))).)))).)))))))))))).))).)))...    reads    mm    sample
    ..........gcaggaagagacgcagcagcuu.................................................    1        0     ya2
    ..........gcaggaagagacgcagcagcuug................................................    2        0     ya2

    ..........gcaggaagagacgcagcagcuu.................................................    1        0     va1
    ..........gcaggaagagacgcagcagcuug................................................    1        0     va1
    ..........gcaggaagagacgcagcagcuugu...............................................    1        0     va1

    ...............................................aagcuucugcaucuuuucuuc.............    3        0     U20
    ................................................agcuucugcaucuuuucuuc.............    1        0     U20
    ................................................agcuucugcaucuuuucuucccu..........    2        0     U20

    ..........gcaggaagagacgcagcagcuug................................................    2        0     va3

    ..........gcaggaagagacgcagcagcuu.................................................    2        0     j27

    ..........gcaggaagagacgcagca.....................................................    1        0     la1

    ..........gcaggaagagacgcagcagc...................................................    1        0     ya4
    ..........gcaggaagagacgcagcagcu..................................................    1        0     ya4

    ..........gcaggaagagacgcagcagcuu.................................................    1        0     s03

    ..........gcaggaagagacgcagcagcuug................................................    1        0     la2

    ..........gcaggaagagacgcagcagcuug................................................    3        0     NL1
    ..........gcaggaagagacgcagcagcuugu...............................................    1        0     NL1
```

Provisional ID	:	hsa_can_1451
Score total	:	1825
Score for star read(s)	:	3.9
Score for read counts	:	1816.1
Score for mfe	:	2
Score for randfold	:	
Score for cons. seed	:	3
Total read count	:	3574
Mature read count	:	3542
Loop read count	:	0
Star read count	:	32

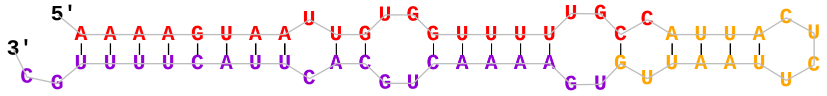

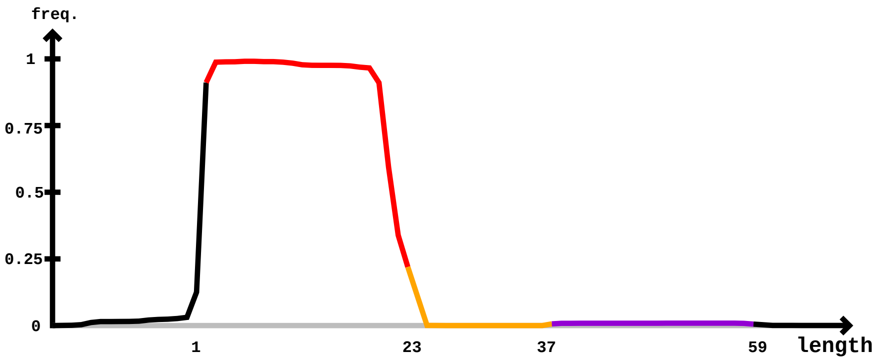

Mature **Star**

```
5'- uauuagguuggugcaaaaguaauugugguuuuugccauuacucuuaauugugaaaacugcacuuacuuuugcaccaaccua  -3'   obs
    uauuagguuggugcaaaaguaauugugguuuuugccauuacucuuaauugugaaaacugcacuuacuuuugcaccaaccua        exp
    uauuagguuggugcaaaaguaauugugguuuuugccauuacucuuaauugugaaaacugcacuuacuuuugcaccaaccua        known
    ...(((((((((((((((((((.((..(((((..(.((((....)))).)..)))))..)).)))))))))))))))))))    reads   mm   sample
    uauuagguuggugcaaaagu.............................................................    2       0    ya1
    ...uagguuggugcaaaaguaauu.........................................................    2       0    ya1
    .............caaaaguaauugugguuuu.................................................    5       0    ya1
    .............caaaaguaauugugguuuuu................................................    22      0    ya1
    .............caaaaguaauugugguuuuug...............................................    1       0    ya1
    ..............aaaaguaauugugguuuu.................................................    16      0    ya1
    ..............aaaaguaauugugguuuuu................................................    111     0    ya1
    ..............aaaaguaauugugguuuuug...............................................    8       0    ya1
    ...............aaaguaauugugguuuuu................................................    19      0    ya1
    ..................................................ugaaaacugcacuuacuuuug..........    1       0    ya1

    ..............aaaaguaauugugguuuuug...............................................    1       0    s04

    uauuagguuggugcaaaaguaauu.........................................................    2       0    j26
    ..uuagguuggugcaaaaguaauug........................................................    1       0    j26
    ..............aaaaguaauugugguuuuug...............................................    1       0    j26

    ........uggugcaaaaguaauuguggu....................................................    1       0    va1
    .............caaaaguaauugugguuuuugc..............................................    1       0    va1
    ..............aaaaguaauugugguuuuu................................................    2       0    va1
    ..............aaaaguaauugugguuuuug...............................................    25      0    va1
    ..............aaaaguaauugugguuuuugc..............................................    55      0    va1
    ..............aaaaguaauugugguuuuugcc.............................................    35      0    va1
    ..............aaaaguaauugugguuuuugcca............................................    3       0    va1
    ...............aaaguaauugugguuuuug...............................................    2       0    va1
    ...............aaaguaauugugguuuuugc..............................................    1       0    va1
    ...............aaaguaauugugguuuuugcc.............................................    1       0    va1

    .......uuggugcaaaaguaauugugguu...................................................    1       0    ku5
    ........uggugcaaaaguaauuguggu....................................................    1       0    ku5
    ..........gugcaaaaguaauugugguu...................................................    1       0    ku5
    .............caaaaguaauugugguuuuug...............................................    1       0    ku5
```

Mature **Star**

```
uauuagguuggugcaaaaguaauugugguuuuugccauuacucuuaauugugaaaacugcacuuacuuuugcaccaaccua
..............aaaaguaauugugguuuuug...............................................    3    0    ku5

.............caaaaguaauugugguuuuu................................................    2    0    k05
.............caaaaguaauugugguuuuug...............................................    2    0    k05
.............caaaaguaauugugguuuuugc..............................................    1    0    k05
..............aaaaguaauugugguuuuug...............................................    3    0    k05
..............aaaaguaauugugguuuuugc..............................................    1    0    k05
...............aaaguaauugugguuuuug...............................................    1    0    k05

..uuagguuggugcaaaaguaauu.........................................................    1    0    s06
.............caaaaguaauugugguuuuug...............................................    1    0    s06
..............aaaaguaauugugguuuuugc..............................................    1    0    s06
..............aaaaguaauugugguuuuugcc.............................................    2    0    s06
..................................................ugaaaacugcacuuacuuuug..........    1    0    s06

..uuagguuggugcaaaaguaauu.........................................................    1    0    HeL
..............aaaaguaauugugguuuuugc..............................................    5    0    HeL
..............aaaaguaauugugguuuuugcc.............................................    49   0    HeL
..............aaaaguaauugugguuuuugcca............................................    6    0    HeL
...............aaaguaauugugguuuuugc..............................................    1    0    HeL
...............aaaguaauugugguuuuugcc.............................................    1    0    HeL
................aaguaauugugguuuuugccauu..........................................    1    0    HeL
..................guaauugugguuuuugcc.............................................    5    0    HeL
..................................................ugaaaacugcacuuacuuuug..........    1    0    HeL

..........gugcaaaaguaauugugguu...................................................    1    0    ku4
..............aaaaguaauugugguuuuu................................................    5    0    ku4
..............aaaaguaauugugguuuuug...............................................    11   0    ku4

..............aaaaguaauugugguuuuugcc.............................................    1    0    s07

..............aaaaguaauugugguuuuug...............................................    2    0    s10
..............aaaaguaauugugguuuuugc..............................................    1    0    s10
..................................................ugaaaacugcacuuacuuuug..........    1    0    s10

.............caaaaguaauugugguuuuu................................................    2    0    k08
.............caaaaguaauugugguuuuug...............................................    1    0    k08
..............aaaaguaauugugguuuuu................................................    1    0    k08
..............aaaaguaauugugguuuuug...............................................    1    0    k08
...............aaaguaauugugguuuuugcc.............................................    1    0    k08

..............aaaaguaauugugguuuu.................................................    1    0    ta3
..............aaaaguaauugugguuuuug...............................................    7    0    ta3
..............aaaaguaauugugguuuuugc..............................................    1    0    ta3
...............aaaguaauugugguuuuug...............................................    1    0    ta3

.............caaaaguaauugugguuuuugc..............................................    1    0    j10
..............aaaaguaauugugguuuuu................................................    2    0    j10
..............aaaaguaauugugguuuuug...............................................    6    0    j10
..............aaaaguaauugugguuuuugc..............................................    5    0    j10

..............aaaaguaauugugguuuuug...............................................    3    0    j04

.............caaaaguaauugugguuuuu................................................    2    0    k14
.............caaaaguaauugugguuuuug...............................................    4    0    k14
..............aaaaguaauugugguuuuu................................................    2    0    k14
..............aaaaguaauugugguuuuug...............................................    4    0    k14
...............aaaguaauugugguuuuug...............................................    1    0    k14

...uagguuggugcaaaaguaauu.........................................................    1    0    j02
...uagguuggugcaaaaguaauug........................................................    1    0    j02
..............aaaaguaauugugguuuu.................................................    1    0    j02
..............aaaaguaauugugguuuuu................................................    5    0    j02
..............aaaaguaauugugguuuuug...............................................    22   0    j02
...............aaaguaauugugguuuuug...............................................    1    0    j02

.auuagguuggugcaaaaguaa...........................................................    1    0    va2
...uagguuggugcaaaaguaauug........................................................    1    0    va2
.............caaaaguaauugugguuuuu................................................    1    0    va2
.............caaaaguaauugugguuuuugc..............................................    1    0    va2
```

Mature **Star**

uauuagguuggugcaaaaguaauugugguuuuugccauuacucuuaaauugugaaaacugcacuuacuuuugcaccaaccua

..............aaaaguaauugugguuuuu...	1	0	va2
..............aaaaguaauugugguuuuug..	4	0	va2
..............aaaaguaauugugguuuuugc...	4	0	va2
...............aaaguaauugugguuuuugc...	1	0	va2
.........ggugcaaaaguaauugugguu..	1	0	j27
.............caaaaguaauugugguuuuu...	1	0	j27
.............caaaaguaauugugguuuuug..	4	0	j27
..............aaaaguaauugugguuu...	1	0	j27
..............aaaaguaauugugguuuuu...	2	0	j27
..............aaaaguaauugugguuuuug..	13	0	j27
...............aaaguaauugugguuuuugc...	1	0	j27
..uuagguuggugcaaaaguaauug...	1	0	j31
.......uuggugcaaaaguaauuguggu...	1	0	j31
........uggugcaaaaguaauugugguu..	1	0	j31
.............caaaaguaauugugguuuuu...	1	0	j31
.............caaaaguaauugugguuuuug..	2	0	j31
..............aaaaguaauugugguuuuug..	1	0	j31
..............aaaaguaauugugguuuuugcc..	1	0	j31
..............aaaaguaauugugguuuuugc...	2	0	s03
....ugaaaacugcacuuacuuuug..........	1	0	s03
....ugaaaacugcacuuacuuuugc.........	1	0	s03
..uuagguuggugcaaaaguaauu..	1	0	H52
.............caaaaguaauugugguuuuugc...	1	0	H52
..............aaaaguaauugugguuuuu...	2	0	H52
..............aaaaguaauugugguuuuugc...	4	0	H52
..............aaaaguaauugugguuuuugcc..	16	0	H52
...............aaaguaauugugguuuuugcca...	1	0	H52
.............caaaaguaauugugguuuuu...	1	0	j22
.............caaaaguaauugugguuuuug..	1	0	j22
.............caaaaguaauugugguuuuugc...	2	0	j22
..............aaaaguaauugugguuuuu...	1	0	j22
..............aaaaguaauugugguuuuug..	33	0	j22
..............aaaaguaauugugguuuuugc...	1	0	j22
..............aaaaguaauugugguuuuugcc..	2	0	j22
...............aaaguaauugugguuuuug..	3	0	j22
...uagguuggugcaaaaguaauug...	1	0	k09
.......uuggugcaaaaguaauugugg..	1	0	k09
...........ugcaaaaguaauugugguuuuu...	1	0	k09
.............caaaaguaauugugguuuuu...	1	0	k09
.............caaaaguaauugugguuuuug..	2	0	k09
..............aaaaguaauugugguuuuu...	2	0	k09
..............aaaaguaauugugguuuuug..	4	0	k09
..............aaaaguaauugugguuuuugc...	1	0	k09
..............aaaaguaauugugguuuuugcc..	1	0	k09
....ugaaaacugcacuuacuuuugc.........	1	0	k09
.............caaaaguaauugugguuuuug..	3	0	B14
.............caaaaguaauugugguuuuugc...	1	0	B14
..............aaaaguaauugugguuuuugc...	6	0	B14
..............aaaaguaauugugguuuuugcc..	15	0	B14
...............aaaguaauugugguuuuugcc..	1	0	B14
.............caaaaguaauugugguuuuug..	1	0	j16
..............aaaaguaauugugguuu...	1	0	j16
..............aaaaguaauugugguuuuu...	12	0	j16
..............aaaaguaauugugguuuuug..	25	0	j16
...............aaaguaauugugguuuuugc...	1	0	j16
.............caaaaguaauugugguuuuu...	2	0	j09
..............aaaaguaauugugguuuuu...	4	0	j09
..............aaaaguaauugugguuuuug..	9	0	j09
...............aaaguaauugugguuuuug..	1	0	j09
.............caaaaguaauugugguuuuu...	3	0	j18

```
              Mature                             Star
uauuagguuggugcaaaaguaauugugguuuuugccauuacucuuaauugugaaaacugcacuuacuuuugcaccaaccua
.............caaaaguaauugugguuuuug...............................................    1    0    j18
..............aaaaguaauugugguuuuug...............................................    1    0    j18

..uuagguuggugcaaaaguaau..........................................................    2    0    ku3
........uggugcaaaaguaauuguggu....................................................    1    0    ku3
.............caaaaguaauugugguuuuug...............................................    1    0    ku3
..............aaaaguaauugugguuuuug...............................................    6    0    ku3

..uuagguuggugcaaaaguaa...........................................................    1    0    ku6
.............caaaaguaauugugguuuu.................................................    1    0    ku6
.............caaaaguaauugugguuuuu................................................    1    0    ku6
..............aaaaguaauugugguuuuug...............................................    2    0    ku6

..............aaaaguaauugugguuuuu................................................    4    0    j11
..............aaaaguaauugugguuuuug...............................................    7    0    j11
...............aaaguaauugugguuuuug...............................................    1    0    j11

..............aaaaguaauugugguuuuug...............................................    4    0    s12
...............aaaguaauugugguuuuug...............................................    2    0    s12
..................................................ugaaaacugcacuuacuuuug..........    1    0    s12

..uuagguuggugcaaaaguaauu.........................................................    1    0    ya3
...uagguuggugcaaaaguaauu.........................................................    1    0    ya3
............gcaaaaguaauugugguuuuu................................................    2    0    ya3
.............caaaaguaauugugguuuu.................................................    9    0    ya3
.............caaaaguaauugugguuuuu................................................    22   0    ya3
..............aaaaguaauugugguuuu.................................................    44   0    ya3
..............aaaaguaauugugguuuuu................................................    238  0    ya3
..............aaaaguaauugugguuuuug...............................................    27   0    ya3
..............aaaaguaauugugguuuuugc..............................................    1    0    ya3
...............aaaguaauugugguuuuu................................................    45   0    ya3
...............aaaguaauugugguuuuug...............................................    8    0    ya3
..................................................ugaaaacugcacuuacuuuu...........    2    0    ya3

.............caaaaguaauugugguuuuu................................................    1    0    la1
.............caaaaguaauugugguuuuugc..............................................    1    0    la1
..............aaaaguaauugugguuuu.................................................    1    0    la1
..............aaaaguaauugugguuuuu................................................    1    0    la1
..............aaaaguaauugugguuuuug...............................................    3    0    la1
..............aaaaguaauugugguuuuugcc.............................................    1    0    la1

........uggugcaaaaguaauugugguu...................................................    1    0    HEK
.............caaaaguaauugugguuuuugc..............................................    7    0    HEK
..............aaaaguaauugugguuuuugc..............................................    9    0    HEK
..............aaaaguaauugugguuuuugcc.............................................    75   0    HEK
...............aaaguaauugugguuuuugcc.............................................    2    0    HEK
...............aaaguaauugugguuuuugcca............................................    1    0    HEK
................aaguaauugugguuuuugcc.............................................    1    0    HEK
................aaguaauugugguuuuugccau...........................................    1    0    HEK

.............caaaaguaauugugguuuuug...............................................    4    0    j15
.............caaaaguaauugugguuuuugc..............................................    1    0    j15
..............aaaaguaauugugguuuu.................................................    1    0    j15
..............aaaaguaauugugguuuuu................................................    3    0    j15
..............aaaaguaauugugguuuuug...............................................    16   0    j15
...............aaaguaauugugguuuuu................................................    1    0    j15
...............aaaguaauugugguuuuug...............................................    1    0    j15

..uuagguuggugcaaaaguaauu.........................................................    1    0    j17
..............aaaaguaauugugguuuu.................................................    1    0    j17
..............aaaaguaauugugguuuuu................................................    8    0    j17
..............aaaaguaauugugguuuuug...............................................    8    0    j17

..uuagguuggugcaaaagu.............................................................    1    0    la2
..............aaaaguaauugugguuuuu................................................    1    0    la2
..............aaaaguaauugugguuuuug...............................................    3    0    la2
..............aaaaguaauugugguuuuugcc.............................................    6    0    la2
...............aaaguaauugugguuuuugcc.............................................    1    0    la2
.....................................................aaacugcacuuacuuuugcacca.....    1    0    la2

........uggugcaaaaguaauugugguu...................................................    1    0    j12
```

Mature **Star**

```
uauuagguuggugcaaaaguaauugugguuuuugccauuacucuuaauugugaaaacugcacuuacuuuugcaccaaccua
.............caaaaguaauugugguuuuug...............................................	5	0	j12
..............aaaaguaauugugguuuuu................................................	6	0	j12
..............aaaaguaauugugguuuuug...............................................	24	0	j12
..............aaaaguaauugugguuuuugc..............................................	2	0	j12
..............aaaaguaauugugguuuuugcc.............................................	1	0	j12
...............aaaguaauugugguuuuug...............................................	2	0	j12

........uggugcaaaaguaauugugguu...................................................	1	0	li2
.............caaaaguaauugugguuuuug...............................................	4	0	li2
.............caaaaguaauugugguuuuugc..............................................	9	0	li2
..............aaaaguaauugugguuuuugc..............................................	56	0	li2
..............aaaaguaauugugguuuuugcc.............................................	3	0	li2
...............aaaguaauugugguuuuugc..............................................	3	0	li2
..................................................ugaaaacugcacuuacuuuugc.........	1	0	li2

.............caaaaguaauugugguuuuug...............................................	1	0	va4
.............caaaaguaauugugguuuuugc..............................................	1	0	va4
..............aaaaguaauugugguuuuu................................................	1	0	va4
..............aaaaguaauugugguuuuug...............................................	5	0	va4
..............aaaaguaauugugguuuuugc..............................................	11	0	va4
..............aaaaguaauugugguuuuugcc.............................................	11	0	va4

.............caaaaguaauugugguuuuug...............................................	2	0	va3
.............caaaaguaauugugguuuuugc..............................................	1	0	va3
..............aaaaguaauugugguuuuu................................................	2	0	va3
..............aaaaguaauugugguuuuug...............................................	10	0	va3
..............aaaaguaauugugguuuuugc..............................................	13	0	va3
..............aaaaguaauugugguuuuugcc.............................................	3	0	va3
...............aaaguaauugugguuuuug...............................................	2	0	va3
...............aaaguaauugugguuuuugcc.............................................	1	0	va3

.auuagguuggugcaaaaguaauu.........................................................	1	0	ya4
...uagguuggugcaaaaguaau..........................................................	4	0	ya4
.............caaaaguaauugugguuuu.................................................	16	0	ya4
.............caaaaguaauugugguuuuu................................................	16	0	ya4
.............caaaaguaauugugguuuuug...............................................	4	0	ya4
..............aaaaguaauugugguuuu.................................................	33	0	ya4
..............aaaaguaauugugguuuuu................................................	213	0	ya4
..............aaaaguaauugugguuuuug...............................................	5	0	ya4
..............aaaaguaauugugguuuuugcc.............................................	4	0	ya4
...............aaaguaauugugguuuuu................................................	43	0	ya4
...............aaaguaauugugguuuuug...............................................	3	0	ya4
..................................................ugaaaacugcacuuacuuuu...........	1	0	ya4

..uuagguuggugcaaaaguaauu.........................................................	1	0	SW4
.............caaaaguaauugugguuuuu................................................	1	0	SW4
.............caaaaguaauugugguuuuugc..............................................	1	0	SW4
..............aaaaguaauugugguuuuu................................................	1	0	SW4
..............aaaaguaauugugguuuuug...............................................	3	0	SW4
..............aaaaguaauugugguuuuugc..............................................	12	0	SW4
..............aaaaguaauugugguuuuugcc.............................................	20	0	SW4
...............aaaguaauugugguuuuugc..............................................	1	0	SW4
...............aaaguaauugugguuuuugcca............................................	1	0	SW4

..uuagguuggugcaaaaguaauu.........................................................	1	0	ku1
.............caaaaguaauugugguuuuu................................................	1	0	ku1
.............caaaaguaauugugguuuuug...............................................	1	0	ku1
.............caaaaguaauugugguuuuugc..............................................	1	0	ku1
..............aaaaguaauugugguuuuu................................................	5	0	ku1
..............aaaaguaauugugguuuuug...............................................	38	0	ku1
..............aaaaguaauugugguuuuugc..............................................	1	0	ku1
...............aaaguaauugugguuuuug...............................................	1	0	ku1

...........ugcaaaaguaauugugguuuuu................................................	1	0	j23
.............caaaaguaauugugguuuuug...............................................	4	0	j23
..............aaaaguaauugugguuuu.................................................	1	0	j23
..............aaaaguaauugugguuuuu................................................	8	0	j23
..............aaaaguaauugugguuuuug...............................................	89	0	j23
..............aaaaguaauugugguuuuugc..............................................	5	0	j23
..............aaaaguaauugugguuuuugcc.............................................	13	0	j23
```

Mature **Star**

```
uauuagguuggugcaaaaguaauugugguuuuugccauuacucuuaauugugaaaacugcacuuacuuuugcaccaaccua
...............aaaguaauugugguuuuug...............................................   11   0   j23
...............aaaguaauugugguuuuugc..............................................   1    0   j23
...............aaaguaauugugguuuuugcc.............................................   2    0   j23

..............aaaaguaauugugguuuuug...............................................   7    0   s05
..............aaaaguaauugugguuuuugc..............................................   1    0   s05
...............aaaguaauugugguuuuug...............................................   1    0   s05

..............aaaaguaauugugguuuuugc..............................................   4    0   ta2
...............aaaguaauugugguuuuug...............................................   1    0   ta2

.........ggugcaaaaguaauugugguuu..................................................   5    0   j24
.............caaaaguaauugugguuuu.................................................   3    0   j24
.............caaaaguaauugugguuuuug...............................................   1    0   j24
..............aaaaguaauugugguuuuu................................................   3    0   j24
..............aaaaguaauugugguuuuug...............................................   8    0   j24

..uuagguuggugcaaaaguaa...........................................................   1    0   k11
..uuagguuggugcaaaaguaau..........................................................   1    0   k11
.............caaaaguaauugugguuuuug...............................................   1    0   k11
..............aaaaguaauugugguuuuu................................................   2    0   k11
..............aaaaguaauugugguuuuug...............................................   2    0   k11
..............aaaaguaauugugguuuuugc..............................................   3    0   k11

..............aaaaguaauugugguuuu.................................................   1    0   ta1
..............aaaaguaauugugguuuuu................................................   1    0   ta1
..............aaaaguaauugugguuuuug...............................................   3    0   ta1
..............aaaaguaauugugguuuuugcc.............................................   1    0   ta1
...............aaaguaauugugguuuuug...............................................   1    0   ta1

.............caaaaguaauugugguuuuug...............................................   1    0   s09
..............aaaaguaauugugguuuuu................................................   1    0   s09
..............aaaaguaauugugguuuuug...............................................   3    0   s09
...............aaaguaauugugguuuuug...............................................   1    0   s09
..................................................ugaaaacugcacuuacuuuug..........   1    0   s09

..uuagguuggugcaaaaguaauu.........................................................   1    0   NL2
.............caaaaguaauugugguuu..................................................   1    0   NL2
.............caaaaguaauugugguuuu.................................................   1    0   NL2
.............caaaaguaauugugguuuuu................................................   4    0   NL2
..............aaaaguaauugugguuuu.................................................   10   0   NL2
..............aaaaguaauugugguuuuu................................................   2    0   NL2
..............aaaaguaauugugguuuuug...............................................   2    0   NL2

..uuagguuggugcaaaaguaauu.........................................................   2    0   k10
........uggugcaaaaguaauugugguu...................................................   1    0   k10
.............caaaaguaauugugguuuuu................................................   1    0   k10
.............caaaaguaauugugguuuuug...............................................   2    0   k10
..............aaaaguaauugugguuuuug...............................................   4    0   k10
..............aaaaguaauugugguuuuugc..............................................   2    0   k10

.............caaaaguaauugugguuuuug...............................................   1    0   j13
.............caaaaguaauugugguuuuugc..............................................   1    0   j13
..............aaaaguaauugugguuuu.................................................   1    0   j13
..............aaaaguaauugugguuuuu................................................   6    0   j13
..............aaaaguaauugugguuuuug...............................................   33   0   j13
..............aaaaguaauugugguuuuugc..............................................   4    0   j13
..............aaaaguaauugugguuuuugcc.............................................   1    0   j13
...............aaaguaauugugguuuuug...............................................   1    0   j13
...............aaaguaauugugguuuuugc..............................................   1    0   j13

.............caaaaguaauugugguuuuu................................................   2    0   j19
.............caaaaguaauugugguuuuug...............................................   1    0   j19
..............aaaaguaauugugguuuu.................................................   2    0   j19
..............aaaaguaauugugguuuuu................................................   5    0   j19
..............aaaaguaauugugguuuuug...............................................   37   0   j19
..............aaaaguaauugugguuuuugc..............................................   2    0   j19

..............aaaaguaauugugguuuuug...............................................   4    0   j03

..............aaaaguaauugugguuuuug...............................................   1    0   k01
```

```
            Mature                      Star
uauuagguuggugcaaaaguaauugugguuuuugccauuacucuuaauugugaaaacugcacuuacuuuugcaccaaccua
..............aaaaguaauugugguuuuugc..............................................      3     0     k01

..uuagguuggugcaaaaguaa...........................................................      1     0     j25
........uggugcaaaaguaauugugguu...................................................      1     0     j25
...........ugcaaaaguaauugugguu...................................................      2     0     j25
.............caaaaguaauugugguuuu.................................................      1     0     j25
..............aaaaguaauugugguuuuug...............................................      10    0     j25

.auuagguuggugcaaaaguaa...........................................................      1     0     per
.auuagguuggugcaaaaguaau..........................................................      2     0     per
..uuagguuggugcaaaaguaa...........................................................      1     0     per
..uuagguuggugcaaaaguaau..........................................................      1     0     per
............gcaaaaguaauugugguuuuug...............................................      1     0     per
.............caaaaguaauugugguuuuug...............................................      1     0     per
..............aaaaguaauugugguuuu.................................................      1     0     per
..............aaaaguaauugugguuuuug...............................................      8     0     per
...............aaaguaauugugguuuuug...............................................      1     0     per

............gcaaaaguaauugugguuuuug...............................................      1     0     s01
..................................................ugaaaacugcacuuacuuuugc.........      1     0     s01

.............caaaaguaauugugguuuuu................................................      2     0     k04
.............caaaaguaauugugguuuuug...............................................      1     0     k04
..............aaaaguaauugugguuuu.................................................      1     0     k04
..............aaaaguaauugugguuuuug...............................................      5     0     k04
..............aaaaguaauugugguuuuugc..............................................      1     0     k04

...uagguuggugcaaaaguaauug........................................................      1     0     ku2
.............caaaaguaauugugguuuuu................................................      2     0     ku2
..............aaaaguaauugugguuuuu................................................      5     0     ku2
..............aaaaguaauugugguuuuug...............................................      16    0     ku2
..............aaaaguaauugugguuuuugc..............................................      1     0     ku2
..............aaaaguaauugugguuuuugcc.............................................      1     0     ku2

........uggugcaaaaguaauuguggu....................................................      2     0     U20
........uggugcaaaaguaauugugguu...................................................      1     0     U20
..........gugcaaaaguaauugugguuuu.................................................      1     0     U20
.............caaaaguaauugugguuuuug...............................................      1     0     U20
.............caaaaguaauugugguuuuugc..............................................      18    0     U20
.............caaaaguaauugugguuuuugcc.............................................      1     0     U20
..............aaaaguaauugugguuuuu................................................      1     0     U20
..............aaaaguaauugugguuuuug...............................................      6     0     U20
..............aaaaguaauugugguuuuugc..............................................      48    0     U20
..............aaaaguaauugugguuuuugcc.............................................      243   0     U20
..............aaaaguaauugugguuuuugcca............................................      3     0     U20
...............aaaguaauugugguuuuugc..............................................      11    0     U20
...............aaaguaauugugguuuuugcc.............................................      24    0     U20
...............aaaguaauugugguuuuugcca............................................      1     0     U20
.................aguaauugugguuuuugc..............................................      1     0     U20

.auuagguuggugcaaaaguaauug........................................................      1     0     j21
.............caaaaguaauugugguuuuu................................................      1     0     j21
.............caaaaguaauugugguuuuug...............................................      3     0     j21
..............aaaaguaauugugguuuuu................................................      1     0     j21
..............aaaaguaauugugguuuuug...............................................      14    0     j21
..............aaaaguaauugugguuuuugc..............................................      3     0     j21
..............aaaaguaauugugguuuuugcc.............................................      2     0     j21
...............aaaguaauugugguuuuug...............................................      3     0     j21
...............aaaguaauugugguuuuugcc.............................................      2     0     j21


..............aaaaguaauugugguuuuug...............................................      1     0     s11

..............aaaaguaauugugguuuuu................................................      4     0     j07
..............aaaaguaauugugguuuuug...............................................      2     0     j07
...............aaaguaauugugguuuuug...............................................      1     0     j07

.............caaaaguaauugugguuuuug...............................................      5     0     j14
..............aaaaguaauugugguuuuug...............................................      9     0     j14
..............aaaaguaauugugguuuuugc..............................................      5     0     j14
```

Mature **Star**

```
uauuagguuggugcaaaaguaauugugguuuuugccauuacucuuaaaugugaaaacugcacuuacuuuugcaccaaccua
..............aaaaguaauugugguuuuugcc.............................................   1    0    j14

..uuagguuggugcaaaaguaau..........................................................   1    0    k02
.............caaaaguaauugugguuuuug...............................................   2    0    k02
..............aaaaguaauugugguuuuu................................................   2    0    k02
..............aaaaguaauugugguuuuugc..............................................   1    0    k02

..uuagguuggugcaaaaguaau..........................................................   1    0    j28
..uuagguuggugcaaaaguaauu.........................................................   1    0    j28
...uagguuggugcaaaaguaauu.........................................................   1    0    j28
.............caaaaguaauugugguuuuu................................................   1    0    j28
..............aaaaguaauugugguuuuu................................................   3    0    j28
..............aaaaguaauugugguuuuug...............................................   17   0    j28

..............aaaaguaauugugguuuuug...............................................   1    0    s08

.............caaaaguaauugugguuuuugc..............................................   1    0    DLD
..............aaaaguaauugugguuuuu................................................   2    0    DLD
..............aaaaguaauugugguuuuug...............................................   1    0    DLD
..............aaaaguaauugugguuuuugc..............................................   13   0    DLD
..............aaaaguaauugugguuuuugcc.............................................   73   0    DLD
..............aaaaguaauugugguuuuugcca............................................   1    0    DLD
...............aaaguaauugugguuuuugcc.............................................   4    0    DLD
..................guaauugugguuuuugcc.............................................   1    0    DLD

......guuggugcaaaaguaauuguggu....................................................   1    0    k03
..............aaaaguaauugugguuuuug...............................................   5    0    k03
..............aaaaguaauugugguuuuugc..............................................   3    0    k03

..uuagguuggugcaaaaguaauu.........................................................   2    0    ya2
.............caaaaguaauugugguuu..................................................   1    0    ya2
.............caaaaguaauugugguuuu.................................................   6    0    ya2
.............caaaaguaauugugguuuuu................................................   13   0    ya2
.............caaaaguaauugugguuuuug...............................................   2    0    ya2
..............aaaaguaauugugguuuu.................................................   24   0    ya2
..............aaaaguaauugugguuuuu................................................   188  0    ya2
..............aaaaguaauugugguuuuug...............................................   17   0    ya2
..............aaaaguaauugugguuuuugcc.............................................   1    0    ya2
..............aaaaguaauugugguuuuugcca............................................   1    0    ya2
...............aaaguaauugugguuuuu................................................   43   0    ya2
...............aaaguaauugugguuuuug...............................................   4    0    ya2
..................................................ugaaaacugcacuuacuuuug..........   1    0    ya2

..uuagguuggugcaaaaguaauu.........................................................   1    0    NL3
.............caaaaguaauugugguuu..................................................   1    0    NL3
.............caaaaguaauugugguuuu.................................................   1    0    NL3
.............caaaaguaauugugguuuuu................................................   4    0    NL3
.............caaaaguaauugugguuuuug...............................................   2    0    NL3
..............aaaaguaauugugguuuu.................................................   15   0    NL3
..............aaaaguaauugugguuuuu................................................   6    0    NL3
..............aaaaguaauugugguuuuug...............................................   5    0    NL3
..................................................ugaaaacugcacuuacuuuug..........   1    0    NL3

.............caaaaguaauugugguuuuug...............................................   1    0    s02
..............aaaaguaauugugguuuuugcc.............................................   1    0    s02
..................................................ugaaaacugcacuuacuuuugc.........   1    0    s02

.............caaaaguaauugugguuuuug...............................................   3    0    k06
..............aaaaguaauugugguuuuug...............................................   2    0    k06

.auuagguuggugcaaaagu..............................................................   2    0    sh2
..uuagguuggugcaaaaguaa...........................................................   1    0    sh2
.....gguuggugcaaaaguaauugu.......................................................   1    0    sh2
.......uuggugcaaaaguaauugugguu...................................................   1    0    sh2
..........gugcaaaaguaauugugguu...................................................   1    0    sh2
.............caaaaguaauugugguuuuu................................................   1    0    sh2
.............caaaaguaauugugguuuuugc..............................................   1    0    sh2
..............aaaaguaauugugguuuuug...............................................   1    0    sh2
..............aaaaguaauugugguuuuugc..............................................   3    0    sh2
..............aaaaguaauugugguuuuugcc.............................................   3    0    sh2
```

Mature   Star

uauuagguuggugcaaaaguaauugugguuuuugccauuacucuuaauugugaaaacugcacuuacuuuugcaccaaccua

..............aaaaguaauugugguuuuugcca..	2	0	sh2
....uuacuuuugcaccaaccua	1	0	sh2
............gcaaaaguaauugugguuuuug...	1	0	j29
.............caaaaguaauugugguuuuug...	3	0	j29
..............aaaaguaauugugguuuuug...	10	0	j29
..............aaaaguaauugugguuuuugc..	8	0	j29
..............aaaaguaauugugguuuuugcc...	1	0	j29
........uggugcaaaaguaauuguggu..	1	0	MCF
.............caaaaguaauugugguuuuugc..	1	0	MCF
..............aaaaguaauugugguuuuug...	4	0	MCF
..............aaaaguaauugugguuuuugc..	11	0	MCF
..............aaaaguaauugugguuuuugcc...	42	0	MCF
..............aaaaguaauugugguuuuugcca..	1	0	MCF
...............aaaguaauugugguuuuugcc...	1	0	MCF
.........ggugcaaaaguaauugugguuu..	1	0	j01
...........ugcaaaaguaauugugguuuu...	3	0	j01
.............caaaaguaauugugguuuuug...	1	0	j01
..............aaaaguaauugugguuuuug...	5	0	j01
.............caaaaguaauugugguuuuugc..	1	0	MBM
..............aaaaguaauugugguuuuug...	2	0	MBM
..............aaaaguaauugugguuuuugc..	14	0	MBM
..............aaaaguaauugugguuuuugcc...	33	0	MBM
..............aaaaguaauugugguuuuugcca..	6	0	MBM
...............aaaguaauugugguuuuugc..	1	0	MBM
...............aaaguaauugugguuuuugcc...	1	0	MBM
..................guaauugugguuuuugcc...	1	0	MBM
....ugaaaacugcacuuacuuuugc.........	3	0	MBM
....gaaaacugcacuuacuuuugc.........	1	0	MBM
..uuagguuggugcaaaaguaau..	2	0	NL1
..............aaaaguaauugugguuuuu..	2	0	NL1
..............aaaaguaauugugguuuuug...	2	0	NL1
..............aaaaguaauugugguuuuugc..	1	0	NL1
.........ggugcaaaaguaauugugguu...	1	0	k07
...........ugcaaaaguaauugugguuuu...	1	0	k07
.............caaaaguaauugugguuuuug...	9	0	k07
..............aaaaguaauugugguuuuug...	3	0	k07
..............aaaaguaauugugguuuuugc..	1	0	k07
...........ugcaaaaguaauugugguuuu...	1	0	sh1
.............caaaaguaauugugguuuuug...	6	0	sh1
..............aaaaguaauugugguuuuug...	4	0	sh1
........uggugcaaaaguaauugugguu...	1	0	li1
.............caaaaguaauugugguuuuug...	3	0	li1
.............caaaaguaauugugguuuuugc..	1	0	li1
..............aaaaguaauugugguuuuugc..	23	0	li1
..............aaaaguaauugugguuuuugcc...	2	0	li1
...............aaaguaauugugguuuuugc..	1	0	li1
....ugaaaacugcacuuacuuuug..........	1	0	li1
....ugaaaacugcacuuacuuuugc.........	2	0	li1
.............caaaaguaauugugguuuuu..	1	0	k13
.............caaaaguaauugugguuuuug...	4	0	k13
..............aaaaguaauugugguuuuug...	1	0	k13
.............caaaaguaauugugguuuu...	1	0	j20
.............caaaaguaauugugguuuuu..	2	0	j20
.............caaaaguaauugugguuuuug...	7	0	j20
..............aaaaguaauugugguuuuu..	3	0	j20
..............aaaaguaauugugguuuuug...	27	0	j20
..............aaaaguaauugugguuuuugcc...	1	0	j20
...............aaaguaauugugguuuuugc..	1	0	j20
............gcaaaaguaauugugguuuuug...	11	0	A54

```
              Mature                             Star
uauuagguuggugcaaaaguaauugugguuuuugccauuacucuuaaauugugaaaacugcacuuacuuuugcaccaaccua
..............aaaaguaauugugguuuuug................................................    3     0     A54
..............aaaaguaauugugguuuuugc...............................................    3     0     A54
..............aaaaguaauugugguuuuugcc..............................................    50    0     A54
..............aaaaguaauugugguuuuugcca.............................................    5     0     A54
...............aaaguaauugugguuuuugcc..............................................    5     0     A54
....................................................gaaaacugcacuuacuuuugc.........    7     0     A54

........uggugcaaaaguaauugugguu....................................................    1     0     j30
..............aaaaguaauugugguuuuug................................................    2     0     j30
..............aaaaguaauugugguuuuugcc..............................................    1     0     j30

.............caaaaguaauugugguuuuu.................................................    8     0     k12
.............caaaaguaauugugguuuuug................................................    12    0     k12
..............aaaaguaauugugguuuuu.................................................    6     0     k12
..............aaaaguaauugugguuuuug................................................    18    0     k12
..............aaaaguaauugugguuuuugc...............................................    2     0     k12
..............aaaaguaauugugguuuuugcc..............................................    1     0     k12
```

Provisional ID	:	hsa_can_1477
Score total	:	0.3
Score for star read(s)	:	-1.3
Score for read counts	:	0
Score for mfe	:	2.2
Score for randfold	:	
Score for cons. seed	:	-0.6
Total read count	:	58
Mature read count	:	58
Loop read count	:	0
Star read count	:	0

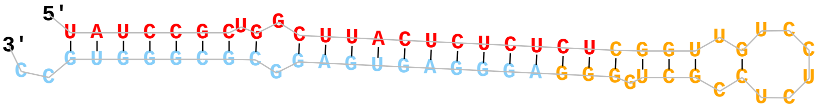

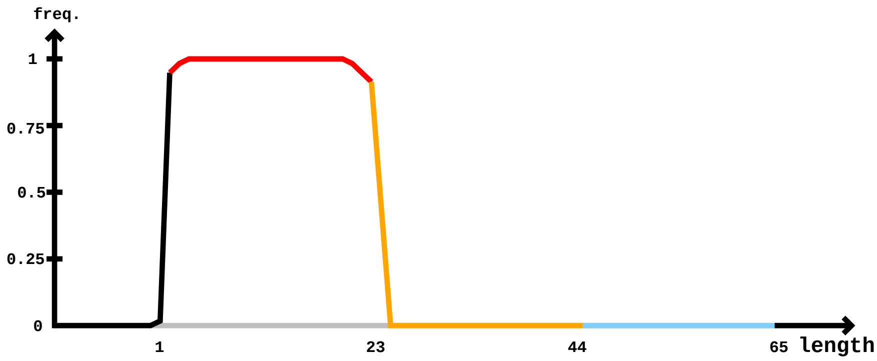

Mature **Star**

```
5'- ucuggcugggUAUCCGCUGGCUUACUCUCUCUcgguuguccucuccgcuggggagggagugaggcgcgggugccggccggg  -3'   exp
    .((((((((.(((((((.(.((((((((((((((((.(......).))).)))))))))))))).)))))))))))))))).   reads   mm   sample
    .........uauccgcuggcuuacucucucu.................................................   1   0   k09

    .........uauccgcuggcuuacucucucu.................................................   2   0   DLD
    .........uauccgcuggcuuacucucucuc................................................   1   0   DLD

    .........uauccgcuggcuuacucuc....................................................   1   0   ya1
    .........uauccgcuggcuuacucucucu.................................................   1   0   ya1
    .........uauccgcuggcuuacucucucuc................................................   1   0   ya1

    .........uauccgcuggcuuacucucucu.................................................   4   0   va1

    .........uauccgcuggcuuacucucucu.................................................   2   0   j24

    .........uauccgcuggcuuacucucucuc................................................   1   0   k05

    .........uauccgcuggcuuacucucucu.................................................   3   0   k11

    .........uauccgcuggcuuacucucucuc................................................   2   0   k06

    .........uauccgcuggcuuacucucucuc................................................   1   0   j29

    .........uauccgcuggcuuacucucuc..................................................   1   0   sh2
    .........uauccgcuggcuuacucucucu.................................................   4   0   sh2
    ..........auccgcuggcuuacucucucu.................................................   2   0   sh2

    .........uauccgcuggcuuacucucucuc................................................   1   0   MCF

    .........uauccgcuggcuuacucucucu.................................................   1   0   j17

    .........uauccgcuggcuuacucucucu.................................................   1   0   k07

    .........uauccgcuggcuuacucucu...................................................   1   0   li2
    .........uauccgcuggcuuacucucucuc................................................   1   0   li2

    .........uauccgcuggcuuacucucucu.................................................   1   0   li1
```

```
           Mature                                      Star
ucuggcugggUAUCCGCUGGCUUACUCUCUCUcgguuguccucuccgcuggggagggagugaggcgcgggugccggccggg
```

```
ucuggcuggguauccgcuggcuuacucucucucgguuguccucuccgcuggggagggagugaggcgcgggugccggccggg

.........guauccgcuggcuuacucucucu.................................................    1    0    k01

..........uauccgcuggcuuacucucucu.................................................    1    0    k13

..........uauccgcuggcuuacucucucuc................................................    1    0    s01

..........uauccgcuggcuuacucucucu.................................................    1    0    per

..........uauccgcuggcuuacucucucu.................................................    1    0    k04

..........uauccgcuggcuuacucucucu.................................................    3    0    U20
..........uauccgcuggcuuacucucucuc................................................    1    0    U20

..........uauccgcuggcuuacucucuc..................................................    1    0    j21
..........uauccgcuggcuuacucucucu.................................................    4    0    j21
..........uauccgcuggcuuacucucucuc................................................    1    0    j21

..........uauccgcuggcuuacucucu...................................................    1    0    va2

..........uauccgcuggcuuacucucucu.................................................    2    0    k14

..........uauccgcuggcuuacucucucuc................................................    1    0    va3

..........uauccgcuggcuuacucucucu.................................................    1    0    j31

..........uauccgcuggcuuacucucucu.................................................    1    0    k12
............uccgcuggcuuacucucucu.................................................    1    0    k12

..........uauccgcuggcuuacucucucu.................................................    3    0    ya4
```

Provisional ID	:	hsa_can_1512
Score total	:	-0.8
Score for star read(s)	:	-1.3
Score for read counts	:	0
Score for mfe	:	1
Score for randfold	:	
Score for cons. seed	:	-0.6
Total read count	:	30
Mature read count	:	30
Loop read count	:	0
Star read count	:	0

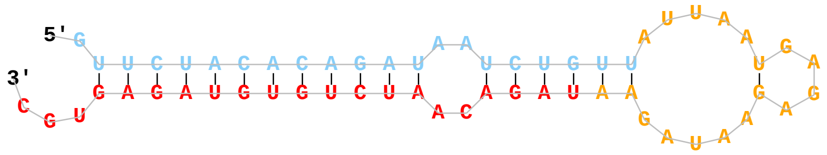

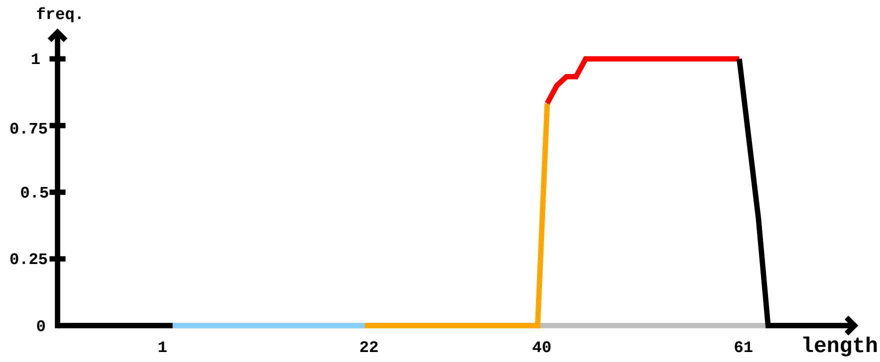

Star Mature

```
5'- ugaguuaaguguucuacacagauaaucuguuauuaaugagagaauagaauagacaaucuguguagagugcuuaaccuggug   -3'    exp
    ...((((((..((((((((((((..((((((.....(....).....))))))..))))))))))))..))))))......     reads      mm         sample
    .................................................uagacaaucuguguagagugcu..........     1          0          s08

    .................................................uagacaaucuguguagagugcu..........     1          0          k09

    .................................................uagacaaucuguguagagugcu..........     1          0          j16

    .................................................uagacaaucuguguagagugcu..........     1          0          va1
    .................................................uagacaaucuguguagagugcuu.........     1          0          va1
    ...................................................gacaaucuguguagagugcuu.........     1          0          va1

    .................................................uagacaaucuguguagagugcu..........     1          0          j24

    .................................................uagacaaucuguguagagugcu..........     1          0          s12

    .................................................uagacaaucuguguagagugcuu.........     1          0          s06

    .................................................uagacaaucuguguagagugcu..........     1          0          sh2

    .................................................uagacaaucuguguagagugcu..........     1          0          HEK

    .................................................uagacaaucuguguagagugcu..........     1          0          ku4

    .................................................uagacaaucuguguagagugcuu.........     2          0          MBM

    .................................................uagacaaucuguguagagugcuu.........     1          0          k07

    .................................................uagacaaucuguguagagugcu..........     1          0          k10
    ..................................................agacaaucuguguagagugcuu.........     1          0          k10

    .................................................uagacaaucuguguagagugc...........     2          0          li1
    .................................................uagacaaucuguguagagugcu..........     1          0          li1

    .................................................uagacaaucuguguagagugc...........     3          0          U20
    .................................................uagacaaucuguguagagugcuu.........     2          0          U20
```

Star **Mature**

```
ugaguuaaguguucuacacagauaaucuguuauuaaugagagaauagaauagacaaucuguguagagugcuuaaccuggug
....................................................caaucuguguagagugcu..........   2   0   U20
.................................................agacaaucuguguagagugcuu.........   1   0   A54
................................................uagacaaucuguguagagugcuu.........   1   0   j30
................................................uagacaaucuguguagagugcuu.........   1   0   ku1
```

Provisional ID : hsa_can_1520
Score total : 98.3
Score for star read(s) : 3.9
Score for read counts : 89.9
Score for mfe : 1.6
Score for randfold :
Score for cons. seed : 3
Total read count : 188
Mature read count : 184
Loop read count : 0
Star read count : 4

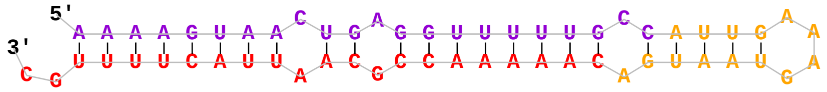

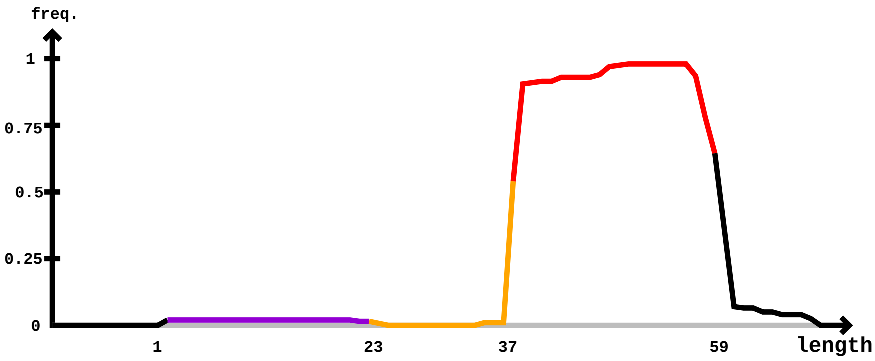

Star **Mature**

```
5'- agguugcugcaaaaguaacugagguuuuugccauugaaaguaaugacaaaaaccgcaauuacuuuugcaccaaccuaaagc  -3'   obs
    agguugcugcaaaaguaacugagguuuuugccauugaaaguaaugacaaaaaccgcaauuacuuuugcaccaaccuaaagc         exp
    agguugcugcaaaaguaacugagguuuuugccauugaaaguaaugacaaaaaccgcaauuacuuuugcaccaaccuaaagc         known
    ((((((.(((((((((((.((.((((((((.(((((....))))).)))))))).)).))))))))))).)))))).....   reads   mm   sample
    ..............................................caaaaaccgcaauuacuuu................   2       0    ya1
    ...............................................aaaaaccgcaauuacuuuu...............   2       0    ya1

    ..............................................caaaaaccgcaauuacuuuu...............   1       0    ku5
    ...............................................aaaaaccgcaauuacuuuug..............   1       0    ku5
    ...............................................aaaaaccgcaauuacuuuugca............   1       0    ku5

    ...............................................aaaaaccgcaauuacuuuu...............   1       0    s09

    ..............................................caaaaaccgcaauuacuuuugc.............   6       0    HeL
    ...............................................aaaaaccgcaauuacuuuugca............   1       0    HeL
    ................................................aaaaccgcaauuacuuuugca............   1       0    HeL

    ...............................................aaaaaccgcaauuacuuuug..............   1       0    s10

    ..............................................caaaaaccgcaauuacuuu................   1       0    NL2
    ..............................................caaaaaccgcaauuacuuuu...............   4       0    NL2
    ..............................................caaaaaccgcaauuacuuuug..............   1       0    NL2
    ...............................................aaaaaccgcaauuacuuu................   1       0    NL2
    ...............................................aaaaaccgcaauuacuuuu...............   2       0    NL2
    ...................................................accgcaauuacuuuugcac...........   1       0    NL2

    ..............................................caaaaaccgcaauuacuuuug..............   1       0    j13

    ..............................................caaaaaccgcaauuacuuuu...............   1       0    j19
    ..............................................caaaaaccgcaauuacuuuug..............   1       0    j19
    ...............................................aaaaaccgcaauuacuuuug..............   1       0    j19
    ...............................................aaaaaccgcaauuacuuuugca............   1       0    j19

    ..........aaaaguaacugagguuuuug...................................................   1       0    per
    ...............................................aaaaaccgcaauuacuuuug..............   1       0    per
    ........................................................aauuacuuuugcaccaaccua....   2       0    per
```

Star **Mature**

agguugcugcaaaaguaacugagguuuuugccauugaaaguaaugacaaaaaccgcaauuacuuuugcaccaaccuaaagc

..........aaaaguaacugagguuuuugcca..	1	0	k04
....ugacaaaaaccgcaauuacuuu................	2	0	U20
....caaaaaccgcaauuacuuuug..............	1	0	U20
....caaaaaccgcaauuacuuuugc.............	12	0	U20
....aaaaaccgcaauuacuuuugc.............	10	0	U20
....aaaaaccgcaauuacuuuugca............	1	0	U20
....caaaaaccgcaauuacuuuu...............	1	0	ku2
....caaaaaccgcaauuacuuuug..............	1	0	ku2
....caauuacuuuugcaccaaccuaa...	1	0	va2
....aaaaaccgcaauuacuuuug..............	1	0	s11
..........aaaaguaacugagguuuuugcc...	1	0	j14
....caaaaaccgcaauuacuuu................	1	0	j28
....caaaaaccgcaauuacuuuu...............	1	0	j28
....aaaaaccgcaauuacuuuug..............	3	0	j28
....caaaaaccgcaauuacuuuugc.............	2	0	H52
....aaaaaccgcaauuacuuuugc.............	2	0	H52
....accgcaauuacuuuugcacca.........	1	0	k09
....caaaaaccgcaauuacuuuugc.............	1	0	s08
....caaaaaccgcaauuacuuuug..............	1	0	B14
....caaaaaccgcaauuacuuuugc.............	2	0	B14
....aaaaaccgcaauuacuuuugc.............	1	0	B14
....aaaaaccgcaauuacuuuugca............	2	0	B14
....caaaaaccgcaauuacuuuugc.............	2	0	DLD
....aaaaaccgcaauuacuuuugc.............	1	0	DLD
....aaaaaccgcaauuacuuuugca............	3	0	j18
....caaaaaccgcaauuacuuuu...............	2	0	ku3
....caaaaaccgcaauuacuuuug..............	1	0	j16
....caaaaaccgcaauuacuuuugc.............	1	0	j16
....aaaaaccgcaauuacuuuug..............	2	0	j16
....aaaaaccgcaauuacuuuugca............	2	0	j16
....caaaaaccgcaauuacuuu................	1	0	NL3
....caaaaaccgcaauuacuuuu...............	1	0	NL3
....auuacuuuugcaccaaccuaa...	1	0	NL3
....caaaaaccgcaauuacuuuu...............	1	0	ku6
....aaaaaccgcaauuacuuuu...............	1	0	ku6
....caaaaaccgcaauuacuuuu...............	1	0	ya2
....aaaaaccgcaauuacuuuu...............	2	0	ya2
....caauuacuuuugcaccaac.......	1	0	ya2
....aauuacuuuugcaccaac.......	1	0	ya2
....caaaaaccgcaauuacuuuu...............	2	0	ya3
....aaaaaccgcaauuacuuu................	1	0	ya3
....aaaaaccgcaauuacuuuu...............	2	0	ya3
....aaaaaccgcaauuacuuuug..............	1	0	ya3
....caaaaaccgcaauuacuuuugc.............	1	0	sh2
....uuacuuuugcaccaaccua....	1	0	sh2
....caaaaaccgcaauuacuuuugc.............	8	0	HEK
....aaaaaccgcaauuacuuuugc.............	2	0	HEK
....aaaaaccgcaauuacuuuugca............	3	0	HEK

```
          Star                                    Mature
agguugcugcaaaaguaacugagguuuuugccauugaaaguaaugacaaaaaccgcaauuacuuuugcaccaaccuaaagc

..............................................caaaaaccgcaauuacuuuug..............    2     0     j15

..............................................caaaaaccgcaauuacuuuugc.............    1     0     MCF
...............................................aaaaaccgcaauuacuuuug..............    1     0     MCF
...............................................aaaaaccgcaauuacuuuugc.............    1     0     MCF
........................................................aauuacuuuugcaccaaccuaa...    2     0     MCF

..........aaaaguaacugagguuuuugcc.................................................    1     0     NL1

..............................................caaaaaccgcaauuacuuuug..............    1     0     MBM
..............................................caaaaaccgcaauuacuuuugc.............    24    0     MBM
...............................................aaaaaccgcaauuacuuuug..............    1     0     MBM
...............................................aaaaaccgcaauuacuuuugc.............    4     0     MBM
...............................................aaaaaccgcaauuacuuuugca............    5     0     MBM

...............................................aaaaaccgcaauuacuuuugc.............    2     0     li1

.................................................aaaccgcaauuacuuuugcacca.........    1     0     k13
........................................................aauuacuuuugcaccaaccuaa...    1     0     k13

...................................................accgcaauuacuuuugcacca.........    1     0     j20

..............................................caaaaaccgcaauuacuuuugc.............    3     0     A54

..............................................caaaaaccgcaauuacuuuu...............    3     0     ya4
...............................................aaaaaccgcaauuacuuuu...............    2     0     ya4

..............................................caaaaaccgcaauuacuuuug..............    3     0     SW4
..............................................caaaaaccgcaauuacuuuugc.............    6     0     SW4
...............................................aaaaaccgcaauuacuuuug..............    1     0     SW4
...............................................aaaaaccgcaauuacuuuugc.............    1     0     SW4
...............................................aaaaaccgcaauuacuuuugca............    2     0     SW4

..............................................caaaaaccgcaauuacuuuu...............    1     0     ku1
```

Provisional ID	:	hsa_can_1556
Score total	:	29.3
Score for star read(s)	:	3.9
Score for read counts	:	25.1
Score for mfe	:	0.9
Score for randfold	:	
Score for cons. seed	:	-0.6
Total read count	:	61
Mature read count	:	37
Loop read count	:	1
Star read count	:	23

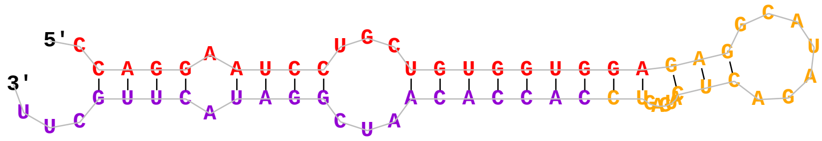

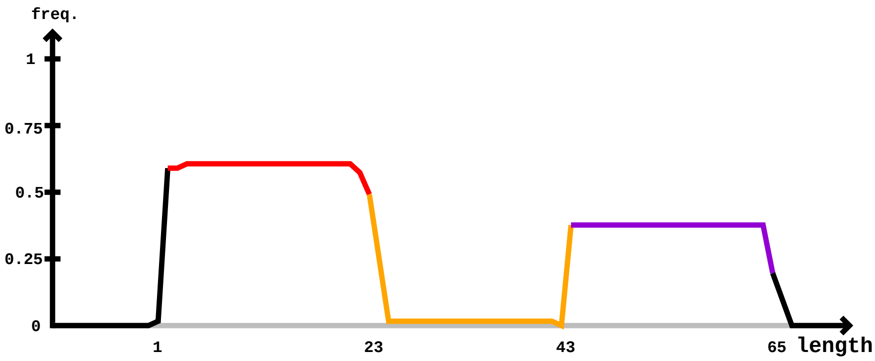

Mature **Star**

```
5'- ggaauggaagccaggaauccugcuguggugga gaggcauagacucaugaguccaccacaaucggauacuugcuucuuuuua  -3'   obs
    ggaauggaagccaggaauccugcugugguggagaggcauagacucaugaguccaccacaaucggauacuugcuucuuuuua        exp
    .(((.((((((.(((.((((...((((((((((((.......))).....)))))))))...)))).))))))))).))).   reads   mm   sample
    ....................................................caccacaaucggauacuugcuu.......   1       0    s08

    ..........ccaggaauccugcuguggug...................................................   1       0    B14
    ..........ccaggaauccugcugugguggu..................................................   1       0    B14
    ..........ccaggaauccugcuguggugga.................................................   3       0    B14
    ..........ccaggaauccugcugugguggag................................................   1       0    B14
    ....................................................caccacaaucggauacuugcuu.......   2       0    B14

    ..........ccaggaauccugcuguggugga.................................................   1       0    k03

    ....................................................caccacaaucggauacuugcu........   1       0    s12

    ....................................................caccacaaucggauacuugcu........   1       0    k05

    ..........ccaggaauccugcugugguggu..................................................   1       0    s06
    ..........ccaggaauccugcuguggugga.................................................   1       0    s06
    ............aggaauccugcugugguggag................................................   1       0    s06

    ..........ccaggaauccugcuguggugga.................................................   4       0    HeL
    ....................................................caccacaaucggauacuugcuu.......   2       0    HeL

    ..........ccaggaauccugcuguggugga.................................................   1       0    sh2

    ....................................................caccacaaucggauacuugcuu.......   1       0    k07

    .........gccaggaauccugcuguggug...................................................   1       0    li2
    ..........ccaggaauccugcugugguggu..................................................   1       0    li2
    ..........ccaggaauccugcuguggugga.................................................   10      0    li2
    ....................................................caccacaaucggauacuugcu........   1       0    li2

    ..........ccaggaauccugcuguggugga.................................................   1       0    k01
    ....................................................caccacaaucggauacuugcuu.......   1       0    k01

    ..........ccaggaauccugcuguggugga.................................................   4       0    U20
```

```
          Mature                                  Star
ggaauggaagccaggaauccugcuguggugga gaggcauagacucaugagucaccacaaucggauacuugcuucuuuuua

...................................................caccacaaucggauacuugcuu.......       1           0           k02

..........ccaggaauccugcuguggugga................................................       1           0           k12
...................................................caccacaaucggauacuugcu........       1           0           k12

..........ccaggaauccugcugugguggg.................................................       2           0           H52
..........ccaggaauccugcuguggugga................................................       2           0           H52
...................................................caccacaaucggauacuugcu........       7           0           H52
...................................................caccacaaucggauacuugcuu.......       3           0           H52

...................................................caccacaaucggauacuugcuu.......       1           0           s03

................................aggcauagacucaugagu..............................       1           0           j22
```

Provisional ID	:	hsa_can_1563
Score total	:	3.5
Score for star read(s)	:	-1.3
Score for read counts	:	0
Score for mfe	:	1.8
Score for randfold	:	
Score for cons. seed	:	3
Total read count	:	422
Mature read count	:	422
Loop read count	:	0
Star read count	:	0

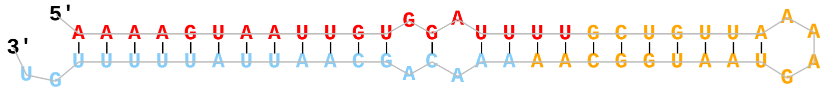

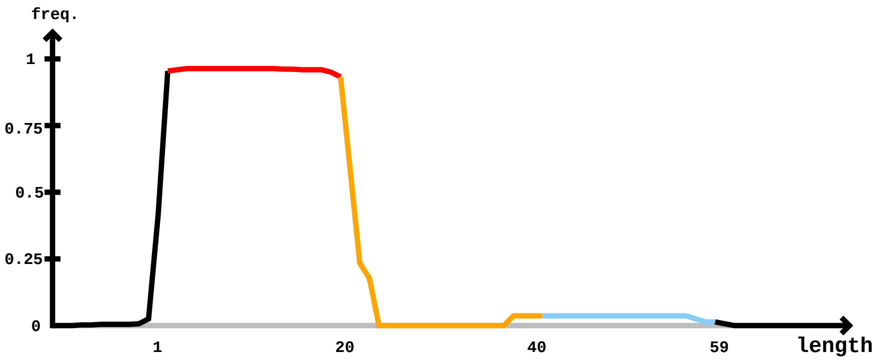

Mature Star

```
5'- agguuggugcaaaaguaauuguggauuuugcuguuaaaaguaauggcaaaaacagcaauuauuuuuguaccagccuaguau  -3'    exp
    agguuggugcaaaaguaauuguggauuuugcuguuaaaaguaauggcaaaaacagcaauuauuuuuguaccagccuaguau          known
    (((((((((((((((((((((.(.(((((((((((....))))))))))).).))))))))))))))))))))).....    reads      mm          sample
    ........gcaaaaguaauuguggauuuu...................................................    1          0           ya1
    .........caaaaguaauuguggauuu....................................................    1          0           ya1
    .........caaaaguaauuguggauuuu...................................................    16         0           ya1
    .........caaaaguaauuguggauuuug..................................................    3          0           ya1
    ..........aaaaguaauuguggauuuu...................................................    6          0           ya1
    ..........aaaaguaauuguggauuuug..................................................    2          0           ya1
    ..........aaaaguaauuguggauuuugc.................................................    2          0           ya1
    ...............................................caaaaacagcaauuauuuu...............    3          0           ya1
    ...............................................caaaaacagcaauuauuuuu..............    1          0           ya1
    ...............................................caaaaacagcaauuauuuuugu............    3          0           ya1

    .........caaaaguaauuguggauuuug..................................................    1          0           j26

    .........caaaaguaauuguggauuuug..................................................    2          0           va1
    ..........aaaaguaauuguggauuuug..................................................    3          0           va1
    ..........aaaaguaauuguggauuuugcu................................................    1          0           va1

    .........caaaaguaauuguggauuuug..................................................    2          0           ku5

    ..........aaaaguaauuguggauuuugc.................................................    1          0           k05
    ..........aaaaguaauuguggauuuugcu................................................    2          0           k05

    .........caaaaguaauuguggauuuug..................................................    2          0           s06

    .........caaaaguaauuguggauuuug..................................................    3          0           ku4
    ..........aaaaguaauuguggauuuu...................................................    1          0           ku4
    ..........aaaaguaauuguggauuuug..................................................    1          0           ku4

    .........caaaaguaauuguggauuuug..................................................    1          0           k08
    ..........aaaaguaauuguggauuuugcu................................................    2          0           k08

    .........caaaaguaauuguggauuuug..................................................    1          0           ta3

    ..........aaaaguaauuguggauuuug..................................................    10         0           j06
```

Mature Star

```
agguuggugcaaaaguaauuguggauuuugcuguuaaaaguaauggcaaaaacagcaauuauuuuuguaccagccuaguau

.........caaaaguaauuguggauuuug...................................................	1	0	j10

.........caaaaguaauuguggauuuug...................................................	1	0	k14
..........aaaaguaauuguggauuuugc..................................................	1	0	k14
..........aaaaguaauuguggauuuugcu.................................................	1	0	k14

..........aaaaguaauuguggauuuug...................................................	2	0	j31

.........caaaaguaauuguggauuuug...................................................	2	0	j27
..........aaaaguaauuguggauuuug...................................................	1	0	j27

........gcaaaaguaauuguggauuuug...................................................	2	0	j22
.........caaaaguaauuguggauuu.....................................................	1	0	j22
.........caaaaguaauuguggauuuug...................................................	6	0	j22
..........aaaaguaauuguggauuuu....................................................	1	0	j22
..........aaaaguaauuguggauuuug...................................................	1	0	j22
..........aaaaguaauuguggauuuugc..................................................	4	0	j22
..........aaaaguaauuguggauuuugcu.................................................	13	0	j22

...uuggugcaaaaguaauugugg.........................................................	1	0	k09
..........aaaaguaauuguggauuuugc..................................................	1	0	k09

..........aaaaguaauuguggauuuu....................................................	1	0	B14

..........aaaaguaauuguggauuuug...................................................	1	0	j16

........gcaaaaguaauuguggauuuug...................................................	2	0	j18
..........aaaaguaauuguggauuuug...................................................	1	0	j18

.........caaaaguaauuguggauuuu....................................................	1	0	ku3
.........caaaaguaauuguggauuuug...................................................	2	0	ku3
..........aaaaguaauuguggauuuug...................................................	1	0	ku3

..........aaaaguaauuguggauuuug...................................................	1	0	ku6

........gcaaaaguaauuguggauuuug...................................................	1	0	j11
.........caaaaguaauuguggauuuug...................................................	4	0	j11

.........caaaaguaauuguggauuu.....................................................	2	0	ya3
.........caaaaguaauuguggauuuu....................................................	16	0	ya3
.........caaaaguaauuguggauuuug...................................................	8	0	ya3
..........aaaaguaauuguggauuu.....................................................	1	0	ya3
..........aaaaguaauuguggauuuu....................................................	13	0	ya3
..............................................caaaaacagcaauuauuuuu...............	1	0	ya3

..........aaaaguaauuguggauuuugcu.................................................	1	0	HEK

.........caaaaguaauuguggauuuug...................................................	1	0	la1

.........caaaaguaauuguggauuuug...................................................	1	0	j15

.........caaaaguaauuguggauuuu....................................................	2	0	j17
.........caaaaguaauuguggauuuug...................................................	5	0	j17
..........aaaaguaauuguggauuuug...................................................	2	0	j17

..........aaaaguaauuguggauuuug...................................................	1	0	la2

.........caaaaguaauuguggauuuug...................................................	1	0	j12

.........caaaaguaauuguggauuuug...................................................	1	0	li2

.........caaaaguaauuguggauuuug...................................................	1	0	va4

........gcaaaaguaauuguggauuuug...................................................	1	0	va3
.........caaaaguaauuguggauuuug...................................................	5	0	va3

..............................................caaaaacagcaauuauuuuugu.............	2	0	SW4

.........caaaaguaauuguggauu......................................................	4	0	ya4
```

```
           Mature                                  Star
agguuggugcaaaaguaauuguggauuuugcuguuaaaaguaauggcaaaaacagcaauuauuuuuguaccagccuaguau
.........caaaaguaauuguggauuuu....................................................    8     0     ya4
.........caaaaguaauuguggauuuug...................................................    5     0     ya4
..........aaaaguaauuguggauuu.....................................................    2     0     ya4
..........aaaaguaauuguggauuuu....................................................    27    0     ya4
..........aaaaguaauuguggauuuug...................................................    4     0     ya4
..........aaaaguaauuguggauuuugcu.................................................    1     0     ya4
...........aaaguaauuguggauuuu....................................................    2     0     ya4
..............................................caaaaacagcaauuauuuu................    1     0     ya4
..............................................caaaaacagcaauuauuuuu...............    3     0     ya4

.......ugcaaaaguaauuguggauuuu....................................................    1     0     j23
........gcaaaaguaauuguggauuuug...................................................    1     0     j23
.........caaaaguaauuguggauuuug...................................................    11    0     j23
..........aaaaguaauuguggauuuu....................................................    1     0     j23
..........aaaaguaauuguggauuuug...................................................    2     0     j23
..........aaaaguaauuguggauuuugc..................................................    2     0     j23
..........aaaaguaauuguggauuuugcu.................................................    1     0     j23
..............................................caaaaacagcaauuauuuuugu.............    1     0     j23

.........caaaaguaauuguggauuuug...................................................    2     0     s05

..........aaaaguaauuguggauuuu....................................................    3     0     j24
..........aaaaguaauuguggauuuug...................................................    4     0     j24
..........aaaaguaauuguggauuuugcu.................................................    5     0     j24

..........aaaaguaauuguggauuuugcu.................................................    1     0     k11

..........aaaaguaauuguggauuuug...................................................    1     0     s09

.........caaaaguaauuguggauuuu....................................................    5     0     NL2
..........aaaaguaauuguggauuuu....................................................    1     0     NL2
..........aaaaguaauuguggauuuugcu.................................................    1     0     NL2

.........caaaaguaauuguggauuuug...................................................    1     0     j13
..........aaaaguaauuguggauuuug...................................................    1     0     j13

..........aaaaguaauuguggauuuug...................................................    2     0     k10
..........aaaaguaauuguggauuuugc..................................................    1     0     k10
..........aaaaguaauuguggauuuugcu.................................................    2     0     k10

.........caaaaguaauuguggauuuug...................................................    1     0     j19

..........aaaaguaauuguggauuuu....................................................    1     0     j03

.........caaaaguaauuguggauuuug...................................................    1     0     j25

..........aaaaguaauuguggauuuugc..................................................    1     0     k01
..........aaaaguaauuguggauuuugcu.................................................    2     0     k01

..........aaaaguaauuguggauuuug...................................................    1     0     k04
..........aaaaguaauuguggauuuugc..................................................    1     0     k04
..........aaaaguaauuguggauuuugcu.................................................    1     0     k04

.........caaaaguaauuguggauuuug...................................................    1     0     ku2
..........aaaaguaauuguggauuuug...................................................    2     0     ku2

.........caaaaguaauuguggauuuug...................................................    5     0     j21


.........caaaaguaauuguggauuuug...................................................    4     0     j14
..........aaaaguaauuguggauuuug...................................................    4     0     j14

.........caaaaguaauuguggauuuug...................................................    1     0     k02
..........aaaaguaauuguggauuuugc..................................................    1     0     k02
..........aaaaguaauuguggauuuugcu.................................................    1     0     k02

.........caaaaguaauuguggauuuug...................................................    2     0     s08

..........aaaaguaauuguggauuuugc..................................................    1     0     k03
..........aaaaguaauuguggauuuugcu.................................................    4     0     k03
```

Mature **Star**

agguuggugcaaaaguaauuguggauuuugcuguuaaaaguaauggcaaaaacagcaauuauuuuuguaccagccuaguau

.........caaaaguaauuguggauuuu..	1	0	NL3
.........caaaaguaauuguggauuuug...	1	0	NL3
..........aaaaguaauuguggauuu...	1	0	NL3
..........aaaaguaauuguggauuuu..	2	0	NL3
.........caaaaguaauuguggauuuu..	8	0	ya2
.........caaaaguaauuguggauuuug...	1	0	ya2
..........aaaaguaauuguggauuuu..	16	0	ya2
..........aaaaguaauuguggauuuug...	2	0	ya2
..........aaaaguaauuguggauuuugc..	1	0	ya2
....caaaaacagcaauuauuuu................	1	0	ya2
.........caaaaguaauuguggauuuug...	1	0	k06
..........aaaaguaauuguggauuuugcu...	4	0	k06
.gguuggugcaaaaguaauugu...	1	0	sh2
..........aaaaguaauuguggauuuugcu...	24	0	sh2
.........caaaaguaauuguggauuuug...	2	0	MCF
..........aaaaguaauuguggauuuugcu...	2	0	MCF
..........aaaaguaauuguggauuuug...	5	0	j01
.........caaaaguaauuguggauuuug...	1	0	MBM
..........aaaaguaauuguggauuuugc..	1	0	NL1
..........aaaaguaauuguggauuuugcu...	2	0	k07
..........aaaaguaauuguggauuuugc..	1	0	sh1
.........caaaaguaauuguggauuuug...	4	0	li1
..........aaaaguaauuguggauuuugc..	5	0	li1
..........aaaaguaauuguggauuuugcu...	4	0	li1
.........caaaaguaauuguggauuuug...	1	0	k13
..........aaaaguaauuguggauuuug...	1	0	k13
..........aaaaguaauuguggauuuugcu...	1	0	k13
.........caaaaguaauuguggauuuu..	2	0	j20
.........caaaaguaauuguggauuuug...	1	0	j20
..........aaaaguaauuguggauuuug...	2	0	j20
.........caaaaguaauuguggauuuug...	3	0	A54
.........caaaaguaauuguggauuuug...	1	0	j30
.........caaaaguaauuguggauuuug...	1	0	k12
..........aaaaguaauuguggauuuug...	2	0	k12
..........aaaaguaauuguggauuuugcu...	1	0	k12
............aaguaauuguggauuuugc..	1	0	k12
............aaguaauuguggauuuugcu...	1	0	k12

Provisional ID	:	hsa_can_1579
Score total	:	34.5
Score for star read(s)	:	3.9
Score for read counts	:	29.7
Score for mfe	:	1.5
Score for randfold	:	
Score for cons. seed	:	-0.6
Total read count	:	70
Mature read count	:	35
Loop read count	:	0
Star read count	:	35

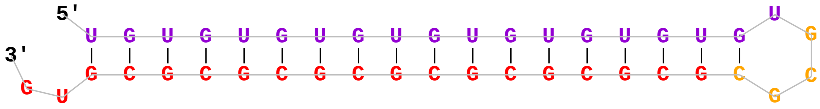

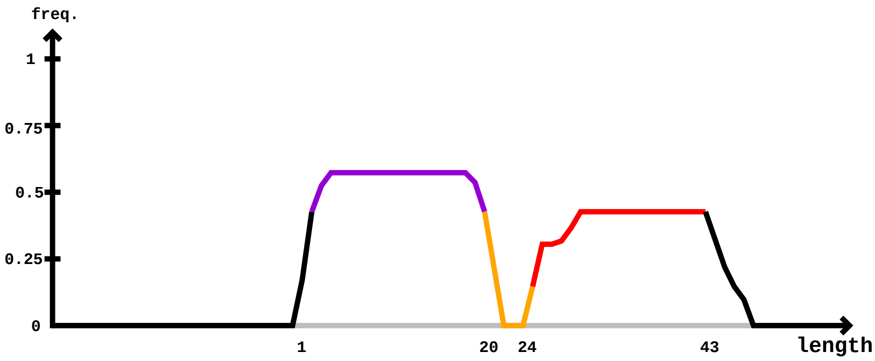


```
                        Star                  Mature
5'- ucuguucaaaaggcugcuggguaagugugugugugugugugugugcgcgcgcgcgcgcgcgcgcgugugguugguucuucag  -3'   obs
    ucuguucaaaaggcugcuggguaagugugugugugugugugugugcgcgcgcgcgcgcgcgcgcgugugguugguucuucag        exp
    ..........((..((..(.((..(((((((((((((((((....)))))))))))))))))..)).)..))..))..   reads   mm   sample
    .........................ugugugugugugugugugu......................................   1       0    k09

    .........................ugugugugugugugugugu......................................   1       0    j23

    ................................................gcgcgcgcgcgcgcgcgugug.............   1       0    DLD
    .....................................................cgcgcgcgcgcguguggu...........   1       0    DLD

    ........................gugugugugugugugugug.......................................   6       0    ya1
    ..........................gugugugugugugugugug.....................................   6       0    ya1

    ........................gugugugugugugugugug.......................................   2       0    B14
    ..........................gugugugugugugugugug.....................................   2       0    B14
    .................................................cgcgcgcgcgcgcgcgug...............   2       0    B14

    ....................................................gcgcgcgcgcgcguguggu...........   1       0    j09

    .........................ugugugugugugugugugug.....................................   2       0    j16
    .................................................cgcgcgcgcgcgcgcgugu..............   1       0    j16

    ........................gugugugugugugugugugu......................................   2       0    j18

    ........................gugugugugugugugugu........................................   2       0    NL3
    ..........................gugugugugugugugugu......................................   2       0    NL3

    ........................gugugugugugugugugu........................................   4       0    ya2
    .........................ugugugugugugugugug.......................................   2       0    ya2
    ..........................gugugugugugugugugu......................................   4       0    ya2
    ...........................ugugugugugugugugug.....................................   2       0    ya2

    .........................ugugugugugugugugugu......................................   2       0    j11

    ....................................................gcgcgcgcgcgcgugugg............   1       0    ya3

    .........................ugugugugugugugugugu......................................   1       0    ta1
```

Star Mature

```
ucuguucaaaaggcugcugguaagugugugugugugugugugugcgcgcgcgcgcgcgcgcgcgugugguuggucuucag
...................................................gcgcgcgcgcgcgugugg...........	1	0	ta1

........................ugugugugugugugugug......................................	2	0	HeL
..........................ugugugugugugugugug....................................	2	0	HeL
...............................................gcgcgcgcgcgcgcgcgugug............	1	0	HeL

................................................cgcgcgcgcgcgcgcgug..............	1	0	HEK

........................ugugugugugugugugugu.....................................	1	0	la1

.......................gugugugugugugugugugu.....................................	1	0	sh2

...............................................gcgcgcgcgcgcgcgcgug..............	1	0	MBM
................................................cgcgcgcgcgcgcgcgug..............	2	0	MBM

........................ugugugugugugugugugu.....................................	1	0	la2
........................ugugugugugugugugugug....................................	1	0	la2

........................ugugugugugugugugug......................................	2	0	s10
........................ugugugugugugugugugu.....................................	1	0	s10
..........................ugugugugugugugugug....................................	2	0	s10

.......................gugugugugugugugugugu.....................................	2	0	NL1

........................ugugugugugugugugugug....................................	1	0	k10

.......................gugugugugugugugugug......................................	2	0	li1
.........................gugugugugugugugugug....................................	2	0	li1
...............................................gcgcgcgcgcgcgcgcgug..............	7	0	li1
...............................................gcgcgcgcgcgcgcgcgugugg...........	1	0	li1
................................................cgcgcgcgcgcgcgcgugugg...........	1	0	li1

........................ugugugugugugugugugu.....................................	1	0	per

.......................gugugugugugugugugugu.....................................	1	0	k04

........................ugugugugugugugugugu.....................................	2	0	U20

........................ugugugugugugugugug......................................	2	0	j21
..........................ugugugugugugugugug....................................	2	0	j21

...................................................gcgcgcgcgcgcguguggu..........	1	0	j27

........................ugugugugugugugugugu.....................................	1	0	H52
...............................................gcgcgcgcgcgcgcgcgugug............	1	0	H52
................................................cgcgcgcgcgcgcgcgug..............	3	0	H52
................................................cgcgcgcgcgcgcgcgugug............	3	0	H52
..................................................cgcgcgcgcgcgcguguggu..........	1	0	H52
....................................................cgcgcgcgcgcguguggu..........	4	0	H52

........................ugugugugugugugugugu.....................................	1	0	j22
```

Provisional ID	:	hsa_can_1591
Score total	:	17.6
Score for star read(s)	:	3.9
Score for read counts	:	12.4
Score for mfe	:	1.9
Score for randfold	:	
Score for cons. seed	:	-0.6
Total read count	:	36
Mature read count	:	33
Loop read count	:	0
Star read count	:	3

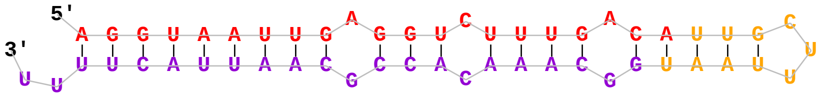

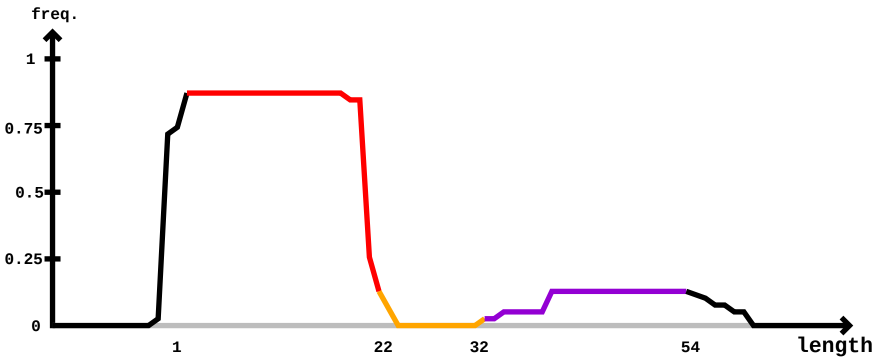

Mature Star

```
5'- agguuggugcaaagguaauugaggucuuugacauugcuuuaauggcaaacaccgcaauuacuuuugcaccagccuaacaaa  -3'    obs
    agguuggugcaaagguaauugaggucuuugacauugcuuuaauggcaaacaccgcaauuacuuuugcaccagccuaacaaa          exp
    ((((((((((((((((((((.(((.((((.(((((...))))).)))).))).)))))))))))))))))))).......    reads      mm         sample
    .................................................accgcaauuacuuuugcacca..........    1          0          k09

    .........aaagguaauugaggucuuugac.................................................    1          0          j23

    .........aaagguaauugaggucuuuga..................................................    2          0          ku3

    .........aaagguaauugaggucuuuga..................................................    1          0          NL3

    .........aaagguaauugaggucuuuga..................................................    1          0          ya2
    ..........................................ggcaaacaccgcaauuacuuuu................    1          0          ya2

    .........aaagguaauugaggucuuuga..................................................    2          0          va1
    .........aaagguaauugaggucuuugac.................................................    1          0          va1

    .........aaagguaauugaggucuuuga..................................................    1          0          ta2

    ..........aagguaauugaggucuuu....................................................    1          0          ya3

    .........aaagguaauugaggucuuuga..................................................    1          0          s10

    .........aaagguaauugaggucuuuga..................................................    1          0          k08

    .........aaagguaauugaggucuuuga..................................................    1          0          NL2
    .................................................accgcaauuacuuuugcac............    1          0          NL2

    .........aaagguaauugaggucuuuga..................................................    1          0          j12

    .........aaagguaauugaggucuuugac.................................................    1          0          sh1

    .........aaagguaauugaggucuuuga..................................................    1          0          j25

    ...........agguaauugaggucuuugaca................................................    1          0          j10

    ...........agguaauugaggucuuugaca................................................    4          0          U20
```

Mature Star

```
agguuggugcaaagguaauugaggucuuugacauugcuuuaauggcaaacaccgcaauuacuuuugcaccagccuaacaaa
.............................................caaacaccgcaauuacuuuugc..............    1    0    U20
..........aaagguaauugaggucuuuga..................................................    2    0    va4
..........aaagguaauugaggucuuuga..................................................    1    0    ku2
..........aaagguaauugaggucuuugac.................................................    1    0    j21
..................................................accgcaauuacuuuugcacca..........    1    0    j20
..........aaagguaauugaggucuuuga..................................................    3    0    j04
.........caaagguaauugaggucuuugac.................................................    1    0    va3
..........aaagguaauugaggucuuuga..................................................    1    0    j14
..........aaagguaauugaggucuuuga..................................................    1    0    j30
..........aaagguaauugaggucuuuga..................................................    2    0    ku1
..........aaagguaauugaggucuuuga..................................................    1    0    j22
```

Provisional ID	:	hsa_can_1617
Score total	:	119.1
Score for star read(s)	:	3.9
Score for read counts	:	114.3
Score for mfe	:	1.4
Score for randfold	:	
Score for cons. seed	:	-0.6
Total read count	:	236
Mature read count	:	220
Loop read count	:	1
Star read count	:	15

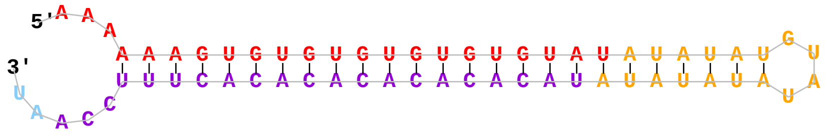

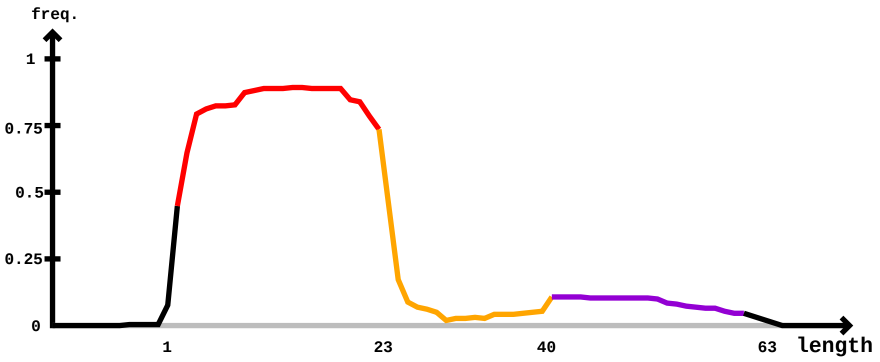

Mature **Star**

```
5'- cucaaaaaaaaaaaaaaguguguguguguguauauauauguauauauauauacacacacacacacuuuccaauaccagucg  -3'   obs
    cucaaaaaaaaaaaaaaguguguguguguguauauauauguauauauauauauacacacacacacacuuuccaauaccagucg        exp
    ..............(((((((((((((((((((((((((....))))))))))))))))))))))))).............     reads   mm   sample
    ...........aaaaaaguguguguguguguau................................................     1       0    j23
    ............aaaaaguguguguguguguaua...............................................     1       0    j23
    ...............aaguguguguguguguau................................................     1       0    j23
    ....................uguguguguguauauauaugu........................................     1       0    j23

    ..........aaaaaaagugugugugugu....................................................     4       0    ya1
    ...........aaaaaagugugugugugu....................................................     1       0    ya1
    ...........aaaaaaguguguguguguguau................................................     4       0    ya1
    ...........aaaaaaguguguguguguguaua...............................................     1       0    ya1
    ............aaaaaguguguguguguguau................................................     3       0    ya1
    ............aaaaaguguguguguguguaua...............................................     2       0    ya1
    .............aaaagugugugugugu....................................................     3       0    ya1
    .............aaaaguguguguguguguau................................................     1       0    ya1
    .............aaaaguguguguguguguauau..............................................     5       0    ya1
    ..............aaaguguguguguguguaua...............................................     1       0    ya1
    ..................uguguguguguguauauau............................................     2       0    ya1
    ...................guguguguguguauauau............................................     1       0    ya1

    ...........aaaaaaguguguguguguguau................................................     10      0    j26
    ...........aaaaaaguguguguguguguaua...............................................     1       0    j26
    ............aaaaagugugugugugu....................................................     1       0    j26
    ............aaaaaguguguguguguguau................................................     1       0    j26
    .............aaaaguguguguguguguau................................................     2       0    j26
    ...............aaguguguguguguguauauaua...........................................     1       0    j26

    ..........aaaaaaaguguguguguguguau................................................     1       0    ta2
    .............aaaaguguguguguguguauau..............................................     1       0    ta2

    .........................................auauauauauacacacacaca...................     2       0    ku5

    ...........aaaaaaguguguguguguguau................................................     2       0    ta1
    .............aaaaguguguguguguguau................................................     2       0    ta1
    ..................................................uacacacacacacacuuucca..........     1       0    ta1
```

```
           Mature                                 Star
cucaaaaaaaaaaaaaaguguguguguguguauauauauguauauauauauacacacacacacacuuuccaauaccagucg
..................................................uacacacacacacacuuuccaa.........    1          0          ta1

............aaaaaguguguguguguguaua...............................................    1          0          s09
..................uguguguguguguauauau............................................    1          0          s09
...............................................auauacacacacacacacuuu.............    1          0          s09

......aaaaaaaaaaagugugugu........................................................    1          0          HeL
.............aaaaguguguguguguguauau..............................................    1          0          HeL

...........aaaaaagugugugugugugu..................................................    1          0          ku4
...........aaaaaagugugugugugugugua...............................................    1          0          ku4
...........aaaaaaguguguguguguguau................................................    1          0          ku4
............aaaaaguguguguguguguauau..............................................    1          0          ku4
.............aaaaguguguguguguguau................................................    1          0          ku4

...........aaaaaaguguguguguguguau................................................    2          0          s07

..................uguguguguguguauaua.............................................    2          0          s10
..................uguguguguguguauauauau..........................................    1          0          s10

...........aaaaaagugugugugugu....................................................    1          0          NL2
...........aaaaaaguguguguguguguau................................................    3          0          NL2
.............aaaaguguguguguguguauau..............................................    2          0          NL2

...........aaaaaagugugugugugugugua...............................................    1          0          ta3
...........aaaaaaguguguguguguguau................................................    4          0          ta3
............aaaaaguguguguguguguau................................................    1          0          ta3
............aaaaaguguguguguguguaua...............................................    2          0          ta3
.............aaaaguguguguguguguau................................................    1          0          ta3
..................................................uacacacacacacacuuucca..........    1          0          ta3
..................................................uacacacacacacacuuuccaa.........    1          0          ta3

............aaaaaguguguguguguguaua...............................................    2          0          j13
.............aaaaguguguguguguguaua...............................................    1          0          j13
....................uguguguguguauauauauguau......................................    1          0          j13
............................................uauauauacacacacacaca.................    1          0          j13

.............aaaaguguguguguguguau................................................    1          0          j19

...........aaaaaagugugugugugugugua...............................................    2          0          j03

..................uguguguguguguauauauau..........................................    1          0          k04

..........aaaaaaagugugugugugugu..................................................    2          0          per
...........aaaaaagugugugugugugu..................................................    2          0          per
...........aaaaaaguguguguguguguau................................................    4          0          per
............aaaaaguguguguguguguau................................................    2          0          per
...............aaguguguguguguguauauaua...........................................    1          0          per
.................guguguguguguguauauauau..........................................    1          0          per
..................uguguguguguguauaua.............................................    1          0          per

...................guguguguguguauauauaugu........................................    1          0          U20
.......................guguguguauauauaugua.......................................    1          0          U20

.............aaaaguguguguguguguauau..............................................    1          0          j10

...........aaaaaagugugugugugugugua...............................................    1          0          ku2
...........aaaaaaguguguguguguguaua...............................................    1          0          ku2
............aaaaaguguguguguguguau................................................    1          0          ku2
.............aaaaguguguguguguguauau..............................................    1          0          ku2
..................................................uacacacacacacacuuuccaa.........    1          0          ku2

............aaaaaguguguguguguguau................................................    1          0          j21
.............aaaaguguguguguguguauau..............................................    1          0          j21
..............aaaguguguguguguguauau..............................................    1          0          j21
.................................................auacacacacacacacuuuc............    1          0          j21

............................................uauauauacacacacacacacu...............    1          0          va2

...........aaaaaaguguguguguguguau................................................    1          0          j02
```

Mature **Star**

cucaaaaaaaaaaaaaaguguguguguguguauauauauguauauauauauacacacacacacacuuuccaauaccagucg			
.............aaaaguguguguguguguaua...	2	0	j02
.............aaaaguguguguguguguauau..	1	0	j02
...........aaaaaaguguguguguguguau..	1	0	s11
....uacacacacacacacuuucca..........	2	0	s11
...........aaaaaaguguguguguguguau..	4	0	j27
............aaaaaguguguguguguguau..	1	0	j27
............aaaaaguguguguguguguaua...	2	0	j27
..........aaaaaaagugugugugugugu..	3	0	j28
..........aaaaaaagugugugugugugua...	1	0	j28
..........aaaaaaaguguguguguguguau..	5	0	j28
...........aaaaaagugugugugugugu..	1	0	j28
...........aaaaaagugugugugugugua...	2	0	j28
...........aaaaaaguguguguguguguau..	13	0	j28
...........aaaaaaguguguguguguguaua...	3	0	j28
............aaaaaguguguguguguguau..	12	0	j28
............aaaaaguguguguguguguaua...	6	0	j28
.............aaaaguguguguguguguau..	1	0	j28
.............aaaaguguguguguguguauau..	1	0	j28
..................uguguguguguguauauau..	1	0	j28
....uauauauauauacacacacaca...................	2	0	j28
....uacacacacacacacuuucca..........	2	0	j28
....uauauauauacacacacac....................	1	0	H52
...........aaaaaaguguguguguguguau..	1	0	j22
.............aaaaguguguguguguguauau..	1	0	j22
..........aaaaaaaguguguguguguguau..	1	0	s08
.............aaaaguguguguguguguauau..	1	0	s08
............aaaaaguguguguguguguau..	1	0	ku3
............aaaaaguguguguguguguaua...	1	0	ku3
.............aaaagugugugugugugua...	1	0	ku3
....uacacacacacacacuuuccaa.........	1	0	ku3
...........aaaaaagugugugugugu..	2	0	ya2
...........aaaaaaguguguguguguguaua...	2	0	ya2
.............aaaaguguguguguguguau..	1	0	ya2
...........aaaaaagugugugugugu..	1	0	NL3
............aaaaaguguguguguguguau..	1	0	NL3
.............aaaaguguguguguguguauau..	1	0	NL3
..................uguguguguguguauauauau..	1	0	NL3
...........aaaaaagugugugugugugua...	3	0	j11
...........aaaaaaguguguguguguguau..	1	0	j11
..............aaaguguguguguguguaua...	2	0	j11
...........aaaaaagugugugugugu..	2	0	ya3
...........aaaaaagugugugugugug...	1	0	ya3
...........aaaaaaguguguguguguguau..	1	0	ya3
.............aaaaguguguguguguguau..	1	0	ya3
..................uguguguguguguauauaua...	1	0	ya3
....uauauauacacacacacac..................	1	0	ya3
.............aaaaguguguguguguguauau..	1	0	j29
...........aaaaaaguguguguguguguau..	1	0	la1
....uacacacacacacacuuucca..........	1	0	j17
[truncated: 579,337 more chars]
